# Identification of m7G Methylation-Related miRNA Signature Associated with Survival and Immune Microenvironment Regulation in Uterine Corpus Endometrial Carcinoma

**DOI:** 10.1155/2022/8776678

**Published:** 2022-11-23

**Authors:** Rujun Chen, Ke Sun, Yue Hou, Jiayu Shen, Jina Chen, Fuyun Dong, Xiaoqin Wang, Lina Yang, Liwen Zhang

**Affiliations:** ^1^Department of Gynecology and Obstetrics, Shanghai Fifth People's Hospital, Fudan University, Shanghai 200240, China; ^2^Central laboratory, Shanghai Fifth People's Hospital, Fudan University, Shanghai 200240, China

## Abstract

**Background:**

N^7^-methylguanosine (m7G) has been implicated in the development of cancer. The role of m7G-related miRNAs in the survival prediction of UCEC patients has not been investigated. Current research was the first to construct an m7G-related miRNA model to accurately predict the survival of patients with uterine corpus endometrial carcinoma (UCEC) and to explore immune cell infiltration and immune activity in the tumor microenvironment.

**Methods:**

RNA-seq data and clinical information of UCEC patients were derived from The Cancer Genome Atlas (TCGA) database. Using the TargetScan online database, we predicted miRNAs linked to the m7G-related genes and identified miRNAs which were significantly associated with the survival in UCEC patients and constructed a risk scoring model. The TCGA-UCEC cases were scored according to the risk model, and the high- and low-risk groups were divided by the median risk value. Gene enrichment analysis and immune cell infiltration and immune function analysis were performed using “clusterProfiler” and “GSVA” packages in R.

**Results:**

The survival prediction model consisted of 9 miRNAs, namely, hsa-miR-1301, hsa-miR-940, hsa-miR-592, hsa-miR-3170, hsa-miR-876, hsa-miR-215, hsa-miR-934, hsa-miR-3920, and hsa-miR-216b. Survival of UCEC patients in the high-risk group was worse than that in the low-risk group (*p* < 0.001). The receiver operating characteristic (ROC) curve showed that the model had good predictive performance, and the area under the curve was 0.800, 0.690, and 0.705 for 1-, 3-, and 5-year survival predictions, respectively. There were differences in the degree of immune cell infiltration and immune activity between the low-risk and high-risk groups. The expression levels of the identified differentially expressed genes correlated with the susceptibility to multiple anticancer drugs.

**Conclusions:**

The survival prediction model constructed based on 9 m7G-related miRNAs had good predictive performance.

## 1. Introduction

Uterine corpus endometrial carcinoma (UCEC) is a common gynecological malignancy that endangers women's health. According to the 2020 global cancer statistics, there were more than 410,000 new UCEC cases and more than 97,000 new deaths worldwide [1]. In recent years, with the identification of an increasing number of markers and signaling pathways, rising novel approaches to the treatment of UCEC have emerged. However, the prognosis prediction of UCEC patients still needs to be explored.

N^7^-methylguanosine, or m7G, is a ubiquitous posttranscriptional RNA modification [2]. A methyl group is added to riboguanosine at the N7 position during transcription initiation [3]. A positively charged modification of RNA results from the modification of m7G at the 5′ cap catalyzed by methyltransferase cotranscriptionally [4]. m7G has been shown to be associated with a variety of biological processes and is involved in the development of various diseases, including cancers [5, 6].

**Figure 1:**
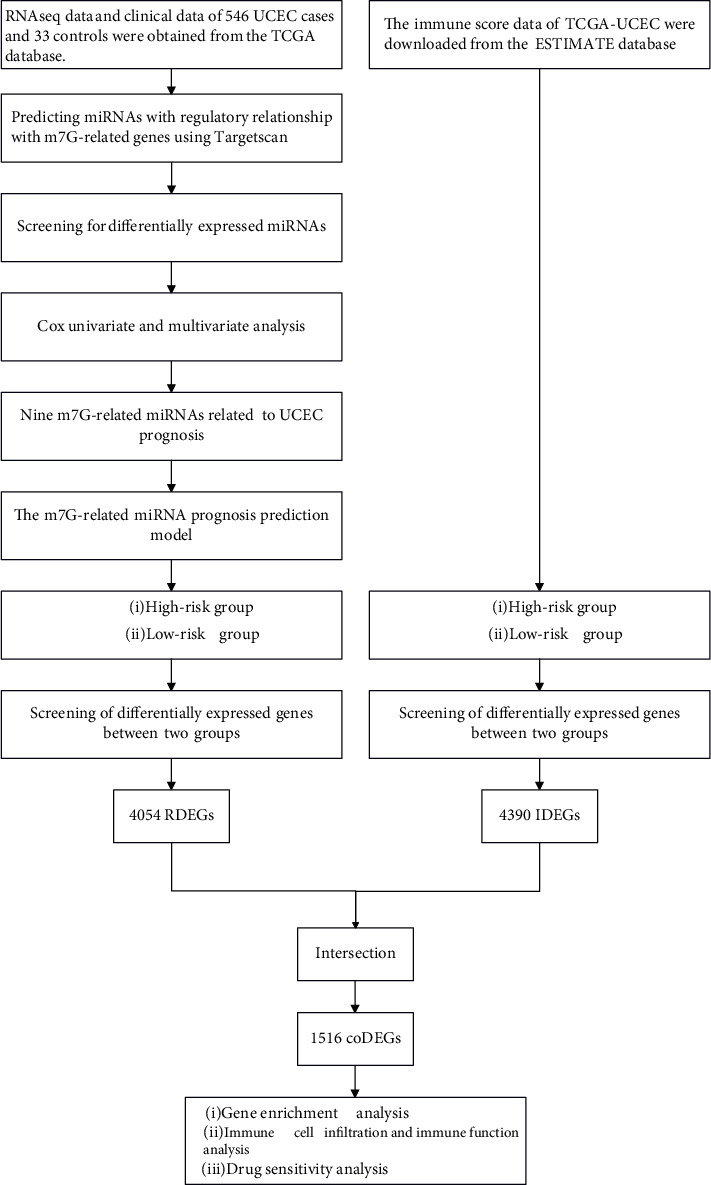
Flowchart of construction of the UCEC prognostic model.

In this study, we constructed a model of m7G-related microRNAs (miRNAs) for predicting survival in UCEC patients. Moreover, UCEC patients were divided into high- and low-risk groups based on risk score results, and differentially expressed genes were analyzed to explore the correlation with immune cell infiltration, immune activity, and anticancer drug sensitivity. The flow chart of our study is demonstrated in Figure 1.

## 2. Materials and Methods

### 2.1. Data Acquisition

RNA-seq expression data and clinical data of UCEC were derived from The Cancer Genome Atlas (TCGA) database (https://portal.gdc.cancer.gov/). A list of twenty-eight m7G-related genes was obtained from a previous literature [7] (Supplementary Table S1).

### 2.2. Construction of the m7G-Related miRNA Prognosis Prediction Model in UCEC

The obtained proteins encoded by 28 m7G-related genes play multiple roles in m7G biological processes, which in turn affect cancer progression [8–11]. It has been demonstrated that miRNAs regulate gene expression posttranscriptionally [12]. miRNAs can affect tumor progression by regulating the expression of downstream m7G-related genes [13, 14]. Based on this, we designed and constructed an m7G-related miRNA prognosis model. Using the TargetScan online database (https://www.targetscan.org/vert_72/) [15], we predicted miRNAs that may have regulatory relationships with m7G-related genes (Supplementary Table S2). Next, based on the transcriptome data obtained from the TCGA-UCEC cohort, differentially expressed miRNAs (DEmiRNAs) between tumor samples and normal samples were obtained by screening with the “edgeR” package in R [16] according to the following conditions: FDR < 0.05 and |log_2_FC| ≥ 1. To explore the relationship between the expression of DEmiRNAs and the survival of UCEC patients, we used the “survival” package in R to perform the Cox univariate and multivariate analyses and screened out DEmiRNAs with *p* value < 0.01. For the purpose of obtaining a generalized linear model and reducing error, 1000 Cox LASSO regression calculations and ten cross-validation cycles were performed with the R package “glmnet” [17]. The obtained DEmiRNAs significantly associated with survival were named SDEmiRNAs, and a risk prognostic model was constructed. Finally, to assess the sensitivity and specificity of the survival risk assessment model, we plotted ROC curves using the “timeROC” package in R for predicting survival at 1-, 3-, and 5-year survival [18].

### 2.3. Calculation of Risk Score

The specific equation for the risk score:

(1) $$\begin{array}{c} \text{Risk score}=\sum \text{C}\text{oef miRNA}\times \log2\left(\text{miRNA expression}+1\right). \end{array}$$

We divided the high- and low-risk groups in UCEC patients by the median risk score.

### 2.4. Screening of Differentially Expressed Genes

A risk score was calculated for each TCGA-UCEC case according to the constructed survival risk model, and the median risk score was used to distinguish high risk from low risk. Following this, we identified differentially expressed genes (DEGs) between the high-risk and low-risk groups by looking at the transcriptome data in the TCGA-UCEC cohort. Genes that were differentially expressed between the high-risk and low-risk groups were found by screening with the “edgeR” package in R under the following conditions: FDR < 0.05 and |log_2_FC| ≥ 1. We named the obtained genes RDEGs. Next, we collected the immune score data of TCGA-UCEC from the ESTIMATE database (https://bioinformatics.mdanderson.org/estimate/disease.html), differentiated the high score group and the low score group based on median immune scores, and designated those differentially expressed genes that met the conditions of FDR < 0.05 and |log_2_FC| ≥ 1 as IDEGs. To obtain codifferentially expressed genes (coDEGs), we took the intersection of RDEGs and IDEGs.

### 2.5. Gene Enrichment Analysis

We performed functional and pathway enrichment analysis of Gene Ontology (GO) and Kyoto Encyclopedia of Genes and Genomes (KEGG) on coDEGs using the “clusterProfiler” package in R [19].

### 2.6. Immune Cell Infiltration and Immune Function Analysis

We used the “GSVA” package in R to quantify immune cell infiltration and immune function in the TCGA-UCEC cohort using the single-sample Gene Set Enrichment Analysis (ssGSEA) algorithm [20]. Subsequently, we analyzed the correlation between different immune cell infiltrations and different immune functions. We also explored the association between risk scores and immune infiltration, as well as the correlation between coDEGs and immune infiltration.

### 2.7. Drug Sensitivity Analysis

We used the GSCA online tool (http://bioinfo.life.hust.edu.cn/GSCA/#/drug) to explore the relationship between the expression of coDEGs and the sensitivity of antitumor drugs, including two major modules: The Cancer Therapeutics Response Portal (CTRP) drug sensitivity analysis and the Genomics of Drug Sensitivity in Cancer (GDSC) drug sensitivity analysis [21].

### 2.8. Statistical Analysis

For statistical studies, R software (version: 4.1.2) was used. To compare the data between two groups, Student's *t*-test was utilized. One-way ANOVA was employed to analyze multiple groups followed by Tukey's post hoc test. All tests were two-sided and statistical significance was defined as *p* < 0.05. The “ggplot2” package in R was used for plotting.

## 3. Results

### 3.1. Construction of a Prognostic Model from m7G-Related miRNAs

**Figure 2:**
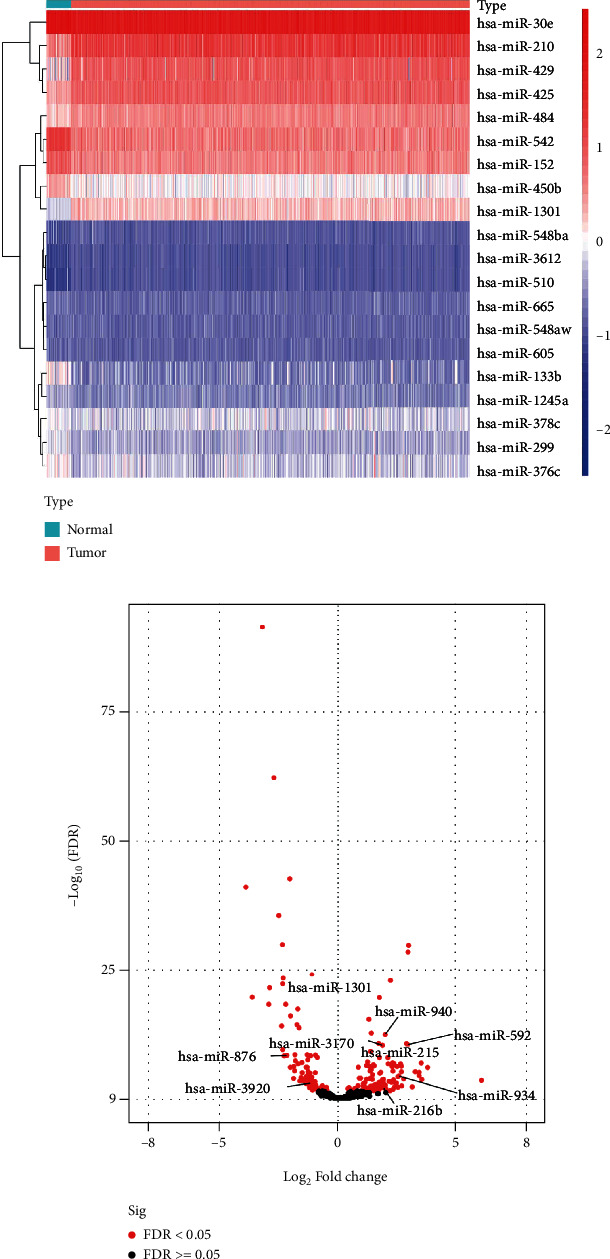
The differentially expressed miRNAs in TCGA-UCEC were identified in the tumor group compared with the normal group. (a) Heatmap of top 20 DEmiRNAs. (b) Volcano plot of all DEmiRNAs.

**Figure 3:**
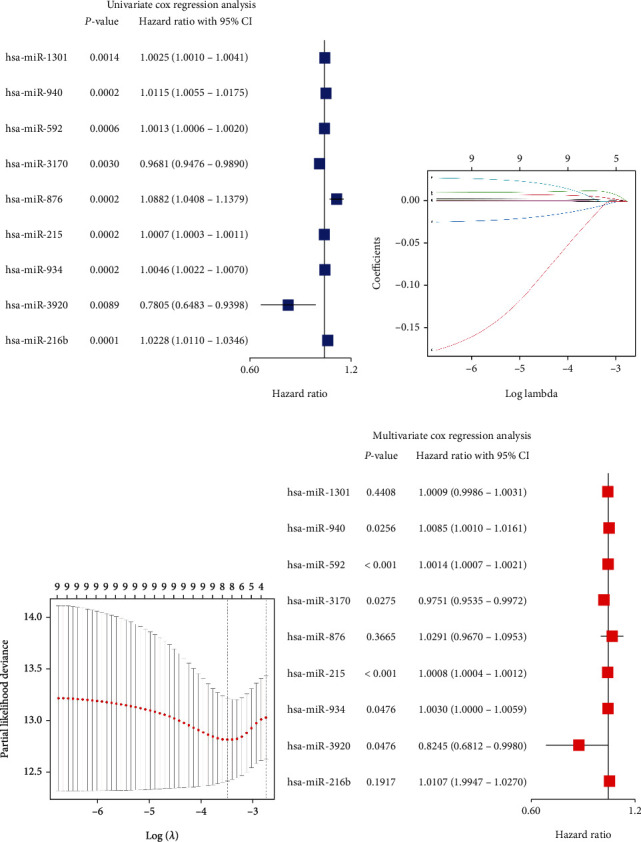
Identification of SDEmiRNAs by univariate and multivariate Cox analyses. (a) The forest plot showed the results of the univariate analysis. (b) The profile of coefficients in the model was plotted at different levels of penalization using the log(lambda) sequence. (c) Cross-validated error tenfold (the first vertical line represents the minimum error, while the second shows the error within a standard deviation). (d) The forest plot showed the results of the multivariate analysis.

**Figure 4:**
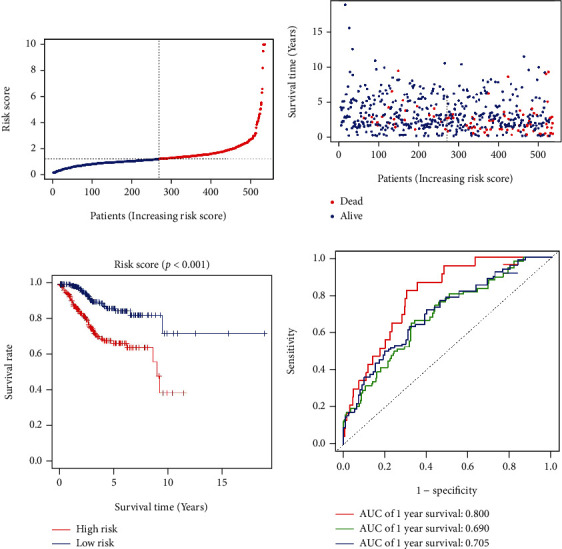
Nine m7G-related SDEmiRNAs constructed a survival prediction model for UCEC patients. (a) Distribution and median values of risk scores. (b) Distributions of overall survival and risk scores. (c) Kaplan–Meier curves showed survival for patients in the high-risk and low-risk groups. (d) The ROC curves showed the performance of the model to predict the 1-, 3-, and 5-year survival of UCEC patients.

We obtained 33 specimens of normal tissue and 546 specimens of tumor tissue from the TCGA-UCEC cohort. The differentially expressed miRNA analysis results of the tumor group compared to the normal group showed 91 upregulated DEmiRNAs and 61 downregulated DEmiRNAs (Supplementary Table S3). In the heatmap (top 20 DEmiRNAs), the differences in the expression of DEmiRNAs between the normal group and the tumor group are shown (Figure 2). Next, we identified 9 SDEmiRNAs that were significantly associated with the overall survival in UCEC patients by the univariate Cox regression analysis and LASSO regression analysis (Figures 3(a)–3(c)). Hence, we computed risk scores using the 9 SDEmiRNAs described above and constructed a multifactorial Cox regression model (Figure 3(d)). Formula used to calculate risk score: 0.00088 × expression level of hsa − miR − 1301 + 0.00850 × expression level of hsa − miR − 940 + 0.00140 × expression level of hsa − miR − 592 − 0.02522 × expression level of hsa − miR − 3170 + 0.02871 × expression level of hsa − miR − 876 + 0.00080 × expression level of hsa − miR − 215 + 0.00296 × expression level of hsa − miR − 934 − 0.19298 × expression level of hsa − miR − 3920 + 0.01063 × expression level of hsa − miR − 216b. The median risk score was used to differentiate between the high- and low-risk groups (Figure 4(a)). It is clear from Figures 4(b) and 4(c) that the high-risk group had a significantly higher mortality rate than the low-risk group and the low-risk group had better survival. The sensitivity and specificity of the survival risk assessment model were evaluated by the ROC curve, and the areas under the curve of the risk score model were 0.800, 0.690, and 0.705 for the prediction of 1-year, 3-year, and 5-year survival probability, respectively (Figure 4(d)).

### 3.2. Predictive Value of Risk Scoring Models Combined with Clinical Parameters

**Figure 5:**
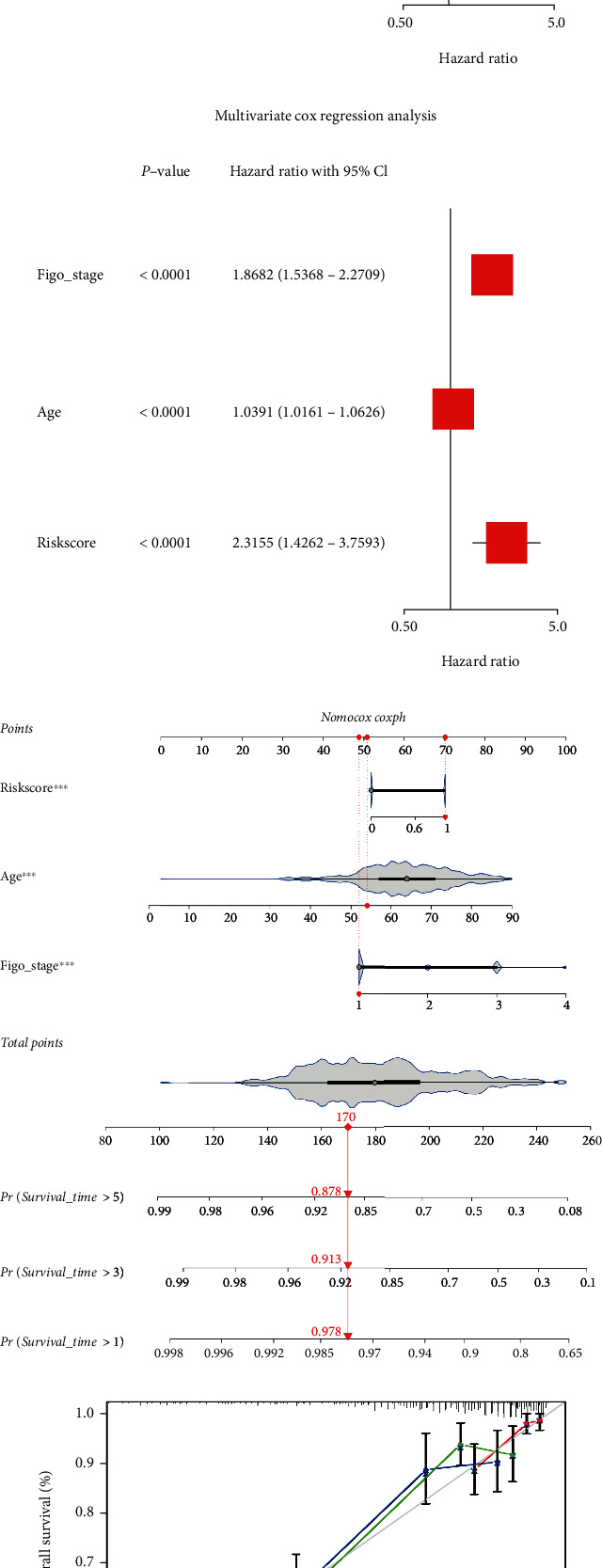
Construction of a composite model of risk scores combined with clinical parameters. (a, b) The risk score model was an independent factor affecting the survival of UCEC patients in univariate and multivariate Cox analyses. (c) Nomogram for model constructed from risk scores combined with clinical parameters (age and FIGO stage). (d) Calibration curve for nomogram. ^∗∗^*p* < 0.01, ^∗∗∗^*p* < 0.001.

We incorporated clinical parameters (age and FIGO stage) and risk score into univariate and multivariate Cox regression analyses models and found that the risk score was an independent factor affecting the survival of UCEC patients (Figures 5(a) and 5(b)). In addition, we drew a nomogram for risk assessment combined with clinical parameters to predict the 1-, 3-, and 5-year survival rates of UCEC patients (Figure 5(c)), and the calibration curve showed that the nomogram had good predictive performance (Figure 5(d)).

### 3.3. Association of SDEmiRNAs with Survival in UCEC Patients

**Figure 6:**
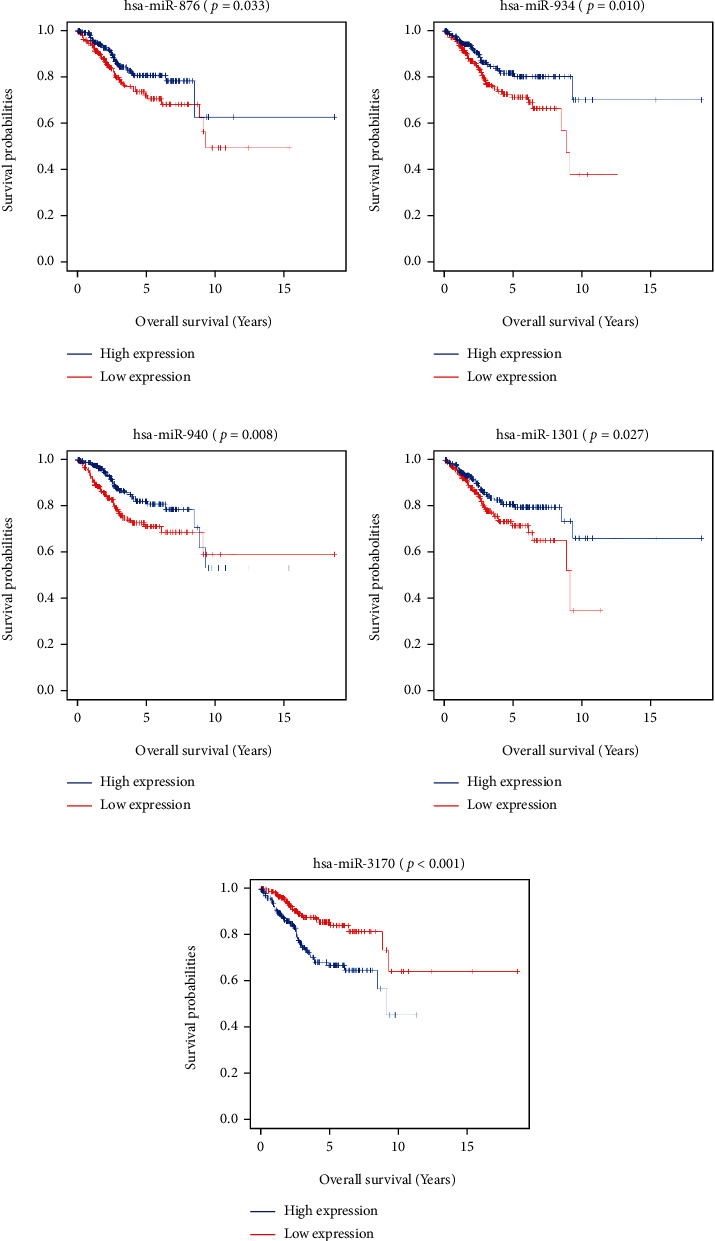
Survival analysis of SDEmiRNA expression in TCGA-UCEC. Kaplan–Meier curves showed survival analysis of high versus low expression of hsa-miR-876 (a), hsa-miR-934 (b), hsa-miR-940 (c), hsa-miR-1301 (d), and hsa-miR-3170 (e) in UCEC patients.

Survival analysis was used to explore the correlation between the expression of SDEmiRNAs and the survival of UCEC patients. In Figure 6, the Kaplan–Meier curves showed that UCEC patients with high expression of hsa-miR-876, hsa-miR-934, hsa-miR-940, and hsa-miR-1301 had a significantly lower survival rate than those with low expression, and the survival rate of UCEC patients with high expression of hsa-miR-3170 was significantly increased compared with those with low expression.

### 3.4. Functional and Pathway Enrichment Analyses

**Figure 7:**
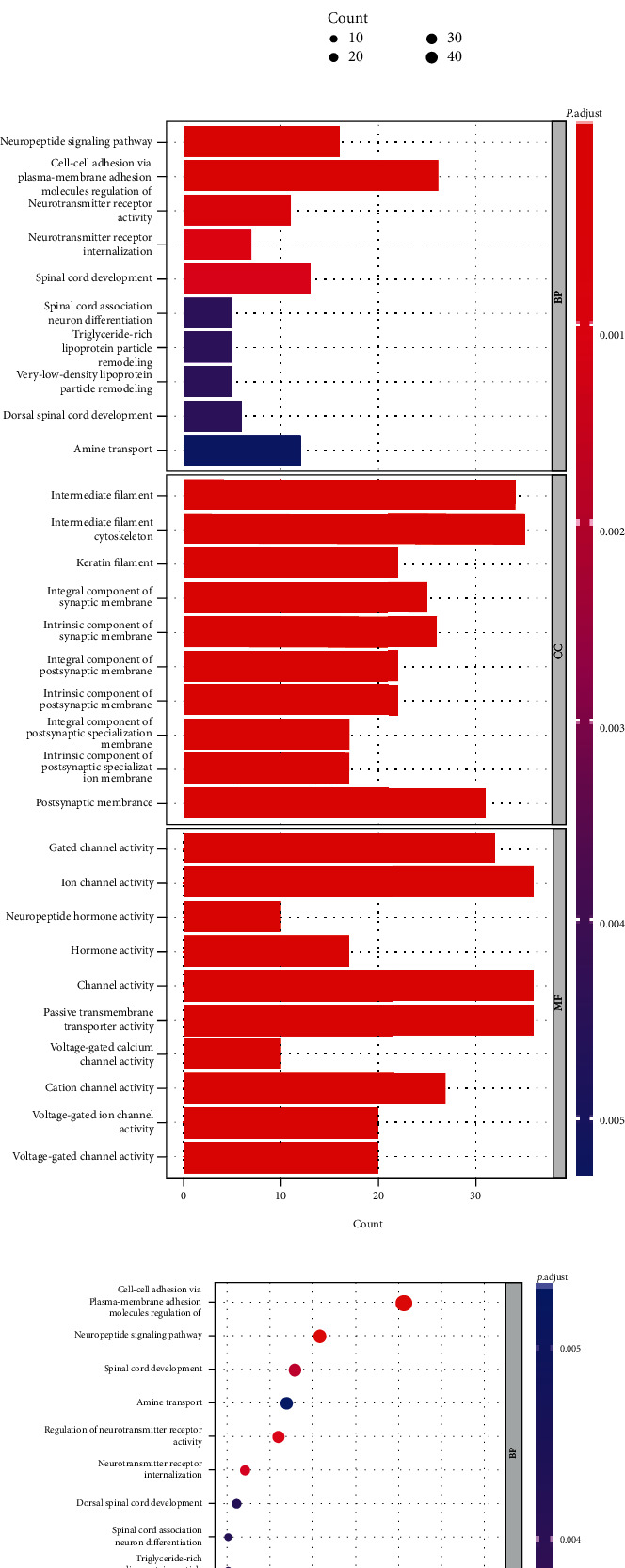
Identification and functional enrichment analysis of coDEGs. (a) The Venn diagram showed that a total of 1516 coDEGs were obtained. (b, c) Bar and dot plots showed the results of KEGG enrichment analysis of coDEGs. (d, e) Bar and dot plots showed the results of GO enrichment analysis of coDEGs.

Using difference analysis, we obtained 4054 RDEGs and 4390 IDEGs and obtained 1516 coDEGs by taking the intersection (Figure 7(a)). We performed enrichment analysis of the obtained coDEGs with KEGG, and the results showed that the top three pathways were neuroactive ligand-receptor interaction, cAMP signaling pathway, and oxytocin signaling pathway (Figures 7(b) and 7(c)). An overview of a given gene's function is provided by GO, which includes molecular function (MF), cellular components (CC), and biological process (BP). In the GO enrichment analysis, BP module's results were especially enriched in cell-cell adhesion via plasma-membrane adhesion molecules, neuropeptide signaling pathway, and spinal cord development; CC module's results were enriched in intermediate filament cytoskeleton, intermediate filament, and postsynaptic membrane; and MF module's results were mainly enriched in ion channel activity, channel activity, and passive transmembrane transporter activity (Figures 7(d) and 7(e)).

### 3.5. Tumor Microenvironment Immune Infiltration

**Figure 8:**
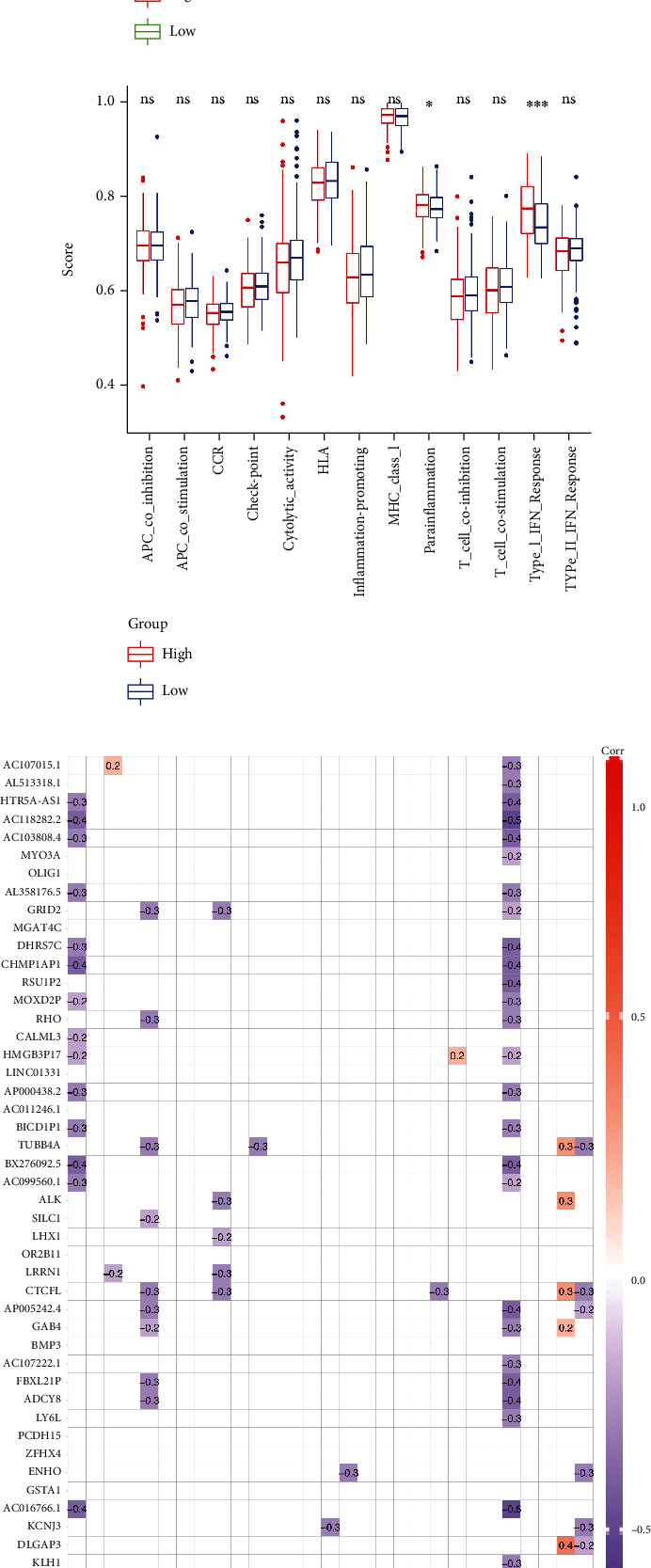
Immune cell infiltration and immune activity in the tumor microenvironment of UCEC and anticancer drug sensitivity analysis of coDEGs. (a) Heatmap of immune cell infiltration and immune activity in the TCGA-UCEC cohort. (b) Correlation analysis between various immune cells. (c) Correlation analysis between various immune functions. (d) Comparison of the degree of infiltration of various immune cells in the high-risk group and the low-risk group. (e) Comparison of the extent of various immune activities in the high-risk group versus the low-risk group. (f) Correlation analysis of coDEG expression with immune cell infiltration and immune activity. (g, h) Anticancer drug sensitivity analysis based on CTRP and GDSC databases for coDEG expression.

The ssGSEA results are represented as a heatmap, and it is likely that different immune cells have different degrees of infiltration in UCEC tumor samples, while at the same time, immune function activities are also different (Figure 8(a)). Then, we examined the correlations between 16 subtypes of immune cells, as well as 13 immune functions (Figures 8(b) and 8(c)). Interestingly, in Figure 8(d), the proportions of CD8+ T cells, DCs, iDCs, neutrophils, and Th2 cells in the high-risk score group were significantly lower than those in the low-risk score group. On the other hand, the proportion of aDCs in the high-risk score group was significantly higher than that in the low-risk group. In addition, among various immune functions, only the degree of parainflammation and type I IFN response was significantly different between the high-risk score group and the low-risk score group (Figure 8(e)). Subsequently, we analyzed the correlation between the expression of coDEGs and 16 different subtypes of immune cells, as well as the correlation between the expression of coDEGs and 13 immune functions. The results showed that the expression of most coDEGs was negatively correlated with the degree of immune cell infiltration and immune functions, except for IGLV3-24 and IGLV9-49 (Figure 8(f)).

### 3.6. Drug Sensitivity Analysis

In order to comprehensively understand the correlation between the expression of coDEGs and the sensitivity of antitumor drugs, we used CTRP and GDSC drug data for mining analysis and the results are shown in Figures 8(g) and 8(h).

## 4. Discussion

Methylation occurs in biological systems via enzymes. The process of methylation can control gene expression, RNA processing, and protein function. In epigenetics, it is considered a key process. The N^7^-methylguanosine (m7G) modification of RNA has recently gained significant attention. m7G modifications affect a variety of RNA molecules, including messenger RNA, ribosomal RNA, microRNA, and transfer RNA, that participate in biological and pathological functions [6]. It is now clear that m7G plays a critical role in the development of human diseases, especially cancers, and aberrant m7G levels are linked to tumorigenesis and progression through the regulation of multiple oncogenes and tumor suppressor genes [22, 23]. Currently, the molecular mechanisms underlying m7G modification in UCEC are not well understood. Therefore, we explored m7G-related miRNA signatures in UCEC and constructed a risk score model for survival prediction of UCEC patients.

In our study, we identified 9 m7G-related miRNAs associated with the survival of UCEC patients by integrated bioinformatics approach, including: hsa-miR-1301, hsa-miR-940, hsa-miR-592, hsa-miR-3170, hsa-miR-876, hsa-miR-215, hsa-miR-934, hsa-miR-3920, and hsa-miR-216b. These SDEmiRNAs were used to construct a risk scoring model for the survival prediction of UCEC patients at 1, 3, and 5 years. Previous studies have shown that these miRNAs are associated with the phenotype of various malignant tumors. According to Wang et al., miR-1301 inhibits tumor cell migration and invasion by regulating the UBE4B-p53 pathway in multiple human cancer cells [24]. In gastric cancer, the miR-1301-3p/KIF23 axis inhibits gastric cancer cell proliferation, migration, and invasion by knocking down circ_0067934 [25]. Guo et al. reported that PVT1 inhibits proliferation and promotes apoptosis in human retinal epithelial cells by binding to microRNA-1301-3p and activating KLF7 [26]. In pancreatic cancer, Zhang et al. reported that miR-1301-3p inhibits epithelial-mesenchymal transition through targeting RhoA [27]. Wang et al. revealed that miR-1301-3p directly binds to METTL3 and regulates hepatocellular carcinoma progression. It has been reported that METTL3 associates with both m7G cap-binding complexes CBP80 and eIF4E [28]. Researchers found that miR-940 increases proliferation and metastasis in endometrial cancer by regulating MRVI1 [29]. Through regulating FOXO3, miR-940 promotes malignant progression of breast cancer [30]. In esophageal squamous cell carcinoma cells, miR-940 inhibits cell proliferation and promotes apoptosis, which may affect the outcome after surgery [31]. By absorbing hsa-mir-940, hsa_circ_0092339 targets C-MYC indirectly and plays an important role in castration-resistant prostate cancer [32]. It is reported that miR-592 enhances medullary thyroid cancer tumorigenesis through cyclin-dependent kinase 8 [33]. MiR-592 suppresses the malignant phenotypes of thyroid cancer through the regulation of lncRNA NEAT1 and downregulation of NOVA1 [34]. A miR-592-mediated activation of mTOR (mammalian target of rapamycin), ERK1/ERK2 signaling, and neuronal differentiation impart group 4 medulloblastoma characteristics [35]. It has been found that lncRNA MEF2C-AS1 inhibits cervical cancer by targeting RSPO1 by suppressing miR-592 [36]. There is no relevant experimental research literature on miR-3170, but one literature incorporates miR-3170 into the prognostic model through bioinformatics analysis and considers it to be a predictor of UCEC [37]. In a glucose-induced tumor microenvironment, the HOXC-AS2/miR-876-5p/HKDC1 axis regulates endometrial cancer progression [38]. By activating the PI3K/AKT signaling pathway, miR-876-5p targets GNG12 and contributes to glioma progression [39]. Through targeting TMED3, miR-876-3p modulates the resistance of gastric cancer cells to cisplatin and their stem-like properties [40]. A study by Liang et al. reported that hsa_circ_0097922 regulates ACTN4 expression via miR-876-3p, thus promoting tamoxifen resistance in breast cancer cells [41]. miR-215 interferes with cell migration and invasion by targeting stearoyl-CoA desaturase in colorectal cancer [42]. Through targeting RB1 and triggering the Wnt/*β*-catenin pathway, miR-215 promotes nasopharyngeal carcinoma progression [43]. By targeting Sox9, miRNA-215-5p inhibits aggressiveness in breast cancer cells [44]. A decrease in miR-215 levels is correlated with an increase in KDM1B levels in enzalutamide-resistant prostate cancer cells that promotes AR-dependent AGR2 transcription and regulates the sensitivity to AR-targeted therapy [45]. In breast cancer, miR-934 regulates PTEN and the epithelial-mesenchymal transition [46]. An increase in the expression of miR-934 has been shown to serve as an independent prognostic factor in lung cancer and contributes to proliferation, migration, and invasion in non-small-cell lung cancer cells [47]. miR-934 derived from colorectal cancer tumors can induce macrophage polarization toward M2 to promote liver metastasis [48]. At present, there is no literature report that miR-3920 is related to tumors, and further research is needed in the future. miR-216b overexpression enhances the activation of PI3K/AKT by partially regulating PXN in gastric cancer cells [49]. Through binding to the 3′-UTR of HDAC8, miR-216b-5p inhibits proliferation and progression in breast cancer cells [50]. Inhibition of osteosarcoma cell proliferation, migration, and invasion is mediated by miRNA 216b targeting Forkhead box M1 [51].

The tumor microenvironment (TME) is a key factor in the occurrence and development of tumors. In particular, various immune cell infiltration and changes in immune activity in the TME affect the biological properties of tumors. Immunotherapy has been known as a promising antitumor regimen in recent years. To propose an appropriate antitumor regimen, it is necessary to first understand the specific conditions of immune cell infiltration and immune activity in the TME. Our findings showed that in UCEC, the high-risk group had a lower degree of immune cell infiltration than the low-risk group. Adaptive immunity is mediated by CD8-positive T cells, a subgroup of MHC class I-restricted cells. These include cytotoxic T cells, which can destroy cancerous or virally infected cells, and CD8-positive suppressor T cells, which suppress specific forms of immunity. Cancer is primarily targeted by CD8+ cytotoxic T lymphocytes (CTLs) [52]. TME-associated immune-related tolerance and immunosuppression causes CTL dysfunction and exhaustion during cancer progression, resulting in adaptive immune resistance [52]. Innate immune cells known as dendritic cells (DCs) infiltrate tumors and present tumor-derived antigens to naïve T cells. Hence, DCs serve as a major therapeutic target for cancer immunotherapy since they play a critical role in priming antitumor immunity [53]. As the most dominant immune cell, neutrophils also play a complex and critical role in cancer. The peripheral blood counts of neutrophils have been found to be elevated in a number of cancer studies. Researchers have demonstrated that the neutrophil-to-lymphocyte ratio can serve as an independent prognostic indicator for cancer patients [54]. Th2 cells have been shown to mediate both pro- and antitumor effects. Studies showed that Th2 cells are indeed involved in mediating antitumor immunity, contrary to the traditional view that type 2 immunity inhibits these responses [55–57]. In addition, we also found differences in parainflammation and type I IFN response between the high- and low-risk groups, which may suggest a potential role in TME.

In addition, we analyzed the correlation between the expression of coDEGs and immune cell infiltration or immune function, and the results showed that the negative correlation was more than the positive correlation. The results of drug sensitivity analysis showed that the expression of coDEGs was significantly correlated with a variety of antitumor drugs, which provided suggestions for the selection of anticancer drugs.

Currently, several studies have reported prognostic prediction models for UCEC. Ni et al. constructed a prognostic model composed of 12 miRNAs for UCEC, which can accurately predict the survival of UCEC [58]. Using redox-related genes, Geng et al. developed a clinical outcome prognostic model [59]. Compared with previous studies, our study started with m7G-related miRNAs and included only 9 miRNAs, which had better accuracy in 1-year prognosis prediction. And the downstream genes regulated by miRNAs included in the model were related to immune cell infiltration in the tumor microenvironment, providing a basis for antitumor drug selection.

## 5. Conclusion

In conclusion, we constructed a model of nine m7G-related miRNAs for predicting survival in UCEC patients. The TCGA-UCEC samples were divided into high- and low-risk groups based on the risk model, and the immune cell infiltration and immune function of each group were explored. Moreover, we performed functional analysis of coDEGs and investigated the relationship between their expression and immune activity, as well as their correlation with anticancer drug susceptibility.

## Data Availability

The data included in the present study are available from the corresponding author upon reasonable request.

## Supplementary Materials

Supplementary 1 Supplementary Table S1: the twenty-eight m7G-related genes list.

Supplementary 2 Supplementary Table S2: the m7G-related miRNAs list.

Supplementary 3 Supplementary Table S3: the differentially expressed miRNA analysis results of tumor group compared to normal group in the TCGA-UCEC cohort.
