# Supplementary material for: Identification of m7G Methylation-Related miRNA Signature Associated with Survival and Immune Microenvironment Regulation in Uterine Corpus Endometrial Carcinoma
Source: Biomed Res Int. 2022 Nov 23;2022:8776678. doi: 10.1155/2022/8776678 (PMC9713471; doi:10.1155/2022/8776678)
Supplement: Supplementary 1 — Supplementary Table S1: the twenty-eight m7G-related genes list. [file 8776678.f1.pdf]

Gene
METTL1
WDR4
NSUN2
DCP2
DCPS
NUDT10
NUDT11
NUDT16
NUDT3
NUDT4
AGO2
CYFIP1
EIF4E
EIF4E1B
EIF4E2
EIF4E3
GEMIN5
LARP1
NCBP1
NCBP2
C17orf85
EIF3D
EIF4A1
EIF4G3
IFIT5
LSM1
NCBP2L
SNUPN
