# Supplementary material for: Identification of m7G Methylation-Related miRNA Signature Associated with Survival and Immune Microenvironment Regulation in Uterine Corpus Endometrial Carcinoma
Source: Biomed Res Int. 2022 Nov 23;2022:8776678. doi: 10.1155/2022/8776678 (PMC9713471; doi:10.1155/2022/8776678)
Supplement: Supplementary 3 — Supplementary Table S3: the differentially expressed miRNA analysis results of tumor group compared to normal group in the TCGA-UCEC cohort. [file 8776678.f3.pdf]

ID	logFC	logCPM	P-Value	FDR
hsa-miR-54	-3.2223697	13.2256858	3.96E-95	2.56E-92
hsa-miR-45	-2.7329807	9.17786596	1.57E-65	5.06E-63
hsa-miR-15	-2.0578723	12.2010282	9.19E-46	1.98E-43
hsa-miR-13	-3.9301519	7.00728354	4.83E-44	7.80E-42
hsa-miR-12	-2.5262043	5.85078418	2.23E-38	2.88E-36
hsa-miR-29	-2.3746602	7.0241048	1.16E-32	1.25E-30
hsa-miR-42	3.00584608	13.7788963	1.85E-32	1.71E-30
hsa-miR-21	2.9794948	15.7147489	4.24E-31	3.43E-29
hsa-miR-30	-1.1163822	18.8899013	1.44E-26	1.03E-24
hsa-miR-54	-2.341593	4.61005985	9.90E-26	6.39E-24
hsa-miR-42	2.23048724	13.6235057	3.14E-25	1.84E-23
hsa-miR-60	-2.3638562	4.49219845	1.57E-24	8.44E-23
hsa-miR-36	-2.9168333	3.89876286	9.61E-24	4.78E-22
hsa-miR-51	-3.6554421	3.92121015	7.44E-22	3.44E-20
hsa-miR-13	1.7584676	10.0717731	9.14E-22	3.94E-20
hsa-miR-54	-2.952467	4.29424864	2.02E-20	7.97E-19
hsa-miR-66	-2.2323913	4.75822679	2.10E-20	7.97E-19
hsa-miR-37	-1.7139627	7.85538264	1.77E-19	6.37E-18
hsa-miR-37	-2.0271443	8.08806189	4.16E-18	1.42E-16
hsa-miR-48	1.31342154	11.8250272	1.94E-17	6.27E-16
hsa-miR-54	-1.7501347	4.45768576	2.25E-16	6.93E-15
hsa-miR-67	-2.4079371	3.91455953	4.33E-16	1.27E-14
hsa-miR-63	-1.6598565	4.78142592	1.01E-15	2.84E-14
hsa-miR-19	1.41465811	8.2946523	1.10E-14	2.95E-13
hsa-miR-94	2.00867368	7.87935755	2.21E-14	5.71E-13
hsa-miR-12	8.31784669	13.2006358	5.41E-14	1.34E-12
hsa-miR-12	1.72789166	9.50147231	1.25E-12	2.98E-11
hsa-miR-63	2.91637784	4.7876372	1.38E-12	3.17E-11
hsa-miR-59	2.95790092	8.92133847	2.35E-12	5.24E-11
hsa-miR-31	1.89461656	7.03668293	3.22E-12	6.94E-11
hsa-miR-38	-2.3643845	6.37446756	2.29E-11	4.77E-10
hsa-miR-36	1.30669118	7.70927836	7.66E-11	1.55E-09
hsa-miR-13	-1.3185131	12.8627073	2.35E-10	4.50E-09
hsa-miR-32	-1.8377346	4.43480245	2.37E-10	4.50E-09
hsa-miR-87	-2.184068	4.92223118	3.32E-10	5.95E-09
hsa-miR-14	-1.1719198	8.72215804	4.00E-10	6.98E-09
hsa-miR-79	-2.3034362	4.17241686	4.33E-10	7.37E-09
hsa-miR-65	2.11850798	9.11694561	1.16E-09	1.87E-08
hsa-miR-77	1.75412083	5.59864936	1.22E-09	1.93E-08
hsa-miR-88	-1.294648	8.02835047	2.82E-09	4.33E-08
hsa-miR-44	-1.817875	4.03529808	4.48E-09	6.74E-08
hsa-miR-94	1.25646003	8.33104682	7.32E-09	1.07E-07
hsa-miR-65	-1.5407275	6.38181256	1.00E-08	1.44E-07
hsa-miR-55	2.31446479	4.67679405	1.20E-08	1.68E-07
hsa-miR-12	3.53580552	4.86793194	1.29E-08	1.78E-07

hsa-miR-21	2.37248271	8.73983445	1.57E-08	2.12E-07
hsa-miR-56	2.13542808	5.01340745	1.96E-08	2.54E-07
hsa-miR-12	2.66071826	4.58780225	1.99E-08	2.54E-07
hsa-miR-46	-1.6979837	3.84357082	2.00E-08	2.54E-07
hsa-miR-76	1.50158579	6.87743852	3.17E-08	3.93E-07
hsa-miR-77	1.21030488	6.62747071	4.60E-08	5.60E-07
hsa-miR-36	1.53931605	6.07430108	5.16E-08	6.17E-07
hsa-miR-47	1.31347076	5.38615366	5.81E-08	6.82E-07
hsa-miR-50	2.69580425	4.39118784	6.34E-08	7.32E-07
hsa-miR-23	-1.8903712	4.70260896	7.54E-08	8.48E-07
hsa-miR-68	2.5078802	4.37733899	7.70E-08	8.48E-07
hsa-miR-31	2.30054843	4.54864479	7.75E-08	8.48E-07
hsa-miR-88	-1.3643152	8.56027423	1.10E-07	1.16E-06
hsa-miR-56	-2.036188	4.04656482	1.18E-07	1.22E-06
hsa-miR-37	-1.3165582	8.05713912	1.19E-07	1.22E-06
hsa-miR-89	3.81163395	11.3803054	1.26E-07	1.27E-06
hsa-miR-13	1.41184744	6.08092403	3.12E-07	3.10E-06
hsa-miR-50	-1.8527185	4.50064367	5.65E-07	5.53E-06
hsa-miR-36	1.36442019	6.20616396	6.15E-07	5.93E-06
hsa-miR-48	2.33371183	5.03841329	6.47E-07	6.14E-06
hsa-miR-46	-1.2777569	5.19822821	8.31E-07	7.78E-06
hsa-miR-44	3.28361894	11.9102712	9.09E-07	8.32E-06
hsa-miR-77	2.69823734	5.80557406	9.15E-07	8.32E-06
hsa-miR-57	1.81853587	9.94612111	1.19E-06	1.07E-05
hsa-miR-52	3.44721639	4.47667581	1.21E-06	1.07E-05
hsa-miR-31	-1.5291504	4.07416116	1.47E-06	1.26E-05
hsa-miR-45	-1.1986534	13.6391083	1.81E-06	1.54E-05
hsa-miR-45	1.77925212	4.53953331	1.96E-06	1.64E-05
hsa-miR-49	-1.2796938	5.99338602	3.62E-06	3.00E-05
hsa-miR-43	1.46765968	7.92389665	3.82E-06	3.12E-05
hsa-miR-12	-1.2270719	4.55570396	4.09E-06	3.30E-05
hsa-miR-31	3.44510354	5.22616612	6.15E-06	4.90E-05
hsa-miR-93	2.58264477	7.07424869	7.31E-06	5.76E-05
hsa-miR-65	-1.2591941	5.07519444	8.23E-06	6.40E-05
hsa-miR-31	-1.3574009	4.17338717	9.19E-06	7.07E-05
hsa-miR-12	2.54659091	8.76447092	1.02E-05	7.77E-05
hsa-miR-44	-1.117934	5.88202532	1.28E-05	9.63E-05
hsa-miR-12	-1.5753583	5.56296582	1.62E-05	0.00012061
hsa-miR-54	-1.233083	6.00626479	2.25E-05	0.0001617
hsa-miR-44	1.36632442	6.32081332	2.35E-05	0.00016653
hsa-miR-12	-1.8945949	4.44990938	2.52E-05	0.00017687
hsa-miR-65	1.35323429	4.72835948	2.84E-05	0.00019717
hsa-miR-12	2.79108522	4.24681412	3.07E-05	0.00021115
hsa-miR-95	1.21503153	7.49661559	3.54E-05	0.00024096
hsa-miR-46	1.9363677	4.09293522	3.72E-05	0.00025059
hsa-miR-31	1.45675965	4.45027959	3.97E-05	0.00026472

hsa-miR-89	3.5617066	5.28455217	4.15E-05	0.0002737
hsa-miR-49	-1.1404339	6.32143432	4.38E-05	0.00028578
hsa-miR-46	1.88334722	4.55984179	5.00E-05	0.00032287
hsa-miR-57	2.35811295	4.20524093	5.17E-05	0.00033096
hsa-miR-12	6.11928215	8.07332934	5.69E-05	0.00036043
hsa-miR-42	-1.4027705	3.90207175	7.43E-05	0.00046617
hsa-miR-45	-1.4700842	4.73626138	7.91E-05	0.00048799
hsa-miR-12	-1.1965987	4.11076439	7.93E-05	0.00048799
hsa-miR-12	-1.4079808	3.85006168	8.97E-05	0.00054663
hsa-miR-77	-1.6249573	4.09024479	9.45E-05	0.00057024
hsa-miR-44	2.17182481	4.68552271	0.000108598	0.00064958
hsa-miR-52	2.27499056	4.59957367	0.000159126	0.00093451
hsa-miR-36	1.74055652	4.33802089	0.000175833	0.00102332
hsa-miR-12	1.14433438	7.18259209	0.000198014	0.00114212
hsa-miR-31	-1.146639	3.84246416	0.000225299	0.00128799
hsa-miR-66	2.43688831	4.21649255	0.00022837	0.0012941
hsa-miR-36	2.48275075	4.15739231	0.00024275	0.00136362
hsa-miR-79	1.93792288	4.69300404	0.000247444	0.00137801
hsa-miR-39	-1.163571	4.70428849	0.000257848	0.00142367
hsa-miR-57	-1.1933075	3.93383392	0.000264199	0.00144638
hsa-miR-12	1.07764738	4.67797771	0.000320633	0.00174058
hsa-miR-47	-1.3487048	3.92284482	0.000359031	0.00193278
hsa-miR-36	1.57942582	4.98391961	0.000493345	0.00263389
hsa-miR-47	2.01248007	4.23706325	0.00070191	0.00368645
hsa-miR-41	-1.0726385	8.59707374	0.00072443	0.00374385
hsa-miR-36	-1.1785576	3.86614099	0.000996336	0.0049894
hsa-miR-22	1.489823	4.13965274	0.001029738	0.00509419
hsa-miR-44	1.06662487	4.90989035	0.001033033	0.00509419
hsa-miR-76	2.71112102	8.95479279	0.001043601	0.00510732
hsa-miR-44	1.78507315	4.50432	0.001052151	0.00511045
hsa-miR-37	1.3866074	18.5143287	0.00121529	0.00585879
hsa-miR-94	1.54046784	4.10203427	0.001242238	0.00594434
hsa-miR-31	1.67814688	4.13652824	0.001436906	0.00672639
hsa-miR-39	3.15753771	4.76218429	0.001647958	0.00765885
hsa-miR-54	-1.008737	3.84159535	0.001778084	0.00820459
hsa-miR-89	2.40139736	4.52769181	0.001836403	0.00841359
hsa-miR-89	2.54901074	5.91938868	0.001936827	0.0088112
hsa-miR-60	1.45564283	4.4671845	0.002014489	0.00910042
hsa-miR-80	1.09438739	4.47011646	0.00219286	0.00976647
hsa-miR-12	1.16631965	7.58211594	0.002207283	0.00976647
hsa-miR-39	-1.220705	4.02845876	0.002518256	0.01091807
hsa-miR-21	1.93745509	4.90556233	0.002891489	0.01237021
hsa-miR-50	-1.0713402	3.85475776	0.002972739	0.01263414
hsa-miR-31	1.24333806	4.17235006	0.003061741	0.01292735
hsa-miR-12	1.66480135	3.98261531	0.003280765	0.01367338
hsa-miR-47	2.02760535	4.9004993	0.003664204	0.01498149

hsa-miR-56	-1.0415613	3.97616295	0.004308213	0.01707427
hsa-miR-54	2.3383022	4.82531178	0.00591352	0.02301285
hsa-miR-31	1.16819634	3.95154425	0.006226615	0.02408619
hsa-miR-58	1.62329305	4.3881095	0.006504396	0.0247167
hsa-miR-44	1.75289029	4.05261176	0.006629998	0.02504666
hsa-miR-45	1.39011593	4.24009806	0.00720973	0.02676716
hsa-miR-88	1.22009401	9.64034639	0.007801186	0.0286339
hsa-miR-46	1.13896133	4.19155329	0.008144875	0.02955949
hsa-miR-39	1.32728339	4.1802428	0.008487197	0.03045961
hsa-miR-19	1.23302408	4.11115584	0.008853324	0.03142443
hsa-miR-37	-1.0927573	5.17218918	0.009246511	0.03246329
hsa-miR-39	1.37217469	3.93978019	0.011413794	0.03985573
hsa-miR-31	1.51118899	6.58705055	0.011663673	0.04050932
hsa-miR-59	2.21289037	5.64352323	0.012559406	0.04338704
hsa-miR-18	1.62102319	10.3252103	0.013687439	0.04703237
