# Supplementary material for: Identification of m7G Methylation-Related miRNA Signature Associated with Survival and Immune Microenvironment Regulation in Uterine Corpus Endometrial Carcinoma
Source: Biomed Res Int. 2022 Nov 23;2022:8776678. doi: 10.1155/2022/8776678 (PMC9713471; doi:10.1155/2022/8776678)
Supplement: Supplementary 2 — Supplementary Table S2: the m7G-related miRNAs list. [file 8776678.f2.pdf]

Gene	miRNA
AGO2	hsa-miR-6715b-3p
AGO2	hsa-miR-3667-5p
AGO2	hsa-miR-7075-3p
AGO2	hsa-miR-7666-3p
AGO2	hsa-miR-7686-3p
AGO2	hsa-miR-6505-3p
AGO2	hsa-miR-291
AGO2	hsa-miR-1777
AGO2	hsa-miR-2369
AGO2	hsa-miR-548ac
AGO2	hsa-miR-205-5p
AGO2	hsa-miR-6980-5p
AGO2	hsa-miR-510-5p
AGO2	hsa-miR-667-3p
AGO2	hsa-miR-487b-5p
AGO2	hsa-miR-7276-5p
AGO2	hsa-miR-107-3p
AGO2	hsa-miR-7033-5p
AGO2	hsa-miR-5001-3p
AGO2	hsa-miR-1704
AGO2	hsa-miR-4277
AGO2	hsa-miR-4275
AGO2	hsa-miR-7069-3p
AGO2	hsa-miR-2352
AGO2	hsa-miR-2370-5p
AGO2	hsa-miR-3596d
AGO2	hsa-miR-7263-3p
AGO2	hsa-miR-460-5p
AGO2	hsa-miR-30ad-3p
AGO2	hsa-miR-6905-5p
AGO2	hsa-miR-6845-5p
AGO2	hsa-miR-17-3p
AGO2	hsa-miR-573
AGO2	hsa-miR-23-3p
AGO2	hsa-miR-6356
AGO2	hsa-miR-6777-3p
AGO2	hsa-miR-153
AGO2	hsa-miR-7468-3p
AGO2	hsa-miR-7457-5p
AGO2	hsa-miR-4472
AGO2	hsa-miR-8097
AGO2	hsa-miR-939-5p
AGO2	hsa-miR-4732-3p
AGO2	hsa-miR-467-3p
AGO2	hsa-miR-7285-5p

AGO2	hsa-miR-1906
AGO2	hsa-miR-6974-3p
AGO2	hsa-miR-290a-5p
AGO2	hsa-miR-6506-5p
AGO2	hsa-miR-1273h-5p
AGO2	hsa-miR-7264-5p
AGO2	hsa-miR-7398k-5p
AGO2	hsa-miR-371-3p
AGO2	hsa-miR-7110-3p
AGO2	hsa-miR-6756-5p
AGO2	hsa-miR-136-3p
AGO2	hsa-miR-7050-5p
AGO2	hsa-miR-6672-3p
AGO2	hsa-miR-1589
AGO2	hsa-miR-4426
AGO2	hsa-miR-7076-5p
AGO2	hsa-miR-4531
AGO2	hsa-miR-1550-3p
AGO2	hsa-miR-4632-3p
AGO2	hsa-miR-7006-5p
AGO2	hsa-miR-6517
AGO2	hsa-miR-714
AGO2	hsa-miR-5125-5p
AGO2	hsa-miR-599
AGO2	hsa-miR-5591-5p
AGO2	hsa-miR-3075
AGO2	hsa-miR-7063-3p
AGO2	hsa-miR-5787
AGO2	hsa-miR-3692-5p
AGO2	hsa-miR-7386b-3p
AGO2	hsa-miR-6909-3p
AGO2	hsa-miR-889-3p
AGO2	hsa-miR-1297
AGO2	hsa-miR-7089-3p
AGO2	hsa-miR-1942
AGO2	hsa-miR-8908b
AGO2	hsa-miR-642b-5p
AGO2	hsa-miR-324-3p
AGO2	hsa-miR-6744-3p
AGO2	hsa-miR-4469
AGO2	hsa-miR-2278
AGO2	hsa-miR-16-1-3p
AGO2	hsa-miR-7085-5p
AGO2	hsa-miR-3622a-3p
AGO2	hsa-miR-19a-5p
AGO2	hsa-miR-7321-5p

AGO2	hsa-miR-7390-3p
AGO2	hsa-miR-873-3p
AGO2	hsa-miR-1772-5p
AGO2	hsa-miR-4668-5p
AGO2	hsa-miR-153-5p
AGO2	hsa-miR-5585-5p
AGO2	hsa-miR-495-3p
AGO2	hsa-miR-4438
AGO2	hsa-miR-6847-5p
AGO2	hsa-miR-7183-3p
AGO2	hsa-miR-1200
AGO2	hsa-miR-7220-5p
AGO2	hsa-miR-7064-5p
AGO2	hsa-miR-219a-5p
AGO2	hsa-miR-2284l
AGO2	hsa-miR-2430
AGO2	hsa-miR-3594-5p
AGO2	hsa-miR-4667-5p
AGO2	hsa-miR-540-5p
AGO2	hsa-miR-7397-3p
AGO2	hsa-miR-4264
AGO2	hsa-let-7g-3p
AGO2	hsa-miR-4632-5p
AGO2	hsa-miR-6764-5p
AGO2	hsa-miR-545-5p
AGO2	hsa-miR-880-5p
AGO2	hsa-miR-365a-5p
AGO2	hsa-miR-190b-3p
AGO2	hsa-miR-31-5p
AGO2	hsa-miR-218-3p
AGO2	hsa-miR-6733-5p
AGO2	hsa-miR-2052
AGO2	hsa-miR-641
AGO2	hsa-miR-6895-5p
AGO2	hsa-miR-1271-3p
AGO2	hsa-miR-2424
AGO2	hsa-miR-1230
AGO2	hsa-miR-881-5p
AGO2	hsa-miR-140-5p
AGO2	hsa-miR-6965-5p
AGO2	hsa-miR-140-3p.1
AGO2	hsa-miR-4530
AGO2	hsa-miR-6354
AGO2	hsa-miR-3080-5p
AGO2	hsa-miR-4652-5p
AGO2	hsa-miR-1815

AGO2	hsa-miR-142-3p.1
AGO2	hsa-miR-9768-5p
AGO2	hsa-miR-6873-3p
AGO2	hsa-miR-6960-5p
AGO2	hsa-miR-3070-2-3p
AGO2	hsa-miR-8061
AGO2	hsa-miR-758-3p
AGO2	hsa-miR-2472
AGO2	hsa-miR-6332
AGO2	hsa-miR-525-5p
AGO2	hsa-miR-34-5p
AGO2	hsa-miR-5699-3p
AGO2	hsa-miR-942-3p
AGO2	hsa-miR-4523
AGO2	hsa-miR-1650
AGO2	hsa-miR-7664-5p
AGO2	hsa-miR-1184
AGO2	hsa-miR-7169-5p
AGO2	hsa-miR-1324
AGO2	hsa-miR-292a-5p
AGO2	hsa-miR-7156-5p
AGO2	hsa-miR-7861
AGO2	hsa-miR-609
AGO2	hsa-miR-513
AGO2	hsa-miR-1662
AGO2	hsa-miR-7649-3p
AGO2	hsa-miR-151-3p
AGO2	hsa-miR-3473
AGO2	hsa-miR-1736-5p
AGO2	hsa-miR-580-5p
AGO2	hsa-miR-7232-3p
AGO2	hsa-miR-509-3-5p
AGO2	hsa-miR-7386l-5p
AGO2	hsa-miR-449c-5p
AGO2	hsa-miR-505-3p
AGO2	hsa-miR-5617-5p
AGO2	hsa-miR-6679-5p
AGO2	hsa-miR-181a-5p
AGO2	hsa-miR-4265
AGO2	hsa-miR-6947-5p
AGO2	hsa-miR-548j-5p
AGO2	hsa-miR-577
AGO2	hsa-miR-6973b-3p
AGO2	hsa-miR-337-3p
AGO2	hsa-miR-7578
AGO2	hsa-miR-6985-5p

AGO2	hsa-miR-7275-3p
AGO2	hsa-miR-556-3p
AGO2	hsa-miR-6980-3p
AGO2	hsa-miR-6598-5p
AGO2	hsa-miR-650
AGO2	hsa-miR-1759-3p
AGO2	hsa-miR-1226-3p
AGO2	hsa-miR-6922-3p
AGO2	hsa-miR-1207-3p
AGO2	hsa-miR-373-3p
AGO2	hsa-miR-6850-3p
AGO2	hsa-miR-6885-5p
AGO2	hsa-miR-200a-3p
AGO2	hsa-miR-17-5p
AGO2	hsa-miR-4688
AGO2	hsa-miR-618
AGO2	hsa-miR-6628-3p
AGO2	hsa-miR-7176-5p
AGO2	hsa-miR-4680-3p
AGO2	hsa-miR-520b
AGO2	hsa-miR-3173-5p
AGO2	hsa-miR-4289
AGO2	hsa-miR-2415-3p
AGO2	hsa-miR-331-3p
AGO2	hsa-miR-1941-3p
AGO2	hsa-miR-8801
AGO2	hsa-miR-4725-5p
AGO2	hsa-miR-1743
AGO2	hsa-miR-4738-5p
AGO2	hsa-miR-2345
AGO2	hsa-miR-96-5p
AGO2	hsa-miR-548ak
AGO2	hsa-miR-483-5p
AGO2	hsa-miR-518c-5p
AGO2	hsa-miR-7000-3p
AGO2	hsa-miR-202-5p
AGO2	hsa-miR-1788-3p
AGO2	hsa-miR-6128
AGO2	hsa-miR-2322-5p
AGO2	hsa-miR-696
AGO2	hsa-miR-6950-3p
AGO2	hsa-miR-344i
AGO2	hsa-miR-7463-5p
AGO2	hsa-miR-7662-5p
AGO2	hsa-miR-7658-3p
AGO2	hsa-miR-4659b-3p

AGO2	hsa-miR-7043-5p
AGO2	hsa-miR-548o-5p
AGO2	hsa-miR-4633-3p
AGO2	hsa-miR-6968-3p
AGO2	hsa-miR-872-5p
AGO2	hsa-miR-382-5p
AGO2	hsa-miR-520-3p
AGO2	hsa-miR-196a-1-3p
AGO2	hsa-miR-380-3p
AGO2	hsa-miR-136-5p
AGO2	hsa-miR-344g-3p
AGO2	hsa-miR-6129
AGO2	hsa-miR-7041-3p
AGO2	hsa-miR-1585
AGO2	hsa-miR-208b-3p
AGO2	hsa-miR-466h-3p
AGO2	hsa-miR-4714-3p
AGO2	hsa-miR-6751-3p
AGO2	hsa-miR-548ab
AGO2	hsa-miR-7084-5p
AGO2	hsa-miR-6910-5p
AGO2	hsa-miR-7051-5p
AGO2	hsa-miR-4752
AGO2	hsa-miR-499a-3p
AGO2	hsa-miR-508-3p
AGO2	hsa-miR-7360-5p
AGO2	hsa-miR-885-5p
AGO2	hsa-miR-1781-5p
AGO2	hsa-miR-330-3p.1
AGO2	hsa-miR-7178-3p
AGO2	hsa-miR-193a
AGO2	hsa-miR-7040-3p
AGO2	hsa-miR-3158-5p
AGO2	hsa-miR-6846-5p
AGO2	hsa-miR-346
AGO2	hsa-miR-6641-5p
AGO2	hsa-miR-106a-5p
AGO2	hsa-miR-5622-5p
AGO2	hsa-miR-208a-3p
AGO2	hsa-miR-6756-3p
AGO2	hsa-miR-7172-5p
AGO2	hsa-miR-4417
AGO2	hsa-miR-1695
AGO2	hsa-miR-3063-3p
AGO2	hsa-miR-548b-3p
AGO2	hsa-miR-486-5p

AGO2	hsa-miR-7160-3p
AGO2	hsa-miR-155-3p
AGO2	hsa-miR-6576-3p
AGO2	hsa-miR-6913-5p
AGO2	hsa-miR-8908e
AGO2	hsa-miR-7258-5p
AGO2	hsa-miR-513b-3p
AGO2	hsa-miR-2454-3p
AGO2	hsa-miR-7185-3p
AGO2	hsa-miR-450b
AGO2	hsa-miR-873b
AGO2	hsa-miR-3551-5p
AGO2	hsa-miR-2355-5p
AGO2	hsa-miR-365a-3p
AGO2	hsa-miR-207
AGO2	hsa-miR-543
AGO2	hsa-miR-8119
AGO2	hsa-miR-1612
AGO2	hsa-miR-128-1-5p
AGO2	hsa-miR-590-5p
AGO2	hsa-miR-3561-5p
AGO2	hsa-miR-4271
AGO2	hsa-miR-548s
AGO2	hsa-miR-7156-3p
AGO2	hsa-miR-6829-3p
AGO2	hsa-miR-4795-5p
AGO2	hsa-miR-4330
AGO2	hsa-miR-4723-3p
AGO2	hsa-miR-1270
AGO2	hsa-miR-6564-3p
AGO2	hsa-miR-7398r-3p
AGO2	hsa-miR-1262-5p
AGO2	hsa-miR-7275-5p
AGO2	hsa-miR-548ay-3p
AGO2	hsa-miR-4697-5p
AGO2	hsa-miR-7051-3p
AGO2	hsa-miR-3593-5p
AGO2	hsa-miR-1207-5p
AGO2	hsa-miR-1288-5p
AGO2	hsa-miR-1277-5p
AGO2	hsa-miR-4495
AGO2	hsa-miR-7118-5p
AGO2	hsa-miR-7346-5p
AGO2	hsa-miR-4529-3p
AGO2	hsa-miR-2130
AGO2	hsa-miR-548ao-5p

AGO2	hsa-miR-1247-5p
AGO2	hsa-miR-3571
AGO2	hsa-miR-2899
AGO2	hsa-miR-7022-5p
AGO2	hsa-miR-7667-5p
AGO2	hsa-miR-7664-3p
AGO2	hsa-miR-1684-3p
AGO2	hsa-miR-5691
AGO2	hsa-miR-101-2-5p
AGO2	hsa-miR-1273e
AGO2	hsa-miR-6809-5p
AGO2	hsa-miR-1715-5p
AGO2	hsa-miR-7374-5p
AGO2	hsa-miR-15-5p
AGO2	hsa-miR-2329-3p
AGO2	hsa-miR-6690-5p
AGO2	hsa-miR-133-3p
AGO2	hsa-miR-7334-3p
AGO2	hsa-miR-7017-5p
AGO2	hsa-miR-5571-5p
AGO2	hsa-miR-495-3p
AGO2	hsa-miR-922
AGO2	hsa-miR-7398m-3p
AGO2	hsa-miR-2127
AGO2	hsa-miR-7336-5p
AGO2	hsa-miR-7386k-3p
AGO2	hsa-miR-1301
AGO2	hsa-miR-1468-3p
AGO2	hsa-miR-297-5p
AGO2	hsa-miR-181a-1-3p
AGO2	hsa-miR-7257-3p
AGO2	hsa-miR-369-3p
AGO2	hsa-let-7afk-3p
AGO2	hsa-miR-379-5p
AGO2	hsa-miR-3549
AGO2	hsa-miR-138-5p
AGO2	hsa-miR-2409
AGO2	hsa-miR-105-5p
AGO2	hsa-miR-8867
AGO2	hsa-miR-561-3p
AGO2	hsa-miR-129b-5p
AGO2	hsa-miR-6524
AGO2	hsa-miR-5571-3p
AGO2	hsa-miR-3944-5p
AGO2	hsa-miR-7216-5p
AGO2	hsa-miR-376a-2-5p

AGO2	hsa-miR-8901
AGO2	hsa-miR-550b-3p
AGO2	hsa-miR-7319-3p
AGO2	hsa-miR-500a-5p
AGO2	hsa-miR-184-3p
AGO2	hsa-miR-589-5p
AGO2	hsa-miR-3173-3p
AGO2	hsa-let-7abf-3p
AGO2	hsa-miR-194-5p
AGO2	hsa-miR-351-5p
AGO2	hsa-miR-1805-3p
AGO2	hsa-miR-6981-5p
AGO2	hsa-miR-6902-5p
AGO2	hsa-miR-362-5p
AGO2	hsa-miR-8906
AGO2	hsa-miR-3157-5p
AGO2	hsa-miR-8104
AGO2	hsa-miR-1982-5p
AGO2	hsa-miR-5046
AGO2	hsa-miR-6376
AGO2	hsa-miR-1798-3p
AGO2	hsa-miR-2392
AGO2	hsa-miR-3470b
AGO2	hsa-miR-762
AGO2	hsa-miR-6762-5p
AGO2	hsa-miR-507
AGO2	hsa-miR-6803-5p
AGO2	hsa-miR-193-5p
AGO2	hsa-miR-6990-5p
AGO2	hsa-miR-3577-3p
AGO2	hsa-miR-3087-5p
AGO2	hsa-miR-7348-3p
AGO2	hsa-miR-8092
AGO2	hsa-miR-320-3p
AGO2	hsa-miR-520f-3p
AGO2	hsa-miR-5114
AGO2	hsa-miR-1467-3p
AGO2	hsa-miR-139-3p
AGO2	hsa-miR-3084bc-5p
AGO2	hsa-miR-7655-3p
AGO2	hsa-miR-140-5p
AGO2	hsa-miR-7344-5p
AGO2	hsa-miR-8095
AGO2	hsa-miR-6843-3p
AGO2	hsa-miR-1304-3p
AGO2	hsa-miR-8102

AGO2	hsa-miR-4322
AGO2	hsa-miR-7157-5p
AGO2	hsa-miR-583
AGO2	hsa-miR-367-5p
AGO2	hsa-miR-7078-5p
AGO2	hsa-miR-6930-3p
AGO2	hsa-miR-592
AGO2	hsa-miR-33a-3p
AGO2	hsa-miR-7052-5p
AGO2	hsa-miR-5129-5p
AGO2	hsa-miR-540-3p
AGO2	hsa-miR-141-5p
AGO2	hsa-miR-548ax
AGO2	hsa-miR-7182-3p
AGO2	hsa-miR-2385-3p
AGO2	hsa-miR-6605-5p
AGO2	hsa-miR-2284-3p
AGO2	hsa-miR-4297
AGO2	hsa-miR-548p
AGO2	hsa-miR-7081-5p
AGO2	hsa-miR-9b-3p
AGO2	hsa-miR-2285h
AGO2	hsa-miR-1266
AGO2	hsa-miR-4498
AGO2	hsa-miR-548ap-3p
AGO2	hsa-miR-6528
AGO2	hsa-miR-7218-3p
AGO2	hsa-miR-7300-5p
AGO2	hsa-miR-508-5p
AGO2	hsa-miR-7164-5p
AGO2	hsa-miR-602
AGO2	hsa-miR-4713-5p
AGO2	hsa-miR-200-3p
AGO2	hsa-miR-6409
AGO2	hsa-miR-760-3p
AGO2	hsa-miR-2455
AGO2	hsa-miR-3972
AGO2	hsa-miR-708-5p
AGO2	hsa-miR-1931
AGO2	hsa-miR-8085
AGO2	hsa-miR-502a
AGO2	hsa-miR-7655-5p
AGO2	hsa-miR-6890-3p
AGO2	hsa-miR-767-3p
AGO2	hsa-miR-6722-3p
AGO2	hsa-miR-2285u

AGO2	hsa-miR-2325b
AGO2	hsa-miR-7677-3p
AGO2	hsa-miR-16-2-3p
AGO2	hsa-miR-92
AGO2	hsa-miR-208a-5p
AGO2	hsa-miR-3170
AGO2	hsa-miR-182-3p
AGO2	hsa-miR-103b
AGO2	hsa-miR-4718
AGO2	hsa-miR-631
AGO2	hsa-miR-200a-3p
AGO2	hsa-miR-7189-5p
AGO2	hsa-miR-4695-5p
AGO2	hsa-miR-7864
AGO2	hsa-miR-622
AGO2	hsa-miR-5580-3p
AGO2	hsa-miR-3078-3p
AGO2	hsa-miR-199-1-5p
AGO2	hsa-miR-571
AGO2	hsa-miR-2341
AGO2	hsa-miR-20-5p
AGO2	hsa-miR-34c-3p
AGO2	hsa-miR-294-5p
AGO2	hsa-miR-4766-3p
AGO2	hsa-miR-499b-3p
AGO2	hsa-miR-624-3p
AGO2	hsa-miR-212-5p
AGO2	hsa-miR-802-5p
AGO2	hsa-miR-3589
AGO2	hsa-miR-193-3p
AGO2	hsa-miR-1460
AGO2	hsa-miR-2276-5p
AGO2	hsa-miR-1545c-5p
AGO2	hsa-miR-7702
AGO2	hsa-miR-3612
AGO2	hsa-miR-6393
AGO2	hsa-miR-4538
AGO2	hsa-miR-6940-5p
AGO2	hsa-miR-1245-5p
AGO2	hsa-miR-3934-3p
AGO2	hsa-miR-1290
AGO2	hsa-miR-4505
AGO2	hsa-miR-5189-5p
AGO2	hsa-miR-6735-5p
AGO2	hsa-miR-2461-3p
AGO2	hsa-miR-7299-3p

AGO2	hsa-miR-6816-3p
AGO2	hsa-miR-30-5p
AGO2	hsa-miR-126-3p.1
AGO2	hsa-miR-148
AGO2	hsa-miR-7026-5p
AGO2	hsa-miR-463-3p
AGO2	hsa-miR-5572
AGO2	hsa-miR-2284w
AGO2	hsa-miR-769
AGO2	hsa-miR-7443-3p
AGO2	hsa-miR-5197-3p
AGO2	hsa-miR-873-5p
AGO2	hsa-miR-7330-5p
AGO2	hsa-miR-497-3p
AGO2	hsa-miR-7291-3p
AGO2	hsa-miR-423-3p
AGO2	hsa-miR-644a
AGO2	hsa-miR-873-5p.2
AGO2	hsa-miR-654-5p
AGO2	hsa-miR-1911-5p
AGO2	hsa-miR-22-5p
AGO2	hsa-miR-454-3p
AGO2	hsa-miR-8820
AGO2	hsa-miR-30e-5p
AGO2	hsa-miR-349
AGO2	hsa-miR-4715-3p
AGO2	hsa-miR-183-5p.2
AGO2	hsa-miR-4716
AGO2	hsa-miR-1617
AGO2	hsa-miR-6889-5p
AGO2	hsa-miR-129-5p
AGO2	hsa-miR-3591-3p
AGO2	hsa-miR-2285af
AGO2	hsa-miR-344f-5p
AGO2	hsa-miR-542-5p
AGO2	hsa-miR-471-3p
AGO2	hsa-miR-4744
AGO2	hsa-miR-1967
AGO2	hsa-miR-6755-5p
AGO2	hsa-miR-548aa
AGO2	hsa-miR-3100-5p
AGO2	hsa-miR-363-3p
AGO2	hsa-miR-7161-5p
AGO2	hsa-miR-3584-5p
AGO2	hsa-miR-7b-3p
AGO2	hsa-miR-4786-3p

AGO2	hsa-miR-369-5p
AGO2	hsa-miR-7653-5p
AGO2	hsa-miR-6590-3p
AGO2	hsa-miR-383-3p
AGO2	hsa-miR-6795-5p
AGO2	hsa-miR-660-5p
AGO2	hsa-miR-196a-3p
AGO2	hsa-miR-3663-3p
AGO2	hsa-miR-548az-5p
AGO2	hsa-miR-7244-3p
AGO2	hsa-miR-7472-5p
AGO2	hsa-miR-3105-5p
AGO2	hsa-miR-6995-5p
AGO2	hsa-miR-7251-3p
AGO2	hsa-miR-3169
AGO2	hsa-miR-3547-5p
AGO2	hsa-miR-141-3p
AGO2	hsa-miR-513b-5p
AGO2	hsa-miR-215-5p
AGO2	hsa-miR-1691
AGO2	hsa-miR-1729-5p
AGO2	hsa-miR-223-3p
AGO2	hsa-miR-518-3p
AGO2	hsa-miR-3119
AGO2	hsa-miR-7166-5p
AGO2	hsa-miR-8064
AGO2	hsa-miR-203-3p.1
AGO2	hsa-miR-8908-3p
AGO2	hsa-miR-19b-5p
AGO2	hsa-miR-7355-3p
AGO2	hsa-miR-6133
AGO2	hsa-miR-128-5p
AGO2	hsa-miR-2465
AGO2	hsa-miR-2483
AGO2	hsa-miR-7010-3p
AGO2	hsa-miR-7060-3p
AGO2	hsa-miR-300-5p
AGO2	hsa-miR-106b-5p
AGO2	hsa-miR-2897
AGO2	hsa-miR-129-2-3p
AGO2	hsa-miR-1760
AGO2	hsa-miR-7058-5p
AGO2	hsa-miR-3099-5p
AGO2	hsa-miR-222-5p
AGO2	hsa-miR-2284c
AGO2	hsa-miR-1228-3p

AGO2	hsa-miR-564
AGO2	hsa-miR-449-3p
AGO2	hsa-miR-7281-3p
AGO2	hsa-miR-6973a-5p
AGO2	hsa-miR-32-5p
AGO2	hsa-miR-1747-3p
AGO2	hsa-miR-6888-5p
AGO2	hsa-miR-216-5p
AGO2	hsa-miR-7323-5p
AGO2	hsa-miR-2381
AGO2	hsa-miR-2448-3p
AGO2	hsa-miR-6991-5p
AGO2	hsa-miR-194-3p
AGO2	hsa-miR-3152-5p
AGO2	hsa-miR-7231-3p
AGO2	hsa-miR-3065-5p
AGO2	hsa-miR-1191b-5p
AGO2	hsa-miR-7163-5p
AGO2	hsa-miR-7202-3p
AGO2	hsa-miR-6630-3p
AGO2	hsa-miR-3686
AGO2	hsa-miR-4428
AGO2	hsa-miR-7246-3p
AGO2	hsa-miR-6499-3p
AGO2	hsa-miR-502-5p
AGO2	hsa-miR-7339-5p
AGO2	hsa-miR-7467-3p
AGO2	hsa-miR-7370i-3p
AGO2	hsa-miR-3062-3p
AGO2	hsa-miR-7386l-3p
AGO2	hsa-miR-6086
AGO2	hsa-miR-5187-3p
AGO2	hsa-miR-6691-5p
AGO2	hsa-miR-2890
AGO2	hsa-miR-7043-3p
AGO2	hsa-miR-4769-3p
AGO2	hsa-miR-708-5p
AGO2	hsa-miR-374-3p
AGO2	hsa-miR-10a-3p
AGO2	hsa-miR-181d-5p
AGO2	hsa-miR-1258-5p
AGO2	hsa-miR-3065-3p
AGO2	hsa-miR-7052-3p
AGO2	hsa-miR-7336-3p
AGO2	hsa-miR-6836-3p
AGO2	hsa-miR-6678-3p

AGO2	hsa-miR-7371a-5p
AGO2	hsa-miR-30e-3p
AGO2	hsa-miR-548ao-3p
AGO2	hsa-miR-92a-2-5p
AGO2	hsa-miR-7180
AGO2	hsa-miR-219b-5p
AGO2	hsa-miR-6979-5p
AGO2	hsa-miR-3094-5p
AGO2	hsa-miR-1292-5p
AGO2	hsa-miR-6833-5p
AGO2	hsa-miR-606
AGO2	hsa-miR-7863
AGO2	hsa-miR-6743-5p
AGO2	hsa-miR-7341-3p
AGO2	hsa-miR-367-3p
AGO2	hsa-miR-6997-3p
AGO2	hsa-miR-199-3p
AGO2	hsa-miR-335-5p
AGO2	hsa-miR-5003-3p
AGO2	hsa-miR-548z
AGO2	hsa-miR-6126
AGO2	hsa-miR-8065
AGO2	hsa-miR-878-3p
AGO2	hsa-miR-8809
AGO2	hsa-miR-210-5p
AGO2	hsa-miR-7374a-3p
AGO2	hsa-miR-8066
AGO2	hsa-miR-16c-3p
AGO2	hsa-miR-4487
AGO2	hsa-miR-3097-3p
AGO2	hsa-miR-3569-3p
AGO2	hsa-miR-7030-3p
AGO2	hsa-miR-4722-3p
AGO2	hsa-miR-1843
AGO2	hsa-miR-7297-5p
AGO2	hsa-miR-460-3p
AGO2	hsa-miR-4694-5p
AGO2	hsa-miR-2461-5p
AGO2	hsa-miR-7077-5p
AGO2	hsa-miR-3919
AGO2	hsa-miR-1722-3p
AGO2	hsa-miR-2129
AGO2	hsa-miR-2414
AGO2	hsa-miR-9768-3p
AGO2	hsa-miR-362-5p
AGO2	hsa-miR-1663-3p

AGO2	hsa-miR-659-5p
AGO2	hsa-miR-6131
AGO2	hsa-miR-6577-3p
AGO2	hsa-let-7ag-3p
AGO2	hsa-miR-561-5p
AGO2	hsa-miR-4422
AGO2	hsa-miR-3101-5p
AGO2	hsa-miR-6649-3p
AGO2	hsa-miR-3912-5p
AGO2	hsa-miR-6927-5p
AGO2	hsa-miR-698-3p
AGO2	hsa-miR-7116-5p
AGO2	hsa-miR-138-3p
AGO2	hsa-miR-770
AGO2	hsa-miR-22-3p
AGO2	hsa-miR-3186-3p
AGO2	hsa-miR-7069-5p
AGO2	hsa-miR-552-5p
AGO2	hsa-miR-3431
AGO2	hsa-miR-7465-3p
AGO2	hsa-miR-2682-3p
AGO2	hsa-miR-6914-5p
AGO2	hsa-miR-7448-3p
AGO2	hsa-miR-2373-5p
AGO2	hsa-miR-3600
AGO2	hsa-miR-7656-3p
AGO2	hsa-miR-6507-5p
AGO2	hsa-miR-3070-5p
AGO2	hsa-miR-7398n-3p
AGO2	hsa-miR-7385a-3p
AGO2	hsa-miR-7359-5p
AGO2	hsa-miR-183-5p
AGO2	hsa-miR-4276
AGO2	hsa-miR-1258
AGO2	hsa-miR-2284q
AGO2	hsa-miR-8113
AGO2	hsa-miR-6730-3p
AGO2	hsa-miR-3102-5p.2-5p
AGO2	hsa-miR-7350-5p
AGO2	hsa-miR-203-3p.2
AGO2	hsa-miR-3650
AGO2	hsa-miR-8844
AGO2	hsa-miR-5582-3p
AGO2	hsa-miR-3109-5p
AGO2	hsa-miR-7290-3p
AGO2	hsa-miR-2329-5p

AGO2	hsa-miR-1537-5p
AGO2	hsa-miR-7037-5p
AGO2	hsa-miR-1434-3p
AGO2	hsa-miR-7222-3p
AGO2	hsa-miR-692
AGO2	hsa-miR-6768-3p
AGO2	hsa-miR-590-3p
AGO2	hsa-miR-6837-3p
AGO2	hsa-miR-4763-5p
AGO2	hsa-miR-2439-5p
AGO2	hsa-miR-16-3p
AGO2	hsa-miR-544-5p
AGO2	hsa-miR-5000-3p
AGO2	hsa-miR-1281
AGO2	hsa-miR-1258-3p
AGO2	hsa-miR-34b-3p
AGO2	hsa-miR-6077
AGO2	hsa-miR-6794-5p
AGO2	hsa-miR-1260a
AGO2	hsa-miR-4765
AGO2	hsa-miR-6549-3p
AGO2	hsa-miR-7462-5p
AGO2	hsa-miR-208a-3p
AGO2	hsa-miR-6982-3p
AGO2	hsa-miR-2401
AGO2	hsa-miR-7277-3p
AGO2	hsa-miR-3155a
AGO2	hsa-miR-6971-5p
AGO2	hsa-miR-322-5p
AGO2	hsa-miR-1702
AGO2	hsa-miR-7329-3p
AGO2	hsa-miR-3069-3p
AGO2	hsa-miR-5004-5p
AGO2	hsa-miR-1185-3p
AGO2	hsa-miR-7073-3p
AGO2	hsa-miR-4329
AGO2	hsa-miR-605-5p
AGO2	hsa-miR-3689a-5p
AGO2	hsa-miR-454-5p
AGO2	hsa-miR-532-5p
AGO2	hsa-miR-3971
AGO2	hsa-miR-145-5p
AGO2	hsa-miR-6399
AGO2	hsa-miR-181b-1-3p
AGO2	hsa-miR-2450a
AGO2	hsa-miR-449b-3p

AGO2	hsa-miR-892c-3p
AGO2	hsa-miR-1462-3p
AGO2	hsa-miR-7437-5p
AGO2	hsa-miR-2467-3p
AGO2	hsa-miR-500b-3p
AGO2	hsa-miR-6710-3p
AGO2	hsa-miR-190b
AGO2	hsa-miR-4649-5p
AGO2	hsa-miR-3090-5p
AGO2	hsa-miR-742-3p
AGO2	hsa-miR-148a-3p
AGO2	hsa-miR-125a-5p
AGO2	hsa-miR-1203
AGO2	hsa-miR-4661-3p
AGO2	hsa-let-7d-5p
AGO2	hsa-miR-8902
AGO2	hsa-miR-7011-3p
AGO2	hsa-miR-4719
AGO2	hsa-miR-320b
AGO2	hsa-miR-7669-3p
AGO2	hsa-miR-1544-3p
AGO2	hsa-miR-142
AGO2	hsa-miR-124-3p.2
AGO2	hsa-miR-1827
AGO2	hsa-miR-2285v
AGO2	hsa-miR-6999-5p
AGO2	hsa-miR-8889
AGO2	hsa-miR-6327
AGO2	hsa-miR-4738-3p
AGO2	hsa-miR-7256-5p
AGO2	hsa-miR-101-3p
AGO2	hsa-miR-6773-3p
AGO2	hsa-miR-6071
AGO2	hsa-miR-7286-3p
AGO2	hsa-miR-346-3p
AGO2	hsa-miR-382-5p
AGO2	hsa-miR-7162-3p
AGO2	hsa-miR-7289-5p
AGO2	hsa-miR-5621-5p
AGO2	hsa-miR-547-3p
AGO2	hsa-miR-1467-5p
AGO2	hsa-miR-548h-3p
AGO2	hsa-miR-10
AGO2	hsa-miR-5118
AGO2	hsa-miR-322-3p
AGO2	hsa-miR-2429

AGO2	hsa-miR-1766
AGO2	hsa-miR-6990-3p
AGO2	hsa-miR-6978-5p
AGO2	hsa-miR-2113
AGO2	hsa-miR-3583-5p
AGO2	hsa-miR-7020-5p
AGO2	hsa-miR-1291
AGO2	hsa-miR-4448
AGO2	hsa-miR-6836-5p
AGO2	hsa-miR-504
AGO2	hsa-miR-7466-5p
AGO2	hsa-miR-4324
AGO2	hsa-miR-8850
AGO2	hsa-miR-7684-3p
AGO2	hsa-miR-7024-3p
AGO2	hsa-miR-3180-5p
AGO2	hsa-miR-7201-3p
AGO2	hsa-miR-3475-3p
AGO2	hsa-miR-6873-5p
AGO2	hsa-miR-1236-3p
AGO2	hsa-miR-7272-5p
AGO2	hsa-miR-216a-5p
AGO2	hsa-miR-548aj-3p
AGO2	hsa-miR-125b-2-3p
AGO2	hsa-miR-216b-5p
AGO2	hsa-let-7f-1-3p
AGO2	hsa-miR-4743-5p
AGO2	hsa-miR-6564-5p
AGO2	hsa-miR-325
AGO2	hsa-miR-138-2-3p
AGO2	hsa-miR-133
AGO2	hsa-miR-8089
AGO2	hsa-miR-2323
AGO2	hsa-miR-4666b
AGO2	hsa-miR-7276-3p
AGO2	hsa-miR-153-3p
AGO2	hsa-miR-3161
AGO2	hsa-miR-1545b-3-3p
AGO2	hsa-miR-488-3p
AGO2	hsa-miR-483-3p
AGO2	hsa-miR-138-5p
AGO2	hsa-miR-194-1-3p
AGO2	hsa-miR-2384
AGO2	hsa-miR-6891-5p
AGO2	hsa-miR-5626-5p
AGO2	hsa-miR-691

AGO2	hsa-miR-697
AGO2	hsa-miR-513a
AGO2	hsa-miR-8856
AGO2	hsa-miR-3192
AGO2	hsa-miR-4527
AGO2	hsa-miR-548
AGO2	hsa-miR-2452
AGO2	hsa-miR-582-3p
AGO2	hsa-miR-578
AGO2	hsa-miR-667-5p
AGO2	hsa-miR-551b-5p
AGO2	hsa-miR-3652
AGO2	hsa-miR-1838
AGO2	hsa-miR-6750-3p
AGO2	hsa-miR-6848-3p
AGO2	hsa-miR-6987-5p
AGO2	hsa-miR-628-3p
AGO2	hsa-miR-7296-3p
AGO2	hsa-miR-6578-3p
AGO2	hsa-miR-551-5p
AGO2	hsa-miR-6551-3p
AGO2	hsa-miR-743a-3p
AGO2	hsa-miR-590-5p
AGO2	hsa-miR-7117-3p
AGO2	hsa-miR-1669
AGO2	hsa-miR-374c-5p
AGO2	hsa-miR-135a-5p
AGO2	hsa-miR-219c-5p
AGO2	hsa-miR-8789
AGO2	hsa-miR-8864
AGO2	hsa-miR-2454-5p
AGO2	hsa-miR-2285y
AGO2	hsa-miR-199
AGO2	hsa-miR-8872
AGO2	hsa-miR-3617-5p
AGO2	hsa-miR-6749-3p
AGO2	hsa-miR-181b-2-3p
AGO2	hsa-miR-3689f
AGO2	hsa-miR-3099-3p
AGO2	hsa-miR-8070
AGO2	hsa-miR-7269-3p
AGO2	hsa-let-7c-1-3p
AGO2	hsa-miR-548az-3p
AGO2	hsa-miR-128
AGO2	hsa-miR-548l
AGO2	hsa-miR-302c-3p

AGO2	hsa-miR-6919-3p
AGO2	hsa-miR-5100
AGO2	hsa-miR-4535
AGO2	hsa-miR-3672
AGO2	hsa-miR-6548-5p
AGO2	hsa-miR-4272
AGO2	hsa-miR-4803
AGO2	hsa-miR-6841-5p
AGO2	hsa-miR-7198-3p
AGO2	hsa-miR-4659a-3p
AGO2	hsa-miR-2483-3p
AGO2	hsa-miR-6842-5p
AGO2	hsa-miR-194-5p
AGO2	hsa-miR-6076
AGO2	hsa-miR-99-5p
AGO2	hsa-miR-4777-5p
AGO2	hsa-miR-6686-3p
AGO2	hsa-miR-6085
AGO2	hsa-miR-3162-3p
AGO2	hsa-miR-7482-5p
AGO2	hsa-miR-7652-3p
AGO2	hsa-miR-4665-5p
AGO2	hsa-miR-2417
AGO2	hsa-miR-6958-5p
AGO2	hsa-miR-20b-3p
AGO2	hsa-miR-2404
AGO2	hsa-miR-890-5p
AGO2	hsa-miR-130b-5p
AGO2	hsa-miR-7317-3p
AGO2	hsa-miR-1807
AGO2	hsa-miR-292b-3p
AGO2	hsa-miR-6950-5p
AGO2	hsa-miR-205-5p
AGO2	hsa-miR-330-3p.2
AGO2	hsa-miR-499b-5p
AGO2	hsa-miR-663b
AGO2	hsa-miR-3153
AGO2	hsa-miR-548ai
AGO2	hsa-miR-7647-3p
AGO2	hsa-miR-2402
AGO2	hsa-miR-718
AGO2	hsa-miR-1571
AGO2	hsa-miR-2370-3p
AGO2	hsa-miR-6529
AGO2	hsa-miR-7663-5p
AGO2	hsa-miR-522-3p

AGO2	hsa-miR-7247-5p
AGO2	hsa-miR-7113-3p
AGO2	hsa-miR-29b-1-5p
AGO2	hsa-miR-425-5p
AGO2	hsa-miR-434-5p
AGO2	hsa-miR-1667-5p
AGO2	hsa-miR-627-3p
AGO2	hsa-miR-154-3p
AGO2	hsa-miR-504-5p
AGO2	hsa-miR-3661
AGO2	hsa-miR-3106-5p
AGO2	hsa-miR-214-5p
AGO2	hsa-miR-874-3p
AGO2	hsa-miR-4316
AGO2	hsa-miR-639
AGO2	hsa-miR-669e-3p
AGO2	hsa-miR-4496
AGO2	hsa-miR-4445-5p
AGO2	hsa-miR-1626-3p
AGO2	hsa-miR-4796-3p
AGO2	hsa-miR-7385-5p
AGO2	hsa-miR-1626-5p
AGO2	hsa-miR-3131
AGO2	hsa-miR-4492
AGO2	hsa-miR-505-5p
AGO2	hsa-miR-6737-5p
AGO2	hsa-miR-5683
AGO2	hsa-miR-883a-5p
AGO2	hsa-miR-4742-5p
AGO2	hsa-miR-106-3p
AGO2	hsa-miR-491-3p
AGO2	hsa-miR-1194
AGO2	hsa-miR-7374c-3p
AGO2	hsa-miR-6337
AGO2	hsa-miR-7175-5p
AGO2	hsa-miR-466ade-3p
AGO2	hsa-miR-8096
AGO2	hsa-miR-1721
AGO2	hsa-miR-7191-5p
AGO2	hsa-miR-3977
AGO2	hsa-miR-1640
AGO2	hsa-miR-877-5p
AGO2	hsa-miR-449c-3p
AGO2	hsa-miR-190-5p
AGO2	hsa-miR-187-3p
AGO2	hsa-miR-2881

AGO2	hsa-miR-7227-3p
AGO2	hsa-miR-124-3p.1
AGO2	hsa-miR-6754-5p
AGO2	hsa-miR-6613-3p
AGO2	hsa-miR-5587-3p
AGO2	hsa-miR-7029-3p
AGO2	hsa-miR-5581-5p
AGO2	hsa-miR-7387-5p
AGO2	hsa-miR-6697-5p
AGO2	hsa-miR-6336
AGO2	hsa-miR-6772-3p
AGO2	hsa-miR-4639-3p
AGO2	hsa-miR-7112-3p
AGO2	hsa-miR-514a-3p
AGO2	hsa-miR-154b
AGO2	hsa-miR-376c
AGO2	hsa-miR-500-3p
AGO2	hsa-miR-3533
AGO2	hsa-miR-3607-5p
AGO2	hsa-miR-224-5p
AGO2	hsa-miR-148b-5p
AGO2	hsa-miR-7307-5p
AGO2	hsa-miR-4446
AGO2	hsa-miR-500-5p
AGO2	hsa-miR-1586
AGO2	hsa-miR-1615
AGO2	hsa-miR-6976-3p
AGO2	hsa-miR-3956
AGO2	hsa-miR-802-3p
AGO2	hsa-miR-7053-5p
AGO2	hsa-miR-373-3p
AGO2	hsa-miR-877-5p
AGO2	hsa-miR-4300
AGO2	hsa-miR-506-5p
AGO2	hsa-miR-1690-5p
AGO2	hsa-miR-676-3p
AGO2	hsa-miR-6523a
AGO2	hsa-miR-7023-5p
AGO2	hsa-miR-613
AGO2	hsa-miR-23a-5p
AGO2	hsa-miR-4423-3p
AGO2	hsa-miR-520c-3p
AGO2	hsa-miR-7672-3p
AGO2	hsa-miR-1632-3p
AGO2	hsa-miR-20-3p
AGO2	hsa-miR-3059-3p

AGO2	hsa-miR-548a-5p
AGO2	hsa-miR-6972-5p
AGO2	hsa-miR-92a-1-5p
AGO2	hsa-miR-8871
AGO2	hsa-miR-6397
AGO2	hsa-miR-3671
AGO2	hsa-miR-154-5p
AGO2	hsa-miR-7373d-3p
AGO2	hsa-miR-655-3p
AGO2	hsa-miR-1251
AGO2	hsa-miR-892a
AGO2	hsa-miR-1225-3p
AGO2	hsa-miR-7107-3p
AGO2	hsa-miR-411-5p
AGO2	hsa-miR-188-3p
AGO2	hsa-miR-509-3p
AGO2	hsa-miR-1193-3p
AGO2	hsa-miR-6795-3p
AGO2	hsa-miR-3942-5p
AGO2	hsa-miR-489-3p
AGO2	hsa-miR-498
AGO2	hsa-miR-4474-3p
AGO2	hsa-miR-3938
AGO2	hsa-miR-7292-5p
AGO2	hsa-miR-7398s-3p
AGO2	hsa-miR-6899-5p
AGO2	hsa-miR-669j
AGO2	hsa-miR-593-5p
AGO2	hsa-miR-2466-3p
AGO2	hsa-miR-6586-5p
AGO2	hsa-miR-2438
AGO2	hsa-miR-6718-5p
AGO2	hsa-miR-548g-3p
AGO2	hsa-miR-615-5p
AGO2	hsa-miR-584-3p
AGO2	hsa-miR-7161-3p
AGO2	hsa-miR-4731-5p
AGO2	hsa-miR-6976-5p
AGO2	hsa-miR-3177-5p
AGO2	hsa-miR-7055-5p
AGO2	hsa-miR-7212-5p
AGO2	hsa-miR-127-5p
AGO2	hsa-miR-5093
AGO2	hsa-miR-6090
AGO2	hsa-miR-3133
AGO2	hsa-miR-6882-5p

AGO2	hsa-miR-7357-5p
AGO2	hsa-miR-3073a-3p
AGO2	hsa-miR-5699-5p
AGO2	hsa-miR-3070-3p
AGO2	hsa-miR-6948-5p
AGO2	hsa-miR-7293-5p
AGO2	hsa-miR-6964-3p
AGO2	hsa-miR-1697
AGO2	hsa-miR-1679
AGO2	hsa-miR-466bco-5p
AGO2	hsa-miR-7044-3p
AGO2	hsa-miR-7333-5p
AGO2	hsa-miR-519d-3p
AGO2	hsa-miR-8842
AGO2	hsa-miR-4747-5p
AGO2	hsa-miR-1643-5p
AGO2	hsa-miR-6544-5p
AGO2	hsa-miR-5008-5p
AGO2	hsa-miR-4488
AGO2	hsa-miR-7287-5p
AGO2	hsa-miR-1207
AGO2	hsa-miR-669a-3-3p
AGO2	hsa-miR-4522
AGO2	hsa-miR-154-5p
AGO2	hsa-miR-330-3p
AGO2	hsa-miR-4261
AGO2	hsa-miR-1705
AGO2	hsa-miR-4303
AGO2	hsa-miR-450b-5p
AGO2	hsa-miR-597-3p
AGO2	hsa-miR-140-3p.2
AGO2	hsa-miR-7073-5p
AGO2	hsa-miR-6997-5p
AGO2	hsa-miR-7014-5p
AGO2	hsa-miR-7665-3p
AGO2	hsa-miR-196
AGO2	hsa-miR-4762-3p
AGO2	hsa-miR-326-5p
AGO2	hsa-miR-125
AGO2	hsa-let-7i-3p
AGO2	hsa-miR-376b-3p
AGO2	hsa-miR-1245b-5p
AGO2	hsa-miR-4780
AGO2	hsa-miR-4432
AGO2	hsa-miR-6769a-5p
AGO2	hsa-miR-4742-3p

AGO2	hsa-miR-1285-5p
AGO2	hsa-miR-7191-3p
AGO2	hsa-miR-31-3p
AGO2	hsa-miR-3121-3p
AGO2	hsa-miR-1225-5p
AGO2	hsa-miR-3116
AGO2	hsa-miR-764
AGO2	hsa-miR-6072
AGO2	hsa-miR-3602
AGO2	hsa-miR-1544-5p
AGO2	hsa-miR-2110
AGO2	hsa-miR-5088-5p
AGO2	hsa-miR-3691-3p
AGO2	hsa-miR-4779
AGO2	hsa-miR-508b
AGO2	hsa-miR-6797-3p
AGO2	hsa-miR-6677-5p
AGO2	hsa-miR-1198-3p
AGO2	hsa-miR-891a-5p
AGO2	hsa-miR-7331-5p
AGO2	hsa-miR-2325
AGO2	hsa-miR-2439-3p
AGO2	hsa-miR-298
AGO2	hsa-miR-7222-5p
AGO2	hsa-miR-545-3p
AGO2	hsa-miR-4470
AGO2	hsa-miR-8908-5p
AGO2	hsa-miR-7179-3p
AGO2	hsa-miR-6910-3p
AGO2	hsa-miR-6683-3p
AGO2	hsa-miR-1543
AGO2	hsa-miR-7340-5p
AGO2	hsa-miR-92b-5p
AGO2	hsa-miR-6801-5p
AGO2	hsa-miR-124c-5p
AGO2	hsa-miR-103-2-5p
AGO2	hsa-miR-1892
AGO2	hsa-miR-1b-5p
AGO2	hsa-miR-4302
AGO2	hsa-miR-548cej-3p
AGO2	hsa-miR-4654
AGO2	hsa-miR-3183
AGO2	hsa-miR-6548-3p
AGO2	hsa-miR-7042-3p
AGO2	hsa-miR-653-3p
AGO2	hsa-miR-7093-5p

AGO2	hsa-miR-872-3p
AGO2	hsa-miR-1909-5p
AGO2	hsa-miR-7229-3p
AGO2	hsa-miR-3649
AGO2	hsa-miR-1197-3p
AGO2	hsa-miR-4797-3p
AGO2	hsa-miR-500a-3p
AGO2	hsa-miR-6641-3p
AGO2	hsa-miR-24-3p
AGO2	hsa-miR-2373-3p
AGO2	hsa-miR-142-3p.2
AGO2	hsa-miR-4468
AGO2	hsa-miR-2428
AGO2	hsa-miR-7257-5p
AGO2	hsa-miR-4418
AGO2	hsa-miR-4690-5p
AGO2	hsa-miR-4762-5p
AGO2	hsa-miR-2343
AGO2	hsa-miR-365-3p
AGO2	hsa-miR-6510-3p
AGO2	hsa-miR-677-5p
AGO2	hsa-miR-7370-5p
AGO2	hsa-miR-7455-3p
AGO2	hsa-miR-1769-5p
AGO2	hsa-miR-2285
AGO2	hsa-miR-8790
AGO2	hsa-miR-6817-3p
AGO2	hsa-miR-4640-3p
AGO2	hsa-miR-371
AGO2	hsa-miR-7071-3p
AGO2	hsa-miR-1-3p
AGO2	hsa-miR-8877
AGO2	hsa-miR-3067-3p
AGO2	hsa-miR-766-3p
AGO2	hsa-miR-4648
AGO2	hsa-miR-562
AGO2	hsa-miR-4278
AGO2	hsa-miR-8832
AGO2	hsa-miR-1298
AGO2	hsa-miR-1323
AGO2	hsa-miR-3120-3p
AGO2	hsa-miR-877-3p
AGO2	hsa-miR-376ab
AGO2	hsa-miR-6545-5p
AGO2	hsa-miR-7332-5p
AGO2	hsa-miR-7464-3p

AGO2	hsa-miR-7388c-5p
AGO2	hsa-miR-487a-3p
AGO2	hsa-miR-655-5p
AGO2	hsa-miR-7226-3p
AGO2	hsa-miR-8796
AGO2	hsa-miR-8825
AGO2	hsa-miR-7324-3p
AGO2	hsa-miR-2284m
AGO2	hsa-miR-3620-3p
AGO2	hsa-miR-7094-3p
AGO2	hsa-miR-598-5p
AGO2	hsa-miR-1839-5p
AGO2	hsa-miR-4777-3p
AGO2	hsa-miR-27-3p
AGO2	hsa-miR-1251-5p
AGO2	hsa-miR-509-3p
AGO2	hsa-miR-6120-3p
AGO2	hsa-miR-569
AGO2	hsa-miR-7353-5p
AGO2	hsa-miR-6913-3p
AGO2	hsa-miR-1193
AGO2	hsa-miR-8793
AGO2	hsa-miR-1655-3p
AGO2	hsa-miR-3095-3p
AGO2	hsa-miR-3918
AGO2	hsa-miR-665-5p
AGO2	hsa-miR-1938
AGO2	hsa-miR-344-3p
AGO2	hsa-miR-1688
AGO2	hsa-miR-5690
AGO2	hsa-miR-4446-3p
AGO2	hsa-miR-6074
AGO2	hsa-miR-2431-5p
AGO2	hsa-miR-1795
AGO2	hsa-miR-5586-5p
AGO2	hsa-miR-7373b-3p
AGO2	hsa-miR-137
AGO2	hsa-miR-509-5p
AGO2	hsa-miR-466c-3p
AGO2	hsa-miR-4708-5p
AGO2	hsa-miR-7687-3p
AGO2	hsa-miR-7332-3p
AGO2	hsa-miR-7159-3p
AGO2	hsa-miR-8068
AGO2	hsa-miR-4268
AGO2	hsa-miR-4484

AGO2	hsa-miR-1236-3p
AGO2	hsa-miR-1234-3p
AGO2	hsa-let-7i-5p
AGO2	hsa-miR-3087-3p
AGO2	hsa-miR-1191b-3p
AGO2	hsa-miR-7087-5p
AGO2	hsa-miR-3473g
AGO2	hsa-miR-32-3p
AGO2	hsa-miR-7274-5p
AGO2	hsa-miR-4725-3p
AGO2	hsa-miR-6510-5p
AGO2	hsa-miR-4656
AGO2	hsa-miR-6941-3p
AGO2	hsa-miR-6511-5p
AGO2	hsa-miR-217-3p
AGO2	hsa-miR-5002-5p
AGO2	hsa-miR-6790-5p
AGO2	hsa-miR-3596a
AGO2	hsa-miR-3609
AGO2	hsa-miR-6888-3p
AGO2	hsa-miR-6875-3p
AGO2	hsa-miR-705
AGO2	hsa-miR-3526
AGO2	hsa-miR-6797-5p
AGO2	hsa-miR-1933-3p
AGO2	hsa-miR-219c-3p
AGO2	hsa-miR-4804-3p
AGO2	hsa-miR-6860
AGO2	hsa-miR-3179
AGO2	hsa-miR-2285t
AGO2	hsa-miR-2311
AGO2	hsa-miR-871-3p
AGO2	hsa-miR-2902
AGO2	hsa-miR-15b-3p
AGO2	hsa-miR-6584-5p
AGO2	hsa-miR-467f
AGO2	hsa-miR-6573-3p
AGO2	hsa-miR-487a
AGO2	hsa-miR-548j-3p
AGO2	hsa-miR-3074-3p
AGO2	hsa-miR-6870-5p
AGO2	hsa-miR-526
AGO2	hsa-miR-6582-3p
AGO2	hsa-miR-3136-3p
AGO2	hsa-miR-7223-3p
AGO2	hsa-miR-152-3p

AGO2	hsa-miR-2116-5p
AGO2	hsa-miR-2331-5p
AGO2	hsa-miR-6738-3p
AGO2	hsa-miR-599-3p
AGO2	hsa-miR-6907-5p
AGO2	hsa-miR-1227-5p
AGO2	hsa-miR-6774-5p
AGO2	hsa-miR-6848-5p
AGO2	hsa-miR-4763-3p
AGO2	hsa-miR-8794
AGO2	hsa-miR-665-3p
AGO2	hsa-miR-30c-2-3p
AGO2	hsa-miR-6614-3p
AGO2	hsa-miR-6963-3p
AGO2	hsa-miR-652-5p
AGO2	hsa-miR-512
AGO2	hsa-miR-7152-3p
AGO2	hsa-miR-452-5p
AGO2	hsa-miR-6988-3p
AGO2	hsa-miR-6569-3p
AGO2	hsa-miR-336-5p
AGO2	hsa-miR-7233-5p
AGO2	hsa-miR-6810-3p
AGO2	hsa-miR-16-5p
AGO2	hsa-miR-200c-5p
AGO2	hsa-miR-197-3p
AGO2	hsa-miR-378
AGO2	hsa-miR-7268-5p
AGO2	hsa-miR-548b-3p
AGO2	hsa-miR-2290
AGO2	hsa-miR-483-3p.2
AGO2	hsa-miR-7088-3p
AGO2	hsa-miR-7108-5p
AGO2	hsa-miR-676-5p
AGO2	hsa-miR-218-1-3p
AGO2	hsa-miR-4711-5p
AGO2	hsa-miR-649
AGO2	hsa-miR-137-3p
AGO2	hsa-miR-188-5p
AGO2	hsa-miR-376abd
AGO2	hsa-miR-8887
AGO2	hsa-miR-7451-3p
AGO2	hsa-miR-1572
AGO2	hsa-miR-5116
AGO2	hsa-miR-5193
AGO2	hsa-miR-547-5p

AGO2	hsa-miR-30c-1-3p
AGO2	hsa-miR-6360
AGO2	hsa-miR-6537-5p
AGO2	hsa-miR-4314
AGO2	hsa-miR-1197
AGO2	hsa-miR-2485
AGO2	hsa-miR-219a-2-3p
AGO2	hsa-miR-6887-5p
AGO2	hsa-miR-548e-3p
AGO2	hsa-miR-8093
AGO2	hsa-miR-6400
AGO2	hsa-miR-6733-3p
AGO2	hsa-miR-6505-5p
AGO2	hsa-miR-1276
AGO2	hsa-miR-4435
AGO2	hsa-miR-106a-3p
AGO2	hsa-miR-15b-5p
AGO2	hsa-miR-107
AGO2	hsa-miR-6765-3p
AGO2	hsa-miR-188-5p
AGO2	hsa-miR-302c-3p.1
AGO2	hsa-miR-3192-5p
AGO2	hsa-miR-7000-5p
AGO2	hsa-miR-642a-5p
AGO2	hsa-miR-574-3p
AGO2	hsa-miR-2486-3p
AGO2	hsa-miR-6949-5p
AGO2	hsa-miR-144-3p
AGO2	hsa-miR-7184-5p
AGO2	hsa-miR-2284n
AGO2	hsa-miR-100-3p
AGO2	hsa-miR-6972-3p
AGO2	hsa-miR-302
AGO2	hsa-miR-2284i
AGO2	hsa-miR-3064
AGO2	hsa-miR-873-5p
AGO2	hsa-miR-2337
AGO2	hsa-miR-4296
AGO2	hsa-miR-1780
AGO2	hsa-miR-7168-3p
AGO2	hsa-miR-135a-3p
AGO2	hsa-miR-6715b-5p
AGO2	hsa-miR-520-5p
AGO2	hsa-miR-155-5p
AGO2	hsa-miR-8845
AGO2	hsa-miR-217

AGO2	hsa-miR-466l-3p
AGO2	hsa-miR-500
AGO2	hsa-miR-6862-5p
AGO2	hsa-miR-26-2-3p
AGO2	hsa-miR-1286
AGO2	hsa-miR-6715-5p
AGO2	hsa-miR-743b-5p
AGO2	hsa-miR-6676-3p
AGO2	hsa-miR-20
AGO2	hsa-miR-24-3p
AGO2	hsa-miR-29c-3p
AGO2	hsa-miR-574-5p
AGO2	hsa-miR-122-5p
AGO2	hsa-miR-7374b-3p
AGO2	hsa-miR-4259
AGO2	hsa-miR-520g-5p
AGO2	hsa-miR-7848-3p
AGO2	hsa-miR-4666a-5p
AGO2	hsa-miR-7011-5p
AGO2	hsa-miR-3147
AGO2	hsa-miR-21-5p
AGO2	hsa-miR-4716-3p
AGO2	hsa-miR-3182
AGO2	hsa-miR-9a-3p
AGO2	hsa-miR-5694
AGO2	hsa-miR-605-3p
AGO2	hsa-miR-2115-3p
AGO2	hsa-miR-4657
AGO2	hsa-miR-6417
AGO2	hsa-miR-6377
AGO2	hsa-miR-30d-5p
AGO2	hsa-miR-3083-5p
AGO2	hsa-miR-885
AGO2	hsa-miR-4768-3p
AGO2	hsa-miR-3614-5p
AGO2	hsa-miR-876
AGO2	hsa-miR-8834a
AGO2	hsa-miR-3177-3p
AGO2	hsa-miR-6654-3p
AGO2	hsa-miR-433
AGO2	hsa-miR-106
AGO2	hsa-miR-4735-5p
AGO2	hsa-miR-6563-5p
AGO2	hsa-miR-3907
AGO2	hsa-miR-7022-3p
AGO2	hsa-miR-548aw

AGO2	hsa-miR-5581-3p
AGO2	hsa-miR-7167-5p
AGO2	hsa-miR-6134
AGO2	hsa-miR-377-3p
AGO2	hsa-miR-603
AGO2	hsa-miR-549a
AGO2	hsa-miR-448-5p
AGO2	hsa-miR-6799-5p
AGO2	hsa-miR-3066-3p
AGO2	hsa-miR-2434
AGO2	hsa-miR-2284d
AGO2	hsa-miR-2297
AGO2	hsa-miR-449a
AGO2	hsa-miR-7351-5p
AGO2	hsa-miR-2309
AGO2	hsa-miR-196b-3p
AGO2	hsa-miR-7119-3p
AGO2	hsa-miR-5110
AGO2	hsa-miR-1272
AGO2	hsa-miR-4436b-5p
AGO2	hsa-miR-4760-3p
AGO2	hsa-miR-519d-5p
AGO2	hsa-miR-3909
AGO2	hsa-miR-8099
AGO2	hsa-miR-6881-5p
AGO2	hsa-miR-142b
AGO2	hsa-miR-6953-5p
AGO2	hsa-miR-7465-5p
AGO2	hsa-miR-1912-3p
AGO2	hsa-miR-3088-5p
AGO2	hsa-miR-6787-3p
AGO2	hsa-miR-290
AGO2	hsa-miR-1676-5p
AGO2	hsa-miR-7002-5p
AGO2	hsa-miR-921
AGO2	hsa-miR-4673
AGO2	hsa-miR-3164
AGO2	hsa-miR-7053-3p
AGO2	hsa-miR-8797
AGO2	hsa-miR-4999-5p
AGO2	hsa-miR-301
AGO2	hsa-miR-8818
AGO2	hsa-miR-5089-5p
AGO2	hsa-miR-2896
AGO2	hsa-miR-95
AGO2	hsa-miR-761

AGO2	hsa-miR-6526
AGO2	hsa-miR-7274-3p
AGO2	hsa-miR-700-5p
AGO2	hsa-miR-1726
AGO2	hsa-miR-190a-5p
AGO2	hsa-miR-767-5p
AGO2	hsa-miR-4753-5p
AGO2	hsa-miR-329b
AGO2	hsa-miR-192-3p
AGO2	hsa-miR-3595
AGO2	hsa-miR-920
AGO2	hsa-miR-4714-5p
AGO2	hsa-miR-5001-5p
AGO2	hsa-miR-301-3p
AGO2	hsa-miR-8071
AGO2	hsa-miR-6964-5p
AGO2	hsa-miR-7263-5p
AGO2	hsa-miR-7012-5p
AGO2	hsa-miR-452-3p
AGO2	hsa-miR-4485-3p
AGO2	hsa-miR-6959-5p
AGO2	hsa-miR-7199-5p
AGO2	hsa-miR-7388b-5p
AGO2	hsa-miR-3064-5p
AGO2	hsa-miR-3071-3p
AGO2	hsa-miR-1552-5p
AGO2	hsa-miR-6124
AGO2	hsa-miR-548y
AGO2	hsa-miR-892-5p
AGO2	hsa-miR-2116-3p
AGO2	hsa-miR-3096-3p
AGO2	hsa-miR-7670-5p
AGO2	hsa-miR-487-3p
AGO2	hsa-miR-3937
AGO2	hsa-miR-642
AGO2	hsa-miR-548bd-3p
AGO2	hsa-miR-619-5p
AGO2	hsa-miR-7472-3p
AGO2	hsa-miR-4282
AGO2	hsa-miR-7386i-5p
AGO2	hsa-miR-2379
AGO2	hsa-miR-1269a
AGO2	hsa-miR-6537-3p
AGO2	hsa-miR-8904a
AGO2	hsa-miR-466q
AGO2	hsa-miR-4686

AGO2	hsa-miR-4311
AGO2	hsa-miR-6121-5p
AGO2	hsa-miR-7113-5p
AGO2	hsa-miR-345-5p
AGO2	hsa-miR-6867-5p
AGO2	hsa-miR-1956-3p
AGO2	hsa-miR-6764-3p
AGO2	hsa-miR-7186-5p
AGO2	hsa-miR-548d-3p
AGO2	hsa-miR-7152-5p
AGO2	hsa-miR-6769b-3p
AGO2	hsa-miR-7441-3p
AGO2	hsa-miR-6081
AGO2	hsa-miR-4499
AGO2	hsa-miR-2364
AGO2	hsa-miR-1252
AGO2	hsa-miR-6851-5p
AGO2	hsa-miR-7398a-3p
AGO2	hsa-miR-6900-5p
AGO2	hsa-miR-6335
AGO2	hsa-miR-2395
AGO2	hsa-miR-616-5p
AGO2	hsa-miR-7363-5p
AGO2	hsa-miR-6784-5p
AGO2	hsa-miR-2398
AGO2	hsa-miR-6542-5p
AGO2	hsa-let-7-5p
AGO2	hsa-miR-6926-3p
AGO2	hsa-miR-2887
AGO2	hsa-miR-3103-5p
AGO2	hsa-miR-694
AGO2	hsa-miR-7361-5p
AGO2	hsa-miR-8800
AGO2	hsa-miR-3675-3p
AGO2	hsa-miR-6914-3p
AGO2	hsa-miR-6516-3p
AGO2	hsa-miR-1548-3p
AGO2	hsa-miR-1271-5p
AGO2	hsa-miR-4789-5p
AGO2	hsa-miR-5187-5p
AGO2	hsa-miR-302f
AGO2	hsa-miR-8063
AGO2	hsa-miR-4797-5p
AGO2	hsa-miR-6833-3p
AGO2	hsa-miR-3134
AGO2	hsa-miR-6963-5p

AGO2	hsa-miR-4524-5p
AGO2	hsa-miR-544a
AGO2	hsa-miR-3941
AGO2	hsa-miR-519
AGO2	hsa-miR-4502
AGO2	hsa-miR-7037-3p
AGO2	hsa-miR-299-3p
AGO2	hsa-miR-6736-5p
AGO2	hsa-miR-485
AGO2	hsa-miR-203a-5p
AGO2	hsa-miR-5009-5p
AGO2	hsa-miR-376c-3p
AGO2	hsa-miR-6922-5p
AGO2	hsa-miR-8833
AGO2	hsa-miR-561-3p
AGO2	hsa-miR-1224
AGO2	hsa-miR-6244
AGO2	hsa-miR-5619-3p
AGO2	hsa-miR-6986-3p
AGO2	hsa-miR-6740-3p
AGO2	hsa-miR-2285n
AGO2	hsa-miR-548as-5p
AGO2	hsa-miR-743-5p
AGO2	hsa-miR-7061-5p
AGO2	hsa-miR-7060-5p
AGO2	hsa-miR-1668
AGO2	hsa-miR-138-1-3p
AGO2	hsa-miR-7031-3p
AGO2	hsa-miR-6655-5p
AGO2	hsa-miR-6123
AGO2	hsa-miR-3122
AGO2	hsa-miR-6891-3p
AGO2	hsa-miR-548ba
AGO2	hsa-miR-6855-3p
AGO2	hsa-miR-1542-5p
AGO2	hsa-miR-518bc-3p
AGO2	hsa-miR-703
AGO2	hsa-miR-9500
AGO2	hsa-miR-556-5p
AGO2	hsa-miR-6412
AGO2	hsa-miR-7649-5p
AGO2	hsa-miR-186-3p
AGO2	hsa-miR-4759
AGO2	hsa-miR-196-2-3p
AGO2	hsa-miR-4464
AGO2	hsa-miR-690

AGO2	hsa-miR-6807-5p
AGO2	hsa-miR-125-5p
AGO2	hsa-miR-29b-5p
AGO2	hsa-miR-200bc
AGO2	hsa-miR-2421
AGO2	hsa-miR-8107
AGO2	hsa-miR-7193-3p
AGO2	hsa-miR-514b-5p
AGO2	hsa-miR-513a-3p
AGO2	hsa-miR-8860
AGO2	hsa-miR-7231-5p
AGO2	hsa-miR-4635
AGO2	hsa-miR-8824
AGO2	hsa-miR-5107-3p
AGO2	hsa-miR-2470
AGO2	hsa-miR-3067-5p
AGO2	hsa-miR-7192-5p
AGO2	hsa-miR-6396
AGO2	hsa-miR-666-3p
AGO2	hsa-miR-132-5p
AGO2	hsa-miR-1456-5p
AGO2	hsa-miR-6826-3p
AGO2	hsa-miR-7243-5p
AGO2	hsa-miR-7277-5p
AGO2	hsa-miR-6357
AGO2	hsa-miR-660-5p
AGO2	hsa-miR-7083-5p
AGO2	hsa-miR-20a-5p
AGO2	hsa-miR-548ah-3p
AGO2	hsa-miR-376a-3p
AGO2	hsa-miR-5692bc
AGO2	hsa-miR-6947-3p
AGO2	hsa-miR-6809-3p
AGO2	hsa-miR-6778-3p
AGO2	hsa-miR-7284-3p
AGO2	hsa-miR-651-3p
AGO2	hsa-miR-7386f-3p
AGO2	hsa-miR-7220-3p
AGO2	hsa-miR-608
AGO2	hsa-miR-7453-5p
AGO2	hsa-miR-145-3p
AGO2	hsa-miR-3084-3p
AGO2	hsa-miR-2284-5p
AGO2	hsa-miR-7168-5p
AGO2	hsa-miR-5113
AGO2	hsa-miR-1841

AGO2	hsa-miR-409-3p
AGO2	hsa-miR-6989-3p
AGO2	hsa-miR-6831-5p
AGO2	hsa-miR-551
AGO2	hsa-let-7ac-3p
AGO2	hsa-miR-1237-5p
AGO2	hsa-miR-4716-5p
AGO2	hsa-miR-3059-5p
AGO2	hsa-miR-4700-5p
AGO2	hsa-miR-3525
AGO2	hsa-miR-2407
AGO2	hsa-miR-323b-5p
AGO2	hsa-miR-3664-5p
AGO2	hsa-miR-3619-5p
AGO2	hsa-miR-184
AGO2	hsa-miR-203-3p
AGO2	hsa-miR-6798-5p
AGO2	hsa-miR-770-3p
AGO2	hsa-miR-466f
AGO2	hsa-miR-2136
AGO2	hsa-miR-3659
AGO2	hsa-miR-5589-5p
AGO2	hsa-miR-6391
AGO2	hsa-miR-6955-3p
AGO2	hsa-miR-154c
AGO2	hsa-miR-466b-4-3p
AGO2	hsa-miR-6877-3p
AGO2	hsa-miR-1470
AGO2	hsa-miR-183-5p
AGO2	hsa-miR-1981-5p
AGO2	hsa-miR-206-3p
AGO2	hsa-miR-6868-5p
AGO2	hsa-miR-6414
AGO2	hsa-let-7b-5p
AGO2	hsa-miR-548d-5p
AGO2	hsa-miR-2388-3p
AGO2	hsa-miR-2310
AGO2	hsa-miR-1968-5p
AGO2	hsa-miR-7009-3p
AGO2	hsa-miR-216b-3p
AGO2	hsa-miR-181-5p
AGO2	hsa-miR-4726-3p
AGO2	hsa-miR-6578-5p
AGO2	hsa-miR-673-5p
AGO2	hsa-miR-5585-3p
AGO2	hsa-miR-4473

AGO2	hsa-miR-300-3p
AGO2	hsa-miR-4425
AGO2	hsa-miR-7289-3p
AGO2	hsa-miR-1953
AGO2	hsa-miR-325-3p
AGO2	hsa-miR-1764-5p
AGO2	hsa-miR-559
AGO2	hsa-miR-6984-5p
AGO2	hsa-miR-6680-3p
AGO2	hsa-miR-1304-5p
AGO2	hsa-miR-2450bd
AGO2	hsa-miR-7021-3p
AGO2	hsa-miR-7371g-5p
AGO2	hsa-miR-7386h-3p
AGO2	hsa-miR-124-3p
AGO2	hsa-miR-6791-3p
AGO2	hsa-miR-8873a
AGO2	hsa-miR-196-5p
AGO2	hsa-miR-4743-5p
AGO2	hsa-miR-7027-5p
AGO2	hsa-miR-589-5p
AGO2	hsa-miR-7482-3p
AGO2	hsa-miR-3578
AGO2	hsa-miR-6127
AGO2	hsa-miR-7109-3p
AGO2	hsa-miR-669i
AGO2	hsa-miR-3112-5p
AGO2	hsa-miR-6906-5p
AGO2	hsa-miR-2400
AGO2	hsa-miR-2347
AGO2	hsa-miR-7232-5p
AGO2	hsa-miR-505-3p.1
AGO2	hsa-miR-1231
AGO2	hsa-miR-3913-5p
AGO2	hsa-miR-323b-3p
AGO2	hsa-miR-3556a
AGO2	hsa-miR-124
AGO2	hsa-miR-1972
AGO2	hsa-miR-605
AGO2	hsa-miR-149-3p
AGO2	hsa-miR-338-3p
AGO2	hsa-miR-7207-3p
AGO2	hsa-miR-5587-5p
AGO2	hsa-miR-4662a-3p
AGO2	hsa-miR-580-3p
AGO2	hsa-miR-4504

AGO2	hsa-miR-6766-5p
AGO2	hsa-miR-6529-5p
AGO2	hsa-miR-3968
AGO2	hsa-miR-4758-3p
AGO2	hsa-miR-450a
AGO2	hsa-miR-421-5p
AGO2	hsa-miR-7323-3p
AGO2	hsa-miR-6779-3p
AGO2	hsa-miR-548as-3p
AGO2	hsa-miR-7686-5p
AGO2	hsa-miR-5624-5p
AGO2	hsa-miR-2681-5p
AGO2	hsa-miR-7335-3p
AGO2	hsa-miR-424-5p
AGO2	hsa-miR-154a
AGO2	hsa-miR-665
AGO2	hsa-miR-3680-3p
AGO2	hsa-miR-6783-5p
AGO2	hsa-miR-144-3p
AGO2	hsa-miR-449a-3p
AGO2	hsa-miR-8069
AGO2	hsa-miR-130ac-5p
AGO2	hsa-miR-7688-3p
AGO2	hsa-miR-6893-5p
AGO2	hsa-miR-429
AGO2	hsa-miR-134-5p
AGO2	hsa-miR-93-5p
AGO2	hsa-miR-5007-5p
AGO2	hsa-miR-548ay-5p
AGO2	hsa-miR-23b-3p
AGO2	hsa-miR-7481-5p
AGO2	hsa-miR-4283
AGO2	hsa-miR-1564-5p
AGO2	hsa-miR-2292
AGO2	hsa-miR-6958-3p
AGO2	hsa-miR-204-3p
AGO2	hsa-let-7g-5p
AGO2	hsa-miR-7398l-3p
AGO2	hsa-miR-1712-3p
AGO2	hsa-miR-4749-3p
AGO2	hsa-miR-4529-5p
AGO2	hsa-miR-501-5p
AGO2	hsa-let-7d-3p
AGO2	hsa-miR-1388-3p
AGO2	hsa-miR-1812-5p
AGO2	hsa-miR-2303

AGO2	hsa-miR-1929-3p
AGO2	hsa-miR-4305
AGO2	hsa-miR-190-3p
AGO2	hsa-miR-3926
AGO2	hsa-miR-6878-5p
AGO2	hsa-miR-892b
AGO2	hsa-miR-7348-5p
AGO2	hsa-miR-130a-5p
AGO2	hsa-miR-1250
AGO2	hsa-miR-8803
AGO2	hsa-miR-4253
AGO2	hsa-miR-6870-3p
AGO2	hsa-miR-151a-3p
AGO2	hsa-miR-943
AGO2	hsa-miR-3089-3p
AGO2	hsa-miR-1913
AGO2	hsa-miR-8869
AGO2	hsa-miR-301b-5p
AGO2	hsa-miR-423-5p
AGO2	hsa-miR-1224-3p
AGO2	hsa-miR-4649-3p
AGO2	hsa-miR-6746-5p
AGO2	hsa-miR-7398p-3p
AGO2	hsa-miR-6992-3p
AGO2	hsa-miR-1260b
AGO2	hsa-miR-4794
AGO2	hsa-miR-7195-5p
AGO2	hsa-miR-1298-5p
AGO2	hsa-miR-741-3p
AGO2	hsa-miR-6986-5p
AGO2	hsa-miR-2882
AGO2	hsa-miR-4433b-5p
AGO2	hsa-miR-883ab-3p
AGO2	hsa-miR-375-3p
AGO2	hsa-miR-450a-3p
AGO2	hsa-miR-4717-3p
AGO2	hsa-miR-7045-3p
AGO2	hsa-miR-103-5p
AGO2	hsa-miR-6811-3p
AGO2	hsa-miR-3162-5p
AGO2	hsa-miR-6801-3p
AGO2	hsa-miR-7193-5p
AGO2	hsa-miR-2335
AGO2	hsa-miR-6509-5p
AGO2	hsa-miR-342-5p
AGO2	hsa-miR-7865

AGO2	hsa-miR-548at-3p
AGO2	hsa-miR-1597-5p
AGO2	hsa-miR-570-3p
AGO2	hsa-miR-1188-3p
AGO2	hsa-miR-2114-5p
AGO2	hsa-miR-1233-3p
AGO2	hsa-miR-1595-5p
AGO2	hsa-miR-1663-5p
AGO2	hsa-miR-1661
AGO2	hsa-miR-1915-5p
AGO2	hsa-miR-1208
AGO2	hsa-miR-6896-3p
AGO2	hsa-miR-331-5p
AGO2	hsa-miR-551b-3p
AGO2	hsa-miR-1279
AGO2	hsa-miR-5132-5p
AGO2	hsa-miR-508a
AGO2	hsa-miR-2459
AGO2	hsa-miR-4722-5p
AGO2	hsa-miR-5680
AGO2	hsa-miR-1796
AGO2	hsa-miR-5580-5p
AGO2	hsa-miR-8082
AGO2	hsa-let-7f-5p
AGO2	hsa-miR-4633-5p
AGO2	hsa-miR-6509-3p
AGO2	hsa-miR-5627-3p
AGO2	hsa-miR-7650-3p
AGO2	hsa-miR-673-3p
AGO2	hsa-miR-6761-5p
AGO2	hsa-miR-6771-3p
AGO2	hsa-miR-1909-3p
AGO2	hsa-miR-7190-5p
AGO2	hsa-miR-6842-3p
AGO2	hsa-miR-1256
AGO2	hsa-miR-4256
AGO2	hsa-miR-302a-3p
AGO2	hsa-miR-6941-5p
AGO2	hsa-miR-7047-5p
AGO2	hsa-miR-7301-5p
AGO2	hsa-miR-3674
AGO2	hsa-miR-195-5p
AGO2	hsa-miR-7663-3p
AGO2	hsa-miR-7158-5p
AGO2	hsa-miR-427
AGO2	hsa-miR-7371f-5p

AGO2	hsa-miR-628-5p
AGO2	hsa-miR-6728-3p
AGO2	hsa-miR-3536
AGO2	hsa-miR-7386e-3p
AGO2	hsa-miR-6821-3p
AGO2	hsa-miR-680
AGO2	hsa-miR-3470a
AGO2	hsa-miR-4690-3p
AGO2	hsa-miR-6928-5p
AGO2	hsa-miR-4645-3p
AGO2	hsa-miR-7398-3p
AGO2	hsa-miR-6932-3p
AGO2	hsa-miR-654-5p
AGO2	hsa-miR-7447-3p
AGO2	hsa-miR-6314
AGO2	hsa-miR-7068-3p
AGO2	hsa-miR-3580-3p
AGO2	hsa-miR-6522
AGO2	hsa-miR-7059-3p
AGO2	hsa-miR-585-5p
AGO2	hsa-miR-2462
AGO2	hsa-miR-291b-3p
AGO2	hsa-miR-1197-5p
AGO2	hsa-miR-3199
AGO2	hsa-miR-6828-5p
AGO2	hsa-miR-4710
AGO2	hsa-miR-1546-5p
AGO2	hsa-miR-2332
AGO2	hsa-miR-939-5p
AGO2	hsa-miR-7003-3p
AGO2	hsa-miR-2885
AGO2	hsa-miR-520a-3p
AGO2	hsa-miR-1273g-5p
AGO2	hsa-miR-1564-3p
AGO2	hsa-miR-7025-3p
AGO2	hsa-miR-8810
AGO2	hsa-miR-299a-5p
AGO2	hsa-miR-3667-3p
AGO2	hsa-miR-1305
AGO2	hsa-miR-661
AGO2	hsa-miR-466f-3p
AGO2	hsa-miR-6625-5p
AGO2	hsa-miR-4709-3p
AGO2	hsa-miR-589-3p
AGO2	hsa-miR-33-3p
AGO2	hsa-miR-2376

AGO2	hsa-miR-1745
AGO2	hsa-miR-3965
AGO2	hsa-miR-1774
AGO2	hsa-miR-3613b
AGO2	hsa-miR-2362
AGO2	hsa-miR-2861
AGO2	hsa-miR-34b-5p
AGO2	hsa-miR-380
AGO2	hsa-miR-6523b
AGO2	hsa-miR-6765-5p
AGO2	hsa-miR-6656-5p
AGO2	hsa-miR-3973
AGO2	hsa-miR-187-3p
AGO2	hsa-miR-2315
AGO2	hsa-miR-6685-5p
AGO2	hsa-miR-2464-3p
AGO2	hsa-miR-493-3p
AGO2	hsa-miR-5739
AGO2	hsa-miR-7234-5p
AGO2	hsa-miR-3068-3p
AGO2	hsa-miR-1948-3p
AGO2	hsa-miR-8817
AGO2	hsa-miR-195-3p
AGO2	hsa-miR-595
AGO2	hsa-miR-767
AGO2	hsa-miR-6519
AGO2	hsa-miR-5010-3p
AGO2	hsa-miR-450a-2-3p
AGO2	hsa-miR-539-5p
AGO2	hsa-miR-1273d
AGO2	hsa-miR-3591
AGO2	hsa-miR-490-3p
AGO2	hsa-miR-6998-5p
AGO2	hsa-miR-7208-3p
AGO2	hsa-miR-485-5p
AGO2	hsa-miR-7656-5p
AGO2	hsa-miR-299
AGO2	hsa-miR-128-3p
AGO2	hsa-miR-7207-5p
AGO2	hsa-miR-93-3p
AGO2	hsa-miR-491-5p
AGO2	hsa-miR-8875
AGO2	hsa-miR-6906-3p
AGO2	hsa-miR-6073
AGO2	hsa-miR-510-3p
AGO2	hsa-miR-4646-5p

AGO2	hsa-miR-1555-5p
AGO2	hsa-miR-8863
AGO2	hsa-miR-190a-3p
AGO2	hsa-miR-7050-3p
AGO2	hsa-miR-891b
AGO2	hsa-miR-1911-3p
AGO2	hsa-miR-4720-3p
AGO2	hsa-miR-625-5p
AGO2	hsa-miR-378d
AGO2	hsa-miR-1284
AGO2	hsa-miR-7048-5p
AGO2	hsa-miR-670
AGO2	hsa-miR-7669-5p
AGO2	hsa-miR-369-3p
AGO2	hsa-miR-216a-3p
AGO2	hsa-miR-3189-5p
AGO2	hsa-miR-6745
AGO2	hsa-miR-451b
AGO2	hsa-miR-7386j-5p
AGO2	hsa-miR-8791
AGO2	hsa-miR-3588
AGO2	hsa-miR-329
AGO2	hsa-miR-6596-3p
AGO2	hsa-miR-548ad-5p
AGO2	hsa-miR-518d-5p
AGO2	hsa-miR-548ae-3p
AGO2	hsa-miR-3910
AGO2	hsa-miR-152-5p
AGO2	hsa-miR-448-3p
AGO2	hsa-miR-29a-3p
AGO2	hsa-miR-200bc-3p
AGO2	hsa-miR-1677-3p
AGO2	hsa-miR-15a-3p
AGO2	hsa-miR-6329
AGO2	hsa-miR-2405
AGO2	hsa-miR-202-3p
AGO2	hsa-miR-4452
AGO2	hsa-miR-3082-3p
AGO2	hsa-miR-669c-3p
AGO2	hsa-miR-4420
AGO2	hsa-miR-646
AGO2	hsa-miR-548w
AGO2	hsa-miR-6627-3p
AGO2	hsa-miR-552
AGO2	hsa-miR-411-5p.2
AGO2	hsa-miR-6815-5p

AGO2	hsa-miR-1976
AGO2	hsa-miR-1912
AGO2	hsa-miR-329-3p
AGO2	hsa-miR-6825-5p
AGO2	hsa-miR-203
AGO2	hsa-miR-8852
AGO2	hsa-miR-106-5p
AGO2	hsa-miR-5626-3p
AGO2	hsa-miR-181a-2-3p
AGO2	hsa-miR-4459
AGO2	hsa-miR-7197-3p
AGO2	hsa-miR-7302-3p
AGO2	hsa-miR-219-3p
AGO2	hsa-miR-295-3p
AGO2	hsa-miR-7079-3p
AGO2	hsa-miR-6839-5p
AGO2	hsa-miR-18
AGO2	hsa-miR-664a-3p
AGO2	hsa-miR-7075-5p
AGO2	hsa-miR-6817-5p
AGO2	hsa-miR-1933-5p
AGO2	hsa-miR-3079-3p
AGO2	hsa-miR-3185
AGO2	hsa-miR-607
AGO2	hsa-miR-7294-5p
AGO2	hsa-miR-548u
AGO2	hsa-miR-548ae-5p
AGO2	hsa-miR-6729-5p
AGO2	hsa-miR-8847
AGO2	hsa-miR-4723-5p
AGO2	hsa-miR-4279
AGO2	hsa-miR-328-3p
AGO2	hsa-miR-8874
AGO2	hsa-miR-7253-5p
AGO2	hsa-miR-6777-5p
AGO2	hsa-miR-7286-5p
AGO2	hsa-miR-3106-3p
AGO2	hsa-miR-6981-3p
AGO2	hsa-miR-6375
AGO2	hsa-miR-1551-3p
AGO2	hsa-miR-3573-5p
AGO2	hsa-miR-8120
AGO2	hsa-miR-376e
AGO2	hsa-miR-1285-3p
AGO2	hsa-miR-1717
AGO2	hsa-miR-7383-5p

AGO2	hsa-miR-4489
AGO2	hsa-miR-5707
AGO2	hsa-miR-29e
AGO2	hsa-miR-8895
AGO2	hsa-miR-410-3p
AGO2	hsa-miR-7680-5p
AGO2	hsa-miR-292b-5p
AGO2	hsa-miR-3078-5p
AGO2	hsa-miR-3619-3p
AGO2	hsa-miR-2888
AGO2	hsa-miR-200b-3p
AGO2	hsa-miR-1963
AGO2	hsa-miR-4520-5p
AGO2	hsa-miR-6533
AGO2	hsa-miR-6902-3p
AGO2	hsa-miR-139-5p
AGO2	hsa-miR-3524-3p
AGO2	hsa-miR-7185-5p
AGO2	hsa-miR-365a-3p
AGO2	hsa-miR-2286
AGO2	hsa-miR-7296-5p
AGO2	hsa-miR-544-3p
AGO2	hsa-miR-7279-3p
AGO2	hsa-miR-335-5p
AGO2	hsa-miR-2444
AGO2	hsa-miR-3925-5p
AGO2	hsa-miR-616-3p
AGO2	hsa-miR-3613-3p
AGO2	hsa-miR-6808-3p
AGO2	hsa-miR-1658-3p
AGO2	hsa-miR-599-5p
AGO2	hsa-miR-7272-3p
AGO2	hsa-miR-7221-5p
AGO2	hsa-miR-3156-3p
AGO2	hsa-miR-7446-5p
AGO2	hsa-miR-3664-3p
AGO2	hsa-miR-548am-5p
AGO2	hsa-miR-376ab-3p
AGO2	hsa-miR-7110-5p
AGO2	hsa-miR-5192
AGO2	hsa-miR-6701-3p
AGO2	hsa-miR-6868-3p
AGO2	hsa-miR-383-5p.2
AGO2	hsa-miR-7381-3p
AGO2	hsa-miR-201-3p
AGO2	hsa-miR-548aq-3p

AGO2	hsa-miR-7668-3p
AGO2	hsa-miR-4800-5p
AGO2	hsa-miR-6575-3p
AGO2	hsa-miR-7377-5p
AGO2	hsa-miR-8905
AGO2	hsa-miR-7092-3p
AGO2	hsa-miR-4494
AGO2	hsa-miR-8060
AGO2	hsa-miR-124a-5p
AGO2	hsa-miR-7278-5p
AGO2	hsa-miR-873-5p.1
AGO2	hsa-miR-7106-3p
AGO2	hsa-miR-1343-5p
AGO2	hsa-miR-1593
AGO2	hsa-miR-514a-3p
AGO2	hsa-miR-6737-3p
AGO2	hsa-miR-7070-5p
AGO2	hsa-miR-19a-3p
AGO2	hsa-miR-6649-5p
AGO2	hsa-miR-6962-5p
AGO2	hsa-miR-6531
AGO2	hsa-miR-1646
AGO2	hsa-miR-3575
AGO2	hsa-miR-7364-5p
AGO2	hsa-miR-7067-5p
AGO2	hsa-miR-3539
AGO2	hsa-miR-7056-5p
AGO2	hsa-miR-350
AGO2	hsa-miR-875-3p
AGO2	hsa-miR-6631-5p
AGO2	hsa-miR-7301-3p
AGO2	hsa-miR-454
AGO2	hsa-miR-1250-5p
AGO2	hsa-miR-301b-3p
AGO2	hsa-miR-6663-5p
AGO2	hsa-miR-1229-3p
AGO2	hsa-miR-3120-5p
AGO2	hsa-miR-4519
AGO2	hsa-miR-129-5p
AGO2	hsa-miR-3473f
AGO2	hsa-miR-204-5p
AGO2	hsa-miR-5197-5p
AGO2	hsa-miR-326-3p
AGO2	hsa-miR-6518
AGO2	hsa-miR-2422
AGO2	hsa-miR-576-3p

AGO2	hsa-miR-4427
AGO2	hsa-miR-20b-5p
AGO2	hsa-miR-548ag
AGO2	hsa-miR-4441
AGO2	hsa-miR-570-3p
AGO2	hsa-miR-3102-5p
AGO2	hsa-miR-92a-3p
AGO2	hsa-miR-7665-5p
AGO2	hsa-miR-655
AGO2	hsa-miR-1397-5p
AGO2	hsa-miR-8853
AGO2	hsa-miR-208-5p
AGO2	hsa-miR-1454
AGO2	hsa-miR-146-5p
AGO2	hsa-miR-7015-3p
AGO2	hsa-miR-1231-3p
AGO2	hsa-miR-4792
AGO2	hsa-miR-6939-5p
AGO2	hsa-miR-7371j-5p
AGO2	hsa-miR-295-5p
AGO2	hsa-miR-520g-3p
AGO2	hsa-miR-6715a-3p
AGO2	hsa-miR-579
AGO2	hsa-miR-2383
AGO2	hsa-miR-1641
AGO2	hsa-miR-3915
AGO2	hsa-miR-145-5p
AGO2	hsa-miR-302-3p
AGO2	hsa-miR-2331-3p
AGO2	hsa-miR-6500-5p
AGO2	hsa-miR-3109-3p
AGO2	hsa-miR-5709-3p
AGO2	hsa-miR-3117-3p
AGO2	hsa-miR-4290
AGO2	hsa-miR-7026-3p
AGO2	hsa-miR-362-3p
AGO2	hsa-miR-7072-5p
AGO2	hsa-miR-1809
AGO2	hsa-miR-8802
AGO2	hsa-miR-217-5p
AGO2	hsa-miR-216a
AGO2	hsa-miR-29b-3p
AGO2	hsa-miR-7241-5p
AGO2	hsa-miR-342-3p
AGO2	hsa-miR-181d-3p
AGO2	hsa-miR-3682-5p

AGO2	hsa-miR-29c-5p
AGO2	hsa-miR-7072-3p
AGO2	hsa-miR-320e
AGO2	hsa-miR-2365
AGO2	hsa-miR-4754
AGO2	hsa-miR-302c-3p.2
AGO2	hsa-miR-552-3p
AGO2	hsa-miR-3061-5p
AGO2	hsa-miR-15c-3p
AGO2	hsa-miR-2302
AGO2	hsa-miR-2285s
AGO2	hsa-miR-7184-3p
AGO2	hsa-miR-3658
AGO2	hsa-miR-3596b
AGO2	hsa-miR-130c-5p
AGO2	hsa-miR-339-5p
AGO2	hsa-miR-2284ab
AGO2	hsa-miR-6904-5p
AGO2	hsa-miR-2327
AGO2	hsa-miR-6846-3p
AGO2	hsa-miR-3145-5p
AGO2	hsa-miR-377-3p
AGO2	hsa-miR-8826
AGO2	hsa-miR-878
AGO2	hsa-miR-548f-3p
AGO2	hsa-miR-3530-5p
AGO2	hsa-miR-100-5p
AGO2	hsa-miR-1934-3p
AGO2	hsa-miR-548aef
AGO2	hsa-miR-6917-5p
AGO2	hsa-miR-361-5p
AGO2	hsa-miR-581
AGO2	hsa-miR-548ar-5p
AGO2	hsa-miR-8880
AGO2	hsa-miR-3148
AGO2	hsa-miR-7679-3p
AGO2	hsa-miR-6726-5p
AGO2	hsa-miR-7079-5p
AGO2	hsa-miR-4260
AGO2	hsa-miR-3058-3p
AGO2	hsa-miR-7678-5p
AGO2	hsa-miR-3110-5p
AGO2	hsa-miR-7480-5p
AGO2	hsa-miR-301a-3p
AGO2	hsa-miR-2284r
AGO2	hsa-miR-302d-5p

AGO2	hsa-miR-101a-3p.2
AGO2	hsa-miR-548t-3p
AGO2	hsa-miR-7450-5p
AGO2	hsa-miR-7160-5p
AGO2	hsa-miR-6741-5p
AGO2	hsa-miR-6216
AGO2	hsa-miR-6344
AGO2	hsa-miR-323-3p
AGO2	hsa-miR-8114
AGO2	hsa-miR-4715-5p
AGO2	hsa-miR-3092-5p
AGO2	hsa-miR-944
AGO2	hsa-miR-3544-5p
AGO2	hsa-miR-378-3p
AGO2	hsa-miR-6872-3p
AGO2	hsa-miR-135b-5p
AGO2	hsa-miR-6804-5p
AGO2	hsa-miR-6566-3p
AGO2	hsa-miR-7078-3p
AGO2	hsa-miR-4786-5p
AGO2	hsa-miR-1675
AGO2	hsa-miR-572
AGO2	hsa-miR-199b-2-5p
AGO2	hsa-miR-27-5p
AGO2	hsa-miR-7187-3p
AGO2	hsa-miR-7151-5p
AGO2	hsa-miR-1388-5p
AGO2	hsa-miR-6900-3p
AGO2	hsa-miR-6398
AGO2	hsa-miR-6597-3p
AGO2	hsa-miR-8804
AGO2	hsa-miR-6823-5p
AGO2	hsa-miR-1948-5p
AGO2	hsa-miR-6779-5p
AGO2	hsa-miR-301a-5p
AGO2	hsa-miR-1262
AGO2	hsa-miR-619-3p
AGO2	hsa-miR-195-5p
AGO2	hsa-miR-3151-5p
AGO2	hsa-miR-6562-5p
AGO2	hsa-miR-7285-3p
AGO2	hsa-miR-4785
AGO2	hsa-miR-4724-5p
AGO2	hsa-miR-192-5p
AGO2	hsa-miR-6798-3p
AGO2	hsa-miR-625-3p

AGO2	hsa-miR-664a-5p
AGO2	hsa-miR-7386m-3p
AGO2	hsa-miR-1837
AGO2	hsa-miR-6781-5p
AGO2	hsa-miR-7444-5p
AGO2	hsa-miR-126-3p
AGO2	hsa-miR-6943-3p
AGO2	hsa-miR-7224-3p
AGO2	hsa-miR-1287-5p
AGO2	hsa-miR-504-5p.1
AGO2	hsa-miR-489-5p
AGO2	hsa-miR-2361
AGO2	hsa-miR-7065-5p
AGO2	hsa-miR-4748
AGO2	hsa-miR-4769-5p
AGO2	hsa-miR-7397-5p
AGO2	hsa-miR-518a-5p
AGO2	hsa-miR-367-3p
AGO2	hsa-miR-3069-5p
AGO2	hsa-miR-1969
AGO2	hsa-miR-7975
AGO2	hsa-miR-679
AGO2	hsa-miR-2284j
AGO2	hsa-miR-1299
AGO2	hsa-miR-7676-5p
AGO2	hsa-miR-206-5p
AGO2	hsa-miR-937-5p
AGO2	hsa-miR-125a-3p
AGO2	hsa-miR-2486-5p
AGO2	hsa-miR-1771
AGO2	hsa-miR-548t-5p
AGO2	hsa-miR-3653-3p
AGO2	hsa-miR-6732-3p
AGO2	hsa-miR-2115-5p
AGO2	hsa-miR-198
AGO2	hsa-miR-1246
AGO2	hsa-miR-8094
AGO2	hsa-miR-3543
AGO2	hsa-miR-6556-5p
AGO2	hsa-miR-30b-3p
AGO2	hsa-miR-4327
AGO2	hsa-miR-4653-5p
AGO2	hsa-miR-466b-2-3p
AGO2	hsa-miR-219-5p
AGO2	hsa-miR-3591-5p
AGO2	hsa-miR-2285r

AGO2	hsa-miR-8868
AGO2	hsa-miR-4643
AGO2	hsa-miR-4687-5p
AGO2	hsa-miR-3173-3p
AGO2	hsa-miR-619
AGO2	hsa-miR-3978
AGO2	hsa-miR-5134-5p
AGO2	hsa-miR-8862
AGO2	hsa-miR-30--3p
AGO2	hsa-miR-3592
AGO2	hsa-miR-1251-3p
AGO2	hsa-miR-30-3p
AGO2	hsa-miR-1231-5p
AGO2	hsa-miR-7388a-5p
AGO2	hsa-miR-1453
AGO2	hsa-miR-1255b-5p
AGO2	hsa-let-7c-3p
AGO2	hsa-miR-588
AGO2	hsa-miR-4638-3p
AGO2	hsa-miR-4310
AGO2	hsa-miR-33-5p
AGO2	hsa-miR-3657
AGO2	hsa-miR-7646-5p
AGO2	hsa-miR-6734-3p
AGO2	hsa-miR-7659-3p
AGO2	hsa-miR-1981-3p
AGO2	hsa-miR-5135
AGO2	hsa-miR-4691-3p
AGO2	hsa-miR-548ad-3p
AGO2	hsa-miR-4457
AGO2	hsa-miR-7685-5p
AGO2	hsa-miR-4516
AGO2	hsa-miR-338-5p
AGO2	hsa-miR-302e
AGO2	hsa-miR-1323-3p
AGO2	hsa-miR-3616-5p
AGO2	hsa-miR-7704
AGO2	hsa-miR-6408
AGO2	hsa-miR-1250-3p
AGO2	hsa-miR-7245-3p
AGO2	hsa-miR-4266
AGO2	hsa-miR-650abc
AGO2	hsa-miR-23a-3p
AGO2	hsa-miR-8831
AGO2	hsa-miR-1681
AGO2	hsa-miR-6758-3p

AGO2	hsa-miR-2285aa
AGO2	hsa-miR-6572-3p
AGO2	hsa-miR-6837-5p
AGO2	hsa-miR-29
AGO2	hsa-miR-215-3p
AGO2	hsa-miR-1185-5p
AGO2	hsa-miR-1947-5p
AGO2	hsa-miR-1180-5p
AGO2	hsa-miR-3927-3p
AGO2	hsa-miR-4540
AGO2	hsa-miR-1302
AGO2	hsa-miR-6921-3p
AGO2	hsa-miR-421-3p
AGO2	hsa-miR-6859-5p
AGO2	hsa-miR-8883
AGO2	hsa-miR-5705
AGO2	hsa-miR-103
AGO2	hsa-miR-8843
AGO2	hsa-miR-3551-3p
AGO2	hsa-miR-8870
AGO2	hsa-miR-6780a-5p
AGO2	hsa-miR-5104
AGO2	hsa-miR-1553-5p
AGO2	hsa-miR-33
AGO2	hsa-miR-6215
AGO2	hsa-miR-548n
AGO2	hsa-miR-8884
AGO2	hsa-miR-6804-3p
AGO2	hsa-miR-543-3p
AGO2	hsa-miR-514a-5p
AGO2	hsa-miR-6819-3p
AGO2	hsa-miR-7316-5p
AGO2	hsa-miR-380-5p
AGO2	hsa-miR-2285ad
AGO2	hsa-miR-4476
AGO2	hsa-miR-422
AGO2	hsa-miR-371a-3p
AGO2	hsa-miR-7199-3p
AGO2	hsa-miR-4685-5p
AGO2	hsa-miR-6903-3p
AGO2	hsa-miR-885-3p
AGO2	hsa-miR-7371e-5p
AGO2	hsa-miR-383-5p
AGO2	hsa-miR-4270
AGO2	hsa-miR-9769-3p
AGO2	hsa-miR-1736-3p

AGO2	hsa-miR-7652-5p
AGO2	hsa-miR-93-5p
AGO2	hsa-miR-466f-5p
AGO2	hsa-miR-7282-3p
AGO2	hsa-miR-4760-5p
AGO2	hsa-miR-2368-3p
AGO2	hsa-miR-5682
AGO2	hsa-miR-1569
AGO2	hsa-miR-2446
AGO2	hsa-miR-7244-5p
AGO2	hsa-miR-6962-3p
AGO2	hsa-miR-1804
AGO2	hsa-miR-7013-5p
AGO2	hsa-miR-1752
AGO2	hsa-miR-3531-3p
AGO2	hsa-miR-1237-3p
AGO2	hsa-miR-1462-5p
AGO2	hsa-miR-3190-3p
AGO2	hsa-miR-4700-3p
AGO2	hsa-miR-4424
AGO2	hsa-miR-1964-5p
AGO2	hsa-miR-1625-3p
AGO2	hsa-miR-7320-3p
AGO2	hsa-miR-6607-5p
AGO2	hsa-miR-4727-5p
AGO2	hsa-miR-873a-5p.1
AGO2	hsa-miR-7324-5p
AGO2	hsa-miR-4668-3p
AGO2	hsa-miR-664-3p
AGO2	hsa-miR-6700-3p
AGO2	hsa-miR-6877-5p
AGO2	hsa-miR-5701
AGO2	hsa-miR-3102
AGO2	hsa-miR-5685
AGO2	hsa-miR-7398n-5p
AGO2	hsa-miR-6624-3p
AGO2	hsa-miR-6132
AGO2	hsa-miR-7177-5p
AGO2	hsa-miR-2469
AGO2	hsa-miR-124-5p
AGO2	hsa-miR-558
AGO2	hsa-miR-103a-3p
AGO2	hsa-miR-548ar-3p
AGO2	hsa-miR-2425-5p
AGO2	hsa-miR-29-5p
AGO2	hsa-miR-4533

AGO2	hsa-miR-7396-5p
AGO2	hsa-miR-2377
AGO2	hsa-miR-5094
AGO2	hsa-miR-874
AGO2	hsa-miR-34a-3p
AGO2	hsa-miR-143-3p
AGO2	hsa-miR-7019-5p
AGO2	hsa-miR-329-5p
AGO2	hsa-miR-449-5p
AGO2	hsa-miR-7398b-3p
AGO2	hsa-miR-7248-5p
AGO2	hsa-miR-520f-5p
AGO2	hsa-miR-8110
AGO2	hsa-miR-6507-3p
AGO2	hsa-miR-643
AGO2	hsa-miR-30a-3p
AGO2	hsa-miR-28-5p
AGO2	hsa-miR-95-5p
AGO2	hsa-miR-1178-5p
AGO2	hsa-miR-7385e-3p
AGO2	hsa-miR-1545b-3p
AGO2	hsa-miR-1248
AGO2	hsa-miR-3175
AGO2	hsa-miR-4660
AGO2	hsa-miR-1295b-5p
AGO2	hsa-miR-26a-3p
AGO2	hsa-miR-3944-3p
AGO2	hsa-miR-7162-5p
AGO2	hsa-miR-1682
AGO2	hsa-miR-410
AGO2	hsa-miR-4667
AGO2	hsa-miR-7235-3p
AGO2	hsa-miR-8112
AGO2	hsa-miR-3689d
AGO2	hsa-miR-460a-5p
AGO2	hsa-miR-26-3p
AGO2	hsa-miR-711
AGO2	hsa-miR-6918-5p
AGO2	hsa-miR-7086-5p
AGO2	hsa-miR-425
AGO2	hsa-miR-3942-3p
AGO2	hsa-miR-4509
AGO2	hsa-miR-4670-3p
AGO2	hsa-miR-669h-5p
AGO2	hsa-miR-660-3p
AGO2	hsa-miR-302bd

AGO2	hsa-miR-651
AGO2	hsa-miR-5627-5p
AGO2	hsa-miR-7392-3p
AGO2	hsa-miR-147-5p
AGO2	hsa-miR-4667-3p
AGO2	hsa-miR-4731-3p
AGO2	hsa-miR-6615-3p
AGO2	hsa-miR-1956
AGO2	hsa-miR-7399-5p
AGO2	hsa-miR-4682
AGO2	hsa-miR-5128
AGO2	hsa-miR-2900
AGO2	hsa-miR-2298
AGO2	hsa-miR-1574-3p
AGO2	hsa-miR-7176-3p
AGO2	hsa-miR-6715-3p
AGO2	hsa-miR-92b-3p
AGO2	hsa-miR-6416-3p
AGO2	hsa-miR-5583-5p
AGO2	hsa-miR-8840
AGO2	hsa-miR-499
AGO2	hsa-miR-2284z
AGO2	hsa-miR-669
AGO2	hsa-miR-181
AGO2	hsa-miR-425-3p
AGO2	hsa-miR-548bb-3p
AGO2	hsa-miR-6969-5p
AGO2	hsa-miR-6894-3p
AGO2	hsa-miR-7215-3p
AGO2	hsa-miR-4647
AGO2	hsa-miR-548c-3p
AGO2	hsa-miR-383
AGO2	hsa-miR-679-5p
AGO2	hsa-miR-693-5p
AGO2	hsa-miR-7679-5p
AGO2	hsa-miR-1227-3p
AGO2	hsa-miR-1843b-5p
AGO2	hsa-miR-5588-3p
AGO2	hsa-miR-6805-5p
AGO2	hsa-miR-8073
AGO2	hsa-miR-503-5p
AGO2	hsa-miR-7843-3p
AGO2	hsa-miR-6508-5p
AGO2	hsa-miR-5106
AGO2	hsa-miR-4642
AGO2	hsa-miR-29b-2-5p

AGO2	hsa-miR-196-1-3p
AGO2	hsa-miR-203b-5p
AGO2	hsa-miR-9-3-3p
AGO2	hsa-miR-6752-3p
AGO2	hsa-miR-1983
AGO2	hsa-miR-139-5p
AGO2	hsa-miR-1811
AGO2	hsa-miR-2284
AGO2	hsa-miR-190a-5p
AGO2	hsa-miR-7850-5p
AGO2	hsa-miR-2285b
AGO2	hsa-miR-6920-5p
AGO2	hsa-miR-7681-5p
AGO2	hsa-miR-1794
AGO2	hsa-miR-548at-5p
AGO2	hsa-miR-3102-3p
AGO2	hsa-miR-3075-3p
AGO2	hsa-miR-8876
AGO2	hsa-miR-744-5p
AGO2	hsa-miR-3587
AGO2	hsa-miR-6835-5p
AGO2	hsa-miR-1247-5p
AGO2	hsa-miR-1283
AGO2	hsa-miR-4795-3p
AGO2	hsa-miR-200a-5p
AGO2	hsa-miR-3110-3p
AGO2	hsa-miR-4663
AGO2	hsa-miR-466l-5p
AGO2	hsa-miR-671-5p
AGO2	hsa-miR-6572-5p
AGO2	hsa-miR-3585-5p
AGO2	hsa-miR-638
AGO2	hsa-miR-8076
AGO2	hsa-miR-7064-3p
AGO2	hsa-miR-3558-5p
AGO2	hsa-miR-185-3p
AGO2	hsa-miR-361-3p
AGO2	hsa-miR-6782-3p
AGO2	hsa-miR-1287
AGO2	hsa-miR-6371
AGO2	hsa-miR-653-5p
AGO2	hsa-miR-7200-3p
AGO2	hsa-miR-3187-5p
AGO2	hsa-miR-4662b
AGO2	hsa-miR-1271-5p
AGO2	hsa-miR-1540-5p

AGO2	hsa-miR-4510
AGO2	hsa-miR-1941-5p
AGO2	hsa-miR-6841-3p
AGO2	hsa-miR-6365
AGO2	hsa-miR-3138
AGO2	hsa-miR-743b-3p
AGO2	hsa-miR-6773-5p
AGO2	hsa-miR-6957-5p
AGO2	hsa-miR-101-5p
AGO2	hsa-miR-1226-3p
AGO2	hsa-miR-8118
AGO2	hsa-miR-1757
AGO2	hsa-miR-7076-3p
AGO2	hsa-miR-597-5p
AGO2	hsa-miR-326
AGO2	hsa-miR-1541
AGO2	hsa-miR-6943-5p
AGO2	hsa-miR-7362-3p
AGO2	hsa-miR-7262b-5p
AGO2	hsa-miR-502-5p
AGO2	hsa-miR-6938-5p
AGO2	hsa-miR-7028-5p
AGO2	hsa-miR-8052
AGO2	hsa-miR-6911-5p
AGO2	hsa-miR-31-5p
AGO2	hsa-miR-7386d-3p
AGO2	hsa-miR-4471
AGO2	hsa-miR-6752-5p
AGO2	hsa-miR-4790-3p
AGO2	hsa-miR-7341-5p
AGO2	hsa-miR-759
AGO2	hsa-miR-3191-5p
AGO2	hsa-miR-7668-5p
AGO2	hsa-miR-485-3p
AGO2	hsa-miR-548bb-5p
AGO2	hsa-miR-7298-5p
AGO2	hsa-miR-889-5p
AGO2	hsa-miR-6791-5p
AGO2	hsa-miR-7313-5p
AGO2	hsa-miR-6989-5p
AGO2	hsa-miR-3559-5p
AGO2	hsa-miR-4751
AGO2	hsa-miR-432-5p
AGO2	hsa-miR-8116
AGO2	hsa-miR-2328-5p
AGO2	hsa-miR-3082-5p

AGO2	hsa-miR-7235-5p
AGO2	hsa-miR-328
AGO2	hsa-miR-4778-5p
AGO2	hsa-miR-6322
AGO2	hsa-miR-4307
AGO2	hsa-miR-8080
AGO2	hsa-miR-6923-5p
AGO2	hsa-miR-492
AGO2	hsa-miR-512-5p
AGO2	hsa-miR-6561-5p
AGO2	hsa-miR-664
AGO2	hsa-miR-214-3p
AGO2	hsa-miR-6704-5p
AGO2	hsa-miR-5121
AGO2	hsa-miR-539-3p
AGO2	hsa-miR-7025-5p
AGO2	hsa-miR-2313-3p
AGO2	hsa-miR-3557-5p
AGO2	hsa-miR-126a-5p
AGO2	hsa-miR-3057-5p
AGO2	hsa-miR-8903
AGO2	hsa-miR-7371-5p
AGO2	hsa-miR-7308-3p
AGO2	hsa-miR-95-3p
AGO2	hsa-miR-4740-5p
AGO2	hsa-miR-6130
AGO2	hsa-miR-4742
AGO2	hsa-miR-873a-5p.2
AGO2	hsa-miR-3159
AGO2	hsa-miR-517
AGO2	hsa-miR-7212-3p
AGO2	hsa-miR-4683
AGO2	hsa-miR-741-5p
AGO2	hsa-miR-7077-3p
AGO2	hsa-miR-6731-5p
AGO2	hsa-miR-548i
AGO2	hsa-miR-7314-5p
AGO2	hsa-miR-147a
AGO2	hsa-miR-629-3p
AGO2	hsa-miR-215
AGO2	hsa-miR-758-3p
AGO2	hsa-miR-7290-5p
AGO2	hsa-miR-503-3p
AGO2	hsa-miR-6742-5p
AGO2	hsa-miR-4515
AGO2	hsa-miR-1797

AGO2	hsa-miR-376a-1-5p
AGO2	hsa-miR-7306-5p
AGO2	hsa-miR-377-5p
AGO2	hsa-miR-669g
AGO2	hsa-miR-4699-3p
AGO2	hsa-miR-7648-3p
AGO2	hsa-miR-5703
AGO2	hsa-miR-7047-3p
AGO2	hsa-miR-684
AGO2	hsa-miR-1199-3p
AGO2	hsa-miR-1786
AGO2	hsa-miR-7352-5p
AGO2	hsa-miR-548av-3p
AGO2	hsa-miR-3924
AGO2	hsa-miR-766-3p
AGO2	hsa-miR-548f-5p
AGO2	hsa-miR-676-3p
AGO2	hsa-let-7c-5p
AGO2	hsa-miR-3123
AGO2	hsa-miR-99a-5p
AGO2	hsa-miR-293-5p
AGO2	hsa-miR-7278-3p
AGO2	hsa-miR-3622b-3p
AGO2	hsa-miR-2277-3p
AGO2	hsa-miR-1262-3p
AGO2	hsa-miR-3104-5p
AGO2	hsa-miR-4782-5p
AGO2	hsa-miR-383-5p.1
AGO2	hsa-miR-1936
AGO2	hsa-miR-429-5p
AGO2	hsa-miR-15a-5p
AGO2	hsa-miR-3074-3p
AGO2	hsa-miR-1973
AGO2	hsa-miR-6769b-5p
AGO2	hsa-miR-6521
AGO2	hsa-miR-516b
AGO2	hsa-miR-27a-5p
AGO2	hsa-miR-1643a-5p
AGO2	hsa-miR-668
AGO2	hsa-miR-3093-3p
AGO2	hsa-miR-133b
AGO2	hsa-miR-3144-5p
AGO2	hsa-miR-7349-3p
AGO2	hsa-miR-1915-3p
AGO2	hsa-miR-6331
AGO2	hsa-miR-28-3p

AGO2	hsa-miR-2333
AGO2	hsa-miR-709
AGO2	hsa-miR-6961-5p
AGO2	hsa-miR-548a-3p
AGO2	hsa-miR-520e
AGO2	hsa-miR-3925-3p
AGO2	hsa-miR-548ah-5p
AGO2	hsa-miR-6125
AGO2	hsa-miR-875-5p
AGO2	hsa-miR-155-5p
AGO2	hsa-miR-7024-5p
AGO2	hsa-miR-6325
AGO2	hsa-miR-6971-3p
AGO2	hsa-miR-548h-5p
AGO2	hsa-miR-1292-5p
AGO2	hsa-miR-7358-3p
AGO2	hsa-miR-7063-5p
AGO2	hsa-miR-98-5p
AGO2	hsa-miR-6730-5p
AGO2	hsa-miR-20a-3p
AGO2	hsa-miR-6760-3p
AGO2	hsa-miR-23b-5p
AGO2	hsa-miR-669hk-3p
AGO2	hsa-miR-4773
AGO2	hsa-miR-491-5p
AGO2	hsa-miR-587
AGO2	hsa-miR-3145-3p
AGO2	hsa-miR-6876-3p
AGO2	hsa-miR-764-5p
AGO2	hsa-miR-2449
AGO2	hsa-miR-7454-3p
AGO2	hsa-miR-6075
AGO2	hsa-miR-26-5p
AGO2	hsa-miR-6504-3p
AGO2	hsa-let-7-3p
AGO2	hsa-miR-890
AGO2	hsa-miR-4503
AGO2	hsa-miR-1307-3p
AGO2	hsa-miR-2892
AGO2	hsa-miR-6780b-5p
AGO2	hsa-miR-7236-5p
AGO2	hsa-miR-1205
AGO2	hsa-miR-2128
AGO2	hsa-miR-2382-5p
AGO2	hsa-miR-674-3p
AGO2	hsa-miR-344-5p

AGO2	hsa-miR-7268-3p
AGO2	hsa-miR-132-3p
AGO2	hsa-miR-1958
AGO2	hsa-miR-7007-5p
AGO2	hsa-miR-3614-3p
AGO2	hsa-miR-378j
AGO2	hsa-miR-3084-5p
AGO2	hsa-miR-7054-3p
AGO2	hsa-miR-4433a-5p
AGO2	hsa-miR-1-1-5p
AGO2	hsa-miR-6857-3p
AGO2	hsa-miR-4312
AGO2	hsa-miR-4729
AGO2	hsa-miR-6574-5p
AGO2	hsa-miR-6820-5p
AGO2	hsa-miR-373-5p
AGO2	hsa-miR-7192-3p
AGO2	hsa-miR-7394a-5p
AGO2	hsa-miR-542-3p
AGO2	hsa-miR-219b-3p
AGO2	hsa-miR-7379-3p
AGO2	hsa-miR-186-5p
AGO2	hsa-miR-185-5p
AGO2	hsa-miR-7165-3p
AGO2	hsa-miR-296-3p
AGO2	hsa-miR-2285q
AGO2	hsa-miR-890-3p
AGO2	hsa-miR-7154-3p
AGO2	hsa-miR-6411
AGO2	hsa-miR-1654
AGO2	hsa-miR-2443
AGO2	hsa-miR-23c
AGO2	hsa-miR-7438-3p
AGO2	hsa-miR-2354
AGO2	hsa-miR-302a-5p
AGO2	hsa-miR-7083-3p
AGO2	hsa-miR-1592
AGO2	hsa-miR-8084
AGO2	hsa-miR-4640-5p
AGO2	hsa-miR-4802-3p
AGO2	hsa-miR-7088-5p
AGO2	hsa-miR-6673-3p
AGO2	hsa-miR-1547-5p
AGO2	hsa-miR-7478-3p
AGO2	hsa-miR-7851-3p
AGO2	hsa-miR-452-5p

AGO2	hsa-miR-2349
AGO2	hsa-miR-302b-5p
AGO2	hsa-miR-5194
AGO2	hsa-miR-1249-5p
AGO2	hsa-miR-6792-5p
AGO2	hsa-miR-6761-3p
AGO2	hsa-miR-8087
AGO2	hsa-miR-496.2
AGO2	hsa-miR-455-5p
AGO2	hsa-miR-889
AGO2	hsa-miR-513c-3p
AGO2	hsa-miR-7155-5p
AGO2	hsa-miR-1647
AGO2	hsa-miR-3646
AGO2	hsa-miR-376bc-5p
AGO2	hsa-miR-6388
AGO2	hsa-miR-6771-5p
AGO2	hsa-miR-4437
AGO2	hsa-miR-6770-3p
AGO2	hsa-miR-6970-3p
AGO2	hsa-miR-3080-3p
AGO2	hsa-miR-5000-5p
AGO2	hsa-miR-520c-3p
AGO2	hsa-miR-486-5p
AGO2	hsa-miR-517a-3p
AGO2	hsa-miR-3060-5p
AGO2	hsa-miR-6719-3p
AGO2	hsa-miR-6693-3p
AGO2	hsa-miR-4449
AGO2	hsa-miR-6887-3p
AGO2	hsa-miR-6855-5p
AGO2	hsa-miR-450b-3p
AGO2	hsa-miR-1814a
AGO2	hsa-miR-577-5p
AGO2	hsa-miR-6984-3p
AGO2	hsa-miR-7688-5p
AGO2	hsa-miR-4292
AGO2	hsa-miR-5007-3p
AGO2	hsa-miR-6955-5p
AGO2	hsa-miR-344a-3p
AGO2	hsa-miR-4251
AGO2	hsa-miR-5697-3p
AGO2	hsa-miR-181b-3p
AGO2	hsa-miR-496a-5p
AGO2	hsa-miR-330-3p
AGO2	hsa-miR-4274

AGO2	hsa-miR-2427
AGO2	hsa-miR-1643-3p
AGO2	hsa-miR-7181-5p
AGO2	hsa-miR-527
AGO2	hsa-miR-320c
AGO2	hsa-miR-7265-5p
AGO2	hsa-miR-1243-5p
AGO2	hsa-miR-496
AGO2	hsa-miR-615-3p
AGO2	hsa-miR-7017-3p
AGO2	hsa-miR-384
AGO2	hsa-miR-1468-5p
AGO2	hsa-miR-4696
AGO2	hsa-miR-6946-5p
AGO2	hsa-miR-22-3p
AGO2	hsa-miR-1955-5p
AGO2	hsa-miR-125b-5p
AGO2	hsa-miR-7243-3p
AGO2	hsa-miR-3920
AGO2	hsa-miR-7398t-3p
AGO2	hsa-miR-939-3p
AGO2	hsa-miR-4280
AGO2	hsa-miR-548x-3p
AGO2	hsa-miR-99
AGO2	hsa-miR-10b-5p
AGO2	hsa-miR-466b-5p
AGO2	hsa-miR-7398p-5p
AGO2	hsa-miR-6529a
AGO2	hsa-miR-758-5p
AGO2	hsa-miR-7219-5p
AGO2	hsa-miR-3574
AGO2	hsa-miR-7034-3p
AGO2	hsa-miR-4687-3p
AGO2	hsa-miR-644a
AGO2	hsa-miR-2399-3p
AGO2	hsa-miR-6948-3p
AGO2	hsa-miR-6387
AGO2	hsa-miR-6824-5p
AGO2	hsa-miR-6404
AGO2	hsa-miR-7208-5p
AGO2	hsa-miR-4750-3p
AGO2	hsa-miR-421
AGO2	hsa-miR-8900
AGO2	hsa-miR-6165
AGO2	hsa-miR-206
AGO2	hsa-miR-3127-3p

AGO2	hsa-miR-4650-3p
AGO2	hsa-miR-4761-5p
AGO2	hsa-miR-647
AGO2	hsa-miR-7671-5p
AGO2	hsa-miR-1a-5p
AGO2	hsa-miR-6709-5p
AGO2	hsa-miR-1329-5p
AGO2	hsa-miR-350-3p
AGO2	hsa-miR-7259-5p
AGO2	hsa-miR-6929-5p
AGO2	hsa-miR-8854
AGO2	hsa-miR-1266-5p
AGO2	hsa-miR-7008-5p
AGO2	hsa-miR-1260a
AGO2	hsa-miR-7438-5p
AGO2	hsa-miR-8100
AGO2	hsa-miR-7381-5p
AGO2	hsa-miR-19-5p
AGO2	hsa-miR-3921
AGO2	hsa-miR-5195-5p
AGO2	hsa-miR-4491
AGO2	hsa-miR-34b-3p
AGO2	hsa-miR-3914
AGO2	hsa-miR-3917
AGO2	hsa-miR-4539
AGO2	hsa-miR-6374
AGO2	hsa-miR-511-5p
AGO2	hsa-miR-371b-5p
AGO2	hsa-miR-5095
AGO2	hsa-miR-7091-5p
AGO2	hsa-miR-1288-3p
AGO2	hsa-miR-208-3p
AGO2	hsa-miR-3535
AGO2	hsa-miR-8805
AGO2	hsa-miR-6747-3p
AGO2	hsa-miR-183-5p.1
AGO2	hsa-miR-876-5p
AGO2	hsa-miR-3622b-5p
AGO2	hsa-miR-149-5p
AGO2	hsa-miR-4486
AGO2	hsa-miR-26-3-3p
AGO2	hsa-miR-8848
AGO2	hsa-miR-103a-2-5p
AGO2	hsa-miR-717
AGO2	hsa-miR-2441
AGO2	hsa-miR-6975-3p

AGO2	hsa-miR-625-5p
AGO2	hsa-miR-7973
AGO2	hsa-miR-7242-3p
AGO2	hsa-miR-3099
AGO2	hsa-miR-1306-3p
AGO2	hsa-miR-6644-3p
AGO2	hsa-miR-150-5p
AGO2	hsa-miR-302a-3p
AGO2	hsa-miR-6954-5p
AGO2	hsa-miR-3558-3p
AGO2	hsa-miR-216
AGO2	hsa-miR-1282
AGO2	hsa-miR-6874-3p
AGO2	hsa-miR-6514-3p
AGO2	hsa-miR-424-3p
AGO2	hsa-miR-6736-3p
AGO2	hsa-miR-192-5p
AGO2	hsa-miR-3607-3p
AGO2	hsa-miR-3075-5p
AGO2	hsa-miR-616-3p
AGO2	hsa-miR-712-5p
AGO2	hsa-miR-4755-5p
AGO2	hsa-miR-6995-3p
AGO2	hsa-miR-222-3p
AGO2	hsa-miR-6956-5p
AGO2	hsa-miR-592-3p
AGO2	hsa-miR-4477a
AGO2	hsa-miR-6334
AGO2	hsa-miR-7845-5p
AGO2	hsa-miR-6776-5p
AGO2	hsa-miR-409
AGO2	hsa-miR-34bc-3p
AGO2	hsa-miR-876-3p
AGO2	hsa-miR-6590-5p
AGO2	hsa-miR-3154
AGO2	hsa-miR-1273g-3p
AGO2	hsa-miR-651-5p
AGO2	hsa-miR-7252-5p
AGO2	hsa-miR-338-3p
AGO2	hsa-miR-1273f
AGO2	hsa-miR-1749-5p
AGO2	hsa-miR-330-5p
AGO2	hsa-miR-6991-3p
AGO2	hsa-miR-4652-3p
AGO2	hsa-miR-6967-3p
AGO2	hsa-miR-532-3p

AGO2	hsa-miR-4775
AGO2	hsa-miR-6819-5p
AGO2	hsa-miR-2426
AGO2	hsa-miR-328-5p
AGO2	hsa-miR-519-5p
AGO2	hsa-miR-27a-3p
AGO2	hsa-miR-1802
AGO2	hsa-miR-520gh
AGO2	hsa-miR-7977
AGO2	hsa-miR-8816
AGO2	hsa-miR-7311-5p
AGO2	hsa-miR-6778-5p
AGO2	hsa-miR-1912-5p
AGO2	hsa-miR-887-5p
AGO2	hsa-miR-1-3p
AGO2	hsa-miR-299b-5p
AGO2	hsa-miR-7859
AGO2	hsa-miR-6794-3p
AGO2	hsa-miR-3663-5p
AGO2	hsa-miR-2411-5p
AGO2	hsa-miR-5133
AGO2	hsa-miR-6501-3p
AGO2	hsa-miR-1190
AGO2	hsa-miR-1960
AGO2	hsa-miR-19
AGO2	hsa-miR-211-5p
AGO2	hsa-miR-1812-3p
AGO2	hsa-miR-2330-5p
AGO2	hsa-miR-1901
AGO2	hsa-miR-8103
AGO2	hsa-miR-218-2-3p
AGO2	hsa-miR-6876-5p
AGO2	hsa-miR-548c-5p
AGO2	hsa-miR-1264-3p
AGO2	hsa-miR-429-3p
AGO2	hsa-miR-149-5p
AGO2	hsa-miR-4419a
AGO2	hsa-miR-4328
AGO2	hsa-miR-7398h-5p
AGO2	hsa-miR-3150b-3p
AGO2	hsa-miR-148-5p
AGO2	hsa-miR-7856-5p
AGO2	hsa-miR-4662-5p
AGO2	hsa-miR-3113-5p
AGO2	hsa-miR-942
AGO2	hsa-miR-6570-5p

AGO2	hsa-miR-6316
AGO2	hsa-miR-143-3p
AGO2	hsa-miR-6859-3p
AGO2	hsa-miR-101b-3p.2
AGO2	hsa-miR-1254
AGO2	hsa-miR-4755-3p
AGO2	hsa-miR-7325-3p
AGO2	hsa-miR-2393
AGO2	hsa-miR-7456-5p
AGO2	hsa-miR-742-5p
AGO2	hsa-miR-135b-3p
AGO2	hsa-miR-6575-5p
AGO2	hsa-miR-5189-3p
AGO2	hsa-miR-494-3p
AGO2	hsa-miR-18-3p
AGO2	hsa-miR-549a
AGO2	hsa-miR-2385-5p
AGO2	hsa-miR-7023-3p
AGO2	hsa-miR-340-5p
AGO2	hsa-miR-1545c-3p
AGO2	hsa-miR-465abc-3p
AGO2	hsa-miR-30d-3p
AGO2	hsa-miR-23-5p
AGO2	hsa-miR-1970
AGO2	hsa-miR-16-5p
AGO2	hsa-miR-3560
AGO2	hsa-miR-3085-3p
AGO2	hsa-miR-7680-3p
AGO2	hsa-miR-222-3p
AGO2	hsa-miR-656
AGO2	hsa-miR-6994-5p
AGO2	hsa-miR-30b-5p
AGO2	hsa-miR-7249-5p
AGO2	hsa-miR-2344
AGO2	hsa-miR-4433b-3p
AGO2	hsa-miR-4308
AGO2	hsa-miR-200b-3p
AGO2	hsa-miR-4252
AGO2	hsa-miR-1245a
AGO2	hsa-miR-6695-5p
AGO2	hsa-miR-653
AGO2	hsa-miR-1839
AGO2	hsa-miR-2188-3p
AGO2	hsa-miR-3544-3p
AGO2	hsa-miR-4713-3p
AGO2	hsa-miR-7462-3p

AGO2	hsa-miR-7460-3p
AGO2	hsa-miR-6960-3p
AGO2	hsa-miR-1620
AGO2	hsa-miR-596
AGO2	hsa-miR-5010-5p
AGO2	hsa-miR-142-3p
AGO2	hsa-miR-632
AGO2	hsa-miR-2306
AGO2	hsa-miR-298-5p
AGO2	hsa-miR-8841
AGO2	hsa-miR-2894
AGO2	hsa-miR-26
AGO2	hsa-miR-7171-3p
AGO2	hsa-miR-506-3p
AGO2	hsa-miR-2340
AGO2	hsa-miR-9-3p
AGO2	hsa-miR-6867-3p
AGO2	hsa-miR-669m-5p
AGO2	hsa-miR-8081
AGO2	hsa-miR-7481-3p
AGO2	hsa-miR-1738
AGO2	hsa-miR-5698
AGO2	hsa-miR-3095-5p
AGO2	hsa-miR-6882-3p
AGO2	hsa-miR-2475
AGO2	hsa-miR-6750-5p
AGO2	hsa-miR-2442
AGO2	hsa-miR-4461
AGO2	hsa-miR-6937-3p
AGO2	hsa-miR-7045-5p
AGO2	hsa-miR-337-3p
AGO2	hsa-miR-7213-5p
AGO2	hsa-miR-381-3p
AGO2	hsa-miR-1950
AGO2	hsa-miR-467g
AGO2	hsa-miR-143-5p
AGO2	hsa-miR-6355
AGO2	hsa-miR-203a-3p.1
AGO2	hsa-miR-669ao-3p
AGO2	hsa-miR-3150a-5p
AGO2	hsa-miR-4698
AGO2	hsa-miR-32-5p
AGO2	hsa-miR-3576
AGO2	hsa-miR-7847-3p
AGO2	hsa-miR-505-3p
AGO2	hsa-miR-4709-5p

AGO2	hsa-miR-624-5p
AGO2	hsa-miR-122-3p
AGO2	hsa-miR-6707-5p
AGO2	hsa-miR-1672
AGO2	hsa-miR-7313-3p
AGO2	hsa-miR-1179
AGO2	hsa-miR-5011-5p
AGO2	hsa-miR-10b-5p
AGO2	hsa-miR-881-3p
AGO2	hsa-miR-6829-5p
AGO2	hsa-miR-6751-5p
AGO2	hsa-miR-6574-3p
AGO2	hsa-miR-500b-5p
AGO2	hsa-miR-4483
AGO2	hsa-miR-513a-5p
AGO2	hsa-miR-7119-5p
AGO2	hsa-miR-502-3p
AGO2	hsa-miR-1540
AGO2	hsa-miR-3682-3p
AGO2	hsa-miR-8083
AGO2	hsa-miR-7382-5p
AGO2	hsa-miR-18-5p
AGO2	hsa-miR-516b-3p
AGO2	hsa-miR-570-5p
AGO2	hsa-miR-196c-3p
AGO2	hsa-miR-6503-5p
AGO2	hsa-miR-7385f-3p
AGO2	hsa-miR-6899-3p
AGO2	hsa-miR-21-3p
AGO2	hsa-miR-7371b-3p
AGO2	hsa-miR-381-5p
AGO2	hsa-miR-1896
AGO2	hsa-miR-2489
AGO2	hsa-miR-597
AGO2	hsa-miR-627-5p
AGO2	hsa-miR-892-3p
AGO2	hsa-miR-525-5p
AGO2	hsa-miR-2477
AGO2	hsa-miR-6743-3p
AGO2	hsa-miR-7111-5p
AGO2	hsa-miR-1894-5p
AGO2	hsa-miR-6849-3p
AGO2	hsa-miR-1546-3p
AGO2	hsa-miR-520d-3p
AGO2	hsa-miR-7299-5p
AGO2	hsa-miR-7223-5p

AGO2	hsa-miR-6892-3p
AGO2	hsa-miR-6740-5p
AGO2	hsa-miR-7356-3p
AGO2	hsa-miR-323a-3p
AGO2	hsa-miR-6588-3p
AGO2	hsa-miR-3076-3p
AGO2	hsa-miR-10b-3p
AGO2	hsa-miR-519-3p
AGO2	hsa-miR-2406
AGO2	hsa-miR-6824-3p
AGO2	hsa-miR-2358
AGO2	hsa-miR-7057-5p
AGO2	hsa-miR-3934-5p
AGO2	hsa-miR-4254
AGO2	hsa-miR-636
AGO2	hsa-miR-331-3p
AGO2	hsa-miR-6589-5p
AGO2	hsa-miR-1468
AGO2	hsa-miR-7066-5p
AGO2	hsa-miR-541-3p
AGO2	hsa-miR-1719
AGO2	hsa-miR-6705-5p
AGO2	hsa-miR-8075
AGO2	hsa-miR-7106-5p
AGO2	hsa-miR-7682-5p
AGO2	hsa-miR-3057-3p
AGO2	hsa-miR-1639
AGO2	hsa-miR-7196-5p
AGO2	hsa-miR-7386g-3p
AGO2	hsa-miR-4481
AGO2	hsa-miR-4798-3p
AGO2	hsa-miR-3100-3p
AGO2	hsa-miR-7-3p
AGO2	hsa-miR-7380-3p
AGO2	hsa-miR-7041-5p
AGO2	hsa-miR-4526
AGO2	hsa-miR-224-3p
AGO2	hsa-miR-30c-5p
AGO2	hsa-miR-182-5p
AGO2	hsa-miR-3085
AGO2	hsa-miR-7238-3p
AGO2	hsa-let-7e-5p
AGO2	hsa-miR-6403
AGO2	hsa-miR-428
AGO2	hsa-miR-6772-5p
AGO2	hsa-miR-7117-5p

AGO2	hsa-miR-92-5p
AGO2	hsa-miR-6861-3p
AGO2	hsa-miR-3129-3p
AGO2	hsa-miR-28-5p
AGO2	hsa-miR-200c-3p
AGO2	hsa-miR-7010-5p
AGO2	hsa-miR-372-3p
AGO2	hsa-miR-7116-3p
AGO2	hsa-miR-218-5p
AGO2	hsa-miR-7262a-5p
AGO2	hsa-miR-6515-5p
AGO2	hsa-miR-7094-1-5p
AGO2	hsa-miR-6087
AGO2	hsa-miR-7386k-5p
AGO2	hsa-miR-2330-3p
AGO2	hsa-miR-7326-5p
AGO2	hsa-miR-382-3p
AGO2	hsa-miR-96-3p
AGO2	hsa-miR-1773-5p
AGO2	hsa-miR-343
AGO2	hsa-miR-7398r-5p
AGO2	hsa-miR-2316
AGO2	hsa-miR-3098-5p
AGO2	hsa-miR-7355-5p
AGO2	hsa-miR-197-3p
AGO2	hsa-miR-7033-3p
AGO2	hsa-miR-7672-5p
AGO2	hsa-miR-2300-3p
AGO2	hsa-miR-7675-3p
AGO2	hsa-miR-7297-3p
AGO2	hsa-miR-6742-3p
AGO2	hsa-miR-3678-3p
AGO2	hsa-miR-5706
AGO2	hsa-miR-2054
AGO2	hsa-miR-2366
AGO2	hsa-let-7a-5p
AGO2	hsa-miR-5696
AGO2	hsa-miR-135
AGO2	hsa-miR-7109-5p
AGO2	hsa-miR-7857
AGO2	hsa-miR-6338
AGO2	hsa-miR-100-5p
AGO2	hsa-miR-7227-5p
AGO2	hsa-miR-223-5p
AGO2	hsa-miR-1943-3p
AGO2	hsa-miR-8819

AGO2	hsa-miR-7439-3p
AGO2	hsa-miR-7189-3p
AGO2	hsa-miR-3664-5p
AGO2	hsa-miR-7976
AGO2	hsa-miR-218a-2-3p
AGO2	hsa-miR-7173-5p
AGO2	hsa-miR-103-3p
AGO2	hsa-miR-4801
AGO2	hsa-miR-7264-3p
AGO2	hsa-miR-6871-3p
AGO2	hsa-miR-1245b-3p
AGO2	hsa-miR-7338-5p
AGO2	hsa-miR-10-5p
AGO2	hsa-miR-4325
AGO2	hsa-miR-743ab-3p
AGO2	hsa-miR-17
AGO2	hsa-miR-4694-3p
AGO2	hsa-miR-320a
AGO2	hsa-miR-6931-5p
AGO2	hsa-miR-3076-5p
AGO2	hsa-miR-8812
AGO2	hsa-miR-98-5p
AGO2	hsa-miR-8090
AGO2	hsa-miR-2360
AGO2	hsa-miR-8837
AGO2	hsa-miR-6852-5p
AGO2	hsa-miR-339-5p
AGO2	hsa-miR-4680
AGO2	hsa-miR-3151
AGO2	hsa-miR-4514
AGO2	hsa-miR-7015-5p
AGO2	hsa-miR-7473-5p
AGO2	hsa-miR-184-5p
AGO2	hsa-miR-142-5p
AGO2	hsa-miR-7085-3p
AGO2	hsa-miR-6571-5p
AGO2	hsa-miR-654-3p
AGO2	hsa-miR-4733-3p
AGO2	hsa-miR-8806
AGO2	hsa-miR-601
AGO2	hsa-miR-548an
AGO2	hsa-miR-7108-3p
AGO2	hsa-miR-9769-5p
AGO2	hsa-miR-5003-5p
AGO2	hsa-miR-4301
AGO2	hsa-miR-7170-3p

AGO2	hsa-miR-7398g-3p
AGO2	hsa-miR-7261-5p
AGO2	hsa-miR-7260-5p
AGO2	hsa-miR-5692a
AGO2	hsa-miR-374-5p
AGO2	hsa-miR-484
AGO2	hsa-miR-134
AGO2	hsa-miR-6858-5p
AGO2	hsa-miR-106b-3p
AGO2	hsa-miR-2399-5p
AGO2	hsa-miR-6119-5p
AGO2	hsa-miR-7008-3p
AGO2	hsa-miR-6368
AGO2	hsa-miR-7328-3p
AGO2	hsa-miR-130a-3p
AGO2	hsa-miR-1957a
AGO2	hsa-miR-4291
AGO2	hsa-miR-4677-5p
AGO2	hsa-miR-8062
AGO2	hsa-miR-2285j
AGO2	hsa-miR-6543-3p
AGO2	hsa-miR-151
AGO2	hsa-miR-598-3p
AGO2	hsa-miR-7249-3p
AGO2	hsa-miR-193a-5p
AGO2	hsa-miR-4768-5p
AGO2	hsa-miR-7334-5p
AGO2	hsa-miR-101c
AGO2	hsa-miR-883-5p
AGO2	hsa-miR-372-5p
AGO2	hsa-miR-4757-5p
AGO2	hsa-miR-200ab-5p
AGO2	hsa-miR-379-3p
AGO2	hsa-miR-4455
AGO2	hsa-miR-5619-5p
AGO2	hsa-miR-455-3p
AGO2	hsa-miR-8799
AGO2	hsa-miR-1547-3p
AGO2	hsa-miR-7g-3p
AGO2	hsa-miR-323a-5p
AGO2	hsa-miR-3085-5p
AGO2	hsa-miR-4699-5p
AGO2	hsa-miR-3569-5p
AGO2	hsa-miR-663a
AGO2	hsa-miR-99b-5p
AGO2	hsa-miR-7158-3p

AGO2	hsa-miR-146b-3p
AGO2	hsa-miR-6856-3p
AGO2	hsa-miR-1301-3p
AGO2	hsa-miR-6889-3p
AGO2	hsa-miR-7018-5p
AGO2	hsa-miR-6965-3p
AGO2	hsa-miR-5615-5p
AGO2	hsa-miR-524-5p
AGO2	hsa-miR-3158-3p
AGO2	hsa-miR-6912-5p
AGO2	hsa-miR-7326-3p
AGO2	hsa-miR-384-5p
AGO2	hsa-miR-3180-3p
AGO2	hsa-miR-6974-5p
AGO2	hsa-miR-3120-3p
AGO2	hsa-miR-6917-3p
AGO2	hsa-miR-2131-5p
AGO2	hsa-miR-3091-3p
AGO2	hsa-miR-710
AGO2	hsa-miR-300
AGO2	hsa-miR-4430
AGO2	hsa-miR-891a
AGO2	hsa-miR-7371b-5p
AGO2	hsa-miR-6516-5p
AGO2	hsa-miR-6516
AGO2	hsa-miR-942-5p
AGO2	hsa-miR-888-5p
AGO2	hsa-miR-122
AGO2	hsa-miR-466mo-3p
AGO2	hsa-miR-6781-3p
AGO2	hsa-miR-1539
AGO2	hsa-miR-7398u-3p
AGO2	hsa-miR-7218-5p
AGO2	hsa-miR-126-5p
AGO2	hsa-miR-6729-3p
AGO2	hsa-miR-6927-3p
AGO2	hsa-miR-7205-3p
AGO2	hsa-miR-6832-3p
AGO2	hsa-miR-3068-5p
AGO2	hsa-miR-7359-3p
AGO2	hsa-miR-8108
AGO2	hsa-miR-211-3p
AGO2	hsa-miR-7343-5p
AGO2	hsa-miR-7-5p
AGO2	hsa-miR-7673-5p
AGO2	hsa-miR-4645-5p

AGO2	hsa-miR-7660-3p
AGO2	hsa-miR-411-5p.1
AGO2	hsa-miR-633
AGO2	hsa-miR-4255
AGO2	hsa-miR-548v
AGO2	hsa-miR-5134-3p
AGO2	hsa-miR-101-3p
AGO2	hsa-miR-1711
AGO2	hsa-miR-576-5p
AGO2	hsa-miR-134-3p
AGO2	hsa-miR-4746-5p
AGO2	hsa-miR-2301
AGO2	hsa-miR-6418-3p
AGO2	hsa-miR-412
AGO2	hsa-miR-455-3p.2
AGO2	hsa-miR-580
AGO2	hsa-miR-3074-5p
AGO2	hsa-miR-1599
AGO2	hsa-miR-4534
AGO2	hsa-miR-6629-5p
AGO2	hsa-miR-2114-3p
AGO2	hsa-miR-2397-5p
AGO2	hsa-miR-1233-3p
AGO2	hsa-miR-3562
AGO2	hsa-miR-4421
AGO2	hsa-miR-672-3p
AGO2	hsa-miR-6481
AGO2	hsa-miR-7339-3p
AGO2	hsa-miR-765
AGO2	hsa-miR-6350
AGO2	hsa-miR-223-3p
AGO2	hsa-miR-150-5p
AGO2	hsa-miR-802
AGO2	hsa-miR-2320-5p
AGO2	hsa-miR-7383-3p
AGO2	hsa-miR-1195
AGO2	hsa-miR-361-5p
AGO2	hsa-miR-6552-5p
AGO2	hsa-miR-466dk-5p
AGO2	hsa-miR-4637
AGO2	hsa-miR-3167-5p
AGO2	hsa-miR-3163
AGO2	hsa-miR-541-5p
AGO2	hsa-miR-1298-3p
AGO2	hsa-miR-568
AGO2	hsa-miR-1687-3p

AGO2	hsa-miR-6321
AGO2	hsa-miR-3690
AGO2	hsa-miR-3174
AGO2	hsa-miR-3137
AGO2	hsa-miR-324-5p
AGO2	hsa-miR-7388b-3p
AGO2	hsa-miR-329a
AGO2	hsa-miR-8907
AGO2	hsa-miR-658
AGO2	hsa-miR-323
AGO2	hsa-miR-6982-5p
AGO2	hsa-miR-6538
AGO2	hsa-miR-141-3p
AGO2	hsa-miR-1343-3p
AGO2	hsa-miR-5130
AGO2	hsa-miR-431-5p
AGO2	hsa-miR-6405
AGO2	hsa-miR-3688-5p
AGO2	hsa-miR-1733
AGO2	hsa-miR-3688-3p
AGO2	hsa-miR-8117
AGO2	hsa-miR-6897-3p
AGO2	hsa-miR-504-3p
AGO2	hsa-miR-6934-3p
AGO2	hsa-miR-5687
AGO2	hsa-miR-7172-3p
AGO2	hsa-miR-7202-5p
AGO2	hsa-miR-6346
AGO2	hsa-miR-7398d-3p
AGO2	hsa-miR-8067
AGO2	hsa-miR-3096-5p
AGO2	hsa-miR-7398e-3p
AGO2	hsa-miR-1560-3p
AGO2	hsa-miR-6407
AGO2	hsa-miR-7030-5p
AGO2	hsa-miR-3976
AGO2	hsa-miR-92a-5p
AGO2	hsa-miR-26b-3p
AGO2	hsa-miR-7213-3p
AGO2	hsa-miR-6747-5p
AGO2	hsa-miR-378c
AGO2	hsa-let-7a-3p
AGO2	hsa-miR-370-5p
AGO2	hsa-miR-1753
AGO2	hsa-miR-3197
AGO2	hsa-miR-5132-3p

AGO2	hsa-miR-3580-5p
AGO2	hsa-miR-3113-3p
AGO2	hsa-miR-6864-3p
AGO2	hsa-miR-107-5p
AGO2	hsa-miR-466aep-5p
AGO2	hsa-miR-6909-5p
AGO2	hsa-miR-148-3p
AGO2	hsa-miR-7155-3p
AGO2	hsa-miR-7090-5p
AGO2	hsa-miR-1930-5p
AGO2	hsa-miR-1202
AGO2	hsa-miR-34
AGO2	hsa-miR-1193-5p
AGO2	hsa-miR-7515
AGO2	hsa-miR-7174-5p
AGO2	hsa-miR-7678-3p
AGO2	hsa-miR-548aj-5p
AGO2	hsa-miR-764-3p
AGO2	hsa-miR-4524a-3p
AGO2	hsa-miR-211-5p
AGO2	hsa-miR-7194-3p
AGO2	hsa-miR-6664-3p
AGO2	hsa-miR-6753-3p
AGO2	hsa-miR-1775-3p
AGO2	hsa-miR-8808
AGO2	hsa-miR-2423
AGO2	hsa-miR-6326
AGO2	hsa-miR-6796-5p
AGO2	hsa-miR-4286
AGO2	hsa-miR-3922-5p
AGO2	hsa-miR-1283a
AGO2	hsa-miR-6083
AGO2	hsa-miR-7342-3p
AGO2	hsa-miR-548au-5p
AGO2	hsa-miR-3165
AGO2	hsa-miR-4796-5p
AGO2	hsa-miR-1233-5p
AGO2	hsa-miR-3590-3p
AGO2	hsa-miR-135a-1-3p
AGO2	hsa-miR-548aq-5p
AGO2	hsa-miR-674-5p
AGO2	hsa-miR-191-5p
AGO2	hsa-miR-1624
AGO2	hsa-miR-704
AGO2	hsa-miR-101a-5p
AGO2	hsa-miR-7215-5p

AGO2	hsa-miR-411-3p
AGO2	hsa-miR-2431-3p
AGO2	hsa-miR-3191-3p
AGO2	hsa-miR-96-5p
AGO2	hsa-miR-582-5p
AGO2	hsa-miR-8886
AGO2	hsa-miR-219a-5p
AGO2	hsa-miR-296-5p
AGO2	hsa-miR-6793-3p
AGO2	hsa-miR-1816
AGO2	hsa-miR-2284v
AGO2	hsa-miR-548av-5p
AGO2	hsa-miR-7312-3p
AGO2	hsa-miR-3908
AGO2	hsa-miR-3117-3p
AGO2	hsa-miR-19-3p
AGO2	hsa-miR-1252-5p
AGO2	hsa-miR-6911-3p
AGO2	hsa-miR-2284k
AGO2	hsa-miR-7352-3p
AGO2	hsa-miR-448
AGO2	hsa-miR-2285x
AGO2	hsa-miR-9406
AGO2	hsa-miR-523-3p
AGO2	hsa-miR-6535
AGO2	hsa-miR-3596
AGO2	hsa-miR-374b-5p
AGO2	hsa-miR-891-3p
AGO2	hsa-miR-130-5p
AGO2	hsa-miR-3089-5p
AGO2	hsa-miR-378g
AGO2	hsa-miR-424-5p
AGO2	hsa-miR-1199-5p
AGO2	hsa-miR-7354-3p
AGO2	hsa-miR-4463
AGO2	hsa-miR-7266-5p
AGO2	hsa-miR-6828-3p
AGO2	hsa-miR-365b-3p
AGO2	hsa-miR-7057-3p
AGO2	hsa-miR-219a-1-3p
AGO2	hsa-miR-6754-3p
AGO2	hsa-miR-7157-3p
AGO2	hsa-miR-548k
AGO2	hsa-miR-6508-3p
AGO2	hsa-miR-6782-5p
AGO2	hsa-miR-7843-5p

AGO2	hsa-miR-1905
AGO2	hsa-miR-6616-5p
AGO2	hsa-miR-187-5p
AGO2	hsa-miR-8814
AGO2	hsa-miR-2319
AGO2	hsa-miR-7387-3p
AGO2	hsa-miR-294
AGO2	hsa-miR-29-3p
AGO2	hsa-miR-4269
AGO2	hsa-miR-7390-5p
AGO2	hsa-miR-7378-3p
AGO2	hsa-miR-1656
AGO2	hsa-miR-4787-5p
AGO2	hsa-miR-92-3p
AGO2	hsa-miR-3160-5p
AGO2	hsa-miR-883-3p
AGO2	hsa-miR-25-5p
AGO2	hsa-miR-5702
AGO2	hsa-miR-3097-5p
AGO2	hsa-miR-1247-3p
AGO2	hsa-miR-191-5p
AGO2	hsa-miR-4776-5p
AGO2	hsa-miR-7046-3p
AGO2	hsa-miR-1930-3p
AGO2	hsa-miR-7327-5p
AGO2	hsa-miR-683
AGO2	hsa-miR-6883-5p
AGO2	hsa-miR-7647-5p
AGO2	hsa-miR-374b-3p
AGO2	hsa-miR-4423-5p
AGO2	hsa-miR-181b-5p
AGO2	hsa-miR-7032-5p
AGO2	hsa-miR-7371a-3p
AGO2	hsa-miR-4658
AGO2	hsa-miR-133a-3p.2
AGO2	hsa-miR-6359
AGO2	hsa-miR-133b-5p
AGO2	hsa-miR-6827-3p
AGO2	hsa-miR-320d
AGO2	hsa-miR-3689-3p
AGO2	hsa-miR-1655-5p
AGO2	hsa-miR-2471-3p
AGO2	hsa-miR-2467-5p
AGO2	hsa-miR-3548
AGO2	hsa-miR-470-5p
AGO2	hsa-miR-7386i-3p

AGO2	hsa-miR-133a-5p
AGO2	hsa-miR-5681a
AGO2	hsa-miR-130
AGO2	hsa-miR-511
AGO2	hsa-miR-548e-5p
AGO2	hsa-miR-1754-3p
AGO2	hsa-miR-297bc-3p
AGO2	hsa-miR-544a
AGO2	hsa-miR-7054-5p
AGO2	hsa-miR-3689e
AGO2	hsa-miR-1814
AGO2	hsa-miR-6684-5p
AGO2	hsa-miR-3713
AGO2	hsa-miR-6785-5p
AGO2	hsa-miR-1603
AGO2	hsa-miR-2970-5p
AGO2	hsa-miR-7112-5p
AGO2	hsa-miR-6975-5p
AGO2	hsa-miR-1434-5p
AGO2	hsa-miR-6802-3p
AGO2	hsa-miR-365b-5p
AGO2	hsa-miR-6579-3p
AGO2	hsa-miR-6970-5p
AGO2	hsa-miR-409-5p
AGO2	hsa-miR-627
AGO2	hsa-miR-8813
AGO2	hsa-miR-5620-5p
AGO2	hsa-miR-7201-5p
AGO2	hsa-miR-3564
AGO2	hsa-miR-3618-3p
AGO2	hsa-miR-6315
AGO2	hsa-miR-7173-3p
AGO2	hsa-miR-1632-5p
AGO2	hsa-miR-7398q-3p
AGO2	hsa-miR-679-3p
AGO2	hsa-miR-216c-5p
AGO2	hsa-miR-2285z
AGO2	hsa-miR-7357-3p
AGO2	hsa-miR-6973a-3p
AGO2	hsa-miR-4703-3p
AGO2	hsa-miR-7849-3p
AGO2	hsa-miR-511-3p
AGO2	hsa-miR-345-3p
AGO2	hsa-miR-2464-5p
AGO2	hsa-miR-7214-5p
AGO2	hsa-miR-411a

AGO2	hsa-miR-1962
AGO2	hsa-miR-7204-5p
AGO2	hsa-miR-8058
AGO2	hsa-miR-6935-5p
AGO2	hsa-miR-6078
AGO2	hsa-miR-4323
AGO2	hsa-miR-8890
AGO2	hsa-miR-2350
AGO2	hsa-miR-6903-5p
AGO2	hsa-miR-4493
AGO2	hsa-miR-2314
AGO2	hsa-miR-25-3p
AGO2	hsa-miR-626
AGO2	hsa-miR-4789-3p
AGO2	hsa-miR-1596-5p
AGO2	hsa-miR-6840-5p
AGO2	hsa-miR-6969-3p
AGO2	hsa-miR-136-5p
AGO2	hsa-miR-6827-5p
AGO2	hsa-miR-1630
AGO2	hsa-miR-7091-3p
AGO2	hsa-miR-642-3p
AGO2	hsa-miR-490-5p
AGO2	hsa-miR-671-5p
AGO2	hsa-miR-3621
AGO2	hsa-miR-6838-3p
AGO2	hsa-miR-4684-3p
AGO2	hsa-miR-2463
AGO2	hsa-miR-4693-3p
AGO2	hsa-miR-1651-3p
AGO2	hsa-miR-5623-5p
AGO2	hsa-miR-1768
AGO2	hsa-miR-3557-3p
AGO2	hsa-miR-2357
AGO2	hsa-miR-4446-5p
AGO2	hsa-miR-6639-5p
AGO2	hsa-miR-6768-5p
AGO2	hsa-miR-3064-3p
AGO2	hsa-miR-520
AGO2	hsa-miR-130b-3p
AGO2	hsa-miR-3552
AGO2	hsa-miR-5101
AGO2	hsa-miR-623
AGO2	hsa-miR-8077
AGO2	hsa-miR-7646-3p
AGO2	hsa-miR-29a-5p

AGO2	hsa-miR-4273
AGO2	hsa-miR-6871-5p
AGO2	hsa-miR-6667-5p
AGO2	hsa-miR-548am-3p
AGO2	hsa-miR-1954
AGO2	hsa-miR-6926-5p
AGO2	hsa-miR-4520-3p
AGO2	hsa-miR-493
AGO2	hsa-miR-2460
AGO2	hsa-miR-3194-5p
AGO2	hsa-miR-320-5p
AGO2	hsa-miR-3911
AGO2	hsa-miR-21-5p
AGO2	hsa-miR-5125
AGO2	hsa-miR-2451
AGO2	hsa-miR-5006-3p
AGO2	hsa-miR-1255a
AGO2	hsa-miR-7271-3p
AGO2	hsa-miR-7031-5p
AGO2	hsa-miR-3927-3p
AGO2	hsa-miR-4650-5p
AGO2	hsa-miR-6786-3p
AGO2	hsa-miR-2284ac
AGO2	hsa-miR-7659-5p
AGO2	hsa-miR-5124a
AGO2	hsa-miR-3135b
AGO2	hsa-miR-545-3p
AGO2	hsa-miR-501-3p
AGO2	hsa-miR-433-3p
AGO2	hsa-miR-7169-3p
AGO2	hsa-miR-132-3p
AGO2	hsa-miR-708-3p
AGO2	hsa-miR-659-3p
AGO2	hsa-miR-2339
AGO2	hsa-miR-677-3p
AGO2	hsa-miR-344b-3p
AGO2	hsa-miR-3680-5p
AGO2	hsa-miR-7039-5p
AGO2	hsa-miR-3665
AGO2	hsa-miR-1556
AGO2	hsa-miR-7312-5p
AGO2	hsa-miR-2305
AGO2	hsa-miR-2131-3p
AGO2	hsa-miR-7369-3p
AGO2	hsa-miR-27b-3p
AGO2	hsa-miR-6879-5p

AGO2	hsa-miR-4447
AGO2	hsa-miR-6525
AGO2	hsa-miR-4692
AGO2	hsa-miR-202-3p
AGO2	hsa-miR-3938-3p
AGO2	hsa-miR-6919-5p
AGO2	hsa-miR-2355-3p
AGO2	hsa-miR-3594-3p
AGO2	hsa-miR-7048-3p
AGO2	hsa-miR-468-5p
AGO2	hsa-miR-7371d-5p
AGO2	hsa-miR-1-2-5p
AGO2	hsa-miR-363-3p
AGO2	hsa-miR-488-3p
AGO2	hsa-miR-6550-5p
AGO2	hsa-miR-1689-5p
AGO2	hsa-miR-7398l-5p
AGO2	hsa-miR-105-5p
AGO2	hsa-miR-3074-2-3p
AGO2	hsa-miR-6830-3p
AGO2	hsa-miR-1343
AGO2	hsa-miR-7074-5p
AGO2	hsa-miR-101-1-5p
AGO2	hsa-miR-769-5p
AGO2	hsa-miR-7020-3p
AGO2	hsa-miR-5586-3p
AGO2	hsa-miR-1183
AGO2	hsa-miR-201-5p
AGO2	hsa-miR-3586-3p
AGO2	hsa-miR-7282-5p
AGO2	hsa-miR-526b-5p
AGO2	hsa-miR-518g-3p
AGO2	hsa-miR-1416-3p
AGO2	hsa-miR-4513
AGO2	hsa-miR-1191a
AGO2	hsa-miR-1776
AGO2	hsa-miR-2917
AGO2	hsa-miR-7019-3p
AGO2	hsa-miR-6069
AGO2	hsa-miR-6857-5p
AGO2	hsa-miR-3611
AGO2	hsa-miR-5103
AGO2	hsa-miR-7641
AGO2	hsa-miR-4298
AGO2	hsa-miR-1756b
AGO2	hsa-miR-3126-5p

AGO2	hsa-miR-6767-3p
AGO2	hsa-miR-2397-3p
AGO2	hsa-miR-4436b-3p
AGO2	hsa-miR-6541
AGO2	hsa-miR-7394b-5p
AGO2	hsa-miR-2389
AGO2	hsa-miR-2408
AGO2	hsa-miR-6694-3p
AGO2	hsa-miR-5582-5p
AGO2	hsa-miR-487a-5p
AGO2	hsa-miR-4708-3p
AGO2	hsa-miR-27
AGO2	hsa-miR-3916
AGO2	hsa-miR-6945-3p
AGO2	hsa-miR-693-3p
AGO2	hsa-miR-702-5p
AGO2	hsa-miR-7681-3p
AGO2	hsa-miR-516-3p
AGO2	hsa-miR-6587-5p
AGO2	hsa-miR-381-3p
AGO2	hsa-miR-4462
AGO2	hsa-miR-6951-5p
AGO2	hsa-miR-8894
AGO2	hsa-miR-6793-5p
AGO2	hsa-miR-7233-3p
AGO2	hsa-miR-6119-3p
AGO2	hsa-miR-5622-3p
AGO2	hsa-miR-1267
AGO2	hsa-miR-4766-5p
AGO2	hsa-miR-5089-3p
AGO2	hsa-miR-302b-3p
AGO2	hsa-miR-3472
AGO2	hsa-miR-6527
AGO2	hsa-miR-3618-5p
AGO2	hsa-miR-499-5p
AGO2	hsa-miR-7338-3p
AGO2	hsa-miR-1275
AGO2	hsa-miR-6865-5p
AGO2	hsa-miR-7384-3p
AGO2	hsa-miR-6858-3p
AGO2	hsa-miR-1255
AGO2	hsa-miR-214-3p
AGO2	hsa-miR-1633
AGO2	hsa-miR-3573-3p
AGO2	hsa-miR-487-5p
AGO2	hsa-miR-2396

AGO2	hsa-miR-508-3p
AGO2	hsa-miR-291-5p
AGO2	hsa-miR-7246-5p
AGO2	hsa-miR-203b-3p
AGO2	hsa-miR-135-5p
AGO2	hsa-miR-7242-5p
AGO2	hsa-miR-2368-5p
AGO2	hsa-miR-370-3p
AGO2	hsa-miR-5011-3p
AGO2	hsa-miR-6640-5p
AGO2	hsa-miR-3121-3p
AGO2	hsa-miR-3092-3p
AGO2	hsa-miR-681
AGO2	hsa-miR-7163-3p
AGO2	hsa-miR-1710
AGO2	hsa-miR-7159-5p
AGO2	hsa-miR-455-3p.1
AGO2	hsa-miR-770-5p
AGO2	hsa-miR-495-5p
AGO2	hsa-miR-4258
AGO2	hsa-miR-2293
AGO2	hsa-miR-6812-5p
AGO2	hsa-miR-7a-1-3p
AGO2	hsa-miR-3072-3p
AGO2	hsa-miR-4317
AGO2	hsa-miR-302c-5p
AGO2	hsa-miR-7347-5p
AGO2	hsa-miR-6659-3p
AGO2	hsa-miR-4482-3p
AGO2	hsa-miR-6682-3p
AGO2	hsa-miR-221-5p
AGO2	hsa-miR-517-3p
AGO2	hsa-miR-7442-5p
AGO2	hsa-miR-8858
AGO2	hsa-miR-1255-5p
AGO2	hsa-miR-7666-5p
AGO2	hsa-miR-7369-5p
AGO2	hsa-miR-940
AGO2	hsa-miR-6814-5p
AGO2	hsa-miR-888-3p
AGO2	hsa-miR-7343-3p
AGO2	hsa-miR-7854-3p
AGO2	hsa-miR-1245a
AGO2	hsa-miR-677
AGO2	hsa-miR-451-3p
AGO2	hsa-miR-292-5p

AGO2	hsa-miR-1296-3p
AGO2	hsa-miR-8827
AGO2	hsa-miR-6988-5p
AGO2	hsa-miR-7181-3p
AGO2	hsa-miR-1225
AGO2	hsa-miR-2447
AGO2	hsa-miR-2411-3p
AGO2	hsa-miR-6358
AGO2	hsa-miR-499b-3p
AGO2	hsa-miR-191-3p
AGO2	hsa-miR-4506
AGO2	hsa-miR-6623-3p
AGO2	hsa-miR-499b-5p
AGO2	hsa-miR-221-3p
AGO2	hsa-miR-6998-3p
AGO2	hsa-miR-7347-3p
AGO2	hsa-miR-6755-3p
AGO2	hsa-miR-466-5p
AGO2	hsa-miR-221-3p
AGO2	hsa-miR-6993-5p
AGO2	hsa-miR-516b-5p
AGO2	hsa-miR-7350-3p
AGO2	hsa-miR-6502-3p
AGO2	hsa-miR-8893
AGO2	hsa-miR-7442-3p
AGO2	hsa-miR-30
AGO2	hsa-miR-706
AGO2	hsa-miR-1723
AGO2	hsa-miR-8849
AGO2	hsa-miR-7200-5p
AGO2	hsa-miR-3176
AGO2	hsa-miR-6775-3p
AGO2	hsa-miR-7018-3p
AGO2	hsa-miR-4757-3p
AGO2	hsa-miR-218-5p
AGO2	hsa-miR-7307-3p
AGO2	hsa-miR-7175-3p
AGO2	hsa-miR-7703
AGO2	hsa-miR-1897-5p
AGO2	hsa-miR-466g
AGO2	hsa-miR-105b
AGO2	hsa-miR-2338
AGO2	hsa-miR-183-3p
AGO2	hsa-miR-148b-3p
AGO2	hsa-miR-516a-3p
AGO2	hsa-miR-4772-3p

AGO2	hsa-miR-7386j-3p
AGO2	hsa-miR-883b-5p
AGO2	hsa-miR-8838
AGO2	hsa-miR-3193
AGO2	hsa-miR-4284
AGO2	hsa-miR-7974
AGO2	hsa-miR-7469-5p
AGO2	hsa-let-7j-3p
AGO2	hsa-miR-8888
AGO2	hsa-miR-548c-3p
AGO2	hsa-miR-2318
AGO2	hsa-miR-7458-3p
AGO2	hsa-miR-6759-5p
AGO2	hsa-miR-6345
AGO2	hsa-miR-6760-5p
AGO2	hsa-miR-7330-3p
AGO2	hsa-miR-7382-3p
AGO2	hsa-miR-101b-3p.1
AGO2	hsa-miR-378a-5p
AGO2	hsa-miR-8899
AGO2	hsa-miR-7309-3p
AGO2	hsa-miR-290b-3p
AGO2	hsa-miR-496a-3p.1
AGO2	hsa-miR-7660-5p
AGO2	hsa-miR-6916-3p
AGO2	hsa-miR-3967
AGO2	hsa-miR-670-5p
AGO2	hsa-miR-4743-3p
AGO2	hsa-miR-5625-3p
AGO2	hsa-miR-216b
AGO2	hsa-miR-374a-3p
AGO2	hsa-miR-936
AGO2	hsa-miR-7351-3p
AGO2	hsa-miR-871-5p
AGO2	hsa-miR-6946-3p
AGO2	hsa-miR-3167-3p
AGO2	hsa-miR-7448-5p
AGO2	hsa-miR-7062-3p
AGO2	hsa-miR-7371e-3p
AGO2	hsa-miR-532-5p
AGO2	hsa-miR-6379
AGO2	hsa-miR-4664-5p
AGO2	hsa-miR-6789-5p
AGO2	hsa-miR-6864-5p
AGO2	hsa-miR-6912-3p
AGO2	hsa-miR-522-3p

AGO2	hsa-let-7
AGO2	hsa-miR-3140-3p
AGO2	hsa-miR-182-5p
AGO2	hsa-miR-374
AGO2	hsa-miR-6513-5p
AGO2	hsa-miR-4756-3p
AGO2	hsa-miR-516a
AGO2	hsa-miR-7651-3p
AGO2	hsa-miR-1667-3p
AGO2	hsa-miR-335-3p
AGO2	hsa-miR-374c-3p
AGO2	hsa-miR-8550
AGO2	hsa-miR-4651
AGO2	hsa-miR-4712-3p
AGO2	hsa-miR-2299-3p
AGO2	hsa-miR-6757-3p
AGO2	hsa-miR-1306-5p
AGO2	hsa-miR-1285-3p
AGO2	hsa-miR-199-5p
AGO2	hsa-miR-1668-5p
AGO2	hsa-miR-664-5p
AGO2	hsa-miR-497-5p
AGO2	hsa-miR-412-3p
AGO2	hsa-miR-8792
AGO2	hsa-miR-3125
AGO2	hsa-miR-340-5p
AGO2	hsa-miR-6944-5p
AGO2	hsa-miR-3102-3p.2-3p
AGO2	hsa-miR-1835
AGO2	hsa-miR-4671-3p
AGO2	hsa-miR-1659
AGO2	hsa-miR-6513-3p
AGO2	hsa-miR-550a-3p
AGO2	hsa-miR-4772-5p
AGO2	hsa-miR-5123
AGO2	hsa-miR-30a-5p
AGO2	hsa-miR-148a-5p
AGO2	hsa-miR-1910-5p
AGO2	hsa-miR-7661-5p
AGO2	hsa-miR-7398o-5p
AGO2	hsa-miR-518
AGO2	hsa-miR-7375-3p
AGO2	hsa-miR-7371h-3p
AGO2	hsa-miR-432-5p
AGO2	hsa-miR-6939-3p
AGO2	hsa-miR-7379-5p

AGO2	hsa-miR-4433a-3p
AGO2	hsa-miR-7254-5p
AGO2	hsa-miR-668-3p
AGO2	hsa-miR-3094-3p
AGO2	hsa-miR-7028-3p
AGO2	hsa-miR-3928-3p
AGO2	hsa-miR-15
AGO2	hsa-miR-3660
AGO2	hsa-miR-2284x
AGO2	hsa-miR-6810-5p
AGO2	hsa-miR-181c-5p
AGO2	hsa-miR-1224-5p
AGO2	hsa-miR-130-3p
AGO2	hsa-miR-4764-3p
AGO2	hsa-miR-874-5p
AGO2	hsa-miR-375
AGO2	hsa-miR-1805-5p
AGO2	hsa-miR-7288-3p
AGO2	hsa-miR-6544-3p
AGO2	hsa-miR-301-5p
AGO2	hsa-miR-135-3p
AGO2	hsa-miR-376-3p
AGO2	hsa-miR-3190-5p
AGO2	hsa-miR-6856-5p
AGO2	hsa-miR-505-3p.2
AGO2	hsa-miR-2394
AGO2	hsa-miR-6785-3p
AGO2	hsa-miR-7151-3p
AGO2	hsa-miR-3685
AGO2	hsa-miR-486-3p
AGO2	hsa-miR-4701-3p
AGO2	hsa-miR-6728-5p
AGO2	hsa-miR-576-5p
AGO2	hsa-miR-924
AGO2	hsa-miR-3202
AGO2	hsa-miR-6822-5p
AGO2	hsa-miR-7216-3p
AGO2	hsa-miR-7214-3p
AGO2	hsa-miR-7378-5p
AGO2	hsa-miR-579-3p
AGO2	hsa-miR-1238-3p
AGO2	hsa-miR-6788-5p
AGO2	hsa-miR-6500-3p
AGO2	hsa-miR-2388-5p
AGO2	hsa-miR-7340-3p
AGO2	hsa-miR-363-5p

AGO2	hsa-miR-152
AGO2	hsa-miR-24-5p
AGO2	hsa-miR-346-5p
AGO2	hsa-miR-4799-5p
AGO2	hsa-miR-181c-3p
AGO2	hsa-miR-3187-3p
AGO2	hsa-miR-5107-5p
AGO2	hsa-miR-2334
AGO2	hsa-miR-7310-5p
AGO2	hsa-miR-539-5p
AGO2	hsa-miR-7087-3p
AGO2	hsa-miR-5196-5p
AGO2	hsa-miR-1618-5p
AGO2	hsa-miR-204-5p
AGO2	hsa-miR-7978
AGO2	hsa-miR-698-5p
AGO2	hsa-miR-212-3p
AGO2	hsa-miR-935
AGO2	hsa-miR-577-3p
AGO2	hsa-miR-3200-5p
AGO2	hsa-miR-891
AGO2	hsa-miR-2433
AGO2	hsa-miR-7262-3p
AGO2	hsa-miR-140-3p
AGO2	hsa-miR-196-3p
AGO2	hsa-miR-6333
AGO2	hsa-miR-4293
AGO2	hsa-miR-937-3p
AGO2	hsa-miR-2435
AGO2	hsa-miR-7368-3p
AGO2	hsa-miR-878-5p
AGO2	hsa-miR-670-3p
AGO2	hsa-miR-466i-3p
AGO2	hsa-miR-7460-5p
AGO2	hsa-miR-6550-3p
AGO2	hsa-miR-371a-5p
AGO2	hsa-miR-423-3p
AGO2	hsa-miR-3922-3p
AGO2	hsa-miR-6885-3p
AGO2	hsa-miR-3583-3p
AGO2	hsa-miR-8908f
AGO2	hsa-miR-6692-5p
AGO2	hsa-miR-548b-5p
AGO2	hsa-miR-4434
AGO2	hsa-miR-185-5p
AGO2	hsa-miR-6362

AGO2	hsa-miR-7094b-2-5p
AGO2	hsa-miR-557
AGO2	hsa-miR-1782
AGO2	hsa-miR-7674-5p
AGO2	hsa-miR-3090-3p
AGO2	hsa-miR-6776-3p
AGO2	hsa-miR-6901-3p
AGO2	hsa-miR-4653-3p
AGO2	hsa-miR-1457
AGO2	hsa-miR-146
AGO2	hsa-miR-549-3p
AGO2	hsa-miR-27b-5p
AGO2	hsa-miR-7354-5p
AGO2	hsa-miR-6865-3p
AGO2	hsa-miR-186-5p
AGO2	hsa-miR-181-3p
AGO2	hsa-miR-553
AGO2	hsa-miR-2380
AGO2	hsa-miR-6529-3p
AGO2	hsa-miR-514-3p
AGO2	hsa-miR-384-3p
AGO2	hsa-miR-431-5p
AGO2	hsa-miR-617
AGO2	hsa-miR-600
AGO2	hsa-miR-6724-5p
AGO2	hsa-miR-2466-5p
AGO2	hsa-miR-5008-3p
AGO2	hsa-miR-2284aa
AGO2	hsa-miR-7251-5p
AGO2	hsa-miR-4753-3p
AGO2	hsa-miR-6612-5p
AGO2	hsa-miR-6727-3p
AGO2	hsa-miR-7844-5p
AGO2	hsa-miR-1239
AGO2	hsa-miR-483-3p.1
AGO2	hsa-miR-4689
AGO2	hsa-miR-7279-5p
AGO2	hsa-miR-1781-3p
AGO2	hsa-miR-324
AGO2	hsa-miR-23
AGO2	hsa-miR-4477b
AGO2	hsa-miR-873a-3p
AGO2	hsa-miR-8823
AGO2	hsa-miR-8836
AGO2	hsa-miR-548x-5p
AGO2	hsa-miR-4781-3p

AGO2	hsa-miR-518-5p
AGO2	hsa-miR-630
AGO2	hsa-miR-340-3p
AGO2	hsa-miR-6813-3p
AGO2	hsa-miR-611
AGO2	hsa-miR-6839-3p
AGO2	hsa-miR-657
AGO2	hsa-miR-879-3p
AGO2	hsa-miR-494
AGO2	hsa-miR-1842
AGO2	hsa-miR-2276-3p
AGO2	hsa-miR-6987-3p
AGO2	hsa-miR-3136-5p
AGO2	hsa-miR-2325c
AGO2	hsa-miR-1188-5p
AGO2	hsa-miR-466
AGO2	hsa-miR-5120
AGO2	hsa-miR-5621-3p
AGO2	hsa-miR-4746-3p
AGO2	hsa-miR-6340
AGO2	hsa-miR-7198-5p
AGO2	hsa-miR-2387
AGO2	hsa-miR-6323
AGO2	hsa-miR-1744-3p
AGO2	hsa-miR-6951-3p
AGO2	hsa-miR-194-2-3p
AGO2	hsa-miR-1843a-5p
AGO2	hsa-miR-29a-3p
AGO2	hsa-miR-1264-5p
AGO2	hsa-miR-5689
AGO2	hsa-miR-7683-3p
AGO2	hsa-miR-6935-3p
AGO2	hsa-miR-506-3p
AGO2	hsa-miR-7004-3p
AGO2	hsa-miR-4520-2-3p
AGO2	hsa-miR-320a
AGO2	hsa-miR-7-5p
AGO2	hsa-miR-3529-3p
AGO2	hsa-miR-7476-3p
AGO2	hsa-miR-370
AGO2	hsa-miR-1293
AGO2	hsa-miR-8059
AGO2	hsa-miR-6800-3p
AGO2	hsa-miR-6897-5p
AGO2	hsa-miR-5096
AGO2	hsa-miR-6923-3p

AGO2	hsa-miR-224-5p
AGO2	hsa-miR-4728-3p
AGO2	hsa-miR-637
AGO2	hsa-miR-1902
AGO2	hsa-miR-507b
AGO2	hsa-miR-488-5p
AGO2	hsa-miR-327
AGO2	hsa-miR-7298-3p
AGO2	hsa-miR-4453
AGO2	hsa-miR-4727-3p
AGO2	hsa-miR-7240-5p
AGO2	hsa-miR-4439
AGO2	hsa-miR-3961
AGO2	hsa-miR-6884-3p
AGO2	hsa-miR-365-5p
AGO2	hsa-miR-4467
AGO2	hsa-miR-4257
AGO2	hsa-miR-25-3p
AGO2	hsa-miR-3940-5p
AGO2	hsa-miR-7013-3p
AGO2	hsa-miR-30c-3p
AGO2	hsa-miR-2458
AGO2	hsa-miR-8106
AGO2	hsa-miR-760-5p
AGO2	hsa-miR-7684-5p
AGO2	hsa-miR-2390
AGO2	hsa-miR-516a-5p
AGO2	hsa-miR-6660-3p
AGO2	hsa-miR-6120-5p
AGO2	hsa-miR-7012-3p
AGO2	hsa-miR-6748-3p
AGO2	hsa-miR-656-3p
AGO2	hsa-miR-4641
AGO2	hsa-miR-8055
AGO2	hsa-miR-2432
AGO2	hsa-miR-1545a-3p
AGO2	hsa-miR-3473c
AGO2	hsa-miR-548ap-5p
AGO2	hsa-miR-3062-5p
AGO2	hsa-miR-7005-3p
AGO2	hsa-miR-686
AGO2	hsa-miR-4739
AGO2	hsa-miR-586
AGO2	hsa-miR-7385d-3p
AGO2	hsa-miR-5579-3p
AGO2	hsa-miR-3606-5p

AGO2	hsa-miR-3136-5p
AGO2	hsa-miR-450a-1-3p
AGO2	hsa-miR-6558-5p
AGO2	hsa-miR-512-3p
AGO2	hsa-miR-1684-5p
AGO2	hsa-miR-7a-2-3p
AGO2	hsa-miR-344d-2-5p
AGO2	hsa-miR-1252-3p
AGO2	hsa-miR-5088-3p
AGO2	hsa-miR-135a-23-3p
AGO2	hsa-miR-372
AGO2	hsa-miR-2359
AGO2	hsa-miR-2117
AGO2	hsa-miR-7180-3p
AGO2	hsa-miR-7304-5p
AGO2	hsa-miR-2299-5p
AGO2	hsa-miR-6637-3p
AGO2	hsa-miR-34c-5p
AGO2	hsa-miR-4745-5p
AGO2	hsa-miR-365-12-5p
AGO2	hsa-miR-8485
AGO2	hsa-miR-3692-3p
AGO2	hsa-miR-8885
AGO2	hsa-miR-6758-5p
AGO2	hsa-miR-3606-3p
AGO2	hsa-miR-6418-5p
AGO2	hsa-miR-3146
AGO2	hsa-miR-3473be
AGO2	hsa-miR-6582-5p
AGO2	hsa-miR-8904b
AGO2	hsa-miR-3058-5p
AGO2	hsa-miR-150-3p
AGO2	hsa-miR-669k-5p
AGO2	hsa-miR-135ab-3p
AGO2	hsa-miR-5700
AGO2	hsa-miR-7009-5p
AGO2	hsa-miR-5681b
AGO2	hsa-miR-3143
AGO2	hsa-miR-290b-5p
AGO2	hsa-miR-1739
AGO2	hsa-miR-6851-3p
AGO2	hsa-miR-1-5p
AGO2	hsa-miR-298-3p
AGO2	hsa-miR-7685-3p
AGO2	hsa-miR-6938-3p
AGO2	hsa-miR-1788-5p

AGO2	hsa-miR-342-3p
AGO2	hsa-miR-6840-3p
AGO2	hsa-miR-8898
AGO2	hsa-miR-3616-3p
AGO2	hsa-miR-4507
AGO2	hsa-miR-3572-5p
AGO2	hsa-miR-7466-3p
AGO2	hsa-miR-2308
AGO2	hsa-miR-628-3p
AGO2	hsa-miR-1784-5p
AGO2	hsa-miR-6600-3p
AGO2	hsa-miR-7365-3p
AGO2	hsa-miR-1552-3p
AGO2	hsa-miR-6536
AGO2	hsa-miR-3105-3p
AGO2	hsa-miR-3150b-5p
AGO2	hsa-miR-2482
AGO2	hsa-miR-3577-5p
AGO2	hsa-miR-3141
AGO2	hsa-miR-7398-5p
AGO2	hsa-miR-6808-5p
AGO2	hsa-miR-3150a-3p
AGO2	hsa-miR-702-3p
AGO2	hsa-miR-1643b-5p
AGO2	hsa-miR-199b-3-5p
AGO2	hsa-miR-5618-5p
AGO2	hsa-miR-7337-3p
AGO2	hsa-miR-1583
AGO2	hsa-miR-6813-5p
AGO2	hsa-miR-1545b-5p
AGO2	hsa-miR-612
AGO2	hsa-miR-302d-3p
AGO2	hsa-miR-650d
AGO2	hsa-miR-2468
AGO2	hsa-miR-6924-5p
AGO2	hsa-miR-763
AGO2	hsa-miR-7360-3p
AGO2	hsa-miR-7005-5p
AGO2	hsa-miR-212-3p
AGO2	hsa-let-7b-3p
AGO2	hsa-miR-1236-5p
AGO2	hsa-miR-493-5p
AGO2	hsa-miR-210
AGO2	hsa-miR-4761-3p
AGO2	hsa-miR-219-2-3p
AGO2	hsa-miR-7389-5p

AGO2	hsa-miR-6874-5p
AGO2	hsa-miR-635
AGO2	hsa-miR-1323-5p
AGO2	hsa-miR-7039-3p
AGO2	hsa-miR-7211-3p
AGO2	hsa-miR-1227-3p
AGO2	hsa-miR-516
AGO2	hsa-miR-99-3p
AGO2	hsa-miR-8088
AGO2	hsa-miR-4511
AGO2	hsa-miR-7065-3p
AGO2	hsa-miR-3072
AGO2	hsa-miR-4697-3p
AGO2	hsa-miR-1952
AGO2	hsa-miR-19b-3p
AGO2	hsa-miR-7682-3p
AGO2	hsa-miR-7035-3p
AGO2	hsa-miR-497-5p
AGO2	hsa-miR-205-3p
AGO2	hsa-miR-7395-3p
AGO2	hsa-miR-29a-1-5p
AGO2	hsa-miR-7386d-5p
AGO2	hsa-miR-2367-3p
AGO2	hsa-miR-7283-3p
AGO2	hsa-miR-769-3p
AGO2	hsa-miR-4776-3p
AGO2	hsa-miR-1735
AGO2	hsa-miR-4518
AGO2	hsa-miR-1249-3p
AGO2	hsa-miR-4728-5p
AGO2	hsa-miR-7203-5p
AGO2	hsa-miR-1740-3p
AGO2	hsa-miR-669b-5p
AGO2	hsa-miR-1683
AGO2	hsa-miR-7049-5p
AGO2	hsa-miR-7111-3p
AGO2	hsa-miR-3966
AGO2	hsa-miR-1894-3p
AGO2	hsa-miR-146a-3p
AGO2	hsa-miR-7197-5p
AGO2	hsa-miR-6739-5p
AGO2	hsa-miR-1680-5p
AGO2	hsa-miR-3601
AGO2	hsa-miR-4524a-5p
AGO2	hsa-miR-4460
AGO2	hsa-miR-6954-3p

AGO2	hsa-miR-7203-3p
AGO2	hsa-miR-3189-3p
AGO2	hsa-miR-1456-3p
AGO2	hsa-miR-2317
AGO2	hsa-miR-30e
AGO2	hsa-miR-7062-5p
AGO2	hsa-miR-3084a-5p
AGO2	hsa-miR-541-3p
AGO2	hsa-miR-7188-3p
AGO2	hsa-miR-8079
AGO2	hsa-miR-3662
AGO2	hsa-let-7a-2-3p
AGO2	hsa-miR-98-3p
AGO2	hsa-miR-8054
AGO2	hsa-miR-582-5p
AGO2	hsa-miR-151a-5p
AGO2	hsa-miR-133c
AGO2	hsa-miR-1943-5p
AGO2	hsa-miR-548m
AGO2	hsa-miR-548i-3p
AGO2	hsa-miR-7032-3p
AGO2	hsa-miR-193b-5p
AGO2	hsa-miR-8115
AGO2	hsa-let-7f-2-3p
AGO2	hsa-miR-7042-5p
AGO2	hsa-miR-3653-5p
AGO2	hsa-miR-6966-3p
AGO2	hsa-miR-4678
AGO2	hsa-miR-200c-3p
AGO2	hsa-miR-6328
AGO2	hsa-miR-4288
AGO2	hsa-miR-345-5p
AGO2	hsa-miR-7114-5p
AGO2	hsa-miR-1237-3p
AGO2	hsa-miR-3121-5p
AGO2	hsa-miR-3689b-5p
AGO2	hsa-miR-2483-5p
AGO2	hsa-miR-4263
AGO2	hsa-miR-548g-5p
AGO2	hsa-miR-6908-3p
AGO2	hsa-miR-4800-3p
AGO2	hsa-miR-591
C17orf85	hsa-miR-4716-5p
C17orf85	hsa-miR-548z
C17orf85	hsa-miR-7198-5p
C17orf85	hsa-miR-643

C17orf85	hsa-miR-3134
C17orf85	hsa-miR-195-5p
C17orf85	hsa-miR-518c-5p
C17orf85	hsa-miR-6790-5p
C17orf85	hsa-miR-6868-5p
C17orf85	hsa-miR-3129-3p
C17orf85	hsa-miR-8879
C17orf85	hsa-miR-2425-5p
C17orf85	hsa-miR-1252-3p
C17orf85	hsa-miR-4524b-3p
C17orf85	hsa-miR-8905
C17orf85	hsa-miR-369-5p
C17orf85	hsa-miR-8119
C17orf85	hsa-miR-6958-5p
C17orf85	hsa-miR-1626-3p
C17orf85	hsa-miR-467g
C17orf85	hsa-miR-6384
C17orf85	hsa-miR-6805-5p
C17orf85	hsa-miR-7460-3p
C17orf85	hsa-miR-5001-5p
C17orf85	hsa-miR-7188-3p
C17orf85	hsa-miR-1251
C17orf85	hsa-miR-2368-5p
C17orf85	hsa-miR-1968-3p
C17orf85	hsa-miR-101-3p
C17orf85	hsa-miR-20-5p
C17orf85	hsa-miR-4271
C17orf85	hsa-miR-365a-3p
C17orf85	hsa-miR-513a
C17orf85	hsa-miR-576-5p
C17orf85	hsa-miR-429-3p
C17orf85	hsa-miR-3110-3p
C17orf85	hsa-miR-133
C17orf85	hsa-miR-553
C17orf85	hsa-miR-4288
C17orf85	hsa-miR-548y
C17orf85	hsa-miR-103-3p
C17orf85	hsa-miR-361-5p
C17orf85	hsa-miR-6952-5p
C17orf85	hsa-miR-141-3p
C17orf85	hsa-miR-944
C17orf85	hsa-miR-27a-3p
C17orf85	hsa-miR-185-3p
C17orf85	hsa-miR-92
C17orf85	hsa-miR-1292-3p
C17orf85	hsa-miR-1258-5p

C17orf85	hsa-miR-454-3p
C17orf85	hsa-miR-4783-5p
C17orf85	hsa-miR-6125
C17orf85	hsa-miR-323b-5p
C17orf85	hsa-miR-3604
C17orf85	hsa-miR-5589-5p
C17orf85	hsa-miR-377-3p
C17orf85	hsa-miR-592
C17orf85	hsa-miR-7225-3p
C17orf85	hsa-miR-1343
C17orf85	hsa-miR-99a-3p
C17orf85	hsa-miR-760-3p
C17orf85	hsa-miR-6806-3p
C17orf85	hsa-miR-3680-3p
C17orf85	hsa-miR-4437
C17orf85	hsa-miR-292b-3p
C17orf85	hsa-miR-384-5p
C17orf85	hsa-miR-3921
C17orf85	hsa-miR-101-2-5p
C17orf85	hsa-miR-548h-5p
C17orf85	hsa-miR-147a
C17orf85	hsa-miR-488-3p
C17orf85	hsa-miR-6715-5p
C17orf85	hsa-miR-4764-3p
C17orf85	hsa-miR-5193
C17orf85	hsa-miR-518g-3p
C17orf85	hsa-miR-5685
C17orf85	hsa-miR-144-5p
C17orf85	hsa-miR-2347
C17orf85	hsa-miR-324-3p
C17orf85	hsa-miR-3060-5p
C17orf85	hsa-miR-7051-5p
C17orf85	hsa-miR-6871-5p
C17orf85	hsa-miR-766-3p
C17orf85	hsa-miR-7216-5p
C17orf85	hsa-miR-30-5p
C17orf85	hsa-miR-1228-3p
C17orf85	hsa-miR-1843b-5p
C17orf85	hsa-miR-526
C17orf85	hsa-miR-141-3p
C17orf85	hsa-miR-2467-3p
C17orf85	hsa-miR-548f-5p
C17orf85	hsa-miR-4696
C17orf85	hsa-miR-342-3p
C17orf85	hsa-miR-7005-5p
C17orf85	hsa-miR-6864-3p

C17orf85	hsa-miR-291b-3p
C17orf85	hsa-miR-6932-5p
C17orf85	hsa-miR-8880
C17orf85	hsa-miR-153-5p
C17orf85	hsa-miR-7213-3p
C17orf85	hsa-miR-7331-5p
C17orf85	hsa-miR-6813-3p
C17orf85	hsa-miR-145-3p
C17orf85	hsa-miR-4314
C17orf85	hsa-miR-1296
C17orf85	hsa-miR-3057-3p
C17orf85	hsa-miR-4640-5p
C17orf85	hsa-miR-17
C17orf85	hsa-miR-697
C17orf85	hsa-miR-6837-3p
C17orf85	hsa-miR-7117-5p
C17orf85	hsa-miR-548j-3p
C17orf85	hsa-miR-145-5p
C17orf85	hsa-miR-1249-3p
C17orf85	hsa-let-7g-3p
C17orf85	hsa-miR-297
C17orf85	hsa-miR-6744-5p
C17orf85	hsa-miR-5186
C17orf85	hsa-miR-4727-5p
C17orf85	hsa-miR-579-3p
C17orf85	hsa-miR-5681b
C17orf85	hsa-miR-6069
C17orf85	hsa-miR-2894
C17orf85	hsa-miR-7076-3p
C17orf85	hsa-miR-6416-3p
C17orf85	hsa-miR-150-3p
C17orf85	hsa-miR-641
C17orf85	hsa-miR-6831-5p
C17orf85	hsa-miR-133a-5p
C17orf85	hsa-miR-1252
C17orf85	hsa-miR-7656-3p
C17orf85	hsa-miR-6752-3p
C17orf85	hsa-miR-216b-5p
C17orf85	hsa-miR-548x-5p
C17orf85	hsa-miR-3678-3p
C17orf85	hsa-miR-4487
C17orf85	hsa-miR-7065-5p
C17orf85	hsa-miR-599-3p
C17orf85	hsa-miR-6832-3p
C17orf85	hsa-miR-7085-5p
C17orf85	hsa-miR-4296

C17orf85	hsa-miR-7221-3p
C17orf85	hsa-miR-4645-5p
C17orf85	hsa-miR-7111-5p
C17orf85	hsa-miR-7010-3p
C17orf85	hsa-miR-1903
C17orf85	hsa-miR-2891
C17orf85	hsa-miR-3177-5p
C17orf85	hsa-miR-4725-5p
C17orf85	hsa-miR-138-3p
C17orf85	hsa-miR-4690-3p
C17orf85	hsa-miR-4538
C17orf85	hsa-miR-4520-2-3p
C17orf85	hsa-miR-513a-3p
C17orf85	hsa-miR-708-3p
C17orf85	hsa-miR-802-5p
C17orf85	hsa-miR-9500
C17orf85	hsa-miR-6937-3p
C17orf85	hsa-miR-3675-3p
C17orf85	hsa-miR-3074-3p
C17orf85	hsa-miR-7011-3p
C17orf85	hsa-miR-669p-3p
C17orf85	hsa-miR-4750-3p
C17orf85	hsa-miR-4302
C17orf85	hsa-miR-1-3p
C17orf85	hsa-miR-4427
C17orf85	hsa-miR-3620-5p
C17orf85	hsa-miR-1816
C17orf85	hsa-miR-6858-5p
C17orf85	hsa-let-7a-2-3p
C17orf85	hsa-miR-18-5p
C17orf85	hsa-miR-343
C17orf85	hsa-miR-1286
C17orf85	hsa-miR-3072-5p
C17orf85	hsa-miR-146
C17orf85	hsa-miR-6732-3p
C17orf85	hsa-miR-1243-5p
C17orf85	hsa-miR-3911
C17orf85	hsa-miR-325
C17orf85	hsa-miR-6972-5p
C17orf85	hsa-miR-128-5p
C17orf85	hsa-miR-7235-5p
C17orf85	hsa-miR-380-3p
C17orf85	hsa-miR-883-5p
C17orf85	hsa-miR-4728-5p
C17orf85	hsa-miR-2909
C17orf85	hsa-miR-2460

C17orf85	hsa-miR-6906-5p
C17orf85	hsa-miR-3590-3p
C17orf85	hsa-miR-1273h-5p
C17orf85	hsa-miR-344g-3p
C17orf85	hsa-miR-632
C17orf85	hsa-miR-17-5p
C17orf85	hsa-miR-509-3p
C17orf85	hsa-miR-6893-5p
C17orf85	hsa-miR-421-3p
C17orf85	hsa-miR-2419-5p
C17orf85	hsa-miR-329-5p
C17orf85	hsa-miR-6216
C17orf85	hsa-miR-7020-5p
C17orf85	hsa-miR-340-3p
C17orf85	hsa-miR-499-5p
C17orf85	hsa-miR-1981-3p
C17orf85	hsa-miR-4433b-3p
C17orf85	hsa-miR-463-3p
C17orf85	hsa-miR-4530
C17orf85	hsa-miR-3098-3p
C17orf85	hsa-miR-184-5p
C17orf85	hsa-miR-135-3p
C17orf85	hsa-miR-4642
C17orf85	hsa-miR-320-3p
C17orf85	hsa-miR-485
C17orf85	hsa-miR-942
C17orf85	hsa-miR-2467-5p
C17orf85	hsa-miR-7683-5p
C17orf85	hsa-miR-7681-5p
C17orf85	hsa-miR-6087
C17orf85	hsa-miR-376a-5p
C17orf85	hsa-miR-4287
C17orf85	hsa-miR-593-3p
C17orf85	hsa-miR-549-3p
C17orf85	hsa-miR-8873b
C17orf85	hsa-miR-597-3p
C17orf85	hsa-miR-506-5p
C17orf85	hsa-miR-8057
C17orf85	hsa-miR-637
C17orf85	hsa-miR-3184-3p
C17orf85	hsa-miR-7224-3p
C17orf85	hsa-miR-597-5p
C17orf85	hsa-miR-483-5p
C17orf85	hsa-miR-579
C17orf85	hsa-miR-1245-3p
C17orf85	hsa-miR-181c-3p

C17orf85	hsa-miR-4749-5p
C17orf85	hsa-miR-3928-3p
C17orf85	hsa-miR-3561-3p
C17orf85	hsa-miR-7053-3p
C17orf85	hsa-miR-4472
C17orf85	hsa-miR-7185-5p
C17orf85	hsa-miR-7111-3p
C17orf85	hsa-miR-7236-5p
C17orf85	hsa-miR-7182-3p
C17orf85	hsa-miR-4422
C17orf85	hsa-miR-5000-5p
C17orf85	hsa-miR-6345
C17orf85	hsa-miR-191-5p
C17orf85	hsa-miR-1231-3p
C17orf85	hsa-miR-6808-5p
C17orf85	hsa-miR-6315
C17orf85	hsa-miR-1897-5p
C17orf85	hsa-miR-8825
C17orf85	hsa-miR-6949-3p
C17orf85	hsa-miR-212-3p
C17orf85	hsa-miR-680
C17orf85	hsa-miR-7171-3p
C17orf85	hsa-miR-5696
C17orf85	hsa-miR-618
C17orf85	hsa-miR-7106-5p
C17orf85	hsa-miR-8908-5p
C17orf85	hsa-miR-6964-3p
C17orf85	hsa-miR-218a-2-3p
C17orf85	hsa-miR-7199-5p
C17orf85	hsa-miR-4755-3p
C17orf85	hsa-miR-199-3p
C17orf85	hsa-miR-4539
C17orf85	hsa-miR-548g-5p
C17orf85	hsa-miR-128-3p
C17orf85	hsa-miR-134
C17orf85	hsa-miR-7653-5p
C17orf85	hsa-miR-548n
C17orf85	hsa-miR-320-5p
C17orf85	hsa-miR-3557-5p
C17orf85	hsa-miR-586
C17orf85	hsa-miR-3689d
C17orf85	hsa-miR-6919-3p
C17orf85	hsa-miR-7243-3p
C17orf85	hsa-miR-3136-5p
C17orf85	hsa-miR-7168-3p
C17orf85	hsa-miR-696

C17orf85	hsa-miR-325-5p
C17orf85	hsa-miR-4419a
C17orf85	hsa-miR-6816-3p
C17orf85	hsa-miR-98-5p
C17orf85	hsa-miR-6072
C17orf85	hsa-miR-6900-3p
C17orf85	hsa-miR-3654
C17orf85	hsa-miR-23a-5p
C17orf85	hsa-miR-3183
C17orf85	hsa-miR-7277-3p
C17orf85	hsa-miR-548c-3p
C17orf85	hsa-miR-6779-3p
C17orf85	hsa-miR-6898-3p
C17orf85	hsa-miR-3944-5p
C17orf85	hsa-miR-3185
C17orf85	hsa-miR-223-3p
C17orf85	hsa-miR-653-3p
C17orf85	hsa-miR-3542
C17orf85	hsa-miR-3609
C17orf85	hsa-miR-6649-3p
C17orf85	hsa-miR-516a-3p
C17orf85	hsa-miR-532-3p
C17orf85	hsa-miR-141-5p
C17orf85	hsa-miR-5623-3p
C17orf85	hsa-miR-103
C17orf85	hsa-miR-3165
C17orf85	hsa-miR-1837
C17orf85	hsa-miR-4650-5p
C17orf85	hsa-miR-7377-5p
C17orf85	hsa-miR-2339
C17orf85	hsa-miR-4494
C17orf85	hsa-miR-1197
C17orf85	hsa-miR-4741
C17orf85	hsa-miR-195-3p
C17orf85	hsa-miR-7019-3p
C17orf85	hsa-miR-468-5p
C17orf85	hsa-miR-674-3p
C17orf85	hsa-miR-700-5p
C17orf85	hsa-miR-216-3p
C17orf85	hsa-miR-8052
C17orf85	hsa-miR-1283a
C17orf85	hsa-miR-7851-3p
C17orf85	hsa-miR-4722-3p
C17orf85	hsa-miR-181a-2-3p
C17orf85	hsa-miR-5011-5p
C17orf85	hsa-miR-664a-3p

C17orf85	hsa-miR-26a-3p
C17orf85	hsa-miR-410-5p
C17orf85	hsa-miR-142-5p
C17orf85	hsa-miR-7178-5p
C17orf85	hsa-miR-3619-5p
C17orf85	hsa-miR-1956-3p
C17orf85	hsa-miR-712-5p
C17orf85	hsa-miR-302-3p
C17orf85	hsa-miR-1911-3p
C17orf85	hsa-miR-4679
C17orf85	hsa-miR-4293
C17orf85	hsa-miR-7228-3p
C17orf85	hsa-miR-133-3p.1
C17orf85	hsa-miR-122
C17orf85	hsa-miR-2277-3p
C17orf85	hsa-miR-4802-3p
C17orf85	hsa-miR-6946-3p
C17orf85	hsa-miR-32-5p
C17orf85	hsa-miR-378g
C17orf85	hsa-miR-628-3p
C17orf85	hsa-miR-8113
C17orf85	hsa-miR-6799-5p
C17orf85	hsa-miR-613
C17orf85	hsa-miR-3692-3p
C17orf85	hsa-miR-5000-3p
C17orf85	hsa-miR-8805
C17orf85	hsa-miR-7185-3p
C17orf85	hsa-miR-6128
C17orf85	hsa-miR-6740-5p
C17orf85	hsa-miR-218-1-3p
C17orf85	hsa-miR-874-5p
C17orf85	hsa-miR-4326
C17orf85	hsa-miR-107-5p
C17orf85	hsa-miR-7042-5p
C17orf85	hsa-miR-758-3p
C17orf85	hsa-miR-4682
C17orf85	hsa-miR-101a-3p.2
C17orf85	hsa-miR-525-5p
C17orf85	hsa-miR-4792
C17orf85	hsa-miR-3089-3p
C17orf85	hsa-miR-8850
C17orf85	hsa-miR-3069-5p
C17orf85	hsa-miR-129-5p
C17orf85	hsa-miR-4793-5p
C17orf85	hsa-miR-7217-3p
C17orf85	hsa-miR-6077

C17orf85	hsa-miR-1188-5p
C17orf85	hsa-miR-151a-5p
C17orf85	hsa-miR-219a-5p
C17orf85	hsa-miR-6707-5p
C17orf85	hsa-miR-2471-5p
C17orf85	hsa-miR-4474-3p
C17orf85	hsa-miR-767-3p
C17orf85	hsa-miR-8082
C17orf85	hsa-miR-3145-3p
C17orf85	hsa-miR-1180-3p
C17orf85	hsa-miR-1815
C17orf85	hsa-miR-7009-5p
C17orf85	hsa-miR-206-5p
C17orf85	hsa-miR-3922-5p
C17orf85	hsa-miR-485-5p
C17orf85	hsa-miR-669b-5p
C17orf85	hsa-miR-6770-5p
C17orf85	hsa-miR-372-5p
C17orf85	hsa-miR-592-3p
C17orf85	hsa-miR-5709-3p
C17orf85	hsa-miR-455-5p
C17orf85	hsa-miR-6510-5p
C17orf85	hsa-miR-3692-5p
C17orf85	hsa-miR-511-5p
C17orf85	hsa-miR-4639-3p
C17orf85	hsa-miR-6865-5p
C17orf85	hsa-miR-495-3p
C17orf85	hsa-miR-125b-2-3p
C17orf85	hsa-miR-6759-5p
C17orf85	hsa-miR-6768-3p
C17orf85	hsa-miR-4760-3p
C17orf85	hsa-miR-3096-5p
C17orf85	hsa-miR-679
C17orf85	hsa-miR-1207-3p
C17orf85	hsa-miR-125-5p
C17orf85	hsa-miR-4687-5p
C17orf85	hsa-miR-6765-5p
C17orf85	hsa-miR-6385
C17orf85	hsa-miR-4656
C17orf85	hsa-miR-26
C17orf85	hsa-miR-99-3p
C17orf85	hsa-miR-6831-3p
C17orf85	hsa-miR-7071-5p
C17orf85	hsa-miR-6793-3p
C17orf85	hsa-miR-3145-5p
C17orf85	hsa-miR-4283

C17orf85	hsa-miR-3965
C17orf85	hsa-miR-7177-3p
C17orf85	hsa-miR-6133
C17orf85	hsa-miR-6825-3p
C17orf85	hsa-miR-2330-3p
C17orf85	hsa-miR-1237-3p
C17orf85	hsa-miR-3913-3p
C17orf85	hsa-miR-6829-3p
C17orf85	hsa-miR-575
C17orf85	hsa-miR-490-3p
C17orf85	hsa-miR-6554-5p
C17orf85	hsa-miR-4711-3p
C17orf85	hsa-miR-494
C17orf85	hsa-miR-7210-5p
C17orf85	hsa-miR-6071
C17orf85	hsa-miR-520f-5p
C17orf85	hsa-miR-548a-3p
C17orf85	hsa-miR-2392
C17orf85	hsa-miR-23b-5p
C17orf85	hsa-miR-631
C17orf85	hsa-miR-381-5p
C17orf85	hsa-miR-8807
C17orf85	hsa-miR-3913-5p
C17orf85	hsa-miR-660-5p
C17orf85	hsa-miR-3148
C17orf85	hsa-miR-4768-3p
C17orf85	hsa-miR-559
C17orf85	hsa-miR-7210-3p
C17orf85	hsa-miR-31-5p
C17orf85	hsa-miR-6908-5p
C17orf85	hsa-miR-7205-3p
C17orf85	hsa-miR-5003-3p
C17orf85	hsa-miR-548ay-3p
C17orf85	hsa-miR-471-3p
C17orf85	hsa-miR-3586-5p
C17orf85	hsa-miR-6789-3p
C17orf85	hsa-miR-6990-3p
C17orf85	hsa-miR-291
C17orf85	hsa-miR-488-5p
C17orf85	hsa-miR-548x-3p
C17orf85	hsa-miR-6843-3p
C17orf85	hsa-miR-6373
C17orf85	hsa-miR-6948-5p
C17orf85	hsa-miR-5131
C17orf85	hsa-miR-4471
C17orf85	hsa-miR-410

C17orf85	hsa-miR-880-5p
C17orf85	hsa-miR-6537-3p
C17orf85	hsa-miR-3151
C17orf85	hsa-miR-548ap-3p
C17orf85	hsa-miR-465a-5p
C17orf85	hsa-miR-4744
C17orf85	hsa-miR-3188
C17orf85	hsa-miR-6365
C17orf85	hsa-miR-8888
C17orf85	hsa-miR-673-5p
C17orf85	hsa-miR-2284ab
C17orf85	hsa-miR-881-3p
C17orf85	hsa-miR-154-5p
C17orf85	hsa-miR-6791-3p
C17orf85	hsa-miR-2284k
C17orf85	hsa-miR-8816
C17orf85	hsa-miR-7038-5p
C17orf85	hsa-miR-548ar-3p
C17orf85	hsa-miR-3187-5p
C17orf85	hsa-miR-7163-3p
C17orf85	hsa-miR-8886
C17orf85	hsa-miR-1256
C17orf85	hsa-miR-216c-5p
C17orf85	hsa-miR-4456
C17orf85	hsa-miR-222-5p
C17orf85	hsa-miR-1904
C17orf85	hsa-miR-5113
C17orf85	hsa-miR-766-5p
C17orf85	hsa-miR-8084
C17orf85	hsa-miR-601
C17orf85	hsa-miR-1234-3p
C17orf85	hsa-miR-1953
C17orf85	hsa-miR-489-3p
C17orf85	hsa-miR-3679-3p
C17orf85	hsa-miR-666-3p
C17orf85	hsa-miR-2399-3p
C17orf85	hsa-miR-505-5p
C17orf85	hsa-miR-122-5p
C17orf85	hsa-miR-887-5p
C17orf85	hsa-miR-5698
C17orf85	hsa-miR-93-5p
C17orf85	hsa-miR-409b
C17orf85	hsa-miR-548bd-3p
C17orf85	hsa-miR-6855-3p
C17orf85	hsa-let-7i-5p
C17orf85	hsa-miR-6529a

C17orf85	hsa-miR-486-3p
C17orf85	hsa-miR-29-3p
C17orf85	hsa-miR-7022-5p
C17orf85	hsa-miR-871-3p
C17orf85	hsa-miR-7021-3p
C17orf85	hsa-miR-410-3p
C17orf85	hsa-miR-4676-5p
C17orf85	hsa-miR-26-3p
C17orf85	hsa-miR-3063-5p
C17orf85	hsa-miR-344a-3p
C17orf85	hsa-miR-204-3p
C17orf85	hsa-miR-6721-5p
C17orf85	hsa-miR-873a-5p.1
C17orf85	hsa-miR-373-5p
C17orf85	hsa-miR-500-5p
C17orf85	hsa-miR-684
C17orf85	hsa-miR-205-5p
C17orf85	hsa-miR-7214-5p
C17orf85	hsa-miR-5697-3p
C17orf85	hsa-miR-1205
C17orf85	hsa-miR-6836-3p
C17orf85	hsa-miR-2486-3p
C17orf85	hsa-miR-4501
C17orf85	hsa-miR-452-3p
C17orf85	hsa-miR-1967
C17orf85	hsa-miR-124
C17orf85	hsa-miR-2285b
C17orf85	hsa-miR-1569
C17orf85	hsa-miR-8804
C17orf85	hsa-miR-7083-3p
C17orf85	hsa-miR-1301
C17orf85	hsa-miR-6377
C17orf85	hsa-miR-5620-3p
C17orf85	hsa-miR-7177-5p
C17orf85	hsa-miR-377-3p
C17orf85	hsa-miR-766-3p
C17orf85	hsa-miR-669k-5p
C17orf85	hsa-miR-2353
C17orf85	hsa-miR-942-5p
C17orf85	hsa-miR-378a-5p
C17orf85	hsa-miR-7212-3p
C17orf85	hsa-miR-3150b-3p
C17orf85	hsa-miR-2286
C17orf85	hsa-miR-4435
C17orf85	hsa-miR-487a-5p
C17orf85	hsa-miR-466

C17orf85	hsa-miR-7269-3p
C17orf85	hsa-miR-543-3p
C17orf85	hsa-miR-1185-5p
C17orf85	hsa-miR-3127-5p
C17orf85	hsa-miR-5004-3p
C17orf85	hsa-miR-4769-3p
C17orf85	hsa-miR-3975
C17orf85	hsa-miR-3120-3p
C17orf85	hsa-miR-344c-3p
C17orf85	hsa-miR-2458
C17orf85	hsa-miR-298
C17orf85	hsa-miR-6736-5p
C17orf85	hsa-miR-876
C17orf85	hsa-miR-146b-3p
C17orf85	hsa-miR-7a-2-3p
C17orf85	hsa-miR-3064-5p
C17orf85	hsa-miR-7660-5p
C17orf85	hsa-miR-499b-5p
C17orf85	hsa-miR-7060-3p
C17orf85	hsa-miR-34b-3p
C17orf85	hsa-miR-216a-5p
C17orf85	hsa-miR-137-3p
C17orf85	hsa-miR-1183
C17orf85	hsa-miR-504-3p
C17orf85	hsa-miR-2861
C17orf85	hsa-miR-3470a
C17orf85	hsa-miR-3141
C17orf85	hsa-miR-337-3p
C17orf85	hsa-miR-4308
C17orf85	hsa-miR-23a-3p
C17orf85	hsa-miR-873a-3p
C17orf85	hsa-miR-155-5p
C17orf85	hsa-miR-1538
C17orf85	hsa-miR-2439-5p
C17orf85	hsa-miR-1193-3p
C17orf85	hsa-miR-449-5p
C17orf85	hsa-miR-943
C17orf85	hsa-miR-18-3p
C17orf85	hsa-miR-7110-3p
C17orf85	hsa-miR-668-3p
C17orf85	hsa-miR-544-5p
C17orf85	hsa-miR-7094-1-5p
C17orf85	hsa-miR-4266
C17orf85	hsa-miR-30a-3p
C17orf85	hsa-miR-6827-3p
C17orf85	hsa-miR-642

C17orf85	hsa-miR-4752
C17orf85	hsa-miR-582-5p
C17orf85	hsa-miR-3689e
C17orf85	hsa-miR-290b-5p
C17orf85	hsa-miR-570-3p
C17orf85	hsa-miR-18b-3p
C17orf85	hsa-miR-7081-3p
C17orf85	hsa-miR-6728-3p
C17orf85	hsa-miR-449a
C17orf85	hsa-miR-2450c
C17orf85	hsa-miR-6877-5p
C17orf85	hsa-miR-6797-3p
C17orf85	hsa-miR-376c-5p
C17orf85	hsa-miR-196-2-3p
C17orf85	hsa-miR-1296-5p
C17orf85	hsa-miR-6781-3p
C17orf85	hsa-miR-1786
C17orf85	hsa-miR-3944-3p
C17orf85	hsa-miR-2681-3p
C17orf85	hsa-miR-3938-3p
C17orf85	hsa-miR-574-5p
C17orf85	hsa-miR-1343-5p
C17orf85	hsa-miR-3174
C17orf85	hsa-miR-4797-3p
C17orf85	hsa-miR-1934-3p
C17orf85	hsa-miR-767
C17orf85	hsa-miR-6899-5p
C17orf85	hsa-miR-130-3p
C17orf85	hsa-miR-5003-5p
C17orf85	hsa-miR-4660
C17orf85	hsa-miR-6511-5p
C17orf85	hsa-miR-6794-3p
C17orf85	hsa-miR-3102
C17orf85	hsa-miR-4511
C17orf85	hsa-miR-7386i-5p
C17orf85	hsa-miR-3065-5p
C17orf85	hsa-miR-423-3p
C17orf85	hsa-miR-5690
C17orf85	hsa-miR-34-5p
C17orf85	hsa-miR-2306
C17orf85	hsa-miR-3616-3p
C17orf85	hsa-miR-7094-3p
C17orf85	hsa-miR-6987-5p
C17orf85	hsa-miR-7227-5p
C17orf85	hsa-miR-2284
C17orf85	hsa-miR-3074-5p

C17orf85	hsa-miR-6826-3p
C17orf85	hsa-miR-216c-3p
C17orf85	hsa-miR-539-3p
C17orf85	hsa-miR-619-3p
C17orf85	hsa-miR-6914-3p
C17orf85	hsa-miR-9-5p
C17orf85	hsa-miR-3659
C17orf85	hsa-miR-1255
C17orf85	hsa-miR-1738
C17orf85	hsa-miR-452-5p
C17orf85	hsa-miR-346
C17orf85	hsa-miR-2407
C17orf85	hsa-miR-92b-5p
C17orf85	hsa-miR-4777-5p
C17orf85	hsa-miR-1912-3p
C17orf85	hsa-miR-200a-5p
C17orf85	hsa-miR-548am-5p
C17orf85	hsa-miR-196a-3p
C17orf85	hsa-miR-195-5p
C17orf85	hsa-miR-344-5p
C17orf85	hsa-miR-363-3p
C17orf85	hsa-miR-496b
C17orf85	hsa-miR-374
C17orf85	hsa-miR-5619-5p
C17orf85	hsa-miR-302bd
C17orf85	hsa-miR-8908c
C17orf85	hsa-miR-6735-3p
C17orf85	hsa-miR-7463-3p
C17orf85	hsa-miR-222-3p
C17orf85	hsa-miR-3061-3p
C17orf85	hsa-miR-6507-5p
C17orf85	hsa-miR-20b-3p
C17orf85	hsa-miR-519d-5p
C17orf85	hsa-miR-96-5p
C17orf85	hsa-miR-8887
C17orf85	hsa-miR-548k
C17orf85	hsa-miR-6891-3p
C17orf85	hsa-miR-576-5p
C17orf85	hsa-miR-7212-5p
C17orf85	hsa-miR-3102-3p.2-3p
C17orf85	hsa-miR-454
C17orf85	hsa-miR-548e-3p
C17orf85	hsa-miR-96-5p
C17orf85	hsa-miR-494-5p
C17orf85	hsa-miR-1190
C17orf85	hsa-miR-1262

C17orf85	hsa-miR-6861-5p
C17orf85	hsa-miR-106b-5p
C17orf85	hsa-miR-516
C17orf85	hsa-miR-548c-3p
C17orf85	hsa-miR-2376
C17orf85	hsa-miR-1943-5p
C17orf85	hsa-miR-6780a-5p
C17orf85	hsa-miR-8077
C17orf85	hsa-miR-370-5p
C17orf85	hsa-miR-6769b-3p
C17orf85	hsa-miR-3473f
C17orf85	hsa-miR-489-5p
C17orf85	hsa-miR-7154-5p
C17orf85	hsa-miR-6916-5p
C17orf85	hsa-miR-512
C17orf85	hsa-miR-4651
C17orf85	hsa-miR-7093-3p
C17orf85	hsa-miR-8860
C17orf85	hsa-miR-5627-5p
C17orf85	hsa-miR-31-5p
C17orf85	hsa-miR-3059-3p
C17orf85	hsa-miR-6330
C17orf85	hsa-miR-2290
C17orf85	hsa-miR-518-3p
C17orf85	hsa-miR-493-3p
C17orf85	hsa-miR-6984-5p
C17orf85	hsa-miR-8854
C17orf85	hsa-miR-6867-5p
C17orf85	hsa-miR-4719
C17orf85	hsa-miR-140-3p
C17orf85	hsa-miR-154b
C17orf85	hsa-miR-639
C17orf85	hsa-miR-7659-5p
C17orf85	hsa-miR-6715b-5p
C17orf85	hsa-miR-4298
C17orf85	hsa-miR-3938
C17orf85	hsa-miR-183-5p
C17orf85	hsa-miR-2340
C17orf85	hsa-miR-6792-3p
C17orf85	hsa-miR-644a
C17orf85	hsa-miR-7193-5p
C17orf85	hsa-miR-187-5p
C17orf85	hsa-miR-376ab-3p
C17orf85	hsa-miR-133-3p
C17orf85	hsa-miR-328-5p
C17orf85	hsa-miR-296-5p

C17orf85	hsa-miR-1180-5p
C17orf85	hsa-miR-664a-5p
C17orf85	hsa-miR-7031-5p
C17orf85	hsa-miR-3473be
C17orf85	hsa-miR-6129
C17orf85	hsa-miR-4794
C17orf85	hsa-let-7-5p
C17orf85	hsa-miR-516b
C17orf85	hsa-miR-4490
C17orf85	hsa-miR-2314
C17orf85	hsa-miR-922
C17orf85	hsa-miR-548ad-5p
C17orf85	hsa-miR-1626-5p
C17orf85	hsa-miR-149-3p
C17orf85	hsa-miR-3927-3p
C17orf85	hsa-miR-2284n
C17orf85	hsa-miR-659-5p
C17orf85	hsa-miR-7048-5p
C17orf85	hsa-miR-30-3p
C17orf85	hsa-miR-2284x
C17orf85	hsa-miR-455-3p.1
C17orf85	hsa-miR-181b-1-3p
C17orf85	hsa-miR-5114
C17orf85	hsa-miR-1-3p
C17orf85	hsa-miR-9769-3p
C17orf85	hsa-miR-124c-5p
C17orf85	hsa-miR-505-3p.2
C17orf85	hsa-miR-7864
C17orf85	hsa-miR-4786-5p
C17orf85	hsa-miR-34b-3p
C17orf85	hsa-miR-7703
C17orf85	hsa-miR-589-3p
C17orf85	hsa-miR-339-5p
C17orf85	hsa-miR-7321-3p
C17orf85	hsa-miR-7199-3p
C17orf85	hsa-miR-607
C17orf85	hsa-miR-200-3p
C17orf85	hsa-miR-3689a-5p
C17orf85	hsa-miR-4773
C17orf85	hsa-miR-7688-3p
C17orf85	hsa-miR-4643
C17orf85	hsa-miR-466b-3p
C17orf85	hsa-miR-1253
C17orf85	hsa-miR-16-5p
C17orf85	hsa-miR-342-5p
C17orf85	hsa-miR-665-3p

C17orf85	hsa-miR-516b-3p
C17orf85	hsa-miR-6346
C17orf85	hsa-miR-921
C17orf85	hsa-miR-378c
C17orf85	hsa-miR-3089-5p
C17orf85	hsa-miR-4680-3p
C17orf85	hsa-miR-669j
C17orf85	hsa-miR-759
C17orf85	hsa-miR-206
C17orf85	hsa-miR-3058-3p
C17orf85	hsa-miR-4532
C17orf85	hsa-miR-548c-5p
C17orf85	hsa-miR-3919
C17orf85	hsa-miR-1240
C17orf85	hsa-miR-224-5p
C17orf85	hsa-miR-7026-5p
C17orf85	hsa-let-7b-3p
C17orf85	hsa-miR-3962
C17orf85	hsa-miR-6740-3p
C17orf85	hsa-miR-107
C17orf85	hsa-miR-7038-3p
C17orf85	hsa-miR-7054-5p
C17orf85	hsa-miR-466g
C17orf85	hsa-miR-1701
C17orf85	hsa-miR-3191-5p
C17orf85	hsa-miR-20-3p
C17orf85	hsa-miR-2284-5p
C17orf85	hsa-miR-196-5p
C17orf85	hsa-miR-5701
C17orf85	hsa-miR-433
C17orf85	hsa-miR-190a-3p
C17orf85	hsa-miR-203-5p
C17orf85	hsa-miR-934-5p
C17orf85	hsa-miR-302c-3p
C17orf85	hsa-miR-3125
C17orf85	hsa-miR-3684
C17orf85	hsa-miR-3929
C17orf85	hsa-miR-4659b-3p
C17orf85	hsa-miR-606
C17orf85	hsa-miR-1266
C17orf85	hsa-miR-138-5p
C17orf85	hsa-miR-6994-3p
C17orf85	hsa-miR-302b-5p
C17orf85	hsa-miR-4739
C17orf85	hsa-miR-2430
C17orf85	hsa-miR-2285u

C17orf85	hsa-miR-4330
C17orf85	hsa-miR-380
C17orf85	hsa-miR-365-5p
C17orf85	hsa-miR-2431-5p
C17orf85	hsa-miR-687
C17orf85	hsa-miR-423-5p
C17orf85	hsa-miR-98-3p
C17orf85	hsa-miR-2188-3p
C17orf85	hsa-miR-6355
C17orf85	hsa-miR-8864
C17orf85	hsa-miR-2294
C17orf85	hsa-miR-6612-5p
C17orf85	hsa-miR-302c-5p
C17orf85	hsa-miR-8813
C17orf85	hsa-miR-1191b-5p
C17orf85	hsa-miR-7069-3p
C17orf85	hsa-miR-8054
C17orf85	hsa-miR-302f
C17orf85	hsa-miR-2411-3p
C17orf85	hsa-miR-6081
C17orf85	hsa-miR-491-3p
C17orf85	hsa-miR-6734-3p
C17orf85	hsa-miR-5703
C17orf85	hsa-miR-1909-5p
C17orf85	hsa-miR-520
C17orf85	hsa-miR-4704-3p
C17orf85	hsa-miR-1254
C17orf85	hsa-miR-648
C17orf85	hsa-miR-92a-2-5p
C17orf85	hsa-miR-5108
C17orf85	hsa-miR-5627-3p
C17orf85	hsa-miR-8116
C17orf85	hsa-miR-532-5p
C17orf85	hsa-miR-6901-3p
C17orf85	hsa-miR-4673
C17orf85	hsa-miR-3058-5p
C17orf85	hsa-miR-1231
C17orf85	hsa-miR-2475
C17orf85	hsa-miR-1596-5p
C17orf85	hsa-miR-1276
C17orf85	hsa-miR-514b-5p
C17orf85	hsa-miR-371b-5p
C17orf85	hsa-miR-2443
C17orf85	hsa-miR-7686-5p
C17orf85	hsa-miR-129-5p
C17orf85	hsa-miR-1224-5p

C17orf85	hsa-miR-5120
C17orf85	hsa-miR-2897
C17orf85	hsa-miR-4438
C17orf85	hsa-miR-204-5p
C17orf85	hsa-miR-3607-5p
C17orf85	hsa-miR-670
C17orf85	hsa-miR-509-5p
C17orf85	hsa-miR-624-3p
C17orf85	hsa-miR-4449
C17orf85	hsa-miR-6982-3p
C17orf85	hsa-miR-1298-3p
C17orf85	hsa-miR-2438
C17orf85	hsa-miR-3664-3p
C17orf85	hsa-miR-7663-5p
C17orf85	hsa-miR-7321-5p
C17orf85	hsa-miR-6845-5p
C17orf85	hsa-miR-548ae-3p
C17orf85	hsa-miR-2466-3p
C17orf85	hsa-miR-432-3p
C17orf85	hsa-miR-30c-2-3p
C17orf85	hsa-miR-1262-3p
C17orf85	hsa-miR-34
C17orf85	hsa-miR-8823
C17orf85	hsa-miR-4667-5p
C17orf85	hsa-miR-468-3p
C17orf85	hsa-miR-3928-5p
C17orf85	hsa-miR-4282
C17orf85	hsa-miR-3122
C17orf85	hsa-let-7a-5p
C17orf85	hsa-miR-3622a-3p
C17orf85	hsa-miR-605
C17orf85	hsa-miR-146-5p
C17orf85	hsa-miR-8485
C17orf85	hsa-miR-1892
C17orf85	hsa-miR-7007-5p
C17orf85	hsa-miR-214-3p
C17orf85	hsa-miR-344d-2-5p
C17orf85	hsa-miR-7024-3p
C17orf85	hsa-miR-101b-3p.1
C17orf85	hsa-miR-6796-5p
C17orf85	hsa-miR-6844
C17orf85	hsa-miR-653
C17orf85	hsa-miR-411-5p
C17orf85	hsa-miR-3099-5p
C17orf85	hsa-miR-370
C17orf85	hsa-miR-7665-3p

C17orf85	hsa-miR-4745-3p
C17orf85	hsa-miR-4477a
C17orf85	hsa-miR-1229-3p
C17orf85	hsa-miR-6739-3p
C17orf85	hsa-miR-8908b
C17orf85	hsa-miR-216b
C17orf85	hsa-miR-7681-3p
C17orf85	hsa-miR-7678-5p
C17orf85	hsa-miR-7646-5p
C17orf85	hsa-miR-4419b
C17orf85	hsa-miR-942-3p
C17orf85	hsa-miR-1896
C17orf85	hsa-miR-5591-3p
C17orf85	hsa-miR-548ak
C17orf85	hsa-miR-1207-5p
C17orf85	hsa-miR-3551-3p
C17orf85	hsa-miR-381-3p
C17orf85	hsa-miR-7666-5p
C17orf85	hsa-miR-3073a-5p
C17orf85	hsa-miR-4753-5p
C17orf85	hsa-miR-6781-5p
C17orf85	hsa-miR-1193
C17orf85	hsa-miR-3690
C17orf85	hsa-miR-6849-3p
C17orf85	hsa-miR-383
C17orf85	hsa-miR-8873a
C17orf85	hsa-miR-6880-5p
C17orf85	hsa-miR-19a-5p
C17orf85	hsa-miR-3571
C17orf85	hsa-miR-548ar-5p
C17orf85	hsa-miR-299-5p
C17orf85	hsa-miR-1202
C17orf85	hsa-miR-6134
C17orf85	hsa-miR-7169-5p
C17orf85	hsa-miR-3600
C17orf85	hsa-miR-5583-3p
C17orf85	hsa-miR-3079-3p
C17orf85	hsa-miR-320a
C17orf85	hsa-miR-4692
C17orf85	hsa-miR-3103-5p
C17orf85	hsa-miR-3140-5p
C17orf85	hsa-miR-504-5p
C17orf85	hsa-miR-4529-3p
C17orf85	hsa-miR-4793-3p
C17orf85	hsa-miR-7467-3p
C17orf85	hsa-miR-6568-3p

C17orf85	hsa-miR-125-3p
C17orf85	hsa-miR-4714-5p
C17orf85	hsa-miR-1247-3p
C17orf85	hsa-miR-1777
C17orf85	hsa-miR-1250-5p
C17orf85	hsa-miR-7006-3p
C17orf85	hsa-miR-7975
C17orf85	hsa-miR-4664-5p
C17orf85	hsa-miR-211-5p
C17orf85	hsa-miR-7227-3p
C17orf85	hsa-miR-466l-3p
C17orf85	hsa-miR-1264
C17orf85	hsa-miR-891a-3p
C17orf85	hsa-let-7afk-3p
C17orf85	hsa-miR-143-3p
C17orf85	hsa-miR-7025-3p
C17orf85	hsa-miR-1941-5p
C17orf85	hsa-let-7c-5p
C17orf85	hsa-miR-6760-5p
C17orf85	hsa-miR-6735-5p
C17orf85	hsa-miR-1537-3p
C17orf85	hsa-miR-5096
C17orf85	hsa-miR-103-5p
C17orf85	hsa-miR-7012-5p
C17orf85	hsa-miR-185-5p
C17orf85	hsa-miR-24-5p
C17orf85	hsa-miR-1785
C17orf85	hsa-miR-4756-3p
C17orf85	hsa-miR-3619-3p
C17orf85	hsa-miR-6992-5p
C17orf85	hsa-let-7-3p
C17orf85	hsa-miR-677-5p
C17orf85	hsa-miR-520-5p
C17orf85	hsa-miR-7017-5p
C17orf85	hsa-miR-8071
C17orf85	hsa-miR-1251-5p
C17orf85	hsa-miR-595
C17orf85	hsa-miR-6879-5p
C17orf85	hsa-miR-6131
C17orf85	hsa-miR-544a
C17orf85	hsa-miR-7205-5p
C17orf85	hsa-miR-1285-3p
C17orf85	hsa-miR-3660
C17orf85	hsa-miR-591
C17orf85	hsa-miR-524-3p
C17orf85	hsa-miR-3193

C17orf85	hsa-miR-378-5p
C17orf85	hsa-miR-5002-3p
C17orf85	hsa-miR-197-5p
C17orf85	hsa-miR-4534
C17orf85	hsa-miR-182-5p
C17orf85	hsa-miR-2896
C17orf85	hsa-miR-5008-3p
C17orf85	hsa-miR-7035-5p
C17orf85	hsa-miR-6130
C17orf85	hsa-miR-624-5p
C17orf85	hsa-miR-550a-3p
C17orf85	hsa-miR-3957
C17orf85	hsa-miR-422
C17orf85	hsa-miR-7064-5p
C17orf85	hsa-miR-744-5p
C17orf85	hsa-miR-2285k
C17orf85	hsa-miR-4447
C17orf85	hsa-miR-669i
C17orf85	hsa-miR-8898
C17orf85	hsa-miR-7857
C17orf85	hsa-miR-2284u
C17orf85	hsa-miR-7087-3p
C17orf85	hsa-miR-5132-5p
C17orf85	hsa-miR-599-5p
C17orf85	hsa-miR-4680-5p
C17orf85	hsa-miR-551-5p
C17orf85	hsa-miR-16-5p
C17orf85	hsa-miR-938
C17orf85	hsa-miR-6779-5p
C17orf85	hsa-miR-6399
C17orf85	hsa-miR-5589-3p
C17orf85	hsa-miR-3085-3p
C17orf85	hsa-miR-2446
C17orf85	hsa-miR-5090
C17orf85	hsa-miR-3649
C17orf85	hsa-miR-4746-5p
C17orf85	hsa-miR-2052
C17orf85	hsa-miR-644a
C17orf85	hsa-miR-564
C17orf85	hsa-miR-1941-3p
C17orf85	hsa-miR-8803
C17orf85	hsa-miR-4462
C17orf85	hsa-miR-542-3p
C17orf85	hsa-miR-26b-3p
C17orf85	hsa-miR-624-3p
C17orf85	hsa-miR-8819

C17orf85	hsa-miR-6729-3p
C17orf85	hsa-miR-1539
C17orf85	hsa-miR-1266-5p
C17orf85	hsa-miR-1812-5p
C17orf85	hsa-miR-548au-5p
C17orf85	hsa-miR-741-3p
C17orf85	hsa-miR-7019-5p
C17orf85	hsa-miR-1687-3p
C17orf85	hsa-miR-3667-5p
C17orf85	hsa-miR-3611
C17orf85	hsa-miR-2379
C17orf85	hsa-miR-6916-3p
C17orf85	hsa-miR-200c-5p
C17orf85	hsa-miR-8055
C17orf85	hsa-miR-548ac
C17orf85	hsa-miR-6124
C17orf85	hsa-miR-6754-3p
C17orf85	hsa-miR-8893
C17orf85	hsa-miR-211-5p
C17orf85	hsa-miR-6881-3p
C17orf85	hsa-miR-578
C17orf85	hsa-miR-548aj-5p
C17orf85	hsa-miR-514a-5p
C17orf85	hsa-miR-215-3p
C17orf85	hsa-miR-2985
C17orf85	hsa-miR-27b-5p
C17orf85	hsa-miR-3652
C17orf85	hsa-miR-3431
C17orf85	hsa-miR-7162-3p
C17orf85	hsa-miR-6965-3p
C17orf85	hsa-miR-608
C17orf85	hsa-miR-134-5p
C17orf85	hsa-miR-3572
C17orf85	hsa-miR-6815-5p
C17orf85	hsa-miR-2114-3p
C17orf85	hsa-miR-2448-3p
C17orf85	hsa-miR-4252
C17orf85	hsa-miR-500-3p
C17orf85	hsa-miR-4509
C17orf85	hsa-miR-6878-5p
C17orf85	hsa-miR-2422
C17orf85	hsa-miR-6321
C17orf85	hsa-miR-6335
C17orf85	hsa-miR-3605-5p
C17orf85	hsa-miR-5101
C17orf85	hsa-miR-1273g-5p

C17orf85	hsa-miR-6710-3p
C17orf85	hsa-miR-6949-5p
C17orf85	hsa-miR-548i
C17orf85	hsa-miR-7116-5p
C17orf85	hsa-miR-216a-3p
C17orf85	hsa-miR-7702
C17orf85	hsa-miR-4328
C17orf85	hsa-miR-7862
C17orf85	hsa-miR-3607-3p
C17orf85	hsa-miR-23b-3p
C17orf85	hsa-miR-451b
C17orf85	hsa-miR-7016-3p
C17orf85	hsa-miR-219b-5p
C17orf85	hsa-miR-7354-3p
C17orf85	hsa-miR-5582-5p
C17orf85	hsa-miR-32-5p
C17orf85	hsa-miR-30--3p
C17orf85	hsa-miR-3474
C17orf85	hsa-miR-2331-3p
C17orf85	hsa-miR-6541
C17orf85	hsa-miR-212-5p
C17orf85	hsa-miR-6120-5p
C17orf85	hsa-miR-6332
C17orf85	hsa-miR-497-3p
C17orf85	hsa-miR-3130-3p
C17orf85	hsa-miR-205-5p
C17orf85	hsa-miR-3617-5p
C17orf85	hsa-miR-4681
C17orf85	hsa-miR-2285r
C17orf85	hsa-miR-6745
C17orf85	hsa-miR-2401
C17orf85	hsa-miR-132-3p
C17orf85	hsa-miR-548ax
C17orf85	hsa-miR-679-3p
C17orf85	hsa-miR-1746
C17orf85	hsa-miR-6737-3p
C17orf85	hsa-miR-4269
C17orf85	hsa-miR-6940-3p
C17orf85	hsa-miR-1249-5p
C17orf85	hsa-miR-3556b
C17orf85	hsa-miR-7268-3p
C17orf85	hsa-miR-196c-3p
C17orf85	hsa-miR-7083-5p
C17orf85	hsa-miR-3099-3p
C17orf85	hsa-miR-877-5p
C17orf85	hsa-miR-2388-3p

C17orf85	hsa-miR-3614-5p
C17orf85	hsa-miR-6750-5p
C17orf85	hsa-miR-548
C17orf85	hsa-miR-6418-5p
C17orf85	hsa-miR-3087-5p
C17orf85	hsa-miR-6803-3p
C17orf85	hsa-miR-8899
C17orf85	hsa-miR-6677-5p
C17orf85	hsa-miR-628-5p
C17orf85	hsa-miR-7184-5p
C17orf85	hsa-miR-488-3p
C17orf85	hsa-miR-5125
C17orf85	hsa-miR-185-5p
C17orf85	hsa-miR-5580-5p
C17orf85	hsa-miR-6515-5p
C17orf85	hsa-miR-8061
C17orf85	hsa-miR-6943-3p
C17orf85	hsa-miR-301-5p
C17orf85	hsa-miR-193-3p
C17orf85	hsa-miR-29-5p
C17orf85	hsa-miR-6086
C17orf85	hsa-miR-6942-5p
C17orf85	hsa-miR-6734-5p
C17orf85	hsa-miR-6857-3p
C17orf85	hsa-miR-181-5p
C17orf85	hsa-miR-544-3p
C17orf85	hsa-miR-1827
C17orf85	hsa-miR-8793
C17orf85	hsa-miR-1465
C17orf85	hsa-miR-7687-5p
C17orf85	hsa-miR-512-3p
C17orf85	hsa-miR-2311
C17orf85	hsa-miR-4999-5p
C17orf85	hsa-miR-4716-3p
C17orf85	hsa-miR-6896-5p
C17orf85	hsa-miR-1584-5p
C17orf85	hsa-miR-378d
C17orf85	hsa-miR-2284a
C17orf85	hsa-miR-548aj-3p
C17orf85	hsa-miR-2410
C17orf85	hsa-miR-487b-5p
C17orf85	hsa-miR-491-5p
C17orf85	hsa-miR-338-3p
C17orf85	hsa-miR-1236-3p
C17orf85	hsa-miR-3194-5p
C17orf85	hsa-miR-6569-5p

C17orf85	hsa-miR-6962-3p
C17orf85	hsa-miR-6914-5p
C17orf85	hsa-miR-6529b
C17orf85	hsa-miR-217
C17orf85	hsa-miR-522-3p
C17orf85	hsa-miR-6973b-3p
C17orf85	hsa-let-7b-5p
C17orf85	hsa-miR-6401
C17orf85	hsa-miR-6819-5p
C17orf85	hsa-miR-1271-5p
C17orf85	hsa-miR-7240-5p
C17orf85	hsa-miR-1560-3p
C17orf85	hsa-miR-598-5p
C17orf85	hsa-miR-6902-3p
C17orf85	hsa-miR-489
C17orf85	hsa-miR-5615-5p
C17orf85	hsa-miR-6870-3p
C17orf85	hsa-miR-4658
C17orf85	hsa-miR-219a-1-3p
C17orf85	hsa-miR-212-3p
C17orf85	hsa-miR-101-3p.2
C17orf85	hsa-miR-3202
C17orf85	hsa-miR-199b-5p
C17orf85	hsa-miR-200a-3p
C17orf85	hsa-miR-7-5p
C17orf85	hsa-miR-4699-3p
C17orf85	hsa-miR-6741-5p
C17orf85	hsa-miR-196-1-3p
C17orf85	hsa-miR-330-3p.2
C17orf85	hsa-miR-2393
C17orf85	hsa-miR-8062
C17orf85	hsa-miR-3620-3p
C17orf85	hsa-miR-188-3p
C17orf85	hsa-miR-1248
C17orf85	hsa-miR-199-5p
C17orf85	hsa-miR-678
C17orf85	hsa-miR-2276-3p
C17orf85	hsa-miR-2381
C17orf85	hsa-miR-3918
C17orf85	hsa-miR-9768-3p
C17orf85	hsa-miR-30
C17orf85	hsa-miR-518d-5p
C17orf85	hsa-miR-7036a-3p
C17orf85	hsa-miR-4277
C17orf85	hsa-miR-6885-5p
C17orf85	hsa-miR-1906

C17orf85	hsa-miR-6780b-5p
C17orf85	hsa-miR-7029-3p
C17orf85	hsa-miR-597
C17orf85	hsa-miR-130b-5p
C17orf85	hsa-miR-584-3p
C17orf85	hsa-miR-7211-5p
C17orf85	hsa-miR-5124a
C17orf85	hsa-miR-2285x
C17orf85	hsa-miR-1343-3p
C17orf85	hsa-miR-424-5p
C17orf85	hsa-miR-2310
C17orf85	hsa-miR-7017-3p
C17orf85	hsa-miR-499b-3p
C17orf85	hsa-miR-5683
C17orf85	hsa-miR-136-5p
C17orf85	hsa-miR-3596c
C17orf85	hsa-miR-1255b-2-3p
C17orf85	hsa-miR-7373a-3p
C17orf85	hsa-miR-3194-3p
C17orf85	hsa-miR-377-5p
C17orf85	hsa-miR-3972
C17orf85	hsa-miR-4514
C17orf85	hsa-miR-466mo-3p
C17orf85	hsa-miR-3682-5p
C17orf85	hsa-miR-10-5p
C17orf85	hsa-miR-6794-5p
C17orf85	hsa-miR-532-5p
C17orf85	hsa-miR-465c-5p
C17orf85	hsa-miR-3613b
C17orf85	hsa-miR-6873-5p
C17orf85	hsa-miR-15a-5p
C17orf85	hsa-miR-3123
C17orf85	hsa-miR-877-5p
C17orf85	hsa-miR-7977
C17orf85	hsa-miR-29b-5p
C17orf85	hsa-miR-544a
C17orf85	hsa-miR-425-3p
C17orf85	hsa-miR-7154-3p
C17orf85	hsa-miR-7660-3p
C17orf85	hsa-miR-4685-5p
C17orf85	hsa-miR-3662
C17orf85	hsa-miR-623
C17orf85	hsa-miR-6357
C17orf85	hsa-miR-2406
C17orf85	hsa-miR-1293
C17orf85	hsa-miR-6854-5p

C17orf85	hsa-miR-7157-3p
C17orf85	hsa-miR-190-3p
C17orf85	hsa-miR-487-5p
C17orf85	hsa-miR-147-5p
C17orf85	hsa-miR-4528
C17orf85	hsa-miR-3153
C17orf85	hsa-miR-2334
C17orf85	hsa-miR-4724-5p
C17orf85	hsa-miR-7667-5p
C17orf85	hsa-miR-2325
C17orf85	hsa-miR-6817-5p
C17orf85	hsa-miR-6814-5p
C17orf85	hsa-miR-5699-3p
C17orf85	hsa-miR-4781-3p
C17orf85	hsa-miR-1969
C17orf85	hsa-miR-7081-5p
C17orf85	hsa-miR-622
C17orf85	hsa-miR-3473g
C17orf85	hsa-miR-540-3p
C17orf85	hsa-miR-375
C17orf85	hsa-miR-3121-3p
C17orf85	hsa-miR-7171-5p
C17orf85	hsa-let-7
C17orf85	hsa-miR-7178-3p
C17orf85	hsa-miR-3613-3p
C17orf85	hsa-miR-1197-3p
C17orf85	hsa-miR-7181-3p
C17orf85	hsa-miR-3140-3p
C17orf85	hsa-miR-8890
C17orf85	hsa-miR-373-3p
C17orf85	hsa-miR-542
C17orf85	hsa-miR-7175-5p
C17orf85	hsa-miR-215-5p
C17orf85	hsa-miR-543
C17orf85	hsa-miR-758-5p
C17orf85	hsa-miR-203
C17orf85	hsa-miR-7850-5p
C17orf85	hsa-miR-19b-5p
C17orf85	hsa-miR-548b-3p
C17orf85	hsa-miR-7044-3p
C17orf85	hsa-miR-1243
C17orf85	hsa-miR-6973a-3p
C17orf85	hsa-miR-208-3p
C17orf85	hsa-miR-130
C17orf85	hsa-miR-1954
C17orf85	hsa-miR-2313-5p

C17orf85	hsa-miR-466b-4-3p
C17orf85	hsa-miR-5692a
C17orf85	hsa-miR-369-3p
C17orf85	hsa-miR-4329
C17orf85	hsa-miR-7037-3p
C17orf85	hsa-miR-7175-3p
C17orf85	hsa-miR-145a-3p
C17orf85	hsa-miR-15b-5p
C17orf85	hsa-miR-338-3p
C17orf85	hsa-miR-510-5p
C17orf85	hsa-miR-3594-5p
C17orf85	hsa-miR-708-5p
C17orf85	hsa-miR-1288
C17orf85	hsa-miR-2895
C17orf85	hsa-miR-194-5p
C17orf85	hsa-miR-202-3p
C17orf85	hsa-miR-8815
C17orf85	hsa-miR-5006-3p
C17orf85	hsa-miR-3612
C17orf85	hsa-miR-2284g
C17orf85	hsa-miR-7450-5p
C17orf85	hsa-miR-26-5p
C17orf85	hsa-miR-3544-3p
C17orf85	hsa-miR-1696
C17orf85	hsa-miR-3144-5p
C17orf85	hsa-miR-8101
C17orf85	hsa-miR-3162-5p
C17orf85	hsa-miR-511-3p
C17orf85	hsa-miR-3548
C17orf85	hsa-miR-625-5p
C17orf85	hsa-miR-4763-5p
C17orf85	hsa-miR-6998-3p
C17orf85	hsa-miR-4691-5p
C17orf85	hsa-miR-1273g-3p
C17orf85	hsa-miR-3160-3p
C17orf85	hsa-miR-4264
C17orf85	hsa-miR-3100-5p
C17orf85	hsa-miR-4722-5p
C17orf85	hsa-miR-6872-3p
C17orf85	hsa-miR-7843-3p
C17orf85	hsa-let-7g-5p
C17orf85	hsa-miR-4510
C17orf85	hsa-miR-207
C17orf85	hsa-miR-551
C17orf85	hsa-miR-17-3p
C17orf85	hsa-miR-4705

C17orf85	hsa-miR-1388-3p
C17orf85	hsa-miR-2300-3p
C17orf85	hsa-miR-7280-5p
C17orf85	hsa-miR-6939-5p
C17orf85	hsa-miR-466b-2-3p
C17orf85	hsa-miR-691
C17orf85	hsa-miR-27-3p
C17orf85	hsa-miR-182-5p
C17orf85	hsa-miR-8904b
C17orf85	hsa-miR-4491
C17orf85	hsa-miR-5196-5p
C17orf85	hsa-miR-872
C17orf85	hsa-miR-6938-3p
C17orf85	hsa-miR-5004-5p
C17orf85	hsa-miR-1247-5p
C17orf85	hsa-miR-450a-1-3p
C17orf85	hsa-miR-93-3p
C17orf85	hsa-miR-424-5p
C17orf85	hsa-miR-204-5p
C17orf85	hsa-miR-6822-5p
C17orf85	hsa-miR-693-5p
C17orf85	hsa-miR-1224
C17orf85	hsa-miR-674-5p
C17orf85	hsa-miR-7193-3p
C17orf85	hsa-miR-876-5p
C17orf85	hsa-miR-4255
C17orf85	hsa-miR-6529-3p
C17orf85	hsa-miR-5088-5p
C17orf85	hsa-miR-127-5p
C17orf85	hsa-miR-142b
C17orf85	hsa-miR-519-5p
C17orf85	hsa-miR-5110
C17orf85	hsa-miR-1281
C17orf85	hsa-miR-6983-5p
C17orf85	hsa-miR-4742-5p
C17orf85	hsa-miR-3577
C17orf85	hsa-miR-683
C17orf85	hsa-miR-7077-3p
C17orf85	hsa-miR-1178-3p
C17orf85	hsa-miR-3966
C17orf85	hsa-miR-378-3p
C17orf85	hsa-miR-149-5p
C17orf85	hsa-miR-4776-5p
C17orf85	hsa-miR-330-3p.1
C17orf85	hsa-miR-3475-5p
C17orf85	hsa-miR-5787

C17orf85	hsa-miR-6771-5p
C17orf85	hsa-miR-8832
C17orf85	hsa-miR-548b-5p
C17orf85	hsa-miR-4716
C17orf85	hsa-miR-7705
C17orf85	hsa-miR-1255b-5p
C17orf85	hsa-miR-513c-3p
C17orf85	hsa-miR-143-3p
C17orf85	hsa-miR-502a
C17orf85	hsa-miR-7015-5p
C17orf85	hsa-miR-431-5p
C17orf85	hsa-miR-1708
C17orf85	hsa-miR-6836-5p
C17orf85	hsa-miR-6795-3p
C17orf85	hsa-miR-412-3p
C17orf85	hsa-miR-7657-3p
C17orf85	hsa-miR-4418
C17orf85	hsa-miR-138-1-3p
C17orf85	hsa-miR-6827-5p
C17orf85	hsa-miR-3618
C17orf85	hsa-miR-619
C17orf85	hsa-miR-2477
C17orf85	hsa-miR-2442
C17orf85	hsa-miR-7661-5p
C17orf85	hsa-miR-350-3p
C17orf85	hsa-miR-3922-3p
C17orf85	hsa-miR-1956
C17orf85	hsa-miR-6694-3p
C17orf85	hsa-miR-548ah-5p
C17orf85	hsa-miR-2400
C17orf85	hsa-miR-144-3p
C17orf85	hsa-miR-302d-5p
C17orf85	hsa-miR-654-5p
C17orf85	hsa-miR-8791
C17orf85	hsa-miR-194-5p
C17orf85	hsa-miR-702-3p
C17orf85	hsa-miR-573
C17orf85	hsa-miR-29a-5p
C17orf85	hsa-miR-541-3p
C17orf85	hsa-miR-2299-3p
C17orf85	hsa-miR-5006-5p
C17orf85	hsa-miR-221-3p
C17orf85	hsa-miR-374b-5p
C17orf85	hsa-miR-6690-5p
C17orf85	hsa-miR-3688-3p
C17orf85	hsa-miR-3529-3p

C17orf85	hsa-miR-2881
C17orf85	hsa-miR-4774-5p
C17orf85	hsa-miR-452-5p
C17orf85	hsa-miR-5009-3p
C17orf85	hsa-miR-4291
C17orf85	hsa-miR-4709-3p
C17orf85	hsa-miR-6499-3p
C17orf85	hsa-miR-4762-5p
C17orf85	hsa-miR-5687
C17orf85	hsa-miR-2284d
C17orf85	hsa-miR-548aa
C17orf85	hsa-miR-548g-3p
C17orf85	hsa-miR-6398
C17orf85	hsa-miR-23-3p
C17orf85	hsa-miR-548au-3p
C17orf85	hsa-miR-505-3p
C17orf85	hsa-miR-2421
C17orf85	hsa-miR-758-3p
C17orf85	hsa-miR-5571-3p
C17orf85	hsa-miR-3646
C17orf85	hsa-miR-151
C17orf85	hsa-miR-1283
C17orf85	hsa-miR-8871
C17orf85	hsa-miR-3591-3p
C17orf85	hsa-miR-2324
C17orf85	hsa-miR-761
C17orf85	hsa-miR-2364
C17orf85	hsa-miR-2292
C17orf85	hsa-miR-302-5p
C17orf85	hsa-miR-1303
C17orf85	hsa-miR-6762-3p
C17orf85	hsa-miR-4439
C17orf85	hsa-miR-326
C17orf85	hsa-miR-4683
C17orf85	hsa-miR-15b-3p
C17orf85	hsa-miR-6663-5p
C17orf85	hsa-miR-614
C17orf85	hsa-miR-7079-3p
C17orf85	hsa-miR-1285-3p
C17orf85	hsa-miR-2284aa
C17orf85	hsa-miR-152-5p
C17orf85	hsa-miR-672-5p
C17orf85	hsa-miR-1781-3p
C17orf85	hsa-miR-4736
C17orf85	hsa-miR-6910-3p
C17orf85	hsa-miR-1252-5p

C17orf85	hsa-miR-7855-5p
C17orf85	hsa-miR-3572-5p
C17orf85	hsa-miR-4732-5p
C17orf85	hsa-miR-2394
C17orf85	hsa-miR-2387
C17orf85	hsa-miR-1306-5p
C17orf85	hsa-miR-2284b
C17orf85	hsa-miR-626
C17orf85	hsa-miR-429
C17orf85	hsa-miR-1298-5p
C17orf85	hsa-miR-742-3p
C17orf85	hsa-miR-4662-5p
C17orf85	hsa-miR-6907-5p
C17orf85	hsa-miR-658
C17orf85	hsa-miR-200c-3p
C17orf85	hsa-miR-3197
C17orf85	hsa-miR-3977
C17orf85	hsa-miR-6954-3p
C17orf85	hsa-miR-548aef
C17orf85	hsa-miR-6314
C17orf85	hsa-miR-4468
C17orf85	hsa-miR-7118-5p
C17orf85	hsa-miR-556-3p
C17orf85	hsa-miR-539-5p
C17orf85	hsa-miR-7107-5p
C17orf85	hsa-miR-105b
C17orf85	hsa-miR-883ab-3p
C17orf85	hsa-miR-4652-5p
C17orf85	hsa-miR-292-5p
C17orf85	hsa-miR-2285z
C17orf85	hsa-miR-6777-3p
C17orf85	hsa-miR-363-5p
C17orf85	hsa-miR-508b
C17orf85	hsa-miR-576-3p
C17orf85	hsa-miR-1271-5p
C17orf85	hsa-miR-2456
C17orf85	hsa-miR-370-3p
C17orf85	hsa-miR-7151-5p
C17orf85	hsa-miR-4297
C17orf85	hsa-miR-4274
C17orf85	hsa-miR-4257
C17orf85	hsa-miR-4518
C17orf85	hsa-miR-8063
C17orf85	hsa-miR-21-3p
C17orf85	hsa-miR-1654
C17orf85	hsa-miR-8795

C17orf85	hsa-miR-3557-3p
C17orf85	hsa-miR-7077-5p
C17orf85	hsa-miR-148-5p
C17orf85	hsa-miR-7216-3p
C17orf85	hsa-miR-3061-5p
C17orf85	hsa-miR-8821
C17orf85	hsa-miR-4796-5p
C17orf85	hsa-miR-7028-3p
C17orf85	hsa-miR-2360
C17orf85	hsa-miR-4789-5p
C17orf85	hsa-miR-1243-3p
C17orf85	hsa-miR-328
C17orf85	hsa-miR-3925-3p
C17orf85	hsa-miR-3606-5p
C17orf85	hsa-miR-4701-3p
C17orf85	hsa-let-7f-5p
C17orf85	hsa-miR-4758-3p
C17orf85	hsa-miR-412
C17orf85	hsa-miR-582-3p
C17orf85	hsa-miR-883b-5p
C17orf85	hsa-miR-1758
C17orf85	hsa-miR-6809-3p
C17orf85	hsa-miR-376b-5p
C17orf85	hsa-miR-130a-5p
C17orf85	hsa-miR-3664-5p
C17orf85	hsa-miR-19
C17orf85	hsa-miR-888-5p
C17orf85	hsa-miR-548j-5p
C17orf85	hsa-miR-495-3p
C17orf85	hsa-miR-1225-5p
C17orf85	hsa-miR-518-5p
C17orf85	hsa-miR-1457
C17orf85	hsa-miR-562
C17orf85	hsa-miR-6917-3p
C17orf85	hsa-miR-6858-3p
C17orf85	hsa-miR-295-5p
C17orf85	hsa-miR-2399-5p
C17orf85	hsa-miR-4684-5p
C17orf85	hsa-miR-889-5p
C17orf85	hsa-miR-344g
C17orf85	hsa-miR-4516
C17orf85	hsa-miR-6833-3p
C17orf85	hsa-miR-664-3p
C17orf85	hsa-miR-8060
C17orf85	hsa-miR-6358
C17orf85	hsa-miR-4280

C17orf85	hsa-miR-3180-5p
C17orf85	hsa-miR-509-3-5p
C17orf85	hsa-miR-383-5p
C17orf85	hsa-miR-6503-3p
C17orf85	hsa-miR-764-5p
C17orf85	hsa-miR-3925-5p
C17orf85	hsa-miR-4638-5p
C17orf85	hsa-miR-323a-5p
C17orf85	hsa-miR-8117
C17orf85	hsa-miR-6119-3p
C17orf85	hsa-miR-432-5p
C17orf85	hsa-miR-642b-5p
C17orf85	hsa-miR-6738-3p
C17orf85	hsa-miR-5710
C17orf85	hsa-miR-144-3p
C17orf85	hsa-miR-3088-3p
C17orf85	hsa-miR-1764-3p
C17orf85	hsa-miR-8059
C17orf85	hsa-miR-760
C17orf85	hsa-miR-4698
C17orf85	hsa-miR-7202-5p
C17orf85	hsa-miR-1255a
C17orf85	hsa-miR-6998-5p
C17orf85	hsa-miR-566
C17orf85	hsa-miR-1958
C17orf85	hsa-miR-513b-3p
C17orf85	hsa-miR-6083
C17orf85	hsa-miR-6350
C17orf85	hsa-miR-9-3-3p
C17orf85	hsa-miR-7650-3p
C17orf85	hsa-miR-1964-5p
C17orf85	hsa-miR-6076
C17orf85	hsa-miR-4446-3p
C17orf85	hsa-miR-1537-5p
C17orf85	hsa-miR-7013-3p
C17orf85	hsa-miR-369-3p
C17orf85	hsa-miR-4755-5p
C17orf85	hsa-miR-6959-5p
C17orf85	hsa-miR-1704
C17orf85	hsa-miR-421-5p
C17orf85	hsa-miR-4778-3p
C17orf85	hsa-miR-6848-3p
C17orf85	hsa-miR-7085-3p
C17orf85	hsa-miR-2284m
C17orf85	hsa-miR-497-5p
C17orf85	hsa-miR-4492

C17orf85	hsa-miR-210
C17orf85	hsa-miR-3167-5p
C17orf85	hsa-miR-499b-5p
C17orf85	hsa-miR-6739-5p
C17orf85	hsa-miR-1323
C17orf85	hsa-miR-2889
C17orf85	hsa-miR-300
C17orf85	hsa-miR-4647
C17orf85	hsa-miR-467a-5p
C17orf85	hsa-miR-483-3p.1
C17orf85	hsa-miR-6680-3p
C17orf85	hsa-miR-6828-3p
C17orf85	hsa-miR-293-5p
C17orf85	hsa-miR-8868
C17orf85	hsa-miR-670-3p
C17orf85	hsa-miR-153-3p
C17orf85	hsa-miR-3127-5p
C17orf85	hsa-miR-129b-3p
C17orf85	hsa-miR-6530
C17orf85	hsa-miR-136-3p
C17orf85	hsa-miR-294-3p
C17orf85	hsa-miR-7201-5p
C17orf85	hsa-miR-1185-3p
C17orf85	hsa-miR-7155-5p
C17orf85	hsa-miR-6969-5p
C17orf85	hsa-miR-7220-5p
C17orf85	hsa-miR-219-3p
C17orf85	hsa-miR-8841
C17orf85	hsa-miR-1930-3p
C17orf85	hsa-miR-1841
C17orf85	hsa-miR-1270
C17orf85	hsa-miR-502-5p
C17orf85	hsa-miR-676-3p
C17orf85	hsa-miR-29e
C17orf85	hsa-miR-6362
C17orf85	hsa-miR-5001-3p
C17orf85	hsa-miR-2362
C17orf85	hsa-miR-5586-3p
C17orf85	hsa-miR-1323-5p
C17orf85	hsa-miR-342-3p
C17orf85	hsa-miR-1245a
C17orf85	hsa-miR-203b-3p
C17orf85	hsa-miR-669bf-3p
C17orf85	hsa-miR-802
C17orf85	hsa-miR-1835
C17orf85	hsa-miR-298-5p

C17orf85	hsa-miR-15-5p
C17orf85	hsa-miR-5107-5p
C17orf85	hsa-miR-7170-3p
C17orf85	hsa-miR-6817-3p
C17orf85	hsa-miR-558
C17orf85	hsa-miR-2452
C17orf85	hsa-miR-6950-3p
C17orf85	hsa-miR-3978
C17orf85	hsa-miR-6918-3p
C17orf85	hsa-miR-193a-5p
C17orf85	hsa-miR-675-3p
C17orf85	hsa-miR-6513-5p
C17orf85	hsa-miR-7184-3p
C17orf85	hsa-miR-200ab-5p
C17orf85	hsa-miR-182-3p
C17orf85	hsa-miR-3616-5p
C17orf85	hsa-miR-344-3p
C17orf85	hsa-miR-6742-5p
C17orf85	hsa-miR-6727-3p
C17orf85	hsa-miR-1178-5p
C17orf85	hsa-miR-7047-5p
C17orf85	hsa-miR-125a-3p
C17orf85	hsa-miR-4251
C17orf85	hsa-miR-889
C17orf85	hsa-miR-3914
C17orf85	hsa-miR-6837-5p
C17orf85	hsa-miR-671-5p
C17orf85	hsa-miR-3667-3p
C17orf85	hsa-miR-2485
C17orf85	hsa-miR-6812-5p
C17orf85	hsa-miR-1302
C17orf85	hsa-miR-2917
C17orf85	hsa-miR-6119-5p
C17orf85	hsa-miR-450b-5p
C17orf85	hsa-miR-8889
C17orf85	hsa-miR-7073-3p
C17orf85	hsa-miR-1468-3p
C17orf85	hsa-miR-28-5p
C17orf85	hsa-miR-743-5p
C17orf85	hsa-miR-221-5p
C17orf85	hsa-miR-150-5p
C17orf85	hsa-miR-1234-3p
C17orf85	hsa-miR-7183-3p
C17orf85	hsa-miR-6749-5p
C17orf85	hsa-miR-4753-3p
C17orf85	hsa-miR-3060-3p

C17orf85	hsa-miR-137
C17orf85	hsa-miR-4261
C17orf85	hsa-miR-3558-3p
C17orf85	hsa-miR-151-3p
C17orf85	hsa-miR-3176
C17orf85	hsa-miR-326-3p
C17orf85	hsa-miR-764-3p
C17orf85	hsa-miR-455-3p.2
C17orf85	hsa-miR-10
C17orf85	hsa-miR-4436b-3p
C17orf85	hsa-miR-183-5p
C17orf85	hsa-miR-7218-5p
C17orf85	hsa-miR-347
C17orf85	hsa-miR-6955-5p
C17orf85	hsa-miR-660-3p
C17orf85	hsa-miR-28-5p
C17orf85	hsa-miR-1285-5p
C17orf85	hsa-miR-1842
C17orf85	hsa-miR-7865
C17orf85	hsa-miR-7676-5p
C17orf85	hsa-miR-4632-5p
C17orf85	hsa-miR-3083-5p
C17orf85	hsa-miR-7978
C17orf85	hsa-miR-208a-3p
C17orf85	hsa-miR-936
C17orf85	hsa-miR-2449
C17orf85	hsa-miR-2300-5p
C17orf85	hsa-miR-599
C17orf85	hsa-miR-3072-3p
C17orf85	hsa-miR-6921-3p
C17orf85	hsa-miR-3136-5p
C17orf85	hsa-miR-7042-3p
C17orf85	hsa-miR-4639-5p
C17orf85	hsa-miR-4505
C17orf85	hsa-miR-6988-5p
C17orf85	hsa-miR-6379
C17orf85	hsa-miR-6733-5p
C17orf85	hsa-miR-6927-3p
C17orf85	hsa-miR-4284
C17orf85	hsa-miR-496a-5p
C17orf85	hsa-miR-548bb-3p
C17orf85	hsa-miR-10b-3p
C17orf85	hsa-miR-7231-5p
C17orf85	hsa-miR-3584-5p
C17orf85	hsa-miR-4693-3p
C17orf85	hsa-miR-7053-5p

C17orf85	hsa-miR-2110
C17orf85	hsa-miR-7655-3p
C17orf85	hsa-miR-6333
C17orf85	hsa-miR-380-5p
C17orf85	hsa-miR-7578
C17orf85	hsa-miR-892-3p
C17orf85	hsa-miR-501-5p
C17orf85	hsa-miR-193-5p
C17orf85	hsa-miR-694
C17orf85	hsa-miR-4723-5p
C17orf85	hsa-miR-5582-3p
C17orf85	hsa-miR-1299
C17orf85	hsa-miR-105-5p
C17orf85	hsa-miR-362-3p
C17orf85	hsa-miR-3127-3p
C17orf85	hsa-miR-1825
C17orf85	hsa-miR-1184
C17orf85	hsa-miR-6962-5p
C17orf85	hsa-miR-7202-3p
C17orf85	hsa-miR-8087
C17orf85	hsa-miR-6895-3p
C17orf85	hsa-miR-7119-3p
C17orf85	hsa-miR-3583-5p
C17orf85	hsa-miR-6882-3p
C17orf85	hsa-miR-7668-3p
C17orf85	hsa-miR-4446
C17orf85	hsa-let-7c-1-3p
C17orf85	hsa-miR-7094b-2-5p
C17orf85	hsa-miR-5194
C17orf85	hsa-miR-7242-3p
C17orf85	hsa-miR-3596d
C17orf85	hsa-miR-502b
C17orf85	hsa-miR-669m-5p
C17orf85	hsa-miR-525-5p
C17orf85	hsa-miR-548i-3p
C17orf85	hsa-miR-16-1-3p
C17orf85	hsa-miR-218-2-3p
C17orf85	hsa-miR-6727-5p
C17orf85	hsa-miR-345-3p
C17orf85	hsa-miR-4524a-5p
C17orf85	hsa-miR-713
C17orf85	hsa-miR-7013-5p
C17orf85	hsa-miR-411-3p
C17orf85	hsa-miR-3137
C17orf85	hsa-miR-4508
C17orf85	hsa-miR-330-5p

C17orf85	hsa-miR-106-5p
C17orf85	hsa-miR-6715-3p
C17orf85	hsa-miR-7648-3p
C17orf85	hsa-miR-7067-5p
C17orf85	hsa-miR-3078-5p
C17orf85	hsa-miR-1271-3p
C17orf85	hsa-miR-495-5p
C17orf85	hsa-miR-7045-3p
C17orf85	hsa-miR-1651-3p
C17orf85	hsa-miR-215
C17orf85	hsa-miR-3105-3p
C17orf85	hsa-miR-6920-5p
C17orf85	hsa-miR-4700-5p
C17orf85	hsa-miR-292a-3p
C17orf85	hsa-miR-3688-5p
C17orf85	hsa-miR-4731-5p
C17orf85	hsa-miR-4259
C17orf85	hsa-miR-96-3p
C17orf85	hsa-miR-196
C17orf85	hsa-miR-299
C17orf85	hsa-miR-642a-5p
C17orf85	hsa-miR-3916
C17orf85	hsa-miR-4678
C17orf85	hsa-miR-290
C17orf85	hsa-miR-216b-3p
C17orf85	hsa-miR-2461-5p
C17orf85	hsa-miR-3658
C17orf85	hsa-miR-7049-3p
C17orf85	hsa-miR-2368-3p
C17orf85	hsa-miR-7159-5p
C17orf85	hsa-miR-744-5p
C17orf85	hsa-miR-6929-5p
C17orf85	hsa-miR-4685-3p
C17orf85	hsa-miR-593-3p
C17orf85	hsa-miR-200c-3p
C17orf85	hsa-miR-19-3p
C17orf85	hsa-miR-331-3p
C17orf85	hsa-miR-6571-3p
C17orf85	hsa-miR-1894-3p
C17orf85	hsa-miR-1208
C17orf85	hsa-miR-4686
C17orf85	hsa-miR-8894
C17orf85	hsa-miR-6737-5p
C17orf85	hsa-miR-6860
C17orf85	hsa-miR-2396
C17orf85	hsa-miR-7173-3p

C17orf85	hsa-miR-3067-5p
C17orf85	hsa-miR-3533
C17orf85	hsa-miR-1273f
C17orf85	hsa-miR-6883-3p
C17orf85	hsa-miR-139-3p
C17orf85	hsa-miR-197-3p
C17orf85	hsa-miR-6870-5p
C17orf85	hsa-miR-6528
C17orf85	hsa-miR-7052-3p
C17orf85	hsa-miR-6758-5p
C17orf85	hsa-miR-1712-3p
C17orf85	hsa-miR-201-3p
C17orf85	hsa-miR-5134-5p
C17orf85	hsa-miR-649
C17orf85	hsa-miR-183-3p
C17orf85	hsa-miR-327
C17orf85	hsa-miR-378
C17orf85	hsa-miR-199
C17orf85	hsa-miR-3158-5p
C17orf85	hsa-miR-5680
C17orf85	hsa-miR-8079
C17orf85	hsa-miR-5739
C17orf85	hsa-miR-6728-5p
C17orf85	hsa-miR-653-5p
C17orf85	hsa-miR-23-5p
C17orf85	hsa-miR-4513
C17orf85	hsa-miR-192-3p
C17orf85	hsa-miR-523-3p
C17orf85	hsa-miR-128
C17orf85	hsa-miR-29b-1-5p
C17orf85	hsa-miR-892a
C17orf85	hsa-miR-483-3p
C17orf85	hsa-miR-6765-3p
C17orf85	hsa-miR-6944-3p
C17orf85	hsa-miR-7370-5p
C17orf85	hsa-miR-7669-3p
C17orf85	hsa-miR-135-5p
C17orf85	hsa-miR-551b-5p
C17orf85	hsa-miR-24-3p
C17orf85	hsa-miR-6868-3p
C17orf85	hsa-miR-1434-5p
C17orf85	hsa-miR-192-5p
C17orf85	hsa-miR-6659-3p
C17orf85	hsa-miR-7113-5p
C17orf85	hsa-miR-1587
C17orf85	hsa-miR-3564

C17orf85	hsa-miR-590-3p
C17orf85	hsa-miR-1836
C17orf85	hsa-miR-467-3p
C17orf85	hsa-miR-6828-5p
C17orf85	hsa-miR-6714-3p
C17orf85	hsa-miR-4638-3p
C17orf85	hsa-miR-6931-5p
C17orf85	hsa-miR-2114-5p
C17orf85	hsa-miR-6886-3p
C17orf85	hsa-miR-4765
C17orf85	hsa-miR-2479
C17orf85	hsa-let-7f-2-3p
C17orf85	hsa-miR-6981-5p
C17orf85	hsa-miR-6797-5p
C17orf85	hsa-miR-1434-3p
C17orf85	hsa-miR-3091-3p
C17orf85	hsa-miR-5129-5p
C17orf85	hsa-miR-6724-5p
C17orf85	hsa-miR-520gh
C17orf85	hsa-miR-4482-5p
C17orf85	hsa-miR-454-5p
C17orf85	hsa-miR-133b-5p
C17orf85	hsa-miR-701-3p
C17orf85	hsa-miR-7048-3p
C17orf85	hsa-miR-4797-5p
C17orf85	hsa-miR-6588-3p
C17orf85	hsa-miR-2323
C17orf85	hsa-miR-548w
C17orf85	hsa-miR-4780
C17orf85	hsa-miR-8847
C17orf85	hsa-miR-6818-5p
C17orf85	hsa-miR-6909-3p
C17orf85	hsa-miR-5583-5p
C17orf85	hsa-miR-6774-5p
C17orf85	hsa-miR-5002-5p
C17orf85	hsa-miR-4706
C17orf85	hsa-miR-677
C17orf85	hsa-miR-4432
C17orf85	hsa-miR-3544
C17orf85	hsa-miR-1914-5p
C17orf85	hsa-miR-6999-5p
C17orf85	hsa-miR-548h-3p
C17orf85	hsa-miR-202-3p
C17orf85	hsa-miR-7066-5p
C17orf85	hsa-miR-290b-3p
C17orf85	hsa-miR-4729

C17orf85	hsa-miR-1182
C17orf85	hsa-miR-409-5p
C17orf85	hsa-miR-383-5p.2
C17orf85	hsa-miR-4646-5p
C17orf85	hsa-miR-100-3p
C17orf85	hsa-miR-130ac-5p
C17orf85	hsa-miR-7844-5p
C17orf85	hsa-miR-629-5p
C17orf85	hsa-miR-1269a
C17orf85	hsa-miR-2313-3p
C17orf85	hsa-miR-291-5p
C17orf85	hsa-miR-7088-5p
C17orf85	hsa-miR-572
C17orf85	hsa-miR-548at-3p
C17orf85	hsa-miR-3973
C17orf85	hsa-miR-1290
C17orf85	hsa-miR-568
C17orf85	hsa-miR-8818
C17orf85	hsa-miR-666-5p
C17orf85	hsa-miR-6833-5p
C17orf85	hsa-miR-3935
C17orf85	hsa-miR-4526
C17orf85	hsa-miR-2374
C17orf85	hsa-miR-6967-3p
C17orf85	hsa-miR-4453
C17orf85	hsa-miR-8814
C17orf85	hsa-miR-2284c
C17orf85	hsa-miR-3664-5p
C17orf85	hsa-miR-2464-5p
C17orf85	hsa-miR-6756-3p
C17orf85	hsa-miR-301-3p
C17orf85	hsa-miR-4667-3p
C17orf85	hsa-miR-4515
C17orf85	hsa-miR-548t-3p
C17orf85	hsa-miR-548as-5p
C17orf85	hsa-miR-133c-5p
C17orf85	hsa-miR-6785-5p
C17orf85	hsa-miR-302a-5p
C17orf85	hsa-miR-193a
C17orf85	hsa-miR-330-3p
C17orf85	hsa-miR-425-5p
C17orf85	hsa-miR-7162-5p
C17orf85	hsa-miR-547-3p
C17orf85	hsa-miR-2453
C17orf85	hsa-miR-1839-5p
C17orf85	hsa-miR-516b-5p

C17orf85	hsa-miR-138-5p
C17orf85	hsa-miR-210-5p
C17orf85	hsa-miR-6376
C17orf85	hsa-miR-7235-3p
C17orf85	hsa-miR-6371
C17orf85	hsa-miR-4436b-5p
C17orf85	hsa-miR-548p
C17orf85	hsa-miR-6762-5p
C17orf85	hsa-miR-6512-5p
C17orf85	hsa-miR-548am-3p
C17orf85	hsa-miR-548f-3p
C17orf85	hsa-miR-2315
C17orf85	hsa-let-7i-3p
C17orf85	hsa-miR-344b-3p
C17orf85	hsa-miR-6941-3p
C17orf85	hsa-miR-466-5p
C17orf85	hsa-miR-596
C17orf85	hsa-miR-6780a-3p
C17orf85	hsa-miR-4531
C17orf85	hsa-miR-589-5p
C17orf85	hsa-miR-549-5p
C17orf85	hsa-miR-499b-3p
C17orf85	hsa-miR-15
C17orf85	hsa-miR-8808
C17orf85	hsa-miR-7230-5p
C17orf85	hsa-miR-6769b-5p
C17orf85	hsa-miR-3689f
C17orf85	hsa-miR-543-5p
C17orf85	hsa-miR-4717-3p
C17orf85	hsa-miR-5046
C17orf85	hsa-miR-1976
C17orf85	hsa-miR-883-3p
C17orf85	hsa-miR-513a-5p
C17orf85	hsa-miR-939-3p
C17orf85	hsa-miR-494-3p
C17orf85	hsa-miR-1909-3p
C17orf85	hsa-miR-219b
C17orf85	hsa-miR-6975-5p
C17orf85	hsa-miR-3121-3p
C17orf85	hsa-miR-2424
C17orf85	hsa-miR-3063-3p
C17orf85	hsa-miR-4325
C17orf85	hsa-miR-1265
C17orf85	hsa-miR-6334
C17orf85	hsa-miR-7211-3p
C17orf85	hsa-miR-3175

C17orf85	hsa-miR-6720-5p
C17orf85	hsa-miR-6769a-5p
C17orf85	hsa-miR-4799-5p
C17orf85	hsa-miR-139-5p
C17orf85	hsa-miR-668
C17orf85	hsa-miR-296-5p
C17orf85	hsa-miR-6322
C17orf85	hsa-miR-7196-5p
C17orf85	hsa-miR-9769-5p
C17orf85	hsa-miR-6417
C17orf85	hsa-miR-2431-3p
C17orf85	hsa-miR-2285n
C17orf85	hsa-miR-711
C17orf85	hsa-miR-6387
C17orf85	hsa-miR-6847-5p
C17orf85	hsa-miR-135b-3p
C17orf85	hsa-miR-548aq-3p
C17orf85	hsa-miR-322-3p
C17orf85	hsa-miR-27b-3p
C17orf85	hsa-miR-507
C17orf85	hsa-miR-8908-3p
C17orf85	hsa-miR-433-3p
C17orf85	hsa-miR-155-3p
C17orf85	hsa-miR-744-3p
C17orf85	hsa-miR-520g-5p
C17orf85	hsa-miR-4299
C17orf85	hsa-miR-8903
C17orf85	hsa-miR-4459
C17orf85	hsa-miR-24-3p
C17orf85	hsa-miR-6883-5p
C17orf85	hsa-miR-522-3p
C17orf85	hsa-miR-6924-5p
C17orf85	hsa-miR-7168-5p
C17orf85	hsa-miR-7306-5p
C17orf85	hsa-miR-879-3p
C17orf85	hsa-miR-4469
C17orf85	hsa-miR-4311
C17orf85	hsa-miR-6132
C17orf85	hsa-miR-4771
C17orf85	hsa-miR-6904-5p
C17orf85	hsa-miR-500
C17orf85	hsa-miR-6516-3p
C17orf85	hsa-miR-7207-3p
C17orf85	hsa-miR-6776-5p
C17orf85	hsa-miR-4659a-5p
C17orf85	hsa-miR-217-3p

C17orf85	hsa-miR-1260b
C17orf85	hsa-miR-27
C17orf85	hsa-miR-1972
C17orf85	hsa-miR-6894-3p
C17orf85	hsa-miR-202-5p
C17orf85	hsa-miR-6920-3p
C17orf85	hsa-miR-5196-3p
C17orf85	hsa-miR-3622b-3p
C17orf85	hsa-miR-4535
C17orf85	hsa-miR-1942
C17orf85	hsa-miR-99b-3p
C17orf85	hsa-miR-4745-5p
C17orf85	hsa-miR-2284j
C17orf85	hsa-miR-548aw
C17orf85	hsa-miR-6372
C17orf85	hsa-miR-612
C17orf85	hsa-miR-3090-5p
C17orf85	hsa-miR-1197-5p
C17orf85	hsa-miR-490-5p
C17orf85	hsa-miR-1839-3p
C17orf85	hsa-miR-499a-3p
C17orf85	hsa-miR-7215-3p
C17orf85	hsa-miR-4732-3p
C17orf85	hsa-let-7c-3p
C17orf85	hsa-miR-2439-3p
C17orf85	hsa-miR-1227-3p
C17orf85	hsa-miR-219b-3p
C17orf85	hsa-miR-3653-5p
C17orf85	hsa-miR-7036b-3p
C17orf85	hsa-miR-381-3p
C17orf85	hsa-miR-668-5p
C17orf85	hsa-miR-7063-3p
C17orf85	hsa-miR-7090-5p
C17orf85	hsa-miR-2426
C17orf85	hsa-miR-33-5p
C17orf85	hsa-miR-2322-5p
C17orf85	hsa-miR-6519
C17orf85	hsa-miR-376c
C17orf85	hsa-miR-101-3p
C17orf85	hsa-miR-570-3p
C17orf85	hsa-miR-8056
C17orf85	hsa-miR-1298
C17orf85	hsa-miR-8066
C17orf85	hsa-miR-6944-5p
C17orf85	hsa-miR-4265
C17orf85	hsa-miR-6418-3p

C17orf85	hsa-miR-4703-5p
C17orf85	hsa-miR-103b
C17orf85	hsa-miR-92a-1-5p
C17orf85	hsa-miR-3593-5p
C17orf85	hsa-miR-5626-3p
C17orf85	hsa-miR-5581-5p
C17orf85	hsa-miR-455-3p
C17orf85	hsa-miR-4483
C17orf85	hsa-miR-556-5p
C17orf85	hsa-miR-6818-3p
C17orf85	hsa-miR-330-3p
C17orf85	hsa-miR-3924
C17orf85	hsa-miR-762
C17orf85	hsa-miR-615-3p
C17orf85	hsa-miR-6875-3p
C17orf85	hsa-miR-139-5p
C17orf85	hsa-miR-8892
C17orf85	hsa-miR-294
C17orf85	hsa-miR-5619-3p
C17orf85	hsa-miR-6981-3p
C17orf85	hsa-miR-455-5p
C17orf85	hsa-miR-4659b-5p
C17orf85	hsa-miR-7156-3p
C17orf85	hsa-miR-3102-3p
C17orf85	hsa-miR-34a-3p
C17orf85	hsa-miR-7051-3p
C17orf85	hsa-miR-6516-5p
C17orf85	hsa-miR-4772-3p
C17orf85	hsa-miR-3556a
C17orf85	hsa-miR-548ap-5p
C17orf85	hsa-miR-4690-5p
C17orf85	hsa-miR-8800
C17orf85	hsa-miR-2367-3p
C17orf85	hsa-miR-8904a
C17orf85	hsa-miR-7082-3p
C17orf85	hsa-miR-891-3p
C17orf85	hsa-miR-1231-5p
C17orf85	hsa-miR-655
C17orf85	hsa-miR-616-5p
C17orf85	hsa-miR-148-3p
C17orf85	hsa-miR-7160-3p
C17orf85	hsa-miR-382-5p
C17orf85	hsa-miR-3200-5p
C17orf85	hsa-miR-675-5p
C17orf85	hsa-miR-3689b-5p
C17orf85	hsa-miR-323-3p

C17orf85	hsa-miR-6759-3p
C17orf85	hsa-miR-295-3p
C17orf85	hsa-miR-548o-5p
C17orf85	hsa-miR-1304-3p
C17orf85	hsa-miR-1207
C17orf85	hsa-let-7e-3p
C17orf85	hsa-miR-1625-3p
C17orf85	hsa-miR-6509-3p
C17orf85	hsa-miR-483-3p.2
C17orf85	hsa-miR-6730-5p
C17orf85	hsa-miR-3081-5p
C17orf85	hsa-miR-8089
C17orf85	hsa-miR-4489
C17orf85	hsa-miR-6862-3p
C17orf85	hsa-miR-5010-3p
C17orf85	hsa-miR-5192
C17orf85	hsa-miR-7658-3p
C17orf85	hsa-let-7d-3p
C17orf85	hsa-miR-3562
C17orf85	hsa-miR-669hk-3p
C17orf85	hsa-miR-548az-5p
C17orf85	hsa-miR-873-5p
C17orf85	hsa-miR-6900-5p
C17orf85	hsa-miR-7158-3p
C17orf85	hsa-miR-3714
C17orf85	hsa-miR-6403
C17orf85	hsa-miR-3958
C17orf85	hsa-miR-1238-3p
C17orf85	hsa-miR-769-5p
C17orf85	hsa-miR-1237-3p
C17orf85	hsa-miR-7641
C17orf85	hsa-miR-1301-3p
C17orf85	hsa-miR-4275
C17orf85	hsa-miR-4694-3p
C17orf85	hsa-miR-7080-5p
C17orf85	hsa-miR-6861-3p
C17orf85	hsa-miR-6856-5p
C17orf85	hsa-miR-550b-3p
C17orf85	hsa-miR-4661-3p
C17orf85	hsa-miR-4423-3p
C17orf85	hsa-miR-1966-3p
C17orf85	hsa-miR-155-5p
C17orf85	hsa-miR-7052-5p
C17orf85	hsa-miR-6995-3p
C17orf85	hsa-miR-1179
C17orf85	hsa-miR-6761-5p

C17orf85	hsa-miR-7057-5p
C17orf85	hsa-miR-7679-3p
C17orf85	hsa-miR-6331
C17orf85	hsa-miR-3653-3p
C17orf85	hsa-miR-600
C17orf85	hsa-miR-7221-5p
C17orf85	hsa-miR-2472
C17orf85	hsa-miR-619-5p
C17orf85	hsa-miR-411-5p.2
C17orf85	hsa-miR-1470
C17orf85	hsa-miR-7649-5p
C17orf85	hsa-miR-3551-5p
C17orf85	hsa-miR-7153-3p
C17orf85	hsa-miR-431-5p
C17orf85	hsa-miR-517
C17orf85	hsa-miR-1284
C17orf85	hsa-miR-4478
C17orf85	hsa-miR-2284v
C17orf85	hsa-miR-2447
C17orf85	hsa-miR-3156-5p
C17orf85	hsa-miR-7680-3p
C17orf85	hsa-miR-939-5p
C17orf85	hsa-miR-3589
C17orf85	hsa-miR-3109-3p
C17orf85	hsa-miR-2412
C17orf85	hsa-miR-4324
C17orf85	hsa-miR-449c-3p
C17orf85	hsa-miR-888-5p
C17orf85	hsa-miR-101-3p.1
C17orf85	hsa-miR-363-3p
C17orf85	hsa-miR-3674
C17orf85	hsa-miR-3075-3p
C17orf85	hsa-miR-8802
C17orf85	hsa-miR-7172-3p
C17orf85	hsa-miR-496-5p
C17orf85	hsa-miR-300-5p
C17orf85	hsa-miR-2890
C17orf85	hsa-miR-351-5p
C17orf85	hsa-miR-665
C17orf85	hsa-miR-548as-3p
C17orf85	hsa-miR-3934-5p
C17orf85	hsa-miR-939-5p
C17orf85	hsa-miR-2478
C17orf85	hsa-miR-876-3p
C17orf85	hsa-miR-374-5p
C17orf85	hsa-miR-6640-5p

C17orf85	hsa-miR-2486-5p
C17orf85	hsa-miR-374c-5p
C17orf85	hsa-miR-4254
C17orf85	hsa-miR-6325
C17orf85	hsa-miR-1761
C17orf85	hsa-miR-6343
C17orf85	hsa-miR-7652-5p
C17orf85	hsa-miR-7176-5p
C17orf85	hsa-miR-1931
C17orf85	hsa-miR-1227-3p
C17orf85	hsa-miR-3103-3p
C17orf85	hsa-miR-7010-5p
C17orf85	hsa-miR-124-5p
C17orf85	hsa-miR-650abc
C17orf85	hsa-miR-7084-5p
C17orf85	hsa-miR-3106-3p
C17orf85	hsa-miR-874
C17orf85	hsa-miR-664
C17orf85	hsa-miR-1843
C17orf85	hsa-miR-7683-3p
C17orf85	hsa-miR-7859
C17orf85	hsa-miR-5694
C17orf85	hsa-miR-2343
C17orf85	hsa-miR-329-3p
C17orf85	hsa-miR-7311-5p
C17orf85	hsa-miR-4693-5p
C17orf85	hsa-miR-3544-5p
C17orf85	hsa-miR-548aq-5p
C17orf85	hsa-miR-3670
C17orf85	hsa-miR-6771-3p
C17orf85	hsa-miR-7376-5p
C17orf85	hsa-miR-8826
C17orf85	hsa-miR-1936
C17orf85	hsa-miR-6523a
C17orf85	hsa-miR-548ah-3p
C17orf85	hsa-miR-6506-5p
C17orf85	hsa-miR-508a
C17orf85	hsa-miR-505-3p
C17orf85	hsa-miR-3166
C17orf85	hsa-miR-1236-5p
C17orf85	hsa-miR-2329-3p
C17orf85	hsa-miR-548ae-5p
C17orf85	hsa-miR-2898
C17orf85	hsa-miR-5623-5p
C17orf85	hsa-miR-650
C17orf85	hsa-miR-3475-3p

C17orf85	hsa-miR-3976
C17orf85	hsa-miR-875-3p
C17orf85	hsa-miR-6932-3p
C17orf85	hsa-miR-203-3p
C17orf85	hsa-miR-298-3p
C17orf85	hsa-miR-503-5p
C17orf85	hsa-miR-5693
C17orf85	hsa-miR-218a-1-3p
C17orf85	hsa-miR-6822-3p
C17orf85	hsa-miR-8900
C17orf85	hsa-miR-6898-5p
C17orf85	hsa-miR-6787-3p
C17orf85	hsa-miR-1245a
C17orf85	hsa-miR-203-3p.2
C17orf85	hsa-miR-4742
C17orf85	hsa-miR-223-5p
C17orf85	hsa-miR-501-3p
C17orf85	hsa-miR-20a-3p
C17orf85	hsa-miR-3064-3p
C17orf85	hsa-miR-502-3p
C17orf85	hsa-miR-4749-3p
C17orf85	hsa-miR-548az-3p
C17orf85	hsa-miR-6783-5p
C17orf85	hsa-miR-7092-5p
C17orf85	hsa-miR-1451-3p
C17orf85	hsa-miR-667-5p
C17orf85	hsa-miR-6918-5p
C17orf85	hsa-miR-143-5p
C17orf85	hsa-miR-3059-5p
C17orf85	hsa-miR-214-3p
C17orf85	hsa-miR-636
C17orf85	hsa-miR-1563
C17orf85	hsa-miR-1897-3p
C17orf85	hsa-miR-3078-3p
C17orf85	hsa-miR-6354
C17orf85	hsa-miR-2888
C17orf85	hsa-miR-7438-5p
C17orf85	hsa-miR-491-5p
C17orf85	hsa-miR-548l
C17orf85	hsa-miR-4653-5p
C17orf85	hsa-miR-335-5p
C17orf85	hsa-miR-152-3p
C17orf85	hsa-miR-376a-2-5p
C17orf85	hsa-miR-1324
C17orf85	hsa-miR-1236-3p
C17orf85	hsa-miR-140-5p

C17orf85	hsa-miR-7650-5p
C17orf85	hsa-miR-6826-5p
C17orf85	hsa-miR-6318
C17orf85	hsa-miR-2366
C17orf85	hsa-miR-6747-5p
C17orf85	hsa-miR-450b-3p
C17orf85	hsa-miR-3578
C17orf85	hsa-miR-140-3p.2
C17orf85	hsa-miR-371
C17orf85	hsa-miR-1194
C17orf85	hsa-miR-743b-5p
C17orf85	hsa-miR-367-3p
C17orf85	hsa-miR-651-3p
C17orf85	hsa-miR-147a
C17orf85	hsa-miR-132-5p
C17orf85	hsa-miR-135a-3p
C17orf85	hsa-miR-2284y
C17orf85	hsa-miR-4667
C17orf85	hsa-miR-6773-5p
C17orf85	hsa-miR-1643-3p
C17orf85	hsa-miR-3076-3p
C17orf85	hsa-miR-25-3p
C17orf85	hsa-let-7d-5p
C17orf85	hsa-miR-6693-3p
C17orf85	hsa-miR-5121
C17orf85	hsa-miR-1241
C17orf85	hsa-miR-881-5p
C17orf85	hsa-miR-126-3p
C17orf85	hsa-miR-23
C17orf85	hsa-miR-6775-5p
C17orf85	hsa-miR-374c-3p
C17orf85	hsa-miR-1226-3p
C17orf85	hsa-miR-1611
C17orf85	hsa-miR-660-5p
C17orf85	hsa-miR-6922-3p
C17orf85	hsa-miR-6515-3p
C17orf85	hsa-miR-7220-3p
C17orf85	hsa-miR-2682-3p
C17orf85	hsa-miR-504-5p.1
C17orf85	hsa-miR-548ab
C17orf85	hsa-miR-4790-5p
C17orf85	hsa-miR-4434
C17orf85	hsa-miR-1307-3p
C17orf85	hsa-miR-7174-5p
C17orf85	hsa-miR-8792
C17orf85	hsa-miR-1224-3p

C17orf85	hsa-miR-340-5p
C17orf85	hsa-miR-6852-5p
C17orf85	hsa-miR-6825-5p
C17orf85	hsa-miR-548e-5p
C17orf85	hsa-miR-376c-3p
C17orf85	hsa-miR-23c
C17orf85	hsa-miR-6328
C17orf85	hsa-miR-548q
C17orf85	hsa-miR-9-3p
C17orf85	hsa-miR-7165-5p
C17orf85	hsa-miR-33-3p
C17orf85	hsa-miR-4768-5p
C17orf85	hsa-miR-5586-5p
C17orf85	hsa-miR-4704-5p
C17orf85	hsa-miR-520c-3p
C17orf85	hsa-miR-7041-3p
C17orf85	hsa-miR-6961-3p
C17orf85	hsa-miR-703
C17orf85	hsa-miR-1178-3p
C17orf85	hsa-miR-361-5p
C17orf85	hsa-miR-4712-3p
C17orf85	hsa-miR-1938
C17orf85	hsa-miR-6726-3p
C17orf85	hsa-miR-433-5p
C17orf85	hsa-miR-8835
C17orf85	hsa-miR-6742-3p
C17orf85	hsa-miR-126a-5p
C17orf85	hsa-miR-1814
C17orf85	hsa-miR-8849
C17orf85	hsa-miR-1273h-3p
C17orf85	hsa-miR-105-5p
C17orf85	hsa-miR-92-3p
C17orf85	hsa-miR-6867-3p
C17orf85	hsa-miR-6945-3p
C17orf85	hsa-miR-5615-3p
C17orf85	hsa-miR-615-5p
C17orf85	hsa-miR-193b-5p
C17orf85	hsa-miR-466bcp-3p
C17orf85	hsa-miR-186-5p
C17orf85	hsa-miR-545-3p
C17orf85	hsa-miR-4669
C17orf85	hsa-miR-4738-3p
C17orf85	hsa-miR-5187-3p
C17orf85	hsa-miR-7025-5p
C17orf85	hsa-miR-6847-3p
C17orf85	hsa-miR-146a-3p

C17orf85	hsa-miR-4423
C17orf85	hsa-miR-200a-3p
C17orf85	hsa-miR-6821-5p
C17orf85	hsa-miR-6778-3p
C17orf85	hsa-miR-193
C17orf85	hsa-miR-2385-5p
C17orf85	hsa-miR-3163
C17orf85	hsa-miR-6881-5p
C17orf85	hsa-miR-605-5p
C17orf85	hsa-miR-367-3p
C17orf85	hsa-miR-335-5p
C17orf85	hsa-miR-7164-5p
C17orf85	hsa-miR-5087
C17orf85	hsa-miR-4789-3p
C17orf85	hsa-miR-1260a
C17orf85	hsa-miR-7027-5p
C17orf85	hsa-miR-6815-3p
C17orf85	hsa-miR-140-5p
C17orf85	hsa-miR-30e-3p
C17orf85	hsa-miR-706
C17orf85	hsa-miR-3068-5p
C17orf85	hsa-miR-340-5p
C17orf85	hsa-miR-6915-3p
C17orf85	hsa-miR-1721
C17orf85	hsa-miR-16-2-3p
C17orf85	hsa-miR-214-5p
C17orf85	hsa-miR-6859-5p
C17orf85	hsa-miR-6991-5p
C17orf85	hsa-miR-7843-5p
C17orf85	hsa-miR-421
C17orf85	hsa-miR-8863
C17orf85	hsa-miR-7191-3p
C17orf85	hsa-miR-6748-5p
C17orf85	hsa-miR-770-5p
C17orf85	hsa-miR-338-5p
C17orf85	hsa-miR-6748-3p
C17orf85	hsa-miR-676-5p
C17orf85	hsa-miR-567
C17orf85	hsa-miR-22-5p
C17orf85	hsa-miR-362-5p
C17orf85	hsa-miR-2416
C17orf85	hsa-miR-4721
C17orf85	hsa-miR-8861
C17orf85	hsa-miR-4657
C17orf85	hsa-miR-4779
C17orf85	hsa-miR-8872

C17orf85	hsa-miR-297bc-3p
C17orf85	hsa-miR-1228-5p
C17orf85	hsa-miR-7001-3p
C17orf85	hsa-miR-191-3p
C17orf85	hsa-miR-765
C17orf85	hsa-miR-8108
C17orf85	hsa-miR-545-5p
C17orf85	hsa-miR-548ao-3p
C17orf85	hsa-miR-3156-3p
C17orf85	hsa-miR-6905-5p
C17orf85	hsa-miR-136-5p
C17orf85	hsa-miR-6769a-3p
C17orf85	hsa-miR-382-3p
C17orf85	hsa-miR-4481
C17orf85	hsa-miR-548bb-5p
C17orf85	hsa-miR-4684-3p
C17orf85	hsa-miR-4699-5p
C17orf85	hsa-miR-8862
C17orf85	hsa-miR-424-3p
C17orf85	hsa-miR-6823-5p
C17orf85	hsa-miR-6925-3p
C17orf85	hsa-miR-517-3p
C17orf85	hsa-miR-1733
C17orf85	hsa-miR-501-5p
C17orf85	hsa-miR-3064
C17orf85	hsa-miR-743b-3p
C17orf85	hsa-miR-5691
C17orf85	hsa-miR-1288-5p
C17orf85	hsa-miR-7150
C17orf85	hsa-miR-6965-5p
C17orf85	hsa-miR-2309
C17orf85	hsa-miR-4668-3p
C17orf85	hsa-miR-3158-3p
C17orf85	hsa-miR-6784-3p
C17orf85	hsa-miR-6747-3p
C17orf85	hsa-miR-708-5p
C17orf85	hsa-miR-365-3p
C17orf85	hsa-miR-4747-5p
C17orf85	hsa-miR-7213-5p
C17orf85	hsa-miR-8855
C17orf85	hsa-miR-8067
C17orf85	hsa-miR-6873-3p
C17orf85	hsa-miR-320e
C17orf85	hsa-miR-1277-5p
C17orf85	hsa-miR-3101-5p
C17orf85	hsa-miR-7b-3p

C17orf85	hsa-miR-1-5p
C17orf85	hsa-miR-7973
C17orf85	hsa-miR-7046-5p
C17orf85	hsa-miR-7160-5p
C17orf85	hsa-miR-6791-5p
C17orf85	hsa-miR-198
C17orf85	hsa-miR-6584-5p
C17orf85	hsa-miR-6889-3p
C17orf85	hsa-miR-3151-5p
C17orf85	hsa-miR-4281
C17orf85	hsa-miR-350
C17orf85	hsa-miR-6165
C17orf85	hsa-miR-7086-5p
C17orf85	hsa-miR-1291
C17orf85	hsa-miR-106-3p
C17orf85	hsa-miR-3941
C17orf85	hsa-miR-181
C17orf85	hsa-miR-604
C17orf85	hsa-miR-8837
C17orf85	hsa-miR-548av-3p
C17orf85	hsa-miR-3086-3p
C17orf85	hsa-miR-4307
C17orf85	hsa-miR-6982-5p
C17orf85	hsa-miR-767-5p
C17orf85	hsa-miR-4263
C17orf85	hsa-miR-7453-3p
C17orf85	hsa-miR-4486
C17orf85	hsa-miR-7200-5p
C17orf85	hsa-miR-4430
C17orf85	hsa-miR-331-5p
C17orf85	hsa-miR-4751
C17orf85	hsa-miR-498
C17orf85	hsa-miR-224-5p
C17orf85	hsa-miR-6804-3p
C17orf85	hsa-miR-296-3p
C17orf85	hsa-miR-4689
C17orf85	hsa-miR-8806
C17orf85	hsa-miR-4426
C17orf85	hsa-miR-3094-5p
C17orf85	hsa-miR-8097
C17orf85	hsa-miR-4524a-3p
C17orf85	hsa-miR-6856-3p
C17orf85	hsa-miR-30b-3p
C17orf85	hsa-miR-3552
C17orf85	hsa-miR-2317
C17orf85	hsa-miR-7659-3p

C17orf85	hsa-miR-3671
C17orf85	hsa-miR-627-3p
C17orf85	hsa-miR-1948-3p
C17orf85	hsa-miR-1304-5p
C17orf85	hsa-miR-6684-5p
C17orf85	hsa-miR-98-5p
C17orf85	hsa-miR-211-3p
C17orf85	hsa-miR-107-3p
C17orf85	hsa-miR-4498
C17orf85	hsa-miR-4718
C17orf85	hsa-miR-6089
C17orf85	hsa-miR-4649-3p
C17orf85	hsa-miR-4502
C17orf85	hsa-miR-933
C17orf85	hsa-miR-743ab-3p
C17orf85	hsa-miR-4663
C17orf85	hsa-miR-582-5p
C17orf85	hsa-miR-3915
C17orf85	hsa-miR-2388-5p
C17orf85	hsa-miR-3622a-5p
C17orf85	hsa-miR-7231-3p
C17orf85	hsa-miR-5189-5p
C17orf85	hsa-miR-448-3p
C17orf85	hsa-miR-5133
C17orf85	hsa-miR-3588
C17orf85	hsa-miR-4723-3p
C17orf85	hsa-miR-2429
C17orf85	hsa-miR-2411-5p
C17orf85	hsa-miR-519
C17orf85	hsa-miR-1558
C17orf85	hsa-miR-6923-3p
C17orf85	hsa-miR-3583-3p
C17orf85	hsa-miR-7688-5p
C17orf85	hsa-let-7a-3p
C17orf85	hsa-miR-7043-3p
C17orf85	hsa-miR-6910-5p
C17orf85	hsa-miR-672-3p
C17orf85	hsa-miR-877-3p
C17orf85	hsa-miR-322-5p
C17orf85	hsa-miR-544b
C17orf85	hsa-miR-7847-3p
C17orf85	hsa-miR-547-5p
C17orf85	hsa-miR-126-5p
C17orf85	hsa-miR-186-3p
C17orf85	hsa-miR-125
C17orf85	hsa-miR-8100

C17orf85	hsa-miR-425
C17orf85	hsa-miR-6832-5p
C17orf85	hsa-miR-7-5p
C17orf85	hsa-miR-6605-5p
C17orf85	hsa-miR-3092-5p
C17orf85	hsa-miR-7039-5p
C17orf85	hsa-miR-6887-3p
C17orf85	hsa-miR-4734
C17orf85	hsa-miR-335-3p
C17orf85	hsa-miR-3068-3p
C17orf85	hsa-miR-524-5p
C17orf85	hsa-miR-4786-3p
C17orf85	hsa-miR-3569-3p
C17orf85	hsa-miR-466c-3p
C17orf85	hsa-miR-3530-3p
C17orf85	hsa-miR-4772-5p
C17orf85	hsa-miR-8836
C17orf85	hsa-miR-5119
C17orf85	hsa-miR-2296
C17orf85	hsa-miR-186-5p
C17orf85	hsa-miR-492
C17orf85	hsa-miR-661
C17orf85	hsa-miR-3685
C17orf85	hsa-miR-3927-3p
C17orf85	hsa-miR-27a-5p
C17orf85	hsa-miR-1910-5p
C17orf85	hsa-miR-4795-3p
C17orf85	hsa-miR-6829-5p
C17orf85	hsa-miR-200
C17orf85	hsa-miR-3130-5p
C17orf85	hsa-miR-3618-3p
C17orf85	hsa-miR-384-3p
C17orf85	hsa-miR-6378
C17orf85	hsa-miR-3672
C17orf85	hsa-miR-512-5p
C17orf85	hsa-miR-7074-5p
C17orf85	hsa-miR-7207-5p
C17orf85	hsa-miR-548b-3p
C17orf85	hsa-miR-2285i
C17orf85	hsa-miR-556-5p
C17orf85	hsa-miR-7166-5p
C17orf85	hsa-miR-1246
C17orf85	hsa-miR-7165-3p
C17orf85	hsa-miR-383-5p.1
C17orf85	hsa-miR-22-3p
C17orf85	hsa-miR-6508-5p

C17orf85	hsa-miR-3124-3p
C17orf85	hsa-miR-6641-3p
C17orf85	hsa-miR-6540-3p
C17orf85	hsa-miR-6890-3p
C17orf85	hsa-miR-4650-3p
C17orf85	hsa-miR-5584-5p
C17orf85	hsa-miR-3182
C17orf85	hsa-miR-7063-5p
C17orf85	hsa-miR-1731-3p
C17orf85	hsa-miR-574-3p
C17orf85	hsa-miR-8103
C17orf85	hsa-miR-686
C17orf85	hsa-miR-4292
C17orf85	hsa-miR-4720-3p
C17orf85	hsa-miR-7039-3p
C17orf85	hsa-miR-470-5p
C17orf85	hsa-miR-7679-5p
C17orf85	hsa-miR-4512
C17orf85	hsa-miR-504
C17orf85	hsa-miR-6857-5p
C17orf85	hsa-miR-218-5p
C17orf85	hsa-miR-3135b
C17orf85	hsa-miR-719
C17orf85	hsa-miR-7232-5p
C17orf85	hsa-miR-3073b-3p
C17orf85	hsa-miR-497-5p
C17orf85	hsa-miR-2285
C17orf85	hsa-miR-382-5p
C17orf85	hsa-miR-2465
C17orf85	hsa-miR-7200-3p
C17orf85	hsa-miR-1199-5p
C17orf85	hsa-miR-4700-3p
C17orf85	hsa-miR-764
C17orf85	hsa-miR-1744-3p
C17orf85	hsa-miR-3152-3p
C17orf85	hsa-let-7abf-3p
C17orf85	hsa-miR-1929-5p
C17orf85	hsa-miR-4763-3p
C17orf85	hsa-miR-4735-5p
C17orf85	hsa-miR-7846-3p
C17orf85	hsa-miR-371a-5p
C17orf85	hsa-miR-6736-3p
C17orf85	hsa-miR-1198-3p
C17orf85	hsa-miR-548d-3p
C17orf85	hsa-miR-6957-5p
C17orf85	hsa-miR-548ao-5p

C17orf85	hsa-miR-32-3p
C17orf85	hsa-miR-376a-1-5p
C17orf85	hsa-miR-4659a-3p
C17orf85	hsa-miR-15a-3p
C17orf85	hsa-miR-6517
C17orf85	hsa-miR-5187-5p
C17orf85	hsa-miR-1933-3p
C17orf85	hsa-miR-4677-5p
C17orf85	hsa-miR-6513-3p
C17orf85	hsa-let-7f-1-3p
C17orf85	hsa-miR-6084
C17orf85	hsa-miR-892b
C17orf85	hsa-miR-7006-5p
C17orf85	hsa-miR-4433a-3p
C17orf85	hsa-miR-704
C17orf85	hsa-miR-548d-5p
C17orf85	hsa-miR-16-3p
C17orf85	hsa-miR-1245b-3p
C17orf85	hsa-miR-6508-3p
C17orf85	hsa-miR-4674
C17orf85	hsa-miR-770-3p
C17orf85	hsa-miR-513c-5p
C17orf85	hsa-miR-145-5p
C17orf85	hsa-miR-6514-3p
C17orf85	hsa-miR-2370-5p
C17orf85	hsa-miR-587
C17orf85	hsa-miR-4710
C17orf85	hsa-miR-101b-3p.2
C17orf85	hsa-miR-4670-3p
C17orf85	hsa-miR-106a-3p
C17orf85	hsa-miR-7456-5p
C17orf85	hsa-miR-25-3p
C17orf85	hsa-miR-18
C17orf85	hsa-miR-6722-3p
C17orf85	hsa-miR-485-3p
C17orf85	hsa-miR-379-3p
C17orf85	hsa-miR-224-3p
C17orf85	hsa-miR-6793-5p
C17orf85	hsa-miR-6511-3p
C17orf85	hsa-miR-548i-5p
C17orf85	hsa-miR-7482-3p
C17orf85	hsa-miR-301
C17orf85	hsa-miR-6546-5p
C17orf85	hsa-miR-203a-5p
C17orf85	hsa-miR-133cd-5p
C17orf85	hsa-miR-7118-3p

C17orf85	hsa-miR-4766-5p
C17orf85	hsa-miR-7114-5p
C17orf85	hsa-miR-4421
C17orf85	hsa-miR-6720-3p
C17orf85	hsa-miR-670-5p
C17orf85	hsa-miR-4444
C17orf85	hsa-miR-4662a-3p
C17orf85	hsa-miR-2284i
C17orf85	hsa-miR-5008-5p
C17orf85	hsa-miR-499
C17orf85	hsa-miR-520g-3p
C17orf85	hsa-miR-2483
C17orf85	hsa-miR-1304-5p
C17orf85	hsa-miR-4726-3p
C17orf85	hsa-miR-466q
C17orf85	hsa-miR-3190-3p
C17orf85	hsa-miR-4666a-3p
C17orf85	hsa-miR-664-5p
C17orf85	hsa-miR-1b-5p
C17orf85	hsa-miR-5112
C17orf85	hsa-miR-1838
C17orf85	hsa-miR-6804-5p
C17orf85	hsa-miR-484
C17orf85	hsa-miR-8064
C17orf85	hsa-miR-4803
C17orf85	hsa-miR-4671-5p
C17orf85	hsa-miR-1200
C17orf85	hsa-miR-203-3p.1
C17orf85	hsa-miR-191-5p
C17orf85	hsa-miR-199a-5p
C17orf85	hsa-miR-7190-3p
C17orf85	hsa-miR-4452
C17orf85	hsa-miR-466ade-3p
C17orf85	hsa-miR-873-3p
C17orf85	hsa-miR-3199
C17orf85	hsa-miR-1255-5p
C17orf85	hsa-miR-2284z
C17orf85	hsa-miR-548a-5p
C17orf85	hsa-miR-539-5p
C17orf85	hsa-miR-508-5p
C17orf85	hsa-miR-4662b
C17orf85	hsa-miR-134-3p
C17orf85	hsa-miR-6945-5p
C17orf85	hsa-miR-323a-5p
C17orf85	hsa-miR-940
C17orf85	hsa-miR-192-5p

C17orf85	hsa-miR-490-3p
C17orf85	hsa-miR-2389
C17orf85	hsa-miR-2461-3p
C17orf85	hsa-miR-3191-3p
C17orf85	hsa-miR-7170-5p
C17orf85	hsa-miR-465-5p
C17orf85	hsa-miR-6808-3p
C17orf85	hsa-miR-302
C17orf85	hsa-miR-200bc-3p
C17orf85	hsa-let-7e-5p
C17orf85	hsa-miR-667-3p
C17orf85	hsa-miR-22-3p
C17orf85	hsa-miR-3074-3p
C17orf85	hsa-miR-5094
C17orf85	hsa-miR-450a-2-3p
C17orf85	hsa-miR-1185-5p
C17orf85	hsa-miR-6127
C17orf85	hsa-miR-129-3p
C17orf85	hsa-miR-129-2-3p
C17orf85	hsa-miR-4267
C17orf85	hsa-miR-3075-5p
C17orf85	hsa-miR-603
C17orf85	hsa-miR-4740-5p
C17orf85	hsa-miR-138-2-3p
C17orf85	hsa-miR-760-5p
C17orf85	hsa-miR-1657
C17orf85	hsa-miR-635
C17orf85	hsa-miR-217-5p
C17orf85	hsa-miR-590-5p
C17orf85	hsa-miR-7192-5p
C17orf85	hsa-miR-7674-5p
C17orf85	hsa-miR-561-3p
C17orf85	hsa-miR-652-5p
C17orf85	hsa-miR-4641
C17orf85	hsa-miR-194-3p
C17orf85	hsa-miR-6943-5p
C17orf85	hsa-miR-7049-5p
C17orf85	hsa-miR-677-3p
C17orf85	hsa-miR-655-3p
C17orf85	hsa-miR-4322
C17orf85	hsa-miR-3154
C17orf85	hsa-miR-3155a
C17orf85	hsa-miR-5125-5p
C17orf85	hsa-miR-6716-5p
C17orf85	hsa-miR-326-5p
C17orf85	hsa-miR-545-3p

C17orf85	hsa-miR-149-5p
C17orf85	hsa-miR-7652-3p
C17orf85	hsa-miR-487a
C17orf85	hsa-miR-6811-3p
C17orf85	hsa-miR-6776-3p
C17orf85	hsa-miR-7113-3p
C17orf85	hsa-miR-6810-5p
C17orf85	hsa-miR-1930-5p
C17orf85	hsa-miR-7664-3p
C17orf85	hsa-miR-769
C17orf85	hsa-miR-7034-5p
C17orf85	hsa-miR-934
C17orf85	hsa-miR-7237-3p
C17orf85	hsa-miR-6526
C17orf85	hsa-miR-34bc-3p
C17orf85	hsa-miR-467f
C17orf85	hsa-miR-1199-3p
C17orf85	hsa-miR-3665
C17orf85	hsa-miR-4708-5p
C17orf85	hsa-miR-4443
C17orf85	hsa-miR-2117
C17orf85	hsa-miR-6940-5p
C17orf85	hsa-miR-6851-5p
C17orf85	hsa-miR-291-3p
C17orf85	hsa-miR-7115-3p
C17orf85	hsa-miR-6500-3p
C17orf85	hsa-miR-7230-3p
C17orf85	hsa-miR-8848
C17orf85	hsa-miR-6958-3p
C17orf85	hsa-miR-557
C17orf85	hsa-miR-6504-3p
C17orf85	hsa-miR-4495
C17orf85	hsa-miR-3113-5p
C17orf85	hsa-miR-466n-3p
C17orf85	hsa-miR-548t-5p
C17orf85	hsa-miR-361-3p
C17orf85	hsa-miR-8846
C17orf85	hsa-miR-384
C17orf85	hsa-miR-4675
C17orf85	hsa-miR-7678-3p
C17orf85	hsa-miR-323b-3p
C17orf85	hsa-miR-3593-3p
C17orf85	hsa-miR-548av-5p
C17orf85	hsa-miR-223-3p
C17orf85	hsa-miR-2900
C17orf85	hsa-miR-6894-5p

C17orf85	hsa-miR-6616-5p
C17orf85	hsa-miR-634
C17orf85	hsa-miR-30c-3p
C17orf85	hsa-miR-6396
C17orf85	hsa-miR-7011-5p
C17orf85	hsa-miR-517a-3p
C17orf85	hsa-miR-7-3p
C17orf85	hsa-miR-6964-5p
C17orf85	hsa-miR-500a-3p
C17orf85	hsa-miR-503-5p
C17orf85	hsa-miR-6788-5p
C17orf85	hsa-miR-548ay-5p
C17orf85	hsa-miR-106b-3p
C17orf85	hsa-miR-2382-3p
C17orf85	hsa-miR-383-3p
C17orf85	hsa-miR-4677-3p
C17orf85	hsa-miR-3587
C17orf85	hsa-miR-2378
C17orf85	hsa-miR-1288-3p
C17orf85	hsa-miR-140-3p.1
C17orf85	hsa-miR-4725-3p
C17orf85	hsa-miR-9a-3p
C17orf85	hsa-miR-2285aa
C17orf85	hsa-miR-7204-5p
C17orf85	hsa-miR-2418
C17orf85	hsa-miR-4668-5p
C17orf85	hsa-miR-1260a
C17orf85	hsa-miR-196b-3p
C17orf85	hsa-miR-101-1-5p
C17orf85	hsa-miR-5681a
C17orf85	hsa-miR-7314-3p
C17orf85	hsa-miR-6913-5p
C17orf85	hsa-miR-3691-5p
C17orf85	hsa-miR-4761-3p
C17orf85	hsa-miR-874-3p
C17orf85	hsa-miR-3651
C17orf85	hsa-miR-3689-3p
C17orf85	hsa-miR-8883
C17orf85	hsa-miR-216-5p
C17orf85	hsa-miR-3150a-3p
C17orf85	hsa-miR-642-3p
C17orf85	hsa-miR-6895-5p
C17orf85	hsa-miR-7203-5p
C17orf85	hsa-miR-4760-5p
C17orf85	hsa-miR-429-5p
C17orf85	hsa-miR-2450a

C17orf85	hsa-miR-3575
C17orf85	hsa-miR-7673-3p
C17orf85	hsa-miR-3606-3p
C17orf85	hsa-miR-320a
C17orf85	hsa-miR-563
C17orf85	hsa-miR-9-5p
CYFIP1	hsa-miR-7220-5p
CYFIP1	hsa-miR-4314
CYFIP1	hsa-miR-3106-3p
CYFIP1	hsa-miR-216-5p
CYFIP1	hsa-miR-1b-5p
CYFIP1	hsa-miR-466aep-5p
CYFIP1	hsa-miR-4646-5p
CYFIP1	hsa-miR-6819-3p
CYFIP1	hsa-miR-548ag
CYFIP1	hsa-miR-6780a-3p
CYFIP1	hsa-miR-763
CYFIP1	hsa-miR-509-3p
CYFIP1	hsa-miR-3688-3p
CYFIP1	hsa-miR-1252-3p
CYFIP1	hsa-miR-8836
CYFIP1	hsa-miR-615-3p
CYFIP1	hsa-miR-4777-5p
CYFIP1	hsa-miR-3173-3p
CYFIP1	hsa-miR-494-3p
CYFIP1	hsa-miR-496.2
CYFIP1	hsa-miR-4803
CYFIP1	hsa-miR-7168-5p
CYFIP1	hsa-miR-1262-3p
CYFIP1	hsa-miR-378
CYFIP1	hsa-miR-1266-3p
CYFIP1	hsa-miR-6909-3p
CYFIP1	hsa-miR-7180-3p
CYFIP1	hsa-miR-6982-5p
CYFIP1	hsa-miR-412
CYFIP1	hsa-miR-1278
CYFIP1	hsa-miR-7192-5p
CYFIP1	hsa-miR-4255
CYFIP1	hsa-miR-683
CYFIP1	hsa-miR-6994-5p
CYFIP1	hsa-miR-4799-5p
CYFIP1	hsa-miR-4263
CYFIP1	hsa-miR-3137
CYFIP1	hsa-miR-1915-3p
CYFIP1	hsa-miR-4796-3p
CYFIP1	hsa-miR-7231-3p

CYFIP1	hsa-miR-3136-5p
CYFIP1	hsa-miR-742-5p
CYFIP1	hsa-miR-93-5p
CYFIP1	hsa-miR-943
CYFIP1	hsa-miR-877-5p
CYFIP1	hsa-miR-3159
CYFIP1	hsa-miR-135
CYFIP1	hsa-miR-216a-5p
CYFIP1	hsa-miR-6730-3p
CYFIP1	hsa-miR-499b-3p
CYFIP1	hsa-miR-486-3p
CYFIP1	hsa-miR-7338-3p
CYFIP1	hsa-miR-6504-3p
CYFIP1	hsa-miR-6348
CYFIP1	hsa-miR-27-3p
CYFIP1	hsa-miR-5101
CYFIP1	hsa-miR-6970-5p
CYFIP1	hsa-miR-877-3p
CYFIP1	hsa-miR-449-5p
CYFIP1	hsa-miR-1388-3p
CYFIP1	hsa-miR-129-5p
CYFIP1	hsa-miR-511
CYFIP1	hsa-miR-548t-5p
CYFIP1	hsa-miR-128-3p
CYFIP1	hsa-miR-2325
CYFIP1	hsa-miR-766-3p
CYFIP1	hsa-miR-619-5p
CYFIP1	hsa-miR-138-5p
CYFIP1	hsa-miR-2367-3p
CYFIP1	hsa-miR-4732-3p
CYFIP1	hsa-miR-2311
CYFIP1	hsa-miR-631
CYFIP1	hsa-miR-3128
CYFIP1	hsa-miR-107-5p
CYFIP1	hsa-miR-7678-3p
CYFIP1	hsa-miR-154-5p
CYFIP1	hsa-miR-23-3p
CYFIP1	hsa-miR-604
CYFIP1	hsa-miR-206-3p
CYFIP1	hsa-miR-8118
CYFIP1	hsa-miR-5119
CYFIP1	hsa-miR-138-2-3p
CYFIP1	hsa-miR-7330-3p
CYFIP1	hsa-miR-7015-3p
CYFIP1	hsa-miR-6810-3p
CYFIP1	hsa-miR-542-5p

CYFIP1	hsa-miR-22-3p
CYFIP1	hsa-miR-340-3p
CYFIP1	hsa-miR-4758-5p
CYFIP1	hsa-miR-192-5p
CYFIP1	hsa-miR-290b-5p
CYFIP1	hsa-miR-466
CYFIP1	hsa-miR-4719
CYFIP1	hsa-miR-8056
CYFIP1	hsa-miR-7388a-3p
CYFIP1	hsa-miR-6506-5p
CYFIP1	hsa-miR-7218-3p
CYFIP1	hsa-miR-3692-3p
CYFIP1	hsa-miR-21-5p
CYFIP1	hsa-miR-92-3p
CYFIP1	hsa-miR-7087-5p
CYFIP1	hsa-miR-4765
CYFIP1	hsa-miR-126-5p
CYFIP1	hsa-miR-4460
CYFIP1	hsa-miR-410-5p
CYFIP1	hsa-miR-2284
CYFIP1	hsa-miR-6722-3p
CYFIP1	hsa-miR-543
CYFIP1	hsa-miR-6858-5p
CYFIP1	hsa-miR-642-3p
CYFIP1	hsa-miR-885-3p
CYFIP1	hsa-miR-607
CYFIP1	hsa-miR-1250-5p
CYFIP1	hsa-miR-708-5p
CYFIP1	hsa-miR-548aef
CYFIP1	hsa-miR-2284k
CYFIP1	hsa-miR-297-5p
CYFIP1	hsa-miR-4632-5p
CYFIP1	hsa-miR-767-3p
CYFIP1	hsa-let-7g-3p
CYFIP1	hsa-miR-585-5p
CYFIP1	hsa-miR-451a
CYFIP1	hsa-miR-29c-5p
CYFIP1	hsa-miR-548m
CYFIP1	hsa-miR-760
CYFIP1	hsa-miR-589-5p
CYFIP1	hsa-miR-6871-5p
CYFIP1	hsa-miR-7172-5p
CYFIP1	hsa-miR-376a-2-5p
CYFIP1	hsa-miR-4793-5p
CYFIP1	hsa-miR-4536-3p
CYFIP1	hsa-miR-669aflp-5p

CYFIP1	hsa-miR-5589-5p
CYFIP1	hsa-miR-6856-5p
CYFIP1	hsa-miR-6777-3p
CYFIP1	hsa-miR-8837
CYFIP1	hsa-miR-6358
CYFIP1	hsa-miR-6526
CYFIP1	hsa-miR-497-5p
CYFIP1	hsa-let-7a-2-3p
CYFIP1	hsa-miR-186-5p
CYFIP1	hsa-miR-8074
CYFIP1	hsa-miR-499
CYFIP1	hsa-miR-4708-5p
CYFIP1	hsa-miR-1744-3p
CYFIP1	hsa-miR-21-5p
CYFIP1	hsa-miR-1668
CYFIP1	hsa-miR-7843-5p
CYFIP1	hsa-miR-197-3p
CYFIP1	hsa-miR-3540
CYFIP1	hsa-miR-2455
CYFIP1	hsa-miR-450b
CYFIP1	hsa-miR-103-3p
CYFIP1	hsa-miR-6841-3p
CYFIP1	hsa-miR-6945-3p
CYFIP1	hsa-miR-7196-5p
CYFIP1	hsa-miR-499a-3p
CYFIP1	hsa-miR-7194-5p
CYFIP1	hsa-miR-3577
CYFIP1	hsa-miR-5690
CYFIP1	hsa-miR-470-5p
CYFIP1	hsa-miR-409-3p
CYFIP1	hsa-miR-8084
CYFIP1	hsa-miR-30a-3p
CYFIP1	hsa-miR-26-3p
CYFIP1	hsa-miR-320a
CYFIP1	hsa-miR-409-5p
CYFIP1	hsa-miR-300
CYFIP1	hsa-miR-2301
CYFIP1	hsa-miR-7287-3p
CYFIP1	hsa-miR-524-5p
CYFIP1	hsa-miR-2489
CYFIP1	hsa-miR-302a-5p
CYFIP1	hsa-miR-7371b-3p
CYFIP1	hsa-let-7f-2-3p
CYFIP1	hsa-miR-7244-5p
CYFIP1	hsa-miR-6577-3p
CYFIP1	hsa-miR-507

CYFIP1	hsa-miR-5591-3p
CYFIP1	hsa-miR-2446
CYFIP1	hsa-miR-523-3p
CYFIP1	hsa-miR-3176
CYFIP1	hsa-miR-563
CYFIP1	hsa-miR-6869-5p
CYFIP1	hsa-miR-183-5p
CYFIP1	hsa-miR-519
CYFIP1	hsa-miR-8068
CYFIP1	hsa-miR-384-3p
CYFIP1	hsa-miR-2401
CYFIP1	hsa-miR-6132
CYFIP1	hsa-miR-185-5p
CYFIP1	hsa-miR-539-3p
CYFIP1	hsa-miR-1276
CYFIP1	hsa-miR-208a-5p
CYFIP1	hsa-miR-5681b
CYFIP1	hsa-miR-16-5p
CYFIP1	hsa-miR-669j
CYFIP1	hsa-miR-7258-5p
CYFIP1	hsa-miR-2183
CYFIP1	hsa-miR-323-3p
CYFIP1	hsa-miR-8061
CYFIP1	hsa-miR-7688-3p
CYFIP1	hsa-miR-8070
CYFIP1	hsa-miR-518d-5p
CYFIP1	hsa-miR-101-3p
CYFIP1	hsa-miR-9500
CYFIP1	hsa-miR-1781-5p
CYFIP1	hsa-miR-7236-3p
CYFIP1	hsa-miR-1206
CYFIP1	hsa-miR-548ar-3p
CYFIP1	hsa-miR-3575
CYFIP1	hsa-miR-1260a
CYFIP1	hsa-miR-193-3p
CYFIP1	hsa-miR-199b-2-5p
CYFIP1	hsa-miR-6943-5p
CYFIP1	hsa-miR-493-3p
CYFIP1	hsa-miR-3064-3p
CYFIP1	hsa-miR-378c
CYFIP1	hsa-miR-1769-3p
CYFIP1	hsa-miR-2284aa
CYFIP1	hsa-miR-548c-3p
CYFIP1	hsa-miR-329-3p
CYFIP1	hsa-miR-182-5p
CYFIP1	hsa-miR-134

CYFIP1	hsa-miR-940
CYFIP1	hsa-miR-4783-3p
CYFIP1	hsa-miR-133a-5p
CYFIP1	hsa-miR-516b-3p
CYFIP1	hsa-miR-210
CYFIP1	hsa-miR-8885
CYFIP1	hsa-miR-2320-5p
CYFIP1	hsa-miR-6925-3p
CYFIP1	hsa-miR-208-5p
CYFIP1	hsa-miR-6508-5p
CYFIP1	hsa-miR-4512
CYFIP1	hsa-miR-1251-3p
CYFIP1	hsa-miR-331-3p
CYFIP1	hsa-miR-144-3p
CYFIP1	hsa-miR-619
CYFIP1	hsa-miR-1397-5p
CYFIP1	hsa-miR-96-5p
CYFIP1	hsa-miR-743ab-3p
CYFIP1	hsa-miR-302b-5p
CYFIP1	hsa-miR-653-5p
CYFIP1	hsa-miR-206
CYFIP1	hsa-miR-6752-3p
CYFIP1	hsa-miR-7257-5p
CYFIP1	hsa-miR-7171-5p
CYFIP1	hsa-let-7e-3p
CYFIP1	hsa-miR-519e-5p
CYFIP1	hsa-miR-4502
CYFIP1	hsa-miR-26a-3p
CYFIP1	hsa-miR-3604
CYFIP1	hsa-miR-6778-3p
CYFIP1	hsa-miR-676-5p
CYFIP1	hsa-miR-410-3p
CYFIP1	hsa-miR-5098
CYFIP1	hsa-miR-2284-5p
CYFIP1	hsa-miR-7207-5p
CYFIP1	hsa-miR-6801-3p
CYFIP1	hsa-miR-670-5p
CYFIP1	hsa-miR-369-3p
CYFIP1	hsa-miR-222-5p
CYFIP1	hsa-miR-4518
CYFIP1	hsa-miR-499b-3p
CYFIP1	hsa-miR-6724-5p
CYFIP1	hsa-miR-1-2-5p
CYFIP1	hsa-miR-707
CYFIP1	hsa-miR-507b
CYFIP1	hsa-miR-128

CYFIP1	hsa-miR-15c-3p
CYFIP1	hsa-miR-2480
CYFIP1	hsa-miR-205-5p
CYFIP1	hsa-miR-6773-5p
CYFIP1	hsa-miR-28-5p
CYFIP1	hsa-miR-126a-5p
CYFIP1	hsa-miR-34
CYFIP1	hsa-miR-669bf-3p
CYFIP1	hsa-miR-409
CYFIP1	hsa-miR-8819
CYFIP1	hsa-miR-374-5p
CYFIP1	hsa-miR-365-3p
CYFIP1	hsa-miR-1238-5p
CYFIP1	hsa-miR-4778-5p
CYFIP1	hsa-miR-548f-3p
CYFIP1	hsa-miR-1298
CYFIP1	hsa-miR-3529-5p
CYFIP1	hsa-miR-1233-3p
CYFIP1	hsa-miR-875
CYFIP1	hsa-miR-133cd-5p
CYFIP1	hsa-miR-204-3p
CYFIP1	hsa-miR-6894-3p
CYFIP1	hsa-miR-9-3p
CYFIP1	hsa-miR-4310
CYFIP1	hsa-miR-5619-5p
CYFIP1	hsa-miR-4653-5p
CYFIP1	hsa-miR-361-3p
CYFIP1	hsa-miR-1304-3p
CYFIP1	hsa-miR-6758-5p
CYFIP1	hsa-miR-130-3p
CYFIP1	hsa-miR-4501
CYFIP1	hsa-miR-660-3p
CYFIP1	hsa-miR-5186
CYFIP1	hsa-miR-2861
CYFIP1	hsa-miR-101b-3p.1
CYFIP1	hsa-miR-6927-3p
CYFIP1	hsa-miR-2393
CYFIP1	hsa-miR-743-5p
CYFIP1	hsa-miR-6848-3p
CYFIP1	hsa-miR-7162-5p
CYFIP1	hsa-miR-669c-5p
CYFIP1	hsa-miR-1294
CYFIP1	hsa-miR-4763-5p
CYFIP1	hsa-miR-143-3p
CYFIP1	hsa-miR-4439
CYFIP1	hsa-miR-6871-3p

CYFIP1	hsa-miR-3184-3p
CYFIP1	hsa-miR-6729-3p
CYFIP1	hsa-miR-4474-5p
CYFIP1	hsa-miR-3143
CYFIP1	hsa-miR-6874-5p
CYFIP1	hsa-miR-1207-3p
CYFIP1	hsa-miR-1582
CYFIP1	hsa-miR-101-5p
CYFIP1	hsa-miR-6814-5p
CYFIP1	hsa-miR-2310
CYFIP1	hsa-miR-7241-3p
CYFIP1	hsa-miR-582-5p
CYFIP1	hsa-miR-649
CYFIP1	hsa-miR-1298-5p
CYFIP1	hsa-miR-7286-3p
CYFIP1	hsa-miR-101-3p
CYFIP1	hsa-miR-3600
CYFIP1	hsa-miR-4287
CYFIP1	hsa-miR-2337
CYFIP1	hsa-miR-202-5p
CYFIP1	hsa-miR-4792
CYFIP1	hsa-miR-495-3p
CYFIP1	hsa-miR-579-3p
CYFIP1	hsa-miR-4755-3p
CYFIP1	hsa-miR-3689d
CYFIP1	hsa-miR-547-5p
CYFIP1	hsa-miR-664-3p
CYFIP1	hsa-miR-7333-3p
CYFIP1	hsa-miR-3532-5p
CYFIP1	hsa-miR-494
CYFIP1	hsa-miR-24-3p
CYFIP1	hsa-miR-4495
CYFIP1	hsa-miR-6790-5p
CYFIP1	hsa-miR-376a-5p
CYFIP1	hsa-let-7c-3p
CYFIP1	hsa-miR-2473
CYFIP1	hsa-miR-497-5p
CYFIP1	hsa-miR-490-5p
CYFIP1	hsa-miR-7673-3p
CYFIP1	hsa-let-7abf-3p
CYFIP1	hsa-miR-143-5p
CYFIP1	hsa-miR-513a-3p
CYFIP1	hsa-miR-7156-3p
CYFIP1	hsa-miR-2285b
CYFIP1	hsa-miR-8825
CYFIP1	hsa-miR-6779-3p

CYFIP1	hsa-miR-7118-3p
CYFIP1	hsa-miR-330-3p.2
CYFIP1	hsa-miR-6928-3p
CYFIP1	hsa-miR-2482
CYFIP1	hsa-miR-514b-5p
CYFIP1	hsa-miR-200-3p
CYFIP1	hsa-miR-7238-3p
CYFIP1	hsa-miR-8908f
CYFIP1	hsa-miR-4679
CYFIP1	hsa-miR-4684-3p
CYFIP1	hsa-miR-5008-3p
CYFIP1	hsa-miR-1271-5p
CYFIP1	hsa-miR-6924-3p
CYFIP1	hsa-miR-155-5p
CYFIP1	hsa-miR-450b-5p
CYFIP1	hsa-miR-183-3p
CYFIP1	hsa-miR-1271-3p
CYFIP1	hsa-miR-2284ab
CYFIP1	hsa-miR-3681-5p
CYFIP1	hsa-miR-19
CYFIP1	hsa-miR-1285-5p
CYFIP1	hsa-miR-196a-3p
CYFIP1	hsa-miR-676-3p
CYFIP1	hsa-miR-5092
CYFIP1	hsa-miR-7031-5p
CYFIP1	hsa-miR-8820
CYFIP1	hsa-miR-6403
CYFIP1	hsa-miR-181a-1-3p
CYFIP1	hsa-miR-7664-5p
CYFIP1	hsa-miR-7202-3p
CYFIP1	hsa-miR-3910
CYFIP1	hsa-miR-1179
CYFIP1	hsa-miR-4422
CYFIP1	hsa-miR-376c-5p
CYFIP1	hsa-miR-3079-5p
CYFIP1	hsa-miR-202-3p
CYFIP1	hsa-miR-5701
CYFIP1	hsa-miR-2307
CYFIP1	hsa-miR-344-3p
CYFIP1	hsa-miR-4328
CYFIP1	hsa-miR-7059-3p
CYFIP1	hsa-miR-4691-5p
CYFIP1	hsa-miR-2293
CYFIP1	hsa-miR-4658
CYFIP1	hsa-miR-217-3p
CYFIP1	hsa-miR-513a

CYFIP1	hsa-miR-508a
CYFIP1	hsa-miR-6836-3p
CYFIP1	hsa-miR-376b-5p
CYFIP1	hsa-miR-7162-3p
CYFIP1	hsa-miR-100-3p
CYFIP1	hsa-miR-876
CYFIP1	hsa-miR-3613-3p
CYFIP1	hsa-miR-5624-5p
CYFIP1	hsa-miR-4746-5p
CYFIP1	hsa-miR-374b-3p
CYFIP1	hsa-miR-6972-3p
CYFIP1	hsa-miR-8058
CYFIP1	hsa-miR-3914
CYFIP1	hsa-miR-7078-5p
CYFIP1	hsa-miR-1260a
CYFIP1	hsa-miR-192-5p
CYFIP1	hsa-miR-1236-5p
CYFIP1	hsa-miR-7284-3p
CYFIP1	hsa-miR-381-3p
CYFIP1	hsa-miR-219c-3p
CYFIP1	hsa-miR-4782-5p
CYFIP1	hsa-miR-526
CYFIP1	hsa-miR-5586-5p
CYFIP1	hsa-miR-1292-5p
CYFIP1	hsa-miR-6944-5p
CYFIP1	hsa-miR-4773
CYFIP1	hsa-miR-7386i-3p
CYFIP1	hsa-miR-30e-3p
CYFIP1	hsa-miR-351-5p
CYFIP1	hsa-miR-211-5p
CYFIP1	hsa-miR-576-5p
CYFIP1	hsa-miR-1-3p
CYFIP1	hsa-miR-7307-3p
CYFIP1	hsa-miR-1185-5p
CYFIP1	hsa-miR-7193-3p
CYFIP1	hsa-miR-6747-3p
CYFIP1	hsa-miR-6751-3p
CYFIP1	hsa-miR-6126
CYFIP1	hsa-miR-880-5p
CYFIP1	hsa-miR-224-5p
CYFIP1	hsa-miR-3072-3p
CYFIP1	hsa-miR-214-3p
CYFIP1	hsa-miR-411-3p
CYFIP1	hsa-miR-203
CYFIP1	hsa-miR-1260b
CYFIP1	hsa-miR-6938-3p

CYFIP1	hsa-miR-6720-5p
CYFIP1	hsa-miR-7977
CYFIP1	hsa-miR-8842
CYFIP1	hsa-miR-3619-5p
CYFIP1	hsa-miR-2486-3p
CYFIP1	hsa-miR-4660
CYFIP1	hsa-miR-6949-3p
CYFIP1	hsa-miR-1184
CYFIP1	hsa-miR-371b-5p
CYFIP1	hsa-miR-6335
CYFIP1	hsa-miR-8821
CYFIP1	hsa-miR-7200-3p
CYFIP1	hsa-miR-509-3p
CYFIP1	hsa-miR-8069
CYFIP1	hsa-miR-6877-3p
CYFIP1	hsa-miR-373-5p
CYFIP1	hsa-miR-518g-3p
CYFIP1	hsa-miR-4685-3p
CYFIP1	hsa-miR-499-5p
CYFIP1	hsa-miR-518-5p
CYFIP1	hsa-miR-29a-5p
CYFIP1	hsa-miR-5787
CYFIP1	hsa-miR-29a-3p
CYFIP1	hsa-miR-488-3p
CYFIP1	hsa-miR-6329
CYFIP1	hsa-miR-196c-3p
CYFIP1	hsa-miR-4727-3p
CYFIP1	hsa-miR-6500-3p
CYFIP1	hsa-miR-136-5p
CYFIP1	hsa-miR-101b-3p.2
CYFIP1	hsa-let-7c-1-3p
CYFIP1	hsa-miR-8877
CYFIP1	hsa-miR-5007-3p
CYFIP1	hsa-miR-429
CYFIP1	hsa-miR-629-3p
CYFIP1	hsa-miR-7215-5p
CYFIP1	hsa-miR-2319
CYFIP1	hsa-miR-98-3p
CYFIP1	hsa-miR-4267
CYFIP1	hsa-miR-3074-5p
CYFIP1	hsa-miR-6081
CYFIP1	hsa-miR-497-3p
CYFIP1	hsa-miR-1191b-3p
CYFIP1	hsa-miR-627-3p
CYFIP1	hsa-miR-6918-5p
CYFIP1	hsa-miR-2284d

CYFIP1	hsa-miR-6792-3p
CYFIP1	hsa-miR-378j
CYFIP1	hsa-miR-425
CYFIP1	hsa-miR-101-3p.2
CYFIP1	hsa-miR-7210-5p
CYFIP1	hsa-miR-1183
CYFIP1	hsa-miR-5696
CYFIP1	hsa-miR-3473f
CYFIP1	hsa-miR-136-5p
CYFIP1	hsa-miR-6072
CYFIP1	hsa-miR-433-3p
CYFIP1	hsa-miR-2284c
CYFIP1	hsa-miR-1787
CYFIP1	hsa-miR-6414
CYFIP1	hsa-miR-4795-3p
CYFIP1	hsa-miR-7269-5p
CYFIP1	hsa-miR-3121-3p
CYFIP1	hsa-miR-7086-5p
CYFIP1	hsa-miR-2335
CYFIP1	hsa-miR-466b-5p
CYFIP1	hsa-miR-1933-5p
CYFIP1	hsa-miR-6122-3p
CYFIP1	hsa-miR-944
CYFIP1	hsa-miR-129-2-3p
CYFIP1	hsa-miR-370-5p
CYFIP1	hsa-miR-3907
CYFIP1	hsa-miR-6343
CYFIP1	hsa-miR-7234-5p
CYFIP1	hsa-miR-299-5p
CYFIP1	hsa-miR-3068-3p
CYFIP1	hsa-miR-600
CYFIP1	hsa-miR-4724-5p
CYFIP1	hsa-miR-133c
CYFIP1	hsa-miR-6892-5p
CYFIP1	hsa-miR-7249-3p
CYFIP1	hsa-miR-8485
CYFIP1	hsa-miR-3691-5p
CYFIP1	hsa-miR-7159-3p
CYFIP1	hsa-miR-29b-5p
CYFIP1	hsa-miR-106a-3p
CYFIP1	hsa-miR-6134
CYFIP1	hsa-miR-6811-3p
CYFIP1	hsa-miR-1207
CYFIP1	hsa-miR-7849-3p
CYFIP1	hsa-miR-335-5p
CYFIP1	hsa-miR-29b-2-5p

CYFIP1	hsa-miR-18-3p
CYFIP1	hsa-miR-1322
CYFIP1	hsa-miR-200c-3p
CYFIP1	hsa-miR-2400
CYFIP1	hsa-miR-655
CYFIP1	hsa-miR-6750-5p
CYFIP1	hsa-miR-6735-5p
CYFIP1	hsa-miR-4762-3p
CYFIP1	hsa-miR-137
CYFIP1	hsa-miR-2303
CYFIP1	hsa-miR-4663
CYFIP1	hsa-miR-6891-3p
CYFIP1	hsa-miR-6757-5p
CYFIP1	hsa-miR-4766-5p
CYFIP1	hsa-miR-6762-3p
CYFIP1	hsa-miR-8791
CYFIP1	hsa-miR-1912
CYFIP1	hsa-miR-466b-3p
CYFIP1	hsa-miR-8829
CYFIP1	hsa-miR-7373b-3p
CYFIP1	hsa-miR-486-5p
CYFIP1	hsa-miR-4662a-3p
CYFIP1	hsa-miR-3915
CYFIP1	hsa-miR-374a-5p
CYFIP1	hsa-miR-7652-3p
CYFIP1	hsa-miR-7230-3p
CYFIP1	hsa-miR-7217-5p
CYFIP1	hsa-miR-2113
CYFIP1	hsa-miR-6572-5p
CYFIP1	hsa-miR-6821-3p
CYFIP1	hsa-miR-326
CYFIP1	hsa-miR-2681-5p
CYFIP1	hsa-miR-185-5p
CYFIP1	hsa-miR-323
CYFIP1	hsa-miR-2369
CYFIP1	hsa-miR-548av-3p
CYFIP1	hsa-miR-302a-3p
CYFIP1	hsa-miR-3163
CYFIP1	hsa-miR-1272
CYFIP1	hsa-miR-616-5p
CYFIP1	hsa-miR-6896-3p
CYFIP1	hsa-miR-508-5p
CYFIP1	hsa-miR-137-3p
CYFIP1	hsa-miR-194-5p
CYFIP1	hsa-miR-347
CYFIP1	hsa-miR-215-5p

CYFIP1	hsa-miR-6320
CYFIP1	hsa-miR-659-3p
CYFIP1	hsa-miR-1298-3p
CYFIP1	hsa-miR-4711-5p
CYFIP1	hsa-miR-1273f
CYFIP1	hsa-miR-655-5p
CYFIP1	hsa-miR-591
CYFIP1	hsa-miR-519e-3p
CYFIP1	hsa-miR-411a
CYFIP1	hsa-miR-6376
CYFIP1	hsa-miR-702-3p
CYFIP1	hsa-miR-1264
CYFIP1	hsa-miR-379-5p
CYFIP1	hsa-miR-105-5p
CYFIP1	hsa-miR-698-3p
CYFIP1	hsa-miR-3144-3p
CYFIP1	hsa-miR-1290
CYFIP1	hsa-miR-1964-5p
CYFIP1	hsa-miR-7324-5p
CYFIP1	hsa-miR-5700
CYFIP1	hsa-miR-4747-5p
CYFIP1	hsa-miR-7006-5p
CYFIP1	hsa-miR-7190-5p
CYFIP1	hsa-miR-874-3p
CYFIP1	hsa-miR-6843-3p
CYFIP1	hsa-miR-30-3p
CYFIP1	hsa-miR-4774-3p
CYFIP1	hsa-miR-1556
CYFIP1	hsa-miR-766-3p
CYFIP1	hsa-miR-3614-5p
CYFIP1	hsa-miR-2373-5p
CYFIP1	hsa-miR-922
CYFIP1	hsa-miR-4680
CYFIP1	hsa-miR-4804-3p
CYFIP1	hsa-miR-2292
CYFIP1	hsa-miR-101-3p.1
CYFIP1	hsa-miR-4448
CYFIP1	hsa-miR-2382-3p
CYFIP1	hsa-miR-30b-3p
CYFIP1	hsa-miR-4780
CYFIP1	hsa-miR-7386g-3p
CYFIP1	hsa-miR-7364-5p
CYFIP1	hsa-miR-2114-5p
CYFIP1	hsa-miR-6960-5p
CYFIP1	hsa-miR-5697-3p
CYFIP1	hsa-miR-6330

CYFIP1	hsa-miR-421
CYFIP1	hsa-miR-6344
CYFIP1	hsa-miR-4261
CYFIP1	hsa-miR-7119-5p
CYFIP1	hsa-miR-3152-5p
CYFIP1	hsa-miR-3102
CYFIP1	hsa-miR-584-5p
CYFIP1	hsa-miR-769
CYFIP1	hsa-miR-190a-3p
CYFIP1	hsa-miR-367-5p
CYFIP1	hsa-miR-150-5p
CYFIP1	hsa-miR-8874
CYFIP1	hsa-miR-28-5p
CYFIP1	hsa-miR-7186-3p
CYFIP1	hsa-miR-7016-3p
CYFIP1	hsa-miR-541-5p
CYFIP1	hsa-miR-1542-3p
CYFIP1	hsa-miR-742-3p
CYFIP1	hsa-miR-330-3p
CYFIP1	hsa-miR-675-3p
CYFIP1	hsa-miR-105b
CYFIP1	hsa-miR-6939-3p
CYFIP1	hsa-miR-1914-5p
CYFIP1	hsa-miR-506-3p
CYFIP1	hsa-miR-4788
CYFIP1	hsa-miR-3174
CYFIP1	hsa-miR-5626-5p
CYFIP1	hsa-miR-8831
CYFIP1	hsa-miR-7192-3p
CYFIP1	hsa-miR-3162-5p
CYFIP1	hsa-miR-146
CYFIP1	hsa-miR-7649-3p
CYFIP1	hsa-miR-2299-5p
CYFIP1	hsa-miR-1-5p
CYFIP1	hsa-miR-9768-3p
CYFIP1	hsa-miR-1843a-5p
CYFIP1	hsa-miR-4753-3p
CYFIP1	hsa-miR-569
CYFIP1	hsa-miR-6850-5p
CYFIP1	hsa-miR-448-3p
CYFIP1	hsa-miR-369-3p
CYFIP1	hsa-miR-135-5p
CYFIP1	hsa-miR-3927-3p
CYFIP1	hsa-miR-4645-3p
CYFIP1	hsa-miR-434-3p
CYFIP1	hsa-miR-378-3p

CYFIP1	hsa-miR-181c-3p
CYFIP1	hsa-miR-101c
CYFIP1	hsa-miR-146b-3p
CYFIP1	hsa-miR-6951-3p
CYFIP1	hsa-miR-6350
CYFIP1	hsa-miR-1837
CYFIP1	hsa-miR-433
CYFIP1	hsa-miR-4514
CYFIP1	hsa-miR-6951-5p
CYFIP1	hsa-miR-141-5p
CYFIP1	hsa-miR-2429
CYFIP1	hsa-miR-527
CYFIP1	hsa-miR-342-3p
CYFIP1	hsa-miR-4282
CYFIP1	hsa-miR-3613-5p
CYFIP1	hsa-miR-5113
CYFIP1	hsa-miR-5004-5p
CYFIP1	hsa-miR-3618-5p
CYFIP1	hsa-miR-7241-5p
CYFIP1	hsa-miR-218-5p
CYFIP1	hsa-miR-7371f-5p
CYFIP1	hsa-miR-193
CYFIP1	hsa-miR-5002-5p
CYFIP1	hsa-miR-761
CYFIP1	hsa-miR-4305
CYFIP1	hsa-miR-636
CYFIP1	hsa-miR-299-3p
CYFIP1	hsa-miR-103
CYFIP1	hsa-miR-6078
CYFIP1	hsa-miR-7398r-3p
CYFIP1	hsa-miR-517
CYFIP1	hsa-miR-2116-3p
CYFIP1	hsa-miR-7450-5p
CYFIP1	hsa-miR-548b-3p
CYFIP1	hsa-miR-203-5p
CYFIP1	hsa-miR-568
CYFIP1	hsa-miR-6853-5p
CYFIP1	hsa-miR-7854-3p
CYFIP1	hsa-miR-651
CYFIP1	hsa-miR-7334-3p
CYFIP1	hsa-miR-3185
CYFIP1	hsa-miR-6891-5p
CYFIP1	hsa-miR-7166-5p
CYFIP1	hsa-miR-374-3p
CYFIP1	hsa-miR-6943-3p
CYFIP1	hsa-miR-3140-3p

CYFIP1	hsa-miR-3573-3p
CYFIP1	hsa-miR-6537-5p
CYFIP1	hsa-miR-698-5p
CYFIP1	hsa-miR-8799
CYFIP1	hsa-miR-7036b-3p
CYFIP1	hsa-miR-3576
CYFIP1	hsa-miR-6832-3p
CYFIP1	hsa-miR-4797-5p
CYFIP1	hsa-miR-302d-5p
CYFIP1	hsa-miR-6930-3p
CYFIP1	hsa-miR-6079
CYFIP1	hsa-miR-668-3p
CYFIP1	hsa-miR-4513
CYFIP1	hsa-miR-7069-3p
CYFIP1	hsa-miR-643
CYFIP1	hsa-miR-2957
CYFIP1	hsa-miR-496-3p
CYFIP1	hsa-miR-3927-3p
CYFIP1	hsa-miR-6514-3p
CYFIP1	hsa-miR-135b-3p
CYFIP1	hsa-miR-3561-5p
CYFIP1	hsa-miR-6938-5p
CYFIP1	hsa-miR-1198-5p
CYFIP1	hsa-miR-21-3p
CYFIP1	hsa-miR-598-5p
CYFIP1	hsa-miR-613
CYFIP1	hsa-miR-6881-3p
CYFIP1	hsa-miR-4520-5p
CYFIP1	hsa-miR-211-5p
CYFIP1	hsa-miR-302c-3p
CYFIP1	hsa-miR-513c-3p
CYFIP1	hsa-miR-3961
CYFIP1	hsa-let-7a-3p
CYFIP1	hsa-miR-7112-3p
CYFIP1	hsa-miR-8809
CYFIP1	hsa-miR-4445-5p
CYFIP1	hsa-miR-3109-3p
CYFIP1	hsa-miR-3173-3p
CYFIP1	hsa-miR-6941-3p
CYFIP1	hsa-miR-7045-3p
CYFIP1	hsa-miR-132-3p
CYFIP1	hsa-miR-485
CYFIP1	hsa-miR-6889-3p
CYFIP1	hsa-miR-1282
CYFIP1	hsa-miR-203-3p.2
CYFIP1	hsa-miR-2421

CYFIP1	hsa-miR-8800
CYFIP1	hsa-miR-7398l-3p
CYFIP1	hsa-miR-331-5p
CYFIP1	hsa-miR-1795
CYFIP1	hsa-miR-7641
CYFIP1	hsa-miR-3958
CYFIP1	hsa-miR-16-1-3p
CYFIP1	hsa-miR-5695
CYFIP1	hsa-miR-7093-3p
CYFIP1	hsa-miR-1224-5p
CYFIP1	hsa-miR-4526
CYFIP1	hsa-miR-489-5p
CYFIP1	hsa-miR-7674-3p
CYFIP1	hsa-miR-1264-3p
CYFIP1	hsa-miR-1a-5p
CYFIP1	hsa-miR-8104
CYFIP1	hsa-miR-96-3p
CYFIP1	hsa-miR-7464-5p
CYFIP1	hsa-miR-6524
CYFIP1	hsa-miR-8846
CYFIP1	hsa-miR-4678
CYFIP1	hsa-miR-154a
CYFIP1	hsa-miR-3607-5p
CYFIP1	hsa-miR-590-3p
CYFIP1	hsa-miR-2399-5p
CYFIP1	hsa-miR-548c-3p
CYFIP1	hsa-miR-93-3p
CYFIP1	hsa-miR-6822-5p
CYFIP1	hsa-miR-675-5p
CYFIP1	hsa-miR-891
CYFIP1	hsa-miR-4530
CYFIP1	hsa-miR-7856-5p
CYFIP1	hsa-miR-7702
CYFIP1	hsa-miR-3130-5p
CYFIP1	hsa-miR-200c-5p
CYFIP1	hsa-miR-183-5p
CYFIP1	hsa-miR-582-5p
CYFIP1	hsa-miR-6346
CYFIP1	hsa-miR-151-3p
CYFIP1	hsa-miR-597-5p
CYFIP1	hsa-miR-323b-3p
CYFIP1	hsa-miR-17-3p
CYFIP1	hsa-miR-208-3p
CYFIP1	hsa-miR-6517
CYFIP1	hsa-miR-5687
CYFIP1	hsa-miR-587

CYFIP1	hsa-miR-1243-3p
CYFIP1	hsa-miR-6957-3p
CYFIP1	hsa-miR-7398a-3p
CYFIP1	hsa-miR-466mo-3p
CYFIP1	hsa-miR-519-5p
CYFIP1	hsa-miR-2284x
CYFIP1	hsa-miR-510
CYFIP1	hsa-miR-3121-5p
CYFIP1	hsa-miR-7382-3p
CYFIP1	hsa-miR-3108-5p
CYFIP1	hsa-miR-3120-3p
CYFIP1	hsa-miR-5118
CYFIP1	hsa-miR-7236-5p
CYFIP1	hsa-miR-6802-3p
CYFIP1	hsa-miR-3145-3p
CYFIP1	hsa-miR-103-5p
CYFIP1	hsa-miR-299a-5p
CYFIP1	hsa-miR-6878-3p
CYFIP1	hsa-let-7f-1-3p
CYFIP1	hsa-miR-516b
CYFIP1	hsa-miR-584-3p
CYFIP1	hsa-miR-1-3p
CYFIP1	hsa-miR-6758-3p
CYFIP1	hsa-miR-3586-5p
CYFIP1	hsa-miR-2483-3p
CYFIP1	hsa-miR-1262-5p
CYFIP1	hsa-miR-700-5p
CYFIP1	hsa-miR-148-3p
CYFIP1	hsa-miR-7-5p
CYFIP1	hsa-miR-219b-5p
CYFIP1	hsa-miR-2485
CYFIP1	hsa-miR-7340-3p
CYFIP1	hsa-miR-7398m-3p
CYFIP1	hsa-miR-1-1-5p
CYFIP1	hsa-miR-218-5p
CYFIP1	hsa-miR-758-3p
CYFIP1	hsa-miR-7398u-3p
CYFIP1	hsa-miR-195-3p
CYFIP1	hsa-miR-4652-3p
CYFIP1	hsa-let-7b-3p
CYFIP1	hsa-miR-4695-3p
CYFIP1	hsa-miR-770-3p
CYFIP1	hsa-miR-6565-3p
CYFIP1	hsa-miR-6131
CYFIP1	hsa-miR-495-3p
CYFIP1	hsa-miR-6828-5p

CYFIP1	hsa-miR-3560
CYFIP1	hsa-miR-5003-5p
CYFIP1	hsa-miR-6829-3p
CYFIP1	hsa-miR-6510-5p
CYFIP1	hsa-miR-93-5p
CYFIP1	hsa-miR-3572-3p
CYFIP1	hsa-miR-7157-5p
CYFIP1	hsa-miR-7398b-3p
CYFIP1	hsa-miR-6919-5p
CYFIP1	hsa-miR-1224-3p
CYFIP1	hsa-miR-2285x
CYFIP1	hsa-miR-1973
CYFIP1	hsa-miR-29
CYFIP1	hsa-miR-9b-3p
CYFIP1	hsa-miR-33-5p
CYFIP1	hsa-miR-152-3p
CYFIP1	hsa-miR-516a-3p
CYFIP1	hsa-miR-717
CYFIP1	hsa-miR-668
CYFIP1	hsa-miR-6827-5p
CYFIP1	hsa-miR-3192
CYFIP1	hsa-miR-2285u
CYFIP1	hsa-miR-4475
CYFIP1	hsa-miR-6910-5p
CYFIP1	hsa-miR-107-3p
CYFIP1	hsa-miR-203b-3p
CYFIP1	hsa-miR-30-5p
CYFIP1	hsa-miR-3065-5p
CYFIP1	hsa-miR-4640-3p
CYFIP1	hsa-miR-6823-5p
CYFIP1	hsa-miR-4279
CYFIP1	hsa-miR-590-5p
CYFIP1	hsa-miR-6764-5p
CYFIP1	hsa-miR-7052-3p
CYFIP1	hsa-miR-466dk-5p
CYFIP1	hsa-miR-8884
CYFIP1	hsa-miR-694
CYFIP1	hsa-miR-6516-3p
CYFIP1	hsa-miR-3652
CYFIP1	hsa-miR-889-3p
CYFIP1	hsa-miR-6839-5p
CYFIP1	hsa-miR-6988-3p
CYFIP1	hsa-miR-381-3p
CYFIP1	hsa-miR-5000-5p
CYFIP1	hsa-miR-7455-3p
CYFIP1	hsa-miR-2285j

CYFIP1	hsa-miR-143-3p
CYFIP1	hsa-miR-1752
CYFIP1	hsa-miR-2353
CYFIP1	hsa-miR-1283a
CYFIP1	hsa-miR-7440-3p
CYFIP1	hsa-miR-548ba
CYFIP1	hsa-miR-6866-5p
CYFIP1	hsa-miR-4471
CYFIP1	hsa-miR-432-3p
CYFIP1	hsa-miR-6124
CYFIP1	hsa-miR-8059
CYFIP1	hsa-miR-7178-5p
CYFIP1	hsa-miR-550a-3p
CYFIP1	hsa-miR-7094-3p
CYFIP1	hsa-miR-340-5p
CYFIP1	hsa-miR-2325b
CYFIP1	hsa-miR-513c-5p
CYFIP1	hsa-miR-8076
CYFIP1	hsa-miR-16-3p
CYFIP1	hsa-miR-5088-3p
CYFIP1	hsa-miR-5196-3p
CYFIP1	hsa-miR-1266
CYFIP1	hsa-miR-4256
CYFIP1	hsa-miR-502a
CYFIP1	hsa-miR-4775
CYFIP1	hsa-miR-1805-3p
CYFIP1	hsa-miR-204-5p
CYFIP1	hsa-miR-190b-3p
CYFIP1	hsa-miR-553
CYFIP1	hsa-miR-3167-3p
CYFIP1	hsa-miR-216-3p
CYFIP1	hsa-miR-196
CYFIP1	hsa-miR-4713-5p
CYFIP1	hsa-miR-6867-3p
CYFIP1	hsa-miR-339-5p
CYFIP1	hsa-miR-6507-5p
CYFIP1	hsa-miR-548az-5p
CYFIP1	hsa-miR-548au-3p
CYFIP1	hsa-miR-6991-5p
CYFIP1	hsa-miR-5009-3p
CYFIP1	hsa-miR-7646-3p
CYFIP1	hsa-miR-7206-5p
CYFIP1	hsa-miR-7445-5p
CYFIP1	hsa-miR-576-5p
CYFIP1	hsa-miR-7670-5p
CYFIP1	hsa-miR-8904b

CYFIP1	hsa-miR-372-5p
CYFIP1	hsa-miR-561-3p
CYFIP1	hsa-miR-6855-3p
CYFIP1	hsa-miR-7114-5p
CYFIP1	hsa-miR-1225-3p
CYFIP1	hsa-miR-8906
CYFIP1	hsa-miR-874-5p
CYFIP1	hsa-miR-194-5p
CYFIP1	hsa-miR-215
CYFIP1	hsa-miR-3938-5p
CYFIP1	hsa-miR-16-2-3p
CYFIP1	hsa-miR-1264-5p
CYFIP1	hsa-miR-148a-5p
CYFIP1	hsa-miR-145a-3p
CYFIP1	hsa-miR-880-3p
CYFIP1	hsa-miR-140-5p
CYFIP1	hsa-miR-346-3p
CYFIP1	hsa-miR-224-5p
CYFIP1	hsa-miR-4477a
CYFIP1	hsa-miR-465c-5p
CYFIP1	hsa-miR-6317
CYFIP1	hsa-miR-7243-5p
CYFIP1	hsa-miR-1301-3p
CYFIP1	hsa-miR-197-3p
CYFIP1	hsa-miR-1202
CYFIP1	hsa-miR-16-5p
CYFIP1	hsa-miR-6831-5p
CYFIP1	hsa-miR-125b-2-3p
CYFIP1	hsa-miR-125
CYFIP1	hsa-miR-6941-5p
CYFIP1	hsa-miR-1199-5p
CYFIP1	hsa-miR-7292-5p
CYFIP1	hsa-miR-1299
CYFIP1	hsa-miR-17-5p
CYFIP1	hsa-miR-6499-3p
CYFIP1	hsa-miR-576-3p
CYFIP1	hsa-miR-3133
CYFIP1	hsa-miR-101-1-5p
CYFIP1	hsa-miR-1915-5p
CYFIP1	hsa-miR-7290-5p
CYFIP1	hsa-miR-451-3p
CYFIP1	hsa-miR-891-3p
CYFIP1	hsa-miR-345-3p
CYFIP1	hsa-miR-34-5p
CYFIP1	hsa-miR-1205
CYFIP1	hsa-miR-2909

CYFIP1	hsa-miR-3664-3p
CYFIP1	hsa-miR-672-3p
CYFIP1	hsa-miR-7024-5p
CYFIP1	hsa-miR-2428
CYFIP1	hsa-miR-7297-3p
CYFIP1	hsa-miR-6515-3p
CYFIP1	hsa-miR-6944-3p
CYFIP1	hsa-miR-146-5p
CYFIP1	hsa-miR-6903-3p
CYFIP1	hsa-miR-1287-5p
CYFIP1	hsa-miR-2426
CYFIP1	hsa-miR-206-5p
CYFIP1	hsa-miR-200bc
CYFIP1	hsa-miR-1252
CYFIP1	hsa-miR-22-3p
CYFIP1	hsa-miR-597
CYFIP1	hsa-miR-483-3p.2
CYFIP1	hsa-miR-4722-3p
CYFIP1	hsa-miR-384
CYFIP1	hsa-miR-8838
CYFIP1	hsa-miR-370-3p
CYFIP1	hsa-miR-670
CYFIP1	hsa-miR-6948-3p
CYFIP1	hsa-miR-4424
CYFIP1	hsa-miR-6377
CYFIP1	hsa-let-7d-3p
CYFIP1	hsa-miR-4729
CYFIP1	hsa-miR-6858-3p
CYFIP1	hsa-miR-7314-5p
CYFIP1	hsa-miR-374c-5p
CYFIP1	hsa-miR-3942-3p
CYFIP1	hsa-miR-34a-3p
CYFIP1	hsa-miR-7188-5p
CYFIP1	hsa-miR-1287
CYFIP1	hsa-miR-2284z
CYFIP1	hsa-miR-5621-5p
CYFIP1	hsa-miR-32-3p
CYFIP1	hsa-miR-1839
CYFIP1	hsa-miR-129b-5p
CYFIP1	hsa-miR-199b-3-5p
CYFIP1	hsa-miR-29b-1-5p
CYFIP1	hsa-miR-302c-5p
CYFIP1	hsa-miR-545-3p
CYFIP1	hsa-miR-6885-3p
CYFIP1	hsa-miR-296-5p
CYFIP1	hsa-miR-7473-5p

CYFIP1	hsa-miR-344b-3p
CYFIP1	hsa-miR-3591-5p
CYFIP1	hsa-miR-2284v
CYFIP1	hsa-miR-1811
CYFIP1	hsa-miR-454-3p
CYFIP1	hsa-miR-570-5p
CYFIP1	hsa-miR-323a-3p
CYFIP1	hsa-miR-7204-5p
CYFIP1	hsa-miR-3682-3p
CYFIP1	hsa-miR-219-5p
CYFIP1	hsa-miR-9769-3p
CYFIP1	hsa-miR-7178-3p
CYFIP1	hsa-miR-8065
CYFIP1	hsa-miR-551-5p
CYFIP1	hsa-miR-1250-3p
CYFIP1	hsa-miR-2326
CYFIP1	hsa-miR-379-3p
CYFIP1	hsa-miR-6872-3p
CYFIP1	hsa-miR-95-5p
CYFIP1	hsa-miR-4680-5p
CYFIP1	hsa-miR-2284w
CYFIP1	hsa-miR-548
CYFIP1	hsa-miR-7336-5p
CYFIP1	hsa-miR-3068-5p
CYFIP1	hsa-miR-7306-5p
CYFIP1	hsa-miR-1295b-5p
CYFIP1	hsa-miR-4716-5p
CYFIP1	hsa-miR-7157-3p
CYFIP1	hsa-miR-3135b
CYFIP1	hsa-miR-5002-3p
CYFIP1	hsa-miR-3596
CYFIP1	hsa-miR-532-3p
CYFIP1	hsa-miR-6849-3p
CYFIP1	hsa-miR-759
CYFIP1	hsa-miR-4760-5p
CYFIP1	hsa-miR-599
CYFIP1	hsa-miR-1255-3p
CYFIP1	hsa-miR-6912-5p
CYFIP1	hsa-miR-466bcp-3p
CYFIP1	hsa-miR-8804
CYFIP1	hsa-miR-589-3p
CYFIP1	hsa-miR-2114-5p
CYFIP1	hsa-miR-1271-5p
CYFIP1	hsa-miR-1178-3p
CYFIP1	hsa-miR-545-5p
CYFIP1	hsa-miR-4659a-5p

CYFIP1	hsa-miR-3095-5p
CYFIP1	hsa-miR-200a-3p
CYFIP1	hsa-miR-2115-3p
CYFIP1	hsa-miR-4666a-3p
CYFIP1	hsa-miR-743b-5p
CYFIP1	hsa-miR-7117-3p
CYFIP1	hsa-miR-671-5p
CYFIP1	hsa-miR-29a-3p
CYFIP1	hsa-miR-692
CYFIP1	hsa-miR-329-5p
CYFIP1	hsa-miR-182-5p
CYFIP1	hsa-miR-465a-5p
CYFIP1	hsa-miR-7457-3p
CYFIP1	hsa-miR-15-5p
CYFIP1	hsa-miR-103a-2-5p
CYFIP1	hsa-miR-3613b
CYFIP1	hsa-miR-6701-3p
CYFIP1	hsa-miR-4474-3p
CYFIP1	hsa-miR-1544-3p
CYFIP1	hsa-miR-3618-3p
CYFIP1	hsa-miR-708-3p
CYFIP1	hsa-miR-2334
CYFIP1	hsa-miR-6736-3p
CYFIP1	hsa-miR-7093-5p
CYFIP1	hsa-miR-7021-3p
CYFIP1	hsa-let-7ag-3p
CYFIP1	hsa-miR-139-5p
CYFIP1	hsa-miR-743b-3p
CYFIP1	hsa-miR-30ad-3p
CYFIP1	hsa-miR-380
CYFIP1	hsa-miR-7348-3p
CYFIP1	hsa-miR-373-3p
CYFIP1	hsa-miR-7116-3p
CYFIP1	hsa-miR-691
CYFIP1	hsa-miR-424-5p
CYFIP1	hsa-miR-543-3p
CYFIP1	hsa-miR-367-3p
CYFIP1	hsa-miR-422
CYFIP1	hsa-miR-6950-5p
CYFIP1	hsa-miR-1976
CYFIP1	hsa-miR-298
CYFIP1	hsa-miR-616-3p
CYFIP1	hsa-miR-186-5p
CYFIP1	hsa-miR-425-5p
CYFIP1	hsa-miR-3116
CYFIP1	hsa-miR-6854-5p

CYFIP1	hsa-miR-6967-3p
CYFIP1	hsa-miR-769-5p
CYFIP1	hsa-miR-196-3p
CYFIP1	hsa-miR-130a-5p
CYFIP1	hsa-miR-140-5p
CYFIP1	hsa-miR-5187-3p
CYFIP1	hsa-miR-5196-5p
CYFIP1	hsa-miR-216b-3p
CYFIP1	hsa-miR-3145-5p
CYFIP1	hsa-miR-4480
CYFIP1	hsa-miR-429-3p
CYFIP1	hsa-miR-4482-3p
CYFIP1	hsa-miR-2116-5p
CYFIP1	hsa-miR-7855-5p
CYFIP1	hsa-miR-5682
CYFIP1	hsa-miR-152-5p
CYFIP1	hsa-miR-4521
CYFIP1	hsa-miR-6769b-5p
CYFIP1	hsa-miR-375
CYFIP1	hsa-miR-7111-3p
CYFIP1	hsa-miR-6835-3p
CYFIP1	hsa-miR-1970
CYFIP1	hsa-miR-29-3p
CYFIP1	hsa-miR-548az-3p
CYFIP1	hsa-miR-5692bc
CYFIP1	hsa-miR-1934-5p
CYFIP1	hsa-miR-6889-5p
CYFIP1	hsa-miR-1243
CYFIP1	hsa-miR-7213-3p
CYFIP1	hsa-miR-654-3p
CYFIP1	hsa-miR-493-5p
CYFIP1	hsa-miR-6502-3p
CYFIP1	hsa-miR-6900-3p
CYFIP1	hsa-miR-6898-5p
CYFIP1	hsa-miR-7677-5p
CYFIP1	hsa-miR-3057-3p
CYFIP1	hsa-miR-2285n
CYFIP1	hsa-miR-871-3p
CYFIP1	hsa-miR-876-5p
CYFIP1	hsa-miR-4522
CYFIP1	hsa-miR-743a-3p
CYFIP1	hsa-miR-34bc-3p
CYFIP1	hsa-miR-1840
CYFIP1	hsa-miR-3065-3p
CYFIP1	hsa-miR-935
CYFIP1	hsa-let-7-3p

CYFIP1	hsa-miR-101a-3p.2
CYFIP1	hsa-miR-410
CYFIP1	hsa-miR-183-5p.1
CYFIP1	hsa-miR-4751
CYFIP1	hsa-miR-3660
CYFIP1	hsa-miR-362-3p
CYFIP1	hsa-miR-7221-5p
CYFIP1	hsa-miR-4659b-5p
CYFIP1	hsa-miR-672-5p
CYFIP1	hsa-miR-7023-5p
CYFIP1	hsa-miR-326-3p
CYFIP1	hsa-miR-101a-5p
CYFIP1	hsa-miR-487a
CYFIP1	hsa-miR-6899-3p
CYFIP1	hsa-miR-6516-5p
CYFIP1	hsa-miR-411-5p
CYFIP1	hsa-miR-7185-3p
CYFIP1	hsa-miR-4689
CYFIP1	hsa-miR-511-3p
CYFIP1	hsa-miR-712-5p
CYFIP1	hsa-miR-653-3p
CYFIP1	hsa-miR-186-3p
CYFIP1	hsa-miR-4520-3p
CYFIP1	hsa-miR-500
CYFIP1	hsa-miR-7169-5p
CYFIP1	hsa-miR-1266-5p
CYFIP1	hsa-miR-7046-3p
CYFIP1	hsa-miR-2392
CYFIP1	hsa-miR-6946-5p
CYFIP1	hsa-miR-201-5p
CYFIP1	hsa-miR-4670-3p
CYFIP1	hsa-miR-1254
CYFIP1	hsa-miR-134-5p
CYFIP1	hsa-miR-1233-3p
CYFIP1	hsa-miR-599-5p
CYFIP1	hsa-miR-7195-5p
CYFIP1	hsa-miR-548l
CYFIP1	hsa-miR-6790-3p
CYFIP1	hsa-miR-7287-5p
CYFIP1	hsa-miR-647
CYFIP1	hsa-miR-7161-3p
CYFIP1	hsa-miR-6844
CYFIP1	hsa-miR-187-5p
CYFIP1	hsa-miR-5580-5p
CYFIP1	hsa-miR-4291
CYFIP1	hsa-miR-424-5p

CYFIP1	hsa-miR-188-3p
CYFIP1	hsa-miR-4733-5p
CYFIP1	hsa-miR-4703-3p
CYFIP1	hsa-miR-3583-5p
CYFIP1	hsa-miR-375-3p
CYFIP1	hsa-miR-510-3p
CYFIP1	hsa-miR-1252-5p
CYFIP1	hsa-miR-6749-3p
CYFIP1	hsa-miR-7176-3p
CYFIP1	hsa-miR-7089-3p
CYFIP1	hsa-miR-298-5p
CYFIP1	hsa-miR-33a-3p
CYFIP1	hsa-miR-433-5p
CYFIP1	hsa-miR-1193-5p
CYFIP1	hsa-miR-8067
CYFIP1	hsa-miR-548e-3p
CYFIP1	hsa-miR-616-3p
CYFIP1	hsa-miR-107
CYFIP1	hsa-miR-653
CYFIP1	hsa-miR-421-5p
CYFIP1	hsa-miR-7109-3p
CYFIP1	hsa-miR-6737-3p
CYFIP1	hsa-miR-3097-3p
CYFIP1	hsa-miR-203a-3p.1
CYFIP1	hsa-miR-6511-3p
CYFIP1	hsa-miR-141-3p
CYFIP1	hsa-miR-127-5p
CYFIP1	hsa-miR-6905-3p
CYFIP1	hsa-miR-4482-5p
CYFIP1	hsa-miR-1955-5p
CYFIP1	hsa-miR-760-5p
CYFIP1	hsa-miR-6933-3p
CYFIP1	hsa-miR-544-5p
CYFIP1	hsa-miR-205-5p
CYFIP1	hsa-miR-3646
CYFIP1	hsa-miR-7347-3p
CYFIP1	hsa-miR-879-3p
CYFIP1	hsa-miR-3921
CYFIP1	hsa-miR-6739-5p
CYFIP1	hsa-miR-2682-3p
CYFIP1	hsa-miR-6842-3p
CYFIP1	hsa-miR-548a-3p
CYFIP1	hsa-miR-146a-3p
CYFIP1	hsa-miR-4680-3p
CYFIP1	hsa-miR-3190-3p
CYFIP1	hsa-miR-5187-5p

CYFIP1	hsa-miR-3472
CYFIP1	hsa-miR-192-3p
CYFIP1	hsa-miR-3110-5p
CYFIP1	hsa-miR-31-5p
CYFIP1	hsa-miR-153
CYFIP1	hsa-miR-741-3p
CYFIP1	hsa-miR-875-5p
CYFIP1	hsa-miR-6847-3p
CYFIP1	hsa-miR-6910-3p
CYFIP1	hsa-miR-6781-3p
CYFIP1	hsa-miR-605-3p
CYFIP1	hsa-miR-6809-5p
CYFIP1	hsa-miR-5580-3p
CYFIP1	hsa-miR-7371e-3p
CYFIP1	hsa-miR-6325
CYFIP1	hsa-miR-3098-3p
CYFIP1	hsa-miR-3064-5p
CYFIP1	hsa-miR-5584-5p
CYFIP1	hsa-miR-7371j-3p
CYFIP1	hsa-miR-6800-5p
CYFIP1	hsa-miR-1292-5p
CYFIP1	hsa-miR-296-3p
CYFIP1	hsa-miR-7386b-3p
CYFIP1	hsa-miR-7202-5p
CYFIP1	hsa-miR-214-5p
CYFIP1	hsa-miR-96-5p
CYFIP1	hsa-miR-199
CYFIP1	hsa-miR-8802
CYFIP1	hsa-miR-181b-3p
CYFIP1	hsa-miR-655-3p
CYFIP1	hsa-miR-5197-3p
CYFIP1	hsa-miR-106-5p
CYFIP1	hsa-miR-3086-5p
CYFIP1	hsa-miR-193b-5p
CYFIP1	hsa-miR-8814
CYFIP1	hsa-miR-6777-5p
CYFIP1	hsa-miR-582-3p
CYFIP1	hsa-miR-466l-3p
CYFIP1	hsa-miR-1258-5p
CYFIP1	hsa-miR-3119
CYFIP1	hsa-miR-466q
CYFIP1	hsa-miR-6735-3p
CYFIP1	hsa-miR-20-5p
CYFIP1	hsa-miR-218-2-3p
CYFIP1	hsa-miR-539-5p
CYFIP1	hsa-miR-148-5p

CYFIP1	hsa-miR-3136-5p
CYFIP1	hsa-miR-3686
CYFIP1	hsa-miR-7317-3p
CYFIP1	hsa-miR-6696-3p
CYFIP1	hsa-miR-142-5p
CYFIP1	hsa-miR-3087-3p
CYFIP1	hsa-miR-7175-5p
CYFIP1	hsa-miR-1814a
CYFIP1	hsa-miR-197-5p
CYFIP1	hsa-miR-1258
CYFIP1	hsa-miR-432-5p
CYFIP1	hsa-miR-4436b-3p
CYFIP1	hsa-miR-3062-5p
CYFIP1	hsa-miR-686
CYFIP1	hsa-miR-3200-5p
CYFIP1	hsa-miR-4493
CYFIP1	hsa-miR-6973b-5p
CYFIP1	hsa-miR-133-5p
CYFIP1	hsa-miR-5006-5p
CYFIP1	hsa-miR-33-3p
CYFIP1	hsa-miR-5588-3p
CYFIP1	hsa-miR-1603
CYFIP1	hsa-miR-7061-5p
CYFIP1	hsa-miR-3121-3p
CYFIP1	hsa-miR-377-5p
CYFIP1	hsa-miR-6733-5p
CYFIP1	hsa-miR-1955-3p
CYFIP1	hsa-miR-7657-5p
CYFIP1	hsa-miR-291-5p
CYFIP1	hsa-miR-579
CYFIP1	hsa-miR-6839-3p
CYFIP1	hsa-miR-301-3p
CYFIP1	hsa-miR-502-5p
CYFIP1	hsa-miR-2399-3p
CYFIP1	hsa-miR-3076-5p
CYFIP1	hsa-miR-8801
CYFIP1	hsa-miR-664a-3p
CYFIP1	hsa-miR-7106-3p
CYFIP1	hsa-miR-466l-5p
CYFIP1	hsa-miR-370
CYFIP1	hsa-miR-4668-5p
CYFIP1	hsa-miR-7233-5p
CYFIP1	hsa-miR-124-5p
CYFIP1	hsa-miR-20b-3p
CYFIP1	hsa-miR-680
CYFIP1	hsa-miR-181b-1-3p

CYFIP1	hsa-miR-4743-3p
CYFIP1	hsa-miR-6821-5p
CYFIP1	hsa-miR-380-3p
CYFIP1	hsa-miR-5616-3p
CYFIP1	hsa-miR-26-3-3p
CYFIP1	hsa-miR-674-3p
CYFIP1	hsa-miR-2891
CYFIP1	hsa-miR-300-3p
CYFIP1	hsa-miR-6851-5p
CYFIP1	hsa-miR-5739
CYFIP1	hsa-miR-23
CYFIP1	hsa-miR-1909-3p
CYFIP1	hsa-miR-676-3p
CYFIP1	hsa-miR-488-5p
CYFIP1	hsa-miR-3606-3p
CYFIP1	hsa-miR-548n
CYFIP1	hsa-miR-344eh-5p
CYFIP1	hsa-miR-324-3p
CYFIP1	hsa-miR-201-3p
CYFIP1	hsa-miR-1227-3p
CYFIP1	hsa-miR-652-5p
CYFIP1	hsa-miR-8892
CYFIP1	hsa-miR-708-5p
CYFIP1	hsa-miR-4254
CYFIP1	hsa-miR-6980-5p
CYFIP1	hsa-miR-7199-3p
CYFIP1	hsa-miR-3662
CYFIP1	hsa-miR-2284j
CYFIP1	hsa-miR-496a-3p.2
CYFIP1	hsa-miR-6857-3p
CYFIP1	hsa-miR-586
CYFIP1	hsa-miR-7183-3p
CYFIP1	hsa-miR-520f-5p
CYFIP1	hsa-miR-1288-5p
CYFIP1	hsa-miR-3572
CYFIP1	hsa-miR-4466
CYFIP1	hsa-miR-5706
CYFIP1	hsa-miR-2285
CYFIP1	hsa-miR-484
CYFIP1	hsa-miR-8888
CYFIP1	hsa-miR-6540-3p
CYFIP1	hsa-miR-2284m
CYFIP1	hsa-miR-7090-5p
CYFIP1	hsa-miR-2371
CYFIP1	hsa-miR-7064-3p
CYFIP1	hsa-miR-4677-5p

CYFIP1	hsa-miR-203a-5p
CYFIP1	hsa-miR-3192-5p
CYFIP1	hsa-miR-6316
CYFIP1	hsa-miR-2312
CYFIP1	hsa-miR-5582-3p
CYFIP1	hsa-miR-153-3p
CYFIP1	hsa-miR-488-3p
CYFIP1	hsa-miR-6884-3p
CYFIP1	hsa-miR-511-5p
CYFIP1	hsa-miR-3158-5p
CYFIP1	hsa-miR-6529
CYFIP1	hsa-miR-1306-3p
CYFIP1	hsa-miR-891b
CYFIP1	hsa-miR-693-3p
CYFIP1	hsa-miR-4307
CYFIP1	hsa-miR-184-5p
CYFIP1	hsa-miR-6728-5p
CYFIP1	hsa-miR-6719-3p
CYFIP1	hsa-miR-195-5p
CYFIP1	hsa-miR-324-5p
CYFIP1	hsa-miR-2285h
CYFIP1	hsa-miR-145-3p
CYFIP1	hsa-miR-29-5p
CYFIP1	hsa-miR-6338
CYFIP1	hsa-miR-377-3p
CYFIP1	hsa-miR-3064
CYFIP1	hsa-miR-5125
CYFIP1	hsa-miR-125a-3p
CYFIP1	hsa-miR-154-5p
CYFIP1	hsa-miR-7150
CYFIP1	hsa-miR-4720-3p
CYFIP1	hsa-miR-25-3p
CYFIP1	hsa-miR-1250
CYFIP1	hsa-miR-138-5p
CYFIP1	hsa-miR-2424
CYFIP1	hsa-miR-214-3p
CYFIP1	hsa-miR-460a-5p
CYFIP1	hsa-miR-4692
CYFIP1	hsa-miR-7650-3p
CYFIP1	hsa-miR-661
CYFIP1	hsa-miR-4752
CYFIP1	hsa-miR-7025-5p
CYFIP1	hsa-miR-7087-3p
CYFIP1	hsa-miR-8839
CYFIP1	hsa-miR-8879
CYFIP1	hsa-miR-2366

CYFIP1	hsa-miR-7267-5p
CYFIP1	hsa-miR-889-5p
CYFIP1	hsa-miR-3057-5p
CYFIP1	hsa-miR-7848-3p
CYFIP1	hsa-miR-3609
CYFIP1	hsa-miR-449a
CYFIP1	hsa-miR-4774-5p
CYFIP1	hsa-miR-125-5p
CYFIP1	hsa-miR-6697-5p
CYFIP1	hsa-miR-130b-5p
CYFIP1	hsa-miR-1178-3p
CYFIP1	hsa-miR-4673
CYFIP1	hsa-miR-6991-3p
CYFIP1	hsa-miR-3922-3p
CYFIP1	hsa-miR-6083
CYFIP1	hsa-miR-487-5p
CYFIP1	hsa-miR-4760-3p
CYFIP1	hsa-miR-195-5p
CYFIP1	hsa-miR-331-3p
CYFIP1	hsa-miR-7160-5p
CYFIP1	hsa-miR-532-5p
CYFIP1	hsa-miR-505-3p
CYFIP1	hsa-miR-519-3p
CYFIP1	hsa-miR-1643b-5p
CYFIP1	hsa-miR-3553
CYFIP1	hsa-miR-4650
CYFIP1	hsa-miR-1283
CYFIP1	hsa-miR-340-5p
CYFIP1	hsa-miR-106
CYFIP1	hsa-miR-371a-5p
CYFIP1	hsa-miR-6961-5p
CYFIP1	hsa-miR-6982-3p
CYFIP1	hsa-miR-2329-5p
CYFIP1	hsa-miR-155-5p
CYFIP1	hsa-miR-516a
CYFIP1	hsa-miR-221-5p
CYFIP1	hsa-miR-502-5p
CYFIP1	hsa-miR-3594-3p
CYFIP1	hsa-miR-629-5p
CYFIP1	hsa-miR-4650-3p
CYFIP1	hsa-miR-549-5p
CYFIP1	hsa-miR-204-5p
CYFIP1	hsa-miR-15
CYFIP1	hsa-miR-210-3p
CYFIP1	hsa-miR-126-3p
CYFIP1	hsa-miR-3596d

CYFIP1	hsa-miR-1843b-5p
CYFIP1	hsa-miR-632
CYFIP1	hsa-miR-548ah-5p
CYFIP1	hsa-miR-4427
CYFIP1	hsa-miR-515-5p
CYFIP1	hsa-miR-8077
CYFIP1	hsa-miR-350-3p
CYFIP1	hsa-miR-2479
CYFIP1	hsa-miR-556-3p
CYFIP1	hsa-miR-7034-3p
CYFIP1	hsa-miR-548e-5p
CYFIP1	hsa-miR-4317
CYFIP1	hsa-miR-3148
CYFIP1	hsa-miR-5583-3p
CYFIP1	hsa-miR-3617-3p
CYFIP1	hsa-miR-376a-1-5p
CYFIP1	hsa-miR-1814
CYFIP1	hsa-miR-697
CYFIP1	hsa-miR-5009-5p
CYFIP1	hsa-miR-1462-5p
CYFIP1	hsa-miR-6908-3p
CYFIP1	hsa-miR-558
CYFIP1	hsa-miR-7341-5p
CYFIP1	hsa-miR-7069-5p
CYFIP1	hsa-miR-7-3p
CYFIP1	hsa-miR-664
CYFIP1	hsa-miR-188-5p
CYFIP1	hsa-miR-581
CYFIP1	hsa-miR-3674
CYFIP1	hsa-miR-338-5p
CYFIP1	hsa-miR-452-3p
CYFIP1	hsa-miR-5692a
CYFIP1	hsa-miR-465-5p
CYFIP1	hsa-miR-2284n
CYFIP1	hsa-miR-516b-5p
CYFIP1	hsa-miR-4638-3p
CYFIP1	hsa-miR-196-1-3p
CYFIP1	hsa-miR-3944-5p
CYFIP1	hsa-miR-6791-3p
CYFIP1	hsa-miR-5193
CYFIP1	hsa-miR-6727-3p
CYFIP1	hsa-let-7j-3p
CYFIP1	hsa-miR-3473c
CYFIP1	hsa-miR-10a-3p
CYFIP1	hsa-miR-4303
CYFIP1	hsa-miR-570-3p

CYFIP1	hsa-miR-494-5p
CYFIP1	hsa-miR-151
CYFIP1	hsa-miR-2352
CYFIP1	hsa-miR-188-5p
CYFIP1	hsa-miR-374b-5p
CYFIP1	hsa-miR-5694
CYFIP1	hsa-miR-335-3p
CYFIP1	hsa-miR-2472
CYFIP1	hsa-miR-5003-3p
CYFIP1	hsa-miR-1897-5p
CYFIP1	hsa-miR-7859
CYFIP1	hsa-miR-7373c-3p
CYFIP1	hsa-miR-374
CYFIP1	hsa-miR-548ai
CYFIP1	hsa-miR-519d-5p
CYFIP1	hsa-miR-26-5p
CYFIP1	hsa-miR-7262a-5p
CYFIP1	hsa-miR-1584-3p
CYFIP1	hsa-miR-509-5p
CYFIP1	hsa-miR-4645-5p
CYFIP1	hsa-miR-376b-3p
CYFIP1	hsa-miR-4642
CYFIP1	hsa-miR-6815-3p
CYFIP1	hsa-miR-4724-3p
CYFIP1	hsa-miR-32-5p
CYFIP1	hsa-miR-3593-5p
CYFIP1	hsa-miR-154-3p
CYFIP1	hsa-miR-674-5p
CYFIP1	hsa-miR-548an
CYFIP1	hsa-miR-3969
CYFIP1	hsa-miR-3153
CYFIP1	hsa-miR-7043-5p
CYFIP1	hsa-miR-651-5p
CYFIP1	hsa-miR-7033-5p
CYFIP1	hsa-miR-669p-3p
CYFIP1	hsa-miR-4505
CYFIP1	hsa-miR-4640-5p
CYFIP1	hsa-miR-5683
CYFIP1	hsa-miR-6992-3p
CYFIP1	hsa-miR-4738-5p
CYFIP1	hsa-miR-892b
CYFIP1	hsa-miR-181b-2-3p
CYFIP1	hsa-miR-330-5p
CYFIP1	hsa-miR-8810
CYFIP1	hsa-miR-6768-3p
CYFIP1	hsa-miR-6783-5p

CYFIP1	hsa-miR-20
CYFIP1	hsa-miR-1247-3p
CYFIP1	hsa-miR-144-3p
CYFIP1	hsa-miR-4428
CYFIP1	hsa-miR-6508-3p
CYFIP1	hsa-miR-6879-5p
CYFIP1	hsa-miR-690
CYFIP1	hsa-miR-4430
CYFIP1	hsa-miR-883a-5p
CYFIP1	hsa-miR-6948-5p
CYFIP1	hsa-miR-881-3p
CYFIP1	hsa-miR-5584-3p
CYFIP1	hsa-miR-153-5p
CYFIP1	hsa-miR-4756-3p
CYFIP1	hsa-miR-7g-3p
CYFIP1	hsa-miR-335-5p
CYFIP1	hsa-miR-3113-5p
CYFIP1	hsa-miR-6830-5p
CYFIP1	hsa-miR-6378
CYFIP1	hsa-miR-548b-3p
CYFIP1	hsa-miR-669i
CYFIP1	hsa-miR-1544b-5p
CYFIP1	hsa-miR-133
CYFIP1	hsa-miR-1771
CYFIP1	hsa-miR-4286
CYFIP1	hsa-miR-7271-3p
CYFIP1	hsa-miR-15a-3p
CYFIP1	hsa-miR-6529-3p
DCP2	hsa-miR-548b-3p
DCP2	hsa-miR-2485
DCP2	hsa-miR-5001-3p
DCP2	hsa-miR-302b-5p
DCP2	hsa-miR-2458
DCP2	hsa-miR-7385c-3p
DCP2	hsa-miR-7030-3p
DCP2	hsa-miR-335-5p
DCP2	hsa-miR-222-5p
DCP2	hsa-miR-6357
DCP2	hsa-miR-8900
DCP2	hsa-miR-2410
DCP2	hsa-miR-1188-3p
DCP2	hsa-miR-125b-2-3p
DCP2	hsa-miR-150-5p
DCP2	hsa-miR-5615-5p
DCP2	hsa-let-7g-3p
DCP2	hsa-miR-3921

DCP2	hsa-miR-541-3p
DCP2	hsa-miR-6720-5p
DCP2	hsa-miR-3084bc-5p
DCP2	hsa-miR-7211-5p
DCP2	hsa-miR-30e-5p
DCP2	hsa-miR-29b-2-5p
DCP2	hsa-miR-3082-3p
DCP2	hsa-miR-1302
DCP2	hsa-miR-335-5p
DCP2	hsa-miR-3166
DCP2	hsa-miR-4299
DCP2	hsa-miR-3106-5p
DCP2	hsa-miR-135b-5p
DCP2	hsa-miR-7337-5p
DCP2	hsa-miR-183-5p.1
DCP2	hsa-miR-2894
DCP2	hsa-miR-467f
DCP2	hsa-miR-7226-3p
DCP2	hsa-miR-513a
DCP2	hsa-miR-5197-5p
DCP2	hsa-miR-548c-3p
DCP2	hsa-miR-8799
DCP2	hsa-miR-514a-3p
DCP2	hsa-miR-7324-5p
DCP2	hsa-miR-6799-5p
DCP2	hsa-miR-7221-5p
DCP2	hsa-miR-581
DCP2	hsa-miR-1233-5p
DCP2	hsa-miR-365-3p
DCP2	hsa-miR-579-3p
DCP2	hsa-miR-6769b-5p
DCP2	hsa-miR-6882-5p
DCP2	hsa-miR-6529-3p
DCP2	hsa-miR-371b-5p
DCP2	hsa-miR-8823
DCP2	hsa-miR-6215
DCP2	hsa-miR-5046
DCP2	hsa-miR-4731-5p
DCP2	hsa-miR-1468-3p
DCP2	hsa-miR-4313
DCP2	hsa-miR-591
DCP2	hsa-let-7-5p
DCP2	hsa-miR-671-5p
DCP2	hsa-miR-216b-5p
DCP2	hsa-miR-129b-3p
DCP2	hsa-miR-6165

DCP2	hsa-miR-7056-5p
DCP2	hsa-miR-431-5p
DCP2	hsa-miR-4769-5p
DCP2	hsa-miR-6831-5p
DCP2	hsa-miR-599-5p
DCP2	hsa-miR-2895
DCP2	hsa-miR-5571-5p
DCP2	hsa-miR-7080-5p
DCP2	hsa-miR-4421
DCP2	hsa-miR-709
DCP2	hsa-miR-6509-3p
DCP2	hsa-miR-133b-5p
DCP2	hsa-miR-3662
DCP2	hsa-miR-1933-3p
DCP2	hsa-miR-7377-5p
DCP2	hsa-miR-7229-3p
DCP2	hsa-miR-3059-5p
DCP2	hsa-miR-466bcp-3p
DCP2	hsa-miR-1894-5p
DCP2	hsa-miR-6959-3p
DCP2	hsa-miR-32-5p
DCP2	hsa-miR-487a-3p
DCP2	hsa-miR-1243-5p
DCP2	hsa-miR-6971-3p
DCP2	hsa-miR-3145-3p
DCP2	hsa-miR-489-3p
DCP2	hsa-miR-513a-5p
DCP2	hsa-miR-7317-5p
DCP2	hsa-miR-1540-5p
DCP2	hsa-miR-5582-3p
DCP2	hsa-miR-2887
DCP2	hsa-miR-548as-5p
DCP2	hsa-miR-4704-5p
DCP2	hsa-miR-4518
DCP2	hsa-miR-7656-3p
DCP2	hsa-miR-7262a-5p
DCP2	hsa-miR-2372
DCP2	hsa-miR-3085-5p
DCP2	hsa-miR-6740-3p
DCP2	hsa-miR-3575
DCP2	hsa-miR-5703
DCP2	hsa-miR-466n-3p
DCP2	hsa-miR-3663-5p
DCP2	hsa-miR-631
DCP2	hsa-miR-561-3p
DCP2	hsa-miR-7332-3p

DCP2	hsa-miR-3682-5p
DCP2	hsa-miR-8833
DCP2	hsa-miR-7681-5p
DCP2	hsa-miR-4469
DCP2	hsa-miR-122-5p
DCP2	hsa-miR-596
DCP2	hsa-miR-30
DCP2	hsa-miR-15a-3p
DCP2	hsa-miR-548ap-3p
DCP2	hsa-miR-543
DCP2	hsa-miR-6943-3p
DCP2	hsa-miR-3119
DCP2	hsa-miR-7296-5p
DCP2	hsa-miR-5694
DCP2	hsa-miR-466mo-3p
DCP2	hsa-miR-6890-3p
DCP2	hsa-miR-1584-3p
DCP2	hsa-miR-132-3p
DCP2	hsa-miR-7168-5p
DCP2	hsa-miR-2326
DCP2	hsa-miR-8871
DCP2	hsa-miR-880-3p
DCP2	hsa-miR-208a-3p
DCP2	hsa-miR-515-5p
DCP2	hsa-miR-5107-3p
DCP2	hsa-miR-101b-3p.1
DCP2	hsa-miR-2464-5p
DCP2	hsa-miR-101a-3p.2
DCP2	hsa-miR-7388a-3p
DCP2	hsa-miR-7216-3p
DCP2	hsa-miR-7368-3p
DCP2	hsa-miR-6992-3p
DCP2	hsa-miR-6827-3p
DCP2	hsa-miR-7257-5p
DCP2	hsa-miR-4727-3p
DCP2	hsa-miR-5107-5p
DCP2	hsa-miR-411-5p.2
DCP2	hsa-miR-2325b
DCP2	hsa-miR-548aef
DCP2	hsa-miR-181b-2-3p
DCP2	hsa-miR-7196-5p
DCP2	hsa-miR-3093-5p
DCP2	hsa-miR-6835-3p
DCP2	hsa-miR-18-3p
DCP2	hsa-miR-5000-3p
DCP2	hsa-miR-1268b

DCP2	hsa-miR-522-3p
DCP2	hsa-miR-15b-3p
DCP2	hsa-miR-6237
DCP2	hsa-miR-6983-3p
DCP2	hsa-miR-624-3p
DCP2	hsa-miR-381-3p
DCP2	hsa-miR-3170
DCP2	hsa-miR-519d-5p
DCP2	hsa-miR-548at-3p
DCP2	hsa-miR-7651-3p
DCP2	hsa-miR-183-5p.2
DCP2	hsa-miR-6904-3p
DCP2	hsa-miR-8801
DCP2	hsa-miR-7021-3p
DCP2	hsa-miR-1747-3p
DCP2	hsa-miR-767-3p
DCP2	hsa-miR-153
DCP2	hsa-miR-542
DCP2	hsa-miR-6900-5p
DCP2	hsa-miR-548k
DCP2	hsa-miR-324-5p
DCP2	hsa-miR-1930-3p
DCP2	hsa-miR-4722-3p
DCP2	hsa-miR-218-2-3p
DCP2	hsa-miR-7222-5p
DCP2	hsa-miR-146
DCP2	hsa-miR-30b-5p
DCP2	hsa-miR-5112
DCP2	hsa-miR-2384
DCP2	hsa-miR-883ab-3p
DCP2	hsa-miR-605
DCP2	hsa-miR-7283-3p
DCP2	hsa-miR-344f-5p
DCP2	hsa-miR-512
DCP2	hsa-miR-2285j
DCP2	hsa-miR-5004-5p
DCP2	hsa-miR-371a-5p
DCP2	hsa-miR-548ab
DCP2	hsa-miR-2428
DCP2	hsa-miR-200-3p
DCP2	hsa-miR-548aq-5p
DCP2	hsa-miR-7216-5p
DCP2	hsa-miR-449a
DCP2	hsa-miR-6365
DCP2	hsa-miR-1-3p
DCP2	hsa-miR-2681-5p

DCP2	hsa-miR-7273-3p
DCP2	hsa-miR-3192-3p
DCP2	hsa-miR-548cej-3p
DCP2	hsa-miR-2483-3p
DCP2	hsa-miR-561-5p
DCP2	hsa-miR-3963
DCP2	hsa-miR-548g-3p
DCP2	hsa-miR-2333
DCP2	hsa-miR-514a-5p
DCP2	hsa-miR-510-3p
DCP2	hsa-miR-744-3p
DCP2	hsa-miR-3113-5p
DCP2	hsa-miR-16-5p
DCP2	hsa-miR-129b-5p
DCP2	hsa-miR-5104
DCP2	hsa-miR-6320
DCP2	hsa-miR-411-5p
DCP2	hsa-miR-6980-3p
DCP2	hsa-miR-133
DCP2	hsa-miR-4723-5p
DCP2	hsa-miR-500b-3p
DCP2	hsa-miR-548av-5p
DCP2	hsa-miR-132-3p
DCP2	hsa-miR-217
DCP2	hsa-miR-7029-3p
DCP2	hsa-miR-499b-5p
DCP2	hsa-miR-7315-5p
DCP2	hsa-miR-186-5p
DCP2	hsa-miR-421-3p
DCP2	hsa-miR-460a-5p
DCP2	hsa-miR-6872-3p
DCP2	hsa-miR-1252
DCP2	hsa-miR-7678-3p
DCP2	hsa-miR-693-3p
DCP2	hsa-miR-10-5p
DCP2	hsa-miR-584-5p
DCP2	hsa-miR-1243
DCP2	hsa-miR-8810
DCP2	hsa-miR-7251-3p
DCP2	hsa-miR-154-3p
DCP2	hsa-miR-944
DCP2	hsa-miR-4666a-3p
DCP2	hsa-miR-320a
DCP2	hsa-miR-138-3p
DCP2	hsa-miR-669p-3p
DCP2	hsa-miR-7199-3p

DCP2	hsa-miR-494-5p
DCP2	hsa-miR-570-3p
DCP2	hsa-miR-4796-5p
DCP2	hsa-miR-642a-5p
DCP2	hsa-miR-3664-3p
DCP2	hsa-miR-5004-3p
DCP2	hsa-miR-599
DCP2	hsa-miR-4424
DCP2	hsa-miR-517-5p
DCP2	hsa-miR-203a-3p.1
DCP2	hsa-miR-2483
DCP2	hsa-miR-2116-3p
DCP2	hsa-miR-7114-5p
DCP2	hsa-miR-3085-3p
DCP2	hsa-miR-10a-3p
DCP2	hsa-miR-7454-5p
DCP2	hsa-miR-597-3p
DCP2	hsa-miR-4430
DCP2	hsa-miR-513c-5p
DCP2	hsa-miR-3622b-3p
DCP2	hsa-miR-7227-5p
DCP2	hsa-miR-381-3p
DCP2	hsa-miR-4752
DCP2	hsa-miR-200bc-3p
DCP2	hsa-miR-7856-5p
DCP2	hsa-miR-4703-5p
DCP2	hsa-miR-1299
DCP2	hsa-miR-3909
DCP2	hsa-miR-5588-3p
DCP2	hsa-miR-1245-3p
DCP2	hsa-miR-3097-5p
DCP2	hsa-miR-7030-5p
DCP2	hsa-miR-4459
DCP2	hsa-miR-629-3p
DCP2	hsa-miR-610
DCP2	hsa-miR-1904
DCP2	hsa-miR-672-3p
DCP2	hsa-miR-7061-5p
DCP2	hsa-miR-654-5p
DCP2	hsa-miR-3590-3p
DCP2	hsa-miR-4270
DCP2	hsa-miR-7194-5p
DCP2	hsa-miR-2329-5p
DCP2	hsa-miR-7374a-3p
DCP2	hsa-miR-3150b-5p
DCP2	hsa-miR-4517

DCP2	hsa-miR-33a-3p
DCP2	hsa-miR-4659b-5p
DCP2	hsa-miR-6973a-3p
DCP2	hsa-miR-4457
DCP2	hsa-miR-6531
DCP2	hsa-miR-194-2-3p
DCP2	hsa-miR-6998-5p
DCP2	hsa-miR-889-3p
DCP2	hsa-miR-362-3p
DCP2	hsa-miR-6949-5p
DCP2	hsa-miR-1545a-3p
DCP2	hsa-miR-876-5p
DCP2	hsa-miR-550-3p
DCP2	hsa-miR-3470b
DCP2	hsa-miR-513b-5p
DCP2	hsa-miR-4463
DCP2	hsa-miR-7046-3p
DCP2	hsa-miR-6131
DCP2	hsa-miR-668-3p
DCP2	hsa-miR-497-5p
DCP2	hsa-miR-326-5p
DCP2	hsa-miR-6407
DCP2	hsa-miR-194-5p
DCP2	hsa-miR-103
DCP2	hsa-miR-656
DCP2	hsa-miR-500b-5p
DCP2	hsa-miR-2393
DCP2	hsa-miR-2355-3p
DCP2	hsa-miR-7033-3p
DCP2	hsa-miR-6835-5p
DCP2	hsa-miR-6920-5p
DCP2	hsa-miR-708-5p
DCP2	hsa-miR-7022-5p
DCP2	hsa-miR-700-5p
DCP2	hsa-miR-7296-3p
DCP2	hsa-miR-217-3p
DCP2	hsa-miR-1255
DCP2	hsa-miR-4495
DCP2	hsa-miR-378
DCP2	hsa-miR-219-1-3p
DCP2	hsa-miR-877-3p
DCP2	hsa-miR-195-5p
DCP2	hsa-miR-6803-5p
DCP2	hsa-miR-7337-3p
DCP2	hsa-miR-219c-3p
DCP2	hsa-miR-548aa

DCP2	hsa-let-7f-2-3p
DCP2	hsa-miR-2322-3p
DCP2	hsa-miR-3475-5p
DCP2	hsa-miR-6941-5p
DCP2	hsa-miR-7023-5p
DCP2	hsa-miR-124
DCP2	hsa-miR-1416-3p
DCP2	hsa-miR-6836-5p
DCP2	hsa-miR-1225-5p
DCP2	hsa-miR-7260-3p
DCP2	hsa-miR-5007-5p
DCP2	hsa-miR-677-5p
DCP2	hsa-miR-5096
DCP2	hsa-miR-1911-5p
DCP2	hsa-miR-2285ad
DCP2	hsa-miR-514a-3p
DCP2	hsa-miR-342-3p
DCP2	hsa-miR-7074-3p
DCP2	hsa-miR-4764-3p
DCP2	hsa-miR-5120
DCP2	hsa-miR-25-3p
DCP2	hsa-miR-7378-3p
DCP2	hsa-miR-4691-5p
DCP2	hsa-miR-216-5p
DCP2	hsa-miR-7244-5p
DCP2	hsa-miR-216a
DCP2	hsa-miR-1262-5p
DCP2	hsa-miR-1-2-5p
DCP2	hsa-miR-10
DCP2	hsa-miR-6814-5p
DCP2	hsa-miR-559
DCP2	hsa-miR-544-3p
DCP2	hsa-miR-7350-3p
DCP2	hsa-miR-891-3p
DCP2	hsa-miR-4774-3p
DCP2	hsa-miR-7647-5p
DCP2	hsa-miR-7246-3p
DCP2	hsa-miR-1248
DCP2	hsa-miR-8901
DCP2	hsa-miR-219-2-3p
DCP2	hsa-miR-487-5p
DCP2	hsa-miR-6715a-3p
DCP2	hsa-miR-3550
DCP2	hsa-miR-183-3p
DCP2	hsa-miR-369-3p
DCP2	hsa-miR-548m

DCP2	hsa-miR-4533
DCP2	hsa-miR-4288
DCP2	hsa-miR-2337
DCP2	hsa-miR-6120-3p
DCP2	hsa-miR-548ag
DCP2	hsa-miR-383-5p
DCP2	hsa-miR-92
DCP2	hsa-miR-122-3p
DCP2	hsa-miR-4650
DCP2	hsa-miR-8824
DCP2	hsa-miR-7164-5p
DCP2	hsa-miR-3618
DCP2	hsa-miR-883b-5p
DCP2	hsa-miR-4724-5p
DCP2	hsa-miR-508-5p
DCP2	hsa-miR-1968-5p
DCP2	hsa-miR-490-5p
DCP2	hsa-miR-5695
DCP2	hsa-miR-148-3p
DCP2	hsa-miR-7373d-3p
DCP2	hsa-miR-888-3p
DCP2	hsa-miR-2284ab
DCP2	hsa-miR-202-3p
DCP2	hsa-miR-764
DCP2	hsa-miR-675-3p
DCP2	hsa-miR-1266-5p
DCP2	hsa-miR-519
DCP2	hsa-miR-7977
DCP2	hsa-miR-1253
DCP2	hsa-miR-548x-3p
DCP2	hsa-miR-4273
DCP2	hsa-miR-2285m
DCP2	hsa-miR-95-5p
DCP2	hsa-miR-144-3p
DCP2	hsa-miR-379-3p
DCP2	hsa-miR-6952-3p
DCP2	hsa-miR-1911-3p
DCP2	hsa-miR-4775
DCP2	hsa-miR-7068-5p
DCP2	hsa-miR-2484
DCP2	hsa-miR-7387-5p
DCP2	hsa-miR-2330-3p
DCP2	hsa-miR-4639-3p
DCP2	hsa-miR-8887
DCP2	hsa-miR-7272-3p
DCP2	hsa-miR-7398m-3p

DCP2	hsa-miR-4722-5p
DCP2	hsa-miR-548aj-3p
DCP2	hsa-miR-692
DCP2	hsa-miR-199-3p
DCP2	hsa-miR-7660-5p
DCP2	hsa-miR-2367-3p
DCP2	hsa-miR-1233-3p
DCP2	hsa-miR-6748-3p
DCP2	hsa-miR-8085
DCP2	hsa-miR-7a-1-3p
DCP2	hsa-miR-1235
DCP2	hsa-miR-2370-5p
DCP2	hsa-miR-4303
DCP2	hsa-miR-7338-5p
DCP2	hsa-miR-2957
DCP2	hsa-miR-6719-3p
DCP2	hsa-miR-301a-3p
DCP2	hsa-miR-491-5p
DCP2	hsa-miR-3593-5p
DCP2	hsa-miR-29e
DCP2	hsa-miR-30-3p
DCP2	hsa-miR-3061-5p
DCP2	hsa-miR-190-5p
DCP2	hsa-miR-2439-3p
DCP2	hsa-miR-2285d
DCP2	hsa-miR-7361-5p
DCP2	hsa-miR-518e-3p
DCP2	hsa-miR-376c-3p
DCP2	hsa-miR-7178-3p
DCP2	hsa-miR-503-3p
DCP2	hsa-miR-29-3p
DCP2	hsa-miR-330-3p
DCP2	hsa-miR-7351-3p
DCP2	hsa-miR-7331-1-3p
DCP2	hsa-miR-1301-5p
DCP2	hsa-miR-4282
DCP2	hsa-miR-454
DCP2	hsa-miR-6358
DCP2	hsa-miR-186-3p
DCP2	hsa-miR-7224-3p
DCP2	hsa-miR-2325
DCP2	hsa-miR-219a-2-3p
DCP2	hsa-miR-7256-5p
DCP2	hsa-miR-27-3p
DCP2	hsa-miR-2399-3p
DCP2	hsa-miR-4714-5p

DCP2	hsa-miR-140-3p.2
DCP2	hsa-miR-1197-5p
DCP2	hsa-miR-6081
DCP2	hsa-miR-6738-3p
DCP2	hsa-miR-125-5p
DCP2	hsa-miR-743ab-3p
DCP2	hsa-miR-3109-3p
DCP2	hsa-miR-4456
DCP2	hsa-miR-18b-3p
DCP2	hsa-miR-574-5p
DCP2	hsa-miR-5619-3p
DCP2	hsa-miR-7683-3p
DCP2	hsa-miR-1964-5p
DCP2	hsa-miR-4531
DCP2	hsa-miR-134-3p
DCP2	hsa-miR-6973b-3p
DCP2	hsa-miR-6804-3p
DCP2	hsa-miR-7215-5p
DCP2	hsa-miR-467a-5p
DCP2	hsa-miR-19a-5p
DCP2	hsa-miR-2489
DCP2	hsa-miR-4524b-3p
DCP2	hsa-miR-7094-1-5p
DCP2	hsa-miR-741-3p
DCP2	hsa-miR-4781-3p
DCP2	hsa-miR-872-5p
DCP2	hsa-miR-1285-3p
DCP2	hsa-miR-2303
DCP2	hsa-miR-3164
DCP2	hsa-miR-6074
DCP2	hsa-miR-5581-5p
DCP2	hsa-miR-3192
DCP2	hsa-miR-7344-3p
DCP2	hsa-miR-493-3p
DCP2	hsa-miR-496-3p
DCP2	hsa-miR-449c-3p
DCP2	hsa-miR-3099-3p
DCP2	hsa-miR-877-5p
DCP2	hsa-miR-374a-5p
DCP2	hsa-miR-7033-5p
DCP2	hsa-miR-1191a
DCP2	hsa-miR-1285-3p
DCP2	hsa-miR-6949-3p
DCP2	hsa-miR-7975
DCP2	hsa-miR-2285x
DCP2	hsa-miR-548a-3p

DCP2	hsa-miR-3916
DCP2	hsa-miR-362-5p
DCP2	hsa-miR-7353-3p
DCP2	hsa-miR-4330
DCP2	hsa-miR-3927-3p
DCP2	hsa-miR-6133
DCP2	hsa-miR-4419a
DCP2	hsa-miR-665-3p
DCP2	hsa-miR-7084-3p
DCP2	hsa-miR-3544-3p
DCP2	hsa-miR-4510
DCP2	hsa-miR-6355
DCP2	hsa-miR-6816-3p
DCP2	hsa-miR-3135b
DCP2	hsa-miR-6920-3p
DCP2	hsa-miR-365b-3p
DCP2	hsa-miR-548am-3p
DCP2	hsa-miR-7076-5p
DCP2	hsa-miR-7179-3p
DCP2	hsa-miR-2284n
DCP2	hsa-miR-6086
DCP2	hsa-miR-7189-3p
DCP2	hsa-miR-548aq-3p
DCP2	hsa-miR-519-5p
DCP2	hsa-miR-7g-3p
DCP2	hsa-miR-5704
DCP2	hsa-miR-660-3p
DCP2	hsa-miR-6800-3p
DCP2	hsa-miR-7398l-3p
DCP2	hsa-miR-6509-5p
DCP2	hsa-miR-2285g
DCP2	hsa-miR-4428
DCP2	hsa-miR-7034-3p
DCP2	hsa-miR-485-5p
DCP2	hsa-miR-6893-5p
DCP2	hsa-miR-7649-5p
DCP2	hsa-miR-934-3p
DCP2	hsa-miR-7066-3p
DCP2	hsa-miR-5699-3p
DCP2	hsa-miR-7328-3p
DCP2	hsa-miR-8848
DCP2	hsa-miR-4779
DCP2	hsa-miR-325-5p
DCP2	hsa-miR-7266-5p
DCP2	hsa-miR-3918
DCP2	hsa-miR-155-3p

DCP2	hsa-miR-1283
DCP2	hsa-miR-5134-5p
DCP2	hsa-miR-874
DCP2	hsa-miR-3576
DCP2	hsa-miR-5681b
DCP2	hsa-miR-6849-3p
DCP2	hsa-miR-1976
DCP2	hsa-miR-3136-5p
DCP2	hsa-miR-6947-5p
DCP2	hsa-miR-7013-3p
DCP2	hsa-miR-3583-3p
DCP2	hsa-miR-466i-3p
DCP2	hsa-miR-96-5p
DCP2	hsa-miR-590-3p
DCP2	hsa-miR-548p
DCP2	hsa-miR-1545b-3-3p
DCP2	hsa-miR-8067
DCP2	hsa-miR-4537
DCP2	hsa-miR-3646
DCP2	hsa-miR-7398s-3p
DCP2	hsa-miR-302bd
DCP2	hsa-miR-934
DCP2	hsa-miR-24-3p
DCP2	hsa-miR-669c-3p
DCP2	hsa-miR-455-3p.1
DCP2	hsa-miR-6868-3p
DCP2	hsa-miR-6722-5p
DCP2	hsa-miR-6371
DCP2	hsa-miR-659-3p
DCP2	hsa-miR-30a-3p
DCP2	hsa-miR-4762-5p
DCP2	hsa-miR-885-5p
DCP2	hsa-miR-4327
DCP2	hsa-miR-642-3p
DCP2	hsa-miR-3082-5p
DCP2	hsa-miR-345-5p
DCP2	hsa-miR-544a
DCP2	hsa-miR-3552
DCP2	hsa-miR-7649-3p
DCP2	hsa-miR-6964-3p
DCP2	hsa-miR-3066-5p
DCP2	hsa-miR-3924
DCP2	hsa-miR-2370-3p
DCP2	hsa-miR-300-5p
DCP2	hsa-miR-499a-3p
DCP2	hsa-miR-7299-5p

DCP2	hsa-miR-548ay-3p
DCP2	hsa-miR-7330-5p
DCP2	hsa-miR-184-3p
DCP2	hsa-miR-4648
DCP2	hsa-miR-683
DCP2	hsa-miR-7386a-3-3p
DCP2	hsa-miR-4672
DCP2	hsa-miR-373-3p
DCP2	hsa-miR-221-3p
DCP2	hsa-miR-7073-5p
DCP2	hsa-miR-6758-3p
DCP2	hsa-let-7abf-3p
DCP2	hsa-miR-3936
DCP2	hsa-miR-8844
DCP2	hsa-miR-2322-5p
DCP2	hsa-miR-8842
DCP2	hsa-miR-374
DCP2	hsa-miR-669b-5p
DCP2	hsa-miR-7342-5p
DCP2	hsa-miR-7325-3p
DCP2	hsa-miR-3596a
DCP2	hsa-miR-6894-3p
DCP2	hsa-miR-4724-3p
DCP2	hsa-miR-518-3p
DCP2	hsa-miR-6991-3p
DCP2	hsa-miR-7349-3p
DCP2	hsa-miR-1835
DCP2	hsa-miR-7113-3p
DCP2	hsa-miR-548ao-3p
DCP2	hsa-miR-2388-3p
DCP2	hsa-miR-7324-3p
DCP2	hsa-miR-31-5p
DCP2	hsa-miR-8076
DCP2	hsa-miR-6523b
DCP2	hsa-miR-6373
DCP2	hsa-miR-7394a-5p
DCP2	hsa-miR-197-3p
DCP2	hsa-miR-1198-3p
DCP2	hsa-miR-219a-5p
DCP2	hsa-miR-653
DCP2	hsa-miR-374-5p
DCP2	hsa-miR-4708-3p
DCP2	hsa-miR-673-5p
DCP2	hsa-miR-135-3p
DCP2	hsa-miR-3926
DCP2	hsa-miR-8908f

DCP2	hsa-miR-466l-3p
DCP2	hsa-miR-545-3p
DCP2	hsa-miR-1814b
DCP2	hsa-miR-548s
DCP2	hsa-miR-7682-5p
DCP2	hsa-miR-6760-5p
DCP2	hsa-miR-544-5p
DCP2	hsa-miR-34
DCP2	hsa-miR-7042-5p
DCP2	hsa-miR-7015-5p
DCP2	hsa-miR-1245b-3p
DCP2	hsa-miR-370
DCP2	hsa-miR-7151-3p
DCP2	hsa-miR-301-3p
DCP2	hsa-miR-200ab-5p
DCP2	hsa-miR-8906
DCP2	hsa-miR-2455
DCP2	hsa-miR-4755-3p
DCP2	hsa-miR-7285-3p
DCP2	hsa-miR-3941
DCP2	hsa-miR-298-5p
DCP2	hsa-miR-3978
DCP2	hsa-miR-7055-5p
DCP2	hsa-miR-374-3p
DCP2	hsa-miR-8876
DCP2	hsa-miR-8908e
DCP2	hsa-miR-1255-3p
DCP2	hsa-miR-7331-2-3p
DCP2	hsa-miR-3654
DCP2	hsa-miR-6852-5p
DCP2	hsa-miR-6525
DCP2	hsa-miR-4760-5p
DCP2	hsa-miR-3689f
DCP2	hsa-miR-4520-2-3p
DCP2	hsa-miR-3573-3p
DCP2	hsa-miR-432-5p
DCP2	hsa-miR-2340
DCP2	hsa-miR-883-3p
DCP2	hsa-miR-377-5p
DCP2	hsa-miR-8063
DCP2	hsa-miR-7680-5p
DCP2	hsa-miR-8103
DCP2	hsa-miR-3160-3p
DCP2	hsa-miR-26b-3p
DCP2	hsa-miR-3100-3p
DCP2	hsa-miR-106-3p

DCP2	hsa-miR-4511
DCP2	hsa-miR-3919
DCP2	hsa-miR-3689-3p
DCP2	hsa-miR-4802-3p
DCP2	hsa-miR-6327
DCP2	hsa-miR-4763-3p
DCP2	hsa-miR-207
DCP2	hsa-miR-1255a
DCP2	hsa-miR-376a-1-5p
DCP2	hsa-miR-2450bd
DCP2	hsa-miR-8809
DCP2	hsa-miR-4478
DCP2	hsa-miR-1284
DCP2	hsa-miR-299-3p
DCP2	hsa-miR-4519
DCP2	hsa-miR-410-5p
DCP2	hsa-miR-6778-3p
DCP2	hsa-miR-126-5p
DCP2	hsa-miR-7855-5p
DCP2	hsa-miR-589-5p
DCP2	hsa-miR-4279
DCP2	hsa-miR-2421
DCP2	hsa-miR-6777-3p
DCP2	hsa-miR-5095
DCP2	hsa-miR-16-3p
DCP2	hsa-miR-7238-3p
DCP2	hsa-miR-133a-3p.2
DCP2	hsa-miR-486-3p
DCP2	hsa-miR-6923-3p
DCP2	hsa-miR-206
DCP2	hsa-miR-367-3p
DCP2	hsa-miR-8891
DCP2	hsa-miR-2285y
DCP2	hsa-miR-7685-5p
DCP2	hsa-miR-7007-5p
DCP2	hsa-miR-365-5p
DCP2	hsa-miR-8873a
DCP2	hsa-miR-126-3p
DCP2	hsa-miR-6737-3p
DCP2	hsa-miR-3154
DCP2	hsa-miR-4689
DCP2	hsa-miR-5698
DCP2	hsa-miR-6833-5p
DCP2	hsa-miR-9-5p
DCP2	hsa-miR-674-5p
DCP2	hsa-miR-606

DCP2	hsa-miR-7215-3p
DCP2	hsa-miR-219b-5p
DCP2	hsa-miR-465-3p
DCP2	hsa-miR-301
DCP2	hsa-miR-7673-5p
DCP2	hsa-miR-524-5p
DCP2	hsa-miR-6967-3p
DCP2	hsa-miR-549-3p
DCP2	hsa-miR-8118
DCP2	hsa-miR-6918-5p
DCP2	hsa-miR-144-3p
DCP2	hsa-miR-7253-3p
DCP2	hsa-miR-7389-5p
DCP2	hsa-miR-6730-5p
DCP2	hsa-miR-7375-5p
DCP2	hsa-miR-7207-3p
DCP2	hsa-miR-3180-5p
DCP2	hsa-miR-468-3p
DCP2	hsa-miR-144-5p
DCP2	hsa-miR-8875
DCP2	hsa-miR-887-5p
DCP2	hsa-miR-6977-5p
DCP2	hsa-miR-297bc-3p
DCP2	hsa-miR-7251-5p
DCP2	hsa-miR-4726-3p
DCP2	hsa-miR-374b-3p
DCP2	hsa-miR-525-5p
DCP2	hsa-miR-330-3p.1
DCP2	hsa-miR-7302-3p
DCP2	hsa-miR-649
DCP2	hsa-miR-146a-3p
DCP2	hsa-let-7ag-3p
DCP2	hsa-miR-330-3p
DCP2	hsa-miR-3109-5p
DCP2	hsa-miR-7326-3p
DCP2	hsa-miR-4427
DCP2	hsa-miR-3129-5p
DCP2	hsa-miR-302f
DCP2	hsa-miR-642b-5p
DCP2	hsa-miR-7181-5p
DCP2	hsa-miR-30a-5p
DCP2	hsa-miR-3095-3p
DCP2	hsa-miR-4774-5p
DCP2	hsa-miR-223-5p
DCP2	hsa-miR-6393
DCP2	hsa-miR-7398l-5p

DCP2	hsa-miR-4423
DCP2	hsa-miR-548ai
DCP2	hsa-miR-194-1-3p
DCP2	hsa-miR-7159-5p
DCP2	hsa-miR-422
DCP2	hsa-miR-8805
DCP2	hsa-miR-320b
DCP2	hsa-miR-1247-3p
DCP2	hsa-miR-291-3p
DCP2	hsa-miR-2284l
DCP2	hsa-miR-940
DCP2	hsa-miR-677
DCP2	hsa-miR-23-5p
DCP2	hsa-miR-763
DCP2	hsa-miR-5709-3p
DCP2	hsa-miR-586
DCP2	hsa-miR-383-3p
DCP2	hsa-miR-645
DCP2	hsa-miR-3689b-5p
DCP2	hsa-miR-671-5p
DCP2	hsa-miR-3551-3p
DCP2	hsa-miR-6331
DCP2	hsa-miR-7068-3p
DCP2	hsa-miR-4534
DCP2	hsa-miR-512-5p
DCP2	hsa-miR-7027-5p
DCP2	hsa-miR-2284d
DCP2	hsa-miR-6887-3p
DCP2	hsa-miR-2402
DCP2	hsa-miR-2447
DCP2	hsa-miR-6819-5p
DCP2	hsa-miR-7010-5p
DCP2	hsa-miR-3613-3p
DCP2	hsa-miR-3152-5p
DCP2	hsa-miR-6905-5p
DCP2	hsa-miR-3120-3p
DCP2	hsa-miR-7850-5p
DCP2	hsa-miR-1343-3p
DCP2	hsa-let-7f-1-3p
DCP2	hsa-miR-1323
DCP2	hsa-miR-4735-5p
DCP2	hsa-miR-3620-3p
DCP2	hsa-miR-3120-3p
DCP2	hsa-miR-6325
DCP2	hsa-miR-6502-3p
DCP2	hsa-miR-483-3p.1

DCP2	hsa-miR-512-3p
DCP2	hsa-miR-3591-5p
DCP2	hsa-miR-6323
DCP2	hsa-miR-1258
DCP2	hsa-miR-4298
DCP2	hsa-miR-487-3p
DCP2	hsa-miR-5690
DCP2	hsa-miR-7075-5p
DCP2	hsa-miR-2286
DCP2	hsa-miR-376c
DCP2	hsa-miR-3667-5p
DCP2	hsa-miR-182-5p
DCP2	hsa-miR-2335
DCP2	hsa-miR-7664-5p
DCP2	hsa-miR-6847-5p
DCP2	hsa-miR-2431-5p
DCP2	hsa-let-7c-1-3p
DCP2	hsa-miR-471-3p
DCP2	hsa-miR-1909-3p
DCP2	hsa-miR-8099
DCP2	hsa-miR-8818
DCP2	hsa-miR-7269-3p
DCP2	hsa-miR-758-3p
DCP2	hsa-miR-4251
DCP2	hsa-miR-2904
DCP2	hsa-miR-2349
DCP2	hsa-miR-3161
DCP2	hsa-miR-7191-3p
DCP2	hsa-miR-3127-5p
DCP2	hsa-miR-3622a-3p
DCP2	hsa-miR-500
DCP2	hsa-miR-7249-3p
DCP2	hsa-miR-7350-5p
DCP2	hsa-miR-1897-5p
DCP2	hsa-miR-7322-5p
DCP2	hsa-miR-5006-3p
DCP2	hsa-miR-6744-3p
DCP2	hsa-miR-7051-5p
DCP2	hsa-miR-7398e-3p
DCP2	hsa-miR-4439
DCP2	hsa-miR-2343
DCP2	hsa-miR-8895
DCP2	hsa-miR-4792
DCP2	hsa-miR-2183
DCP2	hsa-miR-713
DCP2	hsa-miR-1207-3p

DCP2	hsa-miR-1268a
DCP2	hsa-miR-4419b
DCP2	hsa-miR-2431-3p
DCP2	hsa-miR-1267
DCP2	hsa-miR-587
DCP2	hsa-miR-412-3p
DCP2	hsa-miR-1207
DCP2	hsa-miR-1966-3p
DCP2	hsa-miR-507
DCP2	hsa-miR-3943
DCP2	hsa-miR-292b-5p
DCP2	hsa-miR-6812-5p
DCP2	hsa-miR-1982-3p
DCP2	hsa-miR-4742-3p
DCP2	hsa-miR-6751-5p
DCP2	hsa-miR-593-3p
DCP2	hsa-miR-1841
DCP2	hsa-miR-511-5p
DCP2	hsa-miR-3675-5p
DCP2	hsa-miR-494
DCP2	hsa-miR-23-3p
DCP2	hsa-miR-505-5p
DCP2	hsa-miR-665
DCP2	hsa-miR-378a-5p
DCP2	hsa-miR-8087
DCP2	hsa-miR-7342-3p
DCP2	hsa-miR-7374c-3p
DCP2	hsa-miR-7370-3p
DCP2	hsa-miR-890-5p
DCP2	hsa-miR-140-5p
DCP2	hsa-miR-30d-5p
DCP2	hsa-miR-4786-5p
DCP2	hsa-miR-873a-5p.2
DCP2	hsa-miR-6715b-5p
DCP2	hsa-miR-488-5p
DCP2	hsa-miR-7299-3p
DCP2	hsa-miR-7323-5p
DCP2	hsa-miR-2308
DCP2	hsa-miR-101-3p
DCP2	hsa-miR-555
DCP2	hsa-miR-452-3p
DCP2	hsa-miR-4435
DCP2	hsa-miR-4423-3p
DCP2	hsa-miR-942-3p
DCP2	hsa-miR-1956
DCP2	hsa-miR-4685-3p

DCP2	hsa-miR-7282-5p
DCP2	hsa-miR-7680-3p
DCP2	hsa-miR-3606-5p
DCP2	hsa-miR-2277-3p
DCP2	hsa-miR-6412
DCP2	hsa-miR-6590-3p
DCP2	hsa-miR-2435
DCP2	hsa-miR-4274
DCP2	hsa-miR-7075-3p
DCP2	hsa-miR-6914-3p
DCP2	hsa-miR-6779-3p
DCP2	hsa-miR-1286
DCP2	hsa-miR-1813
DCP2	hsa-miR-1228-3p
DCP2	hsa-miR-628-5p
DCP2	hsa-miR-7664-3p
DCP2	hsa-miR-6901-3p
DCP2	hsa-miR-216
DCP2	hsa-miR-624-3p
DCP2	hsa-miR-185-3p
DCP2	hsa-miR-7340-5p
DCP2	hsa-miR-7343-5p
DCP2	hsa-miR-3155a
DCP2	hsa-miR-6996-5p
DCP2	hsa-miR-384-3p
DCP2	hsa-miR-575
DCP2	hsa-miR-5089-3p
DCP2	hsa-miR-369-3p
DCP2	hsa-miR-4719
DCP2	hsa-miR-6715-5p
DCP2	hsa-miR-4712-3p
DCP2	hsa-miR-302
DCP2	hsa-miR-710
DCP2	hsa-miR-6899-5p
DCP2	hsa-miR-548e-5p
DCP2	hsa-miR-134
DCP2	hsa-miR-18
DCP2	hsa-miR-6811-3p
DCP2	hsa-miR-2285
DCP2	hsa-miR-7354-5p
DCP2	hsa-miR-881-3p
DCP2	hsa-miR-4645-5p
DCP2	hsa-miR-375
DCP2	hsa-miR-92-3p
DCP2	hsa-miR-4803
DCP2	hsa-miR-701-3p

DCP2	hsa-miR-660-5p
DCP2	hsa-miR-7049-3p
DCP2	hsa-miR-5701
DCP2	hsa-miR-4490
DCP2	hsa-miR-7109-3p
DCP2	hsa-miR-7111-5p
DCP2	hsa-miR-7204-3p
DCP2	hsa-miR-548bb-5p
DCP2	hsa-miR-762
DCP2	hsa-miR-200a-3p
DCP2	hsa-miR-7386g-3p
DCP2	hsa-miR-664a-3p
DCP2	hsa-miR-8874
DCP2	hsa-miR-7172-5p
DCP2	hsa-miR-181b-3p
DCP2	hsa-miR-2285n
DCP2	hsa-miR-455-5p
DCP2	hsa-miR-19a-3p
DCP2	hsa-miR-890-3p
DCP2	hsa-miR-2388-5p
DCP2	hsa-miR-7233-5p
DCP2	hsa-miR-3167-3p
DCP2	hsa-miR-664-3p
DCP2	hsa-miR-7234-5p
DCP2	hsa-miR-1185-3p
DCP2	hsa-miR-3579
DCP2	hsa-miR-496a-3p.2
DCP2	hsa-miR-7192-3p
DCP2	hsa-miR-30ad-3p
DCP2	hsa-miR-677-3p
DCP2	hsa-miR-6507-3p
DCP2	hsa-miR-3549
DCP2	hsa-let-7a-2-3p
DCP2	hsa-miR-548v
DCP2	hsa-miR-7225-3p
DCP2	hsa-miR-103a-2-5p
DCP2	hsa-miR-192-5p
DCP2	hsa-miR-7371b-3p
DCP2	hsa-miR-4293
DCP2	hsa-miR-7386h-5p
DCP2	hsa-miR-3553
DCP2	hsa-miR-2898
DCP2	hsa-miR-6123
DCP2	hsa-miR-21-3p
DCP2	hsa-miR-8061
DCP2	hsa-miR-4717-5p

DCP2	hsa-miR-129-5p
DCP2	hsa-miR-346-3p
DCP2	hsa-miR-7159-3p
DCP2	hsa-miR-6986-5p
DCP2	hsa-miR-551b-5p
DCP2	hsa-miR-4528
DCP2	hsa-miR-7578
DCP2	hsa-miR-7281-3p
DCP2	hsa-miR-4636
DCP2	hsa-miR-670
DCP2	hsa-miR-4524a-5p
DCP2	hsa-miR-152
DCP2	hsa-miR-8816
DCP2	hsa-miR-4438
DCP2	hsa-miR-190-3p
DCP2	hsa-miR-580-3p
DCP2	hsa-miR-3102
DCP2	hsa-miR-623
DCP2	hsa-miR-23c
DCP2	hsa-miR-4642
DCP2	hsa-miR-499b-3p
DCP2	hsa-miR-380-3p
DCP2	hsa-miR-7398f-3p
DCP2	hsa-miR-878-5p
DCP2	hsa-miR-1934-5p
DCP2	hsa-miR-363-3p
DCP2	hsa-miR-6942-3p
DCP2	hsa-miR-498
DCP2	hsa-miR-7307-3p
DCP2	hsa-miR-548bd-3p
DCP2	hsa-miR-101-3p.1
DCP2	hsa-miR-3927-3p
DCP2	hsa-miR-4694-3p
DCP2	hsa-miR-2400
DCP2	hsa-miR-1844
DCP2	hsa-miR-576-5p
DCP2	hsa-miR-340-5p
DCP2	hsa-miR-7156-3p
DCP2	hsa-miR-8054
DCP2	hsa-miR-4733-3p
DCP2	hsa-miR-2285z
DCP2	hsa-miR-4256
DCP2	hsa-miR-493-5p
DCP2	hsa-miR-2480
DCP2	hsa-miR-101-5p
DCP2	hsa-miR-892-5p

DCP2	hsa-miR-3088-3p
DCP2	hsa-miR-7398b-3p
DCP2	hsa-miR-365a-5p
DCP2	hsa-miR-669j
DCP2	hsa-miR-1185-5p
DCP2	hsa-miR-6843-3p
DCP2	hsa-miR-192-3p
DCP2	hsa-miR-337-3p
DCP2	hsa-miR-376a-2-5p
DCP2	hsa-miR-8843
DCP2	hsa-miR-494-3p
DCP2	hsa-miR-374a-3p
DCP2	hsa-miR-1197-3p
DCP2	hsa-miR-7260-5p
DCP2	hsa-miR-6792-3p
DCP2	hsa-miR-3678-3p
DCP2	hsa-miR-106a-3p
DCP2	hsa-miR-527
DCP2	hsa-miR-200
DCP2	hsa-miR-7703
DCP2	hsa-miR-6891-3p
DCP2	hsa-miR-550b-2-5p
DCP2	hsa-miR-6540-3p
DCP2	hsa-miR-6516
DCP2	hsa-miR-191-5p
DCP2	hsa-miR-520
DCP2	hsa-miR-4526
DCP2	hsa-miR-9a-3p
DCP2	hsa-miR-1224-5p
DCP2	hsa-miR-4536-5p
DCP2	hsa-miR-16-2-3p
DCP2	hsa-miR-8094
DCP2	hsa-miR-4698
DCP2	hsa-miR-6855-3p
DCP2	hsa-miR-7247-5p
DCP2	hsa-miR-3660
DCP2	hsa-miR-297
DCP2	hsa-miR-6999-3p
DCP2	hsa-miR-2310
DCP2	hsa-miR-3148
DCP2	hsa-miR-4797-3p
DCP2	hsa-miR-7339-3p
DCP2	hsa-miR-6898-5p
DCP2	hsa-miR-93-5p
DCP2	hsa-miR-145-3p
DCP2	hsa-miR-2364

DCP2	hsa-miR-1912
DCP2	hsa-miR-4796-3p
DCP2	hsa-miR-571
DCP2	hsa-miR-548ay-5p
DCP2	hsa-miR-626
DCP2	hsa-miR-7364-3p
DCP2	hsa-miR-7158-5p
DCP2	hsa-miR-6374
DCP2	hsa-miR-294-3p
DCP2	hsa-miR-7291-3p
DCP2	hsa-miR-7093-3p
DCP2	hsa-miR-4667
DCP2	hsa-miR-1251-3p
DCP2	hsa-miR-181-5p
DCP2	hsa-miR-323-3p
DCP2	hsa-miR-7347-3p
DCP2	hsa-miR-1252-3p
DCP2	hsa-miR-7446-5p
DCP2	hsa-miR-6080
DCP2	hsa-miR-568
DCP2	hsa-miR-4760-3p
DCP2	hsa-miR-7663-5p
DCP2	hsa-miR-548b-3p
DCP2	hsa-miR-676-3p
DCP2	hsa-miR-7245-3p
DCP2	hsa-miR-556-3p
DCP2	hsa-miR-5697-3p
DCP2	hsa-miR-2354
DCP2	hsa-miR-7019-5p
DCP2	hsa-miR-376b-5p
DCP2	hsa-miR-3149
DCP2	hsa-miR-514-3p
DCP2	hsa-miR-1948-5p
DCP2	hsa-miR-105-5p
DCP2	hsa-miR-143-5p
DCP2	hsa-miR-3116
DCP2	hsa-miR-503-5p
DCP2	hsa-miR-552-5p
DCP2	hsa-miR-8113
DCP2	hsa-miR-295-5p
DCP2	hsa-miR-205-5p
DCP2	hsa-miR-942
DCP2	hsa-miR-486-5p
DCP2	hsa-miR-1343
DCP2	hsa-miR-7006-3p
DCP2	hsa-miR-8095

DCP2	hsa-miR-6529
DCP2	hsa-miR-451-3p
DCP2	hsa-miR-6508-3p
DCP2	hsa-miR-1264
DCP2	hsa-miR-1-1-5p
DCP2	hsa-miR-7278-3p
DCP2	hsa-miR-2411-3p
DCP2	hsa-miR-5189-5p
DCP2	hsa-miR-23a-5p
DCP2	hsa-miR-193-3p
DCP2	hsa-miR-490-3p
DCP2	hsa-miR-8066
DCP2	hsa-miR-299a-5p
DCP2	hsa-miR-6085
DCP2	hsa-miR-4652-3p
DCP2	hsa-miR-2323
DCP2	hsa-miR-548h-5p
DCP2	hsa-miR-655-5p
DCP2	hsa-miR-1912-3p
DCP2	hsa-miR-4801
DCP2	hsa-miR-743-5p
DCP2	hsa-miR-1960
DCP2	hsa-miR-3672
DCP2	hsa-miR-8905
DCP2	hsa-miR-7262b-5p
DCP2	hsa-miR-636
DCP2	hsa-miR-6896-5p
DCP2	hsa-miR-518g-3p
DCP2	hsa-miR-4446-5p
DCP2	hsa-miR-6930-3p
DCP2	hsa-miR-4708-5p
DCP2	hsa-miR-2115-5p
DCP2	hsa-miR-1246
DCP2	hsa-miR-1643-3p
DCP2	hsa-miR-7351-5p
DCP2	hsa-miR-196
DCP2	hsa-miR-19
DCP2	hsa-miR-2452
DCP2	hsa-miR-4742
DCP2	hsa-miR-2347
DCP2	hsa-miR-3078-5p
DCP2	hsa-miR-199b-3-5p
DCP2	hsa-miR-4662-5p
DCP2	hsa-miR-6839-5p
DCP2	hsa-miR-298-3p
DCP2	hsa-miR-493

DCP2	hsa-miR-3577-5p
DCP2	hsa-miR-1295a
DCP2	hsa-miR-1298
DCP2	hsa-miR-545-5p
DCP2	hsa-miR-640
DCP2	hsa-miR-6797-3p
DCP2	hsa-miR-548ar-3p
DCP2	hsa-miR-3130-3p
DCP2	hsa-miR-4643
DCP2	hsa-miR-1434-3p
DCP2	hsa-miR-434-3p
DCP2	hsa-miR-216a-5p
DCP2	hsa-miR-6904-5p
DCP2	hsa-miR-3473f
DCP2	hsa-miR-4789-5p
DCP2	hsa-miR-764-3p
DCP2	hsa-miR-548c-5p
DCP2	hsa-miR-3616-5p
DCP2	hsa-miR-513
DCP2	hsa-miR-3613b
DCP2	hsa-miR-6948-3p
DCP2	hsa-miR-2315
DCP2	hsa-miR-8841
DCP2	hsa-miR-195-3p
DCP2	hsa-miR-496
DCP2	hsa-miR-29c-3p
DCP2	hsa-miR-2426
DCP2	hsa-miR-211-5p
DCP2	hsa-miR-299
DCP2	hsa-miR-7194-3p
DCP2	hsa-miR-3060-3p
DCP2	hsa-miR-6950-5p
DCP2	hsa-miR-7197-3p
DCP2	hsa-miR-4420
DCP2	hsa-miR-219b-3p
DCP2	hsa-miR-7398j-3p
DCP2	hsa-miR-27b-3p
DCP2	hsa-miR-5626-3p
DCP2	hsa-miR-383-5p.2
DCP2	hsa-miR-579
DCP2	hsa-miR-1199-5p
DCP2	hsa-miR-2284g
DCP2	hsa-miR-7059-3p
DCP2	hsa-miR-654-5p
DCP2	hsa-miR-6731-5p
DCP2	hsa-miR-612

DCP2	hsa-miR-7162-3p
DCP2	hsa-miR-511
DCP2	hsa-miR-8858
DCP2	hsa-miR-18-5p
DCP2	hsa-miR-924
DCP2	hsa-miR-1304-5p
DCP2	hsa-miR-294-5p
DCP2	hsa-miR-6844
DCP2	hsa-miR-421
DCP2	hsa-miR-127-5p
DCP2	hsa-miR-4503
DCP2	hsa-miR-103-3p
DCP2	hsa-miR-6881-5p
DCP2	hsa-miR-2317
DCP2	hsa-miR-510
DCP2	hsa-miR-6787-3p
DCP2	hsa-miR-125a-3p
DCP2	hsa-miR-7201-5p
DCP2	hsa-miR-890
DCP2	hsa-miR-8814
DCP2	hsa-miR-6756-3p
DCP2	hsa-miR-6957-5p
DCP2	hsa-miR-210-3p
DCP2	hsa-miR-6899-3p
DCP2	hsa-miR-455-5p
DCP2	hsa-miR-378b
DCP2	hsa-miR-133a-3p.1
DCP2	hsa-miR-4690-5p
DCP2	hsa-miR-7347-5p
DCP2	hsa-miR-7300-5p
DCP2	hsa-miR-371a-5p
DCP2	hsa-miR-7173-5p
DCP2	hsa-miR-7235-3p
DCP2	hsa-miR-6883-5p
DCP2	hsa-miR-3686
DCP2	hsa-miR-5590-5p
DCP2	hsa-miR-183-5p
DCP2	hsa-miR-766-3p
DCP2	hsa-miR-5006-5p
DCP2	hsa-miR-3971
DCP2	hsa-miR-1191b-3p
DCP2	hsa-miR-4328
DCP2	hsa-miR-4738-3p
DCP2	hsa-miR-2376
DCP2	hsa-miR-7321-5p
DCP2	hsa-miR-548an

DCP2	hsa-miR-452-5p
DCP2	hsa-miR-7390-3p
DCP2	hsa-miR-655
DCP2	hsa-miR-223-3p
DCP2	hsa-miR-4769-3p
DCP2	hsa-miR-6216
DCP2	hsa-miR-669d-5p
DCP2	hsa-miR-6954-3p
DCP2	hsa-miR-1-5p
DCP2	hsa-miR-582-3p
DCP2	hsa-miR-499b-5p
DCP2	hsa-miR-7119-3p
DCP2	hsa-miR-7231-3p
DCP2	hsa-miR-550a-5p
DCP2	hsa-miR-6925-3p
DCP2	hsa-miR-483-3p
DCP2	hsa-miR-423-5p
DCP2	hsa-miR-6499-3p
DCP2	hsa-miR-5691
DCP2	hsa-miR-6122-5p
DCP2	hsa-miR-3065-3p
DCP2	hsa-miR-1565
DCP2	hsa-miR-874-3p
DCP2	hsa-miR-7269-5p
DCP2	hsa-miR-7208-3p
DCP2	hsa-miR-7081-3p
DCP2	hsa-miR-4499
DCP2	hsa-miR-6971-5p
DCP2	hsa-miR-1278
DCP2	hsa-miR-106-5p
DCP2	hsa-miR-548i-3p
DCP2	hsa-miR-4751
DCP2	hsa-let-7
DCP2	hsa-miR-302a-3p
DCP2	hsa-miR-7a-2-3p
DCP2	hsa-miR-7357-5p
DCP2	hsa-miR-320-5p
DCP2	hsa-miR-150-3p
DCP2	hsa-miR-615-5p
DCP2	hsa-miR-1273e
DCP2	hsa-miR-593-5p
DCP2	hsa-miR-625-3p
DCP2	hsa-miR-6414
DCP2	hsa-miR-147a
DCP2	hsa-miR-7018-3p
DCP2	hsa-miR-3600

DCP2	hsa-miR-92a-1-5p
DCP2	hsa-miR-5615-3p
DCP2	hsa-miR-6834-5p
DCP2	hsa-miR-409b
DCP2	hsa-miR-7056-3p
DCP2	hsa-miR-6528
DCP2	hsa-miR-186-5p
DCP2	hsa-miR-2338
DCP2	hsa-miR-6362
DCP2	hsa-miR-3194-3p
DCP2	hsa-miR-2443
DCP2	hsa-miR-3557-5p
DCP2	hsa-miR-7308-3p
DCP2	hsa-miR-2284r
DCP2	hsa-miR-294
DCP2	hsa-miR-8813
DCP2	hsa-miR-6769a-3p
DCP2	hsa-miR-601
DCP2	hsa-miR-460-5p
DCP2	hsa-miR-222-3p
DCP2	hsa-miR-624-5p
DCP2	hsa-miR-7373-5p
DCP2	hsa-miR-6806-3p
DCP2	hsa-miR-500a-5p
DCP2	hsa-miR-3127-3p
DCP2	hsa-miR-344i
DCP2	hsa-miR-6876-5p
DCP2	hsa-miR-2278
DCP2	hsa-miR-93-5p
DCP2	hsa-miR-7186-3p
DCP2	hsa-miR-4705
DCP2	hsa-miR-1193-3p
DCP2	hsa-miR-2284b
DCP2	hsa-miR-7336-5p
DCP2	hsa-miR-679-5p
DCP2	hsa-miR-4766-5p
DCP2	hsa-miR-7398-3p
DCP2	hsa-miR-6345
DCP2	hsa-miR-1942
DCP2	hsa-miR-4697-3p
DCP2	hsa-miR-7054-5p
DCP2	hsa-miR-6907-5p
DCP2	hsa-miR-7160-5p
DCP2	hsa-miR-3168
DCP2	hsa-miR-4744
DCP2	hsa-miR-2295

DCP2	hsa-miR-3065-5p
DCP2	hsa-miR-324-3p
DCP2	hsa-miR-7152-5p
DCP2	hsa-miR-22-5p
DCP2	hsa-miR-889-5p
DCP2	hsa-miR-470-5p
DCP2	hsa-miR-7691
DCP2	hsa-miR-323
DCP2	hsa-miR-6821-3p
DCP2	hsa-miR-384-5p
DCP2	hsa-miR-6481
DCP2	hsa-miR-1237-3p
DCP2	hsa-miR-1544-3p
DCP2	hsa-miR-6801-5p
DCP2	hsa-miR-1291
DCP2	hsa-miR-1434-5p
DCP2	hsa-miR-3560
DCP2	hsa-miR-6943-5p
DCP2	hsa-miR-4699-5p
DCP2	hsa-miR-8068
DCP2	hsa-miR-1948-3p
DCP2	hsa-miR-7317-3p
DCP2	hsa-miR-6416-3p
DCP2	hsa-miR-3074-3p
DCP2	hsa-miR-34b-3p
DCP2	hsa-miR-7039-3p
DCP2	hsa-miR-7314-3p
DCP2	hsa-miR-8073
DCP2	hsa-miR-7006-5p
DCP2	hsa-miR-466b-5p
DCP2	hsa-miR-548az-5p
DCP2	hsa-miR-6895-5p
DCP2	hsa-miR-3472
DCP2	hsa-miR-1612
DCP2	hsa-miR-4536-5p
DCP2	hsa-miR-622
DCP2	hsa-miR-214-3p
DCP2	hsa-miR-3078-3p
DCP2	hsa-miR-1273h-5p
DCP2	hsa-miR-2117
DCP2	hsa-miR-548as-3p
DCP2	hsa-miR-290a-5p
DCP2	hsa-miR-349
DCP2	hsa-miR-5684
DCP2	hsa-miR-4762-3p
DCP2	hsa-miR-466h-3p

DCP2	hsa-miR-6927-3p
DCP2	hsa-miR-128-5p
DCP2	hsa-miR-290b-5p
DCP2	hsa-miR-7675-3p
DCP2	hsa-miR-25-3p
DCP2	hsa-miR-3190-5p
DCP2	hsa-miR-1179
DCP2	hsa-miR-27a-3p
DCP2	hsa-miR-7272-5p
DCP2	hsa-miR-6778-5p
DCP2	hsa-miR-6826-3p
DCP2	hsa-miR-7398-5p
DCP2	hsa-miR-4684-5p
DCP2	hsa-miR-6946-3p
DCP2	hsa-miR-7352-3p
DCP2	hsa-miR-7212-3p
DCP2	hsa-miR-200b-5p
DCP2	hsa-miR-6376
DCP2	hsa-miR-6321
DCP2	hsa-miR-1843a-5p
DCP2	hsa-miR-4477a
DCP2	hsa-miR-7024-5p
DCP2	hsa-miR-466ade-3p
DCP2	hsa-miR-3185
DCP2	hsa-miR-3651
DCP2	hsa-miR-2114-5p
DCP2	hsa-miR-605-5p
DCP2	hsa-miR-7201-3p
DCP2	hsa-miR-500-3p
DCP2	hsa-miR-2285r
DCP2	hsa-miR-300
DCP2	hsa-miR-7286-3p
DCP2	hsa-miR-1256
DCP2	hsa-miR-6905-3p
DCP2	hsa-miR-4715-3p
DCP2	hsa-miR-7117-3p
DCP2	hsa-miR-6870-3p
DCP2	hsa-miR-1905
DCP2	hsa-miR-373-5p
DCP2	hsa-miR-4653-5p
DCP2	hsa-miR-7398n-3p
DCP2	hsa-miR-2471-3p
DCP2	hsa-miR-6888-3p
DCP2	hsa-let-7c-3p
DCP2	hsa-miR-1257
DCP2	hsa-miR-520-5p

DCP2	hsa-miR-203-3p.2
DCP2	hsa-miR-2394
DCP2	hsa-miR-6529-5p
DCP2	hsa-miR-2284z
DCP2	hsa-miR-4666b
DCP2	hsa-miR-6776-3p
DCP2	hsa-miR-7202-3p
DCP2	hsa-miR-3571
DCP2	hsa-miR-495-3p
DCP2	hsa-miR-7195-3p
DCP2	hsa-miR-548l
DCP2	hsa-miR-2464-3p
DCP2	hsa-miR-203b-3p
DCP2	hsa-miR-7094-3p
DCP2	hsa-miR-8838
DCP2	hsa-miR-6860
DCP2	hsa-miR-323b-5p
DCP2	hsa-miR-20-3p
DCP2	hsa-miR-694
DCP2	hsa-miR-7183-3p
DCP2	hsa-miR-7352-5p
DCP2	hsa-miR-3691-3p
DCP2	hsa-miR-466f-5p
DCP2	hsa-miR-876
DCP2	hsa-miR-3099
DCP2	hsa-miR-100-3p
DCP2	hsa-miR-4680-3p
DCP2	hsa-miR-6317
DCP2	hsa-miR-22-3p
DCP2	hsa-miR-669i
DCP2	hsa-miR-4452
DCP2	hsa-miR-6878-5p
DCP2	hsa-miR-4446-3p
DCP2	hsa-miR-1544b-5p
DCP2	hsa-miR-654-3p
DCP2	hsa-miR-880-5p
DCP2	hsa-miR-552-3p
DCP2	hsa-miR-1903
DCP2	hsa-miR-6752-3p
DCP2	hsa-miR-5620-3p
DCP2	hsa-miR-4264
DCP2	hsa-miR-3152-3p
DCP2	hsa-miR-7054-3p
DCP2	hsa-miR-3966
DCP2	hsa-miR-2284y
DCP2	hsa-miR-2113

DCP2	hsa-miR-1a-5p
DCP2	hsa-miR-3544-5p
DCP2	hsa-miR-6875-3p
DCP2	hsa-miR-6988-5p
DCP2	hsa-miR-380
DCP2	hsa-miR-3064-5p
DCP2	hsa-miR-204-5p
DCP2	hsa-miR-556-5p
DCP2	hsa-miR-4699-3p
DCP2	hsa-miR-4476
DCP2	hsa-miR-1397-5p
DCP2	hsa-miR-661
DCP2	hsa-miR-382-3p
DCP2	hsa-miR-3925-5p
DCP2	hsa-miR-6387
DCP2	hsa-miR-627-5p
DCP2	hsa-miR-7355-3p
DCP2	hsa-miR-1655-3p
DCP2	hsa-miR-29b-1-5p
DCP2	hsa-miR-2681-3p
DCP2	hsa-miR-4524-5p
DCP2	hsa-miR-6505-5p
DCP2	hsa-miR-1295a
DCP2	hsa-miR-6817-5p
DCP2	hsa-miR-210-5p
DCP2	hsa-miR-1255b-5p
DCP2	hsa-miR-7706
DCP2	hsa-miR-218-1-3p
DCP2	hsa-miR-7191-5p
DCP2	hsa-miR-7398p-3p
DCP2	hsa-miR-484
DCP2	hsa-miR-532-3p
DCP2	hsa-miR-181
DCP2	hsa-miR-7206-3p
DCP2	hsa-miR-3071-5p
DCP2	hsa-miR-6750-3p
DCP2	hsa-miR-3570
DCP2	hsa-miR-135
DCP2	hsa-miR-409-3p
DCP2	hsa-miR-6701-3p
DCP2	hsa-miR-148-5p
DCP2	hsa-miR-1197
DCP2	hsa-miR-152-3p
DCP2	hsa-miR-6882-3p
DCP2	hsa-miR-3614-5p
DCP2	hsa-miR-193a-5p

DCP2	hsa-miR-7363-5p
DCP2	hsa-miR-542-3p
DCP2	hsa-miR-24-3p
DCP2	hsa-miR-4695-5p
DCP2	hsa-miR-3611
DCP2	hsa-miR-211-5p
DCP2	hsa-miR-21-5p
DCP2	hsa-miR-7974
DCP2	hsa-miR-5591-3p
DCP2	hsa-miR-4482-3p
DCP2	hsa-miR-7398i-3p
DCP2	hsa-miR-7073-3p
DCP2	hsa-miR-6076
DCP2	hsa-miR-1954
DCP2	hsa-miR-7069-5p
DCP2	hsa-miR-487a
DCP2	hsa-miR-7312-5p
DCP2	hsa-miR-1958
DCP2	hsa-miR-1929-3p
DCP2	hsa-miR-26-3-3p
DCP2	hsa-miR-4728-3p
DCP2	hsa-miR-190a-5p
DCP2	hsa-miR-670-3p
DCP2	hsa-miR-365a-3p
DCP2	hsa-miR-344eh-5p
DCP2	hsa-miR-8808
DCP2	hsa-miR-2284v
DCP2	hsa-miR-8832
DCP2	hsa-miR-5692bc
DCP2	hsa-miR-1255-5p
DCP2	hsa-miR-429
DCP2	hsa-miR-4668-3p
DCP2	hsa-miR-381-5p
DCP2	hsa-miR-130-3p
DCP2	hsa-miR-137
DCP2	hsa-miR-3073a-3p
DCP2	hsa-miR-548bb-3p
DCP2	hsa-miR-582-5p
DCP2	hsa-miR-487a-5p
DCP2	hsa-miR-550-5p
DCP2	hsa-miR-7291-5p
DCP2	hsa-miR-6737-5p
DCP2	hsa-miR-212-5p
DCP2	hsa-miR-2477
DCP2	hsa-miR-3653-3p
DCP2	hsa-miR-1b-5p

DCP2	hsa-miR-550a-3p
DCP2	hsa-miR-3062-5p
DCP2	hsa-miR-3199
DCP2	hsa-miR-687
DCP2	hsa-miR-3688-5p
DCP2	hsa-miR-4673
DCP2	hsa-miR-7374-5p
DCP2	hsa-miR-1296
DCP2	hsa-miR-6945-3p
DCP2	hsa-miR-451a
DCP2	hsa-miR-7267-3p
DCP2	hsa-miR-7206-5p
DCP2	hsa-miR-505-3p
DCP2	hsa-miR-208-5p
DCP2	hsa-miR-518a-5p
DCP2	hsa-miR-4720-3p
DCP2	hsa-miR-5697
DCP2	hsa-miR-3913-3p
DCP2	hsa-miR-185-5p
DCP2	hsa-miR-547-5p
DCP2	hsa-miR-1304-3p
DCP2	hsa-miR-8881
DCP2	hsa-miR-3200-5p
DCP2	hsa-miR-872-3p
DCP2	hsa-miR-4443
DCP2	hsa-miR-350-3p
DCP2	hsa-miR-370-3p
DCP2	hsa-miR-590-5p
DCP2	hsa-miR-5584-5p
DCP2	hsa-miR-3606-3p
DCP2	hsa-miR-883a-5p
DCP2	hsa-miR-6512-5p
DCP2	hsa-miR-218a-1-3p
DCP2	hsa-miR-208-3p
DCP2	hsa-miR-3618-3p
DCP2	hsa-miR-1545b-5p
DCP2	hsa-miR-1273d
DCP2	hsa-miR-2362
DCP2	hsa-miR-6753-5p
DCP2	hsa-miR-8908-3p
DCP2	hsa-miR-2355-5p
DCP2	hsa-miR-128-3p
DCP2	hsa-miR-4516
DCP2	hsa-miR-651
DCP2	hsa-miR-542-5p
DCP2	hsa-miR-7865

DCP2	hsa-miR-8069
DCP2	hsa-miR-1252-5p
DCP2	hsa-miR-892b
DCP2	hsa-miR-6793-3p
DCP2	hsa-miR-326-3p
DCP2	hsa-miR-548d-3p
DCP2	hsa-miR-182-3p
DCP2	hsa-miR-8071
DCP2	hsa-miR-6939-5p
DCP2	hsa-miR-6848-3p
DCP2	hsa-miR-545-3p
DCP2	hsa-miR-3595
DCP2	hsa-miR-351-5p
DCP2	hsa-miR-5007-3p
DCP2	hsa-miR-4645-3p
DCP2	hsa-miR-5003-3p
DCP2	hsa-miR-3127-5p
DCP2	hsa-miR-514b-5p
DCP2	hsa-miR-19b-3p
DCP2	hsa-miR-5132-5p
DCP2	hsa-miR-145-5p
DCP2	hsa-miR-206-3p
DCP2	hsa-miR-203-3p.1
DCP2	hsa-miR-6981-5p
DCP2	hsa-miR-3071-3p
DCP2	hsa-miR-2285h
DCP2	hsa-miR-129-5p
DCP2	hsa-miR-103-5p
DCP2	hsa-miR-6837-3p
DCP2	hsa-miR-138-1-3p
DCP2	hsa-miR-411-5p.1
DCP2	hsa-miR-936
DCP2	hsa-miR-6388
DCP2	hsa-miR-4653-3p
DCP2	hsa-miR-7045-3p
DCP2	hsa-miR-4482-5p
DCP2	hsa-miR-7287-5p
DCP2	hsa-miR-8092
DCP2	hsa-miR-5195-5p
DCP2	hsa-miR-34bc-3p
DCP2	hsa-miR-6868-5p
DCP2	hsa-miR-4324
DCP2	hsa-miR-153-5p
DCP2	hsa-miR-7253-5p
DCP2	hsa-miR-299-5p
DCP2	hsa-miR-5192

DCP2	hsa-miR-6871-3p
DCP2	hsa-miR-548t-3p
DCP2	hsa-miR-6917-3p
DCP2	hsa-miR-8831
DCP2	hsa-miR-1898
DCP2	hsa-miR-2332
DCP2	hsa-miR-2368-3p
DCP2	hsa-miR-1233-3p
DCP2	hsa-miR-204-3p
DCP2	hsa-miR-3182
DCP2	hsa-miR-7359-5p
DCP2	hsa-miR-922
DCP2	hsa-miR-185-5p
DCP2	hsa-miR-154b
DCP2	hsa-miR-706
DCP2	hsa-miR-1303
DCP2	hsa-miR-7092-3p
DCP2	hsa-miR-30c-2-3p
DCP2	hsa-miR-372-5p
DCP2	hsa-miR-202-5p
DCP2	hsa-miR-23
DCP2	hsa-miR-1266-3p
DCP2	hsa-miR-33
DCP2	hsa-miR-7193-3p
DCP2	hsa-miR-2312
DCP2	hsa-miR-3136-5p
DCP2	hsa-miR-361-5p
DCP2	hsa-miR-323b-3p
DCP2	hsa-miR-1-3p
DCP2	hsa-miR-666-3p
DCP2	hsa-miR-619-5p
DCP2	hsa-miR-22-3p
DCP2	hsa-miR-2902
DCP2	hsa-miR-3652
DCP2	hsa-miR-101-3p
DCP2	hsa-miR-6986-3p
DCP2	hsa-miR-28-3p
DCP2	hsa-miR-548ax
DCP2	hsa-miR-2461-3p
DCP2	hsa-miR-7081-5p
DCP2	hsa-miR-3915
DCP2	hsa-miR-6130
DCP2	hsa-miR-7343-3p
DCP2	hsa-miR-216b
DCP2	hsa-miR-6418-3p
DCP2	hsa-miR-589-3p

DCP2	hsa-miR-6932-5p
DCP2	hsa-miR-6316
DCP2	hsa-miR-7392-3p
DCP2	hsa-miR-7064-3p
DCP2	hsa-miR-607
DCP2	hsa-miR-150-5p
DCP2	hsa-miR-7063-3p
DCP2	hsa-miR-505-3p.2
DCP2	hsa-miR-4742-5p
DCP2	hsa-miR-382-5p
DCP2	hsa-miR-603
DCP2	hsa-miR-7309-5p
DCP2	hsa-miR-2284s
DCP2	hsa-miR-181a-3p
DCP2	hsa-miR-3108-5p
DCP2	hsa-miR-5009-5p
DCP2	hsa-miR-577-5p
DCP2	hsa-miR-3689a-5p
DCP2	hsa-miR-181b-1-3p
DCP2	hsa-miR-888-5p
DCP2	hsa-miR-1207-5p
DCP2	hsa-miR-2305
DCP2	hsa-miR-330-3p.2
DCP2	hsa-miR-3589
DCP2	hsa-miR-6830-5p
DCP2	hsa-miR-2285t
DCP2	hsa-miR-485-3p
DCP2	hsa-miR-703
DCP2	hsa-miR-2896
DCP2	hsa-miR-5193
DCP2	hsa-miR-6073
DCP2	hsa-miR-216c-5p
DCP2	hsa-miR-7014-5p
DCP2	hsa-miR-4685-5p
DCP2	hsa-miR-196b-3p
DCP2	hsa-miR-7010-3p
DCP2	hsa-miR-188-5p
DCP2	hsa-miR-7689-3p
DCP2	hsa-miR-7041-3p
DCP2	hsa-miR-633
DCP2	hsa-miR-6817-3p
DCP2	hsa-miR-5692a
DCP2	hsa-miR-26-2-3p
DCP2	hsa-miR-130
DCP2	hsa-miR-4423-5p
DCP2	hsa-miR-448-3p

DCP2	hsa-miR-3547-3p
DCP2	hsa-miR-548at-5p
DCP2	hsa-miR-5682
DCP2	hsa-miR-330-5p
DCP2	hsa-miR-4659a-3p
DCP2	hsa-miR-1287-3p
DCP2	hsa-miR-3167-5p
DCP2	hsa-miR-10b-3p
DCP2	hsa-miR-4305
DCP2	hsa-miR-96-5p
DCP2	hsa-miR-544a
DCP2	hsa-miR-6906-5p
DCP2	hsa-miR-7180-3p
DCP2	hsa-miR-3914
DCP2	hsa-miR-7032-3p
DCP2	hsa-miR-376c-5p
DCP2	hsa-miR-204-5p
DCP2	hsa-miR-4287
DCP2	hsa-miR-8869
DCP2	hsa-miR-7245-5p
DCP2	hsa-miR-2415-5p
DCP2	hsa-miR-520-3p
DCP2	hsa-miR-4501
DCP2	hsa-miR-562
DCP2	hsa-miR-613
DCP2	hsa-miR-802
DCP2	hsa-miR-669e-3p
DCP2	hsa-miR-3174
DCP2	hsa-miR-3137
DCP2	hsa-miR-6940-3p
DCP2	hsa-miR-3072-5p
DCP2	hsa-miR-7013-5p
DCP2	hsa-miR-1255b-2-3p
DCP2	hsa-miR-126a-5p
DCP2	hsa-miR-8082
DCP2	hsa-miR-433-3p
DCP2	hsa-miR-411-5p
DCP2	hsa-miR-5621-3p
DCP2	hsa-miR-6809-5p
DCP2	hsa-miR-28-5p
DCP2	hsa-miR-7195-5p
DCP2	hsa-miR-6780a-3p
DCP2	hsa-miR-628-3p
DCP2	hsa-miR-7386a-3p
DCP2	hsa-miR-3159
DCP2	hsa-miR-4513

DCP2	hsa-miR-1247-5p
DCP2	hsa-miR-3529-3p
DCP2	hsa-miR-698-5p
DCP2	hsa-miR-3138
DCP2	hsa-miR-7334-3p
DCP2	hsa-miR-676-3p
DCP2	hsa-miR-1240
DCP2	hsa-miR-1258-5p
DCP2	hsa-miR-2284u
DCP2	hsa-miR-5093
DCP2	hsa-miR-3143
DCP2	hsa-miR-7214-5p
DCP2	hsa-miR-570-5p
DCP2	hsa-miR-3058-3p
DCP2	hsa-miR-934-5p
DCP2	hsa-miR-338-3p
DCP2	hsa-miR-3130-5p
DCP2	hsa-miR-4727-5p
DCP2	hsa-miR-6950-3p
DCP2	hsa-miR-8857
DCP2	hsa-miR-6770-5p
DCP2	hsa-miR-4736
DCP2	hsa-miR-142
DCP2	hsa-miR-567
DCP2	hsa-miR-3604
DCP2	hsa-miR-30e-3p
DCP2	hsa-miR-6808-5p
DCP2	hsa-miR-2365
DCP2	hsa-miR-495-5p
DCP2	hsa-miR-433
DCP2	hsa-miR-3658
DCP2	hsa-miR-4677-5p
DCP2	hsa-miR-4317
DCP2	hsa-miR-138-5p
DCP2	hsa-miR-7115-3p
DCP2	hsa-miR-8802
DCP2	hsa-miR-4659b-3p
DCP2	hsa-miR-466b-2-3p
DCP2	hsa-miR-19-3p
DCP2	hsa-miR-138-2-3p
DCP2	hsa-miR-106
DCP2	hsa-miR-7641
DCP2	hsa-miR-4432
DCP2	hsa-miR-3150b-3p
DCP2	hsa-miR-7650-3p
DCP2	hsa-miR-4778-3p

DCP2	hsa-miR-520g-3p
DCP2	hsa-miR-501-3p
DCP2	hsa-miR-8084
DCP2	hsa-miR-4683
DCP2	hsa-miR-350
DCP2	hsa-miR-29-5p
DCP2	hsa-miR-7280-5p
DCP2	hsa-miR-224-5p
DCP2	hsa-miR-19-5p
DCP2	hsa-miR-411a
DCP2	hsa-miR-4771
DCP2	hsa-miR-2292
DCP2	hsa-miR-1588
DCP2	hsa-miR-8865
DCP2	hsa-miR-107-5p
DCP2	hsa-miR-7072-3p
DCP2	hsa-miR-2285s
DCP2	hsa-miR-6902-5p
DCP2	hsa-miR-6504-3p
DCP2	hsa-miR-873-5p.1
DCP2	hsa-miR-4433b-3p
DCP2	hsa-miR-3928-5p
DCP2	hsa-miR-6981-3p
DCP2	hsa-miR-302a-5p
DCP2	hsa-miR-767-5p
DCP2	hsa-miR-215
DCP2	hsa-miR-550b-3p
DCP2	hsa-miR-5088-3p
DCP2	hsa-miR-4766-3p
DCP2	hsa-miR-3061-3p
DCP2	hsa-miR-2284m
DCP2	hsa-miR-214-5p
DCP2	hsa-miR-519-3p
DCP2	hsa-miR-522-3p
DCP2	hsa-miR-1542-5p
DCP2	hsa-miR-1545c-3p
DCP2	hsa-miR-3664-5p
DCP2	hsa-miR-7210-5p
DCP2	hsa-miR-1323-5p
DCP2	hsa-miR-3688-3p
DCP2	hsa-miR-1972
DCP2	hsa-miR-6631-5p
DCP2	hsa-miR-1238-3p
DCP2	hsa-miR-345-3p
DCP2	hsa-miR-7398p-5p
DCP2	hsa-miR-1774

DCP2	hsa-miR-6506-3p
DCP2	hsa-miR-98-3p
DCP2	hsa-miR-343
DCP2	hsa-miR-7398a-3p
DCP2	hsa-miR-1265
DCP2	hsa-miR-1295b-5p
DCP2	hsa-miR-6826-5p
DCP2	hsa-miR-4269
DCP2	hsa-miR-33-5p
DCP2	hsa-miR-7113-5p
DCP2	hsa-miR-2284x
DCP2	hsa-miR-7052-3p
DCP2	hsa-miR-4789-3p
DCP2	hsa-miR-1206
DCP2	hsa-miR-499b-3p
DCP2	hsa-miR-1902
DCP2	hsa-miR-6931-3p
DCP2	hsa-miR-6079
DCP2	hsa-miR-4695-3p
DCP2	hsa-miR-4763-5p
DCP2	hsa-miR-218-5p
DCP2	hsa-miR-627-3p
DCP2	hsa-miR-676-5p
DCP2	hsa-miR-497-5p
DCP2	hsa-miR-6417
DCP2	hsa-miR-197-3p
DCP2	hsa-miR-878
DCP2	hsa-miR-577
DCP2	hsa-miR-4464
DCP2	hsa-miR-7078-5p
DCP2	hsa-miR-2293
DCP2	hsa-miR-216-3p
DCP2	hsa-miR-4768-3p
DCP2	hsa-miR-6883-3p
DCP2	hsa-miR-8898
DCP2	hsa-miR-3973
DCP2	hsa-miR-7198-3p
DCP2	hsa-miR-153-3p
DCP2	hsa-miR-6792-5p
DCP2	hsa-miR-154-5p
DCP2	hsa-miR-7188-3p
DCP2	hsa-miR-3074-3p
DCP2	hsa-miR-4654
DCP2	hsa-miR-650d
DCP2	hsa-miR-5572
DCP2	hsa-miR-1545b-3p

DCP2	hsa-miR-7358-3p
DCP2	hsa-miR-520gh
DCP2	hsa-miR-1250-3p
DCP2	hsa-miR-217-5p
DCP2	hsa-miR-3680-3p
DCP2	hsa-miR-7017-3p
DCP2	hsa-miR-361-5p
DCP2	hsa-miR-4716-5p
DCP2	hsa-miR-5110
DCP2	hsa-miR-3140-5p
DCP2	hsa-miR-212-3p
DCP2	hsa-miR-203-3p
DCP2	hsa-miR-15
DCP2	hsa-miR-331-3p
DCP2	hsa-miR-8077
DCP2	hsa-miR-6332
DCP2	hsa-miR-133b
DCP2	hsa-miR-3089-3p
DCP2	hsa-miR-875-5p
DCP2	hsa-miR-135a-1-3p
DCP2	hsa-miR-8110
DCP2	hsa-miR-7004-3p
DCP2	hsa-miR-93-3p
DCP2	hsa-miR-2284a
DCP2	hsa-miR-190b-3p
DCP2	hsa-miR-211-3p
DCP2	hsa-miR-4729
DCP2	hsa-miR-2352
DCP2	hsa-miR-7163-5p
DCP2	hsa-miR-224-3p
DCP2	hsa-miR-6857-3p
DCP2	hsa-miR-7219-5p
DCP2	hsa-miR-670-5p
DCP2	hsa-miR-151-3p
DCP2	hsa-miR-7668-5p
DCP2	hsa-miR-7667-3p
DCP2	hsa-miR-336-5p
DCP2	hsa-miR-3101-3p
DCP2	hsa-miR-3117-5p
DCP2	hsa-miR-3977
DCP2	hsa-miR-6519
DCP2	hsa-miR-3102-3p.2-3p
DCP2	hsa-miR-5623-3p
DCP2	hsa-miR-5010-3p
DCP2	hsa-miR-133-5p
DCP2	hsa-miR-6071

DCP2	hsa-miR-302-3p
DCP2	hsa-miR-513a-3p
DCP2	hsa-miR-548ak
DCP2	hsa-miR-8863
DCP2	hsa-miR-9b-3p
DCP2	hsa-miR-7236-5p
DCP2	hsa-miR-9-5p
DCP2	hsa-miR-141-3p
DCP2	hsa-miR-526b-5p
DCP2	hsa-miR-6128
DCP2	hsa-miR-548b-5p
DCP2	hsa-miR-4666a-5p
DCP2	hsa-miR-548au-5p
DCP2	hsa-miR-7646-3p
DCP2	hsa-miR-7205-3p
DCP2	hsa-miR-943
DCP2	hsa-miR-196a-3p
DCP2	hsa-miR-6330
DCP2	hsa-miR-6996-3p
DCP2	hsa-miR-2284-3p
DCP2	hsa-miR-7254-5p
DCP2	hsa-miR-302-5p
DCP2	hsa-miR-3140-3p
DCP2	hsa-miR-6742-3p
DCP2	hsa-miR-2284ac
DCP2	hsa-miR-1180-5p
DCP2	hsa-miR-3587
DCP2	hsa-miR-4776-3p
DCP2	hsa-miR-6503-3p
DCP2	hsa-miR-133-3p
DCP2	hsa-miR-7246-5p
DCP2	hsa-miR-1287-5p
DCP2	hsa-miR-1237-3p
DCP2	hsa-miR-5125-5p
DCP2	hsa-miR-7670-5p
DCP2	hsa-miR-3470a
DCP2	hsa-miR-548j-5p
DCP2	hsa-miR-122
DCP2	hsa-miR-466b-3p
DCP2	hsa-miR-7383-5p
DCP2	hsa-miR-642
DCP2	hsa-miR-139-3p
DCP2	hsa-miR-3117-3p
DCP2	hsa-miR-3607-3p
DCP2	hsa-miR-6989-3p
DCP2	hsa-miR-7307-5p

DCP2	hsa-miR-1929-5p
DCP2	hsa-miR-7399-5p
DCP2	hsa-miR-3929
DCP2	hsa-miR-6571-5p
DCP2	hsa-miR-6515-5p
DCP2	hsa-miR-4687-5p
DCP2	hsa-miR-137-3p
DCP2	hsa-miR-208a-5p
DCP2	hsa-miR-7339-5p
DCP2	hsa-miR-7222-3p
DCP2	hsa-miR-7049-5p
DCP2	hsa-miR-7016-3p
DCP2	hsa-miR-470-3p
DCP2	hsa-miR-4276
DCP2	hsa-miR-200bc
DCP2	hsa-miR-6911-3p
DCP2	hsa-miR-4325
DCP2	hsa-miR-6813-5p
DCP2	hsa-miR-577-3p
DCP2	hsa-miR-325-3p
DCP2	hsa-miR-7360-5p
DCP2	hsa-miR-742-3p
DCP2	hsa-miR-378j
DCP2	hsa-miR-4732-3p
DCP2	hsa-miR-6728-3p
DCP2	hsa-miR-8829
DCP2	hsa-miR-548av-3p
DCP2	hsa-miR-7175-3p
DCP2	hsa-miR-136-5p
DCP2	hsa-miR-495-3p
DCP2	hsa-miR-2470
DCP2	hsa-miR-9-3p
DCP2	hsa-miR-1892
DCP2	hsa-miR-7175-5p
DCP2	hsa-miR-98-5p
DCP2	hsa-miR-6876-3p
DCP2	hsa-miR-7385b-3p
DCP2	hsa-miR-7077-3p
DCP2	hsa-miR-339-5p
DCP2	hsa-miR-4704-3p
DCP2	hsa-miR-205-3p
DCP2	hsa-miR-4709-3p
DCP2	hsa-miR-4504
DCP2	hsa-miR-641
DCP2	hsa-miR-578
DCP2	hsa-miR-6791-3p

DCP2	hsa-miR-1839-3p
DCP2	hsa-miR-690
DCP2	hsa-miR-2298
DCP2	hsa-miR-548c-3p
DCP2	hsa-miR-6400
DCP2	hsa-miR-26
DCP2	hsa-miR-548h-3p
DCP2	hsa-miR-873-5p
DCP2	hsa-miR-374c-5p
DCP2	hsa-miR-7843-3p
DCP2	hsa-miR-7301-5p
DCP2	hsa-miR-6529b
DCP2	hsa-miR-7373b-3p
DCP2	hsa-miR-508-3p
DCP2	hsa-miR-184-5p
DCP2	hsa-miR-7088-5p
DCP2	hsa-miR-6119-3p
DCP2	hsa-miR-6937-5p
DCP2	hsa-miR-1269a
DCP2	hsa-miR-7660-3p
DCP2	hsa-miR-4266
DCP2	hsa-miR-152-5p
DCP2	hsa-miR-8847
DCP2	hsa-miR-107
DCP2	hsa-miR-5581-3p
DCP2	hsa-miR-5132-3p
DCP2	hsa-miR-222-3p
DCP2	hsa-miR-4765
DCP2	hsa-miR-337-3p
DCP2	hsa-miR-344a-3p
DCP2	hsa-miR-8055
DCP2	hsa-miR-4795-3p
DCP2	hsa-miR-615-3p
DCP2	hsa-miR-377-3p
DCP2	hsa-miR-5582-5p
DCP2	hsa-miR-2442
DCP2	hsa-miR-504-3p
DCP2	hsa-miR-7243-3p
DCP2	hsa-miR-873-5p
DCP2	hsa-miR-2441
DCP2	hsa-miR-7232-5p
DCP2	hsa-miR-15c-3p
DCP2	hsa-miR-6880-5p
DCP2	hsa-miR-5622-3p
DCP2	hsa-miR-548i
DCP2	hsa-miR-5118

DCP2	hsa-miR-6754-5p
DCP2	hsa-miR-7166-5p
DCP2	hsa-miR-650abc
DCP2	hsa-miR-548am-5p
DCP2	hsa-miR-7050-3p
DCP2	hsa-miR-7053-3p
DCP2	hsa-miR-541-5p
DCP2	hsa-miR-764-5p
DCP2	hsa-miR-6717-5p
DCP2	hsa-miR-1962
DCP2	hsa-miR-7048-3p
DCP2	hsa-miR-7156-5p
DCP2	hsa-miR-8074
DCP2	hsa-miR-3692-3p
DCP2	hsa-miR-2345
DCP2	hsa-miR-215-3p
DCP2	hsa-miR-761
DCP2	hsa-miR-143-3p
DCP2	hsa-miR-29a-3p
DCP2	hsa-miR-4461
DCP2	hsa-miR-3691-5p
DCP2	hsa-miR-6836-3p
DCP2	hsa-miR-21-5p
DCP2	hsa-miR-128
DCP2	hsa-miR-3622a-5p
DCP2	hsa-miR-181a-2-3p
DCP2	hsa-miR-5619-5p
DCP2	hsa-miR-4272
DCP2	hsa-miR-2416
DCP2	hsa-miR-424-5p
DCP2	hsa-miR-7174-5p
DCP2	hsa-miR-548f-3p
DCP2	hsa-miR-4260
DCP2	hsa-miR-7157-5p
DCP2	hsa-miR-450b-5p
DCP2	hsa-miR-548j-3p
DCP2	hsa-miR-4670-3p
DCP2	hsa-miR-4709-5p
DCP2	hsa-miR-1271-3p
DCP2	hsa-miR-410-3p
DCP2	hsa-miR-8888
DCP2	hsa-miR-2446
DCP2	hsa-miR-6972-3p
DCP2	hsa-miR-2115-3p
DCP2	hsa-miR-758-5p
DCP2	hsa-miR-550a-3-5p

DCP2	hsa-miR-7285-5p
DCP2	hsa-miR-7398k-5p
DCP2	hsa-miR-3689e
DCP2	hsa-miR-6403
DCP2	hsa-miR-6985-5p
DCP2	hsa-miR-2285u
DCP2	hsa-miR-6862-5p
DCP2	hsa-miR-582-5p
DCP2	hsa-miR-6529a
DCP2	hsa-miR-552
DCP2	hsa-miR-2284k
DCP2	hsa-miR-3150a-5p
DCP2	hsa-miR-6842-5p
DCP2	hsa-miR-376b-3p
DCP2	hsa-miR-7001-3p
DCP2	hsa-miR-3117-3p
DCP2	hsa-miR-557
DCP2	hsa-miR-702-3p
DCP2	hsa-miR-6541
DCP2	hsa-miR-674-3p
DCP2	hsa-miR-32-3p
DCP2	hsa-miR-4515
DCP2	hsa-miR-140-5p
DCP2	hsa-miR-651-3p
DCP2	hsa-miR-7297-5p
DCP2	hsa-miR-194-3p
DCP2	hsa-miR-6127
DCP2	hsa-miR-3692-5p
DCP2	hsa-miR-548n
DCP2	hsa-miR-2285e
DCP2	hsa-miR-4530
DCP2	hsa-miR-195-5p
DCP2	hsa-miR-7-3p
DCP2	hsa-miR-338-5p
DCP2	hsa-miR-1388-3p
DCP2	hsa-miR-200c-5p
DCP2	hsa-miR-4470
DCP2	hsa-miR-6536
DCP2	hsa-miR-140-3p.1
DCP2	hsa-miR-8836
DCP2	hsa-miR-1271-5p
DCP2	hsa-miR-489
DCP2	hsa-miR-6534
DCP2	hsa-miR-510-5p
DCP2	hsa-miR-224-5p
DCP2	hsa-miR-4780

DCP2	hsa-miR-6771-3p
DCP2	hsa-miR-1542-3p
DCP2	hsa-miR-2329-3p
DCP2	hsa-miR-3585-5p
DCP2	hsa-miR-4668-5p
DCP2	hsa-miR-7328-5p
DCP2	hsa-miR-4286
DCP2	hsa-miR-6768-5p
DCP2	hsa-miR-3105-3p
DCP2	hsa-miR-2284-5p
DCP2	hsa-miR-3617-5p
DCP2	hsa-miR-92a-2-5p
DCP2	hsa-miR-141-3p
DCP2	hsa-miR-6729-3p
DCP2	hsa-miR-27
DCP2	hsa-miR-215-5p
DCP2	hsa-miR-7009-3p
DCP2	hsa-miR-3103-3p
DCP2	hsa-miR-548e-3p
DCP2	hsa-miR-4679
DCP2	hsa-miR-7386i-3p
DCP2	hsa-miR-7657-5p
DCP2	hsa-miR-1290
DCP2	hsa-miR-570-3p
DCP2	hsa-miR-1264-3p
DCP2	hsa-miR-7226-5p
DCP2	hsa-miR-2331-3p
DCP2	hsa-miR-4637
DCP2	hsa-miR-2406
DCP2	hsa-miR-1283a
DCP2	hsa-miR-7158-3p
DCP2	hsa-miR-141-5p
DCP2	hsa-miR-5002-3p
DCP2	hsa-miR-367-5p
DCP2	hsa-miR-6859-5p
DCP2	hsa-miR-7083-3p
DCP2	hsa-miR-4290
DCP2	hsa-miR-7398t-3p
DCP2	hsa-miR-23b-3p
DCP2	hsa-miR-448
DCP2	hsa-miR-27b-5p
DCP2	hsa-miR-2284i
DCP2	hsa-miR-3671
DCP2	hsa-miR-6896-3p
DCP2	hsa-miR-7348-5p
DCP2	hsa-miR-4711-5p

DCP2	hsa-miR-6924-3p
DCP2	hsa-miR-138-5p
DCP2	hsa-miR-569
DCP2	hsa-miR-548ae-3p
DCP2	hsa-miR-5571-3p
DCP2	hsa-miR-1896
DCP2	hsa-miR-455-3p.2
DCP2	hsa-miR-6886-3p
DCP2	hsa-miR-873-5p.2
DCP2	hsa-miR-576-5p
DCP2	hsa-miR-4646-3p
DCP2	hsa-miR-2284c
DCP2	hsa-miR-206-5p
DCP2	hsa-miR-323a-3p
DCP2	hsa-miR-7243-5p
DCP2	hsa-miR-2285c
DCP2	hsa-miR-4799-5p
DCP2	hsa-miR-628-3p
DCP2	hsa-miR-547-3p
DCP2	hsa-miR-450b
DCP2	hsa-miR-1277-5p
DCP2	hsa-miR-872
DCP2	hsa-miR-7188-5p
DCP2	hsa-miR-548f-5p
DCP2	hsa-miR-5580-3p
DCP2	hsa-miR-2339
DCP2	hsa-miR-6913-3p
DCP2	hsa-miR-7015-3p
DCP2	hsa-miR-146-5p
DCP2	hsa-miR-548z
DCP2	hsa-miR-4680
DCP2	hsa-miR-7323-3p
DCP2	hsa-miR-410
DCP2	hsa-miR-8904b
DCP2	hsa-miR-218a-2-3p
DCP2	hsa-miR-1460
DCP2	hsa-miR-188-5p
DCP2	hsa-miR-7398n-5p
DCP2	hsa-miR-373-3p
DCP2	hsa-miR-5583-5p
DCP2	hsa-miR-8064
DCP2	hsa-miR-338-3p
DCP2	hsa-miR-28-5p
DCP2	hsa-miR-6516-3p
DCP2	hsa-miR-7356-5p
DCP2	hsa-miR-7207-5p

DCP2	hsa-miR-4791
DCP2	hsa-miR-4662a-3p
DCP2	hsa-miR-7213-3p
DCP2	hsa-miR-6953-5p
DCP2	hsa-miR-3689d
DCP2	hsa-miR-1843
DCP2	hsa-miR-7258-3p
DCP2	hsa-miR-532-5p
DCP2	hsa-miR-7183-5p
DCP2	hsa-miR-4434
DCP2	hsa-miR-3113-3p
DCP2	hsa-miR-8793
DCP2	hsa-miR-2472
DCP2	hsa-miR-4755-5p
DCP2	hsa-miR-9-3-3p
DCP2	hsa-miR-7665-3p
DCP2	hsa-miR-509-5p
DCP2	hsa-miR-148
DCP2	hsa-miR-6930-5p
DCP2	hsa-miR-142-5p
DCP2	hsa-miR-130a-5p
DCP2	hsa-miR-548y
DCP2	hsa-miR-6970-5p
DCP2	hsa-miR-376-3p
DCP2	hsa-miR-6515-3p
DCP2	hsa-miR-525-5p
DCP2	hsa-miR-2375
DCP2	hsa-miR-1547-3p
DCP2	hsa-miR-23a-3p
DCP2	hsa-miR-323b-5p
DCP2	hsa-miR-3121-5p
DCP2	hsa-miR-3079-3p
DCP2	hsa-miR-6359
DCP2	hsa-miR-200ac-5p
DCP2	hsa-miR-6866-5p
DCP2	hsa-miR-892-3p
DCP2	hsa-miR-1208
DCP2	hsa-miR-2429
DCP2	hsa-miR-5009-3p
DCP2	hsa-miR-5589-5p
DCP2	hsa-miR-2284aa
DCP2	hsa-miR-219a-1-3p
DCP2	hsa-miR-130a-3p
DCP2	hsa-miR-1178-5p
DCP2	hsa-miR-136-5p
DCP2	hsa-miR-7241-5p

DCP2	hsa-miR-2440
DCP2	hsa-miR-3473c
DCP2	hsa-miR-199a-3p
DCP2	hsa-miR-4311
DCP2	hsa-miR-7864
DCP2	hsa-miR-6399
DCP2	hsa-miR-1546-3p
DCP2	hsa-miR-7316-5p
DCP2	hsa-miR-7444-5p
DCP2	hsa-miR-6870-5p
DCP2	hsa-miR-6858-5p
DCP2	hsa-miR-29b-3p
DCP2	hsa-miR-3548
DCP2	hsa-miR-130b-5p
DCP2	hsa-miR-5693
DCP2	hsa-miR-7313-3p
DCP2	hsa-miR-3098-5p
DCP2	hsa-miR-3201
DCP2	hsa-miR-7161-5p
DCP2	hsa-miR-6937-3p
DCP2	hsa-let-7a-3p
DCP2	hsa-miR-6873-3p
DCP2	hsa-miR-8119
DCP2	hsa-miR-322-3p
DCP2	hsa-miR-548a-5p
DCP2	hsa-miR-340-5p
DCP2	hsa-miR-3675-3p
DCP2	hsa-miR-378d
DCP2	hsa-miR-7360-3p
DCP2	hsa-miR-292a-3p
DCP2	hsa-miR-6999-5p
DCP2	hsa-miR-466c-3p
DCP2	hsa-miR-371
DCP2	hsa-miR-6856-5p
DCP2	hsa-miR-4291
DCP2	hsa-miR-6976-3p
DCP2	hsa-miR-7200-5p
DCP2	hsa-miR-653-5p
DCP2	hsa-miR-196-3p
DCP2	hsa-miR-7685-3p
DCP2	hsa-miR-155-5p
DCP2	hsa-miR-2285aa
DCP2	hsa-miR-26-3p
DCP2	hsa-miR-3551-5p
DCP2	hsa-miR-580
DCP2	hsa-miR-4693-5p

DCP2	hsa-miR-1183
DCP2	hsa-miR-3664-5p
DCP2	hsa-miR-6506-5p
DCP2	hsa-miR-331-5p
DCP2	hsa-miR-455-3p
DCP2	hsa-miR-7257-3p
DCP2	hsa-miR-921
DCP2	hsa-miR-8056
DCP2	hsa-miR-7397-5p
DCP2	hsa-miR-7119-5p
DCP2	hsa-miR-421-5p
DCP2	hsa-miR-193b-5p
DCP2	hsa-miR-6348
DCP2	hsa-miR-4687-3p
DCP2	hsa-miR-3612
DCP2	hsa-miR-3682-3p
DCP2	hsa-miR-6906-3p
DCP2	hsa-miR-6120-5p
DCP2	hsa-miR-3096-5p
DCP2	hsa-miR-3129-3p
DCP2	hsa-miR-7396-5p
DCP2	hsa-miR-5000-5p
DCP2	hsa-miR-627
DCP2	hsa-miR-6916-5p
DCP2	hsa-miR-101-1-5p
DCP2	hsa-miR-329-3p
DCP2	hsa-miR-1279
DCP2	hsa-miR-634
DCP2	hsa-miR-365b-5p
DCP2	hsa-miR-6926-5p
DCP2	hsa-miR-3163
DCP2	hsa-miR-7370-5p
DCP2	hsa-miR-8052
DCP2	hsa-miR-196c-3p
DCP2	hsa-miR-7312-3p
DCP2	hsa-miR-4647
DCP2	hsa-miR-3098-3p
DCP2	hsa-miR-573
DCP2	hsa-miR-7355-5p
DCP2	hsa-miR-496b
DCP2	hsa-miR-766-3p
DCP2	hsa-miR-30-5p
DCP2	hsa-miR-7294-3p
DCP2	hsa-miR-149-3p
DCP2	hsa-miR-6773-3p
DCP2	hsa-miR-7688-3p

DCP2	hsa-miR-3121-3p
DCP2	hsa-miR-378-5p
DCP2	hsa-miR-4641
DCP2	hsa-miR-1296-3p
DCP2	hsa-miR-6828-5p
DCP2	hsa-miR-2465
DCP2	hsa-miR-7b-3p
DCP2	hsa-miR-133a-5p
DCP2	hsa-miR-148b-3p
DCP2	hsa-miR-203
DCP2	hsa-miR-6785-5p
DCP2	hsa-miR-4422
DCP2	hsa-miR-6734-3p
DCP2	hsa-miR-548x-5p
DCP2	hsa-miR-500a-3p
DCP2	hsa-miR-7106-5p
DCP2	hsa-miR-409
DCP2	hsa-miR-1582
DCP2	hsa-miR-669m-5p
DCP2	hsa-miR-7366-5p
DCP2	hsa-miR-508a
DCP2	hsa-miR-200a-5p
DCP2	hsa-miR-8846
DCP2	hsa-miR-4436b-5p
DCP2	hsa-miR-548
DCP2	hsa-miR-632
DCP2	hsa-miR-7973
DCP2	hsa-miR-584-3p
DCP2	hsa-miR-7394b-5p
DCP2	hsa-miR-7321-3p
DCP2	hsa-miR-7387-3p
DCP2	hsa-miR-891a-3p
DCP2	hsa-miR-6129
DCP2	hsa-miR-3557-3p
DCP2	hsa-miR-3685
DCP2	hsa-miR-383
DCP2	hsa-miR-5700
DCP2	hsa-miR-380-5p
DCP2	hsa-miR-6722-3p
DCP2	hsa-miR-5008-3p
DCP2	hsa-miR-7377-3p
DCP2	hsa-miR-548aw
DCP2	hsa-miR-7681-3p
DCP2	hsa-miR-2399-5p
DCP2	hsa-miR-532-5p
DCP2	hsa-miR-539-5p

DCP2	hsa-miR-935
DCP2	hsa-miR-519e-3p
DCP2	hsa-miR-7244-3p
DCP2	hsa-miR-148a-3p
DCP2	hsa-miR-3572
DCP2	hsa-miR-1273g-3p
DCP2	hsa-miR-6855-5p
DCP2	hsa-miR-4297
DCP2	hsa-miR-3679-3p
DCP2	hsa-miR-1324
DCP2	hsa-miR-1468-5p
DCP2	hsa-miR-6867-3p
DCP2	hsa-miR-7398r-5p
DCP2	hsa-miR-8093
DCP2	hsa-miR-450b-3p
DCP2	hsa-miR-6736-3p
DCP2	hsa-miR-2387
DCP2	hsa-miR-7217-3p
DCP2	hsa-miR-4746-3p
DCP2	hsa-miR-145-5p
DCP2	hsa-miR-517
DCP2	hsa-miR-20-5p
DCP2	hsa-miR-499-5p
DCP2	hsa-miR-2313-3p
DCP2	hsa-miR-550
DCP2	hsa-miR-7230-5p
DCP2	hsa-miR-6546-5p
DCP2	hsa-miR-125
DCP2	hsa-miR-2390
DCP2	hsa-miR-6932-3p
DCP2	hsa-miR-149-5p
DCP2	hsa-miR-2397-3p
DCP2	hsa-miR-34c-3p
DCP2	hsa-miR-6975-3p
DCP2	hsa-miR-513c-3p
DCP2	hsa-miR-5119
DCP2	hsa-miR-3569-3p
DCP2	hsa-miR-4671-3p
DCP2	hsa-miR-6889-3p
DCP2	hsa-miR-2486-3p
DCP2	hsa-miR-548az-3p
DCP2	hsa-miR-7268-5p
DCP2	hsa-miR-3069-3p
DCP2	hsa-miR-1249-3p
DCP2	hsa-miR-7374b-3p
DCP2	hsa-miR-210

DCP2	hsa-miR-374b-5p
DCP2	hsa-miR-431-5p
DCP2	hsa-miR-7304-3p
DCP2	hsa-miR-6813-3p
DCP2	hsa-miR-6944-3p
DCP2	hsa-miR-27a-5p
DCP2	hsa-miR-29a-5p
DCP2	hsa-miR-6122-3p
DCP2	hsa-miR-6934-3p
DCP2	hsa-miR-1292-3p
DCP2	hsa-miR-548ac
DCP2	hsa-miR-6947-3p
DCP2	hsa-miR-7227-3p
DCP2	hsa-miR-190a-3p
DCP2	hsa-miR-7480-5p
DCP2	hsa-miR-5589-3p
DCP2	hsa-miR-548ae-5p
DCP2	hsa-miR-589-5p
DCP2	hsa-miR-3091-5p
DCP2	hsa-miR-425-5p
DCP2	hsa-miR-467-3p
DCP2	hsa-miR-3120-5p
DCP2	hsa-miR-488-3p
DCP2	hsa-miR-6847-3p
DCP2	hsa-miR-191-3p
DCP2	hsa-miR-382-5p
DCP2	hsa-miR-5108
DCP2	hsa-miR-6954-5p
DCP2	hsa-miR-374c-3p
DCP2	hsa-miR-2319
DCP2	hsa-miR-200a-3p
DCP2	hsa-miR-7370i-3p
DCP2	hsa-miR-466-5p
DCP2	hsa-miR-6326
DCP2	hsa-miR-7844-5p
DCP2	hsa-miR-3474
DCP2	hsa-miR-6795-3p
DCP2	hsa-miR-344c-3p
DCP2	hsa-miR-105-5p
DCP2	hsa-miR-7669-5p
DCP2	hsa-miR-5616-3p
DCP2	hsa-miR-7012-3p
DCP2	hsa-miR-199b-3p
DCP2	hsa-miR-1912-5p
DCP2	hsa-miR-20b-3p
DCP2	hsa-miR-429-5p

DCP2	hsa-miR-548ah-5p
DCP2	hsa-miR-6511-5p
DCP2	hsa-miR-212-3p
DCP2	hsa-miR-8821
DCP2	hsa-miR-4676-5p
DCP2	hsa-miR-216a-3p
DCP2	hsa-miR-5098
DCP2	hsa-miR-2420
DCP2	hsa-miR-6526
DCP2	hsa-miR-1276
DCP2	hsa-miR-411-3p
DCP2	hsa-miR-543-5p
DCP2	hsa-miR-6909-3p
DCP2	hsa-miR-7011-3p
DCP2	hsa-miR-742-5p
DCP2	hsa-miR-548ba
DCP2	hsa-miR-1273f
DCP2	hsa-miR-3713
DCP2	hsa-miR-328
DCP2	hsa-miR-6935-3p
DCP2	hsa-miR-616-3p
DCP2	hsa-miR-199b-2-5p
DCP2	hsa-miR-6502-5p
DCP2	hsa-miR-296-3p
DCP2	hsa-miR-767
DCP2	hsa-miR-497-3p
DCP2	hsa-miR-548ah-3p
DCP2	hsa-miR-6673-3p
DCP2	hsa-miR-187-5p
DCP2	hsa-miR-1241
DCP2	hsa-miR-2311
DCP2	hsa-miR-6994-5p
DCP2	hsa-miR-135a-5p
DCP2	hsa-miR-301b-3p
DCP2	hsa-miR-891b
DCP2	hsa-miR-3087-3p
DCP2	hsa-miR-539-5p
DCP2	hsa-miR-6851-5p
DCP2	hsa-miR-4649-3p
DCP2	hsa-miR-2052
DCP2	hsa-miR-96-3p
DCP2	hsa-miR-6715-3p
DCP2	hsa-miR-1193
DCP2	hsa-miR-181d-3p
DCP2	hsa-miR-506-5p
DCP2	hsa-miR-743a-3p

DCP2	hsa-miR-1236-5p
DCP2	hsa-miR-667-5p
DCP2	hsa-miR-7264-3p
DCP2	hsa-miR-196-5p
DCP2	hsa-miR-7331-5p
DCP2	hsa-miR-7169-5p
DCP2	hsa-miR-6780a-5p
DCP2	hsa-miR-3958
DCP2	hsa-miR-6939-3p
DCP2	hsa-miR-2116-5p
DCP2	hsa-miR-7367-3p
DCP2	hsa-miR-1254
DCP2	hsa-miR-5622-5p
DCP2	hsa-miR-449-3p
DCP2	hsa-miR-4659a-5p
DCP2	hsa-miR-684
DCP2	hsa-miR-7067-5p
DCP2	hsa-miR-511-3p
DCP2	hsa-miR-8853
DCP2	hsa-miR-34c-5p
DCP2	hsa-miR-3431
DCP2	hsa-miR-3561-3p
DCP2	hsa-miR-669k-5p
DCP2	hsa-miR-2467-3p
DCP2	hsa-miR-7365-3p
DCP2	hsa-miR-4259
DCP2	hsa-miR-6760-3p
DCP2	hsa-miR-329-5p
DCP2	hsa-miR-6516-5p
DCP2	hsa-miR-7298-5p
DCP2	hsa-miR-6910-5p
DCP2	hsa-miR-320-3p
DCP2	hsa-miR-202-3p
DCP2	hsa-miR-4509
DCP2	hsa-miR-6864-3p
DCP2	hsa-miR-19b-5p
DCP2	hsa-miR-548ao-5p
DCP2	hsa-miR-3969
DCP2	hsa-miR-4743-3p
DCP2	hsa-miR-6418-5p
DCP2	hsa-miR-7187-3p
DCP2	hsa-miR-3077-3p
DCP2	hsa-miR-223-3p
DCP2	hsa-miR-134-5p
DCP2	hsa-miR-3543
DCP2	hsa-miR-7652-5p

DCP2	hsa-miR-3058-5p
DCP2	hsa-miR-372
DCP2	hsa-miR-551-5p
DCP2	hsa-miR-3187-5p
DCP2	hsa-miR-454-5p
DCP2	hsa-miR-6837-5p
DCP2	hsa-miR-5089-5p
DCP2	hsa-miR-3157-3p
DCP2	hsa-miR-7211-3p
DCP2	hsa-miR-29a-3p
DCP2	hsa-miR-221-5p
DCP2	hsa-miR-6715b-3p
DCP2	hsa-miR-7002-5p
DCP2	hsa-miR-193-5p
DCP2	hsa-miR-3133
DCP2	hsa-miR-1668
DCP2	hsa-miR-6501-5p
DCP2	hsa-miR-449b-3p
DCP2	hsa-miR-4749-3p
DCP2	hsa-miR-3558-3p
DCP2	hsa-miR-20
DCP2	hsa-miR-889
DCP2	hsa-miR-4753-3p
DCP2	hsa-miR-6828-3p
DCP2	hsa-miR-655-3p
DCP2	hsa-miR-219-3p
DCP2	hsa-miR-6794-3p
DCP2	hsa-miR-7705
DCP2	hsa-miR-3667-3p
DCP2	hsa-miR-2396
DCP2	hsa-miR-6965-3p
DCP2	hsa-miR-2324
DCP2	hsa-miR-6338
DCP2	hsa-miR-7386j-3p
DCP2	hsa-miR-6974-5p
DCP2	hsa-miR-7385f-3p
DCP2	hsa-miR-7240-5p
DCP2	hsa-miR-708-3p
DCP2	hsa-miR-7035-3p
DCP2	hsa-miR-342-3p
DCP2	hsa-miR-129-3p
DCP2	hsa-miR-363-3p
DCP2	hsa-miR-98-5p
DCP2	hsa-miR-548ad-5p
DCP2	hsa-miR-7157-3p
DCP2	hsa-miR-7198-5p

DCP2	hsa-miR-7859
DCP2	hsa-miR-1943-3p
DCP2	hsa-miR-938
DCP2	hsa-miR-6854-5p
DCP2	hsa-miR-5116
DCP2	hsa-miR-1231-5p
DCP2	hsa-miR-3609
DCP2	hsa-miR-448-5p
DCP2	hsa-miR-3169
DCP2	hsa-miR-129-2-3p
DCP2	hsa-miR-585-5p
DCP2	hsa-miR-6830-3p
DCP2	hsa-miR-4713-5p
DCP2	hsa-miR-454-3p
DCP2	hsa-miR-6411
DCP2	hsa-miR-7193-5p
DCP2	hsa-miR-6867-5p
DCP2	hsa-let-7-3p
DCP2	hsa-miR-450a-2-3p
DCP2	hsa-miR-548ar-5p
DCP2	hsa-miR-885
DCP2	hsa-miR-6739-3p
DCP2	hsa-miR-4520-3p
DCP2	hsa-miR-8880
DCP2	hsa-miR-4532
DCP2	hsa-miR-7262-3p
DCP2	hsa-miR-6900-3p
DCP2	hsa-miR-7025-3p
DCP2	hsa-miR-664
DCP2	hsa-miR-7110-5p
DCP2	hsa-miR-741-5p
DCP2	hsa-miR-218-5p
DCP2	hsa-miR-6942-5p
DCP2	hsa-miR-7192-5p
DCP2	hsa-miR-6885-3p
DCP2	hsa-miR-3179
DCP2	hsa-miR-429-3p
DCP2	hsa-miR-5696
DCP2	hsa-miR-668
DCP2	hsa-miR-2462
DCP2	hsa-miR-6758-5p
DCP2	hsa-miR-548d-5p
DCP2	hsa-miR-5683
DCP2	hsa-miR-3663-3p
DCP2	hsa-miR-199
DCP2	hsa-miR-5002-5p

DCP2	hsa-miR-1266
DCP2	hsa-miR-802-5p
DCP2	hsa-miR-466q
DCP2	hsa-miR-6749-3p
DCP2	hsa-miR-939-3p
DCP2	hsa-miR-8908-5p
DCP2	hsa-miR-4496
DCP2	hsa-miR-7294-5p
DCP2	hsa-miR-322-5p
DCP2	hsa-miR-6961-3p
DCP2	hsa-miR-671-3p
DCP2	hsa-miR-7270-5p
DCP2	hsa-miR-6754-3p
DCP2	hsa-miR-4731-3p
DCP2	hsa-miR-4307
DCP2	hsa-miR-424-3p
DCP2	hsa-miR-2891
DCP2	hsa-miR-6969-5p
DCP2	hsa-miR-6945-5p
DCP2	hsa-miR-486-5p
DCP2	hsa-miR-1271-5p
DCP2	hsa-miR-6752-5p
DCP2	hsa-miR-301-5p
DCP2	hsa-miR-8850
DCP2	hsa-miR-7652-3p
DCP2	hsa-miR-384
DCP2	hsa-miR-6765-3p
DCP2	hsa-miR-6378
DCP2	hsa-miR-8485
DCP2	hsa-miR-7309-3p
DCP2	hsa-miR-548u
DCP2	hsa-miR-501-5p
DCP2	hsa-miR-6134
DCP2	hsa-miR-3650
DCP2	hsa-miR-4445-5p
DCP2	hsa-miR-592
DCP2	hsa-miR-6997-5p
DCP2	hsa-miR-7065-3p
DCP2	hsa-miR-361-3p
DCP2	hsa-miR-2357
DCP2	hsa-miR-320a
DCP2	hsa-miR-7204-5p
DCP2	hsa-miR-7398h-3p
DCP2	hsa-miR-4715-5p
DCP2	hsa-miR-6322
DCP2	hsa-let-7b-3p

DCP2	hsa-miR-6928-3p
DCP2	hsa-miR-7371d-5p
DCP2	hsa-miR-146b-3p
DCP2	hsa-miR-3125
DCP2	hsa-miR-875-3p
DCP2	hsa-miR-375-3p
DCP2	hsa-miR-7673-3p
DCP2	hsa-miR-6727-3p
DCP2	hsa-miR-7276-5p
DCP2	hsa-miR-8840
DCP2	hsa-miR-7037-3p
DCP2	hsa-miR-8106
DCP2	hsa-miR-618
DCP2	hsa-miR-200c-3p
DCP2	hsa-miR-580-5p
DCP2	hsa-miR-4662b
DCP2	hsa-miR-3068-3p
DCP2	hsa-miR-7663-3p
DCP2	hsa-miR-1702
DCP2	hsa-miR-4778-5p
DCP2	hsa-miR-6514-3p
DCP2	hsa-miR-5197-3p
DCP2	hsa-miR-378-3p
DCP2	hsa-miR-3591-3p
DCP2	hsa-miR-3112-5p
DCP2	hsa-miR-192-5p
DCP2	hsa-miR-6912-3p
DCP2	hsa-miR-2377
DCP2	hsa-miR-7322-3p
DCP2	hsa-miR-616-5p
DCP2	hsa-miR-2469
DCP2	hsa-miR-6829-3p
DCP2	hsa-miR-151a-5p
DCP2	hsa-miR-3190-3p
DCP2	hsa-miR-7252-3p
DCP2	hsa-miR-3057-3p
DCP2	hsa-miR-8868
DCP2	hsa-miR-3649
DCP2	hsa-miR-6914-5p
DCP2	hsa-miR-7398q-3p
DCP2	hsa-miR-7063-5p
DCP2	hsa-miR-155-5p
DCP2	hsa-miR-376a-5p
DCP2	hsa-miR-107-3p
DCP2	hsa-miR-467g
DCP2	hsa-miR-7676-5p

DCP2	hsa-miR-370-5p
DCP2	hsa-let-7j-3p
DCP2	hsa-miR-302c-3p
DCP2	hsa-miR-6372
DCP2	hsa-miR-31-5p
DCP2	hsa-miR-2351
DCP2	hsa-miR-99-3p
DCP2	hsa-miR-16-1-3p
DCP2	hsa-miR-1297
DCP2	hsa-miR-376e
DCP2	hsa-miR-3556a
DCP2	hsa-miR-201-3p
DCP2	hsa-miR-6398
DCP2	hsa-miR-7363-3p
DCP2	hsa-miR-7-5p
DCP2	hsa-miR-4682
DCP2	hsa-miR-4529-3p
DCP2	hsa-miR-2285l
DCP2	hsa-miR-340-3p
DCP2	hsa-miR-8852
DCP2	hsa-miR-669bf-3p
DCP2	hsa-miR-3090-5p
DCP2	hsa-miR-5094
DCP2	hsa-miR-3101-5p
DCP2	hsa-miR-6984-3p
DCP2	hsa-miR-6500-3p
DCP2	hsa-miR-6790-3p
DCP2	hsa-miR-871-5p
DCP2	hsa-miR-3121-3p
DCP2	hsa-miR-8096
DCP2	hsa-miR-3094-3p
DCP2	hsa-miR-548w
DCP2	hsa-miR-2341
DCP2	hsa-miR-548i-5p
DCP2	hsa-let-7afk-3p
DCP2	hsa-miR-412
DCP2	hsa-miR-3559-5p
DCP2	hsa-miR-4524a-3p
DCP2	hsa-miR-4728-5p
DCP2	hsa-miR-1273h-3p
DCP2	hsa-miR-7398o-5p
DCP2	hsa-miR-130b-3p
DCP2	hsa-miR-6730-3p
DCP2	hsa-miR-6747-3p
DCP2	hsa-miR-30b-3p
DCP2	hsa-miR-7213-5p

DCP2	hsa-miR-466g
DCP2	hsa-miR-7449-3p
DCP2	hsa-miR-2422
DCP2	hsa-miR-519e-5p
DCP2	hsa-miR-26-5p
DCP2	hsa-miR-2284
DCP2	hsa-miR-6951-5p
DCP2	hsa-miR-7330-3p
DCP2	hsa-miR-6510-5p
DCP2	hsa-miR-3074-5p
DCP2	hsa-miR-548o-5p
DCP2	hsa-miR-3192-5p
DCP2	hsa-miR-499
DCP2	hsa-miR-6524
DCP2	hsa-miR-2114-3p
DCP2	hsa-miR-7090-5p
DCP2	hsa-miR-3110-3p
DCP2	hsa-miR-2331-5p
DCP2	hsa-miR-6802-3p
DCP2	hsa-miR-1264-5p
DCP2	hsa-miR-7035-5p
DCP2	hsa-miR-2466-3p
DCP2	hsa-miR-7270-3p
DCP2	hsa-miR-1245-5p
DCP2	hsa-miR-7022-3p
DCP2	hsa-miR-29
DCP2	hsa-miR-7023-3p
DCP2	hsa-miR-8053
DCP2	hsa-miR-6997-3p
DCP2	hsa-miR-6908-3p
DCP2	hsa-miR-592-3p
DCP2	hsa-miR-452-5p
DCP2	hsa-miR-487b-5p
DCP2	hsa-miR-7110-3p
DCP2	hsa-miR-7293-5p
DCP2	hsa-miR-101-3p.2
DCP2	hsa-miR-335-3p
DCP2	hsa-miR-4320
DCP2	hsa-miR-614
DCP2	hsa-miR-743b-3p
DCP2	hsa-miR-345-5p
DCP2	hsa-miR-543-3p
DCP2	hsa-miR-7371e-3p
DCP2	hsa-miR-7-5p
DCP2	hsa-miR-7166-3p
DCP2	hsa-miR-1643b-5p

DCP2	hsa-miR-24-5p
DCP2	hsa-miR-553
DCP2	hsa-miR-526
DCP2	hsa-miR-4263
DCP2	hsa-miR-7288-3p
DCP2	hsa-miR-7340-3p
DCP2	hsa-miR-17-5p
DCP2	hsa-miR-378c
DCP2	hsa-miR-7182-3p
DCP2	hsa-miR-4426
DCP2	hsa-miR-5124a
DCP2	hsa-miR-3151-3p
DCP2	hsa-miR-548ap-5p
DCP2	hsa-miR-466f-3p
DCP2	hsa-miR-7232-3p
DCP2	hsa-miR-8885
DCP2	hsa-miR-7079-3p
DCP2	hsa-miR-879-5p
DCP2	hsa-miR-485
DCP2	hsa-miR-2383
DCP2	hsa-miR-7398r-3p
DCP2	hsa-miR-20a-3p
DCP2	hsa-miR-891
DCP2	hsa-miR-7333-5p
DCP2	hsa-miR-1182
DCP2	hsa-miR-4797-5p
DCP2	hsa-miR-3076-3p
DCP2	hsa-miR-7386l-5p
DCP2	hsa-miR-539-3p
DCP2	hsa-miR-4633-5p
DCP2	hsa-miR-16-5p
DCP2	hsa-miR-135-5p
DCP2	hsa-miR-3653-5p
DCP2	hsa-miR-8862
DCP2	hsa-miR-3945
DCP2	hsa-miR-646
DCP2	hsa-miR-518-5p
DCP2	hsa-miR-548g-5p
DCP2	hsa-miR-7655-3p
DCP2	hsa-miR-2379
DCP2	hsa-miR-7043-3p
DCP2	hsa-miR-23b-5p
DCP2	hsa-miR-520f-5p
DCP2	hsa-miR-888-5p
DCP2	hsa-miR-1322
DCP2	hsa-miR-1229-5p

DCP2	hsa-miR-30e
DCP2	hsa-miR-302d-5p
DCP2	hsa-miR-3126-5p
DCP2	hsa-miR-4700-3p
DCP2	hsa-miR-7397-3p
DCP2	hsa-miR-6788-5p
DCP2	hsa-miR-7686-5p
DCP2	hsa-miR-327
DCP2	hsa-miR-140-3p
DCP2	hsa-miR-1955-5p
DCP2	hsa-miR-2359
DCP2	hsa-miR-26a-3p
DCP2	hsa-miR-2284j
DCP2	hsa-miR-490-3p
DCP2	hsa-miR-891a-5p
DCP2	hsa-miR-105b
DCP2	hsa-miR-5689
DCP2	hsa-miR-7012-5p
DCP2	hsa-miR-466b-4-3p
DCP2	hsa-miR-892a
DCP2	hsa-miR-7275-5p
DCP2	hsa-miR-656-3p
DCP2	hsa-miR-7050-5p
DCP2	hsa-miR-5687
DCP2	hsa-miR-8058
DCP2	hsa-miR-7177-5p
DCP2	hsa-miR-7314-5p
DCP2	hsa-miR-4635
DCP2	hsa-miR-4539
DCP2	hsa-miR-5583-3p
DCP2	hsa-miR-6537-3p
DCP2	hsa-miR-34b-3p
DCP2	hsa-miR-7386j-5p
DCP2	hsa-miR-4253
DCP2	hsa-miR-301a-5p
DCP2	hsa-miR-205-5p
DCP2	hsa-miR-657
DCP2	hsa-miR-463-5p
DCP2	hsa-miR-124-5p
DCP2	hsa-miR-7386d-3p
DCP2	hsa-miR-4441
DCP2	hsa-miR-6853-3p
DCP2	hsa-miR-6944-5p
DCP2	hsa-miR-3588
DCP2	hsa-miR-4678
DCP2	hsa-miR-3084-3p

DCP2	hsa-miR-299b-5p
DCP2	hsa-miR-17-3p
DCP2	hsa-miR-4310
DCP2	hsa-miR-5624-5p
DCP2	hsa-miR-5585-5p
DCP2	hsa-miR-183-5p
DCP2	hsa-miR-7369-5p
DCP2	hsa-miR-292a-5p
DCP2	hsa-miR-1193-5p
DCP2	hsa-miR-9768-3p
DCP2	hsa-miR-1950
DCP2	hsa-miR-182-5p
DCP2	hsa-miR-1224
DCP2	hsa-miR-101b-3p.2
DCP2	hsa-miR-32-5p
DCP2	hsa-miR-4663
DCP2	hsa-miR-548t-5p
DCP2	hsa-miR-7386f-3p
DCP2	hsa-miR-704
DCP2	hsa-miR-6845-3p
DCP2	hsa-miR-518
DCP2	hsa-miR-6884-3p
DCP2	hsa-miR-3123
DCP2	hsa-miR-942-5p
DCP2	hsa-miR-502-3p
DCP2	hsa-miR-600
DCP2	hsa-miR-365a-3p
DCP2	hsa-miR-6328
DCP2	hsa-miR-4748
DCP2	hsa-miR-7254-3p
DCP2	hsa-miR-6072
DCP2	hsa-miR-576-3p
DCP2	hsa-miR-1243-3p
DCP2	hsa-miR-6779-5p
DCP2	hsa-miR-6983-5p
DCP2	hsa-miR-7388c-3p
DCP2	hsa-miR-873-3p
DCP2	hsa-miR-6919-3p
DCP2	hsa-miR-759
DCP2	hsa-miR-372-3p
DCP2	hsa-miR-6343
DCP2	hsa-miR-466
DCP2	hsa-miR-15-5p
DCP2	hsa-miR-548aj-5p
DCP2	hsa-miR-637
DCP2	hsa-miR-5586-5p

DCP2	hsa-miR-367-3p
DCP2	hsa-miR-6132
DCP2	hsa-miR-346
DCP2	hsa-miR-5011-5p
DCP2	hsa-miR-2917
DCP2	hsa-miR-7304-5p
DCP2	hsa-miR-6507-5p
DCP2	hsa-miR-3596
DCP2	hsa-miR-3596d
DCP2	hsa-miR-6782-3p
DCP2	hsa-miR-3145-5p
DCP2	hsa-miR-449a-3p
DCP2	hsa-miR-221-3p
DCP2	hsa-miR-593-3p
DCP2	hsa-miR-1298-5p
DCP2	hsa-miR-653-3p
DCP2	hsa-miR-4804-3p
DCP2	hsa-miR-7857
DCP2	hsa-miR-7371b-5p
DCP2	hsa-miR-200b-3p
DCP2	hsa-miR-188-3p
DCP2	hsa-miR-344-3p
DCP2	hsa-miR-1306-5p
DCP2	hsa-miR-6995-3p
DCP2	hsa-miR-7341-5p
DCP2	hsa-miR-1205
DCP2	hsa-miR-181-3p
DCP2	hsa-miR-483-3p.2
DCP2	hsa-miR-2423
DCP2	hsa-miR-33-3p
DCP2	hsa-miR-6780b-3p
DCP2	hsa-miR-302c-5p
DCP2	hsa-miR-143-3p
DCP2	hsa-miR-7259-5p
DCP2	hsa-miR-7359-3p
DCP2	hsa-miR-3072-3p
DCP2	hsa-miR-130c-5p
DCP2	hsa-miR-4521
DCP2	hsa-miR-450a
DCP2	hsa-miR-7398u-3p
DCP2	hsa-miR-8867
DCP2	hsa-miR-219-5p
DCP2	hsa-miR-30c-3p
DCP2	hsa-miR-4650-5p
DCP2	hsa-miR-329
DCP2	hsa-miR-4757-5p

DCP2	hsa-miR-6508-5p
DCP2	hsa-miR-30c-5p
DCP2	hsa-miR-7150
DCP2	hsa-miR-6124
DCP2	hsa-miR-6980-5p
DCP2	hsa-miR-142-3p
DCP2	hsa-miR-4773
DCP2	hsa-miR-3102-3p
DCP2	hsa-miR-556-5p
DCP2	hsa-miR-7057-5p
DCP2	hsa-miR-6356
DCP2	hsa-miR-3124-3p
DCP2	hsa-miR-2353
DCP2	hsa-miR-686
DCP2	hsa-miR-7250-5p
DCP2	hsa-miR-2450c
DCP2	hsa-miR-471-5p
DCP2	hsa-miR-194-5p
DCP2	hsa-miR-301b-5p
DCP2	hsa-miR-5187-3p
DCP2	hsa-miR-8097
DCP2	hsa-miR-295-3p
DCP2	hsa-miR-29b-5p
DCP2	hsa-miR-7233-3p
DCP2	hsa-miR-1827
DCP2	hsa-miR-216b-3p
DCP2	hsa-miR-6995-5p
DCP2	hsa-miR-3934-5p
DCP2	hsa-miR-7279-3p
DCP2	hsa-miR-1270
DCP2	hsa-miR-540-5p
DCP2	hsa-miR-5587-5p
DCP2	hsa-miR-4433a-3p
DCP2	hsa-miR-1814
DCP2	hsa-miR-3165
DCP2	hsa-miR-500-5p
DCP2	hsa-miR-139-5p
DCP2	hsa-miR-7674-5p
DCP2	hsa-miR-4761-5p
DCP2	hsa-miR-488-3p
DCPS	hsa-miR-7051-5p
DCPS	hsa-miR-483-3p.2
DCPS	hsa-miR-15-5p
DCPS	hsa-miR-6960-3p
DCPS	hsa-miR-660-3p
DCPS	hsa-miR-302

DCPS	hsa-miR-5627-3p
DCPS	hsa-miR-8810
DCPS	hsa-miR-5710
DCPS	hsa-miR-3663-5p
DCPS	hsa-miR-883-3p
DCPS	hsa-miR-132-3p
DCPS	hsa-miR-4323
DCPS	hsa-miR-6337
DCPS	hsa-miR-4680-5p
DCPS	hsa-miR-7047-3p
DCPS	hsa-miR-7023-5p
DCPS	hsa-miR-4714-5p
DCPS	hsa-miR-3138
DCPS	hsa-miR-147a
DCPS	hsa-miR-6949-3p
DCPS	hsa-miR-4273
DCPS	hsa-miR-7312-5p
DCPS	hsa-miR-6734-3p
DCPS	hsa-miR-5587-3p
DCPS	hsa-miR-7196-5p
DCPS	hsa-miR-876-5p
DCPS	hsa-miR-550a-5p
DCPS	hsa-miR-506-3p
DCPS	hsa-miR-4723-5p
DCPS	hsa-miR-211-3p
DCPS	hsa-miR-4793-3p
DCPS	hsa-miR-4433b-5p
DCPS	hsa-miR-7372-3p
DCPS	hsa-miR-5107-5p
DCPS	hsa-miR-5591-5p
DCPS	hsa-miR-4792
DCPS	hsa-miR-7039-3p
DCPS	hsa-miR-3921
DCPS	hsa-miR-365-5p
DCPS	hsa-miR-603
DCPS	hsa-miR-4742-5p
DCPS	hsa-miR-199-5p
DCPS	hsa-miR-4303
DCPS	hsa-miR-6901-3p
DCPS	hsa-miR-5087
DCPS	hsa-miR-467f
DCPS	hsa-miR-7069-5p
DCPS	hsa-miR-631
DCPS	hsa-miR-7027-5p
DCPS	hsa-miR-7647-5p
DCPS	hsa-miR-548

DCPS	hsa-miR-378b
DCPS	hsa-miR-3664-3p
DCPS	hsa-miR-4421
DCPS	hsa-miR-650
DCPS	hsa-miR-3619-5p
DCPS	hsa-miR-6340
DCPS	hsa-miR-6831-5p
DCPS	hsa-miR-6741-5p
DCPS	hsa-miR-344f-5p
DCPS	hsa-miR-7201-5p
DCPS	hsa-miR-4469
DCPS	hsa-miR-7163-3p
DCPS	hsa-miR-7056-3p
DCPS	hsa-miR-3915
DCPS	hsa-miR-6804-5p
DCPS	hsa-miR-149-5p
DCPS	hsa-miR-6810-3p
DCPS	hsa-miR-5625-5p
DCPS	hsa-miR-6776-3p
DCPS	hsa-miR-7299-3p
DCPS	hsa-miR-3619-3p
DCPS	hsa-miR-22-5p
DCPS	hsa-miR-216c-3p
DCPS	hsa-miR-3688-5p
DCPS	hsa-miR-7175-5p
DCPS	hsa-miR-641
DCPS	hsa-miR-4430
DCPS	hsa-miR-4519
DCPS	hsa-miR-873-5p
DCPS	hsa-miR-1915-5p
DCPS	hsa-miR-4327
DCPS	hsa-miR-183-5p
DCPS	hsa-miR-296-5p
DCPS	hsa-miR-577-3p
DCPS	hsa-miR-590-5p
DCPS	hsa-miR-578
DCPS	hsa-miR-1228-3p
DCPS	hsa-miR-489-3p
DCPS	hsa-miR-1958
DCPS	hsa-miR-3660
DCPS	hsa-miR-4274
DCPS	hsa-miR-103-3p
DCPS	hsa-miR-504-3p
DCPS	hsa-miR-3195
DCPS	hsa-miR-7113-3p
DCPS	hsa-miR-760

DCPS	hsa-miR-1911-3p
DCPS	hsa-miR-4488
DCPS	hsa-miR-6834-5p
DCPS	hsa-miR-1260b
DCPS	hsa-miR-219-5p
DCPS	hsa-miR-597-5p
DCPS	hsa-miR-450a-1-3p
DCPS	hsa-miR-4447
DCPS	hsa-miR-5001-5p
DCPS	hsa-miR-19
DCPS	hsa-miR-7152-5p
DCPS	hsa-miR-490-5p
DCPS	hsa-miR-1968-5p
DCPS	hsa-miR-3682-5p
DCPS	hsa-miR-206
DCPS	hsa-miR-541-5p
DCPS	hsa-miR-4307
DCPS	hsa-miR-504-5p
DCPS	hsa-miR-297
DCPS	hsa-miR-431-5p
DCPS	hsa-miR-7261-5p
DCPS	hsa-miR-520
DCPS	hsa-miR-6887-3p
DCPS	hsa-miR-575
DCPS	hsa-miR-376c-3p
DCPS	hsa-miR-4435
DCPS	hsa-miR-222-5p
DCPS	hsa-miR-6890-3p
DCPS	hsa-miR-4656
DCPS	hsa-miR-2475
DCPS	hsa-miR-510-3p
DCPS	hsa-miR-29-5p
DCPS	hsa-miR-3092-3p
DCPS	hsa-miR-1343-5p
DCPS	hsa-miR-5620-3p
DCPS	hsa-miR-186-3p
DCPS	hsa-miR-6777-5p
DCPS	hsa-miR-4471
DCPS	hsa-miR-6319
DCPS	hsa-miR-3059-3p
DCPS	hsa-miR-7155-5p
DCPS	hsa-miR-18a-3p
DCPS	hsa-miR-181-5p
DCPS	hsa-miR-7030-5p
DCPS	hsa-miR-1964-5p
DCPS	hsa-miR-7010-5p

DCPS	hsa-miR-1199-5p
DCPS	hsa-miR-1948-3p
DCPS	hsa-miR-4266
DCPS	hsa-miR-1238-5p
DCPS	hsa-miR-6982-5p
DCPS	hsa-miR-7679-5p
DCPS	hsa-miR-692
DCPS	hsa-miR-8089
DCPS	hsa-miR-3140-3p
DCPS	hsa-miR-3070-2-3p
DCPS	hsa-miR-6902-5p
DCPS	hsa-miR-4755-3p
DCPS	hsa-miR-7092-3p
DCPS	hsa-miR-6387
DCPS	hsa-miR-548s
DCPS	hsa-miR-6891-5p
DCPS	hsa-miR-3113-3p
DCPS	hsa-miR-3470b
DCPS	hsa-miR-6350
DCPS	hsa-miR-1293
DCPS	hsa-miR-548au-3p
DCPS	hsa-miR-6946-5p
DCPS	hsa-miR-6933-3p
DCPS	hsa-miR-342-3p
DCPS	hsa-miR-7168-5p
DCPS	hsa-miR-3199
DCPS	hsa-miR-872-3p
DCPS	hsa-miR-2296
DCPS	hsa-miR-15b-3p
DCPS	hsa-miR-465-3p
DCPS	hsa-miR-7036b-3p
DCPS	hsa-miR-4510
DCPS	hsa-miR-132-3p
DCPS	hsa-miR-3145-3p
DCPS	hsa-miR-7398s-5p
DCPS	hsa-miR-671-5p
DCPS	hsa-miR-1298-5p
DCPS	hsa-miR-548q
DCPS	hsa-miR-3591
DCPS	hsa-miR-3084bc-5p
DCPS	hsa-miR-133a-3p.2
DCPS	hsa-miR-669aflp-5p
DCPS	hsa-miR-669m-5p
DCPS	hsa-miR-195-5p
DCPS	hsa-miR-3104-5p
DCPS	hsa-miR-558

DCPS	hsa-miR-8844
DCPS	hsa-miR-105-5p
DCPS	hsa-miR-8075
DCPS	hsa-miR-7312-3p
DCPS	hsa-miR-132-5p
DCPS	hsa-miR-6941-5p
DCPS	hsa-miR-654-5p
DCPS	hsa-miR-5006-5p
DCPS	hsa-miR-134-3p
DCPS	hsa-miR-1248
DCPS	hsa-miR-330-3p
DCPS	hsa-miR-889-5p
DCPS	hsa-miR-547-5p
DCPS	hsa-miR-9-5p
DCPS	hsa-miR-1200
DCPS	hsa-miR-1953
DCPS	hsa-miR-23
DCPS	hsa-miR-717
DCPS	hsa-miR-6779-3p
DCPS	hsa-miR-6872-3p
DCPS	hsa-miR-4468
DCPS	hsa-miR-2483
DCPS	hsa-miR-1266-5p
DCPS	hsa-miR-1910-3p
DCPS	hsa-miR-2347
DCPS	hsa-miR-3105-5p
DCPS	hsa-miR-6836-5p
DCPS	hsa-miR-8084
DCPS	hsa-miR-7646-5p
DCPS	hsa-miR-7228-3p
DCPS	hsa-miR-500-3p
DCPS	hsa-miR-548u
DCPS	hsa-miR-7256-5p
DCPS	hsa-miR-483-3p.1
DCPS	hsa-miR-148-3p
DCPS	hsa-miR-6501-5p
DCPS	hsa-miR-5046
DCPS	hsa-miR-7666-3p
DCPS	hsa-miR-8083
DCPS	hsa-miR-8063
DCPS	hsa-miR-7847-3p
DCPS	hsa-miR-218a-1-3p
DCPS	hsa-miR-532-5p
DCPS	hsa-miR-3925-3p
DCPS	hsa-miR-4652-5p
DCPS	hsa-miR-125

DCPS	hsa-miR-512-5p
DCPS	hsa-miR-1225-3p
DCPS	hsa-miR-1955-5p
DCPS	hsa-miR-7187-5p
DCPS	hsa-miR-6904-5p
DCPS	hsa-miR-3160-3p
DCPS	hsa-miR-7515
DCPS	hsa-miR-1284
DCPS	hsa-miR-6876-5p
DCPS	hsa-miR-501-3p
DCPS	hsa-miR-3071-5p
DCPS	hsa-miR-2291
DCPS	hsa-miR-3128
DCPS	hsa-miR-6841-3p
DCPS	hsa-miR-5615-3p
DCPS	hsa-miR-6759-5p
DCPS	hsa-miR-6828-3p
DCPS	hsa-miR-669b-5p
DCPS	hsa-miR-7042-5p
DCPS	hsa-miR-3613-5p
DCPS	hsa-miR-3083-5p
DCPS	hsa-miR-302-3p
DCPS	hsa-miR-301-3p
DCPS	hsa-miR-604
DCPS	hsa-miR-5579-3p
DCPS	hsa-miR-103
DCPS	hsa-miR-4781-5p
DCPS	hsa-miR-30c-2-3p
DCPS	hsa-miR-4325
DCPS	hsa-miR-6746-3p
DCPS	hsa-miR-7269-5p
DCPS	hsa-miR-4741
DCPS	hsa-miR-6834-3p
DCPS	hsa-miR-6748-5p
DCPS	hsa-miR-7262a-5p
DCPS	hsa-miR-3097-3p
DCPS	hsa-miR-218-2-3p
DCPS	hsa-miR-693-3p
DCPS	hsa-miR-4501
DCPS	hsa-miR-466n-5p
DCPS	hsa-miR-8103
DCPS	hsa-miR-3667-5p
DCPS	hsa-miR-99-3p
DCPS	hsa-miR-129-5p
DCPS	hsa-miR-6737-3p
DCPS	hsa-miR-22-3p

DCPS	hsa-miR-1273h-5p
DCPS	hsa-miR-5684
DCPS	hsa-miR-4283
DCPS	hsa-miR-6074
DCPS	hsa-miR-3907
DCPS	hsa-miR-4533
DCPS	hsa-miR-3972
DCPS	hsa-miR-455-3p.1
DCPS	hsa-miR-7354-3p
DCPS	hsa-miR-8899
DCPS	hsa-miR-1545b-3-3p
DCPS	hsa-miR-6998-5p
DCPS	hsa-miR-6762-3p
DCPS	hsa-miR-3193
DCPS	hsa-miR-18-3p
DCPS	hsa-miR-216b
DCPS	hsa-miR-6905-5p
DCPS	hsa-miR-6867-5p
DCPS	hsa-miR-10-5p
DCPS	hsa-miR-7660-5p
DCPS	hsa-miR-205-5p
DCPS	hsa-miR-6815-3p
DCPS	hsa-miR-1982-5p
DCPS	hsa-miR-298-5p
DCPS	hsa-miR-4496
DCPS	hsa-miR-208-3p
DCPS	hsa-miR-4769-5p
DCPS	hsa-miR-502-5p
DCPS	hsa-miR-1468
DCPS	hsa-miR-1295b-3p
DCPS	hsa-miR-6511a-5p
DCPS	hsa-miR-92-3p
DCPS	hsa-miR-188-5p
DCPS	hsa-miR-6896-3p
DCPS	hsa-miR-4269
DCPS	hsa-miR-32-3p
DCPS	hsa-miR-6816-3p
DCPS	hsa-miR-3927-3p
DCPS	hsa-miR-6885-5p
DCPS	hsa-miR-2117
DCPS	hsa-miR-3149
DCPS	hsa-miR-6953-5p
DCPS	hsa-miR-1966-3p
DCPS	hsa-miR-1841
DCPS	hsa-miR-423-5p
DCPS	hsa-miR-6822-3p

DCPS	hsa-miR-518-3p
DCPS	hsa-miR-7976
DCPS	hsa-miR-1233-3p
DCPS	hsa-miR-4763-3p
DCPS	hsa-miR-532-3p
DCPS	hsa-miR-7194-5p
DCPS	hsa-miR-650abc
DCPS	hsa-miR-196
DCPS	hsa-miR-4688
DCPS	hsa-miR-743-5p
DCPS	hsa-miR-6922-5p
DCPS	hsa-miR-4451
DCPS	hsa-miR-449a
DCPS	hsa-miR-3664-5p
DCPS	hsa-miR-148-5p
DCPS	hsa-miR-920
DCPS	hsa-miR-347
DCPS	hsa-miR-369-3p
DCPS	hsa-miR-4518
DCPS	hsa-miR-7026-3p
DCPS	hsa-miR-3559-5p
DCPS	hsa-miR-4756-3p
DCPS	hsa-miR-1296-3p
DCPS	hsa-miR-6325
DCPS	hsa-miR-328-5p
DCPS	hsa-miR-434-3p
DCPS	hsa-miR-18
DCPS	hsa-miR-649
DCPS	hsa-miR-3926
DCPS	hsa-miR-7215-5p
DCPS	hsa-miR-4324
DCPS	hsa-miR-574-5p
DCPS	hsa-miR-1307-3p
DCPS	hsa-miR-4715-5p
DCPS	hsa-miR-107-5p
DCPS	hsa-miR-6768-5p
DCPS	hsa-miR-3589
DCPS	hsa-miR-669c-3p
DCPS	hsa-miR-6770-5p
DCPS	hsa-miR-4284
DCPS	hsa-miR-183-3p
DCPS	hsa-miR-3689-3p
DCPS	hsa-miR-33b-3p
DCPS	hsa-miR-4693-5p
DCPS	hsa-miR-1587
DCPS	hsa-miR-609

DCPS	hsa-miR-339-5p
DCPS	hsa-miR-7159-3p
DCPS	hsa-miR-449c-3p
DCPS	hsa-miR-3098-3p
DCPS	hsa-miR-8889
DCPS	hsa-miR-653-3p
DCPS	hsa-miR-1914-5p
DCPS	hsa-miR-6999-3p
DCPS	hsa-miR-8898
DCPS	hsa-miR-6848-5p
DCPS	hsa-miR-8882
DCPS	hsa-miR-3594-5p
DCPS	hsa-miR-518-5p
DCPS	hsa-miR-1281
DCPS	hsa-miR-561-3p
DCPS	hsa-miR-675-3p
DCPS	hsa-miR-6797-5p
DCPS	hsa-miR-320e
DCPS	hsa-miR-4707-5p
DCPS	hsa-miR-7200-5p
DCPS	hsa-miR-519d-5p
DCPS	hsa-miR-1237-3p
DCPS	hsa-miR-659-3p
DCPS	hsa-miR-765
DCPS	hsa-miR-3943
DCPS	hsa-miR-551
DCPS	hsa-miR-5618-5p
DCPS	hsa-miR-190a-3p
DCPS	hsa-miR-1250-3p
DCPS	hsa-miR-769-5p
DCPS	hsa-miR-7019-5p
DCPS	hsa-miR-381-3p
DCPS	hsa-miR-7680-5p
DCPS	hsa-miR-205-3p
DCPS	hsa-miR-7005-3p
DCPS	hsa-miR-892-5p
DCPS	hsa-miR-34
DCPS	hsa-miR-34c-5p
DCPS	hsa-miR-3200-5p
DCPS	hsa-miR-516b-3p
DCPS	hsa-miR-6975-5p
DCPS	hsa-miR-6893-5p
DCPS	hsa-miR-186-5p
DCPS	hsa-miR-1305
DCPS	hsa-miR-4725-3p
DCPS	hsa-miR-7034-3p

DCPS	hsa-miR-3622b-5p
DCPS	hsa-miR-380
DCPS	hsa-miR-1178-3p
DCPS	hsa-miR-4667-5p
DCPS	hsa-miR-4313
DCPS	hsa-miR-6401
DCPS	hsa-miR-6320
DCPS	hsa-miR-1262
DCPS	hsa-miR-8076
DCPS	hsa-miR-504-5p.1
DCPS	hsa-miR-6799-5p
DCPS	hsa-miR-3158-5p
DCPS	hsa-miR-1224-5p
DCPS	hsa-miR-375
DCPS	hsa-miR-887-5p
DCPS	hsa-miR-6865-3p
DCPS	hsa-miR-452-5p
DCPS	hsa-miR-7076-5p
DCPS	hsa-miR-4765
DCPS	hsa-miR-141-3p
DCPS	hsa-miR-633
DCPS	hsa-miR-593-3p
DCPS	hsa-miR-6825-5p
DCPS	hsa-miR-1229-3p
DCPS	hsa-miR-5089-5p
DCPS	hsa-miR-6505-5p
DCPS	hsa-miR-6803-5p
DCPS	hsa-miR-6993-5p
DCPS	hsa-miR-466i-3p
DCPS	hsa-miR-6956-5p
DCPS	hsa-miR-4766-3p
DCPS	hsa-miR-1298-3p
DCPS	hsa-miR-3087-5p
DCPS	hsa-miR-185-3p
DCPS	hsa-miR-5088-3p
DCPS	hsa-miR-6824-5p
DCPS	hsa-miR-561-3p
DCPS	hsa-miR-4799-3p
DCPS	hsa-miR-3661
DCPS	hsa-miR-7207-3p
DCPS	hsa-miR-3078-5p
DCPS	hsa-miR-4742
DCPS	hsa-miR-27-3p
DCPS	hsa-let-7-5p
DCPS	hsa-miR-298-3p
DCPS	hsa-miR-125-5p

DCPS	hsa-miR-7088-5p
DCPS	hsa-miR-769-3p
DCPS	hsa-miR-5132-5p
DCPS	hsa-miR-345-5p
DCPS	hsa-miR-1943-5p
DCPS	hsa-miR-7032-3p
DCPS	hsa-miR-197-3p
DCPS	hsa-miR-495-3p
DCPS	hsa-miR-3680-3p
DCPS	hsa-miR-1434-3p
DCPS	hsa-miR-1904
DCPS	hsa-miR-7092-5p
DCPS	hsa-miR-3533
DCPS	hsa-miR-6830-3p
DCPS	hsa-miR-4291
DCPS	hsa-miR-7169-5p
DCPS	hsa-miR-6877-3p
DCPS	hsa-miR-1956-3p
DCPS	hsa-miR-491-5p
DCPS	hsa-miR-499a-3p
DCPS	hsa-miR-526b-5p
DCPS	hsa-miR-664
DCPS	hsa-miR-762
DCPS	hsa-miR-6355
DCPS	hsa-miR-8082
DCPS	hsa-miR-3072-5p
DCPS	hsa-miR-561-5p
DCPS	hsa-miR-433-3p
DCPS	hsa-miR-7033-5p
DCPS	hsa-miR-577
DCPS	hsa-miR-3167-3p
DCPS	hsa-miR-6948-3p
DCPS	hsa-miR-1969
DCPS	hsa-miR-6918-5p
DCPS	hsa-miR-219b-3p
DCPS	hsa-miR-549-3p
DCPS	hsa-miR-3144-5p
DCPS	hsa-miR-7042-3p
DCPS	hsa-miR-6889-5p
DCPS	hsa-miR-6314
DCPS	hsa-miR-7220-5p
DCPS	hsa-miR-583
DCPS	hsa-miR-5125
DCPS	hsa-miR-466mo-3p
DCPS	hsa-miR-367-3p
DCPS	hsa-miR-19b-5p

DCPS	hsa-miR-1238-3p
DCPS	hsa-miR-7019-3p
DCPS	hsa-miR-1322
DCPS	hsa-miR-7094-3p
DCPS	hsa-miR-374-5p
DCPS	hsa-miR-4680-3p
DCPS	hsa-miR-1202
DCPS	hsa-miR-208-5p
DCPS	hsa-miR-5196-5p
DCPS	hsa-miR-2276-3p
DCPS	hsa-miR-6751-5p
DCPS	hsa-miR-1927
DCPS	hsa-miR-3544-3p
DCPS	hsa-miR-7041-3p
DCPS	hsa-miR-3120-5p
DCPS	hsa-miR-30-3p
DCPS	hsa-miR-300-5p
DCPS	hsa-miR-421-3p
DCPS	hsa-miR-203a-3p.1
DCPS	hsa-miR-9-5p
DCPS	hsa-miR-493-3p
DCPS	hsa-miR-3099-5p
DCPS	hsa-miR-670
DCPS	hsa-miR-291-5p
DCPS	hsa-miR-497-5p
DCPS	hsa-miR-1930-3p
DCPS	hsa-miR-7285-5p
DCPS	hsa-miR-6346
DCPS	hsa-miR-16-5p
DCPS	hsa-miR-527
DCPS	hsa-miR-3621
DCPS	hsa-miR-432-5p
DCPS	hsa-miR-3084a-5p
DCPS	hsa-miR-548at-3p
DCPS	hsa-miR-623
DCPS	hsa-miR-577-5p
DCPS	hsa-miR-877-3p
DCPS	hsa-miR-3180-5p
DCPS	hsa-miR-5623-5p
DCPS	hsa-miR-591
DCPS	hsa-miR-8876
DCPS	hsa-miR-7162-3p
DCPS	hsa-miR-3652
DCPS	hsa-miR-4716
DCPS	hsa-miR-875-3p
DCPS	hsa-miR-4489

DCPS	hsa-miR-7211-5p
DCPS	hsa-miR-3178
DCPS	hsa-miR-34b-3p
DCPS	hsa-miR-548c-3p
DCPS	hsa-miR-1251
DCPS	hsa-miR-6500-5p
DCPS	hsa-miR-7648-3p
DCPS	hsa-miR-30e-3p
DCPS	hsa-miR-764-3p
DCPS	hsa-miR-587
DCPS	hsa-miR-4769-3p
DCPS	hsa-miR-573
DCPS	hsa-miR-2386
DCPS	hsa-miR-6404
DCPS	hsa-miR-20
DCPS	hsa-miR-7179-3p
DCPS	hsa-miR-219-1-3p
DCPS	hsa-miR-3076-5p
DCPS	hsa-miR-96-5p
DCPS	hsa-miR-8827
DCPS	hsa-miR-1231-3p
DCPS	hsa-miR-688
DCPS	hsa-miR-7688-3p
DCPS	hsa-miR-4498
DCPS	hsa-miR-25-3p
DCPS	hsa-miR-6081
DCPS	hsa-miR-1287
DCPS	hsa-miR-362-3p
DCPS	hsa-miR-4691-5p
DCPS	hsa-miR-3173-3p
DCPS	hsa-miR-6775-3p
DCPS	hsa-miR-630
DCPS	hsa-miR-7703
DCPS	hsa-miR-940
DCPS	hsa-miR-103a-2-5p
DCPS	hsa-miR-7668-5p
DCPS	hsa-miR-4514
DCPS	hsa-miR-7073-3p
DCPS	hsa-miR-6133
DCPS	hsa-miR-6979-5p
DCPS	hsa-miR-766-3p
DCPS	hsa-miR-1265
DCPS	hsa-miR-3073a-5p
DCPS	hsa-miR-7192-5p
DCPS	hsa-miR-562
DCPS	hsa-miR-1245-5p

DCPS	hsa-miR-6333
DCPS	hsa-miR-340-5p
DCPS	hsa-miR-545-3p
DCPS	hsa-miR-4277
DCPS	hsa-miR-548j-3p
DCPS	hsa-miR-3583-3p
DCPS	hsa-miR-7069-3p
DCPS	hsa-miR-743b-5p
DCPS	hsa-miR-613
DCPS	hsa-miR-496.2
DCPS	hsa-miR-6836-3p
DCPS	hsa-miR-4290
DCPS	hsa-miR-6967-3p
DCPS	hsa-miR-5581-5p
DCPS	hsa-miR-6972-5p
DCPS	hsa-miR-576-5p
DCPS	hsa-miR-8062
DCPS	hsa-miR-5702
DCPS	hsa-miR-544-3p
DCPS	hsa-miR-2284z
DCPS	hsa-miR-508-5p
DCPS	hsa-miR-136-3p
DCPS	hsa-miR-6796-3p
DCPS	hsa-miR-7234-5p
DCPS	hsa-miR-7198-3p
DCPS	hsa-miR-8057
DCPS	hsa-miR-686
DCPS	hsa-miR-3129-3p
DCPS	hsa-miR-526
DCPS	hsa-miR-7195-3p
DCPS	hsa-miR-6891-3p
DCPS	hsa-miR-495-3p
DCPS	hsa-miR-6736-5p
DCPS	hsa-miR-490-3p
DCPS	hsa-miR-615-3p
DCPS	hsa-miR-7160-5p
DCPS	hsa-miR-6080
DCPS	hsa-miR-6808-5p
DCPS	hsa-miR-4654
DCPS	hsa-miR-4781-3p
DCPS	hsa-miR-6130
DCPS	hsa-miR-297bc-3p
DCPS	hsa-miR-6947-5p
DCPS	hsa-miR-197-5p
DCPS	hsa-miR-3191-5p
DCPS	hsa-miR-7007-5p

DCPS	hsa-miR-516a
DCPS	hsa-miR-505-3p
DCPS	hsa-miR-1258-3p
DCPS	hsa-miR-21-3p
DCPS	hsa-miR-3164
DCPS	hsa-miR-106-3p
DCPS	hsa-miR-6821-3p
DCPS	hsa-miR-3934-3p
DCPS	hsa-miR-505-3p
DCPS	hsa-miR-1237-3p
DCPS	hsa-miR-5104
DCPS	hsa-miR-629-5p
DCPS	hsa-miR-6391
DCPS	hsa-miR-371a-5p
DCPS	hsa-miR-338-5p
DCPS	hsa-miR-32-5p
DCPS	hsa-miR-520g-3p
DCPS	hsa-miR-6937-5p
DCPS	hsa-miR-4300
DCPS	hsa-miR-6875-3p
DCPS	hsa-miR-7065-5p
DCPS	hsa-miR-7665-5p
DCPS	hsa-miR-6085
DCPS	hsa-miR-26-5p
DCPS	hsa-miR-550a-3p
DCPS	hsa-miR-548h-5p
DCPS	hsa-miR-6830-5p
DCPS	hsa-miR-6762-5p
DCPS	hsa-miR-651
DCPS	hsa-miR-193b-3p
DCPS	hsa-miR-6818-5p
DCPS	hsa-miR-4286
DCPS	hsa-miR-3184-3p
DCPS	hsa-miR-3135b
DCPS	hsa-miR-336-5p
DCPS	hsa-miR-5186
DCPS	hsa-miR-6873-5p
DCPS	hsa-miR-4698
DCPS	hsa-miR-7243-3p
DCPS	hsa-miR-382-5p
DCPS	hsa-miR-3103-3p
DCPS	hsa-miR-6925-3p
DCPS	hsa-miR-545-3p
DCPS	hsa-miR-4424
DCPS	hsa-miR-484
DCPS	hsa-miR-494

DCPS	hsa-miR-4423-5p
DCPS	hsa-miR-3620-5p
DCPS	hsa-miR-548aq-3p
DCPS	hsa-miR-4660
DCPS	hsa-miR-8904a
DCPS	hsa-miR-4651
DCPS	hsa-miR-2406
DCPS	hsa-miR-3591-5p
DCPS	hsa-miR-1825
DCPS	hsa-miR-214-5p
DCPS	hsa-miR-3558-3p
DCPS	hsa-miR-4478
DCPS	hsa-miR-705
DCPS	hsa-miR-299-3p
DCPS	hsa-miR-7032-5p
DCPS	hsa-miR-7208-3p
DCPS	hsa-miR-615-5p
DCPS	hsa-miR-1193-5p
DCPS	hsa-miR-485
DCPS	hsa-miR-7081-3p
DCPS	hsa-miR-6731-5p
DCPS	hsa-miR-4482-3p
DCPS	hsa-miR-944
DCPS	hsa-miR-2425-5p
DCPS	hsa-miR-669k-5p
DCPS	hsa-miR-7035-5p
DCPS	hsa-miR-34bc-3p
DCPS	hsa-miR-2114-5p
DCPS	hsa-miR-6815-5p
DCPS	hsa-miR-3561-3p
DCPS	hsa-miR-3618-5p
DCPS	hsa-miR-7211-3p
DCPS	hsa-miR-519e-5p
DCPS	hsa-miR-4272
DCPS	hsa-miR-2379
DCPS	hsa-miR-4711-3p
DCPS	hsa-miR-524-5p
DCPS	hsa-miR-642
DCPS	hsa-miR-4722-5p
DCPS	hsa-miR-6886-3p
DCPS	hsa-miR-6745
DCPS	hsa-miR-500
DCPS	hsa-miR-7008-3p
DCPS	hsa-miR-6744-3p
DCPS	hsa-miR-2399-5p
DCPS	hsa-miR-891b

DCPS	hsa-miR-597-3p
DCPS	hsa-miR-3192
DCPS	hsa-miR-488-3p
DCPS	hsa-miR-4422
DCPS	hsa-miR-489
DCPS	hsa-miR-6931-5p
DCPS	hsa-miR-6983-5p
DCPS	hsa-miR-470-3p
DCPS	hsa-miR-122-5p
DCPS	hsa-miR-669ao-3p
DCPS	hsa-miR-508-3p
DCPS	hsa-miR-6740-3p
DCPS	hsa-miR-4793-5p
DCPS	hsa-miR-511
DCPS	hsa-miR-1721
DCPS	hsa-miR-545-5p
DCPS	hsa-miR-6968-3p
DCPS	hsa-miR-2110
DCPS	hsa-miR-876
DCPS	hsa-miR-5110
DCPS	hsa-miR-6864-5p
DCPS	hsa-miR-3067-5p
DCPS	hsa-miR-103-2-5p
DCPS	hsa-miR-323a-5p
DCPS	hsa-miR-4766-5p
DCPS	hsa-miR-568
DCPS	hsa-miR-6837-5p
DCPS	hsa-miR-3689d
DCPS	hsa-miR-296-3p
DCPS	hsa-miR-1910-5p
DCPS	hsa-miR-3672
DCPS	hsa-miR-203-3p
DCPS	hsa-miR-939-5p
DCPS	hsa-miR-6957-5p
DCPS	hsa-miR-15
DCPS	hsa-miR-1237-5p
DCPS	hsa-miR-596
DCPS	hsa-miR-3089-3p
DCPS	hsa-let-7abf-3p
DCPS	hsa-miR-4639-5p
DCPS	hsa-miR-449-5p
DCPS	hsa-miR-4264
DCPS	hsa-miR-6899-3p
DCPS	hsa-miR-370-3p
DCPS	hsa-miR-3686
DCPS	hsa-miR-5590-5p

DCPS	hsa-miR-7169-3p
DCPS	hsa-miR-4786-5p
DCPS	hsa-miR-6990-5p
DCPS	hsa-miR-6928-5p
DCPS	hsa-miR-3552
DCPS	hsa-miR-6943-3p
DCPS	hsa-miR-672-3p
DCPS	hsa-miR-7683-3p
DCPS	hsa-miR-23a-5p
DCPS	hsa-miR-382-3p
DCPS	hsa-miR-4419a
DCPS	hsa-miR-892-3p
DCPS	hsa-miR-6974-3p
DCPS	hsa-miR-5581-3p
DCPS	hsa-miR-7010-3p
DCPS	hsa-miR-665-3p
DCPS	hsa-miR-95-5p
DCPS	hsa-miR-5681b
DCPS	hsa-miR-1262-5p
DCPS	hsa-miR-2284aa
DCPS	hsa-miR-363-5p
DCPS	hsa-miR-548as-3p
DCPS	hsa-miR-3078-3p
DCPS	hsa-miR-373-3p
DCPS	hsa-miR-471-3p
DCPS	hsa-miR-7266-3p
DCPS	hsa-miR-182-3p
DCPS	hsa-miR-4316
DCPS	hsa-miR-6358
DCPS	hsa-miR-6889-3p
DCPS	hsa-miR-207
DCPS	hsa-miR-1290
DCPS	hsa-miR-1271-5p
DCPS	hsa-miR-6785-5p
DCPS	hsa-miR-3106-3p
DCPS	hsa-miR-6722-5p
DCPS	hsa-miR-7863
DCPS	hsa-miR-6895-3p
DCPS	hsa-miR-2895
DCPS	hsa-miR-6899-5p
DCPS	hsa-miR-3106-5p
DCPS	hsa-miR-943
DCPS	hsa-miR-3577-5p
DCPS	hsa-miR-7231-5p
DCPS	hsa-miR-3160-5p
DCPS	hsa-miR-1298

DCPS	hsa-miR-6329
DCPS	hsa-miR-6771-3p
DCPS	hsa-miR-378d
DCPS	hsa-miR-134
DCPS	hsa-miR-2278
DCPS	hsa-miR-7012-5p
DCPS	hsa-miR-608
DCPS	hsa-miR-874-3p
DCPS	hsa-miR-6777-3p
DCPS	hsa-miR-216a-5p
DCPS	hsa-miR-544b
DCPS	hsa-miR-6976-3p
DCPS	hsa-miR-7201-3p
DCPS	hsa-miR-593-5p
DCPS	hsa-miR-193
DCPS	hsa-miR-6769b-5p
DCPS	hsa-miR-6126
DCPS	hsa-miR-619-3p
DCPS	hsa-miR-551-3p
DCPS	hsa-miR-376a-5p
DCPS	hsa-miR-7164-5p
DCPS	hsa-miR-7250-3p
DCPS	hsa-miR-7016-3p
DCPS	hsa-miR-27-5p
DCPS	hsa-miR-8870
DCPS	hsa-miR-548f-5p
DCPS	hsa-miR-2457
DCPS	hsa-miR-23-3p
DCPS	hsa-miR-606
DCPS	hsa-miR-4802-3p
DCPS	hsa-miR-6529-5p
DCPS	hsa-miR-8813
DCPS	hsa-miR-7158-5p
DCPS	hsa-miR-4493
DCPS	hsa-miR-3096-3p
DCPS	hsa-miR-2054
DCPS	hsa-miR-4722-3p
DCPS	hsa-miR-340-3p
DCPS	hsa-miR-6845-5p
DCPS	hsa-miR-1285-3p
DCPS	hsa-miR-6914-5p
DCPS	hsa-miR-550-5p
DCPS	hsa-miR-599-5p
DCPS	hsa-miR-650d
DCPS	hsa-miR-7-5p
DCPS	hsa-miR-1297

DCPS	hsa-miR-5114
DCPS	hsa-miR-6855-3p
DCPS	hsa-miR-548ac
DCPS	hsa-miR-6860
DCPS	hsa-miR-627
DCPS	hsa-miR-4697-3p
DCPS	hsa-miR-671-5p
DCPS	hsa-miR-377-5p
DCPS	hsa-miR-191-3p
DCPS	hsa-miR-4425
DCPS	hsa-miR-7206-5p
DCPS	hsa-miR-7088-3p
DCPS	hsa-miR-7691
DCPS	hsa-miR-7852-3p
DCPS	hsa-miR-548x-3p
DCPS	hsa-miR-8839
DCPS	hsa-miR-200ab-5p
DCPS	hsa-miR-19a-5p
DCPS	hsa-miR-548aj-3p
DCPS	hsa-miR-665
DCPS	hsa-miR-4256
DCPS	hsa-miR-519-5p
DCPS	hsa-miR-3136-5p
DCPS	hsa-miR-6814-5p
DCPS	hsa-miR-548cej-3p
DCPS	hsa-miR-200bc-3p
DCPS	hsa-miR-411-5p
DCPS	hsa-miR-493-5p
DCPS	hsa-miR-6165
DCPS	hsa-miR-7224-5p
DCPS	hsa-miR-548ap-3p
DCPS	hsa-miR-6134
DCPS	hsa-miR-380-3p
DCPS	hsa-miR-3112-3p
DCPS	hsa-let-7
DCPS	hsa-miR-6962-3p
DCPS	hsa-miR-153
DCPS	hsa-miR-7106-5p
DCPS	hsa-miR-6852-5p
DCPS	hsa-miR-3101-3p
DCPS	hsa-miR-19-5p
DCPS	hsa-miR-2894
DCPS	hsa-miR-383-3p
DCPS	hsa-miR-3977
DCPS	hsa-miR-5588-5p
DCPS	hsa-miR-653

DCPS	hsa-miR-505-3p.1
DCPS	hsa-miR-548al
DCPS	hsa-miR-3670
DCPS	hsa-miR-2432
DCPS	hsa-miR-337-3p
DCPS	hsa-miR-711
DCPS	hsa-miR-2116-3p
DCPS	hsa-miR-8055
DCPS	hsa-miR-2904
DCPS	hsa-miR-1253
DCPS	hsa-miR-6954-3p
DCPS	hsa-miR-6732-3p
DCPS	hsa-miR-4666a-5p
DCPS	hsa-miR-548d-5p
DCPS	hsa-miR-3431
DCPS	hsa-miR-939-5p
DCPS	hsa-miR-494-3p
DCPS	hsa-miR-3190-5p
DCPS	hsa-miR-330-5p
DCPS	hsa-miR-193b-5p
DCPS	hsa-miR-5002-3p
DCPS	hsa-miR-4659a-3p
DCPS	hsa-miR-374a-3p
DCPS	hsa-miR-6892-3p
DCPS	hsa-miR-4271
DCPS	hsa-miR-3179
DCPS	hsa-miR-7233-3p
DCPS	hsa-miR-8836
DCPS	hsa-miR-4719
DCPS	hsa-miR-500a-3p
DCPS	hsa-miR-936
DCPS	hsa-miR-3130-3p
DCPS	hsa-miR-133-3p
DCPS	hsa-miR-612
DCPS	hsa-miR-193-3p
DCPS	hsa-miR-7020-5p
DCPS	hsa-miR-4441
DCPS	hsa-miR-3911
DCPS	hsa-miR-138-5p
DCPS	hsa-miR-887
DCPS	hsa-miR-7050-5p
DCPS	hsa-miR-1894-3p
DCPS	hsa-miR-5189-3p
DCPS	hsa-miR-1287-5p
DCPS	hsa-miR-7653-3p
DCPS	hsa-miR-1954

DCPS	hsa-miR-7117-3p
DCPS	hsa-miR-139-3p
DCPS	hsa-miR-1277-5p
DCPS	hsa-miR-3605-3p
DCPS	hsa-miR-373-5p
DCPS	hsa-miR-6734-5p
DCPS	hsa-miR-93-5p
DCPS	hsa-miR-7171-3p
DCPS	hsa-miR-8892
DCPS	hsa-miR-4650
DCPS	hsa-miR-4308
DCPS	hsa-miR-6735-5p
DCPS	hsa-miR-517
DCPS	hsa-miR-7200-3p
DCPS	hsa-miR-7349-3p
DCPS	hsa-miR-145-3p
DCPS	hsa-miR-6925-5p
DCPS	hsa-miR-1188-5p
DCPS	hsa-miR-92a-1-5p
DCPS	hsa-miR-4777-3p
DCPS	hsa-miR-3572-5p
DCPS	hsa-miR-6964-5p
DCPS	hsa-miR-7062-5p
DCPS	hsa-miR-6761-5p
DCPS	hsa-miR-371b-5p
DCPS	hsa-miR-153-3p
DCPS	hsa-miR-6846-5p
DCPS	hsa-miR-2437
DCPS	hsa-miR-136-5p
DCPS	hsa-miR-3653-3p
DCPS	hsa-miR-2477
DCPS	hsa-miR-5697-3p
DCPS	hsa-miR-147-5p
DCPS	hsa-miR-4727-5p
DCPS	hsa-miR-4294
DCPS	hsa-miR-6878-5p
DCPS	hsa-miR-1260a
DCPS	hsa-miR-4716-5p
DCPS	hsa-miR-741-3p
DCPS	hsa-miR-8079
DCPS	hsa-miR-3065-5p
DCPS	hsa-miR-679-5p
DCPS	hsa-miR-2380
DCPS	hsa-miR-590-3p
DCPS	hsa-miR-1948-5p
DCPS	hsa-miR-3690

DCPS	hsa-miR-433
DCPS	hsa-miR-552-5p
DCPS	hsa-miR-656-5p
DCPS	hsa-miR-1838
DCPS	hsa-miR-26
DCPS	hsa-miR-486-5p
DCPS	hsa-miR-1388-3p
DCPS	hsa-miR-4257
DCPS	hsa-miR-135a-1-3p
DCPS	hsa-miR-588
DCPS	hsa-miR-5699-3p
DCPS	hsa-miR-133a-3p.1
DCPS	hsa-miR-106
DCPS	hsa-miR-8085
DCPS	hsa-miR-7212-3p
DCPS	hsa-miR-6963-3p
DCPS	hsa-miR-8849
DCPS	hsa-miR-7108-3p
DCPS	hsa-miR-3074-2-3p
DCPS	hsa-miR-6740-5p
DCPS	hsa-miR-581
DCPS	hsa-miR-16-5p
DCPS	hsa-miR-4733-5p
DCPS	hsa-miR-7082-3p
DCPS	hsa-miR-4674
DCPS	hsa-miR-2285t
DCPS	hsa-miR-201-3p
DCPS	hsa-miR-3542
DCPS	hsa-miR-92a-2-5p
DCPS	hsa-miR-4676-5p
DCPS	hsa-miR-216a-3p
DCPS	hsa-miR-4694-3p
DCPS	hsa-miR-136-5p
DCPS	hsa-miR-627-5p
DCPS	hsa-miR-3473c
DCPS	hsa-miR-1273f
DCPS	hsa-miR-7231-3p
DCPS	hsa-miR-6848-3p
DCPS	hsa-miR-7689-3p
DCPS	hsa-miR-5135
DCPS	hsa-miR-3573-5p
DCPS	hsa-miR-5787
DCPS	hsa-miR-1246
DCPS	hsa-miR-6910-3p
DCPS	hsa-miR-15c-3p
DCPS	hsa-miR-6774-5p

DCPS	hsa-miR-7351-5p
DCPS	hsa-miR-6348
DCPS	hsa-miR-3065-3p
DCPS	hsa-miR-6371
DCPS	hsa-miR-6323
DCPS	hsa-miR-129-5p
DCPS	hsa-miR-6801-3p
DCPS	hsa-miR-875
DCPS	hsa-miR-6131
DCPS	hsa-miR-7162-5p
DCPS	hsa-miR-4649-3p
DCPS	hsa-miR-664b-5p
DCPS	hsa-miR-7017-5p
DCPS	hsa-miR-133-3p.1
DCPS	hsa-miR-1193
DCPS	hsa-miR-4776-3p
DCPS	hsa-miR-4643
DCPS	hsa-miR-4707-3p
DCPS	hsa-miR-761
DCPS	hsa-miR-2052
DCPS	hsa-miR-670-5p
DCPS	hsa-miR-450b-3p
DCPS	hsa-miR-147a
DCPS	hsa-miR-363-3p
DCPS	hsa-miR-891
DCPS	hsa-miR-320-5p
DCPS	hsa-miR-1814a
DCPS	hsa-miR-8831
DCPS	hsa-miR-300
DCPS	hsa-miR-7052-3p
DCPS	hsa-miR-3085
DCPS	hsa-miR-8885
DCPS	hsa-miR-466f-3p
DCPS	hsa-miR-6089
DCPS	hsa-miR-186-5p
DCPS	hsa-miR-432-3p
DCPS	hsa-miR-770
DCPS	hsa-miR-6351
DCPS	hsa-miR-7286-3p
DCPS	hsa-miR-6911-5p
DCPS	hsa-miR-3099-3p
DCPS	hsa-miR-4288
DCPS	hsa-miR-3557-5p
DCPS	hsa-miR-4486
DCPS	hsa-miR-1205
DCPS	hsa-miR-30c-3p

DCPS	hsa-miR-556-5p
DCPS	hsa-miR-298
DCPS	hsa-miR-31-5p
DCPS	hsa-miR-676-3p
DCPS	hsa-miR-219-3p
DCPS	hsa-miR-1273g-3p
DCPS	hsa-miR-4739
DCPS	hsa-miR-709
DCPS	hsa-miR-1266-3p
DCPS	hsa-miR-8080
DCPS	hsa-miR-4692
DCPS	hsa-miR-7090-5p
DCPS	hsa-miR-105b
DCPS	hsa-miR-8908-5p
DCPS	hsa-miR-6331
DCPS	hsa-miR-3132
DCPS	hsa-miR-520gh
DCPS	hsa-miR-20-3p
DCPS	hsa-miR-338-3p
DCPS	hsa-miR-924
DCPS	hsa-miR-8853
DCPS	hsa-miR-6752-5p
DCPS	hsa-miR-6239
DCPS	hsa-miR-28-5p
DCPS	hsa-miR-3187-3p
DCPS	hsa-miR-3175
DCPS	hsa-miR-2461-5p
DCPS	hsa-miR-491-5p
DCPS	hsa-miR-7077-3p
DCPS	hsa-miR-6948-5p
DCPS	hsa-miR-467-3p
DCPS	hsa-miR-522-3p
DCPS	hsa-miR-4690-5p
DCPS	hsa-miR-3913-3p
DCPS	hsa-miR-2308
DCPS	hsa-miR-8108
DCPS	hsa-miR-548g-3p
DCPS	hsa-miR-7199-3p
DCPS	hsa-miR-370
DCPS	hsa-miR-1539
DCPS	hsa-miR-7173-5p
DCPS	hsa-miR-3113-5p
DCPS	hsa-miR-6324
DCPS	hsa-miR-890-5p
DCPS	hsa-miR-3928-3p
DCPS	hsa-miR-216b-5p

DCPS	hsa-miR-6971-3p
DCPS	hsa-miR-4289
DCPS	hsa-miR-708-5p
DCPS	hsa-miR-465abc-3p
DCPS	hsa-miR-6354
DCPS	hsa-miR-4785
DCPS	hsa-miR-548aef
DCPS	hsa-miR-5194
DCPS	hsa-miR-7056-5p
DCPS	hsa-miR-365-3p
DCPS	hsa-miR-486-3p
DCPS	hsa-miR-764
DCPS	hsa-miR-892b
DCPS	hsa-miR-1302
DCPS	hsa-miR-367-3p
DCPS	hsa-miR-6071
DCPS	hsa-miR-1261
DCPS	hsa-miR-622
DCPS	hsa-miR-6895-5p
DCPS	hsa-miR-1252-5p
DCPS	hsa-miR-7218-3p
DCPS	hsa-miR-4708-5p
DCPS	hsa-miR-6817-3p
DCPS	hsa-miR-7846-3p
DCPS	hsa-miR-328-3p
DCPS	hsa-miR-6849-3p
DCPS	hsa-miR-7191-5p
DCPS	hsa-miR-193-5p
DCPS	hsa-miR-3592
DCPS	hsa-miR-3140-3p
DCPS	hsa-miR-7205-3p
DCPS	hsa-miR-1301-5p
DCPS	hsa-miR-514b-5p
DCPS	hsa-miR-6988-3p
DCPS	hsa-miR-4305
DCPS	hsa-miR-141-5p
DCPS	hsa-miR-15a-3p
DCPS	hsa-miR-203-3p.1
DCPS	hsa-miR-3662
DCPS	hsa-miR-3692-3p
DCPS	hsa-miR-8097
DCPS	hsa-miR-4314
DCPS	hsa-miR-103b
DCPS	hsa-miR-4731-5p
DCPS	hsa-miR-5125-5p
DCPS	hsa-miR-7360-5p

DCPS	hsa-miR-635
DCPS	hsa-miR-344i
DCPS	hsa-miR-1843a-5p
DCPS	hsa-miR-33-5p
DCPS	hsa-miR-1273e
DCPS	hsa-miR-3927-3p
DCPS	hsa-miR-5690
DCPS	hsa-miR-6775-5p
DCPS	hsa-miR-153-5p
DCPS	hsa-miR-6514-3p
DCPS	hsa-miR-653-5p
DCPS	hsa-miR-5692bc
DCPS	hsa-miR-421-5p
DCPS	hsa-miR-206-5p
DCPS	hsa-miR-3929
DCPS	hsa-miR-370-5p
DCPS	hsa-miR-4750-3p
DCPS	hsa-miR-7174-5p
DCPS	hsa-miR-8832
DCPS	hsa-miR-6819-3p
DCPS	hsa-miR-204-3p
DCPS	hsa-miR-628-3p
DCPS	hsa-miR-4513
DCPS	hsa-miR-3194-3p
DCPS	hsa-miR-7224-3p
DCPS	hsa-miR-2397-5p
DCPS	hsa-miR-677-5p
DCPS	hsa-miR-3173-3p
DCPS	hsa-miR-130-3p
DCPS	hsa-miR-4535
DCPS	hsa-miR-656
DCPS	hsa-miR-7118-5p
DCPS	hsa-miR-6788-5p
DCPS	hsa-miR-7009-5p
DCPS	hsa-miR-1835
DCPS	hsa-miR-5698
DCPS	hsa-miR-680
DCPS	hsa-miR-301b-5p
DCPS	hsa-miR-1256
DCPS	hsa-miR-7227-5p
DCPS	hsa-miR-22-3p
DCPS	hsa-miR-2902
DCPS	hsa-miR-5093
DCPS	hsa-miR-6513-5p
DCPS	hsa-miR-4534
DCPS	hsa-miR-6806-5p

DCPS	hsa-miR-4791
DCPS	hsa-miR-7175-3p
DCPS	hsa-miR-376e
DCPS	hsa-miR-3074-3p
DCPS	hsa-miR-1267
DCPS	hsa-miR-7068-3p
DCPS	hsa-miR-3577-3p
DCPS	hsa-miR-3116
DCPS	hsa-miR-3663-3p
DCPS	hsa-miR-454-5p
DCPS	hsa-miR-2285m
DCPS	hsa-miR-7218-5p
DCPS	hsa-miR-450-5p
DCPS	hsa-miR-8906
DCPS	hsa-miR-767-3p
DCPS	hsa-miR-4775
DCPS	hsa-miR-4708-3p
DCPS	hsa-miR-1245a
DCPS	hsa-miR-1252
DCPS	hsa-miR-8842
DCPS	hsa-miR-4729
DCPS	hsa-miR-3085-3p
DCPS	hsa-miR-5101
DCPS	hsa-miR-5107-3p
DCPS	hsa-miR-140-3p.1
DCPS	hsa-miR-7083-3p
DCPS	hsa-miR-4279
DCPS	hsa-miR-6980-3p
DCPS	hsa-miR-7210-3p
DCPS	hsa-miR-429-5p
DCPS	hsa-miR-6957-3p
DCPS	hsa-miR-513a
DCPS	hsa-miR-1668
DCPS	hsa-miR-3079-3p
DCPS	hsa-miR-378
DCPS	hsa-miR-6360
DCPS	hsa-miR-1191b-3p
DCPS	hsa-miR-4745-5p
DCPS	hsa-miR-7314-3p
DCPS	hsa-let-7g-3p
DCPS	hsa-miR-409-5p
DCPS	hsa-miR-4668-5p
DCPS	hsa-miR-210-5p
DCPS	hsa-miR-1915-3p
DCPS	hsa-miR-5623-3p
DCPS	hsa-miR-6829-5p

DCPS	hsa-miR-3964
DCPS	hsa-miR-4685-5p
DCPS	hsa-miR-6073
DCPS	hsa-miR-3161
DCPS	hsa-miR-1-3p
DCPS	hsa-miR-3475-3p
DCPS	hsa-miR-3120-3p
DCPS	hsa-miR-7094-1-5p
DCPS	hsa-miR-7073-5p
DCPS	hsa-miR-7111-5p
DCPS	hsa-miR-6527
DCPS	hsa-miR-7214-3p
DCPS	hsa-miR-548az-3p
DCPS	hsa-miR-6315
DCPS	hsa-miR-6760-3p
DCPS	hsa-miR-7851-3p
DCPS	hsa-miR-7008-5p
DCPS	hsa-miR-544a
DCPS	hsa-miR-3157-5p
DCPS	hsa-miR-548ah-3p
DCPS	hsa-miR-3975
DCPS	hsa-miR-3120-3p
DCPS	hsa-miR-6403
DCPS	hsa-miR-6750-3p
DCPS	hsa-miR-759
DCPS	hsa-miR-6756-5p
DCPS	hsa-miR-124-5p
DCPS	hsa-miR-452-3p
DCPS	hsa-miR-7156-5p
DCPS	hsa-miR-5580-5p
DCPS	hsa-miR-4419b
DCPS	hsa-miR-200c-3p
DCPS	hsa-miR-7025-3p
DCPS	hsa-miR-6870-5p
DCPS	hsa-miR-8797
DCPS	hsa-miR-548ae-3p
DCPS	hsa-miR-6779-5p
DCPS	hsa-miR-7176-5p
DCPS	hsa-miR-2358
DCPS	hsa-miR-7195-5p
DCPS	hsa-miR-6788-3p
DCPS	hsa-miR-876-3p
DCPS	hsa-miR-922
DCPS	hsa-miR-2467-5p
DCPS	hsa-miR-4763-5p
DCPS	hsa-miR-1905

DCPS	hsa-miR-6908-5p
DCPS	hsa-miR-4796-3p
DCPS	hsa-miR-4260
DCPS	hsa-miR-499b-3p
DCPS	hsa-miR-4326
DCPS	hsa-miR-1836
DCPS	hsa-miR-492
DCPS	hsa-miR-6514-5p
DCPS	hsa-miR-4483
DCPS	hsa-miR-29b-2-5p
DCPS	hsa-miR-133b-5p
DCPS	hsa-miR-5192
DCPS	hsa-miR-6789-5p
DCPS	hsa-miR-374b-5p
DCPS	hsa-miR-3684
DCPS	hsa-miR-6763-3p
DCPS	hsa-miR-2285x
DCPS	hsa-miR-647
DCPS	hsa-miR-2682-3p
DCPS	hsa-miR-1892
DCPS	hsa-miR-2464-3p
DCPS	hsa-miR-7193-3p
DCPS	hsa-miR-154-5p
DCPS	hsa-miR-2114-5p
DCPS	hsa-miR-1225
DCPS	hsa-miR-6864-3p
DCPS	hsa-miR-657
DCPS	hsa-miR-2401
DCPS	hsa-miR-182-5p
DCPS	hsa-miR-466f-5p
DCPS	hsa-miR-6902-3p
DCPS	hsa-miR-6862-3p
DCPS	hsa-miR-1306-3p
DCPS	hsa-miR-1289
DCPS	hsa-miR-6950-3p
DCPS	hsa-miR-507
DCPS	hsa-miR-6502-5p
DCPS	hsa-miR-766-3p
DCPS	hsa-miR-142
DCPS	hsa-miR-7024-3p
DCPS	hsa-miR-890
DCPS	hsa-miR-7845-5p
DCPS	hsa-miR-7081-5p
DCPS	hsa-miR-548am-3p
DCPS	hsa-miR-293-3p
DCPS	hsa-miR-4251

DCPS	hsa-miR-378a-5p
DCPS	hsa-miR-1285-3p
DCPS	hsa-miR-4470
DCPS	hsa-miR-7091-5p
DCPS	hsa-miR-7189-3p
DCPS	hsa-miR-7-3p
DCPS	hsa-miR-3572-3p
DCPS	hsa-miR-6861-3p
DCPS	hsa-miR-8805
DCPS	hsa-miR-3094-5p
DCPS	hsa-miR-7398l-5p
DCPS	hsa-miR-8887
DCPS	hsa-miR-3075
DCPS	hsa-miR-199-3p
DCPS	hsa-miR-3974
DCPS	hsa-miR-1226-3p
DCPS	hsa-miR-3150a-3p
DCPS	hsa-miR-1247-3p
DCPS	hsa-miR-6764-3p
DCPS	hsa-miR-7657-3p
DCPS	hsa-miR-6824-3p
DCPS	hsa-miR-155-3p
DCPS	hsa-miR-4476
DCPS	hsa-miR-7684-3p
DCPS	hsa-miR-146b-3p
DCPS	hsa-miR-7157-3p
DCPS	hsa-miR-1276
DCPS	hsa-miR-215-5p
DCPS	hsa-miR-6965-3p
DCPS	hsa-miR-632
DCPS	hsa-miR-6976-5p
DCPS	hsa-miR-383
DCPS	hsa-miR-1227-3p
DCPS	hsa-miR-7864
DCPS	hsa-miR-7240-5p
DCPS	hsa-miR-6129
DCPS	hsa-miR-5700
DCPS	hsa-miR-7848-3p
DCPS	hsa-miR-6732-5p
DCPS	hsa-miR-7664-3p
DCPS	hsa-miR-550b-3p
DCPS	hsa-miR-6727-5p
DCPS	hsa-miR-1343-3p
DCPS	hsa-miR-3061-3p
DCPS	hsa-miR-522-3p
DCPS	hsa-miR-743ab-3p

DCPS	hsa-miR-1972
DCPS	hsa-miR-5691
DCPS	hsa-miR-7161-5p
DCPS	hsa-miR-34-5p
DCPS	hsa-miR-7254-5p
DCPS	hsa-miR-409-3p
DCPS	hsa-miR-7028-3p
DCPS	hsa-miR-410
DCPS	hsa-miR-8119
DCPS	hsa-miR-7048-3p
DCPS	hsa-miR-4703-3p
DCPS	hsa-miR-7219-5p
DCPS	hsa-miR-8804
DCPS	hsa-miR-548g-5p
DCPS	hsa-miR-7058-5p
DCPS	hsa-miR-548aj-5p
DCPS	hsa-miR-3649
DCPS	hsa-miR-187-5p
DCPS	hsa-miR-8092
DCPS	hsa-miR-224-5p
DCPS	hsa-miR-5189-5p
DCPS	hsa-miR-4700-3p
DCPS	hsa-miR-548i-5p
DCPS	hsa-miR-7398r-5p
DCPS	hsa-miR-497-3p
DCPS	hsa-miR-6847-5p
DCPS	hsa-miR-8837
DCPS	hsa-miR-667-3p
DCPS	hsa-miR-450b-5p
DCPS	hsa-miR-6932-3p
DCPS	hsa-miR-670-3p
DCPS	hsa-miR-3090-3p
DCPS	hsa-miR-6935-5p
DCPS	hsa-miR-3612
DCPS	hsa-miR-8808
DCPS	hsa-miR-6800-5p
DCPS	hsa-miR-4761-3p
DCPS	hsa-miR-2315
DCPS	hsa-miR-2426
DCPS	hsa-miR-885-3p
DCPS	hsa-miR-6832-5p
DCPS	hsa-miR-4480
DCPS	hsa-miR-4773
DCPS	hsa-miR-6980-5p
DCPS	hsa-miR-6793-5p
DCPS	hsa-miR-6991-3p

DCPS	hsa-miR-519
DCPS	hsa-miR-6784-3p
DCPS	hsa-miR-6825-3p
DCPS	hsa-miR-1547-3p
DCPS	hsa-miR-540-5p
DCPS	hsa-miR-322-5p
DCPS	hsa-miR-6842-3p
DCPS	hsa-miR-8877
DCPS	hsa-miR-3163
DCPS	hsa-miR-7048-5p
DCPS	hsa-miR-6728-3p
DCPS	hsa-miR-107-3p
DCPS	hsa-miR-5572
DCPS	hsa-miR-619-5p
DCPS	hsa-miR-6317
DCPS	hsa-miR-8052
DCPS	hsa-miR-4736
DCPS	hsa-miR-4673
DCPS	hsa-miR-1250
DCPS	hsa-miR-3668
DCPS	hsa-miR-3202
DCPS	hsa-miR-4704-3p
DCPS	hsa-miR-4529-5p
DCPS	hsa-miR-344-5p
DCPS	hsa-miR-518a-5p
DCPS	hsa-miR-3140-5p
DCPS	hsa-miR-8077
DCPS	hsa-miR-7181-5p
DCPS	hsa-miR-140-5p
DCPS	hsa-miR-532-5p
DCPS	hsa-miR-7116-5p
DCPS	hsa-miR-93-3p
DCPS	hsa-miR-5096
DCPS	hsa-miR-7108-5p
DCPS	hsa-miR-185-5p
DCPS	hsa-miR-6883-5p
DCPS	hsa-miR-5703
DCPS	hsa-miR-4632-5p
DCPS	hsa-miR-2382-5p
DCPS	hsa-miR-4633-3p
DCPS	hsa-miR-6512-5p
DCPS	hsa-miR-3973
DCPS	hsa-miR-517-5p
DCPS	hsa-miR-677-3p
DCPS	hsa-miR-3076-3p
DCPS	hsa-miR-6926-3p

DCPS	hsa-miR-2376
DCPS	hsa-miR-661
DCPS	hsa-miR-4433a-5p
DCPS	hsa-miR-6807-5p
DCPS	hsa-miR-1283
DCPS	hsa-miR-673-5p
DCPS	hsa-miR-1952
DCPS	hsa-miR-519e-3p
DCPS	hsa-miR-3940-3p
DCPS	hsa-miR-214-3p
DCPS	hsa-miR-4433b-3p
DCPS	hsa-miR-4516
DCPS	hsa-miR-4492
DCPS	hsa-miR-202-3p
DCPS	hsa-miR-6865-5p
DCPS	hsa-miR-2285s
DCPS	hsa-miR-3617-5p
DCPS	hsa-miR-10
DCPS	hsa-miR-4787-5p
DCPS	hsa-miR-26b-3p
DCPS	hsa-miR-215
DCPS	hsa-miR-674-5p
DCPS	hsa-miR-2447
DCPS	hsa-miR-4743-5p
DCPS	hsa-miR-6918-3p
DCPS	hsa-miR-8873b
DCPS	hsa-miR-6868-3p
DCPS	hsa-miR-3620-3p
DCPS	hsa-miR-2309
DCPS	hsa-miR-6516-5p
DCPS	hsa-miR-6843-3p
DCPS	hsa-miR-6778-3p
DCPS	hsa-miR-496a-3p.1
DCPS	hsa-miR-3122
DCPS	hsa-miR-6857-5p
DCPS	hsa-miR-152-3p
DCPS	hsa-miR-3110-5p
DCPS	hsa-miR-4747-5p
DCPS	hsa-miR-2378
DCPS	hsa-miR-1270
DCPS	hsa-miR-1245a
DCPS	hsa-miR-6920-5p
DCPS	hsa-miR-3096-5p
DCPS	hsa-miR-2486-5p
DCPS	hsa-miR-576-3p
DCPS	hsa-miR-542-3p

DCPS	hsa-miR-7649-3p
DCPS	hsa-miR-2957
DCPS	hsa-miR-885-5p
DCPS	hsa-miR-7173-3p
DCPS	hsa-miR-4725-5p
DCPS	hsa-miR-7113-5p
DCPS	hsa-miR-8093
DCPS	hsa-miR-135b-3p
DCPS	hsa-miR-7655-3p
DCPS	hsa-miR-424-5p
DCPS	hsa-miR-580
DCPS	hsa-miR-6771-5p
DCPS	hsa-miR-6501-3p
DCPS	hsa-miR-1226-3p
DCPS	hsa-miR-3182
DCPS	hsa-miR-7684-5p
DCPS	hsa-miR-185-5p
DCPS	hsa-miR-3664-5p
DCPS	hsa-miR-676-5p
DCPS	hsa-miR-411-5p
DCPS	hsa-miR-4668-3p
DCPS	hsa-miR-668-3p
DCPS	hsa-miR-4797-3p
DCPS	hsa-miR-6528
DCPS	hsa-miR-7049-5p
DCPS	hsa-miR-381-5p
DCPS	hsa-miR-3568
DCPS	hsa-miR-6356
DCPS	hsa-miR-4439
DCPS	hsa-miR-7066-5p
DCPS	hsa-miR-802-3p
DCPS	hsa-miR-194-5p
DCPS	hsa-miR-4318
DCPS	hsa-miR-212-5p
DCPS	hsa-miR-202-3p
DCPS	hsa-miR-2891
DCPS	hsa-miR-4309
DCPS	hsa-miR-7166-3p
DCPS	hsa-miR-361-5p
DCPS	hsa-miR-3136-5p
DCPS	hsa-miR-8893
DCPS	hsa-miR-1304-5p
DCPS	hsa-miR-548bd-3p
DCPS	hsa-miR-3124-3p
DCPS	hsa-miR-8059
DCPS	hsa-miR-4646-5p

DCPS	hsa-miR-192-5p
DCPS	hsa-miR-6938-3p
DCPS	hsa-miR-1545b-3p
DCPS	hsa-miR-1942
DCPS	hsa-miR-6516-3p
DCPS	hsa-miR-2454-3p
DCPS	hsa-miR-1304-3p
DCPS	hsa-miR-6418-3p
DCPS	hsa-miR-7178-3p
DCPS	hsa-miR-3189-3p
DCPS	hsa-miR-4255
DCPS	hsa-miR-4504
DCPS	hsa-miR-1230
DCPS	hsa-miR-4292
DCPS	hsa-miR-138-2-3p
DCPS	hsa-miR-6733-3p
DCPS	hsa-miR-6748-3p
DCPS	hsa-miR-548h-3p
DCPS	hsa-miR-6986-5p
DCPS	hsa-miR-764-5p
DCPS	hsa-miR-411-5p.2
DCPS	hsa-miR-381-3p
DCPS	hsa-miR-6983-3p
DCPS	hsa-miR-499b-3p
DCPS	hsa-miR-1260a
DCPS	hsa-miR-1194
DCPS	hsa-miR-1188-3p
DCPS	hsa-miR-4757-5p
DCPS	hsa-miR-624-3p
DCPS	hsa-miR-6862-5p
DCPS	hsa-miR-204-5p
DCPS	hsa-miR-6365
DCPS	hsa-miR-6835-5p
DCPS	hsa-miR-513a-5p
DCPS	hsa-miR-6511-5p
DCPS	hsa-miR-1226-5p
DCPS	hsa-miR-7075-5p
DCPS	hsa-miR-107
DCPS	hsa-miR-664a-5p
DCPS	hsa-miR-4446-5p
DCPS	hsa-miR-6817-5p
DCPS	hsa-miR-146a-3p
DCPS	hsa-miR-7005-5p
DCPS	hsa-miR-377-3p
DCPS	hsa-miR-5582-5p
DCPS	hsa-miR-6922-3p

DCPS	hsa-miR-1909-3p
DCPS	hsa-miR-636
DCPS	hsa-miR-142-3p.2
DCPS	hsa-miR-6086
DCPS	hsa-miR-25-3p
DCPS	hsa-miR-766-5p
DCPS	hsa-miR-4749-3p
DCPS	hsa-miR-1262-3p
DCPS	hsa-miR-6758-3p
DCPS	hsa-miR-548z
DCPS	hsa-miR-7002-5p
DCPS	hsa-miR-3201
DCPS	hsa-miR-548aw
DCPS	hsa-miR-92
DCPS	hsa-miR-7154-5p
DCPS	hsa-miR-6128
DCPS	hsa-miR-326
DCPS	hsa-miR-1179
DCPS	hsa-miR-519-3p
DCPS	hsa-miR-6845-3p
DCPS	hsa-miR-106-5p
DCPS	hsa-miR-1251-3p
DCPS	hsa-miR-4485-5p
DCPS	hsa-miR-345-3p
DCPS	hsa-miR-431-5p
DCPS	hsa-miR-466ade-3p
DCPS	hsa-miR-569
DCPS	hsa-miR-1178-3p
DCPS	hsa-miR-467g
DCPS	hsa-miR-7177-5p
DCPS	hsa-miR-4539
DCPS	hsa-miR-6856-5p
DCPS	hsa-miR-3671
DCPS	hsa-miR-1234-3p
DCPS	hsa-miR-30b-3p
DCPS	hsa-miR-6940-5p
DCPS	hsa-miR-3070-3p
DCPS	hsa-miR-20-5p
DCPS	hsa-miR-155-5p
DCPS	hsa-miR-669a-3-3p
DCPS	hsa-miR-550
DCPS	hsa-miR-380-5p
DCPS	hsa-miR-6790-3p
DCPS	hsa-miR-7182-3p
DCPS	hsa-miR-365a-3p
DCPS	hsa-miR-4653-5p

DCPS	hsa-miR-3622a-3p
DCPS	hsa-miR-515-3p
DCPS	hsa-miR-4312
DCPS	hsa-miR-2115-3p
DCPS	hsa-miR-410-3p
DCPS	hsa-miR-6894-3p
DCPS	hsa-miR-1204
DCPS	hsa-miR-133
DCPS	hsa-miR-1243-3p
DCPS	hsa-miR-500b-3p
DCPS	hsa-miR-3191-3p
DCPS	hsa-miR-6840-3p
DCPS	hsa-miR-216-3p
DCPS	hsa-miR-514a-5p
DCPS	hsa-miR-32-5p
DCPS	hsa-miR-548bb-3p
DCPS	hsa-miR-7843-5p
DCPS	hsa-miR-7053-5p
DCPS	hsa-miR-4659b-5p
DCPS	hsa-miR-5187-3p
DCPS	hsa-miR-25-5p
DCPS	hsa-miR-550b-2-5p
DCPS	hsa-miR-4293
DCPS	hsa-miR-7183-3p
DCPS	hsa-miR-3165
DCPS	hsa-miR-6842-5p
DCPS	hsa-miR-5583-5p
DCPS	hsa-miR-7067-5p
DCPS	hsa-miR-2371
DCPS	hsa-miR-98-5p
DCPS	hsa-miR-6505-3p
DCPS	hsa-miR-7269-3p
DCPS	hsa-miR-1-3p
DCPS	hsa-miR-3134
DCPS	hsa-miR-421
DCPS	hsa-miR-697
DCPS	hsa-miR-6127
DCPS	hsa-miR-1301-3p
DCPS	hsa-miR-3613-3p
DCPS	hsa-miR-466-5p
DCPS	hsa-miR-194-3p
DCPS	hsa-miR-200c-5p
DCPS	hsa-miR-210-3p
DCPS	hsa-miR-324-3p
DCPS	hsa-miR-505-3p.2
DCPS	hsa-miR-3922-5p

DCPS	hsa-miR-7119-5p
DCPS	hsa-miR-6813-5p
DCPS	hsa-miR-6783-5p
DCPS	hsa-miR-7641
DCPS	hsa-miR-181b-1-3p
DCPS	hsa-miR-6529-3p
DCPS	hsa-miR-2382-3p
DCPS	hsa-miR-4298
DCPS	hsa-miR-6791-5p
DCPS	hsa-miR-642a-5p
DCPS	hsa-miR-7843-3p
DCPS	hsa-miR-128-1-5p
DCPS	hsa-miR-504
DCPS	hsa-miR-152-5p
DCPS	hsa-miR-7074-5p
DCPS	hsa-miR-3606-5p
DCPS	hsa-miR-150-5p
DCPS	hsa-miR-2286
DCPS	hsa-miR-6338
DCPS	hsa-miR-4524a-3p
DCPS	hsa-miR-7667-5p
DCPS	hsa-miR-7065-3p
DCPS	hsa-miR-4659a-5p
DCPS	hsa-miR-466q
DCPS	hsa-miR-3092-5p
DCPS	hsa-miR-584-3p
DCPS	hsa-miR-2284-5p
DCPS	hsa-miR-1966-5p
DCPS	hsa-miR-6939-5p
DCPS	hsa-miR-4640-5p
DCPS	hsa-miR-7657-5p
DCPS	hsa-miR-548d-3p
DCPS	hsa-miR-6975-3p
DCPS	hsa-miR-4762-3p
DCPS	hsa-miR-219a-2-3p
DCPS	hsa-miR-6729-3p
DCPS	hsa-miR-6996-5p
DCPS	hsa-miR-7152-3p
DCPS	hsa-miR-873-5p
DCPS	hsa-miR-454-3p
DCPS	hsa-miR-2331-3p
DCPS	hsa-miR-450a-2-3p
DCPS	hsa-miR-6873-3p
DCPS	hsa-miR-105-5p
DCPS	hsa-miR-548at-5p
DCPS	hsa-miR-6981-5p

DCPS	hsa-miR-448-5p
DCPS	hsa-miR-7681-3p
DCPS	hsa-miR-103-5p
DCPS	hsa-miR-3070-5p
DCPS	hsa-miR-2311
DCPS	hsa-miR-7037-3p
DCPS	hsa-miR-619
DCPS	hsa-miR-548x-5p
DCPS	hsa-miR-1245-3p
DCPS	hsa-miR-6794-3p
DCPS	hsa-miR-6995-3p
DCPS	hsa-miR-1264-5p
DCPS	hsa-miR-4477a
DCPS	hsa-miR-7024-5p
DCPS	hsa-miR-520-3p
DCPS	hsa-miR-4665-3p
DCPS	hsa-miR-6510-5p
DCPS	hsa-miR-4472
DCPS	hsa-miR-7188-5p
DCPS	hsa-miR-196-5p
DCPS	hsa-miR-656-3p
DCPS	hsa-miR-6888-3p
DCPS	hsa-miR-668
DCPS	hsa-miR-2368-3p
DCPS	hsa-miR-3084-5p
DCPS	hsa-miR-519b-3p
DCPS	hsa-miR-7191-3p
DCPS	hsa-miR-4532
DCPS	hsa-miR-6950-5p
DCPS	hsa-miR-6935-3p
DCPS	hsa-miR-5580-3p
DCPS	hsa-miR-7275-3p
DCPS	hsa-miR-6809-5p
DCPS	hsa-miR-429
DCPS	hsa-miR-626
DCPS	hsa-miR-4999-3p
DCPS	hsa-miR-4420
DCPS	hsa-miR-7203-3p
DCPS	hsa-miR-1827
DCPS	hsa-miR-6940-3p
DCPS	hsa-miR-4650-5p
DCPS	hsa-miR-7028-5p
DCPS	hsa-miR-33a-3p
DCPS	hsa-miR-7181-3p
DCPS	hsa-miR-3544
DCPS	hsa-miR-4638-3p

DCPS	hsa-miR-3584-5p
DCPS	hsa-miR-155-5p
DCPS	hsa-miR-7188-3p
DCPS	hsa-miR-5583-3p
DCPS	hsa-miR-3126-5p
DCPS	hsa-miR-3591-3p
DCPS	hsa-miR-450a
DCPS	hsa-miR-7213-3p
DCPS	hsa-miR-3110-3p
DCPS	hsa-miR-301a-5p
DCPS	hsa-miR-6916-5p
DCPS	hsa-miR-939-3p
DCPS	hsa-miR-557
DCPS	hsa-miR-8829
DCPS	hsa-miR-6874-5p
DCPS	hsa-miR-6856-3p
DCPS	hsa-miR-4329
DCPS	hsa-miR-659-5p
DCPS	hsa-miR-4675
DCPS	hsa-miR-488-3p
DCPS	hsa-miR-433-5p
DCPS	hsa-miR-5090
DCPS	hsa-miR-6945-5p
DCPS	hsa-miR-2276-5p
DCPS	hsa-miR-5697
DCPS	hsa-miR-4446-3p
DCPS	hsa-miR-361-5p
DCPS	hsa-miR-4427
DCPS	hsa-miR-8113
DCPS	hsa-miR-6756-3p
DCPS	hsa-miR-513c-5p
DCPS	hsa-miR-628-5p
DCPS	hsa-miR-7296-5p
DCPS	hsa-miR-550a-3-5p
DCPS	hsa-miR-744-3p
DCPS	hsa-miR-8848
DCPS	hsa-miR-4684-5p
DCPS	hsa-miR-7168-3p
DCPS	hsa-miR-1245b-5p
DCPS	hsa-miR-6416-3p
DCPS	hsa-miR-4474-3p
DCPS	hsa-miR-4699-5p
DCPS	hsa-miR-5582-3p
DCPS	hsa-miR-3665
DCPS	hsa-miR-409
DCPS	hsa-miR-515-5p

DCPS	hsa-miR-432-5p
DCPS	hsa-miR-576-5p
DCPS	hsa-miR-4505
DCPS	hsa-miR-542-5p
DCPS	hsa-miR-548ay-3p
DCPS	hsa-miR-5133
DCPS	hsa-miR-200a-5p
DCPS	hsa-miR-2458
DCPS	hsa-miR-6504-3p
DCPS	hsa-miR-3616-3p
DCPS	hsa-miR-644a
DCPS	hsa-miR-742-5p
DCPS	hsa-miR-548e-3p
DCPS	hsa-miR-190-5p
DCPS	hsa-miR-2330-5p
DCPS	hsa-miR-483-3p
DCPS	hsa-miR-8886
DCPS	hsa-miR-2115-5p
DCPS	hsa-miR-374
DCPS	hsa-miR-7087-5p
DCPS	hsa-miR-548aa
DCPS	hsa-miR-4531
DCPS	hsa-miR-4735-5p
DCPS	hsa-miR-192-3p
DCPS	hsa-miR-570-3p
DCPS	hsa-miR-3913-5p
DCPS	hsa-miR-767-5p
DCPS	hsa-miR-6987-5p
DCPS	hsa-miR-8840
DCPS	hsa-miR-1197
DCPS	hsa-miR-142-3p
DCPS	hsa-miR-2373-3p
DCPS	hsa-miR-3167-5p
DCPS	hsa-miR-6392-3p
DCPS	hsa-miR-3614-5p
DCPS	hsa-miR-4797-5p
DCPS	hsa-miR-369-3p
DCPS	hsa-miR-6857-3p
DCPS	hsa-miR-1950
DCPS	hsa-miR-215-3p
DCPS	hsa-miR-6736-3p
DCPS	hsa-miR-6715-5p
DCPS	hsa-miR-6811-3p
DCPS	hsa-miR-7110-5p
DCPS	hsa-miR-4695-5p
DCPS	hsa-miR-1236-5p

DCPS	hsa-miR-6072
DCPS	hsa-miR-743b-3p
DCPS	hsa-miR-708-5p
DCPS	hsa-miR-2301
DCPS	hsa-miR-6749-3p
DCPS	hsa-miR-4726-3p
DCPS	hsa-miR-7018-3p
DCPS	hsa-miR-129-2-3p
DCPS	hsa-miR-7025-5p
DCPS	hsa-miR-137-3p
DCPS	hsa-miR-1184
DCPS	hsa-miR-4746-3p
DCPS	hsa-miR-208a-5p
DCPS	hsa-miR-3541
DCPS	hsa-miR-6992-5p
DCPS	hsa-miR-548t-3p
DCPS	hsa-miR-1895
DCPS	hsa-miR-6823-5p
DCPS	hsa-miR-3174
DCPS	hsa-miR-706
DCPS	hsa-miR-499-5p
DCPS	hsa-miR-7159-5p
DCPS	hsa-miR-6529
DCPS	hsa-miR-7249-3p
DCPS	hsa-miR-2421
DCPS	hsa-miR-7006-3p
DCPS	hsa-miR-6405
DCPS	hsa-miR-372-5p
DCPS	hsa-miR-6747-3p
DCPS	hsa-miR-2897
DCPS	hsa-miR-296-5p
DCPS	hsa-miR-7686-5p
DCPS	hsa-miR-93-5p
DCPS	hsa-miR-7207-5p
DCPS	hsa-miR-7217-5p
DCPS	hsa-miR-6827-5p
DCPS	hsa-miR-7298-3p
DCPS	hsa-miR-6368
DCPS	hsa-miR-29a-5p
DCPS	hsa-miR-7652-3p
DCPS	hsa-miR-3691-3p
DCPS	hsa-miR-1249-5p
DCPS	hsa-miR-6876-3p
DCPS	hsa-miR-184
DCPS	hsa-miR-3543
DCPS	hsa-miR-7186-3p

DCPS	hsa-miR-4743-3p
DCPS	hsa-miR-597
DCPS	hsa-miR-6332
DCPS	hsa-miR-510
DCPS	hsa-miR-4717-5p
DCPS	hsa-miR-3156-3p
DCPS	hsa-miR-5704
DCPS	hsa-miR-339-5p
DCPS	hsa-miR-8814
DCPS	hsa-miR-8872
DCPS	hsa-miR-548n
DCPS	hsa-miR-3622b-3p
DCPS	hsa-miR-3095-3p
DCPS	hsa-miR-6752-3p
DCPS	hsa-miR-7216-5p
DCPS	hsa-miR-6879-3p
DCPS	hsa-miR-379-5p
DCPS	hsa-miR-4779
DCPS	hsa-miR-5693
DCPS	hsa-miR-6766-5p
DCPS	hsa-miR-1197-3p
DCPS	hsa-miR-6743-5p
DCPS	hsa-miR-505-5p
DCPS	hsa-miR-6720-5p
DCPS	hsa-miR-6827-3p
DCPS	hsa-miR-579-3p
DCPS	hsa-miR-3119
DCPS	hsa-miR-7660-3p
DCPS	hsa-miR-539-3p
DCPS	hsa-miR-873a-5p.1
DCPS	hsa-miR-212-3p
DCPS	hsa-miR-7386j-3p
DCPS	hsa-miR-3121-3p
DCPS	hsa-miR-6726-5p
DCPS	hsa-miR-6974-5p
DCPS	hsa-miR-6727-3p
DCPS	hsa-miR-128-3p
DCPS	hsa-miR-5108
DCPS	hsa-miR-1912-3p
DCPS	hsa-miR-7676-5p
DCPS	hsa-miR-615-3p
DCPS	hsa-miR-6802-5p
DCPS	hsa-miR-301-5p
DCPS	hsa-miR-4436b-5p
DCPS	hsa-miR-29-3p
DCPS	hsa-miR-4252

DCPS	hsa-miR-892c-3p
DCPS	hsa-miR-7977
DCPS	hsa-miR-7274-3p
DCPS	hsa-miR-3474
DCPS	hsa-miR-8090
DCPS	hsa-miR-375-3p
DCPS	hsa-miR-6795-3p
DCPS	hsa-miR-1249-3p
DCPS	hsa-miR-4446
DCPS	hsa-miR-7215-3p
DCPS	hsa-miR-4428
DCPS	hsa-miR-203
DCPS	hsa-miR-124-3p.1
DCPS	hsa-miR-3064-5p
DCPS	hsa-miR-3569
DCPS	hsa-miR-3121-3p
DCPS	hsa-miR-30a-3p
DCPS	hsa-miR-6882-3p
DCPS	hsa-miR-3147
DCPS	hsa-miR-3084-3p
DCPS	hsa-miR-4697-5p
DCPS	hsa-miR-518
DCPS	hsa-miR-98-5p
DCPS	hsa-miR-24-3p
DCPS	hsa-miR-6984-5p
DCPS	hsa-miR-5011-5p
DCPS	hsa-miR-548ar-3p
DCPS	hsa-miR-1323-3p
DCPS	hsa-miR-3607-5p
DCPS	hsa-miR-343
DCPS	hsa-miR-3653-5p
DCPS	hsa-miR-2387
DCPS	hsa-miR-6722-3p
DCPS	hsa-miR-128
DCPS	hsa-miR-3074-5p
DCPS	hsa-miR-7110-3p
DCPS	hsa-let-7c-3p
DCPS	hsa-miR-571
DCPS	hsa-miR-6417
DCPS	hsa-miR-7202-5p
DCPS	hsa-miR-145a-3p
DCPS	hsa-miR-424-5p
DCPS	hsa-miR-6077
DCPS	hsa-miR-197-3p
DCPS	hsa-miR-1233-3p
DCPS	hsa-miR-4768-3p

DCPS	hsa-miR-6917-3p
DCPS	hsa-miR-1245b-3p
DCPS	hsa-miR-6721-5p
DCPS	hsa-miR-302bd
DCPS	hsa-miR-294
DCPS	hsa-miR-382-5p
DCPS	hsa-miR-7166-5p
DCPS	hsa-miR-34a-3p
DCPS	hsa-miR-6132
DCPS	hsa-miR-6715b-5p
DCPS	hsa-miR-4710
DCPS	hsa-miR-1323
DCPS	hsa-miR-184-5p
DCPS	hsa-miR-6995-5p
DCPS	hsa-miR-7057-5p
DCPS	hsa-miR-6851-5p
DCPS	hsa-miR-548a-3p
DCPS	hsa-miR-181-3p
DCPS	hsa-miR-33
DCPS	hsa-miR-1982-3p
DCPS	hsa-miR-338-3p
DCPS	hsa-miR-134-5p
DCPS	hsa-miR-223-3p
DCPS	hsa-miR-652-5p
DCPS	hsa-miR-7227-3p
DCPS	hsa-miR-292b-3p
DCPS	hsa-miR-2439-5p
DCPS	hsa-miR-142-3p.1
DCPS	hsa-miR-6930-3p
DCPS	hsa-miR-7354-5p
DCPS	hsa-miR-1976
DCPS	hsa-miR-342-5p
DCPS	hsa-miR-669
DCPS	hsa-miR-1231-5p
DCPS	hsa-miR-669h-5p
DCPS	hsa-miR-607
DCPS	hsa-miR-4742-3p
DCPS	hsa-miR-502-5p
DCPS	hsa-miR-133b
DCPS	hsa-miR-130-5p
DCPS	hsa-miR-19-3p
DCPS	hsa-miR-4434
DCPS	hsa-miR-7185-3p
DCPS	hsa-miR-7850-5p
DCPS	hsa-miR-143-5p
DCPS	hsa-miR-124

DCPS	hsa-miR-5000-3p
DCPS	hsa-miR-195-5p
DCPS	hsa-miR-146-5p
DCPS	hsa-miR-7176-3p
DCPS	hsa-miR-3173-5p
DCPS	hsa-miR-8791
DCPS	hsa-miR-320b
DCPS	hsa-miR-4254
DCPS	hsa-miR-548e-5p
DCPS	hsa-miR-9768-3p
DCPS	hsa-miR-6540-3p
DCPS	hsa-miR-8900
DCPS	hsa-miR-1207-5p
DCPS	hsa-miR-378g
DCPS	hsa-miR-3667-3p
DCPS	hsa-miR-4645-5p
DCPS	hsa-miR-4445-3p
DCPS	hsa-miR-221-5p
DCPS	hsa-miR-7119-3p
DCPS	hsa-miR-6909-3p
DCPS	hsa-miR-6321
DCPS	hsa-miR-4263
DCPS	hsa-miR-6839-3p
DCPS	hsa-miR-4646-3p
DCPS	hsa-miR-361-3p
DCPS	hsa-miR-3557-3p
DCPS	hsa-miR-7222-3p
DCPS	hsa-miR-8846
DCPS	hsa-miR-7236-5p
DCPS	hsa-miR-3942-3p
DCPS	hsa-miR-8100
DCPS	hsa-miR-4520-5p
DCPS	hsa-miR-1266
DCPS	hsa-miR-6804-3p
DCPS	hsa-miR-6773-3p
DCPS	hsa-miR-3685
DCPS	hsa-miR-465d-3p
DCPS	hsa-miR-218-5p
DCPS	hsa-miR-351-5p
DCPS	hsa-miR-3593-5p
DCPS	hsa-miR-24-3p
DCPS	hsa-miR-384-5p
DCPS	hsa-miR-3688-3p
DCPS	hsa-miR-2322-3p
DCPS	hsa-miR-6912-5p
DCPS	hsa-miR-7011-5p

DCPS	hsa-miR-3127-3p
DCPS	hsa-miR-6929-5p
DCPS	hsa-miR-3473
DCPS	hsa-miR-4786-3p
DCPS	hsa-miR-140-3p
DCPS	hsa-miR-5587-5p
DCPS	hsa-miR-6764-5p
DCPS	hsa-miR-7687-3p
DCPS	hsa-miR-879-5p
DCPS	hsa-miR-5588-3p
DCPS	hsa-miR-646
DCPS	hsa-miR-6500-3p
DCPS	hsa-miR-190a-5p
DCPS	hsa-miR-411-5p.1
DCPS	hsa-miR-1224
DCPS	hsa-miR-3655
DCPS	hsa-miR-7184-5p
DCPS	hsa-miR-4728-5p
DCPS	hsa-miR-873-5p.2
DCPS	hsa-miR-667-5p
DCPS	hsa-let-7a-2-3p
DCPS	hsa-miR-548f-3p
DCPS	hsa-miR-26-3p
DCPS	hsa-miR-374b-3p
DCPS	hsa-miR-539-5p
DCPS	hsa-miR-2317
DCPS	hsa-miR-4657
DCPS	hsa-miR-5008-3p
DCPS	hsa-miR-7197-3p
DCPS	hsa-miR-1251-5p
DCPS	hsa-miR-6751-3p
DCPS	hsa-miR-6930-5p
DCPS	hsa-miR-30-5p
DCPS	hsa-miR-7004-3p
DCPS	hsa-miR-7153-3p
DCPS	hsa-miR-669p-3p
DCPS	hsa-miR-548c-3p
DCPS	hsa-miR-329-3p
DCPS	hsa-miR-3187-5p
DCPS	hsa-miR-7242-3p
DCPS	hsa-miR-4687-3p
DCPS	hsa-miR-3198
DCPS	hsa-miR-4433a-3p
DCPS	hsa-miR-637
DCPS	hsa-miR-742-3p
DCPS	hsa-miR-346

DCPS	hsa-miR-4761-5p
DCPS	hsa-miR-6765-3p
DCPS	hsa-miR-188-3p
DCPS	hsa-miR-20a-3p
DCPS	hsa-miR-2053
DCPS	hsa-miR-138-5p
DCPS	hsa-miR-6791-3p
DCPS	hsa-miR-4701-3p
DCPS	hsa-miR-7973
DCPS	hsa-miR-149-3p
DCPS	hsa-miR-2389
DCPS	hsa-miR-2483-5p
DCPS	hsa-miR-302c-3p
DCPS	hsa-miR-6942-5p
DCPS	hsa-miR-7238-3p
DCPS	hsa-miR-6907-5p
DCPS	hsa-miR-8823
DCPS	hsa-miR-4659b-3p
DCPS	hsa-miR-3547-5p
DCPS	hsa-miR-6943-5p
DCPS	hsa-miR-4700-5p
DCPS	hsa-miR-582-3p
DCPS	hsa-miR-4686
DCPS	hsa-miR-4481
DCPS	hsa-miR-1227-3p
DCPS	hsa-miR-210
DCPS	hsa-miR-4278
DCPS	hsa-miR-4443
DCPS	hsa-miR-4530
DCPS	hsa-miR-3714
DCPS	hsa-miR-7022-5p
DCPS	hsa-miR-503-3p
DCPS	hsa-miR-7046-5p
DCPS	hsa-miR-7180
DCPS	hsa-miR-17-3p
DCPS	hsa-miR-290b-5p
DCPS	hsa-miR-4270
DCPS	hsa-miR-4683
DCPS	hsa-miR-5615-5p
DCPS	hsa-miR-3188
DCPS	hsa-miR-4772-3p
DCPS	hsa-miR-873-3p
DCPS	hsa-miR-6730-3p
DCPS	hsa-miR-4663
DCPS	hsa-miR-1941-3p
DCPS	hsa-miR-150-5p

DCPS	hsa-miR-330-3p.1
DCPS	hsa-miR-599
DCPS	hsa-miR-7398-5p
DCPS	hsa-miR-758-3p
DCPS	hsa-miR-6719-3p
DCPS	hsa-miR-4268
DCPS	hsa-miR-6989-5p
DCPS	hsa-miR-452-5p
DCPS	hsa-miR-377-3p
DCPS	hsa-miR-4743-5p
DCPS	hsa-miR-9500
DCPS	hsa-miR-372-3p
DCPS	hsa-miR-320-3p
DCPS	hsa-miR-616-5p
DCPS	hsa-miR-4713-5p
DCPS	hsa-miR-6769a-3p
DCPS	hsa-miR-346-3p
DCPS	hsa-miR-769
DCPS	hsa-miR-1343
DCPS	hsa-miR-7043-5p
DCPS	hsa-miR-140-5p
DCPS	hsa-miR-881-5p
DCPS	hsa-miR-551-5p
DCPS	hsa-miR-506-3p
DCPS	hsa-miR-149-5p
DCPS	hsa-miR-7013-5p
DCPS	hsa-miR-7157-5p
DCPS	hsa-miR-3189-5p
DCPS	hsa-miR-4732-3p
DCPS	hsa-miR-525-5p
DCPS	hsa-miR-698-3p
DCPS	hsa-miR-143-3p
DCPS	hsa-miR-2917
DCPS	hsa-miR-451b
DCPS	hsa-miR-579
DCPS	hsa-miR-6818-3p
DCPS	hsa-miR-6122-3p
DCPS	hsa-miR-4310
DCPS	hsa-miR-1468-3p
DCPS	hsa-miR-485-5p
DCPS	hsa-miR-7150
DCPS	hsa-miR-3976
DCPS	hsa-miR-7022-3p
DCPS	hsa-miR-223-3p
DCPS	hsa-miR-7094b-2-5p
DCPS	hsa-miR-6376

DCPS	hsa-miR-617
DCPS	hsa-miR-2467-3p
DCPS	hsa-miR-498
DCPS	hsa-miR-3544-5p
DCPS	hsa-miR-483-3p
DCPS	hsa-miR-1254
DCPS	hsa-miR-6879-5p
DCPS	hsa-miR-5011-3p
DCPS	hsa-miR-937-5p
DCPS	hsa-miR-5119
DCPS	hsa-miR-4440
DCPS	hsa-miR-28-5p
DCPS	hsa-miR-1227-5p
DCPS	hsa-miR-181
DCPS	hsa-miR-4436b-3p
DCPS	hsa-miR-214-3p
DCPS	hsa-miR-644a
DCPS	hsa-miR-5001-3p
DCPS	hsa-miR-6990-3p
DCPS	hsa-miR-455-3p.2
DCPS	hsa-miR-1208
DCPS	hsa-miR-200a-3p
DCPS	hsa-miR-3157-3p
DCPS	hsa-miR-466
DCPS	hsa-miR-6754-5p
DCPS	hsa-miR-4459
DCPS	hsa-miR-4297
DCPS	hsa-miR-6810-5p
DCPS	hsa-miR-1291
DCPS	hsa-miR-8060
DCPS	hsa-miR-3109-3p
DCPS	hsa-miR-892a
DCPS	hsa-miR-345-5p
DCPS	hsa-miR-8862
DCPS	hsa-miR-4652-3p
DCPS	hsa-miR-4508
DCPS	hsa-miR-3192-5p
DCPS	hsa-miR-1282
DCPS	hsa-miR-7021-3p
DCPS	hsa-miR-5008-5p
DCPS	hsa-miR-8106
DCPS	hsa-miR-1909-5p
DCPS	hsa-miR-938
DCPS	hsa-miR-7026-5p
DCPS	hsa-miR-448
DCPS	hsa-miR-3945

DCPS	hsa-miR-183-5p
DCPS	hsa-miR-6909-5p
DCPS	hsa-miR-7064-5p
DCPS	hsa-miR-7662-5p
DCPS	hsa-miR-6847-3p
DCPS	hsa-miR-487-5p
DCPS	hsa-miR-331-3p
DCPS	hsa-miR-4758-5p
DCPS	hsa-miR-6499-3p
DCPS	hsa-miR-4721
DCPS	hsa-miR-6537-5p
DCPS	hsa-miR-7855-5p
DCPS	hsa-miR-6780b-5p
DCPS	hsa-miR-6790-5p
DCPS	hsa-miR-7079-5p
DCPS	hsa-miR-874
DCPS	hsa-miR-5004-3p
DCPS	hsa-miR-17-5p
DCPS	hsa-miR-1185-5p
DCPS	hsa-miR-549-5p
DCPS	hsa-miR-6792-3p
DCPS	hsa-miR-4667
DCPS	hsa-miR-7685-3p
DCPS	hsa-miR-6934-3p
DCPS	hsa-miR-6826-3p
DCPS	hsa-miR-3194-5p
DCPS	hsa-miR-516a-3p
DCPS	hsa-miR-6506-5p
DCPS	hsa-miR-4253
DCPS	hsa-miR-143-3p
DCPS	hsa-miR-6868-5p
DCPS	hsa-miR-548l
DCPS	hsa-miR-7170-3p
DCPS	hsa-miR-921
DCPS	hsa-miR-6781-3p
DCPS	hsa-miR-593-3p
DCPS	hsa-miR-6829-3p
DCPS	hsa-miR-7331-5p
DCPS	hsa-miR-344g-3p
DCPS	hsa-miR-6511-3p
DCPS	hsa-miR-885
DCPS	hsa-miR-563
DCPS	hsa-miR-4720-5p
DCPS	hsa-miR-1967
DCPS	hsa-miR-2285z
DCPS	hsa-miR-7220-3p

DCPS	hsa-miR-6780a-3p
DCPS	hsa-miR-510-5p
DCPS	hsa-miR-4709-5p
DCPS	hsa-miR-4437
DCPS	hsa-miR-6776-5p
DCPS	hsa-miR-331-3p
DCPS	hsa-miR-4803
DCPS	hsa-miR-6808-3p
DCPS	hsa-miR-2377
DCPS	hsa-miR-499
DCPS	hsa-miR-133cd-5p
DCPS	hsa-miR-8807
DCPS	hsa-miR-3920
DCPS	hsa-miR-2422
DCPS	hsa-miR-3063-3p
DCPS	hsa-miR-378-3p
DCPS	hsa-miR-6885-3p
DCPS	hsa-miR-6418-5p
DCPS	hsa-miR-34b-3p
DCPS	hsa-miR-6513-3p
DCPS	hsa-miR-7184-3p
DCPS	hsa-miR-6985-5p
DCPS	hsa-miR-5120
DCPS	hsa-miR-1224-3p
DCPS	hsa-miR-6758-5p
DCPS	hsa-miR-7082-5p
DCPS	hsa-miR-4794
DCPS	hsa-miR-4802-5p
DCPS	hsa-miR-511-5p
DCPS	hsa-miR-6782-5p
DCPS	hsa-miR-1271-3p
DCPS	hsa-miR-4526
DCPS	hsa-miR-3679-3p
DCPS	hsa-miR-5134-5p
DCPS	hsa-miR-3692-5p
DCPS	hsa-miR-3617-3p
DCPS	hsa-miR-125b-2-3p
DCPS	hsa-miR-1240
DCPS	hsa-miR-3651
DCPS	hsa-miR-7023-3p
DCPS	hsa-let-7ag-3p
DCPS	hsa-miR-20b-3p
DCPS	hsa-miR-677
DCPS	hsa-miR-383-5p.1
DCPS	hsa-miR-6780a-5p
DCPS	hsa-miR-7-5p

DCPS	hsa-miR-3691-5p
DCPS	hsa-miR-4521
DCPS	hsa-miR-7107-5p
DCPS	hsa-miR-3108-3p
DCPS	hsa-miR-5695
DCPS	hsa-miR-6742-3p
DCPS	hsa-miR-497-5p
DCPS	hsa-miR-344-3p
DCPS	hsa-miR-605-3p
DCPS	hsa-miR-6947-3p
DCPS	hsa-miR-6794-5p
DCPS	hsa-miR-6377
DCPS	hsa-miR-7241-3p
DCPS	hsa-miR-4320
DCPS	hsa-miR-760-5p
DCPS	hsa-miR-1962
EIF3D	hsa-miR-590-3p
EIF3D	hsa-miR-4650
EIF3D	hsa-miR-513b-3p
EIF3D	hsa-miR-548u
EIF3D	hsa-miR-3095-3p
EIF3D	hsa-miR-4436b-5p
EIF3D	hsa-miR-7168-5p
EIF3D	hsa-miR-6768-3p
EIF3D	hsa-miR-4514
EIF3D	hsa-miR-6517
EIF3D	hsa-miR-7084-3p
EIF3D	hsa-miR-4419a
EIF3D	hsa-miR-126-3p
EIF3D	hsa-miR-3165
EIF3D	hsa-miR-4655-3p
EIF3D	hsa-miR-4474-5p
EIF3D	hsa-miR-1843
EIF3D	hsa-miR-8088
EIF3D	hsa-miR-7212-3p
EIF3D	hsa-miR-3126-5p
EIF3D	hsa-miR-215
EIF3D	hsa-miR-508-5p
EIF3D	hsa-miR-7161-5p
EIF3D	hsa-miR-6946-5p
EIF3D	hsa-miR-28-5p
EIF3D	hsa-miR-2472
EIF3D	hsa-miR-7683-3p
EIF3D	hsa-miR-3153
EIF3D	hsa-miR-6934-3p
EIF3D	hsa-miR-5584-5p

EIF3D	hsa-miR-6509-3p
EIF3D	hsa-miR-763
EIF3D	hsa-miR-1721
EIF3D	hsa-miR-3069-3p
EIF3D	hsa-miR-6392-3p
EIF3D	hsa-miR-8854
EIF3D	hsa-miR-98-5p
EIF3D	hsa-miR-350
EIF3D	hsa-miR-7032-3p
EIF3D	hsa-miR-451b
EIF3D	hsa-miR-8080
EIF3D	hsa-miR-4686
EIF3D	hsa-miR-185-5p
EIF3D	hsa-miR-6511a-5p
EIF3D	hsa-miR-1910-3p
EIF3D	hsa-miR-190a-3p
EIF3D	hsa-miR-205-3p
EIF3D	hsa-miR-6315
EIF3D	hsa-miR-8485
EIF3D	hsa-miR-7010-5p
EIF3D	hsa-miR-2320-5p
EIF3D	hsa-miR-331-5p
EIF3D	hsa-miR-7014-5p
EIF3D	hsa-miR-7688-5p
EIF3D	hsa-miR-1839
EIF3D	hsa-miR-6786-5p
EIF3D	hsa-miR-766-3p
EIF3D	hsa-miR-6739-5p
EIF3D	hsa-miR-6511-5p
EIF3D	hsa-miR-708-5p
EIF3D	hsa-miR-3202
EIF3D	hsa-miR-302-5p
EIF3D	hsa-miR-5011-5p
EIF3D	hsa-let-7-5p
EIF3D	hsa-miR-192-5p
EIF3D	hsa-miR-6127
EIF3D	hsa-miR-6996-5p
EIF3D	hsa-miR-7027-5p
EIF3D	hsa-miR-452-3p
EIF3D	hsa-miR-484
EIF3D	hsa-miR-3919
EIF3D	hsa-miR-688
EIF3D	hsa-miR-4510
EIF3D	hsa-miR-3059-5p
EIF3D	hsa-miR-6733-5p
EIF3D	hsa-miR-7669-3p

EIF3D	hsa-miR-6119-5p
EIF3D	hsa-miR-670-3p
EIF3D	hsa-miR-3557-3p
EIF3D	hsa-miR-6133
EIF3D	hsa-miR-6129
EIF3D	hsa-miR-4692
EIF3D	hsa-miR-138-3p
EIF3D	hsa-miR-6834-5p
EIF3D	hsa-let-7
EIF3D	hsa-miR-766-3p
EIF3D	hsa-miR-7166-3p
EIF3D	hsa-miR-424-3p
EIF3D	hsa-miR-4756-3p
EIF3D	hsa-miR-3190-3p
EIF3D	hsa-miR-4533
EIF3D	hsa-miR-6130
EIF4A1	hsa-miR-7070-5p
EIF4A1	hsa-miR-1843b-5p
EIF4A1	hsa-miR-1545a-3p
EIF4A1	hsa-miR-5589-5p
EIF4A1	hsa-miR-6836-3p
EIF4A1	hsa-miR-2459
EIF4A1	hsa-miR-6927-5p
EIF4A1	hsa-miR-3109-3p
EIF4A1	hsa-miR-7673-3p
EIF4A1	hsa-miR-409
EIF4A1	hsa-miR-887-5p
EIF4A1	hsa-miR-4272
EIF4A1	hsa-miR-294-5p
EIF4A1	hsa-miR-3912-5p
EIF4A1	hsa-miR-34b-3p
EIF4A1	hsa-miR-664-5p
EIF4A1	hsa-miR-506-5p
EIF4A1	hsa-miR-6856-3p
EIF4A1	hsa-miR-7398u-3p
EIF4A1	hsa-miR-6845-3p
EIF4A1	hsa-miR-30c-1-3p
EIF4A1	hsa-miR-2406
EIF4A1	hsa-miR-7010-3p
EIF4A1	hsa-miR-2324
EIF4A1	hsa-miR-7196-5p
EIF4A1	hsa-miR-133-3p.1
EIF4A1	hsa-miR-330-5p
EIF4A1	hsa-miR-154-5p
EIF4A1	hsa-miR-6976-5p
EIF4A1	hsa-miR-3557-5p

EIF4A1	hsa-let-7a-3p
EIF4A1	hsa-miR-548az-5p
EIF4A1	hsa-miR-6779-5p
EIF4A1	hsa-miR-7041-3p
EIF4A1	hsa-miR-204-3p
EIF4A1	hsa-miR-3562
EIF4A1	hsa-miR-7000-5p
EIF4A1	hsa-miR-8804
EIF4A1	hsa-miR-566
EIF4A1	hsa-miR-516b-5p
EIF4A1	hsa-miR-4763-3p
EIF4A1	hsa-miR-154a
EIF4A1	hsa-miR-7864
EIF4A1	hsa-miR-7178-5p
EIF4A1	hsa-miR-7280-3p
EIF4A1	hsa-miR-7118-5p
EIF4A1	hsa-miR-204-5p
EIF4A1	hsa-miR-3473
EIF4A1	hsa-miR-8880
EIF4A1	hsa-miR-377-3p
EIF4A1	hsa-miR-33-3p
EIF4A1	hsa-miR-4283
EIF4A1	hsa-miR-4505
EIF4A1	hsa-miR-3122
EIF4A1	hsa-miR-145a-3p
EIF4A1	hsa-miR-4314
EIF4A1	hsa-miR-3564
EIF4A1	hsa-miR-7119-5p
EIF4A1	hsa-miR-449c-5p
EIF4A1	hsa-miR-8089
EIF4A1	hsa-miR-23-5p
EIF4A1	hsa-miR-129-5p
EIF4A1	hsa-miR-219a-5p
EIF4A1	hsa-miR-4287
EIF4A1	hsa-miR-7363-5p
EIF4A1	hsa-miR-3192-5p
EIF4A1	hsa-miR-6996-3p
EIF4A1	hsa-miR-7339-5p
EIF4A1	hsa-miR-2438
EIF4A1	hsa-miR-6888-3p
EIF4A1	hsa-miR-8085
EIF4A1	hsa-miR-599
EIF4A1	hsa-miR-599-3p
EIF4A1	hsa-miR-1663-3p
EIF4A1	hsa-miR-409b
EIF4A1	hsa-miR-326-5p

EIF4A1	hsa-miR-8794
EIF4A1	hsa-miR-6134
EIF4A1	hsa-miR-4514
EIF4A1	hsa-miR-4654
EIF4A1	hsa-miR-7364-5p
EIF4A1	hsa-miR-7069-5p
EIF4A1	hsa-miR-2284ac
EIF4A1	hsa-miR-688
EIF4A1	hsa-miR-295-5p
EIF4A1	hsa-miR-4652-5p
EIF4A1	hsa-miR-7665-5p
EIF4A1	hsa-miR-942-5p
EIF4A1	hsa-miR-4255
EIF4A1	hsa-miR-4430
EIF4A1	hsa-miR-2114-5p
EIF4A1	hsa-miR-133a-3p.2
EIF4A1	hsa-miR-3150b-3p
EIF4A1	hsa-miR-548c-3p
EIF4A1	hsa-miR-5197-3p
EIF4A1	hsa-miR-423-5p
EIF4A1	hsa-miR-6776-5p
EIF4A1	hsa-miR-6734-3p
EIF4A1	hsa-miR-4448
EIF4A1	hsa-miR-511-5p
EIF4A1	hsa-miR-6766-5p
EIF4A1	hsa-miR-493-3p
EIF4A1	hsa-miR-4756-3p
EIF4A1	hsa-miR-129b-5p
EIF4A1	hsa-miR-6868-3p
EIF4A1	hsa-miR-660-3p
EIF4A1	hsa-miR-130c-5p
EIF4A1	hsa-miR-1545c-3p
EIF4A1	hsa-miR-1805-5p
EIF4A1	hsa-miR-6398
EIF4A1	hsa-miR-2394
EIF4A1	hsa-miR-27-3p
EIF4A1	hsa-miR-2388-5p
EIF4A1	hsa-miR-6810-5p
EIF4A1	hsa-miR-6997-5p
EIF4A1	hsa-miR-8881
EIF4A1	hsa-miR-5187-3p
EIF4A1	hsa-miR-6745
EIF4A1	hsa-miR-7240-5p
EIF4A1	hsa-miR-345-3p
EIF4A1	hsa-miR-4659b-3p
EIF4A1	hsa-miR-4710

EIF4A1	hsa-miR-516a
EIF4A1	hsa-miR-142-3p
EIF4A1	hsa-miR-339-5p
EIF4A1	hsa-miR-624-3p
EIF4A1	hsa-miR-329a
EIF4A1	hsa-miR-428
EIF4A1	hsa-miR-597-3p
EIF4A1	hsa-miR-6506-5p
EIF4A1	hsa-miR-361-3p
EIF4A1	hsa-miR-3140-5p
EIF4A1	hsa-miR-3586-5p
EIF4A1	hsa-miR-8843
EIF4A1	hsa-miR-3591-5p
EIF4A1	hsa-miR-6873-3p
EIF4A1	hsa-miR-320-5p
EIF4A1	hsa-miR-8861
EIF4A1	hsa-miR-3473be
EIF4A1	hsa-miR-4774-3p
EIF4A1	hsa-miR-6877-3p
EIF4A1	hsa-miR-548c-3p
EIF4A1	hsa-miR-7388a-5p
EIF4A1	hsa-miR-181-5p
EIF4A1	hsa-miR-5001-5p
EIF4A1	hsa-miR-2489
EIF4A1	hsa-miR-7223-3p
EIF4A1	hsa-miR-6809-3p
EIF4A1	hsa-miR-2285c
EIF4A1	hsa-miR-5101
EIF4A1	hsa-miR-7264-3p
EIF4A1	hsa-miR-1233-3p
EIF4A1	hsa-miR-7298-3p
EIF4A1	hsa-miR-30c-3p
EIF4A1	hsa-miR-154b
EIF4A1	hsa-miR-335-5p
EIF4A1	hsa-miR-450b-3p
EIF4A1	hsa-miR-7239-5p
EIF4A1	hsa-miR-331-3p
EIF4A1	hsa-miR-343
EIF4A1	hsa-miR-2355-5p
EIF4A1	hsa-miR-183-5p
EIF4A1	hsa-miR-516b-3p
EIF4A1	hsa-miR-4778-3p
EIF4A1	hsa-miR-892b
EIF4A1	hsa-miR-6769a-3p
EIF4A1	hsa-miR-1562-5p
EIF4A1	hsa-miR-1191b-3p

EIF4A1	hsa-miR-7340-5p
EIF4A1	hsa-miR-3652
EIF4A1	hsa-miR-3577-5p
EIF4A1	hsa-miR-6780a-5p
EIF4A1	hsa-miR-1284
EIF4A1	hsa-miR-5010-3p
EIF4A1	hsa-miR-3077-3p
EIF4A1	hsa-miR-129-5p
EIF4A1	hsa-miR-380-5p
EIF4A1	hsa-miR-4731-5p
EIF4A1	hsa-miR-5189-5p
EIF4A1	hsa-miR-743-5p
EIF4A1	hsa-miR-138-1-3p
EIF4A1	hsa-miR-6860
EIF4A1	hsa-miR-1584-3p
EIF4A1	hsa-miR-872-5p
EIF4A1	hsa-miR-3470a
EIF4A1	hsa-miR-4690-3p
EIF4A1	hsa-miR-1200
EIF4A1	hsa-miR-2285x
EIF4A1	hsa-miR-5000-3p
EIF4A1	hsa-miR-196
EIF4A1	hsa-miR-7315-3p
EIF4A1	hsa-miR-219b-5p
EIF4A1	hsa-miR-129b-3p
EIF4A1	hsa-miR-2385-5p
EIF4A1	hsa-miR-2355-3p
EIF4A1	hsa-miR-4768-5p
EIF4A1	hsa-miR-450a-2-3p
EIF4A1	hsa-miR-6315
EIF4A1	hsa-miR-488-5p
EIF4A1	hsa-miR-5088-3p
EIF4A1	hsa-miR-6787-3p
EIF4A1	hsa-miR-7318-5p
EIF4A1	hsa-miR-4640-3p
EIF4A1	hsa-miR-3573-5p
EIF4A1	hsa-miR-2300-5p
EIF4A1	hsa-miR-548au-3p
EIF4A1	hsa-miR-138-5p
EIF4A1	hsa-miR-423-3p
EIF4A1	hsa-miR-6904-5p
EIF4A1	hsa-miR-4330
EIF4A1	hsa-miR-5186
EIF4A1	hsa-miR-670-3p
EIF4A1	hsa-miR-4469
EIF4A1	hsa-miR-3078-5p

EIF4A1	hsa-miR-377-3p
EIF4A1	hsa-miR-7322-5p
EIF4A1	hsa-miR-2113
EIF4A1	hsa-miR-6941-5p
EIF4A1	hsa-miR-7337-5p
EIF4A1	hsa-miR-7666-5p
EIF4A1	hsa-miR-939-3p
EIF4A1	hsa-miR-7309-3p
EIF4A1	hsa-miR-193b-3p
EIF4A1	hsa-miR-1544-3p
EIF4A1	hsa-miR-133b
EIF4A1	hsa-miR-6541
EIF4A1	hsa-miR-4802-5p
EIF4A1	hsa-miR-872
EIF4A1	hsa-miR-8084
EIF4A1	hsa-miR-95-5p
EIF4A1	hsa-miR-7064-3p
EIF4A1	hsa-miR-3975
EIF4A1	hsa-miR-6348
EIF4A1	hsa-miR-374b-3p
EIF4A1	hsa-miR-4688
EIF4A1	hsa-miR-642a-5p
EIF4A1	hsa-miR-3691-3p
EIF4A1	hsa-miR-6756-5p
EIF4A1	hsa-miR-7320-5p
EIF4A1	hsa-miR-4685-3p
EIF4A1	hsa-miR-3057-5p
EIF4A1	hsa-miR-7007-3p
EIF4A1	hsa-miR-5125-5p
EIF4A1	hsa-miR-7119-3p
EIF4A1	hsa-miR-516a-3p
EIF4A1	hsa-miR-3589
EIF4A1	hsa-miR-3613-3p
EIF4A1	hsa-miR-30-3p
EIF4A1	hsa-miR-4278
EIF4A1	hsa-miR-1286
EIF4A1	hsa-miR-4769-5p
EIF4A1	hsa-miR-4530
EIF4A1	hsa-miR-6833-3p
EIF4A1	hsa-miR-6975-3p
EIF4A1	hsa-miR-680
EIF4A1	hsa-miR-145-3p
EIF4A1	hsa-miR-3182
EIF4A1	hsa-miR-6939-3p
EIF4A1	hsa-miR-1248
EIF4A1	hsa-miR-3152-5p

EIF4A1	hsa-miR-7851-3p
EIF4A1	hsa-miR-185-3p
EIF4A1	hsa-miR-1258-3p
EIF4A1	hsa-miR-7045-3p
EIF4A1	hsa-miR-1273h-5p
EIF4A1	hsa-miR-183-3p
EIF4A1	hsa-miR-454-5p
EIF4A1	hsa-miR-186-3p
EIF4A1	hsa-miR-7113-3p
EIF4A1	hsa-miR-548ah-5p
EIF4A1	hsa-miR-5120
EIF4A1	hsa-miR-3679-3p
EIF4A1	hsa-miR-488-3p
EIF4A1	hsa-miR-7150
EIF4A1	hsa-miR-3541
EIF4A1	hsa-miR-642
EIF4A1	hsa-miR-6851-5p
EIF4A1	hsa-miR-769-3p
EIF4A1	hsa-miR-762
EIF4A1	hsa-miR-30c-2-3p
EIF4A1	hsa-miR-2115-5p
EIF4A1	hsa-miR-4446-5p
EIF4A1	hsa-miR-2361
EIF4A1	hsa-miR-661
EIF4A1	hsa-miR-23b-5p
EIF4A1	hsa-miR-7195-5p
EIF4A1	hsa-miR-1253
EIF4A1	hsa-miR-6842-3p
EIF4A1	hsa-miR-7344-5p
EIF4A1	hsa-miR-548g-3p
EIF4A1	hsa-miR-548t-5p
EIF4A1	hsa-miR-3473g
EIF4A1	hsa-miR-3063-5p
EIF4A1	hsa-miR-335-5p
EIF4A1	hsa-miR-7024-5p
EIF4A1	hsa-miR-4483
EIF4A1	hsa-miR-7269-5p
EIF4A1	hsa-miR-6523a
EIF4A1	hsa-miR-2114-3p
EIF4A1	hsa-miR-381-3p
EIF4A1	hsa-miR-28-5p
EIF4A1	hsa-miR-7038-5p
EIF4A1	hsa-miR-6810-3p
EIF4A1	hsa-miR-1897-5p
EIF4A1	hsa-miR-153-5p
EIF4A1	hsa-miR-1233-3p

EIF4A1	hsa-miR-340-3p
EIF4A1	hsa-miR-8908b
EIF4A1	hsa-miR-7678-3p
EIF4A1	hsa-miR-1275
EIF4A1	hsa-miR-2285d
EIF4A1	hsa-miR-891-3p
EIF4A1	hsa-miR-4292
EIF4A1	hsa-let-7-3p
EIF4A1	hsa-miR-7665-3p
EIF4A1	hsa-miR-7168-3p
EIF4A1	hsa-miR-3596d
EIF4A1	hsa-miR-15b-3p
EIF4A1	hsa-miR-892-5p
EIF4A1	hsa-miR-5787
EIF4A1	hsa-miR-6360
EIF4A1	hsa-miR-381-3p
EIF4A1	hsa-miR-2284aa
EIF4A1	hsa-miR-6740-3p
EIF4A1	hsa-miR-7352-3p
EIF4A1	hsa-miR-5118
EIF4A1	hsa-miR-7391-5p
EIF4A1	hsa-miR-627-3p
EIF4A1	hsa-miR-2893
EIF4A1	hsa-miR-371b-5p
EIF4A1	hsa-miR-361-5p
EIF4A1	hsa-miR-133a-3p.1
EIF4A1	hsa-miR-3689-3p
EIF4A1	hsa-miR-1237-3p
EIF4A1	hsa-miR-2422
EIF4A1	hsa-miR-6772-3p
EIF4A1	hsa-miR-7234-5p
EIF4A1	hsa-miR-4268
EIF4A1	hsa-miR-3670
EIF4A1	hsa-miR-612
EIF4A1	hsa-miR-2390
EIF4A1	hsa-miR-3191-3p
EIF4A1	hsa-miR-4419b
EIF4A1	hsa-miR-2183
EIF4A1	hsa-miR-371
EIF4A1	hsa-let-7f-1-3p
EIF4A1	hsa-miR-2428
EIF4A1	hsa-miR-1240
EIF4A1	hsa-miR-16-2-3p
EIF4A1	hsa-miR-6887-3p
EIF4A1	hsa-miR-539-3p
EIF4A1	hsa-miR-1843a-5p

EIF4A1	hsa-miR-691
EIF4A1	hsa-miR-7674-5p
EIF4A1	hsa-miR-3592
EIF4A1	hsa-miR-7312-3p
EIF4A1	hsa-miR-31-5p
EIF4A1	hsa-miR-7325-5p
EIF4A1	hsa-miR-4765
EIF4A1	hsa-miR-2368-3p
EIF4A1	hsa-miR-7023-5p
EIF4A1	hsa-miR-1892
EIF4A1	hsa-miR-6379
EIF4A1	hsa-miR-7249-3p
EIF4A1	hsa-miR-450b
EIF4A1	hsa-miR-7192-3p
EIF4A1	hsa-let-7abf-3p
EIF4A1	hsa-miR-3909
EIF4A1	hsa-miR-6916-3p
EIF4A1	hsa-miR-7197-3p
EIF4A1	hsa-miR-7201-5p
EIF4A1	hsa-miR-623
EIF4A1	hsa-miR-2466-3p
EIF4A1	hsa-miR-592
EIF4A1	hsa-miR-5702
EIF4A1	hsa-miR-1245b-3p
EIF4A1	hsa-miR-8077
EIF4A1	hsa-miR-6879-3p
EIF4A1	hsa-miR-8057
EIF4A1	hsa-miR-34b-5p
EIF4A1	hsa-miR-2909
EIF4A1	hsa-miR-8068
EIF4A1	hsa-miR-3613-5p
EIF4A1	hsa-miR-216c-3p
EIF4A1	hsa-miR-712-5p
EIF4A1	hsa-miR-1246
EIF4A1	hsa-miR-7861
EIF4A1	hsa-miR-1285-3p
EIF4A1	hsa-miR-516b
EIF4A1	hsa-miR-7283-5p
EIF4A1	hsa-miR-8870
EIF4A1	hsa-miR-4667-3p
EIF4A1	hsa-miR-6743-5p
EIF4A1	hsa-miR-655-5p
EIF4A1	hsa-miR-1250-3p
EIF4A1	hsa-miR-6773-3p
EIF4A1	hsa-miR-378-5p
EIF4A1	hsa-miR-4700-5p

EIF4A1	hsa-miR-188-5p
EIF4A1	hsa-miR-331-3p
EIF4A1	hsa-miR-4492
EIF4A1	hsa-miR-4646-5p
EIF4A1	hsa-miR-549-5p
EIF4A1	hsa-miR-486-3p
EIF4A1	hsa-miR-449-5p
EIF4A1	hsa-miR-2451
EIF4A1	hsa-miR-1782
EIF4A1	hsa-miR-1958
EIF4A1	hsa-miR-6801-3p
EIF4A1	hsa-miR-2311
EIF4A1	hsa-miR-708-5p
EIF4A1	hsa-miR-3473c
EIF4A1	hsa-miR-3064-5p
EIF4A1	hsa-miR-742-3p
EIF4A1	hsa-miR-6878-5p
EIF4A1	hsa-miR-212-3p
EIF4A1	hsa-miR-4694-3p
EIF4A1	hsa-miR-3083-5p
EIF4A1	hsa-miR-215-3p
EIF4A1	hsa-miR-7163-5p
EIF4A1	hsa-miR-2285e
EIF4A1	hsa-miR-1976
EIF4A1	hsa-miR-6512-5p
EIF4A1	hsa-miR-338-3p
EIF4A1	hsa-miR-5703
EIF4A1	hsa-miR-6788-5p
EIF4A1	hsa-miR-1894-5p
EIF4A1	hsa-miR-3655
EIF4A1	hsa-miR-590-3p
EIF4A1	hsa-miR-300
EIF4A1	hsa-miR-6961-3p
EIF4A1	hsa-miR-128-3p
EIF4A1	hsa-miR-7017-3p
EIF4A1	hsa-miR-638
EIF4A1	hsa-miR-193b-5p
EIF4A1	hsa-miR-133
EIF4A1	hsa-miR-7671-5p
EIF4A1	hsa-miR-7350-3p
EIF4A1	hsa-miR-6731-5p
EIF4A1	hsa-miR-4736
EIF4A1	hsa-miR-8865
EIF4A1	hsa-miR-187-5p
EIF4A1	hsa-miR-6807-5p
EIF4A1	hsa-miR-6983-3p

EIF4A1	hsa-miR-7332-3p
EIF4A1	hsa-miR-3943
EIF4A1	hsa-miR-3689d
EIF4A1	hsa-miR-1230
EIF4A1	hsa-miR-328
EIF4A1	hsa-miR-1252-3p
EIF4A1	hsa-miR-4713-3p
EIF4A1	hsa-miR-2284r
EIF4A1	hsa-miR-871-5p
EIF4A1	hsa-miR-6852-5p
EIF4A1	hsa-miR-4713-5p
EIF4A1	hsa-miR-6976-3p
EIF4A1	hsa-miR-6867-3p
EIF4A1	hsa-miR-7661-5p
EIF4A1	hsa-miR-616-5p
EIF4A1	hsa-miR-3144-5p
EIF4A1	hsa-miR-34
EIF4A1	hsa-miR-2328-5p
EIF4A1	hsa-miR-376a-5p
EIF4A1	hsa-miR-3062-3p
EIF4A1	hsa-miR-5107-5p
EIF4A1	hsa-miR-4692
EIF4A1	hsa-miR-6780b-3p
EIF4A1	hsa-miR-7679-5p
EIF4A1	hsa-miR-6980-5p
EIF4A1	hsa-miR-421-5p
EIF4A1	hsa-miR-6782-5p
EIF4A1	hsa-miR-6418-5p
EIF4A1	hsa-miR-7649-3p
EIF4A1	hsa-miR-7192-5p
EIF4A1	hsa-miR-1470
EIF4A1	hsa-miR-1238-3p
EIF4A1	hsa-miR-92a-2-5p
EIF4A1	hsa-miR-1547-3p
EIF4A1	hsa-miR-619-5p
EIF4A1	hsa-miR-6896-3p
EIF4A1	hsa-miR-373-3p
EIF4A1	hsa-miR-1225-3p
EIF4A1	hsa-miR-7081-3p
EIF4A1	hsa-miR-2114-5p
EIF4A1	hsa-miR-619
EIF4A1	hsa-miR-881-5p
EIF4A1	hsa-miR-1956-3p
EIF4A1	hsa-miR-4659a-3p
EIF4A1	hsa-miR-1237-3p
EIF4A1	hsa-miR-5194

EIF4A1	hsa-miR-1224
EIF4A1	hsa-miR-545-3p
EIF4A1	hsa-miR-330-3p
EIF4A1	hsa-miR-4434
EIF4A1	hsa-miR-516-3p
EIF4A1	hsa-miR-154-5p
EIF4A1	hsa-miR-1226-5p
EIF4A1	hsa-miR-294
EIF4A1	hsa-miR-6923-5p
EIF4A1	hsa-miR-669n
EIF4A1	hsa-miR-7051-5p
EIF4A1	hsa-miR-485-5p
EIF4A1	hsa-miR-2285g
EIF4A1	hsa-miR-2479
EIF4A1	hsa-miR-6791-5p
EIF4A1	hsa-miR-326
EIF4A1	hsa-miR-1207-5p
EIF4A1	hsa-miR-2453
EIF4A1	hsa-miR-607
EIF4A1	hsa-miR-1207
EIF4A1	hsa-miR-6824-5p
EIF4A1	hsa-miR-7222-3p
EIF4A1	hsa-miR-1293
EIF4A1	hsa-miR-5002-5p
EIF4A1	hsa-miR-6734-5p
EIF4A1	hsa-miR-4478
EIF4A1	hsa-miR-3074-5p
EIF4A1	hsa-miR-4516
EIF4A1	hsa-miR-4743-3p
EIF4A1	hsa-miR-7236-5p
EIF4A1	hsa-miR-3596a
EIF4A1	hsa-miR-6953-5p
EIF4A1	hsa-miR-2290
EIF4A1	hsa-miR-877-3p
EIF4A1	hsa-miR-7260-5p
EIF4A1	hsa-miR-98-3p
EIF4A1	hsa-miR-139-3p
EIF4A1	hsa-miR-125b-2-3p
EIF4A1	hsa-miR-7386d-3p
EIF4A1	hsa-miR-1903
EIF4A1	hsa-miR-592-5p
EIF4A1	hsa-miR-4666a-3p
EIF4A1	hsa-miR-6546-5p
EIF4A1	hsa-miR-7359-5p
EIF4A1	hsa-miR-3929
EIF4A1	hsa-miR-5685

EIF4A1	hsa-miR-1224-3p
EIF4A1	hsa-miR-7854-3p
EIF4A1	hsa-miR-629-3p
EIF4A1	hsa-miR-8906
EIF4A1	hsa-miR-505-5p
EIF4A1	hsa-miR-2464-5p
EIF4A1	hsa-miR-5584-5p
EIF4A1	hsa-miR-331-5p
EIF4A1	hsa-miR-30b-3p
EIF4A1	hsa-miR-133-3p
EIF4A1	hsa-miR-7106-3p
EIF4A1	hsa-miR-6881-3p
EIF4A1	hsa-miR-4489
EIF4A1	hsa-miR-6789-5p
EIF4A1	hsa-miR-6914-5p
EIF4A1	hsa-miR-4498
EIF4A1	hsa-miR-378a-5p
EIF4A1	hsa-miR-28-5p
EIF4A1	hsa-miR-4780
EIF4A1	hsa-miR-150-5p
EIF4A1	hsa-miR-7323-3p
EIF4A1	hsa-miR-545-3p
EIF4A1	hsa-miR-7207-5p
EIF4A1	hsa-miR-1236-5p
EIF4A1	hsa-miR-338-3p
EIF4A1	hsa-miR-7162-5p
EIF4A1	hsa-miR-3662
EIF4A1	hsa-miR-292b-5p
EIF4A1	hsa-miR-1207-3p
EIF4A1	hsa-miR-2284z
EIF4A1	hsa-miR-1839
EIF4A1	hsa-miR-7862
EIF4A1	hsa-miR-1291
EIF4A1	hsa-miR-6513-5p
EIF4A1	hsa-miR-516
EIF4A1	hsa-miR-7687-3p
EIF4A1	hsa-miR-7354-3p
EIF4A1	hsa-miR-7233-5p
EIF4A1	hsa-miR-5621-5p
EIF4A1	hsa-miR-625-3p
EIF4A1	hsa-miR-7152-5p
EIF4A1	hsa-miR-9500
EIF4A1	hsa-miR-4689
EIF4A1	hsa-miR-212-5p
EIF4A1	hsa-miR-4749-3p
EIF4A1	hsa-miR-3913-5p

EIF4A1	hsa-miR-3596c
EIF4A1	hsa-miR-6819-3p
EIF4A1	hsa-miR-188-5p
EIF4A1	hsa-miR-7162-3p
EIF4A1	hsa-miR-3192
EIF4A1	hsa-miR-5588-3p
EIF4A1	hsa-miR-7278-3p
EIF4A1	hsa-miR-6790-3p
EIF4A1	hsa-miR-5046
EIF4A1	hsa-miR-8876
EIF4A1	hsa-miR-3154
EIF4A1	hsa-miR-670-5p
EIF4A1	hsa-miR-2444
EIF4A1	hsa-miR-8848
EIF4A1	hsa-miR-3187-5p
EIF4A1	hsa-miR-6858-5p
EIF4A1	hsa-miR-4667
EIF4A1	hsa-miR-4729
EIF4A1	hsa-miR-2296
EIF4A1	hsa-miR-6834-5p
EIF4A1	hsa-miR-3674
EIF4A1	hsa-miR-450a-1-3p
EIF4A1	hsa-miR-8793
EIF4A1	hsa-miR-483-3p.1
EIF4A1	hsa-miR-3613a
EIF4A1	hsa-miR-3609
EIF4A1	hsa-miR-3188
EIF4A1	hsa-miR-34b-3p
EIF4A1	hsa-miR-2345
EIF4A1	hsa-miR-6954-5p
EIF4A1	hsa-miR-6765-3p
EIF4A1	hsa-miR-363-5p
EIF4A1	hsa-miR-7082-3p
EIF4A1	hsa-miR-2303
EIF4A1	hsa-miR-6926-3p
EIF4A1	hsa-miR-4495
EIF4A1	hsa-miR-6779-3p
EIF4A1	hsa-miR-2284s
EIF4A1	hsa-miR-6822-5p
EIF4A1	hsa-miR-3059-5p
EIF4A1	hsa-miR-4667-5p
EIF4A1	hsa-miR-370
EIF4A1	hsa-miR-2897
EIF4A1	hsa-miR-142-3p.2
EIF4A1	hsa-miR-8823
EIF4A1	hsa-miR-1225

EIF4A1	hsa-miR-7203-5p
EIF4A1	hsa-miR-1285-3p
EIF4A1	hsa-miR-5123
EIF4A1	hsa-miR-10b-5p
EIF4A1	hsa-miR-7160-5p
EIF4A1	hsa-miR-763
EIF4A1	hsa-miR-7177-5p
EIF4A1	hsa-miR-2467-5p
EIF4A1	hsa-miR-511
EIF4A1	hsa-miR-6070
EIF4A1	hsa-miR-373-5p
EIF4A1	hsa-miR-6750-5p
EIF4A1	hsa-miR-7073-5p
EIF4A1	hsa-miR-298-3p
EIF4A1	hsa-miR-3162-5p
EIF4A1	hsa-miR-5093
EIF4A1	hsa-miR-708-5p
EIF4A1	hsa-miR-466l-3p
EIF4A1	hsa-miR-7269-3p
EIF4A1	hsa-miR-370-3p
EIF4A1	hsa-miR-6720-3p
EIF4A1	hsa-miR-6854-5p
EIF4A1	hsa-let-7b-3p
EIF4E	hsa-miR-548ap-3p
EIF4E	hsa-miR-2292
EIF4E	hsa-miR-1912-3p
EIF4E	hsa-miR-4740-3p
EIF4E	hsa-miR-7254-3p
EIF4E	hsa-miR-148
EIF4E	hsa-miR-25-3p
EIF4E	hsa-miR-297
EIF4E	hsa-miR-543-3p
EIF4E	hsa-miR-465a-5p
EIF4E	hsa-miR-4752
EIF4E	hsa-miR-1649-5p
EIF4E	hsa-miR-302
EIF4E	hsa-miR-4433a-3p
EIF4E	hsa-let-7c-3p
EIF4E	hsa-miR-2285l
EIF4E	hsa-miR-204-3p
EIF4E	hsa-miR-1587
EIF4E	hsa-miR-6779-5p
EIF4E	hsa-miR-4735-5p
EIF4E	hsa-miR-1954
EIF4E	hsa-miR-3150b-3p
EIF4E	hsa-miR-5095

EIF4E	hsa-miR-8054
EIF4E	hsa-miR-6073
EIF4E	hsa-miR-384-3p
EIF4E	hsa-miR-490-3p
EIF4E	hsa-miR-4316
EIF4E	hsa-miR-7194-5p
EIF4E	hsa-miR-132-3p
EIF4E	hsa-miR-409a
EIF4E	hsa-miR-7338-5p
EIF4E	hsa-miR-548ah-3p
EIF4E	hsa-miR-3177-5p
EIF4E	hsa-miR-195-3p
EIF4E	hsa-miR-4291
EIF4E	hsa-miR-412
EIF4E	hsa-miR-3140-3p
EIF4E	hsa-miR-660-3p
EIF4E	hsa-miR-6573-5p
EIF4E	hsa-miR-4691-3p
EIF4E	hsa-miR-942-5p
EIF4E	hsa-miR-6314
EIF4E	hsa-miR-7006-5p
EIF4E	hsa-miR-3530-3p
EIF4E	hsa-miR-5684
EIF4E	hsa-miR-7857
EIF4E	hsa-miR-607
EIF4E	hsa-miR-4509
EIF4E	hsa-miR-141-5p
EIF4E	hsa-miR-103b
EIF4E	hsa-miR-532-3p
EIF4E	hsa-miR-548ar-5p
EIF4E	hsa-miR-6384
EIF4E	hsa-miR-1960
EIF4E	hsa-miR-605
EIF4E	hsa-miR-6757-5p
EIF4E	hsa-miR-6900-3p
EIF4E	hsa-miR-553
EIF4E	hsa-miR-548g-5p
EIF4E	hsa-miR-7273-5p
EIF4E	hsa-miR-224-3p
EIF4E	hsa-miR-7119-5p
EIF4E	hsa-miR-409
EIF4E	hsa-miR-6740-5p
EIF4E	hsa-miR-1814a
EIF4E	hsa-miR-3122
EIF4E	hsa-miR-525-5p
EIF4E	hsa-miR-548t-3p

EIF4E	hsa-miR-466b-5p
EIF4E	hsa-miR-8844
EIF4E	hsa-miR-455-5p
EIF4E	hsa-miR-548b-5p
EIF4E	hsa-miR-499a-3p
EIF4E	hsa-miR-6508-3p
EIF4E	hsa-miR-3907
EIF4E	hsa-miR-22-5p
EIF4E	hsa-miR-548c-3p
EIF4E	hsa-miR-410
EIF4E	hsa-miR-502-5p
EIF4E	hsa-miR-4482-3p
EIF4E	hsa-miR-30c-1-3p
EIF4E	hsa-miR-6808-5p
EIF4E	hsa-miR-2461-3p
EIF4E	hsa-miR-483-3p
EIF4E	hsa-miR-6861-5p
EIF4E	hsa-miR-6398
EIF4E	hsa-miR-1179
EIF4E	hsa-miR-4650-5p
EIF4E	hsa-miR-4474-5p
EIF4E	hsa-miR-6757-3p
EIF4E	hsa-miR-664
EIF4E	hsa-miR-7027-5p
EIF4E	hsa-miR-7069-5p
EIF4E	hsa-miR-4743-3p
EIF4E	hsa-miR-149-5p
EIF4E	hsa-miR-548az-5p
EIF4E	hsa-miR-30b-3p
EIF4E	hsa-miR-6504-3p
EIF4E	hsa-miR-7296-5p
EIF4E	hsa-miR-125-5p
EIF4E	hsa-miR-552-3p
EIF4E	hsa-miR-4790-3p
EIF4E	hsa-miR-4637
EIF4E	hsa-miR-8085
EIF4E	hsa-miR-8089
EIF4E	hsa-miR-7673-3p
EIF4E	hsa-miR-3591-5p
EIF4E	hsa-miR-2482
EIF4E	hsa-let-7c-1-3p
EIF4E	hsa-miR-4767
EIF4E	hsa-miR-6792-3p
EIF4E	hsa-miR-4524b-3p
EIF4E	hsa-miR-377-3p
EIF4E	hsa-miR-1288-3p

EIF4E	hsa-miR-3120-3p
EIF4E	hsa-miR-5683
EIF4E	hsa-miR-8060
EIF4E	hsa-miR-3925-5p
EIF4E	hsa-miR-6090
EIF4E	hsa-miR-6951-3p
EIF4E	hsa-miR-467a-5p
EIF4E	hsa-miR-651
EIF4E	hsa-miR-520-3p
EIF4E	hsa-miR-6830-3p
EIF4E	hsa-miR-4454
EIF4E	hsa-miR-1266
EIF4E	hsa-miR-7116-3p
EIF4E	hsa-miR-7337-5p
EIF4E	hsa-miR-1178-3p
EIF4E	hsa-miR-92
EIF4E	hsa-miR-345-3p
EIF4E	hsa-miR-1304-5p
EIF4E	hsa-miR-6991-5p
EIF4E	hsa-miR-344h-3p
EIF4E	hsa-miR-2376
EIF4E	hsa-miR-8860
EIF4E	hsa-miR-1838
EIF4E	hsa-miR-4672
EIF4E	hsa-miR-193
EIF4E	hsa-miR-8069
EIF4E	hsa-miR-548z
EIF4E	hsa-miR-5696
EIF4E	hsa-miR-6893-5p
EIF4E	hsa-miR-2409
EIF4E	hsa-miR-4284
EIF4E	hsa-miR-25-5p
EIF4E	hsa-miR-103-2-5p
EIF4E	hsa-miR-29a-2-5p
EIF4E	hsa-miR-2371
EIF4E	hsa-miR-6380
EIF4E	hsa-miR-362-5p
EIF4E	hsa-miR-5697-3p
EIF4E	hsa-miR-302c-5p
EIF4E	hsa-miR-122
EIF4E	hsa-miR-370-5p
EIF4E	hsa-miR-7864
EIF4E	hsa-miR-548ah-5p
EIF4E	hsa-miR-4437
EIF4E	hsa-miR-3671
EIF4E	hsa-miR-152-5p

EIF4E	hsa-miR-2285x
EIF4E	hsa-miR-520g-3p
EIF4E	hsa-miR-378d
EIF4E	hsa-miR-1970
EIF4E	hsa-miR-1228-3p
EIF4E	hsa-miR-2284ac
EIF4E	hsa-miR-624-3p
EIF4E	hsa-miR-5692bc
EIF4E	hsa-miR-548aa
EIF4E	hsa-miR-7161-5p
EIF4E	hsa-miR-133c
EIF4E	hsa-miR-449-3p
EIF4E	hsa-miR-3158-5p
EIF4E	hsa-miR-4314
EIF4E	hsa-miR-7153-3p
EIF4E	hsa-miR-8086
EIF4E	hsa-miR-467f
EIF4E	hsa-miR-103
EIF4E	hsa-miR-6801-3p
EIF4E	hsa-miR-4999-5p
EIF4E	hsa-miR-7369-5p
EIF4E	hsa-miR-6338
EIF4E	hsa-miR-6788-5p
EIF4E	hsa-miR-3910
EIF4E	hsa-miR-6879-5p
EIF4E	hsa-miR-691
EIF4E	hsa-miR-95-5p
EIF4E	hsa-miR-183-5p
EIF4E	hsa-miR-4747-5p
EIF4E	hsa-miR-30
EIF4E	hsa-miR-3192-5p
EIF4E	hsa-miR-1268b
EIF4E	hsa-miR-6896-5p
EIF4E	hsa-miR-218-3p
EIF4E	hsa-miR-30-3p
EIF4E	hsa-miR-1271-3p
EIF4E	hsa-miR-4280
EIF4E	hsa-miR-291
EIF4E	hsa-miR-6777-3p
EIF4E	hsa-miR-3622a-3p
EIF4E	hsa-miR-7156-3p
EIF4E	hsa-miR-3121-5p
EIF4E	hsa-miR-8843
EIF4E	hsa-miR-93-5p
EIF4E	hsa-miR-802
EIF4E	hsa-miR-19a-3p

EIF4E	hsa-miR-4796-5p
EIF4E	hsa-miR-381-3p
EIF4E	hsa-miR-545-3p
EIF4E	hsa-miR-6499-3p
EIF4E	hsa-miR-627-3p
EIF4E	hsa-miR-4460
EIF4E	hsa-miR-2114-5p
EIF4E	hsa-miR-211-5p
EIF4E	hsa-miR-2438
EIF4E	hsa-miR-8082
EIF4E	hsa-miR-577
EIF4E	hsa-miR-6679-5p
EIF4E	hsa-miR-4446-5p
EIF4E	hsa-miR-5681a
EIF4E	hsa-miR-3692-5p
EIF4E	hsa-miR-4650
EIF4E	hsa-miR-7206-3p
EIF4E	hsa-miR-548s
EIF4E	hsa-miR-7844-5p
EIF4E	hsa-miR-18-5p
EIF4E	hsa-miR-548h-5p
EIF4E	hsa-miR-1227-3p
EIF4E	hsa-miR-448-5p
EIF4E	hsa-miR-7680-3p
EIF4E	hsa-miR-3613-3p
EIF4E	hsa-miR-3614-3p
EIF4E	hsa-miR-6937-3p
EIF4E	hsa-miR-1760
EIF4E	hsa-miR-342-3p
EIF4E	hsa-miR-5701
EIF4E	hsa-miR-1197
EIF4E	hsa-miR-6131
EIF4E	hsa-miR-191-5p
EIF4E	hsa-miR-323a-5p
EIF4E	hsa-miR-212-3p
EIF4E	hsa-miR-5190
EIF4E	hsa-miR-466b-4-3p
EIF4E	hsa-miR-873-5p
EIF4E	hsa-miR-4684-3p
EIF4E	hsa-miR-4698
EIF4E	hsa-miR-8861
EIF4E	hsa-miR-7118-5p
EIF4E	hsa-miR-879-5p
EIF4E	hsa-miR-6797-5p
EIF4E	hsa-miR-548aq-3p
EIF4E	hsa-miR-7193-5p

EIF4E	hsa-miR-548x-3p
EIF4E	hsa-miR-5094
EIF4E	hsa-miR-494
EIF4E	hsa-miR-380-3p
EIF4E	hsa-miR-449-5p
EIF4E	hsa-miR-6319
EIF4E	hsa-miR-2285ad
EIF4E	hsa-miR-3974
EIF4E	hsa-miR-664-3p
EIF4E	hsa-miR-210-5p
EIF4E	hsa-miR-548m
EIF4E	hsa-miR-1933-5p
EIF4E	hsa-miR-2285g
EIF4E	hsa-miR-330-3p.2
EIF4E	hsa-miR-7328-3p
EIF4E	hsa-miR-1911-3p
EIF4E	hsa-miR-219c-3p
EIF4E	hsa-miR-7216-3p
EIF4E	hsa-miR-2285e
EIF4E	hsa-miR-145a-3p
EIF4E	hsa-miR-4455
EIF4E	hsa-miR-6781-5p
EIF4E	hsa-miR-147-5p
EIF4E	hsa-miR-1286
EIF4E	hsa-miR-549a
EIF4E	hsa-miR-613
EIF4E	hsa-miR-4483
EIF4E	hsa-miR-197-3p
EIF4E	hsa-miR-1233-5p
EIF4E	hsa-miR-7211-5p
EIF4E	hsa-miR-527
EIF4E	hsa-miR-320a
EIF4E	hsa-miR-7655-3p
EIF4E	hsa-miR-1240
EIF4E	hsa-miR-3174
EIF4E	hsa-miR-7207-5p
EIF4E	hsa-miR-2440
EIF4E	hsa-miR-3117-5p
EIF4E	hsa-miR-7168-5p
EIF4E	hsa-miR-7183-5p
EIF4E	hsa-miR-1284
EIF4E	hsa-miR-376a-5p
EIF4E	hsa-miR-7386o-3p
EIF4E	hsa-miR-198
EIF4E	hsa-miR-2471-3p
EIF4E	hsa-miR-129b-5p

EIF4E	hsa-miR-3976
EIF4E	hsa-miR-548j-5p
EIF4E	hsa-miR-6748-5p
EIF4E	hsa-miR-4694-5p
EIF4E	hsa-miR-3689a-5p
EIF4E	hsa-miR-2285aa
EIF4E	hsa-miR-496.1
EIF4E	hsa-miR-142-3p.1
EIF4E	hsa-miR-1303
EIF4E	hsa-miR-2285j
EIF4E	hsa-miR-6714-3p
EIF4E	hsa-miR-3152-5p
EIF4E	hsa-miR-6639-5p
EIF4E	hsa-miR-4261
EIF4E	hsa-miR-6523a
EIF4E	hsa-miR-660-5p
EIF4E	hsa-miR-7651-5p
EIF4E	hsa-miR-743-5p
EIF4E	hsa-miR-609
EIF4E	hsa-miR-1720-3p
EIF4E	hsa-miR-6778-5p
EIF4E	hsa-miR-4427
EIF4E	hsa-miR-2355-5p
EIF4E	hsa-miR-3524-3p
EIF4E	hsa-miR-290b-5p
EIF4E	hsa-miR-695
EIF4E	hsa-miR-330-3p
EIF4E	hsa-miR-6664-3p
EIF4E	hsa-miR-2957
EIF4E	hsa-miR-5128
EIF4E	hsa-miR-6888-3p
EIF4E	hsa-miR-1178-3p
EIF4E	hsa-miR-5615-5p
EIF4E	hsa-miR-7089-3p
EIF4E	hsa-miR-7117-5p
EIF4E	hsa-miR-7854-3p
EIF4E	hsa-miR-4309
EIF4E	hsa-miR-7398a-3p
EIF4E	hsa-miR-7266-3p
EIF4E	hsa-miR-7307-3p
EIF4E	hsa-miR-548c-3p
EIF4E	hsa-miR-570-3p
EIF4E	hsa-miR-6505-3p
EIF4E	hsa-miR-4999-3p
EIF4E	hsa-miR-7292-3p
EIF4E	hsa-miR-2483-3p

EIF4E	hsa-miR-593-3p
EIF4E	hsa-miR-4762-3p
EIF4E	hsa-miR-2452
EIF4E	hsa-miR-6742-3p
EIF4E	hsa-miR-550a-3p
EIF4E	hsa-miR-1225-3p
EIF4E	hsa-miR-548ap-5p
EIF4E	hsa-miR-3078-5p
EIF4E	hsa-miR-5590-5p
EIF4E	hsa-miR-548av-5p
EIF4E	hsa-miR-503-3p
EIF4E	hsa-miR-106a-3p
EIF4E	hsa-miR-92a-5p
EIF4E	hsa-miR-374a-3p
EIF4E	hsa-miR-4450
EIF4E	hsa-miR-619-5p
EIF4E	hsa-miR-701-5p
EIF4E	hsa-miR-3120-3p
EIF4E	hsa-miR-548x-5p
EIF4E	hsa-miR-7234-3p
EIF4E	hsa-miR-15c-3p
EIF4E	hsa-miR-599
EIF4E	hsa-miR-1-5p
EIF4E	hsa-miR-7184-5p
EIF4E	hsa-miR-1266-5p
EIF4E	hsa-miR-7664-3p
EIF4E	hsa-miR-7287-5p
EIF4E	hsa-miR-135a-1-3p
EIF4E	hsa-miR-4480
EIF4E	hsa-miR-567
EIF4E	hsa-miR-363-3p
EIF4E	hsa-miR-5187-5p
EIF4E	hsa-miR-467-3p
EIF4E	hsa-miR-101-3p.2
EIF4E	hsa-miR-942
EIF4E	hsa-miR-5000-3p
EIF4E	hsa-miR-6753-5p
EIF4E	hsa-miR-489-5p
EIF4E	hsa-miR-3596
EIF4E	hsa-miR-4492
EIF4E	hsa-miR-2428
EIF4E	hsa-miR-7371a-5p
EIF4E	hsa-miR-5693
EIF4E	hsa-miR-7179-3p
EIF4E	hsa-miR-3681-5p
EIF4E	hsa-miR-548av-3p

EIF4E	hsa-miR-4520-3p
EIF4E	hsa-miR-770
EIF4E	hsa-miR-450b
EIF4E	hsa-miR-677
EIF4E	hsa-miR-4731-5p
EIF4E	hsa-miR-6619-5p
EIF4E	hsa-miR-6840-3p
EIF4E	hsa-miR-7119-3p
EIF4E	hsa-miR-6827-3p
EIF4E	hsa-miR-450b-5p
EIF4E	hsa-miR-16-5p
EIF4E	hsa-miR-5571-5p
EIF4E	hsa-miR-7211-3p
EIF4E	hsa-miR-4742-3p
EIF4E	hsa-miR-3591-3p
EIF4E	hsa-miR-1257
EIF4E	hsa-miR-6722-5p
EIF4E	hsa-miR-3675-3p
EIF4E	hsa-miR-201-5p
EIF4E	hsa-miR-7371j-5p
EIF4E	hsa-let-7a-3p
EIF4E	hsa-miR-2324
EIF4E	hsa-miR-6900-5p
EIF4E	hsa-let-7a-2-3p
EIF4E	hsa-miR-548ak
EIF4E	hsa-miR-32-5p
EIF4E	hsa-miR-3074-5p
EIF4E	hsa-miR-16-5p
EIF4E	hsa-miR-3688-5p
EIF4E	hsa-miR-548v
EIF4E	hsa-miR-6529-3p
EIF4E	hsa-miR-670-5p
EIF4E	hsa-miR-1207-5p
EIF4E	hsa-miR-99-3p
EIF4E	hsa-miR-4532
EIF4E	hsa-miR-4799-5p
EIF4E	hsa-miR-410-3p
EIF4E	hsa-miR-5624-5p
EIF4E	hsa-miR-3936
EIF4E	hsa-miR-593-5p
EIF4E	hsa-miR-350
EIF4E	hsa-miR-128-5p
EIF4E	hsa-miR-6735-5p
EIF4E	hsa-miR-2053
EIF4E	hsa-miR-15a-3p
EIF4E	hsa-miR-7053-3p

EIF4E	hsa-miR-7169-3p
EIF4E	hsa-miR-5692a
EIF4E	hsa-miR-1976
EIF4E	hsa-miR-1255a
EIF4E	hsa-miR-376b-5p
EIF4E	hsa-miR-6718-5p
EIF4E	hsa-miR-7323-5p
EIF4E	hsa-miR-4528
EIF4E	hsa-miR-3654
EIF4E	hsa-miR-646
EIF4E	hsa-miR-6744-3p
EIF4E	hsa-miR-23
EIF4E	hsa-miR-143-3p
EIF4E	hsa-miR-6832-5p
EIF4E	hsa-miR-351-3p
EIF4E	hsa-miR-666-3p
EIF4E	hsa-miR-548e-5p
EIF4E	hsa-miR-556-3p
EIF4E	hsa-miR-1972
EIF4E	hsa-miR-217
EIF4E	hsa-miR-3573-3p
EIF4E	hsa-miR-450a-3p
EIF4E	hsa-miR-924
EIF4E	hsa-miR-6481
EIF4E	hsa-miR-548h-3p
EIF4E	hsa-miR-466b-2-3p
EIF4E	hsa-miR-3658
EIF4E	hsa-miR-743b-3p
EIF4E	hsa-miR-6881-3p
EIF4E	hsa-miR-555
EIF4E	hsa-miR-4715-3p
EIF4E	hsa-miR-7204-3p
EIF4E	hsa-miR-6884-3p
EIF4E	hsa-miR-8075
EIF4E	hsa-miR-4526
EIF4E	hsa-miR-501-3p
EIF4E	hsa-miR-5003-5p
EIF4E	hsa-miR-24-3p
EIF4E	hsa-miR-3059-3p
EIF4E	hsa-miR-5699-3p
EIF4E	hsa-miR-7649-5p
EIF4E	hsa-miR-5589-3p
EIF4E	hsa-miR-2475
EIF4E	hsa-miR-379-5p
EIF4E	hsa-miR-3653-3p
EIF4E	hsa-miR-593-3p

EIF4E	hsa-miR-21-3p
EIF4E	hsa-miR-874-5p
EIF4E	hsa-miR-455-3p.2
EIF4E	hsa-miR-5002-3p
EIF4E	hsa-miR-6739-3p
EIF4E	hsa-miR-449c-3p
EIF4E	hsa-miR-878
EIF4E	hsa-miR-495-5p
EIF4E	hsa-miR-3591
EIF4E	hsa-miR-1962
EIF4E	hsa-miR-7062-3p
EIF4E	hsa-miR-4478
EIF4E	hsa-miR-6973a-3p
EIF4E	hsa-miR-7037-3p
EIF4E	hsa-miR-2351
EIF4E	hsa-miR-138-2-3p
EIF4E	hsa-miR-512
EIF4E	hsa-miR-2310
EIF4E	hsa-miR-6906-3p
EIF4E	hsa-miR-770-5p
EIF4E	hsa-miR-508a
EIF4E	hsa-miR-7349-3p
EIF4E	hsa-miR-2410
EIF4E	hsa-miR-23a-5p
EIF4E	hsa-miR-8550
EIF4E	hsa-miR-3616-5p
EIF4E	hsa-miR-371a-5p
EIF4E	hsa-miR-376ab
EIF4E	hsa-miR-1243-3p
EIF4E	hsa-miR-424-5p
EIF4E	hsa-miR-1238-3p
EIF4E	hsa-miR-7198-5p
EIF4E	hsa-miR-30a-3p
EIF4E	hsa-miR-873-3p
EIF4E	hsa-miR-1185-5p
EIF4E	hsa-miR-29a-5p
EIF4E	hsa-miR-345-5p
EIF4E	hsa-miR-4292
EIF4E	hsa-miR-513a-5p
EIF4E	hsa-miR-2297
EIF4E	hsa-miR-487-5p
EIF4E	hsa-miR-3617-5p
EIF4E	hsa-miR-891b
EIF4E	hsa-miR-1537-5p
EIF4E	hsa-miR-5703
EIF4E	hsa-miR-433-5p

EIF4E	hsa-miR-3920
EIF4E	hsa-miR-3470b
EIF4E	hsa-miR-6119-3p
EIF4E	hsa-miR-548l
EIF4E	hsa-miR-6736-5p
EIF4E	hsa-miR-2313-5p
EIF4E	hsa-miR-7150
EIF4E	hsa-miR-2370-3p
EIF4E	hsa-miR-5697
EIF4E	hsa-miR-3622a-5p
EIF4E	hsa-miR-2284z
EIF4E	hsa-miR-218-5p
EIF4E	hsa-miR-1273f
EIF4E	hsa-miR-382-3p
EIF4E	hsa-miR-7260-3p
EIF4E	hsa-miR-4670-3p
EIF4E	hsa-miR-208-3p
EIF4E	hsa-miR-8838
EIF4E	hsa-miR-3559-3p
EIF4E	hsa-miR-6509-5p
EIF4E	hsa-miR-669bf-3p
EIF4E	hsa-miR-200c-3p
EIF4E	hsa-miR-429
EIF4E	hsa-miR-103-5p
EIF4E	hsa-miR-1299
EIF4E	hsa-miR-412-3p
EIF4E	hsa-miR-8796
EIF4E	hsa-miR-300-3p
EIF4E	hsa-miR-6823-5p
EIF4E	hsa-miR-134-5p
EIF4E	hsa-miR-145-3p
EIF4E	hsa-miR-130-3p
EIF4E	hsa-miR-502-5p
EIF4E	hsa-miR-2285h
EIF4E	hsa-miR-186-5p
EIF4E	hsa-miR-7053-5p
EIF4E	hsa-miR-550b-2-5p
EIF4E	hsa-miR-377-3p
EIF4E	hsa-miR-2902
EIF4E	hsa-miR-876-3p
EIF4E	hsa-miR-196-2-3p
EIF4E	hsa-miR-154c
EIF4E	hsa-miR-331-3p
EIF4E	hsa-miR-2477
EIF4E	hsa-miR-451b
EIF4E	hsa-miR-6509-3p

EIF4E	hsa-miR-625-5p
EIF4E	hsa-miR-889
EIF4E	hsa-miR-1758
EIF4E	hsa-miR-651-5p
EIF4E	hsa-miR-943
EIF4E	hsa-miR-217-5p
EIF4E	hsa-miR-6936-5p
EIF4E	hsa-miR-6500-5p
EIF4E	hsa-miR-124-3p
EIF4E	hsa-miR-301
EIF4E	hsa-miR-579
EIF4E	hsa-miR-6799-5p
EIF4E	hsa-miR-3190-3p
EIF4E	hsa-miR-6864-3p
EIF4E	hsa-miR-7371d-5p
EIF4E	hsa-miR-148b-5p
EIF4E	hsa-miR-1803
EIF4E	hsa-miR-4728-5p
EIF4E	hsa-miR-6815-5p
EIF4E	hsa-miR-6885-3p
EIF4E	hsa-miR-7281-5p
EIF4E	hsa-miR-513a-3p
EIF4E	hsa-miR-10b-3p
EIF4E	hsa-miR-3663-5p
EIF4E	hsa-miR-6732-3p
EIF4E	hsa-miR-33-5p
EIF4E	hsa-miR-1930-3p
EIF4E	hsa-miR-2417
EIF4E	hsa-miR-488-3p
EIF4E	hsa-miR-2285n
EIF4E	hsa-miR-7398r-3p
EIF4E	hsa-miR-4419b
EIF4E	hsa-miR-4459
EIF4E	hsa-miR-6849-3p
EIF4E	hsa-miR-3189-3p
EIF4E	hsa-miR-7241-3p
EIF4E	hsa-miR-6842-3p
EIF4E	hsa-miR-3163
EIF4E	hsa-miR-606
EIF4E	hsa-miR-6762-3p
EIF4E	hsa-miR-1258
EIF4E	hsa-miR-1712-3p
EIF4E	hsa-miR-539-3p
EIF4E	hsa-miR-3664-3p
EIF4E	hsa-miR-4666a-3p
EIF4E	hsa-miR-152-3p

EIF4E	hsa-miR-372-5p
EIF4E	hsa-miR-2444
EIF4E	hsa-miR-4663
EIF4E	hsa-miR-7258-5p
EIF4E	hsa-miR-224-5p
EIF4E	hsa-miR-2426
EIF4E	hsa-miR-4753-3p
EIF4E	hsa-miR-7059-3p
EIF4E	hsa-miR-7174-5p
EIF4E	hsa-miR-1285-5p
EIF4E	hsa-miR-544b
EIF4E	hsa-miR-216b-5p
EIF4E	hsa-miR-2479
EIF4E	hsa-miR-1-3p
EIF4E	hsa-miR-6773-3p
EIF4E	hsa-miR-465c-5p
EIF4E	hsa-miR-3202
EIF4E	hsa-miR-187-5p
EIF4E	hsa-miR-490-5p
EIF4E	hsa-miR-3064-3p
EIF4E	hsa-miR-30c-3p
EIF4E	hsa-miR-142-3p
EIF4E	hsa-miR-2284g
EIF4E	hsa-miR-200ac-5p
EIF4E	hsa-miR-7024-5p
EIF4E	hsa-miR-135-3p
EIF4E	hsa-miR-2332
EIF4E	hsa-miR-1276
EIF4E	hsa-miR-6890-3p
EIF4E	hsa-miR-342-3p
EIF4E	hsa-miR-6747-3p
EIF4E	hsa-miR-6417
EIF4E	hsa-miR-4716-5p
EIF4E	hsa-miR-7976
EIF4E	hsa-miR-3121-3p
EIF4E	hsa-miR-101a-5p
EIF4E	hsa-miR-141-3p
EIF4E	hsa-miR-124
EIF4E	hsa-miR-297bc-3p
EIF4E	hsa-miR-3690
EIF4E	hsa-miR-3058-5p
EIF4E	hsa-miR-6852-5p
EIF4E	hsa-miR-29-5p
EIF4E	hsa-miR-199
EIF4E	hsa-miR-2284y
EIF4E	hsa-miR-5689

EIF4E	hsa-miR-6683-3p
EIF4E	hsa-miR-1261
EIF4E	hsa-miR-329-3p
EIF4E	hsa-miR-7856-5p
EIF4E	hsa-miR-6553-3p
EIF4E	hsa-miR-182-5p
EIF4E	hsa-miR-1252-3p
EIF4E	hsa-miR-6754-5p
EIF4E	hsa-miR-291b-3p
EIF4E	hsa-miR-669p-3p
EIF4E	hsa-miR-3657
EIF4E	hsa-miR-6594-5p
EIF4E	hsa-miR-6802-5p
EIF4E	hsa-miR-618
EIF4E	hsa-miR-669o-5p
EIF4E	hsa-miR-2314
EIF4E	hsa-miR-526
EIF4E	hsa-miR-4425
EIF4E	hsa-miR-548an
EIF4E	hsa-miR-571
EIF4E	hsa-miR-548au-5p
EIF4E	hsa-miR-5003-3p
EIF4E	hsa-miR-7387-3p
EIF4E	hsa-miR-590-5p
EIF4E	hsa-miR-1253
EIF4E	hsa-miR-2359
EIF4E	hsa-miR-548aw
EIF4E	hsa-miR-7468-3p
EIF4E	hsa-miR-3544-3p
EIF4E	hsa-miR-2681-3p
EIF4E	hsa-miR-96-3p
EIF4E	hsa-miR-6903-3p
EIF4E	hsa-miR-7044-3p
EIF4E	hsa-miR-4725-3p
EIF4E	hsa-miR-507
EIF4E	hsa-miR-3663-3p
EIF4E	hsa-miR-3176
EIF4E	hsa-miR-1293
EIF4E	hsa-miR-4667-5p
EIF4E	hsa-miR-466n-3p
EIF4E	hsa-miR-2439-3p
EIF4E	hsa-miR-2414
EIF4E	hsa-miR-1305
EIF4E	hsa-miR-664a-3p
EIF4E	hsa-miR-4744
EIF4E	hsa-miR-4305

EIF4E	hsa-miR-7388c-3p
EIF4E	hsa-miR-1277-5p
EIF4E	hsa-miR-589-5p
EIF4E	hsa-miR-4420
EIF4E	hsa-miR-6326
EIF4E	hsa-miR-6526
EIF4E	hsa-miR-29b-2-5p
EIF4E	hsa-miR-7192-3p
EIF4E	hsa-miR-5189-3p
EIF4E	hsa-miR-500a-3p
EIF4E	hsa-miR-29-3p
EIF4E	hsa-miR-3646
EIF4E	hsa-miR-155-5p
EIF4E	hsa-miR-3548
EIF4E	hsa-miR-200c-3p
EIF4E	hsa-miR-874
EIF4E	hsa-miR-7190-5p
EIF4E	hsa-miR-767-5p
EIF4E	hsa-miR-579-3p
EIF4E	hsa-miR-582-5p
EIF4E	hsa-miR-6128
EIF4E	hsa-miR-4797-5p
EIF4E	hsa-miR-7231-3p
EIF4E	hsa-miR-891
EIF4E	hsa-miR-8120
EIF4E	hsa-miR-145-5p
EIF4E	hsa-miR-6736-3p
EIF4E	hsa-miR-7658-3p
EIF4E	hsa-miR-1283
EIF4E	hsa-miR-150-5p
EIF4E	hsa-miR-548b-3p
EIF4E	hsa-miR-6748-3p
EIF4E	hsa-miR-570-3p
EIF4E	hsa-miR-372-3p
EIF4E	hsa-miR-155-3p
EIF4E	hsa-miR-6766-5p
EIF4E	hsa-miR-6704-5p
EIF4E	hsa-miR-2431-5p
EIF4E	hsa-miR-3102-3p.2-3p
EIF4E	hsa-miR-2284l
EIF4E	hsa-miR-6929-5p
EIF4E	hsa-miR-6119-5p
EIF4E	hsa-miR-5089-5p
EIF4E	hsa-miR-4714-5p
EIF4E	hsa-miR-4677-5p
EIF4E	hsa-miR-6357

EIF4E	hsa-miR-20
EIF4E	hsa-miR-105-5p
EIF4E	hsa-miR-24-3p
EIF4E	hsa-miR-323-3p
EIF4E	hsa-miR-7166-5p
EIF4E	hsa-miR-1941-3p
EIF4E	hsa-miR-4662-5p
EIF4E	hsa-miR-5010-3p
EIF4E	hsa-miR-203a-3p.1
EIF4E	hsa-miR-29b-1-5p
EIF4E	hsa-miR-7654-3p
EIF4E	hsa-miR-4692
EIF4E	hsa-miR-8804
EIF4E	hsa-miR-7385c-3p
EIF4E	hsa-miR-1791-3p
EIF4E	hsa-miR-3188
EIF4E	hsa-miR-6950-3p
EIF4E	hsa-miR-3190-5p
EIF4E	hsa-miR-631
EIF4E	hsa-miR-1559-5p
EIF4E	hsa-miR-291-3p
EIF4E	hsa-miR-548i
EIF4E	hsa-miR-4699-3p
EIF4E	hsa-miR-3153
EIF4E	hsa-miR-3551-5p
EIF4E	hsa-miR-6510-5p
EIF4E	hsa-miR-485-5p
EIF4E	hsa-miR-130
EIF4E	hsa-miR-541-3p
EIF4E	hsa-miR-6079
EIF4E	hsa-miR-588
EIF4E	hsa-miR-883a-5p
EIF4E	hsa-miR-4259
EIF4E	hsa-miR-30c-2-3p
EIF4E	hsa-miR-215
EIF4E	hsa-miR-185-5p
EIF4E	hsa-miR-3138
EIF4E	hsa-miR-202-3p
EIF4E	hsa-miR-7030-5p
EIF4E	hsa-miR-486-5p
EIF4E	hsa-miR-548u
EIF4E	hsa-miR-3942-3p
EIF4E	hsa-miR-8057
EIF4E	hsa-miR-1696
EIF4E	hsa-miR-346
EIF4E	hsa-miR-4643

EIF4E	hsa-miR-634
EIF4E	hsa-miR-19-5p
EIF4E	hsa-miR-106-5p
EIF4E	hsa-miR-4684-5p
EIF4E	hsa-miR-7863
EIF4E	hsa-miR-29b-5p
EIF4E	hsa-miR-1243
EIF4E	hsa-miR-126-3p
EIF4E	hsa-miR-3162-3p
EIF4E	hsa-miR-2406
EIF4E	hsa-miR-520f-5p
EIF4E	hsa-miR-587
EIF4E	hsa-miR-6753-3p
EIF4E	hsa-miR-6914-3p
EIF4E	hsa-miR-4272
EIF4E	hsa-miR-693-3p
EIF4E	hsa-miR-504-5p.1
EIF4E	hsa-miR-514a-5p
EIF4E	hsa-miR-5583-5p
EIF4E	hsa-miR-4433b-5p
EIF4E	hsa-miR-128-1-5p
EIF4E	hsa-miR-1324
EIF4E	hsa-miR-7198-3p
EIF4E	hsa-miR-6821-3p
EIF4E	hsa-miR-203-3p
EIF4E	hsa-miR-544a
EIF4E	hsa-miR-4693-3p
EIF4E	hsa-miR-5089-3p
EIF4E	hsa-miR-6385
EIF4E	hsa-miR-1b-5p
EIF4E	hsa-miR-4765
EIF4E	hsa-miR-520
EIF4E	hsa-miR-4659b-5p
EIF4E	hsa-miR-20-3p
EIF4E	hsa-miR-7371e-5p
EIF4E	hsa-miR-6513-3p
EIF4E	hsa-miR-8825
EIF4E	hsa-miR-4656
EIF4E	hsa-miR-4271
EIF4E	hsa-miR-1227-3p
EIF4E	hsa-miR-873a-5p.1
EIF4E	hsa-miR-934
EIF4E	hsa-miR-6511-5p
EIF4E	hsa-miR-434-3p
EIF4E	hsa-miR-2372
EIF4E	hsa-miR-6080

EIF4E	hsa-miR-7225-3p
EIF4E	hsa-miR-4461
EIF4E	hsa-miR-6516
EIF4E	hsa-miR-292a-3p
EIF4E	hsa-miR-3613b
EIF4E	hsa-miR-3968
EIF4E	hsa-miR-7090-3p
EIF4E	hsa-miR-765
EIF4E	hsa-miR-4511
EIF4E	hsa-miR-7189-3p
EIF4E	hsa-miR-7170-5p
EIF4E	hsa-miR-2052
EIF4E	hsa-miR-2131-3p
EIF4E	hsa-miR-4518
EIF4E	hsa-miR-525-5p
EIF4E	hsa-miR-8081
EIF4E	hsa-miR-4320
EIF4E	hsa-miR-1227-5p
EIF4E	hsa-miR-4257
EIF4E	hsa-miR-1910-5p
EIF4E	hsa-miR-3660
EIF4E	hsa-miR-542
EIF4E	hsa-miR-1956
EIF4E	hsa-miR-875
EIF4E	hsa-miR-3137
EIF4E	hsa-miR-3533
EIF4E	hsa-miR-1224
EIF4E	hsa-miR-6603-3p
EIF4E	hsa-miR-654-3p
EIF4E	hsa-miR-7468-5p
EIF4E	hsa-miR-616-3p
EIF4E	hsa-miR-6787-3p
EIF4E	hsa-miR-4793-3p
EIF4E	hsa-miR-2363
EIF4E	hsa-miR-141-3p
EIF4E	hsa-miR-3146
EIF4E	hsa-miR-4536-3p
EIF4E	hsa-miR-7670-5p
EIF4E	hsa-miR-7175-3p
EIF4E	hsa-miR-6935-3p
EIF4E	hsa-miR-3133
EIF4E	hsa-miR-1827
EIF4E	hsa-miR-7066-3p
EIF4E	hsa-miR-2458
EIF4E	hsa-miR-20b-5p
EIF4E	hsa-miR-3915

EIF4E	hsa-miR-6726-5p
EIF4E	hsa-miR-4311
EIF4E	hsa-miR-4533
EIF4E	hsa-miR-1956-5p
EIF4E	hsa-miR-573
EIF4E	hsa-miR-422
EIF4E	hsa-miR-337-3p
EIF4E	hsa-miR-1271-5p
EIF4E	hsa-miR-3964
EIF4E	hsa-miR-505-3p.2
EIF4E	hsa-miR-7347-5p
EIF4E	hsa-miR-135b-3p
EIF4E	hsa-miR-3668
EIF4E	hsa-miR-7210-3p
EIF4E	hsa-miR-6124
EIF4E	hsa-miR-9406
EIF4E	hsa-miR-7282-5p
EIF4E	hsa-miR-4433a-5p
EIF4E	hsa-miR-8816
EIF4E	hsa-miR-6531
EIF4E	hsa-miR-8076
EIF4E	hsa-miR-878-5p
EIF4E	hsa-miR-144-5p
EIF4E	hsa-miR-650abc
EIF4E	hsa-miR-150-5p
EIF4E	hsa-miR-2386
EIF4E	hsa-miR-3167-5p
EIF4E	hsa-miR-548c-5p
EIF4E	hsa-miR-670
EIF4E	hsa-miR-429-3p
EIF4E	hsa-miR-7g-3p
EIF4E	hsa-miR-6811-3p
EIF4E	hsa-miR-181-5p
EIF4E	hsa-miR-129-5p
EIF4E	hsa-miR-488-5p
EIF4E	hsa-let-7ag-3p
EIF4E	hsa-miR-205-5p
EIF4E	hsa-miR-33-3p
EIF4E	hsa-miR-7093-3p
EIF4E	hsa-miR-200bc
EIF4E	hsa-miR-8855
EIF4E	hsa-miR-7327-3p
EIF4E	hsa-miR-5625-3p
EIF4E	hsa-miR-519-5p
EIF4E	hsa-miR-7481-5p
EIF4E	hsa-miR-145-5p

EIF4E	hsa-miR-7-3p
EIF4E	hsa-miR-1323-5p
EIF4E	hsa-miR-7334-3p
EIF4E	hsa-miR-7087-3p
EIF4E	hsa-miR-548y
EIF4E	hsa-miR-544a
EIF4E	hsa-miR-20b-3p
EIF4E	hsa-miR-6910-3p
EIF4E	hsa-miR-2290
EIF4E	hsa-miR-2463
EIF4E	hsa-miR-3157-3p
EIF4E	hsa-miR-6838-3p
EIF4E	hsa-miR-7479-5p
EIF4E	hsa-miR-1262-3p
EIF4E	hsa-miR-5124a
EIF4E	hsa-miR-6631-5p
EIF4E	hsa-miR-338-3p
EIF4E	hsa-miR-7111-3p
EIF4E	hsa-miR-875-3p
EIF4E	hsa-miR-2276-3p
EIF4E	hsa-miR-216c-5p
EIF4E	hsa-miR-3585-5p
EIF4E	hsa-miR-8805
EIF4E	hsa-miR-7843-5p
EIF4E	hsa-miR-132-3p
EIF4E	hsa-miR-6804-5p
EIF4E	hsa-miR-5695
EIF4E	hsa-miR-7204-5p
EIF4E	hsa-miR-125a-3p
EIF4E	hsa-miR-138-5p
EIF4E	hsa-miR-4709-3p
EIF4E	hsa-miR-510
EIF4E	hsa-miR-4745-5p
EIF4E	hsa-miR-500b-3p
EIF4E	hsa-miR-6954-3p
EIF4E	hsa-miR-376a-2-5p
EIF4E	hsa-miR-8095
EIF4E	hsa-miR-6876-5p
EIF4E	hsa-miR-676-3p
EIF4E	hsa-miR-335-5p
EIF4E	hsa-miR-619
EIF4E	hsa-miR-4669
EIF4E	hsa-miR-3958
EIF4E	hsa-miR-6905-3p
EIF4E	hsa-miR-295-3p
EIF4E	hsa-miR-6870-5p

EIF4E	hsa-miR-1245a
EIF4E	hsa-miR-7157-3p
EIF4E	hsa-miR-677-5p
EIF4E	hsa-miR-7861
EIF4E	hsa-miR-743ab-3p
EIF4E	hsa-miR-888-5p
EIF4E	hsa-miR-369-3p
EIF4E	hsa-miR-195-5p
EIF4E	hsa-miR-7158-5p
EIF4E	hsa-miR-6516-3p
EIF4E	hsa-miR-1559-3p
EIF4E	hsa-let-7abf-3p
EIF4E	hsa-miR-4434
EIF4E	hsa-miR-583
EIF4E	hsa-miR-4323
EIF4E	hsa-miR-148-5p
EIF4E	hsa-miR-6844
EIF4E	hsa-miR-891a-3p
EIF4E	hsa-miR-3616-3p
EIF4E	hsa-miR-3068-3p
EIF4E	hsa-miR-107
EIF4E	hsa-miR-215-5p
EIF4E	hsa-miR-8883
EIF4E	hsa-miR-4703-3p
EIF4E	hsa-miR-4640-5p
EIF4E	hsa-miR-1689-5p
EIF4E	hsa-miR-3093-3p
EIF4E	hsa-miR-320a
EIF4E	hsa-miR-19b-3p
EIF4E	hsa-miR-669ao-3p
EIF4E	hsa-miR-599-3p
EIF4E	hsa-miR-5616-3p
EIF4E	hsa-miR-4674
EIF4E	hsa-miR-3100-3p
EIF4E	hsa-miR-7859
EIF4E	hsa-miR-8874
EIF4E	hsa-miR-1643-3p
EIF4E	hsa-miR-3614-5p
EIF4E	hsa-miR-139-3p
EIF4E	hsa-miR-7173-3p
EIF4E	hsa-miR-603
EIF4E	hsa-miR-6782-5p
EIF4E	hsa-miR-5009-5p
EIF4E	hsa-miR-8079
EIF4E	hsa-miR-491-3p
EIF4E	hsa-miR-532-5p

EIF4E	hsa-miR-203-3p.2
EIF4E	hsa-miR-4723-3p
EIF4E	hsa-miR-8087
EIF4E	hsa-miR-3471
EIF4E	hsa-miR-124-3p.1
EIF4E	hsa-miR-345-5p
EIF4E	hsa-miR-7663-5p
EIF4E	hsa-miR-4279
EIF4E	hsa-miR-548t-5p
EIF4E	hsa-miR-7321-5p
EIF4E	hsa-miR-1288
EIF4E	hsa-miR-6740-3p
EIF4E	hsa-miR-511-5p
EIF4E	hsa-miR-6514-3p
EIF4E	hsa-miR-6399
EIF4E	hsa-miR-431-5p
EIF4E	hsa-miR-2322-3p
EIF4E	hsa-miR-7330-3p
EIF4E	hsa-miR-4778-5p
EIF4E	hsa-miR-136-5p
EIF4E	hsa-miR-330-5p
EIF4E	hsa-miR-548bd-3p
EIF4E	hsa-miR-496
EIF4E	hsa-miR-301-3p
EIF4E	hsa-miR-8802
EIF4E	hsa-miR-2285r
EIF4E	hsa-miR-7160-5p
EIF4E	hsa-miR-6984-3p
EIF4E	hsa-miR-6853-3p
EIF4E	hsa-miR-1226-3p
EIF4E	hsa-miR-425
EIF4E	hsa-miR-1207-3p
EIF4E	hsa-miR-4714-3p
EIF4E	hsa-miR-584-3p
EIF4E	hsa-miR-323b-5p
EIF4E	hsa-miR-8808
EIF4E	hsa-miR-323
EIF4E	hsa-miR-4649-3p
EIF4E	hsa-miR-4766-5p
EIF4E	hsa-miR-2285
EIF4E	hsa-miR-592-5p
EIF4E	hsa-miR-4652-3p
EIF4E	hsa-miR-1237-3p
EIF4E	hsa-miR-15b-5p
EIF4E	hsa-miR-2465
EIF4E	hsa-miR-5011-5p

EIF4E	hsa-miR-16-3p
EIF4E	hsa-miR-1245-3p
EIF4E	hsa-miR-374-5p
EIF4E	hsa-miR-183-3p
EIF4E	hsa-miR-6981-3p
EIF4E	hsa-miR-1793
EIF4E	hsa-miR-7847-3p
EIF4E	hsa-miR-4727-5p
EIF4E	hsa-miR-133a-5p
EIF4E	hsa-miR-6951-5p
EIF4E	hsa-miR-713
EIF4E	hsa-miR-6696-5p
EIF4E	hsa-miR-7170-3p
EIF4E	hsa-miR-669k-5p
EIF4E	hsa-miR-519d-3p
EIF4E	hsa-miR-5591-3p
EIF4E	hsa-miR-339-5p
EIF4E	hsa-miR-3106-3p
EIF4E	hsa-miR-5589-5p
EIF4E	hsa-miR-6856-3p
EIF4E	hsa-miR-6845-5p
EIF4E	hsa-miR-3198
EIF4E	hsa-miR-1841
EIF4E	hsa-miR-627-5p
EIF4E	hsa-miR-140-3p.2
EIF4E	hsa-miR-4722-5p
EIF4E	hsa-miR-6776-5p
EIF4E	hsa-miR-4711-5p
EIF4E	hsa-miR-7015-5p
EIF4E	hsa-miR-455-3p
EIF4E	hsa-miR-3691-5p
EIF4E	hsa-miR-4436b-5p
EIF4E	hsa-miR-3069-3p
EIF4E	hsa-miR-510-3p
EIF4E	hsa-miR-1456-5p
EIF4E	hsa-miR-367-3p
EIF4E	hsa-miR-7332-5p
EIF4E	hsa-miR-361-5p
EIF4E	hsa-miR-4270
EIF4E	hsa-miR-3577
EIF4E	hsa-miR-508-5p
EIF4E	hsa-miR-7851-3p
EIF4E	hsa-miR-1246
EIF4E	hsa-miR-8058
EIF4E	hsa-miR-181b-1-3p
EIF4E	hsa-miR-660-5p

EIF4E	hsa-miR-208a-3p
EIF4E	hsa-miR-548f-3p
EIF4E	hsa-miR-15b-3p
EIF4E	hsa-miR-4253
EIF4E	hsa-miR-324-3p
EIF4E	hsa-miR-4659a-5p
EIF4E	hsa-miR-6976-5p
EIF4E	hsa-miR-10a-3p
EIF4E	hsa-miR-2325
EIF4E	hsa-miR-1463
EIF4E	hsa-miR-6505-5p
EIF4E	hsa-miR-3129-3p
EIF4E	hsa-miR-6510-3p
EIF4E	hsa-miR-205-3p
EIF4E	hsa-miR-7223-5p
EIF4E	hsa-miR-644a
EIF4E	hsa-miR-200a-3p
EIF4E	hsa-miR-1271-5p
EIF4E	hsa-miR-6692-5p
EIF4E	hsa-miR-374a-5p
EIF4E	hsa-miR-4527
EIF4E	hsa-miR-138-5p
EIF4E	hsa-miR-377-5p
EIF4E	hsa-miR-676-3p
EIF4E	hsa-miR-548ao-5p
EIF4E	hsa-miR-7218-3p
EIF4E	hsa-miR-8827
EIF4E	hsa-miR-7262b-5p
EIF4E	hsa-miR-6791-5p
EIF4E	hsa-miR-7367-5p
EIF4E	hsa-miR-6741-5p
EIF4E	hsa-miR-214-5p
EIF4E	hsa-miR-329
EIF4E	hsa-miR-4732-3p
EIF4E	hsa-miR-4718
EIF4E	hsa-miR-467cd-5p
EIF4E	hsa-miR-34
EIF4E	hsa-miR-548j-3p
EIF4E	hsa-miR-140-5p
EIF4E	hsa-miR-1294
EIF4E	hsa-miR-186-5p
EIF4E	hsa-miR-1258-5p
EIF4E	hsa-miR-3057-3p
EIF4E	hsa-miR-622
EIF4E	hsa-miR-4255
EIF4E	hsa-miR-7112-3p

EIF4E	hsa-miR-144-3p
EIF4E	hsa-miR-8908-3p
EIF4E	hsa-miR-2285c
EIF4E	hsa-miR-1267
EIF4E	hsa-miR-6872-3p
EIF4E	hsa-miR-133a-3p.2
EIF4E	hsa-miR-548bb-3p
EIF4E	hsa-miR-4720-3p
EIF4E	hsa-miR-654-5p
EIF4E	hsa-miR-8055
EIF4E	hsa-miR-34c-3p
EIF4E	hsa-miR-8893
EIF4E	hsa-miR-675-3p
EIF4E	hsa-miR-3105-5p
EIF4E	hsa-miR-3572-3p
EIF4E	hsa-miR-6731-5p
EIF4E	hsa-miR-4506
EIF4E	hsa-miR-1302
EIF4E	hsa-miR-655
EIF4E	hsa-miR-7166-3p
EIF4E	hsa-miR-6780a-3p
EIF4E	hsa-miR-6630-3p
EIF4E	hsa-miR-3094-3p
EIF4E	hsa-miR-1225-5p
EIF4E	hsa-miR-4266
EIF4E	hsa-miR-7023-5p
EIF4E	hsa-miR-543
EIF4E	hsa-miR-6663-5p
EIF4E	hsa-miR-6730-3p
EIF4E	hsa-miR-7163-3p
EIF4E	hsa-miR-2284ab
EIF4E	hsa-miR-499b-3p
EIF4E	hsa-miR-7336-5p
EIF4E	hsa-miR-7155-5p
EIF4E	hsa-miR-3084-3p
EIF4E	hsa-miR-873b
EIF4E	hsa-miR-483-5p
EIF4E	hsa-miR-3544-5p
EIF4E	hsa-miR-669hk-3p
EIF4E	hsa-miR-3072-5p
EIF4E	hsa-miR-2399-5p
EIF4E	hsa-miR-4302
EIF4E	hsa-miR-32-5p
EIF4E	hsa-miR-5011-3p
EIF4E	hsa-miR-7217-5p
EIF4E	hsa-miR-2462

EIF4E	hsa-miR-7481-3p
EIF4E	hsa-miR-517
EIF4E	hsa-miR-686
EIF4E	hsa-miR-8856
EIF4E	hsa-miR-1814
EIF4E	hsa-miR-2345
EIF4E	hsa-miR-7251-5p
EIF4E	hsa-miR-543-5p
EIF4E	hsa-miR-7200-3p
EIF4E	hsa-miR-3927-5p
EIF4E	hsa-miR-8869
EIF4E	hsa-miR-5618-5p
EIF4E	hsa-miR-6371
EIF4E	hsa-miR-6499-5p
EIF4E	hsa-miR-7184-3p
EIF4E	hsa-miR-6329
EIF4E	hsa-miR-24-5p
EIF4E	hsa-miR-489
EIF4E	hsa-miR-4700-5p
EIF4E	hsa-miR-547-5p
EIF4E	hsa-miR-1273g-5p
EIF4E	hsa-miR-1224-5p
EIF4E	hsa-miR-3530-5p
EIF4E	hsa-miR-6912-5p
EIF4E	hsa-miR-329-5p
EIF4E	hsa-miR-26-5p
EIF4E	hsa-miR-344f-3p
EIF4E	hsa-miR-1260a
EIF4E	hsa-miR-4775
EIF4E	hsa-miR-2390
EIF4E	hsa-miR-1591-3p
EIF4E	hsa-miR-16-2-3p
EIF4E	hsa-miR-1237-3p
EIF4E	hsa-miR-548bb-5p
EIF4E	hsa-miR-6506-5p
EIF4E	hsa-miR-7019-5p
EIF4E	hsa-miR-6215
EIF4E	hsa-miR-6895-3p
EIF4E	hsa-miR-186-3p
EIF4E	hsa-miR-124-5p
EIF4E	hsa-miR-7398-3p
EIF4E	hsa-miR-7046-5p
EIF4E	hsa-miR-1454
EIF4E	hsa-miR-548aq-5p
EIF4E	hsa-miR-4464
EIF4E	hsa-miR-3134

EIF4E	hsa-miR-4796-3p
EIF4E	hsa-miR-433
EIF4E	hsa-miR-4742
EIF4E	hsa-miR-8867
EIF4E	hsa-miR-5193
EIF4E	hsa-miR-4794
EIF4E	hsa-miR-7028-3p
EIF4E	hsa-miR-935
EIF4E	hsa-miR-1297
EIF4E	hsa-miR-7229-3p
EIF4E	hsa-miR-3664-5p
EIF4E	hsa-miR-296-5p
EIF4E	hsa-miR-515-5p
EIF4E	hsa-miR-211-3p
EIF4E	hsa-miR-3618-5p
EIF4E	hsa-miR-4435
EIF4E	hsa-miR-2284q
EIF4E	hsa-miR-4529-5p
EIF4E	hsa-miR-1-2-5p
EIF4E	hsa-miR-4776-3p
EIF4E	hsa-miR-4328
EIF4E	hsa-miR-101-3p.1
EIF4E	hsa-miR-505-3p
EIF4E	hsa-miR-6882-5p
EIF4E	hsa-miR-500a-5p
EIF4E	hsa-miR-101b-3p.1
EIF4E	hsa-miR-548at-5p
EIF4E	hsa-miR-514-3p
EIF4E	hsa-miR-4318
EIF4E	hsa-miR-150-3p
EIF4E	hsa-miR-7284-3p
EIF4E	hsa-miR-548as-3p
EIF4E	hsa-miR-873-5p.1
EIF4E	hsa-miR-204-5p
EIF4E	hsa-miR-4514
EIF4E	hsa-miR-6917-5p
EIF4E	hsa-miR-8810
EIF4E	hsa-miR-21-5p
EIF4E	hsa-miR-2284n
EIF4E	hsa-miR-4421
EIF4E	hsa-miR-6836-3p
EIF4E	hsa-miR-7043-5p
EIF4E	hsa-miR-4795-3p
EIF4E	hsa-miR-218-1-3p
EIF4E	hsa-miR-6730-5p
EIF4E	hsa-miR-516-3p

EIF4E	hsa-miR-3620-5p
EIF4E	hsa-miR-4274
EIF4E	hsa-miR-138-3p
EIF4E	hsa-miR-3569
EIF4E	hsa-miR-6087
EIF4E	hsa-miR-7212-5p
EIF4E	hsa-miR-4264
EIF4E	hsa-miR-1262-5p
EIF4E	hsa-miR-190a-3p
EIF4E	hsa-miR-653-3p
EIF4E	hsa-miR-7382-5p
EIF4E	hsa-miR-489-3p
EIF4E	hsa-miR-1354
EIF4E	hsa-miR-2285t
EIF4E	hsa-miR-4781-3p
EIF4E	hsa-miR-8068
EIF4E	hsa-miR-376c-5p
EIF4E	hsa-miR-2329-5p
EIF4E	hsa-miR-612
EIF4E	hsa-miR-411-5p.2
EIF4E	hsa-miR-4517
EIF4E	hsa-miR-365-3p
EIF4E	hsa-miR-1577
EIF4E	hsa-miR-892b
EIF4E	hsa-miR-7299-5p
EIF4E	hsa-miR-5098
EIF4E	hsa-miR-6865-5p
EIF4E	hsa-miR-6810-3p
EIF4E	hsa-miR-6554-5p
EIF4E	hsa-miR-98-5p
EIF4E	hsa-miR-6802-3p
EIF4E	hsa-miR-4524-5p
EIF4E	hsa-miR-2113
EIF4E	hsa-miR-6762-5p
EIF4E	hsa-miR-3092-5p
EIF4E	hsa-miR-5700
EIF4E	hsa-miR-766-3p
EIF4E	hsa-miR-32-3p
EIF4E	hsa-miR-4451
EIF4E	hsa-miR-3674
EIF4E	hsa-miR-502a
EIF4E	hsa-miR-294-3p
EIF4E	hsa-miR-7386g-5p
EIF4E	hsa-miR-7665-5p
EIF4E	hsa-miR-499
EIF4E	hsa-miR-6715b-5p

EIF4E	hsa-miR-6829-5p
EIF4E	hsa-miR-1285-3p
EIF4E	hsa-miR-6960-3p
EIF4E	hsa-miR-7849-3p
EIF4E	hsa-miR-4761-3p
EIF4E	hsa-miR-3928-3p
EIF4E	hsa-miR-1677-5p
EIF4E	hsa-miR-16-1-3p
EIF4E	hsa-miR-4768-3p
EIF4E	hsa-miR-344b-3p
EIF4E	hsa-miR-513c-5p
EIF4E	hsa-miR-496.2
EIF4E	hsa-miR-7383-3p
EIF4E	hsa-miR-6809-3p
EIF4E	hsa-miR-3071-5p
EIF4E	hsa-miR-4540
EIF4E	hsa-miR-4671-3p
EIF4E	hsa-miR-3911
EIF4E	hsa-miR-212-3p
EIF4E	hsa-miR-6878-3p
EIF4E	hsa-miR-6769b-5p
EIF4E	hsa-miR-1182
EIF4E	hsa-miR-208a-5p
EIF4E	hsa-miR-6520
EIF4E	hsa-miR-1592
EIF4E	hsa-miR-4733-5p
EIF4E	hsa-miR-7704
EIF4E	hsa-miR-1273e
EIF4E	hsa-miR-1183
EIF4E	hsa-miR-922
EIF4E	hsa-miR-2284-5p
EIF4E	hsa-miR-8820
EIF4E	hsa-miR-7373b-3p
EIF4E	hsa-miR-8875
EIF4E	hsa-miR-4495
EIF4E	hsa-miR-143-5p
EIF4E	hsa-miR-7398s-5p
EIF4E	hsa-miR-3121-3p
EIF4E	hsa-miR-101-3p
EIF4E	hsa-miR-8800
EIF4E	hsa-miR-300
EIF4E	hsa-miR-329b
EIF4E	hsa-miR-6071
EIF4E	hsa-miR-8908f
EIF4E	hsa-miR-203b-5p
EIF4E	hsa-miR-181b-3p

EIF4E	hsa-miR-5690
EIF4E	hsa-miR-506-3p
EIF4E	hsa-miR-197-5p
EIF4E	hsa-miR-1951
EIF4E	hsa-miR-548d-5p
EIF4E	hsa-miR-4651
EIF4E	hsa-miR-548ax
EIF4E	hsa-miR-2278
EIF4E	hsa-miR-2446
EIF4E	hsa-miR-221-5p
EIF4E	hsa-miR-4717-5p
EIF4E	hsa-miR-466
EIF4E	hsa-miR-152
EIF4E	hsa-miR-556-5p
EIF4E	hsa-miR-7071-3p
EIF4E	hsa-miR-760-5p
EIF4E	hsa-miR-6500-3p
EIF4E	hsa-miR-3688-3p
EIF4E	hsa-miR-6797-3p
EIF4E	hsa-miR-148-3p
EIF4E	hsa-miR-34b-3p
EIF4E	hsa-miR-7005-3p
EIF4E	hsa-miR-7386m-3p
EIF4E	hsa-miR-892a
EIF4E	hsa-miR-6515-5p
EIF4E	hsa-miR-4269
EIF4E	hsa-miR-7109-3p
EIF4E	hsa-miR-383-5p.2
EIF4E	hsa-miR-6986-3p
EIF4E	hsa-miR-1301-3p
EIF4E	hsa-miR-4729
EIF4E	hsa-miR-7398s-3p
EIF4E	hsa-let-7ac-3p
EIF4E	hsa-miR-338-5p
EIF4E	hsa-miR-2970-3p
EIF4E	hsa-miR-596
EIF4E	hsa-miR-362-3p
EIF4E	hsa-miR-4477a
EIF4E	hsa-miR-19
EIF4E	hsa-miR-4286
EIF4E	hsa-miR-6862-5p
EIF4E	hsa-miR-517-3p
EIF4E	hsa-miR-6737-3p
EIF4E	hsa-miR-6829-3p
EIF4E	hsa-miR-6896-3p
EIF4E	hsa-miR-640

EIF4E	hsa-miR-6503-5p
EIF4E	hsa-miR-1636
EIF4E	hsa-miR-6975-5p
EIF4E	hsa-miR-6749-3p
EIF4E	hsa-miR-3618-3p
EIF4E	hsa-miR-6701-3p
EIF4E	hsa-miR-4696
EIF4E	hsa-miR-1260a
EIF4E	hsa-miR-3149
EIF4E	hsa-miR-7-5p
EIF4E	hsa-let-7-3p
EIF4E	hsa-miR-221-3p
EIF4E	hsa-miR-4731-3p
EIF4E	hsa-miR-4766-3p
EIF4E	hsa-miR-383-3p
EIF4E	hsa-miR-4441
EIF4E	hsa-miR-375
EIF4E	hsa-miR-1273h-5p
EIF4E	hsa-miR-2286
EIF4E	hsa-miR-425-5p
EIF4E	hsa-miR-8908b
EIF4E	hsa-miR-6879-3p
EIF4E	hsa-miR-7106-5p
EIF4E	hsa-miR-6837-5p
EIF4E	hsa-miR-424-5p
EIF4E	hsa-miR-880-5p
EIF4E	hsa-miR-7049-3p
EIF4E	hsa-miR-6321
EIF4E	hsa-miR-548i-5p
EIF4E	hsa-miR-8797
EIF4E	hsa-miR-1255b-2-3p
EIF4E	hsa-miR-509-5p
EIF4E	hsa-miR-7386k-3p
EIF4E	hsa-miR-4736
EIF4E	hsa-miR-8837
EIF4E	hsa-miR-192-3p
EIF4E	hsa-miR-96-5p
EIF4E	hsa-miR-6716-5p
EIF4E	hsa-miR-652-5p
EIF4E	hsa-miR-3586-3p
EIF4E	hsa-miR-5585-3p
EIF4E	hsa-miR-548d-3p
EIF4E	hsa-miR-101-2-5p
EIF4E	hsa-miR-6677-5p
EIF4E	hsa-miR-411-5p.1
EIF4E	hsa-miR-3095-5p

EIF4E	hsa-miR-644a
EIF4E	hsa-miR-374
EIF4E	hsa-miR-6863
EIF4E	hsa-miR-1558
EIF4E	hsa-miR-222-5p
EIF4E	hsa-miR-595
EIF4E	hsa-miR-107-5p
EIF4E	hsa-miR-7647-5p
EIF4E	hsa-miR-17
EIF4E	hsa-miR-4723-5p
EIF4E	hsa-miR-1910-3p
EIF4E	hsa-miR-23-3p
EIF4E	hsa-miR-4703-5p
EIF4E	hsa-miR-410-5p
EIF4E	hsa-miR-574-3p
EIF4E	hsa-miR-6964-5p
EIF4E	hsa-miR-7479-3p
EIF4E	hsa-miR-7332-3p
EIF4E	hsa-miR-548ae-3p
EIF4E	hsa-miR-6950-5p
EIF4E	hsa-miR-9-5p
EIF4E	hsa-miR-669b-5p
EIF4E	hsa-miR-2339
EIF4E	hsa-miR-3978
EIF4E	hsa-let-7f-2-3p
EIF4E	hsa-miR-4256
EIF4E	hsa-miR-582-5p
EIF4E	hsa-miR-4516
EIF4E	hsa-miR-6519
EIF4E	hsa-miR-766-3p
EIF4E	hsa-miR-98-3p
EIF4E	hsa-miR-6089
EIF4E	hsa-miR-6127
EIF4E	hsa-miR-3136-5p
EIF4E	hsa-miR-4755-3p
EIF4E	hsa-miR-374c-5p
EIF4E	hsa-miR-4717-3p
EIF4E	hsa-miR-769-3p
EIF4E	hsa-miR-3587
EIF4E	hsa-miR-4432
EIF4E	hsa-miR-3135b
EIF4E	hsa-miR-296-3p
EIF4E	hsa-miR-3560
EIF4E	hsa-miR-6931-5p
EIF4E	hsa-miR-3136-3p
EIF4E	hsa-miR-3606-3p

EIF4E	hsa-miR-1639
EIF4E	hsa-miR-6727-3p
EIF4E	hsa-miR-2461-5p
EIF4E	hsa-miR-466g
EIF4E	hsa-miR-18
EIF4E	hsa-miR-17-3p
EIF4E	hsa-miR-6745
EIF4E	hsa-miR-466l-5p
EIF4E	hsa-miR-6577-3p
EIF4E	hsa-miR-378a-5p
EIF4E	hsa-miR-6129
EIF4E	hsa-miR-3060-5p
EIF4E	hsa-miR-6847-5p
EIF4E	hsa-miR-3148
EIF4E	hsa-miR-1712-5p
EIF4E	hsa-miR-4481
EIF4E	hsa-miR-1224-3p
EIF4E	hsa-miR-7379-3p
EIF4E	hsa-miR-578
EIF4E	hsa-miR-487a
EIF4E	hsa-miR-3194-3p
EIF4E	hsa-miR-1256
EIF4E	hsa-miR-654-5p
EIF4E	hsa-miR-550-5p
EIF4E	hsa-miR-488-3p
EIF4E	hsa-miR-518-3p
EIF4E	hsa-miR-4529-3p
EIF4E	hsa-miR-3583-3p
EIF4E	hsa-miR-890-5p
EIF4E	hsa-miR-4647
EIF4E	hsa-miR-6536
EIF4E	hsa-miR-4774-3p
EIF4E	hsa-miR-31-5p
EIF4E	hsa-miR-577-5p
EIF4E	hsa-miR-105-5p
EIF4E	hsa-miR-2431-3p
EIF4E	hsa-miR-769-5p
EIF4E	hsa-miR-624-3p
EIF4E	hsa-miR-8103
EIF4E	hsa-miR-1805-5p
EIF4E	hsa-miR-5622-5p
EIF4E	hsa-miR-133cd-5p
EIF4E	hsa-miR-374b-3p
EIF4E	hsa-miR-135a-2-3p
EIF4E	hsa-miR-4763-3p
EIF4E	hsa-miR-3558-3p

EIF4E	hsa-miR-6911-3p
EIF4E	hsa-miR-6715-3p
EIF4E	hsa-miR-506-3p
EIF4E	hsa-miR-511
EIF4E	hsa-miR-628-5p
EIF4E	hsa-miR-7073-5p
EIF4E	hsa-miR-3143
EIF4E	hsa-miR-5093
EIF4E	hsa-miR-3187-3p
EIF4E	hsa-miR-7977
EIF4E	hsa-miR-4646-3p
EIF4E	hsa-miR-34-5p
EIF4E	hsa-miR-27
EIF4E	hsa-miR-324-5p
EIF4E	hsa-miR-558
EIF4E	hsa-miR-7159-5p
EIF4E	hsa-miR-92-3p
EIF4E	hsa-miR-592
EIF4E	hsa-miR-2681-5p
EIF4E	hsa-miR-200c-5p
EIF4E	hsa-miR-4632-5p
EIF4E	hsa-miR-1251-5p
EIF4E	hsa-miR-513
EIF4E	hsa-miR-495-3p
EIF4E	hsa-miR-376e
EIF4E	hsa-miR-10-5p
EIF4E	hsa-miR-7398m-3p
EIF4E	hsa-miR-466c-3p
EIF4E	hsa-miR-22-3p
EIF4E	hsa-miR-4312
EIF4E	hsa-miR-7196-3p
EIF4E	hsa-miR-4666a-5p
EIF4E	hsa-miR-137
EIF4E	hsa-miR-8824
EIF4E	hsa-miR-3526
EIF4E	hsa-miR-3571
EIF4E	hsa-miR-4438
EIF4E	hsa-miR-4520-2-3p
EIF4E	hsa-miR-471-3p
EIF4E	hsa-miR-6877-3p
EIF4E	hsa-miR-3692-3p
EIF4E	hsa-miR-153
EIF4E	hsa-miR-4662b
EIF4E	hsa-miR-4307
EIF4E	hsa-miR-196c-3p
EIF4E	hsa-miR-7271-5p

EIF4E	hsa-miR-3102
EIF4E	hsa-miR-7398e-3p
EIF4E	hsa-miR-8908d
EIF4E	hsa-miR-1747-3p
EIF4E	hsa-miR-5707
EIF4E	hsa-miR-920
EIF4E	hsa-miR-302a-3p
EIF4E	hsa-miR-200
EIF4E	hsa-miR-2429
EIF4E	hsa-miR-519d-5p
EIF4E	hsa-miR-331-5p
EIF4E	hsa-miR-4738-3p
EIF4E	hsa-miR-767
EIF4E	hsa-miR-7164-5p
EIF4E	hsa-miR-550
EIF4E	hsa-miR-490-3p
EIF4E	hsa-miR-6768-3p
EIF4E	hsa-miR-219b
EIF4E	hsa-miR-371b-5p
EIF4E	hsa-miR-6825-3p
EIF4E	hsa-miR-2116-5p
EIF4E	hsa-miR-6733-5p
EIF4E	hsa-miR-8881
EIF4E	hsa-miR-2115-3p
EIF4E	hsa-miR-4473
EIF4E	hsa-miR-7667-5p
EIF4E	hsa-miR-3108-5p
EIF4E	hsa-miR-656-5p
EIF4E	hsa-miR-7004-3p
EIF4E	hsa-miR-7457-3p
EIF4E	hsa-miR-139-5p
EIF4E	hsa-miR-1966-5p
EIF4E	hsa-miR-455-3p.1
EIF4E	hsa-miR-7664-5p
EIF4E	hsa-miR-3165
EIF4E	hsa-miR-1778
EIF4E	hsa-miR-221-3p
EIF4E	hsa-miR-7398u-3p
EIF4E	hsa-miR-548am-5p
EIF4E	hsa-miR-1249-5p
EIF4E	hsa-miR-382-5p
EIF4E	hsa-miR-2385-3p
EIF4E	hsa-miR-741-5p
EIF4E	hsa-miR-15-5p
EIF4E	hsa-miR-4633-5p
EIF4E	hsa-miR-7354-3p

EIF4E	hsa-miR-6715b-3p
EIF4E	hsa-miR-1207
EIF4E	hsa-miR-712-5p
EIF4E	hsa-miR-4426
EIF4E	hsa-miR-7116-5p
EIF4E	hsa-miR-153-5p
EIF4E	hsa-miR-1250-3p
EIF4E	hsa-miR-4789-5p
EIF4E	hsa-miR-7691
EIF4E	hsa-miR-6801-5p
EIF4E	hsa-miR-552
EIF4E	hsa-miR-1544-3p
EIF4E	hsa-miR-26b-3p
EIF4E	hsa-miR-7480-5p
EIF4E	hsa-miR-548aj-3p
EIF4E	hsa-miR-3525
EIF4E	hsa-miR-4300
EIF4E	hsa-miR-5186
EIF4E	hsa-miR-568
EIF4E	hsa-miR-466aep-5p
EIF4E	hsa-miR-6374
EIF4E	hsa-miR-3922-3p
EIF4E	hsa-miR-4719
EIF4E	hsa-miR-4728-3p
EIF4E	hsa-miR-3672
EIF4E	hsa-miR-3559-5p
EIF4E	hsa-miR-4494
EIF4E	hsa-miR-3099-5p
EIF4E	hsa-miR-6759-3p
EIF4E	hsa-miR-1243-5p
EIF4E	hsa-miR-692
EIF4E	hsa-miR-7398b-3p
EIF4E	hsa-miR-411-3p
EIF4E	hsa-miR-370
EIF4E	hsa-miR-4275
EIF4E	hsa-miR-5100
EIF4E	hsa-miR-3120-5p
EIF4E	hsa-miR-1229-3p
EIF4E	hsa-miR-7312-5p
EIF4E	hsa-miR-7476-5p
EIF4E	hsa-miR-9-3p
EIF4E	hsa-miR-7185-3p
EIF4E	hsa-miR-2392
EIF4E	hsa-miR-4677-3p
EIF4E	hsa-miR-6817-5p
EIF4E	hsa-miR-6878-5p

EIF4E	hsa-miR-632
EIF4E	hsa-miR-8485
EIF4E	hsa-miR-3686
EIF4E	hsa-miR-5694
EIF4E	hsa-miR-4470
EIF4E	hsa-miR-139-5p
EIF4E	hsa-miR-1304-5p
EIF4E	hsa-miR-7155-3p
EIF4E	hsa-let-7-5p
EIF4E	hsa-miR-196a-3p
EIF4E	hsa-miR-511-3p
EIF4E	hsa-miR-4802-5p
EIF4E	hsa-miR-4695-3p
EIF4E	hsa-miR-1627-3p
EIF4E	hsa-miR-6814-5p
EIF4E	hsa-miR-3941
EIF4E	hsa-miR-190a-5p
EIF4E	hsa-miR-500-5p
EIF4E	hsa-miR-522-3p
EIF4E	hsa-miR-1260b
EIF4E	hsa-miR-6857-5p
EIF4E	hsa-miR-5698
EIF4E	hsa-miR-7257-3p
EIF4E	hsa-miR-7341-3p
EIF4E	hsa-miR-516-5p
EIF4E	hsa-miR-3157-5p
EIF4E	hsa-miR-6769b-3p
EIF4E	hsa-miR-548a-3p
EIF4E	hsa-miR-523-3p
EIF4E	hsa-miR-4753-5p
EIF4E	hsa-miR-4325
EIF4E	hsa-miR-3617-3p
EIF4E	hsa-miR-325-3p
EIF4E	hsa-miR-4685-5p
EIF4E	hsa-miR-1288-5p
EIF4E	hsa-miR-497-5p
EIF4E	hsa-miR-889-3p
EIF4E	hsa-miR-8823
EIF4E	hsa-miR-3173-5p
EIF4E	hsa-miR-2301
EIF4E	hsa-miR-871-5p
EIF4E	hsa-miR-140-3p
EIF4E	hsa-miR-1812-5p
EIF4E	hsa-miR-4724-5p
EIF4E	hsa-miR-7176-3p
EIF4E	hsa-miR-2370-5p

EIF4E	hsa-miR-762
EIF4E	hsa-miR-151-3p
EIF4E	hsa-miR-2311
EIF4E	hsa-miR-2054
EIF4E	hsa-miR-7178-3p
EIF4E	hsa-miR-4298
EIF4E	hsa-miR-7264-5p
EIF4E	hsa-miR-888-5p
EIF4E	hsa-miR-650
EIF4E	hsa-miR-5691
EIF4E	hsa-miR-548az-3p
EIF4E	hsa-miR-185-3p
EIF4E	hsa-miR-1255
EIF4E	hsa-miR-495-3p
EIF4E	hsa-miR-4294
EIF4E	hsa-miR-5583-3p
EIF4E	hsa-miR-1279
EIF4E	hsa-miR-5087
EIF4E	hsa-miR-376ab-3p
EIF4E	hsa-miR-342-5p
EIF4E	hsa-miR-4254
EIF4E	hsa-miR-181-3p
EIF4E	hsa-miR-3557-5p
EIF4E	hsa-miR-3140-3p
EIF4E	hsa-miR-3105-3p
EIF4E	hsa-miR-10b-5p
EIF4E	hsa-miR-5687
EIF4E	hsa-miR-1255-3p
EIF4E	hsa-miR-3584-5p
EIF4E	hsa-miR-6943-5p
EIF4E	hsa-miR-4289
EIF4E	hsa-miR-326
EIF4E	hsa-miR-301b-5p
EIF4E	hsa-miR-5579-5p
EIF4E	hsa-miR-218-5p
EIF4E	hsa-miR-133
EIF4E	hsa-miR-7307-5p
EIF4E	hsa-miR-31-5p
EIF4E	hsa-miR-8905
EIF4E	hsa-miR-2317
EIF4E	hsa-miR-4777-5p
EIF4E	hsa-miR-493-5p
EIF4E	hsa-miR-3071-3p
EIF4E	hsa-miR-1323
EIF4E	hsa-miR-183-5p
EIF4E	hsa-miR-548am-3p

EIF4E	hsa-miR-513a
EIF4E	hsa-miR-2368-3p
EIF4E	hsa-miR-758-3p
EIF4E	hsa-miR-1388-3p
EIF4E	hsa-miR-4293
EIF4E	hsa-miR-8831
EIF4E	hsa-miR-155-5p
EIF4E	hsa-miR-4433b-3p
EIF4E	hsa-miR-350-3p
EIF4E	hsa-miR-4760-5p
EIF4E	hsa-miR-7050-3p
EIF4E	hsa-miR-1247-3p
EIF4E	hsa-miR-6783-5p
EIF4E	hsa-miR-2469
EIF4E	hsa-miR-4777-3p
EIF4E	hsa-miR-3977
EIF4E	hsa-let-7g-3p
EIF4E	hsa-miR-467b-5p
EIF4E	hsa-miR-8848
EIF4E	hsa-miR-3128
EIF4E	hsa-miR-409-3p
EIF4E	hsa-miR-3127-5p
EIF4E	hsa-miR-548f-5p
EIF4E	hsa-miR-4536-5p
EIF4E	hsa-miR-7043-3p
EIF4E	hsa-miR-6715a-3p
EIF4E	hsa-miR-376c-3p
EIF4E	hsa-miR-7158-3p
EIF4E	hsa-miR-153-3p
EIF4E	hsa-miR-7000-3p
EIF4E	hsa-miR-1251
EIF4E	hsa-miR-2110
EIF4E	hsa-miR-2421
EIF4E	hsa-miR-1668-3p
EIF4E	hsa-miR-7202-3p
EIF4E	hsa-miR-1208
EIF4E	hsa-miR-500
EIF4E	hsa-miR-3689e
EIF4E	hsa-miR-3191-5p
EIF4E	hsa-miR-7171-5p
EIF4E	hsa-miR-7201-5p
EIF4E	hsa-miR-513b-3p
EIF4E	hsa-miR-4721
EIF4E	hsa-miR-873-5p
EIF4E	hsa-miR-2397-3p
EIF4E	hsa-miR-122-5p

EIF4E	hsa-miR-4418
EIF4E	hsa-miR-4732-5p
EIF4E	hsa-miR-6834-5p
EIF4E	hsa-miR-659-3p
EIF4E	hsa-miR-147a
EIF4E	hsa-miR-3689b-5p
EIF4E	hsa-miR-8806
EIF4E	hsa-miR-1252
EIF4E	hsa-miR-1251-3p
EIF4E	hsa-miR-881-3p
EIF4E	hsa-miR-6790-3p
EIF4E	hsa-miR-551-5p
EIF4E	hsa-miR-195-5p
EIF4E	hsa-miR-503-5p
EIF4E	hsa-miR-7394-3p
EIF4E	hsa-miR-3578
EIF4E	hsa-miR-548w
EIF4E	hsa-miR-6917-3p
EIF4E	hsa-miR-3714
EIF4E	hsa-miR-3155a
EIF4E	hsa-miR-874-3p
EIF4E	hsa-miR-7094-3p
EIF4E	hsa-miR-1282
EIF4E	hsa-miR-6634-5p
EIF4E	hsa-let-7b-3p
EIF4E	hsa-miR-6855-3p
EIF4E	hsa-miR-872-3p
EIF4E	hsa-miR-4423-3p
EIF4E	hsa-miR-890
EIF4E	hsa-miR-3126-5p
EIF4E	hsa-miR-7386e-5p
EIF4E	hsa-miR-8898
EIF4E	hsa-miR-576-3p
EIF4E	hsa-miR-591
EIF4E	hsa-miR-7973
EIF4E	hsa-miR-554
EIF4E	hsa-miR-302a-5p
EIF4E	hsa-miR-6890-5p
EIF4E	hsa-miR-693-5p
EIF4E	hsa-miR-3926
EIF4E	hsa-miR-513b-5p
EIF4E	hsa-miR-335-3p
EIF4E	hsa-miR-5000-5p
EIF4E	hsa-miR-431-5p
EIF4E	hsa-miR-608
EIF4E	hsa-miR-539-5p

EIF4E	hsa-miR-2284b
EIF4E	hsa-miR-4524a-3p
EIF4E	hsa-miR-6827-5p
EIF4E	hsa-miR-2284aa
EIF4E	hsa-miR-424-3p
EIF4E	hsa-miR-7281-3p
EIF4E	hsa-miR-93-3p
EIF4E	hsa-miR-7051-3p
EIF4E	hsa-miR-597
EIF4E	hsa-miR-655-3p
EIF4E	hsa-miR-432-3p
EIF4E	hsa-miR-466dk-5p
EIF4E	hsa-miR-5007-5p
EIF4E	hsa-miR-518
EIF4E	hsa-miR-6828-3p
EIF4E	hsa-miR-4657
EIF4E	hsa-miR-4690-5p
EIF4E	hsa-miR-5586-3p
EIF4E	hsa-miR-484
EIF4E	hsa-miR-670-3p
EIF4E	hsa-miR-548ac
EIF4E	hsa-miR-514b-5p
EIF4E	hsa-miR-26-3-3p
EIF4E	hsa-miR-6941-5p
EIF4E	hsa-miR-7651-3p
EIF4E	hsa-miR-7191-5p
EIF4E	hsa-miR-559
EIF4E	hsa-miR-6132
EIF4E	hsa-miR-1625-3p
EIF4E	hsa-miR-181c-3p
EIF4E	hsa-miR-7681-3p
EIF4E	hsa-miR-4751
EIF4E	hsa-miR-3682-5p
EIF4E	hsa-miR-2285af
EIF4E	hsa-miR-8084
EIF4E	hsa-miR-224-5p
EIF4E	hsa-miR-548ay-5p
EIF4E	hsa-miR-4283
EIF4E	hsa-miR-6744-5p
EIF4E	hsa-miR-371
EIF4E	hsa-miR-4712-3p
EIF4E	hsa-miR-551b-5p
EIF4E	hsa-miR-1264-3p
EIF4E	hsa-let-7d-3p
EIF4E	hsa-miR-183-5p.2
EIF4E	hsa-miR-2285z

EIF4E	hsa-miR-4804-3p
EIF4E	hsa-miR-3059-5p
EIF4E	hsa-miR-1968-3p
EIF4E	hsa-miR-514a-3p
EIF4E	hsa-miR-1268a
EIF4E	hsa-miR-26b-5p
EIF4E	hsa-miR-2453
EIF4E	hsa-miR-6804-3p
EIF4E	hsa-miR-4513
EIF4E	hsa-miR-7205-3p
EIF4E	hsa-miR-497-5p
EIF4E	hsa-miR-6754-3p
EIF4E	hsa-miR-2319
EIF4E	hsa-miR-1704
EIF4E	hsa-miR-7390-3p
EIF4E	hsa-miR-4439
EIF4E	hsa-miR-1468-3p
EIF4E	hsa-miR-202-5p
EIF4E	hsa-miR-2284k
EIF4E	hsa-miR-4769-3p
EIF4E	hsa-miR-541-5p
EIF4E	hsa-miR-1238-5p
EIF4E	hsa-miR-586
EIF4E	hsa-miR-6720-5p
EIF4E	hsa-miR-6874-5p
EIF4E	hsa-miR-1248
EIF4E	hsa-miR-2284u
EIF4E	hsa-miR-3064
EIF4E	hsa-miR-7156-5p
EIF4E	hsa-miR-103a-2-5p
EIF4E	hsa-miR-887-5p
EIF4E	hsa-miR-193b-5p
EIF4E	hsa-miR-6734-5p
EIF4E	hsa-miR-7075-5p
EIF4E	hsa-miR-193-3p
EIF4E	hsa-miR-6086
EIF4E	hsa-miR-642-3p
EIF4E	hsa-miR-217-3p
EIF4E	hsa-miR-466ade-3p
EIF4E	hsa-miR-4476
EIF4E	hsa-miR-1225
EIF4E	hsa-miR-323a-5p
EIF4E	hsa-miR-5096
EIF4E	hsa-miR-4802-3p
EIF4E	hsa-miR-146b-3p
EIF4E	hsa-miR-548as-5p

EIF4E	hsa-miR-4693-5p
EIF4E	hsa-miR-485-3p
EIF4E	hsa-miR-25-3p
EIF4E	hsa-miR-1714
EIF4E	hsa-miR-467g
EIF4E	hsa-miR-7666-3p
EIF4E	hsa-miR-616-5p
EIF4E	hsa-miR-610
EIF4E	hsa-miR-6772-3p
EIF4E	hsa-miR-580-5p
EIF4E	hsa-miR-1612
EIF4E	hsa-miR-2284i
EIF4E	hsa-miR-7398l-3p
EIF4E	hsa-miR-6706-5p
EIF4E	hsa-miR-3618
EIF4E	hsa-miR-3607-5p
EIF4E	hsa-miR-101b-3p.2
EIF4E	hsa-miR-7197-3p
EIF4E	hsa-miR-7677-3p
EIF4E	hsa-miR-7850-5p
EIF4E	hsa-miR-501-5p
EIF4E	hsa-miR-128
EIF4E	hsa-miR-136-5p
EIF4E	hsa-miR-372
EIF4E	hsa-miR-6077
EIF4E	hsa-miR-518-5p
EIF4E	hsa-miR-6868-3p
EIF4E	hsa-miR-4430
EIF4E	hsa-miR-6831-3p
EIF4E	hsa-miR-3607-3p
EIF4E	hsa-miR-876
EIF4E	hsa-miR-6776-3p
EIF4E	hsa-miR-520f-3p
EIF4E	hsa-miR-6076
EIF4E	hsa-miR-6790-5p
EIF4E	hsa-miR-6728-5p
EIF4E	hsa-miR-182-3p
EIF4E	hsa-miR-8871
EIF4E	hsa-miR-519-3p
EIF4E	hsa-miR-101a-3p.2
EIF4E	hsa-miR-192-5p
EIF4E	hsa-miR-4675
EIF4E	hsa-miR-5580-3p
EIF4E	hsa-miR-5006-5p
EIF4E	hsa-miR-8841
EIF4E	hsa-miR-335-5p

EIF4E	hsa-miR-3689-3p
EIF4E	hsa-miR-8080
EIF4E	hsa-miR-381-3p
EIF4E	hsa-miR-939-3p
EIF4E	hsa-miR-1-3p
EIF4E	hsa-miR-655-5p
EIF4E	hsa-miR-376c
EIF4E	hsa-miR-548at-3p
EIF4E	hsa-miR-2293
EIF4E	hsa-miR-7111-5p
EIF4E	hsa-miR-548i-3p
EIF4E	hsa-miR-15
EIF4E	hsa-miR-1a-5p
EIF4E	hsa-miR-6898-3p
EIF4E	hsa-miR-33a-3p
EIF4E	hsa-miR-7641
EIF4E	hsa-miR-3577-5p
EIF4E	hsa-miR-193b-3p
EIF4E	hsa-miR-3622b-3p
EIF4E	hsa-miR-6134
EIF4E	hsa-miR-299-3p
EIF4E	hsa-miR-7385a-3p
EIF4E	hsa-miR-452-3p
EIF4E	hsa-miR-636
EIF4E	hsa-miR-759
EIF4E	hsa-miR-513c-3p
EIF4E	hsa-miR-196b-3p
EIF4E	hsa-miR-6750-5p
EIF4E	hsa-miR-4764-5p
EIF4E	hsa-miR-757
EIF4E	hsa-miR-552-5p
EIF4E	hsa-miR-7351-3p
EIF4E	hsa-miR-8791
EIF4E	hsa-miR-545-3p
EIF4E	hsa-miR-1640
EIF4E	hsa-miR-4679
EIF4E	hsa-miR-6216
EIF4E	hsa-miR-2457
EIF4E	hsa-miR-7a-1-3p
EIF4E	hsa-miR-3665
EIF4E	hsa-miR-576-5p
EIF4E	hsa-miR-522-3p
EIF4E	hsa-miR-532-5p
EIF4E	hsa-miR-7231-5p
EIF4E	hsa-miR-219-1-3p
EIF4E	hsa-miR-330-3p

EIF4E	hsa-miR-3082-5p
EIF4E	hsa-miR-881-5p
EIF4E	hsa-miR-196-3p
EIF4E	hsa-miR-580
EIF4E	hsa-miR-8892
EIF4E	hsa-miR-450b-3p
EIF4E	hsa-miR-1245a
EIF4E	hsa-miR-512-3p
EIF4E	hsa-miR-4803
EIF4E	hsa-miR-1721
EIF4E	hsa-miR-3576
EIF4E	hsa-miR-4668-3p
EIF4E	hsa-miR-892-5p
EIF4E	hsa-miR-3113-5p
EIF4E	hsa-miR-8872
EIF4E	hsa-miR-3934-5p
EIF4E	hsa-miR-15a-5p
EIF4E	hsa-miR-8799
EIF4E	hsa-miR-26
EIF4E	hsa-miR-382-5p
EIF4E	hsa-miR-5571-3p
EIF4E	hsa-miR-2383
EIF4E	hsa-miR-7177-5p
EIF4E	hsa-miR-2447
EIF4E	hsa-miR-370-3p
EIF4E	hsa-miR-760
EIF4E	hsa-miR-146
EIF4E	hsa-miR-218-2-3p
EIF4E	hsa-miR-4682
EIF4E	hsa-miR-373-3p
EIF4E	hsa-miR-6999-3p
EIF4E	hsa-miR-708-3p
EIF4E	hsa-miR-4252
EIF4E	hsa-miR-3064-5p
EIF4E	hsa-miR-3160-5p
EIF4E	hsa-miR-520gh
EIF4E	hsa-miR-7386g-3p
EIF4E	hsa-miR-216a
EIF4E	hsa-miR-4709-5p
EIF4E	hsa-miR-6826-3p
EIF4E	hsa-miR-4694-3p
EIF4E	hsa-miR-449b-3p
EIF4E	hsa-miR-101-1-5p
EIF4E	hsa-miR-2415-3p
EIF4E	hsa-miR-1206
EIF4E	hsa-miR-2393

EIF4E	hsa-miR-6906-5p
EIF4E	hsa-miR-4748
EIF4E	hsa-miR-322-5p
EIF4E	hsa-miR-3652
EIF4E	hsa-miR-668
EIF4E	hsa-miR-3180-5p
EIF4E	hsa-miR-2352
EIF4E	hsa-miR-1287-3p
EIF4E	hsa-miR-6780b-5p
EIF4E	hsa-miR-669c-5p
EIF4E	hsa-miR-4658
EIF4E	hsa-miR-3612
EIF4E	hsa-miR-1304-3p
EIF4E	hsa-miR-320-5p
EIF4E	hsa-miR-8065
EIF4E	hsa-miR-6735-3p
EIF4E	hsa-miR-4273
EIF4E	hsa-miR-26a-5p
EIF4E	hsa-miR-7188-3p
EIF4E	hsa-miR-222-3p
EIF4E	hsa-miR-509-3p
EIF4E	hsa-miR-1245b-3p
EIF4E	hsa-miR-374c-3p
EIF4E	hsa-miR-647
EIF4E	hsa-miR-4263
EIF4E	hsa-miR-383-5p
EIF4E	hsa-miR-8801
EIF4E	hsa-miR-367-3p
EIF4E	hsa-miR-1306-5p
EIF4E	hsa-miR-4505
EIF4E	hsa-miR-557
EIF4E	hsa-miR-376abd
EIF4E	hsa-miR-2416
EIF4E	hsa-miR-1434-5p
EIF4E	hsa-miR-669a-3-3p
EIF4E	hsa-miR-8873a
EIF4E	hsa-miR-8895
EIF4E	hsa-miR-1550-3p
EIF4E	hsa-miR-1188-3p
EIF4E	hsa-miR-6833-5p
EIF4E	hsa-miR-4778-3p
EIF4E	hsa-miR-1289
EIF4E	hsa-miR-6888-5p
EIF4E	hsa-miR-7168-3p
EIF4E	hsa-miR-34bc-3p
EIF4E	hsa-miR-674-5p

EIF4E	hsa-miR-665
EIF4E	hsa-miR-6237
EIF4E	hsa-miR-7646-5p
EIF4E	hsa-miR-1200
EIF4E	hsa-miR-2468
EIF4E	hsa-miR-512-5p
EIF4E	hsa-miR-561-3p
EIF4E	hsa-miR-374b-5p
EIF4E	hsa-miR-3065-3p
EIF4E	hsa-miR-205-5p
EIF4E	hsa-miR-4531
EIF4E	hsa-miR-2400
EIF4E	hsa-miR-3609
EIF4E	hsa-miR-4667
EIF4E	hsa-miR-4786-5p
EIF4E	hsa-miR-192-5p
EIF4E	hsa-miR-216a-5p
EIF4E	hsa-miR-4704-5p
EIF4E	hsa-miR-4708-5p
EIF4E	hsa-miR-548b-3p
EIF4E	hsa-miR-122-3p
EIF4E	hsa-miR-2418
EIF4E	hsa-miR-20a-3p
EIF4E	hsa-miR-1-1-5p
EIF4E	hsa-miR-4635
EIF4E	hsa-miR-3109-5p
EIF4E	hsa-miR-5002-5p
EIF4E	hsa-miR-30e
EIF4E	hsa-miR-146-5p
EIF4E	hsa-miR-3170
EIF4E	hsa-miR-548g-3p
EIF4E	hsa-miR-7478-3p
EIF4E	hsa-miR-2365
EIF4E	hsa-miR-4666b
EIF4E	hsa-miR-133b
EIF4E	hsa-miR-5591-5p
EIF4E	hsa-miR-590-3p
EIF4E	hsa-miR-4440
EIF4E	hsa-miR-539-5p
EIF4E	hsa-miR-449a
EIF4E	hsa-miR-7678-3p
EIF4E	hsa-miR-7978
EIF4E	hsa-miR-3145-5p
EIF4E	hsa-miR-6544-3p
EIF4E	hsa-miR-6841-3p
EIF4E	hsa-miR-4789-3p

EIF4E	hsa-miR-373-3p
EIF4E	hsa-miR-138-1-3p
EIF4E	hsa-miR-7647-3p
EIF4E	hsa-miR-6403
EIF4E	hsa-miR-376-3p
EIF4E	hsa-miR-2284j
EIF4E	hsa-miR-518d-5p
EIF4E	hsa-miR-6130
EIF4E	hsa-miR-6822-5p
EIF4E	hsa-miR-31-3p
EIF4E	hsa-miR-6511a-5p
EIF4E	hsa-miR-373-5p
EIF4E	hsa-miR-4317
EIF4E	hsa-miR-6807-5p
EIF4E	hsa-miR-7092-3p
EIF4E	hsa-miR-1255-5p
EIF4E	hsa-miR-4772-5p
EIF4E	hsa-miR-3062-5p
EIF4E	hsa-miR-7207-3p
EIF4E	hsa-miR-5196-5p
EIF4E	hsa-miR-2455
EIF4E	hsa-miR-6739-5p
EIF4E	hsa-miR-4662a-3p
EIF4E	hsa-miR-2291
EIF4E	hsa-miR-509-3p
EIF4E	hsa-miR-4800-3p
EIF4E	hsa-miR-9768-3p
EIF4E	hsa-miR-548aef
EIF4E	hsa-miR-6729-3p
EIF4E	hsa-miR-4428
EIF4E	hsa-miR-4742-5p
EIF4E	hsa-miR-148a-5p
EIF4E	hsa-miR-7386j-3p
EIF4E	hsa-miR-7107-3p
EIF4E	hsa-miR-219a-1-3p
EIF4E	hsa-miR-3101-3p
EIF4E	hsa-miR-548aj-5p
EIF4E	hsa-miR-6778-3p
EIF4E	hsa-miR-4448
EIF4E	hsa-miR-1781-3p
EIF4E	hsa-miR-6728-3p
EIF4E	hsa-miR-365b-5p
EIF4E	hsa-miR-548e-3p
EIF4E	hsa-miR-2485
EIF4E	hsa-miR-500b-5p
EIF4E	hsa-miR-7005-5p

EIF4E	hsa-miR-34c-5p
EIF4E	hsa-miR-3529-3p
EIF4E	hsa-miR-1540-5p
EIF4E	hsa-miR-589-5p
EIF4E	hsa-miR-7163-5p
EIF4E	hsa-miR-4484
EIF4E	hsa-miR-7-5p
EIF4E	hsa-miR-4536-5p
EIF4E	hsa-miR-7394a-5p
EIF4E	hsa-miR-5582-3p
EIF4E	hsa-miR-2284m
EIF4E	hsa-miR-1283a
EIF4E	hsa-miR-668-3p
EIF4E	hsa-miR-383
EIF4E	hsa-miR-6764-3p
EIF4E	hsa-miR-6848-3p
EIF4E	hsa-miR-6791-3p
EIF4E	hsa-miR-3185
EIF4E	hsa-miR-2361
EIF4E	hsa-miR-6937-5p
EIF4E	hsa-miR-6834-3p
EIF4E	hsa-miR-199-3p
EIF4E	hsa-miR-548n
EIF4E	hsa-miR-140-5p
EIF4E	hsa-miR-519
EIF4E	hsa-miR-3651
EIF4E	hsa-miR-103-3p
EIF4E	hsa-miR-1273g-3p
EIF4E	hsa-miR-4757-5p
EIF4E	hsa-miR-1969
EIF4E	hsa-miR-6344
EIF4E	hsa-miR-301a-5p
EIF4E	hsa-miR-499b-5p
EIF4E	hsa-miR-6972-3p
EIF4E	hsa-miR-219-3p
EIF4E	hsa-miR-6966-3p
EIF4E	hsa-miR-6756-5p
EIF4E	hsa-miR-669h-5p
EIF4E	hsa-miR-8077
EIF4E	hsa-miR-4798-3p
EIF4E	hsa-miR-6793-3p
EIF4E	hsa-miR-1285-3p
EIF4E	hsa-miR-548a-5p
EIF4E	hsa-miR-944
EIF4E	hsa-miR-3065-5p
EIF4E	hsa-miR-548

EIF4E	hsa-miR-4701-3p
EIF4E	hsa-miR-6409
EIF4E	hsa-miR-149-5p
EIF4E	hsa-miR-1262
EIF4E	hsa-miR-7036b-3p
EIF4E	hsa-miR-1323-3p
EIF4E	hsa-miR-934-5p
EIF4E	hsa-miR-1255b-5p
EIF4E	hsa-miR-190-5p
EIF4E	hsa-miR-7372-3p
EIF4E	hsa-miR-6355
EIF4E	hsa-miR-331-3p
EIF4E	hsa-miR-3095-3p
EIF4E	hsa-miR-101-3p
EIF4E	hsa-miR-1789
EIF4E	hsa-miR-5195-5p
EIF4E	hsa-miR-8066
EIF4E	hsa-miR-4534
EIF4E	hsa-miR-6719-3p
EIF4E	hsa-miR-548ar-3p
EIF4E	hsa-miR-3918
EIF4E	hsa-miR-5007-3p
EIF4E	hsa-miR-1651-3p
EIF4E	hsa-miR-504-3p
EIF4E	hsa-miR-3622b-5p
EIF4E	hsa-miR-6165
EIF4E	hsa-miR-6502-3p
EIF4E	hsa-miR-564
EIF4E	hsa-miR-8063
EIF4E	hsa-miR-124-3p.2
EIF4E	hsa-miR-1562-5p
EIF4E	hsa-miR-5787
EIF4E	hsa-miR-7152-5p
EIF4E	hsa-miR-125
EIF4E	hsa-miR-4499
EIF4E	hsa-miR-411-5p
EIF4E	hsa-miR-302c-3p
EIF4E	hsa-miR-4801
EIF4E	hsa-miR-6819-3p
EIF4E	hsa-miR-604
EIF4E	hsa-miR-1746
EIF4E	hsa-miR-143-3p
EIF4E	hsa-miR-500-3p
EIF4E	hsa-miR-365a-3p
EIF4E	hsa-miR-3572-5p
EIF4E	hsa-miR-499b-3p

EIF4E	hsa-miR-2379
EIF4E	hsa-miR-432-5p
EIF4E	hsa-miR-4268
EIF4E	hsa-miR-203-3p.1
EIF4E	hsa-miR-7201-3p
EIF4E	hsa-miR-5584-5p
EIF4E	hsa-miR-28-3p
EIF4E	hsa-miR-6870-3p
EIF4E	hsa-miR-7390-5p
EIF4E	hsa-miR-133b-5p
EIF4E	hsa-miR-767-3p
EIF4E	hsa-miR-1258-3p
EIF4E	hsa-miR-6880-5p
EIF4E	hsa-miR-27-3p
EIF4E	hsa-miR-672-3p
EIF4E	hsa-miR-6378
EIF4E	hsa-miR-330-3p.1
EIF4E	hsa-miR-7683-3p
EIF4E	hsa-miR-3973
EIF4E	hsa-miR-7437-5p
EIF4E	hsa-miR-1269a
EIF4E	hsa-miR-2384
EIF4E	hsa-miR-19a-5p
EIF4E	hsa-miR-7298-3p
EIF4E	hsa-miR-3182
EIF4E	hsa-miR-1766
EIF4E	hsa-miR-2284a
EIF4E	hsa-miR-614
EIF4E	hsa-miR-4750-5p
EIF4E	hsa-miR-7092-5p
EIF4E	hsa-miR-106b-5p
EIF4E	hsa-miR-2891
EIF4E	hsa-miR-519e-5p
EIF4E	hsa-miR-320-3p
EIF4E	hsa-miR-664b-5p
EIF4E	hsa-miR-9-5p
EIF4E	hsa-miR-101b-5p
EIF4E	hsa-miR-4498
EIF4E	hsa-miR-548ad-5p
EIF4E	hsa-miR-4699-5p
EIF4E	hsa-miR-1226-5p
EIF4E	hsa-miR-1843a-5p
EIF4E	hsa-miR-6407
EIF4E	hsa-miR-524-5p
EIF4E	hsa-miR-6529-5p
EIF4E	hsa-miR-6992-5p

EIF4E	hsa-miR-365a-5p
EIF4E	hsa-miR-210
EIF4E	hsa-miR-7668-5p
EIF4E	hsa-miR-651-3p
EIF4E	hsa-miR-2284x
EIF4E	hsa-miR-3183
EIF4E	hsa-miR-200a-5p
EIF4E	hsa-miR-340-3p
EIF4E	hsa-miR-7386k-5p
EIF4E	hsa-miR-30-5p
EIF4E	hsa-miR-1205
EIF4E	hsa-miR-5582-5p
EIF4E	hsa-miR-7364-3p
EIF4E	hsa-miR-1465
EIF4E	hsa-miR-302f
EIF4E	hsa-miR-147a
EIF4E	hsa-miR-361-5p
EIF4E	hsa-miR-378
EIF4E	hsa-miR-873-5p.2
EIF4E	hsa-miR-2480
EIF4E	hsa-miR-6843-3p
EIF4E	hsa-miR-7269-5p
EIF4E	hsa-miR-6769a-5p
EIF4E	hsa-miR-135ab-3p
EIF4E	hsa-miR-548ab
EIF4E	hsa-miR-498
EIF4E	hsa-miR-4650-3p
EIF4E	hsa-miR-4741
EIF4E	hsa-miR-1184
EIF4E	hsa-miR-637
EIF4E	hsa-miR-5619-3p
EIF4E	hsa-miR-340-5p
EIF4E	hsa-miR-6883-5p
EIF4E	hsa-miR-3588
EIF4E	hsa-miR-7041-5p
EIF4E	hsa-miR-619-3p
EIF4E	hsa-miR-4722-3p
EIF4E	hsa-miR-203b-3p
EIF4E	hsa-miR-491-5p
EIF4E	hsa-miR-2335
EIF4E	hsa-miR-936
EIF4E	hsa-miR-6590-3p
EIF4E	hsa-miR-200-3p
EIF4E	hsa-miR-656
EIF4E	hsa-miR-7371-5p
EIF4E	hsa-miR-6824-3p

EIF4E	hsa-miR-7374a-3p
EIF4E	hsa-miR-302c-3p.2
EIF4E	hsa-miR-548o-5p
EIF4E	hsa-miR-301-5p
EIF4E	hsa-miR-4539
EIF4E	hsa-miR-338-3p
EIF4E	hsa-miR-1343
EIF4E	hsa-miR-378-3p
EIF4E	hsa-miR-337-3p
EIF4E	hsa-miR-4759
EIF4E	hsa-miR-6855-5p
EIF4E	hsa-miR-6902-3p
EIF4E	hsa-miR-4474-3p
EIF4E	hsa-miR-8108
EIF4E	hsa-miR-376a-1-5p
EIF4E	hsa-miR-4510
EIF4E	hsa-miR-3604
EIF4E	hsa-miR-2887
EIF4E	hsa-miR-4760-3p
EIF4E	hsa-miR-323a-3p
EIF4E	hsa-miR-3680-3p
EIF4E	hsa-miR-542-3p
EIF4E	hsa-miR-34a-3p
EIF4E	hsa-miR-379-3p
EIF4E	hsa-miR-2442
EIF4E	hsa-miR-194-5p
EIF4E	hsa-miR-23-5p
EIF4E	hsa-miR-6813-3p
EIF4E	hsa-miR-20-5p
EIF4E	hsa-miR-4633-3p
EIF4E	hsa-miR-4762-5p
EIF4E	hsa-miR-545-5p
EIF4E	hsa-miR-6081
EIF4E	hsa-miR-4772-3p
EIF4E	hsa-miR-6942-3p
EIF4E	hsa-miR-548ae-5p
EIF4E	hsa-miR-6907-5p
EIF4E	hsa-miR-6589-5p
EIF4E	hsa-miR-1273d
EIF4E	hsa-miR-2430
EIF4E	hsa-miR-6613-3p
EIF4E	hsa-miR-4436b-3p
EIF4E	hsa-miR-501-5p
EIF4E	hsa-miR-7687-3p
EIF4E	hsa-miR-3192
EIF4E	hsa-let-7f-1-3p

EIF4E	hsa-miR-7671-3p
EIF4E	hsa-miR-550a-3-5p
EIF4E	hsa-miR-133-3p
EIF4E	hsa-miR-615-5p
EIF4E	hsa-miR-518c-5p
EIF4E	hsa-miR-3664-5p
EIF4E	hsa-miR-656-3p
EIF4E	hsa-miR-1287
EIF4E	hsa-miR-2284v
EIF4E	hsa-miR-18-3p
EIF4E	hsa-miR-8067
EIF4E	hsa-miR-4645-3p
EIF4E	hsa-miR-206-3p
EIF4E	hsa-miR-5699-5p
EIF4E	hsa-miR-6720-3p
EIF4E	hsa-miR-628-3p
EIF4E	hsa-miR-3913-5p
EIF4E	hsa-miR-8854
EIF4E	hsa-miR-125b-2-3p
EIF4E	hsa-miR-151
EIF4E	hsa-miR-4758-5p
EIF4E	hsa-miR-1239
EIF4E	hsa-miR-9500
EIF4E	hsa-miR-6971-5p
EIF4E	hsa-miR-200ab-5p
EIF4E	hsa-miR-7398t-3p
EIF4E	hsa-miR-6857-3p
EIF4E	hsa-miR-6333
EIF4E	hsa-miR-508b
EIF4E	hsa-miR-7442-3p
EIF4E	hsa-miR-4773
EIF4E	hsa-miR-3924
EIF4E	hsa-miR-124a-5p
EIF4E	hsa-miR-6933-3p
EIF4E	hsa-miR-3536
EIF4E	hsa-miR-1263
EIF4E	hsa-miR-7113-3p
EIF4E	hsa-miR-142-3p.2
EIF4E	hsa-miR-34b-3p
EIF4E	hsa-miR-1584-3p
EIF4E	hsa-miR-149-3p
EIF4E	hsa-miR-1272
EIF4E	hsa-miR-487b-5p
EIF4E	hsa-miR-216-5p
EIF4E	hsa-miR-92a-2-5p
EIF4E	hsa-miR-137-3p

EIF4E	hsa-miR-450a-1-3p
EIF4E	hsa-miR-4422
EIF4E	hsa-miR-548p
EIF4E	hsa-miR-429-5p
EIF4E	hsa-miR-7160-3p
EIF4E	hsa-miR-96-5p
EIF4E	hsa-miR-645
EIF4E	hsa-miR-7206-5p
EIF4E	hsa-miR-5001-5p
EIF4E	hsa-miR-7236-5p
EIF4E	hsa-miR-891-3p
EIF4E	hsa-miR-3200-5p
EIF4E	hsa-miR-6083
EIF4E	hsa-miR-206
EIF4E	hsa-miR-2467-3p
EIF4E	hsa-miR-188-5p
EIF4E	hsa-miR-6507-5p
EIF4E	hsa-miR-6366
EIF4E	hsa-miR-207
EIF4E	hsa-miR-223-3p
EIF4E	hsa-miR-465-5p
EIF4E	hsa-miR-133ab
EIF4E	hsa-miR-2464-5p
EIF4E	hsa-miR-3140-5p
EIF4E	hsa-miR-3127-5p
EIF4E	hsa-miR-6772-5p
EIF4E	hsa-miR-29
EIF4E	hsa-miR-6818-3p
EIF4E	hsa-miR-4452
EIF4E	hsa-miR-1582
EIF4E	hsa-miR-582-3p
EIF4E	hsa-miR-5008-3p
EIF4E	hsa-miR-4680-5p
EIF4E	hsa-miR-561-3p
EIF4E	hsa-miR-6133
EIF4E	hsa-miR-7472-3p
EIF4E	hsa-miR-549a
EIF4E	hsa-miR-6416-5p
EIF4E	hsa-miR-661
EIF4E	hsa-miR-4468
EIF4E	hsa-miR-4501
EIF4E	hsa-miR-4755-5p
EIF4E	hsa-miR-324
EIF4E	hsa-miR-1797
EIF4E	hsa-miR-1278
EIF4E	hsa-miR-200a-3p

EIF4E	hsa-miR-199-5p
EIF4E	hsa-miR-5189-5p
EIF4E	hsa-miR-2367-3p
EIF4E	hsa-miR-3199
EIF4E	hsa-miR-4419a
EIF4E	hsa-miR-5191
EIF4E	hsa-miR-590-5p
EIF4E	hsa-miR-885
EIF4E	hsa-miR-518a-5p
EIF4E	hsa-miR-6418-3p
EIF4E	hsa-miR-7199-5p
EIF4E	hsa-miR-129-5p
EIF4E	hsa-miR-8906
EIF4E	hsa-miR-7392-3p
EIF4E	hsa-miR-4443
EIF4E	hsa-miR-6537-3p
EIF4E	hsa-miR-1236-5p
EIF4E	hsa-miR-433-3p
EIF4E	hsa-miR-7068-5p
EIF4E	hsa-miR-7247-5p
EIF4E	hsa-miR-503-5p
EIF4E	hsa-miR-340-5p
EIF4E	hsa-miR-6874-3p
EIF4E	hsa-miR-20a-5p
EIF4E	hsa-miR-367-5p
EIF4E	hsa-miR-369-3p
EIF4E	hsa-miR-183-5p.1
EIF4E	hsa-miR-3662
EIF4E	hsa-miR-4463
EIF4E	hsa-miR-106
EIF4E	hsa-miR-769
EIF4E	hsa-miR-940
EIF4E	hsa-miR-549-3p
EIF4E	hsa-miR-291-5p
EIF4E	hsa-miR-7661-5p
EIF4E	hsa-miR-8866
EIF4E	hsa-miR-485
EIF4E	hsa-miR-212-5p
EIF4E	hsa-miR-524-3p
EIF4E	hsa-miR-6980-5p
EIF4E	hsa-miR-600
EIF4E	hsa-miR-6760-5p
EIF4E	hsa-miR-6908-5p
EIF4E	hsa-miR-6985-5p
EIF4E	hsa-miR-605-5p
EIF4E	hsa-miR-2486-5p

EIF4E	hsa-miR-2285d
EIF4E	hsa-miR-106a-5p
EIF4E	hsa-miR-641
EIF4E	hsa-miR-3929
EIF4E	hsa-miR-5004-5p
EIF4E	hsa-miR-3152-3p
EIF4E	hsa-miR-7852-3p
EIF4E	hsa-miR-507b
EIF4E	hsa-miR-657
EIF4E	hsa-miR-3611
EIF4E	hsa-miR-6836-5p
EIF4E	hsa-miR-649
EIF4E	hsa-miR-302-3p
EIF4E	hsa-miR-5680
EIF4E	hsa-miR-378g
EIF4E	hsa-miR-7466-3p
EIF4E	hsa-miR-7151-3p
EIF4E	hsa-miR-1343-3p
EIF4E	hsa-miR-7011-3p
EIF4E	hsa-miR-3655
EIF4E	hsa-miR-2284c
EIF4E	hsa-miR-411-5p
EIF4E	hsa-miR-126b-5p
EIF4E	hsa-miR-4793-5p
EIF4E	hsa-miR-2366
EIF4E	hsa-miR-7315-3p
EIF4E	hsa-miR-6889-3p
EIF4E	hsa-miR-5585-5p
EIF4E	hsa-miR-200b-3p
EIF4E	hsa-miR-7208-3p
EIF4E	hsa-miR-6513-5p
EIF4E	hsa-miR-7180-5p
EIF4E	hsa-miR-107-3p
EIF4E	hsa-miR-181
EIF4E	hsa-miR-363-5p
EIF4E	hsa-miR-4683
EIF4E	hsa-miR-4739
EIF4E	hsa-miR-146a-3p
EIF4E	hsa-miR-548k
EIF4E	hsa-miR-6508-5p
EIF4E	hsa-miR-188-3p
EIF4E	hsa-miR-6345
EIF4E	hsa-miR-7042-3p
EIF4E	hsa-miR-6918-5p
EIF4E	hsa-miR-1434-3p
EIF4E	hsa-miR-514a-3p

EIF4E	hsa-miR-10b-5p
EIF4E	hsa-miR-201-3p
EIF4E	hsa-miR-4634
EIF4E	hsa-miR-1388-5p
EIF4E	hsa-miR-93-5p
EIF4E	hsa-miR-7361-5p
EIF4E	hsa-miR-487a-5p
EIF4E	hsa-miR-664a-5p
EIF4E	hsa-miR-222-3p
EIF4E	hsa-miR-6989-3p
EIF4E	hsa-miR-4496
EIF4E	hsa-miR-6534
EIF4E	hsa-miR-421
EIF4E	hsa-miR-505-5p
EIF4E	hsa-miR-7074-5p
EIF4E	hsa-miR-7010-5p
EIF4E	hsa-miR-3085-3p
EIF4E	hsa-miR-1555-5p
EIF4E	hsa-miR-92a-1-5p
EIF4E	hsa-miR-938
EIF4E	hsa-miR-580-3p
EIF4E	hsa-miR-142
EIF4E	hsa-miR-362-5p
EIF4E	hsa-miR-144-3p
EIF4E	hsa-miR-325
EIF4E	hsa-miR-7162-3p
EIF4E	hsa-miR-1753
EIF4E	hsa-miR-8073
EIF4E	hsa-miR-454-3p
EIF4E	hsa-miR-19-3p
EIF4E	hsa-miR-509-3-5p
EIF4E	hsa-miR-3653-5p
EIF4E	hsa-miR-5006-3p
EIF4E	hsa-miR-7333-5p
EIF4E	hsa-miR-3689f
EIF4E	hsa-miR-550a-5p
EIF4E	hsa-miR-1322
EIF4E	hsa-miR-3945
EIF4E	hsa-miR-6785-5p
EIF4E	hsa-miR-763
EIF4E	hsa-miR-6516-5p
EIF4E	hsa-miR-3192-3p
EIF4E	hsa-miR-3078-3p
EIF4E	hsa-miR-33
EIF4E	hsa-miR-379-5p
EIF4E	hsa-miR-6554-3p

EIF4E	hsa-miR-4487
EIF4E	hsa-miR-7297-3p
EIF4E	hsa-miR-448
EIF4E	hsa-miR-8052
EIF4E	hsa-miR-4797-3p
EIF4E	hsa-miR-6529a
EIF4E	hsa-miR-675-5p
EIF4E	hsa-miR-7703
EIF4E	hsa-miR-764
EIF4E	hsa-miR-3123
EIF4E	hsa-miR-4668-5p
EIF4E	hsa-miR-650d
EIF4E	hsa-miR-5009-3p
EIF4E	hsa-miR-2285u
EIF4E	hsa-miR-7079-3p
EIF4E	hsa-miR-6883-3p
EIF4E	hsa-miR-1254
EIF4E	hsa-miR-298
EIF4E	hsa-miR-450-5p
EIF4E	hsa-miR-223-5p
EIF4E	hsa-miR-203
EIF4E	hsa-miR-3162-5p
EIF4E	hsa-miR-200bc-3p
EIF4E	hsa-miR-1664-3p
EIF4E	hsa-miR-4691-5p
EIF4E	hsa-miR-4282
EIF4E	hsa-miR-6771-3p
EIF4E	hsa-miR-2322-5p
EIF4E	hsa-miR-8056
EIF4E	hsa-miR-502-3p
EIF4E	hsa-miR-190-3p
EIF4E	hsa-miR-625-3p
EIF4E	hsa-miR-4700-3p
EIF4E	hsa-miR-7175-5p
EIF4E	hsa-miR-215-3p
EIF4E	hsa-miR-3090-5p
EIF4E	hsa-miR-7220-3p
EIF4E	hsa-miR-8817
EIF4E	hsa-miR-181b-2-3p
EIF4E	hsa-miR-8061
EIF4E	hsa-miR-6910-5p
EIF4E	hsa-miR-942-3p
EIF4E	hsa-miR-597-5p
EIF4E	hsa-miR-4524a-5p
EIF4E	hsa-miR-454
EIF4E	hsa-miR-8062

EIF4E	hsa-miR-452-5p
EIF4E	hsa-miR-1812-3p
EIF4E	hsa-miR-17-5p
EIF4E	hsa-miR-486-5p
EIF4E	hsa-miR-494-5p
EIF4E	hsa-miR-6983-3p
EIF4E	hsa-miR-3187-5p
EIF4E	hsa-miR-128-3p
EIF4E	hsa-miR-6780a-5p
EIF4E	hsa-miR-142-5p
EIF4E	hsa-miR-4489
EIF4E	hsa-miR-7394b-5p
EIF4E	hsa-miR-2284d
EIF4E	hsa-miR-3580-3p
EIF4E	hsa-miR-1708
EIF4E	hsa-miR-7045-5p
EIF4E	hsa-miR-499-5p
EIF4E	hsa-miR-6860
EIF4E	hsa-miR-2285y
EIF4E	hsa-miR-1768
EIF4E	hsa-miR-7076-5p
EIF4E	hsa-miR-197-3p
EIF4E	hsa-miR-6758-3p
EIF4E	hsa-miR-432-5p
EIF4E	hsa-miR-885-5p
EIF4E	hsa-miR-1551-5p
EIF4E	hsa-miR-497-3p
EIF4E	hsa-miR-7108-5p
EIF4E	hsa-miR-6867-5p
EIF4E	hsa-miR-6765-3p
EIF4E	hsa-miR-1804
EIF4E	hsa-miR-3561-5p
EIF4E	hsa-miR-194-5p
EIF4E	hsa-miR-5623-3p
EIF4E	hsa-miR-126-5p
EIF4E	hsa-miR-1185-3p
EIF4E	hsa-miR-548ay-3p
EIF4E	hsa-miR-1667-5p
EIF4E	hsa-miR-5588-3p
EIF4E	hsa-miR-577-3p
EIF4E	hsa-miR-6652-5p
EIF4E	hsa-miR-105b
EIF4E	hsa-miR-6514-5p
EIF4E	hsa-miR-7203-5p
EIF4E	hsa-miR-4522
EIF4E	hsa-miR-7386h-5p

EIF4E	hsa-miR-380
EIF4E	hsa-miR-569
EIF4E	hsa-miR-562
EIF4E	hsa-miR-6760-3p
EIF4E	hsa-miR-576-5p
EIF4E	hsa-miR-423-3p
EIF4E	hsa-miR-6782-3p
EIF4E	hsa-miR-3145-3p
EIF4E	hsa-miR-1270
EIF4E	hsa-miR-3116
EIF4E1B	hsa-miR-152-3p
EIF4E1B	hsa-miR-328
EIF4E1B	hsa-miR-6784-3p
EIF4E1B	hsa-miR-5008-5p
EIF4E1B	hsa-miR-30c-2-3p
EIF4E1B	hsa-miR-3667-3p
EIF4E1B	hsa-miR-6407
EIF4E1B	hsa-miR-1293
EIF4E1B	hsa-miR-30-3p
EIF4E1B	hsa-miR-485-5p
EIF4E1B	hsa-miR-500-3p
EIF4E1B	hsa-miR-455-3p.1
EIF4E1B	hsa-let-7c-3p
EIF4E1B	hsa-miR-1470
EIF4E1B	hsa-miR-208-3p
EIF4E1B	hsa-miR-421
EIF4E1B	hsa-miR-376c
EIF4E1B	hsa-miR-1191a
EIF4E1B	hsa-miR-3934-3p
EIF4E1B	hsa-miR-4494
EIF4E1B	hsa-miR-7386o-3p
EIF4E1B	hsa-miR-8887
EIF4E1B	hsa-miR-1225
EIF4E1B	hsa-miR-4667-5p
EIF4E1B	hsa-miR-188-5p
EIF4E1B	hsa-miR-7045-3p
EIF4E1B	hsa-miR-6851-3p
EIF4E1B	hsa-miR-885-5p
EIF4E1B	hsa-miR-4650-5p
EIF4E1B	hsa-miR-7017-5p
EIF4E1B	hsa-miR-3176
EIF4E1B	hsa-miR-4483
EIF4E1B	hsa-miR-107-5p
EIF4E1B	hsa-miR-328-3p
EIF4E1B	hsa-miR-24-3p
EIF4E1B	hsa-miR-8790

EIF4E1B	hsa-miR-4742
EIF4E1B	hsa-miR-2461-5p
EIF4E1B	hsa-miR-92a-2-5p
EIF4E1B	hsa-miR-874-5p
EIF4E1B	hsa-miR-874
EIF4E1B	hsa-miR-548j-5p
EIF4E1B	hsa-miR-4518
EIF4E1B	hsa-miR-350
EIF4E1B	hsa-miR-6825-5p
EIF4E1B	hsa-miR-3965
EIF4E1B	hsa-miR-686
EIF4E1B	hsa-miR-1260a
EIF4E1B	hsa-miR-1284
EIF4E1B	hsa-miR-2483
EIF4E1B	hsa-miR-1266-5p
EIF4E1B	hsa-miR-1777
EIF4E1B	hsa-miR-3120-5p
EIF4E1B	hsa-miR-8827
EIF4E1B	hsa-miR-2454-3p
EIF4E1B	hsa-miR-6852-5p
EIF4E1B	hsa-miR-15-5p
EIF4E1B	hsa-miR-2284ab
EIF4E1B	hsa-miR-8804
EIF4E1B	hsa-miR-670-3p
EIF4E1B	hsa-miR-30e-3p
EIF4E1B	hsa-miR-873-5p
EIF4E1B	hsa-miR-346-5p
EIF4E1B	hsa-miR-4731-5p
EIF4E1B	hsa-miR-939-5p
EIF4E1B	hsa-miR-6895-3p
EIF4E1B	hsa-miR-1266
EIF4E1B	hsa-miR-6766-5p
EIF4E1B	hsa-miR-4433b-5p
EIF4E1B	hsa-miR-3076-5p
EIF4E1B	hsa-miR-1841
EIF4E1B	hsa-miR-3614-5p
EIF4E1B	hsa-miR-6841-3p
EIF4E1B	hsa-miR-548ap-5p
EIF4E1B	hsa-miR-3659
EIF4E1B	hsa-miR-1199-5p
EIF4E1B	hsa-miR-1241
EIF4E1B	hsa-miR-761
EIF4E1B	hsa-miR-939-5p
EIF4E1B	hsa-miR-6770-3p
EIF4E1B	hsa-miR-7094-1-5p
EIF4E1B	hsa-miR-4511

EIF4E1B	hsa-miR-1584-3p
EIF4E1B	hsa-miR-188-3p
EIF4E1B	hsa-miR-9768-3p
EIF4E1B	hsa-miR-349
EIF4E1B	hsa-miR-1224-3p
EIF4E1B	hsa-miR-545-3p
EIF4E1B	hsa-miR-2300-5p
EIF4E1B	hsa-miR-4717-5p
EIF4E1B	hsa-miR-6876-3p
EIF4E1B	hsa-miR-8850
EIF4E1B	hsa-miR-6888-3p
EIF4E1B	hsa-miR-1930-3p
EIF4E1B	hsa-miR-2284n
EIF4E1B	hsa-miR-940
EIF4E1B	hsa-miR-494
EIF4E1B	hsa-miR-6990-3p
EIF4E1B	hsa-miR-7068-5p
EIF4E1B	hsa-miR-6734-3p
EIF4E1B	hsa-miR-1225-5p
EIF4E1B	hsa-miR-6742-3p
EIF4E1B	hsa-miR-3922-5p
EIF4E1B	hsa-miR-146
EIF4E1B	hsa-miR-455-3p
EIF4E1B	hsa-miR-378-3p
EIF4E1B	hsa-miR-4715-3p
EIF4E1B	hsa-miR-338-3p
EIF4E1B	hsa-miR-4266
EIF4E1B	hsa-miR-302a-3p
EIF4E1B	hsa-miR-409a
EIF4E1B	hsa-miR-370
EIF4E1B	hsa-miR-2373-3p
EIF4E1B	hsa-miR-7177-3p
EIF4E1B	hsa-miR-2334
EIF4E1B	hsa-miR-1-3p
EIF4E1B	hsa-miR-2284k
EIF4E1B	hsa-miR-4258
EIF4E1B	hsa-miR-6089
EIF4E1B	hsa-miR-2343
EIF4E1B	hsa-miR-361-5p
EIF4E1B	hsa-miR-7855-5p
EIF4E1B	hsa-miR-8089
EIF4E1B	hsa-miR-3189-5p
EIF4E1B	hsa-miR-4695-5p
EIF4E1B	hsa-miR-622
EIF4E1B	hsa-miR-7035-3p
EIF4E1B	hsa-miR-4708-3p

EIF4E1B	hsa-miR-203
EIF4E1B	hsa-miR-21-5p
EIF4E1B	hsa-miR-6077
EIF4E1B	hsa-miR-3192-3p
EIF4E1B	hsa-miR-5000-3p
EIF4E1B	hsa-miR-3915
EIF4E1B	hsa-miR-4710
EIF4E1B	hsa-miR-548am-5p
EIF4E1B	hsa-miR-7976
EIF4E1B	hsa-miR-3154
EIF4E1B	hsa-miR-494-3p
EIF4E1B	hsa-miR-6809-5p
EIF4E1B	hsa-miR-3577-3p
EIF4E1B	hsa-miR-3929
EIF4E1B	hsa-miR-548b-3p
EIF4E1B	hsa-miR-1207-3p
EIF4E1B	hsa-miR-7-5p
EIF4E1B	hsa-miR-545-3p
EIF4E1B	hsa-miR-194-3p
EIF4E1B	hsa-miR-4792
EIF4E1B	hsa-miR-5004-5p
EIF4E1B	hsa-miR-200a-3p
EIF4E1B	hsa-miR-660-3p
EIF4E1B	hsa-miR-548w
EIF4E1B	hsa-miR-1291
EIF4E1B	hsa-miR-760-5p
EIF4E1B	hsa-miR-627-5p
EIF4E1B	hsa-miR-1249-3p
EIF4E1B	hsa-miR-1910-5p
EIF4E1B	hsa-miR-4649-3p
EIF4E1B	hsa-miR-548d-5p
EIF4E1B	hsa-miR-3664-3p
EIF4E1B	hsa-miR-7201-3p
EIF4E1B	hsa-miR-24-3p
EIF4E1B	hsa-miR-7327-5p
EIF4E1B	hsa-miR-6840-3p
EIF4E1B	hsa-miR-6090
EIF4E1B	hsa-miR-4323
EIF4E1B	hsa-miR-2319
EIF4E1B	hsa-miR-548h-5p
EIF4E1B	hsa-miR-195-5p
EIF4E1B	hsa-miR-483-3p
EIF4E1B	hsa-miR-2326
EIF4E1B	hsa-miR-4316
EIF4E1B	hsa-miR-352
EIF4E1B	hsa-miR-2957

EIF4E1B	hsa-miR-2284d
EIF4E1B	hsa-miR-5002-3p
EIF4E1B	hsa-miR-4640-3p
EIF4E1B	hsa-miR-3966
EIF4E1B	hsa-miR-125-5p
EIF4E1B	hsa-miR-2284v
EIF4E1B	hsa-miR-370-5p
EIF4E1B	hsa-miR-3664-5p
EIF4E1B	hsa-miR-214-3p
EIF4E1B	hsa-miR-4780
EIF4E1B	hsa-miR-6776-5p
EIF4E1B	hsa-miR-2894
EIF4E1B	hsa-miR-4516
EIF4E1B	hsa-miR-1306-5p
EIF4E1B	hsa-miR-143-5p
EIF4E1B	hsa-miR-6858-5p
EIF4E1B	hsa-miR-7157-3p
EIF4E1B	hsa-miR-422
EIF4E1B	hsa-miR-3115
EIF4E1B	hsa-miR-548ad-5p
EIF4E1B	hsa-miR-4264
EIF4E1B	hsa-miR-324-5p
EIF4E1B	hsa-miR-3973
EIF4E1B	hsa-miR-376c-3p
EIF4E1B	hsa-miR-338-3p
EIF4E1B	hsa-miR-1266-3p
EIF4E1B	hsa-miR-8903
EIF4E1B	hsa-miR-665
EIF4E1B	hsa-miR-18-3p
EIF4E1B	hsa-miR-141-3p
EIF4E1B	hsa-miR-208-5p
EIF4E1B	hsa-miR-601
EIF4E1B	hsa-miR-186-3p
EIF4E1B	hsa-miR-5627-5p
EIF4E1B	hsa-miR-561-3p
EIF4E1B	hsa-miR-7169-5p
EIF4E1B	hsa-miR-6862-3p
EIF4E1B	hsa-miR-4720-3p
EIF4E1B	hsa-miR-7191-3p
EIF4E1B	hsa-miR-33-3p
EIF4E1B	hsa-miR-148-3p
EIF4E1B	hsa-miR-3151
EIF4E1B	hsa-miR-361-3p
EIF4E1B	hsa-miR-4766-3p
EIF4E1B	hsa-miR-7386m-3p
EIF4E1B	hsa-miR-6893-5p

EIF4E1B	hsa-miR-548aq-5p
EIF4E1B	hsa-miR-149-5p
EIF4E1B	hsa-miR-2284-5p
EIF4E1B	hsa-miR-497-5p
EIF4E1B	hsa-miR-4795-3p
EIF4E1B	hsa-miR-4433a-3p
EIF4E1B	hsa-miR-2284-3p
EIF4E1B	hsa-miR-938
EIF4E1B	hsa-miR-629-5p
EIF4E1B	hsa-miR-4311
EIF4E1B	hsa-miR-1207
EIF4E1B	hsa-miR-2389
EIF4E1B	hsa-miR-5587-5p
EIF4E1B	hsa-miR-4689
EIF4E1B	hsa-miR-3110-5p
EIF4E1B	hsa-miR-629-3p
EIF4E1B	hsa-miR-516-3p
EIF4E1B	hsa-miR-206
EIF4E1B	hsa-miR-10-5p
EIF4E1B	hsa-miR-3620-3p
EIF4E1B	hsa-miR-589-5p
EIF4E1B	hsa-miR-181a-2-3p
EIF4E1B	hsa-miR-322-5p
EIF4E1B	hsa-miR-7386k-3p
EIF4E1B	hsa-miR-3975
EIF4E1B	hsa-miR-1276
EIF4E1B	hsa-miR-6944-5p
EIF4E1B	hsa-miR-6393
EIF4E1B	hsa-miR-33-5p
EIF4E1B	hsa-miR-3151-3p
EIF4E1B	hsa-miR-4434
EIF4E1B	hsa-miR-182-5p
EIF4E1B	hsa-miR-2355-5p
EIF4E1B	hsa-miR-626
EIF4E1B	hsa-miR-3922-3p
EIF4E1B	hsa-miR-302-3p
EIF4E1B	hsa-miR-654-3p
EIF4E1B	hsa-miR-27a-5p
EIF4E1B	hsa-miR-548ay-5p
EIF4E1B	hsa-miR-7171-3p
EIF4E1B	hsa-miR-6745
EIF4E1B	hsa-miR-188-5p
EIF4E1B	hsa-miR-3106-3p
EIF4E1B	hsa-miR-1914-5p
EIF4E1B	hsa-miR-2276-5p
EIF4E1B	hsa-miR-2284c

EIF4E1B	hsa-miR-197-3p
EIF4E1B	hsa-miR-6813-3p
EIF4E1B	hsa-miR-5699-5p
EIF4E1B	hsa-miR-7231-3p
EIF4E1B	hsa-miR-3175
EIF4E1B	hsa-miR-1324
EIF4E1B	hsa-miR-548b-3p
EIF4E1B	hsa-miR-520-5p
EIF4E1B	hsa-miR-362-3p
EIF4E1B	hsa-miR-6889-3p
EIF4E1B	hsa-miR-525-5p
EIF4E1B	hsa-miR-452-5p
EIF4E1B	hsa-miR-2368-3p
EIF4E1B	hsa-miR-708-5p
EIF4E1B	hsa-miR-4742-5p
EIF4E1B	hsa-miR-7063-5p
EIF4E1B	hsa-miR-702-3p
EIF4E1B	hsa-miR-4478
EIF4E1B	hsa-miR-6770-5p
EIF4E1B	hsa-miR-31-5p
EIF4E1B	hsa-miR-6729-3p
EIF4E1B	hsa-miR-548bb-5p
EIF4E1B	hsa-miR-4467
EIF4E1B	hsa-miR-548ae-5p
EIF4E1B	hsa-miR-1955-3p
EIF4E1B	hsa-miR-1258-5p
EIF4E1B	hsa-miR-7108-3p
EIF4E1B	hsa-miR-1233-3p
EIF4E1B	hsa-miR-7166-5p
EIF4E1B	hsa-miR-7664-5p
EIF4E1B	hsa-miR-1287-3p
EIF4E1B	hsa-miR-2439-5p
EIF4E1B	hsa-miR-6886-3p
EIF4E1B	hsa-miR-3059-5p
EIF4E1B	hsa-miR-548b-5p
EIF4E1B	hsa-miR-6763-3p
EIF4E1B	hsa-miR-642a-5p
EIF4E1B	hsa-miR-4667
EIF4E1B	hsa-miR-6818-3p
EIF4E1B	hsa-miR-6839-3p
EIF4E1B	hsa-miR-548
EIF4E1B	hsa-miR-663b
EIF4E1B	hsa-miR-499-5p
EIF4E1B	hsa-miR-545-5p
EIF4E1B	hsa-miR-548as-5p
EIF4E1B	hsa-miR-3075-3p

EIF4E1B	hsa-miR-9-5p
EIF4E1B	hsa-miR-4485-5p
EIF4E1B	hsa-miR-873-5p.1
EIF4E1B	hsa-miR-548y
EIF4E1B	hsa-miR-6503-3p
EIF4E1B	hsa-miR-4436b-5p
EIF4E1B	hsa-miR-1255b-2-3p
EIF4E1B	hsa-miR-6737-3p
EIF4E1B	hsa-miR-2466-3p
EIF4E1B	hsa-miR-766-3p
EIF4E1B	hsa-miR-6769a-3p
EIF4E1B	hsa-miR-100-3p
EIF4E1B	hsa-miR-6529-3p
EIF4E1B	hsa-miR-1306-3p
EIF4E1B	hsa-miR-6912-5p
EIF4E1B	hsa-miR-3190-5p
EIF4E1B	hsa-miR-873-5p
EIF4E1B	hsa-miR-6356
EIF4E1B	hsa-miR-5008-3p
EIF4E1B	hsa-miR-670
EIF4E1B	hsa-miR-186-5p
EIF4E1B	hsa-miR-7033-3p
EIF4E1B	hsa-miR-7657-3p
EIF4E1B	hsa-miR-140-3p.1
EIF4E1B	hsa-miR-4261
EIF4E1B	hsa-miR-7179-3p
EIF4E1B	hsa-miR-662
EIF4E1B	hsa-miR-6856-3p
EIF4E1B	hsa-miR-7197-3p
EIF4E1B	hsa-miR-125
EIF4E1B	hsa-miR-5092
EIF4E1B	hsa-miR-33
EIF4E1B	hsa-miR-2284j
EIF4E1B	hsa-miR-7364-5p
EIF4E1B	hsa-miR-4804-3p
EIF4E1B	hsa-miR-4797-3p
EIF4E1B	hsa-miR-8908-5p
EIF4E1B	hsa-miR-4999-3p
EIF4E1B	hsa-miR-8836
EIF4E1B	hsa-miR-6807-5p
EIF4E1B	hsa-miR-1343-3p
EIF4E1B	hsa-miR-4700-5p
EIF4E1B	hsa-miR-125a-3p
EIF4E1B	hsa-miR-1343
EIF4E1B	hsa-miR-8805
EIF4E1B	hsa-miR-548c-5p

EIF4E1B	hsa-miR-7110-3p
EIF4E1B	hsa-miR-300-5p
EIF4E1B	hsa-miR-593-3p
EIF4E1B	hsa-miR-544b
EIF4E1B	hsa-miR-6500-3p
EIF4E1B	hsa-miR-4687-5p
EIF4E1B	hsa-miR-559
EIF4E1B	hsa-miR-4768-3p
EIF4E1B	hsa-miR-3680-3p
EIF4E1B	hsa-miR-7108-5p
EIF4E1B	hsa-miR-5125
EIF4E1B	hsa-miR-548au-5p
EIF4E1B	hsa-miR-212-5p
EIF4E1B	hsa-miR-1296-5p
EIF4E1B	hsa-miR-3179
EIF4E1B	hsa-miR-3167-5p
EIF4E1B	hsa-miR-7-5p
EIF4E1B	hsa-miR-8834b
EIF4E1B	hsa-miR-4459
EIF4E1B	hsa-miR-759
EIF4E1B	hsa-miR-7152-5p
EIF4E1B	hsa-miR-642
EIF4E1B	hsa-miR-583
EIF4E1B	hsa-miR-6892-3p
EIF4E1B	hsa-miR-5007-5p
EIF4E1B	hsa-miR-186-5p
EIF4E1B	hsa-miR-197-5p
EIF4E1B	hsa-miR-1233-3p
EIF4E1B	hsa-miR-193-3p
EIF4E1B	hsa-miR-8901
EIF4E1B	hsa-miR-2433
EIF4E1B	hsa-miR-7656-3p
EIF4E1B	hsa-miR-6779-3p
EIF4E1B	hsa-miR-2284m
EIF4E1B	hsa-miR-3188
EIF4E1B	hsa-miR-1225-3p
EIF4E1B	hsa-miR-378
EIF4E1B	hsa-miR-7646-5p
EIF4E1B	hsa-miR-6802-3p
EIF4E1B	hsa-miR-3120-3p
EIF4E1B	hsa-miR-6808-5p
EIF4E1B	hsa-miR-2327
EIF4E1B	hsa-miR-6878-5p
EIF4E1B	hsa-miR-1962
EIF4E1B	hsa-miR-7069-3p
EIF4E1B	hsa-miR-4433a-5p

EIF4E1B	hsa-miR-7167-5p
EIF4E1B	hsa-miR-548a-5p
EIF4E1B	hsa-miR-5693
EIF4E1B	hsa-miR-6958-3p
EIF4E1B	hsa-miR-2306
EIF4E1B	hsa-miR-499b-5p
EIF4E1B	hsa-miR-329
EIF4E1B	hsa-miR-370-3p
EIF4E1B	hsa-miR-8832
EIF4E1B	hsa-miR-363-5p
EIF4E1B	hsa-miR-548o-5p
EIF4E1B	hsa-miR-2467-5p
EIF4E1B	hsa-miR-384
EIF4E1B	hsa-miR-7224-3p
EIF4E1B	hsa-miR-4667-3p
EIF4E1B	hsa-miR-6793-3p
EIF4E1B	hsa-miR-4419b
EIF4E1B	hsa-miR-4751
EIF4E1B	hsa-miR-455-5p
EIF4E1B	hsa-miR-548ab
EIF4E1B	hsa-miR-4290
EIF4E1B	hsa-miR-3943
EIF4E1B	hsa-miR-30-5p
EIF4E1B	hsa-miR-539-5p
EIF4E1B	hsa-miR-325
EIF4E1B	hsa-miR-8905
EIF4E1B	hsa-miR-4779
EIF4E1B	hsa-miR-1972
EIF4E1B	hsa-miR-548ar-5p
EIF4E1B	hsa-miR-181b-2-3p
EIF4E1B	hsa-miR-879-5p
EIF4E1B	hsa-miR-939-3p
EIF4E1B	hsa-miR-674-5p
EIF4E1B	hsa-miR-1827
EIF4E1B	hsa-miR-16-5p
EIF4E1B	hsa-miR-128-3p
EIF4E1B	hsa-miR-4446-3p
EIF4E1B	hsa-miR-3692-3p
EIF4E1B	hsa-miR-3140-3p
EIF4E1B	hsa-miR-548i
EIF4E1B	hsa-miR-22-3p
EIF4E1B	hsa-miR-548ak
EIF4E1B	hsa-miR-7188-5p
EIF4E1B	hsa-miR-1224-5p
EIF4E1B	hsa-miR-5703
EIF4E1B	hsa-miR-6756-5p

EIF4E1B	hsa-miR-502-5p
EIF4E1B	hsa-miR-645
EIF4E1B	hsa-miR-593-3p
EIF4E1B	hsa-miR-2285
EIF4E1B	hsa-miR-6798-3p
EIF4E1B	hsa-miR-2373-5p
EIF4E1B	hsa-miR-1343-5p
EIF4E1B	hsa-miR-6775-3p
EIF4E1B	hsa-miR-200a-3p
EIF4E1B	hsa-miR-6865-3p
EIF4E1B	hsa-miR-3150a-3p
EIF4E1B	hsa-miR-5627-3p
EIF4E2	hsa-miR-7270-3p
EIF4E2	hsa-miR-218a-1-3p
EIF4E2	hsa-miR-3559-3p
EIF4E2	hsa-miR-302a-3p
EIF4E2	hsa-miR-8079
EIF4E2	hsa-miR-7323-3p
EIF4E2	hsa-miR-7243-3p
EIF4E2	hsa-miR-8799
EIF4E2	hsa-miR-6970-3p
EIF4E2	hsa-miR-3912-5p
EIF4E2	hsa-miR-7177-5p
EIF4E2	hsa-miR-7039-3p
EIF4E2	hsa-miR-374-3p
EIF4E2	hsa-miR-6786-3p
EIF4E2	hsa-miR-6780a-3p
EIF4E2	hsa-miR-1266-3p
EIF4E2	hsa-miR-2471-5p
EIF4E2	hsa-miR-295-5p
EIF4E2	hsa-miR-1190
EIF4E2	hsa-miR-6994-5p
EIF4E2	hsa-miR-3067-5p
EIF4E2	hsa-miR-20-3p
EIF4E2	hsa-miR-302c-5p
EIF4E2	hsa-miR-3154
EIF4E2	hsa-miR-540-5p
EIF4E2	hsa-miR-185-3p
EIF4E2	hsa-miR-7077-3p
EIF4E2	hsa-miR-29c-3p
EIF4E2	hsa-miR-3655
EIF4E2	hsa-miR-193-5p
EIF4E2	hsa-miR-758-3p
EIF4E2	hsa-miR-877-3p
EIF4E2	hsa-miR-589-3p
EIF4E2	hsa-miR-1796

EIF4E2	hsa-miR-1207-3p
EIF4E2	hsa-miR-218-1-3p
EIF4E2	hsa-miR-292b-5p
EIF4E2	hsa-miR-1912-3p
EIF4E2	hsa-miR-20b-3p
EIF4E2	hsa-miR-7222-3p
EIF4E2	hsa-miR-292a-5p
EIF4E2	hsa-miR-7316-5p
EIF4E2	hsa-miR-767
EIF4E2	hsa-miR-6881-3p
EIF4E2	hsa-miR-496b
EIF4E2	hsa-miR-7191-5p
EIF4E2	hsa-miR-8898
EIF4E2	hsa-miR-6971-3p
EIF4E2	hsa-miR-290
EIF4E2	hsa-miR-22-3p
EIF4E2	hsa-miR-7111-3p
EIF4E2	hsa-miR-132-5p
EIF4E2	hsa-miR-7050-3p
EIF4E2	hsa-miR-1207
EIF4E2	hsa-miR-665
EIF4E2	hsa-miR-7641
EIF4E2	hsa-miR-592-5p
EIF4E2	hsa-miR-683
EIF4E2	hsa-miR-6932-3p
EIF4E2	hsa-miR-302a-5p
EIF4E2	hsa-miR-299-5p
EIF4E2	hsa-miR-29b-1-5p
EIF4E2	hsa-miR-29a-3p
EIF4E2	hsa-miR-7048-3p
EIF4E2	hsa-miR-3163
EIF4E2	hsa-miR-184
EIF4E2	hsa-miR-293-5p
EIF4E2	hsa-miR-7682-5p
EIF4E2	hsa-miR-4477a
EIF4E2	hsa-miR-374b-3p
EIF4E2	hsa-miR-7086-3p
EIF4E2	hsa-miR-22-3p
EIF4E2	hsa-miR-9769-3p
EIF4E2	hsa-miR-585-5p
EIF4E2	hsa-miR-3102-3p
EIF4E2	hsa-miR-2325c
EIF4E2	hsa-miR-597
EIF4E2	hsa-miR-3085-5p
EIF4E2	hsa-miR-5000-3p
EIF4E2	hsa-miR-3613-5p

EIF4E2	hsa-miR-449c-3p
EIF4E2	hsa-miR-669p-3p
EIF4E2	hsa-miR-5682
EIF4E2	hsa-miR-7389-5p
EIF4E2	hsa-miR-290a-5p
EIF4E2	hsa-miR-299a-5p
EIF4E2	hsa-miR-665-3p
EIF4E2	hsa-miR-26-3p
EIF4E2	hsa-miR-1236-3p
EIF4E2	hsa-miR-5124a
EIF4E2	hsa-miR-6871-3p
EIF4E2	hsa-miR-224-3p
EIF4E2	hsa-miR-322-3p
EIF4E2	hsa-miR-1245a
EIF4E2	hsa-miR-6961-3p
EIF4E2	hsa-miR-7243-5p
EIF4E2	hsa-miR-8096
EIF4E2	hsa-miR-3059-5p
EIF4E2	hsa-miR-6348
EIF4E2	hsa-miR-292-5p
EIF4E2	hsa-miR-3099-5p
EIF4E2	hsa-miR-29-3p
EIF4E2	hsa-miR-8069
EIF4E2	hsa-miR-7686-5p
EIF4E2	hsa-miR-6928-3p
EIF4E2	hsa-miR-7037-5p
EIF4E2	hsa-miR-6947-3p
EIF4E2	hsa-miR-767-5p
EIF4E2	hsa-miR-3613a
EIF4E2	hsa-miR-4803
EIF4E2	hsa-miR-2116-5p
EIF4E2	hsa-miR-6403
EIF4E2	hsa-miR-1236-3p
EIF4E2	hsa-miR-29
EIF4E2	hsa-miR-4520-5p
EIF4E2	hsa-miR-1245a
EIF4E2	hsa-miR-1956-5p
EIF4E2	hsa-miR-29a-3p
EIF4E2	hsa-miR-294-5p
EIF4E2	hsa-miR-7864
EIF4E2	hsa-miR-7480-5p
EIF4E2	hsa-miR-4729
EIF4E2	hsa-miR-8875
EIF4E2	hsa-miR-543-5p
EIF4E2	hsa-miR-7173-5p
EIF4E2	hsa-miR-1956

EIF4E2	hsa-miR-26b-3p
EIF4E2	hsa-miR-21-3p
EIF4E2	hsa-miR-4695-3p
EIF4E2	hsa-miR-1553-5p
EIF4E2	hsa-miR-1567
EIF4E2	hsa-miR-7646-5p
EIF4E2	hsa-miR-7365-3p
EIF4E2	hsa-miR-3662
EIF4E2	hsa-miR-6787-3p
EIF4E2	hsa-miR-22-5p
EIF4E2	hsa-miR-763
EIF4E2	hsa-miR-198
EIF4E2	hsa-miR-6398
EIF4E2	hsa-miR-29b-3p
EIF4E2	hsa-miR-6958-3p
EIF4E2	hsa-miR-4677-5p
EIF4E2	hsa-miR-627-3p
EIF4E2	hsa-miR-141-5p
EIF4E2	hsa-miR-7032-3p
EIF4E2	hsa-miR-6549-3p
EIF4E3	hsa-miR-19a-3p
EIF4E3	hsa-miR-4437
EIF4E3	hsa-miR-409
EIF4E3	hsa-miR-5697-5p
EIF4E3	hsa-miR-4691-3p
EIF4E3	hsa-miR-7659-5p
EIF4E3	hsa-miR-7398a-3p
EIF4E3	hsa-miR-2450bd
EIF4E3	hsa-miR-8089
EIF4E3	hsa-miR-1744-5p
EIF4E3	hsa-miR-195-3p
EIF4E3	hsa-miR-216b-3p
EIF4E3	hsa-miR-4482-3p
EIF4E3	hsa-miR-548g-5p
EIF4E3	hsa-miR-377-3p
EIF4E3	hsa-miR-891b
EIF4E3	hsa-miR-3075
EIF4E3	hsa-miR-6740-5p
EIF4E3	hsa-miR-1252-5p
EIF4E3	hsa-miR-7308-5p
EIF4E3	hsa-miR-553
EIF4E3	hsa-miR-4700-5p
EIF4E3	hsa-miR-2284b
EIF4E3	hsa-miR-8099
EIF4E3	hsa-miR-6751-3p
EIF4E3	hsa-miR-7050-5p

EIF4E3	hsa-miR-455-5p
EIF4E3	hsa-miR-4261
EIF4E3	hsa-miR-3920
EIF4E3	hsa-miR-6384
EIF4E3	hsa-miR-3908
EIF4E3	hsa-let-7c-3p
EIF4E3	hsa-miR-525-5p
EIF4E3	hsa-miR-8826
EIF4E3	hsa-miR-6876-5p
EIF4E3	hsa-miR-3622a-3p
EIF4E3	hsa-miR-608
EIF4E3	hsa-miR-548b-5p
EIF4E3	hsa-miR-18-5p
EIF4E3	hsa-miR-6508-3p
EIF4E3	hsa-miR-365a-3p
EIF4E3	hsa-miR-6779-5p
EIF4E3	hsa-miR-606
EIF4E3	hsa-miR-4524b-3p
EIF4E3	hsa-miR-4526
EIF4E3	hsa-miR-7183-5p
EIF4E3	hsa-miR-548au-5p
EIF4E3	hsa-miR-216-5p
EIF4E3	hsa-miR-1682
EIF4E3	hsa-miR-4308
EIF4E3	hsa-miR-1954
EIF4E3	hsa-miR-1178-3p
EIF4E3	hsa-miR-4637
EIF4E3	hsa-miR-3135b
EIF4E3	hsa-miR-103-2-5p
EIF4E3	hsa-miR-1814a
EIF4E3	hsa-miR-6120-5p
EIF4E3	hsa-miR-448-5p
EIF4E3	hsa-miR-6372
EIF4E3	hsa-miR-204-5p
EIF4E3	hsa-miR-4472
EIF4E3	hsa-miR-6999-5p
EIF4E3	hsa-miR-654-3p
EIF4E3	hsa-miR-6871-5p
EIF4E3	hsa-miR-3144-3p
EIF4E3	hsa-miR-362-5p
EIF4E3	hsa-miR-7270-3p
EIF4E3	hsa-miR-7398d-3p
EIF4E3	hsa-miR-3564
EIF4E3	hsa-miR-3122
EIF4E3	hsa-miR-7157-5p
EIF4E3	hsa-miR-5683

EIF4E3	hsa-miR-7175-3p
EIF4E3	hsa-miR-16-1-3p
EIF4E3	hsa-miR-3533
EIF4E3	hsa-miR-1955-5p
EIF4E3	hsa-miR-2411-3p
EIF4E3	hsa-miR-211-5p
EIF4E3	hsa-miR-6801-3p
EIF4E3	hsa-miR-548ar-5p
EIF4E3	hsa-miR-548ah-5p
EIF4E3	hsa-miR-5190
EIF4E3	hsa-miR-1649-5p
EIF4E3	hsa-miR-7069-5p
EIF4E3	hsa-miR-224-3p
EIF4E3	hsa-miR-6845-3p
EIF4E3	hsa-miR-1934-3p
EIF4E3	hsa-miR-2459
EIF4E3	hsa-miR-365b-3p
EIF4E3	hsa-miR-7062-5p
EIF4E3	hsa-miR-6504-3p
EIF4E3	hsa-miR-378d
EIF4E3	hsa-miR-6324
EIF4E3	hsa-miR-2285
EIF4E3	hsa-miR-6757-5p
EIF4E3	hsa-miR-4480
EIF4E3	hsa-miR-2399-3p
EIF4E3	hsa-miR-29b-3p
EIF4E3	hsa-miR-7300-5p
EIF4E3	hsa-miR-219a-2-3p
EIF4E3	hsa-miR-4454
EIF4E3	hsa-miR-1179
EIF4E3	hsa-miR-148
EIF4E3	hsa-miR-7058-5p
EIF4E3	hsa-miR-24-3p
EIF4E3	hsa-miR-599-3p
EIF4E3	hsa-miR-544-3p
EIF4E3	hsa-miR-290b-5p
EIF4E3	hsa-miR-7374c-3p
EIF4E3	hsa-miR-660-5p
EIF4E3	hsa-miR-3936
EIF4E3	hsa-miR-494
EIF4E3	hsa-miR-5696
EIF4E3	hsa-miR-6879-5p
EIF4E3	hsa-miR-24-5p
EIF4E3	hsa-miR-7237-5p
EIF4E3	hsa-miR-4460
EIF4E3	hsa-miR-6338

EIF4E3	hsa-miR-1696
EIF4E3	hsa-miR-328-3p
EIF4E3	hsa-miR-4291
EIF4E3	hsa-miR-6886-3p
EIF4E3	hsa-miR-4314
EIF4E3	hsa-miR-4752
EIF4E3	hsa-miR-2354
EIF4E3	hsa-miR-8085
EIF4E3	hsa-miR-22-5p
EIF4E3	hsa-miR-887
EIF4E3	hsa-miR-3562
EIF4E3	hsa-miR-6849-3p
EIF4E3	hsa-miR-4280
EIF4E3	hsa-miR-339-5p
EIF4E3	hsa-miR-703
EIF4E3	hsa-miR-378-5p
EIF4E3	hsa-miR-7026-5p
EIF4E3	hsa-miR-2285x
EIF4E3	hsa-miR-7337-5p
EIF4E3	hsa-miR-3531-3p
EIF4E3	hsa-miR-6976-3p
EIF4E3	hsa-miR-7065-3p
EIF4E3	hsa-miR-7359-3p
EIF4E3	hsa-miR-8805
EIF4E3	hsa-miR-135-5p
EIF4E3	hsa-miR-616-3p
EIF4E3	hsa-miR-615-3p
EIF4E3	hsa-miR-2284r
EIF4E3	hsa-miR-6505-3p
EIF4E3	hsa-miR-547-5p
EIF4E3	hsa-miR-4425
EIF4E3	hsa-miR-6398
EIF4E3	hsa-miR-6798-5p
EIF4E3	hsa-miR-129-3p
EIF4E3	hsa-miR-1680-5p
EIF4E3	hsa-miR-4799-3p
EIF4E3	hsa-miR-5110
EIF4E3	hsa-miR-5571-5p
EIF4E3	hsa-miR-128-5p
EIF4E3	hsa-miR-4705
EIF4E3	hsa-miR-556-5p
EIF4E3	hsa-miR-7156-3p
EIF4E3	hsa-miR-302
EIF4E3	hsa-miR-7172-3p
EIF4E3	hsa-miR-7116-3p
EIF4E3	hsa-miR-6811-3p

EIF4E3	hsa-miR-3928-3p
EIF4E3	hsa-miR-6973a-3p
EIF4E3	hsa-miR-4686
EIF4E3	hsa-miR-1970
EIF4E3	hsa-miR-1949
EIF4E3	hsa-miR-25-5p
EIF4E3	hsa-miR-8086
EIF4E3	hsa-miR-802
EIF4E3	hsa-miR-4742-3p
EIF4E3	hsa-miR-873a-3p
EIF4E3	hsa-miR-5120
EIF4E3	hsa-let-7c-1-3p
EIF4E3	hsa-miR-130-3p
EIF4E3	hsa-miR-1296-3p
EIF4E3	hsa-miR-6929-5p
EIF4E3	hsa-miR-549a
EIF4E3	hsa-miR-7015-3p
EIF4E3	hsa-miR-487-5p
EIF4E3	hsa-miR-2479
EIF4E3	hsa-miR-298-5p
EIF4E3	hsa-miR-361-3p
EIF4E3	hsa-miR-1976
EIF4E3	hsa-let-7ag-3p
EIF4E3	hsa-miR-532-3p
EIF4E3	hsa-miR-7118-5p
EIF4E3	hsa-miR-7202-3p
EIF4E3	hsa-miR-30
EIF4E3	hsa-miR-513a-5p
EIF4E3	hsa-miR-329
EIF4E3	hsa-miR-1560-3p
EIF4E3	hsa-miR-4762-3p
EIF4E3	hsa-miR-3136-5p
EIF4E3	hsa-miR-424-5p
EIF4E3	hsa-miR-1915-3p
EIF4E3	hsa-miR-2452
EIF4E3	hsa-miR-2461-3p
EIF4E3	hsa-miR-3167-3p
EIF4E3	hsa-miR-187-5p
EIF4E3	hsa-miR-8844
EIF4E3	hsa-miR-875
EIF4E3	hsa-miR-197-3p
EIF4E3	hsa-miR-4292
EIF4E3	hsa-miR-374a-3p
EIF4E3	hsa-miR-210-5p
EIF4E3	hsa-miR-5128
EIF4E3	hsa-miR-423-5p

EIF4E3	hsa-miR-7311-5p
EIF4E3	hsa-miR-1-3p
EIF4E3	hsa-miR-465abc-3p
EIF4E3	hsa-miR-4650
EIF4E3	hsa-miR-8889
EIF4E3	hsa-miR-6393
EIF4E3	hsa-miR-129-2-3p
EIF4E3	hsa-miR-1197
EIF4E3	hsa-miR-4509
EIF4E3	hsa-miR-3681-5p
EIF4E3	hsa-miR-350
EIF4E3	hsa-miR-5125
EIF4E3	hsa-miR-8076
EIF4E3	hsa-miR-380-3p
EIF4E3	hsa-miR-7302-5p
EIF4E3	hsa-miR-217
EIF4E3	hsa-miR-345-5p
EIF4E3	hsa-miR-3557-3p
EIF4E3	hsa-miR-7252-3p
EIF4E3	hsa-miR-135a-1-3p
EIF4E3	hsa-miR-2285g
EIF4E3	hsa-miR-7358-3p
EIF4E3	hsa-miR-7235-3p
EIF4E3	hsa-miR-520-3p
EIF4E3	hsa-miR-879-5p
EIF4E3	hsa-miR-590-5p
EIF4E3	hsa-miR-4796-5p
EIF4E3	hsa-miR-4647
EIF4E3	hsa-miR-7046-5p
EIF4E3	hsa-miR-204-3p
EIF4E3	hsa-miR-4287
EIF4E3	hsa-miR-5094
EIF4E3	hsa-miR-3686
EIF4E3	hsa-miR-3653-3p
EIF4E3	hsa-miR-23a-3p
EIF4E3	hsa-miR-2277-3p
EIF4E3	hsa-miR-2292
EIF4E3	hsa-miR-1238-3p
EIF4E3	hsa-miR-3074-5p
EIF4E3	hsa-miR-1264
EIF4E3	hsa-miR-29b-1-5p
EIF4E3	hsa-miR-493-5p
EIF4E3	hsa-miR-3613-3p
EIF4E3	hsa-miR-5697-3p
EIF4E3	hsa-miR-488-3p
EIF4E3	hsa-miR-7067-5p

EIF4E3	hsa-miR-345-3p
EIF4E3	hsa-miR-7249-3p
EIF4E3	hsa-miR-148b-3p
EIF4E3	hsa-miR-513a
EIF4E3	hsa-miR-6356
EIF4E3	hsa-miR-688
EIF4E3	hsa-miR-6715b-5p
EIF4E3	hsa-miR-7330-5p
EIF4E3	hsa-miR-1574-5p
EIF4E3	hsa-miR-656-5p
EIF4E3	hsa-miR-342-3p
EIF4E3	hsa-miR-1246
EIF4E3	hsa-miR-3082-5p
EIF4E3	hsa-miR-449-3p
EIF4E3	hsa-miR-3141
EIF4E3	hsa-miR-1545a-3p
EIF4E3	hsa-miR-486-5p
EIF4E3	hsa-miR-618
EIF4E3	hsa-miR-6481
EIF4E3	hsa-miR-2343
EIF4E3	hsa-miR-635
EIF4E3	hsa-miR-2284q
EIF4E3	hsa-miR-5132-3p
EIF4E3	hsa-miR-6380
EIF4E3	hsa-miR-6757-3p
EIF4E3	hsa-miR-450a
EIF4E3	hsa-miR-200c-3p
EIF4E3	hsa-miR-154-5p
EIF4E3	hsa-miR-1758
EIF4E3	hsa-miR-3154
EIF4E3	hsa-miR-1912-3p
EIF4E3	hsa-miR-6942-5p
EIF4E3	hsa-miR-5681b
EIF4E3	hsa-miR-7014-5p
EIF4E3	hsa-miR-3473be
EIF4E3	hsa-miR-506-5p
EIF4E3	hsa-miR-30--3p
EIF4E3	hsa-miR-2329-5p
EIF4E3	hsa-miR-197-5p
EIF4E3	hsa-miR-496b
EIF4E3	hsa-miR-660-5p
EIF4E3	hsa-miR-7048-3p
EIF4E3	hsa-miR-200c-3p
EIF4E3	hsa-miR-2483-3p
EIF4E3	hsa-miR-7385f-3p
EIF4E3	hsa-miR-879-3p

EIF4E3	hsa-miR-2316
EIF4E3	hsa-miR-4301
EIF4E3	hsa-miR-625-5p
EIF4E3	hsa-miR-669p-3p
EIF4E3	hsa-miR-7178-5p
EIF4E3	hsa-miR-4735-5p
EIF4E3	hsa-miR-719
EIF4E3	hsa-miR-2395
EIF4E3	hsa-miR-3591-5p
EIF4E3	hsa-miR-3942-3p
EIF4E3	hsa-miR-669g
EIF4E3	hsa-miR-3573-5p
EIF4E3	hsa-miR-622
EIF4E3	hsa-miR-6918-3p
EIF4E3	hsa-miR-2394
EIF4E3	hsa-miR-8888
EIF4E3	hsa-miR-627-3p
EIF4E3	hsa-miR-297bc-3p
EIF4E3	hsa-miR-527
EIF4E3	hsa-miR-6937-3p
EIF4E3	hsa-miR-2285j
EIF4E3	hsa-miR-548u
EIF4E3	hsa-miR-7194-5p
EIF4E3	hsa-miR-4514
EIF4E3	hsa-miR-6934-3p
EIF4E3	hsa-miR-548ap-3p
EIF4E3	hsa-miR-7253-5p
EIF4E3	hsa-miR-1911-3p
EIF4E3	hsa-miR-544b
EIF4E3	hsa-miR-138-2-3p
EIF4E3	hsa-miR-376c-5p
EIF4E3	hsa-miR-7369-5p
EIF4E3	hsa-miR-7386o-3p
EIF4E3	hsa-miR-548ay-5p
EIF4E3	hsa-miR-330-3p.2
EIF4E3	hsa-miR-548s
EIF4E3	hsa-miR-7349-3p
EIF4E3	hsa-miR-133
EIF4E3	hsa-miR-329-3p
EIF4E3	hsa-miR-7111-3p
EIF4E3	hsa-miR-30a-3p
EIF4E3	hsa-miR-292-3p
EIF4E3	hsa-miR-3938-5p
EIF4E3	hsa-miR-5703
EIF4E3	hsa-miR-7861
EIF4E3	hsa-miR-609

EIF4E3	hsa-miR-145-5p
EIF4E3	hsa-miR-6885-3p
EIF4E3	hsa-miR-7660-3p
EIF4E3	hsa-miR-6732-3p
EIF4E3	hsa-miR-6844
EIF4E3	hsa-miR-2957
EIF4E3	hsa-miR-1598
EIF4E3	hsa-miR-8886
EIF4E3	hsa-miR-3138
EIF4E3	hsa-miR-4692
EIF4E3	hsa-miR-8075
EIF4E3	hsa-miR-1243-3p
EIF4E3	hsa-miR-460b-5p
EIF4E3	hsa-miR-4713-3p
EIF4E3	hsa-miR-1608
EIF4E3	hsa-miR-7864
EIF4E3	hsa-miR-556-3p
EIF4E3	hsa-miR-6325
EIF4E3	hsa-miR-7223-5p
EIF4E3	hsa-miR-4311
EIF4E3	hsa-miR-1968-5p
EIF4E3	hsa-miR-429-3p
EIF4E3	hsa-miR-376ab
EIF4E3	hsa-miR-581
EIF4E3	hsa-miR-1258-5p
EIF4E3	hsa-miR-290a-5p
EIF4E3	hsa-miR-7386e-3p
EIF4E3	hsa-miR-2297
EIF4E3	hsa-miR-2114-5p
EIF4E3	hsa-miR-515
EIF4E3	hsa-miR-2428
EIF4E3	hsa-miR-4753-3p
EIF4E3	hsa-miR-8816
EIF4E3	hsa-miR-2285aa
EIF4E3	hsa-miR-4799-5p
EIF4E3	hsa-miR-502-5p
EIF4E3	hsa-miR-7354-5p
EIF4E3	hsa-miR-6971-3p
EIF4E3	hsa-miR-3070-5p
EIF4E3	hsa-miR-2341
EIF4E3	hsa-miR-7110-3p
EIF4E3	hsa-miR-6988-5p
EIF4E3	hsa-miR-326-5p
EIF4E3	hsa-miR-1185-5p
EIF4E3	hsa-miR-701-3p
EIF4E3	hsa-miR-6778-5p

EIF4E3	hsa-miR-6969-5p
EIF4E3	hsa-miR-1244
EIF4E3	hsa-miR-7371e-5p
EIF4E3	hsa-miR-7072-5p
EIF4E3	hsa-miR-891a
EIF4E3	hsa-miR-4758-3p
EIF4E3	hsa-miR-6976-5p
EIF4E3	hsa-miR-6656-5p
EIF4E3	hsa-miR-3102-5p
EIF4E3	hsa-miR-5003-3p
EIF4E3	hsa-miR-7673-3p
EIF4E3	hsa-let-7a-3p
EIF4E3	hsa-miR-6664-3p
EIF4E3	hsa-miR-543-3p
EIF4E3	hsa-miR-214-3p
EIF4E3	hsa-miR-548aq-3p
EIF4E3	hsa-miR-7398h-3p
EIF4E3	hsa-miR-6954-3p
EIF4E3	hsa-miR-6919-5p
EIF4E3	hsa-miR-593-3p
EIF4E3	hsa-miR-542-5p
EIF4E3	hsa-miR-532-5p
EIF4E3	hsa-miR-5693
EIF4E3	hsa-miR-6828-3p
EIF4E3	hsa-miR-6799-5p
EIF4E3	hsa-miR-4267
EIF4E3	hsa-miR-4297
EIF4E3	hsa-miR-883a-5p
EIF4E3	hsa-miR-203-3p.2
EIF4E3	hsa-miR-410-3p
EIF4E3	hsa-miR-2439-3p
EIF4E3	hsa-miR-638
EIF4E3	hsa-miR-4529-5p
EIF4E3	hsa-miR-7169-3p
EIF4E3	hsa-miR-7232-3p
EIF4E3	hsa-miR-103-5p
EIF4E3	hsa-miR-466f-3p
EIF4E3	hsa-miR-619-5p
EIF4E3	hsa-miR-4271
EIF4E3	hsa-miR-5699-3p
EIF4E3	hsa-miR-29b-2-5p
EIF4E3	hsa-miR-4749-5p
EIF4E3	hsa-miR-6896-5p
EIF4E3	hsa-miR-2484
EIF4E3	hsa-miR-7224-5p
EIF4E3	hsa-miR-1271-3p

EIF4E3	hsa-miR-7g-3p
EIF4E3	hsa-miR-698-5p
EIF4E3	hsa-miR-3152-5p
EIF4E3	hsa-miR-3149
EIF4E3	hsa-miR-8818
EIF4E3	hsa-miR-570-3p
EIF4E3	hsa-miR-3109-3p
EIF4E3	hsa-miR-7371j-5p
EIF4E3	hsa-miR-4433a-3p
EIF4E3	hsa-miR-7119-5p
EIF4E3	hsa-miR-4733-3p
EIF4E3	hsa-miR-6766-5p
EIF4E3	hsa-miR-4664-5p
EIF4E3	hsa-miR-7237-3p
EIF4E3	hsa-miR-1261
EIF4E3	hsa-miR-124-3p
EIF4E3	hsa-miR-2351
EIF4E3	hsa-miR-23a-5p
EIF4E3	hsa-miR-382-3p
EIF4E3	hsa-miR-7b-3p
EIF4E3	hsa-miR-372-5p
EIF4E3	hsa-miR-7260-5p
EIF4E3	hsa-miR-301
EIF4E3	hsa-miR-4744
EIF4E3	hsa-miR-487a
EIF4E3	hsa-miR-4697-3p
EIF4E3	hsa-miR-182-5p
EIF4E3	hsa-miR-2370-3p
EIF4E3	hsa-miR-8087
EIF4E3	hsa-miR-495-3p
EIF4E3	hsa-miR-1250-3p
EIF4E3	hsa-miR-6764-5p
EIF4E3	hsa-miR-3150b-5p
EIF4E3	hsa-miR-24-3p
EIF4E3	hsa-miR-7670-5p
EIF4E3	hsa-miR-3976
EIF4E3	hsa-miR-873-3p
EIF4E3	hsa-miR-8082
EIF4E3	hsa-miR-548c-3p
EIF4E3	hsa-miR-103
EIF4E3	hsa-miR-29-3p
EIF4E3	hsa-miR-101c
EIF4E3	hsa-miR-6215
EIF4E3	hsa-miR-607
EIF4E3	hsa-miR-1257
EIF4E3	hsa-miR-4677-5p

EIF4E3	hsa-miR-4303
EIF4E3	hsa-miR-3966
EIF4E3	hsa-miR-223-3p
EIF4E3	hsa-miR-297
EIF4E3	hsa-miR-7482-3p
EIF4E3	hsa-miR-7323-3p
EIF4E3	hsa-miR-889
EIF4E3	hsa-miR-124
EIF4E3	hsa-miR-203-3p
EIF4E3	hsa-miR-508a
EIF4E3	hsa-miR-496a-3p.2
EIF4E3	hsa-miR-3660
EIF4E3	hsa-miR-598-3p
EIF4E3	hsa-miR-6794-5p
EIF4E3	hsa-miR-7334-3p
EIF4E3	hsa-miR-29-5p
EIF4E3	hsa-miR-296-5p
EIF4E3	hsa-miR-6959-5p
EIF4E3	hsa-miR-4709-5p
EIF4E3	hsa-miR-7207-5p
EIF4E3	hsa-miR-3113-3p
EIF4E3	hsa-miR-6346
EIF4E3	hsa-miR-21-5p
EIF4E3	hsa-miR-7854-3p
EIF4E3	hsa-miR-5089-5p
EIF4E3	hsa-miR-4632-5p
EIF4E3	hsa-miR-6897-5p
EIF4E3	hsa-miR-300-3p
EIF4E3	hsa-miR-2285e
EIF4E3	hsa-miR-2369
EIF4E3	hsa-miR-7653-5p
EIF4E3	hsa-miR-2363
EIF4E3	hsa-miR-493
EIF4E3	hsa-miR-7190-5p
EIF4E3	hsa-miR-455-5p
EIF4E3	hsa-miR-8107
EIF4E3	hsa-miR-4455
EIF4E3	hsa-miR-6884-3p
EIF4E3	hsa-miR-5692bc
EIF4E3	hsa-miR-343
EIF4E3	hsa-miR-6816-3p
EIF4E3	hsa-miR-323b-5p
EIF4E3	hsa-miR-3910
EIF4E3	hsa-miR-7652-3p
EIF4E3	hsa-miR-669m-5p
EIF4E3	hsa-miR-7183-3p

EIF4E3	hsa-miR-466i-3p
EIF4E3	hsa-miR-7658-3p
EIF4E3	hsa-miR-7035-3p
EIF4E3	hsa-miR-570-3p
EIF4E3	hsa-miR-6821-3p
EIF4E3	hsa-miR-6916-5p
EIF4E3	hsa-miR-540-3p
EIF4E3	hsa-miR-7381-3p
EIF4E3	hsa-miR-708-3p
EIF4E3	hsa-miR-2052
EIF4E3	hsa-miR-372-3p
EIF4E3	hsa-miR-6957-5p
EIF4E3	hsa-miR-487a-3p
EIF4E3	hsa-miR-4699-3p
EIF4E3	hsa-miR-7350-3p
EIF4E3	hsa-miR-702-3p
EIF4E3	hsa-miR-650abc
EIF4E3	hsa-miR-7360-3p
EIF4E3	hsa-miR-302b-5p
EIF4E3	hsa-miR-7341-5p
EIF4E3	hsa-miR-7085-5p
EIF4E3	hsa-miR-1590
EIF4E3	hsa-miR-217-5p
EIF4E3	hsa-miR-691
EIF4E3	hsa-miR-6743-5p
EIF4E3	hsa-miR-1193
EIF4E3	hsa-miR-548j-3p
EIF4E3	hsa-miR-7024-5p
EIF4E3	hsa-miR-646
EIF4E3	hsa-miR-874-5p
EIF4E3	hsa-miR-7682-3p
EIF4E3	hsa-miR-8092
EIF4E3	hsa-miR-6374
EIF4E3	hsa-miR-548az-3p
EIF4E3	hsa-miR-8116
EIF4E3	hsa-miR-548ai
EIF4E3	hsa-miR-7287-5p
EIF4E3	hsa-miR-330-5p
EIF4E3	hsa-miR-3121-3p
EIF4E3	hsa-miR-1960
EIF4E3	hsa-miR-660-3p
EIF4E3	hsa-miR-6852-5p
EIF4E3	hsa-miR-4666a-3p
EIF4E3	hsa-miR-7249-5p
EIF4E3	hsa-miR-4259
EIF4E3	hsa-miR-6906-3p

EIF4E3	hsa-miR-6842-5p
EIF4E3	hsa-miR-4502
EIF4E3	hsa-miR-1258
EIF4E3	hsa-miR-6722-3p
EIF4E3	hsa-miR-6781-3p
EIF4E3	hsa-miR-8838
EIF4E3	hsa-miR-152-3p
EIF4E3	hsa-miR-2284l
EIF4E3	hsa-miR-507
EIF4E3	hsa-miR-555
EIF4E3	hsa-miR-6735-5p
EIF4E3	hsa-miR-567
EIF4E3	hsa-miR-1578
EIF4E3	hsa-miR-5134-5p
EIF4E3	hsa-miR-7068-5p
EIF4E3	hsa-miR-6705-5p
EIF4E3	hsa-miR-344b-3p
EIF4E3	hsa-miR-6840-3p
EIF4E3	hsa-miR-2377
EIF4E3	hsa-miR-26-5p
EIF4E3	hsa-miR-142-3p
EIF4E3	hsa-miR-6330
EIF4E3	hsa-miR-133a-5p
EIF4E3	hsa-miR-548ah-3p
EIF4E3	hsa-miR-1237-3p
EIF4E3	hsa-miR-4665-5p
EIF4E3	hsa-miR-325-5p
EIF4E3	hsa-miR-548t-3p
EIF4E3	hsa-miR-8814
EIF4E3	hsa-miR-3150a-5p
EIF4E3	hsa-miR-520g-3p
EIF4E3	hsa-miR-4639-3p
EIF4E3	hsa-miR-204-5p
EIF4E3	hsa-miR-7398u-3p
EIF4E3	hsa-miR-3602
EIF4E3	hsa-miR-7024-3p
EIF4E3	hsa-miR-199
EIF4E3	hsa-miR-7389-3p
EIF4E3	hsa-miR-2284y
EIF4E3	hsa-miR-619
EIF4E3	hsa-miR-1841
EIF4E3	hsa-miR-218-5p
EIF4E3	hsa-miR-6911-5p
EIF4E3	hsa-miR-8823
EIF4E3	hsa-miR-6732-5p
EIF4E3	hsa-miR-7212-5p

EIF4E3	hsa-miR-491-3p
EIF4E3	hsa-miR-7215-3p
EIF4E3	hsa-miR-2310
EIF4E3	hsa-miR-6526
EIF4E3	hsa-miR-98-3p
EIF4E3	hsa-miR-2414
EIF4E3	hsa-miR-632
EIF4E3	hsa-miR-3174
EIF4E3	hsa-miR-6983-5p
EIF4E3	hsa-miR-7378-3p
EIF4E3	hsa-miR-216b-5p
EIF4E3	hsa-miR-2446
EIF4E3	hsa-miR-330-3p
EIF4E3	hsa-miR-1668
EIF4E3	hsa-miR-4643
EIF4E3	hsa-miR-4277
EIF4E3	hsa-miR-2376
EIF4E3	hsa-miR-140-5p
EIF4E3	hsa-miR-6808-3p
EIF4E3	hsa-miR-19-5p
EIF4E3	hsa-miR-8891
EIF4E3	hsa-miR-7216-3p
EIF4E3	hsa-miR-6812-5p
EIF4E3	hsa-miR-7219-5p
EIF4E3	hsa-miR-7047-5p
EIF4E3	hsa-miR-4430
EIF4E3	hsa-miR-3614-3p
EIF4E3	hsa-miR-543-5p
EIF4E3	hsa-miR-7199-3p
EIF4E3	hsa-miR-7070-5p
EIF4E3	hsa-miR-655
EIF4E3	hsa-miR-1712-3p
EIF4E3	hsa-miR-653-5p
EIF4E3	hsa-miR-7845-5p
EIF4E3	hsa-miR-574-3p
EIF4E3	hsa-miR-6354
EIF4E3	hsa-miR-6867-3p
EIF4E3	hsa-miR-518
EIF4E3	hsa-miR-4511
EIF4E3	hsa-miR-483-3p.1
EIF4E3	hsa-miR-6792-5p
EIF4E3	hsa-miR-7059-5p
EIF4E3	hsa-miR-2301
EIF4E3	hsa-miR-218a-1-3p
EIF4E3	hsa-miR-520
EIF4E3	hsa-miR-5626-5p

EIF4E3	hsa-miR-328
EIF4E3	hsa-miR-7282-5p
EIF4E3	hsa-miR-6730-3p
EIF4E3	hsa-miR-190a-3p
EIF4E3	hsa-miR-4777-5p
EIF4E3	hsa-miR-3620-3p
EIF4E3	hsa-miR-6891-5p
EIF4E3	hsa-miR-1619
EIF4E3	hsa-miR-92a-5p
EIF4E3	hsa-miR-7245-5p
EIF4E3	hsa-miR-6930-3p
EIF4E3	hsa-miR-7398r-3p
EIF4E3	hsa-miR-5695
EIF4E3	hsa-miR-411-5p.2
EIF4E3	hsa-miR-5002-3p
EIF4E3	hsa-miR-155-5p
EIF4E3	hsa-miR-504
EIF4E3	hsa-miR-4696
EIF4E3	hsa-miR-1966-5p
EIF4E3	hsa-miR-4469
EIF4E3	hsa-miR-942-5p
EIF4E3	hsa-miR-3140-3p
EIF4E3	hsa-miR-6998-3p
EIF4E3	hsa-miR-7296-5p
EIF4E3	hsa-miR-96-3p
EIF4E3	hsa-miR-291-3p
EIF4E3	hsa-miR-7340-3p
EIF4E3	hsa-miR-802-3p
EIF4E3	hsa-miR-8060
EIF4E3	hsa-miR-1191a
EIF4E3	hsa-miR-337-3p
EIF4E3	hsa-miR-4669
EIF4E3	hsa-miR-629-3p
EIF4E3	hsa-miR-6990-3p
EIF4E3	hsa-miR-7385e-3p
EIF4E3	hsa-miR-3144-5p
EIF4E3	hsa-miR-664a-3p
EIF4E3	hsa-miR-1264-5p
EIF4E3	hsa-miR-190-5p
EIF4E3	hsa-miR-7280-5p
EIF4E3	hsa-miR-2276-3p
EIF4E3	hsa-miR-512
EIF4E3	hsa-miR-5132-5p
EIF4E3	hsa-miR-8837
EIF4E3	hsa-miR-27b-5p
EIF4E3	hsa-miR-3202

EIF4E3	hsa-miR-5003-5p
EIF4E3	hsa-miR-2284g
EIF4E3	hsa-miR-509-5p
EIF4E3	hsa-miR-3068-3p
EIF4E3	hsa-miR-3589
EIF4E3	hsa-miR-3146
EIF4E3	hsa-miR-6788-5p
EIF4E3	hsa-miR-195-5p
EIF4E3	hsa-miR-2889
EIF4E3	hsa-miR-212-3p
EIF4E3	hsa-miR-122
EIF4E3	hsa-miR-3524-3p
EIF4E3	hsa-miR-6951-5p
EIF4E3	hsa-miR-669i
EIF4E3	hsa-miR-6800-5p
EIF4E3	hsa-miR-7371e-3p
EIF4E3	hsa-miR-351-3p
EIF4E3	hsa-miR-1814
EIF4E3	hsa-miR-7843-3p
EIF4E3	hsa-miR-6509-3p
EIF4E3	hsa-miR-299
EIF4E3	hsa-miR-3153
EIF4E3	hsa-miR-6321
EIF4E3	hsa-miR-3614-5p
EIF4E3	hsa-miR-8862
EIF4E3	hsa-miR-2444
EIF4E3	hsa-miR-2284-5p
EIF4E3	hsa-miR-8902
EIF4E3	hsa-miR-3189-5p
EIF4E3	hsa-miR-7116-5p
EIF4E3	hsa-miR-7059-3p
EIF4E3	hsa-miR-7375-5p
EIF4E3	hsa-miR-8113
EIF4E3	hsa-miR-182-5p
EIF4E3	hsa-miR-7374b-3p
EIF4E3	hsa-miR-7118-3p
EIF4E3	hsa-miR-550
EIF4E3	hsa-miR-3909
EIF4E3	hsa-miR-371b-5p
EIF4E3	hsa-miR-3167-5p
EIF4E3	hsa-miR-7042-5p
EIF4E3	hsa-miR-6946-5p
EIF4E3	hsa-miR-2396
EIF4E3	hsa-miR-374c-5p
EIF4E3	hsa-miR-548at-5p
EIF4E3	hsa-miR-7053-3p

EIF4E3	hsa-miR-2371
EIF4E3	hsa-miR-4278
EIF4E3	hsa-miR-3085
EIF4E3	hsa-miR-669c-3p
EIF4E3	hsa-miR-624-3p
EIF4E3	hsa-miR-29c-3p
EIF4E3	hsa-miR-216-3p
EIF4E3	hsa-miR-6665-5p
EIF4E3	hsa-miR-664-5p
EIF4E3	hsa-miR-6963-3p
EIF4E3	hsa-miR-7005-3p
EIF4E3	hsa-miR-466l-5p
EIF4E3	hsa-miR-548az-5p
EIF4E3	hsa-miR-4674
EIF4E3	hsa-miR-141-3p
EIF4E3	hsa-miR-4506
EIF4E3	hsa-miR-208-3p
EIF4E3	hsa-miR-4484
EIF4E3	hsa-miR-2136
EIF4E3	hsa-miR-323b-5p
EIF4E3	hsa-miR-7386h-3p
EIF4E3	hsa-miR-3058-5p
EIF4E3	hsa-miR-1283
EIF4E3	hsa-miR-6130
EIF4E3	hsa-miR-466bcp-3p
EIF4E3	hsa-miR-3062-3p
EIF4E3	hsa-miR-3125
EIF4E3	hsa-miR-3658
EIF4E3	hsa-miR-338-3p
EIF4E3	hsa-miR-1282
EIF4E3	hsa-miR-3098-3p
EIF4E3	hsa-miR-185-5p
EIF4E3	hsa-miR-8849
EIF4E3	hsa-miR-7303-5p
EIF4E3	hsa-miR-1909-5p
EIF4E3	hsa-miR-1953
EIF4E3	hsa-miR-548x-3p
EIF4E3	hsa-miR-4528
EIF4E3	hsa-miR-7264-3p
EIF4E3	hsa-miR-3078-5p
EIF4E3	hsa-miR-3682-5p
EIF4E3	hsa-miR-1260a
EIF4E3	hsa-miR-6348
EIF4E3	hsa-miR-7338-5p
EIF4E3	hsa-miR-548av-3p
EIF4E3	hsa-miR-7007-5p

EIF4E3	hsa-miR-2305
EIF4E3	hsa-miR-1564-5p
EIF4E3	hsa-miR-2355-5p
EIF4E3	hsa-miR-3096-5p
EIF4E3	hsa-miR-1896
EIF4E3	hsa-miR-1178-3p
EIF4E3	hsa-miR-7472-5p
EIF4E3	hsa-miR-6873-3p
EIF4E3	hsa-miR-6870-5p
EIF4E3	hsa-miR-765
EIF4E3	hsa-miR-6792-3p
EIF4E3	hsa-miR-708-5p
EIF4E3	hsa-miR-3110-5p
EIF4E3	hsa-miR-7277-3p
EIF4E3	hsa-miR-4482-5p
EIF4E3	hsa-miR-3551-5p
EIF4E3	hsa-miR-7388a-3p
EIF4E3	hsa-miR-1599
EIF4E3	hsa-miR-3102-3p.2-3p
EIF4E3	hsa-miR-1931
EIF4E3	hsa-miR-153-5p
EIF4E3	hsa-miR-6965-5p
EIF4E3	hsa-miR-499
EIF4E3	hsa-miR-20-3p
EIF4E3	hsa-miR-7349-5p
EIF4E3	hsa-miR-627
EIF4E3	hsa-miR-592
EIF4E3	hsa-miR-98-5p
EIF4E3	hsa-miR-2401
EIF4E3	hsa-miR-2390
EIF4E3	hsa-miR-7301-3p
EIF4E3	hsa-miR-203a-3p.1
EIF4E3	hsa-miR-3064-3p
EIF4E3	hsa-miR-7119-3p
EIF4E3	hsa-miR-942
EIF4E3	hsa-miR-4520-3p
EIF4E3	hsa-miR-2285h
EIF4E3	hsa-miR-1807
EIF4E3	hsa-miR-107
EIF4E3	hsa-miR-4743-3p
EIF4E3	hsa-miR-7314-5p
EIF4E3	hsa-miR-6505-5p
EIF4E3	hsa-miR-9b-3p
EIF4E3	hsa-miR-3925-5p
EIF4E3	hsa-miR-3173-3p
EIF4E3	hsa-miR-550a-5p

EIF4E3	hsa-miR-29a-3p
EIF4E3	hsa-miR-5625-3p
EIF4E3	hsa-miR-294-5p
EIF4E3	hsa-miR-490-3p
EIF4E3	hsa-miR-4697-5p
EIF4E3	hsa-miR-770-3p
EIF4E3	hsa-miR-1197-5p
EIF4E3	hsa-miR-503-5p
EIF4E3	hsa-miR-6510-5p
EIF4E3	hsa-miR-1942
EIF4E3	hsa-miR-224-5p
EIF4E3	hsa-miR-4766-5p
EIF4E3	hsa-miR-4771
EIF4E3	hsa-miR-592-5p
EIF4E3	hsa-miR-219b-3p
EIF4E3	hsa-miR-7373b-3p
EIF4E3	hsa-miR-764-3p
EIF4E3	hsa-miR-513c-5p
EIF4E3	hsa-miR-4723-3p
EIF4E3	hsa-miR-3592
EIF4E3	hsa-miR-4724-5p
EIF4E3	hsa-miR-331-5p
EIF4E3	hsa-miR-4276
EIF4E3	hsa-miR-743ab-3p
EIF4E3	hsa-miR-291b-3p
EIF4E3	hsa-miR-7263-3p
EIF4E3	hsa-miR-7680-3p
EIF4E3	hsa-miR-145-3p
EIF4E3	hsa-miR-3594-3p
EIF4E3	hsa-miR-6511-3p
EIF4E3	hsa-miR-584-5p
EIF4E3	hsa-miR-7112-3p
EIF4E3	hsa-miR-3061-5p
EIF4E3	hsa-miR-1231-5p
EIF4E3	hsa-miR-27a-5p
EIF4E3	hsa-miR-7650-3p
EIF4E3	hsa-miR-145a-3p
EIF4E3	hsa-miR-7193-5p
EIF4E3	hsa-miR-495-3p
EIF4E3	hsa-miR-412
EIF4E3	hsa-miR-216
EIF4E3	hsa-miR-873a-5p.2
EIF4E3	hsa-miR-587
EIF4E3	hsa-miR-548j-5p
EIF4E3	hsa-miR-4654
EIF4E3	hsa-miR-378a-5p

EIF4E3	hsa-miR-449c-5p
EIF4E3	hsa-miR-6748-3p
EIF4E3	hsa-miR-4263
EIF4E3	hsa-miR-548b-3p
EIF4E3	hsa-miR-1301-3p
EIF4E3	hsa-miR-301b-5p
EIF4E3	hsa-miR-7355-5p
EIF4E3	hsa-miR-1305
EIF4E3	hsa-miR-1933-5p
EIF4E3	hsa-miR-6754-5p
EIF4E3	hsa-miR-21-3p
EIF4E3	hsa-miR-203b-5p
EIF4E3	hsa-miR-7221-5p
EIF4E3	hsa-miR-1262-3p
EIF4E3	hsa-miR-6605-5p
EIF4E3	hsa-miR-6561-3p
EIF4E3	hsa-miR-7090-5p
EIF4E3	hsa-miR-5194
EIF4E3	hsa-miR-548i-3p
EIF4E3	hsa-miR-7323-5p
EIF4E3	hsa-miR-33-5p
EIF4E3	hsa-miR-7279-5p
EIF4E3	hsa-miR-887-5p
EIF4E3	hsa-miR-344i
EIF4E3	hsa-miR-375-3p
EIF4E3	hsa-miR-3646
EIF4E3	hsa-miR-548aa
EIF4E3	hsa-miR-372
EIF4E3	hsa-miR-6080
EIF4E3	hsa-miR-579-3p
EIF4E3	hsa-miR-4445-3p
EIF4E3	hsa-miR-5098
EIF4E3	hsa-miR-211-5p
EIF4E3	hsa-miR-6774-5p
EIF4E3	hsa-miR-295-5p
EIF4E3	hsa-miR-7366-3p
EIF4E3	hsa-miR-934-3p
EIF4E3	hsa-miR-424-3p
EIF4E3	hsa-miR-7231-3p
EIF4E3	hsa-miR-301a-3p
EIF4E3	hsa-miR-7265-5p
EIF4E3	hsa-miR-4679
EIF4E3	hsa-miR-2285q
EIF4E3	hsa-miR-140-3p.2
EIF4E3	hsa-miR-5700
EIF4E3	hsa-miR-2285b

EIF4E3	hsa-miR-323
EIF4E3	hsa-miR-4264
EIF4E3	hsa-miR-8809
EIF4E3	hsa-miR-888-5p
EIF4E3	hsa-miR-8807
EIF4E3	hsa-miR-7351-5p
EIF4E3	hsa-miR-4270
EIF4E3	hsa-miR-152-5p
EIF4E3	hsa-miR-4439
EIF4E3	hsa-miR-8485
EIF4E3	hsa-miR-6715-5p
EIF4E3	hsa-miR-3191-3p
EIF4E3	hsa-miR-663a
EIF4E3	hsa-miR-2285ad
EIF4E3	hsa-miR-1-5p
EIF4E3	hsa-miR-652-5p
EIF4E3	hsa-miR-7688-3p
EIF4E3	hsa-miR-1255a
EIF4E3	hsa-miR-3556a
EIF4E3	hsa-miR-7019-5p
EIF4E3	hsa-miR-7328-3p
EIF4E3	hsa-miR-6952-5p
EIF4E3	hsa-miR-4659a-5p
EIF4E3	hsa-miR-30b-3p
EIF4E3	hsa-miR-1193-5p
EIF4E3	hsa-miR-141-5p
EIF4E3	hsa-miR-7161-5p
EIF4E3	hsa-miR-320a
EIF4E3	hsa-miR-2434
EIF4E3	hsa-miR-548c-3p
EIF4E3	hsa-miR-154b
EIF4E3	hsa-miR-1454
EIF4E3	hsa-miR-2426
EIF4E3	hsa-miR-588
EIF4E3	hsa-miR-671-5p
EIF4E3	hsa-miR-433
EIF4E3	hsa-miR-92b-5p
EIF4E3	hsa-miR-4667-5p
EIF4E3	hsa-miR-4325
EIF4E3	hsa-miR-138-5p
EIF4E3	hsa-miR-4670-3p
EIF4E3	hsa-miR-4796-3p
EIF4E3	hsa-miR-6903-3p
EIF4E3	hsa-miR-99-3p
EIF4E3	hsa-miR-5089-3p
EIF4E3	hsa-miR-6948-3p

EIF4E3	hsa-miR-214-5p
EIF4E3	hsa-miR-1709
EIF4E3	hsa-miR-6531
EIF4E3	hsa-miR-181b-3p
EIF4E3	hsa-miR-7049-5p
EIF4E3	hsa-miR-7679-3p
EIF4E3	hsa-miR-6938-3p
EIF4E3	hsa-miR-3657
EIF4E3	hsa-miR-6919-3p
EIF4E3	hsa-miR-8881
EIF4E3	hsa-miR-6347
EIF4E3	hsa-miR-548ad-3p
EIF4E3	hsa-miR-101b-3p.1
EIF4E3	hsa-miR-873-5p
EIF4E3	hsa-miR-6514-3p
EIF4E3	hsa-miR-4717-5p
EIF4E3	hsa-miR-3575
EIF4E3	hsa-miR-8840
EIF4E3	hsa-miR-6685-5p
EIF4E3	hsa-miR-6861-5p
EIF4E3	hsa-miR-7386b-3p
EIF4E3	hsa-miR-4524-5p
EIF4E3	hsa-miR-8810
EIF4E3	hsa-miR-1982-5p
EIF4E3	hsa-miR-3544-3p
EIF4E3	hsa-miR-7976
EIF4E3	hsa-miR-1943-3p
EIF4E3	hsa-miR-664-3p
EIF4E3	hsa-miR-8908-5p
EIF4E3	hsa-miR-764-5p
EIF4E3	hsa-miR-7216-5p
EIF4E3	hsa-miR-1239
EIF4E3	hsa-miR-181d-3p
EIF4E3	hsa-miR-7113-5p
EIF4E3	hsa-miR-6847-5p
EIF4E3	hsa-miR-504-5p.1
EIF4E3	hsa-let-7f-2-3p
EIF4E3	hsa-miR-6124
EIF4E3	hsa-miR-515-5p
EIF4E3	hsa-miR-1470
EIF4E3	hsa-miR-543
EIF4E3	hsa-miR-939-3p
EIF4E3	hsa-miR-7281-5p
EIF4E3	hsa-miR-548aef
EIF4E3	hsa-miR-548at-3p
EIF4E3	hsa-miR-293-5p

EIF4E3	hsa-miR-7214-3p
EIF4E3	hsa-miR-653-3p
EIF4E3	hsa-miR-589-5p
EIF4E3	hsa-miR-7246-3p
EIF4E3	hsa-miR-3084-3p
EIF4E3	hsa-miR-4517
EIF4E3	hsa-miR-4419b
EIF4E3	hsa-miR-758-3p
EIF4E3	hsa-miR-7011-5p
EIF4E3	hsa-miR-767-5p
EIF4E3	hsa-miR-6899-5p
EIF4E3	hsa-miR-568
EIF4E3	hsa-miR-3156-3p
EIF4E3	hsa-miR-3099-5p
EIF4E3	hsa-miR-135a-2-3p
EIF4E3	hsa-miR-339-5p
EIF4E3	hsa-miR-3120-3p
EIF4E3	hsa-miR-6696-5p
EIF4E3	hsa-miR-205-3p
EIF4E3	hsa-miR-1687-5p
EIF4E3	hsa-miR-6537-5p
EIF4E3	hsa-miR-130b-5p
EIF4E3	hsa-miR-7106-5p
EIF4E3	hsa-miR-3473g
EIF4E3	hsa-miR-1273h-5p
EIF4E3	hsa-miR-7313-5p
EIF4E3	hsa-miR-502a
EIF4E3	hsa-miR-7865
EIF4E3	hsa-miR-4711-5p
EIF4E3	hsa-miR-4646-3p
EIF4E3	hsa-miR-30e-3p
EIF4E3	hsa-miR-3569-5p
EIF4E3	hsa-miR-712-5p
EIF4E3	hsa-miR-8071
EIF4E3	hsa-miR-421-3p
EIF4E3	hsa-miR-29a-5p
EIF4E3	hsa-miR-1677-5p
EIF4E3	hsa-miR-7211-5p
EIF4E3	hsa-miR-1468-3p
EIF4E3	hsa-miR-301b-3p
EIF4E3	hsa-miR-148-5p
EIF4E3	hsa-miR-500
EIF4E3	hsa-miR-1224-5p
EIF4E3	hsa-miR-6979-5p
EIF4E3	hsa-miR-7094-1-5p
EIF4E3	hsa-miR-3089-5p

EIF4E3	hsa-miR-547-3p
EIF4E3	hsa-miR-105-5p
EIF4E3	hsa-miR-7342-3p
EIF4E3	hsa-miR-7844-5p
EIF4E3	hsa-miR-6069
EIF4E3	hsa-miR-545-3p
EIF4E3	hsa-miR-4676-5p
EIF4E3	hsa-miR-6506-5p
EIF4E3	hsa-miR-124-5p
EIF4E3	hsa-miR-186-3p
EIF4E3	hsa-miR-194-5p
EIF4E3	hsa-miR-4662b
EIF4E3	hsa-miR-6958-3p
EIF4E3	hsa-miR-2285r
EIF4E3	hsa-miR-2380
EIF4E3	hsa-miR-6822-5p
EIF4E3	hsa-miR-2340
EIF4E3	hsa-miR-548w
EIF4E3	hsa-miR-197-3p
EIF4E3	hsa-miR-7347-5p
EIF4E3	hsa-miR-7481-5p
EIF4E3	hsa-miR-195-5p
EIF4E3	hsa-miR-4786-3p
EIF4E3	hsa-miR-146b-3p
EIF4E3	hsa-miR-7235-5p
EIF4E3	hsa-miR-3664-5p
EIF4E3	hsa-miR-130b-3p
EIF4E3	hsa-miR-369-3p
EIF4E3	hsa-miR-329b
EIF4E3	hsa-miR-6739-5p
EIF4E3	hsa-miR-6320
EIF4E3	hsa-miR-548t-5p
EIF4E3	hsa-miR-7515
EIF4E3	hsa-miR-670
EIF4E3	hsa-miR-576-3p
EIF4E3	hsa-miR-466bco-5p
EIF4E3	hsa-miR-4279
EIF4E3	hsa-miR-6947-3p
EIF4E3	hsa-miR-2404
EIF4E3	hsa-miR-6901-3p
EIF4E3	hsa-miR-7663-3p
EIF4E3	hsa-miR-514-3p
EIF4E3	hsa-miR-466
EIF4E3	hsa-miR-8903
EIF4E3	hsa-miR-7667-5p
EIF4E3	hsa-miR-379-5p

EIF4E3	hsa-miR-3688-3p
EIF4E3	hsa-miR-6809-5p
EIF4E3	hsa-miR-376ab-3p
EIF4E3	hsa-miR-676-5p
EIF4E3	hsa-miR-148-3p
EIF4E3	hsa-miR-6925-3p
EIF4E3	hsa-miR-342-5p
EIF4E3	hsa-miR-2482
EIF4E3	hsa-miR-664
EIF4E3	hsa-miR-147-5p
EIF4E3	hsa-miR-1838
EIF4E3	hsa-miR-487-3p
EIF4E3	hsa-miR-7057-5p
EIF4E3	hsa-miR-548aw
EIF4E3	hsa-miR-216b
EIF4E3	hsa-miR-29c-5p
EIF4E3	hsa-miR-3919
EIF4E3	hsa-miR-7168-5p
EIF4E3	hsa-miR-150-5p
EIF4E3	hsa-miR-6129
EIF4E3	hsa-miR-326
EIF4E3	hsa-miR-3655
EIF4E3	hsa-miR-7023-5p
EIF4E3	hsa-miR-6825-5p
EIF4E3	hsa-miR-6868-5p
EIF4E3	hsa-miR-19
EIF4E3	hsa-miR-7398n-5p
EIF4E3	hsa-miR-2985
EIF4E3	hsa-miR-4653-5p
EIF4E3	hsa-miR-3570
EIF4E3	hsa-miR-7037-5p
EIF4E3	hsa-miR-374-5p
EIF4E3	hsa-miR-6925-5p
EIF4E3	hsa-miR-6132
EIF4E3	hsa-miR-5589-3p
EIF4E3	hsa-miR-1781-3p
EIF4E3	hsa-miR-1236-3p
EIF4E3	hsa-miR-503-3p
EIF4E3	hsa-miR-2285n
EIF4E3	hsa-miR-7273-5p
EIF4E3	hsa-miR-7240-5p
EIF4E3	hsa-miR-2114-5p
EIF4E3	hsa-miR-219-3p
EIF4E3	hsa-miR-224-5p
EIF4E3	hsa-miR-3173-3p
EIF4E3	hsa-miR-741-3p

EIF4E3	hsa-miR-380-5p
EIF4E3	hsa-miR-25-3p
EIF4E3	hsa-miR-692
EIF4E3	hsa-miR-3092-3p
EIF4E3	hsa-miR-7388c-5p
EIF4E3	hsa-let-7g-3p
EIF4E3	hsa-miR-7398b-3p
EIF4E3	hsa-miR-7676-5p
EIF4E3	hsa-miR-4698
EIF4E3	hsa-miR-7212-3p
EIF4E3	hsa-miR-463-5p
EIF4E3	hsa-miR-2284c
EIF4E3	hsa-miR-7184-3p
EIF4E3	hsa-miR-6964-3p
EIF4E3	hsa-miR-4728-5p
EIF4E3	hsa-miR-489-3p
EIF4E3	hsa-miR-501-3p
EIF4E3	hsa-miR-190b-3p
EIF4E3	hsa-miR-7201-3p
EIF4E3	hsa-miR-1775-3p
EIF4E3	hsa-miR-548ae-5p
EIF4E3	hsa-miR-365a-5p
EIF4E3	hsa-miR-133b-5p
EIF4E3	hsa-miR-3077-3p
EIF4E3	hsa-miR-20
EIF4E3	hsa-miR-329-5p
EIF4E3	hsa-miR-7398m-3p
EIF4E3	hsa-miR-1755
EIF4E3	hsa-miR-30c-3p
EIF4E3	hsa-miR-7065-5p
EIF4E3	hsa-miR-4253
EIF4E3	hsa-miR-7007-3p
EIF4E3	hsa-miR-548f-3p
EIF4E3	hsa-miR-8094
EIF4E3	hsa-miR-450a-5p
EIF4E3	hsa-miR-2284
EIF4E3	hsa-miR-1458
EIF4E3	hsa-miR-130c-5p
EIF4E3	hsa-miR-471-3p
EIF4E3	hsa-miR-7385c-3p
EIF4E3	hsa-miR-322-3p
EIF4E3	hsa-miR-201-5p
EIF4E3	hsa-miR-6785-5p
EIF4E3	hsa-miR-7181-3p
EIF4E3	hsa-miR-1388-5p
EIF4E3	hsa-miR-4326

EIF4E3	hsa-miR-8884
EIF4E3	hsa-miR-6079
EIF4E3	hsa-let-7
EIF4E3	hsa-miR-454
EIF4E3	hsa-miR-1297
EIF4E3	hsa-miR-218-2-3p
EIF4E3	hsa-miR-3121-3p
EIF4E3	hsa-miR-548ae-3p
EIF4E3	hsa-miR-7093-5p
EIF4E3	hsa-miR-2381
EIF4E3	hsa-miR-7290-3p
EIF4E3	hsa-miR-211-3p
EIF4E3	hsa-miR-3689a-5p
EIF4E3	hsa-miR-877-3p
EIF4E3	hsa-miR-3618-5p
EIF4E3	hsa-miR-2909
EIF4E3	hsa-miR-449b-3p
EIF4E3	hsa-miR-4651
EIF4E3	hsa-miR-5617-5p
EIF4E3	hsa-miR-548d-5p
EIF4E3	hsa-miR-8796
EIF4E3	hsa-miR-6755-5p
EIF4E3	hsa-miR-7357-5p
EIF4E3	hsa-miR-6944-5p
EIF4E3	hsa-miR-877-5p
EIF4E3	hsa-miR-193
EIF4E3	hsa-miR-8069
EIF4E3	hsa-miR-93-5p
EIF4E3	hsa-miR-144-3p
EIF4E3	hsa-miR-3654
EIF4E3	hsa-miR-4684-5p
EIF4E3	hsa-miR-6872-3p
EIF4E3	hsa-miR-2312
EIF4E3	hsa-miR-539-5p
EIF4E3	hsa-miR-143-3p
EIF4E3	hsa-miR-1237-5p
EIF4E3	hsa-miR-6996-3p
EIF4E3	hsa-miR-23
EIF4E3	hsa-miR-494-3p
EIF4E3	hsa-miR-221-3p
EIF4E3	hsa-miR-8871
EIF4E3	hsa-miR-7043-5p
EIF4E3	hsa-miR-664b-5p
EIF4E3	hsa-miR-684
EIF4E3	hsa-miR-548y
EIF4E3	hsa-miR-5121

EIF4E3	hsa-miR-544a
EIF4E3	hsa-miR-1224-3p
EIF4E3	hsa-miR-3074-2-3p
EIF4E3	hsa-miR-3094-3p
EIF4E3	hsa-miR-7056-5p
EIF4E3	hsa-miR-3193
EIF4E3	hsa-miR-7368-3p
EIF4E3	hsa-miR-4477a
EIF4E3	hsa-miR-2464-3p
EIF4E3	hsa-miR-7077-3p
EIF4E3	hsa-miR-1262-5p
EIF4E3	hsa-miR-7671-5p
EIF4E3	hsa-miR-2382-5p
EIF4E3	hsa-miR-890-5p
EIF4E3	hsa-miR-548av-5p
EIF4E3	hsa-miR-6513-3p
EIF4E3	hsa-miR-1323
EIF4E3	hsa-miR-100-3p
EIF4E3	hsa-miR-7843-5p
EIF4E3	hsa-miR-128
EIF4E3	hsa-miR-4727-5p
EIF4E3	hsa-miR-291
EIF4E3	hsa-miR-7078-3p
EIF4E3	hsa-miR-6859-5p
EIF4E3	hsa-miR-6744-5p
EIF4E3	hsa-miR-2384
EIF4E3	hsa-miR-1199-5p
EIF4E3	hsa-miR-6940-3p
EIF4E3	hsa-miR-651
EIF4E3	hsa-miR-3072-3p
EIF4E3	hsa-miR-381-3p
EIF4E3	hsa-miR-370
EIF4E3	hsa-miR-7060-5p
EIF4E3	hsa-miR-7012-3p
EIF4E3	hsa-miR-3977
EIF4E3	hsa-miR-2469
EIF4E3	hsa-miR-2325c
EIF4E3	hsa-miR-375
EIF4E3	hsa-miR-8802
EIF4E3	hsa-miR-6994-5p
EIF4E3	hsa-miR-3092-5p
EIF4E3	hsa-miR-9-3p
EIF4E3	hsa-miR-2392
EIF4E3	hsa-miR-4501
EIF4E3	hsa-miR-489
EIF4E3	hsa-miR-7674-5p

EIF4E3	hsa-miR-465c-5p
EIF4E3	hsa-miR-6516-3p
EIF4E3	hsa-miR-153-3p
EIF4E3	hsa-miR-4761-3p
EIF4E3	hsa-miR-146-5p
EIF4E3	hsa-miR-7374a-3p
EIF4E3	hsa-miR-3087-5p
EIF4E3	hsa-miR-5107-5p
EIF4E3	hsa-miR-1190
EIF4E3	hsa-miR-616-3p
EIF4E3	hsa-miR-7227-5p
EIF4E3	hsa-miR-4768-3p
EIF4E3	hsa-miR-7064-5p
EIF4E3	hsa-miR-1783
EIF4E3	hsa-miR-7343-3p
EIF4E3	hsa-miR-3158-5p
EIF4E3	hsa-miR-6932-3p
EIF4E3	hsa-miR-1277-5p
EIF4E3	hsa-miR-6752-3p
EIF4E3	hsa-miR-8883
EIF4E3	hsa-miR-6777-3p
EIF4E3	hsa-miR-1537-5p
EIF4E3	hsa-miR-8806
EIF4E3	hsa-miR-1892
EIF4E3	hsa-miR-1225-3p
EIF4E3	hsa-miR-17
EIF4E3	hsa-miR-302-5p
EIF4E3	hsa-miR-502-5p
EIF4E3	hsa-miR-487b-5p
EIF4E3	hsa-miR-3601
EIF4E3	hsa-miR-3102
EIF4E3	hsa-miR-8867
EIF4E3	hsa-miR-4794
EIF4E3	hsa-miR-146
EIF4E3	hsa-miR-6996-5p
EIF4E3	hsa-miR-541-3p
EIF4E3	hsa-miR-7276-3p
EIF4E3	hsa-miR-6582-3p
EIF4E3	hsa-miR-633
EIF4E3	hsa-miR-7270-5p
EIF4E3	hsa-miR-7335-3p
EIF4E3	hsa-miR-8812
EIF4E3	hsa-miR-4516
EIF4E3	hsa-miR-5001-3p
EIF4E3	hsa-miR-5584-5p
EIF4E3	hsa-miR-1271-5p

EIF4E3	hsa-miR-7094-3p
EIF4E3	hsa-miR-4776-3p
EIF4E3	hsa-miR-4328
EIF4E3	hsa-miR-769-3p
EIF4E3	hsa-miR-320e
EIF4E3	hsa-miR-7386f-3p
EIF4E3	hsa-miR-434-5p
EIF4E3	hsa-miR-1664-5p
EIF4E3	hsa-miR-6952-3p
EIF4E3	hsa-miR-743-5p
EIF4E3	hsa-miR-7030-5p
EIF4E3	hsa-miR-150-3p
EIF4E3	hsa-miR-7053-5p
EIF4E3	hsa-miR-6841-3p
EIF4E3	hsa-miR-6846-5p
EIF4E3	hsa-miR-8790
EIF4E3	hsa-miR-205-5p
EIF4E3	hsa-miR-219c-3p
EIF4E3	hsa-miR-1233-5p
EIF4E3	hsa-miR-2284n
EIF4E3	hsa-miR-4445-5p
EIF4E3	hsa-miR-10
EIF4E3	hsa-miR-2364
EIF4E3	hsa-miR-7399-3p
EIF4E3	hsa-miR-292b-5p
EIF4E3	hsa-miR-23b-3p
EIF4E3	hsa-miR-3140-3p
EIF4E3	hsa-miR-107-3p
EIF4E3	hsa-miR-7655-5p
EIF4E3	hsa-miR-7379-3p
EIF4E3	hsa-miR-466dk-5p
EIF4E3	hsa-miR-584-3p
EIF4E3	hsa-miR-4657
EIF4E3	hsa-miR-6910-3p
EIF4E3	hsa-miR-5010-5p
EIF4E3	hsa-miR-7366-5p
EIF4E3	hsa-miR-4729
EIF4E3	hsa-miR-770-5p
EIF4E3	hsa-miR-488-3p
EIF4E3	hsa-miR-804
EIF4E3	hsa-miR-3070-3p
EIF4E3	hsa-miR-1285-5p
EIF4E3	hsa-miR-483-3p
EIF4E3	hsa-miR-6536
EIF4E3	hsa-miR-2465
EIF4E3	hsa-miR-7309-3p

EIF4E3	hsa-miR-624-3p
EIF4E3	hsa-miR-136-5p
EIF4E3	hsa-miR-548am-3p
EIF4E3	hsa-miR-147a
EIF4E3	hsa-miR-129-5p
EIF4E3	hsa-miR-335-3p
EIF4E3	hsa-miR-892b
EIF4E3	hsa-miR-1200
EIF4E3	hsa-miR-626
EIF4E3	hsa-miR-683
EIF4E3	hsa-miR-6120-3p
EIF4E3	hsa-miR-3915
EIF4E3	hsa-miR-3163
EIF4E3	hsa-miR-4733-5p
EIF4E3	hsa-miR-183-5p.2
EIF4E3	hsa-miR-548a-5p
EIF4E3	hsa-miR-7114-5p
EIF4E3	hsa-miR-8908b
EIF4E3	hsa-miR-548aq-5p
EIF4E3	hsa-miR-4275
EIF4E3	hsa-miR-6122-3p
EIF4E3	hsa-miR-3187-3p
EIF4E3	hsa-miR-6911-3p
EIF4E3	hsa-miR-7078-5p
EIF4E3	hsa-miR-644a
EIF4E3	hsa-miR-7057-3p
EIF4E3	hsa-miR-8100
EIF4E3	hsa-let-7abf-3p
EIF4E3	hsa-miR-5704
EIF4E3	hsa-miR-7031-5p
EIF4E3	hsa-miR-7390-3p
EIF4E3	hsa-miR-203-3p.1
EIF4E3	hsa-miR-4736
EIF4E3	hsa-miR-192-3p
EIF4E3	hsa-miR-7386k-3p
EIF4E3	hsa-miR-181b-1-3p
EIF4E3	hsa-miR-669e-3p
EIF4E3	hsa-miR-5694
EIF4E3	hsa-miR-33b-3p
EIF4E3	hsa-miR-3613-5p
EIF4E3	hsa-miR-3689e
EIF4E3	hsa-miR-301-5p
EIF4E3	hsa-miR-7243-5p
EIF4E3	hsa-miR-466c-3p
EIF4E3	hsa-miR-103-3p
EIF4E3	hsa-miR-4470

EIF4E3	hsa-miR-5010-3p
EIF4E3	hsa-miR-2332
EIF4E3	hsa-miR-7324-5p
EIF4E3	hsa-miR-1276
EIF4E3	hsa-miR-1b-5p
EIF4E3	hsa-miR-6890-3p
EIF4E3	hsa-miR-199-5p
EIF4E3	hsa-miR-410
EIF4E3	hsa-miR-6836-5p
EIF4E3	hsa-miR-1843
EIF4E3	hsa-miR-3157-5p
EIF4E3	hsa-miR-7278-3p
EIF4E3	hsa-miR-1718
EIF4E3	hsa-miR-29
EIF4E3	hsa-miR-135b-3p
EIF4E3	hsa-miR-1746
EIF4E3	hsa-miR-7109-5p
EIF4E3	hsa-miR-5096
EIF4E3	hsa-miR-8890
EIF4E3	hsa-miR-325-3p
EIF4E3	hsa-miR-4641
EIF4E3	hsa-miR-939-5p
EIF4E3	hsa-miR-300
EIF4E3	hsa-miR-874-3p
EIF4E3	hsa-miR-762
EIF4E3	hsa-miR-7689-3p
EIF4E3	hsa-miR-3100-5p
EIF4E3	hsa-miR-7055-3p
EIF4E3	hsa-miR-7656-3p
EIF4E3	hsa-miR-345-5p
EIF4E3	hsa-miR-133a-3p.1
EIF4E3	hsa-miR-623
EIF4E3	hsa-miR-1683
EIF4E3	hsa-miR-1951
EIF4E3	hsa-miR-2284u
EIF4E3	hsa-miR-4725-3p
EIF4E3	hsa-miR-8875
EIF4E3	hsa-miR-2115-3p
EIF4E3	hsa-miR-5088-5p
EIF4E3	hsa-miR-634
EIF4E3	hsa-miR-15a-3p
EIF4E3	hsa-miR-4648
EIF4E3	hsa-miR-7387-3p
EIF4E3	hsa-miR-6998-5p
EIF4E3	hsa-miR-152
EIF4E3	hsa-miR-548bd-3p

EIF4E3	hsa-miR-7246-5p
EIF4E3	hsa-miR-5197-3p
EIF4E3	hsa-miR-5589-5p
EIF4E3	hsa-miR-6950-3p
EIF4E3	hsa-miR-1908-5p
EIF4E3	hsa-miR-605
EIF4E3	hsa-miR-7664-3p
EIF4E3	hsa-miR-5116
EIF4E3	hsa-miR-693-3p
EIF4E3	hsa-miR-631
EIF4E3	hsa-miR-125b-1-3p
EIF4E3	hsa-miR-34b-3p
EIF4E3	hsa-miR-3074-3p
EIF4E3	hsa-miR-7398s-3p
EIF4E3	hsa-miR-3168
EIF4E3	hsa-miR-6978-5p
EIF4E3	hsa-miR-6731-5p
EIF4E3	hsa-miR-362-3p
EIF4E3	hsa-miR-4765
EIF4E3	hsa-miR-1903
EIF4E3	hsa-miR-4286
EIF4E3	hsa-miR-203-5p
EIF4E3	hsa-miR-1237-3p
EIF4E3	hsa-miR-552
EIF4E3	hsa-miR-6704-5p
EIF4E3	hsa-miR-7111-5p
EIF4E3	hsa-miR-376a-5p
EIF4E3	hsa-miR-3690
EIF4E3	hsa-miR-7192-3p
EIF4E3	hsa-miR-3099-3p
EIF4E3	hsa-miR-500a-3p
EIF4E3	hsa-miR-1248
EIF4E3	hsa-miR-3104-3p
EIF4E3	hsa-miR-508-3p
EIF4E3	hsa-miR-455-3p.2
EIF4E3	hsa-miR-4680-3p
EIF4E3	hsa-miR-6340
EIF4E3	hsa-miR-7313-3p
EIF4E3	hsa-miR-3101-5p
EIF4E3	hsa-miR-2890
EIF4E3	hsa-miR-2893
EIF4E3	hsa-miR-1929-3p
EIF4E3	hsa-miR-2454-3p
EIF4E3	hsa-miR-520f-5p
EIF4E3	hsa-miR-516b-5p
EIF4E3	hsa-miR-548ba

EIF4E3	hsa-miR-8074
EIF4E3	hsa-miR-6779-3p
EIF4E3	hsa-miR-1273h-3p
EIF4E3	hsa-miR-7200-3p
EIF4E3	hsa-miR-7005-5p
EIF4E3	hsa-miR-455-3p
EIF4E3	hsa-miR-6927-3p
EIF4E3	hsa-miR-1814b
EIF4E3	hsa-miR-7665-5p
EIF4E3	hsa-miR-4706
EIF4E3	hsa-miR-3958
EIF4E3	hsa-miR-7398t-3p
EIF4E3	hsa-miR-522-3p
EIF4E3	hsa-miR-7158-3p
EIF4E3	hsa-miR-8908e
EIF4E3	hsa-miR-1544b-5p
EIF4E3	hsa-miR-2421
EIF4E3	hsa-miR-132-5p
EIF4E3	hsa-miR-2361
EIF4E3	hsa-miR-667-3p
EIF4E3	hsa-miR-1273g-5p
EIF4E3	hsa-miR-4787-5p
EIF4E3	hsa-miR-1260a
EIF4E3	hsa-miR-2888
EIF4E3	hsa-miR-141-3p
EIF4E3	hsa-miR-2325
EIF4E3	hsa-miR-6975-3p
EIF4E3	hsa-miR-4756-3p
EIF4E3	hsa-miR-1702
EIF4E3	hsa-miR-883ab-3p
EIF4E3	hsa-miR-222-5p
EIF4E3	hsa-miR-483-3p
EIF4E3	hsa-miR-550a-3p
EIF4E3	hsa-miR-96-5p
EIF4E3	hsa-miR-1284
EIF4E3	hsa-miR-6775-3p
EIF4E3	hsa-miR-3470b
EIF4E3	hsa-miR-292b-3p
EIF4E3	hsa-miR-342-3p
EIF4E3	hsa-miR-4446-3p
EIF4E3	hsa-miR-3084-5p
EIF4E3	hsa-miR-21-5p
EIF4E3	hsa-miR-6654-3p
EIF4E3	hsa-miR-3103-5p
EIF4E3	hsa-miR-6869-5p
EIF4E3	hsa-miR-2387

EIF4E3	hsa-miR-545-5p
EIF4E3	hsa-miR-603
EIF4E3	hsa-miR-6964-5p
EIF4E3	hsa-miR-6999-3p
EIF4E3	hsa-miR-384-3p
EIF4E3	hsa-miR-6791-5p
EIF4E3	hsa-miR-544a
EIF4E3	hsa-miR-6948-5p
EIF4E3	hsa-miR-3663-3p
EIF4E3	hsa-miR-1938
EIF4E3	hsa-miR-4663
EIF4E3	hsa-miR-743b-3p
EIF4E3	hsa-miR-6401
EIF4E3	hsa-miR-1225
EIF4E3	hsa-miR-575
EIF4E3	hsa-miR-6882-5p
EIF4E3	hsa-miR-4446-5p
EIF4E3	hsa-miR-2370-5p
EIF4E3	hsa-miR-499b-5p
EIF4E3	hsa-miR-2386
EIF4E3	hsa-miR-7312-3p
EIF4E3	hsa-miR-890
EIF4E3	hsa-miR-3577-5p
EIF4E3	hsa-miR-7352-3p
EIF4E3	hsa-miR-760-5p
EIF4E3	hsa-miR-679-3p
EIF4E3	hsa-miR-7863
EIF4E3	hsa-miR-6523b
EIF4E3	hsa-miR-548bb-3p
EIF4E3	hsa-miR-4639-5p
EIF4E3	hsa-miR-1911-5p
EIF4E3	hsa-miR-6122-5p
EIF4E3	hsa-miR-196-5p
EIF4E3	hsa-miR-126-3p
EIF4E3	hsa-miR-6927-5p
EIF4E3	hsa-miR-106-3p
EIF4E3	hsa-miR-892a
EIF4E3	hsa-miR-26a-5p
EIF4E3	hsa-miR-7353-3p
EIF4E3	hsa-miR-10b-5p
EIF4E3	hsa-miR-655-3p
EIF4E3	hsa-miR-421-5p
EIF4E3	hsa-miR-3088-3p
EIF4E3	hsa-miR-154-5p
EIF4E3	hsa-miR-7166-3p
EIF4E3	hsa-miR-4720-5p

EIF4E3	hsa-miR-9-5p
EIF4E3	hsa-miR-6986-5p
EIF4E3	hsa-miR-31-5p
EIF4E3	hsa-miR-381-3p
EIF4E3	hsa-miR-2344
EIF4E3	hsa-miR-6864-5p
EIF4E3	hsa-miR-8842
EIF4E3	hsa-miR-548ad-5p
EIF4E3	hsa-miR-1943-5p
EIF4E3	hsa-miR-516-3p
EIF4E3	hsa-miR-6716-3p
EIF4E3	hsa-miR-7211-3p
EIF4E3	hsa-miR-384-5p
EIF4E3	hsa-miR-873b
EIF4E3	hsa-miR-7256-5p
EIF4E3	hsa-miR-501-5p
EIF4E3	hsa-miR-292-5p
EIF4E3	hsa-miR-449-5p
EIF4E3	hsa-miR-2372
EIF4E3	hsa-miR-2399-5p
EIF4E3	hsa-miR-127-5p
EIF4E3	hsa-miR-8103
EIF4E3	hsa-miR-5571-3p
EIF4E3	hsa-miR-383-3p
EIF4E3	hsa-miR-6077
EIF4E3	hsa-let-7-3p
EIF4E3	hsa-miR-4462
EIF4E3	hsa-miR-8845
EIF4E3	hsa-miR-2405
EIF4E3	hsa-miR-7027-5p
EIF4E3	hsa-miR-6759-3p
EIF4E3	hsa-miR-6715-3p
EIF4E3	hsa-miR-4273
EIF4E3	hsa-miR-2284aa
EIF4E3	hsa-miR-6119-3p
EIF4E3	hsa-miR-545-3p
EIF4E3	hsa-miR-6776-5p
EIF4E3	hsa-miR-880-5p
EIF4E3	hsa-miR-187-3p
EIF4E3	hsa-miR-433-3p
EIF4E3	hsa-miR-26b-5p
EIF4E3	hsa-miR-7386m-5p
EIF4E3	hsa-miR-3962
EIF4E3	hsa-miR-1629
EIF4E3	hsa-miR-532-5p
EIF4E3	hsa-miR-7088-3p

EIF4E3	hsa-miR-3080-5p
EIF4E3	hsa-miR-139-5p
EIF4E3	hsa-miR-8058
EIF4E3	hsa-miR-669n
EIF4E3	hsa-miR-7117-3p
EIF4E3	hsa-miR-4714-5p
EIF4E3	hsa-miR-96-5p
EIF4E3	hsa-miR-8846
EIF4E3	hsa-miR-8904b
EIF4E3	hsa-let-7-5p
EIF4E3	hsa-miR-653
EIF4E3	hsa-miR-5627-5p
EIF4E3	hsa-miR-7152-5p
EIF4E3	hsa-miR-4661-3p
EIF4E3	hsa-miR-6935-3p
EIF4E3	hsa-miR-3133
EIF4E3	hsa-miR-3068-5p
EIF4E3	hsa-miR-6403
EIF4E3	hsa-miR-2887
EIF4E3	hsa-miR-759
EIF4E3	hsa-miR-5046
EIF4E3	hsa-miR-7669-5p
EIF4E3	hsa-miR-654-5p
EIF4E3	hsa-miR-467a-5p
EIF4E3	hsa-miR-4474-5p
EIF4E3	hsa-miR-7160-5p
EIF4E3	hsa-miR-7218-3p
EIF4E3	hsa-miR-6511a-5p
EIF4E3	hsa-miR-34a-3p
EIF4E3	hsa-miR-4464
EIF4E3	hsa-miR-718
EIF4E3	hsa-miR-7208-3p
EIF4E3	hsa-miR-105b
EIF4E3	hsa-miR-6962-3p
EIF4E3	hsa-miR-3957
EIF4E3	hsa-miR-7272-5p
EIF4E3	hsa-miR-7856-5p
EIF4E3	hsa-miR-670-5p
EIF4E3	hsa-miR-2368-5p
EIF4E3	hsa-miR-520gh
EIF4E3	hsa-miR-188-5p
EIF4E3	hsa-miR-4256
EIF4E3	hsa-miR-4435
EIF4E3	hsa-miR-3079-3p
EIF4E3	hsa-miR-3659
EIF4E3	hsa-miR-610

EIF4E3	hsa-miR-591
EIF4E3	hsa-miR-3164
EIF4E3	hsa-miR-7178-3p
EIF4E3	hsa-miR-1913
EIF4E3	hsa-miR-134-5p
EIF4E3	hsa-miR-6391
EIF4E3	hsa-miR-450b-5p
EIF4E3	hsa-miR-129-5p
EIF4E3	hsa-miR-4255
EIF4E3	hsa-miR-6817-3p
EIF4E3	hsa-miR-7218-5p
EIF4E3	hsa-miR-92
EIF4E3	hsa-miR-8794
EIF4E3	hsa-miR-154c
EIF4E3	hsa-miR-669j
EIF4E3	hsa-miR-3912-5p
EIF4E3	hsa-miR-320a
EIF4E3	hsa-miR-3583-5p
EIF4E3	hsa-miR-3117-5p
EIF4E3	hsa-miR-465a-5p
EIF4E3	hsa-miR-8876
EIF4E3	hsa-miR-4269
EIF4E3	hsa-miR-470-5p
EIF4E3	hsa-miR-326-3p
EIF4E3	hsa-miR-1249-5p
EIF4E3	hsa-miR-383-5p
EIF4E3	hsa-miR-15-5p
EIF4E3	hsa-miR-101a-3p.2
EIF4E3	hsa-miR-1910-5p
EIF4E3	hsa-miR-8836
EIF4E3	hsa-miR-548i
EIF4E3	hsa-miR-6322
EIF4E3	hsa-miR-7191-5p
EIF4E3	hsa-miR-557
EIF4E3	hsa-miR-6414
EIF4E3	hsa-miR-6979-3p
EIF4E3	hsa-miR-124c-5p
EIF4E3	hsa-miR-1750
EIF4E3	hsa-miR-888-3p
EIF4E3	hsa-miR-16-3p
EIF4E3	hsa-miR-412-3p
EIF4E3	hsa-miR-7077-5p
EIF4E3	hsa-miR-7214-5p
EIF4E3	hsa-miR-6326
EIF4E3	hsa-miR-365-3p
EIF4E3	hsa-miR-630

EIF4E3	hsa-miR-125a-3p
EIF4E3	hsa-miR-3201
EIF4E3	hsa-miR-1711
EIF4E3	hsa-miR-378
EIF4E3	hsa-miR-5623-5p
EIF4E3	hsa-miR-8118
EIF4E3	hsa-miR-376a-3p
EIF4E3	hsa-miR-3677-5p
EIF4E3	hsa-miR-3921
EIF4E3	hsa-miR-510
EIF4E3	hsa-miR-411-5p
EIF4E3	hsa-miR-8887
EIF4E3	hsa-miR-8117
EIF4E3	hsa-miR-7013-3p
EIF4E3	hsa-miR-6968-5p
EIF4E3	hsa-miR-6803-5p
EIF4E3	hsa-miR-3085-5p
EIF4E3	hsa-miR-548
EIF4E3	hsa-miR-506-3p
EIF4E3	hsa-miR-4329
EIF4E3	hsa-miR-7186-5p
EIF4E3	hsa-miR-2454-5p
EIF4E3	hsa-miR-30ad-3p
EIF4E3	hsa-miR-6833-3p
EIF4E3	hsa-miR-467f
EIF4E3	hsa-miR-1241
EIF4E3	hsa-miR-6817-5p
EIF4E3	hsa-miR-8081
EIF4E3	hsa-miR-4772-3p
EIF4E3	hsa-miR-6754-3p
EIF4E3	hsa-miR-2416
EIF4E3	hsa-miR-802-5p
EIF4E3	hsa-miR-1251
EIF4E3	hsa-miR-2474
EIF4E3	hsa-miR-16c-3p
EIF4E3	hsa-miR-2284k
EIF4E3	hsa-miR-340-3p
EIF4E3	hsa-miR-511-3p
EIF4E3	hsa-miR-15b-3p
EIF4E3	hsa-miR-148a-5p
EIF4E3	hsa-miR-6809-3p
EIF4E3	hsa-miR-6381
EIF4E3	hsa-miR-6770-5p
EIF4E3	hsa-miR-6895-3p
EIF4E3	hsa-miR-10a-3p
EIF4E3	hsa-miR-3689b-5p

EIF4E3	hsa-miR-1306-3p
EIF4E3	hsa-miR-1260b
EIF4E3	hsa-miR-5701
EIF4E3	hsa-miR-7228-3p
EIF4E3	hsa-miR-191-5p
EIF4E3	hsa-miR-763
EIF4E3	hsa-miR-200bc-3p
EIF4E3	hsa-miR-3080-3p
EIF4E3	hsa-miR-410-5p
EIF4E3	hsa-miR-518d-5p
EIF4E3	hsa-miR-505-5p
EIF4E3	hsa-miR-4307
EIF4E3	hsa-miR-7398k-5p
EIF4E3	hsa-miR-142-5p
EIF4E3	hsa-miR-5108
EIF4E3	hsa-miR-7295-3p
EIF4E3	hsa-miR-5009-5p
EIF4E3	hsa-miR-373-3p
EIF4E3	hsa-miR-4685-5p
EIF4E3	hsa-miR-7685-5p
EIF4E3	hsa-miR-379-3p
EIF4E3	hsa-miR-1630
EIF4E3	hsa-miR-6851-3p
EIF4E3	hsa-miR-4423-3p
EIF4E3	hsa-miR-7028-5p
EIF4E3	hsa-miR-1542-3p
EIF4E3	hsa-miR-140-5p
EIF4E3	hsa-miR-766-3p
EIF4E3	hsa-miR-6984-5p
EIF4E3	hsa-miR-2323
EIF4E3	hsa-miR-5584-3p
EIF4E3	hsa-miR-669o-5p
EIF4E3	hsa-miR-2314
EIF4E3	hsa-miR-6720-5p
EIF4E3	hsa-miR-181-5p
EIF4E3	hsa-miR-6866-5p
EIF4E3	hsa-miR-613
EIF4E3	hsa-miR-151-3p
EIF4E3	hsa-miR-1188-5p
EIF4E3	hsa-miR-599
EIF4E3	hsa-miR-8908-3p
EIF4E3	hsa-miR-6500-3p
EIF4E3	hsa-miR-5681a
EIF4E3	hsa-miR-1253
EIF4E3	hsa-miR-7208-5p
EIF4E3	hsa-miR-7684-3p

EIF4E3	hsa-miR-3066-3p
EIF4E3	hsa-miR-6747-5p
EIF4E3	hsa-miR-4533
EIF4E3	hsa-miR-4272
EIF4E3	hsa-miR-7386m-3p
EIF4E3	hsa-miR-6914-3p
EIF4E3	hsa-miR-383-5p.2
EIF4E3	hsa-miR-1545c-3p
EIF4E3	hsa-miR-7159-3p
EIF4E3	hsa-miR-7354-3p
EIF4E3	hsa-miR-9500
EIF4E3	hsa-miR-8104
EIF4E3	hsa-miR-338-5p
EIF4E3	hsa-miR-7262b-5p
EIF4E3	hsa-miR-648
EIF4E3	hsa-miR-7373-5p
EIF4E3	hsa-miR-4426
EIF4E3	hsa-miR-1745
EIF4E3	hsa-miR-101-3p
EIF4E3	hsa-miR-6896-3p
EIF4E3	hsa-miR-1245-3p
EIF4E3	hsa-miR-891-5p
EIF4E3	hsa-miR-429
EIF4E3	hsa-miR-7061-5p
EIF4E3	hsa-miR-3580-5p
EIF4E3	hsa-miR-5011-5p
EIF4E3	hsa-miR-7006-3p
EIF4E3	hsa-miR-183-3p
EIF4E3	hsa-miR-883-5p
EIF4E3	hsa-miR-1764-3p
EIF4E3	hsa-miR-3544-5p
EIF4E3	hsa-miR-669k-5p
EIF4E3	hsa-miR-7-5p
EIF4E3	hsa-miR-2466-5p
EIF4E3	hsa-miR-7305-5p
EIF4E3	hsa-miR-7370-5p
EIF4E3	hsa-miR-669c-5p
EIF4E3	hsa-miR-185-3p
EIF4E3	hsa-miR-6913-3p
EIF4E3	hsa-miR-6388
EIF4E3	hsa-miR-669e-5p
EIF4E3	hsa-miR-504-5p
EIF4E3	hsa-miR-551b-5p
EIF4E3	hsa-miR-674-3p
EIF4E3	hsa-miR-217-3p
EIF4E3	hsa-miR-411-3p

EIF4E3	hsa-miR-137-3p
EIF4E3	hsa-miR-1967
EIF4E3	hsa-miR-425-5p
EIF4E3	hsa-miR-6997-5p
EIF4E3	hsa-miR-2367-5p
EIF4E3	hsa-miR-1240
EIF4E3	hsa-miR-666-3p
EIF4E3	hsa-miR-362-5p
EIF4E3	hsa-miR-6515-3p
EIF4E3	hsa-miR-526b-5p
EIF4E3	hsa-miR-7a-1-3p
EIF4E3	hsa-miR-1291
EIF4E3	hsa-miR-200c-5p
EIF4E3	hsa-miR-6878-5p
EIF4E3	hsa-miR-2681-5p
EIF4E3	hsa-miR-516b
EIF4E3	hsa-miR-3473c
EIF4E3	hsa-miR-1643b-5p
EIF4E3	hsa-miR-7386k-5p
EIF4E3	hsa-miR-2374
EIF4E3	hsa-miR-208a-3p
EIF4E3	hsa-miR-7285-3p
EIF4E3	hsa-miR-505-3p.1
EIF4E3	hsa-miR-196
EIF4E3	hsa-miR-2313-3p
EIF4E3	hsa-miR-592-3p
EIF4E3	hsa-miR-4724-3p
EIF4E3	hsa-miR-597-3p
EIF4E3	hsa-miR-6878-3p
EIF4E3	hsa-miR-6647-5p
EIF4E3	hsa-miR-4640-5p
EIF4E3	hsa-miR-1287-5p
EIF4E3	hsa-miR-7075-5p
EIF4E3	hsa-miR-669h-5p
EIF4E3	hsa-miR-659-3p
EIF4E3	hsa-miR-30d-3p
EIF4E3	hsa-miR-508-3p
EIF4E3	hsa-miR-5112
EIF4E3	hsa-miR-499-5p
EIF4E3	hsa-miR-454-3p
EIF4E3	hsa-miR-4797-3p
EIF4E3	hsa-miR-207
EIF4E3	hsa-miR-4682
EIF4E3	hsa-miR-1776
EIF4E3	hsa-miR-3090-3p
EIF4E3	hsa-miR-1233-3p

EIF4E3	hsa-miR-497-5p
EIF4E3	hsa-miR-7052-5p
EIF4E3	hsa-miR-5583-5p
EIF4E3	hsa-miR-7063-3p
EIF4E3	hsa-miR-676-3p
EIF4E3	hsa-miR-30c-2-3p
EIF4E3	hsa-miR-216a-3p
EIF4E3	hsa-miR-3967
EIF4E3	hsa-miR-3200-5p
EIF4E3	hsa-miR-767
EIF4E3	hsa-miR-8829
EIF4E3	hsa-miR-7072-3p
EIF4E3	hsa-miR-2284z
EIF4E3	hsa-miR-150-5p
EIF4E3	hsa-miR-673-5p
EIF4E3	hsa-miR-124-3p.1
EIF4E3	hsa-miR-2054
EIF4E3	hsa-miR-186-5p
EIF4E3	hsa-miR-7117-5p
EIF4E3	hsa-miR-550b-2-5p
EIF4E3	hsa-miR-3108-5p
EIF4E3	hsa-miR-106-5p
EIF4E3	hsa-miR-5620-3p
EIF4E3	hsa-miR-7030-3p
EIF4E3	hsa-miR-7684-5p
EIF4E3	hsa-miR-2406
EIF4E3	hsa-miR-6899-3p
EIF4E3	hsa-miR-135
EIF4E3	hsa-miR-1236-3p
EIF4E3	hsa-miR-1298-3p
EIF4E3	hsa-miR-518df-3p
EIF4E3	hsa-miR-7010-3p
EIF4E3	hsa-miR-517-5p
EIF4E3	hsa-miR-589-5p
EIF4E3	hsa-miR-6970-5p
EIF4E3	hsa-miR-200b-5p
EIF4E3	hsa-miR-6752-5p
EIF4E3	hsa-miR-95-3p
EIF4E3	hsa-miR-4795-3p
EIF4E3	hsa-miR-7-5p
EIF4E3	hsa-miR-4690-5p
EIF4E3	hsa-miR-702-5p
EIF4E3	hsa-miR-539-3p
EIF4E3	hsa-miR-514b-5p
EIF4E3	hsa-miR-758-3p
EIF4E3	hsa-miR-4685-3p

EIF4E3	hsa-miR-670-3p
EIF4E3	hsa-miR-181
EIF4E3	hsa-miR-518-3p
EIF4E3	hsa-miR-6941-5p
EIF4E3	hsa-miR-550-5p
EIF4E3	hsa-miR-4652-3p
EIF4E3	hsa-miR-1603
EIF4E3	hsa-miR-5697
EIF4E3	hsa-miR-6362
EIF4E3	hsa-miR-1958
EIF4E3	hsa-miR-302a-5p
EIF4E3	hsa-miR-1728-3p
EIF4E3	hsa-miR-3145-5p
EIF4E3	hsa-miR-1964-5p
EIF4E3	hsa-miR-4668-3p
EIF4E3	hsa-miR-548h-5p
EIF4E3	hsa-miR-32-5p
EIF4E3	hsa-miR-513c-3p
EIF4E3	hsa-miR-8872
EIF4E3	hsa-miR-292a-3p
EIF4E3	hsa-miR-350-3p
EIF4E3	hsa-miR-4766-3p
EIF4E3	hsa-miR-3140-5p
EIF4E3	hsa-miR-876
EIF4E3	hsa-miR-6076
EIF4E3	hsa-miR-7342-5p
EIF4E3	hsa-miR-6997-3p
EIF4E3	hsa-miR-4768-5p
EIF4E3	hsa-miR-3065-5p
EIF4E3	hsa-miR-183-5p
EIF4E3	hsa-miR-8791
EIF4E3	hsa-miR-7094b-2-5p
EIF4E3	hsa-miR-5006-3p
EIF4E3	hsa-miR-6805-5p
EIF4E3	hsa-miR-3200-3p
EIF4E3	hsa-miR-467b-5p
EIF4E3	hsa-miR-451b
EIF4E3	hsa-miR-7043-3p
EIF4E3	hsa-miR-491-5p
EIF4E3	hsa-miR-576-5p
EIF4E3	hsa-miR-4652-5p
EIF4E3	hsa-miR-8865
EIF4E3	hsa-miR-206
EIF4E3	hsa-miR-154-3p
EIF4E3	hsa-miR-20a-3p
EIF4E3	hsa-miR-5007-3p

EIF4E3	hsa-miR-518a-5p
EIF4E3	hsa-miR-7000-3p
EIF4E3	hsa-miR-3071-5p
EIF4E3	hsa-miR-196a-3p
EIF4E3	hsa-miR-3191-5p
EIF4E3	hsa-miR-8904a
EIF4E3	hsa-miR-1295b-5p
EIF4E3	hsa-miR-181c-3p
EIF4E3	hsa-miR-1558
EIF4E3	hsa-miR-6989-5p
EIF4E3	hsa-miR-4434
EIF4E3	hsa-miR-361-5p
EIF4E3	hsa-miR-8847
EIF4E3	hsa-miR-1668-3p
EIF4E3	hsa-miR-3561-5p
EIF4E3	hsa-miR-137
EIF4E3	hsa-miR-6514-5p
EIF4E3	hsa-miR-8065
EIF4E3	hsa-miR-7226-5p
EIF4E3	hsa-miR-3472
EIF4E3	hsa-miR-881-3p
EIF4E3	hsa-miR-922
EIF4E3	hsa-miR-6985-5p
EIF4E3	hsa-miR-4646-5p
EIF4E3	hsa-miR-3089-3p
EIF4E3	hsa-miR-1839
EIF4E3	hsa-miR-143-3p
EIF4E3	hsa-miR-133-5p
EIF4E3	hsa-miR-1927
EIF4E3	hsa-miR-2470
EIF4E3	hsa-miR-4495
EIF4E3	hsa-miR-1584-5p
EIF4E3	hsa-miR-519d-5p
EIF4E3	hsa-miR-6071
EIF4E3	hsa-miR-6119-5p
EIF4E3	hsa-miR-939-5p
EIF4E3	hsa-miR-6525
EIF4E3	hsa-miR-139-3p
EIF4E3	hsa-miR-3913-5p
EIF4E3	hsa-miR-4791
EIF4E3	hsa-miR-4732-3p
EIF4E3	hsa-miR-1715-5p
EIF4E3	hsa-miR-1981-5p
EIF4E3	hsa-miR-4717-3p
EIF4E3	hsa-miR-593-3p
EIF4E3	hsa-miR-1589

EIF4E3	hsa-miR-1955-3p
EIF4E3	hsa-miR-4432
EIF4E3	hsa-miR-548an
EIF4E3	hsa-miR-5692a
EIF4E3	hsa-miR-693-5p
EIF4E3	hsa-miR-7663-5p
EIF4E3	hsa-miR-3473
EIF4E3	hsa-miR-6862-3p
EIF4E3	hsa-miR-548as-3p
EIF4E3	hsa-miR-1193-3p
EIF4E3	hsa-miR-376a-2-5p
EIF4E3	hsa-miR-7093-3p
EIF4E3	hsa-miR-709
EIF4E3	hsa-miR-2408
EIF4E3	hsa-miR-8863
EIF4E3	hsa-miR-370-5p
EIF4E3	hsa-miR-2284s
EIF4E3	hsa-miR-3105-3p
EIF4E3	hsa-miR-7334-5p
EIF4E3	hsa-miR-3076-5p
EIF4E3	hsa-miR-2350
EIF4E3	hsa-miR-466f-5p
EIF4E3	hsa-miR-335-5p
EIF4E3	hsa-miR-6332
EIF4E3	hsa-miR-7398i-3p
EIF4E3	hsa-miR-4481
EIF4E3	hsa-miR-6909-3p
EIF4E3	hsa-miR-7232-5p
EIF4E3	hsa-miR-4274
EIF4E3	hsa-miR-2284m
EIF4E3	hsa-miR-1-3p
EIF4E3	hsa-miR-6973b-5p
EIF4E3	hsa-miR-4715-3p
EIF4E3	hsa-miR-6862-5p
EIF4E3	hsa-miR-1544-3p
EIF4E3	hsa-miR-7347-3p
EIF4E3	hsa-miR-1252-3p
EIF4E3	hsa-miR-2329-3p
EIF4E3	hsa-miR-216c-5p
EIF4E3	hsa-miR-3590-5p
EIF4E3	hsa-miR-674-5p
EIF4E3	hsa-miR-5581-3p
EIF4E3	hsa-miR-301a-5p
EIF4E3	hsa-miR-2285af
EIF4E3	hsa-miR-8835
EIF4E3	hsa-miR-4745-5p

EIF4E3	hsa-miR-582-3p
EIF4E3	hsa-miR-466aep-5p
EIF4E3	hsa-miR-6858-3p
EIF4E3	hsa-miR-1255
EIF4E3	hsa-miR-7397-3p
EIF4E3	hsa-miR-6810-3p
EIF4E3	hsa-miR-6995-3p
EIF4E3	hsa-miR-3188
EIF4E3	hsa-miR-4451
EIF4E3	hsa-miR-7252-5p
EIF4E3	hsa-miR-3925-3p
EIF4E3	hsa-miR-296-5p
EIF4E3	hsa-miR-7009-3p
EIF4E3	hsa-miR-694
EIF4E3	hsa-miR-2400
EIF4E3	hsa-miR-6409
EIF4E3	hsa-miR-4749-3p
EIF4E3	hsa-miR-149-5p
EIF4E3	hsa-miR-365-5p
EIF4E3	hsa-miR-599-5p
EIF4E3	hsa-miR-6404
EIF4E3	hsa-miR-7015-5p
EIF4E3	hsa-miR-6939-3p
EIF4E3	hsa-miR-580
EIF4E3	hsa-miR-1255b-2-3p
EIF4E3	hsa-miR-1571
EIF4E3	hsa-miR-6992-5p
EIF4E3	hsa-miR-3070-2-3p
EIF4E3	hsa-miR-297-5p
EIF4E3	hsa-miR-15
EIF4E3	hsa-miR-1275
EIF4E3	hsa-miR-548al
EIF4E3	hsa-miR-7195-3p
EIF4E3	hsa-miR-7060-3p
EIF4E3	hsa-miR-6721-5p
EIF4E3	hsa-miR-6880-5p
EIF4E3	hsa-miR-7275-5p
EIF4E3	hsa-miR-7373a-3p
EIF4E3	hsa-miR-351-5p
EIF4E3	hsa-miR-490-5p
EIF4E3	hsa-miR-7000-5p
EIF4E3	hsa-miR-4731-5p
EIF4E3	hsa-miR-466c-5p
EIF4E3	hsa-miR-4775
EIF4E3	hsa-miR-374b-5p
EIF4E3	hsa-miR-6776-3p

EIF4E3	hsa-miR-769
EIF4E3	hsa-miR-4716-3p
EIF4E3	hsa-miR-6837-3p
EIF4E3	hsa-miR-548ab
EIF4E3	hsa-miR-328-5p
EIF4E3	hsa-miR-621
EIF4E3	hsa-miR-3569-3p
EIF4E3	hsa-miR-6600-3p
EIF4E3	hsa-miR-7028-3p
EIF4E3	hsa-miR-6072
EIF4E3	hsa-miR-6902-3p
EIF4E3	hsa-miR-549a
EIF4E3	hsa-miR-7384-3p
EIF4E3	hsa-miR-143-5p
EIF4E3	hsa-miR-7247-5p
EIF4E3	hsa-miR-125b-2-3p
EIF4E3	hsa-miR-7661-5p
EIF4E3	hsa-miR-2335
EIF4E3	hsa-miR-210
EIF4E3	hsa-miR-130
EIF4E3	hsa-miR-6953-5p
EIF4E3	hsa-miR-6848-5p
EIF4E3	hsa-miR-6894-5p
EIF4E3	hsa-miR-6802-5p
EIF4E3	hsa-miR-6768-5p
EIF4E3	hsa-miR-677
EIF4E3	hsa-miR-548h-3p
EIF4E3	hsa-miR-2328-3p
EIF4E3	hsa-miR-2475
EIF4E3	hsa-miR-101b-3p.2
EIF4E3	hsa-miR-6740-3p
EIF4E3	hsa-miR-34b-5p
EIF4E3	hsa-miR-466mo-3p
EIF4E3	hsa-miR-3587
EIF4E3	hsa-miR-136-5p
EIF4E3	hsa-miR-700-5p
EIF4E3	hsa-miR-377-3p
EIF4E3	hsa-miR-2285c
EIF4E3	hsa-miR-7330-3p
EIF4E3	hsa-miR-571
EIF4E3	hsa-miR-3120-3p
EIF4E3	hsa-miR-496
EIF4E3	hsa-miR-6888-3p
EIF4E3	hsa-miR-563
EIF4E3	hsa-miR-3061-3p
EIF4E3	hsa-miR-3105-5p

EIF4E3	hsa-miR-7081-5p
EIF4E3	hsa-miR-6323
EIF4E3	hsa-miR-7171-3p
EIF4E3	hsa-miR-3551-3p
EIF4E3	hsa-miR-344-3p
EIF4E3	hsa-miR-6954-5p
EIF4E3	hsa-miR-8801
EIF4E3	hsa-miR-7041-3p
EIF4E3	hsa-miR-1323-5p
EIF4E3	hsa-miR-95
EIF4E3	hsa-miR-19b-5p
EIF4E3	hsa-miR-6343
EIF4E3	hsa-miR-6543-3p
EIF4E3	hsa-miR-8080
EIF4E3	hsa-miR-4633-5p
EIF4E3	hsa-miR-617
EIF4E3	hsa-miR-496-5p
EIF4E3	hsa-miR-2183
EIF4E3	hsa-miR-7154-3p
EIF4E3	hsa-miR-544-5p
EIF4E3	hsa-miR-7371d-5p
EIF4E3	hsa-miR-6943-3p
EIF4E3	hsa-miR-6981-3p
EIF4E3	hsa-miR-7173-5p
EIF4E3	hsa-miR-3059-3p
EIF4E3	hsa-miR-3060-3p
EIF4E3	hsa-miR-208-5p
EIF4E3	hsa-miR-4423-5p
EIF4E3	hsa-miR-3171
EIF4E3	hsa-miR-770
EIF4E3	hsa-miR-6134
EIF4E3	hsa-miR-191-5p
EIF4E3	hsa-miR-5133
EIF4E3	hsa-miR-3607-3p
EIF4E3	hsa-miR-7655-3p
EIF4E3	hsa-miR-125
EIF4E3	hsa-miR-140-3p.1
EIF4E3	hsa-miR-495-5p
EIF4E3	hsa-miR-4499
EIF4E3	hsa-miR-517
EIF4E3	hsa-miR-1264-3p
EIF4E3	hsa-miR-2113
EIF4E3	hsa-miR-8843
EIF4E3	hsa-miR-1243-5p
EIF4E3	hsa-miR-3596c
EIF4E3	hsa-miR-873-5p

EIF4E3	hsa-miR-466q
EIF4E3	hsa-miR-300-5p
EIF4E3	hsa-miR-1968-3p
EIF4E3	hsa-miR-1272
EIF4E3	hsa-miR-5093
EIF4E3	hsa-miR-1902
EIF4E3	hsa-miR-6873-5p
EIF4E3	hsa-miR-1255b-5p
EIF4E3	hsa-miR-6083
EIF4E3	hsa-miR-687
EIF4E3	hsa-miR-7187-3p
EIF4E3	hsa-miR-3124-3p
EIF4E3	hsa-miR-7213-3p
EIF4E3	hsa-miR-1205
EIF4E3	hsa-miR-515-3p
EIF4E3	hsa-miR-4750-3p
EIF4E3	hsa-miR-376c-3p
EIF4E3	hsa-miR-872
EIF4E3	hsa-miR-4769-3p
EIF4E3	hsa-miR-644a
EIF4E3	hsa-miR-548bb-5p
EIF4E3	hsa-miR-8067
EIF4E3	hsa-miR-6995-5p
EIF4E3	hsa-miR-7386i-5p
EIF4E3	hsa-miR-4728-3p
EIF4E3	hsa-miR-2116-3p
EIF4E3	hsa-miR-7289-5p
EIF4E3	hsa-miR-7032-3p
EIF4E3	hsa-miR-3604
EIF4E3	hsa-miR-4665-3p
EIF4E3	hsa-miR-4672
EIF4E3	hsa-miR-500b-5p
EIF4E3	hsa-miR-6633-5p
EIF4E3	hsa-miR-6861-3p
EIF4E3	hsa-miR-7274-5p
EIF4E3	hsa-miR-4521
EIF4E3	hsa-miR-8073
EIF4E3	hsa-miR-6400
EIF4E3	hsa-miR-200a-3p
EIF4E3	hsa-miR-667-5p
EIF4E3	hsa-miR-6771-3p
EIF4E3	hsa-miR-7283-3p
EIF4E3	hsa-miR-496-3p
EIF4E3	hsa-miR-19b-3p
EIF4E3	hsa-miR-6554-3p
EIF4E3	hsa-miR-7-3p

EIF4E3	hsa-miR-8874
EIF4E3	hsa-miR-669b-5p
EIF4E3	hsa-miR-7316-5p
EIF4E3	hsa-miR-7331-2-3p
EIF4E3	hsa-let-7b-3p
EIF4E3	hsa-miR-6127
EIF4E3	hsa-miR-4503
EIF4E3	hsa-miR-548g-3p
EIF4E3	hsa-miR-7641
EIF4E3	hsa-miR-6974-5p
EIF4E3	hsa-miR-2284i
EIF4E3	hsa-miR-450b-3p
EIF4E3	hsa-miR-7973
EIF4E3	hsa-miR-4298
EIF4E3	hsa-let-7a-2-3p
EIF4E3	hsa-miR-29e
EIF4E3	hsa-miR-5627-3p
EIF4E3	hsa-miR-193b-5p
EIF4E3	hsa-miR-5709-3p
EIF4E3	hsa-miR-4778-5p
EIF4E3	hsa-miR-6946-3p
EIF4E3	hsa-miR-8908c
EIF4E3	hsa-miR-3605-5p
EIF4E3	hsa-miR-6977-5p
EIF4E3	hsa-miR-7074-3p
EIF4E3	hsa-miR-3613b
EIF4E3	hsa-miR-6947-5p
EIF4E3	hsa-miR-4720-3p
EIF4E3	hsa-miR-6876-3p
EIF4E3	hsa-miR-7286-3p
EIF4E3	hsa-miR-181-3p
EIF4E3	hsa-miR-519e-3p
EIF4E3	hsa-miR-3148
EIF4E3	hsa-miR-5580-3p
EIF4E3	hsa-miR-9a-3p
EIF4E3	hsa-miR-519-3p
EIF4E3	hsa-miR-647
EIF4E3	hsa-miR-182-3p
EIF4E3	hsa-miR-7242-5p
EIF4E3	hsa-miR-7365-3p
EIF4E3	hsa-miR-2284ac
EIF4E3	hsa-miR-5586-3p
EIF4E3	hsa-miR-1273d
EIF4E3	hsa-miR-1249-3p
EIF4E3	hsa-miR-218-5p
EIF4E3	hsa-miR-7691

EIF4E3	hsa-miR-6801-5p
EIF4E3	hsa-miR-4534
EIF4E3	hsa-miR-943
EIF4E3	hsa-miR-7336-5p
EIF4E3	hsa-miR-4659b-5p
EIF4E3	hsa-miR-371a-5p
EIF4E3	hsa-miR-6907-5p
EIF4E3	hsa-miR-4999-3p
EIF4E3	hsa-miR-3072-5p
EIF4E3	hsa-miR-19a-5p
EIF4E3	hsa-miR-7022-5p
EIF4E3	hsa-miR-892c-3p
EIF4E3	hsa-miR-365b-5p
EIF4E3	hsa-miR-2489
EIF4E3	hsa-miR-5011-3p
EIF4E3	hsa-miR-4474-3p
EIF4E3	hsa-miR-2486-3p
EIF4E3	hsa-miR-449c-3p
EIF4E3	hsa-miR-1842
EIF4E3	hsa-miR-875-5p
EIF4E3	hsa-miR-4760-3p
EIF4E3	hsa-miR-2303
EIF4E3	hsa-miR-6865-3p
EIF4E3	hsa-miR-486-5p
EIF4E3	hsa-miR-3609
EIF4E3	hsa-miR-628-5p
EIF4E3	hsa-miR-6804-3p
EIF4E3	hsa-miR-7054-5p
EIF4E3	hsa-miR-2299-5p
EIF4E3	hsa-miR-4422
EIF4E3	hsa-miR-8869
EIF4E3	hsa-miR-122-3p
EIF4E3	hsa-miR-7002-5p
EIF4E3	hsa-miR-452-5p
EIF4E3	hsa-miR-7324-3p
EIF4E3	hsa-miR-6350
EIF4E3	hsa-miR-7185-3p
EIF4E3	hsa-miR-4436b-3p
EIF4E3	hsa-miR-450a-2-3p
EIF4E3	hsa-miR-503-5p
EIF4E3	hsa-miR-3130-3p
EIF4E3	hsa-miR-2115-5p
EIF4E3	hsa-miR-2110
EIF4E3	hsa-miR-3110-3p
EIF4E3	hsa-miR-583
EIF4E3	hsa-miR-4788

EIF4E3	hsa-miR-673-3p
EIF4E3	hsa-miR-212-3p
EIF4E3	hsa-miR-200-3p
EIF4E3	hsa-miR-6953-3p
EIF4E3	hsa-miR-586
EIF4E3	hsa-miR-4666a-5p
EIF4E3	hsa-miR-3619-5p
EIF4E3	hsa-miR-324-3p
EIF4E3	hsa-miR-1897-5p
EIF4E3	hsa-miR-2320-5p
EIF4E3	hsa-miR-1983
EIF4E3	hsa-miR-200ac-5p
EIF4E3	hsa-miR-8119
EIF4E3	hsa-miR-16-5p
EIF4E3	hsa-miR-382-5p
EIF4E3	hsa-miR-6883-3p
EIF4E3	hsa-miR-5092
EIF4E3	hsa-miR-8853
EIF4E3	hsa-miR-298-3p
EIF4E3	hsa-miR-2412
EIF4E3	hsa-miR-3578
EIF4E3	hsa-miR-890-3p
EIF4E3	hsa-miR-302c-3p
EIF4E3	hsa-miR-3714
EIF4E3	hsa-miR-3159
EIF4E3	hsa-miR-4755-5p
EIF4E3	hsa-miR-2447
EIF4E3	hsa-miR-200
EIF4E3	hsa-miR-7277-5p
EIF4E3	hsa-miR-7386g-3p
EIF4E3	hsa-miR-510-3p
EIF4E3	hsa-miR-2898
EIF4E3	hsa-miR-8908f
EIF4E3	hsa-miR-7207-3p
EIF4E3	hsa-miR-377-5p
EIF4E3	hsa-miR-2425-5p
EIF4E3	hsa-miR-871-5p
EIF4E3	hsa-miR-6938-5p
EIF4E3	hsa-miR-4694-3p
EIF4E3	hsa-miR-467cd-5p
EIF4E3	hsa-miR-505-3p
EIF4E3	hsa-miR-186-5p
EIF4E3	hsa-miR-6733-5p
EIF4E3	hsa-miR-7156-5p
EIF4E3	hsa-miR-344-5p
EIF4E3	hsa-miR-7063-5p

EIF4E3	hsa-miR-488-5p
EIF4E3	hsa-miR-466-5p
EIF4E3	hsa-miR-450b
EIF4E3	hsa-miR-2114-3p
EIF4E3	hsa-miR-561-3p
EIF4E3	hsa-miR-4659b-3p
EIF4E3	hsa-miR-3058-3p
EIF4E3	hsa-miR-6917-5p
EIF4E3	hsa-miR-4421
EIF4E3	hsa-miR-707
EIF4E3	hsa-miR-9768-5p
EIF4E3	hsa-miR-6368
EIF4E3	hsa-miR-8893
EIF4E3	hsa-miR-597
EIF4E3	hsa-miR-509-3p
EIF4E3	hsa-miR-3100-3p
EIF4E3	hsa-miR-6912-3p
EIF4E3	hsa-miR-7051-3p
EIF4E3	hsa-miR-3165
EIF4E3	hsa-miR-6943-5p
EIF4E3	hsa-miR-2373-3p
EIF4E3	hsa-miR-6715b-3p
EIF4E3	hsa-miR-3572-3p
EIF4E3	hsa-miR-578
EIF4E3	hsa-miR-200a-5p
EIF4E3	hsa-miR-3689-3p
EIF4E3	hsa-miR-340-5p
EIF4E3	hsa-miR-2284x
EIF4E3	hsa-miR-548ac
EIF4E3	hsa-miR-760-3p
EIF4E3	hsa-miR-2285t
EIF4E3	hsa-miR-2284ab
EIF4E3	hsa-miR-548cej-3p
EIF4E3	hsa-miR-1556
EIF4E3	hsa-miR-7386c-5p
EIF4E3	hsa-miR-669
EIF4E3	hsa-miR-7083-3p
EIF4E3	hsa-miR-324
EIF4E3	hsa-miR-6131
EIF4E3	hsa-miR-5186
EIF4E3	hsa-miR-2896
EIF4E3	hsa-miR-6842-3p
EIF4E3	hsa-miR-758-5p
EIF4E3	hsa-miR-6529b
EIF4E3	hsa-miR-582-5p
EIF4E3	hsa-miR-3594-5p

EIF4E3	hsa-miR-1643a-5p
EIF4E3	hsa-miR-7217-5p
EIF4E3	hsa-miR-4731-3p
EIF4E3	hsa-miR-6751-5p
EIF4E3	hsa-miR-205-5p
EIF4E3	hsa-miR-198
EIF4E3	hsa-miR-3099
EIF4E3	hsa-miR-2285z
EIF4E3	hsa-miR-4661-5p
EIF4E3	hsa-miR-1197-3p
EIF4E3	hsa-miR-548l
EIF4E3	hsa-miR-7210-5p
EIF4E3	hsa-miR-1322
EIF4E3	hsa-miR-4443
EIF4E3	hsa-miR-8056
EIF4E3	hsa-miR-7466-3p
EIF4E3	hsa-miR-659-5p
EIF4E3	hsa-miR-222-3p
EIF4E3	hsa-miR-4539
EIF4E3	hsa-miR-200b-3p
EIF4E3	hsa-miR-876-5p
EIF4E3	hsa-miR-6902-5p
EIF4E3	hsa-miR-889-5p
EIF4E3	hsa-miR-331-3p
EIF4E3	hsa-miR-3577
EIF4E3	hsa-miR-202-5p
EIF4E3	hsa-miR-548d-3p
EIF4E3	hsa-miR-7360-5p
EIF4E3	hsa-miR-881-5p
EIF4E3	hsa-miR-4687-5p
EIF4E3	hsa-miR-7672-5p
EIF4E3	hsa-miR-7168-3p
EIF4E3	hsa-miR-196a-2-3p
EIF4E3	hsa-miR-1912-5p
EIF4E3	hsa-miR-4802-5p
EIF4E3	hsa-miR-6416-3p
EIF4E3	hsa-miR-4473
EIF4E3	hsa-miR-7052-3p
EIF4E3	hsa-miR-4475
EIF4E3	hsa-miR-500-5p
EIF4E3	hsa-miR-7280-3p
EIF4E3	hsa-miR-1304-3p
EIF4E3	hsa-miR-7175-5p
EIF4E3	hsa-miR-4438
EIF4E3	hsa-miR-302-3p
EIF4E3	hsa-miR-6750-5p

EIF4E3	hsa-miR-7012-5p
EIF4E3	hsa-miR-144-3p
EIF4E3	hsa-miR-5193
EIF4E3	hsa-miR-323a-5p
EIF4E3	hsa-miR-1459
EIF4E3	hsa-miR-4753-5p
EIF4E3	hsa-miR-1910-3p
EIF4E3	hsa-miR-365a-3p
EIF4E3	hsa-miR-7456-5p
EIF4E3	hsa-miR-101-3p
EIF4E3	hsa-miR-467g
EIF4E3	hsa-miR-6568-3p
EIF4E3	hsa-miR-3062-5p
EIF4E3	hsa-miR-7666-3p
EIF4E3	hsa-miR-892-3p
EIF4E3	hsa-miR-5130
EIF4E3	hsa-miR-500-3p
EIF4E3	hsa-miR-616-5p
EIF4E3	hsa-miR-580-5p
EIF4E3	hsa-miR-7164-5p
EIF4E3	hsa-miR-3617-3p
EIF4E3	hsa-miR-4742
EIF4E3	hsa-miR-7374-5p
EIF4E3	hsa-miR-290
EIF4E3	hsa-miR-34
EIF4E3	hsa-miR-6768-3p
EIF4E3	hsa-miR-526
EIF4E3	hsa-miR-3057-3p
EIF4E3	hsa-miR-7667-3p
EIF4E3	hsa-miR-101-3p.1
EIF4E3	hsa-miR-95-5p
EIF4E3	hsa-miR-3679-3p
EIF4E3	hsa-miR-1266-3p
EIF4E3	hsa-miR-669hk-3p
EIF4E3	hsa-miR-686
EIF4E3	hsa-miR-6945-3p
EIF4E3	hsa-miR-5583-3p
EIF4E3	hsa-miR-301-3p
EIF4E3	hsa-miR-6972-5p
EIF4E3	hsa-miR-6853-3p
EIF4E3	hsa-miR-302d-5p
EIF4E3	hsa-miR-7109-3p
EIF4E3	hsa-miR-6836-3p
EIF4E3	hsa-miR-1906
EIF4E3	hsa-miR-885
EIF4E3	hsa-miR-4758-5p

EIF4E3	hsa-miR-383
EIF4E3	hsa-miR-34c-3p
EIF4E3	hsa-miR-4680
EIF4E3	hsa-miR-1283a
EIF4E3	hsa-miR-1302
EIF4E3	hsa-miR-432-3p
EIF4E3	hsa-miR-7344-5p
EIF4E3	hsa-miR-1178-5p
EIF4E3	hsa-miR-7386j-3p
EIF4E3	hsa-miR-6074
EIF4E3	hsa-miR-28-3p
EIF4E3	hsa-miR-3185
EIF4E3	hsa-miR-6885-5p
EIF4E3	hsa-miR-651-3p
EIF4E3	hsa-miR-491-5p
EIF4E3	hsa-miR-376b-5p
EIF4E3	hsa-miR-875-3p
EIF4E3	hsa-miR-6529-3p
EIF4E3	hsa-miR-5572
EIF4E3	hsa-miR-381-5p
EIF4E3	hsa-miR-302f
EIF4E3	hsa-miR-6955-3p
EIF4E3	hsa-miR-548x-5p
EIF4E3	hsa-miR-7978
EIF4E3	hsa-miR-1303
EIF4E3	hsa-miR-148a-3p
EIF4E3	hsa-miR-518-5p
EIF4E3	hsa-miR-4695-3p
EIF4E3	hsa-miR-7463-3p
EIF4E3	hsa-miR-329a
EIF4E3	hsa-miR-3612
EIF4E3	hsa-miR-98-5p
EIF4E3	hsa-miR-378b
EIF4E3	hsa-miR-6802-3p
EIF4E3	hsa-miR-7087-5p
EIF4E3	hsa-miR-7363-5p
EIF4E3	hsa-miR-4659a-3p
EIF4E3	hsa-miR-552-5p
EIF4E3	hsa-miR-302bd
EIF4E3	hsa-miR-19-3p
EIF4E3	hsa-miR-3924
EIF4E3	hsa-miR-637
EIF4E3	hsa-miR-208b-3p
EIF4E3	hsa-miR-1229-3p
EIF4E3	hsa-miR-32-3p
EIF4E3	hsa-miR-7064-3p

EIF4E3	hsa-miR-7319-3p
EIF4E3	hsa-miR-6837-5p
EIF4E3	hsa-miR-27
EIF4E3	hsa-miR-6874-3p
EIF4E3	hsa-miR-3572
EIF4E3	hsa-miR-627-5p
EIF4E3	hsa-miR-6764-3p
EIF4E3	hsa-miR-196-3p
EIF4E3	hsa-miR-496.2
EIF4E3	hsa-miR-497-5p
EIF4E3	hsa-miR-504-3p
EIF4E3	hsa-miR-492
EIF4E3	hsa-miR-7159-5p
EIF4E3	hsa-miR-680
EIF4E3	hsa-miR-539-5p
EIF4E3	hsa-miR-7681-3p
EIF4E3	hsa-miR-4778-3p
EIF4E3	hsa-miR-6928-5p
EIF4E3	hsa-miR-361-5p
EIF4E3	hsa-miR-434-3p
EIF4E3	hsa-miR-7681-5p
EIF4E3	hsa-miR-1932
EIF4E3	hsa-miR-876-3p
EIF4E3	hsa-miR-8824
EIF4E3	hsa-miR-7297-5p
EIF4E3	hsa-miR-3965
EIF4E3	hsa-miR-1732
EIF4E3	hsa-miR-327
EIF4E3	hsa-miR-147a
EIF4E3	hsa-miR-4520-2-3p
EIF4E3	hsa-miR-4251
EIF4E3	hsa-miR-6929-3p
EIF4E3	hsa-miR-2382-3p
EIF4E3	hsa-miR-33
EIF4E3	hsa-miR-425-3p
EIF4E3	hsa-miR-6818-3p
EIF4E3	hsa-miR-9-5p
EIF4E3	hsa-miR-7862
EIF4E3	hsa-miR-1255-5p
EIF4E3	hsa-miR-20-5p
EIF4E3	hsa-miR-2429
EIF4E3	hsa-miR-7229-3p
EIF4E3	hsa-miR-2294
EIF4E3	hsa-miR-7258-5p
EIF4E3	hsa-miR-6738-3p
EIF4E3	hsa-miR-6913-5p

EIF4E3	hsa-miR-7017-3p
EIF4E3	hsa-miR-561-5p
EIF4E3	hsa-miR-2291
EIF4E3	hsa-miR-6940-5p
EIF4E3	hsa-miR-3678-3p
EIF4E3	hsa-miR-2450c
EIF4E3	hsa-miR-6237
EIF4E3	hsa-miR-883-3p
EIF4E3	hsa-miR-3541
EIF4E3	hsa-miR-3596
EIF4E3	hsa-miR-4708-3p
EIF4E3	hsa-miR-7379-5p
EIF4E3	hsa-miR-3911
EIF4E3	hsa-miR-880-3p
EIF4E3	hsa-miR-5590-5p
EIF4E3	hsa-miR-3106-5p
EIF4E3	hsa-miR-4310
EIF4E3	hsa-miR-221-5p
EIF4E3	hsa-miR-2347
EIF4E3	hsa-miR-7659-3p
EIF4E3	hsa-miR-466g
EIF4E3	hsa-miR-8799
EIF4E3	hsa-miR-6875-3p
EIF4E3	hsa-miR-1639
EIF4E3	hsa-miR-3591
EIF4E3	hsa-miR-2398
EIF4E3	hsa-miR-3098-5p
EIF4E3	hsa-miR-675-3p
EIF4E3	hsa-miR-222-3p
EIF4E3	hsa-miR-7355-3p
EIF4E3	hsa-miR-6730-5p
EIF4E3	hsa-miR-7281-3p
EIF4E3	hsa-miR-5114
EIF4E3	hsa-miR-5582-3p
EIF4E3	hsa-miR-8793
EIF4E3	hsa-miR-8120
EIF4E3	hsa-miR-5579-5p
EIF4E3	hsa-miR-145-5p
EIF4E3	hsa-miR-9769-5p
EIF4E3	hsa-miR-465-3p
EIF4E3	hsa-miR-7705
EIF4E3	hsa-miR-2378
EIF4E3	hsa-miR-1249-3p
EIF4E3	hsa-miR-6719-3p
EIF4E3	hsa-miR-7380-3p
EIF4E3	hsa-miR-2317

EIF4E3	hsa-miR-6737-5p
EIF4E3	hsa-miR-8905
EIF4E3	hsa-miR-376c
EIF4E3	hsa-miR-369-3p
EIF4E3	hsa-miR-7155-5p
EIF4E3	hsa-miR-3166
EIF4E3	hsa-miR-761
EIF4E3	hsa-miR-183-5p
EIF4E3	hsa-miR-5187-3p
EIF4E3	hsa-miR-103a-2-5p
EIF4E3	hsa-miR-1191b-5p
EIF4E3	hsa-miR-888-5p
EIF4E3	hsa-miR-4302
EIF4E3	hsa-miR-3559-5p
EIF4E3	hsa-miR-7020-5p
EIF4E3	hsa-miR-1247-3p
EIF4E3	hsa-miR-8817
EIF4E3	hsa-miR-7371a-3p
EIF4E3	hsa-miR-589-3p
EIF4E3	hsa-miR-188-5p
EIF4E3	hsa-miR-4773
EIF4E3	hsa-miR-2117
EIF4E3	hsa-miR-6128
EIF4E3	hsa-miR-5100
EIF4E3	hsa-miR-6516-5p
EIF4E3	hsa-miR-548f-5p
EIF4E3	hsa-miR-7046-3p
EIF4E3	hsa-miR-7258-3p
EIF4E3	hsa-miR-7371-3p
EIF4E3	hsa-miR-1759-3p
EIF4E3	hsa-miR-2427
EIF4E3	hsa-miR-485
EIF4E3	hsa-miR-541-5p
EIF4E3	hsa-miR-3973
EIF4E3	hsa-miR-7231-5p
EIF4E3	hsa-miR-7292-5p
EIF4E3	hsa-miR-7371b-3p
EIF4E3	hsa-miR-1265
EIF4E3	hsa-miR-4748
EIF4E3	hsa-miR-5113
EIF4E3	hsa-miR-697
EIF4E3	hsa-miR-548ar-3p
EIF4E3	hsa-miR-1251-5p
EIF4E3	hsa-miR-302c-3p.2
EIF4E3	hsa-miR-449a
EIF4E3	hsa-miR-1295b-3p

EIF4E3	hsa-miR-6974-3p
EIF4E3	hsa-miR-8892
EIF4E3	hsa-miR-2352
EIF4E3	hsa-miR-5004-3p
EIF4E3	hsa-miR-6939-5p
EIF4E3	hsa-miR-3136-5p
EIF4E3	hsa-miR-522-3p
EIF4E3	hsa-miR-1933-3p
EIF4E3	hsa-miR-7371b-5p
EIF4E3	hsa-miR-3112-5p
EIF4E3	hsa-miR-7013-5p
EIF4E3	hsa-miR-7386-5p
EIF4E3	hsa-miR-562
EIF4E3	hsa-miR-380
EIF4E3	hsa-miR-3586-5p
EIF4E3	hsa-miR-6980-5p
EIF4E3	hsa-miR-4801
EIF4E3	hsa-miR-6418-3p
EIF4E3	hsa-miR-5104
EIF4E3	hsa-miR-7703
EIF4E3	hsa-miR-1343-3p
EIF4E3	hsa-miR-1747-3p
EIF4E3	hsa-miR-3600
EIF4E3	hsa-miR-373-5p
EIF4E3	hsa-miR-4252
EIF4E3	hsa-miR-202-3p
EIF4E3	hsa-miR-6887-5p
EIF4E3	hsa-miR-8820
EIF4E3	hsa-miR-6355
EIF4E3	hsa-miR-519
EIF4E3	hsa-miR-582-5p
EIF4E3	hsa-miR-6960-5p
EIF4E3	hsa-miR-3607-5p
EIF4E3	hsa-miR-548a-3p
EIF4E3	hsa-miR-7859
EIF4E3	hsa-miR-7344-3p
EIF4E3	hsa-miR-7398p-5p
EIF4E3	hsa-miR-2116-5p
EIF4E3	hsa-miR-4260
EIF4E3	hsa-miR-500a-5p
EIF4E3	hsa-miR-4722-3p
EIF4E3	hsa-miR-548c-5p
EIF4E3	hsa-miR-7476-3p
EIF4E3	hsa-miR-3064
EIF4E3	hsa-miR-7284-3p
EIF4E3	hsa-miR-8070

EIF4E3	hsa-miR-129b-3p
EIF4E3	hsa-miR-33-3p
EIF4E3	hsa-miR-4491
EIF4E3	hsa-miR-6868-3p
EIF4E3	hsa-miR-672-5p
EIF4E3	hsa-miR-4254
EIF4E3	hsa-miR-669d-5p
EIF4E3	hsa-miR-3535
EIF4E3	hsa-miR-125-5p
EIF4E3	hsa-miR-1226-3p
EIF4E3	hsa-miR-2287
EIF4E3	hsa-miR-6795-5p
EIF4E3	hsa-miR-3557-5p
EIF4E3	hsa-miR-2324
EIF4E3	hsa-miR-6973b-3p
EIF4E3	hsa-miR-2682-3p
EIF4E3	hsa-miR-605-3p
EIF4E3	hsa-miR-4699-5p
EIF4E3	hsa-miR-2330-5p
EIF4E3	hsa-miR-3664-5p
EIF4E3	hsa-miR-3971
EIF4E3	hsa-miR-4488
EIF4E3	hsa-miR-7259-5p
EIF4E3	hsa-miR-2467-3p
EIF4E3	hsa-miR-2448-3p
EIF4E3	hsa-miR-4505
EIF4E3	hsa-miR-2293
EIF4E3	hsa-miR-7039-3p
EIF4E3	hsa-miR-708-5p
EIF4E3	hsa-miR-2455
EIF4E3	hsa-miR-513a-3p
EIF4E3	hsa-miR-7286-5p
EIF4E3	hsa-miR-4447
EIF4E3	hsa-miR-3583-3p
EIF4E3	hsa-miR-202-3p
EIF4E3	hsa-miR-4680-5p
EIF4E3	hsa-miR-4716
EIF4E3	hsa-miR-4477b
EIF4E3	hsa-miR-219-2-3p
EIF4E3	hsa-miR-3926
EIF4E3	hsa-miR-8831
EIF4E3	hsa-miR-6405
EIF4E3	hsa-miR-371
EIF4E3	hsa-miR-2468
EIF4E3	hsa-miR-221-3p
EIF4E3	hsa-miR-8792

EIF4E3	hsa-miR-7045-3p
EIF4E3	hsa-miR-2285y
EIF4E3	hsa-miR-3680-3p
EIF4E3	hsa-miR-944
EIF4E3	hsa-miR-7073-5p
EIF4E3	hsa-miR-519e-5p
EIF4E3	hsa-miR-3083-5p
EIF4E3	hsa-miR-7687-5p
EIF4E3	hsa-miR-6908-5p
EIF4E3	hsa-miR-643
EIF4E3	hsa-miR-3143
EIF4E3	hsa-miR-3558-5p
EIF4E3	hsa-miR-7386g-5p
EIF4E3	hsa-miR-336-3p
EIF4E3	hsa-miR-2473
EIF4E3	hsa-miR-7680-5p
EIF4E3	hsa-miR-1323-3p
EIF4E3	hsa-miR-7086-5p
EIF4E3	hsa-miR-6810-5p
EIF4E3	hsa-miR-2285d
EIF4E3	hsa-miR-2365
EIF4E3	hsa-miR-891a-5p
EIF4E3	hsa-miR-6833-5p
EIF4E3	hsa-miR-219-5p
EIF4E3	hsa-miR-4711-3p
EIF4E3	hsa-miR-3543
EIF4E3	hsa-miR-4688
EIF4E3	hsa-miR-5101
EIF4E3	hsa-miR-548ao-3p
EIF4E3	hsa-miR-1208
EIF4E3	hsa-miR-3941
EIF4E3	hsa-miR-1542-5p
EIF4E3	hsa-miR-296-3p
EIF4E3	hsa-miR-6880-3p
EIF4E3	hsa-miR-615-3p
EIF4E3	hsa-miR-139-5p
EIF4E3	hsa-miR-22-3p
EIF4E3	hsa-miR-16-2-3p
EIF4E3	hsa-miR-5123
EIF4E3	hsa-miR-7437-3p
EIF4E3	hsa-miR-5088-3p
EIF4E3	hsa-miR-196b-3p
EIF4E3	hsa-miR-1252
EIF4E3	hsa-miR-7021-3p
EIF4E3	hsa-miR-6819-3p
EIF4E3	hsa-miR-193-3p

EIF4E3	hsa-miR-7035-5p
EIF4E3	hsa-miR-2318
EIF4E3	hsa-miR-519-5p
EIF4E3	hsa-miR-7370i-3p
EIF4E3	hsa-miR-8800
EIF4E3	hsa-miR-7194-3p
EIF4E3	hsa-miR-7092-3p
EIF4E3	hsa-miR-4793-5p
EIF4E3	hsa-miR-7110-5p
EIF4E3	hsa-miR-26a-3p
EIF4E3	hsa-miR-3593-5p
EIF4E3	hsa-miR-5192
EIF4E3	hsa-miR-2339
EIF4E3	hsa-miR-4423
EIF4E3	hsa-miR-4666b
EIF4E3	hsa-miR-6782-5p
EIF4E3	hsa-miR-6541
EIF4E3	hsa-miR-6796-3p
EIF4E3	hsa-miR-628-3p
EIF4E3	hsa-miR-6702-5p
EIF4E3	hsa-miR-6888-5p
EIF4E3	hsa-miR-148b-5p
EIF4E3	hsa-miR-3685
EIF4E3	hsa-miR-7386e-5p
EIF4E3	hsa-miR-6898-3p
EIF4E3	hsa-miR-1721
EIF4E3	hsa-miR-4803
EIF4E3	hsa-miR-3542
EIF4E3	hsa-miR-6672-3p
EIF4E3	hsa-miR-2128
EIF4E3	hsa-miR-452-3p
EIF4E3	hsa-miR-16-5p
EIF4E3	hsa-miR-766-5p
EIF4E3	hsa-miR-3585-3p
EIF4E3	hsa-miR-6951-3p
EIF4E3	hsa-miR-29b-5p
EIF4E3	hsa-miR-7033-3p
EIF4E3	hsa-miR-3135a
EIF4E3	hsa-miR-7242-3p
EIF4E3	hsa-miR-1296
EIF4E3	hsa-miR-8855
EIF4E3	hsa-miR-8054
EIF4E3	hsa-miR-7010-5p
EIF4E3	hsa-miR-425
EIF4E3	hsa-miR-2362
EIF4E3	hsa-miR-4424

EIF4E3	hsa-miR-93-3p
EIF4E3	hsa-miR-8899
EIF4E3	hsa-miR-3584-5p
EIF4E3	hsa-miR-6800-3p
EIF4E3	hsa-miR-6794-3p
EIF4E3	hsa-miR-151
EIF4E3	hsa-miR-294
EIF4E3	hsa-miR-8066
EIF4E3	hsa-miR-7668-5p
EIF4E3	hsa-miR-5581-5p
EIF4E3	hsa-miR-6507-3p
EIF4E3	hsa-miR-548e-5p
EIF4E3	hsa-miR-1681
EIF4E3	hsa-miR-3071-3p
EIF4E3	hsa-miR-548au-3p
EIF4E3	hsa-miR-559
EIF4E3	hsa-miR-490-3p
EIF4E3	hsa-miR-4742-5p
EIF4E3	hsa-miR-3097-5p
EIF4E3	hsa-miR-3180-5p
EIF4E3	hsa-miR-1684-3p
EIF4E3	hsa-miR-554
EIF4E3	hsa-miR-449a-3p
EIF4E3	hsa-miR-1580
EIF4E3	hsa-miR-499b-5p
EIF4E3	hsa-miR-133cd-5p
EIF4E3	hsa-miR-4418
EIF4E3	hsa-miR-550-3p
EIF4E3	hsa-miR-347
EIF4E3	hsa-miR-122-5p
EIF4E3	hsa-miR-940
EIF4E3	hsa-miR-7088-5p
EIF4E3	hsa-miR-498
EIF4E3	hsa-miR-4441
EIF4E3	hsa-miR-6516
EIF4E3	hsa-miR-2285m
EIF4E3	hsa-miR-6819-5p
EIF4E3	hsa-miR-133-3p
EIF4E3	hsa-miR-4653-3p
EIF4E3	hsa-miR-6933-5p
EIF4E3	hsa-miR-6534
EIF4E3	hsa-miR-766-3p
EIF4E3	hsa-miR-200ab-5p
EIF4E3	hsa-miR-1969
EIF4E3	hsa-miR-344g-3p
EIF4E3	hsa-miR-7234-5p

EIF4E3	hsa-miR-7243-3p
EIF4E3	hsa-miR-3691-5p
EIF4E3	hsa-miR-330-3p
EIF4E3	hsa-miR-330-3p.1
EIF4E3	hsa-miR-656
EIF4E3	hsa-miR-3057-5p
EIF4E3	hsa-miR-27-3p
EIF4E3	hsa-miR-30e
EIF4E3	hsa-miR-199-3p
EIF4E3	hsa-miR-2368-3p
EIF4E3	hsa-miR-3547-3p
EIF4E3	hsa-miR-7651-3p
EIF4E3	hsa-miR-936
EIF4E3	hsa-miR-4420
EIF4E3	hsa-miR-8825
EIF4E3	hsa-miR-511-5p
EIF4E3	hsa-miR-3560
EIF4E3	hsa-miR-6904-3p
EIF4E3	hsa-miR-892-5p
EIF4E3	hsa-miR-2284v
EIF4E3	hsa-miR-7154-5p
EIF4E3	hsa-miR-7660-5p
EIF4E3	hsa-miR-466f
EIF4E3	hsa-miR-1185-3p
EIF4E3	hsa-miR-7333-5p
EIF4E3	hsa-miR-6316
EIF4E3	hsa-miR-7307-3p
EIF4E3	hsa-miR-4650-3p
EIF4E3	hsa-miR-523-3p
EIF4E3	hsa-miR-6891-3p
EIF4E3	hsa-miR-548z
EIF4E3	hsa-miR-4668-5p
EIF4E3	hsa-miR-4476
EIF4E3	hsa-miR-3150a-3p
EIF4E3	hsa-miR-5710
EIF4E3	hsa-miR-290b-3p
EIF4E3	hsa-miR-7283-5p
EIF4E3	hsa-miR-6535
EIF4E3	hsa-miR-140-3p
EIF4E3	hsa-miR-3067-5p
EIF4E3	hsa-miR-3667-3p
EIF4E3	hsa-miR-5580-5p
EIF4E3	hsa-miR-3190-3p
EIF4E3	hsa-miR-5002-5p
EIF4E3	hsa-miR-376b-3p
EIF4E3	hsa-miR-505-3p.2

EIF4E3	hsa-miR-3104-5p
EIF4E3	hsa-miR-134-3p
EIF4E3	hsa-miR-1909-3p
EIF4E3	hsa-miR-3652
EIF4E3	hsa-miR-324-5p
EIF4E3	hsa-miR-525-5p
EIF4E3	hsa-miR-7975
EIF4E3	hsa-miR-7370-3p
EIF4E3	hsa-miR-542
EIF4E3	hsa-miR-6981-5p
EIF4E3	hsa-miR-743a-3p
EIF4E3	hsa-miR-650
EIF4E3	hsa-miR-548aj-3p
EIF4E3	hsa-miR-6966-3p
EIF4E3	hsa-miR-4789-3p
EIF4E3	hsa-miR-7004-3p
EIF4E3	hsa-miR-8900
EIF4E3	hsa-miR-570-5p
EIF4E3	hsa-miR-548ak
EIF4E3	hsa-miR-431-5p
EIF4E3	hsa-miR-2461-5p
EIF4E3	hsa-miR-6728-5p
EIF4E3	hsa-miR-4691-5p
EIF4E3	hsa-miR-214-3p
EIF4E3	hsa-miR-885-3p
EIF4E3	hsa-miR-706
EIF4E3	hsa-miR-7329-3p
EIF4E3	hsa-miR-505-3p
EIF4E3	hsa-miR-8821
EIF4E3	hsa-miR-103b
EIF4E3	hsa-miR-6934-5p
EIF4E3	hsa-miR-4726-3p
EIF4E3	hsa-miR-1245b-3p
EIF4E3	hsa-miR-878-3p
EIF4E3	hsa-miR-1843a-5p
EIF4E3	hsa-miR-6992-3p
EIF4E3	hsa-miR-1434-5p
EIF4E3	hsa-miR-6528
EIF4E3	hsa-miR-3183
EIF4E3	hsa-miR-519e-3p
EIF4E3	hsa-miR-367-3p
EIF4E3	hsa-miR-138-3p
EIF4E3	hsa-miR-2393
EIF4E3	hsa-miR-9768-3p
EIF4E3	hsa-miR-7652-5p
EIF4E3	hsa-miR-322-5p

EIF4E3	hsa-miR-7398q-3p
EIF4E3	hsa-miR-30-5p
EIF4E3	hsa-miR-6987-5p
EIF4E3	hsa-miR-7398l-3p
EIF4E3	hsa-miR-193b-3p
EIF4E3	hsa-miR-612
EIF4E3	hsa-miR-3179
EIF4E3	hsa-miR-3548
EIF4E3	hsa-miR-548ap-5p
EIF4E3	hsa-miR-3147
EIF4E3	hsa-miR-1226-3p
EIF4E3	hsa-miR-6728-3p
EIF4E3	hsa-miR-6749-3p
EIF4E3	hsa-miR-299-3p
EIF4E3	hsa-miR-30-3p
EIF4E3	hsa-miR-291-5p
EIF4E3	hsa-miR-4459
EIF4E3	hsa-miR-7351-3p
EIF4E3	hsa-miR-577-3p
EIF4E3	hsa-miR-130a-3p
EIF4E3	hsa-miR-2904
EIF4E3	hsa-miR-4536-5p
EIF4E3	hsa-miR-2894
EIF4E3	hsa-miR-548b-3p
EIF4E3	hsa-miR-1555-5p
EIF4E3	hsa-miR-1684-5p
EIF4E3	hsa-miR-188-3p
EIF4E3	hsa-miR-294-3p
EIF4E3	hsa-miR-7304-3p
EIF4E3	hsa-miR-7340-5p
EIF4E3	hsa-miR-203b-3p
EIF4E3	hsa-miR-3552
EIF4E3	hsa-miR-219a-1-3p
EIF4E3	hsa-miR-2442
EIF4E3	hsa-miR-6715a-3p
EIF4E3	hsa-miR-4436b-5p
EIF4E3	hsa-miR-7398o-5p
EIF4E3	hsa-miR-219-1-3p
EIF4E3	hsa-miR-6824-3p
EIF4E3	hsa-miR-2337
EIF4E3	hsa-miR-26-3p
EIF4E3	hsa-miR-6513-5p
EIF4E3	hsa-miR-8101
EIF4E3	hsa-miR-657
EIF4E3	hsa-miR-26b-3p
EIF4E3	hsa-miR-7449-3p

EIF4E3	hsa-miR-8108
EIF4E3	hsa-miR-1540
EIF4E3	hsa-miR-8063
EIF4E3	hsa-miR-1231-3p
EIF4E3	hsa-miR-2411-5p
EIF4E3	hsa-miR-7457-5p
EIF4E3	hsa-miR-2284d
EIF4E3	hsa-miR-1290
EIF4E3	hsa-miR-1540-3p
EIF4E3	hsa-miR-6758-3p
EIF4E3	hsa-miR-2284j
EIF4E3	hsa-miR-4419a
EIF4E3	hsa-miR-7224-3p
EIF4E3	hsa-miR-448
EIF4E3	hsa-miR-1966-3p
EIF4E3	hsa-miR-4802-3p
EIF4E3	hsa-miR-6418-5p
EIF4E3	hsa-miR-384
EIF4E3	hsa-miR-6807-5p
EIF4E3	hsa-miR-3572-5p
EIF4E3	hsa-miR-2375
EIF4E3	hsa-miR-485-3p
EIF4E3	hsa-miR-6986-3p
EIF4E3	hsa-miR-2437
EIF4E3	hsa-miR-7092-5p
EIF4E3	hsa-miR-6983-3p
EIF4E3	hsa-miR-937-5p
EIF4E3	hsa-miR-218-1-3p
EIF4E3	hsa-miR-7262a-5p
EIF4E3	hsa-miR-6961-3p
EIF4E3	hsa-miR-292a-5p
EIF4E3	hsa-miR-1288-5p
EIF4E3	hsa-miR-92a-2-5p
EIF4E3	hsa-miR-10-5p
EIF4E3	hsa-miR-454-5p
EIF4E3	hsa-let-7f-1-3p
EIF4E3	hsa-miR-6416-5p
EIF4E3	hsa-miR-216a
EIF4E3	hsa-miR-33a-3p
EIF4E3	hsa-miR-4478
EIF4E3	hsa-miR-31-5p
EIF4E3	hsa-miR-6924-3p
EIF4E3	hsa-miR-5787
EIF4E3	hsa-miR-124-3p.2
EIF4E3	hsa-miR-3571
EIF4E3	hsa-miR-200bc

EIF4E3	hsa-miR-1930-3p
EIF4E3	hsa-miR-7686-5p
EIF4E3	hsa-miR-5000-5p
EIF4E3	hsa-miR-7169-5p
EIF4E3	hsa-miR-2306
EIF4E3	hsa-miR-6931-5p
EIF4E3	hsa-miR-7090-3p
EIF4E3	hsa-miR-4536-5p
EIF4E3	hsa-miR-2891
EIF4E3	hsa-miR-7180-5p
EIF4E3	hsa-miR-6918-5p
EIF4E3	hsa-miR-6988-3p
EIF4E3	hsa-miR-615-5p
EIF4E3	hsa-miR-4504
EIF4E3	hsa-miR-320-3p
EIF4E3	hsa-miR-335-5p
EIF4E3	hsa-miR-149-3p
EIF4E3	hsa-miR-6815-3p
EIF4E3	hsa-miR-448-3p
EIF4E3	hsa-miR-7170-5p
EIF4E3	hsa-miR-520-5p
EIF4E3	hsa-miR-295-3p
EIF4E3	hsa-miR-1306-5p
EIF4E3	hsa-miR-1661
EIF4E3	hsa-miR-4324
EIF4E3	hsa-miR-7315-3p
EIF4E3	hsa-miR-7251-5p
EIF4E3	hsa-miR-5588-5p
EIF4E3	hsa-miR-7977
EIF4E3	hsa-miR-208a-3p
EIF4E3	hsa-miR-7238-3p
EIF4E3	hsa-miR-466b-3p
EIF4E3	hsa-miR-512-3p
EIF4E3	hsa-miR-7022-3p
EIF4E3	hsa-miR-142b
EIF4E3	hsa-miR-6344
EIF4E3	hsa-miR-2480
EIF4E3	hsa-miR-409-5p
EIF4E3	hsa-miR-2397-3p
EIF4E3	hsa-miR-483-3p.2
EIF4E3	hsa-miR-2284a
EIF4E3	hsa-miR-2325b
EIF4E3	hsa-miR-3667-5p
EIF4E3	hsa-miR-883b-5p
EIF4E3	hsa-miR-669aflp-5p
EIF4E3	hsa-miR-3059-5p

EIF4E3	hsa-miR-2333
EIF4E3	hsa-miR-223-5p
EIF4E3	hsa-miR-28-5p
EIF4E3	hsa-miR-8848
EIF4E3	hsa-miR-421
EIF4E3	hsa-miR-6905-5p
EIF4E3	hsa-miR-7294-5p
EIF4E3	hsa-miR-3529-3p
EIF4E3	hsa-miR-514a-3p
EIF4E3	hsa-miR-6920-3p
EIF4E3	hsa-miR-7669-3p
EIF4E3	hsa-miR-6784-3p
EIF4E3	hsa-miR-3064-5p
EIF4E3	hsa-miR-23b-5p
EIF4E3	hsa-miR-641
EIF4E3	hsa-miR-465-5p
EIF4E3	hsa-miR-7205-3p
EIF4E3	hsa-miR-743b-5p
EIF4E3	hsa-miR-27-5p
EIF4E3	hsa-miR-3103-3p
EIF4E3	hsa-miR-668-3p
EIF4E3	hsa-miR-7648-3p
EIF4E3	hsa-miR-742-5p
EIF4E3	hsa-miR-561-3p
EIF4E3	hsa-miR-193-5p
EIF4E3	hsa-miR-200a-3p
EIF4E3	hsa-miR-3622b-5p
EIF4E3	hsa-miR-556-5p
EIF4E3	hsa-miR-7162-3p
EIF4E3	hsa-miR-579
EIF4E3	hsa-miR-3073a-5p
EIF4E3	hsa-miR-887-3p
EIF4E3	hsa-miR-7038-3p
EIF4E3	hsa-miR-429-5p
EIF4E3	hsa-miR-764
EIF4E3	hsa-miR-6508-5p
EIF4E3	hsa-miR-5588-3p
EIF4E3	hsa-miR-6742-3p
EIF4E3	hsa-miR-206-3p
EIF4E3	hsa-miR-7341-3p
EIF4E3	hsa-miR-6769b-3p
EIF4E3	hsa-miR-551-5p
EIF4E3	hsa-miR-6790-3p
EIF4E3	hsa-miR-379-5p
EIF4E3	hsa-miR-206-5p
EIF4E3	hsa-miR-93-5p

EIF4E3	hsa-miR-302a-3p
EIF4E3	hsa-miR-8795
EIF4E3	hsa-miR-3067-3p
EIF4E3	hsa-miR-548as-5p
EIF4E3	hsa-miR-7056-3p
EIF4E3	hsa-miR-3120-5p
EIF4E3	hsa-miR-409-3p
EIF4E3	hsa-miR-6123
EIF4E3	hsa-miR-2379
EIF4E3	hsa-miR-4320
EIF4E3	hsa-miR-574-5p
EIF4E3	hsa-miR-6832-3p
EIF4E3	hsa-miR-7386d-3p
EIF4E3	hsa-miR-590-3p
EIF4E3	hsa-miR-6956-5p
EIF4E3	hsa-miR-34-5p
EIF4E3	hsa-miR-8839
EIF4E3	hsa-miR-580-3p
EIF4E3	hsa-miR-1343
EIF4E3	hsa-miR-938
EIF4E3	hsa-miR-1465
EIF4E3	hsa-miR-3173-5p
EIF4E3	hsa-miR-6932-5p
EIF4E3	hsa-miR-7359-5p
EIF4E3	hsa-miR-376a-1-5p
EIF4E3	hsa-miR-4759
EIF4E3	hsa-miR-18-3p
EIF4E3	hsa-miR-3606-3p
EIF4E3	hsa-miR-6797-3p
EIF4E3	hsa-miR-1267
EIF4E3	hsa-miR-28-5p
EIF4E3	hsa-miR-8077
EIF4E3	hsa-miR-639
EIF4E3	hsa-miR-6727-3p
EIF4E3	hsa-miR-17-3p
EIF4E3	hsa-miR-212-5p
EIF4E3	hsa-miR-2477
EIF4E3	hsa-miR-676-3p
EIF4E3	hsa-miR-4523
EIF4E3	hsa-miR-1243
EIF4E3	hsa-miR-4790-3p
EIF4E3	hsa-miR-2284w
EIF4E3	hsa-miR-455-3p.1
EIF4E3	hsa-miR-203a-5p
EIF4E3	hsa-miR-6780b-3p
EIF4E3	hsa-miR-8106

EIF4E3	hsa-miR-7069-3p
EIF4E3	hsa-miR-338-3p
EIF4E3	hsa-miR-891
EIF4E3	hsa-miR-18b-3p
EIF4E3	hsa-miR-29a-1-5p
EIF4E3	hsa-miR-3650
EIF4E3	hsa-miR-7683-3p
EIF4E3	hsa-miR-6903-5p
EIF4E3	hsa-miR-2345
EIF4E3	hsa-miR-6378
EIF4E3	hsa-miR-7396-5p
EIF4E3	hsa-miR-194-3p
EIF4E3	hsa-miR-424-5p
EIF4E3	hsa-miR-7193-3p
EIF4E3	hsa-miR-2389
EIF4E3	hsa-miR-7004-5p
EIF4E3	hsa-miR-3916
EIF4E3	hsa-miR-7199-5p
EIF4E3	hsa-miR-744-3p
EIF4E3	hsa-miR-1952
EIF4E3	hsa-miR-8822
EIF4E3	hsa-miR-130a-5p
EIF4E3	hsa-miR-7847-3p
EIF4E3	hsa-miR-106a-3p
EIF4E3	hsa-miR-3914
EIF4E3	hsa-miR-432-5p
EIF4E3	hsa-miR-4494
EIF4E3	hsa-miR-874
EIF4E3	hsa-miR-2462
EIF4E3	hsa-miR-4703-5p
EIF4E3	hsa-miR-507b
EIF4E3	hsa-miR-4764-5p
EIF4E3	hsa-miR-7045-5p
EIF4E3	hsa-miR-7029-3p
EIF4E3	hsa-miR-3128
EIF4E3	hsa-miR-7089-5p
EIF4E3	hsa-miR-7041-5p
EIF4E3	hsa-miR-23c
EIF4E3	hsa-miR-216a-5p
EIF4E3	hsa-miR-7036b-3p
EIF4E3	hsa-miR-7230-3p
EIF4E3	hsa-miR-889-3p
EIF4E3	hsa-miR-3152-3p
EIF4E3	hsa-miR-1236-5p
EIF4E3	hsa-miR-6760-5p
EIF4E3	hsa-miR-34bc-3p

EIF4E3	hsa-miR-320b
EIF4E3	hsa-miR-1434-3p
EIF4E3	hsa-miR-742-3p
EIF4E3	hsa-miR-1584-3p
EIF4E3	hsa-miR-550a-3-5p
EIF4E3	hsa-miR-8879
EIF4E3	hsa-miR-8854
EIF4E3	hsa-miR-548o-5p
EIF4E3	hsa-miR-7091-3p
EIF4E3	hsa-miR-5626-3p
EIF4E3	hsa-miR-8861
EIF4E3	hsa-miR-2423
EIF4E3	hsa-miR-23-3p
EIF4E3	hsa-miR-2308
EIF4E3	hsa-miR-3101-3p
EIF4E3	hsa-miR-337-3p
EIF4E3	hsa-miR-3622b-3p
EIF4E3	hsa-miR-3129-5p
EIF4E3	hsa-miR-7257-3p
EIF4E3	hsa-miR-678
EIF4E3	hsa-miR-4727-3p
EIF4E3	hsa-miR-107-5p
EIF4E3	hsa-miR-1251-3p
EIF4E3	hsa-miR-2464-5p
EIF4E3	hsa-miR-5702
EIF4E3	hsa-miR-1238-5p
EIF4E3	hsa-miR-466b-5p
EIF4E3	hsa-miR-6867-5p
EIF4E3	hsa-miR-193a
EIF4E3	hsa-miR-92a-1-5p
EIF4E3	hsa-miR-2895
EIF4E3	hsa-miR-134
EIF4E3	hsa-miR-711
EIF4E3	hsa-miR-2322-5p
EIF4E3	hsa-miR-3475-5p
EIF4E3	hsa-miR-1263
EIF4E3	hsa-miR-3075-3p
EIF4E3	hsa-miR-376-3p
EIF4E3	hsa-miR-142
EIF4E3	hsa-miR-34c-5p
EIF4E3	hsa-miR-2319
EIF4E3	hsa-miR-7074-5p
EIF4E3	hsa-miR-6537-3p
EIF4E3	hsa-miR-1298-5p
EIF4E3	hsa-miR-7388b-5p
EIF4E3	hsa-miR-4327

EIF4E3	hsa-miR-1948-3p
EIF4E3	hsa-miR-6761-5p
EIF4E3	hsa-miR-383-5p.1
EIF4E3	hsa-miR-8833
EIF4E3	hsa-miR-5579-3p
EIF4E3	hsa-miR-7042-3p
EIF4E3	hsa-miR-524-5p
EIF4E3	hsa-miR-2285s
EIF4E3	hsa-miR-7325-3p
EIF4E3	hsa-miR-320-5p
EIF4E3	hsa-miR-138-1-3p
EIF4E3	hsa-miR-1941-5p
EIF4E3	hsa-miR-194-5p
EIF4E3	hsa-miR-6971-5p
EIF4E3	hsa-miR-373-3p
EIF4E3	hsa-miR-4760-5p
EIF4E3	hsa-miR-542-3p
EIF4E3	hsa-miR-3175
EIF4E3	hsa-miR-339-3p
EIF4E3	hsa-miR-7082-3p
EIF4E3	hsa-miR-518c-5p
EIF4E3	hsa-miR-4645-5p
EIF4E3	hsa-miR-548am-5p
EIF4E3	hsa-miR-2276-5p
EIF4E3	hsa-miR-548ag
EIF4E3	hsa-miR-5623-3p
EIF4E3	hsa-miR-155-5p
EIF4E3	hsa-miR-1184
EIF4E3	hsa-miR-142-3p.2
EIF4E3	hsa-miR-6923-3p
EIF4E3	hsa-miR-540-5p
EIF4E3	hsa-miR-7312-5p
EIF4E3	hsa-miR-363-3p
EIF4E3	hsa-miR-4282
EIF4E3	hsa-miR-187-3p
EIF4E3	hsa-miR-548n
EIF4E3	hsa-miR-7678-3p
EIF4E3	hsa-miR-411-5p
EIF4E3	hsa-miR-2053
EIF4E3	hsa-miR-6550-5p
EIF4E3	hsa-miR-3184-3p
EIF4E3	hsa-miR-7113-3p
EIF4E3	hsa-miR-3475-3p
EIF4E3	hsa-miR-6627-3p
EIF4E3	hsa-miR-6676-3p
EIF4E3	hsa-miR-6765-3p

EIF4E3	hsa-miR-672-3p
EIF4E3	hsa-miR-3945
EIF4E3	hsa-miR-625-3p
EIF4E3	hsa-miR-3182
EIF4E3	hsa-miR-665
EIF4E3	hsa-miR-3076-3p
EIF4E3	hsa-miR-656-3p
EIF4E3	hsa-miR-7296-3p
EIF4E3	hsa-miR-636
EIF4E3	hsa-miR-4468
EIF4E3	hsa-miR-4281
EIF4E3	hsa-miR-8093
EIF4E3	hsa-miR-935
EIF4E3	hsa-miR-3123
EIF4E3	hsa-miR-2357
EIF4E3	hsa-miR-8813
EIF4E3	hsa-miR-1894-3p
EIF4E3	hsa-miR-223-3p
EIF4E3	hsa-miR-4427
EIF4E3	hsa-miR-6958-5p
EIF4E3	hsa-miR-466n-5p
EIF4E3	hsa-miR-605-5p
EIF4E3	hsa-miR-6975-5p
EIF4E3	hsa-miR-26
EIF4E3	hsa-miR-466l-3p
EIF4E3	hsa-miR-7394b-5p
EIF4E3	hsa-miR-6883-5p
EIF4E3	hsa-miR-497-3p
EIF4E3	hsa-miR-7185-5p
EIF4E3	hsa-miR-3929
EIF4E3	hsa-miR-4667-3p
EIF4E3	hsa-miR-6806-5p
EIF4E3	hsa-miR-8068
EIF4E3	hsa-miR-5698
EIF4E3	hsa-miR-4769-5p
EIF4E3	hsa-miR-3085-3p
EIF4E3	hsa-miR-450-5p
EIF4E3	hsa-miR-3119
EIF4E3	hsa-miR-2331-5p
EIF4E3	hsa-miR-6982-3p
EIF4E3	hsa-miR-7398e-3p
EIF4E3	hsa-miR-370-3p
EIF4E3	hsa-miR-7173-3p
EIF4E3	hsa-miR-548e-3p
EIF4E3	hsa-miR-34b-3p
EIF4E3	hsa-miR-1343-5p

EIF4E3	hsa-miR-6818-5p
EIF4E3	hsa-miR-514a-3p
EIF4E3	hsa-miR-10b-3p
EIF4E3	hsa-miR-132-3p
EIF4E3	hsa-miR-8061
EIF4E3	hsa-miR-942-3p
EIF4E3	hsa-miR-4496
EIF4E3	hsa-miR-642-3p
EIF4E3	hsa-miR-3134
EIF4E3	hsa-miR-6908-3p
EIF4E3	hsa-miR-7054-3p
EIF4E3	hsa-miR-2355-3p
EIF4E3	hsa-miR-7008-3p
EIF4E3	hsa-miR-3682-3p
EIF4E3	hsa-miR-7186-3p
EIF4E3	hsa-miR-3132
EIF4E3	hsa-miR-6693-3p
EIF4E3	hsa-miR-323a-3p
EIF4E3	hsa-miR-3964
EIF4E3	hsa-miR-1298
EIF4E3	hsa-miR-3130-5p
EIF4E3	hsa-miR-5699-5p
EIF4E3	hsa-miR-7080-5p
EIF4E3	hsa-miR-1547-5p
EIF4E3	hsa-miR-106
EIF4E3	hsa-miR-3689f
EIF4E3	hsa-miR-668
EIF4E3	hsa-miR-5680
EIF4E3	hsa-miR-6756-5p
EIF4E3	hsa-miR-3065-3p
EIF4E3	hsa-miR-1287
EIF4E3	hsa-miR-7225-3p
EIF4E3	hsa-miR-509-3-5p
EIF4E3	hsa-miR-7685-3p
EIF4E3	hsa-miR-4712-3p
EIF4E3	hsa-miR-7026-3p
EIF4E3	hsa-miR-7319-5p
EIF4E3	hsa-miR-17-5p
EIF4E3	hsa-miR-7331-1-3p
EIF4E3	hsa-miR-3156-5p
EIF4E3	hsa-miR-6835-5p
EIF4E3	hsa-miR-6780a-5p
EIF4E3	hsa-miR-6906-5p
EIF4E3	hsa-miR-664a-5p
EIF4E3	hsa-miR-423-3p
EIF4E3	hsa-miR-215-3p

EIF4E3	hsa-miR-8877
EIF4E3	hsa-miR-7395-3p
EIF4E3	hsa-miR-126-5p
EIF4E3	hsa-miR-7016-3p
EIF4E3	hsa-miR-3617-5p
EIF4E3	hsa-miR-7649-3p
EIF4E3	hsa-miR-3547-5p
EIF4E3	hsa-miR-516
EIF4E3	hsa-miR-183-5p.1
EIF4E3	hsa-miR-6989-3p
EIF4E3	hsa-miR-7657-5p
EIF4E3	hsa-miR-7297-3p
EIF4E3	hsa-miR-7215-5p
EIF4E3	hsa-miR-10b-5p
EIF4E3	hsa-miR-2296
EIF4E3	hsa-miR-22-3p
EIF4E3	hsa-miR-3662
EIF4E3	hsa-miR-891-3p
EIF4E3	hsa-miR-548k
EIF4E3	hsa-miR-2367-3p
EIF4E3	hsa-miR-452-5p
EIF4E3	hsa-miR-1273e
EIF4E3	hsa-miR-584-5p
EIF4E3	hsa-miR-576-5p
EIF4E3	hsa-miR-7662-5p
EIF4E3	hsa-miR-363-5p
EIF4E3	hsa-miR-3653-5p
EIF4E3	hsa-miR-6920-5p
EIF4E3	hsa-miR-1233-3p
EIF4E3	hsa-miR-6333
EIF4E3	hsa-miR-7227-3p
EIF4E3	hsa-miR-4762-5p
EIF4E3	hsa-miR-4531
EIF4E3	hsa-miR-8880
EIF4E3	hsa-miR-7371f-5p
EIF4E3	hsa-miR-548ay-3p
EIF4E3	hsa-miR-7675-3p
EIF4E3	hsa-miR-185-5p
EIF4E3	hsa-miR-6793-3p
EIF4E3	hsa-miR-201-3p
EIF4E3	hsa-miR-2486-5p
EIF4E3	hsa-miR-7079-3p
EIF4E3	hsa-miR-18a-3p
EIF4E3	hsa-miR-146a-3p
EIF4E3	hsa-miR-1545b-5p
EIF4E3	hsa-miR-4519

EIF4E3	hsa-miR-450a-1-3p
EIF4E3	hsa-miR-7226-3p
EIF4E3	hsa-miR-6760-3p
EIF4E3	hsa-miR-6782-3p
EIF4E3	hsa-miR-574-3p
EIF4E3	hsa-miR-6877-3p
EIF4E3	hsa-miR-548aj-5p
EIF4E3	hsa-miR-2285u
EIF4E3	hsa-miR-8062
EIF4E3	hsa-miR-877-5p
EIF4E3	hsa-miR-6780b-5p
EIF4E3	hsa-miR-340-5p
EIF4E3	hsa-miR-4535
EIF4E3	hsa-miR-6972-3p
EIF4E3	hsa-miR-7398-3p
EIF4E3	hsa-miR-7256-3p
EIF4E3	hsa-miR-487a-5p
EIF4E3	hsa-miR-3145-3p
EIF4E3	hsa-miR-7079-5p
EIF4E3	hsa-miR-3113-5p
EIF4E3	hsa-miR-376bc-5p
EIF4E3	hsa-miR-4719
EIF4E3	hsa-miR-7197-3p
EIF4E3	hsa-miR-2356
EIF4E3	hsa-miR-3549
EIF4E3	hsa-miR-6133
EIF4E3	hsa-miR-6529a
EIF4E3	hsa-miR-1799
EIF4E3	hsa-miR-7285-5p
EIF4E3	hsa-miR-494-5p
EIF4E3	hsa-miR-7a-2-3p
EIF4E3	hsa-miR-2349
EIF4E3	hsa-miR-6540-3p
EIF4E3	hsa-miR-4723-5p
EIF4E3	hsa-miR-7688-5p
EIF4E3	hsa-miR-3942-5p
EIF4E3	hsa-miR-1270
EIF4E3	hsa-miR-3106-3p
EIF4E3	hsa-miR-1912
EIF4E3	hsa-miR-302c-5p
EIF4E3	hsa-miR-7076-5p
EIF4E3	hsa-miR-710
EIF4E3	hsa-miR-4673
EIF4E3	hsa-miR-1183
EIF4E3	hsa-miR-2402
EIF4E3	hsa-miR-3470a

EIF4E3	hsa-miR-2458
EIF4E3	hsa-miR-6857-5p
EIF4E3	hsa-miR-4780
EIF4E3	hsa-miR-138-5p
EIF4E3	hsa-miR-7320-5p
EIF4E3	hsa-miR-7001-3p
EIF4E3	hsa-miR-597-5p
EIF4E3	hsa-miR-6373
EIF4E3	hsa-miR-4667
EIF4E3	hsa-miR-7192-5p
EIF4E3	hsa-miR-548p
EIF4E3	hsa-miR-3162-5p
EIF4E3	hsa-miR-3692-3p
EIF4E3	hsa-miR-486-3p
EIF4E3	hsa-miR-7398j-3p
EIF4E3	hsa-miR-6366
EIF4E3	hsa-miR-190-3p
EIF4E3	hsa-miR-105-5p
EIF4E3	hsa-miR-128-3p
EIF4E3	hsa-miR-4510
EIF4E3	hsa-miR-8857
EIF4E3	hsa-miR-645
EIF4E3	hsa-miR-3129-3p
EIF4E3	hsa-miR-7704
EIF4E3	hsa-miR-466ade-3p
EIF4E3	hsa-miR-6957-3p
EIF4E3	hsa-miR-6735-3p
EIF4E3	hsa-miR-3692-5p
EIF4E3	hsa-miR-203
EIF4E3	hsa-miR-6944-3p
EIF4E3	hsa-miR-7314-3p
EIF4E3	hsa-miR-298
EIF4G3	hsa-miR-3524-3p
EIF4G3	hsa-miR-6878-3p
EIF4G3	hsa-miR-2394
EIF4G3	hsa-miR-7157-5p
EIF4G3	hsa-miR-6932-3p
EIF4G3	hsa-miR-129-5p
EIF4G3	hsa-miR-4728-5p
EIF4G3	hsa-miR-2284
EIF4G3	hsa-miR-4676-5p
EIF4G3	hsa-miR-7245-5p
EIF4G3	hsa-miR-6558-5p
EIF4G3	hsa-miR-369-3p
EIF4G3	hsa-miR-186-3p
EIF4G3	hsa-miR-646

EIF4G3	hsa-miR-2325
EIF4G3	hsa-miR-6817-3p
EIF4G3	hsa-miR-193b-5p
EIF4G3	hsa-miR-4799-3p
EIF4G3	hsa-miR-6995-5p
EIF4G3	hsa-miR-567
EIF4G3	hsa-miR-4638-3p
EIF4G3	hsa-miR-1266
EIF4G3	hsa-miR-148a-5p
EIF4G3	hsa-miR-5101
EIF4G3	hsa-miR-6809-3p
EIF4G3	hsa-miR-1306-3p
EIF4G3	hsa-miR-1680-5p
EIF4G3	hsa-miR-1689-5p
EIF4G3	hsa-miR-7183-5p
EIF4G3	hsa-miR-7317-3p
EIF4G3	hsa-miR-3571
EIF4G3	hsa-miR-101c
EIF4G3	hsa-miR-6976-5p
EIF4G3	hsa-miR-7386l-3p
EIF4G3	hsa-miR-7191-3p
EIF4G3	hsa-miR-6516-3p
EIF4G3	hsa-miR-8847
EIF4G3	hsa-miR-6573-5p
EIF4G3	hsa-miR-1237-3p
EIF4G3	hsa-miR-4692
EIF4G3	hsa-miR-760-5p
EIF4G3	hsa-miR-6551-5p
EIF4G3	hsa-miR-3606-3p
EIF4G3	hsa-miR-1185-5p
EIF4G3	hsa-miR-5703
EIF4G3	hsa-miR-1773-3p
EIF4G3	hsa-miR-7053-3p
EIF4G3	hsa-miR-6863
EIF4G3	hsa-miR-6399
EIF4G3	hsa-miR-6882-5p
EIF4G3	hsa-miR-107
EIF4G3	hsa-miR-3613-3p
EIF4G3	hsa-miR-495-3p
EIF4G3	hsa-miR-1463
EIF4G3	hsa-miR-4724-3p
EIF4G3	hsa-miR-7398m-3p
EIF4G3	hsa-miR-4677-5p
EIF4G3	hsa-miR-7673-3p
EIF4G3	hsa-miR-4536-3p
EIF4G3	hsa-miR-3124-3p

EIF4G3	hsa-miR-141-3p
EIF4G3	hsa-miR-1598
EIF4G3	hsa-miR-548at-5p
EIF4G3	hsa-miR-103-3p
EIF4G3	hsa-miR-8485
EIF4G3	hsa-miR-6840-3p
EIF4G3	hsa-miR-4635
EIF4G3	hsa-miR-548ah-5p
EIF4G3	hsa-miR-33-3p
EIF4G3	hsa-miR-7207-5p
EIF4G3	hsa-miR-7108-3p
EIF4G3	hsa-miR-669c-3p
EIF4G3	hsa-miR-7330-3p
EIF4G3	hsa-miR-544-3p
EIF4G3	hsa-miR-2363
EIF4G3	hsa-miR-3179
EIF4G3	hsa-miR-4434
EIF4G3	hsa-miR-7314-5p
EIF4G3	hsa-miR-883-5p
EIF4G3	hsa-miR-6619-5p
EIF4G3	hsa-miR-5696
EIF4G3	hsa-miR-2346
EIF4G3	hsa-miR-6830-3p
EIF4G3	hsa-miR-8850
EIF4G3	hsa-miR-4748
EIF4G3	hsa-miR-7094-1-5p
EIF4G3	hsa-miR-2284g
EIF4G3	hsa-miR-590-5p
EIF4G3	hsa-miR-5003-3p
EIF4G3	hsa-miR-7352-3p
EIF4G3	hsa-miR-5709-3p
EIF4G3	hsa-miR-883ab-3p
EIF4G3	hsa-miR-4793-3p
EIF4G3	hsa-miR-7243-5p
EIF4G3	hsa-miR-3618-5p
EIF4G3	hsa-miR-3573-5p
EIF4G3	hsa-miR-2284k
EIF4G3	hsa-miR-6376
EIF4G3	hsa-miR-381-3p
EIF4G3	hsa-miR-2284b
EIF4G3	hsa-miR-466g
EIF4G3	hsa-miR-302c-3p.2
EIF4G3	hsa-miR-376e
EIF4G3	hsa-miR-7663-5p
EIF4G3	hsa-miR-29b-2-5p
EIF4G3	hsa-miR-6755-5p

EIF4G3	hsa-miR-7398q-3p
EIF4G3	hsa-miR-5691
EIF4G3	hsa-miR-5615-5p
EIF4G3	hsa-miR-3058-3p
EIF4G3	hsa-miR-145-3p
EIF4G3	hsa-miR-6701-3p
EIF4G3	hsa-miR-133b-5p
EIF4G3	hsa-miR-2284l
EIF4G3	hsa-miR-7250-3p
EIF4G3	hsa-miR-6625-5p
EIF4G3	hsa-miR-9-3p
EIF4G3	hsa-miR-4473
EIF4G3	hsa-miR-7681-5p
EIF4G3	hsa-miR-7192-3p
EIF4G3	hsa-miR-520
EIF4G3	hsa-miR-133c
EIF4G3	hsa-miR-297-5p
EIF4G3	hsa-miR-7264-3p
EIF4G3	hsa-miR-883-3p
EIF4G3	hsa-miR-6357
EIF4G3	hsa-miR-4768-3p
EIF4G3	hsa-miR-7461-5p
EIF4G3	hsa-miR-3121-3p
EIF4G3	hsa-miR-5692a
EIF4G3	hsa-miR-7438-5p
EIF4G3	hsa-miR-1237-3p
EIF4G3	hsa-miR-517a-3p
EIF4G3	hsa-miR-936
EIF4G3	hsa-miR-3595
EIF4G3	hsa-miR-1814a
EIF4G3	hsa-miR-7854-3p
EIF4G3	hsa-miR-2329-3p
EIF4G3	hsa-miR-3577
EIF4G3	hsa-miR-3191-5p
EIF4G3	hsa-miR-7650-5p
EIF4G3	hsa-miR-8886
EIF4G3	hsa-miR-6823-5p
EIF4G3	hsa-miR-2446
EIF4G3	hsa-miR-7022-3p
EIF4G3	hsa-miR-6973a-3p
EIF4G3	hsa-miR-8796
EIF4G3	hsa-miR-4789-5p
EIF4G3	hsa-miR-98-3p
EIF4G3	hsa-miR-6684-5p
EIF4G3	hsa-miR-22-5p
EIF4G3	hsa-miR-1934-5p

EIF4G3	hsa-miR-548c-3p
EIF4G3	hsa-miR-134-3p
EIF4G3	hsa-miR-6868-3p
EIF4G3	hsa-miR-7254-3p
EIF4G3	hsa-miR-6874-3p
EIF4G3	hsa-miR-7341-5p
EIF4G3	hsa-miR-3596d
EIF4G3	hsa-miR-103
EIF4G3	hsa-miR-7270-3p
EIF4G3	hsa-miR-1771
EIF4G3	hsa-miR-378f
EIF4G3	hsa-miR-329-3p
EIF4G3	hsa-miR-6632-5p
EIF4G3	hsa-miR-148b-5p
EIF4G3	hsa-miR-8070
EIF4G3	hsa-miR-2337
EIF4G3	hsa-miR-7110-3p
EIF4G3	hsa-miR-7183-3p
EIF4G3	hsa-miR-3586-3p
EIF4G3	hsa-miR-6620-5p
EIF4G3	hsa-miR-6714-3p
EIF4G3	hsa-miR-2334
EIF4G3	hsa-miR-466f-3p
EIF4G3	hsa-miR-7447-3p
EIF4G3	hsa-miR-2285af
EIF4G3	hsa-miR-7052-3p
EIF4G3	hsa-miR-7174-5p
EIF4G3	hsa-miR-1262-5p
EIF4G3	hsa-miR-713
EIF4G3	hsa-miR-7065-3p
EIF4G3	hsa-miR-7251-3p
EIF4G3	hsa-miR-7398l-3p
EIF4G3	hsa-miR-3941
EIF4G3	hsa-miR-290b-5p
EIF4G3	hsa-miR-525-5p
EIF4G3	hsa-miR-33a-3p
EIF4G3	hsa-miR-496.1
EIF4G3	hsa-miR-7680-3p
EIF4G3	hsa-miR-7059-3p
EIF4G3	hsa-miR-4309
EIF4G3	hsa-miR-6554-5p
EIF4G3	hsa-miR-5589-3p
EIF4G3	hsa-miR-2284ab
EIF4G3	hsa-miR-3082-3p
EIF4G3	hsa-miR-409
EIF4G3	hsa-miR-7515

EIF4G3	hsa-miR-6981-3p
EIF4G3	hsa-miR-329
EIF4G3	hsa-miR-452-5p
EIF4G3	hsa-miR-378-5p
EIF4G3	hsa-miR-1416-5p
EIF4G3	hsa-miR-450b-5p
EIF4G3	hsa-miR-2284u
EIF4G3	hsa-miR-7350-3p
EIF4G3	hsa-miR-7215-3p
EIF4G3	hsa-miR-7386g-3p
EIF4G3	hsa-miR-6083
EIF4G3	hsa-miR-150-5p
EIF4G3	hsa-miR-183-3p
EIF4G3	hsa-miR-6825-5p
EIF4G3	hsa-miR-6068
EIF4G3	hsa-miR-762
EIF4G3	hsa-miR-712-5p
EIF4G3	hsa-miR-372-5p
EIF4G3	hsa-miR-7389-3p
EIF4G3	hsa-miR-7039-3p
EIF4G3	hsa-miR-7323-5p
EIF4G3	hsa-miR-2127
EIF4G3	hsa-miR-2365
EIF4G3	hsa-miR-1641
EIF4G3	hsa-miR-1748
EIF4G3	hsa-miR-6503-3p
EIF4G3	hsa-let-7a-3p
EIF4G3	hsa-miR-7398s-3p
EIF4G3	hsa-miR-7106-3p
EIF4G3	hsa-miR-1552-5p
EIF4G3	hsa-miR-1651-3p
EIF4G3	hsa-miR-488-3p
EIF4G3	hsa-miR-2404
EIF4G3	hsa-miR-6951-5p
EIF4G3	hsa-miR-4643
EIF4G3	hsa-miR-7448-3p
EIF4G3	hsa-miR-7193-3p
EIF4G3	hsa-miR-4762-5p
EIF4G3	hsa-miR-3664-3p
EIF4G3	hsa-miR-378a-3p
EIF4G3	hsa-miR-2284q
EIF4G3	hsa-miR-7298-5p
EIF4G3	hsa-miR-8826
EIF4G3	hsa-miR-7159-3p
EIF4G3	hsa-miR-6901-3p
EIF4G3	hsa-miR-5689

EIF4G3	hsa-miR-381-3p
EIF4G3	hsa-miR-883a-5p
EIF4G3	hsa-miR-6864-5p
EIF4G3	hsa-miR-3156-3p
EIF4G3	hsa-miR-7398r-3p
EIF4G3	hsa-miR-7193-5p
EIF4G3	hsa-miR-216
EIF4G3	hsa-miR-687
EIF4G3	hsa-miR-1912-3p
EIF4G3	hsa-miR-7282-5p
EIF4G3	hsa-miR-2052
EIF4G3	hsa-miR-2284m
EIF4G3	hsa-miR-4301
EIF4G3	hsa-miR-4762-3p
EIF4G3	hsa-miR-4446-5p
EIF4G3	hsa-miR-26-2-3p
EIF4G3	hsa-miR-616-5p
EIF4G3	hsa-miR-300
EIF4G3	hsa-miR-875
EIF4G3	hsa-miR-455-3p
EIF4G3	hsa-miR-6845-3p
EIF4G3	hsa-miR-4765
EIF4G3	hsa-miR-512
EIF4G3	hsa-miR-7474-3p
EIF4G3	hsa-miR-34bc-3p
EIF4G3	hsa-miR-1781-3p
EIF4G3	hsa-miR-6930-3p
EIF4G3	hsa-miR-7188-5p
EIF4G3	hsa-miR-1642
EIF4G3	hsa-miR-7063-5p
EIF4G3	hsa-miR-101-3p.1
EIF4G3	hsa-miR-1603
EIF4G3	hsa-miR-520f-5p
EIF4G3	hsa-miR-6832-3p
EIF4G3	hsa-miR-1241
EIF4G3	hsa-miR-6651-5p
EIF4G3	hsa-miR-3105-5p
EIF4G3	hsa-miR-362-5p
EIF4G3	hsa-miR-4666a-3p
EIF4G3	hsa-miR-466i-3p
EIF4G3	hsa-miR-516-3p
EIF4G3	hsa-miR-7386d-5p
EIF4G3	hsa-miR-191-3p
EIF4G3	hsa-miR-770-5p
EIF4G3	hsa-miR-7338-5p
EIF4G3	hsa-miR-153-5p

EIF4G3	hsa-miR-378d
EIF4G3	hsa-miR-423-5p
EIF4G3	hsa-miR-6667-5p
EIF4G3	hsa-miR-7469-3p
EIF4G3	hsa-miR-6896-3p
EIF4G3	hsa-miR-8829
EIF4G3	hsa-miR-7286-5p
EIF4G3	hsa-miR-1256
EIF4G3	hsa-miR-1798-3p
EIF4G3	hsa-miR-4652-3p
EIF4G3	hsa-miR-293-5p
EIF4G3	hsa-miR-2284x
EIF4G3	hsa-miR-3530-5p
EIF4G3	hsa-miR-7191-5p
EIF4G3	hsa-miR-6887-3p
EIF4G3	hsa-miR-6833-3p
EIF4G3	hsa-miR-149-3p
EIF4G3	hsa-miR-1704
EIF4G3	hsa-miR-5588-5p
EIF4G3	hsa-miR-8839
EIF4G3	hsa-miR-3167-5p
EIF4G3	hsa-miR-6549-3p
EIF4G3	hsa-miR-140-5p
EIF4G3	hsa-miR-7463-3p
EIF4G3	hsa-miR-3679-3p
EIF4G3	hsa-miR-7299-5p
EIF4G3	hsa-miR-374-5p
EIF4G3	hsa-miR-670-3p
EIF4G3	hsa-miR-495-3p
EIF4G3	hsa-miR-8064
EIF4G3	hsa-miR-8844
EIF4G3	hsa-miR-4482-3p
EIF4G3	hsa-miR-2359
EIF4G3	hsa-miR-7678-5p
EIF4G3	hsa-miR-3561-3p
EIF4G3	hsa-miR-539-3p
EIF4G3	hsa-miR-290a-5p
EIF4G3	hsa-miR-6990-5p
EIF4G3	hsa-miR-7371d-5p
EIF4G3	hsa-miR-8841
EIF4G3	hsa-let-7abf-3p
EIF4G3	hsa-miR-7856-5p
EIF4G3	hsa-miR-466b-2-3p
EIF4G3	hsa-miR-1966-3p
EIF4G3	hsa-miR-520gh
EIF4G3	hsa-miR-422

EIF4G3	hsa-miR-7049-5p
EIF4G3	hsa-miR-7169-3p
EIF4G3	hsa-miR-6940-5p
EIF4G3	hsa-miR-2284z
EIF4G3	hsa-miR-1759-5p
EIF4G3	hsa-miR-4428
EIF4G3	hsa-miR-6738-3p
EIF4G3	hsa-miR-26-3p
EIF4G3	hsa-miR-580
EIF4G3	hsa-let-7f-1-3p
EIF4G3	hsa-miR-1550-3p
EIF4G3	hsa-miR-1786
EIF4G3	hsa-miR-7011-3p
EIF4G3	hsa-miR-2373-5p
EIF4G3	hsa-miR-7678-3p
EIF4G3	hsa-miR-19-5p
EIF4G3	hsa-miR-6529b
EIF4G3	hsa-miR-933
EIF4G3	hsa-miR-511-5p
EIF4G3	hsa-miR-3182
EIF4G3	hsa-miR-299b-5p
EIF4G3	hsa-miR-7156-5p
EIF4G3	hsa-miR-140-5p
EIF4G3	hsa-miR-561-3p
EIF4G3	hsa-miR-3613b
EIF4G3	hsa-miR-6873-3p
EIF4G3	hsa-miR-342-5p
EIF4G3	hsa-miR-335-5p
EIF4G3	hsa-miR-294-5p
EIF4G3	hsa-miR-6558-3p
EIF4G3	hsa-miR-655
EIF4G3	hsa-miR-29-5p
EIF4G3	hsa-miR-6358
EIF4G3	hsa-miR-8793
EIF4G3	hsa-miR-2343
EIF4G3	hsa-miR-3616-5p
EIF4G3	hsa-miR-2301
EIF4G3	hsa-miR-19b-5p
EIF4G3	hsa-miR-2970-3p
EIF4G3	hsa-miR-2451
EIF4G3	hsa-miR-1306-5p
EIF4G3	hsa-miR-4744
EIF4G3	hsa-miR-509-3p
EIF4G3	hsa-miR-6613-3p
EIF4G3	hsa-let-7b-3p
EIF4G3	hsa-miR-548t-5p

EIF4G3	hsa-miR-5132-5p
EIF4G3	hsa-miR-5130
EIF4G3	hsa-miR-7032-5p
EIF4G3	hsa-miR-1434-3p
EIF4G3	hsa-miR-3572
EIF4G3	hsa-miR-548n
EIF4G3	hsa-miR-3075-3p
EIF4G3	hsa-miR-577-3p
EIF4G3	hsa-miR-561-3p
EIF4G3	hsa-miR-2116-5p
EIF4G3	hsa-miR-6962-5p
EIF4G3	hsa-miR-626
EIF4G3	hsa-miR-770
EIF4G3	hsa-miR-7386e-5p
EIF4G3	hsa-miR-21-3p
EIF4G3	hsa-miR-1260a
EIF4G3	hsa-miR-6338
EIF4G3	hsa-miR-3102-3p
EIF4G3	hsa-miR-4509
EIF4G3	hsa-miR-496
EIF4G3	hsa-miR-4433a-3p
EIF4G3	hsa-miR-125b-1-3p
EIF4G3	hsa-miR-6842-5p
EIF4G3	hsa-miR-6595-5p
EIF4G3	hsa-miR-7682-5p
EIF4G3	hsa-miR-4720-5p
EIF4G3	hsa-miR-4477a
EIF4G3	hsa-miR-619
EIF4G3	hsa-miR-2284y
EIF4G3	hsa-miR-6572-5p
EIF4G3	hsa-miR-1324
EIF4G3	hsa-miR-6407
EIF4G3	hsa-miR-4516
EIF4G3	hsa-miR-517-3p
EIF4G3	hsa-miR-6401
EIF4G3	hsa-miR-8867
EIF4G3	hsa-miR-7331-2-3p
EIF4G3	hsa-miR-665-3p
EIF4G3	hsa-miR-2293
EIF4G3	hsa-miR-292b-5p
EIF4G3	hsa-miR-3121-3p
EIF4G3	hsa-miR-7442-3p
EIF4G3	hsa-miR-373-3p
EIF4G3	hsa-miR-5702
EIF4G3	hsa-miR-2473
EIF4G3	hsa-miR-7351-3p

EIF4G3	hsa-miR-6761-5p
EIF4G3	hsa-miR-2453
EIF4G3	hsa-miR-3059-5p
EIF4G3	hsa-miR-143-5p
EIF4G3	hsa-miR-224-5p
EIF4G3	hsa-miR-181-5p
EIF4G3	hsa-miR-8881
EIF4G3	hsa-miR-6795-3p
EIF4G3	hsa-miR-2901
EIF4G3	hsa-miR-1250-3p
EIF4G3	hsa-miR-1983
EIF4G3	hsa-miR-466b-4-3p
EIF4G3	hsa-miR-7857
EIF4G3	hsa-miR-1238-3p
EIF4G3	hsa-miR-548az-5p
EIF4G3	hsa-miR-4445-5p
EIF4G3	hsa-miR-214-3p
EIF4G3	hsa-miR-3531-3p
EIF4G3	hsa-miR-335-5p
EIF4G3	hsa-miR-2284n
EIF4G3	hsa-miR-6792-3p
EIF4G3	hsa-miR-362-3p
EIF4G3	hsa-miR-7366-5p
EIF4G3	hsa-miR-544a
EIF4G3	hsa-miR-6794-3p
EIF4G3	hsa-miR-154-5p
EIF4G3	hsa-miR-1910-5p
EIF4G3	hsa-miR-4699-3p
EIF4G3	hsa-miR-302
EIF4G3	hsa-miR-6528
EIF4G3	hsa-miR-1249-5p
EIF4G3	hsa-miR-290
EIF4G3	hsa-miR-1305
EIF4G3	hsa-miR-7648-3p
EIF4G3	hsa-miR-7031-5p
EIF4G3	hsa-miR-292a-5p
EIF4G3	hsa-miR-5125-5p
EIF4G3	hsa-miR-7680-5p
EIF4G3	hsa-miR-6883-5p
EIF4G3	hsa-miR-146a-3p
EIF4G3	hsa-miR-3200-3p
EIF4G3	hsa-miR-4452
EIF4G3	hsa-miR-1894-3p
EIF4G3	hsa-miR-4653-3p
EIF4G3	hsa-miR-1711
EIF4G3	hsa-miR-7078-3p

EIF4G3	hsa-miR-2466-5p
EIF4G3	hsa-miR-216a
EIF4G3	hsa-miR-147a
EIF4G3	hsa-miR-695
EIF4G3	hsa-miR-518-5p
EIF4G3	hsa-miR-7469-5p
EIF4G3	hsa-miR-7200-5p
EIF4G3	hsa-miR-7212-3p
EIF4G3	hsa-miR-6808-5p
EIF4G3	hsa-miR-7657-3p
EIF4G3	hsa-miR-154a
EIF4G3	hsa-miR-361-3p
EIF4G3	hsa-miR-3066-3p
EIF4G3	hsa-miR-1278
EIF4G3	hsa-miR-145-5p
EIF4G3	hsa-miR-2385-3p
EIF4G3	hsa-miR-326
EIF4G3	hsa-miR-7262b-5p
EIF4G3	hsa-miR-7188-3p
EIF4G3	hsa-miR-142-5p
EIF4G3	hsa-miR-4738-3p
EIF4G3	hsa-miR-7372-5p
EIF4G3	hsa-miR-669b-5p
EIF4G3	hsa-miR-8096
EIF4G3	hsa-miR-432-5p
EIF4G3	hsa-miR-3664-5p
EIF4G3	hsa-miR-573
EIF4G3	hsa-miR-4258
EIF4G3	hsa-miR-575
EIF4G3	hsa-miR-378-3p
EIF4G3	hsa-miR-6628-3p
EIF4G3	hsa-miR-1896
EIF4G3	hsa-miR-1720-5p
EIF4G3	hsa-miR-452-5p
EIF4G3	hsa-miR-467f
EIF4G3	hsa-miR-6627-3p
EIF4G3	hsa-miR-8802
EIF4G3	hsa-miR-497-3p
EIF4G3	hsa-miR-378h
EIF4G3	hsa-miR-3587
EIF4G3	hsa-miR-371b-5p
EIF4G3	hsa-miR-375-3p
EIF4G3	hsa-miR-7480-5p
EIF4G3	hsa-miR-1655-3p
EIF4G3	hsa-miR-106a-3p
EIF4G3	hsa-miR-532-5p

EIF4G3	hsa-miR-517
EIF4G3	hsa-miR-2285x
EIF4G3	hsa-miR-466l-3p
EIF4G3	hsa-miR-382-5p
EIF4G3	hsa-miR-345-3p
EIF4G3	hsa-let-7j-3p
EIF4G3	hsa-miR-17-3p
EIF4G3	hsa-miR-520g-3p
EIF4G3	hsa-miR-4636
EIF4G3	hsa-miR-2285b
EIF4G3	hsa-miR-150-5p
EIF4G3	hsa-miR-4472
EIF4G3	hsa-miR-141-3p
EIF4G3	hsa-miR-4713-5p
EIF4G3	hsa-miR-7290-3p
EIF4G3	hsa-miR-202-3p
EIF4G3	hsa-miR-1844
EIF4G3	hsa-miR-514b-5p
EIF4G3	hsa-miR-1647
EIF4G3	hsa-let-7f-2-3p
EIF4G3	hsa-miR-1657
EIF4G3	hsa-miR-10
EIF4G3	hsa-miR-2471-3p
EIF4G3	hsa-miR-302a-3p
EIF4G3	hsa-miR-3057-5p
EIF4G3	hsa-miR-6871-3p
EIF4G3	hsa-miR-666-3p
EIF4G3	hsa-miR-378i
EIF4G3	hsa-miR-876-5p
EIF4G3	hsa-miR-3105-3p
EIF4G3	hsa-miR-432-5p
EIF4G3	hsa-miR-6951-3p
EIF4G3	hsa-miR-130b-5p
EIF4G3	hsa-miR-7677-3p
EIF4G3	hsa-miR-6649-5p
EIF4G3	hsa-miR-7006-3p
EIF4G3	hsa-miR-570-3p
EIF4G3	hsa-miR-6955-3p
EIF4G3	hsa-miR-6835-5p
EIF4G3	hsa-miR-4753-3p
EIF4G3	hsa-miR-4671-3p
EIF4G3	hsa-miR-129-5p
EIF4G3	hsa-miR-6802-3p
EIF4G3	hsa-miR-8823
EIF4G3	hsa-miR-8838
EIF4G3	hsa-miR-937-5p

EIF4G3	hsa-miR-7385b-3p
EIF4G3	hsa-miR-709
EIF4G3	hsa-miR-532-3p
EIF4G3	hsa-miR-107-3p
EIF4G3	hsa-miR-6752-5p
EIF4G3	hsa-miR-205-5p
EIF4G3	hsa-miR-6893-5p
EIF4G3	hsa-miR-470-5p
EIF4G3	hsa-miR-1301-5p
EIF4G3	hsa-miR-6216
EIF4G3	hsa-miR-1460
EIF4G3	hsa-miR-495-5p
EIF4G3	hsa-miR-7650-3p
EIF4G3	hsa-miR-4514
EIF4G3	hsa-miR-145-5p
EIF4G3	hsa-miR-1190
EIF4G3	hsa-miR-7222-3p
EIF4G3	hsa-miR-29a-1-5p
EIF4G3	hsa-miR-6826-3p
EIF4G3	hsa-miR-513c-5p
EIF4G3	hsa-miR-3536
EIF4G3	hsa-miR-7027-3p
EIF4G3	hsa-miR-1544-3p
EIF4G3	hsa-miR-544a
EIF4G3	hsa-miR-541-3p
EIF4G3	hsa-miR-6565-3p
EIF4G3	hsa-miR-29b-5p
EIF4G3	hsa-miR-7020-3p
EIF4G3	hsa-miR-378
EIF4G3	hsa-miR-942-5p
EIF4G3	hsa-miR-1756b
EIF4G3	hsa-miR-95-5p
EIF4G3	hsa-miR-4310
EIF4G3	hsa-miR-8825
EIF4G3	hsa-miR-140-3p.1
EIF4G3	hsa-miR-703
EIF4G3	hsa-miR-292-5p
EIF4G3	hsa-miR-513a-3p
EIF4G3	hsa-miR-3144-3p
EIF4G3	hsa-miR-2362
EIF4G3	hsa-miR-3198
EIF4G3	hsa-miR-4639-3p
EIF4G3	hsa-miR-6938-5p
EIF4G3	hsa-miR-26-5p
EIF4G3	hsa-miR-509-3p
EIF4G3	hsa-miR-3924

EIF4G3	hsa-miR-6997-3p
EIF4G3	hsa-miR-655-3p
EIF4G3	hsa-miR-6639-5p
EIF4G3	hsa-miR-6126
EIF4G3	hsa-miR-633
EIF4G3	hsa-miR-5584-5p
EIF4G3	hsa-miR-543-5p
EIF4G3	hsa-miR-6331
EIF4G3	hsa-miR-7035-5p
EIF4G3	hsa-miR-6803-5p
EIF4G3	hsa-miR-3200-3p
EIF4G3	hsa-miR-3615
EIF4G3	hsa-miR-2421
EIF4G3	hsa-miR-467-3p
EIF4G3	hsa-miR-148-5p
EIF4G3	hsa-miR-1721
EIF4G3	hsa-miR-8068
EIF4G3	hsa-miR-6797-5p
EIF4G3	hsa-miR-320-5p
EIF4G3	hsa-miR-224-5p
EIF4G3	hsa-miR-3591-5p
EIF4G3	hsa-miR-375
EIF4G3	hsa-miR-297
EIF4G3	hsa-miR-532-5p
EIF4G3	hsa-miR-1643-3p
EIF4G3	hsa-miR-548av-3p
EIF4G3	hsa-miR-455-3p.2
EIF4G3	hsa-miR-579-3p
EIF4G3	hsa-miR-2325c
EIF4G3	hsa-miR-223-5p
EIF4G3	hsa-miR-876
EIF4G3	hsa-miR-5619-3p
EIF4G3	hsa-miR-2284a
EIF4G3	hsa-miR-101-3p
EIF4G3	hsa-miR-92b-5p
EIF4G3	hsa-miR-4639-5p
EIF4G3	hsa-miR-2284aa
EIF4G3	hsa-miR-1904
EIF4G3	hsa-miR-7319-5p
EIF4G3	hsa-miR-7665-5p
EIF4G3	hsa-miR-4691-5p
EIF4G3	hsa-miR-4447
EIF4G3	hsa-miR-6924-3p
EIF4G3	hsa-miR-5586-5p
EIF4G3	hsa-miR-26b-3p
EIF4G3	hsa-miR-22-3p

EIF4G3	hsa-miR-607
EIF4G3	hsa-miR-2284j
EIF4G3	hsa-miR-7661-5p
EIF4G3	hsa-miR-379-3p
EIF4G3	hsa-miR-29-3p
EIF4G3	hsa-miR-340-3p
EIF4G3	hsa-miR-1737
EIF4G3	hsa-miR-511
EIF4G3	hsa-miR-8843
EIF4G3	hsa-miR-3609
EIF4G3	hsa-miR-7386g-5p
EIF4G3	hsa-miR-6608-3p
EIF4G3	hsa-miR-7347-3p
EIF4G3	hsa-miR-7276-3p
EIF4G3	hsa-miR-4670-3p
EIF4G3	hsa-miR-6751-5p
EIF4G3	hsa-miR-411-3p
EIF4G3	hsa-miR-7110-5p
EIF4G3	hsa-miR-4518
EIF4G3	hsa-miR-2310
EIF4G3	hsa-miR-1248
EIF4G3	hsa-miR-3973
EIF4G3	hsa-miR-7481-5p
EIF4G3	hsa-miR-4307
EIF4G3	hsa-miR-369-3p
EIF4G3	hsa-miR-7244-3p
EIF4G3	hsa-miR-1933-3p
EIF4G3	hsa-miR-130a-5p
EIF4G3	hsa-miR-6582-3p
EIF4G3	hsa-miR-8884
EIF4G3	hsa-miR-7034-3p
EIF4G3	hsa-miR-603
EIF4G3	hsa-miR-3140-3p
EIF4G3	hsa-miR-548c-3p
EIF4G3	hsa-miR-4679
EIF4G3	hsa-miR-4459
EIF4G3	hsa-miR-6134
EIF4G3	hsa-miR-31-3p
EIF4G3	hsa-miR-1625-3p
EIF4G3	hsa-miR-8848
EIF4G3	hsa-miR-1597-5p
EIF4G3	hsa-miR-6885-3p
EIF4G3	hsa-miR-6782-3p
EIF4G3	hsa-miR-378c
EIF4G3	hsa-miR-1910-3p
EIF4G3	hsa-miR-520-3p

EIF4G3	hsa-miR-3533
EIF4G3	hsa-miR-7293-5p
EIF4G3	hsa-miR-674-3p
EIF4G3	hsa-miR-147-3p
EIF4G3	hsa-miR-4768-5p
EIF4G3	hsa-miR-520f-3p
EIF4G3	hsa-miR-7691
EIF4G3	hsa-miR-2393
EIF4G3	hsa-miR-2285
EIF4G3	hsa-miR-295-5p
EIF4G3	hsa-miR-1304-3p
EIF4G3	hsa-miR-659-3p
EIF4G3	hsa-miR-1814
EIF4G3	hsa-miR-3675-3p
EIF4G3	hsa-miR-6119-3p
EIF4G3	hsa-let-7afk-3p
EIF4G3	hsa-miR-7333-5p
EIF4G3	hsa-miR-3083-5p
EIF4G3	hsa-miR-503-3p
EIF4G3	hsa-miR-5623-3p
EIF4G3	hsa-miR-6545-5p
EIF4G3	hsa-miR-3473f
EIF4G3	hsa-miR-2284v
EIF4G3	hsa-miR-2367-3p
EIF4G3	hsa-miR-7371j-5p
EIF4G3	hsa-miR-875-5p
EIF4G3	hsa-miR-183-5p
EIF4G3	hsa-miR-2305
EIF4G3	hsa-miR-2116-3p
EIF4G3	hsa-miR-1663-5p
EIF4G3	hsa-miR-6502-5p
EIF4G3	hsa-miR-6516
EIF4G3	hsa-miR-652-3p
EIF4G3	hsa-miR-103-5p
EIF4G3	hsa-miR-4273
EIF4G3	hsa-miR-940
EIF4G3	hsa-miR-434-3p
EIF4G3	hsa-miR-18b-3p
EIF4G3	hsa-miR-129b-5p
EIF4G3	hsa-miR-7864
EIF4G3	hsa-miR-7398e-3p
EIF4G3	hsa-miR-7459-5p
EIF4G3	hsa-miR-200a-3p
EIF4G3	hsa-miR-6858-3p
EIF4G3	hsa-miR-2284-5p
EIF4G3	hsa-miR-3089-3p

EIF4G3	hsa-miR-3090-3p
EIF4G3	hsa-miR-373-5p
EIF4G3	hsa-miR-763
EIF4G3	hsa-miR-6892-3p
EIF4G3	hsa-miR-1930-5p
EIF4G3	hsa-miR-7466-3p
EIF4G3	hsa-miR-6749-3p
EIF4G3	hsa-miR-6757-3p
EIF4G3	hsa-miR-6958-3p
EIF4G3	hsa-miR-627-3p
EIF4G3	hsa-miR-2284d
EIF4G3	hsa-miR-6785-5p
EIF4G3	hsa-let-7-3p
EIF4G3	hsa-miR-2284c
EIF4G3	hsa-miR-1773-5p
EIF4G3	hsa-miR-6511a-5p
EIF4G3	hsa-miR-371a-5p
EIF4G3	hsa-miR-2388-5p
EIF4G3	hsa-miR-2342
EIF4G3	hsa-miR-1897-5p
EIF4G3	hsa-miR-107-5p
EIF4G3	hsa-miR-2285z
EIF4G3	hsa-miR-4433b-3p
EIF4G3	hsa-miR-7394b-5p
EIF4G3	hsa-miR-7114-5p
EIF4G3	hsa-miR-6345
EIF4G3	hsa-miR-7166-5p
EIF4G3	hsa-miR-6516-5p
EIF4G3	hsa-miR-3594-5p
EIF4G3	hsa-miR-361-5p
EIF4G3	hsa-miR-512-3p
EIF4G3	hsa-miR-7116-3p
EIF4G3	hsa-miR-1827
EIF4G3	hsa-miR-6548-3p
EIF4G3	hsa-miR-8824
EIF4G3	hsa-miR-8062
EIF4G3	hsa-miR-7437-3p
EIF4G3	hsa-miR-1948-5p
EIF4G3	hsa-miR-488-3p
EIF4G3	hsa-miR-7258-3p
EIF4G3	hsa-miR-7226-5p
EIF4G3	hsa-miR-498
EIF4G3	hsa-miR-378b
EIF4G3	hsa-miR-7257-3p
EIF4G3	hsa-miR-339-3p
EIF4G3	hsa-miR-1251-3p

EIF4G3	hsa-miR-196b-3p
EIF4G3	hsa-miR-6728-3p
EIF4G3	hsa-miR-1266-5p
EIF4G3	hsa-miR-4666a-5p
EIF4G3	hsa-miR-450a
EIF4G3	hsa-miR-1693
EIF4G3	hsa-miR-1185-3p
EIF4G3	hsa-miR-3145-3p
EIF4G3	hsa-miR-6739-3p
EIF4G3	hsa-miR-3065-3p
EIF4G3	hsa-miR-6650-5p
EIF4G3	hsa-miR-6813-3p
EIF4G3	hsa-miR-378e
EIF4G3	hsa-miR-7278-3p
EIF4G3	hsa-miR-382-5p
EIF4G3	hsa-miR-21-5p
EIF4G3	hsa-miR-3080-3p
EIF4G3	hsa-miR-3547
EIF4G3	hsa-miR-665
EIF4G3	hsa-miR-6540-3p
EIF4G3	hsa-miR-7331-1-3p
EIF4G3	hsa-miR-540-5p
EIF4G3	hsa-miR-513c-3p
EIF4G3	hsa-miR-7386h-5p
EIF4G3	hsa-miR-2399-5p
EIF4G3	hsa-miR-429-5p
EIF4G3	hsa-miR-4464
GEMIN5	hsa-miR-1245a
GEMIN5	hsa-miR-6998-3p
GEMIN5	hsa-miR-21-3p
GEMIN5	hsa-miR-6808-5p
GEMIN5	hsa-miR-6989-5p
GEMIN5	hsa-miR-7398t-3p
GEMIN5	hsa-miR-296-5p
GEMIN5	hsa-miR-124
GEMIN5	hsa-miR-6507-5p
GEMIN5	hsa-miR-8880
GEMIN5	hsa-miR-7068-3p
GEMIN5	hsa-miR-3974
GEMIN5	hsa-miR-1199-5p
GEMIN5	hsa-miR-505-3p.1
GEMIN5	hsa-miR-2285r
GEMIN5	hsa-miR-3559-5p
GEMIN5	hsa-miR-33-3p
GEMIN5	hsa-miR-548q
GEMIN5	hsa-miR-3562

GEMIN5	hsa-miR-2284l
GEMIN5	hsa-miR-6944-5p
GEMIN5	hsa-miR-548ay-3p
GEMIN5	hsa-miR-292a-5p
GEMIN5	hsa-miR-548f-3p
GEMIN5	hsa-miR-2285j
GEMIN5	hsa-miR-1230
GEMIN5	hsa-miR-4322
GEMIN5	hsa-miR-371
GEMIN5	hsa-miR-34b-3p
GEMIN5	hsa-miR-568
GEMIN5	hsa-miR-642a-5p
GEMIN5	hsa-miR-6719-3p
GEMIN5	hsa-miR-507b
GEMIN5	hsa-miR-181c-3p
GEMIN5	hsa-miR-6823-5p
GEMIN5	hsa-miR-3569
GEMIN5	hsa-miR-1245b-3p
GEMIN5	hsa-miR-369-3p
GEMIN5	hsa-miR-5118
GEMIN5	hsa-miR-607
GEMIN5	hsa-miR-3108-5p
GEMIN5	hsa-miR-3057-3p
GEMIN5	hsa-miR-548aj-5p
GEMIN5	hsa-miR-4792
GEMIN5	hsa-miR-3658
GEMIN5	hsa-miR-6905-3p
GEMIN5	hsa-miR-880-3p
GEMIN5	hsa-miR-505-3p
GEMIN5	hsa-miR-379-3p
GEMIN5	hsa-miR-7217-5p
GEMIN5	hsa-miR-3533
GEMIN5	hsa-miR-7017-3p
GEMIN5	hsa-miR-6907-5p
GEMIN5	hsa-miR-7087-3p
GEMIN5	hsa-miR-17-5p
GEMIN5	hsa-miR-7854-3p
GEMIN5	hsa-miR-450b-3p
GEMIN5	hsa-miR-2339
GEMIN5	hsa-miR-3964
GEMIN5	hsa-miR-7379-5p
GEMIN5	hsa-miR-205-3p
GEMIN5	hsa-miR-6888-5p
GEMIN5	hsa-miR-374
GEMIN5	hsa-miR-570-3p
GEMIN5	hsa-miR-7116-5p

GEMIN5	hsa-miR-6971-3p
GEMIN5	hsa-miR-1262
GEMIN5	hsa-miR-579
GEMIN5	hsa-miR-6922-3p
GEMIN5	hsa-miR-3136-5p
GEMIN5	hsa-miR-7396-3p
GEMIN5	hsa-miR-500b-5p
GEMIN5	hsa-miR-224-5p
GEMIN5	hsa-miR-581
GEMIN5	hsa-miR-7351-5p
GEMIN5	hsa-miR-654-5p
GEMIN5	hsa-miR-1184
GEMIN5	hsa-miR-338-5p
GEMIN5	hsa-miR-656
GEMIN5	hsa-miR-4763-3p
GEMIN5	hsa-miR-690
GEMIN5	hsa-miR-6715b-3p
GEMIN5	hsa-miR-3151-3p
GEMIN5	hsa-miR-20
GEMIN5	hsa-miR-548x-5p
GEMIN5	hsa-miR-570-3p
GEMIN5	hsa-miR-186-5p
GEMIN5	hsa-miR-1546-5p
GEMIN5	hsa-miR-3154
GEMIN5	hsa-miR-642-3p
GEMIN5	hsa-miR-4256
GEMIN5	hsa-miR-6770-5p
GEMIN5	hsa-miR-6741-5p
GEMIN5	hsa-miR-331-5p
GEMIN5	hsa-miR-344f-5p
GEMIN5	hsa-miR-943
GEMIN5	hsa-miR-2285
GEMIN5	hsa-miR-215-3p
GEMIN5	hsa-miR-7280-5p
GEMIN5	hsa-miR-2284j
GEMIN5	hsa-miR-7039-3p
GEMIN5	hsa-miR-7386o-5p
GEMIN5	hsa-miR-362-3p
GEMIN5	hsa-miR-6817-5p
GEMIN5	hsa-miR-6758-5p
GEMIN5	hsa-miR-1550-3p
GEMIN5	hsa-miR-551-5p
GEMIN5	hsa-miR-105b
GEMIN5	hsa-miR-4799-5p
GEMIN5	hsa-miR-1912-3p
GEMIN5	hsa-miR-8810

GEMIN5	hsa-miR-143-3p
GEMIN5	hsa-miR-1468-3p
GEMIN5	hsa-miR-4714-3p
GEMIN5	hsa-miR-1251-5p
GEMIN5	hsa-miR-4769-3p
GEMIN5	hsa-miR-323b-3p
GEMIN5	hsa-miR-548ax
GEMIN5	hsa-miR-4786-5p
GEMIN5	hsa-miR-4693-5p
GEMIN5	hsa-miR-7228-3p
GEMIN5	hsa-miR-548g-5p
GEMIN5	hsa-miR-7169-3p
GEMIN5	hsa-miR-660-5p
GEMIN5	hsa-miR-7053-5p
GEMIN5	hsa-miR-6936-5p
GEMIN5	hsa-miR-656-3p
GEMIN5	hsa-miR-4744
GEMIN5	hsa-miR-7672-5p
GEMIN5	hsa-miR-34b-5p
GEMIN5	hsa-miR-2387
GEMIN5	hsa-miR-124-3p.2
GEMIN5	hsa-miR-433-3p
GEMIN5	hsa-miR-9-5p
GEMIN5	hsa-miR-7028-3p
GEMIN5	hsa-miR-208a-5p
GEMIN5	hsa-miR-373-5p
GEMIN5	hsa-miR-200-3p
GEMIN5	hsa-miR-532-3p
GEMIN5	hsa-miR-2489
GEMIN5	hsa-miR-6864-5p
GEMIN5	hsa-miR-6792-5p
GEMIN5	hsa-miR-7011-5p
GEMIN5	hsa-miR-3089-3p
GEMIN5	hsa-miR-132-3p
GEMIN5	hsa-miR-449c-5p
GEMIN5	hsa-miR-3526
GEMIN5	hsa-miR-5107-5p
GEMIN5	hsa-miR-1200
GEMIN5	hsa-miR-5582-3p
GEMIN5	hsa-miR-6403
GEMIN5	hsa-miR-515-5p
GEMIN5	hsa-miR-26-3p
GEMIN5	hsa-miR-548ac
GEMIN5	hsa-miR-127-5p
GEMIN5	hsa-miR-700-5p
GEMIN5	hsa-miR-337-3p

GEMIN5	hsa-miR-2303
GEMIN5	hsa-miR-3925-3p
GEMIN5	hsa-miR-4666a-5p
GEMIN5	hsa-miR-616-5p
GEMIN5	hsa-miR-3606-3p
GEMIN5	hsa-miR-214-3p
GEMIN5	hsa-miR-924
GEMIN5	hsa-miR-6507-3p
GEMIN5	hsa-miR-7685-5p
GEMIN5	hsa-miR-627-3p
GEMIN5	hsa-miR-425-5p
GEMIN5	hsa-miR-1205
GEMIN5	hsa-miR-2284b
GEMIN5	hsa-miR-7051-3p
GEMIN5	hsa-miR-433
GEMIN5	hsa-miR-7201-5p
GEMIN5	hsa-miR-4540
GEMIN5	hsa-miR-484
GEMIN5	hsa-miR-548f-5p
GEMIN5	hsa-miR-186-3p
GEMIN5	hsa-miR-7063-5p
GEMIN5	hsa-miR-4778-3p
GEMIN5	hsa-miR-3119
GEMIN5	hsa-miR-292b-5p
GEMIN5	hsa-miR-4282
GEMIN5	hsa-miR-7-3p
GEMIN5	hsa-miR-7182-3p
GEMIN5	hsa-miR-500a-5p
GEMIN5	hsa-miR-8068
GEMIN5	hsa-miR-7006-3p
GEMIN5	hsa-miR-5585-5p
GEMIN5	hsa-miR-494-3p
GEMIN5	hsa-miR-548ae-3p
GEMIN5	hsa-miR-6924-5p
GEMIN5	hsa-miR-1981-5p
GEMIN5	hsa-miR-548ao-5p
GEMIN5	hsa-miR-6988-5p
GEMIN5	hsa-miR-3653-3p
GEMIN5	hsa-miR-742-3p
GEMIN5	hsa-miR-181b-2-3p
GEMIN5	hsa-miR-351-3p
GEMIN5	hsa-miR-320a
GEMIN5	hsa-miR-2466-5p
GEMIN5	hsa-miR-5007-3p
GEMIN5	hsa-miR-676-5p
GEMIN5	hsa-miR-6736-5p

GEMIN5	hsa-miR-7365-3p
GEMIN5	hsa-miR-1305
GEMIN5	hsa-miR-6927-3p
GEMIN5	hsa-miR-6959-5p
GEMIN5	hsa-miR-383
GEMIN5	hsa-miR-4418
GEMIN5	hsa-miR-7850-5p
GEMIN5	hsa-miR-7005-5p
GEMIN5	hsa-miR-203
GEMIN5	hsa-miR-3059-3p
GEMIN5	hsa-miR-143-3p
GEMIN5	hsa-miR-8069
GEMIN5	hsa-miR-130b-5p
GEMIN5	hsa-miR-17-3p
GEMIN5	hsa-miR-6958-3p
GEMIN5	hsa-miR-153-5p
GEMIN5	hsa-miR-7019-5p
GEMIN5	hsa-miR-7048-5p
GEMIN5	hsa-miR-3660
GEMIN5	hsa-miR-4710
GEMIN5	hsa-miR-7118-5p
GEMIN5	hsa-miR-548i-5p
GEMIN5	hsa-miR-1182
GEMIN5	hsa-miR-425
GEMIN5	hsa-miR-191-3p
GEMIN5	hsa-miR-2284i
GEMIN5	hsa-miR-136-5p
GEMIN5	hsa-miR-561-3p
GEMIN5	hsa-miR-1967
GEMIN5	hsa-miR-2347
GEMIN5	hsa-miR-6529-5p
GEMIN5	hsa-miR-3920
GEMIN5	hsa-miR-335-3p
GEMIN5	hsa-miR-4265
GEMIN5	hsa-miR-29a-5p
GEMIN5	hsa-miR-592
GEMIN5	hsa-miR-6974-3p
GEMIN5	hsa-miR-664
GEMIN5	hsa-miR-7231-5p
GEMIN5	hsa-miR-411-3p
GEMIN5	hsa-miR-509-5p
GEMIN5	hsa-miR-875-5p
GEMIN5	hsa-miR-561-3p
GEMIN5	hsa-miR-8079
GEMIN5	hsa-miR-7849-3p
GEMIN5	hsa-miR-100-3p

GEMIN5	hsa-miR-1298-3p
GEMIN5	hsa-miR-761
GEMIN5	hsa-miR-4503
GEMIN5	hsa-miR-96-5p
GEMIN5	hsa-miR-677
GEMIN5	hsa-miR-199-5p
GEMIN5	hsa-miR-1257
GEMIN5	hsa-miR-1188-5p
GEMIN5	hsa-miR-1245a
GEMIN5	hsa-miR-186-5p
GEMIN5	hsa-miR-6581-5p
GEMIN5	hsa-miR-34
GEMIN5	hsa-miR-654-3p
GEMIN5	hsa-miR-3594-5p
GEMIN5	hsa-miR-6943-5p
GEMIN5	hsa-miR-488-3p
GEMIN5	hsa-miR-2284z
GEMIN5	hsa-miR-200a-3p
GEMIN5	hsa-miR-590-3p
GEMIN5	hsa-miR-105-5p
GEMIN5	hsa-miR-5584-3p
GEMIN5	hsa-miR-7212-5p
GEMIN5	hsa-miR-8868
GEMIN5	hsa-miR-30--3p
GEMIN5	hsa-miR-2480
GEMIN5	hsa-miR-2284u
GEMIN5	hsa-miR-3099
GEMIN5	hsa-miR-3136-5p
GEMIN5	hsa-miR-5623-5p
GEMIN5	hsa-miR-7283-5p
GEMIN5	hsa-miR-3084-5p
GEMIN5	hsa-miR-2284ab
GEMIN5	hsa-miR-26b-3p
GEMIN5	hsa-miR-2312
GEMIN5	hsa-miR-1271-5p
GEMIN5	hsa-miR-770-5p
GEMIN5	hsa-miR-666-3p
GEMIN5	hsa-miR-4279
GEMIN5	hsa-miR-2285h
GEMIN5	hsa-miR-200a-3p
GEMIN5	hsa-miR-3163
GEMIN5	hsa-miR-132-3p
GEMIN5	hsa-miR-9-5p
GEMIN5	hsa-miR-217-3p
GEMIN5	hsa-miR-2284w
GEMIN5	hsa-miR-3158-5p

GEMIN5	hsa-miR-31-3p
GEMIN5	hsa-miR-7215-5p
GEMIN5	hsa-miR-3093-3p
GEMIN5	hsa-miR-5098
GEMIN5	hsa-miR-1271-5p
GEMIN5	hsa-miR-5009-3p
GEMIN5	hsa-miR-7055-3p
GEMIN5	hsa-miR-196-1-3p
GEMIN5	hsa-miR-7347-5p
GEMIN5	hsa-miR-7652-3p
GEMIN5	hsa-miR-3618-5p
GEMIN5	hsa-miR-373-3p
GEMIN5	hsa-miR-4456
GEMIN5	hsa-miR-513c-3p
GEMIN5	hsa-miR-7200-3p
GEMIN5	hsa-miR-665
GEMIN5	hsa-miR-3068-3p
GEMIN5	hsa-miR-8108
GEMIN5	hsa-miR-6074
GEMIN5	hsa-miR-7340-5p
GEMIN5	hsa-miR-490-3p
GEMIN5	hsa-miR-4316
GEMIN5	hsa-miR-2352
GEMIN5	hsa-miR-194-5p
GEMIN5	hsa-miR-1231
GEMIN5	hsa-miR-5120
GEMIN5	hsa-miR-629-3p
GEMIN5	hsa-miR-548x-3p
GEMIN5	hsa-miR-377-3p
GEMIN5	hsa-miR-2284c
GEMIN5	hsa-miR-6931-5p
GEMIN5	hsa-miR-5616-3p
GEMIN5	hsa-miR-877-3p
GEMIN5	hsa-miR-6950-5p
GEMIN5	hsa-miR-200bc-3p
GEMIN5	hsa-miR-1191a
GEMIN5	hsa-miR-149-5p
GEMIN5	hsa-miR-6906-3p
GEMIN5	hsa-miR-181a-2-3p
GEMIN5	hsa-miR-3121-3p
GEMIN5	hsa-miR-505-3p
GEMIN5	hsa-miR-548ap-3p
GEMIN5	hsa-miR-2468
GEMIN5	hsa-miR-6929-5p
GEMIN5	hsa-miR-4659b-3p
GEMIN5	hsa-miR-2917

GEMIN5	hsa-miR-548at-3p
GEMIN5	hsa-miR-146
GEMIN5	hsa-miR-6973b-5p
GEMIN5	hsa-miR-7355-3p
GEMIN5	hsa-miR-3475-3p
GEMIN5	hsa-miR-640
GEMIN5	hsa-miR-6875-3p
GEMIN5	hsa-miR-340-5p
GEMIN5	hsa-miR-3665
GEMIN5	hsa-miR-6832-5p
GEMIN5	hsa-miR-6893-5p
GEMIN5	hsa-miR-351-5p
GEMIN5	hsa-miR-330-5p
GEMIN5	hsa-miR-433-5p
GEMIN5	hsa-miR-2370-3p
GEMIN5	hsa-miR-33-5p
GEMIN5	hsa-miR-2359
GEMIN5	hsa-miR-6945-3p
GEMIN5	hsa-miR-101-3p
GEMIN5	hsa-miR-4699-3p
GEMIN5	hsa-miR-5132-3p
GEMIN5	hsa-miR-1950
GEMIN5	hsa-miR-96-5p
GEMIN5	hsa-miR-7014-5p
GEMIN5	hsa-miR-670-5p
GEMIN5	hsa-miR-1892
GEMIN5	hsa-miR-2284m
GEMIN5	hsa-miR-181
GEMIN5	hsa-miR-4526
GEMIN5	hsa-miR-202-5p
GEMIN5	hsa-miR-449a
GEMIN5	hsa-miR-32-3p
GEMIN5	hsa-miR-504-5p
GEMIN5	hsa-miR-7202-5p
GEMIN5	hsa-miR-5586-3p
GEMIN5	hsa-miR-7-5p
GEMIN5	hsa-miR-6334
GEMIN5	hsa-miR-519e-5p
GEMIN5	hsa-miR-936
GEMIN5	hsa-miR-4296
GEMIN5	hsa-miR-362-5p
GEMIN5	hsa-miR-7440-3p
GEMIN5	hsa-miR-199-3p
GEMIN5	hsa-miR-3115
GEMIN5	hsa-miR-342-5p
GEMIN5	hsa-miR-1229-5p

GEMIN5	hsa-miR-7042-3p
GEMIN5	hsa-miR-379-5p
GEMIN5	hsa-miR-449a-3p
GEMIN5	hsa-miR-219-2-3p
GEMIN5	hsa-miR-1288-5p
GEMIN5	hsa-miR-548aw
GEMIN5	hsa-miR-513c-5p
GEMIN5	hsa-miR-6972-5p
GEMIN5	hsa-miR-2909
GEMIN5	hsa-miR-124-5p
GEMIN5	hsa-miR-677-3p
GEMIN5	hsa-miR-144-3p
GEMIN5	hsa-miR-5187-3p
GEMIN5	hsa-miR-6939-5p
GEMIN5	hsa-miR-6932-3p
GEMIN5	hsa-miR-506-3p
GEMIN5	hsa-miR-3149
GEMIN5	hsa-miR-7023-3p
GEMIN5	hsa-miR-548bd-3p
GEMIN5	hsa-miR-4299
GEMIN5	hsa-miR-6938-3p
GEMIN5	hsa-miR-4694-5p
GEMIN5	hsa-miR-673-5p
GEMIN5	hsa-miR-2439-5p
GEMIN5	hsa-miR-323
GEMIN5	hsa-miR-4668-5p
GEMIN5	hsa-miR-504
GEMIN5	hsa-miR-375
GEMIN5	hsa-miR-5100
GEMIN5	hsa-miR-185-3p
GEMIN5	hsa-miR-7150
GEMIN5	hsa-miR-494
GEMIN5	hsa-miR-3067-5p
GEMIN5	hsa-miR-1843-3p
GEMIN5	hsa-miR-1902
GEMIN5	hsa-miR-463-5p
GEMIN5	hsa-miR-1953
GEMIN5	hsa-miR-4661-3p
GEMIN5	hsa-miR-1250-3p
GEMIN5	hsa-miR-4659a-3p
GEMIN5	hsa-miR-6964-5p
GEMIN5	hsa-miR-548h-5p
GEMIN5	hsa-miR-6520
GEMIN5	hsa-miR-2891
GEMIN5	hsa-miR-27a-5p
GEMIN5	hsa-miR-580

GEMIN5	hsa-miR-6979-5p
GEMIN5	hsa-miR-34-5p
GEMIN5	hsa-miR-2375
GEMIN5	hsa-miR-7855-5p
GEMIN5	hsa-miR-7185-3p
GEMIN5	hsa-miR-181a-3p
GEMIN5	hsa-miR-770
GEMIN5	hsa-miR-7188-5p
GEMIN5	hsa-miR-4417
GEMIN5	hsa-miR-7320-3p
GEMIN5	hsa-miR-1668
GEMIN5	hsa-miR-194-5p
GEMIN5	hsa-miR-514b-5p
GEMIN5	hsa-miR-3552
GEMIN5	hsa-miR-2284v
GEMIN5	hsa-miR-548ar-3p
GEMIN5	hsa-miR-2454-3p
GEMIN5	hsa-miR-466
GEMIN5	hsa-miR-296-3p
GEMIN5	hsa-miR-378-5p
GEMIN5	hsa-miR-4701-3p
GEMIN5	hsa-miR-8864
GEMIN5	hsa-miR-3120-3p
GEMIN5	hsa-miR-8831
GEMIN5	hsa-miR-892a
GEMIN5	hsa-miR-1966-3p
GEMIN5	hsa-miR-421-5p
GEMIN5	hsa-miR-4275
GEMIN5	hsa-miR-548ah-3p
GEMIN5	hsa-miR-188-3p
GEMIN5	hsa-miR-9769-5p
GEMIN5	hsa-miR-6856-5p
GEMIN5	hsa-miR-6903-5p
GEMIN5	hsa-miR-4324
GEMIN5	hsa-miR-2464-3p
GEMIN5	hsa-miR-450a-2-3p
GEMIN5	hsa-miR-6826-5p
GEMIN5	hsa-miR-141-3p
GEMIN5	hsa-miR-519e-3p
GEMIN5	hsa-miR-6975-5p
GEMIN5	hsa-miR-30c-1-3p
GEMIN5	hsa-miR-329
GEMIN5	hsa-miR-603
GEMIN5	hsa-miR-4427
GEMIN5	hsa-miR-7067-5p
GEMIN5	hsa-miR-8093

GEMIN5	hsa-miR-630
GEMIN5	hsa-miR-7117-5p
GEMIN5	hsa-miR-5695
GEMIN5	hsa-miR-3132
GEMIN5	hsa-miR-2486-3p
GEMIN5	hsa-miR-7227-5p
GEMIN5	hsa-miR-2284k
GEMIN5	hsa-miR-1178-5p
GEMIN5	hsa-miR-7278-5p
GEMIN5	hsa-miR-548aj-3p
GEMIN5	hsa-miR-4768-3p
GEMIN5	hsa-miR-7332-3p
GEMIN5	hsa-miR-657
GEMIN5	hsa-miR-7280-3p
GEMIN5	hsa-miR-548b-3p
GEMIN5	hsa-miR-7288-5p
GEMIN5	hsa-miR-452-5p
GEMIN5	hsa-miR-134
GEMIN5	hsa-miR-627
GEMIN5	hsa-miR-4772-5p
GEMIN5	hsa-miR-7688-3p
GEMIN5	hsa-miR-513a-3p
GEMIN5	hsa-miR-606
GEMIN5	hsa-miR-6076
GEMIN5	hsa-miR-28-5p
GEMIN5	hsa-miR-6966-3p
GEMIN5	hsa-miR-2430
GEMIN5	hsa-miR-599
GEMIN5	hsa-miR-598-5p
GEMIN5	hsa-miR-7978
GEMIN5	hsa-miR-7047-3p
GEMIN5	hsa-miR-5584-5p
GEMIN5	hsa-miR-7183-3p
GEMIN5	hsa-miR-138-3p
GEMIN5	hsa-miR-2311
GEMIN5	hsa-miR-4257
GEMIN5	hsa-miR-23
GEMIN5	hsa-miR-1968-5p
GEMIN5	hsa-miR-7856-5p
GEMIN5	hsa-miR-454-5p
GEMIN5	hsa-miR-3584-5p
GEMIN5	hsa-miR-3152-3p
GEMIN5	hsa-miR-371a-5p
GEMIN5	hsa-miR-2284n
GEMIN5	hsa-miR-2136
GEMIN5	hsa-miR-344-5p

GEMIN5	hsa-miR-2424
GEMIN5	hsa-miR-692
GEMIN5	hsa-miR-3179
GEMIN5	hsa-miR-3192-3p
GEMIN5	hsa-miR-548g-3p
GEMIN5	hsa-miR-217
GEMIN5	hsa-miR-409-3p
GEMIN5	hsa-miR-693-3p
GEMIN5	hsa-miR-181a-1-3p
GEMIN5	hsa-miR-3200-5p
GEMIN5	hsa-miR-548z
GEMIN5	hsa-miR-130a-5p
GEMIN5	hsa-miR-642
GEMIN5	hsa-miR-548aq-3p
GEMIN5	hsa-miR-6898-5p
GEMIN5	hsa-miR-2315
GEMIN5	hsa-miR-8802
GEMIN5	hsa-miR-4511
GEMIN5	hsa-miR-224-3p
GEMIN5	hsa-miR-124-3p
GEMIN5	hsa-miR-107-3p
GEMIN5	hsa-miR-294-5p
GEMIN5	hsa-miR-30e
GEMIN5	hsa-miR-204-3p
GEMIN5	hsa-miR-23-3p
GEMIN5	hsa-miR-4762-3p
GEMIN5	hsa-miR-290a-5p
GEMIN5	hsa-miR-3092-5p
GEMIN5	hsa-miR-15b-3p
GEMIN5	hsa-miR-548az-3p
GEMIN5	hsa-miR-7275-3p
GEMIN5	hsa-miR-3678-3p
GEMIN5	hsa-miR-138-2-3p
GEMIN5	hsa-miR-7245-3p
GEMIN5	hsa-miR-3934-5p
GEMIN5	hsa-miR-671-5p
GEMIN5	hsa-miR-7046-3p
GEMIN5	hsa-miR-215
GEMIN5	hsa-miR-1827
GEMIN5	hsa-miR-7864
GEMIN5	hsa-miR-548t-3p
GEMIN5	hsa-miR-3121-3p
GEMIN5	hsa-miR-2415-5p
GEMIN5	hsa-miR-449-5p
GEMIN5	hsa-miR-7329-3p
GEMIN5	hsa-miR-5002-3p

GEMIN5	hsa-miR-548
GEMIN5	hsa-miR-940
GEMIN5	hsa-miR-1685-3p
GEMIN5	hsa-miR-372-5p
GEMIN5	hsa-miR-4680-3p
GEMIN5	hsa-miR-141-5p
GEMIN5	hsa-miR-199
GEMIN5	hsa-miR-7845-5p
GEMIN5	hsa-miR-7262a-5p
GEMIN5	hsa-miR-2429
GEMIN5	hsa-miR-8081
GEMIN5	hsa-miR-548e-3p
GEMIN5	hsa-miR-378a-5p
GEMIN5	hsa-miR-3580-5p
GEMIN5	hsa-miR-7342-3p
GEMIN5	hsa-miR-633
GEMIN5	hsa-miR-8485
GEMIN5	hsa-miR-586
GEMIN5	hsa-miR-2467-3p
GEMIN5	hsa-miR-509-3-5p
GEMIN5	hsa-miR-500-5p
GEMIN5	hsa-miR-2475
GEMIN5	hsa-miR-2284y
GEMIN5	hsa-miR-6854-3p
GEMIN5	hsa-miR-200c-3p
GEMIN5	hsa-miR-548aef
GEMIN5	hsa-miR-8823
GEMIN5	hsa-miR-106
GEMIN5	hsa-miR-4509
GEMIN5	hsa-miR-34b-3p
GEMIN5	hsa-miR-2392
GEMIN5	hsa-miR-466aep-5p
GEMIN5	hsa-miR-1910-3p
GEMIN5	hsa-miR-340-5p
GEMIN5	hsa-miR-2444
GEMIN5	hsa-miR-223-3p
GEMIN5	hsa-miR-379-5p
GEMIN5	hsa-miR-374-5p
GEMIN5	hsa-miR-142-5p
GEMIN5	hsa-miR-4753-5p
GEMIN5	hsa-miR-708-5p
GEMIN5	hsa-miR-7674-5p
GEMIN5	hsa-miR-148b-5p
GEMIN5	hsa-miR-548m
GEMIN5	hsa-miR-873-3p
GEMIN5	hsa-miR-4796-5p

GEMIN5	hsa-miR-3604
GEMIN5	hsa-miR-7515
GEMIN5	hsa-miR-548au-3p
GEMIN5	hsa-miR-8100
GEMIN5	hsa-miR-4732-5p
GEMIN5	hsa-miR-410
GEMIN5	hsa-miR-622
GEMIN5	hsa-miR-125-5p
GEMIN5	hsa-miR-653
GEMIN5	hsa-miR-759
GEMIN5	hsa-miR-197-5p
GEMIN5	hsa-miR-2285n
GEMIN5	hsa-miR-653-5p
GEMIN5	hsa-miR-383-3p
GEMIN5	hsa-miR-142-3p
GEMIN5	hsa-miR-216b-3p
GEMIN5	hsa-miR-196
GEMIN5	hsa-miR-8893
GEMIN5	hsa-miR-295-5p
GEMIN5	hsa-miR-6838-3p
GEMIN5	hsa-miR-5132-5p
GEMIN5	hsa-miR-7291-3p
GEMIN5	hsa-miR-544b
GEMIN5	hsa-miR-885
GEMIN5	hsa-miR-7398q-3p
GEMIN5	hsa-miR-7347-3p
GEMIN5	hsa-miR-548as-3p
GEMIN5	hsa-miR-324-3p
GEMIN5	hsa-miR-488-3p
GEMIN5	hsa-miR-627-5p
GEMIN5	hsa-miR-3572-5p
GEMIN5	hsa-miR-548b-3p
GEMIN5	hsa-miR-181b-1-3p
GEMIN5	hsa-miR-7293-5p
GEMIN5	hsa-miR-3662
GEMIN5	hsa-miR-6797-3p
GEMIN5	hsa-miR-7370-5p
GEMIN5	hsa-miR-7242-3p
GEMIN5	hsa-miR-1470
GEMIN5	hsa-miR-5192
GEMIN5	hsa-miR-497-3p
GEMIN5	hsa-miR-365-5p
GEMIN5	hsa-miR-103
GEMIN5	hsa-miR-610
GEMIN5	hsa-miR-3541
GEMIN5	hsa-miR-3167-5p

GEMIN5	hsa-miR-4318
GEMIN5	hsa-miR-548bb-3p
GEMIN5	hsa-miR-519d-5p
GEMIN5	hsa-miR-3613b
GEMIN5	hsa-miR-4272
GEMIN5	hsa-miR-182-5p
GEMIN5	hsa-miR-2284a
GEMIN5	hsa-miR-8095
GEMIN5	hsa-miR-6874-3p
GEMIN5	hsa-miR-6730-3p
GEMIN5	hsa-miR-370-3p
GEMIN5	hsa-miR-134-3p
GEMIN5	hsa-miR-524-5p
GEMIN5	hsa-miR-2351
GEMIN5	hsa-miR-548a-3p
GEMIN5	hsa-miR-3090-5p
GEMIN5	hsa-miR-338-3p
GEMIN5	hsa-miR-589-5p
GEMIN5	hsa-miR-7356-3p
GEMIN5	hsa-miR-212-3p
GEMIN5	hsa-miR-107
GEMIN5	hsa-miR-103-3p
GEMIN5	hsa-miR-1982-5p
GEMIN5	hsa-miR-548d-3p
GEMIN5	hsa-miR-182-5p
GEMIN5	hsa-miR-2284-5p
GEMIN5	hsa-miR-3671
GEMIN5	hsa-miR-1279
GEMIN5	hsa-miR-493-3p
GEMIN5	hsa-miR-672-3p
GEMIN5	hsa-miR-7154-5p
GEMIN5	hsa-miR-2367-3p
GEMIN5	hsa-miR-2414
GEMIN5	hsa-miR-501-5p
GEMIN5	hsa-miR-7218-5p
GEMIN5	hsa-miR-7089-5p
GEMIN5	hsa-miR-3575
GEMIN5	hsa-miR-1207-5p
GEMIN5	hsa-miR-548d-5p
GEMIN5	hsa-miR-547-5p
GEMIN5	hsa-miR-548h-3p
GEMIN5	hsa-miR-548aa
GEMIN5	hsa-miR-2358
GEMIN5	hsa-miR-429
GEMIN5	hsa-miR-22-5p
GEMIN5	hsa-miR-383-5p.1

GEMIN5	hsa-miR-187-3p
GEMIN5	hsa-miR-6747-3p
GEMIN5	hsa-miR-141-3p
GEMIN5	hsa-miR-500
GEMIN5	hsa-miR-6947-3p
GEMIN5	hsa-miR-7257-3p
GEMIN5	hsa-miR-7078-5p
GEMIN5	hsa-miR-698-5p
GEMIN5	hsa-miR-883-5p
GEMIN5	hsa-miR-1786
GEMIN5	hsa-miR-676-3p
GEMIN5	hsa-miR-344eh-5p
GEMIN5	hsa-miR-31-5p
GEMIN5	hsa-miR-1255-5p
GEMIN5	hsa-miR-6982-5p
GEMIN5	hsa-miR-181-5p
GEMIN5	hsa-miR-510-3p
GEMIN5	hsa-miR-6998-5p
GEMIN5	hsa-miR-6806-5p
GEMIN5	hsa-miR-8901
GEMIN5	hsa-miR-7684-5p
GEMIN5	hsa-miR-371b-5p
GEMIN5	hsa-miR-2284g
GEMIN5	hsa-miR-195-3p
GEMIN5	hsa-miR-3168
GEMIN5	hsa-miR-7018-3p
GEMIN5	hsa-miR-93-5p
GEMIN5	hsa-miR-7676-5p
GEMIN5	hsa-miR-511-3p
GEMIN5	hsa-miR-679-3p
GEMIN5	hsa-miR-219a-5p
GEMIN5	hsa-miR-2285af
GEMIN5	hsa-miR-4538
GEMIN5	hsa-miR-2284d
GEMIN5	hsa-miR-4453
GEMIN5	hsa-miR-937-5p
GEMIN5	hsa-miR-5004-3p
GEMIN5	hsa-miR-2370-5p
GEMIN5	hsa-miR-4667-3p
GEMIN5	hsa-miR-466f-3p
GEMIN5	hsa-miR-495-3p
GEMIN5	hsa-miR-548j-3p
GEMIN5	hsa-miR-3967
GEMIN5	hsa-miR-7859
GEMIN5	hsa-miR-702-5p
GEMIN5	hsa-miR-192-5p

GEMIN5	hsa-miR-548cej-3p
GEMIN5	hsa-miR-6511a-5p
GEMIN5	hsa-miR-9-3p
GEMIN5	hsa-miR-6881-3p
GEMIN5	hsa-miR-2398
GEMIN5	hsa-miR-4439
GEMIN5	hsa-miR-2361
GEMIN5	hsa-miR-548am-3p
GEMIN5	hsa-miR-7168-3p
GEMIN5	hsa-miR-2284aa
GEMIN5	hsa-miR-7080-5p
GEMIN5	hsa-miR-7087-5p
GEMIN5	hsa-miR-1198-5p
IFIT5	hsa-miR-3200-5p
IFIT5	hsa-miR-548c-5p
IFIT5	hsa-miR-4689
IFIT5	hsa-miR-7172-5p
IFIT5	hsa-miR-7166-3p
IFIT5	hsa-miR-556-5p
IFIT5	hsa-miR-3606-3p
IFIT5	hsa-miR-7849-3p
IFIT5	hsa-miR-8873a
IFIT5	hsa-miR-2054
IFIT5	hsa-miR-1224-5p
IFIT5	hsa-miR-361-5p
IFIT5	hsa-miR-26c
IFIT5	hsa-miR-373-3p
IFIT5	hsa-miR-518-5p
IFIT5	hsa-miR-6878-5p
IFIT5	hsa-miR-374-5p
IFIT5	hsa-miR-429
IFIT5	hsa-miR-493-3p
IFIT5	hsa-miR-4762-3p
IFIT5	hsa-miR-302a-3p
IFIT5	hsa-miR-3911
IFIT5	hsa-miR-4695-5p
IFIT5	hsa-miR-4682
IFIT5	hsa-miR-623
IFIT5	hsa-miR-548j-3p
IFIT5	hsa-miR-7298-5p
IFIT5	hsa-miR-548aef
IFIT5	hsa-miR-4732-3p
IFIT5	hsa-miR-376ab-3p
IFIT5	hsa-miR-8063
IFIT5	hsa-miR-4786-3p
IFIT5	hsa-miR-576-5p

IFIT5	hsa-miR-892a
IFIT5	hsa-miR-2439-3p
IFIT5	hsa-miR-4668-5p
IFIT5	hsa-miR-548ae-3p
IFIT5	hsa-miR-7856-5p
IFIT5	hsa-miR-7207-5p
IFIT5	hsa-miR-8807
IFIT5	hsa-miR-23-3p
IFIT5	hsa-miR-548ak
IFIT5	hsa-miR-8818
IFIT5	hsa-miR-223-5p
IFIT5	hsa-miR-6801-3p
IFIT5	hsa-miR-2398
IFIT5	hsa-miR-1277-3p
IFIT5	hsa-miR-203b-3p
IFIT5	hsa-miR-513c-3p
IFIT5	hsa-miR-363-3p
IFIT5	hsa-miR-548c-3p
IFIT5	hsa-miR-1288-3p
IFIT5	hsa-miR-7347-3p
IFIT5	hsa-miR-503-3p
IFIT5	hsa-miR-7286-3p
IFIT5	hsa-miR-1434-3p
IFIT5	hsa-miR-340-3p
IFIT5	hsa-miR-137-3p
IFIT5	hsa-miR-6864-3p
IFIT5	hsa-miR-499
IFIT5	hsa-miR-7244-5p
IFIT5	hsa-miR-7864
IFIT5	hsa-miR-5196-3p
IFIT5	hsa-miR-548n
IFIT5	hsa-miR-2394
IFIT5	hsa-miR-7386b-3p
IFIT5	hsa-miR-548h-3p
IFIT5	hsa-miR-876-5p
IFIT5	hsa-miR-551-5p
IFIT5	hsa-miR-590-3p
IFIT5	hsa-miR-340-5p
IFIT5	hsa-miR-6733-5p
IFIT5	hsa-miR-200bc
IFIT5	hsa-miR-8908e
IFIT5	hsa-miR-4778-3p
IFIT5	hsa-miR-2387
IFIT5	hsa-miR-3609
IFIT5	hsa-miR-150-5p
IFIT5	hsa-miR-7-5p

IFIT5	hsa-miR-345-5p
IFIT5	hsa-miR-300
IFIT5	hsa-miR-136-5p
IFIT5	hsa-miR-2284aa
IFIT5	hsa-miR-5582-3p
IFIT5	hsa-miR-8904b
IFIT5	hsa-miR-4791
IFIT5	hsa-miR-7159-3p
IFIT5	hsa-miR-3165
IFIT5	hsa-miR-1243
IFIT5	hsa-miR-371a-5p
IFIT5	hsa-miR-6790-5p
IFIT5	hsa-miR-3613b
IFIT5	hsa-miR-4731-3p
IFIT5	hsa-miR-5700
IFIT5	hsa-miR-124-5p
IFIT5	hsa-miR-302
IFIT5	hsa-miR-6858-5p
IFIT5	hsa-miR-616-5p
IFIT5	hsa-miR-6891-3p
IFIT5	hsa-miR-1250-3p
IFIT5	hsa-miR-2284n
IFIT5	hsa-miR-1277-3p
IFIT5	hsa-miR-628-5p
IFIT5	hsa-miR-31-5p
IFIT5	hsa-miR-548a-5p
IFIT5	hsa-miR-3684
IFIT5	hsa-miR-33-5p
IFIT5	hsa-miR-6876-3p
IFIT5	hsa-miR-5694
IFIT5	hsa-miR-338-5p
IFIT5	hsa-miR-3138
IFIT5	hsa-miR-410
IFIT5	hsa-miR-4269
IFIT5	hsa-miR-6788-5p
IFIT5	hsa-miR-8061
IFIT5	hsa-miR-204-5p
IFIT5	hsa-miR-1260a
IFIT5	hsa-miR-670-5p
IFIT5	hsa-miR-548ay-3p
IFIT5	hsa-miR-125a-3p
IFIT5	hsa-miR-1236-3p
IFIT5	hsa-miR-619
IFIT5	hsa-miR-154-5p
IFIT5	hsa-miR-2893
IFIT5	hsa-miR-101-3p

IFIT5	hsa-miR-548bd-3p
IFIT5	hsa-miR-3938
IFIT5	hsa-miR-3201
IFIT5	hsa-miR-4266
IFIT5	hsa-miR-335-3p
IFIT5	hsa-miR-8824
IFIT5	hsa-miR-4482-3p
IFIT5	hsa-miR-17-5p
IFIT5	hsa-miR-371b-5p
IFIT5	hsa-miR-548i
IFIT5	hsa-miR-95
IFIT5	hsa-miR-2332
IFIT5	hsa-miR-183-5p
IFIT5	hsa-miR-5584-3p
IFIT5	hsa-miR-3200-3p
IFIT5	hsa-miR-7385e-3p
IFIT5	hsa-miR-548x-3p
IFIT5	hsa-miR-872
IFIT5	hsa-miR-4259
IFIT5	hsa-miR-7398d-3p
IFIT5	hsa-miR-18
IFIT5	hsa-miR-551b-5p
IFIT5	hsa-miR-7245-3p
IFIT5	hsa-miR-2284-5p
IFIT5	hsa-miR-6779-3p
IFIT5	hsa-miR-548o-5p
IFIT5	hsa-miR-519-3p
IFIT5	hsa-miR-106
IFIT5	hsa-miR-8908-3p
IFIT5	hsa-miR-548av-5p
IFIT5	hsa-miR-17-3p
IFIT5	hsa-miR-26-5p
IFIT5	hsa-miR-7-5p
IFIT5	hsa-miR-1262-5p
IFIT5	hsa-miR-6804-5p
IFIT5	hsa-miR-2285u
IFIT5	hsa-miR-7270-5p
IFIT5	hsa-miR-3934-5p
IFIT5	hsa-miR-376a-2-5p
IFIT5	hsa-miR-708-5p
IFIT5	hsa-miR-1323-5p
IFIT5	hsa-miR-664
IFIT5	hsa-miR-6512-5p
IFIT5	hsa-miR-548ab
IFIT5	hsa-miR-2406
IFIT5	hsa-miR-10b-5p

IFIT5	hsa-miR-7334-5p
IFIT5	hsa-miR-2298
IFIT5	hsa-miR-501-5p
IFIT5	hsa-miR-603
IFIT5	hsa-miR-511
IFIT5	hsa-miR-4519
IFIT5	hsa-miR-508a
IFIT5	hsa-miR-889-5p
IFIT5	hsa-miR-548k
IFIT5	hsa-miR-2373-5p
IFIT5	hsa-miR-1252
IFIT5	hsa-miR-548t-5p
IFIT5	hsa-miR-93-5p
IFIT5	hsa-miR-195-5p
IFIT5	hsa-miR-4491
IFIT5	hsa-miR-627-3p
IFIT5	hsa-miR-4740-5p
IFIT5	hsa-miR-136-5p
IFIT5	hsa-miR-2372
IFIT5	hsa-miR-548ah-5p
IFIT5	hsa-miR-888-5p
IFIT5	hsa-miR-323a-3p
IFIT5	hsa-miR-4684-5p
IFIT5	hsa-miR-2284u
IFIT5	hsa-miR-4804-3p
IFIT5	hsa-miR-500a-5p
IFIT5	hsa-miR-125b-2-3p
IFIT5	hsa-miR-6834-5p
IFIT5	hsa-miR-4662-5p
IFIT5	hsa-miR-203
IFIT5	hsa-miR-497-5p
IFIT5	hsa-miR-2285x
IFIT5	hsa-miR-330-3p.2
IFIT5	hsa-miR-8065
IFIT5	hsa-miR-4677-5p
IFIT5	hsa-miR-7398r-5p
IFIT5	hsa-miR-1323
IFIT5	hsa-miR-495-3p
IFIT5	hsa-miR-2285
IFIT5	hsa-miR-4775
IFIT5	hsa-miR-4715-5p
IFIT5	hsa-miR-3938-3p
IFIT5	hsa-miR-3604
IFIT5	hsa-miR-502-5p
IFIT5	hsa-miR-1343-3p
IFIT5	hsa-miR-3682-3p

IFIT5	hsa-miR-3616-5p
IFIT5	hsa-miR-33-3p
IFIT5	hsa-miR-3924
IFIT5	hsa-miR-7386g-3p
IFIT5	hsa-miR-1243-3p
IFIT5	hsa-miR-1273h-3p
IFIT5	hsa-miR-331-3p
IFIT5	hsa-miR-548a-3p
IFIT5	hsa-miR-129-2-3p
IFIT5	hsa-miR-216-5p
IFIT5	hsa-miR-188-3p
IFIT5	hsa-miR-372
IFIT5	hsa-miR-7166-5p
IFIT5	hsa-miR-610
IFIT5	hsa-miR-7398s-3p
IFIT5	hsa-miR-4691-3p
IFIT5	hsa-miR-629-3p
IFIT5	hsa-miR-3074-5p
IFIT5	hsa-miR-145-5p
IFIT5	hsa-miR-802
IFIT5	hsa-miR-4276
IFIT5	hsa-miR-369-3p
IFIT5	hsa-miR-567
IFIT5	hsa-miR-2482
IFIT5	hsa-miR-7262b-5p
IFIT5	hsa-miR-2421
IFIT5	hsa-miR-1237-3p
IFIT5	hsa-miR-580
IFIT5	hsa-miR-4468
IFIT5	hsa-miR-7373d-3p
IFIT5	hsa-miR-488-3p
IFIT5	hsa-miR-195-3p
IFIT5	hsa-miR-5006-3p
IFIT5	hsa-miR-7297-3p
IFIT5	hsa-miR-373-5p
IFIT5	hsa-miR-3129-5p
IFIT5	hsa-miR-2291
IFIT5	hsa-miR-126-5p
IFIT5	hsa-miR-3116
IFIT5	hsa-miR-6791-5p
IFIT5	hsa-miR-7192-3p
IFIT5	hsa-miR-891a-3p
IFIT5	hsa-miR-633
IFIT5	hsa-miR-323-3p
IFIT5	hsa-miR-548y
IFIT5	hsa-miR-370-5p

IFIT5	hsa-miR-448
IFIT5	hsa-miR-548e-3p
IFIT5	hsa-miR-323b-3p
IFIT5	hsa-miR-3533
IFIT5	hsa-miR-4724-3p
IFIT5	hsa-miR-105-5p
IFIT5	hsa-miR-4712-3p
IFIT5	hsa-miR-5692a
IFIT5	hsa-miR-200-3p
IFIT5	hsa-miR-583
IFIT5	hsa-miR-141-3p
IFIT5	hsa-miR-2293
IFIT5	hsa-miR-4768-3p
IFIT5	hsa-miR-204-5p
IFIT5	hsa-miR-4418
IFIT5	hsa-miR-1237-3p
IFIT5	hsa-miR-548az-5p
IFIT5	hsa-miR-3065-5p
IFIT5	hsa-miR-548ar-5p
IFIT5	hsa-miR-8900
IFIT5	hsa-miR-513a-3p
IFIT5	hsa-miR-3915
IFIT5	hsa-miR-96-5p
IFIT5	hsa-miR-1236-3p
IFIT5	hsa-miR-5583-5p
IFIT5	hsa-miR-589-5p
IFIT5	hsa-miR-196-3p
IFIT5	hsa-miR-449a-3p
IFIT5	hsa-miR-8808
IFIT5	hsa-miR-222-3p
IFIT5	hsa-miR-8843
IFIT5	hsa-miR-16-5p
IFIT5	hsa-miR-7338-3p
IFIT5	hsa-miR-2284k
IFIT5	hsa-miR-7844-5p
IFIT5	hsa-miR-944
IFIT5	hsa-miR-503-5p
IFIT5	hsa-miR-3159
IFIT5	hsa-miR-22-5p
IFIT5	hsa-miR-4311
IFIT5	hsa-miR-8870
IFIT5	hsa-miR-4529-5p
IFIT5	hsa-miR-875-3p
IFIT5	hsa-miR-494
IFIT5	hsa-miR-5009-3p
IFIT5	hsa-miR-1233-3p

IFIT5	hsa-miR-934-5p
IFIT5	hsa-miR-424-5p
IFIT5	hsa-miR-374a-5p
IFIT5	hsa-miR-6758-3p
IFIT5	hsa-miR-216
IFIT5	hsa-miR-5096
IFIT5	hsa-miR-647
IFIT5	hsa-miR-8084
IFIT5	hsa-miR-3130-3p
IFIT5	hsa-miR-422
IFIT5	hsa-miR-548ad-5p
IFIT5	hsa-miR-8083
IFIT5	hsa-miR-4766-5p
IFIT5	hsa-miR-5579-5p
IFIT5	hsa-miR-587
IFIT5	hsa-miR-885-5p
IFIT5	hsa-miR-510-3p
IFIT5	hsa-miR-4717-3p
IFIT5	hsa-miR-4517
IFIT5	hsa-miR-653
IFIT5	hsa-miR-5580-3p
IFIT5	hsa-miR-548w
IFIT5	hsa-miR-7370i-3p
IFIT5	hsa-miR-496.1
IFIT5	hsa-miR-345-3p
IFIT5	hsa-miR-552-5p
IFIT5	hsa-miR-7371e-3p
IFIT5	hsa-miR-934
IFIT5	hsa-miR-3658
IFIT5	hsa-miR-7152-5p
IFIT5	hsa-miR-8908b
IFIT5	hsa-miR-323
IFIT5	hsa-miR-7193-5p
IFIT5	hsa-miR-2418
IFIT5	hsa-miR-8884
IFIT5	hsa-miR-1304-5p
IFIT5	hsa-miR-3187-3p
IFIT5	hsa-miR-7374a-3p
IFIT5	hsa-miR-496
IFIT5	hsa-miR-548g-5p
IFIT5	hsa-miR-2285s
IFIT5	hsa-miR-1200
IFIT5	hsa-miR-2357
IFIT5	hsa-miR-6079
IFIT5	hsa-miR-7168-5p
IFIT5	hsa-miR-5011-5p

IFIT5	hsa-miR-6856-3p
IFIT5	hsa-miR-211-5p
IFIT5	hsa-miR-2284j
IFIT5	hsa-miR-2284d
IFIT5	hsa-miR-3667-3p
IFIT5	hsa-miR-4711-3p
IFIT5	hsa-miR-888-5p
IFIT5	hsa-miR-433
IFIT5	hsa-miR-3182
IFIT5	hsa-miR-557
IFIT5	hsa-miR-7398t-3p
IFIT5	hsa-miR-6894-5p
IFIT5	hsa-miR-6772-5p
IFIT5	hsa-miR-507
IFIT5	hsa-miR-3120-5p
IFIT5	hsa-miR-8895
IFIT5	hsa-miR-8865
IFIT5	hsa-miR-3153
IFIT5	hsa-miR-7194-5p
IFIT5	hsa-miR-421
IFIT5	hsa-miR-23
IFIT5	hsa-miR-4646-5p
IFIT5	hsa-miR-29a-5p
IFIT5	hsa-miR-125
IFIT5	hsa-miR-138-3p
IFIT5	hsa-miR-200b-3p
IFIT5	hsa-miR-6529-5p
IFIT5	hsa-miR-548z
IFIT5	hsa-miR-2284a
IFIT5	hsa-miR-382-3p
IFIT5	hsa-miR-2284c
IFIT5	hsa-miR-9-3p
IFIT5	hsa-miR-670
IFIT5	hsa-miR-30-5p
IFIT5	hsa-miR-429-3p
IFIT5	hsa-miR-764
IFIT5	hsa-miR-3923
IFIT5	hsa-miR-1285-5p
IFIT5	hsa-miR-30c-2-3p
IFIT5	hsa-miR-369-3p
IFIT5	hsa-miR-2352
IFIT5	hsa-miR-7333-5p
IFIT5	hsa-miR-2284z
IFIT5	hsa-miR-1303
IFIT5	hsa-miR-374
IFIT5	hsa-miR-876

IFIT5	hsa-miR-4515
IFIT5	hsa-miR-486-5p
IFIT5	hsa-miR-150-3p
IFIT5	hsa-miR-433-3p
IFIT5	hsa-miR-132-3p
IFIT5	hsa-miR-2284g
IFIT5	hsa-miR-7154-3p
IFIT5	hsa-miR-4528
IFIT5	hsa-miR-299-3p
IFIT5	hsa-miR-6852-5p
IFIT5	hsa-miR-4692
IFIT5	hsa-miR-3680-3p
IFIT5	hsa-miR-6739-5p
IFIT5	hsa-miR-183-3p
IFIT5	hsa-miR-3672
IFIT5	hsa-miR-1287-3p
IFIT5	hsa-miR-2369
IFIT5	hsa-miR-7386j-3p
IFIT5	hsa-miR-2284b
IFIT5	hsa-miR-30-3p
IFIT5	hsa-miR-15a-3p
IFIT5	hsa-miR-3145-3p
IFIT5	hsa-miR-4687-5p
IFIT5	hsa-miR-5703
IFIT5	hsa-miR-7312-5p
IFIT5	hsa-miR-373-3p
IFIT5	hsa-miR-224-5p
IFIT5	hsa-miR-3150a-3p
IFIT5	hsa-miR-4803
IFIT5	hsa-miR-331-3p
IFIT5	hsa-miR-3179
IFIT5	hsa-miR-186-5p
IFIT5	hsa-miR-7257-3p
IFIT5	hsa-miR-1912
IFIT5	hsa-let-7-5p
IFIT5	hsa-miR-4764-3p
IFIT5	hsa-miR-105b
IFIT5	hsa-miR-375
IFIT5	hsa-miR-664a-3p
IFIT5	hsa-miR-548am-3p
IFIT5	hsa-miR-302-3p
IFIT5	hsa-miR-1322
IFIT5	hsa-miR-383-5p.2
IFIT5	hsa-miR-559
IFIT5	hsa-miR-655
IFIT5	hsa-miR-7394b-5p

IFIT5	hsa-miR-4742-5p
IFIT5	hsa-miR-509-3-5p
IFIT5	hsa-miR-631
IFIT5	hsa-miR-3200-3p
IFIT5	hsa-miR-154a
IFIT5	hsa-miR-30c-3p
IFIT5	hsa-miR-539-5p
IFIT5	hsa-miR-146a-3p
IFIT5	hsa-miR-6830-5p
IFIT5	hsa-miR-548ar-3p
IFIT5	hsa-miR-6072
IFIT5	hsa-miR-8838
IFIT5	hsa-miR-526
IFIT5	hsa-miR-148
IFIT5	hsa-miR-1-5p
IFIT5	hsa-miR-10a-3p
IFIT5	hsa-miR-3925-5p
IFIT5	hsa-miR-19-5p
IFIT5	hsa-miR-4760-5p
IFIT5	hsa-miR-28-5p
IFIT5	hsa-miR-2466-5p
IFIT5	hsa-miR-2452
IFIT5	hsa-miR-502-5p
IFIT5	hsa-miR-7371b-3p
IFIT5	hsa-miR-548aj-3p
IFIT5	hsa-miR-215
IFIT5	hsa-miR-2404
IFIT5	hsa-miR-582-5p
IFIT5	hsa-miR-135-5p
IFIT5	hsa-miR-4533
IFIT5	hsa-miR-6074
IFIT5	hsa-miR-548e-5p
IFIT5	hsa-miR-6809-5p
IFIT5	hsa-miR-142-3p
IFIT5	hsa-miR-7199-5p
IFIT5	hsa-miR-7173-3p
IFIT5	hsa-miR-506-5p
IFIT5	hsa-miR-6128
IFIT5	hsa-miR-4434
IFIT5	hsa-miR-7386e-3p
IFIT5	hsa-miR-7264-3p
IFIT5	hsa-miR-21-3p
IFIT5	hsa-miR-222-5p
IFIT5	hsa-miR-548az-3p
IFIT5	hsa-miR-3662
IFIT5	hsa-miR-20

IFIT5	hsa-miR-7281-3p
IFIT5	hsa-miR-153
IFIT5	hsa-miR-10b-3p
IFIT5	hsa-miR-367-3p
IFIT5	hsa-miR-6509-5p
IFIT5	hsa-miR-3163
IFIT5	hsa-miR-513c-5p
IFIT5	hsa-miR-758-3p
IFIT5	hsa-miR-892-5p
IFIT5	hsa-miR-8852
IFIT5	hsa-miR-548bb-3p
IFIT5	hsa-miR-6509-3p
IFIT5	hsa-miR-4286
IFIT5	hsa-miR-1246
IFIT5	hsa-miR-6833-5p
IFIT5	hsa-miR-7324-3p
IFIT5	hsa-miR-7109-3p
IFIT5	hsa-miR-548j-5p
IFIT5	hsa-miR-7387-3p
IFIT5	hsa-miR-939-3p
IFIT5	hsa-miR-9a-3p
IFIT5	hsa-miR-4678
IFIT5	hsa-miR-3140-3p
IFIT5	hsa-miR-577
IFIT5	hsa-miR-548h-5p
IFIT5	hsa-miR-624-3p
IFIT5	hsa-miR-6742-3p
IFIT5	hsa-miR-6077
IFIT5	hsa-miR-625-3p
IFIT5	hsa-miR-4999-5p
IFIT5	hsa-miR-186-3p
IFIT5	hsa-miR-6734-5p
IFIT5	hsa-miR-4273
IFIT5	hsa-miR-7371d-5p
IFIT5	hsa-miR-34
IFIT5	hsa-miR-139-5p
IFIT5	hsa-miR-877-3p
IFIT5	hsa-miR-143-3p
IFIT5	hsa-miR-7195-3p
IFIT5	hsa-miR-8066
IFIT5	hsa-miR-4693-5p
IFIT5	hsa-miR-4761-5p
IFIT5	hsa-miR-4443
IFIT5	hsa-miR-7199-3p
IFIT5	hsa-miR-6506-5p
IFIT5	hsa-miR-2110

IFIT5	hsa-miR-586
IFIT5	hsa-miR-548aq-5p
IFIT5	hsa-miR-5682
IFIT5	hsa-miR-4536-5p
IFIT5	hsa-miR-298
IFIT5	hsa-miR-3180-3p
IFIT5	hsa-miR-204-3p
IFIT5	hsa-miR-7398r-3p
IFIT5	hsa-miR-105-5p
IFIT5	hsa-miR-148-3p
IFIT5	hsa-miR-197-3p
IFIT5	hsa-miR-129-5p
IFIT5	hsa-miR-8881
IFIT5	hsa-miR-532-3p
IFIT5	hsa-miR-378-3p
IFIT5	hsa-miR-1226-5p
IFIT5	hsa-miR-5197-5p
IFIT5	hsa-miR-6730-5p
IFIT5	hsa-miR-4650
IFIT5	hsa-miR-8054
IFIT5	hsa-miR-4699-3p
IFIT5	hsa-miR-6715a-3p
IFIT5	hsa-miR-192-3p
IFIT5	hsa-miR-372-3p
IFIT5	hsa-miR-380
IFIT5	hsa-miR-573
IFIT5	hsa-miR-4455
IFIT5	hsa-miR-5000-3p
IFIT5	hsa-miR-3675-5p
IFIT5	hsa-miR-200c-3p
IFIT5	hsa-miR-3660
IFIT5	hsa-miR-425-5p
IFIT5	hsa-miR-7349-3p
IFIT5	hsa-miR-2116-5p
IFIT5	hsa-miR-302c-3p.2
IFIT5	hsa-miR-27-3p
IFIT5	hsa-miR-95-5p
IFIT5	hsa-miR-376c-3p
IFIT5	hsa-miR-5697-3p
IFIT5	hsa-miR-654-3p
IFIT5	hsa-miR-3928-3p
IFIT5	hsa-miR-543
IFIT5	hsa-miR-3912-5p
IFIT5	hsa-miR-1193
IFIT5	hsa-miR-374a-3p
IFIT5	hsa-miR-4670-3p

IFIT5	hsa-miR-892b
IFIT5	hsa-miR-589-3p
IFIT5	hsa-miR-217
IFIT5	hsa-miR-520-3p
IFIT5	hsa-miR-6757-3p
IFIT5	hsa-miR-221-3p
IFIT5	hsa-miR-1276
IFIT5	hsa-miR-153-5p
IFIT5	hsa-miR-342-5p
IFIT5	hsa-miR-8085
IFIT5	hsa-miR-7247-5p
IFIT5	hsa-miR-3156-5p
IFIT5	hsa-miR-548ah-3p
IFIT5	hsa-miR-4317
IFIT5	hsa-miR-3646
IFIT5	hsa-miR-214-3p
IFIT5	hsa-miR-32-3p
IFIT5	hsa-miR-7289-5p
IFIT5	hsa-miR-4260
IFIT5	hsa-miR-224-5p
IFIT5	hsa-miR-4795-3p
IFIT5	hsa-miR-6515-5p
IFIT5	hsa-miR-4282
IFIT5	hsa-miR-548d-3p
IFIT5	hsa-miR-142-5p
IFIT5	hsa-miR-2284y
IFIT5	hsa-miR-8874
IFIT5	hsa-miR-29a-3p
IFIT5	hsa-miR-374b-3p
IFIT5	hsa-miR-3115
IFIT5	hsa-miR-1972
IFIT5	hsa-miR-7177-5p
IFIT5	hsa-miR-4753-5p
IFIT5	hsa-miR-7398m-3p
IFIT5	hsa-miR-376c-5p
IFIT5	hsa-miR-590-5p
IFIT5	hsa-miR-509-5p
IFIT5	hsa-miR-7273-5p
IFIT5	hsa-miR-520g-3p
IFIT5	hsa-miR-7201-5p
IFIT5	hsa-miR-765
IFIT5	hsa-miR-8823
IFIT5	hsa-miR-1295b-5p
IFIT5	hsa-miR-2286
IFIT5	hsa-miR-410-3p
IFIT5	hsa-miR-4713-5p

IFIT5	hsa-miR-299-5p
IFIT5	hsa-miR-6516
IFIT5	hsa-miR-2429
IFIT5	hsa-miR-6082
IFIT5	hsa-miR-4318
IFIT5	hsa-miR-6846-3p
IFIT5	hsa-miR-450b
IFIT5	hsa-miR-548ap-5p
IFIT5	hsa-miR-449c-3p
IFIT5	hsa-miR-3607-3p
IFIT5	hsa-miR-1289
IFIT5	hsa-miR-548d-5p
IFIT5	hsa-miR-7398g-3p
IFIT5	hsa-miR-6083
IFIT5	hsa-miR-2483-3p
IFIT5	hsa-miR-2284l
IFIT5	hsa-miR-216b
IFIT5	hsa-miR-4729
IFIT5	hsa-miR-3680-5p
IFIT5	hsa-miR-137
IFIT5	hsa-miR-27a-5p
IFIT5	hsa-miR-4480
IFIT5	hsa-miR-107-3p
IFIT5	hsa-miR-511-5p
IFIT5	hsa-miR-4801
IFIT5	hsa-miR-34c-3p
IFIT5	hsa-miR-7170-5p
IFIT5	hsa-miR-8902
IFIT5	hsa-miR-7371j-5p
IFIT5	hsa-miR-27b-5p
IFIT5	hsa-miR-599-3p
IFIT5	hsa-miR-6778-5p
IFIT5	hsa-miR-144-3p
IFIT5	hsa-miR-7156-5p
IFIT5	hsa-miR-612
IFIT5	hsa-miR-4670-5p
IFIT5	hsa-miR-7178-3p
IFIT5	hsa-miR-2447
IFIT5	hsa-miR-4531
IFIT5	hsa-miR-2446
IFIT5	hsa-miR-1266
IFIT5	hsa-miR-604
IFIT5	hsa-miR-548at-3p
IFIT5	hsa-miR-548ay-5p
IFIT5	hsa-miR-365-5p
IFIT5	hsa-miR-7351-3p

IFIT5	hsa-miR-450a-2-3p
IFIT5	hsa-miR-2116-3p
IFIT5	hsa-miR-488-3p
IFIT5	hsa-miR-1537-5p
IFIT5	hsa-miR-8821
IFIT5	hsa-miR-580-3p
IFIT5	hsa-miR-4279
IFIT5	hsa-miR-130a-5p
IFIT5	hsa-miR-3129-3p
IFIT5	hsa-miR-2320-5p
IFIT5	hsa-miR-483-3p.1
IFIT5	hsa-miR-3690
IFIT5	hsa-miR-154-5p
IFIT5	hsa-miR-3169
IFIT5	hsa-miR-93-5p
IFIT5	hsa-miR-3692-3p
IFIT5	hsa-miR-3167-3p
IFIT5	hsa-miR-297
IFIT5	hsa-miR-340-5p
IFIT5	hsa-miR-152
IFIT5	hsa-miR-3678-3p
IFIT5	hsa-miR-455-3p
IFIT5	hsa-miR-548aq-3p
IFIT5	hsa-miR-3167-5p
IFIT5	hsa-miR-4272
IFIT5	hsa-miR-2284v
IFIT5	hsa-miR-4292
IFIT5	hsa-miR-7360-5p
IFIT5	hsa-miR-216a-5p
IFIT5	hsa-miR-4650-5p
IFIT5	hsa-miR-16-3p
IFIT5	hsa-miR-491-5p
IFIT5	hsa-miR-626
IFIT5	hsa-miR-548bb-5p
IFIT5	hsa-miR-450b-5p
IFIT5	hsa-miR-125-5p
IFIT5	hsa-miR-299
IFIT5	hsa-miR-6739-3p
IFIT5	hsa-miR-4716-5p
IFIT5	hsa-miR-761
IFIT5	hsa-miR-4536-5p
IFIT5	hsa-miR-152-3p
IFIT5	hsa-miR-4475
IFIT5	hsa-miR-16-2-3p
IFIT5	hsa-miR-7386l-3p
IFIT5	hsa-let-7-3p

IFIT5	hsa-miR-1299
IFIT5	hsa-miR-7385d-3p
IFIT5	hsa-miR-570-3p
IFIT5	hsa-miR-561-3p
IFIT5	hsa-miR-1842
IFIT5	hsa-miR-7293-5p
IFIT5	hsa-miR-376ab
IFIT5	hsa-miR-1277-5p
IFIT5	hsa-miR-24-3p
IFIT5	hsa-miR-503-5p
IFIT5	hsa-miR-2471-5p
IFIT5	hsa-miR-376a-5p
IFIT5	hsa-miR-2485
IFIT5	hsa-miR-548ac
IFIT5	hsa-miR-2278
IFIT5	hsa-miR-520
IFIT5	hsa-miR-7515
IFIT5	hsa-miR-380-3p
IFIT5	hsa-miR-548am-5p
IFIT5	hsa-miR-5681a
IFIT5	hsa-miR-301-5p
IFIT5	hsa-miR-532-5p
IFIT5	hsa-miR-6813-3p
IFIT5	hsa-miR-1266-5p
IFIT5	hsa-miR-6800-5p
IFIT5	hsa-miR-711
IFIT5	hsa-miR-6810-3p
IFIT5	hsa-miR-150-5p
IFIT5	hsa-miR-4760-3p
IFIT5	hsa-miR-661
IFIT5	hsa-miR-378a-5p
IFIT5	hsa-miR-302f
IFIT5	hsa-miR-2311
IFIT5	hsa-miR-103-3p
IFIT5	hsa-miR-5692bc
IFIT5	hsa-miR-101-3p.2
IFIT5	hsa-miR-2114-5p
IFIT5	hsa-miR-4446-5p
IFIT5	hsa-miR-376b-5p
IFIT5	hsa-miR-4789-3p
IFIT5	hsa-miR-29a-3p
IFIT5	hsa-miR-374b-5p
IFIT5	hsa-miR-539-5p
IFIT5	hsa-miR-4720-3p
IFIT5	hsa-miR-4755-5p
IFIT5	hsa-miR-766-5p

IFIT5	hsa-miR-190a-3p
IFIT5	hsa-miR-4756-3p
IFIT5	hsa-miR-15-5p
IFIT5	hsa-miR-7398s-5p
IFIT5	hsa-miR-548as-5p
IFIT5	hsa-miR-1185-5p
IFIT5	hsa-miR-6872-3p
IFIT5	hsa-miR-200c-3p
IFIT5	hsa-miR-5584-5p
IFIT5	hsa-miR-302bd
IFIT5	hsa-miR-526b-5p
IFIT5	hsa-miR-5088-3p
IFIT5	hsa-miR-520gh
IFIT5	hsa-miR-145-3p
IFIT5	hsa-miR-512-3p
IFIT5	hsa-miR-4518
IFIT5	hsa-miR-6864-5p
IFIT5	hsa-miR-326
IFIT5	hsa-miR-7206-5p
IFIT5	hsa-miR-8069
IFIT5	hsa-miR-133
IFIT5	hsa-miR-7246-3p
IFIT5	hsa-miR-8802
IFIT5	hsa-miR-378
IFIT5	hsa-miR-7180-3p
IFIT5	hsa-miR-6507-3p
IFIT5	hsa-miR-6884-3p
IFIT5	hsa-miR-873-3p
IFIT5	hsa-miR-3120-3p
IFIT5	hsa-miR-7252-5p
IFIT5	hsa-miR-7260-3p
IFIT5	hsa-miR-3688-5p
IFIT5	hsa-miR-577-5p
IFIT5	hsa-miR-106-5p
IFIT5	hsa-miR-7197-3p
IFIT5	hsa-miR-548c-3p
IFIT5	hsa-miR-708-5p
IFIT5	hsa-miR-6854-3p
IFIT5	hsa-miR-376-3p
IFIT5	hsa-miR-2437
IFIT5	hsa-miR-4254
IFIT5	hsa-miR-548b-5p
IFIT5	hsa-miR-548au-5p
IFIT5	hsa-miR-2370-5p
IFIT5	hsa-miR-208-5p
IFIT5	hsa-miR-129-5p

IFIT5	hsa-miR-5003-3p
IFIT5	hsa-miR-3162-5p
IFIT5	hsa-miR-7398b-3p
IFIT5	hsa-miR-1251-3p
IFIT5	hsa-miR-7337-3p
IFIT5	hsa-miR-449b-3p
IFIT5	hsa-miR-585-5p
IFIT5	hsa-miR-8863
IFIT5	hsa-miR-3127-3p
IFIT5	hsa-miR-510-5p
IFIT5	hsa-miR-4777-5p
IFIT5	hsa-miR-4718
IFIT5	hsa-miR-2440
IFIT5	hsa-miR-1252-5p
IFIT5	hsa-miR-185-5p
IFIT5	hsa-miR-331-5p
IFIT5	hsa-miR-3123
IFIT5	hsa-miR-1269a
IFIT5	hsa-miR-6715b-5p
IFIT5	hsa-miR-4735-5p
IFIT5	hsa-miR-651-5p
IFIT5	hsa-miR-3664-3p
IFIT5	hsa-miR-3150b-3p
IFIT5	hsa-miR-5572
IFIT5	hsa-miR-619-5p
IFIT5	hsa-miR-7374b-3p
IFIT5	hsa-miR-651
IFIT5	hsa-miR-7398l-3p
IFIT5	hsa-miR-483-3p
IFIT5	hsa-miR-3202
IFIT5	hsa-miR-505-5p
IFIT5	hsa-miR-382-5p
IFIT5	hsa-miR-8867
IFIT5	hsa-miR-6768-3p
IFIT5	hsa-miR-618
IFIT5	hsa-miR-216a
IFIT5	hsa-miR-6756-3p
IFIT5	hsa-miR-5680
IFIT5	hsa-miR-6881-3p
IFIT5	hsa-miR-8815
IFIT5	hsa-miR-1248
IFIT5	hsa-miR-1233-5p
IFIT5	hsa-miR-6810-5p
IFIT5	hsa-miR-6735-3p
IFIT5	hsa-miR-484
IFIT5	hsa-miR-562

IFIT5	hsa-miR-6830-3p
IFIT5	hsa-miR-3613-3p
IFIT5	hsa-miR-5696
IFIT5	hsa-miR-7859
IFIT5	hsa-miR-885
IFIT5	hsa-miR-3686
IFIT5	hsa-miR-3148
IFIT5	hsa-miR-504
IFIT5	hsa-miR-512
IFIT5	hsa-miR-199-3p
IFIT5	hsa-miR-182-5p
IFIT5	hsa-miR-362-5p
IFIT5	hsa-miR-4516
IFIT5	hsa-miR-4307
IFIT5	hsa-miR-1263
IFIT5	hsa-miR-383
IFIT5	hsa-miR-677
IFIT5	hsa-miR-7304-3p
IFIT5	hsa-miR-190-3p
IFIT5	hsa-miR-203a-3p.1
IFIT5	hsa-miR-2052
IFIT5	hsa-miR-518-3p
IFIT5	hsa-miR-4773
IFIT5	hsa-miR-609
IFIT5	hsa-miR-33
IFIT5	hsa-miR-514b-5p
IFIT5	hsa-miR-211-5p
IFIT5	hsa-miR-7363-5p
IFIT5	hsa-miR-6802-5p
IFIT5	hsa-miR-7264-5p
IFIT5	hsa-miR-7169-5p
IFIT5	hsa-miR-548l
IFIT5	hsa-miR-936
IFIT5	hsa-miR-561-5p
IFIT5	hsa-miR-1254
IFIT5	hsa-miR-4450
IFIT5	hsa-miR-516b
IFIT5	hsa-miR-2285j
IFIT5	hsa-miR-7976
IFIT5	hsa-miR-2371
IFIT5	hsa-miR-548ae-5p
IFIT5	hsa-miR-5583-3p
IFIT5	hsa-miR-6875-3p
IFIT5	hsa-miR-519
IFIT5	hsa-miR-1288
IFIT5	hsa-miR-660-5p

IFIT5	hsa-miR-6762-3p
IFIT5	hsa-miR-6857-5p
IFIT5	hsa-miR-655-3p
IFIT5	hsa-miR-7385f-3p
IFIT5	hsa-miR-192-5p
IFIT5	hsa-miR-4796-3p
IFIT5	hsa-miR-138-2-3p
IFIT5	hsa-miR-2284m
IFIT5	hsa-miR-4779
IFIT5	hsa-miR-7845-5p
IFIT5	hsa-miR-579-3p
IFIT5	hsa-miR-2365
IFIT5	hsa-miR-9b-3p
IFIT5	hsa-miR-7398a-3p
IFIT5	hsa-miR-2424
IFIT5	hsa-miR-3618
IFIT5	hsa-miR-5687
IFIT5	hsa-miR-2284ab
IFIT5	hsa-miR-8485
IFIT5	hsa-miR-7344-5p
IFIT5	hsa-miR-4502
IFIT5	hsa-miR-2472
IFIT5	hsa-miR-381-3p
IFIT5	hsa-miR-6731-5p
IFIT5	hsa-miR-6890-5p
IFIT5	hsa-miR-1233-3p
IFIT5	hsa-miR-6715b-3p
IFIT5	hsa-miR-4679
IFIT5	hsa-miR-6869-5p
IFIT5	hsa-miR-4742
IFIT5	hsa-miR-6867-3p
IFIT5	hsa-miR-3190-3p
IFIT5	hsa-miR-589-5p
IFIT5	hsa-miR-654-5p
IFIT5	hsa-miR-7388c-3p
IFIT5	hsa-miR-1584-3p
IFIT5	hsa-miR-376a-1-5p
IFIT5	hsa-miR-28-5p
IFIT5	hsa-miR-495-3p
IFIT5	hsa-miR-4657
IFIT5	hsa-miR-7269-5p
IFIT5	hsa-miR-7398l-5p
IFIT5	hsa-miR-548f-3p
IFIT5	hsa-miR-3920
IFIT5	hsa-miR-2290
IFIT5	hsa-miR-4698

IFIT5	hsa-miR-548
IFIT5	hsa-miR-877-5p
IFIT5	hsa-miR-579
IFIT5	hsa-miR-3973
IFIT5	hsa-miR-20-5p
IFIT5	hsa-miR-1272
IFIT5	hsa-miR-4436b-5p
IFIT5	hsa-miR-95-3p
IFIT5	hsa-miR-4514
IFIT5	hsa-miR-2362
IFIT5	hsa-miR-199
IFIT5	hsa-miR-500
LARP1	hsa-miR-4278
LARP1	hsa-miR-4746-5p
LARP1	hsa-miR-8100
LARP1	hsa-miR-199-5p
LARP1	hsa-miR-450b
LARP1	hsa-miR-6812-5p
LARP1	hsa-miR-7306-5p
LARP1	hsa-miR-6529a
LARP1	hsa-miR-4492
LARP1	hsa-miR-511
LARP1	hsa-miR-6891-5p
LARP1	hsa-miR-6795-5p
LARP1	hsa-miR-6993-5p
LARP1	hsa-miR-3613-3p
LARP1	hsa-miR-7386e-5p
LARP1	hsa-miR-570-3p
LARP1	hsa-miR-6561-5p
LARP1	hsa-miR-6569-5p
LARP1	hsa-miR-7070-5p
LARP1	hsa-miR-3138
LARP1	hsa-miR-124-3p
LARP1	hsa-miR-5693
LARP1	hsa-miR-7340-5p
LARP1	hsa-miR-2434
LARP1	hsa-miR-7a-2-3p
LARP1	hsa-miR-7012-5p
LARP1	hsa-miR-4303
LARP1	hsa-miR-3131
LARP1	hsa-miR-5011-5p
LARP1	hsa-miR-2116-3p
LARP1	hsa-miR-5193
LARP1	hsa-miR-6751-3p
LARP1	hsa-miR-124-3p.2
LARP1	hsa-miR-201-3p

LARP1	hsa-miR-1277-5p
LARP1	hsa-miR-6974-3p
LARP1	hsa-miR-450b-3p
LARP1	hsa-miR-6771-3p
LARP1	hsa-miR-6789-5p
LARP1	hsa-miR-21-3p
LARP1	hsa-miR-365b-5p
LARP1	hsa-miR-1976
LARP1	hsa-miR-556-5p
LARP1	hsa-miR-207
LARP1	hsa-miR-7289-3p
LARP1	hsa-miR-7114-5p
LARP1	hsa-miR-2366
LARP1	hsa-miR-3943
LARP1	hsa-miR-7268-3p
LARP1	hsa-miR-1710
LARP1	hsa-miR-376c-5p
LARP1	hsa-miR-7062-5p
LARP1	hsa-miR-124
LARP1	hsa-miR-4781-3p
LARP1	hsa-miR-6948-3p
LARP1	hsa-miR-1651-5p
LARP1	hsa-miR-619-5p
LARP1	hsa-miR-7168-5p
LARP1	hsa-miR-7386g-5p
LARP1	hsa-miR-1271-5p
LARP1	hsa-miR-1842
LARP1	hsa-miR-3120-3p
LARP1	hsa-miR-7007-5p
LARP1	hsa-miR-519e-5p
LARP1	hsa-miR-378g
LARP1	hsa-miR-128-1-5p
LARP1	hsa-miR-3548
LARP1	hsa-miR-148-5p
LARP1	hsa-miR-7320-5p
LARP1	hsa-miR-7357-5p
LARP1	hsa-miR-667-5p
LARP1	hsa-miR-6968-5p
LARP1	hsa-miR-635
LARP1	hsa-miR-3526
LARP1	hsa-miR-6516-5p
LARP1	hsa-miR-3658
LARP1	hsa-miR-664-5p
LARP1	hsa-miR-6320
LARP1	hsa-miR-6605-5p
LARP1	hsa-miR-1814

LARP1	hsa-miR-7194-3p
LARP1	hsa-miR-515-3p
LARP1	hsa-miR-6864-3p
LARP1	hsa-miR-1295b-5p
LARP1	hsa-miR-6244
LARP1	hsa-miR-3061-3p
LARP1	hsa-miR-301
LARP1	hsa-miR-6728-3p
LARP1	hsa-miR-7040-3p
LARP1	hsa-miR-383-3p
LARP1	hsa-miR-670-3p
LARP1	hsa-miR-3183
LARP1	hsa-miR-7368-3p
LARP1	hsa-miR-508-5p
LARP1	hsa-miR-8056
LARP1	hsa-miR-4441
LARP1	hsa-miR-8808
LARP1	hsa-miR-5588-5p
LARP1	hsa-miR-1642
LARP1	hsa-miR-6737-5p
LARP1	hsa-miR-421
LARP1	hsa-miR-184
LARP1	hsa-miR-4732-5p
LARP1	hsa-miR-371b-3p
LARP1	hsa-miR-136-5p
LARP1	hsa-miR-5619-3p
LARP1	hsa-miR-7299-5p
LARP1	hsa-miR-7388b-3p
LARP1	hsa-miR-8904a
LARP1	hsa-miR-525-5p
LARP1	hsa-miR-197-3p
LARP1	hsa-miR-6939-5p
LARP1	hsa-miR-548e-3p
LARP1	hsa-miR-223-5p
LARP1	hsa-miR-7264-3p
LARP1	hsa-miR-3692-3p
LARP1	hsa-miR-216
LARP1	hsa-miR-942
LARP1	hsa-miR-758-3p
LARP1	hsa-miR-7081-5p
LARP1	hsa-miR-3097-3p
LARP1	hsa-miR-8832
LARP1	hsa-miR-7386l-3p
LARP1	hsa-miR-148a-5p
LARP1	hsa-miR-548c-3p
LARP1	hsa-miR-6356

LARP1	hsa-miR-1224-5p
LARP1	hsa-miR-7187-3p
LARP1	hsa-miR-1612
LARP1	hsa-miR-5100
LARP1	hsa-miR-6922-5p
LARP1	hsa-miR-4286
LARP1	hsa-miR-184-3p
LARP1	hsa-miR-6216
LARP1	hsa-miR-8824
LARP1	hsa-miR-7687-5p
LARP1	hsa-miR-518c-5p
LARP1	hsa-miR-7065-3p
LARP1	hsa-miR-7034-5p
LARP1	hsa-miR-874-5p
LARP1	hsa-miR-4327
LARP1	hsa-miR-1958
LARP1	hsa-miR-193b-5p
LARP1	hsa-miR-3660
LARP1	hsa-miR-615-3p
LARP1	hsa-miR-1956
LARP1	hsa-miR-6965-5p
LARP1	hsa-miR-6547-3p
LARP1	hsa-miR-3547-3p
LARP1	hsa-miR-7305-5p
LARP1	hsa-miR-7684-5p
LARP1	hsa-miR-7050-3p
LARP1	hsa-miR-519
LARP1	hsa-miR-31-5p
LARP1	hsa-miR-22-3p
LARP1	hsa-miR-7451-5p
LARP1	hsa-miR-7398h-5p
LARP1	hsa-miR-3689-3p
LARP1	hsa-miR-7084-5p
LARP1	hsa-miR-6886-5p
LARP1	hsa-miR-7155-5p
LARP1	hsa-miR-3577
LARP1	hsa-miR-1732
LARP1	hsa-miR-6317
LARP1	hsa-miR-550b-2-5p
LARP1	hsa-miR-4517
LARP1	hsa-miR-29b-5p
LARP1	hsa-miR-3558-3p
LARP1	hsa-miR-1967
LARP1	hsa-miR-7221-5p
LARP1	hsa-miR-337-3p
LARP1	hsa-miR-3679-3p

LARP1	hsa-miR-873-5p
LARP1	hsa-miR-3094-5p
LARP1	hsa-miR-3163
LARP1	hsa-miR-222-3p
LARP1	hsa-miR-218a-1-3p
LARP1	hsa-miR-4713-5p
LARP1	hsa-miR-4723-3p
LARP1	hsa-miR-6947-3p
LARP1	hsa-miR-4676-5p
LARP1	hsa-miR-1457
LARP1	hsa-miR-2399-3p
LARP1	hsa-miR-762
LARP1	hsa-miR-1827
LARP1	hsa-miR-7288-5p
LARP1	hsa-miR-6865-5p
LARP1	hsa-miR-129-2-3p
LARP1	hsa-miR-514a-3p
LARP1	hsa-miR-6917-3p
LARP1	hsa-miR-152-5p
LARP1	hsa-miR-506-3p
LARP1	hsa-miR-522-3p
LARP1	hsa-miR-6834-3p
LARP1	hsa-miR-6582-3p
LARP1	hsa-miR-7440-3p
LARP1	hsa-miR-6958-5p
LARP1	hsa-miR-650abc
LARP1	hsa-miR-2357
LARP1	hsa-miR-6908-3p
LARP1	hsa-miR-7158-3p
LARP1	hsa-miR-592-3p
LARP1	hsa-miR-6741-3p
LARP1	hsa-miR-466bco-5p
LARP1	hsa-miR-518
LARP1	hsa-miR-450a-5p
LARP1	hsa-miR-3678-3p
LARP1	hsa-miR-3065-3p
LARP1	hsa-miR-5118
LARP1	hsa-miR-2406
LARP1	hsa-miR-1238-3p
LARP1	hsa-miR-3156-3p
LARP1	hsa-miR-7005-3p
LARP1	hsa-miR-410
LARP1	hsa-miR-2368-3p
LARP1	hsa-miR-587
LARP1	hsa-miR-7111-5p
LARP1	hsa-miR-3157-5p

LARP1	hsa-miR-7046-5p
LARP1	hsa-miR-2116-5p
LARP1	hsa-miR-676-3p
LARP1	hsa-miR-4641
LARP1	hsa-miR-6999-5p
LARP1	hsa-miR-7053-5p
LARP1	hsa-miR-20-5p
LARP1	hsa-miR-2985
LARP1	hsa-miR-4745-5p
LARP1	hsa-miR-7069-3p
LARP1	hsa-miR-3180-5p
LARP1	hsa-miR-9-5p
LARP1	hsa-miR-30e-5p
LARP1	hsa-miR-195-5p
LARP1	hsa-miR-6914-3p
LARP1	hsa-miR-548az-3p
LARP1	hsa-miR-6499-3p
LARP1	hsa-miR-7359-5p
LARP1	hsa-miR-185-3p
LARP1	hsa-miR-1680-5p
LARP1	hsa-miR-764-3p
LARP1	hsa-miR-216a-3p
LARP1	hsa-miR-669
LARP1	hsa-miR-6748-5p
LARP1	hsa-miR-8884
LARP1	hsa-miR-7047-5p
LARP1	hsa-miR-506-5p
LARP1	hsa-miR-616-3p
LARP1	hsa-miR-7184-3p
LARP1	hsa-miR-2324
LARP1	hsa-miR-7022-5p
LARP1	hsa-miR-1451-5p
LARP1	hsa-miR-1296
LARP1	hsa-miR-381-3p
LARP1	hsa-miR-1273h-5p
LARP1	hsa-miR-4778-3p
LARP1	hsa-miR-7020-3p
LARP1	hsa-miR-4689
LARP1	hsa-miR-7372-3p
LARP1	hsa-miR-6726-5p
LARP1	hsa-miR-6385
LARP1	hsa-miR-1285-3p
LARP1	hsa-miR-506-3p
LARP1	hsa-miR-496-3p
LARP1	hsa-miR-24-3p
LARP1	hsa-miR-7386-5p

LARP1	hsa-miR-8863
LARP1	hsa-miR-6652-5p
LARP1	hsa-miR-7243-3p
LARP1	hsa-miR-6404
LARP1	hsa-miR-411-3p
LARP1	hsa-miR-2431-3p
LARP1	hsa-miR-5681a
LARP1	hsa-miR-3084-5p
LARP1	hsa-miR-1635
LARP1	hsa-miR-673-3p
LARP1	hsa-miR-9768-3p
LARP1	hsa-miR-6501-5p
LARP1	hsa-miR-1894-5p
LARP1	hsa-miR-7289-5p
LARP1	hsa-miR-2462
LARP1	hsa-miR-454
LARP1	hsa-miR-8803
LARP1	hsa-miR-7068-5p
LARP1	hsa-miR-6535
LARP1	hsa-miR-7254-3p
LARP1	hsa-miR-676-3p
LARP1	hsa-miR-6852-5p
LARP1	hsa-miR-2471-3p
LARP1	hsa-miR-7342-3p
LARP1	hsa-miR-1911-3p
LARP1	hsa-miR-105b
LARP1	hsa-miR-7170-3p
LARP1	hsa-miR-653
LARP1	hsa-miR-2131-5p
LARP1	hsa-miR-6849-3p
LARP1	hsa-miR-6573-5p
LARP1	hsa-miR-1741
LARP1	hsa-miR-467-3p
LARP1	hsa-miR-412
LARP1	hsa-miR-8092
LARP1	hsa-miR-6418-3p
LARP1	hsa-miR-2364
LARP1	hsa-miR-595
LARP1	hsa-miR-7202-3p
LARP1	hsa-miR-3691-3p
LARP1	hsa-miR-502-5p
LARP1	hsa-miR-1915-3p
LARP1	hsa-miR-154-5p
LARP1	hsa-miR-7261-3p
LARP1	hsa-miR-5098
LARP1	hsa-miR-4765

LARP1	hsa-miR-6935-5p
LARP1	hsa-miR-6513-3p
LARP1	hsa-miR-2312
LARP1	hsa-miR-6081
LARP1	hsa-miR-4502
LARP1	hsa-miR-349
LARP1	hsa-miR-26-5p
LARP1	hsa-miR-201-5p
LARP1	hsa-miR-1266-5p
LARP1	hsa-miR-7119-3p
LARP1	hsa-miR-7459-3p
LARP1	hsa-miR-26-2-3p
LARP1	hsa-miR-130c-5p
LARP1	hsa-miR-7193-3p
LARP1	hsa-miR-7198-5p
LARP1	hsa-miR-6683-3p
LARP1	hsa-miR-7088-5p
LARP1	hsa-miR-6715b-5p
LARP1	hsa-miR-336-5p
LARP1	hsa-miR-7351-5p
LARP1	hsa-miR-3609
LARP1	hsa-miR-7008-3p
LARP1	hsa-miR-4659a-3p
LARP1	hsa-miR-7059-3p
LARP1	hsa-miR-6777-3p
LARP1	hsa-miR-672-5p
LARP1	hsa-miR-129-5p
LARP1	hsa-miR-7191-3p
LARP1	hsa-miR-7398a-3p
LARP1	hsa-miR-6830-3p
LARP1	hsa-miR-6755-5p
LARP1	hsa-miR-146a-3p
LARP1	hsa-miR-583
LARP1	hsa-miR-7681-3p
LARP1	hsa-miR-1185-3p
LARP1	hsa-miR-1587
LARP1	hsa-miR-7259-3p
LARP1	hsa-miR-6780a-5p
LARP1	hsa-miR-3176
LARP1	hsa-miR-3167-5p
LARP1	hsa-miR-101a-3p.2
LARP1	hsa-miR-519-3p
LARP1	hsa-miR-1952
LARP1	hsa-miR-624-3p
LARP1	hsa-miR-6778-3p
LARP1	hsa-miR-4322

LARP1	hsa-miR-3577-5p
LARP1	hsa-miR-1584-3p
LARP1	hsa-miR-7341-5p
LARP1	hsa-miR-6610-3p
LARP1	hsa-miR-204-3p
LARP1	hsa-miR-28-5p
LARP1	hsa-miR-367-5p
LARP1	hsa-miR-6617-3p
LARP1	hsa-miR-200c-5p
LARP1	hsa-miR-873a-5p.2
LARP1	hsa-miR-2407
LARP1	hsa-miR-3925-3p
LARP1	hsa-miR-6759-5p
LARP1	hsa-miR-186-3p
LARP1	hsa-miR-7035-5p
LARP1	hsa-miR-1894-3p
LARP1	hsa-miR-6165
LARP1	hsa-miR-18
LARP1	hsa-miR-29a-5p
LARP1	hsa-miR-7335-5p
LARP1	hsa-miR-7044-5p
LARP1	hsa-miR-5001-5p
LARP1	hsa-miR-6773-5p
LARP1	hsa-miR-1787
LARP1	hsa-miR-30a-3p
LARP1	hsa-miR-2424
LARP1	hsa-miR-7386h-3p
LARP1	hsa-miR-4747-5p
LARP1	hsa-miR-6967-3p
LARP1	hsa-miR-2385-5p
LARP1	hsa-miR-7390-5p
LARP1	hsa-miR-7286-3p
LARP1	hsa-miR-6813-5p
LARP1	hsa-miR-214-3p
LARP1	hsa-miR-2888
LARP1	hsa-miR-3552
LARP1	hsa-miR-7256-5p
LARP1	hsa-miR-608
LARP1	hsa-miR-32-3p
LARP1	hsa-miR-4768-3p
LARP1	hsa-miR-1705
LARP1	hsa-miR-7075-3p
LARP1	hsa-miR-7398j-3p
LARP1	hsa-miR-298-5p
LARP1	hsa-miR-618
LARP1	hsa-miR-539-5p

LARP1	hsa-miR-6072
LARP1	hsa-miR-7461-5p
LARP1	hsa-miR-1233-5p
LARP1	hsa-miR-2129
LARP1	hsa-miR-7019-5p
LARP1	hsa-miR-2412
LARP1	hsa-miR-2285
LARP1	hsa-miR-144-3p
LARP1	hsa-miR-1270
LARP1	hsa-miR-200b-5p
LARP1	hsa-miR-7224-3p
LARP1	hsa-miR-3059-3p
LARP1	hsa-miR-7975
LARP1	hsa-miR-760-3p
LARP1	hsa-miR-409-3p
LARP1	hsa-miR-7159-3p
LARP1	hsa-miR-548ah-5p
LARP1	hsa-miR-642-3p
LARP1	hsa-miR-4516
LARP1	hsa-miR-7082-3p
LARP1	hsa-miR-6810-5p
LARP1	hsa-miR-6990-5p
LARP1	hsa-miR-7371-5p
LARP1	hsa-miR-2285r
LARP1	hsa-miR-8847
LARP1	hsa-miR-7078-5p
LARP1	hsa-miR-382-5p
LARP1	hsa-miR-1305
LARP1	hsa-miR-6396
LARP1	hsa-miR-6991-3p
LARP1	hsa-miR-3058-5p
LARP1	hsa-miR-4668-3p
LARP1	hsa-miR-3136-3p
LARP1	hsa-miR-199-3p
LARP1	hsa-miR-6867-3p
LARP1	hsa-miR-3085-3p
LARP1	hsa-miR-3057-3p
LARP1	hsa-miR-4433a-3p
LARP1	hsa-miR-2467-3p
LARP1	hsa-miR-936
LARP1	hsa-miR-5698
LARP1	hsa-miR-19-5p
LARP1	hsa-miR-7269-5p
LARP1	hsa-miR-1663-3p
LARP1	hsa-miR-466b-4-3p
LARP1	hsa-miR-664

LARP1	hsa-miR-1794
LARP1	hsa-miR-150-5p
LARP1	hsa-miR-187-5p
LARP1	hsa-miR-4468
LARP1	hsa-miR-3596
LARP1	hsa-miR-6365
LARP1	hsa-miR-3061-5p
LARP1	hsa-miR-1568
LARP1	hsa-miR-597-5p
LARP1	hsa-miR-6373
LARP1	hsa-miR-205-5p
LARP1	hsa-miR-548a-3p
LARP1	hsa-miR-2450a
LARP1	hsa-miR-3135b
LARP1	hsa-miR-6608-5p
LARP1	hsa-miR-8119
LARP1	hsa-miR-8107
LARP1	hsa-miR-743b-3p
LARP1	hsa-miR-939-5p
LARP1	hsa-miR-298-3p
LARP1	hsa-miR-892b
LARP1	hsa-miR-15b-3p
LARP1	hsa-miR-616-5p
LARP1	hsa-miR-766-3p
LARP1	hsa-miR-6940-3p
LARP1	hsa-miR-4296
LARP1	hsa-miR-6810-3p
LARP1	hsa-let-7j-3p
LARP1	hsa-miR-504-3p
LARP1	hsa-miR-5703
LARP1	hsa-miR-145-3p
LARP1	hsa-miR-103b
LARP1	hsa-miR-6952-5p
LARP1	hsa-miR-7081-3p
LARP1	hsa-miR-4664-5p
LARP1	hsa-miR-6802-5p
LARP1	hsa-miR-6817-5p
LARP1	hsa-miR-2284s
LARP1	hsa-miR-8085
LARP1	hsa-miR-766-3p
LARP1	hsa-miR-524-5p
LARP1	hsa-miR-7006-5p
LARP1	hsa-miR-210-5p
LARP1	hsa-miR-6868-5p
LARP1	hsa-miR-374
LARP1	hsa-miR-6862-3p

LARP1	hsa-miR-6734-5p
LARP1	hsa-miR-513a
LARP1	hsa-miR-7399-5p
LARP1	hsa-miR-4526
LARP1	hsa-miR-6796-5p
LARP1	hsa-miR-6954-5p
LARP1	hsa-miR-4751
LARP1	hsa-miR-2285z
LARP1	hsa-miR-3596d
LARP1	hsa-miR-6338
LARP1	hsa-miR-2114-5p
LARP1	hsa-miR-365-12-5p
LARP1	hsa-miR-8052
LARP1	hsa-miR-6930-3p
LARP1	hsa-miR-1199-5p
LARP1	hsa-miR-432-5p
LARP1	hsa-miR-548d-5p
LARP1	hsa-miR-6826-5p
LARP1	hsa-miR-124-3p.1
LARP1	hsa-miR-7076-5p
LARP1	hsa-miR-1664-3p
LARP1	hsa-miR-1188-5p
LARP1	hsa-miR-1545a-5p
LARP1	hsa-miR-7352-3p
LARP1	hsa-miR-194-2-3p
LARP1	hsa-miR-4731-3p
LARP1	hsa-miR-7386l-5p
LARP1	hsa-miR-429-5p
LARP1	hsa-miR-463-3p
LARP1	hsa-miR-6598-5p
LARP1	hsa-miR-7376-3p
LARP1	hsa-miR-548i-3p
LARP1	hsa-miR-7657-5p
LARP1	hsa-miR-211-5p
LARP1	hsa-miR-2904
LARP1	hsa-miR-6586-5p
LARP1	hsa-miR-6860
LARP1	hsa-miR-6762-5p
LARP1	hsa-miR-7442-5p
LARP1	hsa-miR-466c-3p
LARP1	hsa-miR-6934-3p
LARP1	hsa-miR-6418-5p
LARP1	hsa-miR-3083-5p
LARP1	hsa-miR-6732-5p
LARP1	hsa-miR-6822-5p
LARP1	hsa-miR-145a-3p

LARP1	hsa-miR-218-5p
LARP1	hsa-miR-25-5p
LARP1	hsa-miR-98-3p
LARP1	hsa-miR-7052-5p
LARP1	hsa-miR-142
LARP1	hsa-miR-6953-3p
LARP1	hsa-miR-2893
LARP1	hsa-miR-193-3p
LARP1	hsa-miR-7385f-3p
LARP1	hsa-miR-329-5p
LARP1	hsa-miR-6984-3p
LARP1	hsa-miR-181a-2-3p
LARP1	hsa-miR-7856-5p
LARP1	hsa-miR-7110-3p
LARP1	hsa-miR-29-5p
LARP1	hsa-miR-6923-3p
LARP1	hsa-miR-654-5p
LARP1	hsa-miR-452-3p
LARP1	hsa-miR-488-5p
LARP1	hsa-miR-1614-3p
LARP1	hsa-miR-1982-3p
LARP1	hsa-miR-8075
LARP1	hsa-miR-377-3p
LARP1	hsa-miR-6819-5p
LARP1	hsa-miR-770-3p
LARP1	hsa-miR-7398-5p
LARP1	hsa-miR-7371b-5p
LARP1	hsa-miR-1929-3p
LARP1	hsa-miR-4756-3p
LARP1	hsa-miR-6793-5p
LARP1	hsa-miR-744-5p
LARP1	hsa-miR-489-3p
LARP1	hsa-miR-7003-3p
LARP1	hsa-miR-6986-5p
LARP1	hsa-miR-4789-5p
LARP1	hsa-miR-2442
LARP1	hsa-miR-6931-5p
LARP1	hsa-miR-585-5p
LARP1	hsa-miR-4667-5p
LARP1	hsa-miR-7207-5p
LARP1	hsa-miR-3110-5p
LARP1	hsa-miR-561-5p
LARP1	hsa-miR-3068-5p
LARP1	hsa-miR-665-3p
LARP1	hsa-miR-6576-3p
LARP1	hsa-miR-6962-3p

LARP1	hsa-miR-17-3p
LARP1	hsa-miR-7297-3p
LARP1	hsa-miR-1456-5p
LARP1	hsa-miR-193b-3p
LARP1	hsa-miR-4744
LARP1	hsa-miR-6829-3p
LARP1	hsa-miR-4796-5p
LARP1	hsa-miR-5124a
LARP1	hsa-miR-2335
LARP1	hsa-miR-2329-3p
LARP1	hsa-miR-1231-3p
LARP1	hsa-miR-4495
LARP1	hsa-miR-363-5p
LARP1	hsa-miR-7368-5p
LARP1	hsa-miR-140-5p
LARP1	hsa-miR-6900-3p
LARP1	hsa-miR-6907-5p
LARP1	hsa-miR-101-3p
LARP1	hsa-miR-133a-3p.2
LARP1	hsa-miR-5114
LARP1	hsa-miR-7314-5p
LARP1	hsa-miR-2285ad
LARP1	hsa-miR-216a
LARP1	hsa-miR-6751-5p
LARP1	hsa-miR-327
LARP1	hsa-miR-7688-5p
LARP1	hsa-miR-380-5p
LARP1	hsa-miR-129-3p
LARP1	hsa-miR-1720-5p
LARP1	hsa-miR-3123
LARP1	hsa-miR-344i
LARP1	hsa-miR-6917-5p
LARP1	hsa-miR-743a-3p
LARP1	hsa-miR-7303-5p
LARP1	hsa-miR-7160-5p
LARP1	hsa-miR-6842-5p
LARP1	hsa-miR-1301-3p
LARP1	hsa-miR-3125
LARP1	hsa-miR-26
LARP1	hsa-miR-103-5p
LARP1	hsa-miR-135a-2-3p
LARP1	hsa-miR-592
LARP1	hsa-miR-6832-5p
LARP1	hsa-miR-4749-3p
LARP1	hsa-miR-1199-3p
LARP1	hsa-miR-188-3p

LARP1	hsa-miR-7057-5p
LARP1	hsa-miR-4763-3p
LARP1	hsa-miR-6916-5p
LARP1	hsa-miR-7462-3p
LARP1	hsa-miR-5125
LARP1	hsa-miR-6873-5p
LARP1	hsa-miR-708-5p
LARP1	hsa-miR-4649-3p
LARP1	hsa-miR-382-3p
LARP1	hsa-miR-7064-5p
LARP1	hsa-miR-4667-3p
LARP1	hsa-miR-1943-5p
LARP1	hsa-miR-3922-3p
LARP1	hsa-miR-6951-3p
LARP1	hsa-miR-6553-3p
LARP1	hsa-miR-7481-3p
LARP1	hsa-miR-7325-5p
LARP1	hsa-miR-196a-3p
LARP1	hsa-miR-320e
LARP1	hsa-miR-1953
LARP1	hsa-miR-6730-5p
LARP1	hsa-miR-1226-5p
LARP1	hsa-miR-770-5p
LARP1	hsa-miR-4722-3p
LARP1	hsa-miR-6855-5p
LARP1	hsa-miR-7386a-5p
LARP1	hsa-miR-4722-5p
LARP1	hsa-miR-351-5p
LARP1	hsa-miR-3073a-3p
LARP1	hsa-miR-7397-3p
LARP1	hsa-miR-5685
LARP1	hsa-miR-3969
LARP1	hsa-miR-3619-5p
LARP1	hsa-miR-6371
LARP1	hsa-miR-140-3p.2
LARP1	hsa-miR-6541
LARP1	hsa-miR-7367-5p
LARP1	hsa-miR-371a-5p
LARP1	hsa-miR-450b-5p
LARP1	hsa-miR-1548-5p
LARP1	hsa-miR-205-5p
LARP1	hsa-miR-1790
LARP1	hsa-miR-196-5p
LARP1	hsa-miR-3091-3p
LARP1	hsa-miR-3189-3p
LARP1	hsa-miR-1786

LARP1	hsa-miR-3116
LARP1	hsa-miR-8089
LARP1	hsa-miR-7249-5p
LARP1	hsa-miR-432-3p
LARP1	hsa-miR-7691
LARP1	hsa-miR-7324-3p
LARP1	hsa-miR-655
LARP1	hsa-miR-4430
LARP1	hsa-miR-372-5p
LARP1	hsa-miR-6790-5p
LARP1	hsa-miR-291
LARP1	hsa-miR-1229-5p
LARP1	hsa-miR-1237-3p
LARP1	hsa-miR-6724-5p
LARP1	hsa-miR-6344
LARP1	hsa-miR-4446-3p
LARP1	hsa-miR-1728-5p
LARP1	hsa-miR-5591-3p
LARP1	hsa-miR-6955-5p
LARP1	hsa-miR-1912
LARP1	hsa-miR-4313
LARP1	hsa-miR-3617-5p
LARP1	hsa-miR-3557-5p
LARP1	hsa-miR-939-3p
LARP1	hsa-let-7a-2-3p
LARP1	hsa-miR-6800-5p
LARP1	hsa-miR-22-5p
LARP1	hsa-miR-548aef
LARP1	hsa-miR-196b-3p
LARP1	hsa-miR-2429
LARP1	hsa-miR-2360
LARP1	hsa-miR-496.2
LARP1	hsa-miR-30-5p
LARP1	hsa-miR-203
LARP1	hsa-miR-671-5p
LARP1	hsa-miR-4310
LARP1	hsa-miR-224-5p
LARP1	hsa-miR-6417
LARP1	hsa-miR-5582-3p
LARP1	hsa-miR-101-1-5p
LARP1	hsa-miR-5088-3p
LARP1	hsa-miR-2466-3p
LARP1	hsa-miR-1225
LARP1	hsa-miR-3128
LARP1	hsa-miR-214-5p
LARP1	hsa-miR-2421

LARP1	hsa-miR-4793-5p
LARP1	hsa-miR-6132
LARP1	hsa-miR-4780
LARP1	hsa-miR-193-5p
LARP1	hsa-miR-330-3p
LARP1	hsa-miR-335-5p
LARP1	hsa-miR-3651
LARP1	hsa-miR-6981-5p
LARP1	hsa-miR-7240-5p
LARP1	hsa-miR-371a-3p
LARP1	hsa-miR-4520-5p
LARP1	hsa-miR-7185-3p
LARP1	hsa-miR-1193-5p
LARP1	hsa-miR-4316
LARP1	hsa-miR-7117-3p
LARP1	hsa-miR-7071-5p
LARP1	hsa-miR-6868-3p
LARP1	hsa-miR-548i-5p
LARP1	hsa-miR-6540-3p
LARP1	hsa-miR-6630-3p
LARP1	hsa-miR-1283
LARP1	hsa-miR-1545c-3p
LARP1	hsa-miR-3085-5p
LARP1	hsa-miR-8908f
LARP1	hsa-miR-7385d-3p
LARP1	hsa-miR-5699-5p
LARP1	hsa-miR-7216-5p
LARP1	hsa-miR-4758-3p
LARP1	hsa-miR-96-5p
LARP1	hsa-miR-6837-5p
LARP1	hsa-miR-369-3p
LARP1	hsa-miR-4423-5p
LARP1	hsa-miR-378
LARP1	hsa-miR-7357-3p
LARP1	hsa-miR-7080-5p
LARP1	hsa-miR-760-5p
LARP1	hsa-miR-24-3p
LARP1	hsa-miR-4490
LARP1	hsa-miR-4711-3p
LARP1	hsa-miR-7668-3p
LARP1	hsa-miR-96-5p
LARP1	hsa-miR-1329-5p
LARP1	hsa-miR-2317
LARP1	hsa-miR-7676-5p
LARP1	hsa-miR-1809
LARP1	hsa-miR-4684-5p

LARP1	hsa-miR-1453
LARP1	hsa-miR-3910
LARP1	hsa-miR-5621-3p
LARP1	hsa-miR-503-5p
LARP1	hsa-miR-1773-5p
LARP1	hsa-miR-7009-5p
LARP1	hsa-miR-2325
LARP1	hsa-miR-7000-5p
LARP1	hsa-miR-3661
LARP1	hsa-miR-1841
LARP1	hsa-miR-6995-3p
LARP1	hsa-miR-4799-3p
LARP1	hsa-miR-1693
LARP1	hsa-miR-7214-3p
LARP1	hsa-miR-7386h-5p
LARP1	hsa-miR-4498
LARP1	hsa-miR-338-5p
LARP1	hsa-miR-6779-3p
LARP1	hsa-miR-6989-3p
LARP1	hsa-miR-7093-5p
LARP1	hsa-miR-1556
LARP1	hsa-miR-1938
LARP1	hsa-miR-498
LARP1	hsa-miR-8093
LARP1	hsa-miR-628-3p
LARP1	hsa-miR-7220-5p
LARP1	hsa-miR-6735-3p
LARP1	hsa-miR-5591-5p
LARP1	hsa-miR-1251-5p
LARP1	hsa-miR-8111
LARP1	hsa-miR-449-5p
LARP1	hsa-miR-34b-3p
LARP1	hsa-miR-648
LARP1	hsa-miR-28-5p
LARP1	hsa-miR-3590-5p
LARP1	hsa-miR-873-5p
LARP1	hsa-miR-1772-5p
LARP1	hsa-miR-4447
LARP1	hsa-miR-6721-5p
LARP1	hsa-miR-370
LARP1	hsa-miR-138-5p
LARP1	hsa-miR-27-5p
LARP1	hsa-miR-6411
LARP1	hsa-miR-328
LARP1	hsa-miR-93-5p
LARP1	hsa-miR-1791-5p

LARP1	hsa-miR-4771
LARP1	hsa-miR-2431-5p
LARP1	hsa-miR-544b
LARP1	hsa-miR-1659
LARP1	hsa-miR-7054-5p
LARP1	hsa-miR-6556-5p
LARP1	hsa-miR-8059
LARP1	hsa-miR-7454-3p
LARP1	hsa-miR-4253
LARP1	hsa-miR-7466-3p
LARP1	hsa-miR-7168-3p
LARP1	hsa-miR-361-5p
LARP1	hsa-miR-7042-3p
LARP1	hsa-miR-30e-3p
LARP1	hsa-miR-185-5p
LARP1	hsa-miR-8062
LARP1	hsa-miR-451-3p
LARP1	hsa-miR-362-5p
LARP1	hsa-miR-7001-3p
LARP1	hsa-miR-467f
LARP1	hsa-miR-132-3p
LARP1	hsa-miR-7020-5p
LARP1	hsa-miR-300-5p
LARP1	hsa-miR-758-5p
LARP1	hsa-miR-659-3p
LARP1	hsa-miR-3079-3p
LARP1	hsa-miR-6766-5p
LARP1	hsa-miR-5087
LARP1	hsa-miR-27a-5p
LARP1	hsa-miR-665
LARP1	hsa-miR-7321-5p
LARP1	hsa-miR-7085-5p
LARP1	hsa-miR-7031-3p
LARP1	hsa-miR-3064-3p
LARP1	hsa-miR-1248
LARP1	hsa-miR-26b-3p
LARP1	hsa-miR-1903
LARP1	hsa-miR-221-5p
LARP1	hsa-miR-6742-3p
LARP1	hsa-miR-6572-3p
LARP1	hsa-miR-7287-3p
LARP1	hsa-miR-184-5p
LARP1	hsa-miR-1287-3p
LARP1	hsa-miR-6813-3p
LARP1	hsa-miR-346-3p
LARP1	hsa-miR-6834-5p

LARP1	hsa-miR-7039-5p
LARP1	hsa-miR-7365-3p
LARP1	hsa-miR-2287
LARP1	hsa-miR-4801
LARP1	hsa-miR-6120-3p
LARP1	hsa-miR-5106
LARP1	hsa-miR-1982-5p
LARP1	hsa-miR-7117-5p
LARP1	hsa-miR-329
LARP1	hsa-miR-7184-5p
LARP1	hsa-miR-199-1-5p
LARP1	hsa-miR-6077
LARP1	hsa-miR-7335-3p
LARP1	hsa-miR-542-5p
LARP1	hsa-miR-23
LARP1	hsa-miR-2489
LARP1	hsa-miR-6325
LARP1	hsa-miR-5196-3p
LARP1	hsa-miR-1233-3p
LARP1	hsa-miR-670
LARP1	hsa-miR-3664-3p
LARP1	hsa-miR-7250-3p
LARP1	hsa-miR-6953-5p
LARP1	hsa-miR-3919
LARP1	hsa-miR-7308-3p
LARP1	hsa-miR-6978-5p
LARP1	hsa-miR-7276-3p
LARP1	hsa-miR-6748-3p
LARP1	hsa-miR-4428
LARP1	hsa-miR-92a-5p
LARP1	hsa-miR-705
LARP1	hsa-miR-3937
LARP1	hsa-miR-499a-3p
LARP1	hsa-miR-2432
LARP1	hsa-miR-877-5p
LARP1	hsa-miR-6800-3p
LARP1	hsa-miR-3613b
LARP1	hsa-miR-27b-5p
LARP1	hsa-miR-518-5p
LARP1	hsa-miR-3153
LARP1	hsa-miR-7150
LARP1	hsa-miR-6510-5p
LARP1	hsa-miR-7025-5p
LARP1	hsa-miR-491-5p
LARP1	hsa-miR-347
LARP1	hsa-miR-6506-5p

LARP1	hsa-miR-182-3p
LARP1	hsa-let-7b-3p
LARP1	hsa-miR-27
LARP1	hsa-miR-4719
LARP1	hsa-miR-6372
LARP1	hsa-miR-7156-5p
LARP1	hsa-miR-7386m-5p
LARP1	hsa-miR-1808
LARP1	hsa-miR-7363-5p
LARP1	hsa-miR-4256
LARP1	hsa-miR-6699-5p
LARP1	hsa-miR-8827
LARP1	hsa-miR-362-5p
LARP1	hsa-miR-7074-5p
LARP1	hsa-miR-370-5p
LARP1	hsa-miR-1594
LARP1	hsa-miR-194-5p
LARP1	hsa-miR-499
LARP1	hsa-miR-623
LARP1	hsa-miR-6809-5p
LARP1	hsa-miR-6715a-3p
LARP1	hsa-miR-1737
LARP1	hsa-miR-2405
LARP1	hsa-miR-2446
LARP1	hsa-miR-551b-5p
LARP1	hsa-miR-7316-5p
LARP1	hsa-miR-1747-3p
LARP1	hsa-miR-7351-3p
LARP1	hsa-miR-7454-5p
LARP1	hsa-miR-7371-3p
LARP1	hsa-miR-6885-5p
LARP1	hsa-miR-5697
LARP1	hsa-miR-2285y
LARP1	hsa-miR-1955-5p
LARP1	hsa-miR-7346-3p
LARP1	hsa-miR-1343
LARP1	hsa-miR-4696
LARP1	hsa-miR-2474
LARP1	hsa-miR-8818
LARP1	hsa-miR-8098
LARP1	hsa-miR-6780a-3p
LARP1	hsa-miR-544a
LARP1	hsa-miR-18-5p
LARP1	hsa-miR-125-5p
LARP1	hsa-miR-873b
LARP1	hsa-miR-493-5p

LARP1	hsa-miR-15-5p
LARP1	hsa-miR-149-5p
LARP1	hsa-miR-609
LARP1	hsa-miR-7155-3p
LARP1	hsa-miR-6888-5p
LARP1	hsa-miR-4328
LARP1	hsa-miR-6518
LARP1	hsa-miR-6835-5p
LARP1	hsa-miR-7482-3p
LARP1	hsa-miR-548ai
LARP1	hsa-miR-3619-3p
LARP1	hsa-miR-7317-3p
LARP1	hsa-miR-1813
LARP1	hsa-miR-297bc-3p
LARP1	hsa-miR-500b-5p
LARP1	hsa-miR-143-3p
LARP1	hsa-miR-885
LARP1	hsa-miR-6695-5p
LARP1	hsa-miR-6760-5p
LARP1	hsa-miR-6405
LARP1	hsa-miR-101a-5p
LARP1	hsa-miR-212-3p
LARP1	hsa-miR-216c-3p
LARP1	hsa-miR-133b
LARP1	hsa-miR-7118-5p
LARP1	hsa-miR-6624-3p
LARP1	hsa-miR-6881-3p
LARP1	hsa-miR-1777
LARP1	hsa-miR-4779
LARP1	hsa-miR-1608
LARP1	hsa-miR-1207-5p
LARP1	hsa-miR-5189-5p
LARP1	hsa-miR-511-5p
LARP1	hsa-miR-6804-5p
LARP1	hsa-miR-516a
LARP1	hsa-miR-539-5p
LARP1	hsa-miR-3587
LARP1	hsa-miR-383-5p.1
LARP1	hsa-miR-7217-5p
LARP1	hsa-miR-6873-3p
LARP1	hsa-miR-7270-3p
LARP1	hsa-miR-8905
LARP1	hsa-miR-6752-5p
LARP1	hsa-let-7ag-3p
LARP1	hsa-miR-5006-3p
LARP1	hsa-miR-551-5p

LARP1	hsa-miR-2285af
LARP1	hsa-miR-205-3p
LARP1	hsa-miR-712-5p
LARP1	hsa-miR-107-3p
LARP1	hsa-miR-204-5p
LARP1	hsa-miR-7354-5p
LARP1	hsa-miR-891
LARP1	hsa-miR-4471
LARP1	hsa-miR-4726-3p
LARP1	hsa-miR-132-3p
LARP1	hsa-miR-6758-3p
LARP1	hsa-miR-7237-3p
LARP1	hsa-miR-7478-5p
LARP1	hsa-miR-6825-5p
LARP1	hsa-miR-877-3p
LARP1	hsa-miR-466-5p
LARP1	hsa-miR-466i-3p
LARP1	hsa-miR-3090-3p
LARP1	hsa-miR-455-3p
LARP1	hsa-miR-1740-3p
LARP1	hsa-miR-4446-5p
LARP1	hsa-miR-4652-3p
LARP1	hsa-miR-431-3p
LARP1	hsa-miR-6661-5p
LARP1	hsa-miR-6545-5p
LARP1	hsa-miR-6858-5p
LARP1	hsa-miR-7022-3p
LARP1	hsa-miR-3471
LARP1	hsa-miR-6768-3p
LARP1	hsa-miR-130
LARP1	hsa-miR-2330-5p
LARP1	hsa-miR-341-5p
LARP1	hsa-miR-2347
LARP1	hsa-miR-3101-5p
LARP1	hsa-miR-3187-5p
LARP1	hsa-miR-4752
LARP1	hsa-miR-6120-5p
LARP1	hsa-miR-759
LARP1	hsa-miR-300
LARP1	hsa-miR-92a-1-5p
LARP1	hsa-miR-6720-5p
LARP1	hsa-miR-6851-3p
LARP1	hsa-miR-4419b
LARP1	hsa-miR-296-5p
LARP1	hsa-miR-3618-3p
LARP1	hsa-miR-7242-5p

LARP1	hsa-miR-2355-5p
LARP1	hsa-miR-483-3p
LARP1	hsa-miR-3914
LARP1	hsa-miR-6937-3p
LARP1	hsa-miR-7087-5p
LARP1	hsa-miR-143-5p
LARP1	hsa-miR-7255-5p
LARP1	hsa-miR-2450bd
LARP1	hsa-miR-656
LARP1	hsa-miR-3544
LARP1	hsa-miR-7314-3p
LARP1	hsa-miR-145-5p
LARP1	hsa-miR-3103-5p
LARP1	hsa-miR-3607-3p
LARP1	hsa-miR-7206-3p
LARP1	hsa-miR-133ab
LARP1	hsa-miR-4802-5p
LARP1	hsa-miR-7385a-3p
LARP1	hsa-miR-95-5p
LARP1	hsa-miR-2368-5p
LARP1	hsa-miR-105-5p
LARP1	hsa-miR-1968-3p
LARP1	hsa-miR-4436b-5p
LARP1	hsa-miR-496
LARP1	hsa-miR-7177-5p
LARP1	hsa-miR-7355-5p
LARP1	hsa-miR-19b-5p
LARP1	hsa-miR-549a
LARP1	hsa-miR-1679
LARP1	hsa-miR-7211-5p
LARP1	hsa-miR-6843-3p
LARP1	hsa-miR-7110-5p
LARP1	hsa-miR-6836-5p
LARP1	hsa-miR-7652-5p
LARP1	hsa-miR-6898-3p
LARP1	hsa-miR-322-5p
LARP1	hsa-miR-144-3p
LARP1	hsa-miR-2399-5p
LARP1	hsa-miR-7319-5p
LARP1	hsa-miR-7030-3p
LARP1	hsa-miR-3569
LARP1	hsa-miR-883b-5p
LARP1	hsa-miR-6362
LARP1	hsa-miR-629-3p
LARP1	hsa-miR-634
LARP1	hsa-miR-4662b

LARP1	hsa-miR-7072-5p
LARP1	hsa-miR-1814a
LARP1	hsa-miR-140-5p
LARP1	hsa-miR-1265
LARP1	hsa-miR-6384
LARP1	hsa-miR-138-3p
LARP1	hsa-miR-210-3p
LARP1	hsa-miR-8829
LARP1	hsa-miR-6926-5p
LARP1	hsa-let-7f-2-3p
LARP1	hsa-miR-1661
LARP1	hsa-miR-7688-3p
LARP1	hsa-miR-7244-5p
LARP1	hsa-miR-2472
LARP1	hsa-miR-6946-5p
LARP1	hsa-miR-6895-3p
LARP1	hsa-miR-7437-3p
LARP1	hsa-miR-5197-3p
LARP1	hsa-let-7abf-3p
LARP1	hsa-miR-3084-3p
LARP1	hsa-miR-875
LARP1	hsa-miR-338-3p
LARP1	hsa-miR-4270
LARP1	hsa-miR-6552-3p
LARP1	hsa-miR-7273-5p
LARP1	hsa-miR-296-3p
LARP1	hsa-miR-7056-3p
LARP1	hsa-miR-707
LARP1	hsa-miR-628-3p
LARP1	hsa-miR-7278-3p
LARP1	hsa-miR-1297
LARP1	hsa-miR-33-3p
LARP1	hsa-miR-7208-3p
LARP1	hsa-miR-5622-5p
LARP1	hsa-miR-2305
LARP1	hsa-miR-3934-5p
LARP1	hsa-miR-8904b
LARP1	hsa-miR-6964-5p
LARP1	hsa-miR-2400
LARP1	hsa-miR-6403
LARP1	hsa-miR-7166-3p
LARP1	hsa-miR-7010-5p
LARP1	hsa-miR-6956-5p
LARP1	hsa-miR-140-3p.1
LARP1	hsa-miR-7191-5p
LARP1	hsa-miR-578

LARP1	hsa-miR-4642
LARP1	hsa-miR-6891-3p
LARP1	hsa-miR-7028-3p
LARP1	hsa-miR-2394
LARP1	hsa-miR-6589-5p
LARP1	hsa-miR-371b-5p
LARP1	hsa-miR-3058-3p
LARP1	hsa-miR-3714
LARP1	hsa-miR-637
LARP1	hsa-miR-3130-5p
LARP1	hsa-miR-4697-3p
LARP1	hsa-miR-6862-5p
LARP1	hsa-miR-126-5p
LARP1	hsa-miR-451b
LARP1	hsa-miR-497-5p
LARP1	hsa-miR-655-3p
LARP1	hsa-miR-1722-5p
LARP1	hsa-miR-7467-3p
LARP1	hsa-miR-432-5p
LARP1	hsa-miR-1225-5p
LARP1	hsa-miR-211-3p
LARP1	hsa-miR-122
LARP1	hsa-miR-7652-3p
LARP1	hsa-miR-1252
LARP1	hsa-miR-6951-5p
LARP1	hsa-miR-6878-5p
LARP1	hsa-miR-8870
LARP1	hsa-miR-3170
LARP1	hsa-miR-6815-5p
LARP1	hsa-miR-548ba
LARP1	hsa-miR-381-3p
LARP1	hsa-miR-467g
LARP1	hsa-miR-650
LARP1	hsa-miR-574-3p
LARP1	hsa-miR-8888
LARP1	hsa-miR-3620-3p
LARP1	hsa-miR-541-3p
LARP1	hsa-miR-299b-5p
LARP1	hsa-miR-8891
LARP1	hsa-miR-8908e
LARP1	hsa-miR-7307-5p
LARP1	hsa-miR-4743-3p
LARP1	hsa-miR-369-3p
LARP1	hsa-miR-222-5p
LARP1	hsa-miR-6523a
LARP1	hsa-miR-23a-5p

LARP1	hsa-miR-29b-2-5p
LARP1	hsa-miR-6779-5p
LARP1	hsa-miR-7977
LARP1	hsa-miR-6909-3p
LARP1	hsa-miR-6905-5p
LARP1	hsa-miR-3974
LARP1	hsa-miR-6355
LARP1	hsa-miR-8800
LARP1	hsa-miR-6930-5p
LARP1	hsa-miR-326
LARP1	hsa-miR-7053-3p
LARP1	hsa-miR-3470a
LARP1	hsa-miR-4448
LARP1	hsa-miR-219-5p
LARP1	hsa-miR-6727-3p
LARP1	hsa-miR-1343-5p
LARP1	hsa-miR-6878-3p
LARP1	hsa-miR-1226-3p
LARP1	hsa-miR-7333-3p
LARP1	hsa-miR-7200-5p
LARP1	hsa-miR-7261-5p
LARP1	hsa-miR-6632-5p
LARP1	hsa-miR-6704-5p
LARP1	hsa-miR-3070-3p
LARP1	hsa-miR-7483-3p
LARP1	hsa-miR-93-3p
LARP1	hsa-miR-7372-5p
LARP1	hsa-miR-4741
LARP1	hsa-miR-1266
LARP1	hsa-miR-4518
LARP1	hsa-miR-4470
LARP1	hsa-miR-6845-5p
LARP1	hsa-miR-6987-5p
LARP1	hsa-miR-454-5p
LARP1	hsa-miR-3148
LARP1	hsa-miR-330-3p
LARP1	hsa-miR-6866-5p
LARP1	hsa-miR-324
LARP1	hsa-miR-3584-5p
LARP1	hsa-miR-4273
LARP1	hsa-miR-1241
LARP1	hsa-miR-1930-3p
LARP1	hsa-miR-4323
LARP1	hsa-miR-567
LARP1	hsa-miR-3928-3p
LARP1	hsa-miR-7481-5p

LARP1	hsa-miR-320-5p
LARP1	hsa-miR-3533
LARP1	hsa-miR-423-5p
LARP1	hsa-miR-6596-3p
LARP1	hsa-miR-7658-3p
LARP1	hsa-miR-3611
LARP1	hsa-miR-7277-3p
LARP1	hsa-miR-8817
LARP1	hsa-miR-3070-5p
LARP1	hsa-miR-9-5p
LARP1	hsa-miR-6949-3p
LARP1	hsa-miR-92b-5p
LARP1	hsa-miR-4438
LARP1	hsa-miR-873-3p
LARP1	hsa-miR-654-5p
LARP1	hsa-miR-2389
LARP1	hsa-miR-3665
LARP1	hsa-miR-6337
LARP1	hsa-miR-6791-3p
LARP1	hsa-miR-4437
LARP1	hsa-miR-7386i-3p
LARP1	hsa-miR-2323
LARP1	hsa-miR-4265
LARP1	hsa-miR-1956-3p
LARP1	hsa-miR-2464-3p
LARP1	hsa-miR-1632-3p
LARP1	hsa-miR-769-3p
LARP1	hsa-miR-5616-3p
LARP1	hsa-miR-29b-1-5p
LARP1	hsa-miR-541-3p
LARP1	hsa-miR-185-5p
LARP1	hsa-miR-4297
LARP1	hsa-miR-4530
LARP1	hsa-miR-743ab-3p
LARP1	hsa-miR-6750-5p
LARP1	hsa-miR-6688-3p
LARP1	hsa-miR-371
LARP1	hsa-miR-7193-5p
LARP1	hsa-miR-3475-3p
LARP1	hsa-miR-2449
LARP1	hsa-miR-222-3p
LARP1	hsa-miR-4534
LARP1	hsa-miR-182-5p
LARP1	hsa-miR-1275
LARP1	hsa-miR-144-5p
LARP1	hsa-miR-1781-5p

LARP1	hsa-miR-2465
LARP1	hsa-miR-106-3p
LARP1	hsa-miR-1762
LARP1	hsa-miR-4705
LARP1	hsa-miR-4269
LARP1	hsa-miR-669bf-3p
LARP1	hsa-miR-4257
LARP1	hsa-miR-7344-5p
LARP1	hsa-miR-3622b-5p
LARP1	hsa-miR-30ad-3p
LARP1	hsa-miR-7260-3p
LARP1	hsa-miR-6963-3p
LARP1	hsa-miR-8801
LARP1	hsa-miR-335-3p
LARP1	hsa-miR-2881
LARP1	hsa-miR-1271-5p
LARP1	hsa-miR-510
LARP1	hsa-miR-6599-5p
LARP1	hsa-miR-128-5p
LARP1	hsa-miR-219b-5p
LARP1	hsa-miR-6996-5p
LARP1	hsa-miR-6702-5p
LARP1	hsa-miR-7259-5p
LARP1	hsa-miR-7106-3p
LARP1	hsa-miR-701-5p
LARP1	hsa-miR-4669
LARP1	hsa-miR-7113-5p
LARP1	hsa-miR-1545b-3p
LARP1	hsa-miR-6547-5p
LARP1	hsa-miR-7-5p
LARP1	hsa-miR-7214-5p
LARP1	hsa-let-7afk-3p
LARP1	hsa-miR-3192-5p
LARP1	hsa-miR-7386d-5p
LARP1	hsa-miR-3569-3p
LARP1	hsa-miR-584-3p
LARP1	hsa-miR-1307-3p
LARP1	hsa-miR-574-3p
LARP1	hsa-miR-5089-3p
LARP1	hsa-miR-382-5p
LARP1	hsa-miR-3150a-3p
LARP1	hsa-miR-7109-3p
LARP1	hsa-miR-216c
LARP1	hsa-miR-8106
LARP1	hsa-miR-8813
LARP1	hsa-miR-493-3p

LARP1	hsa-miR-92-5p
LARP1	hsa-miR-34
LARP1	hsa-miR-6983-3p
LARP1	hsa-miR-2308
LARP1	hsa-miR-7398u-3p
LARP1	hsa-miR-1715-5p
LARP1	hsa-miR-344f-5p
LARP1	hsa-miR-326-3p
LARP1	hsa-miR-7004-3p
LARP1	hsa-miR-6613-3p
LARP1	hsa-miR-7669-3p
LARP1	hsa-miR-8823
LARP1	hsa-miR-5680
LARP1	hsa-miR-33a-3p
LARP1	hsa-miR-1193-3p
LARP1	hsa-miR-6823-5p
LARP1	hsa-miR-6333
LARP1	hsa-miR-135b-3p
LARP1	hsa-miR-6772-3p
LARP1	hsa-miR-875-5p
LARP1	hsa-miR-1550-5p
LARP1	hsa-miR-6594-5p
LARP1	hsa-miR-1966-5p
LARP1	hsa-miR-525-5p
LARP1	hsa-miR-4796-3p
LARP1	hsa-miR-4268
LARP1	hsa-miR-632
LARP1	hsa-miR-516b-3p
LARP1	hsa-miR-2285u
LARP1	hsa-miR-135-5p
LARP1	hsa-miR-516-5p
LARP1	hsa-miR-153-3p
LARP1	hsa-miR-7164-5p
LARP1	hsa-miR-2346
LARP1	hsa-let-7-3p
LARP1	hsa-miR-133-3p
LARP1	hsa-miR-1685-5p
LARP1	hsa-miR-7048-5p
LARP1	hsa-miR-684
LARP1	hsa-miR-328-3p
LARP1	hsa-miR-505-5p
LARP1	hsa-miR-7353-5p
LARP1	hsa-miR-6814-5p
LARP1	hsa-miR-1224-3p
LARP1	hsa-miR-133c
LARP1	hsa-miR-373-5p

LARP1	hsa-miR-940
LARP1	hsa-miR-7016-3p
LARP1	hsa-miR-1258
LARP1	hsa-miR-1609
LARP1	hsa-miR-7297-5p
LARP1	hsa-miR-691
LARP1	hsa-miR-4656
LARP1	hsa-miR-1912-5p
LARP1	hsa-miR-3547
LARP1	hsa-miR-2355-3p
LARP1	hsa-miR-147-5p
LARP1	hsa-miR-1972
LARP1	hsa-miR-1225-3p
LARP1	hsa-miR-1739
LARP1	hsa-miR-7094b-2-5p
LARP1	hsa-miR-141-5p
LARP1	hsa-miR-98-5p
LARP1	hsa-miR-6357
LARP1	hsa-miR-3918
LARP1	hsa-miR-7452-5p
LARP1	hsa-miR-6764-5p
LARP1	hsa-miR-3104-5p
LARP1	hsa-miR-624-3p
LARP1	hsa-miR-3177-5p
LARP1	hsa-miR-3616-3p
LARP1	hsa-miR-6971-3p
LARP1	hsa-miR-1677-3p
LARP1	hsa-miR-4699-5p
LARP1	hsa-miR-5589-5p
LARP1	hsa-miR-7378-5p
LARP1	hsa-miR-2415-3p
LARP1	hsa-miR-3594-5p
LARP1	hsa-miR-6910-3p
LARP1	hsa-miR-7662-5p
LARP1	hsa-miR-4647
LARP1	hsa-miR-2896
LARP1	hsa-miR-4533
LARP1	hsa-miR-7265-3p
LARP1	hsa-miR-6509-3p
LARP1	hsa-miR-5132-5p
LARP1	hsa-miR-30c-2-3p
LARP1	hsa-miR-198
LARP1	hsa-miR-575
LARP1	hsa-miR-4680-5p
LARP1	hsa-miR-4759
LARP1	hsa-miR-708-3p

LARP1	hsa-miR-6770-5p
LARP1	hsa-miR-6886-3p
LARP1	hsa-miR-181-5p
LARP1	hsa-miR-7260-5p
LARP1	hsa-miR-194-1-3p
LARP1	hsa-miR-4675
LARP1	hsa-miR-2301
LARP1	hsa-miR-3144-3p
LARP1	hsa-miR-547-5p
LARP1	hsa-miR-6656-5p
LARP1	hsa-miR-7b-3p
LARP1	hsa-miR-8848
LARP1	hsa-miR-7073-3p
LARP1	hsa-miR-6964-3p
LARP1	hsa-miR-6992-5p
LARP1	hsa-miR-496a-3p.2
LARP1	hsa-miR-3926
LARP1	hsa-miR-2404
LARP1	hsa-miR-6972-5p
LARP1	hsa-miR-223-3p
LARP1	hsa-miR-33
LARP1	hsa-miR-7656-3p
LARP1	hsa-miR-1260a
LARP1	hsa-miR-6554-5p
LARP1	hsa-miR-4289
LARP1	hsa-miR-4301
LARP1	hsa-miR-6927-5p
LARP1	hsa-miR-7515
LARP1	hsa-miR-7329-5p
LARP1	hsa-miR-214-3p
LARP1	hsa-miR-4709-5p
LARP1	hsa-miR-485-5p
LARP1	hsa-miR-2395
LARP1	hsa-miR-7111-3p
LARP1	hsa-miR-6719-3p
LARP1	hsa-miR-143-3p
LARP1	hsa-miR-7061-5p
LARP1	hsa-miR-1227-3p
LARP1	hsa-miR-4519
LARP1	hsa-miR-7371f-5p
LARP1	hsa-miR-7356-3p
LARP1	hsa-miR-4645-5p
LARP1	hsa-miR-2459
LARP1	hsa-miR-181d-3p
LARP1	hsa-miR-7163-3p
LARP1	hsa-miR-7653-5p

LARP1	hsa-miR-6769b-5p
LARP1	hsa-miR-646
LARP1	hsa-miR-2114-3p
LARP1	hsa-miR-6574-5p
LARP1	hsa-miR-4687-5p
LARP1	hsa-miR-6817-3p
LARP1	hsa-miR-350
LARP1	hsa-miR-4665-5p
LARP1	hsa-miR-4736
LARP1	hsa-miR-7360-5p
LARP1	hsa-miR-891b
LARP1	hsa-miR-460-3p
LARP1	hsa-miR-3578
LARP1	hsa-miR-204-5p
LARP1	hsa-miR-3547-5p
LARP1	hsa-miR-571
LARP1	hsa-miR-154b
LARP1	hsa-miR-6769a-5p
LARP1	hsa-miR-483-3p.1
LARP1	hsa-miR-6555-5p
LARP1	hsa-miR-7677-5p
LARP1	hsa-miR-466b-2-3p
LARP1	hsa-miR-892-5p
LARP1	hsa-miR-6564-3p
LARP1	hsa-miR-3074-5p
LARP1	hsa-miR-6856-3p
LARP1	hsa-miR-7296-3p
LARP1	hsa-miR-2970-5p
LARP1	hsa-miR-3071-3p
LARP1	hsa-miR-2460
LARP1	hsa-miR-3577-3p
LARP1	hsa-miR-6503-3p
LARP1	hsa-miR-6672-3p
LARP1	hsa-miR-421-5p
LARP1	hsa-miR-3070-2-3p
LARP1	hsa-miR-7288-3p
LARP1	hsa-miR-7055-3p
LARP1	hsa-miR-7083-5p
LARP1	hsa-miR-2475
LARP1	hsa-miR-3119
LARP1	hsa-miR-5692bc
LARP1	hsa-miR-1773-3p
LARP1	hsa-miR-4263
LARP1	hsa-miR-149-3p
LARP1	hsa-miR-6619-5p
LARP1	hsa-miR-194-3p

LARP1	hsa-miR-4774-3p
LARP1	hsa-miR-515-5p
LARP1	hsa-miR-320b
LARP1	hsa-miR-7312-3p
LARP1	hsa-miR-470-5p
LARP1	hsa-miR-8097
LARP1	hsa-miR-3664-5p
LARP1	hsa-miR-6920-3p
LARP1	hsa-miR-4472
LARP1	hsa-miR-375
LARP1	hsa-miR-7032-5p
LARP1	hsa-miR-1545a-3p
LARP1	hsa-miR-2423
LARP1	hsa-miR-342-3p
LARP1	hsa-miR-3534
LARP1	hsa-miR-6079
LARP1	hsa-miR-4459
LARP1	hsa-miR-2373-3p
LARP1	hsa-miR-2433
LARP1	hsa-miR-30d-5p
LARP1	hsa-miR-920
LARP1	hsa-miR-4712-3p
LARP1	hsa-miR-18-3p
LARP1	hsa-miR-3177-3p
LARP1	hsa-miR-6332
LARP1	hsa-miR-139-3p
LARP1	hsa-miR-486-3p
LARP1	hsa-miR-1188-3p
LARP1	hsa-miR-4512
LARP1	hsa-miR-3092-5p
LARP1	hsa-miR-549a
LARP1	hsa-miR-2284aa
LARP1	hsa-miR-4700-3p
LARP1	hsa-miR-6829-5p
LARP1	hsa-miR-1198-3p
LARP1	hsa-miR-6793-3p
LARP1	hsa-miR-1721
LARP1	hsa-miR-293-3p
LARP1	hsa-miR-1969
LARP1	hsa-miR-1545b-3-3p
LARP1	hsa-miR-4723-5p
LARP1	hsa-miR-1292-5p
LARP1	hsa-let-7a-3p
LARP1	hsa-miR-5627-5p
LARP1	hsa-miR-6756-3p
LARP1	hsa-miR-450a-2-3p

LARP1	hsa-miR-1237-3p
LARP1	hsa-miR-5586-3p
LARP1	hsa-miR-371-3p
LARP1	hsa-miR-873-5p.1
LARP1	hsa-miR-502-5p
LARP1	hsa-miR-660-3p
LARP1	hsa-miR-329-3p
LARP1	hsa-miR-7366-5p
LARP1	hsa-miR-4417
LARP1	hsa-miR-1789
LARP1	hsa-miR-7669-5p
LARP1	hsa-miR-1607
LARP1	hsa-miR-22-3p
LARP1	hsa-miR-497-3p
LARP1	hsa-miR-196-3p
LARP1	hsa-miR-2439-5p
LARP1	hsa-miR-7262b-5p
LARP1	hsa-miR-3104-3p
LARP1	hsa-miR-374c-5p
LARP1	hsa-miR-1289
LARP1	hsa-miR-3978
LARP1	hsa-miR-6123
LARP1	hsa-miR-466n-5p
LARP1	hsa-miR-2428
LARP1	hsa-miR-154-5p
LARP1	hsa-miR-510-5p
LARP1	hsa-miR-2438
LARP1	hsa-miR-3120-3p
LARP1	hsa-miR-7386o-3p
LARP1	hsa-miR-1782
LARP1	hsa-miR-503-5p
LARP1	hsa-miR-1207-3p
LARP1	hsa-miR-8836
LARP1	hsa-miR-5004-5p
LARP1	hsa-miR-4721
LARP1	hsa-miR-6858-3p
LARP1	hsa-miR-674-5p
LARP1	hsa-miR-6784-3p
LARP1	hsa-miR-6895-5p
LARP1	hsa-miR-1298-3p
LARP1	hsa-miR-3667-3p
LARP1	hsa-miR-4716
LARP1	hsa-miR-4259
LARP1	hsa-miR-10a-3p
LARP1	hsa-miR-6528
LARP1	hsa-miR-6875-3p

LARP1	hsa-miR-7371a-3p
LARP1	hsa-miR-146-5p
LARP1	hsa-miR-361-3p
LARP1	hsa-miR-6754-3p
LARP1	hsa-miR-190a-3p
LARP1	hsa-miR-455-3p.1
LARP1	hsa-miR-6331
LARP1	hsa-miR-2417
LARP1	hsa-miR-3561-3p
LARP1	hsa-miR-4455
LARP1	hsa-miR-1260a
LARP1	hsa-miR-880-5p
LARP1	hsa-miR-7067-5p
LARP1	hsa-let-7g-3p
LARP1	hsa-miR-2397-3p
LARP1	hsa-miR-4761-5p
LARP1	hsa-miR-5192
LARP1	hsa-miR-5695
LARP1	hsa-miR-4725-3p
LARP1	hsa-miR-328-5p
LARP1	hsa-miR-3162-5p
LARP1	hsa-miR-3189-5p
LARP1	hsa-miR-1292-5p
LARP1	hsa-miR-8819
LARP1	hsa-miR-6824-3p
LARP1	hsa-miR-350-3p
LARP1	hsa-miR-6827-5p
LARP1	hsa-miR-2480
LARP1	hsa-miR-7646-5p
LARP1	hsa-miR-2284x
LARP1	hsa-miR-6787-3p
LARP1	hsa-miR-6975-5p
LARP1	hsa-miR-340-5p
LARP1	hsa-miR-494
LARP1	hsa-miR-30a-5p
LARP1	hsa-miR-4762-3p
LARP1	hsa-miR-7685-5p
LARP1	hsa-miR-5113
LARP1	hsa-miR-6774-5p
LARP1	hsa-miR-3078-3p
LARP1	hsa-miR-455-3p.2
LARP1	hsa-miR-889-5p
LARP1	hsa-miR-7212-5p
LARP1	hsa-miR-7311-5p
LARP1	hsa-miR-323-3p
LARP1	hsa-miR-8886

LARP1	hsa-miR-3942-3p
LARP1	hsa-miR-7254-5p
LARP1	hsa-miR-7005-5p
LARP1	hsa-miR-542-3p
LARP1	hsa-miR-4486
LARP1	hsa-miR-598-5p
LARP1	hsa-miR-3121-5p
LARP1	hsa-miR-330-3p.2
LARP1	hsa-miR-485
LARP1	hsa-miR-153-5p
LARP1	hsa-miR-2451
LARP1	hsa-miR-1946b
LARP1	hsa-miR-5572
LARP1	hsa-miR-101-3p.1
LARP1	hsa-miR-3154
LARP1	hsa-miR-345-5p
LARP1	hsa-miR-761
LARP1	hsa-miR-8876
LARP1	hsa-miR-6911-5p
LARP1	hsa-miR-7272-3p
LARP1	hsa-miR-1296-3p
LARP1	hsa-miR-1644
LARP1	hsa-miR-2374
LARP1	hsa-miR-7256-3p
LARP1	hsa-miR-7647-3p
LARP1	hsa-miR-7315-3p
LARP1	hsa-miR-514a-3p
LARP1	hsa-miR-7683-3p
LARP1	hsa-miR-519e-3p
LARP1	hsa-miR-2381
LARP1	hsa-miR-7252-3p
LARP1	hsa-miR-6754-5p
LARP1	hsa-miR-6368
LARP1	hsa-miR-7090-5p
LARP1	hsa-miR-7198-3p
LARP1	hsa-miR-6700-5p
LARP1	hsa-miR-6597-3p
LARP1	hsa-miR-1843b-5p
LARP1	hsa-miR-4433b-5p
LARP1	hsa-miR-1260b
LARP1	hsa-miR-3191-3p
LARP1	hsa-miR-5101
LARP1	hsa-miR-4481
LARP1	hsa-miR-6733-3p
LARP1	hsa-miR-7291-5p
LARP1	hsa-miR-802

LARP1	hsa-miR-1779
LARP1	hsa-miR-329a
LARP1	hsa-miR-7370-3p
LARP1	hsa-miR-7476-3p
LARP1	hsa-miR-3062-3p
LARP1	hsa-miR-6646-3p
LARP1	hsa-miR-6753-5p
LARP1	hsa-miR-7094-1-5p
LARP1	hsa-miR-7032-3p
LARP1	hsa-miR-10-5p
LARP1	hsa-miR-182-5p
LARP1	hsa-miR-4716-5p
LARP1	hsa-miR-25-3p
LARP1	hsa-miR-2891
LARP1	hsa-miR-26a-5p
LARP1	hsa-miR-512
LARP1	hsa-miR-4422
LARP1	hsa-miR-1540
LARP1	hsa-miR-664b-5p
LARP1	hsa-miR-4659b-3p
LARP1	hsa-miR-3191-5p
LARP1	hsa-miR-3193
LARP1	hsa-miR-7398g-3p
LARP1	hsa-miR-642
LARP1	hsa-miR-3075
LARP1	hsa-miR-661
LARP1	hsa-miR-7083-3p
LARP1	hsa-miR-3473
LARP1	hsa-miR-1197
LARP1	hsa-miR-6785-5p
LARP1	hsa-miR-2117
LARP1	hsa-miR-3151-5p
LARP1	hsa-miR-524-3p
LARP1	hsa-miR-6729-3p
LARP1	hsa-miR-3614-3p
LARP1	hsa-miR-1586
LARP1	hsa-miR-741-3p
LARP1	hsa-miR-4434
LARP1	hsa-miR-24-5p
LARP1	hsa-miR-322-3p
LARP1	hsa-miR-2286
LARP1	hsa-miR-4727-5p
LARP1	hsa-miR-709
LARP1	hsa-let-7f-1-3p
LARP1	hsa-miR-7109-5p
LARP1	hsa-miR-3617-3p

LARP1	hsa-miR-7332-3p
LARP1	hsa-miR-484
LARP1	hsa-miR-1943-3p
LARP1	hsa-miR-7090-3p
LARP1	hsa-miR-6801-5p
LARP1	hsa-miR-7084-3p
LARP1	hsa-miR-1625-5p
LARP1	hsa-miR-16-5p
LARP1	hsa-miR-1231-5p
LARP1	hsa-miR-370-3p
LARP1	hsa-miR-2293
LARP1	hsa-miR-4795-5p
LARP1	hsa-miR-1748
LARP1	hsa-miR-6938-5p
LARP1	hsa-miR-8108
LARP1	hsa-miR-6922-3p
LARP1	hsa-miR-1205
LARP1	hsa-miR-5584-5p
LARP1	hsa-miR-301-3p
LARP1	hsa-miR-23-3p
LARP1	hsa-miR-8887
LARP1	hsa-miR-6819-3p
LARP1	hsa-miR-7210-5p
LARP1	hsa-miR-6575-5p
LARP1	hsa-miR-6840-3p
LARP1	hsa-miR-604
LARP1	hsa-miR-6969-5p
LARP1	hsa-miR-344-3p
LARP1	hsa-miR-541-5p
LARP1	hsa-miR-2486-3p
LARP1	hsa-miR-3664-5p
LARP1	hsa-miR-6549-5p
LARP1	hsa-miR-6864-5p
LARP1	hsa-miR-6824-5p
LARP1	hsa-miR-6662-3p
LARP1	hsa-miR-6950-5p
LARP1	hsa-miR-507b
LARP1	hsa-miR-1896
LARP1	hsa-miR-8061
LARP1	hsa-miR-514-3p
LARP1	hsa-miR-471-3p
LARP1	hsa-miR-581
LARP1	hsa-miR-2376
LARP1	hsa-miR-7353-3p
LARP1	hsa-miR-6950-3p
LARP1	hsa-miR-4714-5p

LARP1	hsa-miR-2285n
LARP1	hsa-miR-7036b-3p
LARP1	hsa-miR-450a-1-3p
LARP1	hsa-miR-23b-5p
LARP1	hsa-miR-4724-3p
LARP1	hsa-miR-197-3p
LARP1	hsa-miR-6782-3p
LARP1	hsa-miR-92
LARP1	hsa-miR-4668-5p
LARP1	hsa-miR-1284
LARP1	hsa-miR-2291
LARP1	hsa-miR-6747-5p
LARP1	hsa-miR-3152-3p
LARP1	hsa-miR-2284ac
LARP1	hsa-miR-3173-3p
LARP1	hsa-miR-1694
LARP1	hsa-miR-539-3p
LARP1	hsa-miR-3093-5p
LARP1	hsa-miR-6830-5p
LARP1	hsa-miR-584-5p
LARP1	hsa-miR-491-5p
LARP1	hsa-miR-3127-5p
LARP1	hsa-miR-7648-3p
LARP1	hsa-miR-3150b-3p
LARP1	hsa-miR-758-3p
LARP1	hsa-miR-3529-5p
LARP1	hsa-miR-212-5p
LARP1	hsa-miR-6883-5p
LARP1	hsa-miR-1646
LARP1	hsa-miR-7188-5p
LARP1	hsa-miR-1636
LARP1	hsa-miR-130-3p
LARP1	hsa-miR-30d-3p
LARP1	hsa-miR-3100-3p
LARP1	hsa-miR-3544-3p
LARP1	hsa-miR-680
LARP1	hsa-miR-1252-5p
LARP1	hsa-miR-125a-3p
LARP1	hsa-miR-501-5p
LARP1	hsa-miR-6742-5p
LARP1	hsa-miR-7665-5p
LARP1	hsa-miR-5584-3p
LARP1	hsa-miR-130b-5p
LARP1	hsa-miR-1298
LARP1	hsa-miR-1306-5p
LARP1	hsa-miR-4687-3p

LARP1	hsa-miR-7002-5p
LARP1	hsa-miR-7113-3p
LARP1	hsa-miR-34b-3p
LARP1	hsa-miR-126-3p
LARP1	hsa-miR-2297
LARP1	hsa-miR-411b
LARP1	hsa-miR-3059-5p
LARP1	hsa-miR-3152-5p
LARP1	hsa-miR-1668
LARP1	hsa-miR-6563-3p
LARP1	hsa-miR-6929-5p
LARP1	hsa-miR-513a-5p
LARP1	hsa-miR-6615-5p
LARP1	hsa-miR-2294
LARP1	hsa-miR-6976-5p
LARP1	hsa-miR-6119-5p
LARP1	hsa-miR-141-3p
LARP1	hsa-miR-1273h-3p
LARP1	hsa-miR-7864
LARP1	hsa-miR-6348
LARP1	hsa-miR-1544-3p
LARP1	hsa-miR-3124-3p
LARP1	hsa-miR-2356
LARP1	hsa-miR-126a-5p
LARP1	hsa-miR-669b-5p
LARP1	hsa-miR-33b-3p
LARP1	hsa-miR-3076-5p
LARP1	hsa-miR-702-5p
LARP1	hsa-miR-3620-5p
LARP1	hsa-miR-7092-5p
LARP1	hsa-miR-3550
LARP1	hsa-miR-1285-3p
LARP1	hsa-miR-3907
LARP1	hsa-miR-483-3p
LARP1	hsa-miR-548ag
LARP1	hsa-miR-7055-5p
LARP1	hsa-miR-875-3p
LARP1	hsa-miR-8792
LARP1	hsa-miR-4426
LARP1	hsa-miR-3062-5p
LARP1	hsa-miR-6764-3p
LARP1	hsa-miR-1837
LARP1	hsa-miR-563
LARP1	hsa-miR-133
LARP1	hsa-miR-6877-3p
LARP1	hsa-miR-1843a-5p

LARP1	hsa-miR-1233-3p
LARP1	hsa-miR-8113
LARP1	hsa-miR-672-3p
LARP1	hsa-miR-3672
LARP1	hsa-miR-3075-5p
LARP1	hsa-miR-1906
LARP1	hsa-miR-7030-5p
LARP1	hsa-miR-4505
LARP1	hsa-miR-548ar-3p
LARP1	hsa-miR-3668
LARP1	hsa-miR-1772-3p
LARP1	hsa-miR-642a-5p
LARP1	hsa-miR-7649-5p
LARP1	hsa-miR-3541
LARP1	hsa-miR-7195-5p
LARP1	hsa-miR-8903
LARP1	hsa-miR-6731-5p
LARP1	hsa-miR-656-3p
LARP1	hsa-miR-5615-5p
LARP1	hsa-miR-615-5p
LARP1	hsa-miR-190-3p
LARP1	hsa-miR-698-5p
LARP1	hsa-miR-3084bc-5p
LARP1	hsa-miR-6650-5p
LARP1	hsa-miR-188-5p
LARP1	hsa-miR-150-5p
LARP1	hsa-miR-409
LARP1	hsa-miR-7157-5p
LARP1	hsa-miR-7398s-5p
LARP1	hsa-miR-2338
LARP1	hsa-miR-6519
LARP1	hsa-miR-7033-5p
LARP1	hsa-miR-6780b-5p
LARP1	hsa-miR-6701-3p
LARP1	hsa-miR-106-5p
LARP1	hsa-miR-1897-3p
LARP1	hsa-miR-6330
LARP1	hsa-miR-584-5p
LARP1	hsa-miR-6914-5p
LARP1	hsa-miR-2300-3p
LARP1	hsa-miR-2310
LARP1	hsa-miR-107-5p
LARP1	hsa-miR-7349-5p
LARP1	hsa-miR-153
LARP1	hsa-miR-599
LARP1	hsa-miR-3688-3p

LARP1	hsa-miR-6782-5p
LARP1	hsa-miR-7034-3p
LARP1	hsa-miR-6559-5p
LARP1	hsa-miR-374a-5p
LARP1	hsa-miR-127-5p
LARP1	hsa-miR-2327
LARP1	hsa-miR-7322-5p
LARP1	hsa-miR-5134-5p
LARP1	hsa-miR-324-3p
LARP1	hsa-miR-362-3p
LARP1	hsa-miR-939-5p
LARP1	hsa-miR-7235-5p
LARP1	hsa-miR-2328-5p
LARP1	hsa-miR-4443
LARP1	hsa-miR-631
LARP1	hsa-miR-6799-5p
LARP1	hsa-miR-29-3p
LARP1	hsa-miR-4446
LARP1	hsa-miR-9b-3p
LARP1	hsa-miR-8899
LARP1	hsa-miR-4720-5p
LARP1	hsa-miR-6776-3p
LARP1	hsa-miR-7292-5p
LARP1	hsa-miR-6942-5p
LARP1	hsa-miR-6897-3p
LARP1	hsa-miR-489
LARP1	hsa-miR-141-3p
LARP1	hsa-miR-136-3p
LARP1	hsa-miR-1285-5p
LARP1	hsa-miR-449b-3p
LARP1	hsa-miR-1584-5p
LARP1	hsa-miR-5003-5p
LARP1	hsa-miR-7116-3p
LARP1	hsa-miR-1640
LARP1	hsa-miR-7049-5p
LARP1	hsa-miR-384
LARP1	hsa-miR-6086
LARP1	hsa-miR-7169-5p
LARP1	hsa-miR-3162-3p
LARP1	hsa-miR-200a-3p
LARP1	hsa-miR-1224
LARP1	hsa-miR-6690-5p
LARP1	hsa-miR-101-3p.2
LARP1	hsa-miR-7304-3p
LARP1	hsa-miR-3551-3p
LARP1	hsa-miR-5191

LARP1	hsa-miR-548g-3p
LARP1	hsa-miR-6715-3p
LARP1	hsa-miR-4494
LARP1	hsa-miR-7378-3p
LARP1	hsa-miR-365-3p
LARP1	hsa-miR-8082
LARP1	hsa-miR-5110
LARP1	hsa-miR-4311
LARP1	hsa-miR-7075-5p
LARP1	hsa-miR-7844-5p
LARP1	hsa-miR-3557-3p
LARP1	hsa-miR-296-5p
LARP1	hsa-miR-3102-5p
LARP1	hsa-miR-6620-5p
LARP1	hsa-miR-1306-3p
LARP1	hsa-miR-503-3p
LARP1	hsa-miR-1254
LARP1	hsa-miR-1803
LARP1	hsa-miR-7226-5p
LARP1	hsa-miR-3127-5p
LARP1	hsa-miR-3920
LARP1	hsa-miR-26-1-3p
LARP1	hsa-miR-1656
LARP1	hsa-miR-1788-5p
LARP1	hsa-miR-6768-5p
LARP1	hsa-miR-692
LARP1	hsa-miR-374b-5p
LARP1	hsa-miR-669m-5p
LARP1	hsa-miR-3618-5p
LARP1	hsa-miR-211-5p
LARP1	hsa-miR-140-3p
LARP1	hsa-miR-6739-5p
LARP1	hsa-miR-1546-5p
LARP1	hsa-miR-377-5p
LARP1	hsa-miR-532-3p
LARP1	hsa-miR-7045-3p
LARP1	hsa-miR-7293-5p
LARP1	hsa-miR-7862
LARP1	hsa-miR-5112
LARP1	hsa-miR-4685-5p
LARP1	hsa-miR-1695
LARP1	hsa-miR-2467-5p
LARP1	hsa-miR-200-3p
LARP1	hsa-miR-6998-5p
LARP1	hsa-miR-3964
LARP1	hsa-miR-299-3p

LARP1	hsa-miR-1729-5p
LARP1	hsa-miR-101-3p
LARP1	hsa-miR-3175
LARP1	hsa-miR-2127
LARP1	hsa-miR-2382-5p
LARP1	hsa-miR-3572-5p
LARP1	hsa-miR-1603
LARP1	hsa-miR-3102-5p.2-5p
LARP1	hsa-miR-342-5p
LARP1	hsa-miR-934-3p
LARP1	hsa-miR-7166-5p
LARP1	hsa-miR-4271
LARP1	hsa-miR-1287-5p
LARP1	hsa-miR-32-5p
LARP1	hsa-miR-7316-3p
LARP1	hsa-miR-3551-5p
LARP1	hsa-miR-4731-5p
LARP1	hsa-miR-4650
LARP1	hsa-miR-6848-3p
LARP1	hsa-miR-6747-3p
LARP1	hsa-miR-326-5p
LARP1	hsa-miR-6899-3p
LARP1	hsa-miR-760
LARP1	hsa-miR-669c-3p
LARP1	hsa-miR-151a-5p
LARP1	hsa-miR-291b-3p
LARP1	hsa-miR-3158-5p
LARP1	hsa-miR-2136
LARP1	hsa-miR-4716-3p
LARP1	hsa-miR-376b-5p
LARP1	hsa-miR-540-3p
LARP1	hsa-miR-7162-5p
LARP1	hsa-miR-1686
LARP1	hsa-miR-3127-3p
LARP1	hsa-miR-7445-3p
LARP1	hsa-miR-3149
LARP1	hsa-miR-5583-3p
LARP1	hsa-miR-129b-5p
LARP1	hsa-miR-7091-3p
LARP1	hsa-miR-6985-5p
LARP1	hsa-miR-6581-3p
LARP1	hsa-miR-7215-3p
LARP1	hsa-miR-7058-5p
LARP1	hsa-miR-2454-5p
LARP1	hsa-miR-466n-3p
LARP1	hsa-miR-570-5p

LARP1	hsa-miR-7210-3p
LARP1	hsa-miR-548e-5p
LARP1	hsa-miR-6923-5p
LARP1	hsa-miR-1845
LARP1	hsa-miR-4760-3p
LARP1	hsa-miR-5194
LARP1	hsa-miR-7025-3p
LARP1	hsa-miR-6801-3p
LARP1	hsa-miR-5582-5p
LARP1	hsa-miR-6756-5p
LARP1	hsa-miR-6775-3p
LARP1	hsa-miR-146b-3p
LARP1	hsa-miR-10b-3p
LARP1	hsa-miR-302c-5p
LARP1	hsa-miR-3086-3p
LARP1	hsa-miR-2887
LARP1	hsa-miR-2337
LARP1	hsa-miR-323a-5p
LARP1	hsa-miR-8896
LARP1	hsa-miR-7309-5p
LARP1	hsa-miR-3612
LARP1	hsa-miR-219-1-3p
LARP1	hsa-miR-7033-3p
LARP1	hsa-miR-1227-5p
LARP1	hsa-miR-1962
LARP1	hsa-miR-654-3p
LARP1	hsa-miR-6566-3p
LARP1	hsa-miR-6733-5p
LARP1	hsa-miR-7106-5p
LARP1	hsa-miR-298
LARP1	hsa-miR-34a-3p
LARP1	hsa-miR-1343-3p
LARP1	hsa-miR-129b-3p
LARP1	hsa-miR-7011-5p
LARP1	hsa-miR-1543
LARP1	hsa-miR-466f-3p
LARP1	hsa-miR-767
LARP1	hsa-miR-1956-5p
LARP1	hsa-miR-519d-5p
LARP1	hsa-miR-7682-3p
LARP1	hsa-miR-3431
LARP1	hsa-miR-7386f-3p
LARP1	hsa-miR-1563
LARP1	hsa-miR-3202
LARP1	hsa-miR-3185
LARP1	hsa-miR-7373b-3p

LARP1	hsa-miR-4694-5p
LARP1	hsa-miR-7327-5p
LARP1	hsa-miR-5196-5p
LARP1	hsa-miR-6734-3p
LARP1	hsa-miR-26b-5p
LARP1	hsa-miR-7217-3p
LARP1	hsa-miR-2110
LARP1	hsa-miR-547-3p
LARP1	hsa-miR-3144-5p
LARP1	hsa-miR-3573-5p
LARP1	hsa-miR-30-3p
LARP1	hsa-miR-7226-3p
LARP1	hsa-miR-3151
LARP1	hsa-miR-7063-5p
LARP1	hsa-miR-6595-5p
LARP1	hsa-miR-3474
LARP1	hsa-miR-3652
LARP1	hsa-miR-4531
LARP1	hsa-miR-1245b-3p
LARP1	hsa-miR-27-3p
LARP1	hsa-miR-101b-3p.2
LARP1	hsa-miR-4769-3p
LARP1	hsa-miR-3179
LARP1	hsa-miR-4515
LARP1	hsa-miR-15a-3p
LARP1	hsa-miR-1771
LARP1	hsa-miR-4651
LARP1	hsa-miR-4482-5p
LARP1	hsa-miR-3616-5p
LARP1	hsa-miR-1964-5p
LARP1	hsa-miR-6792-5p
LARP1	hsa-miR-2339
LARP1	hsa-miR-8104
LARP1	hsa-miR-1753
LARP1	hsa-miR-6520
LARP1	hsa-miR-7398b-3p
LARP1	hsa-miR-5108
LARP1	hsa-miR-4677-3p
LARP1	hsa-miR-7112-5p
LARP1	hsa-miR-512-3p
LARP1	hsa-miR-657
LARP1	hsa-miR-4760-5p
LARP1	hsa-miR-200ac-5p
LARP1	hsa-miR-532-5p
LARP1	hsa-miR-4283
LARP1	hsa-miR-3588

LARP1	hsa-miR-625-5p
LARP1	hsa-miR-224-5p
LARP1	hsa-miR-1811
LARP1	hsa-miR-30
LARP1	hsa-miR-6758-5p
LARP1	hsa-miR-2392
LARP1	hsa-miR-5107-5p
LARP1	hsa-miR-1913
LARP1	hsa-miR-2957
LARP1	hsa-miR-6643-5p
LARP1	hsa-miR-2343
LARP1	hsa-miR-4764-3p
LARP1	hsa-miR-6870-3p
LARP1	hsa-miR-1602
LARP1	hsa-miR-3549
LARP1	hsa-miR-593-5p
LARP1	hsa-miR-6913-5p
LARP1	hsa-miR-1574-3p
LARP1	hsa-miR-7296-5p
LARP1	hsa-miR-3113-3p
LARP1	hsa-miR-3106-3p
LARP1	hsa-miR-7326-3p
LARP1	hsa-miR-2470
LARP1	hsa-miR-483-3p.2
LARP1	hsa-miR-1544b-5p
LARP1	hsa-miR-4524a-5p
LARP1	hsa-miR-6761-5p
LARP1	hsa-miR-641
LARP1	hsa-miR-1744-3p
LARP1	hsa-miR-7162-3p
LARP1	hsa-miR-2897
LARP1	hsa-miR-410-3p
LARP1	hsa-miR-30c-1-3p
LARP1	hsa-miR-1805-3p
LARP1	hsa-miR-6715b-3p
LARP1	hsa-miR-7007-3p
LARP1	hsa-miR-1825
LARP1	hsa-miR-5627-3p
LARP1	hsa-miR-1968-5p
LARP1	hsa-miR-6529b
LARP1	hsa-miR-1207
LARP1	hsa-miR-3087-5p
LARP1	hsa-miR-5000-3p
LARP1	hsa-miR-4252
LARP1	hsa-miR-7262a-5p
LARP1	hsa-miR-6945-5p

LARP1	hsa-miR-2284z
LARP1	hsa-miR-23-5p
LARP1	hsa-miR-6323
LARP1	hsa-miR-2285j
LARP1	hsa-miR-412-5p
LARP1	hsa-miR-7398i-3p
LARP1	hsa-miR-7054-3p
LARP1	hsa-miR-7258-5p
LARP1	hsa-miR-520-5p
LARP1	hsa-miR-466ade-3p
LARP1	hsa-miR-6085
LARP1	hsa-miR-6631-5p
LARP1	hsa-miR-6550-3p
LARP1	hsa-miR-5009-3p
LARP1	hsa-miR-7079-5p
LARP1	hsa-miR-6740-3p
LARP1	hsa-miR-5697-3p
LARP1	hsa-miR-765
LARP1	hsa-miR-7231-5p
LARP1	hsa-miR-642b-5p
LARP1	hsa-miR-4786-5p
LARP1	hsa-miR-4690-3p
LARP1	hsa-miR-384-5p
LARP1	hsa-miR-1249-5p
LARP1	hsa-miR-1540-3p
LARP1	hsa-miR-3145-3p
LARP1	hsa-miR-331-3p
LARP1	hsa-miR-5581-5p
LARP1	hsa-miR-6794-3p
LARP1	hsa-miR-6955-3p
LARP1	hsa-miR-7271-3p
LARP1	hsa-miR-3602
LARP1	hsa-miR-2326
LARP1	hsa-miR-383-5p
LARP1	hsa-miR-375-3p
LARP1	hsa-miR-3571
LARP1	hsa-miR-6973a-3p
LARP1	hsa-miR-7156-3p
LARP1	hsa-miR-3929
LARP1	hsa-miR-651-3p
LARP1	hsa-miR-8485
LARP1	hsa-miR-6926-3p
LARP1	hsa-miR-7163-5p
LARP1	hsa-miR-1452
LARP1	hsa-miR-512-5p
LARP1	hsa-miR-103-3p

LARP1	hsa-miR-8794
LARP1	hsa-miR-7298-5p
LARP1	hsa-miR-200a-3p
LARP1	hsa-miR-519e-3p
LARP1	hsa-miR-345-3p
LARP1	hsa-miR-5787
LARP1	hsa-miR-7678-3p
LARP1	hsa-miR-17-5p
LARP1	hsa-miR-150-3p
LARP1	hsa-miR-1291
LARP1	hsa-miR-8060
LARP1	hsa-miR-5684
LARP1	hsa-miR-7041-5p
LARP1	hsa-miR-466q
LARP1	hsa-miR-4300
LARP1	hsa-miR-4755-5p
LARP1	hsa-miR-412-3p
LARP1	hsa-miR-7364-3p
LARP1	hsa-miR-4739
LARP1	hsa-miR-5133
LARP1	hsa-miR-1892
LARP1	hsa-miR-7249-3p
LARP1	hsa-miR-7456-3p
LARP1	hsa-miR-1294
LARP1	hsa-miR-92a-2-5p
LARP1	hsa-let-7-5p
LARP1	hsa-miR-655-5p
LARP1	hsa-miR-1185-5p
LARP1	hsa-miR-1239
LARP1	hsa-miR-922
LARP1	hsa-miR-6966-3p
LARP1	hsa-miR-3192
LARP1	hsa-miR-5195-5p
LARP1	hsa-miR-1706
LARP1	hsa-miR-7277-5p
LARP1	hsa-miR-6667-5p
LARP1	hsa-miR-1470
LARP1	hsa-miR-3916
LARP1	hsa-miR-7086-5p
LARP1	hsa-miR-6752-3p
LARP1	hsa-miR-4667
LARP1	hsa-miR-4728-5p
LARP1	hsa-miR-515
LARP1	hsa-miR-6979-5p
LARP1	hsa-miR-7073-5p
LARP1	hsa-miR-879-5p

LARP1	hsa-miR-2373-5p
LARP1	hsa-miR-449c-3p
LARP1	hsa-miR-6345
LARP1	hsa-miR-6803-5p
LARP1	hsa-miR-8854
LARP1	hsa-miR-6511-5p
LARP1	hsa-miR-1912-3p
LARP1	hsa-miR-6778-5p
LARP1	hsa-miR-7008-5p
LARP1	hsa-miR-885-3p
LARP1	hsa-miR-3199
LARP1	hsa-miR-7479-3p
LARP1	hsa-miR-340-5p
LARP1	hsa-miR-1632-5p
LARP1	hsa-miR-6562-5p
LARP1	hsa-miR-6865-3p
LARP1	hsa-miR-3093-3p
LARP1	hsa-miR-2334
LARP1	hsa-miR-514a-5p
LARP1	hsa-miR-7386m-3p
LARP1	hsa-miR-188-5p
LARP1	hsa-miR-1669
LARP1	hsa-miR-606
LARP1	hsa-miR-6783-5p
LARP1	hsa-miR-1247-3p
LARP1	hsa-miR-8906
LARP1	hsa-miR-1599
LARP1	hsa-miR-4732-3p
LARP1	hsa-miR-7384-3p
LARP1	hsa-miR-6901-3p
LARP1	hsa-miR-516-3p
LARP1	hsa-miR-8908-5p
LARP1	hsa-miR-568
LARP1	hsa-miR-8898
LARP1	hsa-miR-4646-3p
LARP1	hsa-miR-6730-3p
LARP1	hsa-miR-30b-3p
LARP1	hsa-miR-7859
LARP1	hsa-miR-3200-5p
LARP1	hsa-miR-3064-5p
LARP1	hsa-miR-365a-5p
LARP1	hsa-miR-873-5p.2
LARP1	hsa-miR-4693-3p
LARP1	hsa-miR-7021-5p
LARP1	hsa-miR-135
LARP1	hsa-miR-6940-5p

LARP1	hsa-miR-6924-5p
LARP1	hsa-miR-3535
LARP1	hsa-miR-670-5p
LARP1	hsa-miR-7381-3p
LARP1	hsa-miR-3064
LARP1	hsa-miR-573
LARP1	hsa-miR-3158-3p
LARP1	hsa-miR-129-5p
LARP1	hsa-miR-7358-5p
LARP1	hsa-miR-4661-5p
LARP1	hsa-miR-6946-3p
LARP1	hsa-miR-6944-3p
LARP1	hsa-miR-1933-3p
LARP1	hsa-miR-1754-3p
LARP1	hsa-miR-4524a-3p
LARP1	hsa-miR-7665-3p
LARP1	hsa-miR-3192-3p
LARP1	hsa-miR-6794-5p
LARP1	hsa-miR-6769b-3p
LARP1	hsa-miR-3971
LARP1	hsa-miR-2188-5p
LARP1	hsa-miR-365-5p
LARP1	hsa-miR-1288-5p
LARP1	hsa-miR-7681-5p
LARP1	hsa-miR-1933-5p
LARP1	hsa-miR-7267-5p
LARP1	hsa-miR-3082-5p
LARP1	hsa-miR-6989-5p
LARP1	hsa-miR-7052-3p
LARP1	hsa-miR-6997-5p
LARP1	hsa-miR-7018-5p
LARP1	hsa-miR-4274
LARP1	hsa-miR-1910-5p
LARP1	hsa-miR-383-5p.2
LARP1	hsa-miR-221-3p
LARP1	hsa-miR-7152-5p
LARP1	hsa-miR-6549-3p
LARP1	hsa-miR-3691-5p
LARP1	hsa-miR-6870-5p
LARP1	hsa-miR-764-5p
LARP1	hsa-miR-4673
LARP1	hsa-miR-550-3p
LARP1	hsa-miR-717
LARP1	hsa-miR-6887-5p
LARP1	hsa-miR-373-3p
LARP1	hsa-miR-744-3p

LARP1	hsa-miR-124-5p
LARP1	hsa-miR-7028-5p
LARP1	hsa-miR-6565-5p
LARP1	hsa-miR-31-5p
LARP1	hsa-miR-490-3p
LARP1	hsa-miR-377-3p
LARP1	hsa-miR-8897
LARP1	hsa-miR-195-5p
LARP1	hsa-miR-128-3p
LARP1	hsa-miR-544-5p
LARP1	hsa-miR-548c-3p
LARP1	hsa-miR-597
LARP1	hsa-miR-679
LARP1	hsa-miR-6608-3p
LARP1	hsa-miR-652-5p
LARP1	hsa-miR-1286
LARP1	hsa-miR-2900
LARP1	hsa-miR-4679
LARP1	hsa-miR-101b-3p.1
LARP1	hsa-miR-490-5p
LARP1	hsa-miR-6407
LARP1	hsa-miR-7976
LARP1	hsa-miR-125b-1-3p
LARP1	hsa-miR-3173-3p
LARP1	hsa-miR-3109-3p
LARP1	hsa-miR-4639-3p
LARP1	hsa-miR-2320-5p
LARP1	hsa-miR-6715-5p
LARP1	hsa-miR-4733-3p
LARP1	hsa-miR-485-3p
LARP1	hsa-miR-8807
LARP1	hsa-miR-2188-3p
LARP1	hsa-miR-885-5p
LARP1	hsa-miR-4650-5p
LARP1	hsa-miR-612
LARP1	hsa-miR-30b-5p
LARP1	hsa-miR-548f-3p
LARP1	hsa-miR-455-5p
LARP1	hsa-miR-9500
LARP1	hsa-miR-7438-5p
LARP1	hsa-miR-1749-3p
LARP1	hsa-miR-4795-3p
LARP1	hsa-miR-6859-5p
LARP1	hsa-miR-4700-5p
LARP1	hsa-miR-3090-5p
LARP1	hsa-miR-5197-5p

LARP1	hsa-miR-7456-5p
LARP1	hsa-miR-516a-3p
LARP1	hsa-miR-9769-3p
LARP1	hsa-miR-551
LARP1	hsa-miR-3113-5p
LARP1	hsa-miR-2387
LARP1	hsa-miR-7275-5p
LARP1	hsa-miR-3621
LARP1	hsa-miR-7370i-3p
LARP1	hsa-miR-6757-5p
LARP1	hsa-miR-7377-3p
LARP1	hsa-miR-8853
LARP1	hsa-miR-138-5p
LARP1	hsa-miR-1273f
LARP1	hsa-miR-7218-5p
LARP1	hsa-miR-6780b-3p
LARP1	hsa-miR-7674-3p
LARP1	hsa-miR-496b
LARP1	hsa-miR-301-5p
LARP1	hsa-miR-6847-5p
LARP1	hsa-miR-335-5p
LARP1	hsa-miR-5132-3p
LARP1	hsa-miR-1606
LARP1	hsa-miR-6856-5p
LARP1	hsa-miR-212-3p
LARP1	hsa-miR-3472
LARP1	hsa-miR-7282-5p
LARP1	hsa-miR-134-3p
LARP1	hsa-miR-2861
LARP1	hsa-miR-7362-3p
LARP1	hsa-miR-331-3p
LARP1	hsa-miR-125
LARP1	hsa-miR-4478
LARP1	hsa-miR-6836-3p
LARP1	hsa-miR-7238-3p
LARP1	hsa-miR-30c-5p
LARP1	hsa-miR-4463
LARP1	hsa-miR-1941-5p
LARP1	hsa-miR-4776-5p
LARP1	hsa-miR-8846
LARP1	hsa-miR-433-5p
LARP1	hsa-miR-21-5p
LARP1	hsa-miR-6388
LARP1	hsa-miR-6960-5p
LARP1	hsa-miR-8842
LARP1	hsa-miR-7679-5p

LARP1	hsa-miR-1a-5p
LARP1	hsa-miR-4674
LARP1	hsa-miR-942-5p
LARP1	hsa-miR-6607-5p
LARP1	hsa-miR-192-3p
LARP1	hsa-miR-7364-5p
LARP1	hsa-miR-3561-5p
LARP1	hsa-miR-4708-5p
LARP1	hsa-miR-611
LARP1	hsa-miR-6982-5p
LARP1	hsa-miR-218-1-3p
LARP1	hsa-miR-330-5p
LARP1	hsa-miR-697
LARP1	hsa-miR-2306
LARP1	hsa-miR-374-5p
LARP1	hsa-miR-665-5p
LARP1	hsa-miR-3069-5p
LARP1	hsa-miR-564
LARP1	hsa-miR-3473be
LARP1	hsa-miR-6994-5p
LARP1	hsa-miR-3924
LARP1	hsa-miR-2461-3p
LARP1	hsa-miR-7197-3p
LARP1	hsa-miR-7027-5p
LARP1	hsa-miR-522-3p
LARP1	hsa-miR-1682
LARP1	hsa-miR-1276
LARP1	hsa-miR-1648-5p
LARP1	hsa-miR-1226-3p
LARP1	hsa-miR-6090
LARP1	hsa-miR-4715-5p
LARP1	hsa-miR-7379-3p
LARP1	hsa-miR-6904-5p
LARP1	hsa-miR-6517
LARP1	hsa-miR-674-3p
LARP1	hsa-miR-4761-3p
LARP1	hsa-miR-7229-3p
LARP1	hsa-miR-7386k-3p
LARP1	hsa-miR-7385e-3p
LARP1	hsa-miR-7-5p
LARP1	hsa-miR-2485
LARP1	hsa-miR-9769-5p
LARP1	hsa-miR-3604
LARP1	hsa-miR-452-5p
LARP1	hsa-miR-4652-5p
LARP1	hsa-miR-6772-5p

LARP1	hsa-miR-6943-5p
LARP1	hsa-miR-7037-5p
LARP1	hsa-miR-183-5p
LARP1	hsa-miR-7468-3p
LSM1	hsa-miR-1252-5p
LSM1	hsa-miR-607
LSM1	hsa-miR-3556a
LSM1	hsa-miR-7328-5p
LSM1	hsa-miR-139-5p
LSM1	hsa-miR-7204-3p
LSM1	hsa-miR-4684-3p
LSM1	hsa-miR-219-5p
LSM1	hsa-miR-2451
LSM1	hsa-miR-340-5p
LSM1	hsa-miR-4791
LSM1	hsa-miR-6818-5p
LSM1	hsa-miR-4735-5p
LSM1	hsa-miR-30a-3p
LSM1	hsa-miR-1902
LSM1	hsa-miR-6513-5p
LSM1	hsa-miR-489-3p
LSM1	hsa-miR-548ah-5p
LSM1	hsa-miR-133a-5p
LSM1	hsa-miR-3064-3p
LSM1	hsa-miR-4677-3p
LSM1	hsa-miR-27b-5p
LSM1	hsa-miR-622
LSM1	hsa-miR-7341-5p
LSM1	hsa-miR-8868
LSM1	hsa-miR-7319-5p
LSM1	hsa-miR-411-3p
LSM1	hsa-miR-6794-3p
LSM1	hsa-miR-2320-3p
LSM1	hsa-miR-5621-5p
LSM1	hsa-miR-892-3p
LSM1	hsa-miR-1-2-5p
LSM1	hsa-miR-2284-3p
LSM1	hsa-miR-3164
LSM1	hsa-miR-4477a
LSM1	hsa-miR-548z
LSM1	hsa-miR-183-5p
LSM1	hsa-miR-181b-2-3p
LSM1	hsa-miR-669d-5p
LSM1	hsa-miR-548ac
LSM1	hsa-miR-5003-3p
LSM1	hsa-miR-4679

LSM1	hsa-miR-8876
LSM1	hsa-miR-7678-5p
LSM1	hsa-miR-548az-5p
LSM1	hsa-miR-743a-3p
LSM1	hsa-miR-570-3p
LSM1	hsa-miR-384-5p
LSM1	hsa-miR-141-3p
LSM1	hsa-miR-7039-5p
LSM1	hsa-miR-3913-5p
LSM1	hsa-miR-335-5p
LSM1	hsa-miR-2307
LSM1	hsa-miR-15c-3p
LSM1	hsa-miR-8870
LSM1	hsa-miR-452-5p
LSM1	hsa-miR-2467-5p
LSM1	hsa-miR-6999-3p
LSM1	hsa-miR-450b-3p
LSM1	hsa-let-7f-1-3p
LSM1	hsa-miR-1-1-5p
LSM1	hsa-miR-140-5p
LSM1	hsa-miR-2317
LSM1	hsa-miR-34bc-3p
LSM1	hsa-miR-1542-5p
LSM1	hsa-miR-4294
LSM1	hsa-miR-2294
LSM1	hsa-miR-3471
LSM1	hsa-miR-3470a
LSM1	hsa-miR-5706
LSM1	hsa-miR-548c-3p
LSM1	hsa-miR-6973b-3p
LSM1	hsa-miR-7041-5p
LSM1	hsa-miR-6980-3p
LSM1	hsa-miR-7026-5p
LSM1	hsa-miR-3609
LSM1	hsa-miR-380-3p
LSM1	hsa-miR-219c-3p
LSM1	hsa-miR-3201
LSM1	hsa-miR-548c-3p
LSM1	hsa-miR-6815-5p
LSM1	hsa-miR-6856-3p
LSM1	hsa-miR-105-5p
LSM1	hsa-miR-200a-3p
LSM1	hsa-miR-2395
LSM1	hsa-miR-4703-3p
LSM1	hsa-miR-128
LSM1	hsa-miR-186-5p

LSM1	hsa-miR-450b
LSM1	hsa-miR-3059-3p
LSM1	hsa-miR-2367-5p
LSM1	hsa-miR-3573-5p
LSM1	hsa-miR-4444
LSM1	hsa-miR-542-3p
LSM1	hsa-miR-548d-3p
LSM1	hsa-miR-1265
LSM1	hsa-miR-5089-5p
LSM1	hsa-miR-4699-3p
LSM1	hsa-miR-618
LSM1	hsa-miR-7083-3p
LSM1	hsa-miR-3084bc-5p
LSM1	hsa-miR-7685-3p
LSM1	hsa-miR-3577-3p
LSM1	hsa-miR-7671-5p
LSM1	hsa-miR-339-5p
LSM1	hsa-miR-1208
LSM1	hsa-miR-680
LSM1	hsa-miR-548as-3p
LSM1	hsa-miR-2388-5p
LSM1	hsa-miR-7031-5p
LSM1	hsa-miR-3185
LSM1	hsa-miR-5692a
LSM1	hsa-miR-125a-3p
LSM1	hsa-miR-7186-3p
LSM1	hsa-miR-340-5p
LSM1	hsa-miR-743b-3p
LSM1	hsa-miR-6970-3p
LSM1	hsa-miR-7319-3p
LSM1	hsa-miR-7854-3p
LSM1	hsa-miR-542
LSM1	hsa-miR-2383
LSM1	hsa-miR-30e
LSM1	hsa-miR-5186
LSM1	hsa-miR-1251-5p
LSM1	hsa-miR-7235-5p
LSM1	hsa-miR-153-3p
LSM1	hsa-miR-7371b-5p
LSM1	hsa-miR-1227-3p
LSM1	hsa-miR-338-5p
LSM1	hsa-miR-7389-3p
LSM1	hsa-miR-186-5p
LSM1	hsa-miR-3133
LSM1	hsa-miR-140-3p.1
LSM1	hsa-miR-105-5p

LSM1	hsa-miR-7360-3p
LSM1	hsa-miR-1323-5p
LSM1	hsa-miR-5108
LSM1	hsa-miR-362-5p
LSM1	hsa-miR-153
LSM1	hsa-miR-1243-5p
LSM1	hsa-miR-92a-1-5p
LSM1	hsa-miR-15b-3p
LSM1	hsa-miR-668-3p
LSM1	hsa-miR-676-5p
LSM1	hsa-miR-371
LSM1	hsa-miR-300-5p
LSM1	hsa-miR-302c-3p
LSM1	hsa-miR-1898
LSM1	hsa-miR-6875-3p
LSM1	hsa-miR-1294
LSM1	hsa-miR-1252
LSM1	hsa-miR-3138
LSM1	hsa-miR-8843
LSM1	hsa-miR-5616-3p
LSM1	hsa-miR-466aep-5p
LSM1	hsa-miR-6516-3p
LSM1	hsa-miR-7668-5p
LSM1	hsa-miR-8831
LSM1	hsa-miR-3613b
LSM1	hsa-miR-7063-3p
LSM1	hsa-miR-133b-5p
LSM1	hsa-miR-138-5p
LSM1	hsa-miR-103-2-5p
LSM1	hsa-miR-302b-5p
LSM1	hsa-miR-4276
LSM1	hsa-miR-335-5p
LSM1	hsa-miR-30d-3p
LSM1	hsa-miR-29c-5p
LSM1	hsa-miR-4782-5p
LSM1	hsa-miR-30e-3p
LSM1	hsa-miR-7094-3p
LSM1	hsa-miR-367-3p
LSM1	hsa-miR-7325-3p
LSM1	hsa-miR-212-3p
LSM1	hsa-miR-132-3p
LSM1	hsa-miR-7324-3p
LSM1	hsa-miR-7387-5p
LSM1	hsa-miR-452-5p
LSM1	hsa-miR-425
LSM1	hsa-miR-548t-5p

LSM1	hsa-miR-511-3p
LSM1	hsa-miR-5697-5p
LSM1	hsa-miR-1934-5p
LSM1	hsa-miR-128-3p
LSM1	hsa-miR-19b-5p
LSM1	hsa-miR-540-3p
LSM1	hsa-miR-3607-3p
LSM1	hsa-miR-1193-3p
LSM1	hsa-miR-30-3p
LSM1	hsa-miR-5190
LSM1	hsa-miR-7344-5p
LSM1	hsa-miR-548q
LSM1	hsa-miR-3646
LSM1	hsa-miR-548n
LSM1	hsa-miR-29a-1-5p
LSM1	hsa-miR-1252-3p
LSM1	hsa-miR-331-3p
LSM1	hsa-miR-203-3p.2
LSM1	hsa-miR-3103-5p
LSM1	hsa-miR-1244
LSM1	hsa-miR-514-3p
LSM1	hsa-miR-3100-3p
LSM1	hsa-miR-1297
LSM1	hsa-miR-6946-3p
LSM1	hsa-miR-1291
LSM1	hsa-miR-668
LSM1	hsa-miR-3065-3p
LSM1	hsa-miR-2277-3p
LSM1	hsa-miR-3062-5p
LSM1	hsa-miR-450a-1-3p
LSM1	hsa-miR-2486-5p
LSM1	hsa-miR-7292-5p
LSM1	hsa-miR-1305
LSM1	hsa-miR-383
LSM1	hsa-miR-4659a-3p
LSM1	hsa-miR-7271-3p
LSM1	hsa-miR-6515-3p
LSM1	hsa-miR-1943-5p
LSM1	hsa-miR-466l-5p
LSM1	hsa-miR-769-3p
LSM1	hsa-miR-6882-5p
LSM1	hsa-miR-5007-3p
LSM1	hsa-miR-7349-3p
LSM1	hsa-miR-8055
LSM1	hsa-miR-470-5p
LSM1	hsa-miR-8900

LSM1	hsa-miR-5624-5p
LSM1	hsa-miR-210-5p
LSM1	hsa-miR-3117-5p
LSM1	hsa-miR-302d-5p
LSM1	hsa-miR-30-5p
LSM1	hsa-miR-7047-5p
LSM1	hsa-miR-7674-5p
LSM1	hsa-miR-7354-5p
LSM1	hsa-miR-4659b-3p
LSM1	hsa-miR-7649-5p
LSM1	hsa-miR-7190-5p
LSM1	hsa-miR-548bb-3p
LSM1	hsa-miR-466n-5p
LSM1	hsa-miR-425-5p
LSM1	hsa-miR-8062
LSM1	hsa-miR-1224-3p
LSM1	hsa-miR-7205-3p
LSM1	hsa-miR-2309
LSM1	hsa-miR-6728-5p
LSM1	hsa-miR-105b
LSM1	hsa-miR-2293
LSM1	hsa-miR-301-5p
LSM1	hsa-miR-2329-3p
LSM1	hsa-miR-206
LSM1	hsa-miR-7168-5p
LSM1	hsa-miR-887-5p
LSM1	hsa-miR-181-5p
LSM1	hsa-miR-1323
LSM1	hsa-miR-31-3p
LSM1	hsa-miR-4435
LSM1	hsa-miR-7374a-3p
LSM1	hsa-miR-2458
LSM1	hsa-miR-138-5p
LSM1	hsa-miR-1-3p
LSM1	hsa-miR-4252
LSM1	hsa-miR-758-3p
LSM1	hsa-miR-6131
LSM1	hsa-miR-6865-5p
LSM1	hsa-miR-495-3p
LSM1	hsa-miR-2285x
LSM1	hsa-miR-448
LSM1	hsa-miR-153-5p
LSM1	hsa-miR-6965-5p
LSM1	hsa-miR-593-3p
LSM1	hsa-miR-6134
LSM1	hsa-miR-548h-3p

LSM1	hsa-miR-142-5p
LSM1	hsa-miR-2438
LSM1	hsa-miR-743ab-3p
LSM1	hsa-miR-4495
LSM1	hsa-miR-7398q-3p
LSM1	hsa-miR-6769a-3p
LSM1	hsa-miR-423-5p
LSM1	hsa-miR-380
LSM1	hsa-miR-5093
LSM1	hsa-miR-3978
LSM1	hsa-miR-3611
LSM1	hsa-miR-8881
LSM1	hsa-miR-1279
LSM1	hsa-miR-3122
LSM1	hsa-miR-1227-3p
METTL1	hsa-miR-7321-5p
METTL1	hsa-miR-8866
METTL1	hsa-miR-8100
METTL1	hsa-miR-4768-5p
METTL1	hsa-miR-302-3p
METTL1	hsa-miR-193a
METTL1	hsa-miR-92a-1-5p
METTL1	hsa-miR-712-5p
METTL1	hsa-miR-4267
METTL1	hsa-miR-4276
METTL1	hsa-miR-4441
METTL1	hsa-miR-216b-3p
METTL1	hsa-miR-3589
METTL1	hsa-miR-6902-5p
METTL1	hsa-miR-6332
METTL1	hsa-miR-6941-5p
METTL1	hsa-miR-7059-5p
METTL1	hsa-miR-7307-3p
METTL1	hsa-miR-7250-3p
METTL1	hsa-miR-2363
METTL1	hsa-miR-30
METTL1	hsa-miR-6920-5p
METTL1	hsa-miR-6982-5p
METTL1	hsa-miR-214-3p
METTL1	hsa-miR-17-3p
METTL1	hsa-miR-6907-5p
METTL1	hsa-miR-499
METTL1	hsa-miR-1225-3p
METTL1	hsa-miR-3913-5p
METTL1	hsa-miR-8880
METTL1	hsa-miR-191-3p

METTL1	hsa-miR-302d-5p
METTL1	hsa-miR-7222-3p
METTL1	hsa-miR-8836
METTL1	hsa-miR-7213-5p
METTL1	hsa-miR-5710
METTL1	hsa-miR-1905
METTL1	hsa-miR-151-3p
METTL1	hsa-miR-193a-5p
METTL1	hsa-miR-5193
METTL1	hsa-miR-4760-5p
METTL1	hsa-miR-367-5p
METTL1	hsa-miR-3145-5p
METTL1	hsa-miR-3910
METTL1	hsa-miR-4254
METTL1	hsa-miR-8848
METTL1	hsa-miR-7247-5p
METTL1	hsa-miR-8876
METTL1	hsa-miR-5125
METTL1	hsa-miR-1254
METTL1	hsa-miR-1251-3p
METTL1	hsa-miR-6799-5p
METTL1	hsa-miR-7340-5p
METTL1	hsa-miR-486-5p
METTL1	hsa-miR-6808-5p
METTL1	hsa-miR-6384
METTL1	hsa-miR-6529-5p
METTL1	hsa-miR-432-5p
METTL1	hsa-miR-661
METTL1	hsa-miR-34-5p
METTL1	hsa-miR-7244-5p
METTL1	hsa-miR-140-3p.2
METTL1	hsa-miR-7069-5p
METTL1	hsa-miR-887-5p
METTL1	hsa-miR-412-3p
METTL1	hsa-miR-3150b-3p
METTL1	hsa-miR-5087
METTL1	hsa-miR-612
METTL1	hsa-miR-1285-5p
METTL1	hsa-miR-6980-5p
METTL1	hsa-miR-1207-3p
METTL1	hsa-miR-466i-3p
METTL1	hsa-miR-7398s-3p
METTL1	hsa-miR-133-5p
METTL1	hsa-miR-2451
METTL1	hsa-miR-8801
METTL1	hsa-miR-1224-3p

METTL1	hsa-miR-3927-3p
METTL1	hsa-miR-7066-5p
METTL1	hsa-miR-7298-3p
METTL1	hsa-miR-4727-5p
METTL1	hsa-miR-6972-5p
METTL1	hsa-miR-4646-5p
METTL1	hsa-miR-7009-5p
METTL1	hsa-miR-2385-5p
METTL1	hsa-miR-4318
METTL1	hsa-miR-3689-3p
METTL1	hsa-miR-7056-5p
METTL1	hsa-miR-7-5p
METTL1	hsa-miR-6894-5p
METTL1	hsa-miR-149-5p
METTL1	hsa-miR-4451
METTL1	hsa-miR-320-5p
METTL1	hsa-miR-942-5p
METTL1	hsa-miR-642
METTL1	hsa-miR-6758-3p
METTL1	hsa-miR-449-5p
METTL1	hsa-miR-378-5p
METTL1	hsa-miR-510
METTL1	hsa-miR-2364
METTL1	hsa-miR-6503-3p
METTL1	hsa-miR-6731-5p
METTL1	hsa-miR-455-5p
METTL1	hsa-miR-4251
METTL1	hsa-miR-2114-3p
METTL1	hsa-miR-10b-5p
METTL1	hsa-miR-4697-3p
METTL1	hsa-miR-302b-5p
METTL1	hsa-miR-1264
METTL1	hsa-miR-4667-3p
METTL1	hsa-miR-1915-3p
METTL1	hsa-miR-7054-3p
METTL1	hsa-miR-3614-3p
METTL1	hsa-miR-4264
METTL1	hsa-miR-6979-5p
METTL1	hsa-miR-485-3p
METTL1	hsa-miR-452-3p
METTL1	hsa-miR-7356-5p
METTL1	hsa-miR-125b-2-3p
METTL1	hsa-miR-3124-3p
METTL1	hsa-miR-504
METTL1	hsa-miR-8818
METTL1	hsa-miR-4689

METTL1	hsa-miR-6903-5p
METTL1	hsa-miR-4457
METTL1	hsa-miR-1251-5p
METTL1	hsa-miR-4286
METTL1	hsa-miR-136-5p
METTL1	hsa-miR-4728-5p
METTL1	hsa-miR-4711-3p
METTL1	hsa-miR-4761-5p
METTL1	hsa-miR-6754-5p
METTL1	hsa-miR-3569-3p
METTL1	hsa-miR-3162-5p
METTL1	hsa-miR-6792-5p
METTL1	hsa-miR-1b-5p
METTL1	hsa-miR-532-3p
METTL1	hsa-miR-6404
METTL1	hsa-miR-3192-5p
METTL1	hsa-miR-7074-5p
METTL1	hsa-miR-3138
METTL1	hsa-miR-18a-3p
METTL1	hsa-miR-3099-5p
METTL1	hsa-miR-6837-3p
METTL1	hsa-miR-717
METTL1	hsa-miR-329-3p
METTL1	hsa-miR-3591-3p
METTL1	hsa-miR-4433b-3p
METTL1	hsa-miR-7318-5p
METTL1	hsa-miR-3652
METTL1	hsa-miR-764-3p
METTL1	hsa-miR-8061
METTL1	hsa-miR-6831-5p
METTL1	hsa-miR-2450a
METTL1	hsa-miR-6795-3p
METTL1	hsa-miR-193b-5p
METTL1	hsa-miR-7192-3p
METTL1	hsa-miR-1540-5p
METTL1	hsa-miR-548ba
METTL1	hsa-miR-4459
METTL1	hsa-miR-7350-5p
METTL1	hsa-miR-3122
METTL1	hsa-miR-412
METTL1	hsa-miR-466q
METTL1	hsa-miR-4743-3p
METTL1	hsa-miR-463-3p
METTL1	hsa-miR-3605-5p
METTL1	hsa-miR-2900
METTL1	hsa-miR-3177-5p

METTL1	hsa-miR-6878-5p
METTL1	hsa-miR-1840
METTL1	hsa-miR-186-5p
METTL1	hsa-miR-1285-3p
METTL1	hsa-miR-23-5p
METTL1	hsa-miR-1278
METTL1	hsa-miR-142
METTL1	hsa-miR-7051-3p
METTL1	hsa-miR-194-3p
METTL1	hsa-miR-511-3p
METTL1	hsa-miR-6901-3p
METTL1	hsa-miR-4639-5p
METTL1	hsa-miR-7845-5p
METTL1	hsa-miR-3187-5p
METTL1	hsa-miR-7081-5p
METTL1	hsa-miR-379-5p
METTL1	hsa-miR-7211-5p
METTL1	hsa-miR-6883-5p
METTL1	hsa-miR-466c-3p
METTL1	hsa-miR-624-3p
METTL1	hsa-miR-6780a-5p
METTL1	hsa-miR-506-3p
METTL1	hsa-miR-6791-5p
METTL1	hsa-miR-643
METTL1	hsa-miR-8886
METTL1	hsa-miR-539-5p
METTL1	hsa-miR-450b-3p
METTL1	hsa-miR-3061-5p
METTL1	hsa-miR-6125
METTL1	hsa-miR-3551-3p
METTL1	hsa-miR-1285-3p
METTL1	hsa-miR-4693-5p
METTL1	hsa-miR-7068-3p
METTL1	hsa-miR-7398e-3p
METTL1	hsa-miR-129b-5p
METTL1	hsa-miR-467f
METTL1	hsa-miR-4324
METTL1	hsa-miR-30c-3p
METTL1	hsa-miR-146b-3p
METTL1	hsa-miR-3126-5p
METTL1	hsa-miR-150-3p
METTL1	hsa-miR-200c-5p
METTL1	hsa-miR-508b
METTL1	hsa-miR-8789
METTL1	hsa-miR-6077
METTL1	hsa-miR-9500

METTL1	hsa-miR-6926-5p
METTL1	hsa-miR-7854-3p
METTL1	hsa-miR-7152-5p
METTL1	hsa-miR-7362-3p
METTL1	hsa-miR-1200
METTL1	hsa-miR-8845
METTL1	hsa-miR-615-3p
METTL1	hsa-miR-7655-5p
METTL1	hsa-miR-8791
METTL1	hsa-miR-4270
METTL1	hsa-miR-3106-3p
METTL1	hsa-miR-3548
METTL1	hsa-miR-4521
METTL1	hsa-miR-7197-3p
METTL1	hsa-miR-6958-5p
METTL1	hsa-miR-5196-5p
METTL1	hsa-miR-7277-5p
METTL1	hsa-miR-194-1-3p
METTL1	hsa-miR-4733-5p
METTL1	hsa-miR-2392
METTL1	hsa-miR-517
METTL1	hsa-miR-7092-5p
METTL1	hsa-miR-30--3p
METTL1	hsa-miR-548ag
METTL1	hsa-miR-466b-4-3p
METTL1	hsa-miR-3064-3p
METTL1	hsa-miR-361-3p
METTL1	hsa-miR-2459
METTL1	hsa-miR-554
METTL1	hsa-miR-936
METTL1	hsa-miR-3173-5p
METTL1	hsa-miR-4428
METTL1	hsa-miR-874-3p
METTL1	hsa-miR-192-5p
METTL1	hsa-miR-3100-5p
METTL1	hsa-miR-6738-3p
METTL1	hsa-miR-2339
METTL1	hsa-miR-2350
METTL1	hsa-miR-6720-5p
METTL1	hsa-miR-329
METTL1	hsa-miR-8823
METTL1	hsa-miR-6891-3p
METTL1	hsa-miR-6408
METTL1	hsa-miR-7170-3p
METTL1	hsa-miR-7864
METTL1	hsa-miR-7119-5p

METTL1	hsa-miR-519-3p
METTL1	hsa-miR-6984-3p
METTL1	hsa-miR-449c-3p
METTL1	hsa-miR-1248
METTL1	hsa-miR-328
METTL1	hsa-miR-376a-1-5p
METTL1	hsa-miR-2897
METTL1	hsa-miR-6917-3p
METTL1	hsa-miR-5046
METTL1	hsa-miR-8853
METTL1	hsa-miR-764-5p
METTL1	hsa-miR-520-3p
METTL1	hsa-miR-664-5p
METTL1	hsa-miR-1193-3p
METTL1	hsa-miR-221-5p
METTL1	hsa-miR-3116
METTL1	hsa-miR-6826-3p
METTL1	hsa-miR-7024-5p
METTL1	hsa-miR-3593-5p
METTL1	hsa-miR-6840-3p
METTL1	hsa-miR-4308
METTL1	hsa-miR-31-5p
METTL1	hsa-miR-1-3p
METTL1	hsa-miR-6893-5p
METTL1	hsa-miR-219b-5p
METTL1	hsa-miR-6515-5p
METTL1	hsa-miR-183-5p.2
METTL1	hsa-miR-423-3p
METTL1	hsa-miR-3551-5p
METTL1	hsa-miR-330-3p
METTL1	hsa-miR-679
METTL1	hsa-miR-1470
METTL1	hsa-miR-2387
METTL1	hsa-miR-3667-5p
METTL1	hsa-miR-188-3p
METTL1	hsa-miR-6131
METTL1	hsa-miR-4726-3p
METTL1	hsa-miR-4789-3p
METTL1	hsa-miR-6368
METTL1	hsa-miR-124-3p.1
METTL1	hsa-miR-6950-5p
METTL1	hsa-miR-29b-1-5p
METTL1	hsa-miR-3141
METTL1	hsa-miR-6779-5p
METTL1	hsa-miR-346-3p
METTL1	hsa-miR-6764-5p

METTL1	hsa-miR-493
METTL1	hsa-miR-8831
METTL1	hsa-miR-2894
METTL1	hsa-miR-693-5p
METTL1	hsa-miR-139-5p
METTL1	hsa-miR-4643
METTL1	hsa-miR-204-3p
METTL1	hsa-miR-130a-5p
METTL1	hsa-miR-548ai
METTL1	hsa-miR-7338-3p
METTL1	hsa-miR-30c-2-3p
METTL1	hsa-miR-508-5p
METTL1	hsa-miR-373-3p
METTL1	hsa-miR-628-3p
METTL1	hsa-miR-2895
METTL1	hsa-miR-763
METTL1	hsa-miR-2411-3p
METTL1	hsa-miR-874
METTL1	hsa-miR-29-5p
METTL1	hsa-miR-3074-5p
METTL1	hsa-miR-466f-3p
METTL1	hsa-miR-7334-5p
METTL1	hsa-miR-1956-5p
METTL1	hsa-miR-4692
METTL1	hsa-miR-547-5p
METTL1	hsa-miR-449a
METTL1	hsa-miR-6875-3p
METTL1	hsa-miR-450a-1-3p
METTL1	hsa-miR-145-5p
METTL1	hsa-miR-576-3p
METTL1	hsa-miR-188-5p
METTL1	hsa-miR-7386j-3p
METTL1	hsa-miR-6842-3p
METTL1	hsa-miR-3094-5p
METTL1	hsa-miR-29a-5p
METTL1	hsa-miR-7260-3p
METTL1	hsa-miR-4668-5p
METTL1	hsa-miR-6754-3p
METTL1	hsa-miR-1249-5p
METTL1	hsa-miR-483-3p
METTL1	hsa-miR-4298
METTL1	hsa-miR-217
METTL1	hsa-miR-6750-5p
METTL1	hsa-miR-7184-3p
METTL1	hsa-miR-21-3p
METTL1	hsa-miR-4659a-3p

METTL1	hsa-miR-7976
METTL1	hsa-miR-1273h-5p
METTL1	hsa-miR-497-5p
METTL1	hsa-miR-338-3p
METTL1	hsa-miR-342-3p
METTL1	hsa-miR-7106-5p
METTL1	hsa-miR-7196-3p
METTL1	hsa-miR-6362
METTL1	hsa-miR-211-5p
METTL1	hsa-miR-5584-5p
METTL1	hsa-miR-591
METTL1	hsa-miR-7685-5p
METTL1	hsa-miR-370-3p
METTL1	hsa-miR-6909-5p
METTL1	hsa-miR-504-3p
METTL1	hsa-miR-4297
METTL1	hsa-miR-450a-2-3p
METTL1	hsa-miR-362-3p
METTL1	hsa-miR-8808
METTL1	hsa-miR-7319-5p
METTL1	hsa-miR-4795-5p
METTL1	hsa-miR-181-5p
METTL1	hsa-miR-1207
METTL1	hsa-miR-3192
METTL1	hsa-miR-548au-3p
METTL1	hsa-miR-205-3p
METTL1	hsa-miR-874-5p
METTL1	hsa-miR-149-3p
METTL1	hsa-miR-4514
METTL1	hsa-miR-6929-5p
METTL1	hsa-miR-3064-5p
METTL1	hsa-miR-3475-3p
METTL1	hsa-miR-669c-3p
METTL1	hsa-miR-624-3p
METTL1	hsa-miR-6318
METTL1	hsa-miR-3121-5p
METTL1	hsa-miR-3092-3p
METTL1	hsa-miR-765
METTL1	hsa-miR-4666a-5p
METTL1	hsa-miR-6785-5p
METTL1	hsa-miR-150-5p
METTL1	hsa-miR-6739-5p
METTL1	hsa-miR-152-5p
METTL1	hsa-miR-8060
METTL1	hsa-miR-4650-3p
METTL1	hsa-miR-4645-5p

METTL1	hsa-miR-3613b
METTL1	hsa-miR-5197-5p
METTL1	hsa-miR-6398
METTL1	hsa-miR-1976
METTL1	hsa-miR-145-3p
METTL1	hsa-miR-92a-2-5p
METTL1	hsa-miR-7252-3p
METTL1	hsa-miR-215-5p
METTL1	hsa-miR-544a
METTL1	hsa-miR-139-3p
METTL1	hsa-miR-1545a-3p
METTL1	hsa-miR-769
METTL1	hsa-miR-3564
METTL1	hsa-miR-7035-3p
METTL1	hsa-miR-4684-5p
METTL1	hsa-miR-7386e-3p
METTL1	hsa-miR-3135a
METTL1	hsa-miR-4747-5p
METTL1	hsa-miR-513b-5p
METTL1	hsa-miR-2449
METTL1	hsa-miR-2881
METTL1	hsa-miR-7216-5p
METTL1	hsa-miR-1953
METTL1	hsa-miR-6366
METTL1	hsa-miR-5108
METTL1	hsa-miR-200b-3p
METTL1	hsa-miR-939-3p
METTL1	hsa-miR-1239
METTL1	hsa-miR-6809-3p
METTL1	hsa-miR-4704-3p
METTL1	hsa-miR-6936-5p
METTL1	hsa-miR-338-3p
METTL1	hsa-miR-4797-3p
METTL1	hsa-miR-4433a-5p
METTL1	hsa-miR-7678-3p
METTL1	hsa-miR-4660
METTL1	hsa-miR-3432a
METTL1	hsa-miR-6890-3p
METTL1	hsa-miR-570-5p
METTL1	hsa-miR-3057-3p
METTL1	hsa-miR-2411-5p
METTL1	hsa-miR-6797-5p
METTL1	hsa-miR-16-5p
METTL1	hsa-miR-3927-3p
METTL1	hsa-miR-7116-3p
METTL1	hsa-miR-367-3p

METTL1	hsa-miR-1251
METTL1	hsa-miR-467g
METTL1	hsa-miR-342-5p
METTL1	hsa-miR-1193
METTL1	hsa-miR-5190
METTL1	hsa-miR-124
METTL1	hsa-miR-8904b
METTL1	hsa-miR-2330-3p
METTL1	hsa-miR-5001-3p
METTL1	hsa-miR-6523a
METTL1	hsa-miR-1277-3p
METTL1	hsa-miR-6736-3p
METTL1	hsa-miR-6356
METTL1	hsa-miR-7688-3p
METTL1	hsa-miR-379-5p
METTL1	hsa-miR-615-5p
METTL1	hsa-miR-7011-5p
METTL1	hsa-miR-4659b-3p
METTL1	hsa-miR-8841
METTL1	hsa-miR-630
METTL1	hsa-miR-7847-3p
METTL1	hsa-miR-7023-5p
METTL1	hsa-miR-7372-3p
METTL1	hsa-miR-193-5p
METTL1	hsa-miR-101-1-5p
METTL1	hsa-miR-892a
METTL1	hsa-miR-670-3p
METTL1	hsa-miR-1226-3p
METTL1	hsa-miR-6822-5p
METTL1	hsa-miR-6760-5p
METTL1	hsa-miR-7218-5p
METTL1	hsa-miR-466b-5p
METTL1	hsa-miR-6733-5p
METTL1	hsa-miR-1267
METTL1	hsa-miR-6517
METTL1	hsa-miR-5581-5p
METTL1	hsa-miR-145-5p
METTL1	hsa-miR-3619-5p
METTL1	hsa-miR-6991-3p
METTL1	hsa-miR-2902
METTL1	hsa-miR-4662b
METTL1	hsa-miR-6807-5p
METTL1	hsa-miR-924
METTL1	hsa-miR-4426
METTL1	hsa-miR-3085-3p
METTL1	hsa-miR-7055-5p

METTL1	hsa-miR-760-5p
METTL1	hsa-miR-7035-5p
METTL1	hsa-miR-7386b-3p
METTL1	hsa-miR-204-5p
METTL1	hsa-miR-8837
METTL1	hsa-miR-6072
METTL1	hsa-miR-3658
METTL1	hsa-miR-5189-5p
METTL1	hsa-miR-7261-5p
METTL1	hsa-miR-186-3p
METTL1	hsa-miR-470-5p
METTL1	hsa-miR-4323
METTL1	hsa-miR-7237-3p
METTL1	hsa-miR-7385a-3p
METTL1	hsa-miR-7377-3p
METTL1	hsa-miR-3154
METTL1	hsa-miR-940
METTL1	hsa-miR-544b
METTL1	hsa-miR-7652-3p
METTL1	hsa-miR-15
METTL1	hsa-miR-2114-5p
METTL1	hsa-miR-342-3p
METTL1	hsa-miR-3058-5p
METTL1	hsa-miR-372-3p
METTL1	hsa-miR-146-5p
METTL1	hsa-miR-8832
METTL1	hsa-miR-8847
METTL1	hsa-miR-4433b-5p
METTL1	hsa-miR-8085
METTL1	hsa-miR-196-3p
METTL1	hsa-miR-5787
METTL1	hsa-miR-7150
METTL1	hsa-miR-4661-3p
METTL1	hsa-miR-6959-5p
METTL1	hsa-miR-34
METTL1	hsa-miR-9769-5p
METTL1	hsa-miR-6995-5p
METTL1	hsa-miR-181a-2-3p
METTL1	hsa-miR-4696
METTL1	hsa-miR-486-5p
METTL1	hsa-miR-202-3p
METTL1	hsa-miR-7684-5p
METTL1	hsa-miR-3583-3p
METTL1	hsa-miR-6914-5p
METTL1	hsa-miR-605-3p
METTL1	hsa-miR-140-3p.1

METTL1	hsa-miR-3153
METTL1	hsa-miR-207
METTL1	hsa-miR-540-3p
METTL1	hsa-miR-7230-5p
METTL1	hsa-miR-3083-5p
METTL1	hsa-miR-673-3p
METTL1	hsa-miR-324-3p
METTL1	hsa-miR-6788-5p
METTL1	hsa-miR-7217-3p
METTL1	hsa-miR-6910-5p
METTL1	hsa-miR-6507-3p
METTL1	hsa-miR-8796
METTL1	hsa-miR-216a-3p
METTL1	hsa-miR-5004-5p
METTL1	hsa-miR-378d
METTL1	hsa-miR-6793-5p
METTL1	hsa-miR-7277-3p
METTL1	hsa-miR-513
METTL1	hsa-miR-6372
METTL1	hsa-miR-7354-3p
METTL1	hsa-miR-18-3p
METTL1	hsa-miR-6930-5p
METTL1	hsa-miR-1956-3p
METTL1	hsa-miR-7386l-3p
METTL1	hsa-miR-6794-3p
METTL1	hsa-miR-206-5p
METTL1	hsa-miR-297bc-3p
METTL1	hsa-miR-7006-5p
METTL1	hsa-miR-7356-3p
METTL1	hsa-miR-370
METTL1	hsa-miR-7373b-3p
METTL1	hsa-miR-124-3p.2
METTL1	hsa-miR-8908-5p
METTL1	hsa-miR-6834-3p
METTL1	hsa-miR-2428
METTL1	hsa-miR-759
METTL1	hsa-miR-7018-3p
METTL1	hsa-miR-3938-3p
METTL1	hsa-miR-4446-5p
METTL1	hsa-miR-885-5p
METTL1	hsa-miR-6951-3p
METTL1	hsa-miR-6858-5p
METTL1	hsa-miR-2355-5p
METTL1	hsa-miR-30b-3p
METTL1	hsa-miR-7192-5p
METTL1	hsa-miR-6860

METTL1	hsa-miR-223-3p
METTL1	hsa-miR-3547-5p
METTL1	hsa-miR-8857
METTL1	hsa-miR-7240-5p
METTL1	hsa-miR-299-3p
METTL1	hsa-miR-148-5p
METTL1	hsa-miR-7091-5p
METTL1	hsa-miR-7304-3p
METTL1	hsa-miR-4292
METTL1	hsa-miR-30-5p
METTL1	hsa-miR-6904-5p
METTL1	hsa-miR-889-5p
METTL1	hsa-miR-429
METTL1	hsa-miR-7293-5p
METTL1	hsa-miR-520f-5p
METTL1	hsa-miR-7023-3p
METTL1	hsa-miR-378a-5p
METTL1	hsa-miR-7279-5p
METTL1	hsa-miR-1843
METTL1	hsa-miR-133
METTL1	hsa-miR-550a-3p
METTL1	hsa-miR-4652-3p
METTL1	hsa-miR-627-3p
METTL1	hsa-miR-6938-3p
METTL1	hsa-miR-4430
METTL1	hsa-miR-2324
METTL1	hsa-miR-510-5p
METTL1	hsa-miR-2447
METTL1	hsa-miR-6787-3p
METTL1	hsa-miR-672-3p
METTL1	hsa-miR-4680
METTL1	hsa-miR-1277-3p
METTL1	hsa-miR-7309-5p
METTL1	hsa-miR-6405
METTL1	hsa-miR-6833-3p
METTL1	hsa-miR-873-3p
METTL1	hsa-miR-384-5p
METTL1	hsa-miR-539-5p
METTL1	hsa-miR-7-5p
METTL1	hsa-miR-6134
METTL1	hsa-miR-505-5p
METTL1	hsa-miR-466b-2-3p
METTL1	hsa-miR-4505
METTL1	hsa-miR-518-5p
METTL1	hsa-miR-519-5p
METTL1	hsa-miR-466ade-3p

METTL1	hsa-miR-576-5p
METTL1	hsa-miR-455-3p
METTL1	hsa-miR-1957a
METTL1	hsa-miR-7160-5p
METTL1	hsa-miR-194-2-3p
METTL1	hsa-miR-2360
METTL1	hsa-miR-518-3p
METTL1	hsa-miR-2486-5p
METTL1	hsa-miR-766-5p
METTL1	hsa-miR-1982-5p
METTL1	hsa-miR-27-3p
METTL1	hsa-miR-651-3p
METTL1	hsa-miR-136-5p
METTL1	hsa-miR-7154-5p
METTL1	hsa-miR-96-5p
METTL1	hsa-miR-1899
METTL1	hsa-miR-6721-5p
METTL1	hsa-miR-642a-5p
METTL1	hsa-miR-6887-3p
METTL1	hsa-miR-7286-3p
METTL1	hsa-miR-324
METTL1	hsa-miR-8812
METTL1	hsa-miR-5680
METTL1	hsa-miR-3097-3p
METTL1	hsa-miR-7383-3p
METTL1	hsa-miR-7289-5p
METTL1	hsa-miR-7050-3p
METTL1	hsa-miR-466bcp-3p
METTL1	hsa-miR-4647
METTL1	hsa-miR-8905
METTL1	hsa-miR-6974-3p
METTL1	hsa-miR-6740-5p
METTL1	hsa-miR-6969-5p
METTL1	hsa-miR-766-3p
METTL1	hsa-miR-451b
METTL1	hsa-miR-6513-5p
METTL1	hsa-miR-4530
METTL1	hsa-miR-1911-3p
METTL1	hsa-miR-4695-3p
METTL1	hsa-miR-3104-5p
METTL1	hsa-miR-185-5p
METTL1	hsa-miR-1-5p
METTL1	hsa-miR-466
METTL1	hsa-miR-192-5p
METTL1	hsa-miR-7019-5p
METTL1	hsa-miR-7154-3p

METTL1	hsa-miR-215
METTL1	hsa-miR-766-3p
METTL1	hsa-miR-2442
METTL1	hsa-miR-195-5p
METTL1	hsa-miR-1545c-3p
METTL1	hsa-miR-3075-5p
METTL1	hsa-miR-345-3p
METTL1	hsa-miR-7185-5p
METTL1	hsa-miR-6946-3p
METTL1	hsa-miR-7088-3p
METTL1	hsa-miR-185-5p
METTL1	hsa-miR-10b-5p
METTL1	hsa-miR-466mo-3p
METTL1	hsa-miR-3584-5p
METTL1	hsa-miR-30-3p
METTL1	hsa-miR-128-3p
METTL1	hsa-miR-4673
METTL1	hsa-miR-1952
METTL1	hsa-miR-1302
METTL1	hsa-miR-7038-3p
METTL1	hsa-miR-10b-3p
METTL1	hsa-miR-5110
METTL1	hsa-miR-129-2-3p
METTL1	hsa-miR-1913
METTL1	hsa-miR-769-3p
METTL1	hsa-miR-146
METTL1	hsa-miR-4274
METTL1	hsa-miR-7270-3p
METTL1	hsa-miR-7382-5p
NCBP1	hsa-miR-215-5p
NCBP1	hsa-miR-1250-3p
NCBP1	hsa-miR-6506-5p
NCBP1	hsa-miR-871-5p
NCBP1	hsa-miR-188-5p
NCBP1	hsa-miR-7352-3p
NCBP1	hsa-miR-4709-3p
NCBP1	hsa-miR-743ab-3p
NCBP1	hsa-miR-2468
NCBP1	hsa-miR-3475-5p
NCBP1	hsa-miR-942-5p
NCBP1	hsa-miR-4282
NCBP1	hsa-miR-7280-5p
NCBP1	hsa-miR-5698
NCBP1	hsa-miR-543
NCBP1	hsa-miR-19b-5p
NCBP1	hsa-miR-3159

NCBP1	hsa-miR-8792
NCBP1	hsa-miR-125-5p
NCBP1	hsa-miR-2461-3p
NCBP1	hsa-miR-8843
NCBP1	hsa-miR-7389-3p
NCBP1	hsa-miR-4490
NCBP1	hsa-miR-3170
NCBP1	hsa-miR-6122-3p
NCBP1	hsa-miR-6085
NCBP1	hsa-miR-664a-5p
NCBP1	hsa-miR-2284d
NCBP1	hsa-miR-5699-3p
NCBP1	hsa-miR-488-3p
NCBP1	hsa-miR-544-3p
NCBP1	hsa-miR-2355-5p
NCBP1	hsa-miR-7257-3p
NCBP1	hsa-miR-3653-5p
NCBP1	hsa-miR-7043-5p
NCBP1	hsa-miR-883-3p
NCBP1	hsa-miR-575
NCBP1	hsa-miR-6715a-3p
NCBP1	hsa-miR-330-3p
NCBP1	hsa-miR-3103-3p
NCBP1	hsa-miR-6742-3p
NCBP1	hsa-miR-4666b
NCBP1	hsa-miR-551b-5p
NCBP1	hsa-miR-1258
NCBP1	hsa-miR-2113
NCBP1	hsa-miR-6810-5p
NCBP1	hsa-miR-6723-5p
NCBP1	hsa-miR-3152-3p
NCBP1	hsa-miR-6128
NCBP1	hsa-miR-5702
NCBP1	hsa-miR-301-5p
NCBP1	hsa-miR-603
NCBP1	hsa-miR-691
NCBP1	hsa-miR-4796-3p
NCBP1	hsa-miR-5589-5p
NCBP1	hsa-miR-3605-5p
NCBP1	hsa-let-7c-1-3p
NCBP1	hsa-miR-411-5p
NCBP1	hsa-miR-483-3p
NCBP1	hsa-miR-6534
NCBP1	hsa-miR-5697-5p
NCBP1	hsa-miR-3622a-3p
NCBP1	hsa-miR-4676-5p

NCBP1	hsa-miR-30e-3p
NCBP1	hsa-miR-548a-5p
NCBP1	hsa-miR-5579-5p
NCBP1	hsa-miR-659-3p
NCBP1	hsa-miR-3928-5p
NCBP1	hsa-miR-212-3p
NCBP1	hsa-miR-4518
NCBP1	hsa-miR-3167-5p
NCBP1	hsa-miR-892a
NCBP1	hsa-miR-5706
NCBP1	hsa-miR-5196-3p
NCBP1	hsa-miR-142-3p
NCBP1	hsa-miR-6379
NCBP1	hsa-miR-7074-5p
NCBP1	hsa-miR-582-5p
NCBP1	hsa-miR-7207-3p
NCBP1	hsa-miR-4711-5p
NCBP1	hsa-miR-338-3p
NCBP1	hsa-miR-1283
NCBP1	hsa-miR-3095-5p
NCBP1	hsa-miR-33-5p
NCBP1	hsa-miR-8119
NCBP1	hsa-miR-7334-5p
NCBP1	hsa-miR-3125
NCBP1	hsa-miR-519-3p
NCBP1	hsa-let-7b-3p
NCBP1	hsa-miR-1912-5p
NCBP1	hsa-miR-7032-3p
NCBP1	hsa-miR-764-5p
NCBP1	hsa-miR-1236-3p
NCBP1	hsa-miR-1271-3p
NCBP1	hsa-miR-153-5p
NCBP1	hsa-miR-7226-3p
NCBP1	hsa-miR-7269-3p
NCBP1	hsa-miR-3681-5p
NCBP1	hsa-miR-9-3p
NCBP1	hsa-miR-3191-3p
NCBP1	hsa-miR-150-5p
NCBP1	hsa-miR-3157-5p
NCBP1	hsa-miR-2337
NCBP1	hsa-miR-211-3p
NCBP1	hsa-miR-8108
NCBP1	hsa-miR-129-5p
NCBP1	hsa-miR-6411
NCBP1	hsa-miR-8056
NCBP1	hsa-miR-1244

NCBP1	hsa-miR-450a-1-3p
NCBP1	hsa-miR-548aq-5p
NCBP1	hsa-miR-4723-5p
NCBP1	hsa-miR-669ao-3p
NCBP1	hsa-miR-1273f
NCBP1	hsa-miR-628-3p
NCBP1	hsa-miR-378j
NCBP1	hsa-miR-200a-3p
NCBP1	hsa-miR-203-3p.1
NCBP1	hsa-miR-5571-3p
NCBP1	hsa-miR-4298
NCBP1	hsa-miR-2482
NCBP1	hsa-miR-4771
NCBP1	hsa-miR-6943-3p
NCBP1	hsa-miR-548ah-3p
NCBP1	hsa-miR-4252
NCBP1	hsa-miR-26
NCBP1	hsa-miR-8120
NCBP1	hsa-miR-4454
NCBP1	hsa-miR-598-3p
NCBP1	hsa-miR-23a-3p
NCBP1	hsa-miR-672-5p
NCBP1	hsa-miR-101a-5p
NCBP1	hsa-miR-3570
NCBP1	hsa-miR-3473f
NCBP1	hsa-miR-569
NCBP1	hsa-miR-7267-5p
NCBP1	hsa-miR-6517
NCBP1	hsa-miR-3173-3p
NCBP1	hsa-miR-196b-3p
NCBP1	hsa-miR-654-5p
NCBP1	hsa-miR-540-5p
NCBP1	hsa-miR-203
NCBP1	hsa-miR-6715b-3p
NCBP1	hsa-miR-6077
NCBP1	hsa-miR-693-5p
NCBP1	hsa-let-7ag-3p
NCBP1	hsa-miR-548g-5p
NCBP1	hsa-miR-6780b-5p
NCBP1	hsa-miR-199-3p
NCBP1	hsa-miR-7398b-3p
NCBP1	hsa-miR-3662
NCBP1	hsa-miR-7160-5p
NCBP1	hsa-miR-514a-3p
NCBP1	hsa-miR-4524a-5p
NCBP1	hsa-miR-4498

NCBP1	hsa-miR-633
NCBP1	hsa-miR-548av-3p
NCBP1	hsa-miR-877-5p
NCBP1	hsa-miR-2053
NCBP1	hsa-miR-548ab
NCBP1	hsa-miR-944
NCBP1	hsa-miR-431-5p
NCBP1	hsa-miR-1958
NCBP1	hsa-miR-3121-5p
NCBP1	hsa-miR-4777-3p
NCBP1	hsa-miR-296-3p
NCBP1	hsa-miR-7284-3p
NCBP1	hsa-miR-1279
NCBP1	hsa-miR-548b-3p
NCBP1	hsa-miR-205-3p
NCBP1	hsa-miR-590-5p
NCBP1	hsa-miR-218-1-3p
NCBP1	hsa-miR-1284
NCBP1	hsa-miR-619-3p
NCBP1	hsa-miR-1952
NCBP1	hsa-miR-7246-5p
NCBP1	hsa-miR-4657
NCBP1	hsa-miR-185-3p
NCBP1	hsa-miR-5582-3p
NCBP1	hsa-miR-8834b
NCBP1	hsa-miR-3168
NCBP1	hsa-miR-193b-5p
NCBP1	hsa-miR-105-5p
NCBP1	hsa-miR-1231-3p
NCBP1	hsa-miR-141-3p
NCBP1	hsa-miR-6385
NCBP1	hsa-miR-548f-3p
NCBP1	hsa-miR-204-3p
NCBP1	hsa-miR-3094-3p
NCBP1	hsa-miR-4690-3p
NCBP1	hsa-miR-345-5p
NCBP1	hsa-miR-412-3p
NCBP1	hsa-miR-138-1-3p
NCBP1	hsa-miR-582-5p
NCBP1	hsa-miR-5104
NCBP1	hsa-miR-3923
NCBP1	hsa-miR-32-5p
NCBP1	hsa-miR-5089-3p
NCBP1	hsa-miR-501-5p
NCBP1	hsa-miR-425-5p
NCBP1	hsa-miR-153-3p

NCBP1	hsa-miR-3190-3p
NCBP1	hsa-miR-8055
NCBP1	hsa-miR-290
NCBP1	hsa-miR-8864
NCBP1	hsa-miR-875-5p
NCBP1	hsa-miR-24-3p
NCBP1	hsa-miR-499a-3p
NCBP1	hsa-miR-6756-3p
NCBP1	hsa-miR-3557-3p
NCBP1	hsa-miR-194-5p
NCBP1	hsa-miR-626
NCBP1	hsa-miR-376b-5p
NCBP1	hsa-miR-1951
NCBP1	hsa-miR-548ay-5p
NCBP1	hsa-miR-3174
NCBP1	hsa-miR-3680-3p
NCBP1	hsa-miR-6729-3p
NCBP1	hsa-miR-556-5p
NCBP1	hsa-miR-6733-5p
NCBP1	hsa-miR-6947-3p
NCBP1	hsa-miR-137
NCBP1	hsa-miR-2313-3p
NCBP1	hsa-miR-548
NCBP1	hsa-miR-7371a-5p
NCBP1	hsa-miR-6730-5p
NCBP1	hsa-miR-92
NCBP1	hsa-miR-216b
NCBP1	hsa-miR-203a-3p.1
NCBP1	hsa-miR-5008-3p
NCBP1	hsa-miR-937-5p
NCBP1	hsa-miR-7213-5p
NCBP1	hsa-miR-548e-5p
NCBP1	hsa-miR-497-3p
NCBP1	hsa-miR-877-3p
NCBP1	hsa-miR-6728-5p
NCBP1	hsa-miR-7019-5p
NCBP1	hsa-miR-505-5p
NCBP1	hsa-miR-2477
NCBP1	hsa-miR-7370i-3p
NCBP1	hsa-miR-551-5p
NCBP1	hsa-miR-6773-3p
NCBP1	hsa-miR-653-3p
NCBP1	hsa-miR-489
NCBP1	hsa-miR-1234-3p
NCBP1	hsa-miR-292-5p
NCBP1	hsa-miR-561-5p

NCBP1	hsa-miR-548d-3p
NCBP1	hsa-miR-301-3p
NCBP1	hsa-miR-7173-5p
NCBP1	hsa-miR-145-5p
NCBP1	hsa-miR-6838-3p
NCBP1	hsa-miR-489-3p
NCBP1	hsa-miR-124-5p
NCBP1	hsa-miR-3471
NCBP1	hsa-miR-876
NCBP1	hsa-miR-93-5p
NCBP1	hsa-miR-380-5p
NCBP1	hsa-miR-7660-3p
NCBP1	hsa-miR-2329-5p
NCBP1	hsa-miR-517
NCBP1	hsa-miR-520c-3p
NCBP1	hsa-miR-581
NCBP1	hsa-miR-2332
NCBP1	hsa-miR-548am-5p
NCBP1	hsa-miR-6837-3p
NCBP1	hsa-miR-4725-5p
NCBP1	hsa-miR-2437
NCBP1	hsa-miR-4503
NCBP1	hsa-miR-329-5p
NCBP1	hsa-miR-224-5p
NCBP1	hsa-miR-520g-3p
NCBP1	hsa-miR-4288
NCBP1	hsa-miR-3590-3p
NCBP1	hsa-miR-6754-3p
NCBP1	hsa-miR-548f-5p
NCBP1	hsa-miR-421-3p
NCBP1	hsa-miR-7207-5p
NCBP1	hsa-miR-411-3p
NCBP1	hsa-miR-654-3p
NCBP1	hsa-miR-7388a-3p
NCBP1	hsa-miR-1835
NCBP1	hsa-miR-4524b-3p
NCBP1	hsa-miR-1950
NCBP1	hsa-miR-8874
NCBP1	hsa-miR-365a-5p
NCBP1	hsa-miR-411-5p.2
NCBP1	hsa-miR-577
NCBP1	hsa-miR-4803
NCBP1	hsa-miR-6839-5p
NCBP1	hsa-miR-548ae-5p
NCBP1	hsa-miR-2386
NCBP1	hsa-miR-6891-5p

NCBP1	hsa-miR-556-3p
NCBP1	hsa-miR-7194-5p
NCBP1	hsa-miR-374b-3p
NCBP1	hsa-miR-6348
NCBP1	hsa-miR-1226-3p
NCBP1	hsa-miR-3590-5p
NCBP1	hsa-miR-122
NCBP1	hsa-miR-5190
NCBP1	hsa-miR-6823-5p
NCBP1	hsa-miR-5685
NCBP1	hsa-miR-2351
NCBP1	hsa-miR-4703-3p
NCBP1	hsa-miR-374-5p
NCBP1	hsa-miR-411-5p
NCBP1	hsa-miR-7218-5p
NCBP1	hsa-miR-4659a-5p
NCBP1	hsa-miR-502-5p
NCBP1	hsa-miR-4742-3p
NCBP1	hsa-miR-1291
NCBP1	hsa-miR-6404
NCBP1	hsa-miR-6938-3p
NCBP1	hsa-miR-3544-5p
NCBP1	hsa-miR-6927-3p
NCBP1	hsa-miR-3144-5p
NCBP1	hsa-miR-7275-3p
NCBP1	hsa-miR-548as-3p
NCBP1	hsa-miR-1915-3p
NCBP1	hsa-miR-513c-3p
NCBP1	hsa-miR-504-3p
NCBP1	hsa-miR-4310
NCBP1	hsa-miR-4699-5p
NCBP1	hsa-miR-155-5p
NCBP1	hsa-miR-4289
NCBP1	hsa-miR-7208-5p
NCBP1	hsa-miR-559
NCBP1	hsa-miR-767
NCBP1	hsa-miR-154-5p
NCBP1	hsa-miR-127-5p
NCBP1	hsa-miR-3916
NCBP1	hsa-miR-506-5p
NCBP1	hsa-miR-874-3p
NCBP1	hsa-miR-6508-5p
NCBP1	hsa-miR-2479
NCBP1	hsa-miR-3123
NCBP1	hsa-miR-1323
NCBP1	hsa-miR-513b-3p

NCBP1	hsa-miR-17-5p
NCBP1	hsa-miR-223-3p
NCBP1	hsa-miR-873a-5p.1
NCBP1	hsa-miR-7394b-5p
NCBP1	hsa-miR-7007-3p
NCBP1	hsa-miR-6737-3p
NCBP1	hsa-miR-4795-5p
NCBP1	hsa-miR-876-3p
NCBP1	hsa-miR-4760-3p
NCBP1	hsa-miR-2284m
NCBP1	hsa-miR-548as-5p
NCBP1	hsa-miR-4728-3p
NCBP1	hsa-miR-98-3p
NCBP1	hsa-miR-7241-5p
NCBP1	hsa-miR-215-3p
NCBP1	hsa-miR-562
NCBP1	hsa-miR-3129-5p
NCBP1	hsa-miR-3099-5p
NCBP1	hsa-miR-7001-3p
NCBP1	hsa-miR-7191-5p
NCBP1	hsa-miR-6840-3p
NCBP1	hsa-miR-450a
NCBP1	hsa-miR-6999-3p
NCBP1	hsa-let-7a-3p
NCBP1	hsa-miR-891a-3p
NCBP1	hsa-miR-600
NCBP1	hsa-miR-293-5p
NCBP1	hsa-miR-2322-3p
NCBP1	hsa-miR-376c
NCBP1	hsa-miR-883ab-3p
NCBP1	hsa-miR-548bb-5p
NCBP1	hsa-miR-7650-5p
NCBP1	hsa-miR-9-5p
NCBP1	hsa-miR-299
NCBP1	hsa-miR-2454-3p
NCBP1	hsa-miR-550a-3-5p
NCBP1	hsa-miR-802
NCBP1	hsa-miR-1933-3p
NCBP1	hsa-miR-7b-3p
NCBP1	hsa-miR-361-5p
NCBP1	hsa-miR-758-5p
NCBP1	hsa-miR-4729
NCBP1	hsa-miR-126-3p
NCBP1	hsa-miR-4700-5p
NCBP1	hsa-miR-6321
NCBP1	hsa-miR-7220-3p

NCBP1	hsa-miR-1286
NCBP1	hsa-miR-548b-5p
NCBP1	hsa-miR-449a
NCBP1	hsa-miR-452-5p
NCBP1	hsa-miR-4782-5p
NCBP1	hsa-miR-669b-5p
NCBP1	hsa-miR-4520-5p
NCBP1	hsa-miR-548at-3p
NCBP1	hsa-miR-17
NCBP1	hsa-miR-548n
NCBP1	hsa-miR-7660-5p
NCBP1	hsa-miR-2424
NCBP1	hsa-miR-203-3p
NCBP1	hsa-miR-377-3p
NCBP1	hsa-miR-6958-3p
NCBP1	hsa-miR-881-3p
NCBP1	hsa-let-7abf-3p
NCBP1	hsa-miR-7349-3p
NCBP1	hsa-miR-7277-3p
NCBP1	hsa-miR-320-5p
NCBP1	hsa-miR-1902
NCBP1	hsa-miR-4468
NCBP1	hsa-miR-550a-5p
NCBP1	hsa-miR-8854
NCBP1	hsa-miR-2356
NCBP1	hsa-miR-132-3p
NCBP1	hsa-miR-6765-3p
NCBP1	hsa-miR-545-3p
NCBP1	hsa-miR-6771-3p
NCBP1	hsa-miR-3966
NCBP1	hsa-miR-6909-3p
NCBP1	hsa-miR-463-5p
NCBP1	hsa-miR-7262b-5p
NCBP1	hsa-miR-7386g-3p
NCBP1	hsa-miR-674-5p
NCBP1	hsa-miR-4271
NCBP1	hsa-miR-636
NCBP1	hsa-miR-6813-3p
NCBP1	hsa-miR-541-5p
NCBP1	hsa-miR-376b-3p
NCBP1	hsa-miR-372-3p
NCBP1	hsa-miR-548i-3p
NCBP1	hsa-miR-4438
NCBP1	hsa-miR-7388a-5p
NCBP1	hsa-miR-7013-3p
NCBP1	hsa-miR-130-3p

NCBP1	hsa-miR-6759-3p
NCBP1	hsa-miR-7188-3p
NCBP1	hsa-miR-4263
NCBP1	hsa-miR-107-5p
NCBP1	hsa-miR-4778-5p
NCBP1	hsa-miR-1267
NCBP1	hsa-miR-619
NCBP1	hsa-miR-669p-3p
NCBP1	hsa-miR-567
NCBP1	hsa-miR-548t-3p
NCBP1	hsa-miR-6782-5p
NCBP1	hsa-miR-627-3p
NCBP1	hsa-miR-548aw
NCBP1	hsa-miR-373-3p
NCBP1	hsa-miR-3595
NCBP1	hsa-miR-1194
NCBP1	hsa-miR-4780
NCBP1	hsa-miR-6848-3p
NCBP1	hsa-miR-190a-5p
NCBP1	hsa-miR-6937-3p
NCBP1	hsa-miR-197-5p
NCBP1	hsa-miR-7647-5p
NCBP1	hsa-miR-1298-5p
NCBP1	hsa-miR-33a-3p
NCBP1	hsa-miR-326
NCBP1	hsa-miR-548bd-3p
NCBP1	hsa-miR-3146
NCBP1	hsa-miR-8052
NCBP1	hsa-miR-367-5p
NCBP1	hsa-miR-1266-5p
NCBP1	hsa-miR-7359-5p
NCBP1	hsa-miR-23b-3p
NCBP1	hsa-miR-455-5p
NCBP1	hsa-miR-224-5p
NCBP1	hsa-miR-361-3p
NCBP1	hsa-miR-125a-3p
NCBP1	hsa-miR-4516
NCBP1	hsa-miR-365b-5p
NCBP1	hsa-miR-7379-5p
NCBP1	hsa-miR-487-5p
NCBP1	hsa-miR-7370-5p
NCBP1	hsa-miR-3120-3p
NCBP1	hsa-miR-297
NCBP1	hsa-miR-18
NCBP1	hsa-miR-2472
NCBP1	hsa-miR-6919-3p

NCBP1	hsa-miR-875
NCBP1	hsa-miR-4463
NCBP1	hsa-miR-708-5p
NCBP1	hsa-miR-7151-3p
NCBP1	hsa-miR-6914-5p
NCBP1	hsa-miR-3090-5p
NCBP1	hsa-miR-4708-5p
NCBP1	hsa-miR-676-3p
NCBP1	hsa-miR-130c-5p
NCBP1	hsa-miR-6807-5p
NCBP1	hsa-miR-302-3p
NCBP1	hsa-miR-6975-3p
NCBP1	hsa-miR-7398q-3p
NCBP1	hsa-miR-372
NCBP1	hsa-miR-6758-5p
NCBP1	hsa-miR-186-5p
NCBP1	hsa-miR-7174-5p
NCBP1	hsa-miR-545-5p
NCBP1	hsa-miR-8846
NCBP1	hsa-miR-6931-3p
NCBP1	hsa-miR-3691-5p
NCBP1	hsa-miR-6928-5p
NCBP1	hsa-miR-192-5p
NCBP1	hsa-miR-6513-5p
NCBP1	hsa-miR-6969-5p
NCBP1	hsa-miR-6739-5p
NCBP1	hsa-miR-221-3p
NCBP1	hsa-miR-412
NCBP1	hsa-miR-624-3p
NCBP1	hsa-miR-2285z
NCBP1	hsa-miR-3077-3p
NCBP1	hsa-miR-544a
NCBP1	hsa-miR-2054
NCBP1	hsa-miR-351-5p
NCBP1	hsa-miR-7219-3p
NCBP1	hsa-miR-211-5p
NCBP1	hsa-miR-509-5p
NCBP1	hsa-miR-6907-5p
NCBP1	hsa-miR-598-5p
NCBP1	hsa-miR-4720-3p
NCBP1	hsa-miR-7195-5p
NCBP1	hsa-miR-548az-3p
NCBP1	hsa-miR-548ad-5p
NCBP1	hsa-miR-483-3p.1
NCBP1	hsa-miR-550
NCBP1	hsa-miR-548am-3p

NCBP1	hsa-miR-4256
NCBP1	hsa-miR-2369
NCBP1	hsa-miR-190-3p
NCBP1	hsa-miR-7033-5p
NCBP1	hsa-miR-2284x
NCBP1	hsa-miR-6913-3p
NCBP1	hsa-miR-500-3p
NCBP1	hsa-miR-6504-3p
NCBP1	hsa-miR-7268-5p
NCBP1	hsa-miR-2301
NCBP1	hsa-miR-182-3p
NCBP1	hsa-miR-3560
NCBP1	hsa-miR-337-5p
NCBP1	hsa-miR-1343-5p
NCBP1	hsa-miR-2284k
NCBP1	hsa-miR-3154
NCBP1	hsa-miR-362-5p
NCBP1	hsa-miR-410
NCBP1	hsa-miR-548c-3p
NCBP1	hsa-miR-323
NCBP1	hsa-miR-550a-3p
NCBP1	hsa-miR-555
NCBP1	hsa-miR-8093
NCBP1	hsa-miR-2402
NCBP1	hsa-miR-6814-5p
NCBP1	hsa-miR-8871
NCBP1	hsa-miR-3613-3p
NCBP1	hsa-miR-24-5p
NCBP1	hsa-miR-6920-5p
NCBP1	hsa-miR-495-3p
NCBP1	hsa-miR-628-5p
NCBP1	hsa-miR-23
NCBP1	hsa-miR-155-5p
NCBP1	hsa-miR-7851-3p
NCBP1	hsa-miR-7279-5p
NCBP1	hsa-miR-9-5p
NCBP1	hsa-miR-570-3p
NCBP1	hsa-miR-206
NCBP1	hsa-miR-3611
NCBP1	hsa-miR-25-3p
NCBP1	hsa-miR-8067
NCBP1	hsa-miR-496
NCBP1	hsa-miR-6859-5p
NCBP1	hsa-miR-2397-3p
NCBP1	hsa-miR-4434
NCBP1	hsa-miR-1299

NCBP1	hsa-miR-144-3p
NCBP1	hsa-miR-6122-5p
NCBP1	hsa-miR-7387-5p
NCBP1	hsa-miR-6792-5p
NCBP1	hsa-miR-7005-5p
NCBP1	hsa-miR-500b-5p
NCBP1	hsa-miR-7166-5p
NCBP1	hsa-miR-3127-3p
NCBP1	hsa-miR-361-5p
NCBP1	hsa-miR-26-5p
NCBP1	hsa-miR-6971-3p
NCBP1	hsa-miR-465-5p
NCBP1	hsa-miR-4446-5p
NCBP1	hsa-miR-629-5p
NCBP1	hsa-miR-21-5p
NCBP1	hsa-miR-499-5p
NCBP1	hsa-miR-6764-3p
NCBP1	hsa-miR-548ae-3p
NCBP1	hsa-miR-2364
NCBP1	hsa-miR-145-5p
NCBP1	hsa-miR-6502-5p
NCBP1	hsa-miR-632
NCBP1	hsa-miR-105-5p
NCBP1	hsa-miR-362-3p
NCBP1	hsa-miR-3135b
NCBP1	hsa-miR-669hk-3p
NCBP1	hsa-miR-423-3p
NCBP1	hsa-miR-4793-5p
NCBP1	hsa-miR-92-3p
NCBP1	hsa-miR-7040-3p
NCBP1	hsa-miR-878
NCBP1	hsa-miR-465a-5p
NCBP1	hsa-miR-578
NCBP1	hsa-miR-34a-3p
NCBP1	hsa-miR-374a-5p
NCBP1	hsa-miR-7026-3p
NCBP1	hsa-miR-7067-5p
NCBP1	hsa-miR-201-3p
NCBP1	hsa-miR-7169-5p
NCBP1	hsa-miR-613
NCBP1	hsa-miR-504
NCBP1	hsa-miR-520-3p
NCBP1	hsa-miR-693-3p
NCBP1	hsa-miR-6867-5p
NCBP1	hsa-miR-4540
NCBP1	hsa-miR-6778-3p

NCBP1	hsa-miR-6368
NCBP1	hsa-miR-4691-3p
NCBP1	hsa-miR-3664-5p
NCBP1	hsa-miR-1273g-3p
NCBP1	hsa-miR-6855-5p
NCBP1	hsa-miR-4751
NCBP1	hsa-miR-548b-3p
NCBP1	hsa-miR-630
NCBP1	hsa-miR-577-3p
NCBP1	hsa-miR-7360-3p
NCBP1	hsa-miR-7112-3p
NCBP1	hsa-miR-2399-3p
NCBP1	hsa-miR-1302
NCBP1	hsa-miR-8065
NCBP1	hsa-miR-378
NCBP1	hsa-miR-152-5p
NCBP1	hsa-miR-4496
NCBP1	hsa-miR-141-5p
NCBP1	hsa-miR-93-5p
NCBP1	hsa-miR-452-3p
NCBP1	hsa-miR-6354
NCBP1	hsa-miR-106b-5p
NCBP1	hsa-miR-7856-5p
NCBP1	hsa-miR-431-5p
NCBP1	hsa-miR-3201
NCBP1	hsa-miR-6764-5p
NCBP1	hsa-miR-375
NCBP1	hsa-miR-7a-2-3p
NCBP1	hsa-miR-216c-5p
NCBP1	hsa-miR-548c-3p
NCBP1	hsa-miR-7193-5p
NCBP1	hsa-miR-4731-5p
NCBP1	hsa-miR-7185-5p
NCBP1	hsa-miR-7373d-3p
NCBP1	hsa-miR-556-5p
NCBP1	hsa-miR-548ay-3p
NCBP1	hsa-miR-6876-5p
NCBP1	hsa-miR-589-5p
NCBP1	hsa-miR-3572
NCBP1	hsa-miR-4420
NCBP1	hsa-miR-382-3p
NCBP1	hsa-miR-4491
NCBP1	hsa-miR-4428
NCBP1	hsa-miR-584-5p
NCBP1	hsa-miR-520g-5p
NCBP1	hsa-miR-7034-5p

NCBP1	hsa-miR-7273-3p
NCBP1	hsa-let-7f-1-3p
NCBP1	hsa-miR-3547-5p
NCBP1	hsa-miR-505-3p.1
NCBP1	hsa-miR-367-3p
NCBP1	hsa-miR-4521
NCBP1	hsa-miR-3600
NCBP1	hsa-miR-344b-3p
NCBP1	hsa-miR-516b
NCBP1	hsa-miR-8081
NCBP1	hsa-miR-448-5p
NCBP1	hsa-miR-6967-3p
NCBP1	hsa-miR-4652-5p
NCBP1	hsa-miR-5108
NCBP1	hsa-miR-4499
NCBP1	hsa-miR-8796
NCBP1	hsa-miR-471-3p
NCBP1	hsa-miR-19
NCBP1	hsa-miR-4684-5p
NCBP1	hsa-miR-4651
NCBP1	hsa-miR-543-3p
NCBP1	hsa-miR-548o-5p
NCBP1	hsa-miR-223-5p
NCBP1	hsa-miR-340-3p
NCBP1	hsa-miR-7186-3p
NCBP1	hsa-miR-619-5p
NCBP1	hsa-miR-369-3p
NCBP1	hsa-miR-649
NCBP1	hsa-miR-295-5p
NCBP1	hsa-miR-5703
NCBP1	hsa-miR-142-3p.2
NCBP1	hsa-miR-7200-5p
NCBP1	hsa-miR-8118
NCBP1	hsa-miR-561-3p
NCBP1	hsa-miR-548ac
NCBP1	hsa-miR-4668-5p
NCBP1	hsa-miR-924
NCBP1	hsa-miR-8113
NCBP1	hsa-miR-608
NCBP1	hsa-miR-1290
NCBP1	hsa-miR-6238
NCBP1	hsa-miR-2904
NCBP1	hsa-miR-4255
NCBP1	hsa-miR-129-5p
NCBP1	hsa-miR-5700
NCBP1	hsa-miR-495-3p

NCBP1	hsa-miR-30-3p
NCBP1	hsa-miR-6858-3p
NCBP1	hsa-miR-6908-3p
NCBP1	hsa-miR-3071-5p
NCBP1	hsa-miR-4668-3p
NCBP1	hsa-miR-7267-3p
NCBP1	hsa-miR-548h-3p
NCBP1	hsa-miR-1976
NCBP1	hsa-miR-6750-3p
NCBP1	hsa-miR-5187-5p
NCBP1	hsa-miR-1468-3p
NCBP1	hsa-miR-449a-3p
NCBP1	hsa-miR-376c-3p
NCBP1	hsa-miR-3928-3p
NCBP1	hsa-miR-2447
NCBP1	hsa-miR-300
NCBP1	hsa-miR-1814a
NCBP1	hsa-miR-25-3p
NCBP1	hsa-miR-5681b
NCBP1	hsa-miR-7193-3p
NCBP1	hsa-miR-488-5p
NCBP1	hsa-miR-323-3p
NCBP1	hsa-miR-8903
NCBP1	hsa-miR-6806-3p
NCBP1	hsa-miR-6388
NCBP1	hsa-miR-669o-5p
NCBP1	hsa-miR-2114-3p
NCBP1	hsa-miR-3908
NCBP1	hsa-miR-5125-5p
NCBP1	hsa-miR-341-3p
NCBP1	hsa-miR-2406
NCBP1	hsa-miR-466mo-3p
NCBP1	hsa-miR-707
NCBP1	hsa-miR-6165
NCBP1	hsa-miR-6870-5p
NCBP1	hsa-miR-222-3p
NCBP1	hsa-miR-4789-5p
NCBP1	hsa-miR-7079-3p
NCBP1	hsa-miR-7157-3p
NCBP1	hsa-miR-498
NCBP1	hsa-miR-8089
NCBP1	hsa-miR-6881-3p
NCBP1	hsa-miR-548au-5p
NCBP1	hsa-miR-6989-5p
NCBP1	hsa-miR-450b-5p
NCBP1	hsa-miR-1968-5p

NCBP1	hsa-miR-6798-5p
NCBP1	hsa-miR-8838
NCBP1	hsa-miR-4700-3p
NCBP1	hsa-miR-106
NCBP1	hsa-miR-1179
NCBP1	hsa-miR-7202-3p
NCBP1	hsa-miR-1-5p
NCBP1	hsa-miR-1892
NCBP1	hsa-miR-7671-5p
NCBP1	hsa-miR-122-5p
NCBP1	hsa-miR-3919
NCBP1	hsa-miR-20-5p
NCBP1	hsa-miR-888-5p
NCBP1	hsa-miR-6854-5p
NCBP1	hsa-miR-3958
NCBP1	hsa-miR-5623-3p
NCBP1	hsa-miR-7288-5p
NCBP1	hsa-miR-6760-3p
NCBP1	hsa-miR-3682-3p
NCBP1	hsa-miR-135-3p
NCBP1	hsa-miR-137-3p
NCBP1	hsa-miR-690
NCBP1	hsa-miR-202-3p
NCBP1	hsa-miR-6499-3p
NCBP1	hsa-miR-5000-3p
NCBP1	hsa-miR-2392
NCBP1	hsa-miR-125
NCBP1	hsa-miR-4690-5p
NCBP1	hsa-miR-369-3p
NCBP1	hsa-miR-548ap-3p
NCBP1	hsa-miR-4659a-3p
NCBP1	hsa-miR-7386i-5p
NCBP1	hsa-miR-1255b-2-3p
NCBP1	hsa-miR-3664-3p
NCBP1	hsa-miR-548ar-3p
NCBP1	hsa-miR-3651
NCBP1	hsa-miR-590-3p
NCBP1	hsa-miR-2114-5p
NCBP1	hsa-miR-465c-5p
NCBP1	hsa-miR-4768-5p
NCBP1	hsa-miR-743-5p
NCBP1	hsa-miR-8881
NCBP1	hsa-miR-1298
NCBP1	hsa-miR-677
NCBP1	hsa-miR-4531
NCBP1	hsa-miR-7-5p

NCBP1	hsa-miR-6523b
NCBP1	hsa-miR-1-3p
NCBP1	hsa-miR-7307-5p
NCBP1	hsa-miR-218-3p
NCBP1	hsa-miR-8817
NCBP1	hsa-miR-367-3p
NCBP1	hsa-miR-1232
NCBP1	hsa-miR-3173-3p
NCBP1	hsa-miR-7370-3p
NCBP1	hsa-miR-7386o-3p
NCBP1	hsa-miR-21-3p
NCBP1	hsa-miR-548aef
NCBP1	hsa-miR-517-5p
NCBP1	hsa-miR-500a-3p
NCBP1	hsa-miR-324
NCBP1	hsa-miR-302c-5p
NCBP1	hsa-miR-500b-3p
NCBP1	hsa-miR-1839-5p
NCBP1	hsa-miR-7026-5p
NCBP1	hsa-miR-29b-2-5p
NCBP1	hsa-miR-4791
NCBP1	hsa-miR-3617-5p
NCBP1	hsa-miR-3163
NCBP1	hsa-miR-7008-3p
NCBP1	hsa-miR-2410
NCBP1	hsa-miR-376e
NCBP1	hsa-miR-6843-3p
NCBP1	hsa-miR-3078-5p
NCBP1	hsa-miR-3572-3p
NCBP1	hsa-miR-449b-3p
NCBP1	hsa-miR-1277-5p
NCBP1	hsa-miR-881-5p
NCBP1	hsa-miR-151-3p
NCBP1	hsa-miR-1262-5p
NCBP1	hsa-miR-8070
NCBP1	hsa-miR-377-3p
NCBP1	hsa-miR-548h-5p
NCBP1	hsa-miR-670-3p
NCBP1	hsa-miR-4520-2-3p
NCBP1	hsa-miR-106-3p
NCBP1	hsa-miR-6832-5p
NCBP1	hsa-miR-7650-3p
NCBP1	hsa-miR-6884-3p
NCBP1	hsa-miR-7245-3p
NCBP1	hsa-miR-511-3p
NCBP1	hsa-miR-627-5p

NCBP1	hsa-miR-505-3p
NCBP1	hsa-miR-6373
NCBP1	hsa-miR-3593-3p
NCBP1	hsa-miR-2382-5p
NCBP1	hsa-miR-634
NCBP1	hsa-miR-5011-5p
NCBP1	hsa-miR-6745
NCBP1	hsa-miR-7330-3p
NCBP1	hsa-miR-151
NCBP1	hsa-miR-384
NCBP1	hsa-miR-660-5p
NCBP1	hsa-miR-4423
NCBP1	hsa-miR-872
NCBP1	hsa-miR-2367-3p
NCBP1	hsa-miR-7043-3p
NCBP1	hsa-miR-4704-3p
NCBP1	hsa-miR-563
NCBP1	hsa-miR-8057
NCBP1	hsa-miR-4659b-5p
NCBP1	hsa-miR-194-5p
NCBP1	hsa-miR-7386d-3p
NCBP1	hsa-miR-548ak
NCBP1	hsa-miR-143-5p
NCBP1	hsa-miR-380
NCBP1	hsa-miR-3199
NCBP1	hsa-miR-3529-3p
NCBP1	hsa-miR-6826-3p
NCBP1	hsa-miR-2480
NCBP1	hsa-miR-3533
NCBP1	hsa-miR-4291
NCBP1	hsa-miR-6988-5p
NCBP1	hsa-miR-6890-5p
NCBP1	hsa-miR-32-5p
NCBP1	hsa-miR-339-5p
NCBP1	hsa-miR-200a-3p
NCBP1	hsa-miR-4422
NCBP1	hsa-miR-7341-5p
NCBP1	hsa-miR-8831
NCBP1	hsa-miR-2897
NCBP1	hsa-miR-5693
NCBP1	hsa-miR-6405
NCBP1	hsa-miR-5010-3p
NCBP1	hsa-miR-548aj-3p
NCBP1	hsa-miR-524-5p
NCBP1	hsa-miR-3680-5p
NCBP1	hsa-miR-6990-3p

NCBP1	hsa-miR-379-3p
NCBP1	hsa-miR-548g-3p
NCBP1	hsa-miR-302
NCBP1	hsa-miR-503-3p
NCBP1	hsa-miR-548aq-3p
NCBP1	hsa-miR-6512-5p
NCBP1	hsa-miR-6074
NCBP1	hsa-let-7a-2-3p
NCBP1	hsa-miR-6776-3p
NCBP1	hsa-miR-1322
NCBP1	hsa-miR-502a
NCBP1	hsa-miR-7154-5p
NCBP1	hsa-miR-125b-2-3p
NCBP1	hsa-miR-7086-5p
NCBP1	hsa-miR-1266
NCBP1	hsa-miR-140-5p
NCBP1	hsa-miR-1236-3p
NCBP1	hsa-miR-880-3p
NCBP1	hsa-miR-7118-3p
NCBP1	hsa-miR-885
NCBP1	hsa-miR-24-3p
NCBP1	hsa-miR-508a
NCBP1	hsa-miR-518-5p
NCBP1	hsa-miR-607
NCBP1	hsa-miR-548d-5p
NCBP1	hsa-miR-2285x
NCBP1	hsa-miR-6955-3p
NCBP1	hsa-miR-3672
NCBP1	hsa-miR-2389
NCBP1	hsa-miR-4726-3p
NCBP1	hsa-miR-411a
NCBP1	hsa-miR-450a-2-3p
NCBP1	hsa-miR-376c-5p
NCBP1	hsa-miR-7398u-3p
NCBP1	hsa-miR-148-3p
NCBP1	hsa-miR-4707-3p
NCBP1	hsa-miR-4328
NCBP1	hsa-miR-759
NCBP1	hsa-miR-496.2
NCBP1	hsa-miR-208a-3p
NCBP1	hsa-miR-6882-5p
NCBP1	hsa-miR-8813
NCBP1	hsa-miR-548aa
NCBP1	hsa-miR-548z
NCBP1	hsa-miR-3470b
NCBP1	hsa-miR-7203-5p

NCBP1	hsa-miR-154-3p
NCBP1	hsa-miR-6391
NCBP1	hsa-miR-6132
NCBP1	hsa-miR-4772-5p
NCBP1	hsa-miR-2285u
NCBP1	hsa-miR-7093-3p
NCBP1	hsa-miR-3127-5p
NCBP1	hsa-miR-31-3p
NCBP1	hsa-miR-3137
NCBP1	hsa-miR-548x-5p
NCBP1	hsa-miR-490-3p
NCBP1	hsa-miR-4725-3p
NCBP1	hsa-miR-4716-5p
NCBP1	hsa-miR-144-3p
NCBP1	hsa-miR-548at-5p
NCBP1	hsa-miR-2370-3p
NCBP1	hsa-miR-4694-3p
NCBP1	hsa-miR-143-3p
NCBP1	hsa-miR-4637
NCBP1	hsa-miR-7063-5p
NCBP1	hsa-miR-4528
NCBP1	hsa-miR-4667
NCBP1	hsa-miR-10-5p
NCBP1	hsa-miR-6997-5p
NCBP1	hsa-miR-4477b
NCBP1	hsa-miR-7386m-3p
NCBP1	hsa-miR-363-3p
NCBP1	hsa-miR-141-3p
NCBP1	hsa-miR-28-5p
NCBP1	hsa-miR-676-5p
NCBP1	hsa-miR-196c-3p
NCBP1	hsa-miR-2371
NCBP1	hsa-miR-6876-3p
NCBP1	hsa-miR-337-3p
NCBP1	hsa-miR-6417
NCBP1	hsa-miR-1180-5p
NCBP1	hsa-miR-4662-5p
NCBP1	hsa-miR-641
NCBP1	hsa-miR-4419b
NCBP1	hsa-let-7g-3p
NCBP1	hsa-miR-4775
NCBP1	hsa-miR-519-5p
NCBP1	hsa-miR-382-5p
NCBP1	hsa-miR-8810
NCBP1	hsa-miR-6131
NCBP1	hsa-miR-3069-3p

NCBP1	hsa-miR-144-5p
NCBP1	hsa-miR-2421
NCBP1	hsa-miR-2387
NCBP1	hsa-miR-1603
NCBP1	hsa-miR-877-5p
NCBP1	hsa-miR-3918
NCBP1	hsa-miR-3171
NCBP1	hsa-miR-1323-5p
NCBP1	hsa-miR-3140-3p
NCBP1	hsa-miR-382-5p
NCBP1	hsa-miR-5586-3p
NCBP1	hsa-miR-5095
NCBP1	hsa-miR-6945-3p
NCBP1	hsa-miR-7317-3p
NCBP1	hsa-miR-4284
NCBP1	hsa-miR-7-3p
NCBP1	hsa-miR-2052
NCBP1	hsa-miR-466b-5p
NCBP1	hsa-miR-2322-5p
NCBP1	hsa-miR-7168-5p
NCBP1	hsa-miR-2417
NCBP1	hsa-miR-584-5p
NCBP1	hsa-miR-5681a
NCBP1	hsa-miR-4667-5p
NCBP1	hsa-miR-6809-3p
NCBP1	hsa-miR-3127-5p
NCBP1	hsa-miR-373-3p
NCBP1	hsa-miR-17-3p
NCBP1	hsa-miR-668
NCBP1	hsa-miR-139-5p
NCBP1	hsa-miR-7225-3p
NCBP1	hsa-miR-4495
NCBP1	hsa-miR-152-3p
NCBP1	hsa-miR-1966-5p
NCBP1	hsa-miR-2284v
NCBP1	hsa-miR-8902
NCBP1	hsa-miR-8845
NCBP1	hsa-miR-186-3p
NCBP1	hsa-miR-218-5p
NCBP1	hsa-miR-4794
NCBP1	hsa-miR-19-3p
NCBP1	hsa-miR-3934-3p
NCBP1	hsa-miR-4662a-3p
NCBP1	hsa-miR-335-5p
NCBP1	hsa-miR-2970-3p
NCBP1	hsa-miR-708-3p

NCBP1	hsa-miR-190a-3p
NCBP1	hsa-miR-200c-5p
NCBP1	hsa-miR-6757-5p
NCBP1	hsa-miR-3090-3p
NCBP1	hsa-miR-4433a-3p
NCBP1	hsa-miR-5691
NCBP1	hsa-miR-3929
NCBP1	hsa-miR-516b-5p
NCBP1	hsa-miR-1296-3p
NCBP1	hsa-miR-2442
NCBP1	hsa-miR-291-5p
NCBP1	hsa-miR-1272
NCBP1	hsa-miR-6969-3p
NCBP1	hsa-miR-5089-5p
NCBP1	hsa-miR-3112-5p
NCBP1	hsa-miR-346
NCBP1	hsa-miR-675-3p
NCBP1	hsa-miR-7844-5p
NCBP1	hsa-miR-135b-3p
NCBP1	hsa-miR-6332
NCBP1	hsa-miR-7291-3p
NCBP1	hsa-miR-299-5p
NCBP1	hsa-miR-669a-3-3p
NCBP1	hsa-miR-1542-3p
NCBP1	hsa-miR-8850
NCBP1	hsa-miR-3915
NCBP1	hsa-miR-1251
NCBP1	hsa-miR-5000-5p
NCBP1	hsa-miR-3121-3p
NCBP1	hsa-miR-330-3p
NCBP1	hsa-miR-2335
NCBP1	hsa-miR-3909
NCBP1	hsa-miR-6216
NCBP1	hsa-miR-550b-2-5p
NCBP1	hsa-miR-6505-5p
NCBP1	hsa-miR-3973
NCBP1	hsa-miR-1265
NCBP1	hsa-miR-6904-5p
NCBP1	hsa-miR-2284n
NCBP1	hsa-miR-548a-3p
NCBP1	hsa-miR-7386k-3p
NCBP1	hsa-miR-6524
NCBP1	hsa-miR-6824-3p
NCBP1	hsa-miR-380-3p
NCBP1	hsa-miR-323a-3p
NCBP1	hsa-miR-216b-3p

NCBP1	hsa-miR-4750-3p
NCBP1	hsa-miR-4277
NCBP1	hsa-miR-4520-3p
NCBP1	hsa-miR-3580-3p
NCBP1	hsa-miR-802-5p
NCBP1	hsa-miR-133c
NCBP1	hsa-miR-7111-5p
NCBP1	hsa-miR-7119-3p
NCBP1	hsa-miR-548ar-5p
NCBP1	hsa-miR-3065-5p
NCBP1	hsa-miR-138-3p
NCBP1	hsa-miR-5697
NCBP1	hsa-miR-501-3p
NCBP1	hsa-miR-374b-5p
NCBP1	hsa-miR-7281-3p
NCBP1	hsa-miR-6839-3p
NCBP1	hsa-miR-3084-5p
NCBP1	hsa-miR-3663-5p
NCBP1	hsa-miR-199-1-5p
NCBP1	hsa-miR-3606-3p
NCBP1	hsa-miR-106-5p
NCBP1	hsa-miR-1844
NCBP1	hsa-miR-187-3p
NCBP1	hsa-miR-466i-3p
NCBP1	hsa-miR-548y
NCBP1	hsa-miR-466f-3p
NCBP1	hsa-miR-3145-3p
NCBP1	hsa-miR-23-3p
NCBP1	hsa-miR-6335
NCBP1	hsa-miR-7393-3p
NCBP1	hsa-miR-1251-5p
NCBP1	hsa-miR-4436b-5p
NCBP1	hsa-miR-124-3p
NCBP1	hsa-miR-7211-5p
NCBP1	hsa-miR-532-3p
NCBP1	hsa-miR-470-5p
NCBP1	hsa-miR-1929-5p
NCBP1	hsa-miR-7850-5p
NCBP1	hsa-miR-3091-3p
NCBP1	hsa-miR-6902-5p
NCBP1	hsa-miR-222-5p
NCBP1	hsa-miR-139-3p
NCBP1	hsa-miR-4795-3p
NCBP1	hsa-miR-2488
NCBP1	hsa-miR-365a-3p
NCBP1	hsa-miR-1931

NCBP1	hsa-miR-2284-5p
NCBP1	hsa-miR-23c
NCBP1	hsa-miR-410-3p
NCBP1	hsa-miR-470-3p
NCBP1	hsa-miR-344h-3p
NCBP1	hsa-miR-2284l
NCBP1	hsa-miR-513a-3p
NCBP1	hsa-miR-1185-5p
NCBP1	hsa-miR-3089-3p
NCBP1	hsa-miR-5001-5p
NCBP1	hsa-miR-1206
NCBP1	hsa-miR-2284c
NCBP1	hsa-miR-5003-5p
NCBP1	hsa-miR-7343-3p
NCBP1	hsa-miR-4698
NCBP1	hsa-miR-1273d
NCBP1	hsa-miR-432-5p
NCBP1	hsa-miR-711
NCBP1	hsa-miR-3686
NCBP1	hsa-miR-669n
NCBP1	hsa-miR-672-3p
NCBP1	hsa-miR-371a-5p
NCBP1	hsa-miR-20b-3p
NCBP1	hsa-miR-7205-3p
NCBP1	hsa-miR-451b
NCBP1	hsa-miR-19-5p
NCBP1	hsa-miR-6974-3p
NCBP1	hsa-miR-6997-3p
NCBP1	hsa-miR-673-3p
NCBP1	hsa-miR-192-5p
NCBP1	hsa-miR-561-3p
NCBP1	hsa-miR-1540-5p
NCBP1	hsa-miR-7075-3p
NCBP1	hsa-miR-7394-3p
NCBP1	hsa-miR-365-3p
NCBP1	hsa-miR-96-3p
NCBP1	hsa-miR-599
NCBP1	hsa-miR-7315-5p
NCBP1	hsa-miR-548bb-3p
NCBP1	hsa-miR-6975-5p
NCBP1	hsa-miR-146a-3p
NCBP1	hsa-miR-6730-3p
NCBP1	hsa-miR-342-3p
NCBP1	hsa-miR-1434-5p
NCBP1	hsa-miR-520gh
NCBP1	hsa-miR-6827-5p

NCBP1	hsa-miR-8904a
NCBP1	hsa-miR-6080
NCBP1	hsa-miR-2110
NCBP1	hsa-miR-511-5p
NCBP1	hsa-miR-6536
NCBP1	hsa-miR-656
NCBP1	hsa-miR-6748-3p
NCBP1	hsa-miR-6833-3p
NCBP1	hsa-miR-1178-5p
NCBP1	hsa-miR-490-3p
NCBP1	hsa-miR-6813-5p
NCBP1	hsa-miR-587
NCBP1	hsa-miR-140-5p
NCBP1	hsa-miR-409
NCBP1	hsa-miR-512
NCBP1	hsa-miR-4261
NCBP1	hsa-miR-7296-5p
NCBP1	hsa-miR-4476
NCBP1	hsa-miR-32-3p
NCBP1	hsa-miR-322-3p
NCBP1	hsa-miR-126-5p
NCBP1	hsa-miR-7-5p
NCBP1	hsa-miR-376ab
NCBP1	hsa-miR-6861-5p
NCBP1	hsa-miR-302a-5p
NCBP1	hsa-miR-1544-3p
NCBP1	hsa-miR-709
NCBP1	hsa-miR-7231-5p
NCBP1	hsa-miR-6821-3p
NCBP1	hsa-miR-3161
NCBP1	hsa-miR-2681-3p
NCBP1	hsa-miR-1905
NCBP1	hsa-miR-3550
NCBP1	hsa-miR-3925-5p
NCBP1	hsa-miR-20
NCBP1	hsa-miR-8083
NCBP1	hsa-miR-876-5p
NCBP1	hsa-miR-3607-3p
NCBP1	hsa-miR-7689-3p
NCBP1	hsa-miR-8063
NCBP1	hsa-miR-548j-5p
NCBP1	hsa-miR-511
NCBP1	hsa-miR-206-3p
NCBP1	hsa-let-7f-2-3p
NCBP1	hsa-miR-606
NCBP1	hsa-miR-568

NCBP1	hsa-miR-2296
NCBP1	hsa-miR-181b-1-3p
NCBP1	hsa-miR-514a-3p
NCBP1	hsa-miR-4708-3p
NCBP1	hsa-miR-769-3p
NCBP1	hsa-miR-514a-5p
NCBP1	hsa-miR-6946-5p
NCBP1	hsa-miR-6072
NCBP1	hsa-miR-3121-3p
NCBP1	hsa-miR-628-3p
NCBP1	hsa-miR-548w
NCBP1	hsa-miR-5120
NCBP1	hsa-miR-132-3p
NCBP1	hsa-miR-101-3p
NCBP1	hsa-miR-20-3p
NCBP1	hsa-miR-3167-3p
NCBP1	hsa-miR-376a-3p
NCBP1	hsa-miR-570-3p
NCBP1	hsa-miR-7005-3p
NCBP1	hsa-miR-3153
NCBP1	hsa-miR-204-5p
NCBP1	hsa-miR-576-5p
NCBP1	hsa-miR-372-5p
NCBP1	hsa-miR-1185-3p
NCBP1	hsa-miR-3162-5p
NCBP1	hsa-miR-496b
NCBP1	hsa-miR-548az-5p
NCBP1	hsa-miR-5003-3p
NCBP1	hsa-miR-516-3p
NCBP1	hsa-miR-7382-3p
NCBP1	hsa-miR-548e-3p
NCBP1	hsa-miR-3671
NCBP1	hsa-miR-6790-3p
NCBP1	hsa-miR-7031-5p
NCBP1	hsa-miR-8840
NCBP1	hsa-miR-584-3p
NCBP1	hsa-miR-548x-3p
NCBP1	hsa-miR-2484
NCBP1	hsa-miR-548m
NCBP1	hsa-miR-5692bc
NCBP1	hsa-miR-660-3p
NCBP1	hsa-miR-181-3p
NCBP1	hsa-miR-7222-3p
NCBP1	hsa-miR-6787-3p
NCBP1	hsa-miR-7307-3p
NCBP1	hsa-miR-193b-3p

NCBP1	hsa-miR-6856-5p
NCBP1	hsa-miR-625-3p
NCBP1	hsa-miR-302c-3p
NCBP1	hsa-miR-499b-5p
NCBP1	hsa-miR-7304-3p
NCBP1	hsa-miR-3571
NCBP1	hsa-miR-374
NCBP1	hsa-miR-665-3p
NCBP1	hsa-miR-22-3p
NCBP1	hsa-miR-7334-3p
NCBP1	hsa-miR-1805-5p
NCBP1	hsa-miR-4421
NCBP1	hsa-miR-7165-3p
NCBP1	hsa-miR-183-3p
NCBP1	hsa-miR-2366
NCBP1	hsa-miR-7651-5p
NCBP1	hsa-miR-1643b-5p
NCBP1	hsa-miR-345-3p
NCBP1	hsa-miR-4752
NCBP1	hsa-miR-363-5p
NCBP1	hsa-miR-7386n-3p
NCBP1	hsa-miR-489-5p
NCBP1	hsa-miR-320-3p
NCBP1	hsa-miR-872-3p
NCBP1	hsa-miR-33-3p
NCBP1	hsa-miR-6977-3p
NCBP1	hsa-miR-7266-5p
NCBP1	hsa-miR-8098
NCBP1	hsa-miR-548cej-3p
NCBP1	hsa-miR-548j-3p
NCBP1	hsa-miR-3149
NCBP1	hsa-miR-154-5p
NCBP1	hsa-miR-488-3p
NCBP1	hsa-miR-450b
NCBP1	hsa-miR-1229-5p
NCBP1	hsa-miR-7182-3p
NCBP1	hsa-miR-6501-3p
NCBP1	hsa-miR-4430
NCBP1	hsa-miR-519
NCBP1	hsa-miR-520
NCBP1	hsa-miR-4492
NCBP1	hsa-miR-6962-5p
NCBP1	hsa-miR-93-3p
NCBP1	hsa-miR-9a-3p
NCBP1	hsa-miR-6891-3p
NCBP1	hsa-miR-150-5p

NCBP1	hsa-miR-4659b-3p
NCBP1	hsa-miR-6853-3p
NCBP1	hsa-miR-4699-3p
NCBP1	hsa-miR-6525
NCBP1	hsa-miR-3144-3p
NCBP1	hsa-miR-548aj-5p
NCBP1	hsa-miR-448
NCBP1	hsa-miR-7172-3p
NCBP1	hsa-miR-6864-3p
NCBP1	hsa-miR-8869
NCBP1	hsa-miR-6875-3p
NCBP1	hsa-miR-762
NCBP1	hsa-miR-7681-3p
NCBP1	hsa-miR-512-3p
NCBP1	hsa-miR-6974-5p
NCBP1	hsa-miR-4524a-3p
NCBP1	hsa-miR-7975
NCBP1	hsa-miR-181-5p
NCBP1	hsa-miR-3064-3p
NCBP1	hsa-miR-383
NCBP1	hsa-miR-548c-5p
NCBP1	hsa-miR-7157-5p
NCBP1	hsa-miR-9-3-3p
NCBP1	hsa-miR-3658
NCBP1	hsa-miR-291
NCBP1	hsa-miR-294
NCBP1	hsa-miR-8803
NCBP1	hsa-miR-215
NCBP1	hsa-miR-6869-5p
NCBP1	hsa-miR-3108-3p
NCBP1	hsa-miR-6852-5p
NCBP1	hsa-miR-656-3p
NCBP1	hsa-miR-7000-3p
NCBP1	hsa-miR-154a
NCBP1	hsa-miR-5004-3p
NCBP1	hsa-miR-2319
NCBP1	hsa-miR-491-5p
NCBP1	hsa-miR-7177-5p
NCBP1	hsa-miR-2460
NCBP1	hsa-miR-4735-5p
NCBP1	hsa-miR-548t-5p
NCBP1	hsa-miR-7977
NCBP1	hsa-miR-342-3p
NCBP1	hsa-miR-4478
NCBP1	hsa-miR-1208
NCBP1	hsa-miR-411-5p.1

NCBP1	hsa-miR-153
NCBP1	hsa-miR-4804-3p
NCBP1	hsa-miR-7213-3p
NCBP1	hsa-miR-3622b-3p
NCBP1	hsa-miR-126a-5p
NCBP1	hsa-miR-1301-5p
NCBP1	hsa-miR-1283a
NCBP1	hsa-miR-6340
NCBP1	hsa-miR-143-3p
NCBP1	hsa-miR-146-5p
NCBP1	hsa-miR-4279
NCBP1	hsa-miR-5011-3p
NCBP1	hsa-miR-190b-3p
NCBP1	hsa-miR-218-5p
NCBP1	hsa-miR-362-5p
NCBP1	hsa-miR-3652
NCBP1	hsa-miR-8812
NCBP1	hsa-miR-518-3p
NCBP1	hsa-miR-7337-3p
NCBP1	hsa-miR-504-5p
NCBP1	hsa-miR-200-3p
NCBP1	hsa-miR-7057-5p
NCBP1	hsa-miR-4776-3p
NCBP1	hsa-miR-103-5p
NCBP1	hsa-miR-1231
NCBP1	hsa-miR-450b-3p
NCBP1	hsa-miR-7071-5p
NCBP1	hsa-miR-548i
NCBP1	hsa-miR-2284ab
NCBP1	hsa-miR-2278
NCBP1	hsa-miR-7398a-3p
NCBP1	hsa-miR-194-3p
NCBP1	hsa-miR-1303
NCBP1	hsa-miR-6885-3p
NCBP1	hsa-miR-548ap-5p
NCBP1	hsa-miR-1185-5p
NCBP1	hsa-miR-129b-5p
NCBP1	hsa-miR-188-5p
NCBP1	hsa-miR-1324
NCBP1	hsa-miR-7371d-5p
NCBP1	hsa-miR-4999-3p
NCBP1	hsa-miR-320a
NCBP1	hsa-miR-5590-5p
NCBP1	hsa-miR-3074-3p
NCBP1	hsa-miR-7668-3p
NCBP1	hsa-miR-767-5p

NCBP1	hsa-miR-1304-3p
NCBP1	hsa-miR-208-3p
NCBP1	hsa-miR-1306-5p
NCBP1	hsa-miR-3653-3p
NCBP1	hsa-miR-7012-5p
NCBP1	hsa-miR-8898
NCBP1	hsa-miR-2343
NCBP1	hsa-miR-4477a
NCBP1	hsa-miR-6856-3p
NCBP1	hsa-miR-548p
NCBP1	hsa-miR-2284j
NCBP1	hsa-miR-1224
NCBP1	hsa-miR-135ab-3p
NCBP1	hsa-miR-329-3p
NCBP1	hsa-miR-3586-5p
NCBP1	hsa-miR-8060
NCBP1	hsa-miR-3132
NCBP1	hsa-miR-7045-3p
NCBP1	hsa-miR-378a-5p
NCBP1	hsa-miR-4704-5p
NCBP1	hsa-miR-8825
NCBP1	hsa-miR-665
NCBP1	hsa-miR-3200-5p
NCBP1	hsa-miR-30a-3p
NCBP1	hsa-miR-7011-3p
NCBP1	hsa-miR-1-3p
NCBP1	hsa-miR-6715-3p
NCBP1	hsa-miR-1226-3p
NCBP1	hsa-miR-7234-5p
NCBP1	hsa-miR-6366
NCBP1	hsa-miR-130b-5p
NCBP1	hsa-miR-30-5p
NCBP1	hsa-miR-550-5p
NCBP1	hsa-miR-199
NCBP1	hsa-miR-7262-3p
NCBP2	hsa-miR-148a-5p
NCBP2	hsa-miR-879-5p
NCBP2	hsa-miR-3082-3p
NCBP2	hsa-miR-669b-5p
NCBP2	hsa-miR-7-3p
NCBP2	hsa-miR-344-3p
NCBP2	hsa-miR-494-3p
NCBP2	hsa-miR-483-3p
NCBP2	hsa-miR-6418-5p
NCBP2	hsa-miR-326-3p
NCBP2	hsa-miR-203b-5p

NCBP2	hsa-miR-6832-3p
NCBP2	hsa-miR-7398s-3p
NCBP2	hsa-miR-3157-5p
NCBP2	hsa-miR-147-5p
NCBP2	hsa-miR-466b-5p
NCBP2	hsa-miR-509-3p
NCBP2	hsa-miR-545-5p
NCBP2	hsa-miR-6800-5p
NCBP2	hsa-miR-1839
NCBP2	hsa-miR-3653-5p
NCBP2	hsa-miR-706
NCBP2	hsa-miR-2358
NCBP2	hsa-miR-8838
NCBP2	hsa-miR-1668
NCBP2	hsa-miR-2468
NCBP2	hsa-miR-291-5p
NCBP2	hsa-miR-135-5p
NCBP2	hsa-miR-129-5p
NCBP2	hsa-miR-6338
NCBP2	hsa-miR-101-3p.2
NCBP2	hsa-miR-3938-3p
NCBP2	hsa-miR-3171
NCBP2	hsa-miR-7152-5p
NCBP2	hsa-miR-4446-5p
NCBP2	hsa-miR-4750-3p
NCBP2	hsa-miR-21-5p
NCBP2	hsa-miR-183-5p
NCBP2	hsa-miR-363-3p
NCBP2	hsa-miR-7221-5p
NCBP2	hsa-miR-744-3p
NCBP2	hsa-miR-7042-5p
NCBP2	hsa-miR-494
NCBP2	hsa-miR-4493
NCBP2	hsa-miR-3154
NCBP2	hsa-miR-6746-5p
NCBP2	hsa-miR-4683
NCBP2	hsa-miR-2284-5p
NCBP2	hsa-miR-7192-3p
NCBP2	hsa-miR-2284d
NCBP2	hsa-miR-7850-5p
NCBP2	hsa-miR-1584-3p
NCBP2	hsa-miR-1892
NCBP2	hsa-miR-7074-5p
NCBP2	hsa-miR-7288-3p
NCBP2	hsa-miR-6966-3p
NCBP2	hsa-miR-3073a-3p

NCBP2	hsa-miR-6908-5p
NCBP2	hsa-miR-6973a-3p
NCBP2	hsa-miR-3659
NCBP2	hsa-miR-378
NCBP2	hsa-miR-7172-3p
NCBP2	hsa-miR-498
NCBP2	hsa-miR-4460
NCBP2	hsa-miR-3152-3p
NCBP2	hsa-miR-16-3p
NCBP2	hsa-miR-1958
NCBP2	hsa-miR-93-3p
NCBP2	hsa-miR-8085
NCBP2	hsa-miR-3680-3p
NCBP2	hsa-miR-378-3p
NCBP2	hsa-miR-3654
NCBP2	hsa-miR-642
NCBP2	hsa-miR-877-5p
NCBP2	hsa-miR-548bb-3p
NCBP2	hsa-miR-942-5p
NCBP2	hsa-miR-3162-3p
NCBP2	hsa-miR-6903-5p
NCBP2	hsa-miR-6350
NCBP2	hsa-miR-2406
NCBP2	hsa-miR-19
NCBP2	hsa-miR-6404
NCBP2	hsa-miR-655
NCBP2	hsa-miR-9-3-3p
NCBP2	hsa-miR-888-5p
NCBP2	hsa-miR-7229-3p
NCBP2	hsa-miR-518-5p
NCBP2	hsa-miR-873-5p
NCBP2	hsa-let-7-3p
NCBP2	hsa-miR-6988-3p
NCBP2	hsa-miR-873a-3p
NCBP2	hsa-miR-6326
NCBP2	hsa-miR-216a
NCBP2	hsa-miR-3133
NCBP2	hsa-miR-548ah-3p
NCBP2	hsa-miR-592-5p
NCBP2	hsa-miR-5007-3p
NCBP2	hsa-miR-4639-5p
NCBP2	hsa-miR-877-3p
NCBP2	hsa-miR-1208
NCBP2	hsa-miR-4709-5p
NCBP2	hsa-miR-222-5p
NCBP2	hsa-miR-582-5p

NCBP2	hsa-miR-549-3p
NCBP2	hsa-miR-1304-5p
NCBP2	hsa-miR-340-5p
NCBP2	hsa-miR-222-3p
NCBP2	hsa-miR-636
NCBP2	hsa-miR-208a-3p
NCBP2	hsa-miR-598-3p
NCBP2	hsa-miR-205-3p
NCBP2	hsa-miR-548bd-3p
NCBP2	hsa-miR-4270
NCBP2	hsa-miR-129-2-3p
NCBP2	hsa-miR-6381
NCBP2	hsa-miR-148b-5p
NCBP2	hsa-miR-344
NCBP2	hsa-miR-141-5p
NCBP2	hsa-miR-126-3p.2
NCBP2	hsa-miR-520gh
NCBP2	hsa-miR-6955-3p
NCBP2	hsa-miR-3120-5p
NCBP2	hsa-miR-548c-3p
NCBP2	hsa-miR-487b-5p
NCBP2	hsa-miR-6795-3p
NCBP2	hsa-miR-802
NCBP2	hsa-miR-330-3p
NCBP2	hsa-miR-6499-3p
NCBP2	hsa-miR-425
NCBP2	hsa-miR-616-5p
NCBP2	hsa-miR-2431-5p
NCBP2	hsa-miR-452-3p
NCBP2	hsa-miR-1388-5p
NCBP2	hsa-miR-6072
NCBP2	hsa-miR-6616-5p
NCBP2	hsa-miR-486-5p
NCBP2	hsa-miR-431-5p
NCBP2	hsa-miR-2883
NCBP2	hsa-miR-7194-3p
NCBP2	hsa-miR-1267
NCBP2	hsa-miR-3542
NCBP2	hsa-miR-5699-5p
NCBP2	hsa-miR-150-5p
NCBP2	hsa-miR-942-3p
NCBP2	hsa-miR-7114-5p
NCBP2	hsa-miR-6943-3p
NCBP2	hsa-miR-6912-3p
NCBP2	hsa-miR-219-5p
NCBP2	hsa-miR-1237-3p

NCBP2	hsa-miR-759
NCBP2	hsa-miR-3586-3p
NCBP2	hsa-miR-499b-5p
NCBP2	hsa-miR-344g-3p
NCBP2	hsa-miR-425-5p
NCBP2	hsa-miR-1324
NCBP2	hsa-miR-4291
NCBP2	hsa-miR-605-3p
NCBP2	hsa-miR-136-5p
NCBP2	hsa-miR-101-3p.1
NCBP2	hsa-miR-6736-3p
NCBP2	hsa-miR-6501-5p
NCBP2	hsa-miR-6960-3p
NCBP2	hsa-miR-2113
NCBP2	hsa-miR-877-5p
NCBP2	hsa-miR-3081-5p
NCBP2	hsa-miR-7214-5p
NCBP2	hsa-miR-125b-2-3p
NCBP2	hsa-miR-1639
NCBP2	hsa-miR-7386g-3p
NCBP2	hsa-miR-1188-5p
NCBP2	hsa-miR-7166-3p
NCBP2	hsa-miR-24-5p
NCBP2	hsa-miR-510-3p
NCBP2	hsa-miR-499b-3p
NCBP2	hsa-miR-513a
NCBP2	hsa-miR-642a-5p
NCBP2	hsa-miR-7371b-5p
NCBP2	hsa-miR-548at-3p
NCBP2	hsa-miR-511-5p
NCBP2	hsa-miR-3692-5p
NCBP2	hsa-miR-548
NCBP2	hsa-miR-4490
NCBP2	hsa-miR-4689
NCBP2	hsa-miR-5685
NCBP2	hsa-miR-6505-5p
NCBP2	hsa-miR-6883-3p
NCBP2	hsa-miR-873-5p.2
NCBP2	hsa-miR-6728-5p
NCBP2	hsa-miR-1562-5p
NCBP2	hsa-miR-6935-3p
NCBP2	hsa-miR-548am-3p
NCBP2	hsa-miR-539-5p
NCBP2	hsa-miR-376ab-3p
NCBP2	hsa-miR-653-5p
NCBP2	hsa-miR-217

NCBP2	hsa-miR-329
NCBP2	hsa-miR-190a-3p
NCBP2	hsa-miR-204-5p
NCBP2	hsa-miR-1546-5p
NCBP2	hsa-miR-31-3p
NCBP2	hsa-miR-631
NCBP2	hsa-miR-873-5p
NCBP2	hsa-miR-105b
NCBP2	hsa-miR-29b-5p
NCBP2	hsa-miR-561-3p
NCBP2	hsa-miR-331-3p
NCBP2	hsa-miR-7059-3p
NCBP2	hsa-miR-2297
NCBP2	hsa-miR-2400
NCBP2	hsa-miR-190-3p
NCBP2	hsa-miR-500b-5p
NCBP2	hsa-miR-30-5p
NCBP2	hsa-miR-548an
NCBP2	hsa-miR-8097
NCBP2	hsa-miR-1298
NCBP2	hsa-miR-141-3p
NCBP2	hsa-miR-8073
NCBP2	hsa-miR-3112-5p
NCBP2	hsa-miR-5100
NCBP2	hsa-miR-7657-3p
NCBP2	hsa-miR-7053-5p
NCBP2	hsa-miR-211-3p
NCBP2	hsa-miR-7670-5p
NCBP2	hsa-miR-520
NCBP2	hsa-miR-539-5p
NCBP2	hsa-miR-3685
NCBP2	hsa-miR-1225-3p
NCBP2	hsa-miR-185-5p
NCBP2	hsa-miR-3915
NCBP2	hsa-miR-487a-5p
NCBP2	hsa-miR-4289
NCBP2	hsa-miR-325-3p
NCBP2	hsa-miR-26a-3p
NCBP2	hsa-miR-1812-3p
NCBP2	hsa-miR-499a-3p
NCBP2	hsa-miR-433
NCBP2	hsa-miR-224-3p
NCBP2	hsa-miR-6504-3p
NCBP2	hsa-miR-495-3p
NCBP2	hsa-miR-6507-5p
NCBP2	hsa-miR-136-5p

NCBP2	hsa-miR-383-5p.2
NCBP2	hsa-miR-7646-3p
NCBP2	hsa-miR-299
NCBP2	hsa-miR-194-5p
NCBP2	hsa-miR-6969-5p
NCBP2	hsa-miR-1603
NCBP2	hsa-miR-661
NCBP2	hsa-miR-4686
NCBP2	hsa-miR-3074-5p
NCBP2	hsa-miR-3074-3p
NCBP2	hsa-miR-495-3p
NCBP2	hsa-miR-2287
NCBP2	hsa-miR-2285x
NCBP2	hsa-miR-548h-3p
NCBP2	hsa-miR-871-3p
NCBP2	hsa-miR-344d-2-5p
NCBP2	hsa-miR-3569-5p
NCBP2	hsa-miR-27-3p
NCBP2	hsa-miR-7012-3p
NCBP2	hsa-miR-6744-3p
NCBP2	hsa-miR-514b-5p
NCBP2	hsa-miR-599
NCBP2	hsa-miR-1897-5p
NCBP2	hsa-miR-656
NCBP2	hsa-miR-6854-5p
NCBP2	hsa-miR-2114-3p
NCBP2	hsa-miR-4726-3p
NCBP2	hsa-miR-191-3p
NCBP2	hsa-miR-1270
NCBP2	hsa-miR-6866-5p
NCBP2	hsa-miR-25-3p
NCBP2	hsa-miR-450a-1-3p
NCBP2	hsa-miR-8854
NCBP2	hsa-miR-1190
NCBP2	hsa-miR-526
NCBP2	hsa-miR-4420
NCBP2	hsa-miR-2284n
NCBP2	hsa-miR-6913-3p
NCBP2	hsa-miR-6858-5p
NCBP2	hsa-miR-7152-3p
NCBP2	hsa-miR-6793-3p
NCBP2	hsa-miR-26-5p
NCBP2	hsa-miR-1692
NCBP2	hsa-miR-3079-3p
NCBP2	hsa-miR-344b-3p
NCBP2	hsa-miR-186-5p

NCBP2	hsa-miR-372-5p
NCBP2	hsa-miR-513a-5p
NCBP2	hsa-miR-5617-5p
NCBP2	hsa-miR-4433b-3p
NCBP2	hsa-miR-4424
NCBP2	hsa-miR-467eh-5p
NCBP2	hsa-miR-7159-5p
NCBP2	hsa-miR-680
NCBP2	hsa-miR-7237-3p
NCBP2	hsa-miR-548ae-3p
NCBP2	hsa-miR-935
NCBP2	hsa-miR-6385
NCBP2	hsa-miR-8841
NCBP2	hsa-miR-377-5p
NCBP2	hsa-miR-2902
NCBP2	hsa-miR-6830-3p
NCBP2	hsa-miR-7180-3p
NCBP2	hsa-miR-8068
NCBP2	hsa-miR-1252-5p
NCBP2	hsa-miR-208-3p
NCBP2	hsa-miR-676-5p
NCBP2	hsa-miR-557
NCBP2	hsa-miR-411-3p
NCBP2	hsa-miR-4758-3p
NCBP2	hsa-miR-3102-3p.2-3p
NCBP2	hsa-miR-6876-5p
NCBP2	hsa-miR-548z
NCBP2	hsa-miR-669e-5p
NCBP2	hsa-miR-33-5p
NCBP2	hsa-miR-6891-3p
NCBP2	hsa-miR-5093
NCBP2	hsa-let-7a-2-3p
NCBP2	hsa-miR-655-5p
NCBP2	hsa-miR-212-5p
NCBP2	hsa-miR-3163
NCBP2	hsa-miR-382-3p
NCBP2	hsa-miR-374c-5p
NCBP2	hsa-miR-124-3p.2
NCBP2	hsa-miR-4484
NCBP2	hsa-miR-33a-3p
NCBP2	hsa-miR-6758-5p
NCBP2	hsa-miR-449-5p
NCBP2	hsa-miR-101a-3p.2
NCBP2	hsa-miR-449c-5p
NCBP2	hsa-miR-187-5p
NCBP2	hsa-miR-1243

NCBP2	hsa-miR-5626-5p
NCBP2	hsa-miR-6720-5p
NCBP2	hsa-miR-2284m
NCBP2	hsa-miR-4643
NCBP2	hsa-miR-3166
NCBP2	hsa-miR-3473f
NCBP2	hsa-miR-497-3p
NCBP2	hsa-miR-671-5p
NCBP2	hsa-miR-574-5p
NCBP2	hsa-miR-4280
NCBP2	hsa-miR-6790-3p
NCBP2	hsa-miR-7021-3p
NCBP2	hsa-miR-2284c
NCBP2	hsa-miR-548aw
NCBP2	hsa-miR-7469-3p
NCBP2	hsa-miR-3190-5p
NCBP2	hsa-miR-3714
NCBP2	hsa-miR-337-3p
NCBP2	hsa-miR-4677-3p
NCBP2	hsa-miR-450b
NCBP2	hsa-miR-548ay-3p
NCBP2	hsa-miR-10a-3p
NCBP2	hsa-miR-7309-3p
NCBP2	hsa-miR-6754-5p
NCBP2	hsa-miR-3577-3p
NCBP2	hsa-miR-6359
NCBP2	hsa-miR-6821-3p
NCBP2	hsa-miR-424-3p
NCBP2	hsa-miR-34bc-3p
NCBP2	hsa-miR-194-5p
NCBP2	hsa-miR-548ac
NCBP2	hsa-miR-3938
NCBP2	hsa-miR-7156-5p
NCBP2	hsa-miR-4682
NCBP2	hsa-miR-1933-3p
NCBP2	hsa-miR-34-5p
NCBP2	hsa-miR-1241
NCBP2	hsa-miR-656-3p
NCBP2	hsa-miR-384
NCBP2	hsa-miR-192-5p
NCBP2	hsa-miR-185-5p
NCBP2	hsa-miR-5186
NCBP2	hsa-miR-6792-3p
NCBP2	hsa-miR-325
NCBP2	hsa-miR-4735-5p
NCBP2	hsa-miR-2114-5p

NCBP2	hsa-miR-548d-5p
NCBP2	hsa-miR-8058
NCBP2	hsa-miR-455-3p.1
NCBP2	hsa-miR-200bc
NCBP2	hsa-miR-4762-3p
NCBP2	hsa-miR-1-3p
NCBP2	hsa-miR-3926
NCBP2	hsa-miR-4323
NCBP2	hsa-miR-1273h-3p
NCBP2	hsa-miR-375
NCBP2	hsa-miR-4302
NCBP2	hsa-miR-101b-3p.2
NCBP2	hsa-miR-6760-3p
NCBP2	hsa-miR-2390
NCBP2	hsa-miR-545-3p
NCBP2	hsa-miR-505-3p
NCBP2	hsa-miR-590-5p
NCBP2	hsa-miR-4668-5p
NCBP2	hsa-miR-2356
NCBP2	hsa-miR-1914-5p
NCBP2	hsa-miR-7350-3p
NCBP2	hsa-miR-381-3p
NCBP2	hsa-miR-6131
NCBP2	hsa-miR-429
NCBP2	hsa-miR-127-5p
NCBP2	hsa-miR-2115-5p
NCBP2	hsa-miR-2284q
NCBP2	hsa-miR-19-5p
NCBP2	hsa-miR-19-3p
NCBP2	hsa-miR-3547-5p
NCBP2	hsa-miR-5579-5p
NCBP2	hsa-miR-463-5p
NCBP2	hsa-miR-7092-3p
NCBP2	hsa-miR-1243-5p
NCBP2	hsa-miR-590-3p
NCBP2	hsa-miR-499b-3p
NCBP2	hsa-miR-8871
NCBP2	hsa-miR-4691-5p
NCBP2	hsa-miR-133c
NCBP2	hsa-miR-92-3p
NCBP2	hsa-miR-7164-5p
NCBP2	hsa-miR-885
NCBP2	hsa-miR-642-3p
NCBP2	hsa-miR-4441
NCBP2	hsa-miR-7350-5p
NCBP2	hsa-miR-4781-3p

NCBP2	hsa-miR-6405
NCBP2	hsa-miR-4263
NCBP2	hsa-let-7f-2-3p
NCBP2	hsa-miR-7179-3p
NCBP2	hsa-miR-943
NCBP2	hsa-miR-7191-5p
NCBP2	hsa-miR-567
NCBP2	hsa-miR-9-3p
NCBP2	hsa-miR-4256
NCBP2	hsa-miR-2285j
NCBP2	hsa-miR-874-3p
NCBP2	hsa-miR-137-3p
NCBP2	hsa-miR-7222-3p
NCBP2	hsa-miR-6537-5p
NCBP2	hsa-miR-3660
NCBP2	hsa-miR-690
NCBP2	hsa-miR-527
NCBP2	hsa-miR-4743-3p
NCBP2	hsa-miR-3580-5p
NCBP2	hsa-miR-2284aa
NCBP2	hsa-miR-518a-5p
NCBP2	hsa-miR-466i-3p
NCBP2	hsa-miR-767
NCBP2	hsa-miR-702-3p
NCBP2	hsa-miR-6509-5p
NCBP2	hsa-miR-5584-5p
NCBP2	hsa-miR-6565-3p
NCBP2	hsa-miR-7118-3p
NCBP2	hsa-miR-507
NCBP2	hsa-miR-5009-5p
NCBP2	hsa-miR-6071
NCBP2	hsa-miR-6582-3p
NCBP2	hsa-miR-4519
NCBP2	hsa-miR-576-5p
NCBP2	hsa-miR-335-3p
NCBP2	hsa-miR-133ab
NCBP2	hsa-miR-377-3p
NCBP2	hsa-miR-670-3p
NCBP2	hsa-miR-1976
NCBP2	hsa-miR-410-3p
NCBP2	hsa-miR-1721
NCBP2	hsa-miR-1972
NCBP2	hsa-miR-4678
NCBP2	hsa-miR-7086-3p
NCBP2	hsa-miR-6876-3p
NCBP2	hsa-miR-2285

NCBP2	hsa-miR-215-5p
NCBP2	hsa-miR-6895-3p
NCBP2	hsa-miR-139-5p
NCBP2	hsa-miR-769-3p
NCBP2	hsa-miR-4677-5p
NCBP2	hsa-miR-677-3p
NCBP2	hsa-miR-369-3p
NCBP2	hsa-miR-22-5p
NCBP2	hsa-miR-3944-5p
NCBP2	hsa-miR-1191b-5p
NCBP2	hsa-miR-7208-3p
NCBP2	hsa-miR-140-5p
NCBP2	hsa-miR-455-3p
NCBP2	hsa-miR-4797-3p
NCBP2	hsa-miR-6826-3p
NCBP2	hsa-miR-2469
NCBP2	hsa-miR-8087
NCBP2	hsa-miR-134-5p
NCBP2	hsa-miR-6802-5p
NCBP2	hsa-miR-4320
NCBP2	hsa-miR-3182
NCBP2	hsa-miR-664
NCBP2	hsa-miR-493-5p
NCBP2	hsa-miR-323
NCBP2	hsa-miR-3661
NCBP2	hsa-miR-141-3p
NCBP2	hsa-miR-24-3p
NCBP2	hsa-miR-6878-3p
NCBP2	hsa-miR-3065-3p
NCBP2	hsa-miR-138-5p
NCBP2	hsa-miR-5193
NCBP2	hsa-miR-7184-3p
NCBP2	hsa-miR-6719-3p
NCBP2	hsa-miR-679
NCBP2	hsa-miR-6515-3p
NCBP2	hsa-miR-4778-5p
NCBP2	hsa-miR-3611
NCBP2	hsa-miR-548c-3p
NCBP2	hsa-miR-7090-3p
NCBP2	hsa-miR-888-5p
NCBP2	hsa-miR-6764-5p
NCBP2	hsa-miR-218a-1-3p
NCBP2	hsa-miR-593-3p
NCBP2	hsa-miR-7678-3p
NCBP2	hsa-miR-5579-3p
NCBP2	hsa-miR-548x-3p

NCBP2	hsa-miR-7013-5p
NCBP2	hsa-miR-302-5p
NCBP2	hsa-miR-187-3p
NCBP2	hsa-miR-548aj-3p
NCBP2	hsa-miR-5124a
NCBP2	hsa-miR-221-3p
NCBP2	hsa-miR-34b-5p
NCBP2	hsa-miR-2337
NCBP2	hsa-miR-6947-5p
NCBP2	hsa-miR-7254-5p
NCBP2	hsa-miR-202-5p
NCBP2	hsa-miR-466c-5p
NCBP2	hsa-miR-3547
NCBP2	hsa-miR-512-3p
NCBP2	hsa-miR-8908e
NCBP2	hsa-let-7c-1-3p
NCBP2	hsa-miR-4659a-3p
NCBP2	hsa-miR-3935
NCBP2	hsa-miR-7075-3p
NCBP2	hsa-miR-33-3p
NCBP2	hsa-miR-6977-3p
NCBP2	hsa-miR-4786-5p
NCBP2	hsa-miR-880-3p
NCBP2	hsa-miR-1775-3p
NCBP2	hsa-miR-5589-3p
NCBP2	hsa-miR-376a-2-5p
NCBP2	hsa-miR-573
NCBP2	hsa-miR-4328
NCBP2	hsa-miR-582-3p
NCBP2	hsa-miR-340-5p
NCBP2	hsa-miR-422
NCBP2	hsa-miR-367-3p
NCBP2	hsa-miR-2365
NCBP2	hsa-miR-543-3p
NCBP2	hsa-miR-7227-5p
NCBP2	hsa-miR-742-3p
NCBP2	hsa-miR-3595
NCBP2	hsa-miR-34
NCBP2	hsa-miR-668
NCBP2	hsa-miR-421
NCBP2	hsa-miR-7163-5p
NCBP2	hsa-miR-6768-3p
NCBP2	hsa-miR-132-3p
NCBP2	hsa-miR-6950-3p
NCBP2	hsa-miR-181b-2-3p
NCBP2	hsa-miR-500-3p

NCBP2	hsa-miR-373-3p
NCBP2	hsa-miR-193-5p
NCBP2	hsa-miR-4326
NCBP2	hsa-miR-1188-3p
NCBP2	hsa-miR-7261-5p
NCBP2	hsa-miR-500
NCBP2	hsa-miR-29-5p
NCBP2	hsa-miR-6874-3p
NCBP2	hsa-miR-674-5p
NCBP2	hsa-miR-2284ab
NCBP2	hsa-miR-4482-5p
NCBP2	hsa-miR-499-5p
NCBP2	hsa-miR-2284k
NCBP2	hsa-miR-549a
NCBP2	hsa-miR-625-5p
NCBP2	hsa-miR-6896-3p
NCBP2	hsa-miR-703
NCBP2	hsa-miR-468-5p
NCBP2	hsa-miR-1205
NCBP2	hsa-miR-6120-5p
NCBP2	hsa-miR-105-5p
NCBP2	hsa-miR-1251-3p
NCBP2	hsa-miR-1252
NCBP2	hsa-miR-5113
NCBP2	hsa-miR-584-5p
NCBP2	hsa-miR-377-3p
NCBP2	hsa-miR-653-3p
NCBP2	hsa-miR-3129-5p
NCBP2	hsa-miR-548j-3p
NCBP2	hsa-miR-7187-3p
NCBP2	hsa-miR-568
NCBP2	hsa-miR-212-3p
NCBP2	hsa-miR-299-5p
NCBP2	hsa-miR-3188
NCBP2	hsa-miR-4732-5p
NCBP2	hsa-miR-186-5p
NCBP2	hsa-miR-373-5p
NCBP2	hsa-miR-700-5p
NCBP2	hsa-miR-3529-3p
NCBP2	hsa-miR-7208-5p
NCBP2	hsa-miR-208-5p
NCBP2	hsa-miR-7091-3p
NCBP2	hsa-miR-3192-3p
NCBP2	hsa-miR-4474-5p
NCBP2	hsa-miR-1912-3p
NCBP2	hsa-miR-676-3p

NCBP2	hsa-miR-892b
NCBP2	hsa-miR-3106-3p
NCBP2	hsa-miR-4476
NCBP2	hsa-miR-7037-5p
NCBP2	hsa-miR-3145-3p
NCBP2	hsa-miR-6892-3p
NCBP2	hsa-miR-6513-5p
NCBP2	hsa-miR-3667-3p
NCBP2	hsa-miR-4272
NCBP2	hsa-miR-192-5p
NCBP2	hsa-miR-7650-3p
NCBP2	hsa-miR-28-3p
NCBP2	hsa-miR-6730-3p
NCBP2	hsa-miR-4267
NCBP2	hsa-miR-598-5p
NCBP2	hsa-miR-133a-5p
NCBP2	hsa-miR-211-5p
NCBP2	hsa-miR-134
NCBP2	hsa-miR-5135
NCBP2	hsa-miR-593-3p
NCBP2	hsa-miR-135
NCBP2	hsa-miR-7062-3p
NCBP2	hsa-miR-4432
NCBP2	hsa-miR-6731-5p
NCBP2	hsa-miR-524-5p
NCBP2	hsa-miR-5692a
NCBP2	hsa-miR-7090-5p
NCBP2	hsa-miR-532-5p
NCBP2	hsa-miR-6875-3p
NCBP2	hsa-miR-5010-3p
NCBP2	hsa-miR-544-5p
NCBP2	hsa-miR-3176
NCBP2	hsa-miR-200-3p
NCBP2	hsa-miR-143-5p
NCBP2	hsa-miR-181-3p
NCBP2	hsa-miR-548s
NCBP2	hsa-miR-186-3p
NCBP2	hsa-miR-5680
NCBP2	hsa-miR-554
NCBP2	hsa-miR-2301
NCBP2	hsa-miR-467b-5p
NCBP2	hsa-miR-452-5p
NCBP2	hsa-miR-518g-3p
NCBP2	hsa-miR-188-3p
NCBP2	hsa-miR-584-5p
NCBP2	hsa-miR-4690-3p

NCBP2	hsa-miR-1237-3p
NCBP2	hsa-miR-6324
NCBP2	hsa-miR-324-5p
NCBP2	hsa-miR-3473be
NCBP2	hsa-miR-6902-5p
NCBP2	hsa-miR-100-3p
NCBP2	hsa-miR-2303
NCBP2	hsa-miR-151-3p
NCBP2	hsa-miR-607
NCBP2	hsa-miR-374-5p
NCBP2	hsa-miR-146a-3p
NCBP2	hsa-miR-4652-3p
NCBP2	hsa-miR-26b-3p
NCBP2	hsa-miR-3066-3p
NCBP2	hsa-miR-7161-5p
NCBP2	hsa-miR-6528
NCBP2	hsa-miR-7847-3p
NCBP2	hsa-miR-337-3p
NCBP2	hsa-miR-543
NCBP2	hsa-miR-8866
NCBP2	hsa-miR-7578
NCBP2	hsa-miR-7680-5p
NCBP2	hsa-miR-6856-5p
NCBP2	hsa-miR-1616
NCBP2	hsa-miR-7088-3p
NCBP2	hsa-miR-4733-5p
NCBP2	hsa-miR-548aq-3p
NCBP2	hsa-miR-2344
NCBP2	hsa-miR-1298-5p
NCBP2	hsa-miR-2284v
NCBP2	hsa-miR-450b-5p
NCBP2	hsa-miR-195-3p
NCBP2	hsa-miR-2397-3p
NCBP2	hsa-miR-7064-5p
NCBP2	hsa-miR-888-3p
NCBP2	hsa-miR-489-3p
NCBP2	hsa-miR-938
NCBP2	hsa-miR-5694
NCBP2	hsa-let-7
NCBP2	hsa-miR-200b-3p
NCBP2	hsa-miR-499b-5p
NCBP2	hsa-miR-891-3p
NCBP2	hsa-miR-556-5p
NCBP2	hsa-miR-1953
NCBP2	hsa-miR-6718-5p
NCBP2	hsa-miR-1814

NCBP2	hsa-miR-203-3p.1
NCBP2	hsa-miR-2332
NCBP2	hsa-miR-223-3p
NCBP2	hsa-miR-486-5p
NCBP2	hsa-miR-142-3p
NCBP2	hsa-miR-1956
NCBP2	hsa-miR-802-5p
NCBP2	hsa-miR-343
NCBP2	hsa-miR-1283a
NCBP2	hsa-miR-548d-3p
NCBP2	hsa-miR-6997-5p
NCBP2	hsa-miR-653
NCBP2	hsa-miR-181b-1-3p
NCBP2	hsa-miR-4504
NCBP2	hsa-miR-34a-3p
NCBP2	hsa-miR-331-5p
NCBP2	hsa-miR-883ab-3p
NCBP2	hsa-miR-7113-3p
NCBP2	hsa-miR-138-5p
NCBP2	hsa-miR-349
NCBP2	hsa-miR-7031-5p
NCBP2	hsa-miR-4650
NCBP2	hsa-miR-6322
NCBP2	hsa-miR-449a
NCBP2	hsa-miR-152-5p
NCBP2	hsa-miR-7666-5p
NCBP2	hsa-miR-3473c
NCBP2	hsa-miR-2421
NCBP2	hsa-miR-7323-3p
NCBP2	hsa-miR-1266-3p
NCBP2	hsa-miR-371a-5p
NCBP2	hsa-miR-570-3p
NCBP2	hsa-miR-6828-5p
NCBP2	hsa-miR-6976-3p
NCBP2	hsa-miR-7002-5p
NCBP2	hsa-miR-3130-5p
NCBP2	hsa-miR-2286
NCBP2	hsa-miR-3078-5p
NCBP2	hsa-miR-200a-3p
NCBP2	hsa-miR-8806
NCBP2	hsa-miR-216b-3p
NCBP2	hsa-miR-7106-3p
NCBP2	hsa-miR-8074
NCBP2	hsa-miR-3620-3p
NCBP2	hsa-miR-3568
NCBP2	hsa-miR-2466-3p

NCBP2	hsa-miR-211-5p
NCBP2	hsa-miR-3583-3p
NCBP2	hsa-miR-6847-5p
NCBP2	hsa-miR-199-3p
NCBP2	hsa-miR-4762-5p
NCBP2	hsa-miR-873-3p
NCBP2	hsa-miR-139-5p
NCBP2	hsa-miR-4722-5p
NCBP2	hsa-miR-339-5p
NCBP2	hsa-miR-1224
NCBP2	hsa-miR-6356
NCBP2	hsa-miR-3147
NCBP2	hsa-miR-7234-5p
NCBP2	hsa-miR-6872-3p
NCBP2	hsa-miR-8853
NCBP2	hsa-miR-2429
NCBP2	hsa-miR-5004-3p
NCBP2	hsa-miR-429-3p
NCBP2	hsa-miR-3662
NCBP2	hsa-miR-668-3p
NCBP2	hsa-miR-9-5p
NCBP2	hsa-miR-337-5p
NCBP2	hsa-miR-548ap-3p
NCBP2	hsa-miR-944
NCBP2	hsa-miR-202-3p
NCBP2	hsa-miR-7154-5p
NCBP2	hsa-miR-19b-5p
NCBP2	hsa-miR-1739
NCBP2	hsa-miR-892-5p
NCBP2	hsa-miR-501-5p
NCBP2	hsa-miR-2452
NCBP2	hsa-miR-6768-5p
NCBP2	hsa-miR-936
NCBP2	hsa-miR-7664-5p
NCBP2	hsa-miR-1544-3p
NCBP2	hsa-miR-4729
NCBP2	hsa-miR-549-5p
NCBP2	hsa-miR-5195-5p
NCBP2	hsa-miR-1254
NCBP2	hsa-miR-2276-5p
NCBP2	hsa-miR-2325
NCBP2	hsa-miR-687
NCBP2	hsa-miR-4799-5p
NCBP2	hsa-miR-8839
NCBP2	hsa-miR-330-3p
NCBP2	hsa-miR-570-3p

NCBP2	hsa-miR-6516
NCBP2	hsa-miR-98-5p
NCBP2	hsa-miR-4717-3p
NCBP2	hsa-miR-6840-3p
NCBP2	hsa-miR-4437
NCBP2	hsa-miR-323-3p
NCBP2	hsa-miR-4679
NCBP2	hsa-miR-548as-3p
NCBP2	hsa-miR-7033-5p
NCBP2	hsa-miR-3587
NCBP2	hsa-miR-6502-3p
NCBP2	hsa-miR-1286
NCBP2	hsa-miR-7198-3p
NCBP2	hsa-miR-7340-5p
NCBP2	hsa-miR-6215
NCBP2	hsa-miR-496-3p
NCBP2	hsa-miR-1240
NCBP2	hsa-miR-626
NCBP2	hsa-miR-144-3p
NCBP2	hsa-miR-1463
NCBP2	hsa-miR-3674
NCBP2	hsa-miR-8118
NCBP2	hsa-miR-2293
NCBP2	hsa-miR-3092-5p
NCBP2	hsa-miR-7201-3p
NCBP2	hsa-miR-694
NCBP2	hsa-miR-7649-3p
NCBP2	hsa-miR-3590-5p
NCBP2	hsa-miR-130a-5p
NCBP2	hsa-miR-371b-5p
NCBP2	hsa-miR-330-3p.2
NCBP2	hsa-miR-7214-3p
NCBP2	hsa-miR-7172-5p
NCBP2	hsa-miR-4282
NCBP2	hsa-miR-6408
NCBP2	hsa-miR-1981-5p
NCBP2	hsa-miR-6750-3p
NCBP2	hsa-miR-6079
NCBP2	hsa-miR-4287
NCBP2	hsa-miR-1285-5p
NCBP2	hsa-miR-5683
NCBP2	hsa-miR-873-5p.1
NCBP2	hsa-miR-7292-3p
NCBP2	hsa-miR-450b-3p
NCBP2	hsa-miR-153-5p
NCBP2	hsa-miR-4273

NCBP2	hsa-miR-7653-3p
NCBP2	hsa-miR-5089-5p
NCBP2	hsa-miR-142
NCBP2	hsa-miR-219-3p
NCBP2	hsa-miR-889-5p
NCBP2	hsa-miR-7232-5p
NCBP2	hsa-miR-1200
NCBP2	hsa-miR-761
NCBP2	hsa-miR-2284j
NCBP2	hsa-miR-3140-3p
NCBP2	hsa-miR-3922-3p
NCBP2	hsa-miR-218-5p
NCBP2	hsa-miR-6368
NCBP2	hsa-miR-579
NCBP2	hsa-miR-532-3p
NCBP2	hsa-miR-23a-5p
NCBP2	hsa-miR-216c-3p
NCBP2	hsa-miR-6975-3p
NCBP2	hsa-miR-466n-3p
NCBP2	hsa-miR-7193-3p
NCBP2	hsa-miR-6973b-3p
NCBP2	hsa-miR-3116
NCBP2	hsa-miR-322-3p
NCBP2	hsa-miR-21-5p
NCBP2	hsa-miR-329-3p
NCBP2	hsa-miR-7060-3p
NCBP2	hsa-miR-203
NCBP2	hsa-miR-6346
NCBP2	hsa-miR-3148
NCBP2	hsa-miR-3713
NCBP2	hsa-miR-3942-3p
NCBP2	hsa-miR-6979-3p
NCBP2	hsa-miR-6749-3p
NCBP2	hsa-miR-3910
NCBP2	hsa-miR-600
NCBP2	hsa-miR-4526
NCBP2	hsa-miR-5586-5p
NCBP2	hsa-miR-7671-5p
NCBP2	hsa-miR-3590-3p
NCBP2	hsa-miR-6888-3p
NCBP2	hsa-miR-1283
NCBP2	hsa-miR-1304-3p
NCBP2	hsa-miR-466
NCBP2	hsa-miR-6126
NCBP2	hsa-miR-3086-5p
NCBP2	hsa-miR-767-5p

NCBP2	hsa-miR-6739-3p
NCBP2	hsa-miR-763
NCBP2	hsa-miR-345-3p
NCBP2	hsa-miR-1281
NCBP2	hsa-miR-2116-5p
NCBP2	hsa-miR-505-3p.2
NCBP2	hsa-miR-4757-5p
NCBP2	hsa-miR-7224-5p
NCBP2	hsa-miR-193-3p
NCBP2	hsa-miR-204-5p
NCBP2	hsa-miR-32-3p
NCBP2	hsa-miR-129-5p
NCBP2	hsa-miR-483-5p
NCBP2	hsa-miR-206-3p
NCBP2	hsa-miR-429-5p
NCBP2	hsa-miR-4257
NCBP2	hsa-miR-7188-5p
NCBP2	hsa-miR-378a-5p
NCBP2	hsa-miR-323a-3p
NCBP2	hsa-miR-2380
NCBP2	hsa-miR-145-5p
NCBP2	hsa-miR-764-3p
NCBP2	hsa-miR-1245-3p
NCBP2	hsa-miR-493-3p
NCBP2	hsa-miR-1252-3p
NCBP2	hsa-miR-489
NCBP2	hsa-miR-466g
NCBP2	hsa-miR-6073
NCBP2	hsa-miR-5101
NCBP2	hsa-miR-585-5p
NCBP2	hsa-miR-382-5p
NCBP2	hsa-miR-346
NCBP2	hsa-miR-499
NCBP2	hsa-miR-3978
NCBP2	hsa-miR-511
NCBP2	hsa-miR-1224-3p
NCBP2	hsa-miR-6373
NCBP2	hsa-miR-7371f-5p
NCBP2	hsa-miR-6807-5p
NCBP2	hsa-miR-669d-5p
NCBP2	hsa-miR-7089-3p
NCBP2	hsa-miR-7016-3p
NCBP2	hsa-miR-1303
NCBP2	hsa-miR-2338
NCBP2	hsa-miR-23-3p
NCBP2	hsa-miR-548u

NCBP2	hsa-miR-4744
NCBP2	hsa-miR-2384
NCBP2	hsa-miR-27b-5p
NCBP2	hsa-miR-6887-3p
NCBP2	hsa-miR-605
NCBP2	hsa-miR-214-3p
NCBP2	hsa-miR-548t-5p
NCBP2	hsa-miR-623
NCBP2	hsa-miR-7201-5p
NCBP2	hsa-miR-874-5p
NCBP2	hsa-miR-3668
NCBP2	hsa-miR-4425
NCBP2	hsa-miR-142-3p.1
NCBP2	hsa-miR-500a-3p
NCBP2	hsa-miR-101-1-5p
NCBP2	hsa-miR-27
NCBP2	hsa-miR-431-5p
NCBP2	hsa-miR-3164
NCBP2	hsa-miR-221-5p
NCBP2	hsa-miR-4775
NCBP2	hsa-miR-512
NCBP2	hsa-miR-2285b
NCBP2	hsa-miR-7235-3p
NCBP2	hsa-miR-466c-3p
NCBP2	hsa-miR-8846
NCBP2	hsa-miR-365-5p
NCBP2	hsa-miR-181-5p
NCBP2	hsa-miR-3922-5p
NCBP2	hsa-miR-660-3p
NCBP2	hsa-miR-632
NCBP2	hsa-miR-6778-3p
NCBP2	hsa-miR-3646
NCBP2	hsa-miR-3100-5p
NCBP2	hsa-miR-3913-3p
NCBP2	hsa-miR-129b-5p
NCBP2	hsa-miR-4672
NCBP2	hsa-miR-6832-5p
NCBP2	hsa-miR-101b-3p.1
NCBP2	hsa-miR-548cej-3p
NCBP2	hsa-miR-124-5p
NCBP2	hsa-miR-367-5p
NCBP2	hsa-miR-642b-5p
NCBP2	hsa-miR-7386o-3p
NCBP2	hsa-miR-7287-5p
NCBP2	hsa-miR-4685-3p
NCBP2	hsa-miR-1224-5p

NCBP2	hsa-miR-7063-3p
NCBP2	hsa-miR-181
NCBP2	hsa-miR-384-5p
NCBP2	hsa-miR-32-5p
NCBP2	hsa-miR-2345
NCBP2	hsa-miR-1193
NCBP2	hsa-miR-122-5p
NCBP2	hsa-miR-208a-5p
NCBP2	hsa-miR-7230-3p
NCBP2	hsa-miR-6818-3p
NCBP2	hsa-miR-320a
NCBP2	hsa-miR-362-5p
NCBP2	hsa-miR-676-3p
NCBP2	hsa-miR-1306-5p
NCBP2	hsa-miR-4330
NCBP2	hsa-miR-150-5p
NCBP2	hsa-miR-129b-3p
NCBP2	hsa-miR-330-5p
NCBP2	hsa-miR-520g-3p
NCBP2	hsa-miR-8060
NCBP2	hsa-miR-3085-5p
NCBP2	hsa-miR-1915-3p
NCBP2	hsa-miR-3942-5p
NCBP2	hsa-miR-6734-3p
NCBP2	hsa-miR-215
NCBP2	hsa-miR-548aa
NCBP2	hsa-miR-4674
NCBP2	hsa-miR-7207-5p
NCBP2	hsa-miR-4423-5p
NCBP2	hsa-miR-3928-3p
NCBP2	hsa-miR-548t-3p
NCBP2	hsa-miR-3473g
NCBP2	hsa-miR-669hk-3p
NCBP2	hsa-miR-4659b-3p
NCBP2	hsa-miR-4771
NCBP2	hsa-miR-2387
NCBP2	hsa-miR-1839-3p
NCBP2	hsa-miR-6959-5p
NCBP2	hsa-miR-338-3p
NCBP2	hsa-miR-181b-3p
NCBP2	hsa-miR-6953-3p
NCBP2	hsa-miR-2310
NCBP2	hsa-miR-362-3p
NCBP2	hsa-miR-549a
NCBP2	hsa-miR-769-5p
NCBP2	hsa-miR-5132-3p

NCBP2	hsa-miR-3200-5p
NCBP2	hsa-miR-7007-5p
NCBP2	hsa-miR-3075-3p
NCBP2	hsa-miR-2368-3p
NCBP2	hsa-miR-7442-5p
NCBP2	hsa-miR-379-5p
NCBP2	hsa-miR-583
NCBP2	hsa-miR-374b-5p
NCBP2	hsa-miR-193a-5p
NCBP2	hsa-miR-2284z
NCBP2	hsa-miR-7472-3p
NCBP2	hsa-miR-3914
NCBP2	hsa-miR-501-3p
NCBP2	hsa-miR-548az-5p
NCBP2	hsa-miR-380
NCBP2	hsa-miR-4528
NCBP2	hsa-miR-548n
NCBP2	hsa-miR-10b-3p
NCBP2	hsa-miR-122
NCBP2	hsa-miR-1264-3p
NCBP2	hsa-miR-6755-3p
NSUN2	hsa-miR-7031-5p
NSUN2	hsa-miR-7333-5p
NSUN2	hsa-miR-1929-5p
NSUN2	hsa-miR-578
NSUN2	hsa-miR-148-5p
NSUN2	hsa-miR-129-5p
NSUN2	hsa-miR-500a-3p
NSUN2	hsa-miR-506-3p
NSUN2	hsa-miR-548e-5p
NSUN2	hsa-miR-5582-5p
NSUN2	hsa-miR-2284y
NSUN2	hsa-miR-548p
NSUN2	hsa-miR-143-3p
NSUN2	hsa-miR-3938-3p
NSUN2	hsa-miR-5618-5p
NSUN2	hsa-miR-6736-3p
NSUN2	hsa-miR-7398j-3p
NSUN2	hsa-miR-7340-3p
NSUN2	hsa-miR-208-3p
NSUN2	hsa-miR-7221-3p
NSUN2	hsa-miR-3470b
NSUN2	hsa-miR-875-3p
NSUN2	hsa-miR-1277-5p
NSUN2	hsa-miR-665
NSUN2	hsa-miR-495-3p

NSUN2	hsa-miR-492
NSUN2	hsa-miR-329-3p
NSUN2	hsa-miR-29a-5p
NSUN2	hsa-miR-101-3p
NSUN2	hsa-miR-7243-5p
NSUN2	hsa-miR-7084-3p
NSUN2	hsa-miR-526b-5p
NSUN2	hsa-miR-95-5p
NSUN2	hsa-miR-7025-3p
NSUN2	hsa-miR-1972
NSUN2	hsa-miR-32-5p
NSUN2	hsa-miR-944
NSUN2	hsa-miR-572
NSUN2	hsa-miR-7355-3p
NSUN2	hsa-miR-5680
NSUN2	hsa-miR-132-3p
NSUN2	hsa-miR-5625-3p
NSUN2	hsa-miR-4455
NSUN2	hsa-miR-5119
NSUN2	hsa-miR-5194
NSUN2	hsa-miR-3096-5p
NSUN2	hsa-miR-665-3p
NSUN2	hsa-miR-2399-5p
NSUN2	hsa-miR-199
NSUN2	hsa-miR-6505-3p
NSUN2	hsa-miR-5002-5p
NSUN2	hsa-miR-362-3p
NSUN2	hsa-miR-7151-3p
NSUN2	hsa-miR-7399-5p
NSUN2	hsa-miR-6320
NSUN2	hsa-miR-200bc-3p
NSUN2	hsa-miR-548al
NSUN2	hsa-miR-466-5p
NSUN2	hsa-miR-4482-3p
NSUN2	hsa-miR-3660
NSUN2	hsa-miR-7158-5p
NSUN2	hsa-miR-3963
NSUN2	hsa-miR-548
NSUN2	hsa-miR-17-3p
NSUN2	hsa-miR-216b-3p
NSUN2	hsa-miR-1193-3p
NSUN2	hsa-miR-513
NSUN2	hsa-miR-544-3p
NSUN2	hsa-miR-769-5p
NSUN2	hsa-miR-5697-3p
NSUN2	hsa-miR-99-3p

NSUN2	hsa-miR-7011-3p
NSUN2	hsa-miR-607
NSUN2	hsa-miR-878-3p
NSUN2	hsa-miR-9769-5p
NSUN2	hsa-miR-6972-3p
NSUN2	hsa-miR-7300-5p
NSUN2	hsa-miR-7270-5p
NSUN2	hsa-miR-142
NSUN2	hsa-miR-140-5p
NSUN2	hsa-miR-412
NSUN2	hsa-miR-4717-5p
NSUN2	hsa-miR-511-3p
NSUN2	hsa-miR-7274-5p
NSUN2	hsa-miR-6953-3p
NSUN2	hsa-miR-545-3p
NSUN2	hsa-miR-193a
NSUN2	hsa-miR-4453
NSUN2	hsa-miR-526
NSUN2	hsa-miR-6788-5p
NSUN2	hsa-miR-6738-3p
NSUN2	hsa-miR-15c-3p
NSUN2	hsa-miR-224-5p
NSUN2	hsa-miR-7047-3p
NSUN2	hsa-miR-199-3p
NSUN2	hsa-miR-19
NSUN2	hsa-miR-2415-3p
NSUN2	hsa-miR-7026-5p
NSUN2	hsa-miR-218-5p
NSUN2	hsa-miR-466n-3p
NSUN2	hsa-miR-6403
NSUN2	hsa-miR-4772-3p
NSUN2	hsa-miR-5622-5p
NSUN2	hsa-miR-6804-3p
NSUN2	hsa-miR-6379
NSUN2	hsa-miR-7262a-5p
NSUN2	hsa-miR-6731-5p
NSUN2	hsa-miR-539-5p
NSUN2	hsa-miR-499
NSUN2	hsa-miR-9a-3p
NSUN2	hsa-miR-7230-5p
NSUN2	hsa-miR-350
NSUN2	hsa-miR-16-3p
NSUN2	hsa-miR-7398r-3p
NSUN2	hsa-miR-4328
NSUN2	hsa-miR-136-5p
NSUN2	hsa-miR-548g-5p

NSUN2	hsa-miR-4438
NSUN2	hsa-miR-29b-2-5p
NSUN2	hsa-miR-7327-5p
NSUN2	hsa-miR-1546-3p
NSUN2	hsa-miR-6981-3p
NSUN2	hsa-miR-4314
NSUN2	hsa-miR-2469
NSUN2	hsa-miR-5691
NSUN2	hsa-miR-6724-5p
NSUN2	hsa-miR-6366
NSUN2	hsa-miR-6838-3p
NSUN2	hsa-miR-296-5p
NSUN2	hsa-miR-382-5p
NSUN2	hsa-miR-4538
NSUN2	hsa-miR-2285x
NSUN2	hsa-miR-138-3p
NSUN2	hsa-miR-7379-5p
NSUN2	hsa-miR-380-3p
NSUN2	hsa-miR-21-5p
NSUN2	hsa-miR-3561-5p
NSUN2	hsa-miR-509-5p
NSUN2	hsa-miR-4296
NSUN2	hsa-miR-7383-5p
NSUN2	hsa-miR-2389
NSUN2	hsa-miR-133-5p
NSUN2	hsa-miR-2425-5p
NSUN2	hsa-miR-7181-5p
NSUN2	hsa-miR-340-5p
NSUN2	hsa-miR-2387
NSUN2	hsa-miR-330-3p
NSUN2	hsa-miR-4422
NSUN2	hsa-miR-101-1-5p
NSUN2	hsa-miR-208a-3p
NSUN2	hsa-miR-326
NSUN2	hsa-miR-1260a
NSUN2	hsa-miR-450a-5p
NSUN2	hsa-miR-7386l-5p
NSUN2	hsa-miR-26a-3p
NSUN2	hsa-miR-7854-3p
NSUN2	hsa-miR-374-5p
NSUN2	hsa-miR-3606-3p
NSUN2	hsa-miR-1584-3p
NSUN2	hsa-miR-2284b
NSUN2	hsa-miR-1838
NSUN2	hsa-miR-410
NSUN2	hsa-miR-299-3p

NSUN2	hsa-miR-669m-5p
NSUN2	hsa-miR-937-3p
NSUN2	hsa-miR-1260a
NSUN2	hsa-miR-221-5p
NSUN2	hsa-miR-3944-5p
NSUN2	hsa-miR-7068-3p
NSUN2	hsa-miR-683
NSUN2	hsa-miR-1184
NSUN2	hsa-miR-3613b
NSUN2	hsa-miR-27-3p
NSUN2	hsa-miR-642-3p
NSUN2	hsa-miR-25-3p
NSUN2	hsa-miR-200c-3p
NSUN2	hsa-miR-2284u
NSUN2	hsa-miR-7339-5p
NSUN2	hsa-miR-6418-3p
NSUN2	hsa-miR-1983
NSUN2	hsa-miR-7-3p
NSUN2	hsa-miR-450-5p
NSUN2	hsa-miR-4649-3p
NSUN2	hsa-miR-153-3p
NSUN2	hsa-miR-5192
NSUN2	hsa-miR-4316
NSUN2	hsa-miR-1188-5p
NSUN2	hsa-miR-7051-5p
NSUN2	hsa-miR-433-3p
NSUN2	hsa-miR-4632-5p
NSUN2	hsa-miR-550b-3p
NSUN2	hsa-miR-144-3p
NSUN2	hsa-miR-449-5p
NSUN2	hsa-miR-8062
NSUN2	hsa-miR-3187-3p
NSUN2	hsa-miR-383-3p
NSUN2	hsa-miR-425
NSUN2	hsa-miR-5706
NSUN2	hsa-miR-3192
NSUN2	hsa-miR-4418
NSUN2	hsa-miR-6130
NSUN2	hsa-miR-7341-5p
NSUN2	hsa-miR-519d-5p
NSUN2	hsa-miR-2368-3p
NSUN2	hsa-miR-367-3p
NSUN2	hsa-miR-143-5p
NSUN2	hsa-miR-2426
NSUN2	hsa-miR-1933-5p
NSUN2	hsa-miR-3158-5p

NSUN2	hsa-miR-2284a
NSUN2	hsa-miR-376a-1-5p
NSUN2	hsa-miR-2362
NSUN2	hsa-miR-3938
NSUN2	hsa-miR-2053
NSUN2	hsa-miR-140-3p.1
NSUN2	hsa-miR-203
NSUN2	hsa-miR-7018-3p
NSUN2	hsa-miR-149-5p
NSUN2	hsa-miR-7159-3p
NSUN2	hsa-miR-7185-3p
NSUN2	hsa-miR-137-3p
NSUN2	hsa-miR-19-3p
NSUN2	hsa-miR-548ao-3p
NSUN2	hsa-miR-3590-3p
NSUN2	hsa-miR-6735-5p
NSUN2	hsa-miR-34-5p
NSUN2	hsa-miR-6943-3p
NSUN2	hsa-miR-328-3p
NSUN2	hsa-miR-322-3p
NSUN2	hsa-miR-3065-5p
NSUN2	hsa-miR-30c-3p
NSUN2	hsa-miR-506-3p
NSUN2	hsa-miR-513a
NSUN2	hsa-miR-16-1-3p
NSUN2	hsa-miR-21-5p
NSUN2	hsa-miR-4322
NSUN2	hsa-miR-7277-3p
NSUN2	hsa-miR-4265
NSUN2	hsa-miR-2284g
NSUN2	hsa-miR-7646-3p
NSUN2	hsa-miR-6516-3p
NSUN2	hsa-miR-3667-5p
NSUN2	hsa-miR-2284z
NSUN2	hsa-miR-7023-3p
NSUN2	hsa-miR-8883
NSUN2	hsa-miR-3472
NSUN2	hsa-miR-369-3p
NSUN2	hsa-miR-6879-5p
NSUN2	hsa-miR-7977
NSUN2	hsa-miR-3185
NSUN2	hsa-miR-6996-5p
NSUN2	hsa-miR-6974-3p
NSUN2	hsa-miR-124-5p
NSUN2	hsa-miR-8085
NSUN2	hsa-miR-4782-5p

NSUN2	hsa-miR-6994-5p
NSUN2	hsa-miR-374a-3p
NSUN2	hsa-miR-622
NSUN2	hsa-miR-2284l
NSUN2	hsa-miR-575
NSUN2	hsa-miR-659-3p
NSUN2	hsa-miR-1545a-3p
NSUN2	hsa-miR-7292-3p
NSUN2	hsa-miR-7398a-3p
NSUN2	hsa-miR-1930-3p
NSUN2	hsa-miR-330-3p.2
NSUN2	hsa-miR-495-3p
NSUN2	hsa-miR-382-3p
NSUN2	hsa-miR-378j
NSUN2	hsa-miR-7286-3p
NSUN2	hsa-miR-26-3p
NSUN2	hsa-miR-7089-3p
NSUN2	hsa-miR-4417
NSUN2	hsa-miR-124
NSUN2	hsa-miR-6089
NSUN2	hsa-miR-514a-5p
NSUN2	hsa-miR-4714-5p
NSUN2	hsa-miR-891b
NSUN2	hsa-miR-2320-5p
NSUN2	hsa-miR-350-3p
NSUN2	hsa-miR-513c-3p
NSUN2	hsa-miR-548an
NSUN2	hsa-miR-7350-3p
NSUN2	hsa-miR-499b-5p
NSUN2	hsa-miR-215-3p
NSUN2	hsa-miR-6127
NSUN2	hsa-miR-302-5p
NSUN2	hsa-miR-423-5p
NSUN2	hsa-miR-1185-3p
NSUN2	hsa-miR-6955-3p
NSUN2	hsa-miR-19-5p
NSUN2	hsa-miR-676-3p
NSUN2	hsa-miR-878
NSUN2	hsa-miR-6839-5p
NSUN2	hsa-miR-200b-5p
NSUN2	hsa-miR-7647-3p
NSUN2	hsa-miR-3200-5p
NSUN2	hsa-miR-7351-5p
NSUN2	hsa-miR-7398l-3p
NSUN2	hsa-miR-6878-5p
NSUN2	hsa-miR-4286

NSUN2	hsa-miR-590-5p
NSUN2	hsa-miR-194-5p
NSUN2	hsa-miR-6998-3p
NSUN2	hsa-miR-548j-5p
NSUN2	hsa-miR-455-3p.2
NSUN2	hsa-miR-4477a
NSUN2	hsa-miR-892-5p
NSUN2	hsa-miR-290b-5p
NSUN2	hsa-miR-1294
NSUN2	hsa-miR-7373a-3p
NSUN2	hsa-miR-1301-3p
NSUN2	hsa-miR-2347
NSUN2	hsa-miR-4263
NSUN2	hsa-miR-590-3p
NSUN2	hsa-miR-548aj-5p
NSUN2	hsa-miR-4274
NSUN2	hsa-miR-7222-3p
NSUN2	hsa-miR-1273g-3p
NSUN2	hsa-miR-619-5p
NSUN2	hsa-miR-3965
NSUN2	hsa-miR-4660
NSUN2	hsa-miR-548c-3p
NSUN2	hsa-miR-545-3p
NSUN2	hsa-miR-6129
NSUN2	hsa-miR-494-3p
NSUN2	hsa-miR-576-5p
NSUN2	hsa-miR-6506-5p
NSUN2	hsa-miR-7370-5p
NSUN2	hsa-miR-204-3p
NSUN2	hsa-miR-323b-3p
NSUN2	hsa-miR-32-3p
NSUN2	hsa-miR-7004-3p
NSUN2	hsa-miR-7398q-3p
NSUN2	hsa-miR-510-3p
NSUN2	hsa-miR-590-5p
NSUN2	hsa-miR-450a
NSUN2	hsa-miR-6762-3p
NSUN2	hsa-miR-375
NSUN2	hsa-miR-8872
NSUN2	hsa-miR-3580-3p
NSUN2	hsa-miR-151-3p
NSUN2	hsa-miR-487a
NSUN2	hsa-miR-4646-5p
NSUN2	hsa-miR-101-3p
NSUN2	hsa-miR-1324
NSUN2	hsa-miR-7318-5p

NSUN2	hsa-miR-1952
NSUN2	hsa-miR-544a
NSUN2	hsa-miR-6365
NSUN2	hsa-miR-7386l-3p
NSUN2	hsa-miR-876-5p
NSUN2	hsa-miR-497-3p
NSUN2	hsa-miR-200a-3p
NSUN2	hsa-miR-6734-5p
NSUN2	hsa-miR-124-3p.1
NSUN2	hsa-miR-6348
NSUN2	hsa-miR-7370-3p
NSUN2	hsa-miR-202-5p
NSUN2	hsa-miR-3071-5p
NSUN2	hsa-miR-126-5p
NSUN2	hsa-miR-4804-3p
NSUN2	hsa-miR-7398b-3p
NSUN2	hsa-miR-4708-3p
NSUN2	hsa-miR-336-5p
NSUN2	hsa-miR-343
NSUN2	hsa-miR-376a-5p
NSUN2	hsa-miR-3587
NSUN2	hsa-miR-8797
NSUN2	hsa-miR-3664-5p
NSUN2	hsa-miR-219a-1-3p
NSUN2	hsa-miR-140-5p
NSUN2	hsa-miR-548f-5p
NSUN2	hsa-miR-7386j-3p
NSUN2	hsa-miR-378
NSUN2	hsa-miR-548au-3p
NSUN2	hsa-miR-380
NSUN2	hsa-miR-7680-5p
NSUN2	hsa-miR-134-5p
NSUN2	hsa-miR-301-3p
NSUN2	hsa-miR-455-3p.1
NSUN2	hsa-miR-4436b-3p
NSUN2	hsa-miR-544a
NSUN2	hsa-miR-3130-3p
NSUN2	hsa-miR-2467-3p
NSUN2	hsa-miR-8807
NSUN2	hsa-miR-425-5p
NSUN2	hsa-miR-5095
NSUN2	hsa-miR-320a
NSUN2	hsa-miR-2323
NSUN2	hsa-miR-4445-3p
NSUN2	hsa-miR-603
NSUN2	hsa-miR-8850

NSUN2	hsa-miR-2284aa
NSUN2	hsa-miR-3926
NSUN2	hsa-miR-124-3p
NSUN2	hsa-miR-7073-3p
NSUN2	hsa-miR-7848-3p
NSUN2	hsa-miR-2411-5p
NSUN2	hsa-miR-498
NSUN2	hsa-miR-1260b
NSUN2	hsa-miR-7200-3p
NSUN2	hsa-miR-205-3p
NSUN2	hsa-miR-6120-5p
NSUN2	hsa-miR-452-3p
NSUN2	hsa-miR-7843-5p
NSUN2	hsa-miR-1239
NSUN2	hsa-miR-92-3p
NSUN2	hsa-miR-153-5p
NSUN2	hsa-miR-200-3p
NSUN2	hsa-miR-223-5p
NSUN2	hsa-miR-628-3p
NSUN2	hsa-miR-548s
NSUN2	hsa-miR-548c-3p
NSUN2	hsa-miR-1179
NSUN2	hsa-miR-5008-5p
NSUN2	hsa-miR-6363
NSUN2	hsa-miR-4666b
NSUN2	hsa-miR-548x-5p
NSUN2	hsa-miR-1547-3p
NSUN2	hsa-miR-7398i-3p
NSUN2	hsa-miR-488-3p
NSUN2	hsa-miR-468-3p
NSUN2	hsa-miR-15a-3p
NSUN2	hsa-miR-588
NSUN2	hsa-miR-1894-3p
NSUN2	hsa-miR-569
NSUN2	hsa-miR-4661-3p
NSUN2	hsa-miR-3194-3p
NSUN2	hsa-miR-7056-3p
NSUN2	hsa-miR-3942-3p
NSUN2	hsa-miR-93-3p
NSUN2	hsa-miR-6747-5p
NSUN2	hsa-miR-5692a
NSUN2	hsa-miR-3088-3p
NSUN2	hsa-miR-6773-5p
NSUN2	hsa-miR-505-3p
NSUN2	hsa-miR-4704-5p
NSUN2	hsa-miR-5585-5p

NSUN2	hsa-miR-142-5p
NSUN2	hsa-miR-21-3p
NSUN2	hsa-miR-2355-5p
NSUN2	hsa-miR-7398t-3p
NSUN2	hsa-miR-429
NSUN2	hsa-miR-4786-3p
NSUN2	hsa-miR-130-3p
NSUN2	hsa-miR-2363
NSUN2	hsa-miR-320a
NSUN2	hsa-miR-217
NSUN2	hsa-miR-7385f-3p
NSUN2	hsa-miR-1982-3p
NSUN2	hsa-miR-2276-3p
NSUN2	hsa-miR-5132-3p
NSUN2	hsa-miR-135-5p
NSUN2	hsa-miR-7398m-3p
NSUN2	hsa-miR-509-3-5p
NSUN2	hsa-miR-190-3p
NSUN2	hsa-miR-6731-3p
NSUN2	hsa-miR-6134
NSUN2	hsa-miR-9-3p
NSUN2	hsa-miR-8792
NSUN2	hsa-miR-1224-3p
NSUN2	hsa-miR-5190
NSUN2	hsa-miR-6782-5p
NSUN2	hsa-miR-6909-3p
NSUN2	hsa-miR-518c-5p
NSUN2	hsa-miR-7168-5p
NSUN2	hsa-miR-181a-2-3p
NSUN2	hsa-miR-7374a-3p
NSUN2	hsa-miR-3079-5p
NSUN2	hsa-miR-7094-3p
NSUN2	hsa-miR-892b
NSUN2	hsa-miR-513a-3p
NSUN2	hsa-miR-138-5p
NSUN2	hsa-miR-4330
NSUN2	hsa-miR-195-3p
NSUN2	hsa-miR-7215-5p
NSUN2	hsa-miR-6957-5p
NSUN2	hsa-miR-29-5p
NSUN2	hsa-miR-1290
NSUN2	hsa-miR-873-5p
NSUN2	hsa-miR-516
NSUN2	hsa-miR-1205
NSUN2	hsa-miR-7281-3p
NSUN2	hsa-miR-6821-5p

NSUN2	hsa-miR-6133
NSUN2	hsa-miR-4435
NSUN2	hsa-miR-499-5p
NSUN2	hsa-miR-193-3p
NSUN2	hsa-miR-3192-5p
NSUN2	hsa-miR-429-3p
NSUN2	hsa-miR-7162-3p
NSUN2	hsa-miR-1250-3p
NSUN2	hsa-miR-7033-5p
NSUN2	hsa-miR-1910-5p
NSUN2	hsa-miR-136-5p
NSUN2	hsa-miR-6793-3p
NSUN2	hsa-miR-1343-3p
NSUN2	hsa-miR-1262-5p
NSUN2	hsa-miR-2896
NSUN2	hsa-miR-8832
NSUN2	hsa-miR-7217-5p
NSUN2	hsa-miR-1540-5p
NSUN2	hsa-miR-3098-5p
NSUN2	hsa-miR-4676-5p
NSUN2	hsa-miR-2681-5p
NSUN2	hsa-miR-4733-5p
NSUN2	hsa-miR-335-3p
NSUN2	hsa-miR-7297-3p
NSUN2	hsa-miR-631
NSUN2	hsa-miR-141-3p
NSUN2	hsa-miR-488-3p
NSUN2	hsa-miR-1297
NUDT10	hsa-miR-2446
NUDT10	hsa-miR-6828-3p
NUDT10	hsa-miR-7688-3p
NUDT10	hsa-miR-33-5p
NUDT10	hsa-miR-6907-5p
NUDT10	hsa-miR-4294
NUDT10	hsa-miR-6952-3p
NUDT10	hsa-miR-212-5p
NUDT10	hsa-miR-130b-5p
NUDT10	hsa-miR-294-5p
NUDT10	hsa-miR-195-3p
NUDT10	hsa-miR-216a-5p
NUDT10	hsa-miR-3146
NUDT10	hsa-miR-7173-3p
NUDT10	hsa-miR-653
NUDT10	hsa-miR-186-5p
NUDT10	hsa-miR-8819
NUDT10	hsa-miR-8075

NUDT10	hsa-miR-500b-5p
NUDT10	hsa-miR-7119-5p
NUDT10	hsa-miR-23-5p
NUDT10	hsa-miR-6829-3p
NUDT10	hsa-miR-3658
NUDT10	hsa-miR-4703-3p
NUDT10	hsa-miR-1913
NUDT10	hsa-miR-3079-5p
NUDT10	hsa-miR-5187-5p
NUDT10	hsa-miR-7174-5p
NUDT10	hsa-miR-1255
NUDT10	hsa-miR-6926-3p
NUDT10	hsa-miR-889
NUDT10	hsa-miR-5588-5p
NUDT10	hsa-miR-2355-3p
NUDT10	hsa-miR-3085-5p
NUDT10	hsa-miR-7229-3p
NUDT10	hsa-miR-6914-3p
NUDT10	hsa-miR-4325
NUDT10	hsa-miR-7a-1-3p
NUDT10	hsa-miR-4714-5p
NUDT10	hsa-miR-143-3p
NUDT10	hsa-miR-2411-3p
NUDT10	hsa-miR-214-3p
NUDT10	hsa-miR-141-3p
NUDT10	hsa-miR-487-3p
NUDT10	hsa-miR-7214-5p
NUDT10	hsa-miR-1264
NUDT10	hsa-miR-342-3p
NUDT10	hsa-miR-544b
NUDT10	hsa-miR-4691-3p
NUDT10	hsa-miR-295-5p
NUDT10	hsa-miR-599
NUDT10	hsa-miR-654-5p
NUDT10	hsa-miR-130-3p
NUDT10	hsa-miR-590-5p
NUDT10	hsa-miR-7157-3p
NUDT10	hsa-miR-2285g
NUDT10	hsa-miR-7094-1-5p
NUDT10	hsa-miR-3157-3p
NUDT10	hsa-miR-6818-3p
NUDT10	hsa-miR-4669
NUDT10	hsa-miR-6841-3p
NUDT10	hsa-miR-2334
NUDT10	hsa-miR-541-5p
NUDT10	hsa-miR-6728-3p

NUDT10	hsa-miR-6743-5p
NUDT10	hsa-miR-6948-3p
NUDT10	hsa-miR-8839
NUDT10	hsa-miR-556-3p
NUDT10	hsa-miR-4659a-3p
NUDT10	hsa-miR-1958
NUDT10	hsa-miR-18-3p
NUDT10	hsa-miR-302c-3p
NUDT10	hsa-miR-322-3p
NUDT10	hsa-miR-7069-5p
NUDT10	hsa-miR-3116
NUDT10	hsa-miR-501-5p
NUDT10	hsa-miR-3191-5p
NUDT10	hsa-miR-3163
NUDT10	hsa-miR-548ap-5p
NUDT10	hsa-miR-409-3p
NUDT10	hsa-miR-7008-3p
NUDT10	hsa-miR-9768-3p
NUDT10	hsa-miR-5116
NUDT10	hsa-miR-5001-3p
NUDT10	hsa-miR-2054
NUDT10	hsa-miR-4524b-3p
NUDT10	hsa-miR-3613b
NUDT10	hsa-miR-4799-5p
NUDT10	hsa-miR-4688
NUDT10	hsa-miR-8895
NUDT10	hsa-miR-6071
NUDT10	hsa-miR-7084-5p
NUDT10	hsa-miR-34b-3p
NUDT10	hsa-miR-6512-5p
NUDT10	hsa-miR-4422
NUDT10	hsa-miR-6938-3p
NUDT10	hsa-miR-7212-3p
NUDT10	hsa-miR-1284
NUDT10	hsa-miR-5011-5p
NUDT10	hsa-miR-1277-5p
NUDT10	hsa-miR-126b-5p
NUDT10	hsa-miR-6744-5p
NUDT10	hsa-miR-6920-5p
NUDT10	hsa-miR-7159-5p
NUDT10	hsa-miR-2409
NUDT10	hsa-miR-6969-5p
NUDT10	hsa-miR-378g
NUDT10	hsa-miR-4312
NUDT10	hsa-miR-6951-5p
NUDT10	hsa-miR-4699-3p

NUDT10	hsa-miR-381-3p
NUDT10	hsa-miR-2426
NUDT10	hsa-miR-1254
NUDT10	hsa-miR-421-5p
NUDT10	hsa-miR-26
NUDT10	hsa-miR-6507-5p
NUDT10	hsa-miR-8843
NUDT10	hsa-miR-694
NUDT10	hsa-miR-2284z
NUDT10	hsa-miR-376a-2-5p
NUDT10	hsa-miR-130a-3p
NUDT10	hsa-miR-26a-3p
NUDT10	hsa-miR-466ade-3p
NUDT10	hsa-miR-2431-3p
NUDT10	hsa-miR-6960-5p
NUDT10	hsa-miR-2285af
NUDT10	hsa-miR-7658-3p
NUDT10	hsa-miR-323b-5p
NUDT10	hsa-miR-136-5p
NUDT10	hsa-miR-7202-3p
NUDT10	hsa-miR-452-5p
NUDT10	hsa-miR-7659-3p
NUDT10	hsa-miR-1434-3p
NUDT10	hsa-miR-500b-3p
NUDT10	hsa-miR-1193
NUDT10	hsa-miR-3544-3p
NUDT10	hsa-miR-350-3p
NUDT10	hsa-miR-494
NUDT10	hsa-miR-5190
NUDT10	hsa-miR-1-5p
NUDT10	hsa-miR-3622b-5p
NUDT10	hsa-miR-580-3p
NUDT10	hsa-miR-7049-5p
NUDT10	hsa-miR-4254
NUDT10	hsa-miR-9500
NUDT10	hsa-miR-2325b
NUDT10	hsa-miR-1968-3p
NUDT10	hsa-miR-4799-3p
NUDT10	hsa-miR-2284i
NUDT10	hsa-miR-384
NUDT10	hsa-miR-6927-3p
NUDT10	hsa-miR-7195-3p
NUDT10	hsa-miR-6728-5p
NUDT10	hsa-miR-764
NUDT10	hsa-miR-1178-3p
NUDT10	hsa-miR-8103

NUDT10	hsa-miR-6540-3p
NUDT10	hsa-miR-7234-5p
NUDT10	hsa-miR-429-3p
NUDT10	hsa-miR-5681a
NUDT10	hsa-miR-8832
NUDT10	hsa-miR-4309
NUDT10	hsa-miR-7856-5p
NUDT10	hsa-miR-5692a
NUDT10	hsa-miR-1956
NUDT10	hsa-miR-376e
NUDT10	hsa-miR-2284-5p
NUDT10	hsa-miR-488-5p
NUDT10	hsa-miR-6945-3p
NUDT10	hsa-miR-651
NUDT10	hsa-miR-302a-5p
NUDT10	hsa-miR-8073
NUDT10	hsa-miR-470-5p
NUDT10	hsa-miR-664-3p
NUDT10	hsa-miR-619-3p
NUDT10	hsa-miR-650abc
NUDT10	hsa-miR-6509-5p
NUDT10	hsa-miR-30-3p
NUDT10	hsa-miR-499b-5p
NUDT10	hsa-miR-367-3p
NUDT10	hsa-miR-1296
NUDT10	hsa-miR-495-3p
NUDT10	hsa-miR-2285
NUDT10	hsa-miR-1957a
NUDT10	hsa-miR-619
NUDT10	hsa-miR-1287-3p
NUDT10	hsa-miR-7009-3p
NUDT10	hsa-miR-4789-3p
NUDT10	hsa-miR-3962
NUDT10	hsa-miR-7191-5p
NUDT10	hsa-miR-651-3p
NUDT10	hsa-miR-548i
NUDT10	hsa-miR-6899-3p
NUDT10	hsa-miR-6791-3p
NUDT10	hsa-miR-2285b
NUDT10	hsa-miR-136-5p
NUDT10	hsa-miR-4253
NUDT10	hsa-miR-640
NUDT10	hsa-miR-4762-5p
NUDT10	hsa-miR-454
NUDT10	hsa-miR-5119
NUDT10	hsa-miR-556-5p

NUDT10	hsa-miR-7024-5p
NUDT10	hsa-miR-4480
NUDT10	hsa-miR-513b-5p
NUDT10	hsa-miR-301a-3p
NUDT10	hsa-miR-425-5p
NUDT10	hsa-miR-6971-3p
NUDT10	hsa-miR-487a-5p
NUDT10	hsa-miR-1943-3p
NUDT10	hsa-miR-7161-5p
NUDT10	hsa-miR-7675-3p
NUDT10	hsa-miR-8796
NUDT10	hsa-miR-7117-3p
NUDT10	hsa-miR-7199-5p
NUDT10	hsa-miR-1245-3p
NUDT10	hsa-miR-484
NUDT10	hsa-miR-6995-3p
NUDT10	hsa-miR-200a-3p
NUDT10	hsa-miR-6768-3p
NUDT10	hsa-miR-3160-3p
NUDT10	hsa-miR-4496
NUDT10	hsa-miR-7019-5p
NUDT10	hsa-miR-2285e
NUDT10	hsa-miR-1827
NUDT10	hsa-miR-556-5p
NUDT10	hsa-miR-3934-3p
NUDT10	hsa-miR-2317
NUDT10	hsa-miR-548ad-5p
NUDT10	hsa-miR-541-3p
NUDT10	hsa-miR-520gh
NUDT10	hsa-miR-2439-3p
NUDT10	hsa-miR-149-3p
NUDT10	hsa-miR-3935
NUDT10	hsa-miR-6889-3p
NUDT10	hsa-miR-6373
NUDT10	hsa-miR-493
NUDT10	hsa-miR-938
NUDT10	hsa-miR-2420
NUDT10	hsa-miR-2384
NUDT10	hsa-miR-409
NUDT10	hsa-miR-137-3p
NUDT10	hsa-miR-3120-3p
NUDT10	hsa-miR-1283
NUDT10	hsa-miR-4753-5p
NUDT10	hsa-miR-2329-3p
NUDT10	hsa-miR-7678-3p
NUDT10	hsa-miR-153-5p

NUDT10	hsa-miR-3156-5p
NUDT10	hsa-miR-2320-5p
NUDT10	hsa-miR-340-5p
NUDT10	hsa-miR-6947-3p
NUDT10	hsa-miR-548e-5p
NUDT10	hsa-miR-342-3p
NUDT10	hsa-miR-1285-5p
NUDT10	hsa-miR-4803
NUDT10	hsa-miR-3692-5p
NUDT10	hsa-miR-1178-3p
NUDT10	hsa-let-7ag-3p
NUDT10	hsa-miR-421
NUDT10	hsa-miR-142
NUDT10	hsa-miR-1243-5p
NUDT10	hsa-miR-22-3p
NUDT10	hsa-miR-582-5p
NUDT10	hsa-miR-5706
NUDT10	hsa-miR-693-5p
NUDT10	hsa-miR-1-3p
NUDT10	hsa-miR-616-3p
NUDT10	hsa-miR-196a-3p
NUDT10	hsa-miR-487a-3p
NUDT10	hsa-miR-7176-3p
NUDT10	hsa-miR-3182
NUDT10	hsa-miR-8901
NUDT10	hsa-miR-4635
NUDT10	hsa-miR-548y
NUDT10	hsa-miR-6511a-5p
NUDT10	hsa-miR-133b-5p
NUDT10	hsa-miR-301-5p
NUDT10	hsa-miR-585-3p
NUDT10	hsa-miR-7238-3p
NUDT10	hsa-miR-134-5p
NUDT10	hsa-miR-141-3p
NUDT10	hsa-miR-4659b-3p
NUDT10	hsa-miR-7182-3p
NUDT10	hsa-miR-20-3p
NUDT10	hsa-miR-3657
NUDT10	hsa-miR-4724-5p
NUDT10	hsa-miR-487b-5p
NUDT10	hsa-miR-292b-5p
NUDT10	hsa-miR-544-5p
NUDT10	hsa-miR-548as-3p
NUDT10	hsa-miR-6955-3p
NUDT10	hsa-miR-382-3p
NUDT10	hsa-miR-7037-3p

NUDT10	hsa-miR-23a-3p
NUDT10	hsa-miR-16-2-3p
NUDT10	hsa-miR-6958-3p
NUDT10	hsa-miR-5703
NUDT10	hsa-miR-6813-5p
NUDT10	hsa-miR-548ar-5p
NUDT10	hsa-miR-23c
NUDT10	hsa-miR-636
NUDT10	hsa-miR-550
NUDT10	hsa-miR-363-3p
NUDT10	hsa-miR-7641
NUDT10	hsa-miR-3119
NUDT10	hsa-miR-8118
NUDT10	hsa-miR-607
NUDT10	hsa-miR-4753-3p
NUDT10	hsa-miR-6120-5p
NUDT10	hsa-miR-1721
NUDT10	hsa-miR-2339
NUDT10	hsa-miR-548h-5p
NUDT10	hsa-miR-3668
NUDT10	hsa-miR-3924
NUDT10	hsa-miR-380-3p
NUDT10	hsa-miR-1843
NUDT10	hsa-miR-934
NUDT10	hsa-miR-369-3p
NUDT10	hsa-miR-339-5p
NUDT10	hsa-miR-344d-2-5p
NUDT10	hsa-miR-3977
NUDT10	hsa-miR-548ak
NUDT10	hsa-miR-654-3p
NUDT10	hsa-miR-7026-3p
NUDT10	hsa-miR-673-5p
NUDT10	hsa-miR-4782-5p
NUDT10	hsa-miR-3074-2-3p
NUDT10	hsa-miR-548
NUDT10	hsa-miR-199-5p
NUDT10	hsa-let-7g-3p
NUDT10	hsa-miR-33a-3p
NUDT10	hsa-miR-4691-5p
NUDT10	hsa-miR-593-3p
NUDT10	hsa-miR-1224-5p
NUDT10	hsa-miR-2284aa
NUDT10	hsa-miR-548au-5p
NUDT10	hsa-miR-2479
NUDT10	hsa-miR-351-3p
NUDT10	hsa-miR-548am-5p

NUDT10	hsa-miR-200a-5p
NUDT10	hsa-miR-2464-3p
NUDT10	hsa-miR-345-3p
NUDT10	hsa-miR-518a-5p
NUDT10	hsa-miR-466mo-3p
NUDT10	hsa-miR-218-5p
NUDT10	hsa-miR-324
NUDT10	hsa-miR-6742-3p
NUDT10	hsa-miR-2393
NUDT10	hsa-miR-2459
NUDT10	hsa-miR-527
NUDT10	hsa-miR-3074-3p
NUDT10	hsa-miR-3922-5p
NUDT10	hsa-miR-490-3p
NUDT10	hsa-miR-4751
NUDT10	hsa-miR-653-5p
NUDT10	hsa-miR-7687-3p
NUDT10	hsa-miR-1953
NUDT10	hsa-miR-6529-3p
NUDT10	hsa-miR-549-5p
NUDT10	hsa-miR-2367-5p
NUDT10	hsa-miR-582-5p
NUDT10	hsa-miR-616-3p
NUDT10	hsa-miR-26-5p
NUDT10	hsa-miR-4788
NUDT10	hsa-miR-7652-3p
NUDT10	hsa-miR-409b
NUDT10	hsa-miR-4263
NUDT10	hsa-miR-6344
NUDT10	hsa-miR-7-3p
NUDT10	hsa-miR-4445-5p
NUDT10	hsa-miR-548a-5p
NUDT10	hsa-miR-219-5p
NUDT10	hsa-miR-3071-5p
NUDT10	hsa-miR-6391
NUDT10	hsa-miR-548j-5p
NUDT10	hsa-miR-9769-5p
NUDT10	hsa-miR-467g
NUDT10	hsa-miR-7187-3p
NUDT10	hsa-miR-181b-1-3p
NUDT10	hsa-miR-3670
NUDT10	hsa-miR-224-5p
NUDT10	hsa-miR-550a-5p
NUDT10	hsa-miR-548d-5p
NUDT10	hsa-miR-5582-5p
NUDT10	hsa-miR-206

NUDT10	hsa-miR-2458
NUDT10	hsa-miR-3973
NUDT10	hsa-miR-376abd
NUDT10	hsa-miR-376ab
NUDT10	hsa-miR-4528
NUDT10	hsa-miR-3617-3p
NUDT10	hsa-miR-6529-5p
NUDT10	hsa-miR-2418
NUDT10	hsa-miR-548aw
NUDT10	hsa-miR-656
NUDT10	hsa-miR-1952
NUDT10	hsa-miR-129-5p
NUDT10	hsa-miR-374
NUDT10	hsa-miR-486-5p
NUDT10	hsa-miR-324-3p
NUDT10	hsa-miR-5583-3p
NUDT10	hsa-miR-592-3p
NUDT10	hsa-miR-3057-3p
NUDT10	hsa-miR-6998-3p
NUDT10	hsa-miR-3157-5p
NUDT10	hsa-miR-137
NUDT10	hsa-miR-7078-3p
NUDT10	hsa-miR-628-3p
NUDT10	hsa-miR-871-5p
NUDT10	hsa-miR-518-5p
NUDT10	hsa-miR-143-3p
NUDT10	hsa-miR-1668
NUDT10	hsa-miR-216b
NUDT10	hsa-miR-548as-5p
NUDT10	hsa-miR-23
NUDT10	hsa-miR-548aa
NUDT10	hsa-miR-6537-5p
NUDT10	hsa-miR-6937-3p
NUDT10	hsa-miR-3154
NUDT10	hsa-miR-7844-5p
NUDT10	hsa-miR-4320
NUDT10	hsa-miR-578
NUDT10	hsa-miR-16-1-3p
NUDT10	hsa-miR-3112-3p
NUDT10	hsa-miR-4671-3p
NUDT10	hsa-miR-4717-5p
NUDT10	hsa-miR-1256
NUDT10	hsa-miR-3198
NUDT10	hsa-miR-504-3p
NUDT10	hsa-miR-5622-5p
NUDT10	hsa-miR-4504

NUDT10	hsa-miR-532-5p
NUDT10	hsa-miR-1b-5p
NUDT10	hsa-miR-302bd
NUDT10	hsa-miR-5101
NUDT10	hsa-miR-7849-3p
NUDT10	hsa-miR-7033-3p
NUDT10	hsa-miR-200c-3p
NUDT10	hsa-miR-2284q
NUDT10	hsa-miR-3074-1-3p
NUDT10	hsa-miR-452-3p
NUDT10	hsa-miR-502-5p
NUDT10	hsa-miR-200c-3p
NUDT10	hsa-miR-205-5p
NUDT10	hsa-miR-34a-3p
NUDT10	hsa-miR-7650-3p
NUDT10	hsa-let-7-3p
NUDT10	hsa-miR-548c-5p
NUDT10	hsa-miR-669p-3p
NUDT10	hsa-miR-1298-3p
NUDT10	hsa-miR-301
NUDT10	hsa-miR-18b-3p
NUDT10	hsa-miR-3117-5p
NUDT10	hsa-miR-548b-5p
NUDT10	hsa-miR-7227-5p
NUDT10	hsa-miR-548w
NUDT10	hsa-miR-3071-3p
NUDT10	hsa-miR-7002-5p
NUDT10	hsa-miR-5196-3p
NUDT10	hsa-miR-6818-5p
NUDT10	hsa-miR-330-3p.2
NUDT10	hsa-miR-27b-5p
NUDT10	hsa-miR-934-5p
NUDT10	hsa-miR-548u
NUDT10	hsa-miR-7093-5p
NUDT10	hsa-miR-301b-3p
NUDT10	hsa-miR-3612
NUDT10	hsa-miR-203-3p.1
NUDT10	hsa-miR-3664-5p
NUDT10	hsa-miR-677
NUDT10	hsa-miR-675-3p
NUDT10	hsa-miR-520
NUDT10	hsa-miR-7057-5p
NUDT10	hsa-miR-4437
NUDT10	hsa-miR-1265
NUDT10	hsa-miR-1251-3p
NUDT10	hsa-miR-7230-3p

NUDT10	hsa-miR-1981-5p
NUDT10	hsa-miR-4795-3p
NUDT10	hsa-miR-382-5p
NUDT10	hsa-miR-516
NUDT10	hsa-miR-7086-5p
NUDT10	hsa-miR-7023-5p
NUDT10	hsa-miR-3200-5p
NUDT10	hsa-miR-30
NUDT10	hsa-miR-508a
NUDT10	hsa-miR-378
NUDT10	hsa-miR-501-3p
NUDT10	hsa-miR-3065-5p
NUDT10	hsa-miR-654-5p
NUDT10	hsa-miR-4289
NUDT10	hsa-miR-6980-5p
NUDT10	hsa-miR-17-3p
NUDT10	hsa-miR-593-3p
NUDT10	hsa-miR-2364
NUDT10	hsa-miR-3942-5p
NUDT10	hsa-miR-4649-3p
NUDT10	hsa-miR-548ay-5p
NUDT10	hsa-miR-4434
NUDT10	hsa-miR-3138
NUDT10	hsa-miR-3145-3p
NUDT10	hsa-miR-6388
NUDT10	hsa-miR-2285d
NUDT10	hsa-miR-2369
NUDT10	hsa-miR-8104
NUDT10	hsa-miR-3160-5p
NUDT10	hsa-miR-3097-5p
NUDT10	hsa-miR-3618
NUDT10	hsa-miR-505-3p.2
NUDT10	hsa-miR-2284ac
NUDT10	hsa-miR-425
NUDT10	hsa-miR-550-5p
NUDT10	hsa-miR-7667-5p
NUDT10	hsa-miR-660-3p
NUDT10	hsa-miR-129-5p
NUDT10	hsa-miR-429
NUDT10	hsa-miR-1255-5p
NUDT10	hsa-miR-548bb-5p
NUDT10	hsa-miR-1264-3p
NUDT10	hsa-miR-3121-3p
NUDT10	hsa-miR-8881
NUDT10	hsa-miR-10a-3p
NUDT10	hsa-miR-325

NUDT10	hsa-miR-1255b-5p
NUDT10	hsa-miR-490-3p
NUDT10	hsa-miR-6852-5p
NUDT10	hsa-miR-8902
NUDT10	hsa-miR-301-3p
NUDT10	hsa-let-7a-2-3p
NUDT10	hsa-miR-3084-5p
NUDT10	hsa-miR-2307
NUDT10	hsa-miR-761
NUDT10	hsa-miR-3121-3p
NUDT10	hsa-miR-5683
NUDT10	hsa-miR-6833-3p
NUDT10	hsa-miR-130b-3p
NUDT10	hsa-miR-127-5p
NUDT10	hsa-miR-574-3p
NUDT10	hsa-miR-192-3p
NUDT10	hsa-miR-362-5p
NUDT10	hsa-miR-2473
NUDT10	hsa-miR-6882-3p
NUDT10	hsa-miR-302b-5p
NUDT10	hsa-miR-542-3p
NUDT10	hsa-miR-181b-3p
NUDT10	hsa-miR-548t-3p
NUDT10	hsa-miR-5008-3p
NUDT10	hsa-miR-154-3p
NUDT10	hsa-miR-92a-5p
NUDT10	hsa-miR-18a-3p
NUDT10	hsa-miR-449b-3p
NUDT10	hsa-miR-122
NUDT10	hsa-miR-684
NUDT10	hsa-miR-2475
NUDT10	hsa-miR-23b-3p
NUDT10	hsa-miR-221-5p
NUDT10	hsa-miR-1250-3p
NUDT10	hsa-miR-9-3p
NUDT10	hsa-miR-8871
NUDT10	hsa-miR-2957
NUDT10	hsa-miR-7021-3p
NUDT10	hsa-miR-432-5p
NUDT10	hsa-miR-1200
NUDT10	hsa-miR-412-3p
NUDT10	hsa-miR-4443
NUDT10	hsa-miR-8805
NUDT10	hsa-miR-6947-5p
NUDT10	hsa-miR-1343-3p
NUDT10	hsa-miR-6959-5p

NUDT10	hsa-miR-290b-5p
NUDT10	hsa-miR-1a-5p
NUDT10	hsa-miR-200ab-5p
NUDT10	hsa-miR-2898
NUDT10	hsa-miR-502-5p
NUDT10	hsa-miR-8821
NUDT10	hsa-miR-93-3p
NUDT10	hsa-miR-9-3-3p
NUDT10	hsa-miR-7151-5p
NUDT10	hsa-miR-4720-5p
NUDT10	hsa-miR-653-3p
NUDT10	hsa-miR-6789-5p
NUDT10	hsa-miR-130
NUDT10	hsa-miR-6740-5p
NUDT10	hsa-miR-432-5p
NUDT10	hsa-miR-8113
NUDT10	hsa-miR-548o-5p
NUDT10	hsa-miR-669c-5p
NUDT10	hsa-miR-552-5p
NUDT10	hsa-miR-4531
NUDT10	hsa-miR-7056-3p
NUDT10	hsa-miR-1193-3p
NUDT10	hsa-miR-6870-3p
NUDT10	hsa-miR-6964-3p
NUDT10	hsa-miR-6792-3p
NUDT10	hsa-miR-4727-5p
NUDT10	hsa-miR-5625-3p
NUDT10	hsa-miR-300-5p
NUDT10	hsa-miR-4276
NUDT10	hsa-miR-6085
NUDT10	hsa-miR-508-3p
NUDT10	hsa-miR-6950-3p
NUDT10	hsa-miR-3143
NUDT10	hsa-miR-552-3p
NUDT10	hsa-miR-3068-5p
NUDT10	hsa-miR-32-3p
NUDT10	hsa-miR-548ae-5p
NUDT10	hsa-miR-877-5p
NUDT10	hsa-miR-15b-3p
NUDT10	hsa-miR-944
NUDT10	hsa-miR-410-3p
NUDT10	hsa-miR-6895-3p
NUDT10	hsa-miR-4769-3p
NUDT10	hsa-miR-216a-3p
NUDT10	hsa-miR-488-3p
NUDT10	hsa-miR-5088-3p

NUDT10	hsa-miR-4674
NUDT10	hsa-miR-7225-3p
NUDT10	hsa-miR-6348
NUDT10	hsa-miR-219b-5p
NUDT10	hsa-miR-5124a
NUDT10	hsa-miR-22-3p
NUDT10	hsa-miR-539-5p
NUDT10	hsa-miR-4539
NUDT10	hsa-miR-500-3p
NUDT10	hsa-miR-3100-3p
NUDT10	hsa-miR-514a-5p
NUDT10	hsa-miR-6965-3p
NUDT10	hsa-miR-6884-3p
NUDT10	hsa-miR-2469
NUDT10	hsa-miR-7053-3p
NUDT10	hsa-miR-292a-5p
NUDT10	hsa-miR-344-3p
NUDT10	hsa-miR-6798-3p
NUDT10	hsa-miR-4314
NUDT10	hsa-miR-889-3p
NUDT10	hsa-miR-1185-5p
NUDT10	hsa-miR-4280
NUDT10	hsa-miR-2355-5p
NUDT10	hsa-miR-200-3p
NUDT10	hsa-miR-6941-5p
NUDT10	hsa-miR-6416-3p
NUDT10	hsa-miR-4793-5p
NUDT10	hsa-miR-340-5p
NUDT10	hsa-miR-548at-3p
NUDT10	hsa-miR-4516
NUDT10	hsa-miR-674-5p
NUDT10	hsa-miR-6738-3p
NUDT10	hsa-miR-661
NUDT10	hsa-miR-374b-5p
NUDT10	hsa-miR-520g-3p
NUDT10	hsa-miR-7646-5p
NUDT10	hsa-miR-7660-3p
NUDT10	hsa-miR-676-5p
NUDT10	hsa-miR-6737-3p
NUDT10	hsa-miR-558
NUDT10	hsa-miR-454-3p
NUDT10	hsa-miR-669o-5p
NUDT10	hsa-miR-7030-3p
NUDT10	hsa-miR-606
NUDT10	hsa-miR-320a
NUDT10	hsa-miR-200

NUDT10	hsa-miR-2358
NUDT10	hsa-miR-1248
NUDT10	hsa-miR-4744
NUDT10	hsa-miR-494-3p
NUDT10	hsa-miR-7843-3p
NUDT10	hsa-miR-3613-3p
NUDT10	hsa-miR-101-3p.2
NUDT10	hsa-miR-548aq-5p
NUDT10	hsa-miR-4324
NUDT10	hsa-miR-26-3p
NUDT10	hsa-miR-7061-5p
NUDT10	hsa-miR-552
NUDT10	hsa-miR-2285c
NUDT10	hsa-miR-3075-5p
NUDT10	hsa-miR-122-5p
NUDT10	hsa-miR-3607-5p
NUDT10	hsa-miR-2284w
NUDT10	hsa-miR-222-5p
NUDT10	hsa-miR-1276
NUDT10	hsa-miR-6896-3p
NUDT10	hsa-miR-655
NUDT10	hsa-miR-8880
NUDT10	hsa-miR-548ab
NUDT10	hsa-miR-6839-5p
NUDT10	hsa-miR-468-5p
NUDT10	hsa-miR-7012-5p
NUDT10	hsa-miR-7168-5p
NUDT10	hsa-miR-758-3p
NUDT10	hsa-miR-3192-5p
NUDT10	hsa-miR-619-5p
NUDT10	hsa-miR-139-5p
NUDT10	hsa-miR-580-5p
NUDT10	hsa-miR-4433b-3p
NUDT10	hsa-miR-6817-5p
NUDT10	hsa-miR-6885-3p
NUDT10	hsa-miR-548ay-3p
NUDT10	hsa-miR-7865
NUDT10	hsa-miR-218-5p
NUDT10	hsa-miR-374c-5p
NUDT10	hsa-miR-7169-5p
NUDT10	hsa-miR-742-3p
NUDT10	hsa-miR-524-5p
NUDT10	hsa-miR-548cej-3p
NUDT10	hsa-miR-2392
NUDT10	hsa-miR-3976
NUDT10	hsa-miR-2448-3p

NUDT10	hsa-miR-4261
NUDT10	hsa-miR-7207-3p
NUDT10	hsa-miR-630
NUDT10	hsa-miR-7177-3p
NUDT10	hsa-miR-302d-5p
NUDT10	hsa-miR-6809-3p
NUDT10	hsa-miR-3185
NUDT10	hsa-miR-6758-3p
NUDT10	hsa-miR-7684-5p
NUDT10	hsa-miR-580
NUDT10	hsa-miR-550b-2-5p
NUDT10	hsa-miR-3184-3p
NUDT10	hsa-miR-939-3p
NUDT10	hsa-miR-3653-5p
NUDT10	hsa-miR-126-5p
NUDT10	hsa-miR-576-5p
NUDT10	hsa-miR-3059-3p
NUDT10	hsa-miR-6862-5p
NUDT10	hsa-miR-302c-5p
NUDT10	hsa-miR-5004-5p
NUDT10	hsa-miR-4768-5p
NUDT10	hsa-miR-547-5p
NUDT10	hsa-miR-130a-5p
NUDT10	hsa-miR-204-3p
NUDT10	hsa-miR-4646-5p
NUDT10	hsa-miR-4509
NUDT10	hsa-miR-6403
NUDT10	hsa-miR-219-3p
NUDT10	hsa-miR-516b-5p
NUDT10	hsa-miR-561-5p
NUDT10	hsa-miR-8119
NUDT10	hsa-miR-486-5p
NUDT10	hsa-miR-7158-3p
NUDT10	hsa-miR-550a-3-5p
NUDT10	hsa-miR-574-5p
NUDT10	hsa-miR-7192-3p
NUDT10	hsa-miR-2421
NUDT10	hsa-miR-224-5p
NUDT10	hsa-miR-1306-3p
NUDT10	hsa-miR-487a
NUDT10	hsa-miR-1983
NUDT10	hsa-miR-513
NUDT10	hsa-miR-140-3p.1
NUDT10	hsa-miR-290a-5p
NUDT10	hsa-miR-559
NUDT10	hsa-miR-599-5p

NUDT10	hsa-miR-6966-3p
NUDT10	hsa-miR-3075-3p
NUDT10	hsa-miR-1271-3p
NUDT10	hsa-miR-7646-3p
NUDT10	hsa-miR-942-5p
NUDT10	hsa-miR-4521
NUDT10	hsa-miR-147a
NUDT10	hsa-miR-677-5p
NUDT10	hsa-miR-642-3p
NUDT10	hsa-miR-3155a
NUDT10	hsa-miR-212-3p
NUDT10	hsa-miR-1915-5p
NUDT10	hsa-miR-450b-5p
NUDT10	hsa-miR-2325c
NUDT10	hsa-miR-6919-5p
NUDT10	hsa-miR-410
NUDT10	hsa-miR-200a-3p
NUDT10	hsa-miR-487-5p
NUDT10	hsa-miR-6506-5p
NUDT10	hsa-miR-29
NUDT10	hsa-miR-3189-3p
NUDT10	hsa-miR-223-3p
NUDT10	hsa-miR-20b-3p
NUDT10	hsa-miR-297bc-3p
NUDT10	hsa-miR-16-3p
NUDT10	hsa-miR-190a-5p
NUDT10	hsa-miR-6903-5p
NUDT10	hsa-miR-548ap-3p
NUDT10	hsa-miR-216a
NUDT10	hsa-miR-223-5p
NUDT10	hsa-miR-3161
NUDT10	hsa-miR-2404
NUDT10	hsa-miR-4487
NUDT10	hsa-miR-23-3p
NUDT10	hsa-miR-383-3p
NUDT10	hsa-miR-378j
NUDT10	hsa-miR-4305
NUDT10	hsa-miR-1896
NUDT10	hsa-miR-1255a
NUDT10	hsa-miR-3671
NUDT10	hsa-miR-4695-3p
NUDT10	hsa-miR-1910-3p
NUDT10	hsa-miR-6381
NUDT10	hsa-miR-4517
NUDT10	hsa-miR-628-3p
NUDT10	hsa-miR-335-5p

NUDT10	hsa-miR-503-3p
NUDT10	hsa-miR-4662-5p
NUDT11	hsa-miR-212-5p
NUDT11	hsa-miR-7068-3p
NUDT11	hsa-miR-26
NUDT11	hsa-miR-31-5p
NUDT11	hsa-miR-650
NUDT11	hsa-miR-760-5p
NUDT11	hsa-miR-3059-5p
NUDT11	hsa-miR-6800-5p
NUDT11	hsa-miR-8074
NUDT11	hsa-miR-4693-5p
NUDT11	hsa-miR-2285
NUDT11	hsa-miR-376c
NUDT11	hsa-miR-4662b
NUDT11	hsa-miR-502-3p
NUDT11	hsa-miR-3622a-3p
NUDT11	hsa-miR-190-3p
NUDT11	hsa-miR-889
NUDT11	hsa-miR-556-5p
NUDT11	hsa-miR-8861
NUDT11	hsa-miR-8845
NUDT11	hsa-miR-548g-3p
NUDT11	hsa-miR-10-5p
NUDT11	hsa-miR-670-5p
NUDT11	hsa-miR-548d-5p
NUDT11	hsa-miR-545-5p
NUDT11	hsa-miR-4524b-3p
NUDT11	hsa-miR-33a-3p
NUDT11	hsa-miR-4294
NUDT11	hsa-miR-4680-3p
NUDT11	hsa-miR-5692a
NUDT11	hsa-let-7c-1-3p
NUDT11	hsa-miR-5684
NUDT11	hsa-miR-8868
NUDT11	hsa-miR-132-3p
NUDT11	hsa-miR-4660
NUDT11	hsa-miR-342-3p
NUDT11	hsa-miR-2285h
NUDT11	hsa-miR-7046-3p
NUDT11	hsa-miR-2315
NUDT11	hsa-miR-654-5p
NUDT11	hsa-miR-140-5p
NUDT11	hsa-miR-1284
NUDT11	hsa-miR-3120-3p
NUDT11	hsa-miR-3613b

NUDT11	hsa-miR-6501-3p
NUDT11	hsa-miR-7081-3p
NUDT11	hsa-miR-330-3p
NUDT11	hsa-miR-596
NUDT11	hsa-miR-549a
NUDT11	hsa-miR-7683-3p
NUDT11	hsa-miR-2475
NUDT11	hsa-miR-3128
NUDT11	hsa-miR-323
NUDT11	hsa-miR-33-5p
NUDT11	hsa-miR-7236-5p
NUDT11	hsa-miR-351-5p
NUDT11	hsa-miR-669c-3p
NUDT11	hsa-miR-7859
NUDT11	hsa-miR-6960-5p
NUDT11	hsa-miR-6822-3p
NUDT11	hsa-miR-299a-5p
NUDT11	hsa-miR-1434-3p
NUDT11	hsa-miR-548e-5p
NUDT11	hsa-miR-7041-5p
NUDT11	hsa-miR-425-5p
NUDT11	hsa-miR-3065-3p
NUDT11	hsa-miR-6715b-3p
NUDT11	hsa-miR-548al
NUDT11	hsa-miR-1305
NUDT11	hsa-miR-205-3p
NUDT11	hsa-miR-1296
NUDT11	hsa-miR-219-3p
NUDT11	hsa-miR-2409
NUDT11	hsa-miR-892a
NUDT11	hsa-miR-199
NUDT11	hsa-miR-29a-3p
NUDT11	hsa-miR-548d-3p
NUDT11	hsa-miR-7191-3p
NUDT11	hsa-miR-1301-3p
NUDT11	hsa-miR-2406
NUDT11	hsa-miR-651-3p
NUDT11	hsa-miR-3061-3p
NUDT11	hsa-miR-578
NUDT11	hsa-miR-2285t
NUDT11	hsa-miR-4275
NUDT11	hsa-miR-3166
NUDT11	hsa-miR-1943-3p
NUDT11	hsa-miR-4680-5p
NUDT11	hsa-miR-2397-3p
NUDT11	hsa-miR-29b-3p

NUDT11	hsa-miR-33-3p
NUDT11	hsa-miR-7065-3p
NUDT11	hsa-miR-644a
NUDT11	hsa-miR-8089
NUDT11	hsa-miR-5685
NUDT11	hsa-miR-597
NUDT11	hsa-miR-340-5p
NUDT11	hsa-miR-7670-5p
NUDT11	hsa-miR-7864
NUDT11	hsa-miR-7221-5p
NUDT11	hsa-miR-3150b-3p
NUDT11	hsa-miR-141-3p
NUDT11	hsa-miR-1950
NUDT11	hsa-miR-421
NUDT11	hsa-miR-224-3p
NUDT11	hsa-miR-30
NUDT11	hsa-miR-539-3p
NUDT11	hsa-miR-6892-3p
NUDT11	hsa-miR-4263
NUDT11	hsa-miR-452-5p
NUDT11	hsa-miR-3173-3p
NUDT11	hsa-miR-188-5p
NUDT11	hsa-miR-582-5p
NUDT11	hsa-miR-3157-3p
NUDT11	hsa-miR-6879-5p
NUDT11	hsa-miR-4799-3p
NUDT11	hsa-miR-548ae-3p
NUDT11	hsa-miR-7843-5p
NUDT11	hsa-miR-710
NUDT11	hsa-miR-5007-3p
NUDT11	hsa-miR-4690-3p
NUDT11	hsa-miR-6979-5p
NUDT11	hsa-miR-6731-3p
NUDT11	hsa-miR-5003-3p
NUDT11	hsa-miR-4433b-5p
NUDT11	hsa-miR-532-5p
NUDT11	hsa-miR-8809
NUDT11	hsa-miR-5110
NUDT11	hsa-miR-30e-3p
NUDT11	hsa-miR-3926
NUDT11	hsa-miR-335-3p
NUDT11	hsa-miR-499
NUDT11	hsa-miR-23-5p
NUDT11	hsa-miR-345-3p
NUDT11	hsa-miR-7011-3p
NUDT11	hsa-miR-5187-5p

NUDT11	hsa-miR-7117-3p
NUDT11	hsa-miR-376c-3p
NUDT11	hsa-miR-6867-5p
NUDT11	hsa-miR-8075
NUDT11	hsa-miR-31-3p
NUDT11	hsa-miR-653-3p
NUDT11	hsa-miR-2681-3p
NUDT11	hsa-miR-6721-5p
NUDT11	hsa-miR-135-5p
NUDT11	hsa-miR-2052
NUDT11	hsa-miR-4766-5p
NUDT11	hsa-miR-144-3p
NUDT11	hsa-miR-200a-5p
NUDT11	hsa-miR-18-5p
NUDT11	hsa-miR-183-5p
NUDT11	hsa-miR-708-5p
NUDT11	hsa-miR-1255
NUDT11	hsa-miR-6783-5p
NUDT11	hsa-miR-105b
NUDT11	hsa-miR-1468-3p
NUDT11	hsa-miR-9500
NUDT11	hsa-miR-2339
NUDT11	hsa-miR-5009-3p
NUDT11	hsa-miR-2483-3p
NUDT11	hsa-miR-548j-3p
NUDT11	hsa-miR-6906-5p
NUDT11	hsa-miR-29c-3p
NUDT11	hsa-miR-8904a
NUDT11	hsa-miR-466f-3p
NUDT11	hsa-miR-8795
NUDT11	hsa-miR-4677-5p
NUDT11	hsa-miR-6378
NUDT11	hsa-miR-5692bc
NUDT11	hsa-miR-522-3p
NUDT11	hsa-miR-4652-5p
NUDT11	hsa-miR-350
NUDT11	hsa-miR-6792-5p
NUDT11	hsa-miR-6891-5p
NUDT11	hsa-miR-153-5p
NUDT11	hsa-miR-8853
NUDT11	hsa-miR-3065-5p
NUDT11	hsa-miR-203-5p
NUDT11	hsa-miR-181-3p
NUDT11	hsa-miR-200a-3p
NUDT11	hsa-miR-545-3p
NUDT11	hsa-miR-5588-5p

NUDT11	hsa-miR-644a
NUDT11	hsa-miR-4667
NUDT11	hsa-miR-7084-3p
NUDT11	hsa-miR-142-3p
NUDT11	hsa-miR-2284y
NUDT11	hsa-miR-3177-5p
NUDT11	hsa-miR-495-3p
NUDT11	hsa-miR-4643
NUDT11	hsa-miR-1827
NUDT11	hsa-miR-2458
NUDT11	hsa-miR-4426
NUDT11	hsa-miR-7054-5p
NUDT11	hsa-miR-1835
NUDT11	hsa-miR-4738-3p
NUDT11	hsa-miR-642-3p
NUDT11	hsa-miR-5003-5p
NUDT11	hsa-miR-6958-5p
NUDT11	hsa-miR-7214-5p
NUDT11	hsa-miR-4635
NUDT11	hsa-miR-6984-5p
NUDT11	hsa-miR-4318
NUDT11	hsa-miR-382-5p
NUDT11	hsa-miR-4290
NUDT11	hsa-miR-2462
NUDT11	hsa-miR-7202-3p
NUDT11	hsa-miR-557
NUDT11	hsa-miR-6739-3p
NUDT11	hsa-miR-4799-5p
NUDT11	hsa-miR-295-5p
NUDT11	hsa-miR-742-5p
NUDT11	hsa-miR-3173-3p
NUDT11	hsa-miR-4293
NUDT11	hsa-miR-518-5p
NUDT11	hsa-miR-6877-3p
NUDT11	hsa-miR-150-5p
NUDT11	hsa-miR-126a-5p
NUDT11	hsa-miR-2483-5p
NUDT11	hsa-miR-7198-3p
NUDT11	hsa-miR-7037-3p
NUDT11	hsa-miR-101c
NUDT11	hsa-miR-5697-3p
NUDT11	hsa-miR-466l-3p
NUDT11	hsa-miR-30-5p
NUDT11	hsa-miR-6949-3p
NUDT11	hsa-miR-548aef
NUDT11	hsa-miR-206

NUDT11	hsa-miR-494
NUDT11	hsa-miR-8893
NUDT11	hsa-miR-5706
NUDT11	hsa-miR-4774-5p
NUDT11	hsa-let-7a-3p
NUDT11	hsa-miR-30d-3p
NUDT11	hsa-miR-5190
NUDT11	hsa-miR-2284-5p
NUDT11	hsa-miR-6776-3p
NUDT11	hsa-miR-6948-3p
NUDT11	hsa-miR-599
NUDT11	hsa-miR-3074-3p
NUDT11	hsa-miR-4700-5p
NUDT11	hsa-miR-203-3p.2
NUDT11	hsa-let-7f-1-3p
NUDT11	hsa-miR-676-3p
NUDT11	hsa-miR-5571-5p
NUDT11	hsa-miR-3148
NUDT11	hsa-miR-2285af
NUDT11	hsa-miR-6929-5p
NUDT11	hsa-miR-7038-5p
NUDT11	hsa-miR-4667-5p
NUDT11	hsa-miR-216-3p
NUDT11	hsa-miR-1912-3p
NUDT11	hsa-miR-2681-5p
NUDT11	hsa-miR-2285z
NUDT11	hsa-miR-548bd-3p
NUDT11	hsa-miR-26a-5p
NUDT11	hsa-miR-199-5p
NUDT11	hsa-miR-101-3p
NUDT11	hsa-miR-7201-5p
NUDT11	hsa-miR-708-5p
NUDT11	hsa-miR-8821
NUDT11	hsa-miR-379-3p
NUDT11	hsa-miR-4307
NUDT11	hsa-miR-6781-3p
NUDT11	hsa-miR-384
NUDT11	hsa-miR-3102-5p.2-5p
NUDT11	hsa-miR-3101-5p
NUDT11	hsa-miR-624-5p
NUDT11	hsa-miR-6856-3p
NUDT11	hsa-miR-4330
NUDT11	hsa-miR-3160-3p
NUDT11	hsa-miR-4496
NUDT11	hsa-miR-2284j
NUDT11	hsa-miR-27b-5p

NUDT11	hsa-miR-182-5p
NUDT11	hsa-miR-3606-3p
NUDT11	hsa-miR-4291
NUDT11	hsa-miR-549a
NUDT11	hsa-miR-1297
NUDT11	hsa-miR-527
NUDT11	hsa-miR-6946-3p
NUDT11	hsa-miR-6908-3p
NUDT11	hsa-miR-140-3p.2
NUDT11	hsa-miR-144-3p
NUDT11	hsa-miR-6925-5p
NUDT11	hsa-miR-5094
NUDT11	hsa-miR-2682-3p
NUDT11	hsa-miR-493-5p
NUDT11	hsa-miR-669e-5p
NUDT11	hsa-miR-1198-5p
NUDT11	hsa-miR-640
NUDT11	hsa-miR-1283
NUDT11	hsa-miR-3202
NUDT11	hsa-miR-6509-3p
NUDT11	hsa-miR-516-3p
NUDT11	hsa-miR-186-5p
NUDT11	hsa-miR-548aq-3p
NUDT11	hsa-miR-377-3p
NUDT11	hsa-miR-7119-3p
NUDT11	hsa-miR-679-3p
NUDT11	hsa-miR-7023-3p
NUDT11	hsa-miR-21-3p
NUDT11	hsa-miR-7681-3p
NUDT11	hsa-miR-3617-5p
NUDT11	hsa-miR-454-5p
NUDT11	hsa-miR-3070-2-3p
NUDT11	hsa-miR-4433a-5p
NUDT11	hsa-miR-2909
NUDT11	hsa-miR-2410
NUDT11	hsa-miR-8839
NUDT11	hsa-miR-344b-3p
NUDT11	hsa-miR-764-5p
NUDT11	hsa-miR-450a
NUDT11	hsa-miR-4762-3p
NUDT11	hsa-miR-4471
NUDT11	hsa-miR-3651
NUDT11	hsa-miR-126b-5p
NUDT11	hsa-miR-2285u
NUDT11	hsa-miR-6126
NUDT11	hsa-miR-6924-3p

NUDT11	hsa-miR-2459
NUDT11	hsa-miR-548b-3p
NUDT11	hsa-miR-1238-3p
NUDT11	hsa-miR-3100-3p
NUDT11	hsa-miR-8881
NUDT11	hsa-miR-598-5p
NUDT11	hsa-miR-5197-5p
NUDT11	hsa-miR-6511a-5p
NUDT11	hsa-miR-5693
NUDT11	hsa-miR-3911
NUDT11	hsa-miR-646
NUDT11	hsa-miR-4309
NUDT11	hsa-miR-544-3p
NUDT11	hsa-miR-3657
NUDT11	hsa-miR-137
NUDT11	hsa-miR-124-5p
NUDT11	hsa-miR-513a-3p
NUDT11	hsa-miR-8485
NUDT11	hsa-miR-741-3p
NUDT11	hsa-miR-6981-5p
NUDT11	hsa-miR-127-5p
NUDT11	hsa-miR-942
NUDT11	hsa-miR-30-3p
NUDT11	hsa-miR-200
NUDT11	hsa-miR-7030-3p
NUDT11	hsa-miR-690
NUDT11	hsa-miR-29-3p
NUDT11	hsa-miR-8892
NUDT11	hsa-miR-7089-3p
NUDT11	hsa-miR-1273g-3p
NUDT11	hsa-miR-4795-5p
NUDT11	hsa-miR-505-3p
NUDT11	hsa-miR-7075-3p
NUDT11	hsa-miR-3944-5p
NUDT11	hsa-miR-7027-5p
NUDT11	hsa-miR-2284a
NUDT11	hsa-miR-6079
NUDT11	hsa-miR-369-3p
NUDT11	hsa-miR-4723-3p
NUDT11	hsa-miR-2284i
NUDT11	hsa-miR-4533
NUDT11	hsa-miR-7180-5p
NUDT11	hsa-miR-4776-3p
NUDT11	hsa-miR-490-3p
NUDT11	hsa-miR-1245b-3p
NUDT11	hsa-miR-6350

NUDT11	hsa-miR-518-3p
NUDT11	hsa-miR-380-3p
NUDT11	hsa-miR-7666-3p
NUDT11	hsa-miR-7110-3p
NUDT11	hsa-miR-452-3p
NUDT11	hsa-miR-6940-3p
NUDT11	hsa-miR-6858-5p
NUDT11	hsa-miR-15b-3p
NUDT11	hsa-miR-371a-5p
NUDT11	hsa-miR-26-5p
NUDT11	hsa-miR-4782-5p
NUDT11	hsa-miR-4724-5p
NUDT11	hsa-miR-3163
NUDT11	hsa-miR-548ah-3p
NUDT11	hsa-miR-1245-3p
NUDT11	hsa-miR-7194-5p
NUDT11	hsa-miR-6122-3p
NUDT11	hsa-miR-98-3p
NUDT11	hsa-miR-6999-5p
NUDT11	hsa-miR-185-5p
NUDT11	hsa-miR-544-5p
NUDT11	hsa-miR-605-3p
NUDT11	hsa-miR-1256
NUDT11	hsa-miR-2352
NUDT11	hsa-miR-7211-5p
NUDT11	hsa-miR-1289
NUDT11	hsa-miR-4670-3p
NUDT11	hsa-miR-3124-3p
NUDT11	hsa-miR-4273
NUDT11	hsa-miR-7025-3p
NUDT11	hsa-miR-7000-5p
NUDT11	hsa-miR-4717-3p
NUDT11	hsa-let-7abf-3p
NUDT11	hsa-miR-532-5p
NUDT11	hsa-miR-497-5p
NUDT11	hsa-miR-7093-5p
NUDT11	hsa-miR-6983-3p
NUDT11	hsa-miR-2418
NUDT11	hsa-miR-302
NUDT11	hsa-miR-6934-3p
NUDT11	hsa-miR-2285j
NUDT11	hsa-miR-1292-5p
NUDT11	hsa-miR-8885
NUDT11	hsa-miR-7238-3p
NUDT11	hsa-miR-373-3p
NUDT11	hsa-miR-5619-3p

NUDT11	hsa-miR-677-5p
NUDT11	hsa-miR-4659b-3p
NUDT11	hsa-miR-486-5p
NUDT11	hsa-miR-508a
NUDT11	hsa-miR-3167-5p
NUDT11	hsa-miR-3074-2-3p
NUDT11	hsa-miR-5011-5p
NUDT11	hsa-miR-2387
NUDT11	hsa-miR-628-3p
NUDT11	hsa-miR-551b-5p
NUDT11	hsa-miR-7848-3p
NUDT11	hsa-miR-9769-5p
NUDT11	hsa-miR-194-5p
NUDT11	hsa-miR-3145-3p
NUDT11	hsa-miR-7063-3p
NUDT11	hsa-miR-540-5p
NUDT11	hsa-miR-3662
NUDT11	hsa-miR-1255a
NUDT11	hsa-miR-539-5p
NUDT11	hsa-miR-4470
NUDT11	hsa-miR-589-5p
NUDT11	hsa-miR-7227-3p
NUDT11	hsa-miR-6920-5p
NUDT11	hsa-miR-548ar-3p
NUDT11	hsa-miR-3198
NUDT11	hsa-miR-98-5p
NUDT11	hsa-miR-142
NUDT11	hsa-miR-3144-5p
NUDT11	hsa-miR-518a-5p
NUDT11	hsa-miR-8818
NUDT11	hsa-miR-6758-5p
NUDT11	hsa-miR-743ab-3p
NUDT11	hsa-miR-6875-3p
NUDT11	hsa-miR-5585-3p
NUDT11	hsa-miR-764
NUDT11	hsa-miR-374
NUDT11	hsa-miR-6898-3p
NUDT11	hsa-miR-627-5p
NUDT11	hsa-miR-6077
NUDT11	hsa-miR-1241
NUDT11	hsa-miR-6944-3p
NUDT11	hsa-miR-32-3p
NUDT11	hsa-miR-653
NUDT11	hsa-miR-1839
NUDT11	hsa-miR-7234-3p
NUDT11	hsa-miR-323a-3p

NUDT11	hsa-miR-3646
NUDT11	hsa-miR-130b-5p
NUDT11	hsa-miR-3071-3p
NUDT11	hsa-miR-4536-5p
NUDT11	hsa-miR-6871-3p
NUDT11	hsa-miR-934-3p
NUDT11	hsa-miR-302f
NUDT11	hsa-miR-6975-3p
NUDT11	hsa-miR-650abc
NUDT11	hsa-miR-3654
NUDT11	hsa-miR-2358
NUDT11	hsa-miR-381-3p
NUDT11	hsa-miR-2333
NUDT11	hsa-miR-6371
NUDT11	hsa-miR-16-1-3p
NUDT11	hsa-miR-4493
NUDT11	hsa-miR-8825
NUDT11	hsa-miR-641
NUDT11	hsa-miR-873-3p
NUDT11	hsa-miR-4796-5p
NUDT11	hsa-miR-374-5p
NUDT11	hsa-miR-4740-5p
NUDT11	hsa-miR-17
NUDT11	hsa-miR-3660
NUDT11	hsa-miR-302bd
NUDT11	hsa-miR-138-3p
NUDT11	hsa-miR-2290
NUDT11	hsa-miR-1250-3p
NUDT11	hsa-miR-518
NUDT11	hsa-miR-302a-5p
NUDT11	hsa-miR-1277-5p
NUDT11	hsa-miR-597-5p
NUDT11	hsa-miR-545-3p
NUDT11	hsa-miR-19-5p
NUDT11	hsa-miR-1296-5p
NUDT11	hsa-miR-7156-5p
NUDT11	hsa-miR-20-3p
NUDT11	hsa-miR-7166-3p
NUDT11	hsa-miR-516b
NUDT11	hsa-miR-6960-3p
NUDT11	hsa-miR-6346
NUDT11	hsa-miR-200ab-5p
NUDT11	hsa-miR-365-5p
NUDT11	hsa-miR-7163-3p
NUDT11	hsa-miR-372-5p
NUDT11	hsa-miR-292b-5p

NUDT11	hsa-miR-7151-5p
NUDT11	hsa-miR-371b-5p
NUDT11	hsa-miR-489
NUDT11	hsa-miR-883b-5p
NUDT11	hsa-miR-7844-5p
NUDT11	hsa-miR-463-3p
NUDT11	hsa-miR-6716-5p
NUDT11	hsa-miR-7081-5p
NUDT11	hsa-miR-4742-3p
NUDT11	hsa-miR-760
NUDT11	hsa-miR-6899-3p
NUDT11	hsa-miR-8118
NUDT11	hsa-miR-548c-3p
NUDT11	hsa-miR-4778-3p
NUDT11	hsa-miR-378b
NUDT11	hsa-miR-873-5p.2
NUDT11	hsa-miR-6124
NUDT11	hsa-miR-371
NUDT11	hsa-miR-6922-3p
NUDT11	hsa-miR-6418-3p
NUDT11	hsa-miR-6507-5p
NUDT11	hsa-miR-1265
NUDT11	hsa-miR-3682-5p
NUDT11	hsa-miR-561-3p
NUDT11	hsa-miR-329-3p
NUDT11	hsa-miR-1178-3p
NUDT11	hsa-miR-7013-5p
NUDT11	hsa-miR-2285r
NUDT11	hsa-miR-4684-5p
NUDT11	hsa-miR-4276
NUDT11	hsa-miR-2370-3p
NUDT11	hsa-miR-2400
NUDT11	hsa-miR-570-3p
NUDT11	hsa-miR-466i-3p
NUDT11	hsa-miR-6416-3p
NUDT11	hsa-miR-874-3p
NUDT11	hsa-miR-375
NUDT11	hsa-miR-3544-5p
NUDT11	hsa-miR-600
NUDT11	hsa-miR-217
NUDT11	hsa-miR-3138
NUDT11	hsa-miR-4803
NUDT11	hsa-miR-6357
NUDT11	hsa-miR-2326
NUDT11	hsa-miR-350-3p
NUDT11	hsa-let-7-5p

NUDT11	hsa-miR-330-3p.2
NUDT11	hsa-miR-6929-3p
NUDT11	hsa-miR-7208-3p
NUDT11	hsa-miR-122
NUDT11	hsa-miR-3103-3p
NUDT11	hsa-miR-6540-3p
NUDT11	hsa-miR-470-5p
NUDT11	hsa-miR-6913-3p
NUDT11	hsa-miR-4704-5p
NUDT11	hsa-miR-7243-3p
NUDT11	hsa-miR-203-3p.1
NUDT11	hsa-miR-1-3p
NUDT11	hsa-miR-7018-3p
NUDT11	hsa-miR-3099-5p
NUDT11	hsa-miR-200-3p
NUDT11	hsa-miR-6887-3p
NUDT11	hsa-miR-199-3p
NUDT11	hsa-miR-548aw
NUDT11	hsa-miR-496a-3p.2
NUDT11	hsa-miR-655-3p
NUDT11	hsa-miR-6945-3p
NUDT11	hsa-miR-520f-5p
NUDT11	hsa-miR-218-5p
NUDT11	hsa-miR-6787-3p
NUDT11	hsa-miR-5680
NUDT11	hsa-miR-2893
NUDT11	hsa-miR-466n-5p
NUDT11	hsa-miR-409
NUDT11	hsa-miR-450a-5p
NUDT11	hsa-let-7-3p
NUDT11	hsa-miR-5196-5p
NUDT11	hsa-miR-627-3p
NUDT11	hsa-miR-373-3p
NUDT11	hsa-miR-548bb-3p
NUDT11	hsa-miR-2284g
NUDT11	hsa-miR-3928-3p
NUDT11	hsa-miR-695
NUDT11	hsa-miR-3135a
NUDT11	hsa-miR-550a-3p
NUDT11	hsa-miR-6514-5p
NUDT11	hsa-miR-670-3p
NUDT11	hsa-miR-672-3p
NUDT11	hsa-miR-4495
NUDT11	hsa-miR-194-3p
NUDT11	hsa-miR-3915
NUDT11	hsa-miR-2368-3p

NUDT11	hsa-miR-212-3p
NUDT11	hsa-miR-5702
NUDT11	hsa-miR-377-3p
NUDT11	hsa-miR-95-5p
NUDT11	hsa-miR-520
NUDT11	hsa-miR-1982-5p
NUDT11	hsa-miR-4436b-5p
NUDT11	hsa-miR-3976
NUDT11	hsa-miR-6902-5p
NUDT11	hsa-miR-494-3p
NUDT11	hsa-miR-7843-3p
NUDT11	hsa-miR-411-3p
NUDT11	hsa-miR-7849-3p
NUDT11	hsa-miR-29
NUDT11	hsa-miR-380
NUDT11	hsa-miR-6757-5p
NUDT11	hsa-miR-3123
NUDT11	hsa-miR-548l
NUDT11	hsa-miR-190a-3p
NUDT11	hsa-miR-2425-5p
NUDT11	hsa-miR-4687-5p
NUDT11	hsa-miR-635
NUDT11	hsa-miR-6774-5p
NUDT11	hsa-miR-4731-3p
NUDT11	hsa-miR-5010-3p
NUDT11	hsa-miR-7646-3p
NUDT11	hsa-miR-558
NUDT11	hsa-miR-5007-5p
NUDT11	hsa-miR-125b-2-3p
NUDT11	hsa-miR-8831
NUDT11	hsa-miR-93-3p
NUDT11	hsa-miR-143-3p
NUDT11	hsa-miR-216b
NUDT11	hsa-miR-642b-5p
NUDT11	hsa-miR-489-3p
NUDT11	hsa-miR-6481
NUDT11	hsa-miR-6525
NUDT11	hsa-miR-2284b
NUDT11	hsa-miR-513c-3p
NUDT11	hsa-miR-26b-5p
NUDT11	hsa-miR-3133
NUDT11	hsa-miR-693-5p
NUDT11	hsa-miR-26-3p
NUDT11	hsa-miR-3612
NUDT11	hsa-miR-326
NUDT11	hsa-miR-7188-3p

NUDT11	hsa-miR-553
NUDT11	hsa-miR-383-3p
NUDT11	hsa-miR-4766-3p
NUDT11	hsa-miR-3613-3p
NUDT11	hsa-miR-7192-5p
NUDT11	hsa-miR-200c-5p
NUDT11	hsa-miR-495-3p
NUDT11	hsa-miR-6961-3p
NUDT11	hsa-miR-548e-3p
NUDT11	hsa-miR-802
NUDT11	hsa-miR-4443
NUDT11	hsa-miR-676-3p
NUDT11	hsa-miR-4524-5p
NUDT11	hsa-miR-149-3p
NUDT11	hsa-miR-200c-3p
NUDT11	hsa-miR-296-5p
NUDT11	hsa-miR-466aep-5p
NUDT11	hsa-miR-759
NUDT11	hsa-miR-516b-5p
NUDT11	hsa-miR-4420
NUDT11	hsa-miR-6927-3p
NUDT11	hsa-miR-7164-5p
NUDT11	hsa-miR-2322-3p
NUDT11	hsa-miR-3087-3p
NUDT11	hsa-miR-568
NUDT11	hsa-miR-382-3p
NUDT11	hsa-miR-23a-5p
NUDT11	hsa-miR-208-5p
NUDT11	hsa-miR-3913-3p
NUDT11	hsa-miR-294-5p
NUDT11	hsa-miR-6360
NUDT11	hsa-miR-6835-3p
NUDT11	hsa-miR-6881-3p
NUDT11	hsa-miR-181b-3p
NUDT11	hsa-miR-2284d
NUDT11	hsa-miR-3470b
NUDT11	hsa-miR-7061-5p
NUDT11	hsa-miR-292a-5p
NUDT11	hsa-miR-6876-3p
NUDT11	hsa-miR-6516
NUDT11	hsa-miR-3653-3p
NUDT11	hsa-miR-3063-3p
NUDT11	hsa-miR-1286
NUDT11	hsa-miR-3671
NUDT11	hsa-miR-142-5p
NUDT11	hsa-miR-384-5p

NUDT11	hsa-miR-129-5p
NUDT11	hsa-miR-6946-5p
NUDT11	hsa-miR-567
NUDT11	hsa-miR-503-5p
NUDT11	hsa-miR-4261
NUDT11	hsa-miR-548h-3p
NUDT11	hsa-miR-7706
NUDT11	hsa-miR-486-5p
NUDT11	hsa-miR-466l-5p
NUDT11	hsa-miR-7044-5p
NUDT11	hsa-miR-616-5p
NUDT11	hsa-miR-548ac
NUDT11	hsa-miR-4689
NUDT11	hsa-miR-3070-3p
NUDT11	hsa-miR-1949
NUDT11	hsa-miR-6398
NUDT11	hsa-miR-4715-3p
NUDT11	hsa-miR-669bf-3p
NUDT11	hsa-miR-7675-3p
NUDT11	hsa-miR-613
NUDT11	hsa-miR-505-5p
NUDT11	hsa-miR-4632-5p
NUDT11	hsa-miR-7661-5p
NUDT11	hsa-miR-519-3p
NUDT11	hsa-miR-1388-3p
NUDT11	hsa-miR-1178-3p
NUDT11	hsa-miR-874
NUDT11	hsa-miR-26b-3p
NUDT11	hsa-miR-8104
NUDT11	hsa-miR-888-5p
NUDT11	hsa-miR-5096
NUDT11	hsa-miR-337-5p
NUDT11	hsa-miR-633
NUDT11	hsa-miR-6957-5p
NUDT11	hsa-miR-9-5p
NUDT11	hsa-miR-677
NUDT11	hsa-miR-2392
NUDT11	hsa-miR-2457
NUDT11	hsa-miR-7654-3p
NUDT11	hsa-miR-4709-5p
NUDT11	hsa-miR-143-5p
NUDT11	hsa-miR-1252-3p
NUDT11	hsa-miR-942-5p
NUDT11	hsa-miR-7021-3p
NUDT11	hsa-miR-141-3p
NUDT11	hsa-miR-3714

NUDT11	hsa-miR-1255b-2-3p
NUDT11	hsa-miR-8854
NUDT11	hsa-miR-2896
NUDT11	hsa-miR-3068-5p
NUDT11	hsa-miR-3607-3p
NUDT11	hsa-miR-4697-3p
NUDT11	hsa-miR-16-5p
NUDT11	hsa-miR-487a
NUDT11	hsa-miR-4311
NUDT11	hsa-miR-707
NUDT11	hsa-miR-5189-3p
NUDT11	hsa-miR-7865
NUDT11	hsa-miR-7178-5p
NUDT11	hsa-miR-3154
NUDT11	hsa-miR-3179
NUDT11	hsa-miR-3978
NUDT11	hsa-miR-6826-3p
NUDT11	hsa-miR-500-5p
NUDT11	hsa-miR-5690
NUDT11	hsa-miR-29a-3p
NUDT11	hsa-miR-4428
NUDT11	hsa-miR-524-5p
NUDT11	hsa-miR-692
NUDT11	hsa-miR-548n
NUDT11	hsa-miR-4761-3p
NUDT11	hsa-miR-3622b-3p
NUDT11	hsa-miR-8814
NUDT11	hsa-miR-6798-3p
NUDT11	hsa-miR-6844
NUDT11	hsa-miR-130a-5p
NUDT11	hsa-miR-3191-3p
NUDT11	hsa-miR-1931
NUDT11	hsa-miR-216a
NUDT11	hsa-miR-7183-3p
NUDT11	hsa-miR-6782-3p
NUDT11	hsa-miR-653-5p
NUDT11	hsa-miR-450a-1-3p
NUDT11	hsa-miR-291-5p
NUDT11	hsa-miR-3964
NUDT11	hsa-miR-2284x
NUDT11	hsa-miR-7007-5p
NUDT11	hsa-miR-2415-5p
NUDT11	hsa-miR-4795-3p
NUDT11	hsa-miR-548b-3p
NUDT11	hsa-miR-222-5p
NUDT11	hsa-miR-205-5p

NUDT11	hsa-miR-216b-3p
NUDT11	hsa-miR-2284v
NUDT11	hsa-miR-4666a-3p
NUDT11	hsa-miR-302a-3p
NUDT11	hsa-miR-23
NUDT11	hsa-miR-30a-3p
NUDT11	hsa-miR-3062-3p
NUDT11	hsa-miR-203
NUDT11	hsa-miR-6769b-3p
NUDT11	hsa-miR-4305
NUDT11	hsa-miR-433-5p
NUDT11	hsa-miR-548s
NUDT11	hsa-miR-142-3p.1
NUDT11	hsa-miR-7668-5p
NUDT11	hsa-miR-520-3p
NUDT11	hsa-miR-548aj-5p
NUDT11	hsa-miR-4642
NUDT11	hsa-miR-6973a-3p
NUDT11	hsa-miR-429
NUDT11	hsa-miR-344f-5p
NUDT11	hsa-miR-497-5p
NUDT11	hsa-miR-3183
NUDT11	hsa-miR-6819-3p
NUDT11	hsa-miR-2284c
NUDT11	hsa-miR-2364
NUDT11	hsa-miR-548a-3p
NUDT11	hsa-miR-577-3p
NUDT11	hsa-miR-2311
NUDT11	hsa-miR-6884-3p
NUDT11	hsa-miR-4633-3p
NUDT11	hsa-miR-2285b
NUDT11	hsa-miR-3169
NUDT11	hsa-miR-1299
NUDT11	hsa-miR-498
NUDT11	hsa-miR-2285x
NUDT11	hsa-miR-101-3p.2
NUDT11	hsa-miR-4445-3p
NUDT11	hsa-miR-2284u
NUDT11	hsa-miR-582-5p
NUDT11	hsa-miR-6740-3p
NUDT11	hsa-miR-6899-5p
NUDT11	hsa-miR-7163-5p
NUDT11	hsa-miR-7215-3p
NUDT11	hsa-miR-5618-3p
NUDT11	hsa-miR-892-3p
NUDT11	hsa-miR-212-3p

NUDT11	hsa-miR-323-3p
NUDT11	hsa-miR-2398
NUDT11	hsa-miR-5682
NUDT11	hsa-miR-574-3p
NUDT11	hsa-miR-875-3p
NUDT11	hsa-miR-1842
NUDT11	hsa-miR-3686
NUDT11	hsa-miR-15-5p
NUDT11	hsa-miR-3473g
NUDT11	hsa-miR-6995-5p
NUDT11	hsa-miR-6119-5p
NUDT11	hsa-miR-373-5p
NUDT11	hsa-miR-369-3p
NUDT11	hsa-miR-1255b-5p
NUDT11	hsa-miR-8059
NUDT11	hsa-miR-2284k
NUDT11	hsa-miR-579
NUDT11	hsa-miR-1243
NUDT11	hsa-miR-5691
NUDT11	hsa-miR-6985-5p
NUDT11	hsa-miR-6814-5p
NUDT11	hsa-miR-7676-3p
NUDT11	hsa-miR-2471-5p
NUDT11	hsa-miR-3074-1-3p
NUDT11	hsa-miR-3064-3p
NUDT11	hsa-miR-2284ab
NUDT11	hsa-miR-300
NUDT11	hsa-miR-548x-5p
NUDT11	hsa-miR-372-3p
NUDT11	hsa-miR-3925-5p
NUDT11	hsa-miR-6925-3p
NUDT11	hsa-miR-101-3p.1
NUDT11	hsa-miR-96-3p
NUDT11	hsa-miR-549-3p
NUDT11	hsa-miR-6837-3p
NUDT11	hsa-miR-6735-5p
NUDT11	hsa-miR-548av-3p
NUDT11	hsa-miR-6951-3p
NUDT11	hsa-miR-3685
NUDT11	hsa-miR-3924
NUDT11	hsa-miR-1958
NUDT11	hsa-miR-374c-5p
NUDT11	hsa-miR-20a-3p
NUDT11	hsa-miR-7086-3p
NUDT11	hsa-miR-4720-5p
NUDT11	hsa-miR-207

NUDT11	hsa-miR-509-5p
NUDT11	hsa-miR-551-5p
NUDT11	hsa-miR-8843
NUDT11	hsa-miR-338-3p
NUDT11	hsa-miR-302-3p
NUDT11	hsa-miR-570-3p
NUDT11	hsa-miR-7206-3p
NUDT11	hsa-miR-421-3p
NUDT11	hsa-miR-1-3p
NUDT11	hsa-miR-340-5p
NUDT11	hsa-miR-548f-5p
NUDT11	hsa-miR-548f-3p
NUDT11	hsa-miR-3071-5p
NUDT11	hsa-miR-194-5p
NUDT11	hsa-miR-410
NUDT11	hsa-miR-2404
NUDT11	hsa-miR-122-5p
NUDT11	hsa-miR-6810-5p
NUDT11	hsa-miR-433
NUDT11	hsa-miR-671-5p
NUDT11	hsa-miR-1249-5p
NUDT11	hsa-miR-6973b-3p
NUDT11	hsa-miR-2284n
NUDT11	hsa-miR-4801
NUDT11	hsa-miR-3102-3p
NUDT11	hsa-miR-8807
NUDT11	hsa-miR-451b
NUDT11	hsa-miR-382-5p
NUDT11	hsa-miR-1292-5p
NUDT11	hsa-miR-4657
NUDT11	hsa-miR-290a-5p
NUDT11	hsa-miR-200a-3p
NUDT11	hsa-miR-6964-3p
NUDT11	hsa-miR-7660-3p
NUDT11	hsa-miR-7663-3p
NUDT11	hsa-miR-140-3p
NUDT11	hsa-miR-7010-5p
NUDT11	hsa-miR-502b
NUDT11	hsa-miR-7233-5p
NUDT11	hsa-miR-542-3p
NUDT11	hsa-miR-3089-5p
NUDT11	hsa-miR-361-5p
NUDT11	hsa-miR-5697
NUDT11	hsa-miR-2421
NUDT11	hsa-miR-146-5p
NUDT11	hsa-miR-6994-5p

NUDT11	hsa-miR-125-5p
NUDT11	hsa-miR-6989-5p
NUDT11	hsa-miR-5108
NUDT11	hsa-miR-327
NUDT11	hsa-miR-5624-5p
NUDT11	hsa-miR-2284l
NUDT11	hsa-miR-298-5p
NUDT11	hsa-miR-3529-3p
NUDT11	hsa-miR-298
NUDT11	hsa-miR-4436b-3p
NUDT11	hsa-miR-4661-5p
NUDT11	hsa-miR-2446
NUDT11	hsa-miR-522-3p
NUDT11	hsa-miR-548g-5p
NUDT11	hsa-miR-5582-3p
NUDT11	hsa-miR-431-5p
NUDT11	hsa-miR-140-5p
NUDT11	hsa-miR-655
NUDT11	hsa-miR-4282
NUDT11	hsa-miR-183-3p
NUDT11	hsa-miR-101-3p
NUDT11	hsa-miR-28-5p
NUDT11	hsa-miR-548az-3p
NUDT11	hsa-miR-3688-3p
NUDT11	hsa-miR-1434-5p
NUDT11	hsa-miR-105-5p
NUDT11	hsa-miR-548am-3p
NUDT11	hsa-miR-2480
NUDT11	hsa-miR-4650
NUDT11	hsa-miR-432-5p
NUDT11	hsa-miR-4503
NUDT11	hsa-miR-4747-5p
NUDT11	hsa-miR-16-3p
NUDT11	hsa-miR-7215-5p
NUDT11	hsa-miR-4659a-3p
NUDT11	hsa-miR-4487
NUDT11	hsa-miR-548aj-3p
NUDT11	hsa-miR-5121
NUDT11	hsa-miR-449b-3p
NUDT11	hsa-miR-8836
NUDT11	hsa-miR-548c-3p
NUDT11	hsa-miR-23-3p
NUDT11	hsa-miR-450-5p
NUDT11	hsa-miR-4536-5p
NUDT11	hsa-miR-6500-5p
NUDT11	hsa-miR-4647

NUDT11	hsa-miR-8057
NUDT11	hsa-miR-1185-5p
NUDT11	hsa-miR-129-5p
NUDT11	hsa-miR-5687
NUDT11	hsa-miR-3909
NUDT11	hsa-miR-576-5p
NUDT11	hsa-miR-2276-5p
NUDT11	hsa-miR-381-3p
NUDT11	hsa-miR-6919-3p
NUDT11	hsa-miR-1910-3p
NUDT11	hsa-miR-195-5p
NUDT11	hsa-miR-6750-3p
NUDT11	hsa-miR-7169-5p
NUDT11	hsa-miR-7116-3p
NUDT11	hsa-miR-1247-3p
NUDT11	hsa-miR-7217-3p
NUDT11	hsa-miR-4694-3p
NUDT11	hsa-miR-6765-3p
NUDT11	hsa-miR-1912-5p
NUDT11	hsa-miR-147a
NUDT11	hsa-miR-7192-3p
NUDT11	hsa-miR-6868-5p
NUDT11	hsa-miR-139-5p
NUDT11	hsa-miR-2355-3p
NUDT11	hsa-miR-3655
NUDT11	hsa-miR-374b-5p
NUDT11	hsa-miR-1904
NUDT11	hsa-let-7b-3p
NUDT11	hsa-miR-505-3p.2
NUDT11	hsa-miR-2429
NUDT11	hsa-miR-372
NUDT11	hsa-miR-424-5p
NUDT11	hsa-miR-3075-3p
NUDT11	hsa-miR-626
NUDT11	hsa-miR-362-3p
NUDT11	hsa-miR-181a-2-3p
NUDT11	hsa-miR-6384
NUDT11	hsa-miR-2284m
NUDT11	hsa-miR-455-5p
NUDT11	hsa-miR-450b-5p
NUDT11	hsa-miR-548x-3p
NUDT11	hsa-miR-1283a
NUDT11	hsa-miR-3569-3p
NUDT11	hsa-miR-1843
NUDT11	hsa-miR-667-5p
NUDT11	hsa-miR-449a

NUDT11	hsa-miR-6795-3p
NUDT11	hsa-miR-6827-5p
NUDT11	hsa-miR-6856-5p
NUDT11	hsa-miR-6356
NUDT11	hsa-miR-877-3p
NUDT11	hsa-miR-4724-3p
NUDT11	hsa-miR-2482
NUDT11	hsa-miR-28-3p
NUDT11	hsa-miR-516
NUDT11	hsa-miR-6403
NUDT11	hsa-miR-548z
NUDT11	hsa-miR-7074-3p
NUDT11	hsa-miR-590-3p
NUDT11	hsa-miR-607
NUDT11	hsa-miR-2330-3p
NUDT11	hsa-miR-4722-5p
NUDT11	hsa-miR-4433b-3p
NUDT11	hsa-miR-7219-5p
NUDT11	hsa-miR-7024-3p
NUDT11	hsa-miR-425
NUDT11	hsa-miR-144-5p
NUDT11	hsa-miR-6345
NUDT11	hsa-miR-2437
NUDT11	hsa-miR-28-5p
NUDT16	hsa-miR-654-5p
NUDT16	hsa-miR-541-5p
NUDT16	hsa-miR-2116-3p
NUDT16	hsa-miR-148b-5p
NUDT16	hsa-miR-7040-3p
NUDT16	hsa-miR-883-3p
NUDT16	hsa-miR-205-3p
NUDT16	hsa-miR-105-5p
NUDT16	hsa-miR-4752
NUDT16	hsa-miR-3152-5p
NUDT16	hsa-miR-889-3p
NUDT16	hsa-miR-6766-5p
NUDT16	hsa-miR-3474
NUDT16	hsa-miR-7116-5p
NUDT16	hsa-miR-7202-5p
NUDT16	hsa-miR-6986-5p
NUDT16	hsa-miR-6943-3p
NUDT16	hsa-miR-203a-5p
NUDT16	hsa-miR-1305
NUDT16	hsa-miR-7009-3p
NUDT16	hsa-miR-6807-5p
NUDT16	hsa-miR-4452

NUDT16	hsa-miR-212-3p
NUDT16	hsa-miR-6742-3p
NUDT16	hsa-miR-1839-5p
NUDT16	hsa-miR-383-5p.2
NUDT16	hsa-miR-4286
NUDT16	hsa-miR-1283
NUDT16	hsa-miR-8908-5p
NUDT16	hsa-miR-596
NUDT16	hsa-miR-346-3p
NUDT16	hsa-miR-517
NUDT16	hsa-miR-31-5p
NUDT16	hsa-miR-4738-3p
NUDT16	hsa-miR-3173-3p
NUDT16	hsa-miR-6958-5p
NUDT16	hsa-miR-215-5p
NUDT16	hsa-miR-181b-3p
NUDT16	hsa-miR-5011-3p
NUDT16	hsa-miR-3117-3p
NUDT16	hsa-miR-6870-3p
NUDT16	hsa-miR-875-5p
NUDT16	hsa-miR-6871-3p
NUDT16	hsa-miR-8893
NUDT16	hsa-miR-7024-5p
NUDT16	hsa-miR-8068
NUDT16	hsa-miR-7675-3p
NUDT16	hsa-miR-6818-3p
NUDT16	hsa-miR-20-5p
NUDT16	hsa-miR-4802-5p
NUDT16	hsa-miR-5681b
NUDT16	hsa-miR-3146
NUDT16	hsa-miR-5006-5p
NUDT16	hsa-miR-145-5p
NUDT16	hsa-miR-3113-5p
NUDT16	hsa-miR-6748-3p
NUDT16	hsa-miR-2376
NUDT16	hsa-miR-467-3p
NUDT16	hsa-miR-1264-5p
NUDT16	hsa-miR-203b-3p
NUDT16	hsa-miR-3151-5p
NUDT16	hsa-miR-3664-3p
NUDT16	hsa-miR-375-3p
NUDT16	hsa-miR-136-5p
NUDT16	hsa-miR-7204-3p
NUDT16	hsa-miR-4320
NUDT16	hsa-miR-7177-5p
NUDT16	hsa-miR-6797-5p

NUDT16	hsa-miR-149-5p
NUDT16	hsa-miR-6794-3p
NUDT16	hsa-miR-5008-3p
NUDT16	hsa-miR-497-3p
NUDT16	hsa-miR-214-3p
NUDT16	hsa-miR-6769a-5p
NUDT16	hsa-miR-4303
NUDT16	hsa-miR-7068-3p
NUDT16	hsa-miR-1323-5p
NUDT16	hsa-miR-6715-3p
NUDT16	hsa-miR-6358
NUDT16	hsa-miR-106-5p
NUDT16	hsa-miR-6388
NUDT16	hsa-miR-874-5p
NUDT16	hsa-miR-495-5p
NUDT16	hsa-miR-291b-3p
NUDT16	hsa-miR-2285t
NUDT16	hsa-miR-4652-3p
NUDT16	hsa-miR-7649-5p
NUDT16	hsa-miR-3194-3p
NUDT16	hsa-miR-7864
NUDT16	hsa-miR-3095-3p
NUDT16	hsa-miR-551b-5p
NUDT16	hsa-miR-888-5p
NUDT16	hsa-miR-4492
NUDT16	hsa-miR-7685-5p
NUDT16	hsa-miR-1225-5p
NUDT16	hsa-miR-3473c
NUDT16	hsa-miR-3095-5p
NUDT16	hsa-miR-6952-3p
NUDT16	hsa-miR-7113-3p
NUDT16	hsa-miR-188-5p
NUDT16	hsa-miR-181-3p
NUDT16	hsa-miR-605
NUDT16	hsa-miR-3090-5p
NUDT16	hsa-miR-34c-5p
NUDT16	hsa-miR-7007-5p
NUDT16	hsa-miR-8058
NUDT16	hsa-miR-7152-5p
NUDT16	hsa-miR-3120-3p
NUDT16	hsa-miR-518c-5p
NUDT16	hsa-miR-6922-3p
NUDT16	hsa-miR-3140-3p
NUDT16	hsa-miR-687
NUDT16	hsa-miR-6317
NUDT16	hsa-miR-374-5p

NUDT16	hsa-miR-4456
NUDT16	hsa-miR-4760-5p
NUDT16	hsa-miR-1238-3p
NUDT16	hsa-miR-3194-5p
NUDT16	hsa-miR-9768-3p
NUDT16	hsa-miR-600
NUDT16	hsa-miR-6895-3p
NUDT16	hsa-miR-323
NUDT16	hsa-miR-6910-3p
NUDT16	hsa-miR-532-5p
NUDT16	hsa-miR-6793-3p
NUDT16	hsa-miR-4668-3p
NUDT16	hsa-miR-193a-5p
NUDT16	hsa-miR-6930-5p
NUDT16	hsa-miR-873-5p
NUDT16	hsa-miR-31-3p
NUDT16	hsa-miR-6720-5p
NUDT16	hsa-miR-4489
NUDT16	hsa-miR-6832-5p
NUDT16	hsa-miR-2467-3p
NUDT16	hsa-miR-4793-3p
NUDT16	hsa-miR-578
NUDT16	hsa-miR-421
NUDT16	hsa-miR-3157-3p
NUDT16	hsa-miR-330-3p
NUDT16	hsa-miR-8119
NUDT16	hsa-miR-4774-5p
NUDT16	hsa-miR-130-3p
NUDT16	hsa-miR-132-3p
NUDT16	hsa-miR-8075
NUDT16	hsa-miR-6806-5p
NUDT16	hsa-miR-7855-5p
NUDT16	hsa-miR-1226-3p
NUDT16	hsa-miR-3119
NUDT16	hsa-miR-5625-5p
NUDT16	hsa-miR-513a-5p
NUDT16	hsa-miR-548az-5p
NUDT16	hsa-miR-4284
NUDT16	hsa-miR-3612
NUDT16	hsa-miR-376c
NUDT16	hsa-miR-6873-3p
NUDT16	hsa-miR-1255-5p
NUDT16	hsa-miR-518
NUDT16	hsa-miR-5589-3p
NUDT16	hsa-miR-378d
NUDT16	hsa-miR-7213-5p

NUDT16	hsa-miR-7107-3p
NUDT16	hsa-miR-628-5p
NUDT16	hsa-miR-10
NUDT16	hsa-miR-5129-5p
NUDT16	hsa-miR-652-5p
NUDT16	hsa-miR-6983-5p
NUDT16	hsa-miR-6732-5p
NUDT16	hsa-miR-8801
NUDT16	hsa-miR-155-3p
NUDT16	hsa-miR-7201-5p
NUDT16	hsa-miR-6907-5p
NUDT16	hsa-miR-294-3p
NUDT16	hsa-miR-3663-5p
NUDT16	hsa-miR-128-3p
NUDT16	hsa-miR-6780b-3p
NUDT16	hsa-miR-6752-5p
NUDT16	hsa-miR-4725-3p
NUDT16	hsa-miR-582-5p
NUDT16	hsa-miR-8857
NUDT16	hsa-miR-6982-3p
NUDT16	hsa-miR-142
NUDT16	hsa-miR-580-5p
NUDT16	hsa-miR-1191b-5p
NUDT16	hsa-miR-587
NUDT16	hsa-miR-1296
NUDT16	hsa-miR-208a-5p
NUDT16	hsa-miR-938
NUDT16	hsa-miR-4795-5p
NUDT16	hsa-miR-4756-3p
NUDT16	hsa-miR-3689-3p
NUDT16	hsa-miR-3606-3p
NUDT16	hsa-miR-7052-5p
NUDT16	hsa-miR-3147
NUDT16	hsa-miR-660-3p
NUDT16	hsa-miR-570-3p
NUDT16	hsa-miR-34b-5p
NUDT16	hsa-miR-466ade-3p
NUDT16	hsa-miR-1836
NUDT16	hsa-miR-6883-5p
NUDT16	hsa-miR-3078-3p
NUDT16	hsa-miR-4498
NUDT16	hsa-miR-3164
NUDT16	hsa-miR-362-5p
NUDT16	hsa-miR-3064
NUDT16	hsa-miR-703
NUDT16	hsa-miR-8842

NUDT16	hsa-miR-551-5p
NUDT16	hsa-miR-2681-3p
NUDT16	hsa-miR-3187-3p
NUDT16	hsa-miR-4794
NUDT16	hsa-miR-506-3p
NUDT16	hsa-miR-181a-3p
NUDT16	hsa-miR-6905-3p
NUDT16	hsa-miR-3662
NUDT16	hsa-miR-6890-3p
NUDT16	hsa-miR-4758-5p
NUDT16	hsa-miR-708-3p
NUDT16	hsa-miR-3138
NUDT16	hsa-miR-3622b-3p
NUDT16	hsa-miR-3176
NUDT16	hsa-miR-6397
NUDT16	hsa-miR-6824-3p
NUDT16	hsa-miR-1254
NUDT16	hsa-miR-6901-3p
NUDT16	hsa-miR-4463
NUDT16	hsa-miR-3671
NUDT16	hsa-miR-206-3p
NUDT16	hsa-miR-96-3p
NUDT16	hsa-miR-2350
NUDT16	hsa-miR-7159-3p
NUDT16	hsa-miR-6957-5p
NUDT16	hsa-miR-6824-5p
NUDT16	hsa-miR-4522
NUDT16	hsa-miR-6949-5p
NUDT16	hsa-miR-491-3p
NUDT16	hsa-miR-1292-5p
NUDT16	hsa-miR-324-3p
NUDT16	hsa-miR-7206-5p
NUDT16	hsa-miR-199-3p
NUDT16	hsa-miR-549-5p
NUDT16	hsa-miR-1839
NUDT16	hsa-miR-6318
NUDT16	hsa-miR-943
NUDT16	hsa-miR-7221-5p
NUDT16	hsa-miR-8819
NUDT16	hsa-miR-6888-3p
NUDT16	hsa-miR-454-3p
NUDT16	hsa-miR-295-5p
NUDT16	hsa-miR-4435
NUDT16	hsa-miR-624-5p
NUDT16	hsa-miR-520g-5p
NUDT16	hsa-miR-302d-5p

NUDT16	hsa-miR-374c-5p
NUDT16	hsa-miR-665-5p
NUDT16	hsa-miR-598-3p
NUDT16	hsa-miR-224-5p
NUDT16	hsa-miR-93-5p
NUDT16	hsa-miR-7688-5p
NUDT16	hsa-miR-655-5p
NUDT16	hsa-miR-17-3p
NUDT16	hsa-miR-4711-3p
NUDT16	hsa-miR-4792
NUDT16	hsa-miR-146b-3p
NUDT16	hsa-miR-7656-3p
NUDT16	hsa-miR-216b-5p
NUDT16	hsa-miR-666-3p
NUDT16	hsa-miR-7020-5p
NUDT16	hsa-miR-200c-5p
NUDT16	hsa-miR-8804
NUDT16	hsa-miR-136-5p
NUDT16	hsa-miR-29-5p
NUDT16	hsa-miR-1207-3p
NUDT16	hsa-miR-513
NUDT16	hsa-miR-3568
NUDT16	hsa-miR-6802-3p
NUDT16	hsa-miR-4775
NUDT16	hsa-miR-7156-5p
NUDT16	hsa-miR-582-5p
NUDT16	hsa-miR-4727-3p
NUDT16	hsa-miR-340-5p
NUDT16	hsa-miR-409
NUDT16	hsa-miR-7170-3p
NUDT16	hsa-miR-6723-5p
NUDT16	hsa-miR-506-3p
NUDT16	hsa-miR-516a
NUDT16	hsa-miR-6855-3p
NUDT16	hsa-miR-6966-5p
NUDT16	hsa-miR-8077
NUDT16	hsa-miR-7084-3p
NUDT16	hsa-miR-6981-5p
NUDT16	hsa-miR-4439
NUDT16	hsa-miR-7106-5p
NUDT16	hsa-miR-2115-3p
NUDT16	hsa-miR-203-5p
NUDT16	hsa-miR-7118-5p
NUDT16	hsa-miR-2368-3p
NUDT16	hsa-miR-1322
NUDT16	hsa-miR-3590-5p

NUDT16	hsa-miR-15
NUDT16	hsa-miR-2276-3p
NUDT16	hsa-miR-5696
NUDT16	hsa-miR-1255
NUDT16	hsa-miR-4524b-3p
NUDT16	hsa-miR-3126-5p
NUDT16	hsa-miR-215-3p
NUDT16	hsa-miR-6529-5p
NUDT16	hsa-miR-7208-5p
NUDT16	hsa-miR-548ar-3p
NUDT16	hsa-miR-516a-3p
NUDT16	hsa-miR-6976-3p
NUDT16	hsa-miR-6990-3p
NUDT16	hsa-miR-29b-1-5p
NUDT16	hsa-miR-664a-5p
NUDT16	hsa-miR-758-5p
NUDT16	hsa-miR-6845-5p
NUDT16	hsa-miR-5582-3p
NUDT16	hsa-miR-669d-5p
NUDT16	hsa-miR-6754-5p
NUDT16	hsa-miR-6728-3p
NUDT16	hsa-miR-8851
NUDT16	hsa-miR-1188-5p
NUDT16	hsa-miR-586
NUDT16	hsa-miR-5095
NUDT16	hsa-miR-4315
NUDT16	hsa-miR-22-3p
NUDT16	hsa-miR-6797-3p
NUDT16	hsa-miR-1207
NUDT16	hsa-miR-1323
NUDT16	hsa-miR-5196-3p
NUDT16	hsa-miR-301a-5p
NUDT16	hsa-miR-4724-5p
NUDT16	hsa-miR-219-5p
NUDT16	hsa-miR-7669-3p
NUDT16	hsa-miR-8814
NUDT16	hsa-miR-6790-5p
NUDT16	hsa-miR-7185-5p
NUDT16	hsa-miR-1894-3p
NUDT16	hsa-miR-693-5p
NUDT16	hsa-miR-1237-3p
NUDT16	hsa-miR-548an
NUDT16	hsa-miR-7012-3p
NUDT16	hsa-miR-4441
NUDT16	hsa-miR-4504
NUDT16	hsa-miR-7224-5p

NUDT16	hsa-miR-3929
NUDT16	hsa-miR-4446-5p
NUDT16	hsa-miR-605-5p
NUDT16	hsa-miR-1298
NUDT16	hsa-miR-138-1-3p
NUDT16	hsa-miR-3060-3p
NUDT16	hsa-miR-454
NUDT16	hsa-miR-503-5p
NUDT16	hsa-miR-6761-5p
NUDT16	hsa-miR-4750-3p
NUDT16	hsa-miR-676-3p
NUDT16	hsa-miR-597
NUDT16	hsa-miR-6849-3p
NUDT16	hsa-miR-24-5p
NUDT16	hsa-miR-589-5p
NUDT16	hsa-miR-646
NUDT16	hsa-miR-18
NUDT16	hsa-miR-2861
NUDT16	hsa-miR-499a-3p
NUDT16	hsa-miR-328
NUDT16	hsa-miR-7207-5p
NUDT16	hsa-miR-3136-5p
NUDT16	hsa-miR-873-5p
NUDT16	hsa-miR-101-1-5p
NUDT16	hsa-miR-105-5p
NUDT16	hsa-miR-758-3p
NUDT16	hsa-miR-1899
NUDT16	hsa-miR-550a-3p
NUDT16	hsa-miR-16-2-3p
NUDT16	hsa-miR-7007-3p
NUDT16	hsa-miR-197-3p
NUDT16	hsa-miR-485-3p
NUDT16	hsa-miR-514a-3p
NUDT16	hsa-miR-1284
NUDT16	hsa-miR-5131
NUDT16	hsa-miR-5623-3p
NUDT16	hsa-miR-1228-3p
NUDT16	hsa-miR-141-3p
NUDT16	hsa-miR-1286
NUDT16	hsa-miR-660-5p
NUDT16	hsa-miR-4700-3p
NUDT16	hsa-miR-4490
NUDT16	hsa-miR-548a-3p
NUDT16	hsa-miR-6126
NUDT16	hsa-miR-6829-3p
NUDT16	hsa-miR-4742

NUDT16	hsa-miR-1299
NUDT16	hsa-miR-7187-5p
NUDT16	hsa-miR-3668
NUDT16	hsa-miR-4684-5p
NUDT16	hsa-miR-6344
NUDT16	hsa-miR-1241
NUDT16	hsa-miR-1343-3p
NUDT16	hsa-miR-301-3p
NUDT16	hsa-miR-93-5p
NUDT16	hsa-miR-3925-3p
NUDT16	hsa-miR-4292
NUDT16	hsa-miR-4499
NUDT16	hsa-miR-4664-5p
NUDT16	hsa-miR-24-3p
NUDT16	hsa-miR-4419b
NUDT16	hsa-miR-1231-5p
NUDT16	hsa-miR-1224-3p
NUDT16	hsa-miR-4692
NUDT16	hsa-miR-6846-5p
NUDT16	hsa-miR-6826-3p
NUDT16	hsa-miR-7681-3p
NUDT16	hsa-miR-7-3p
NUDT16	hsa-miR-7197-3p
NUDT16	hsa-miR-211-3p
NUDT16	hsa-miR-3151-3p
NUDT16	hsa-miR-708-5p
NUDT16	hsa-miR-3586-5p
NUDT16	hsa-miR-6316
NUDT16	hsa-miR-7684-3p
NUDT16	hsa-miR-3611
NUDT16	hsa-miR-343
NUDT16	hsa-miR-4446
NUDT16	hsa-miR-6127
NUDT16	hsa-miR-4291
NUDT16	hsa-miR-1239
NUDT16	hsa-miR-3613-3p
NUDT16	hsa-miR-669j
NUDT16	hsa-miR-4312
NUDT16	hsa-miR-291-5p
NUDT16	hsa-miR-6950-5p
NUDT16	hsa-miR-140-3p.2
NUDT16	hsa-miR-764
NUDT16	hsa-miR-7189-3p
NUDT16	hsa-miR-455-3p
NUDT16	hsa-miR-6970-5p
NUDT16	hsa-miR-6889-3p

NUDT16	hsa-miR-125a-3p
NUDT16	hsa-miR-376a-2-5p
NUDT16	hsa-miR-7000-3p
NUDT16	hsa-miR-7659-5p
NUDT16	hsa-miR-7226-3p
NUDT16	hsa-miR-107-3p
NUDT16	hsa-miR-214-3p
NUDT16	hsa-miR-290
NUDT16	hsa-miR-6780a-3p
NUDT16	hsa-miR-1236-3p
NUDT16	hsa-miR-8853
NUDT16	hsa-miR-548g-3p
NUDT16	hsa-miR-362-5p
NUDT16	hsa-miR-466
NUDT16	hsa-miR-6764-5p
NUDT16	hsa-miR-7106-3p
NUDT16	hsa-miR-6857-3p
NUDT16	hsa-miR-301
NUDT16	hsa-miR-3065-5p
NUDT16	hsa-miR-371b-3p
NUDT16	hsa-miR-4539
NUDT16	hsa-miR-6874-3p
NUDT16	hsa-miR-888-5p
NUDT16	hsa-miR-30-3p
NUDT16	hsa-miR-345-5p
NUDT16	hsa-miR-3973
NUDT16	hsa-miR-6529
NUDT16	hsa-miR-7172-3p
NUDT16	hsa-miR-124-3p.2
NUDT16	hsa-miR-520
NUDT16	hsa-miR-4483
NUDT16	hsa-miR-6730-3p
NUDT16	hsa-miR-7054-3p
NUDT16	hsa-miR-450a
NUDT16	hsa-miR-4663
NUDT16	hsa-miR-1-3p
NUDT16	hsa-miR-7186-3p
NUDT16	hsa-miR-595
NUDT16	hsa-miR-142-5p
NUDT16	hsa-miR-101a-5p
NUDT16	hsa-miR-487-5p
NUDT16	hsa-miR-19
NUDT16	hsa-miR-1249-5p
NUDT16	hsa-miR-219c-3p
NUDT16	hsa-miR-5094
NUDT16	hsa-miR-4708-5p

NUDT16	hsa-miR-1255a
NUDT16	hsa-miR-483-5p
NUDT16	hsa-miR-465abc-3p
NUDT16	hsa-miR-4759
NUDT16	hsa-miR-874
NUDT16	hsa-miR-510
NUDT16	hsa-miR-3102-3p
NUDT16	hsa-miR-6887-3p
NUDT16	hsa-miR-8892
NUDT16	hsa-miR-4659a-3p
NUDT16	hsa-miR-6348
NUDT16	hsa-miR-767
NUDT16	hsa-miR-3921
NUDT16	hsa-miR-4457
NUDT16	hsa-miR-6836-3p
NUDT16	hsa-miR-126-3p.1
NUDT16	hsa-miR-4269
NUDT16	hsa-miR-7184-5p
NUDT16	hsa-miR-3548
NUDT16	hsa-miR-200a-3p
NUDT16	hsa-miR-4308
NUDT16	hsa-miR-384-3p
NUDT16	hsa-miR-33-5p
NUDT16	hsa-miR-889
NUDT16	hsa-miR-6800-5p
NUDT16	hsa-miR-872-3p
NUDT16	hsa-miR-3975
NUDT16	hsa-miR-1184
NUDT16	hsa-miR-6914-3p
NUDT16	hsa-miR-883a-5p
NUDT16	hsa-miR-8097
NUDT16	hsa-miR-6322
NUDT16	hsa-miR-8901
NUDT16	hsa-miR-129-5p
NUDT16	hsa-miR-141-3p
NUDT16	hsa-miR-1288-5p
NUDT16	hsa-miR-302a-5p
NUDT16	hsa-miR-6392-5p
NUDT16	hsa-miR-7974
NUDT16	hsa-miR-6510-5p
NUDT16	hsa-miR-532-3p
NUDT16	hsa-miR-6751-5p
NUDT16	hsa-miR-6845-3p
NUDT16	hsa-miR-8795
NUDT16	hsa-miR-6750-5p
NUDT16	hsa-miR-7194-3p

NUDT16	hsa-miR-8863
NUDT16	hsa-miR-15-5p
NUDT16	hsa-miR-1273h-3p
NUDT16	hsa-miR-187-3p
NUDT16	hsa-miR-550
NUDT16	hsa-miR-4289
NUDT16	hsa-miR-1668
NUDT16	hsa-miR-326-3p
NUDT16	hsa-miR-107-5p
NUDT16	hsa-miR-6823-5p
NUDT16	hsa-miR-345-5p
NUDT16	hsa-miR-367-5p
NUDT16	hsa-miR-484
NUDT16	hsa-miR-19b-5p
NUDT16	hsa-miR-182-5p
NUDT16	hsa-miR-23-5p
NUDT16	hsa-miR-4275
NUDT16	hsa-miR-323b-3p
NUDT16	hsa-miR-101-3p
NUDT16	hsa-miR-1188-3p
NUDT16	hsa-miR-7183-3p
NUDT16	hsa-miR-329
NUDT16	hsa-miR-7226-5p
NUDT16	hsa-miR-7168-3p
NUDT16	hsa-miR-302
NUDT16	hsa-miR-766-3p
NUDT16	hsa-miR-4653-5p
NUDT16	hsa-miR-7031-5p
NUDT16	hsa-miR-504-5p
NUDT16	hsa-miR-4254
NUDT16	hsa-miR-4795-3p
NUDT16	hsa-miR-5587-5p
NUDT16	hsa-miR-137-3p
NUDT16	hsa-miR-7178-3p
NUDT16	hsa-miR-532-5p
NUDT16	hsa-miR-585-5p
NUDT16	hsa-miR-371a-5p
NUDT16	hsa-miR-3544-5p
NUDT16	hsa-miR-1306-5p
NUDT16	hsa-miR-6911-5p
NUDT16	hsa-miR-653
NUDT16	hsa-miR-335-3p
NUDT16	hsa-miR-1894-5p
NUDT16	hsa-miR-7668-5p
NUDT16	hsa-miR-1298-3p
NUDT16	hsa-miR-876-5p

NUDT16	hsa-miR-7076-5p
NUDT16	hsa-miR-511-5p
NUDT16	hsa-miR-4474-3p
NUDT16	hsa-miR-3145-3p
NUDT16	hsa-miR-6829-5p
NUDT16	hsa-miR-146a-3p
NUDT16	hsa-miR-6832-3p
NUDT16	hsa-miR-122
NUDT16	hsa-miR-218-5p
NUDT16	hsa-miR-5622-3p
NUDT16	hsa-miR-513c-3p
NUDT16	hsa-miR-6814-5p
NUDT16	hsa-miR-3098-3p
NUDT16	hsa-miR-505-3p.2
NUDT16	hsa-miR-8865
NUDT16	hsa-miR-296-3p
NUDT16	hsa-miR-126-5p
NUDT16	hsa-miR-1968-3p
NUDT16	hsa-miR-4659b-5p
NUDT16	hsa-miR-3575
NUDT16	hsa-miR-152-3p
NUDT16	hsa-miR-4697-5p
NUDT16	hsa-miR-4726-3p
NUDT16	hsa-miR-384-5p
NUDT16	hsa-miR-3100-5p
NUDT16	hsa-miR-577-5p
NUDT16	hsa-miR-7166-5p
NUDT16	hsa-miR-361-3p
NUDT16	hsa-miR-7030-5p
NUDT16	hsa-miR-2893
NUDT16	hsa-miR-8873a
NUDT16	hsa-miR-455-3p.1
NUDT16	hsa-miR-6973a-3p
NUDT16	hsa-miR-4659b-3p
NUDT16	hsa-miR-4473
NUDT16	hsa-miR-7175-5p
NUDT16	hsa-miR-6855-5p
NUDT16	hsa-miR-505-5p
NUDT16	hsa-miR-1255b-5p
NUDT16	hsa-miR-188-5p
NUDT16	hsa-miR-679-3p
NUDT16	hsa-miR-7865
NUDT16	hsa-miR-7703
NUDT16	hsa-miR-6833-3p
NUDT16	hsa-miR-3106-3p
NUDT16	hsa-miR-4521

NUDT16	hsa-miR-340-5p
NUDT16	hsa-miR-297bc-3p
NUDT16	hsa-miR-1237-5p
NUDT16	hsa-miR-6896-3p
NUDT16	hsa-miR-672-5p
NUDT16	hsa-miR-3070-5p
NUDT16	hsa-miR-544b
NUDT16	hsa-miR-3113-3p
NUDT16	hsa-miR-6992-5p
NUDT16	hsa-miR-7042-5p
NUDT16	hsa-miR-1185-5p
NUDT16	hsa-miR-1930-5p
NUDT16	hsa-miR-494-3p
NUDT16	hsa-miR-1226-3p
NUDT16	hsa-miR-1982-3p
NUDT16	hsa-miR-372-3p
NUDT16	hsa-miR-3156-3p
NUDT16	hsa-miR-7066-5p
NUDT16	hsa-miR-7015-5p
NUDT16	hsa-miR-6848-5p
NUDT16	hsa-miR-19-5p
NUDT16	hsa-miR-7017-5p
NUDT16	hsa-miR-879-3p
NUDT16	hsa-miR-486-5p
NUDT16	hsa-miR-7001-3p
NUDT16	hsa-miR-222-3p
NUDT16	hsa-miR-7665-3p
NUDT16	hsa-miR-6529b
NUDT16	hsa-miR-8847
NUDT16	hsa-miR-20-3p
NUDT16	hsa-miR-6396
NUDT16	hsa-miR-8827
NUDT16	hsa-miR-6767-3p
NUDT16	hsa-miR-1245-3p
NUDT16	hsa-miR-556-5p
NUDT16	hsa-miR-124
NUDT16	hsa-miR-6134
NUDT16	hsa-miR-384
NUDT16	hsa-miR-6130
NUDT16	hsa-miR-378a-5p
NUDT16	hsa-miR-5187-3p
NUDT16	hsa-miR-6933-3p
NUDT16	hsa-miR-708-5p
NUDT16	hsa-miR-6792-3p
NUDT16	hsa-miR-3559-5p
NUDT16	hsa-miR-6945-5p

NUDT16	hsa-miR-873-5p.2
NUDT16	hsa-miR-548h-5p
NUDT16	hsa-miR-5197-3p
NUDT16	hsa-miR-939-5p
NUDT16	hsa-miR-2393
NUDT16	hsa-miR-3591
NUDT16	hsa-miR-547-3p
NUDT16	hsa-miR-10a-3p
NUDT16	hsa-miR-4747-5p
NUDT16	hsa-miR-147-5p
NUDT16	hsa-miR-4290
NUDT16	hsa-miR-4299
NUDT16	hsa-miR-344
NUDT16	hsa-miR-599
NUDT16	hsa-miR-2415-3p
NUDT16	hsa-miR-1253
NUDT16	hsa-miR-7087-3p
NUDT16	hsa-miR-202-3p
NUDT16	hsa-miR-6912-5p
NUDT16	hsa-miR-5192
NUDT16	hsa-miR-542-3p
NUDT16	hsa-miR-4769-3p
NUDT16	hsa-miR-204-5p
NUDT16	hsa-miR-676-5p
NUDT16	hsa-miR-3978
NUDT16	hsa-miR-520g-3p
NUDT16	hsa-miR-3594-5p
NUDT16	hsa-miR-467g
NUDT16	hsa-miR-7075-5p
NUDT16	hsa-miR-526b-5p
NUDT16	hsa-miR-293-5p
NUDT16	hsa-miR-344h-3p
NUDT16	hsa-miR-4418
NUDT16	hsa-miR-6904-5p
NUDT16	hsa-miR-344-5p
NUDT16	hsa-miR-6905-5p
NUDT16	hsa-miR-466mo-3p
NUDT16	hsa-miR-302b-5p
NUDT16	hsa-miR-449-5p
NUDT16	hsa-miR-3192
NUDT16	hsa-miR-1226-5p
NUDT16	hsa-miR-6985-5p
NUDT16	hsa-miR-6076
NUDT16	hsa-miR-6810-5p
NUDT16	hsa-miR-3682-3p
NUDT16	hsa-miR-383-3p

NUDT16	hsa-miR-3938-3p
NUDT16	hsa-miR-1931
NUDT16	hsa-miR-1265
NUDT16	hsa-miR-98-5p
NUDT16	hsa-miR-6784-3p
NUDT16	hsa-miR-6898-3p
NUDT16	hsa-miR-204-3p
NUDT16	hsa-miR-302-3p
NUDT16	hsa-miR-379-5p
NUDT16	hsa-miR-5125
NUDT16	hsa-miR-520f-5p
NUDT16	hsa-miR-6963-3p
NUDT16	hsa-miR-128-5p
NUDT16	hsa-miR-203-3p.2
NUDT16	hsa-miR-561-5p
NUDT16	hsa-miR-1892
NUDT16	hsa-miR-136-3p
NUDT16	hsa-miR-193
NUDT16	hsa-miR-151
NUDT16	hsa-miR-378j
NUDT16	hsa-miR-7231-5p
NUDT16	hsa-miR-206-5p
NUDT16	hsa-miR-650abc
NUDT16	hsa-miR-625-5p
NUDT16	hsa-miR-519b-3p
NUDT16	hsa-miR-1537-5p
NUDT16	hsa-miR-6501-5p
NUDT16	hsa-miR-6541
NUDT16	hsa-miR-6923-3p
NUDT16	hsa-miR-7032-3p
NUDT16	hsa-miR-495-3p
NUDT16	hsa-miR-4648
NUDT16	hsa-miR-9-5p
NUDT16	hsa-miR-3063-3p
NUDT16	hsa-miR-588
NUDT16	hsa-miR-6956-5p
NUDT16	hsa-miR-194-5p
NUDT16	hsa-miR-4529-5p
NUDT16	hsa-miR-509-3-5p
NUDT16	hsa-miR-6798-5p
NUDT16	hsa-miR-1911-5p
NUDT16	hsa-miR-584-5p
NUDT16	hsa-miR-584-3p
NUDT16	hsa-miR-2290
NUDT16	hsa-miR-1983
NUDT16	hsa-miR-7068-5p

NUDT16	hsa-miR-8854
NUDT16	hsa-miR-890
NUDT16	hsa-miR-6941-5p
NUDT16	hsa-miR-514-3p
NUDT16	hsa-miR-1960
NUDT16	hsa-miR-34bc-3p
NUDT16	hsa-miR-890-3p
NUDT16	hsa-miR-655
NUDT16	hsa-miR-677-5p
NUDT16	hsa-miR-7648-3p
NUDT16	hsa-miR-204-5p
NUDT16	hsa-miR-7218-3p
NUDT16	hsa-miR-3135b
NUDT16	hsa-miR-124-5p
NUDT16	hsa-miR-6867-3p
NUDT16	hsa-miR-5572
NUDT16	hsa-miR-33-3p
NUDT16	hsa-miR-7052-3p
NUDT16	hsa-miR-330-3p.2
NUDT16	hsa-miR-1178-3p
NUDT16	hsa-miR-3670
NUDT16	hsa-miR-379-3p
NUDT16	hsa-miR-1207-5p
NUDT16	hsa-miR-3154
NUDT16	hsa-miR-8829
NUDT16	hsa-miR-5582-5p
NUDT16	hsa-miR-3124-3p
NUDT16	hsa-miR-95-5p
NUDT16	hsa-miR-3189-5p
NUDT16	hsa-miR-6964-5p
NUDT16	hsa-miR-4271
NUDT16	hsa-miR-8791
NUDT16	hsa-miR-18-3p
NUDT16	hsa-miR-7057-5p
NUDT16	hsa-miR-669h-5p
NUDT16	hsa-miR-1912
NUDT16	hsa-miR-471-3p
NUDT16	hsa-miR-516
NUDT16	hsa-miR-2882
NUDT16	hsa-miR-6984-5p
NUDT16	hsa-miR-185-3p
NUDT16	hsa-miR-6791-3p
NUDT16	hsa-miR-3682-5p
NUDT16	hsa-miR-3096-3p
NUDT16	hsa-miR-598-5p
NUDT16	hsa-miR-8866

NUDT16	hsa-miR-548e-3p
NUDT16	hsa-miR-4719
NUDT16	hsa-miR-1236-3p
NUDT16	hsa-miR-134-3p
NUDT16	hsa-miR-1208
NUDT16	hsa-miR-6325
NUDT16	hsa-miR-3180-5p
NUDT16	hsa-miR-23b-5p
NUDT16	hsa-miR-1266-5p
NUDT16	hsa-miR-193-3p
NUDT16	hsa-miR-548az-3p
NUDT16	hsa-miR-181b-2-3p
NUDT16	hsa-miR-3692-5p
NUDT16	hsa-miR-5120
NUDT16	hsa-miR-15a-3p
NUDT16	hsa-miR-4267
NUDT16	hsa-miR-3544-3p
NUDT16	hsa-miR-7686-5p
NUDT16	hsa-miR-7064-5p
NUDT16	hsa-miR-4531
NUDT16	hsa-miR-5591-5p
NUDT16	hsa-miR-6716-5p
NUDT16	hsa-miR-4314
NUDT16	hsa-miR-6843-3p
NUDT16	hsa-miR-7048-5p
NUDT16	hsa-miR-8886
NUDT16	hsa-miR-619
NUDT16	hsa-miR-8083
NUDT16	hsa-miR-3651
NUDT16	hsa-miR-8818
NUDT16	hsa-miR-1185-5p
NUDT16	hsa-miR-487-3p
NUDT16	hsa-miR-3121-5p
NUDT16	hsa-miR-548ao-3p
NUDT16	hsa-miR-8106
NUDT16	hsa-miR-466c-5p
NUDT16	hsa-miR-5581-3p
NUDT16	hsa-miR-6932-3p
NUDT16	hsa-miR-4720-3p
NUDT16	hsa-miR-133b-5p
NUDT16	hsa-miR-7649-3p
NUDT16	hsa-miR-647
NUDT16	hsa-miR-4772-3p
NUDT16	hsa-miR-6914-5p
NUDT16	hsa-miR-8052
NUDT16	hsa-miR-2985

NUDT16	hsa-miR-7110-5p
NUDT16	hsa-miR-3654
NUDT16	hsa-miR-6734-5p
NUDT16	hsa-miR-519
NUDT16	hsa-miR-16-5p
NUDT16	hsa-miR-582-3p
NUDT16	hsa-miR-4325
NUDT16	hsa-miR-7223-5p
NUDT16	hsa-miR-7676-5p
NUDT16	hsa-miR-4322
NUDT16	hsa-miR-6401
NUDT16	hsa-miR-3064-5p
NUDT16	hsa-miR-330-3p
NUDT16	hsa-miR-7084-5p
NUDT16	hsa-miR-4519
NUDT16	hsa-miR-4695-5p
NUDT16	hsa-miR-6974-3p
NUDT16	hsa-miR-6842-3p
NUDT16	hsa-miR-5692a
NUDT16	hsa-miR-1913
NUDT16	hsa-miR-548as-3p
NUDT16	hsa-miR-6955-3p
NUDT16	hsa-miR-5118
NUDT16	hsa-miR-6801-3p
NUDT16	hsa-miR-3584-5p
NUDT16	hsa-miR-8888
NUDT16	hsa-miR-4536-3p
NUDT16	hsa-miR-372
NUDT16	hsa-miR-291-3p
NUDT16	hsa-miR-4268
NUDT16	hsa-miR-4698
NUDT16	hsa-miR-7204-5p
NUDT16	hsa-miR-544a
NUDT16	hsa-miR-432-5p
NUDT16	hsa-miR-449a
NUDT16	hsa-miR-7092-5p
NUDT16	hsa-miR-3150a-5p
NUDT16	hsa-miR-3665
NUDT16	hsa-miR-6930-3p
NUDT16	hsa-miR-6739-3p
NUDT16	hsa-miR-4739
NUDT16	hsa-miR-20a-3p
NUDT16	hsa-miR-181-5p
NUDT16	hsa-miR-7849-3p
NUDT16	hsa-miR-490-3p
NUDT16	hsa-miR-3942-3p

NUDT16	hsa-miR-7008-3p
NUDT16	hsa-miR-649
NUDT16	hsa-miR-4278
NUDT16	hsa-miR-605-3p
NUDT16	hsa-miR-7205-3p
NUDT16	hsa-miR-489-3p
NUDT16	hsa-miR-8821
NUDT16	hsa-miR-8799
NUDT16	hsa-miR-3174
NUDT16	hsa-miR-7191-3p
NUDT16	hsa-miR-3186-5p
NUDT16	hsa-miR-383-5p.1
NUDT16	hsa-miR-4680-3p
NUDT16	hsa-miR-7019-5p
NUDT16	hsa-miR-3541
NUDT16	hsa-miR-542-5p
NUDT16	hsa-miR-4529-3p
NUDT16	hsa-miR-6841-3p
NUDT16	hsa-miR-6515-3p
NUDT16	hsa-miR-7111-3p
NUDT16	hsa-miR-361-5p
NUDT16	hsa-miR-2297
NUDT16	hsa-miR-7976
NUDT16	hsa-miR-502-5p
NUDT16	hsa-miR-552
NUDT16	hsa-miR-296-5p
NUDT16	hsa-miR-694
NUDT16	hsa-miR-3977
NUDT16	hsa-miR-3200-5p
NUDT16	hsa-miR-323a-5p
NUDT16	hsa-miR-4460
NUDT16	hsa-miR-5001-5p
NUDT16	hsa-miR-6830-5p
NUDT16	hsa-miR-4524a-3p
NUDT16	hsa-miR-4743-3p
NUDT16	hsa-miR-217
NUDT16	hsa-miR-6817-3p
NUDT16	hsa-miR-485
NUDT16	hsa-miR-7164-5p
NUDT16	hsa-miR-885-3p
NUDT16	hsa-miR-7035-5p
NUDT16	hsa-miR-7671-5p
NUDT16	hsa-miR-650d
NUDT16	hsa-miR-4999-3p
NUDT16	hsa-miR-6481
NUDT16	hsa-miR-548av-5p

NUDT16	hsa-miR-3110-5p
NUDT16	hsa-miR-338-5p
NUDT16	hsa-miR-98-5p
NUDT16	hsa-miR-3170
NUDT16	hsa-miR-517a-3p
NUDT16	hsa-miR-6873-5p
NUDT16	hsa-miR-504-5p.1
NUDT16	hsa-miR-28-3p
NUDT16	hsa-miR-6727-3p
NUDT16	hsa-miR-8869
NUDT16	hsa-miR-146-5p
NUDT16	hsa-miR-6888-5p
NUDT16	hsa-miR-3653-3p
NUDT16	hsa-miR-5591-3p
NUDT16	hsa-miR-339-5p
NUDT16	hsa-miR-5006-3p
NUDT16	hsa-miR-3173-3p
NUDT16	hsa-miR-516b-3p
NUDT16	hsa-miR-6756-5p
NUDT16	hsa-miR-423-3p
NUDT16	hsa-miR-6899-5p
NUDT16	hsa-miR-548f-3p
NUDT16	hsa-miR-2110
NUDT16	hsa-miR-361-5p
NUDT16	hsa-miR-139-5p
NUDT16	hsa-miR-6858-5p
NUDT16	hsa-miR-508-3p
NUDT16	hsa-miR-3089-5p
NUDT16	hsa-miR-3924
NUDT16	hsa-miR-518bc-3p
NUDT16	hsa-miR-216c-3p
NUDT16	hsa-miR-876
NUDT16	hsa-miR-21-3p
NUDT16	hsa-miR-1343-5p
NUDT16	hsa-miR-192-3p
NUDT16	hsa-miR-221-3p
NUDT16	hsa-miR-6737-5p
NUDT16	hsa-miR-4270
NUDT16	hsa-miR-4714-3p
NUDT16	hsa-miR-219-3p
NUDT16	hsa-miR-376c-3p
NUDT16	hsa-miR-5584-5p
NUDT16	hsa-miR-6392-3p
NUDT16	hsa-miR-3189-3p
NUDT16	hsa-miR-302a-3p
NUDT16	hsa-miR-331-3p

NUDT16	hsa-miR-6847-5p
NUDT16	hsa-miR-365a-3p
NUDT16	hsa-miR-669c-3p
NUDT16	hsa-miR-6828-3p
NUDT16	hsa-miR-7231-3p
NUDT16	hsa-miR-424-5p
NUDT16	hsa-miR-6319
NUDT16	hsa-miR-6403
NUDT16	hsa-miR-7016-3p
NUDT16	hsa-miR-6886-3p
NUDT16	hsa-miR-3928-5p
NUDT16	hsa-miR-651
NUDT16	hsa-miR-3190-5p
NUDT16	hsa-miR-514a-3p
NUDT16	hsa-miR-211-5p
NUDT16	hsa-miR-223-5p
NUDT16	hsa-miR-137
NUDT16	hsa-miR-1296-5p
NUDT16	hsa-miR-1193-5p
NUDT16	hsa-miR-370-5p
NUDT16	hsa-miR-7078-3p
NUDT16	hsa-miR-216b-3p
NUDT16	hsa-miR-21-5p
NUDT16	hsa-miR-382-5p
NUDT16	hsa-miR-4252
NUDT16	hsa-miR-8834b
NUDT16	hsa-miR-34c-3p
NUDT16	hsa-miR-7074-5p
NUDT16	hsa-miR-150-5p
NUDT16	hsa-miR-7664-5p
NUDT16	hsa-miR-409-5p
NUDT16	hsa-miR-6803-5p
NUDT16	hsa-let-7g-3p
NUDT16	hsa-miR-451a
NUDT16	hsa-miR-4742-3p
NUDT16	hsa-miR-130b-5p
NUDT16	hsa-miR-1915-3p
NUDT16	hsa-miR-7081-5p
NUDT16	hsa-miR-4514
NUDT16	hsa-miR-3074-5p
NUDT16	hsa-miR-6949-3p
NUDT16	hsa-miR-20
NUDT16	hsa-miR-920
NUDT16	hsa-miR-206
NUDT16	hsa-miR-7039-3p
NUDT16	hsa-miR-506-5p

NUDT16	hsa-miR-4718
NUDT16	hsa-miR-378g
NUDT16	hsa-miR-495-3p
NUDT16	hsa-miR-7198-5p
NUDT16	hsa-miR-1962
NUDT16	hsa-miR-450-5p
NUDT16	hsa-miR-4799-3p
NUDT16	hsa-miR-7211-3p
NUDT16	hsa-miR-465d-3p
NUDT16	hsa-miR-6852-5p
NUDT16	hsa-miR-6997-5p
NUDT16	hsa-miR-4763-3p
NUDT16	hsa-miR-3099-3p
NUDT16	hsa-miR-6817-5p
NUDT16	hsa-miR-504
NUDT16	hsa-miR-149-3p
NUDT16	hsa-miR-643
NUDT16	hsa-miR-30a-3p
NUDT16	hsa-miR-6966-3p
NUDT16	hsa-miR-592-5p
NUDT16	hsa-miR-1949
NUDT16	hsa-miR-7180-3p
NUDT16	hsa-miR-6789-5p
NUDT16	hsa-miR-4674
NUDT16	hsa-miR-18b-3p
NUDT16	hsa-miR-4803
NUDT16	hsa-miR-1285-5p
NUDT16	hsa-miR-670-3p
NUDT16	hsa-miR-4677-3p
NUDT16	hsa-miR-525-5p
NUDT16	hsa-miR-466g
NUDT16	hsa-miR-589-5p
NUDT16	hsa-miR-3103-3p
NUDT16	hsa-miR-7050-5p
NUDT16	hsa-miR-767-3p
NUDT16	hsa-miR-6366
NUDT16	hsa-miR-154-3p
NUDT16	hsa-miR-8843
NUDT16	hsa-miR-3160-5p
NUDT16	hsa-miR-4724-3p
NUDT16	hsa-miR-8900
NUDT16	hsa-miR-3938
NUDT16	hsa-miR-6964-3p
NUDT16	hsa-miR-6086
NUDT16	hsa-miR-1271-3p
NUDT16	hsa-miR-6758-3p

NUDT16	hsa-miR-491-5p
NUDT16	hsa-miR-4261
NUDT16	hsa-miR-3616-3p
NUDT16	hsa-miR-3936
NUDT16	hsa-miR-3173-5p
NUDT16	hsa-miR-432-5p
NUDT16	hsa-miR-550b-2-5p
NUDT16	hsa-miR-6872-3p
NUDT16	hsa-miR-139-5p
NUDT16	hsa-miR-4797-3p
NUDT16	hsa-miR-6834-5p
NUDT16	hsa-miR-4434
NUDT16	hsa-miR-4448
NUDT16	hsa-miR-6724-5p
NUDT16	hsa-miR-30-5p
NUDT16	hsa-miR-6090
NUDT16	hsa-miR-17-5p
NUDT16	hsa-miR-6975-3p
NUDT16	hsa-miR-7157-3p
NUDT16	hsa-miR-151-3p
NUDT16	hsa-miR-5194
NUDT16	hsa-miR-127-5p
NUDT16	hsa-miR-873-3p
NUDT16	hsa-miR-4493
NUDT16	hsa-miR-7065-3p
NUDT16	hsa-miR-1243-5p
NUDT16	hsa-miR-34-5p
NUDT16	hsa-miR-520c-3p
NUDT16	hsa-miR-6944-5p
NUDT16	hsa-miR-299b-5p
NUDT16	hsa-miR-623
NUDT16	hsa-miR-1b-5p
NUDT16	hsa-miR-2183
NUDT16	hsa-miR-7079-5p
NUDT16	hsa-miR-872-5p
NUDT16	hsa-miR-7656-5p
NUDT16	hsa-miR-935
NUDT16	hsa-miR-8120
NUDT16	hsa-miR-3549
NUDT16	hsa-miR-301-5p
NUDT16	hsa-miR-140-5p
NUDT16	hsa-miR-555
NUDT16	hsa-miR-631
NUDT16	hsa-miR-7163-5p
NUDT16	hsa-miR-125-5p
NUDT16	hsa-miR-664a-3p

NUDT16	hsa-miR-335-5p
NUDT16	hsa-miR-526
NUDT16	hsa-miR-7212-3p
NUDT16	hsa-miR-890-5p
NUDT16	hsa-miR-153-3p
NUDT16	hsa-miR-592-3p
NUDT16	hsa-miR-8059
NUDT16	hsa-miR-6760-3p
NUDT16	hsa-miR-7227-5p
NUDT16	hsa-miR-6771-3p
NUDT16	hsa-miR-4313
NUDT16	hsa-miR-6881-3p
NUDT16	hsa-miR-577
NUDT16	hsa-miR-3679-3p
NUDT16	hsa-miR-1278
NUDT16	hsa-miR-874-3p
NUDT16	hsa-miR-374c-3p
NUDT16	hsa-miR-1291
NUDT16	hsa-miR-7153-3p
NUDT16	hsa-miR-550a-5p
NUDT16	hsa-miR-1273h-5p
NUDT16	hsa-miR-7031-3p
NUDT16	hsa-miR-6791-5p
NUDT16	hsa-miR-1290
NUDT16	hsa-miR-448-3p
NUDT16	hsa-miR-548i-3p
NUDT16	hsa-miR-888-3p
NUDT16	hsa-miR-607
NUDT16	hsa-miR-141-5p
NUDT16	hsa-miR-188-3p
NUDT16	hsa-miR-651-3p
NUDT16	hsa-miR-1297
NUDT16	hsa-miR-3150b-5p
NUDT16	hsa-miR-6529-3p
NUDT16	hsa-miR-223-3p
NUDT16	hsa-miR-677
NUDT16	hsa-miR-192-5p
NUDT16	hsa-miR-4635
NUDT16	hsa-miR-653-5p
NUDT16	hsa-miR-2467-5p
NUDT16	hsa-miR-4778-5p
NUDT16	hsa-miR-770-5p
NUDT16	hsa-miR-7005-3p
NUDT16	hsa-miR-6378
NUDT16	hsa-miR-4326
NUDT16	hsa-miR-4753-5p

NUDT16	hsa-miR-493-5p
NUDT16	hsa-miR-7026-5p
NUDT16	hsa-miR-7237-5p
NUDT16	hsa-miR-704
NUDT16	hsa-miR-6822-3p
NUDT16	hsa-miR-761
NUDT16	hsa-miR-145-5p
NUDT16	hsa-miR-377-3p
NUDT16	hsa-miR-7078-5p
NUDT16	hsa-miR-3667-3p
NUDT16	hsa-miR-692
NUDT16	hsa-miR-514a-5p
NUDT16	hsa-miR-520-5p
NUDT16	hsa-miR-4470
NUDT16	hsa-miR-1287-5p
NUDT16	hsa-miR-466bcp-3p
NUDT16	hsa-miR-4263
NUDT16	hsa-miR-3062-5p
NUDT16	hsa-miR-26-3p
NUDT16	hsa-miR-326
NUDT16	hsa-miR-4423-5p
NUDT16	hsa-miR-3127-3p
NUDT16	hsa-miR-7515
NUDT16	hsa-miR-634
NUDT16	hsa-miR-22-5p
NUDT16	hsa-miR-5579-5p
NUDT16	hsa-miR-2114-5p
NUDT16	hsa-miR-7112-3p
NUDT16	hsa-miR-4300
NUDT16	hsa-miR-939-3p
NUDT16	hsa-miR-6963-5p
NUDT16	hsa-miR-655-3p
NUDT16	hsa-miR-7977
NUDT16	hsa-miR-507
NUDT16	hsa-miR-8872
NUDT16	hsa-miR-6831-5p
NUDT16	hsa-miR-27a-5p
NUDT16	hsa-miR-8904a
NUDT16	hsa-miR-4765
NUDT16	hsa-miR-6132
NUDT16	hsa-miR-665
NUDT16	hsa-miR-187-5p
NUDT16	hsa-miR-5627-5p
NUDT16	hsa-miR-3064-3p
NUDT16	hsa-miR-877-5p
NUDT16	hsa-miR-6834-3p

NUDT16	hsa-miR-3098-5p
NUDT16	hsa-miR-129-5p
NUDT16	hsa-miR-1470
NUDT16	hsa-miR-934-3p
NUDT16	hsa-miR-3199
NUDT16	hsa-miR-4721
NUDT16	hsa-miR-5186
NUDT16	hsa-miR-4330
NUDT16	hsa-miR-6856-3p
NUDT16	hsa-miR-465-3p
NUDT16	hsa-miR-669b-5p
NUDT16	hsa-miR-4307
NUDT16	hsa-miR-5707
NUDT16	hsa-miR-3544
NUDT16	hsa-miR-6501-3p
NUDT16	hsa-miR-195-3p
NUDT16	hsa-miR-483-3p.1
NUDT16	hsa-miR-3944-5p
NUDT16	hsa-miR-4502
NUDT16	hsa-miR-650
NUDT16	hsa-miR-885-5p
NUDT16	hsa-miR-410-5p
NUDT16	hsa-miR-509-3p
NUDT16	hsa-miR-3920
NUDT16	hsa-miR-6755-5p
NUDT16	hsa-miR-1245b-3p
NUDT16	hsa-miR-651-5p
NUDT16	hsa-miR-7032-5p
NUDT16	hsa-miR-1976
NUDT16	hsa-miR-1273d
NUDT16	hsa-miR-3076-5p
NUDT16	hsa-miR-3661
NUDT16	hsa-miR-659-3p
NUDT16	hsa-miR-543
NUDT16	hsa-miR-4732-5p
NUDT16	hsa-miR-4526
NUDT16	hsa-miR-612
NUDT16	hsa-miR-883-5p
NUDT16	hsa-miR-518-3p
NUDT16	hsa-miR-1292-5p
NUDT16	hsa-miR-4780
NUDT16	hsa-miR-7193-5p
NUDT16	hsa-miR-425-5p
NUDT16	hsa-miR-744-3p
NUDT16	hsa-miR-4640-5p
NUDT16	hsa-miR-7083-3p

NUDT16	hsa-miR-433-3p
NUDT16	hsa-miR-6857-5p
NUDT16	hsa-miR-4743-5p
NUDT16	hsa-miR-2370-5p
NUDT16	hsa-miR-4279
NUDT16	hsa-miR-879-5p
NUDT16	hsa-miR-217-5p
NUDT16	hsa-miR-3085
NUDT16	hsa-miR-5697-3p
NUDT16	hsa-miR-8070
NUDT16	hsa-miR-3065-3p
NUDT16	hsa-miR-1943-3p
NUDT16	hsa-miR-545-5p
NUDT16	hsa-miR-6082
NUDT16	hsa-miR-6893-5p
NUDT16	hsa-miR-2115-5p
NUDT16	hsa-miR-6839-5p
NUDT16	hsa-miR-409-3p
NUDT16	hsa-miR-3691-3p
NUDT16	hsa-miR-669k-5p
NUDT16	hsa-miR-3066-3p
NUDT16	hsa-miR-3116
NUDT16	hsa-miR-1178-5p
NUDT16	hsa-miR-1238-5p
NUDT16	hsa-miR-342-3p
NUDT16	hsa-miR-6908-5p
NUDT16	hsa-miR-4520-3p
NUDT16	hsa-miR-583
NUDT16	hsa-miR-8887
NUDT16	hsa-miR-1199-5p
NUDT16	hsa-miR-4478
NUDT16	hsa-miR-4474-5p
NUDT16	hsa-miR-544-3p
NUDT16	hsa-miR-6806-3p
NUDT16	hsa-miR-4477a
NUDT16	hsa-miR-5196-5p
NUDT16	hsa-miR-1193
NUDT16	hsa-miR-5590-5p
NUDT16	hsa-miR-6937-3p
NUDT16	hsa-miR-698-5p
NUDT16	hsa-miR-6848-3p
NUDT16	hsa-miR-7067-5p
NUDT16	hsa-miR-629-3p
NUDT16	hsa-miR-129-2-3p
NUDT16	hsa-miR-155-5p
NUDT16	hsa-miR-335-5p

NUDT16	hsa-miR-4777-3p
NUDT16	hsa-miR-4518
NUDT16	hsa-miR-329-5p
NUDT16	hsa-miR-29b-2-5p
NUDT16	hsa-miR-9b-3p
NUDT16	hsa-miR-4661-5p
NUDT16	hsa-miR-4722-5p
NUDT16	hsa-miR-200bc-3p
NUDT16	hsa-miR-1-3p
NUDT16	hsa-miR-211-5p
NUDT16	hsa-miR-3943
NUDT16	hsa-miR-28-5p
NUDT16	hsa-miR-6919-3p
NUDT16	hsa-miR-7018-3p
NUDT16	hsa-miR-2355-5p
NUDT16	hsa-miR-1896
NUDT16	hsa-miR-661
NUDT16	hsa-miR-3680-3p
NUDT16	hsa-miR-8908c
NUDT16	hsa-miR-218-5p
NUDT16	hsa-miR-1266-3p
NUDT16	hsa-miR-7108-5p
NUDT16	hsa-miR-7062-5p
NUDT16	hsa-miR-7085-3p
NUDT16	hsa-miR-1942
NUDT16	hsa-miR-297
NUDT16	hsa-miR-7653-3p
NUDT16	hsa-miR-544a
NUDT16	hsa-miR-5703
NUDT16	hsa-miR-8840
NUDT16	hsa-miR-7687-5p
NUDT16	hsa-miR-6387
NUDT16	hsa-miR-7029-3p
NUDT16	hsa-miR-148a-5p
NUDT16	hsa-miR-4482-3p
NUDT16	hsa-miR-365-3p
NUDT16	hsa-miR-21-5p
NUDT16	hsa-miR-4265
NUDT16	hsa-miR-30c-2-3p
NUDT16	hsa-miR-3087-5p
NUDT16	hsa-miR-6899-3p
NUDT16	hsa-miR-3938-5p
NUDT16	hsa-miR-570-3p
NUDT16	hsa-miR-558
NUDT16	hsa-miR-201-3p
NUDT16	hsa-miR-133b

NUDT16	hsa-miR-4510
NUDT16	hsa-miR-3658
NUDT16	hsa-miR-1270
NUDT16	hsa-miR-1279
NUDT16	hsa-miR-302c-3p.2
NUDT16	hsa-miR-4294
NUDT16	hsa-miR-205-5p
NUDT16	hsa-miR-6854-5p
NUDT16	hsa-miR-496-5p
NUDT16	hsa-miR-7021-3p
NUDT16	hsa-miR-5195-5p
NUDT16	hsa-miR-186-3p
NUDT16	hsa-miR-5133
NUDT16	hsa-miR-2114-5p
NUDT16	hsa-miR-6925-3p
NUDT16	hsa-miR-3652
NUDT16	hsa-miR-4472
NUDT16	hsa-miR-7689-3p
NUDT16	hsa-miR-295-3p
NUDT16	hsa-miR-6953-3p
NUDT16	hsa-miR-1293
NUDT16	hsa-miR-1233-3p
NUDT16	hsa-miR-1964-5p
NUDT16	hsa-miR-8832
NUDT16	hsa-miR-143-3p
NUDT16	hsa-miR-670
NUDT16	hsa-miR-4444
NUDT16	hsa-miR-3090-3p
NUDT16	hsa-miR-216b
NUDT16	hsa-miR-7674-3p
NUDT16	hsa-miR-5110
NUDT16	hsa-miR-4653-3p
NUDT16	hsa-miR-1273g-3p
NUDT16	hsa-miR-6502-5p
NUDT16	hsa-miR-518g-3p
NUDT16	hsa-miR-3622a-3p
NUDT16	hsa-miR-4778-3p
NUDT16	hsa-miR-344a-3p
NUDT16	hsa-miR-27b-5p
NUDT16	hsa-miR-124-3p
NUDT16	hsa-miR-185-5p
NUDT16	hsa-miR-497-5p
NUDT16	hsa-miR-6514-3p
NUDT16	hsa-miR-6897-5p
NUDT16	hsa-miR-1910-5p
NUDT16	hsa-miR-299-3p

NUDT16	hsa-miR-6776-5p
NUDT16	hsa-miR-6124
NUDT16	hsa-miR-5693
NUDT16	hsa-miR-3617-3p
NUDT16	hsa-miR-8054
NUDT16	hsa-miR-3200-3p
NUDT16	hsa-miR-6365
NUDT16	hsa-miR-486-3p
NUDT16	hsa-miR-3653-5p
NUDT16	hsa-miR-382-5p
NUDT16	hsa-miR-3169
NUDT16	hsa-miR-3106-5p
NUDT16	hsa-miR-5618-3p
NUDT16	hsa-miR-4689
NUDT16	hsa-miR-4297
NUDT16	hsa-miR-6416-3p
NUDT16	hsa-miR-924
NUDT16	hsa-miR-6882-3p
NUDT16	hsa-miR-539-5p
NUDT16	hsa-miR-370
NUDT16	hsa-miR-6940-5p
NUDT16	hsa-miR-205-5p
NUDT16	hsa-miR-3686
NUDT16	hsa-miR-7194-5p
NUDT16	hsa-miR-503-5p
NUDT16	hsa-miR-6737-3p
NUDT16	hsa-miR-7578
NUDT16	hsa-miR-30e
NUDT16	hsa-miR-3130-5p
NUDT16	hsa-miR-5700
NUDT16	hsa-miR-7852-3p
NUDT16	hsa-miR-7227-3p
NUDT16	hsa-miR-6129
NUDT16	hsa-miR-4471
NUDT16	hsa-miR-5706
NUDT16	hsa-miR-92a-5p
NUDT16	hsa-miR-338-3p
NUDT16	hsa-miR-4762-3p
NUDT16	hsa-miR-146
NUDT16	hsa-miR-657
NUDT16	hsa-miR-4742-5p
NUDT16	hsa-miR-2117
NUDT16	hsa-miR-3909
NUDT16	hsa-miR-4753-3p
NUDT16	hsa-miR-8824
NUDT16	hsa-miR-2276-5p

NUDT16	hsa-miR-877-3p
NUDT16	hsa-miR-216-5p
NUDT16	hsa-miR-103b
NUDT16	hsa-miR-6875-3p
NUDT16	hsa-miR-7181-5p
NUDT16	hsa-miR-4646-5p
NUDT16	hsa-miR-1904
NUDT16	hsa-miR-140-3p.1
NUDT16	hsa-miR-7704
NUDT16	hsa-miR-4481
NUDT16	hsa-miR-196b-3p
NUDT16	hsa-miR-7180
NUDT16	hsa-miR-216
NUDT16	hsa-miR-344g-3p
NUDT16	hsa-miR-6997-3p
NUDT16	hsa-miR-6954-3p
NUDT16	hsa-miR-653-3p
NUDT16	hsa-miR-3192-3p
NUDT16	hsa-miR-3160-3p
NUDT16	hsa-miR-513a
NUDT16	hsa-miR-1966-3p
NUDT16	hsa-miR-470-5p
NUDT16	hsa-miR-7022-3p
NUDT16	hsa-miR-372-5p
NUDT16	hsa-miR-6133
NUDT16	hsa-miR-8846
NUDT16	hsa-miR-4266
NUDT16	hsa-miR-1231
NUDT16	hsa-miR-5626-5p
NUDT16	hsa-miR-881-5p
NUDT16	hsa-miR-5622-5p
NUDT16	hsa-miR-7662-5p
NUDT16	hsa-miR-3650
NUDT16	hsa-miR-6902-5p
NUDT16	hsa-miR-449b-3p
NUDT16	hsa-miR-4513
NUDT16	hsa-miR-138-3p
NUDT16	hsa-miR-4487
NUDT16	hsa-miR-3917
NUDT16	hsa-miR-6796-3p
NUDT16	hsa-miR-5787
NUDT16	hsa-miR-548m
NUDT16	hsa-miR-6960-3p
NUDT16	hsa-miR-450a-2-3p
NUDT16	hsa-miR-1970
NUDT16	hsa-miR-8807

NUDT16	hsa-miR-4468
NUDT16	hsa-miR-5113
NUDT16	hsa-miR-1250-3p
NUDT16	hsa-miR-4296
NUDT16	hsa-miR-125b-2-3p
NUDT16	hsa-miR-3922-3p
NUDT16	hsa-miR-5684
NUDT16	hsa-miR-878-3p
NUDT16	hsa-miR-6835-5p
NUDT16	hsa-miR-6971-3p
NUDT16	hsa-miR-489
NUDT16	hsa-miR-4777-5p
NUDT16	hsa-miR-130
NUDT16	hsa-miR-1909-5p
NUDT16	hsa-miR-485-5p
NUDT16	hsa-miR-3606-5p
NUDT16	hsa-miR-30b-3p
NUDT16	hsa-miR-3470a
NUDT16	hsa-miR-1246
NUDT16	hsa-miR-4482-5p
NUDT16	hsa-miR-494
NUDT16	hsa-miR-7681-5p
NUDT16	hsa-miR-760
NUDT16	hsa-miR-3191-5p
NUDT16	hsa-miR-7657-3p
NUDT16	hsa-miR-5099
NUDT16	hsa-miR-4650
NUDT16	hsa-miR-6961-3p
NUDT16	hsa-miR-7033-5p
NUDT16	hsa-miR-4515
NUDT16	hsa-miR-6321
NUDT16	hsa-miR-4755-3p
NUDT16	hsa-miR-1261
NUDT16	hsa-miR-147a
NUDT16	hsa-miR-6729-3p
NUDT16	hsa-miR-3058-3p
NUDT16	hsa-miR-218-2-3p
NUDT16	hsa-miR-1-5p
NUDT16	hsa-miR-448
NUDT16	hsa-miR-4743-5p
NUDT16	hsa-miR-320e
NUDT16	hsa-miR-142-3p.2
NUDT16	hsa-miR-573
NUDT16	hsa-miR-6715b-5p
NUDT16	hsa-miR-1269a
NUDT16	hsa-miR-134-5p

NUDT16	hsa-miR-5189-3p
NUDT16	hsa-miR-126-3p
NUDT16	hsa-miR-4274
NUDT16	hsa-miR-3577
NUDT16	hsa-miR-8550
NUDT16	hsa-miR-186-5p
NUDT16	hsa-miR-3127-5p
NUDT16	hsa-miR-7196-5p
NUDT16	hsa-miR-6859-5p
NUDT16	hsa-miR-6998-5p
NUDT16	hsa-miR-579
NUDT16	hsa-miR-1231-3p
NUDT16	hsa-miR-2113
NUDT16	hsa-miR-153-5p
NUDT16	hsa-miR-3941
NUDT16	hsa-miR-2483
NUDT16	hsa-miR-590-5p
NUDT16	hsa-miR-1183
NUDT16	hsa-miR-764-5p
NUDT16	hsa-miR-6764-3p
NUDT16	hsa-miR-7702
NUDT16	hsa-miR-3073b-3p
NUDT16	hsa-miR-3552
NUDT16	hsa-miR-203-3p.1
NUDT16	hsa-miR-552-3p
NUDT16	hsa-miR-3192-5p
NUDT16	hsa-miR-219a-5p
NUDT16	hsa-miR-1225-3p
NUDT16	hsa-miR-615-5p
NUDT16	hsa-miR-7200-5p
NUDT16	hsa-miR-7663-3p
NUDT16	hsa-miR-6511a-5p
NUDT16	hsa-miR-486-5p
NUDT16	hsa-miR-3557-3p
NUDT16	hsa-miR-3935
NUDT16	hsa-miR-1952
NUDT16	hsa-miR-466l-5p
NUDT16	hsa-miR-4694-5p
NUDT16	hsa-miR-6507-3p
NUDT16	hsa-miR-327
NUDT16	hsa-miR-2378
NUDT16	hsa-miR-6128
NUDT16	hsa-miR-223-3p
NUDT16	hsa-miR-6769a-3p
NUDT16	hsa-miR-7178-5p
NUDT16	hsa-miR-8899

NUDT16	hsa-miR-124-3p.1
NUDT16	hsa-miR-7211-5p
NUDT16	hsa-miR-155-5p
NUDT16	hsa-miR-518d-5p
NUDT16	hsa-miR-1838
NUDT16	hsa-miR-7199-3p
NUDT16	hsa-miR-766-3p
NUDT16	hsa-miR-1260b
NUDT16	hsa-miR-512
NUDT16	hsa-miR-508-3p
NUDT16	hsa-miR-190-5p
NUDT16	hsa-miR-1260a
NUDT16	hsa-miR-1938
NUDT16	hsa-miR-33a-3p
NUDT16	hsa-miR-7049-5p
NUDT16	hsa-miR-106
NUDT16	hsa-miR-6742-5p
NUDT16	hsa-miR-4251
NUDT16	hsa-miR-2298
NUDT16	hsa-miR-6900-5p
NUDT16	hsa-miR-4776-3p
NUDT16	hsa-miR-6087
NUDT16	hsa-miR-103-3p
NUDT16	hsa-miR-8085
NUDT16	hsa-miR-5685
NUDT16	hsa-miR-7090-5p
NUDT16	hsa-miR-143-5p
NUDT16	hsa-miR-29
NUDT16	hsa-miR-7033-3p
NUDT16	hsa-miR-7043-5p
NUDT16	hsa-miR-221-5p
NUDT16	hsa-miR-922
NUDT16	hsa-miR-200c-3p
NUDT16	hsa-miR-4530
NUDT16	hsa-miR-411-3p
NUDT16	hsa-miR-4461
NUDT16	hsa-miR-6842-5p
NUDT16	hsa-miR-4421
NUDT16	hsa-miR-518-5p
NUDT16	hsa-miR-93-3p
NUDT16	hsa-miR-664
NUDT16	hsa-miR-2285aa
NUDT16	hsa-miR-548t-5p
NUDT16	hsa-miR-7011-3p
NUDT16	hsa-miR-7173-5p
NUDT16	hsa-miR-6813-5p

NUDT16	hsa-miR-6756-3p
NUDT16	hsa-miR-1295b-5p
NUDT16	hsa-miR-665-3p
NUDT16	hsa-miR-7187-3p
NUDT16	hsa-miR-3059-5p
NUDT16	hsa-miR-4430
NUDT16	hsa-miR-593-3p
NUDT16	hsa-miR-1587
NUDT16	hsa-miR-150-5p
NUDT16	hsa-miR-29a-3p
NUDT16	hsa-miR-7081-3p
NUDT16	hsa-miR-490-5p
NUDT16	hsa-miR-3934-3p
NUDT16	hsa-miR-3058-5p
NUDT16	hsa-miR-3685
NUDT16	hsa-miR-664b-5p
NUDT16	hsa-miR-4524-5p
NUDT16	hsa-miR-6363
NUDT16	hsa-miR-762
NUDT16	hsa-miR-7156-3p
NUDT16	hsa-miR-3085-3p
NUDT16	hsa-miR-466n-5p
NUDT16	hsa-miR-7179-3p
NUDT16	hsa-miR-7975
NUDT16	hsa-miR-5699-3p
NUDT16	hsa-miR-1266
NUDT16	hsa-miR-6996-3p
NUDT16	hsa-miR-3162-5p
NUDT16	hsa-miR-27-3p
NUDT16	hsa-miR-8113
NUDT16	hsa-miR-6762-5p
NUDT16	hsa-miR-6379
NUDT16	hsa-miR-590-5p
NUDT16	hsa-miR-6821-3p
NUDT16	hsa-miR-8841
NUDT16	hsa-miR-3162-3p
NUDT16	hsa-miR-548s
NUDT16	hsa-miR-5100
NUDT16	hsa-miR-3967
NUDT16	hsa-miR-373-3p
NUDT16	hsa-miR-181b-1-3p
NUDT16	hsa-miR-199-5p
NUDT16	hsa-miR-3475-3p
NUDT16	hsa-miR-7053-3p
NUDT16	hsa-miR-450b-3p
NUDT16	hsa-miR-709

NUDT16	hsa-miR-2392
NUDT16	hsa-miR-7074-3p
NUDT16	hsa-miR-5571-3p
NUDT16	hsa-miR-6894-3p
NUDT16	hsa-miR-1911-3p
NUDT16	hsa-miR-103-5p
NUDT16	hsa-miR-1180-5p
NUDT16	hsa-miR-494-5p
NUDT16	hsa-miR-6759-5p
NUDT16	hsa-miR-6811-3p
NUDT16	hsa-miR-4277
NUDT16	hsa-miR-7162-3p
NUDT16	hsa-miR-4509
NUDT16	hsa-miR-3593-5p
NUDT16	hsa-miR-6736-3p
NUDT16	hsa-miR-28-5p
NUDT16	hsa-miR-3155a
NUDT16	hsa-miR-3912-5p
NUDT16	hsa-miR-4695-3p
NUDT16	hsa-miR-3167-3p
NUDT16	hsa-miR-3188
NUDT16	hsa-miR-32-3p
NUDT16	hsa-miR-509-5p
NUDT16	hsa-miR-1205
NUDT16	hsa-miR-613
NUDT16	hsa-miR-7661-5p
NUDT16	hsa-miR-875-3p
NUDT16	hsa-miR-6833-5p
NUDT16	hsa-miR-524-5p
NUDT16	hsa-miR-3091-5p
NUDT16	hsa-miR-642
NUDT16	hsa-miR-4720-5p
NUDT16	hsa-miR-2331-3p
NUDT16	hsa-miR-5683
NUDT16	hsa-miR-4328
NUDT16	hsa-miR-4288
NUDT16	hsa-miR-8875
NUDT16	hsa-miR-194-3p
NUDT16	hsa-miR-4491
NUDT16	hsa-miR-3613-5p
NUDT16	hsa-miR-193b-5p
NUDT16	hsa-miR-7691
NUDT16	hsa-miR-344f-3p
NUDT16	hsa-miR-499-5p
NUDT16	hsa-miR-6972-5p
NUDT16	hsa-miR-489-5p

NUDT16	hsa-miR-4745-5p
NUDT16	hsa-miR-3182
NUDT16	hsa-miR-200a-3p
NUDT16	hsa-miR-7077-5p
NUDT16	hsa-miR-4323
NUDT16	hsa-miR-4755-5p
NUDT16	hsa-let-7a-2-3p
NUDT16	hsa-miR-122-5p
NUDT16	hsa-miR-149-5p
NUDT16	hsa-let-7c-3p
NUDT16	hsa-miR-195-5p
NUDT16	hsa-miR-5615-3p
NUDT16	hsa-miR-6752-3p
NUDT16	hsa-miR-4687-5p
NUDT16	hsa-miR-557
NUDT16	hsa-miR-654-3p
NUDT16	hsa-miR-6747-3p
NUDT16	hsa-miR-3202
NUDT16	hsa-miR-4758-3p
NUDT16	hsa-miR-7043-3p
NUDT16	hsa-miR-7169-3p
NUDT16	hsa-miR-4255
NUDT16	hsa-miR-301b-5p
NUDT16	hsa-miR-143-3p
NUDT16	hsa-miR-6734-3p
NUDT16	hsa-miR-2052
NUDT16	hsa-miR-3572-3p
NUDT16	hsa-miR-7183-5p
NUDT16	hsa-miR-7051-3p
NUDT16	hsa-miR-483-3p
NUDT16	hsa-miR-1827
NUDT16	hsa-miR-7641
NUDT16	hsa-miR-3075
NUDT16	hsa-miR-940
NUDT16	hsa-miR-3616-5p
NUDT16	hsa-miR-675-3p
NUDT16	hsa-miR-208-3p
NUDT16	hsa-miR-4437
NUDT16	hsa-miR-6783-5p
NUDT16	hsa-miR-6751-3p
NUDT16	hsa-miR-487a-3p
NUDT16	hsa-miR-4447
NUDT16	hsa-miR-3620-5p
NUDT16	hsa-miR-154-5p
NUDT16	hsa-miR-2355-3p
NUDT16	hsa-miR-371

NUDT16	hsa-miR-7687-3p
NUDT16	hsa-miR-7069-3p
NUDT16	hsa-miR-329-3p
NUDT16	hsa-miR-5088-3p
NUDT16	hsa-miR-593-3p
NUDT16	hsa-miR-702-3p
NUDT16	hsa-miR-7652-5p
NUDT16	hsa-miR-6825-5p
NUDT16	hsa-miR-6903-3p
NUDT16	hsa-miR-1914-5p
NUDT16	hsa-miR-516b
NUDT16	hsa-miR-6546-5p
NUDT16	hsa-miR-5107-5p
NUDT16	hsa-miR-6772-3p
NUDT16	hsa-miR-3557-5p
NUDT16	hsa-miR-3473be
NUDT16	hsa-miR-7160-5p
NUDT16	hsa-miR-466q
NUDT16	hsa-miR-6769b-5p
NUDT16	hsa-miR-337-3p
NUDT16	hsa-miR-4675
NUDT16	hsa-miR-4257
NUDT16	hsa-miR-182-3p
NUDT16	hsa-miR-218-1-3p
NUDT16	hsa-miR-512-3p
NUDT16	hsa-miR-6773-5p
NUDT16	hsa-miR-4438
NUDT16	hsa-miR-3140-5p
NUDT16	hsa-miR-4273
NUDT16	hsa-miR-377-3p
NUDT16	hsa-miR-1343
NUDT16	hsa-miR-374b-3p
NUDT16	hsa-miR-802-5p
NUDT16	hsa-miR-5621-5p
NUDT16	hsa-miR-3916
NUDT16	hsa-miR-1225
NUDT16	hsa-miR-1301-5p
NUDT16	hsa-miR-6809-3p
NUDT16	hsa-miR-3678-3p
NUDT16	hsa-miR-7242-5p
NUDT16	hsa-miR-24-3p
NUDT16	hsa-miR-7048-3p
NUDT16	hsa-miR-3971
NUDT16	hsa-miR-1249-3p
NUDT16	hsa-miR-584-5p
NUDT16	hsa-miR-8881

NUDT16	hsa-miR-3084-3p
NUDT16	hsa-miR-6778-5p
NUDT16	hsa-miR-140-3p
NUDT16	hsa-miR-5108
NUDT16	hsa-miR-921
NUDT16	hsa-miR-302c-5p
NUDT16	hsa-miR-4638-3p
NUDT16	hsa-miR-6968-5p
NUDT16	hsa-miR-7172-5p
NUDT16	hsa-miR-6515-5p
NUDT16	hsa-miR-5682
NUDT16	hsa-miR-4642
NUDT16	hsa-miR-4646-3p
NUDT16	hsa-miR-148-5p
NUDT16	hsa-miR-767-5p
NUDT16	hsa-miR-203b-5p
NUDT16	hsa-miR-4774-3p
NUDT16	hsa-miR-548aef
NUDT16	hsa-miR-6936-5p
NUDT16	hsa-miR-4329
NUDT16	hsa-miR-6920-3p
NUDT16	hsa-miR-1927
NUDT16	hsa-miR-8823
NUDT16	hsa-miR-3922-5p
NUDT16	hsa-miR-3546
NUDT16	hsa-miR-619-5p
NUDT16	hsa-miR-4666b
NUDT16	hsa-miR-1248
NUDT16	hsa-miR-138-2-3p
NUDT16	hsa-miR-3083-5p
NUDT16	hsa-miR-6537-3p
NUDT16	hsa-miR-550-5p
NUDT16	hsa-miR-6762-3p
NUDT16	hsa-miR-6827-5p
NUDT16	hsa-miR-221-3p
NUDT16	hsa-miR-8802
NUDT16	hsa-miR-6909-3p
NUDT16	hsa-miR-3130-3p
NUDT16	hsa-miR-6165
NUDT16	hsa-miR-452-3p
NUDT16	hsa-miR-373-5p
NUDT16	hsa-miR-654-5p
NUDT16	hsa-miR-4782-5p
NUDT16	hsa-miR-455-5p
NUDT16	hsa-miR-4659a-5p
NUDT16	hsa-miR-4772-5p

NUDT16	hsa-miR-516-3p
NUDT16	hsa-miR-6973b-3p
NUDT16	hsa-miR-2464-5p
NUDT16	hsa-miR-544-5p
NUDT16	hsa-miR-3179
NUDT16	hsa-miR-7028-5p
NUDT16	hsa-miR-6917-3p
NUDT16	hsa-miR-1304-3p
NUDT16	hsa-miR-4667
NUDT16	hsa-miR-4713-5p
NUDT16	hsa-miR-7705
NUDT16	hsa-miR-30c-3p
NUDT16	hsa-miR-202-3p
NUDT16	hsa-miR-4496
NUDT16	hsa-miR-7228-3p
NUDT16	hsa-miR-3150a-3p
NUDT16	hsa-miR-5009-3p
NUDT16	hsa-miR-7039-5p
NUDT16	hsa-miR-7188-3p
NUDT16	hsa-miR-6531
NUDT16	hsa-miR-6400
NUDT16	hsa-miR-548e-5p
NUDT16	hsa-miR-7181-3p
NUDT16	hsa-miR-1323-3p
NUDT16	hsa-miR-539-5p
NUDT16	hsa-miR-184
NUDT16	hsa-miR-8862
NUDT16	hsa-miR-6948-3p
NUDT16	hsa-miR-591
NUDT16	hsa-miR-871-3p
NUDT16	hsa-miR-513b-5p
NUDT16	hsa-miR-300
NUDT16	hsa-miR-7192-5p
NUDT16	hsa-miR-373-3p
NUDT16	hsa-miR-662
NUDT16	hsa-miR-3907
NUDT16	hsa-miR-7022-5p
NUDT16	hsa-miR-622
NUDT16	hsa-miR-7028-3p
NUDT16	hsa-miR-8810
NUDT16	hsa-miR-3586-3p
NUDT16	hsa-miR-8877
NUDT16	hsa-miR-509-3p
NUDT16	hsa-miR-3117-3p
NUDT16	hsa-miR-3167-5p
NUDT16	hsa-miR-200ab-5p

NUDT16	hsa-miR-331-3p
NUDT16	hsa-miR-7158-5p
NUDT16	hsa-miR-7063-3p
NUDT16	hsa-miR-1249-3p
NUDT16	hsa-miR-4324
NUDT16	hsa-miR-6861-5p
NUDT16	hsa-miR-7239-5p
NUDT16	hsa-miR-642b-5p
NUDT16	hsa-miR-628-3p
NUDT16	hsa-miR-6505-5p
NUDT16	hsa-miR-3093-5p
NUDT16	hsa-let-7
NUDT16	hsa-miR-6911-3p
NUDT16	hsa-miR-7151-3p
NUDT16	hsa-miR-4728-5p
NUDT16	hsa-miR-6810-3p
NUDT16	hsa-miR-6516-3p
NUDT16	hsa-miR-4651
NUDT16	hsa-miR-3185
NUDT16	hsa-miR-4433b-3p
NUDT16	hsa-miR-7667-5p
NUDT16	hsa-miR-6506-5p
NUDT16	hsa-miR-7219-5p
NUDT16	hsa-miR-4735-5p
NUDT16	hsa-miR-4688
NUDT16	hsa-miR-7222-5p
NUDT16	hsa-miR-200-3p
NUDT16	hsa-miR-195-5p
NUDT16	hsa-miR-6354
NUDT16	hsa-miR-153
NUDT16	hsa-miR-497-5p
NUDT16	hsa-miR-1283a
NUDT16	hsa-miR-344c-3p
NUDT16	hsa-miR-6726-5p
NUDT16	hsa-miR-6980-5p
NUDT16	hsa-miR-516b-5p
NUDT16	hsa-miR-548q
NUDT16	hsa-miR-19-3p
NUDT16	hsa-miR-6080
NUDT16	hsa-miR-6868-5p
NUDT16	hsa-miR-1229-3p
NUDT16	hsa-miR-4685-3p
NUDT16	hsa-miR-6918-5p
NUDT16	hsa-miR-4305
NUDT16	hsa-miR-3128
NUDT16	hsa-miR-300-3p

NUDT16	hsa-miR-203
NUDT16	hsa-miR-939-5p
NUDT16	hsa-miR-6947-3p
NUDT16	hsa-miR-379-5p
NUDT16	hsa-miR-8092
NUDT16	hsa-miR-212-3p
NUDT16	hsa-miR-4264
NUDT16	hsa-miR-577-3p
NUDT16	hsa-miR-4660
NUDT16	hsa-miR-324-5p
NUDT16	hsa-miR-292b-3p
NUDT16	hsa-miR-4733-5p
NUDT16	hsa-miR-7119-5p
NUDT16	hsa-miR-4469
NUDT16	hsa-miR-2284-5p
NUDT16	hsa-miR-8867
NUDT16	hsa-miR-4749-3p
NUDT16	hsa-miR-9500
NUDT16	hsa-miR-3136-5p
NUDT16	hsa-miR-769-5p
NUDT16	hsa-miR-3714
NUDT16	hsa-miR-6508-3p
NUDT16	hsa-miR-217-3p
NUDT16	hsa-miR-346
NUDT16	hsa-miR-210-5p
NUDT16	hsa-miR-648
NUDT16	hsa-miR-3074-2-3p
NUDT16	hsa-miR-154a
NUDT16	hsa-miR-520-3p
NUDT16	hsa-miR-1237-3p
NUDT16	hsa-miR-3972
NUDT16	hsa-miR-763
NUDT16	hsa-miR-673-5p
NUDT16	hsa-miR-8794
NUDT16	hsa-miR-7162-5p
NUDT16	hsa-miR-500-5p
NUDT16	hsa-miR-7856-5p
NUDT16	hsa-miR-8485
NUDT16	hsa-miR-448-5p
NUDT16	hsa-miR-26a-3p
NUDT16	hsa-miR-3151
NUDT16	hsa-miR-6780a-5p
NUDT16	hsa-let-7ag-3p
NUDT16	hsa-miR-892a
NUDT16	hsa-miR-3622b-5p
NUDT16	hsa-miR-7155-5p

NUDT16	hsa-miR-18a-3p
NUDT16	hsa-miR-328-5p
NUDT16	hsa-miR-330-5p
NUDT16	hsa-miR-6788-5p
NUDT16	hsa-miR-371b-5p
NUDT16	hsa-miR-376a-1-5p
NUDT16	hsa-miR-19a-5p
NUDT16	hsa-miR-1227-3p
NUDT16	hsa-miR-6715b-3p
NUDT16	hsa-miR-2114-3p
NUDT16	hsa-miR-5699-5p
NUDT16	hsa-miR-6908-3p
NUDT16	hsa-miR-7053-5p
NUDT16	hsa-miR-3143
NUDT16	hsa-miR-5046
NUDT16	hsa-miR-125
NUDT16	hsa-miR-423-5p
NUDT16	hsa-miR-1296-3p
NUDT16	hsa-miR-5581-5p
NUDT16	hsa-miR-34a-3p
NUDT16	hsa-miR-1841
NUDT16	hsa-miR-1262-5p
NUDT16	hsa-miR-8891
NUDT16	hsa-miR-16-5p
NUDT16	hsa-miR-766-5p
NUDT16	hsa-miR-875
NUDT16	hsa-miR-322-3p
NUDT16	hsa-miR-6769b-3p
NUDT16	hsa-miR-3163
NUDT16	hsa-miR-6072
NUDT16	hsa-miR-496
NUDT16	hsa-miR-106a-3p
NUDT16	hsa-miR-4664-3p
NUDT16	hsa-miR-663b
NUDT16	hsa-miR-4696
NUDT16	hsa-miR-619-3p
NUDT16	hsa-miR-548
NUDT16	hsa-miR-5691
NUDT16	hsa-miR-3200-3p
NUDT16	hsa-miR-4419a
NUDT16	hsa-miR-6808-5p
NUDT16	hsa-miR-3075-5p
NUDT16	hsa-miR-572
NUDT16	hsa-miR-548g-5p
NUDT16	hsa-miR-22-3p
NUDT16	hsa-miR-344-3p

NUDT16	hsa-miR-7207-3p
NUDT16	hsa-miR-4769-5p
NUDT16	hsa-miR-6812-5p
NUDT16	hsa-miR-3150b-3p
NUDT16	hsa-miR-2426
NUDT16	hsa-miR-3091-3p
NUDT16	hsa-miR-6858-3p
NUDT16	hsa-miR-1182
NUDT16	hsa-miR-101-3p.1
NUDT16	hsa-miR-4667-3p
NUDT16	hsa-miR-1298-5p
NUDT16	hsa-miR-374
NUDT16	hsa-miR-8848
NUDT16	hsa-miR-567
NUDT16	hsa-miR-5571-5p
NUDT16	hsa-miR-224-5p
NUDT16	hsa-miR-3927-3p
NUDT16	hsa-miR-6785-5p
NUDT16	hsa-miR-1912-5p
NUDT16	hsa-miR-4420
NUDT16	hsa-miR-3572-5p
NUDT16	hsa-miR-4282
NUDT16	hsa-miR-6747-5p
NUDT16	hsa-miR-377-5p
NUDT16	hsa-miR-6867-5p
NUDT16	hsa-miR-5121
NUDT16	hsa-miR-322-5p
NUDT16	hsa-miR-6841-5p
NUDT16	hsa-miR-214-5p
NUDT16	hsa-miR-450b
NUDT16	hsa-miR-185-5p
NUDT16	hsa-miR-6338
NUDT16	hsa-miR-7023-3p
NUDT16	hsa-miR-23
NUDT16	hsa-miR-31-5p
NUDT16	hsa-miR-7150
NUDT16	hsa-miR-4736
NUDT16	hsa-miR-7658-3p
NUDT16	hsa-miR-193-5p
NUDT16	hsa-miR-7056-3p
NUDT16	hsa-let-7abf-3p
NUDT16	hsa-miR-3159
NUDT16	hsa-miR-711
NUDT16	hsa-miR-669i
NUDT16	hsa-miR-4641
NUDT16	hsa-miR-344d-2-5p

NUDT16	hsa-miR-6995-5p
NUDT16	hsa-miR-671-5p
NUDT16	hsa-miR-1255b-2-3p
NUDT16	hsa-miR-877-5p
NUDT16	hsa-miR-4763-5p
NUDT16	hsa-miR-4761-5p
NUDT16	hsa-miR-7082-3p
NUDT16	hsa-miR-378
NUDT16	hsa-miR-616-5p
NUDT16	hsa-miR-642a-5p
NUDT16	hsa-miR-6753-5p
NUDT16	hsa-miR-7026-3p
NUDT16	hsa-miR-196c-3p
NUDT16	hsa-miR-548av-3p
NUDT16	hsa-miR-676-3p
NUDT16	hsa-miR-7210-5p
NUDT16	hsa-miR-6770-5p
NUDT16	hsa-miR-3127-5p
NUDT16	hsa-miR-142-3p.1
NUDT16	hsa-miR-375
NUDT16	hsa-miR-8061
NUDT16	hsa-miR-1190
NUDT16	hsa-miR-7077-3p
NUDT16	hsa-miR-7238-5p
NUDT16	hsa-miR-7225-5p
NUDT16	hsa-miR-2909
NUDT16	hsa-miR-324
NUDT16	hsa-miR-4428
NUDT16	hsa-miR-4690-3p
NUDT16	hsa-miR-350-3p
NUDT16	hsa-miR-592
NUDT16	hsa-miR-2410
NUDT16	hsa-miR-466dk-5p
NUDT16	hsa-miR-9-5p
NUDT16	hsa-miR-2465
NUDT16	hsa-miR-539-3p
NUDT16	hsa-miR-148-3p
NUDT16	hsa-miR-130a-5p
NUDT16	hsa-miR-6865-3p
NUDT16	hsa-miR-637
NUDT16	hsa-miR-380-5p
NUDT16	hsa-miR-212-5p
NUDT16	hsa-miR-4717-3p
NUDT16	hsa-miR-660-5p
NUDT16	hsa-miR-541-3p
NUDT16	hsa-miR-6376

NUDT16	hsa-miR-362-3p
NUDT16	hsa-miR-669bf-3p
NUDT16	hsa-miR-632
NUDT16	hsa-miR-760-3p
NUDT16	hsa-miR-134
NUDT16	hsa-miR-5586-3p
NUDT16	hsa-miR-1915-5p
NUDT16	hsa-miR-770
NUDT16	hsa-miR-1247-3p
NUDT16	hsa-miR-764-3p
NUDT16	hsa-miR-7847-3p
NUDT16	hsa-miR-10-5p
NUDT16	hsa-miR-466f-3p
NUDT16	hsa-miR-603
NUDT16	hsa-miR-3120-5p
NUDT16	hsa-miR-382-3p
NUDT16	hsa-miR-883ab-3p
NUDT16	hsa-miR-5004-3p
NUDT16	hsa-miR-7978
NUDT16	hsa-miR-342-3p
NUDT16	hsa-miR-7061-5p
NUDT16	hsa-miR-7195-3p
NUDT16	hsa-miR-345-3p
NUDT16	hsa-miR-502a
NUDT16	hsa-miR-92a-1-5p
NUDT16	hsa-miR-6864-5p
NUDT16	hsa-miR-8073
NUDT16	hsa-miR-6520
NUDT16	hsa-miR-7023-5p
NUDT16	hsa-miR-292a-5p
NUDT16	hsa-miR-4796-5p
NUDT16	hsa-miR-4533
NUDT16	hsa-miR-7-5p
NUDT16	hsa-miR-3144-3p
NUDT16	hsa-miR-1933-3p
NUDT16	hsa-miR-576-5p
NUDT16	hsa-miR-4524a-5p
NUDT16	hsa-miR-6883-3p
NUDT16	hsa-miR-3059-3p
NUDT16	hsa-miR-197-3p
NUDT16	hsa-miR-4488
NUDT16	hsa-miR-765
NUDT16	hsa-miR-7110-3p
NUDT16	hsa-miR-451b
NUDT16	hsa-miR-8808
NUDT16	hsa-miR-759

NUDT16	hsa-miR-7215-5p
NUDT16	hsa-miR-365b-5p
NUDT16	hsa-miR-7238-3p
NUDT16	hsa-miR-7237-3p
NUDT16	hsa-miR-3092-5p
NUDT16	hsa-miR-7154-3p
NUDT16	hsa-miR-520gh
NUDT16	hsa-miR-1273f
NUDT16	hsa-miR-298-3p
NUDT16	hsa-miR-6984-3p
NUDT16	hsa-miR-8057
NUDT16	hsa-miR-222-3p
NUDT16	hsa-miR-3649
NUDT16	hsa-miR-5000-3p
NUDT16	hsa-miR-4505
NUDT16	hsa-miR-8093
NUDT16	hsa-miR-449c-5p
NUDT16	hsa-miR-6961-5p
NUDT16	hsa-miR-383
NUDT16	hsa-miR-3927-3p
NUDT16	hsa-miR-878
NUDT16	hsa-miR-483-3p.2
NUDT16	hsa-miR-429-3p
NUDT16	hsa-miR-29a-5p
NUDT16	hsa-miR-3592
NUDT16	hsa-miR-6085
NUDT16	hsa-miR-351-5p
NUDT16	hsa-miR-4793-5p
NUDT16	hsa-miR-339-5p
NUDT16	hsa-miR-3074-3p
NUDT16	hsa-miR-6919-5p
NUDT16	hsa-miR-6796-5p
NUDT16	hsa-miR-4710
NUDT16	hsa-miR-1903
NUDT16	hsa-miR-7848-3p
NUDT16	hsa-miR-1227-3p
NUDT16	hsa-miR-4512
NUDT16	hsa-miR-3125
NUDT16	hsa-miR-6743-5p
NUDT16	hsa-miR-7176-3p
NUDT16	hsa-miR-6965-3p
NUDT16	hsa-miR-4443
NUDT16	hsa-miR-7073-3p
NUDT16	hsa-miR-5627-3p
NUDT16	hsa-miR-3620-3p
NUDT16	hsa-miR-450b-5p

NUDT16	hsa-miR-3072
NUDT16	hsa-miR-6844
NUDT16	hsa-miR-4459
NUDT16	hsa-miR-6324
NUDT16	hsa-miR-3158-3p
NUDT16	hsa-miR-6414
NUDT16	hsa-miR-513a-3p
NUDT16	hsa-miR-374a-3p
NUDT16	hsa-miR-8906
NUDT16	hsa-miR-92a-2-5p
NUDT16	hsa-miR-26b-3p
NUDT16	hsa-miR-6781-3p
NUDT16	hsa-miR-7171-5p
NUDT16	hsa-miR-292-5p
NUDT16	hsa-miR-6878-5p
NUDT16	hsa-miR-4501
NUDT16	hsa-miR-192-5p
NUDT16	hsa-miR-6804-5p
NUDT16	hsa-miR-615-3p
NUDT16	hsa-miR-6840-3p
NUDT16	hsa-miR-1236-5p
NUDT16	hsa-miR-3161
NUDT16	hsa-miR-503-3p
NUDT16	hsa-miR-4656
NUDT16	hsa-miR-7006-3p
NUDT16	hsa-miR-8849
NUDT16	hsa-miR-6894-5p
NUDT16	hsa-miR-6799-5p
NUDT16	hsa-miR-517-5p
NUDT16	hsa-miR-490-3p
NUDT16	hsa-miR-7069-5p
NUDT16	hsa-miR-3193
NUDT16	hsa-miR-1264-3p
NUDT16	hsa-miR-543-3p
NUDT16	hsa-miR-6862-3p
NUDT16	hsa-miR-5588-5p
NUDT16	hsa-miR-7851-3p
NUDT16	hsa-miR-10b-3p
NUDT16	hsa-miR-608
NUDT16	hsa-miR-4657
NUDT16	hsa-miR-4999-5p
NUDT16	hsa-miR-683
NUDT16	hsa-miR-1233-5p
NUDT16	hsa-miR-885
NUDT16	hsa-miR-6749-3p
NUDT16	hsa-miR-487a

NUDT16	hsa-miR-4283
NUDT16	hsa-miR-6719-3p
NUDT16	hsa-miR-3660
NUDT16	hsa-miR-7117-3p
NUDT16	hsa-miR-7004-3p
NUDT16	hsa-miR-207
NUDT16	hsa-miR-7109-3p
NUDT16	hsa-miR-1910-3p
NUDT16	hsa-miR-6868-3p
NUDT16	hsa-miR-6503-3p
NUDT16	hsa-miR-1950
NUDT16	hsa-miR-579-3p
NUDT16	hsa-miR-190a-3p
NUDT16	hsa-miR-1200
NUDT16	hsa-miR-6801-5p
NUDT16	hsa-miR-3086-5p
NUDT16	hsa-miR-8844
NUDT16	hsa-miR-4768-5p
NUDT16	hsa-miR-4797-5p
NUDT16	hsa-miR-338-3p
NUDT16	hsa-miR-429
NUDT16	hsa-miR-4691-5p
NUDT16	hsa-miR-365a-5p
NUDT16	hsa-miR-5193
NUDT16	hsa-miR-6780b-5p
NUDT16	hsa-miR-7215-3p
NUDT16	hsa-miR-3667-5p
NUDT16	hsa-miR-1251-3p
NUDT16	hsa-miR-23a-5p
NUDT16	hsa-miR-6891-3p
NUDT16	hsa-miR-150-3p
NUDT16	hsa-miR-942
NUDT16	hsa-miR-7192-3p
NUDT16	hsa-miR-871-5p
NUDT16	hsa-miR-5580-3p
NUDT16	hsa-miR-463-5p
NUDT16	hsa-miR-1842
NUDT16	hsa-miR-370-3p
NUDT16	hsa-miR-6356
NUDT16	hsa-miR-6372
NUDT16	hsa-miR-4480
NUDT16	hsa-miR-667-5p
NUDT16	hsa-miR-3069-3p
NUDT16	hsa-miR-6511-3p
NUDT16	hsa-miR-760-5p
NUDT16	hsa-miR-30e-3p

NUDT16	hsa-miR-7195-5p
NUDT16	hsa-miR-6071
NUDT16	hsa-miR-7038-3p
NUDT16	hsa-miR-6740-3p
NUDT16	hsa-miR-6990-5p
NUDT16	hsa-miR-4722-3p
NUDT16	hsa-miR-6393
NUDT16	hsa-miR-6753-3p
NUDT16	hsa-miR-7045-3p
NUDT16	hsa-miR-208-5p
NUDT16	hsa-miR-4446-3p
NUDT16	hsa-miR-488-3p
NUDT16	hsa-miR-3918
NUDT16	hsa-miR-350
NUDT16	hsa-miR-4728-3p
NUDT16	hsa-miR-190a-5p
NUDT16	hsa-miR-6795-3p
NUDT16	hsa-miR-6765-3p
NUDT16	hsa-miR-4697-3p
NUDT16	hsa-miR-670-5p
NUDT16	hsa-miR-568
NUDT16	hsa-miR-7684-5p
NUDT16	hsa-miR-7093-3p
NUDT16	hsa-miR-455-5p
NUDT16	hsa-miR-873a-5p.2
NUDT16	hsa-miR-1388-3p
NUDT16	hsa-miR-7-5p
NUDT16	hsa-miR-6938-5p
NUDT16	hsa-miR-138-5p
NUDT16	hsa-miR-20b-3p
NUDT16	hsa-miR-550-3p
NUDT16	hsa-miR-6958-3p
NUDT16	hsa-miR-133a-3p.2
NUDT16	hsa-miR-6819-5p
NUDT16	hsa-miR-3120-3p
NUDT16	hsa-miR-434-3p
NUDT16	hsa-miR-3175
NUDT16	hsa-miR-3934-5p
NUDT16	hsa-miR-7009-5p
NUDT16	hsa-miR-6779-5p
NUDT16	hsa-miR-215
NUDT16	hsa-miR-627-3p
NUDT16	hsa-miR-4683
NUDT16	hsa-miR-467f
NUDT16	hsa-miR-302c-3p
NUDT16	hsa-miR-7243-3p

NUDT16	hsa-miR-378-5p
NUDT16	hsa-miR-4432
NUDT16	hsa-miR-2682-3p
NUDT16	hsa-miR-574-5p
NUDT16	hsa-miR-6822-5p
NUDT16	hsa-miR-6731-5p
NUDT16	hsa-miR-133
NUDT16	hsa-miR-4666a-5p
NUDT16	hsa-miR-501-5p
NUDT16	hsa-miR-6891-5p
NUDT16	hsa-miR-6892-3p
NUDT16	hsa-miR-4725-5p
NUDT16	hsa-miR-299
NUDT16	hsa-miR-342-5p
NUDT16	hsa-miR-3075-3p
NUDT16	hsa-miR-548v
NUDT16	hsa-miR-466n-3p
NUDT16	hsa-miR-519-5p
NUDT16	hsa-miR-686
NUDT16	hsa-miR-540-3p
NUDT16	hsa-miR-7151-5p
NUDT16	hsa-miR-519-3p
NUDT16	hsa-miR-3655
NUDT16	hsa-miR-6800-3p
NUDT16	hsa-miR-8817
NUDT16	hsa-miR-7086-5p
NUDT16	hsa-miR-4287
NUDT16	hsa-miR-7193-3p
NUDT16	hsa-miR-290a-5p
NUDT16	hsa-miR-942-5p
NUDT16	hsa-miR-1202
NUDT16	hsa-miR-502-5p
NUDT16	hsa-miR-1843b-5p
NUDT16	hsa-miR-4450
NUDT16	hsa-miR-4711-5p
NUDT16	hsa-miR-1260a
NUDT16	hsa-miR-1912-3p
NUDT16	hsa-miR-4654
NUDT16	hsa-miR-548k
NUDT16	hsa-miR-1233-3p
NUDT16	hsa-miR-3591-5p
NUDT16	hsa-miR-5701
NUDT16	hsa-miR-8874
NUDT16	hsa-miR-424-5p
NUDT16	hsa-miR-523-3p
NUDT16	hsa-let-7-5p

NUDT16	hsa-miR-525-5p
NUDT16	hsa-miR-7012-5p
NUDT16	hsa-miR-100-3p
NUDT16	hsa-miR-3619-5p
NUDT16	hsa-miR-4516
NUDT16	hsa-miR-550a-3-5p
NUDT16	hsa-miR-5009-5p
NUDT16	hsa-miR-4741
NUDT3	hsa-miR-548p
NUDT3	hsa-miR-7691
NUDT3	hsa-miR-7068-3p
NUDT3	hsa-miR-302c-3p.2
NUDT3	hsa-miR-2285
NUDT3	hsa-miR-7657-3p
NUDT3	hsa-miR-106-3p
NUDT3	hsa-miR-511-3p
NUDT3	hsa-miR-2284a
NUDT3	hsa-miR-219-5p
NUDT3	hsa-miR-4275
NUDT3	hsa-miR-650
NUDT3	hsa-miR-542
NUDT3	hsa-miR-3154
NUDT3	hsa-miR-2406
NUDT3	hsa-miR-2054
NUDT3	hsa-miR-3144-3p
NUDT3	hsa-miR-6828-5p
NUDT3	hsa-miR-548d-5p
NUDT3	hsa-miR-34c-3p
NUDT3	hsa-miR-8799
NUDT3	hsa-miR-148b-5p
NUDT3	hsa-miR-2052
NUDT3	hsa-miR-4699-5p
NUDT3	hsa-miR-8837
NUDT3	hsa-miR-92b-5p
NUDT3	hsa-miR-1587
NUDT3	hsa-miR-6719-3p
NUDT3	hsa-miR-8905
NUDT3	hsa-miR-548g-3p
NUDT3	hsa-miR-7386f-3p
NUDT3	hsa-miR-7480-5p
NUDT3	hsa-miR-7078-3p
NUDT3	hsa-miR-526
NUDT3	hsa-miR-878
NUDT3	hsa-miR-3137
NUDT3	hsa-miR-7278-5p
NUDT3	hsa-miR-1717

NUDT3	hsa-miR-33b-3p
NUDT3	hsa-miR-3078-5p
NUDT3	hsa-miR-4760-5p
NUDT3	hsa-miR-2431-3p
NUDT3	hsa-miR-512
NUDT3	hsa-miR-1264
NUDT3	hsa-miR-548as-3p
NUDT3	hsa-miR-6860
NUDT3	hsa-miR-384-3p
NUDT3	hsa-miR-891-3p
NUDT3	hsa-miR-367-5p
NUDT3	hsa-miR-2399-5p
NUDT3	hsa-miR-6562-5p
NUDT3	hsa-let-7j-3p
NUDT3	hsa-miR-7251-3p
NUDT3	hsa-miR-6400
NUDT3	hsa-miR-382-5p
NUDT3	hsa-miR-889-3p
NUDT3	hsa-miR-7187-3p
NUDT3	hsa-miR-20-3p
NUDT3	hsa-miR-7379-3p
NUDT3	hsa-miR-539-3p
NUDT3	hsa-miR-6870-5p
NUDT3	hsa-miR-7156-3p
NUDT3	hsa-miR-542-5p
NUDT3	hsa-miR-5009-3p
NUDT3	hsa-miR-2115-3p
NUDT3	hsa-miR-154-5p
NUDT3	hsa-miR-1229-5p
NUDT3	hsa-miR-1263
NUDT3	hsa-miR-6825-5p
NUDT3	hsa-miR-322-3p
NUDT3	hsa-miR-8119
NUDT3	hsa-miR-199-5p
NUDT3	hsa-miR-6981-3p
NUDT3	hsa-miR-515-3p
NUDT3	hsa-miR-7000-5p
NUDT3	hsa-miR-7334-5p
NUDT3	hsa-miR-30c-2-3p
NUDT3	hsa-miR-3150a-3p
NUDT3	hsa-miR-1227-3p
NUDT3	hsa-miR-4664-5p
NUDT3	hsa-miR-2317
NUDT3	hsa-miR-6351
NUDT3	hsa-miR-2370-5p
NUDT3	hsa-miR-1964-5p

NUDT3	hsa-miR-219b
NUDT3	hsa-miR-411-5p.1
NUDT3	hsa-miR-1-1-5p
NUDT3	hsa-miR-1287
NUDT3	hsa-miR-105-5p
NUDT3	hsa-miR-517
NUDT3	hsa-miR-3713
NUDT3	hsa-miR-6808-5p
NUDT3	hsa-miR-6791-5p
NUDT3	hsa-miR-8067
NUDT3	hsa-miR-203a-3p.1
NUDT3	hsa-miR-7389-3p
NUDT3	hsa-miR-2377
NUDT3	hsa-miR-508b
NUDT3	hsa-miR-221-5p
NUDT3	hsa-miR-2284y
NUDT3	hsa-miR-628-5p
NUDT3	hsa-miR-344f-3p
NUDT3	hsa-miR-3173-3p
NUDT3	hsa-miR-6922-3p
NUDT3	hsa-miR-7454-3p
NUDT3	hsa-miR-7371f-5p
NUDT3	hsa-miR-372-5p
NUDT3	hsa-miR-222-3p
NUDT3	hsa-miR-6561-5p
NUDT3	hsa-miR-3076-5p
NUDT3	hsa-miR-7153-3p
NUDT3	hsa-miR-1597-5p
NUDT3	hsa-miR-6318
NUDT3	hsa-miR-3189-3p
NUDT3	hsa-miR-665-5p
NUDT3	hsa-miR-8089
NUDT3	hsa-miR-101-3p
NUDT3	hsa-miR-6914-3p
NUDT3	hsa-miR-3926
NUDT3	hsa-miR-8074
NUDT3	hsa-miR-1789
NUDT3	hsa-miR-6833-3p
NUDT3	hsa-miR-8796
NUDT3	hsa-miR-1306-5p
NUDT3	hsa-miR-323a-5p
NUDT3	hsa-miR-201-3p
NUDT3	hsa-miR-4330
NUDT3	hsa-miR-7681-5p
NUDT3	hsa-miR-130-3p
NUDT3	hsa-miR-4758-5p

NUDT3	hsa-miR-8073
NUDT3	hsa-miR-2296
NUDT3	hsa-miR-582-5p
NUDT3	hsa-miR-142
NUDT3	hsa-miR-2373-5p
NUDT3	hsa-miR-1753
NUDT3	hsa-miR-3174
NUDT3	hsa-miR-6890-3p
NUDT3	hsa-miR-4649-3p
NUDT3	hsa-miR-871-5p
NUDT3	hsa-miR-466
NUDT3	hsa-miR-1291
NUDT3	hsa-miR-3081-5p
NUDT3	hsa-miR-1894-3p
NUDT3	hsa-miR-2354
NUDT3	hsa-miR-149-5p
NUDT3	hsa-miR-7300-3p
NUDT3	hsa-miR-1839
NUDT3	hsa-miR-2472
NUDT3	hsa-miR-6943-3p
NUDT3	hsa-miR-2136
NUDT3	hsa-miR-7197-3p
NUDT3	hsa-miR-4496
NUDT3	hsa-miR-133-3p.1
NUDT3	hsa-miR-449-3p
NUDT3	hsa-miR-548as-5p
NUDT3	hsa-miR-499b-5p
NUDT3	hsa-miR-7299-3p
NUDT3	hsa-miR-4456
NUDT3	hsa-miR-7023-3p
NUDT3	hsa-miR-4505
NUDT3	hsa-miR-7115-3p
NUDT3	hsa-miR-3145-5p
NUDT3	hsa-miR-183-3p
NUDT3	hsa-miR-1843
NUDT3	hsa-miR-21-5p
NUDT3	hsa-miR-6529b
NUDT3	hsa-miR-552-5p
NUDT3	hsa-miR-1254
NUDT3	hsa-miR-4492
NUDT3	hsa-miR-4663
NUDT3	hsa-miR-7113-5p
NUDT3	hsa-miR-6598-5p
NUDT3	hsa-miR-7386h-5p
NUDT3	hsa-miR-132-3p
NUDT3	hsa-miR-95-5p

NUDT3	hsa-miR-1279
NUDT3	hsa-miR-3124-3p
NUDT3	hsa-miR-4452
NUDT3	hsa-miR-679-3p
NUDT3	hsa-miR-96-5p
NUDT3	hsa-miR-382-3p
NUDT3	hsa-miR-7173-3p
NUDT3	hsa-miR-6952-3p
NUDT3	hsa-miR-4691-3p
NUDT3	hsa-miR-26-3-3p
NUDT3	hsa-miR-2467-3p
NUDT3	hsa-miR-92
NUDT3	hsa-miR-5579-3p
NUDT3	hsa-miR-6845-3p
NUDT3	hsa-miR-8810
NUDT3	hsa-miR-4286
NUDT3	hsa-miR-295-3p
NUDT3	hsa-miR-5683
NUDT3	hsa-miR-873a-3p
NUDT3	hsa-miR-19b-5p
NUDT3	hsa-miR-3577-3p
NUDT3	hsa-miR-6732-5p
NUDT3	hsa-miR-708-5p
NUDT3	hsa-miR-2284g
NUDT3	hsa-miR-873-5p
NUDT3	hsa-miR-4724-5p
NUDT3	hsa-miR-22-3p
NUDT3	hsa-miR-4289
NUDT3	hsa-miR-7398p-5p
NUDT3	hsa-miR-145-5p
NUDT3	hsa-miR-6953-5p
NUDT3	hsa-miR-501-5p
NUDT3	hsa-miR-379-3p
NUDT3	hsa-miR-98-3p
NUDT3	hsa-miR-7333-5p
NUDT3	hsa-miR-770-3p
NUDT3	hsa-miR-4690-3p
NUDT3	hsa-miR-323b-3p
NUDT3	hsa-miR-7378-5p
NUDT3	hsa-miR-7339-5p
NUDT3	hsa-miR-3140-3p
NUDT3	hsa-miR-760-5p
NUDT3	hsa-miR-19a-5p
NUDT3	hsa-miR-2285d
NUDT3	hsa-miR-7-3p
NUDT3	hsa-miR-2462

NUDT3	hsa-miR-3531-5p
NUDT3	hsa-miR-23b-5p
NUDT3	hsa-miR-704
NUDT3	hsa-miR-3473f
NUDT3	hsa-miR-4433b-5p
NUDT3	hsa-miR-7472-5p
NUDT3	hsa-miR-7452-5p
NUDT3	hsa-miR-7182-3p
NUDT3	hsa-miR-1233-3p
NUDT3	hsa-miR-7649-5p
NUDT3	hsa-miR-691
NUDT3	hsa-miR-1208
NUDT3	hsa-miR-6590-3p
NUDT3	hsa-miR-7317-3p
NUDT3	hsa-miR-350
NUDT3	hsa-miR-4482-3p
NUDT3	hsa-miR-1807
NUDT3	hsa-miR-8093
NUDT3	hsa-miR-3160-3p
NUDT3	hsa-miR-1966-3p
NUDT3	hsa-miR-16-2-3p
NUDT3	hsa-miR-888-5p
NUDT3	hsa-miR-1273h-3p
NUDT3	hsa-miR-4535
NUDT3	hsa-miR-8853
NUDT3	hsa-miR-1936
NUDT3	hsa-miR-3662
NUDT3	hsa-miR-302b-5p
NUDT3	hsa-miR-590-5p
NUDT3	hsa-miR-548ar-3p
NUDT3	hsa-miR-518c-5p
NUDT3	hsa-miR-345-5p
NUDT3	hsa-miR-4757-5p
NUDT3	hsa-miR-217-5p
NUDT3	hsa-miR-889
NUDT3	hsa-miR-873a-5p.2
NUDT3	hsa-miR-4637
NUDT3	hsa-miR-301
NUDT3	hsa-miR-3104-5p
NUDT3	hsa-miR-743b-3p
NUDT3	hsa-miR-5587-5p
NUDT3	hsa-miR-483-3p.2
NUDT3	hsa-miR-107-3p
NUDT3	hsa-miR-3194-3p
NUDT3	hsa-miR-6892-5p
NUDT3	hsa-miR-2682-3p

NUDT3	hsa-miR-6715-3p
NUDT3	hsa-miR-2299-5p
NUDT3	hsa-miR-8866
NUDT3	hsa-miR-6931-3p
NUDT3	hsa-miR-601
NUDT3	hsa-miR-7112-5p
NUDT3	hsa-miR-664a-5p
NUDT3	hsa-miR-8908b
NUDT3	hsa-miR-7274-5p
NUDT3	hsa-miR-7185-5p
NUDT3	hsa-miR-499
NUDT3	hsa-miR-644a
NUDT3	hsa-miR-3679-3p
NUDT3	hsa-miR-7398d-3p
NUDT3	hsa-miR-1898
NUDT3	hsa-miR-6134
NUDT3	hsa-miR-4756-3p
NUDT3	hsa-miR-212-3p
NUDT3	hsa-miR-1913
NUDT3	hsa-miR-7843-5p
NUDT3	hsa-miR-571
NUDT3	hsa-miR-215-5p
NUDT3	hsa-miR-7279-5p
NUDT3	hsa-miR-4510
NUDT3	hsa-miR-6896-3p
NUDT3	hsa-miR-7-5p
NUDT3	hsa-miR-6979-5p
NUDT3	hsa-miR-216b-5p
NUDT3	hsa-miR-193-5p
NUDT3	hsa-miR-516a-3p
NUDT3	hsa-miR-4751
NUDT3	hsa-miR-371
NUDT3	hsa-miR-548e-3p
NUDT3	hsa-miR-1261
NUDT3	hsa-miR-6744-5p
NUDT3	hsa-miR-1179
NUDT3	hsa-miR-2294
NUDT3	hsa-miR-5003-3p
NUDT3	hsa-miR-7398r-5p
NUDT3	hsa-miR-4772-3p
NUDT3	hsa-miR-938
NUDT3	hsa-miR-4472
NUDT3	hsa-miR-654-5p
NUDT3	hsa-miR-3665
NUDT3	hsa-miR-200ac-5p
NUDT3	hsa-miR-758-3p

NUDT3	hsa-miR-612
NUDT3	hsa-miR-7339-3p
NUDT3	hsa-miR-3201
NUDT3	hsa-miR-6826-3p
NUDT3	hsa-miR-4633-5p
NUDT3	hsa-miR-669b-5p
NUDT3	hsa-miR-7398j-3p
NUDT3	hsa-miR-6876-5p
NUDT3	hsa-miR-8842
NUDT3	hsa-miR-2113
NUDT3	hsa-miR-34-5p
NUDT3	hsa-miR-1257
NUDT3	hsa-miR-15
NUDT3	hsa-miR-6933-3p
NUDT3	hsa-miR-2380
NUDT3	hsa-miR-6502-5p
NUDT3	hsa-miR-492
NUDT3	hsa-miR-6130
NUDT3	hsa-miR-224-5p
NUDT3	hsa-miR-2319
NUDT3	hsa-miR-669h-5p
NUDT3	hsa-miR-578
NUDT3	hsa-miR-7451-3p
NUDT3	hsa-miR-497-3p
NUDT3	hsa-miR-4725-3p
NUDT3	hsa-miR-2483-3p
NUDT3	hsa-miR-3961
NUDT3	hsa-miR-142b
NUDT3	hsa-miR-200c-5p
NUDT3	hsa-miR-1953
NUDT3	hsa-miR-6984-5p
NUDT3	hsa-miR-2415-3p
NUDT3	hsa-miR-6929-5p
NUDT3	hsa-miR-4719
NUDT3	hsa-miR-1250-3p
NUDT3	hsa-miR-371a-5p
NUDT3	hsa-miR-712-5p
NUDT3	hsa-miR-7028-3p
NUDT3	hsa-miR-548bd-3p
NUDT3	hsa-miR-516-3p
NUDT3	hsa-miR-142-3p.2
NUDT3	hsa-miR-6995-3p
NUDT3	hsa-miR-6752-5p
NUDT3	hsa-miR-7388a-5p
NUDT3	hsa-miR-7856-5p
NUDT3	hsa-miR-4476

NUDT3	hsa-miR-454-3p
NUDT3	hsa-miR-3938-5p
NUDT3	hsa-miR-1276
NUDT3	hsa-miR-548x-5p
NUDT3	hsa-miR-1954
NUDT3	hsa-miR-3120-5p
NUDT3	hsa-miR-2285y
NUDT3	hsa-miR-7194-5p
NUDT3	hsa-miR-6525
NUDT3	hsa-miR-122
NUDT3	hsa-miR-4769-5p
NUDT3	hsa-miR-6843-3p
NUDT3	hsa-miR-124-3p.2
NUDT3	hsa-miR-2439-5p
NUDT3	hsa-miR-190b-3p
NUDT3	hsa-miR-4441
NUDT3	hsa-miR-2466-3p
NUDT3	hsa-miR-2309
NUDT3	hsa-miR-514b-5p
NUDT3	hsa-miR-6841-3p
NUDT3	hsa-miR-1941-3p
NUDT3	hsa-miR-4299
NUDT3	hsa-miR-6893-5p
NUDT3	hsa-miR-1550-5p
NUDT3	hsa-miR-7398l-3p
NUDT3	hsa-miR-4765
NUDT3	hsa-miR-493
NUDT3	hsa-miR-4708-5p
NUDT3	hsa-miR-549-5p
NUDT3	hsa-miR-548bb-5p
NUDT3	hsa-miR-1464
NUDT3	hsa-miR-7649-3p
NUDT3	hsa-miR-335-3p
NUDT3	hsa-miR-7021-3p
NUDT3	hsa-miR-2425-5p
NUDT3	hsa-miR-125a-3p
NUDT3	hsa-miR-3180-5p
NUDT3	hsa-miR-191-5p
NUDT3	hsa-miR-6573-5p
NUDT3	hsa-miR-4677-5p
NUDT3	hsa-miR-7199-3p
NUDT3	hsa-miR-135-3p
NUDT3	hsa-miR-93-3p
NUDT3	hsa-miR-7674-3p
NUDT3	hsa-miR-526b-5p
NUDT3	hsa-miR-4715-3p

NUDT3	hsa-miR-101-2-5p
NUDT3	hsa-miR-2331-3p
NUDT3	hsa-miR-7865
NUDT3	hsa-miR-4256
NUDT3	hsa-miR-6890-5p
NUDT3	hsa-miR-203
NUDT3	hsa-miR-5001-3p
NUDT3	hsa-miR-136-5p
NUDT3	hsa-miR-6847-3p
NUDT3	hsa-miR-4792
NUDT3	hsa-miR-6790-3p
NUDT3	hsa-miR-6974-3p
NUDT3	hsa-miR-3573-5p
NUDT3	hsa-miR-7079-5p
NUDT3	hsa-miR-194-5p
NUDT3	hsa-miR-329b
NUDT3	hsa-miR-3668
NUDT3	hsa-miR-527
NUDT3	hsa-miR-508-3p
NUDT3	hsa-miR-5008-5p
NUDT3	hsa-miR-7276-5p
NUDT3	hsa-miR-2467-5p
NUDT3	hsa-miR-1952
NUDT3	hsa-miR-7111-5p
NUDT3	hsa-miR-2896
NUDT3	hsa-miR-29
NUDT3	hsa-miR-5681a
NUDT3	hsa-miR-5119
NUDT3	hsa-miR-134-3p
NUDT3	hsa-miR-8847
NUDT3	hsa-miR-455-5p
NUDT3	hsa-miR-6822-3p
NUDT3	hsa-miR-8908f
NUDT3	hsa-miR-340-5p
NUDT3	hsa-miR-31-3p
NUDT3	hsa-miR-466q
NUDT3	hsa-miR-301a-5p
NUDT3	hsa-miR-568
NUDT3	hsa-miR-767-5p
NUDT3	hsa-miR-2434
NUDT3	hsa-miR-4713-5p
NUDT3	hsa-miR-4693-3p
NUDT3	hsa-miR-8829
NUDT3	hsa-miR-6823-5p
NUDT3	hsa-miR-672-3p
NUDT3	hsa-miR-1909-5p

NUDT3	hsa-miR-2464-3p
NUDT3	hsa-miR-6534
NUDT3	hsa-miR-7313-3p
NUDT3	hsa-miR-8070
NUDT3	hsa-miR-6782-5p
NUDT3	hsa-miR-888-3p
NUDT3	hsa-miR-6523a
NUDT3	hsa-miR-8791
NUDT3	hsa-miR-1193-3p
NUDT3	hsa-miR-505-3p
NUDT3	hsa-miR-29b-5p
NUDT3	hsa-miR-199-3p
NUDT3	hsa-miR-1251-5p
NUDT3	hsa-miR-4778-5p
NUDT3	hsa-miR-6654-3p
NUDT3	hsa-miR-5571-5p
NUDT3	hsa-miR-4318
NUDT3	hsa-miR-4524a-3p
NUDT3	hsa-miR-585-5p
NUDT3	hsa-miR-5001-5p
NUDT3	hsa-miR-7235-3p
NUDT3	hsa-miR-496.2
NUDT3	hsa-miR-8807
NUDT3	hsa-miR-7283-5p
NUDT3	hsa-miR-3166
NUDT3	hsa-miR-4766-5p
NUDT3	hsa-miR-3117-5p
NUDT3	hsa-miR-4780
NUDT3	hsa-miR-7106-5p
NUDT3	hsa-miR-214-5p
NUDT3	hsa-miR-4282
NUDT3	hsa-miR-609
NUDT3	hsa-miR-2300-3p
NUDT3	hsa-miR-192-5p
NUDT3	hsa-miR-3602
NUDT3	hsa-miR-7386d-3p
NUDT3	hsa-miR-145-5p
NUDT3	hsa-miR-6874-3p
NUDT3	hsa-miR-1204
NUDT3	hsa-miR-29a-3p
NUDT3	hsa-miR-6790-5p
NUDT3	hsa-miR-7224-5p
NUDT3	hsa-miR-8103
NUDT3	hsa-miR-4423-5p
NUDT3	hsa-miR-1593
NUDT3	hsa-miR-7321-3p

NUDT3	hsa-miR-1245a
NUDT3	hsa-miR-3680-5p
NUDT3	hsa-miR-6742-3p
NUDT3	hsa-miR-490-5p
NUDT3	hsa-miR-4694-5p
NUDT3	hsa-miR-2439-3p
NUDT3	hsa-miR-152-5p
NUDT3	hsa-miR-4522
NUDT3	hsa-miR-491-5p
NUDT3	hsa-miR-7358-3p
NUDT3	hsa-miR-3557-3p
NUDT3	hsa-miR-493-3p
NUDT3	hsa-miR-6756-5p
NUDT3	hsa-miR-455-3p
NUDT3	hsa-miR-8881
NUDT3	hsa-miR-34
NUDT3	hsa-miR-6528
NUDT3	hsa-miR-7671-3p
NUDT3	hsa-miR-7373a-3p
NUDT3	hsa-miR-1199-5p
NUDT3	hsa-miR-4773
NUDT3	hsa-miR-2898
NUDT3	hsa-miR-101-1-5p
NUDT3	hsa-miR-6780b-3p
NUDT3	hsa-miR-2486-5p
NUDT3	hsa-miR-1323
NUDT3	hsa-miR-874
NUDT3	hsa-miR-1285-3p
NUDT3	hsa-miR-3663-3p
NUDT3	hsa-miR-340-3p
NUDT3	hsa-miR-6688-3p
NUDT3	hsa-miR-7267-3p
NUDT3	hsa-miR-4328
NUDT3	hsa-miR-6745
NUDT3	hsa-miR-1744-5p
NUDT3	hsa-miR-6847-5p
NUDT3	hsa-miR-7282-3p
NUDT3	hsa-miR-2285h
NUDT3	hsa-miR-33a-3p
NUDT3	hsa-miR-190-5p
NUDT3	hsa-miR-5191
NUDT3	hsa-miR-4308
NUDT3	hsa-miR-3689-3p
NUDT3	hsa-miR-214-3p
NUDT3	hsa-miR-6399
NUDT3	hsa-miR-129-5p

NUDT3	hsa-miR-6889-3p
NUDT3	hsa-miR-6392-5p
NUDT3	hsa-miR-1966-5p
NUDT3	hsa-miR-7355-5p
NUDT3	hsa-miR-6886-3p
NUDT3	hsa-miR-373-5p
NUDT3	hsa-miR-103-3p
NUDT3	hsa-miR-7666-3p
NUDT3	hsa-miR-706
NUDT3	hsa-miR-616-5p
NUDT3	hsa-miR-411-5p.2
NUDT3	hsa-miR-200c-3p
NUDT3	hsa-miR-2681-5p
NUDT3	hsa-miR-4665-5p
NUDT3	hsa-miR-7226-3p
NUDT3	hsa-miR-4252
NUDT3	hsa-miR-7864
NUDT3	hsa-miR-6779-5p
NUDT3	hsa-miR-7327-5p
NUDT3	hsa-miR-603
NUDT3	hsa-miR-5121
NUDT3	hsa-miR-378a-5p
NUDT3	hsa-miR-2468
NUDT3	hsa-miR-7664-5p
NUDT3	hsa-miR-1799
NUDT3	hsa-miR-449c-5p
NUDT3	hsa-miR-6803-5p
NUDT3	hsa-miR-2383
NUDT3	hsa-miR-384
NUDT3	hsa-miR-4279
NUDT3	hsa-miR-4802-5p
NUDT3	hsa-let-7ag-3p
NUDT3	hsa-miR-7201-5p
NUDT3	hsa-miR-4430
NUDT3	hsa-miR-8862
NUDT3	hsa-miR-4494
NUDT3	hsa-miR-500b-5p
NUDT3	hsa-miR-2411-5p
NUDT3	hsa-miR-7198-5p
NUDT3	hsa-miR-548b-3p
NUDT3	hsa-miR-7398i-3p
NUDT3	hsa-miR-2891
NUDT3	hsa-miR-7226-5p
NUDT3	hsa-miR-6739-5p
NUDT3	hsa-miR-2351
NUDT3	hsa-miR-6878-5p

NUDT3	hsa-miR-101a-5p
NUDT3	hsa-miR-6690-5p
NUDT3	hsa-miR-6761-5p
NUDT3	hsa-miR-6897-3p
NUDT3	hsa-miR-6912-5p
NUDT3	hsa-miR-200c-3p
NUDT3	hsa-miR-508-5p
NUDT3	hsa-miR-4659b-3p
NUDT3	hsa-miR-548aq-3p
NUDT3	hsa-miR-485
NUDT3	hsa-miR-3120-3p
NUDT3	hsa-miR-1651-5p
NUDT3	hsa-miR-5584-5p
NUDT3	hsa-miR-7386a-3p
NUDT3	hsa-miR-2473
NUDT3	hsa-miR-8062
NUDT3	hsa-miR-3162-3p
NUDT3	hsa-miR-7224-3p
NUDT3	hsa-miR-7482-3p
NUDT3	hsa-miR-548s
NUDT3	hsa-miR-6859-5p
NUDT3	hsa-miR-548av-5p
NUDT3	hsa-miR-3170
NUDT3	hsa-miR-411-5p
NUDT3	hsa-miR-616-3p
NUDT3	hsa-miR-4539
NUDT3	hsa-miR-8903
NUDT3	hsa-miR-1237-3p
NUDT3	hsa-miR-513c-3p
NUDT3	hsa-miR-2409
NUDT3	hsa-miR-605
NUDT3	hsa-miR-1-2-5p
NUDT3	hsa-miR-346-3p
NUDT3	hsa-miR-7273-5p
NUDT3	hsa-miR-1835
NUDT3	hsa-miR-4798-3p
NUDT3	hsa-miR-7269-3p
NUDT3	hsa-miR-3100-5p
NUDT3	hsa-miR-133b-5p
NUDT3	hsa-miR-5585-3p
NUDT3	hsa-miR-5125-5p
NUDT3	hsa-miR-7042-3p
NUDT3	hsa-miR-460-5p
NUDT3	hsa-miR-676-3p
NUDT3	hsa-miR-324-3p
NUDT3	hsa-miR-8885

NUDT3	hsa-miR-1647
NUDT3	hsa-miR-532-3p
NUDT3	hsa-miR-376a-2-5p
NUDT3	hsa-miR-8827
NUDT3	hsa-miR-1836
NUDT3	hsa-miR-4799-5p
NUDT3	hsa-miR-5007-5p
NUDT3	hsa-miR-518-5p
NUDT3	hsa-miR-3609
NUDT3	hsa-miR-193
NUDT3	hsa-miR-1343-5p
NUDT3	hsa-miR-192-3p
NUDT3	hsa-miR-105-5p
NUDT3	hsa-miR-6133
NUDT3	hsa-miR-6547-3p
NUDT3	hsa-miR-7341-5p
NUDT3	hsa-miR-7670-5p
NUDT3	hsa-miR-188-5p
NUDT3	hsa-miR-7675-3p
NUDT3	hsa-miR-3548
NUDT3	hsa-miR-376c-3p
NUDT3	hsa-miR-17-3p
NUDT3	hsa-miR-17
NUDT3	hsa-miR-4700-3p
NUDT3	hsa-miR-7386j-3p
NUDT3	hsa-miR-655-5p
NUDT3	hsa-miR-7174-5p
NUDT3	hsa-miR-5002-3p
NUDT3	hsa-miR-1248
NUDT3	hsa-miR-669
NUDT3	hsa-miR-6794-5p
NUDT3	hsa-miR-1783
NUDT3	hsa-miR-302a-5p
NUDT3	hsa-miR-9-3p
NUDT3	hsa-miR-4764-5p
NUDT3	hsa-miR-181a-2-3p
NUDT3	hsa-miR-2290
NUDT3	hsa-miR-143-5p
NUDT3	hsa-miR-8895
NUDT3	hsa-miR-546
NUDT3	hsa-miR-758-5p
NUDT3	hsa-miR-6733-5p
NUDT3	hsa-miR-6953-3p
NUDT3	hsa-miR-134-5p
NUDT3	hsa-miR-93-5p
NUDT3	hsa-miR-7302-3p

NUDT3	hsa-miR-2889
NUDT3	hsa-miR-2285t
NUDT3	hsa-miR-6939-3p
NUDT3	hsa-miR-548b-5p
NUDT3	hsa-miR-30
NUDT3	hsa-miR-150-5p
NUDT3	hsa-miR-7682-3p
NUDT3	hsa-miR-5110
NUDT3	hsa-miR-2285aa
NUDT3	hsa-miR-7377-5p
NUDT3	hsa-miR-1178-3p
NUDT3	hsa-miR-4653-3p
NUDT3	hsa-miR-7473-5p
NUDT3	hsa-miR-484
NUDT3	hsa-miR-3072-3p
NUDT3	hsa-miR-2284s
NUDT3	hsa-miR-3189-5p
NUDT3	hsa-miR-562
NUDT3	hsa-miR-20-5p
NUDT3	hsa-miR-2459
NUDT3	hsa-miR-920
NUDT3	hsa-miR-669i
NUDT3	hsa-miR-219-1-3p
NUDT3	hsa-miR-411-5p
NUDT3	hsa-miR-148a-3p
NUDT3	hsa-miR-6986-5p
NUDT3	hsa-miR-702-3p
NUDT3	hsa-miR-3153
NUDT3	hsa-miR-329-3p
NUDT3	hsa-miR-679
NUDT3	hsa-miR-429-3p
NUDT3	hsa-miR-7178-3p
NUDT3	hsa-miR-3148
NUDT3	hsa-miR-4477b
NUDT3	hsa-miR-569
NUDT3	hsa-miR-8805
NUDT3	hsa-miR-7398u-3p
NUDT3	hsa-miR-4687-5p
NUDT3	hsa-miR-548ap-5p
NUDT3	hsa-miR-4788
NUDT3	hsa-miR-4714-3p
NUDT3	hsa-miR-2284n
NUDT3	hsa-miR-5588-5p
NUDT3	hsa-miR-6817-3p
NUDT3	hsa-miR-432-3p
NUDT3	hsa-miR-3607-5p

NUDT3	hsa-miR-18b-3p
NUDT3	hsa-miR-6608-5p
NUDT3	hsa-miR-362-5p
NUDT3	hsa-miR-7262b-5p
NUDT3	hsa-miR-4489
NUDT3	hsa-miR-181b-3p
NUDT3	hsa-miR-4320
NUDT3	hsa-miR-589-5p
NUDT3	hsa-miR-2458
NUDT3	hsa-miR-6669-3p
NUDT3	hsa-miR-619
NUDT3	hsa-miR-4483
NUDT3	hsa-miR-6832-5p
NUDT3	hsa-miR-7341-3p
NUDT3	hsa-miR-22-5p
NUDT3	hsa-miR-6643-5p
NUDT3	hsa-miR-6766-5p
NUDT3	hsa-miR-138-1-3p
NUDT3	hsa-miR-216a
NUDT3	hsa-miR-4775
NUDT3	hsa-miR-6758-5p
NUDT3	hsa-miR-6875-3p
NUDT3	hsa-miR-548aw
NUDT3	hsa-miR-2285b
NUDT3	hsa-miR-1197
NUDT3	hsa-miR-6333
NUDT3	hsa-miR-6787-3p
NUDT3	hsa-miR-2323
NUDT3	hsa-miR-921
NUDT3	hsa-miR-653-5p
NUDT3	hsa-miR-656
NUDT3	hsa-miR-7179-3p
NUDT3	hsa-miR-6396
NUDT3	hsa-miR-369-5p
NUDT3	hsa-miR-370-5p
NUDT3	hsa-miR-7207-5p
NUDT3	hsa-miR-542-3p
NUDT3	hsa-miR-5697-5p
NUDT3	hsa-miR-6737-5p
NUDT3	hsa-miR-7398k-5p
NUDT3	hsa-miR-2313-3p
NUDT3	hsa-miR-550a-3p
NUDT3	hsa-miR-452-5p
NUDT3	hsa-miR-128-3p
NUDT3	hsa-miR-758-3p
NUDT3	hsa-miR-3133

NUDT3	hsa-miR-1284
NUDT3	hsa-miR-4708-3p
NUDT3	hsa-miR-302d-5p
NUDT3	hsa-miR-8075
NUDT3	hsa-miR-1434-3p
NUDT3	hsa-miR-5190
NUDT3	hsa-miR-8908-5p
NUDT3	hsa-miR-3613-5p
NUDT3	hsa-miR-6701-3p
NUDT3	hsa-miR-4436b-5p
NUDT3	hsa-miR-668-3p
NUDT3	hsa-miR-545-3p
NUDT3	hsa-miR-3975
NUDT3	hsa-miR-5582-3p
NUDT3	hsa-miR-1976
NUDT3	hsa-miR-4715-5p
NUDT3	hsa-miR-344eh-5p
NUDT3	hsa-miR-7090-5p
NUDT3	hsa-miR-4698
NUDT3	hsa-miR-8116
NUDT3	hsa-miR-7235-5p
NUDT3	hsa-miR-7665-3p
NUDT3	hsa-miR-7110-3p
NUDT3	hsa-miR-142-3p
NUDT3	hsa-miR-7359-5p
NUDT3	hsa-miR-2285x
NUDT3	hsa-miR-7087-3p
NUDT3	hsa-miR-410-3p
NUDT3	hsa-miR-924
NUDT3	hsa-miR-8868
NUDT3	hsa-miR-6885-3p
NUDT3	hsa-miR-624-3p
NUDT3	hsa-miR-8485
NUDT3	hsa-miR-4777-5p
NUDT3	hsa-miR-489
NUDT3	hsa-miR-8061
NUDT3	hsa-miR-8906
NUDT3	hsa-miR-4742-3p
NUDT3	hsa-miR-3606-5p
NUDT3	hsa-miR-666-5p
NUDT3	hsa-miR-425-3p
NUDT3	hsa-miR-7464-3p
NUDT3	hsa-miR-130
NUDT3	hsa-miR-30c-3p
NUDT3	hsa-miR-3097-5p
NUDT3	hsa-miR-1246

NUDT3	hsa-miR-3191-5p
NUDT3	hsa-miR-1267
NUDT3	hsa-miR-7261-5p
NUDT3	hsa-miR-7250-3p
NUDT3	hsa-miR-4771
NUDT3	hsa-miR-3126-5p
NUDT3	hsa-miR-105b
NUDT3	hsa-miR-580-5p
NUDT3	hsa-miR-6925-5p
NUDT3	hsa-miR-302
NUDT3	hsa-miR-591
NUDT3	hsa-miR-6373
NUDT3	hsa-miR-561-3p
NUDT3	hsa-miR-1599
NUDT3	hsa-miR-6965-5p
NUDT3	hsa-miR-599-3p
NUDT3	hsa-miR-3085-3p
NUDT3	hsa-miR-513b-5p
NUDT3	hsa-miR-2399-3p
NUDT3	hsa-miR-5699-3p
NUDT3	hsa-miR-2897
NUDT3	hsa-miR-1236-3p
NUDT3	hsa-miR-378
NUDT3	hsa-miR-336-5p
NUDT3	hsa-miR-7332-5p
NUDT3	hsa-miR-3167-5p
NUDT3	hsa-miR-7160-5p
NUDT3	hsa-miR-3646
NUDT3	hsa-miR-4284
NUDT3	hsa-miR-7394a-5p
NUDT3	hsa-miR-7271-3p
NUDT3	hsa-miR-5187-5p
NUDT3	hsa-miR-2314
NUDT3	hsa-miR-513a-3p
NUDT3	hsa-miR-888-5p
NUDT3	hsa-miR-4433a-5p
NUDT3	hsa-miR-6628-3p
NUDT3	hsa-miR-3160-5p
NUDT3	hsa-miR-7398-5p
NUDT3	hsa-miR-8852
NUDT3	hsa-miR-2285e
NUDT3	hsa-miR-1225-5p
NUDT3	hsa-miR-6764-3p
NUDT3	hsa-miR-6985-5p
NUDT3	hsa-miR-126b-5p
NUDT3	hsa-miR-584-3p

NUDT3	hsa-miR-433
NUDT3	hsa-miR-3098-3p
NUDT3	hsa-miR-452-5p
NUDT3	hsa-miR-7324-3p
NUDT3	hsa-miR-6778-5p
NUDT3	hsa-miR-764
NUDT3	hsa-miR-2357
NUDT3	hsa-miR-134
NUDT3	hsa-miR-8106
NUDT3	hsa-miR-6831-5p
NUDT3	hsa-miR-6765-5p
NUDT3	hsa-miR-2367-3p
NUDT3	hsa-miR-196b-3p
NUDT3	hsa-miR-1303
NUDT3	hsa-miR-2442
NUDT3	hsa-miR-7371-5p
NUDT3	hsa-miR-6575-5p
NUDT3	hsa-miR-5095
NUDT3	hsa-miR-7322-3p
NUDT3	hsa-miR-6997-3p
NUDT3	hsa-miR-4270
NUDT3	hsa-miR-6090
NUDT3	hsa-miR-582-3p
NUDT3	hsa-miR-1646
NUDT3	hsa-miR-8816
NUDT3	hsa-miR-183-5p
NUDT3	hsa-miR-934-3p
NUDT3	hsa-miR-7398p-3p
NUDT3	hsa-miR-6936-5p
NUDT3	hsa-miR-31-5p
NUDT3	hsa-miR-7256-3p
NUDT3	hsa-miR-6944-5p
NUDT3	hsa-miR-2485
NUDT3	hsa-miR-1252
NUDT3	hsa-miR-2894
NUDT3	hsa-miR-181-3p
NUDT3	hsa-miR-18-5p
NUDT3	hsa-miR-6842-3p
NUDT3	hsa-miR-31-5p
NUDT3	hsa-miR-4774-5p
NUDT3	hsa-miR-2455
NUDT3	hsa-miR-7662-5p
NUDT3	hsa-miR-1252-3p
NUDT3	hsa-miR-6949-5p
NUDT3	hsa-miR-4268
NUDT3	hsa-miR-487a-5p

NUDT3	hsa-miR-7159-5p
NUDT3	hsa-miR-3714
NUDT3	hsa-miR-3569-3p
NUDT3	hsa-miR-6755-5p
NUDT3	hsa-miR-302c-3p
NUDT3	hsa-miR-186-3p
NUDT3	hsa-miR-1307-3p
NUDT3	hsa-miR-766-3p
NUDT3	hsa-miR-7092-5p
NUDT3	hsa-miR-669e-5p
NUDT3	hsa-miR-742-5p
NUDT3	hsa-miR-214-3p
NUDT3	hsa-miR-3087-3p
NUDT3	hsa-miR-4732-5p
NUDT3	hsa-miR-8863
NUDT3	hsa-miR-4428
NUDT3	hsa-miR-5011-5p
NUDT3	hsa-miR-7011-3p
NUDT3	hsa-miR-7205-3p
NUDT3	hsa-miR-1224-5p
NUDT3	hsa-miR-128-1-5p
NUDT3	hsa-miR-7083-3p
NUDT3	hsa-miR-4422
NUDT3	hsa-miR-7845-5p
NUDT3	hsa-miR-489-3p
NUDT3	hsa-miR-1245a
NUDT3	hsa-miR-3927-3p
NUDT3	hsa-miR-2681-3p
NUDT3	hsa-miR-3059-5p
NUDT3	hsa-miR-330-5p
NUDT3	hsa-miR-502a
NUDT3	hsa-miR-3675-5p
NUDT3	hsa-miR-548e-5p
NUDT3	hsa-miR-5583-5p
NUDT3	hsa-miR-150-5p
NUDT3	hsa-miR-29a-3p
NUDT3	hsa-miR-4656
NUDT3	hsa-miR-548ad-5p
NUDT3	hsa-miR-2115-5p
NUDT3	hsa-miR-5046
NUDT3	hsa-miR-7005-5p
NUDT3	hsa-miR-632
NUDT3	hsa-miR-1804
NUDT3	hsa-miR-27
NUDT3	hsa-miR-7667-3p
NUDT3	hsa-miR-7347-3p

NUDT3	hsa-miR-3165
NUDT3	hsa-miR-6728-3p
NUDT3	hsa-miR-29a-5p
NUDT3	hsa-miR-7677-3p
NUDT3	hsa-miR-1643-3p
NUDT3	hsa-miR-3942-3p
NUDT3	hsa-miR-5010-3p
NUDT3	hsa-miR-8099
NUDT3	hsa-miR-7338-5p
NUDT3	hsa-miR-2287
NUDT3	hsa-miR-7328-3p
NUDT3	hsa-miR-217
NUDT3	hsa-miR-106a-3p
NUDT3	hsa-miR-6731-5p
NUDT3	hsa-miR-6730-5p
NUDT3	hsa-miR-8055
NUDT3	hsa-miR-373-3p
NUDT3	hsa-miR-551-5p
NUDT3	hsa-miR-1909-3p
NUDT3	hsa-miR-7204-3p
NUDT3	hsa-miR-344-3p
NUDT3	hsa-miR-4474-3p
NUDT3	hsa-miR-6844
NUDT3	hsa-miR-30e
NUDT3	hsa-miR-6741-3p
NUDT3	hsa-miR-6811-3p
NUDT3	hsa-miR-5000-5p
NUDT3	hsa-miR-5112
NUDT3	hsa-miR-2325
NUDT3	hsa-miR-6397
NUDT3	hsa-miR-4722-5p
NUDT3	hsa-miR-106b-5p
NUDT3	hsa-miR-7159-3p
NUDT3	hsa-miR-7362-3p
NUDT3	hsa-miR-7663-3p
NUDT3	hsa-miR-6797-5p
NUDT3	hsa-miR-302-3p
NUDT3	hsa-miR-300-5p
NUDT3	hsa-miR-7386m-3p
NUDT3	hsa-miR-362-3p
NUDT3	hsa-miR-27a-5p
NUDT3	hsa-miR-3935
NUDT3	hsa-miR-7085-3p
NUDT3	hsa-miR-5580-5p
NUDT3	hsa-miR-2284v
NUDT3	hsa-miR-4743-3p

NUDT3	hsa-miR-1943-5p
NUDT3	hsa-miR-7194-3p
NUDT3	hsa-miR-6881-5p
NUDT3	hsa-miR-3192-3p
NUDT3	hsa-miR-875-3p
NUDT3	hsa-miR-4720-5p
NUDT3	hsa-miR-2312
NUDT3	hsa-miR-328-3p
NUDT3	hsa-miR-7169-5p
NUDT3	hsa-miR-7277-3p
NUDT3	hsa-miR-23
NUDT3	hsa-miR-21-5p
NUDT3	hsa-miR-1329-5p
NUDT3	hsa-miR-873-5p.1
NUDT3	hsa-miR-618
NUDT3	hsa-miR-7118-5p
NUDT3	hsa-miR-1674
NUDT3	hsa-miR-4733-5p
NUDT3	hsa-miR-3473c
NUDT3	hsa-miR-1260a
NUDT3	hsa-miR-411a
NUDT3	hsa-miR-1704
NUDT3	hsa-miR-666-3p
NUDT3	hsa-miR-7398l-5p
NUDT3	hsa-miR-4502
NUDT3	hsa-miR-6900-5p
NUDT3	hsa-miR-4776-3p
NUDT3	hsa-miR-4709-5p
NUDT3	hsa-miR-7281-5p
NUDT3	hsa-miR-664-5p
NUDT3	hsa-miR-346
NUDT3	hsa-miR-3061-5p
NUDT3	hsa-miR-4790-3p
NUDT3	hsa-miR-1193-5p
NUDT3	hsa-miR-6578-3p
NUDT3	hsa-miR-3652
NUDT3	hsa-miR-7017-5p
NUDT3	hsa-miR-6873-3p
NUDT3	hsa-miR-7001-3p
NUDT3	hsa-miR-6925-3p
NUDT3	hsa-miR-4659a-5p
NUDT3	hsa-miR-570-3p
NUDT3	hsa-miR-7034-5p
NUDT3	hsa-miR-486-5p
NUDT3	hsa-miR-2454-5p
NUDT3	hsa-miR-8834b

NUDT3	hsa-miR-4693-5p
NUDT3	hsa-miR-8845
NUDT3	hsa-miR-8832
NUDT3	hsa-miR-301b-5p
NUDT3	hsa-miR-124
NUDT3	hsa-miR-6744-3p
NUDT3	hsa-miR-521
NUDT3	hsa-miR-7448-3p
NUDT3	hsa-miR-4455
NUDT3	hsa-miR-2360
NUDT3	hsa-miR-7015-3p
NUDT3	hsa-miR-4740-3p
NUDT3	hsa-miR-4764-3p
NUDT3	hsa-miR-1290
NUDT3	hsa-miR-331-5p
NUDT3	hsa-miR-20
NUDT3	hsa-miR-6992-3p
NUDT3	hsa-miR-6127
NUDT3	hsa-miR-5197-3p
NUDT3	hsa-miR-378g
NUDT3	hsa-miR-215-3p
NUDT3	hsa-miR-471-3p
NUDT3	hsa-miR-495-3p
NUDT3	hsa-miR-7186-3p
NUDT3	hsa-miR-7386d-5p
NUDT3	hsa-miR-2325b
NUDT3	hsa-miR-3613b
NUDT3	hsa-miR-3672
NUDT3	hsa-miR-5709-3p
NUDT3	hsa-miR-7381-3p
NUDT3	hsa-miR-320e
NUDT3	hsa-miR-4667-5p
NUDT3	hsa-miR-7288-5p
NUDT3	hsa-miR-149-3p
NUDT3	hsa-miR-4297
NUDT3	hsa-miR-4753-5p
NUDT3	hsa-miR-1296-3p
NUDT3	hsa-miR-525-5p
NUDT3	hsa-miR-6239
NUDT3	hsa-miR-676-5p
NUDT3	hsa-miR-27-3p
NUDT3	hsa-miR-3473be
NUDT3	hsa-miR-804
NUDT3	hsa-miR-6612-5p
NUDT3	hsa-miR-539-5p
NUDT3	hsa-miR-1929-5p

NUDT3	hsa-miR-7399-5p
NUDT3	hsa-miR-670-3p
NUDT3	hsa-miR-6605-5p
NUDT3	hsa-miR-7398o-5p
NUDT3	hsa-miR-7279-3p
NUDT3	hsa-miR-7578
NUDT3	hsa-miR-3941
NUDT3	hsa-miR-218-5p
NUDT3	hsa-miR-548aq-5p
NUDT3	hsa-let-7e-3p
NUDT3	hsa-miR-7151-3p
NUDT3	hsa-miR-431-5p
NUDT3	hsa-miR-1-3p
NUDT3	hsa-miR-383-3p
NUDT3	hsa-miR-2325c
NUDT3	hsa-miR-518a-5p
NUDT3	hsa-miR-6768-5p
NUDT3	hsa-miR-4261
NUDT3	hsa-miR-1299
NUDT3	hsa-miR-7109-5p
NUDT3	hsa-miR-6899-5p
NUDT3	hsa-miR-6923-5p
NUDT3	hsa-miR-6946-5p
NUDT3	hsa-miR-6978-5p
NUDT3	hsa-miR-4720-3p
NUDT3	hsa-miR-1737
NUDT3	hsa-miR-507
NUDT3	hsa-miR-140-3p.1
NUDT3	hsa-miR-2378
NUDT3	hsa-miR-7703
NUDT3	hsa-miR-642
NUDT3	hsa-miR-21-3p
NUDT3	hsa-miR-4280
NUDT3	hsa-miR-17-5p
NUDT3	hsa-miR-216
NUDT3	hsa-miR-3689b-5p
NUDT3	hsa-miR-487a
NUDT3	hsa-miR-5682
NUDT3	hsa-miR-30-5p
NUDT3	hsa-miR-623
NUDT3	hsa-miR-3958
NUDT3	hsa-miR-7340-5p
NUDT3	hsa-miR-7455-5p
NUDT3	hsa-miR-4774-3p
NUDT3	hsa-miR-876
NUDT3	hsa-miR-3110-3p

NUDT3	hsa-miR-6920-3p
NUDT3	hsa-miR-323
NUDT3	hsa-miR-2475
NUDT3	hsa-miR-6542-5p
NUDT3	hsa-miR-193a
NUDT3	hsa-miR-138-2-3p
NUDT3	hsa-miR-5622-5p
NUDT3	hsa-miR-7258-5p
NUDT3	hsa-miR-6798-5p
NUDT3	hsa-miR-1306-3p
NUDT3	hsa-miR-1245b-3p
NUDT3	hsa-miR-19-5p
NUDT3	hsa-miR-139-3p
NUDT3	hsa-miR-7398t-3p
NUDT3	hsa-miR-6848-5p
NUDT3	hsa-miR-101-3p.2
NUDT3	hsa-miR-1288-5p
NUDT3	hsa-miR-7156-5p
NUDT3	hsa-miR-301-3p
NUDT3	hsa-miR-8902
NUDT3	hsa-miR-3125
NUDT3	hsa-miR-4690-5p
NUDT3	hsa-miR-2329-3p
NUDT3	hsa-miR-7398m-3p
NUDT3	hsa-miR-7350-5p
NUDT3	hsa-miR-34bc-3p
NUDT3	hsa-miR-592-3p
NUDT3	hsa-miR-106-5p
NUDT3	hsa-miR-744-3p
NUDT3	hsa-miR-4450
NUDT3	hsa-miR-544-5p
NUDT3	hsa-miR-125b-2-3p
NUDT3	hsa-miR-7077-3p
NUDT3	hsa-miR-1285-3p
NUDT3	hsa-miR-6516-5p
NUDT3	hsa-miR-874-3p
NUDT3	hsa-miR-7456-3p
NUDT3	hsa-miR-488-5p
NUDT3	hsa-miR-2410
NUDT3	hsa-miR-298-3p
NUDT3	hsa-miR-1256
NUDT3	hsa-miR-668
NUDT3	hsa-miR-1769-3p
NUDT3	hsa-miR-1664-5p
NUDT3	hsa-miR-548ar-5p
NUDT3	hsa-miR-539-5p

NUDT3	hsa-miR-587
NUDT3	hsa-miR-7035-3p
NUDT3	hsa-miR-3620-5p
NUDT3	hsa-miR-2418
NUDT3	hsa-miR-6982-3p
NUDT3	hsa-miR-548c-3p
NUDT3	hsa-miR-642-3p
NUDT3	hsa-miR-7303-5p
NUDT3	hsa-miR-7214-5p
NUDT3	hsa-miR-3060-3p
NUDT3	hsa-miR-185-3p
NUDT3	hsa-miR-4635
NUDT3	hsa-miR-7461-3p
NUDT3	hsa-miR-152-3p
NUDT3	hsa-miR-681
NUDT3	hsa-miR-7185-3p
NUDT3	hsa-miR-1-5p
NUDT3	hsa-miR-7202-3p
NUDT3	hsa-miR-138-5p
NUDT3	hsa-miR-7026-5p
NUDT3	hsa-miR-1739
NUDT3	hsa-miR-7287-5p
NUDT3	hsa-miR-4779
NUDT3	hsa-miR-2285z
NUDT3	hsa-miR-2346
NUDT3	hsa-miR-4274
NUDT3	hsa-miR-1240
NUDT3	hsa-miR-2285g
NUDT3	hsa-miR-193-3p
NUDT3	hsa-miR-1298-3p
NUDT3	hsa-miR-4470
NUDT3	hsa-miR-1287-5p
NUDT3	hsa-miR-7686-5p
NUDT3	hsa-miR-4762-3p
NUDT3	hsa-miR-6556-5p
NUDT3	hsa-miR-7319-5p
NUDT3	hsa-miR-374b-3p
NUDT3	hsa-miR-2285k
NUDT3	hsa-miR-4728-5p
NUDT3	hsa-miR-338-3p
NUDT3	hsa-miR-519d-5p
NUDT3	hsa-miR-7388c-5p
NUDT3	hsa-miR-2423
NUDT3	hsa-miR-764-5p
NUDT3	hsa-miR-2315
NUDT3	hsa-miR-7398q-3p

NUDT3	hsa-miR-4766-3p
NUDT3	hsa-miR-98-5p
NUDT3	hsa-miR-7646-5p
NUDT3	hsa-miR-4742
NUDT3	hsa-miR-4509
NUDT3	hsa-miR-3671
NUDT3	hsa-miR-7166-5p
NUDT3	hsa-miR-3591-3p
NUDT3	hsa-miR-491-5p
NUDT3	hsa-miR-5125
NUDT3	hsa-miR-873a-5p.1
NUDT3	hsa-miR-187-5p
NUDT3	hsa-miR-1956-3p
NUDT3	hsa-miR-1757
NUDT3	hsa-miR-7371-3p
NUDT3	hsa-miR-7286-3p
NUDT3	hsa-miR-3064-3p
NUDT3	hsa-miR-8892
NUDT3	hsa-miR-4685-3p
NUDT3	hsa-miR-19-3p
NUDT3	hsa-miR-6512-5p
NUDT3	hsa-miR-8819
NUDT3	hsa-miR-345-3p
NUDT3	hsa-miR-194-3p
NUDT3	hsa-miR-2396
NUDT3	hsa-miR-3177-5p
NUDT3	hsa-miR-508-3p
NUDT3	hsa-miR-8052
NUDT3	hsa-miR-3944-5p
NUDT3	hsa-miR-1286
NUDT3	hsa-miR-7386k-5p
NUDT3	hsa-miR-195-3p
NUDT3	hsa-miR-548aa
NUDT3	hsa-miR-1838
NUDT3	hsa-miR-2284-3p
NUDT3	hsa-miR-7361-3p
NUDT3	hsa-miR-1908-5p
NUDT3	hsa-miR-6754-3p
NUDT3	hsa-miR-8802
NUDT3	hsa-miR-942-5p
NUDT3	hsa-miR-374a-3p
NUDT3	hsa-miR-7674-5p
NUDT3	hsa-miR-200b-3p
NUDT3	hsa-miR-4511
NUDT3	hsa-miR-563
NUDT3	hsa-miR-1185-5p

NUDT3	hsa-miR-3596
NUDT3	hsa-miR-7195-5p
NUDT3	hsa-miR-7170-5p
NUDT3	hsa-miR-3569
NUDT3	hsa-miR-380-3p
NUDT3	hsa-miR-7974
NUDT3	hsa-miR-4638-3p
NUDT3	hsa-miR-518-3p
NUDT3	hsa-miR-103
NUDT3	hsa-miR-490-3p
NUDT3	hsa-miR-378d
NUDT3	hsa-miR-7849-3p
NUDT3	hsa-miR-6740-5p
NUDT3	hsa-miR-15-5p
NUDT3	hsa-miR-181-5p
NUDT3	hsa-miR-943
NUDT3	hsa-miR-516a
NUDT3	hsa-miR-7345-3p
NUDT3	hsa-miR-1273e
NUDT3	hsa-miR-330-3p.2
NUDT3	hsa-miR-670
NUDT3	hsa-miR-6768-3p
NUDT3	hsa-miR-1295b-5p
NUDT3	hsa-miR-7386k-3p
NUDT3	hsa-miR-6857-5p
NUDT3	hsa-miR-7191-3p
NUDT3	hsa-miR-3622b-5p
NUDT3	hsa-miR-148
NUDT3	hsa-miR-2310
NUDT3	hsa-miR-5697-3p
NUDT3	hsa-miR-3604
NUDT3	hsa-miR-3559-5p
NUDT3	hsa-miR-1227-3p
NUDT3	hsa-miR-6908-3p
NUDT3	hsa-miR-719
NUDT3	hsa-miR-548az-5p
NUDT3	hsa-miR-8880
NUDT3	hsa-miR-6750-3p
NUDT3	hsa-miR-942-3p
NUDT3	hsa-miR-6515-3p
NUDT3	hsa-miR-762
NUDT3	hsa-miR-6518
NUDT3	hsa-miR-2400
NUDT3	hsa-miR-4423
NUDT3	hsa-miR-6544-3p
NUDT3	hsa-miR-532-5p

NUDT3	hsa-miR-4520-3p
NUDT3	hsa-miR-583
NUDT3	hsa-miR-548q
NUDT3	hsa-miR-3911
NUDT3	hsa-miR-7360-5p
NUDT3	hsa-miR-7164-5p
NUDT3	hsa-miR-130-5p
NUDT3	hsa-miR-7221-5p
NUDT3	hsa-miR-1912-3p
NUDT3	hsa-miR-4501
NUDT3	hsa-miR-4480
NUDT3	hsa-miR-1193
NUDT3	hsa-miR-5690
NUDT3	hsa-miR-6780a-5p
NUDT3	hsa-miR-7106-3p
NUDT3	hsa-miR-2284c
NUDT3	hsa-miR-335-5p
NUDT3	hsa-miR-802-3p
NUDT3	hsa-miR-6508-5p
NUDT3	hsa-miR-6871-5p
NUDT3	hsa-miR-371b-3p
NUDT3	hsa-miR-381-3p
NUDT3	hsa-miR-2368-3p
NUDT3	hsa-miR-146-5p
NUDT3	hsa-miR-503-3p
NUDT3	hsa-miR-1298
NUDT3	hsa-miR-1902
NUDT3	hsa-miR-7074-3p
NUDT3	hsa-miR-1933-3p
NUDT3	hsa-miR-1298-5p
NUDT3	hsa-miR-7018-3p
NUDT3	hsa-miR-3192-5p
NUDT3	hsa-miR-101-3p.1
NUDT3	hsa-miR-135a-1-3p
NUDT3	hsa-miR-372-3p
NUDT3	hsa-miR-5581-3p
NUDT3	hsa-miR-516b-3p
NUDT3	hsa-miR-3925-5p
NUDT3	hsa-miR-1982-3p
NUDT3	hsa-miR-5107-5p
NUDT3	hsa-miR-429
NUDT3	hsa-miR-2343
NUDT3	hsa-miR-4642
NUDT3	hsa-miR-320a
NUDT3	hsa-miR-151-3p
NUDT3	hsa-miR-6747-5p

NUDT3	hsa-miR-4448
NUDT3	hsa-miR-6878-3p
NUDT3	hsa-miR-3120-3p
NUDT3	hsa-miR-524-5p
NUDT3	hsa-miR-2419-3p
NUDT3	hsa-miR-365-3p
NUDT3	hsa-miR-548aj-5p
NUDT3	hsa-let-7-5p
NUDT3	hsa-miR-2463
NUDT3	hsa-miR-2340
NUDT3	hsa-miR-8792
NUDT3	hsa-miR-544a
NUDT3	hsa-miR-6587-5p
NUDT3	hsa-miR-190-3p
NUDT3	hsa-miR-1814a
NUDT3	hsa-miR-499-5p
NUDT3	hsa-miR-5586-5p
NUDT3	hsa-miR-4668-3p
NUDT3	hsa-miR-4311
NUDT3	hsa-miR-8844
NUDT3	hsa-miR-502-3p
NUDT3	hsa-miR-6337
NUDT3	hsa-miR-431-5p
NUDT3	hsa-miR-8848
NUDT3	hsa-miR-291-3p
NUDT3	hsa-miR-148b-3p
NUDT3	hsa-miR-656-3p
NUDT3	hsa-miR-6845-5p
NUDT3	hsa-miR-2322-5p
NUDT3	hsa-miR-3190-5p
NUDT3	hsa-miR-7166-3p
NUDT3	hsa-miR-4762-5p
NUDT3	hsa-miR-149-5p
NUDT3	hsa-miR-216-3p
NUDT3	hsa-miR-6804-5p
NUDT3	hsa-miR-1787
NUDT3	hsa-miR-642a-5p
NUDT3	hsa-miR-216b-3p
NUDT3	hsa-miR-211-5p
NUDT3	hsa-miR-3057-3p
NUDT3	hsa-miR-4498
NUDT3	hsa-miR-3577-5p
NUDT3	hsa-miR-4652-3p
NUDT3	hsa-miR-381-3p
NUDT3	hsa-miR-4714-5p
NUDT3	hsa-miR-7844-5p

NUDT3	hsa-miR-187-3p
NUDT3	hsa-miR-33-3p
NUDT3	hsa-miR-6089
NUDT3	hsa-miR-3664-5p
NUDT3	hsa-miR-556-5p
NUDT3	hsa-miR-363-3p
NUDT3	hsa-miR-200a-5p
NUDT3	hsa-miR-7246-5p
NUDT3	hsa-miR-363-3p
NUDT3	hsa-miR-3086-3p
NUDT3	hsa-miR-1273g-3p
NUDT3	hsa-miR-6864-3p
NUDT3	hsa-miR-548ae-3p
NUDT3	hsa-miR-497-5p
NUDT3	hsa-miR-2285m
NUDT3	hsa-miR-6751-3p
NUDT3	hsa-miR-299-3p
NUDT3	hsa-miR-6120-5p
NUDT3	hsa-miR-3617-3p
NUDT3	hsa-miR-936
NUDT3	hsa-miR-548d-3p
NUDT3	hsa-miR-1265
NUDT3	hsa-miR-5093
NUDT3	hsa-miR-382-5p
NUDT3	hsa-miR-342-3p
NUDT3	hsa-miR-6774-5p
NUDT3	hsa-miR-202-3p
NUDT3	hsa-let-7-3p
NUDT3	hsa-miR-7244-5p
NUDT3	hsa-miR-4731-3p
NUDT3	hsa-miR-486-3p
NUDT3	hsa-miR-1554
NUDT3	hsa-miR-4758-3p
NUDT3	hsa-miR-4478
NUDT3	hsa-miR-7203-5p
NUDT3	hsa-miR-4689
NUDT3	hsa-miR-6547-5p
NUDT3	hsa-miR-698-3p
NUDT3	hsa-miR-3686
NUDT3	hsa-miR-5196-3p
NUDT3	hsa-miR-548j-5p
NUDT3	hsa-miR-1967
NUDT3	hsa-miR-4677-3p
NUDT3	hsa-miR-1574-3p
NUDT3	hsa-miR-892b
NUDT3	hsa-miR-548an

NUDT3	hsa-miR-3973
NUDT3	hsa-miR-1681
NUDT3	hsa-miR-548a-5p
NUDT3	hsa-miR-8822
NUDT3	hsa-miR-7852-3p
NUDT3	hsa-miR-1301-5p
NUDT3	hsa-miR-7370-5p
NUDT3	hsa-miR-92a-5p
NUDT3	hsa-miR-2365
NUDT3	hsa-miR-2117
NUDT3	hsa-miR-3682-3p
NUDT3	hsa-miR-4524a-5p
NUDT3	hsa-miR-7011-5p
NUDT3	hsa-miR-6084
NUDT3	hsa-miR-7249-5p
NUDT3	hsa-miR-7018-5p
NUDT3	hsa-miR-219a-5p
NUDT3	hsa-miR-3564
NUDT3	hsa-miR-548at-5p
NUDT3	hsa-miR-4735-5p
NUDT3	hsa-miR-3910
NUDT3	hsa-miR-2110
NUDT3	hsa-miR-466c-5p
NUDT3	hsa-miR-6603-3p
NUDT3	hsa-miR-6834-5p
NUDT3	hsa-miR-1573
NUDT3	hsa-miR-3075-3p
NUDT3	hsa-miR-222-5p
NUDT3	hsa-miR-1912-5p
NUDT3	hsa-miR-6989-5p
NUDT3	hsa-miR-1234-3p
NUDT3	hsa-miR-765
NUDT3	hsa-miR-6713-3p
NUDT3	hsa-miR-5004-3p
NUDT3	hsa-miR-449a-3p
NUDT3	hsa-miR-1275
NUDT3	hsa-miR-3663-5p
NUDT3	hsa-miR-505-3p
NUDT3	hsa-miR-4486
NUDT3	hsa-miR-4659a-3p
NUDT3	hsa-miR-513a
NUDT3	hsa-miR-1843b-5p
NUDT3	hsa-miR-7161-5p
NUDT3	hsa-miR-8857
NUDT3	hsa-miR-1766
NUDT3	hsa-miR-548a-3p

NUDT3	hsa-miR-1271-5p
NUDT3	hsa-miR-141-3p
NUDT3	hsa-miR-3158-5p
NUDT3	hsa-miR-5623-3p
NUDT3	hsa-miR-1468-3p
NUDT3	hsa-miR-7033-3p
NUDT3	hsa-miR-6829-3p
NUDT3	hsa-miR-584-5p
NUDT3	hsa-miR-4786-3p
NUDT3	hsa-miR-8071
NUDT3	hsa-miR-4281
NUDT3	hsa-miR-369-3p
NUDT3	hsa-miR-2384
NUDT3	hsa-miR-4436b-3p
NUDT3	hsa-miR-6344
NUDT3	hsa-miR-5113
NUDT3	hsa-miR-3928-5p
NUDT3	hsa-miR-196-2-3p
NUDT3	hsa-miR-8884
NUDT3	hsa-miR-3121-3p
NUDT3	hsa-miR-6780a-3p
NUDT3	hsa-miR-182-5p
NUDT3	hsa-miR-3925-3p
NUDT3	hsa-miR-4722-3p
NUDT3	hsa-miR-124-5p
NUDT3	hsa-miR-6881-3p
NUDT3	hsa-miR-347
NUDT3	hsa-miR-1190
NUDT3	hsa-miR-7092-3p
NUDT3	hsa-miR-5000-3p
NUDT3	hsa-miR-409a
NUDT3	hsa-miR-517-5p
NUDT3	hsa-miR-1288
NUDT3	hsa-miR-7028-5p
NUDT3	hsa-miR-7031-3p
NUDT3	hsa-miR-207
NUDT3	hsa-miR-450b
NUDT3	hsa-miR-4736
NUDT3	hsa-miR-10b-3p
NUDT3	hsa-miR-1273h-5p
NUDT3	hsa-miR-6558-5p
NUDT3	hsa-miR-4271
NUDT3	hsa-miR-323a-5p
NUDT3	hsa-miR-760
NUDT3	hsa-miR-7234-5p
NUDT3	hsa-miR-188-3p

NUDT3	hsa-miR-677
NUDT3	hsa-miR-1297
NUDT3	hsa-miR-2432
NUDT3	hsa-miR-7094b-2-5p
NUDT3	hsa-miR-7033-5p
NUDT3	hsa-miR-887-5p
NUDT3	hsa-miR-6323
NUDT3	hsa-miR-373-3p
NUDT3	hsa-miR-4326
NUDT3	hsa-miR-7157-5p
NUDT3	hsa-miR-2285n
NUDT3	hsa-miR-1251-3p
NUDT3	hsa-miR-25-3p
NUDT3	hsa-miR-1304-5p
NUDT3	hsa-miR-7262a-5p
NUDT3	hsa-miR-483-5p
NUDT3	hsa-miR-7000-3p
NUDT3	hsa-miR-1969
NUDT3	hsa-miR-455-3p.2
NUDT3	hsa-miR-3164
NUDT3	hsa-miR-126-3p
NUDT3	hsa-miR-2307
NUDT3	hsa-miR-466f-3p
NUDT3	hsa-miR-2328-5p
NUDT3	hsa-miR-351-3p
NUDT3	hsa-miR-4802-3p
NUDT3	hsa-miR-133a-5p
NUDT3	hsa-miR-4462
NUDT3	hsa-miR-501-3p
NUDT3	hsa-miR-4531
NUDT3	hsa-miR-7334-3p
NUDT3	hsa-miR-520-5p
NUDT3	hsa-miR-6816-3p
NUDT3	hsa-miR-378b
NUDT3	hsa-miR-93-5p
NUDT3	hsa-miR-7343-3p
NUDT3	hsa-miR-326
NUDT3	hsa-miR-8886
NUDT3	hsa-miR-657
NUDT3	hsa-miR-6824-3p
NUDT3	hsa-miR-1837
NUDT3	hsa-miR-197-3p
NUDT3	hsa-miR-146
NUDT3	hsa-miR-206-3p
NUDT3	hsa-miR-7306-5p
NUDT3	hsa-miR-1712-5p

NUDT3	hsa-miR-200-3p
NUDT3	hsa-miR-6760-5p
NUDT3	hsa-miR-3616-3p
NUDT3	hsa-miR-6898-3p
NUDT3	hsa-miR-4433a-3p
NUDT3	hsa-miR-4744
NUDT3	hsa-miR-6785-5p
NUDT3	hsa-miR-7266-5p
NUDT3	hsa-miR-4797-3p
NUDT3	hsa-miR-223-3p
NUDT3	hsa-miR-7371d-5p
NUDT3	hsa-miR-513b-3p
NUDT3	hsa-let-7f-1-3p
NUDT3	hsa-miR-767
NUDT3	hsa-miR-5004-5p
NUDT3	hsa-miR-3614-5p
NUDT3	hsa-miR-30c-1-3p
NUDT3	hsa-miR-6614-3p
NUDT3	hsa-miR-6888-3p
NUDT3	hsa-miR-200a-3p
NUDT3	hsa-miR-1224-3p
NUDT3	hsa-miR-644a
NUDT3	hsa-miR-4301
NUDT3	hsa-miR-379-5p
NUDT3	hsa-miR-7365-3p
NUDT3	hsa-miR-4639-3p
NUDT3	hsa-miR-3544
NUDT3	hsa-miR-4695-5p
NUDT3	hsa-miR-641
NUDT3	hsa-miR-766-3p
NUDT3	hsa-miR-500a-5p
NUDT3	hsa-miR-6079
NUDT3	hsa-miR-5189-5p
NUDT3	hsa-miR-2440
NUDT3	hsa-miR-6545-5p
NUDT3	hsa-miR-7843-3p
NUDT3	hsa-miR-548f-5p
NUDT3	hsa-miR-452-3p
NUDT3	hsa-miR-890-3p
NUDT3	hsa-miR-6608-3p
NUDT3	hsa-miR-8085
NUDT3	hsa-miR-3561-5p
NUDT3	hsa-miR-769-5p
NUDT3	hsa-miR-1643b-5p
NUDT3	hsa-miR-1343-3p
NUDT3	hsa-miR-2331-5p

NUDT3	hsa-miR-7031-5p
NUDT3	hsa-miR-6513-3p
NUDT3	hsa-miR-513
NUDT3	hsa-miR-7274-3p
NUDT3	hsa-miR-2285af
NUDT3	hsa-miR-654-3p
NUDT3	hsa-miR-6750-5p
NUDT3	hsa-miR-6119-3p
NUDT3	hsa-miR-320b
NUDT3	hsa-miR-548w
NUDT3	hsa-miR-548t-5p
NUDT3	hsa-miR-4451
NUDT3	hsa-miR-4640-5p
NUDT3	hsa-miR-2895
NUDT3	hsa-miR-8078
NUDT3	hsa-miR-4421
NUDT3	hsa-miR-6846-5p
NUDT3	hsa-miR-199-1-5p
NUDT3	hsa-miR-1915-3p
NUDT3	hsa-miR-7280-3p
NUDT3	hsa-miR-211-3p
NUDT3	hsa-miR-939-5p
NUDT3	hsa-miR-3085
NUDT3	hsa-miR-107-5p
NUDT3	hsa-miR-548i
NUDT3	hsa-miR-3664-5p
NUDT3	hsa-miR-4786-5p
NUDT3	hsa-miR-2332
NUDT3	hsa-miR-107
NUDT3	hsa-miR-3611
NUDT3	hsa-miR-144-3p
NUDT3	hsa-miR-4700-5p
NUDT3	hsa-miR-4524-5p
NUDT3	hsa-miR-3575
NUDT3	hsa-miR-7111-3p
NUDT3	hsa-miR-3691-3p
NUDT3	hsa-miR-4443
NUDT3	hsa-miR-8806
NUDT3	hsa-miR-2285r
NUDT3	hsa-miR-454
NUDT3	hsa-miR-615-3p
NUDT3	hsa-miR-7652-3p
NUDT3	hsa-miR-26-5p
NUDT3	hsa-miR-676-3p
NUDT3	hsa-miR-34a-3p
NUDT3	hsa-miR-3919

NUDT3	hsa-miR-6996-3p
NUDT3	hsa-miR-892-5p
NUDT3	hsa-miR-3151-5p
NUDT3	hsa-miR-4728-3p
NUDT3	hsa-miR-7170-3p
NUDT3	hsa-miR-7035-5p
NUDT3	hsa-miR-650d
NUDT3	hsa-miR-1234-3p
NUDT3	hsa-miR-6481
NUDT3	hsa-miR-4424
NUDT3	hsa-miR-7373d-3p
NUDT3	hsa-miR-3145-3p
NUDT3	hsa-miR-3110-5p
NUDT3	hsa-miR-146a-3p
NUDT3	hsa-miR-1544b-5p
NUDT3	hsa-miR-6913-3p
NUDT3	hsa-miR-4678
NUDT3	hsa-miR-6635-5p
NUDT3	hsa-miR-3542
NUDT3	hsa-miR-28-3p
NUDT3	hsa-miR-4661-5p
NUDT3	hsa-miR-4433b-3p
NUDT3	hsa-miR-1668-3p
NUDT3	hsa-miR-554
NUDT3	hsa-miR-7318-5p
NUDT3	hsa-miR-3059-3p
NUDT3	hsa-miR-8064
NUDT3	hsa-miR-4277
NUDT3	hsa-miR-1896
NUDT3	hsa-miR-3680-3p
NUDT3	hsa-miR-6883-3p
NUDT3	hsa-miR-7007-5p
NUDT3	hsa-miR-4459
NUDT3	hsa-miR-154-5p
NUDT3	hsa-miR-7668-3p
NUDT3	hsa-miR-1307-3p
NUDT3	hsa-miR-25-3p
NUDT3	hsa-miR-193b-3p
NUDT3	hsa-miR-4699-3p
NUDT3	hsa-miR-4704-3p
NUDT3	hsa-miR-2413
NUDT3	hsa-miR-6868-3p
NUDT3	hsa-miR-6131
NUDT3	hsa-miR-101-3p
NUDT3	hsa-miR-7175-5p
NUDT3	hsa-miR-136-3p

NUDT3	hsa-miR-133a-3p.1
NUDT3	hsa-miR-7029-3p
NUDT3	hsa-miR-10b-5p
NUDT3	hsa-miR-7382-5p
NUDT3	hsa-miR-505-5p
NUDT3	hsa-miR-6722-3p
NUDT3	hsa-miR-1235
NUDT3	hsa-miR-2449
NUDT3	hsa-miR-33-5p
NUDT3	hsa-miR-294-3p
NUDT3	hsa-miR-1643a-5p
NUDT3	hsa-miR-488-3p
NUDT3	hsa-miR-7309-3p
NUDT3	hsa-miR-1237-5p
NUDT3	hsa-miR-7152-5p
NUDT3	hsa-miR-193b-5p
NUDT3	hsa-miR-6776-3p
NUDT3	hsa-miR-106
NUDT3	hsa-miR-8904b
NUDT3	hsa-miR-4791
NUDT3	hsa-miR-301-5p
NUDT3	hsa-miR-7302-5p
NUDT3	hsa-miR-7168-5p
NUDT3	hsa-miR-718
NUDT3	hsa-miR-195-5p
NUDT3	hsa-miR-7337-5p
NUDT3	hsa-miR-1255b-2-3p
NUDT3	hsa-miR-4747-5p
NUDT3	hsa-miR-154a
NUDT3	hsa-miR-137
NUDT3	hsa-miR-922
NUDT3	hsa-miR-1748
NUDT3	hsa-miR-2301
NUDT3	hsa-miR-7025-5p
NUDT3	hsa-miR-323-3p
NUDT3	hsa-miR-6760-3p
NUDT3	hsa-miR-3173-3p
NUDT3	hsa-miR-487b-5p
NUDT3	hsa-miR-1272
NUDT3	hsa-miR-500-5p
NUDT3	hsa-miR-3649
NUDT3	hsa-miR-7169-3p
NUDT3	hsa-miR-30e-3p
NUDT3	hsa-miR-7008-3p
NUDT3	hsa-miR-4999-5p
NUDT3	hsa-miR-7336-5p

NUDT3	hsa-miR-1584-3p
NUDT3	hsa-miR-1635
NUDT3	hsa-miR-1207-5p
NUDT3	hsa-miR-208-3p
NUDT3	hsa-miR-7328-5p
NUDT3	hsa-miR-3101-5p
NUDT3	hsa-miR-2276-3p
NUDT3	hsa-miR-3074-5p
NUDT3	hsa-miR-553
NUDT3	hsa-miR-556-3p
NUDT3	hsa-miR-421
NUDT3	hsa-miR-4723-3p
NUDT3	hsa-miR-6514-3p
NUDT3	hsa-miR-873-5p.2
NUDT3	hsa-miR-4717-3p
NUDT3	hsa-miR-939-5p
NUDT3	hsa-miR-144-3p
NUDT3	hsa-miR-7211-3p
NUDT3	hsa-miR-7013-5p
NUDT3	hsa-miR-2904
NUDT3	hsa-miR-599
NUDT3	hsa-miR-124a-5p
NUDT3	hsa-miR-504
NUDT3	hsa-miR-29b-1-5p
NUDT3	hsa-miR-3105-5p
NUDT3	hsa-miR-8054
NUDT3	hsa-miR-3092-3p
NUDT3	hsa-miR-7290-3p
NUDT3	hsa-miR-548ay-5p
NUDT3	hsa-miR-6769b-3p
NUDT3	hsa-miR-6804-3p
NUDT3	hsa-miR-6806-3p
NUDT3	hsa-miR-8113
NUDT3	hsa-miR-199b-2-5p
NUDT3	hsa-miR-4419b
NUDT3	hsa-miR-204-5p
NUDT3	hsa-miR-873-5p
NUDT3	hsa-miR-9769-5p
NUDT3	hsa-miR-3942-5p
NUDT3	hsa-miR-92a-2-5p
NUDT3	hsa-miR-2469
NUDT3	hsa-miR-483-3p
NUDT3	hsa-miR-5700
NUDT3	hsa-miR-3130-5p
NUDT3	hsa-miR-6505-3p
NUDT3	hsa-miR-7047-5p

NUDT3	hsa-miR-592
NUDT3	hsa-miR-212-3p
NUDT3	hsa-miR-22-3p
NUDT3	hsa-miR-7297-3p
NUDT3	hsa-miR-6516
NUDT3	hsa-miR-4446-5p
NUDT3	hsa-miR-3938
NUDT3	hsa-miR-30ad-3p
NUDT3	hsa-miR-6964-3p
NUDT3	hsa-miR-7201-3p
NUDT3	hsa-miR-4753-3p
NUDT3	hsa-miR-520
NUDT3	hsa-miR-7663-5p
NUDT3	hsa-miR-3155a
NUDT3	hsa-miR-139-5p
NUDT3	hsa-miR-5586-3p
NUDT3	hsa-miR-6602-5p
NUDT3	hsa-miR-1264-3p
NUDT3	hsa-miR-4653-5p
NUDT3	hsa-miR-1288-3p
NUDT3	hsa-miR-5627-5p
NUDT3	hsa-miR-18
NUDT3	hsa-miR-302c-5p
NUDT3	hsa-miR-7172-5p
NUDT3	hsa-miR-6795-3p
NUDT3	hsa-miR-7293-5p
NUDT3	hsa-miR-6789-3p
NUDT3	hsa-miR-6948-5p
NUDT3	hsa-miR-1233-5p
NUDT3	hsa-miR-7398b-3p
NUDT3	hsa-miR-7118-3p
NUDT3	hsa-miR-466l-3p
NUDT3	hsa-miR-4260
NUDT3	hsa-miR-3569-5p
NUDT3	hsa-miR-1631
NUDT3	hsa-miR-6721-5p
NUDT3	hsa-miR-3080-3p
NUDT3	hsa-miR-1255b-5p
NUDT3	hsa-miR-7184-5p
NUDT3	hsa-miR-9-5p
NUDT3	hsa-miR-7296-3p
NUDT3	hsa-miR-1228-3p
NUDT3	hsa-miR-550-5p
NUDT3	hsa-miR-7216-5p
NUDT3	hsa-miR-330-3p
NUDT3	hsa-miR-4529-5p

NUDT3	hsa-miR-4490
NUDT3	hsa-miR-3103-5p
NUDT3	hsa-miR-3182
NUDT3	hsa-miR-4650-3p
NUDT3	hsa-miR-4251
NUDT3	hsa-miR-1572
NUDT3	hsa-miR-6356
NUDT3	hsa-miR-3529-3p
NUDT3	hsa-miR-3130-3p
NUDT3	hsa-miR-543-3p
NUDT3	hsa-miR-5685
NUDT3	hsa-miR-548l
NUDT3	hsa-miR-8864
NUDT3	hsa-miR-4782-5p
NUDT3	hsa-miR-545-3p
NUDT3	hsa-miR-7395-5p
NUDT3	hsa-miR-7066-3p
NUDT3	hsa-miR-411-3p
NUDT3	hsa-miR-518
NUDT3	hsa-miR-548ac
NUDT3	hsa-miR-3134
NUDT3	hsa-miR-1294
NUDT3	hsa-miR-548au-5p
NUDT3	hsa-miR-7354-3p
NUDT3	hsa-miR-6801-5p
NUDT3	hsa-miR-143-3p
NUDT3	hsa-miR-1825
NUDT3	hsa-miR-7374c-3p
NUDT3	hsa-miR-4667
NUDT3	hsa-miR-202-3p
NUDT3	hsa-miR-7109-3p
NUDT3	hsa-miR-4495
NUDT3	hsa-miR-2288
NUDT3	hsa-miR-1955-3p
NUDT3	hsa-miR-5695
NUDT3	hsa-miR-3151-3p
NUDT3	hsa-miR-371b-5p
NUDT3	hsa-miR-30b-3p
NUDT3	hsa-miR-6786-3p
NUDT3	hsa-miR-4650
NUDT3	hsa-miR-1281
NUDT3	hsa-miR-7260-5p
NUDT3	hsa-miR-7651-3p
NUDT3	hsa-miR-186-5p
NUDT3	hsa-miR-2456
NUDT3	hsa-miR-1188-3p

NUDT3	hsa-miR-4676-5p
NUDT3	hsa-miR-5087
NUDT3	hsa-miR-342-5p
NUDT3	hsa-miR-7152-3p
NUDT3	hsa-miR-6894-5p
NUDT3	hsa-miR-1963
NUDT3	hsa-miR-8800
NUDT3	hsa-miR-802
NUDT3	hsa-miR-3682-5p
NUDT3	hsa-miR-7192-5p
NUDT3	hsa-miR-7214-3p
NUDT3	hsa-miR-7851-3p
NUDT3	hsa-miR-7386a-3-3p
NUDT3	hsa-miR-1707
NUDT3	hsa-miR-6325
NUDT3	hsa-miR-365-5p
NUDT3	hsa-miR-1292-3p
NUDT3	hsa-miR-6976-5p
NUDT3	hsa-miR-370-3p
NUDT3	hsa-miR-6880-5p
NUDT3	hsa-miR-1249-3p
NUDT3	hsa-miR-3127-5p
NUDT3	hsa-miR-181b-2-3p
NUDT3	hsa-miR-548u
NUDT3	hsa-miR-6861-5p
NUDT3	hsa-miR-540-5p
NUDT3	hsa-miR-5691
NUDT3	hsa-miR-500b-3p
NUDT3	hsa-miR-4686
NUDT3	hsa-miR-5106
NUDT3	hsa-miR-6873-5p
NUDT3	hsa-miR-7679-3p
NUDT3	hsa-miR-7064-5p
NUDT3	hsa-miR-30-3p
NUDT3	hsa-miR-7515
NUDT3	hsa-miR-8812
NUDT3	hsa-miR-652-5p
NUDT3	hsa-miR-2116-3p
NUDT3	hsa-miR-4300
NUDT3	hsa-miR-450a
NUDT3	hsa-miR-605-5p
NUDT3	hsa-miR-626
NUDT3	hsa-miR-6660-3p
NUDT3	hsa-miR-6639-5p
NUDT3	hsa-miR-1225-3p
NUDT3	hsa-miR-567

NUDT3	hsa-miR-7677-5p
NUDT3	hsa-miR-26-2-3p
NUDT3	hsa-miR-4667-3p
NUDT3	hsa-miR-4726-3p
NUDT3	hsa-miR-663a
NUDT3	hsa-miR-142-5p
NUDT3	hsa-miR-3586-3p
NUDT3	hsa-miR-2893
NUDT3	hsa-miR-3620-3p
NUDT3	hsa-miR-6554-3p
NUDT3	hsa-miR-298
NUDT3	hsa-miR-6887-3p
NUDT3	hsa-miR-8057
NUDT3	hsa-miR-1470
NUDT3	hsa-miR-548aef
NUDT3	hsa-miR-7366-3p
NUDT3	hsa-miR-518d-5p
NUDT3	hsa-miR-155-5p
NUDT3	hsa-miR-6851-5p
NUDT3	hsa-miR-2285s
NUDT3	hsa-miR-625-5p
NUDT3	hsa-miR-6501-5p
NUDT3	hsa-miR-3587
NUDT3	hsa-miR-4457
NUDT3	hsa-miR-1762
NUDT3	hsa-miR-3064-5p
NUDT3	hsa-miR-2284ab
NUDT3	hsa-miR-103-2-5p
NUDT3	hsa-miR-424-5p
NUDT3	hsa-miR-7355-3p
NUDT3	hsa-miR-133
NUDT3	hsa-miR-6919-5p
NUDT3	hsa-miR-4533
NUDT3	hsa-miR-4294
NUDT3	hsa-miR-6742-5p
NUDT3	hsa-miR-147a
NUDT3	hsa-miR-7032-5p
NUDT3	hsa-miR-4672
NUDT3	hsa-miR-4745-5p
NUDT3	hsa-miR-466-5p
NUDT3	hsa-miR-122-5p
NUDT3	hsa-miR-4302
NUDT3	hsa-miR-6733-3p
NUDT3	hsa-miR-8888
NUDT3	hsa-miR-575
NUDT3	hsa-miR-4293

NUDT3	hsa-miR-499b-5p
NUDT3	hsa-miR-450a-2-3p
NUDT3	hsa-miR-7272-5p
NUDT3	hsa-miR-5589-3p
NUDT3	hsa-miR-3956
NUDT3	hsa-miR-6739-3p
NUDT3	hsa-miR-7371a-5p
NUDT3	hsa-miR-6911-5p
NUDT3	hsa-miR-1541
NUDT3	hsa-miR-6981-5p
NUDT3	hsa-miR-1301
NUDT3	hsa-miR-664
NUDT3	hsa-miR-5572
NUDT3	hsa-miR-7372-5p
NUDT3	hsa-miR-7173-5p
NUDT3	hsa-miR-1245-3p
NUDT3	hsa-miR-602
NUDT3	hsa-miR-3151
NUDT3	hsa-miR-940
NUDT3	hsa-miR-2483-5p
NUDT3	hsa-miR-6779-3p
NUDT3	hsa-miR-7398n-3p
NUDT3	hsa-miR-2917
NUDT3	hsa-miR-674-3p
NUDT3	hsa-miR-3606-3p
NUDT3	hsa-miR-665-3p
NUDT3	hsa-miR-7006-3p
NUDT3	hsa-miR-1943-3p
NUDT3	hsa-miR-409-3p
NUDT3	hsa-miR-6775-3p
NUDT3	hsa-miR-769-3p
NUDT3	hsa-miR-1745
NUDT3	hsa-miR-125
NUDT3	hsa-miR-296-5p
NUDT3	hsa-miR-4254
NUDT3	hsa-miR-122-3p
NUDT3	hsa-miR-4795-3p
NUDT3	hsa-miR-6883-5p
NUDT3	hsa-miR-423-5p
NUDT3	hsa-miR-4316
NUDT3	hsa-miR-7278-3p
NUDT3	hsa-miR-622
NUDT3	hsa-miR-3977
NUDT3	hsa-miR-663a
NUDT3	hsa-miR-544-3p
NUDT3	hsa-miR-6748-3p

NUDT3	hsa-miR-1747-3p
NUDT3	hsa-miR-7439-3p
NUDT3	hsa-miR-875-5p
NUDT3	hsa-miR-1715-5p
NUDT3	hsa-miR-2326
NUDT3	hsa-miR-590-5p
NUDT3	hsa-miR-512-3p
NUDT3	hsa-miR-4794
NUDT3	hsa-miR-1778
NUDT3	hsa-miR-98-5p
NUDT3	hsa-miR-5100
NUDT3	hsa-miR-510-5p
NUDT3	hsa-miR-6520
NUDT3	hsa-miR-7208-3p
NUDT3	hsa-miR-1252-5p
NUDT3	hsa-miR-2465
NUDT3	hsa-miR-548z
NUDT3	hsa-miR-1180-5p
NUDT3	hsa-miR-7369-5p
NUDT3	hsa-miR-548g-5p
NUDT3	hsa-miR-590-3p
NUDT3	hsa-miR-7206-3p
NUDT3	hsa-miR-6809-5p
NUDT3	hsa-miR-548ap-3p
NUDT3	hsa-miR-6738-3p
NUDT3	hsa-miR-3689e
NUDT3	hsa-miR-23-3p
NUDT3	hsa-miR-6990-5p
NUDT3	hsa-miR-7680-3p
NUDT3	hsa-miR-216-5p
NUDT3	hsa-miR-634
NUDT3	hsa-miR-3431
NUDT3	hsa-miR-8082
NUDT3	hsa-miR-6511a-5p
NUDT3	hsa-miR-2358
NUDT3	hsa-miR-3972
NUDT3	hsa-miR-4657
NUDT3	hsa-miR-6746-5p
NUDT3	hsa-miR-7385e-3p
NUDT3	hsa-miR-297
NUDT3	hsa-miR-4685-5p
NUDT3	hsa-miR-6855-5p
NUDT3	hsa-miR-7070-5p
NUDT3	hsa-miR-8840
NUDT3	hsa-miR-99-3p
NUDT3	hsa-miR-4521

NUDT3	hsa-miR-6867-5p
NUDT3	hsa-miR-4801
NUDT3	hsa-miR-7200-3p
NUDT3	hsa-miR-8801
NUDT3	hsa-miR-23c
NUDT3	hsa-miR-4266
NUDT3	hsa-miR-6903-5p
NUDT3	hsa-let-7a-3p
NUDT3	hsa-miR-548bb-3p
NUDT3	hsa-miR-7396-3p
NUDT3	hsa-miR-216a-5p
NUDT3	hsa-miR-4796-5p
NUDT3	hsa-miR-588
NUDT3	hsa-miR-1270
NUDT3	hsa-miR-7654-3p
NUDT3	hsa-miR-504-3p
NUDT3	hsa-miR-7009-3p
NUDT3	hsa-miR-3156-3p
NUDT3	hsa-miR-2429
NUDT3	hsa-miR-4755-5p
NUDT3	hsa-miR-671-5p
NUDT3	hsa-miR-885
NUDT3	hsa-miR-514-3p
NUDT3	hsa-miR-631
NUDT3	hsa-miR-5195-5p
NUDT3	hsa-let-7a-2-3p
NUDT3	hsa-miR-6757-5p
NUDT3	hsa-miR-1293
NUDT3	hsa-miR-141-3p
NUDT3	hsa-miR-132-3p
NUDT3	hsa-miR-34b-5p
NUDT3	hsa-miR-6550-3p
NUDT3	hsa-miR-133-3p
NUDT3	hsa-miR-6544-5p
NUDT3	hsa-miR-135
NUDT3	hsa-miR-673-5p
NUDT3	hsa-miR-6822-5p
NUDT3	hsa-miR-1283
NUDT3	hsa-miR-4652-5p
NUDT3	hsa-miR-1197-3p
NUDT3	hsa-miR-2284j
NUDT3	hsa-miR-6734-3p
NUDT3	hsa-miR-7973
NUDT3	hsa-miR-636
NUDT3	hsa-miR-500a-3p
NUDT3	hsa-miR-7081-5p

NUDT3	hsa-miR-7025-3p
NUDT3	hsa-miR-3600
NUDT3	hsa-miR-518g-3p
NUDT3	hsa-miR-669j
NUDT3	hsa-miR-6792-3p
NUDT3	hsa-miR-4778-3p
NUDT3	hsa-miR-708-3p
NUDT3	hsa-miR-7368-5p
NUDT3	hsa-miR-27b-5p
NUDT3	hsa-miR-4447
NUDT3	hsa-miR-6777-3p
NUDT3	hsa-miR-2285u
NUDT3	hsa-miR-6776-5p
NUDT3	hsa-miR-3619-3p
NUDT3	hsa-miR-5681b
NUDT3	hsa-miR-140-3p.2
NUDT3	hsa-miR-1178-3p
NUDT3	hsa-miR-7082-3p
NUDT3	hsa-miR-1253
NUDT3	hsa-miR-6998-3p
NUDT3	hsa-miR-1914-5p
NUDT3	hsa-miR-1841
NUDT3	hsa-miR-8793
NUDT3	hsa-miR-5581-5p
NUDT3	hsa-miR-6762-5p
NUDT3	hsa-miR-6882-3p
NUDT3	hsa-miR-30a-3p
NUDT3	hsa-miR-6869-5p
NUDT3	hsa-miR-516b
NUDT3	hsa-miR-743b-5p
NUDT3	hsa-miR-6680-3p
NUDT3	hsa-miR-6586-5p
NUDT3	hsa-miR-883ab-3p
NUDT3	hsa-miR-520g-3p
NUDT3	hsa-miR-621
NUDT3	hsa-miR-4257
NUDT3	hsa-miR-2342
NUDT3	hsa-miR-2284d
NUDT3	hsa-miR-6716-5p
NUDT3	hsa-miR-466mo-3p
NUDT3	hsa-miR-7075-5p
NUDT3	hsa-miR-548c-3p
NUDT3	hsa-miR-7398-3p
NUDT3	hsa-miR-4795-5p
NUDT3	hsa-miR-4273
NUDT3	hsa-miR-6864-5p

NUDT3	hsa-miR-8843
NUDT3	hsa-miR-1911-3p
NUDT3	hsa-miR-8087
NUDT3	hsa-miR-3091-3p
NUDT3	hsa-miR-7004-3p
NUDT3	hsa-miR-7221-3p
NUDT3	hsa-miR-4314
NUDT3	hsa-miR-541-3p
NUDT3	hsa-miR-3121-3p
NUDT3	hsa-miR-1910-3p
NUDT3	hsa-miR-510-3p
NUDT3	hsa-miR-3551-5p
NUDT3	hsa-miR-7222-5p
NUDT3	hsa-miR-3653-3p
NUDT3	hsa-miR-944
NUDT3	hsa-miR-7062-5p
NUDT3	hsa-miR-3167-3p
NUDT3	hsa-miR-608
NUDT3	hsa-miR-7263-3p
NUDT3	hsa-miR-2421
NUDT3	hsa-miR-1247-3p
NUDT3	hsa-miR-1904
NUDT3	hsa-miR-2285ad
NUDT3	hsa-miR-4481
NUDT3	hsa-miR-7312-3p
NUDT3	hsa-miR-665
NUDT3	hsa-miR-1233-3p
NUDT3	hsa-miR-7041-5p
NUDT3	hsa-miR-197-5p
NUDT3	hsa-miR-891a-3p
NUDT3	hsa-miR-7386i-3p
NUDT3	hsa-miR-6652-5p
NUDT3	hsa-miR-1540-5p
NUDT3	hsa-miR-7a-1-3p
NUDT3	hsa-miR-6501-3p
NUDT3	hsa-miR-7325-5p
NUDT3	hsa-miR-3976
NUDT3	hsa-miR-3088-5p
NUDT3	hsa-miR-3106-3p
NUDT3	hsa-miR-197-3p
NUDT3	hsa-miR-551b-5p
NUDT3	hsa-miR-570-3p
NUDT3	hsa-miR-660-5p
NUDT3	hsa-miR-196-3p
NUDT3	hsa-miR-3920
NUDT3	hsa-miR-6629-5p

NUDT3	hsa-miR-4469
NUDT3	hsa-miR-6126
NUDT3	hsa-miR-674-5p
NUDT3	hsa-miR-221-3p
NUDT3	hsa-miR-6081
NUDT3	hsa-miR-6087
NUDT3	hsa-miR-8871
NUDT3	hsa-miR-1903
NUDT3	hsa-miR-555
NUDT3	hsa-miR-190a-3p
NUDT3	hsa-miR-2306
NUDT3	hsa-miR-4468
NUDT3	hsa-miR-514a-3p
NUDT3	hsa-miR-210-5p
NUDT3	hsa-miR-3923
NUDT3	hsa-miR-557
NUDT3	hsa-miR-6510-5p
NUDT3	hsa-miR-3135b
NUDT3	hsa-miR-4759
NUDT3	hsa-miR-6770-5p
NUDT3	hsa-miR-2461-3p
NUDT3	hsa-miR-942
NUDT3	hsa-miR-1224
NUDT3	hsa-miR-6615-3p
NUDT3	hsa-miR-7241-5p
NUDT3	hsa-miR-892a
NUDT3	hsa-miR-7705
NUDT3	hsa-miR-4264
NUDT3	hsa-miR-409-5p
NUDT3	hsa-miR-485-5p
NUDT3	hsa-miR-7681-3p
NUDT3	hsa-miR-9-3-3p
NUDT3	hsa-miR-2284b
NUDT3	hsa-miR-376b-5p
NUDT3	hsa-miR-6334
NUDT3	hsa-miR-5618-5p
NUDT3	hsa-miR-6788-5p
NUDT3	hsa-miR-607
NUDT3	hsa-miR-7665-5p
NUDT3	hsa-miR-6802-5p
NUDT3	hsa-miR-6071
NUDT3	hsa-miR-18-3p
NUDT3	hsa-miR-1946a
NUDT3	hsa-miR-872
NUDT3	hsa-miR-1622
NUDT3	hsa-miR-3058-3p

NUDT3	hsa-miR-7176-3p
NUDT3	hsa-miR-1912
NUDT3	hsa-miR-7374-5p
NUDT3	hsa-miR-6791-3p
NUDT3	hsa-miR-7236-5p
NUDT3	hsa-miR-1238-5p
NUDT3	hsa-miR-136-5p
NUDT3	hsa-miR-6997-5p
NUDT3	hsa-miR-760-3p
NUDT3	hsa-miR-7386j-5p
NUDT3	hsa-miR-499a-3p
NUDT3	hsa-miR-7392-3p
NUDT3	hsa-miR-7468-5p
NUDT3	hsa-miR-466n-3p
NUDT3	hsa-miR-3692-5p
NUDT3	hsa-miR-510
NUDT3	hsa-miR-3193
NUDT3	hsa-miR-8803
NUDT3	hsa-miR-6499-3p
NUDT3	hsa-miR-761
NUDT3	hsa-miR-3065-5p
NUDT3	hsa-miR-4324
NUDT3	hsa-miR-3667-3p
NUDT3	hsa-miR-6998-5p
NUDT3	hsa-miR-6829-5p
NUDT3	hsa-miR-6408
NUDT3	hsa-miR-2424
NUDT3	hsa-miR-548
NUDT3	hsa-miR-4263
NUDT3	hsa-miR-3140-5p
NUDT3	hsa-miR-350-3p
NUDT3	hsa-miR-1805-5p
NUDT3	hsa-miR-449-5p
NUDT3	hsa-miR-1968-3p
NUDT3	hsa-miR-2053
NUDT3	hsa-miR-1226-3p
NUDT3	hsa-miR-3612
NUDT3	hsa-miR-7244-3p
NUDT3	hsa-miR-7047-3p
NUDT3	hsa-miR-7977
NUDT3	hsa-miR-1546-5p
NUDT3	hsa-miR-5680
NUDT3	hsa-miR-513c-5p
NUDT3	hsa-miR-877-3p
NUDT3	hsa-miR-548ah-5p
NUDT3	hsa-miR-421-3p

NUDT3	hsa-miR-625-5p
NUDT3	hsa-miR-2450a
NUDT3	hsa-miR-1249-5p
NUDT3	hsa-miR-2382-5p
NUDT3	hsa-miR-16-5p
NUDT3	hsa-miR-6420
NUDT3	hsa-miR-2885
NUDT3	hsa-miR-574-3p
NUDT3	hsa-miR-3135a
NUDT3	hsa-miR-606
NUDT3	hsa-miR-4732-3p
NUDT3	hsa-miR-3128
NUDT3	hsa-miR-6918-5p
NUDT3	hsa-miR-206-5p
NUDT3	hsa-miR-322-5p
NUDT3	hsa-miR-5588-3p
NUDT3	hsa-miR-624-5p
NUDT3	hsa-miR-3928-3p
NUDT3	hsa-miR-2394
NUDT3	hsa-miR-4519
NUDT3	hsa-miR-7249-3p
NUDT3	hsa-miR-495-3p
NUDT3	hsa-miR-2888
NUDT3	hsa-miR-532-5p
NUDT3	hsa-miR-3922-5p
NUDT3	hsa-miR-669c-3p
NUDT3	hsa-miR-582-5p
NUDT3	hsa-miR-7378-3p
NUDT3	hsa-miR-3152-5p
NUDT3	hsa-miR-6892-3p
NUDT3	hsa-miR-4749-3p
NUDT3	hsa-miR-2284-5p
NUDT3	hsa-miR-140-5p
NUDT3	hsa-miR-6796-5p
NUDT3	hsa-miR-7071-5p
NUDT3	hsa-miR-2368-5p
NUDT3	hsa-miR-4761-5p
NUDT3	hsa-miR-7385c-3p
NUDT3	hsa-miR-8100
NUDT3	hsa-miR-2286
NUDT3	hsa-miR-544a
NUDT3	hsa-miR-548m
NUDT3	hsa-miR-6693-3p
NUDT3	hsa-miR-3074-2-3p
NUDT3	hsa-miR-335-5p
NUDT3	hsa-miR-204-5p

NUDT3	hsa-miR-548ak
NUDT3	hsa-miR-20a-3p
NUDT3	hsa-miR-6927-3p
NUDT3	hsa-miR-506-3p
NUDT3	hsa-miR-2381
NUDT3	hsa-miR-1388-5p
NUDT3	hsa-miR-6975-5p
NUDT3	hsa-miR-2116-5p
NUDT3	hsa-miR-8108
NUDT3	hsa-miR-7685-3p
NUDT3	hsa-miR-8110
NUDT3	hsa-miR-2316
NUDT3	hsa-miR-383
NUDT3	hsa-let-7g-3p
NUDT3	hsa-miR-3670
NUDT3	hsa-miR-7039-5p
NUDT3	hsa-miR-7398n-5p
NUDT3	hsa-miR-2303
NUDT3	hsa-miR-6778-3p
NUDT3	hsa-miR-4291
NUDT3	hsa-miR-182-5p
NUDT3	hsa-miR-548i-3p
NUDT3	hsa-miR-5696
NUDT3	hsa-miR-151a-5p
NUDT3	hsa-miR-6949-3p
NUDT3	hsa-miR-3058-5p
NUDT3	hsa-miR-7168-3p
NUDT3	hsa-miR-361-5p
NUDT3	hsa-miR-3934-3p
NUDT3	hsa-miR-140-5p
NUDT3	hsa-miR-2370-3p
NUDT3	hsa-miR-3116
NUDT3	hsa-miR-2334
NUDT3	hsa-miR-8887
NUDT3	hsa-miR-3169
NUDT3	hsa-miR-8851
NUDT3	hsa-miR-4750-3p
NUDT3	hsa-miR-2278
NUDT3	hsa-miR-645
NUDT3	hsa-miR-96-3p
NUDT3	hsa-miR-519e-3p
NUDT3	hsa-miR-208a-3p
NUDT3	hsa-miR-323a-3p
NUDT3	hsa-miR-1236-3p
NUDT3	hsa-miR-7037-5p
NUDT3	hsa-miR-1468-5p

NUDT3	hsa-miR-496
NUDT3	hsa-miR-6764-5p
NUDT3	hsa-miR-3967
NUDT3	hsa-miR-3095-3p
NUDT3	hsa-miR-3138
NUDT3	hsa-miR-3915
NUDT3	hsa-miR-1808
NUDT3	hsa-miR-3184-3p
NUDT3	hsa-miR-514a-3p
NUDT3	hsa-miR-6715a-3p
NUDT3	hsa-miR-4768-3p
NUDT3	hsa-miR-18a-3p
NUDT3	hsa-miR-7375-3p
NUDT3	hsa-miR-2392
NUDT3	hsa-miR-128
NUDT3	hsa-miR-6814-5p
NUDT3	hsa-miR-6727-3p
NUDT3	hsa-miR-5589-5p
NUDT3	hsa-miR-6888-5p
NUDT3	hsa-miR-4423-3p
NUDT3	hsa-miR-298-5p
NUDT3	hsa-miR-6931-5p
NUDT3	hsa-miR-3688-3p
NUDT3	hsa-miR-4776-5p
NUDT3	hsa-miR-8824
NUDT3	hsa-miR-4659b-5p
NUDT3	hsa-miR-2356
NUDT3	hsa-miR-1684-5p
NUDT3	hsa-miR-1266-3p
NUDT3	hsa-miR-7252-3p
NUDT3	hsa-miR-6955-5p
NUDT3	hsa-miR-4682
NUDT3	hsa-miR-6735-5p
NUDT3	hsa-miR-204-3p
NUDT3	hsa-miR-6724-5p
NUDT3	hsa-miR-7661-5p
NUDT3	hsa-miR-8826
NUDT3	hsa-miR-7110-5p
NUDT3	hsa-miR-2489
NUDT3	hsa-miR-2324
NUDT3	hsa-miR-2417
NUDT3	hsa-miR-6973a-3p
NUDT3	hsa-miR-653-3p
NUDT3	hsa-miR-1273f
NUDT3	hsa-miR-7356-5p
NUDT3	hsa-miR-619-3p

NUDT3	hsa-miR-32-5p
NUDT3	hsa-miR-1608
NUDT3	hsa-miR-7478-5p
NUDT3	hsa-miR-374c-5p
NUDT3	hsa-miR-96-5p
NUDT3	hsa-miR-6923-3p
NUDT3	hsa-miR-8797
NUDT3	hsa-miR-6625-5p
NUDT3	hsa-miR-8846
NUDT3	hsa-miR-6769a-5p
NUDT3	hsa-miR-6549-3p
NUDT3	hsa-miR-7220-3p
NUDT3	hsa-miR-375-3p
NUDT3	hsa-miR-290b-3p
NUDT3	hsa-miR-8836
NUDT3	hsa-miR-6819-5p
NUDT3	hsa-miR-372
NUDT3	hsa-miR-154c
NUDT3	hsa-miR-138-3p
NUDT3	hsa-miR-451-3p
NUDT3	hsa-miR-6533
NUDT3	hsa-miR-551b-3p
NUDT3	hsa-miR-494-3p
NUDT3	hsa-miR-1200
NUDT3	hsa-miR-5787
NUDT3	hsa-miR-125-5p
NUDT3	hsa-miR-7440-5p
NUDT3	hsa-miR-449a
NUDT3	hsa-miR-648
NUDT3	hsa-miR-4485-5p
NUDT3	hsa-miR-328
NUDT3	hsa-miR-1323-5p
NUDT3	hsa-miR-3934-5p
NUDT3	hsa-miR-3202
NUDT3	hsa-miR-1956-5p
NUDT3	hsa-miR-7162-5p
NUDT3	hsa-miR-6500-3p
NUDT3	hsa-miR-4255
NUDT3	hsa-miR-383-5p
NUDT3	hsa-miR-6621-5p
NUDT3	hsa-miR-7863
NUDT3	hsa-miR-7861
NUDT3	hsa-miR-324
NUDT3	hsa-miR-6988-3p
NUDT3	hsa-miR-7155-5p
NUDT3	hsa-miR-3129-3p

NUDT3	hsa-miR-3688-5p
NUDT3	hsa-miR-2339
NUDT3	hsa-miR-7057-5p
NUDT3	hsa-miR-6815-3p
NUDT3	hsa-miR-130b-5p
NUDT3	hsa-miR-3093-3p
NUDT3	hsa-miR-548h-5p
NUDT3	hsa-miR-6870-3p
NUDT3	hsa-miR-330-3p
NUDT3	hsa-miR-4799-3p
NUDT3	hsa-miR-4290
NUDT3	hsa-miR-515
NUDT3	hsa-miR-2453
NUDT3	hsa-miR-6852-5p
NUDT3	hsa-miR-7107-5p
NUDT3	hsa-miR-2416
NUDT3	hsa-miR-4680-5p
NUDT3	hsa-miR-552
NUDT3	hsa-miR-409b
NUDT3	hsa-miR-8804
NUDT3	hsa-miR-367-3p
NUDT3	hsa-miR-516b-5p
NUDT3	hsa-miR-4420
NUDT3	hsa-miR-7253-5p
NUDT3	hsa-miR-7063-5p
NUDT3	hsa-miR-520-3p
NUDT3	hsa-miR-8850
NUDT3	hsa-miR-6754-5p
NUDT3	hsa-miR-433-5p
NUDT3	hsa-miR-6769b-5p
NUDT3	hsa-miR-370
NUDT3	hsa-miR-548az-3p
NUDT3	hsa-miR-4675
NUDT3	hsa-miR-466g
NUDT3	hsa-miR-8855
NUDT3	hsa-miR-2387
NUDT3	hsa-miR-6551-3p
NUDT3	hsa-miR-191-3p
NUDT3	hsa-miR-642b-5p
NUDT3	hsa-miR-881-3p
NUDT3	hsa-miR-2452
NUDT3	hsa-miR-6129
NUDT3	hsa-miR-5622-3p
NUDT3	hsa-miR-8893
NUDT3	hsa-miR-7242-5p
NUDT3	hsa-miR-4651

NUDT3	hsa-miR-183-5p.1
NUDT3	hsa-miR-7336-3p
NUDT3	hsa-miR-1343
NUDT3	hsa-miR-3176
NUDT3	hsa-miR-2285l
NUDT3	hsa-miR-2426
NUDT3	hsa-miR-7048-3p
NUDT3	hsa-miR-552-3p
NUDT3	hsa-miR-548b-3p
NUDT3	hsa-miR-615-5p
NUDT3	hsa-miR-6716-3p
NUDT3	hsa-miR-218-5p
NUDT3	hsa-miR-548f-3p
NUDT3	hsa-miR-4754
NUDT3	hsa-miR-4638-5p
NUDT3	hsa-miR-137-5p
NUDT3	hsa-miR-2447
NUDT3	hsa-miR-7154-3p
NUDT3	hsa-miR-2438
NUDT3	hsa-miR-7034-3p
NUDT3	hsa-miR-6765-3p
NUDT3	hsa-miR-1685-3p
NUDT3	hsa-miR-1947-5p
NUDT3	hsa-miR-650abc
NUDT3	hsa-miR-4646-3p
NUDT3	hsa-miR-7466-5p
NUDT3	hsa-miR-208-5p
NUDT3	hsa-miR-2379
NUDT3	hsa-miR-124-3p.1
NUDT3	hsa-miR-611
NUDT3	hsa-miR-5186
NUDT3	hsa-miR-1590
NUDT3	hsa-miR-8081
NUDT3	hsa-miR-6123
NUDT3	hsa-miR-6631-5p
NUDT3	hsa-miR-23b-3p
NUDT3	hsa-miR-619-5p
NUDT3	hsa-miR-1542-5p
NUDT3	hsa-miR-1619
NUDT3	hsa-miR-6840-3p
NUDT3	hsa-miR-509-3-5p
NUDT3	hsa-miR-885-5p
NUDT3	hsa-miR-2298
NUDT3	hsa-miR-7161-3p
NUDT3	hsa-miR-6827-5p
NUDT3	hsa-miR-651

NUDT3	hsa-miR-5114
NUDT3	hsa-miR-9500
NUDT3	hsa-miR-380
NUDT3	hsa-miR-466aep-5p
NUDT3	hsa-miR-15b-3p
NUDT3	hsa-miR-365a-5p
NUDT3	hsa-miR-759
NUDT3	hsa-miR-2284u
NUDT3	hsa-miR-1545a-3p
NUDT3	hsa-miR-3927-3p
NUDT3	hsa-miR-6165
NUDT3	hsa-miR-6759-3p
NUDT3	hsa-miR-183-5p.2
NUDT3	hsa-miR-8080
NUDT3	hsa-miR-7648-3p
NUDT3	hsa-miR-3179
NUDT3	hsa-miR-215
NUDT3	hsa-miR-3667-5p
NUDT3	hsa-miR-143-3p
NUDT3	hsa-miR-2441
NUDT3	hsa-miR-3060-5p
NUDT3	hsa-miR-216b
NUDT3	hsa-miR-1304-3p
NUDT3	hsa-miR-7383-3p
NUDT3	hsa-miR-2284aa
NUDT3	hsa-miR-7150
NUDT3	hsa-miR-7051-3p
NUDT3	hsa-miR-2305
NUDT3	hsa-miR-185-5p
NUDT3	hsa-miR-4520-2-3p
NUDT3	hsa-miR-494
NUDT3	hsa-miR-7165-5p
NUDT3	hsa-miR-5625-3p
NUDT3	hsa-miR-7286-5p
NUDT3	hsa-miR-7088-5p
NUDT3	hsa-miR-1239
NUDT3	hsa-miR-3613-3p
NUDT3	hsa-miR-4738-3p
NUDT3	hsa-miR-4515
NUDT3	hsa-miR-6579-3p
NUDT3	hsa-miR-7208-5p
NUDT3	hsa-miR-320-5p
NUDT3	hsa-miR-2355-3p
NUDT3	hsa-miR-7680-5p
NUDT3	hsa-miR-3907
NUDT3	hsa-miR-7976

NUDT3	hsa-miR-152
NUDT3	hsa-miR-541-3p
NUDT3	hsa-miR-763
NUDT3	hsa-miR-766-5p
NUDT3	hsa-miR-519e-3p
NUDT3	hsa-miR-5088-3p
NUDT3	hsa-miR-450a-1-3p
NUDT3	hsa-miR-7305-5p
NUDT3	hsa-miR-7847-3p
NUDT3	hsa-miR-362-5p
NUDT3	hsa-miR-653
NUDT3	hsa-miR-329a
NUDT3	hsa-miR-290b-5p
NUDT3	hsa-miR-4419a
NUDT3	hsa-miR-186-5p
NUDT3	hsa-miR-7371j-3p
NUDT3	hsa-miR-4723-5p
NUDT3	hsa-miR-3472
NUDT3	hsa-miR-2375
NUDT3	hsa-miR-25-5p
NUDT3	hsa-miR-5591-5p
NUDT3	hsa-miR-579
NUDT3	hsa-let-7
NUDT3	hsa-miR-633
NUDT3	hsa-miR-3617-5p
NUDT3	hsa-miR-3082-3p
NUDT3	hsa-miR-7257-3p
NUDT3	hsa-miR-8869
NUDT3	hsa-miR-2284r
NUDT3	hsa-miR-5698
NUDT3	hsa-miR-1185-5p
NUDT3	hsa-miR-2114-5p
NUDT3	hsa-miR-8818
NUDT3	hsa-miR-637
NUDT3	hsa-miR-7189-3p
NUDT3	hsa-miR-702-5p
NUDT3	hsa-miR-502b
NUDT3	hsa-miR-7386i-5p
NUDT3	hsa-miR-195-5p
NUDT3	hsa-miR-6927-5p
NUDT3	hsa-miR-6858-3p
NUDT3	hsa-miR-497-5p
NUDT3	hsa-miR-548ae-5p
NUDT3	hsa-miR-140-3p
NUDT3	hsa-miR-6077
NUDT3	hsa-miR-660-3p

NUDT3	hsa-miR-7002-5p
NUDT3	hsa-miR-6834-3p
NUDT3	hsa-miR-8890
NUDT3	hsa-miR-4435
NUDT3	hsa-miR-6939-5p
NUDT3	hsa-miR-7229-3p
NUDT3	hsa-miR-7978
NUDT3	hsa-miR-460-3p
NUDT3	hsa-miR-2131-5p
NUDT3	hsa-miR-7398r-3p
NUDT3	hsa-miR-509-5p
NUDT3	hsa-miR-2300-5p
NUDT3	hsa-miR-3921
NUDT3	hsa-miR-6984-3p
NUDT3	hsa-miR-1603
NUDT3	hsa-miR-135-5p
NUDT3	hsa-miR-2386
NUDT3	hsa-miR-6524
NUDT3	hsa-miR-8823
NUDT3	hsa-miR-383-5p.2
NUDT3	hsa-miR-5626-5p
NUDT3	hsa-miR-684
NUDT3	hsa-miR-3532-5p
NUDT3	hsa-miR-2284z
NUDT3	hsa-miR-6794-3p
NUDT3	hsa-miR-6800-5p
NUDT3	hsa-miR-188-5p
NUDT3	hsa-miR-1305
NUDT3	hsa-miR-7386m-5p
NUDT3	hsa-miR-522-3p
NUDT3	hsa-miR-3689a-5p
NUDT3	hsa-miR-2114-5p
NUDT3	hsa-miR-7655-5p
NUDT3	hsa-miR-8854
NUDT3	hsa-miR-3190-3p
NUDT3	hsa-miR-490-3p
NUDT3	hsa-miR-713
NUDT3	hsa-miR-181d-3p
NUDT3	hsa-miR-501-5p
NUDT3	hsa-miR-4772-5p
NUDT3	hsa-miR-7682-5p
NUDT3	hsa-miR-3690
NUDT3	hsa-miR-1230
NUDT3	hsa-miR-543
NUDT3	hsa-miR-6069
NUDT3	hsa-miR-7193-5p

NUDT3	hsa-miR-4739
NUDT3	hsa-miR-7442-5p
NUDT3	hsa-miR-1388-3p
NUDT3	hsa-miR-6517
NUDT3	hsa-miR-507b
NUDT3	hsa-miR-649
NUDT3	hsa-miR-548am-5p
NUDT3	hsa-miR-133ab
NUDT3	hsa-miR-184-5p
NUDT3	hsa-miR-4691-5p
NUDT3	hsa-miR-3132
NUDT3	hsa-miR-6593-5p
NUDT3	hsa-miR-483-3p
NUDT3	hsa-miR-7386e-3p
NUDT3	hsa-miR-1271-5p
NUDT3	hsa-miR-7349-3p
NUDT3	hsa-miR-1721
NUDT3	hsa-miR-23-5p
NUDT3	hsa-miR-185-5p
NUDT3	hsa-miR-7330-3p
NUDT3	hsa-miR-4312
NUDT3	hsa-miR-3065-3p
NUDT3	hsa-miR-6950-3p
NUDT3	hsa-miR-548y
NUDT3	hsa-miR-4536-5p
NUDT3	hsa-miR-7323-5p
NUDT3	hsa-miR-1736-3p
NUDT3	hsa-miR-16-5p
NUDT3	hsa-miR-498
NUDT3	hsa-miR-502-5p
NUDT3	hsa-miR-3072-5p
NUDT3	hsa-miR-616-3p
NUDT3	hsa-miR-34c-5p
NUDT3	hsa-miR-548j-3p
NUDT3	hsa-miR-5089-3p
NUDT3	hsa-miR-2285j
NUDT3	hsa-miR-6619-5p
NUDT3	hsa-miR-5132-5p
NUDT3	hsa-miR-2129
NUDT3	hsa-miR-4674
NUDT3	hsa-miR-9768-3p
NUDT3	hsa-miR-7386g-5p
NUDT3	hsa-miR-7671-5p
NUDT3	hsa-miR-5590-5p
NUDT3	hsa-miR-506-3p
NUDT3	hsa-miR-2405

NUDT3	hsa-miR-2285i
NUDT3	hsa-miR-6630-3p
NUDT3	hsa-miR-2364
NUDT3	hsa-miR-338-5p
NUDT3	hsa-miR-548n
NUDT3	hsa-miR-425-5p
NUDT3	hsa-miR-9-5p
NUDT3	hsa-miR-2437
NUDT3	hsa-miR-425
NUDT3	hsa-miR-8825
NUDT3	hsa-miR-7296-5p
NUDT3	hsa-miR-7467-3p
NUDT3	hsa-miR-200bc-3p
NUDT3	hsa-miR-8117
NUDT3	hsa-miR-3929
NUDT3	hsa-miR-7260-3p
NUDT3	hsa-miR-28-5p
NUDT3	hsa-miR-576-5p
NUDT3	hsa-miR-8107
NUDT3	hsa-let-7b-3p
NUDT3	hsa-miR-7162-3p
NUDT3	hsa-miR-16-3p
NUDT3	hsa-miR-2311
NUDT3	hsa-miR-3936
NUDT3	hsa-miR-939-3p
NUDT3	hsa-miR-451b
NUDT3	hsa-miR-2355-5p
NUDT3	hsa-miR-4694-3p
NUDT3	hsa-miR-548k
NUDT3	hsa-miR-892-3p
NUDT3	hsa-miR-7108-5p
NUDT3	hsa-miR-875
NUDT3	hsa-miR-7155-3p
NUDT3	hsa-miR-6858-5p
NUDT3	hsa-miR-1205
NUDT3	hsa-miR-6866-5p
NUDT3	hsa-miR-9a-3p
NUDT3	hsa-miR-3539
NUDT3	hsa-miR-7390-5p
NUDT3	hsa-miR-3194-5p
NUDT3	hsa-miR-7350-3p
NUDT3	hsa-let-7afk-3p
NUDT3	hsa-miR-7089-3p
NUDT3	hsa-miR-3062-3p
NUDT3	hsa-miR-577-3p
NUDT3	hsa-miR-182-3p

NUDT3	hsa-miR-1278
NUDT3	hsa-miR-1571
NUDT3	hsa-miR-4437
NUDT3	hsa-miR-873-3p
NUDT3	hsa-miR-23a-3p
NUDT3	hsa-miR-7386e-5p
NUDT3	hsa-miR-654-5p
NUDT3	hsa-miR-6994-5p
NUDT3	hsa-miR-7180-5p
NUDT3	hsa-miR-3689d
NUDT3	hsa-miR-146b-3p
NUDT3	hsa-miR-422
NUDT3	hsa-miR-4488
NUDT3	hsa-miR-6796-3p
NUDT3	hsa-miR-6902-5p
NUDT3	hsa-miR-670-5p
NUDT3	hsa-miR-378-3p
NUDT3	hsa-miR-7684-5p
NUDT3	hsa-miR-7398a-3p
NUDT3	hsa-miR-7291-3p
NUDT3	hsa-miR-7077-5p
NUDT3	hsa-miR-6995-5p
NUDT3	hsa-miR-7351-3p
NUDT3	hsa-miR-4666a-3p
NUDT3	hsa-miR-8808
NUDT3	hsa-miR-6747-3p
NUDT3	hsa-miR-6751-5p
NUDT3	hsa-miR-3127-5p
NUDT3	hsa-miR-3175
NUDT3	hsa-miR-376c
NUDT3	hsa-miR-8066
NUDT3	hsa-miR-8063
NUDT3	hsa-miR-340-5p
NUDT3	hsa-miR-7377-3p
NUDT3	hsa-miR-375
NUDT3	hsa-miR-4475
NUDT3	hsa-miR-3090-3p
NUDT3	hsa-miR-7352-3p
NUDT3	hsa-miR-1827
NUDT3	hsa-miR-200bc
NUDT3	hsa-miR-525-5p
NUDT3	hsa-miR-2320-5p
NUDT3	hsa-miR-344-5p
NUDT3	hsa-miR-548t-3p
NUDT3	hsa-miR-206
NUDT3	hsa-miR-6838-3p

NUDT3	hsa-miR-7183-3p
NUDT3	hsa-miR-5007-3p
NUDT3	hsa-miR-708-5p
NUDT3	hsa-miR-2393
NUDT3	hsa-miR-92-3p
NUDT3	hsa-miR-1981-5p
NUDT3	hsa-miR-6582-5p
NUDT3	hsa-miR-6124
NUDT3	hsa-miR-124-3p
NUDT3	hsa-miR-148-3p
NUDT3	hsa-miR-3653-5p
NUDT3	hsa-miR-6749-3p
NUDT3	hsa-miR-3105-3p
NUDT3	hsa-miR-6362
NUDT3	hsa-miR-3187-5p
NUDT3	hsa-miR-1-3p
NUDT3	hsa-miR-1548-5p
NUDT3	hsa-miR-7366-5p
NUDT3	hsa-miR-208a-5p
NUDT3	hsa-miR-6799-5p
NUDT3	hsa-miR-677-3p
NUDT3	hsa-miR-6416-3p
NUDT3	hsa-miR-8550
NUDT3	hsa-miR-6837-5p
NUDT3	hsa-miR-3557-5p
NUDT3	hsa-miR-1582
NUDT3	hsa-miR-2322-3p
NUDT3	hsa-miR-218-1-3p
NUDT3	hsa-miR-3689f
NUDT3	hsa-miR-6940-5p
NUDT3	hsa-miR-4418
NUDT3	hsa-miR-2338
NUDT3	hsa-miR-4704-5p
NUDT3	hsa-miR-577
NUDT3	hsa-miR-6503-3p
NUDT3	hsa-miR-548ah-3p
NUDT3	hsa-miR-6905-3p
NUDT3	hsa-miR-3574
NUDT3	hsa-miR-8898
NUDT3	hsa-miR-33
NUDT3	hsa-miR-3149
NUDT3	hsa-miR-7373b-3p
NUDT3	hsa-miR-4679
NUDT3	hsa-miR-4528
NUDT3	hsa-miR-336-3p
NUDT3	hsa-miR-876-3p

NUDT3	hsa-miR-1625-3p
NUDT3	hsa-miR-6758-3p
NUDT3	hsa-miR-7-5p
NUDT3	hsa-miR-3909
NUDT3	hsa-miR-2470
NUDT3	hsa-miR-2292
NUDT3	hsa-miR-6996-5p
NUDT3	hsa-miR-1271-3p
NUDT3	hsa-miR-7203-3p
NUDT3	hsa-miR-7091-3p
NUDT3	hsa-miR-7012-5p
NUDT3	hsa-miR-361-5p
NUDT3	hsa-miR-487-5p
NUDT3	hsa-miR-5089-5p
NUDT3	hsa-miR-4287
NUDT3	hsa-miR-3533
NUDT3	hsa-miR-7180
NUDT3	hsa-miR-6772-3p
NUDT3	hsa-miR-7266-3p
NUDT3	hsa-miR-7191-5p
NUDT3	hsa-miR-450b-5p
NUDT3	hsa-miR-219-3p
NUDT3	hsa-miR-6958-3p
NUDT3	hsa-miR-7220-5p
NUDT3	hsa-miR-129-5p
NUDT3	hsa-miR-7676-5p
NUDT3	hsa-miR-8875
NUDT3	hsa-miR-6918-3p
NUDT3	hsa-miR-4267
NUDT3	hsa-miR-6819-3p
NUDT3	hsa-miR-7348-5p
NUDT3	hsa-miR-3112-3p
NUDT3	hsa-miR-8076
NUDT3	hsa-miR-2344
NUDT3	hsa-miR-127-5p
NUDT3	hsa-miR-210
NUDT3	hsa-miR-196
NUDT3	hsa-miR-561-3p
NUDT3	hsa-miR-339-5p
NUDT3	hsa-miR-5003-5p
NUDT3	hsa-miR-7193-3p
NUDT3	hsa-miR-100-3p
NUDT3	hsa-miR-4303
NUDT3	hsa-miR-1814
NUDT3	hsa-miR-6613-3p
NUDT3	hsa-miR-6762-3p

NUDT3	hsa-miR-7013-3p
NUDT3	hsa-miR-6934-5p
NUDT3	hsa-miR-3157-5p
NUDT3	hsa-miR-617
NUDT3	hsa-miR-423-3p
NUDT3	hsa-miR-7354-5p
NUDT3	hsa-miR-7672-5p
NUDT3	hsa-miR-1460
NUDT3	hsa-miR-3082-5p
NUDT3	hsa-miR-7389-5p
NUDT3	hsa-miR-7678-3p
NUDT3	hsa-miR-4632-5p
NUDT3	hsa-miR-6818-5p
NUDT3	hsa-miR-6842-5p
NUDT3	hsa-miR-6983-5p
NUDT3	hsa-miR-3136-3p
NUDT3	hsa-miR-6880-3p
NUDT3	hsa-miR-200ab-5p
NUDT3	hsa-miR-488-3p
NUDT3	hsa-miR-3584-3p
NUDT3	hsa-miR-1811
NUDT3	hsa-miR-1229-3p
NUDT3	hsa-miR-3102-3p
NUDT3	hsa-miR-342-3p
NUDT3	hsa-miR-454-5p
NUDT3	hsa-miR-2488
NUDT3	hsa-miR-675-3p
NUDT3	hsa-miR-181b-1-3p
NUDT3	hsa-miR-6773-5p
NUDT3	hsa-miR-2909
NUDT3	hsa-miR-7188-3p
NUDT3	hsa-miR-4482-5p
NUDT3	hsa-miR-4668-5p
NUDT3	hsa-miR-516
NUDT3	hsa-miR-192-5p
NUDT3	hsa-miR-212-5p
NUDT3	hsa-miR-8849
NUDT3	hsa-miR-519e-5p
NUDT3	hsa-miR-4645-3p
NUDT3	hsa-miR-7386l-3p
NUDT3	hsa-miR-7177-5p
NUDT3	hsa-miR-659-5p
NUDT3	hsa-miR-7653-5p
NUDT3	hsa-miR-3143
NUDT3	hsa-miR-7158-3p
NUDT3	hsa-miR-6828-3p

NUDT3	hsa-miR-2890
NUDT3	hsa-miR-3079-3p
NUDT3	hsa-miR-2443
NUDT3	hsa-miR-7232-5p
NUDT3	hsa-miR-3614-3p
NUDT3	hsa-miR-522-3p
NUDT3	hsa-miR-3162-5p
NUDT3	hsa-miR-7163-3p
NUDT3	hsa-miR-6773-3p
NUDT3	hsa-miR-589-3p
NUDT3	hsa-miR-519-3p
NUDT3	hsa-miR-579-3p
NUDT3	hsa-miR-377-3p
NUDT3	hsa-miR-7196-5p
NUDT3	hsa-miR-2284k
NUDT3	hsa-miR-5694
NUDT3	hsa-miR-6832-3p
NUDT3	hsa-miR-300-3p
NUDT3	hsa-miR-367-3p
NUDT3	hsa-miR-3183
NUDT3	hsa-miR-6735-3p
NUDT3	hsa-miR-610
NUDT3	hsa-miR-3087-5p
NUDT3	hsa-miR-6384
NUDT3	hsa-miR-202-5p
NUDT3	hsa-miR-6812-5p
NUDT3	hsa-miR-130a-5p
NUDT3	hsa-miR-7307-3p
NUDT3	hsa-miR-3914
NUDT3	hsa-miR-4697-3p
NUDT3	hsa-miR-7281-3p
NUDT3	hsa-miR-5006-3p
NUDT3	hsa-miR-338-3p
NUDT3	hsa-miR-4742-5p
NUDT3	hsa-miR-3173-5p
NUDT3	hsa-miR-6856-5p
NUDT3	hsa-miR-7309-5p
NUDT3	hsa-miR-3692-3p
NUDT3	hsa-miR-6793-3p
NUDT3	hsa-miR-6810-5p
NUDT3	hsa-miR-7367-3p
NUDT3	hsa-miR-3912-5p
NUDT3	hsa-miR-6132
NUDT3	hsa-miR-690
NUDT3	hsa-miR-3535
NUDT3	hsa-miR-6128

NUDT3	hsa-miR-4687-3p
NUDT3	hsa-miR-496a-3p.1
NUDT3	hsa-miR-378c
NUDT3	hsa-miR-548ad-3p
NUDT3	hsa-miR-520gh
NUDT3	hsa-miR-4658
NUDT3	hsa-miR-4283
NUDT3	hsa-miR-4325
NUDT3	hsa-miR-7385-5p
NUDT3	hsa-miR-26b-3p
NUDT3	hsa-miR-519
NUDT3	hsa-miR-7076-5p
NUDT3	hsa-miR-629-3p
NUDT3	hsa-miR-4705
NUDT3	hsa-miR-7053-3p
NUDT3	hsa-miR-7222-3p
NUDT3	hsa-miR-669m-5p
NUDT3	hsa-miR-505-3p.2
NUDT3	hsa-miR-2362
NUDT3	hsa-miR-6807-5p
NUDT3	hsa-miR-3109-3p
NUDT3	hsa-miR-7859
NUDT3	hsa-miR-4654
NUDT3	hsa-miR-7688-3p
NUDT3	hsa-miR-7086-5p
NUDT3	hsa-miR-2284l
NUDT3	hsa-miR-223-5p
NUDT3	hsa-miR-5708
NUDT3	hsa-miR-412-3p
NUDT3	hsa-miR-6935-3p
NUDT3	hsa-miR-7245-3p
NUDT3	hsa-miR-205-5p
NUDT3	hsa-miR-24-3p
NUDT3	hsa-miR-7218-5p
NUDT3	hsa-miR-7300-5p
NUDT3	hsa-miR-7301-3p
NUDT3	hsa-miR-1615
NUDT3	hsa-miR-4763-3p
NUDT3	hsa-miR-6526
NUDT3	hsa-miR-548c-5p
NUDT3	hsa-miR-4530
NUDT3	hsa-miR-3544-5p
NUDT3	hsa-miR-6509-3p
NUDT3	hsa-miR-6891-5p
NUDT3	hsa-miR-7398h-3p
NUDT3	hsa-miR-4477a

NUDT3	hsa-miR-23a-5p
NUDT3	hsa-miR-142-3p.1
NUDT3	hsa-miR-34b-3p
NUDT3	hsa-miR-8899
NUDT3	hsa-miR-6514-5p
NUDT3	hsa-miR-7442-3p
NUDT3	hsa-miR-8833
NUDT3	hsa-miR-6895-5p
NUDT3	hsa-miR-337-3p
NUDT3	hsa-miR-153-5p
NUDT3	hsa-miR-15a-3p
NUDT3	hsa-miR-6836-3p
NUDT3	hsa-miR-7655-3p
NUDT3	hsa-miR-3605-5p
NUDT3	hsa-miR-7038-3p
NUDT3	hsa-miR-15c-3p
NUDT3	hsa-miR-434-5p
NUDT3	hsa-miR-7456-5p
NUDT3	hsa-miR-5193
NUDT3	hsa-miR-4697-5p
NUDT3	hsa-miR-624-3p
NUDT3	hsa-miR-5693
NUDT3	hsa-miR-6792-5p
NUDT3	hsa-miR-1545b-5p
NUDT3	hsa-miR-1769-5p
NUDT3	hsa-miR-6321
NUDT3	hsa-miR-424-3p
NUDT3	hsa-miR-200a-3p
NUDT3	hsa-miR-6837-3p
NUDT3	hsa-miR-2313-5p
NUDT3	hsa-miR-515-5p
NUDT3	hsa-miR-32-3p
NUDT3	hsa-miR-3188
NUDT3	hsa-miR-6772-5p
NUDT3	hsa-miR-5192
NUDT3	hsa-miR-7268-3p
NUDT3	hsa-miR-1547-5p
NUDT3	hsa-miR-7246-3p
NUDT3	hsa-miR-4793-3p
NUDT3	hsa-miR-7215-3p
NUDT3	hsa-miR-7371b-5p
NUDT3	hsa-miR-7314-5p
NUDT3	hsa-miR-7067-5p
NUDT3	hsa-miR-92a-1-5p
NUDT3	hsa-miR-519-5p
NUDT3	hsa-miR-7363-5p

NUDT3	hsa-miR-6554-5p
NUDT3	hsa-miR-502-5p
NUDT3	hsa-miR-3187-3p
NUDT3	hsa-miR-331-3p
NUDT3	hsa-miR-92-5p
NUDT3	hsa-miR-6728-5p
NUDT3	hsa-miR-6617-3p
NUDT3	hsa-miR-7024-3p
NUDT3	hsa-miR-489-5p
NUDT3	hsa-miR-203b-3p
NUDT3	hsa-miR-32-5p
NUDT3	hsa-miR-2341
NUDT3	hsa-miR-5706
NUDT3	hsa-miR-8865
NUDT3	hsa-miR-103-5p
NUDT3	hsa-miR-2114-3p
NUDT3	hsa-miR-549-3p
NUDT3	hsa-miR-211-5p
NUDT3	hsa-miR-513a-5p
NUDT3	hsa-miR-1575
NUDT3	hsa-miR-877-5p
NUDT3	hsa-miR-8838
NUDT3	hsa-miR-2328-3p
NUDT3	hsa-miR-3658
NUDT3	hsa-miR-216c-5p
NUDT3	hsa-miR-4646-5p
NUDT3	hsa-miR-2284
NUDT3	hsa-miR-1237-3p
NUDT3	hsa-miR-424-5p
NUDT3	hsa-miR-550b-3p
NUDT3	hsa-miR-1772-5p
NUDT3	hsa-miR-7444-3p
NUDT3	hsa-miR-496b
NUDT3	hsa-miR-640
NUDT3	hsa-miR-409
NUDT3	hsa-miR-2295
NUDT3	hsa-miR-6835-5p
NUDT3	hsa-miR-7660-5p
NUDT3	hsa-miR-6340
NUDT3	hsa-miR-6238
NUDT3	hsa-miR-2446
NUDT3	hsa-miR-664a-3p
NUDT3	hsa-miR-1544-3p
NUDT3	hsa-miR-2284m
NUDT3	hsa-miR-2483
NUDT3	hsa-miR-3126-3p

NUDT3	hsa-miR-4683
NUDT3	hsa-miR-7177-3p
NUDT3	hsa-miR-769
NUDT3	hsa-miR-3908
NUDT3	hsa-miR-6560-3p
NUDT3	hsa-miR-544b
NUDT3	hsa-miR-7656-3p
NUDT3	hsa-miR-7192-3p
NUDT3	hsa-miR-6624-3p
NUDT3	hsa-miR-3123
NUDT3	hsa-miR-635
NUDT3	hsa-miR-7280-5p
NUDT3	hsa-miR-145-3p
NUDT3	hsa-miR-7171-5p
NUDT3	hsa-miR-1796
NUDT3	hsa-miR-144-5p
NUDT3	hsa-miR-4781-3p
NUDT3	hsa-miR-1955-5p
NUDT3	hsa-miR-6884-3p
NUDT3	hsa-miR-671-5p
NUDT3	hsa-miR-6821-3p
NUDT3	hsa-miR-329
NUDT3	hsa-miR-2347
NUDT3	hsa-miR-1958
NUDT3	hsa-miR-5684
NUDT3	hsa-miR-5196-5p
NUDT3	hsa-miR-4438
NUDT3	hsa-miR-7319-3p
NUDT3	hsa-miR-6877-3p
NUDT3	hsa-miR-7180-3p
NUDT3	hsa-miR-3103-3p
NUDT3	hsa-miR-6781-3p
NUDT3	hsa-miR-514a-5p
NUDT3	hsa-miR-5621-3p
NUDT3	hsa-miR-548x-3p
NUDT3	hsa-miR-6861-3p
NUDT3	hsa-miR-1636
NUDT3	hsa-miR-196a-3p
NUDT3	hsa-miR-4716
NUDT3	hsa-miR-1650
NUDT3	hsa-miR-5626-3p
NUDT3	hsa-miR-1184
NUDT3	hsa-miR-7679-5p
NUDT3	hsa-miR-5580-3p
NUDT3	hsa-miR-3552
NUDT3	hsa-miR-3197

NUDT3	hsa-miR-1249-3p
NUDT3	hsa-miR-4666a-5p
NUDT3	hsa-miR-344c-3p
NUDT3	hsa-miR-4789-3p
NUDT3	hsa-miR-3978
NUDT3	hsa-miR-8860
NUDT3	hsa-miR-8795
NUDT3	hsa-miR-24-3p
NUDT3	hsa-miR-493-5p
NUDT3	hsa-miR-559
NUDT3	hsa-miR-1255a
NUDT3	hsa-miR-7042-5p
NUDT3	hsa-miR-6879-5p
NUDT3	hsa-miR-3922-3p
NUDT3	hsa-miR-4310
NUDT3	hsa-miR-6647-5p
NUDT3	hsa-miR-700-5p
NUDT3	hsa-miR-6945-3p
NUDT3	hsa-miR-2285c
NUDT3	hsa-miR-876-5p
NUDT3	hsa-miR-6848-3p
NUDT3	hsa-miR-126-5p
NUDT3	hsa-miR-6780b-5p
NUDT3	hsa-miR-6917-5p
NUDT3	hsa-miR-6509-5p
NUDT3	hsa-miR-295-5p
NUDT3	hsa-miR-1191b-3p
NUDT3	hsa-miR-3583-5p
NUDT3	hsa-miR-29e
NUDT3	hsa-miR-500
NUDT3	hsa-miR-7357-3p
NUDT3	hsa-miR-7181-5p
NUDT3	hsa-miR-4793-5p
NUDT3	hsa-miR-6865-3p
NUDT3	hsa-miR-2385-5p
NUDT3	hsa-miR-8873a
NUDT3	hsa-miR-7658-5p
NUDT3	hsa-miR-4729
NUDT3	hsa-miR-3063-3p
NUDT3	hsa-miR-1202
NUDT3	hsa-miR-627-3p
NUDT3	hsa-miR-365b-5p
NUDT3	hsa-miR-4731-5p
NUDT3	hsa-miR-6946-3p
NUDT3	hsa-miR-6122-5p
NUDT3	hsa-miR-548av-3p

NUDT3	hsa-miR-3092-5p
NUDT3	hsa-miR-8874
NUDT3	hsa-miR-7392-5p
NUDT3	hsa-miR-5096
NUDT3	hsa-miR-3924
NUDT3	hsa-miR-3150b-3p
NUDT3	hsa-miR-4292
NUDT3	hsa-miR-7335-5p
NUDT3	hsa-miR-3584-5p
NUDT3	hsa-miR-8101
NUDT3	hsa-miR-6809-3p
NUDT3	hsa-miR-520f-5p
NUDT3	hsa-miR-505-3p.1
NUDT3	hsa-miR-7217-3p
NUDT3	hsa-miR-878-3p
NUDT3	hsa-miR-7364-3p
NUDT3	hsa-miR-363-5p
NUDT3	hsa-miR-3083-5p
NUDT3	hsa-miR-3528
NUDT3	hsa-miR-6516-3p
NUDT3	hsa-miR-2454-3p
NUDT3	hsa-miR-1842
NUDT3	hsa-miR-6418-5p
NUDT3	hsa-miR-300
NUDT3	hsa-miR-1897-3p
NUDT3	hsa-miR-3084-3p
NUDT3	hsa-miR-5010-5p
NUDT3	hsa-miR-223-3p
NUDT3	hsa-miR-2430
NUDT3	hsa-miR-6783-5p
NUDT3	hsa-miR-6973a-5p
NUDT3	hsa-miR-2887
NUDT3	hsa-miR-6987-5p
NUDT3	hsa-miR-1777
NUDT3	hsa-miR-2127
NUDT3	hsa-miR-3971
NUDT3	hsa-miR-2395
NUDT3	hsa-miR-512-5p
NUDT3	hsa-miR-8079
NUDT3	hsa-miR-4276
NUDT3	hsa-miR-4503
NUDT3	hsa-miR-196-5p
NUDT3	hsa-miR-3199
NUDT3	hsa-miR-7390-3p
NUDT3	hsa-miR-1238-3p
NUDT3	hsa-miR-7371e-5p

NUDT3	hsa-miR-29-3p
NUDT3	hsa-miR-2466-5p
NUDT3	hsa-miR-6726-5p
NUDT3	hsa-miR-1195
NUDT3	hsa-miR-466f-5p
NUDT3	hsa-miR-6872-3p
NUDT3	hsa-miR-4741
NUDT3	hsa-miR-6506-5p
NUDT3	hsa-miR-6504-3p
NUDT3	hsa-miR-7273-3p
NUDT3	hsa-miR-2293
NUDT3	hsa-miR-7190-5p
NUDT3	hsa-miR-8891
NUDT3	hsa-miR-183-5p
NUDT3	hsa-miR-7237-3p
NUDT3	hsa-miR-139-5p
NUDT3	hsa-miR-7862
NUDT3	hsa-miR-4760-3p
NUDT3	hsa-miR-377-3p
NUDT3	hsa-miR-3192
NUDT3	hsa-miR-6527
NUDT3	hsa-miR-577-5p
NUDT3	hsa-miR-6957-3p
NUDT3	hsa-miR-34b-3p
NUDT3	hsa-miR-193a-5p
NUDT3	hsa-miR-361-3p
NUDT3	hsa-miR-432-5p
NUDT3	hsa-miR-467f
NUDT3	hsa-miR-1915-5p
NUDT3	hsa-miR-6959-5p
NUDT3	hsa-miR-26c
NUDT3	hsa-miR-4534
NUDT3	hsa-miR-548h-3p
NUDT3	hsa-miR-138-5p
NUDT3	hsa-miR-548ab
NUDT3	hsa-miR-4323
NUDT3	hsa-miR-128-5p
NUDT3	hsa-miR-2411-3p
NUDT3	hsa-miR-20b-3p
NUDT3	hsa-miR-1540
NUDT3	hsa-miR-6836-5p
NUDT3	hsa-miR-1679
NUDT3	hsa-miR-1251
NUDT3	hsa-miR-7291-5p
NUDT3	hsa-miR-3102-3p.2-3p
NUDT3	hsa-miR-2291

NUDT3	hsa-miR-4524b-3p
NUDT3	hsa-miR-7285-5p
NUDT3	hsa-miR-7364-5p
NUDT3	hsa-miR-2330-3p
NUDT3	hsa-miR-548am-3p
NUDT3	hsa-miR-4716-5p
NUDT3	hsa-miR-1255-3p
NUDT3	hsa-miR-6359
NUDT3	hsa-miR-651-3p
NUDT3	hsa-miR-548aj-3p
NUDT3	hsa-miR-3916
NUDT3	hsa-miR-6381
NUDT3	hsa-miR-199b-3-5p
NUDT3	hsa-miR-742-3p
NUDT3	hsa-miR-141-5p
NUDT3	hsa-miR-380-5p
NUDT3	hsa-miR-7030-5p
NUDT3	hsa-miR-7269-5p
NUDT3	hsa-miR-7053-5p
NUDT3	hsa-miR-599-5p
NUDT3	hsa-miR-1283a
NUDT3	hsa-miR-500-3p
NUDT3	hsa-miR-6736-3p
NUDT3	hsa-miR-548o-5p
NUDT3	hsa-miR-6800-3p
NUDT3	hsa-miR-6507-5p
NUDT3	hsa-miR-4768-5p
NUDT3	hsa-miR-1227-5p
NUDT3	hsa-miR-767-3p
NUDT3	hsa-miR-3918
NUDT3	hsa-miR-4724-3p
NUDT3	hsa-miR-2471-5p
NUDT3	hsa-miR-7438-5p
NUDT3	hsa-miR-7089-5p
NUDT3	hsa-miR-10
NUDT3	hsa-miR-2284x
NUDT3	hsa-miR-129b-3p
NUDT3	hsa-miR-661
NUDT3	hsa-miR-7315-5p
NUDT3	hsa-miR-5697
NUDT3	hsa-miR-224-5p
NUDT3	hsa-miR-379-5p
NUDT3	hsa-miR-7850-5p
NUDT3	hsa-miR-3157-3p
NUDT3	hsa-miR-4797-5p
NUDT3	hsa-miR-26

NUDT3	hsa-miR-7264-3p
NUDT3	hsa-miR-410
NUDT3	hsa-miR-935
NUDT3	hsa-miR-129-2-3p
NUDT3	hsa-miR-365a-3p
NUDT3	hsa-miR-5627-3p
NUDT3	hsa-miR-495-5p
NUDT3	hsa-miR-7164-3p
NUDT3	hsa-miR-412
NUDT3	hsa-miR-129-3p
NUDT3	hsa-miR-6511-3p
NUDT4	hsa-miR-330-3p
NUDT4	hsa-miR-7079-5p
NUDT4	hsa-miR-548ae-3p
NUDT4	hsa-miR-496.1
NUDT4	hsa-miR-6847-3p
NUDT4	hsa-miR-494
NUDT4	hsa-miR-194-5p
NUDT4	hsa-miR-1255
NUDT4	hsa-miR-201-3p
NUDT4	hsa-miR-154-5p
NUDT4	hsa-miR-19b-5p
NUDT4	hsa-miR-6841-3p
NUDT4	hsa-miR-487a-3p
NUDT4	hsa-miR-2370-5p
NUDT4	hsa-miR-2276-3p
NUDT4	hsa-miR-2054
NUDT4	hsa-miR-296-5p
NUDT4	hsa-miR-7273-5p
NUDT4	hsa-miR-7251-3p
NUDT4	hsa-miR-5617-5p
NUDT4	hsa-miR-1913
NUDT4	hsa-miR-1434-3p
NUDT4	hsa-miR-19a-5p
NUDT4	hsa-miR-4662a-3p
NUDT4	hsa-miR-593-3p
NUDT4	hsa-miR-195-3p
NUDT4	hsa-miR-411-3p
NUDT4	hsa-miR-549a
NUDT4	hsa-miR-3126-5p
NUDT4	hsa-miR-200a-5p
NUDT4	hsa-miR-511-3p
NUDT4	hsa-miR-3689-3p
NUDT4	hsa-miR-18-5p
NUDT4	hsa-miR-21-5p
NUDT4	hsa-miR-211-3p

NUDT4	hsa-miR-539-3p
NUDT4	hsa-miR-4495
NUDT4	hsa-miR-497-3p
NUDT4	hsa-miR-7081-5p
NUDT4	hsa-miR-7039-5p
NUDT4	hsa-miR-4505
NUDT4	hsa-miR-878
NUDT4	hsa-miR-1262
NUDT4	hsa-miR-4510
NUDT4	hsa-miR-6529b
NUDT4	hsa-miR-4444
NUDT4	hsa-miR-7703
NUDT4	hsa-miR-221-5p
NUDT4	hsa-miR-411-5p.1
NUDT4	hsa-miR-6828-5p
NUDT4	hsa-miR-1273h-5p
NUDT4	hsa-miR-4700-3p
NUDT4	hsa-miR-6315
NUDT4	hsa-miR-4725-3p
NUDT4	hsa-miR-508b
NUDT4	hsa-miR-7031-5p
NUDT4	hsa-miR-5116
NUDT4	hsa-miR-5134-5p
NUDT4	hsa-miR-130-3p
NUDT4	hsa-miR-3652
NUDT4	hsa-miR-7198-3p
NUDT4	hsa-miR-3073a-3p
NUDT4	hsa-miR-16-1-3p
NUDT4	hsa-miR-4802-5p
NUDT4	hsa-miR-874-5p
NUDT4	hsa-miR-6938-3p
NUDT4	hsa-miR-148b-5p
NUDT4	hsa-miR-582-5p
NUDT4	hsa-miR-331-5p
NUDT4	hsa-miR-6910-3p
NUDT4	hsa-miR-323-3p
NUDT4	hsa-miR-548az-5p
NUDT4	hsa-miR-2277-3p
NUDT4	hsa-miR-6892-5p
NUDT4	hsa-miR-5589-3p
NUDT4	hsa-miR-30c-2-3p
NUDT4	hsa-miR-548ar-5p
NUDT4	hsa-miR-3090-5p
NUDT4	hsa-miR-4650
NUDT4	hsa-miR-7389-3p
NUDT4	hsa-miR-548ad-5p

NUDT4	hsa-miR-19-5p
NUDT4	hsa-miR-1844
NUDT4	hsa-miR-205-3p
NUDT4	hsa-miR-7398r-5p
NUDT4	hsa-miR-7333-5p
NUDT4	hsa-miR-7232-3p
NUDT4	hsa-miR-4713-5p
NUDT4	hsa-miR-3057-3p
NUDT4	hsa-miR-30c-3p
NUDT4	hsa-miR-1183
NUDT4	hsa-miR-8118
NUDT4	hsa-miR-8805
NUDT4	hsa-miR-138-5p
NUDT4	hsa-miR-515-3p
NUDT4	hsa-miR-323
NUDT4	hsa-miR-6788-5p
NUDT4	hsa-miR-3548
NUDT4	hsa-miR-291-3p
NUDT4	hsa-miR-322-3p
NUDT4	hsa-miR-302a-5p
NUDT4	hsa-miR-1246
NUDT4	hsa-miR-7856-5p
NUDT4	hsa-miR-580-3p
NUDT4	hsa-miR-4664-5p
NUDT4	hsa-miR-22-5p
NUDT4	hsa-miR-3473c
NUDT4	hsa-miR-6848-5p
NUDT4	hsa-miR-200ac-5p
NUDT4	hsa-miR-8059
NUDT4	hsa-miR-619
NUDT4	hsa-miR-1291
NUDT4	hsa-miR-6808-5p
NUDT4	hsa-miR-6885-3p
NUDT4	hsa-miR-2184
NUDT4	hsa-miR-5582-5p
NUDT4	hsa-miR-6896-3p
NUDT4	hsa-miR-2414
NUDT4	hsa-miR-6972-5p
NUDT4	hsa-miR-4472
NUDT4	hsa-miR-1296
NUDT4	hsa-miR-199
NUDT4	hsa-miR-33b-3p
NUDT4	hsa-miR-1188-5p
NUDT4	hsa-miR-424-5p
NUDT4	hsa-miR-7282-5p
NUDT4	hsa-miR-8905

NUDT4	hsa-miR-3113-5p
NUDT4	hsa-miR-3473f
NUDT4	hsa-miR-6391
NUDT4	hsa-miR-3112-5p
NUDT4	hsa-miR-570-3p
NUDT4	hsa-miR-5697-3p
NUDT4	hsa-miR-141-3p
NUDT4	hsa-miR-6715-3p
NUDT4	hsa-miR-6913-3p
NUDT4	hsa-miR-351-5p
NUDT4	hsa-miR-6780a-3p
NUDT4	hsa-miR-7157-5p
NUDT4	hsa-miR-7395-3p
NUDT4	hsa-miR-7182-3p
NUDT4	hsa-miR-7032-3p
NUDT4	hsa-miR-105-5p
NUDT4	hsa-miR-1260a
NUDT4	hsa-miR-7019-5p
NUDT4	hsa-miR-548ap-5p
NUDT4	hsa-miR-3127-3p
NUDT4	hsa-miR-92a-5p
NUDT4	hsa-miR-758-5p
NUDT4	hsa-miR-4733-5p
NUDT4	hsa-miR-7257-5p
NUDT4	hsa-miR-618
NUDT4	hsa-miR-6833-3p
NUDT4	hsa-miR-448-3p
NUDT4	hsa-miR-532-3p
NUDT4	hsa-miR-7666-3p
NUDT4	hsa-miR-636
NUDT4	hsa-miR-199-1-5p
NUDT4	hsa-miR-3651
NUDT4	hsa-miR-7078-3p
NUDT4	hsa-miR-371a-5p
NUDT4	hsa-miR-2457
NUDT4	hsa-miR-549-5p
NUDT4	hsa-miR-548e-5p
NUDT4	hsa-miR-506-5p
NUDT4	hsa-miR-1972
NUDT4	hsa-miR-650
NUDT4	hsa-miR-4260
NUDT4	hsa-miR-7117-3p
NUDT4	hsa-miR-2285af
NUDT4	hsa-miR-499
NUDT4	hsa-miR-302b-5p
NUDT4	hsa-miR-376c-3p

NUDT4	hsa-miR-5692a
NUDT4	hsa-miR-455-5p
NUDT4	hsa-miR-7387-5p
NUDT4	hsa-miR-201-5p
NUDT4	hsa-miR-6528
NUDT4	hsa-miR-2361
NUDT4	hsa-miR-134-5p
NUDT4	hsa-miR-6953-3p
NUDT4	hsa-miR-491-3p
NUDT4	hsa-miR-3124-3p
NUDT4	hsa-miR-7382-5p
NUDT4	hsa-miR-98-3p
NUDT4	hsa-miR-4766-5p
NUDT4	hsa-miR-548e-3p
NUDT4	hsa-miR-7299-3p
NUDT4	hsa-miR-3119
NUDT4	hsa-miR-7199-3p
NUDT4	hsa-miR-1260b
NUDT4	hsa-miR-203
NUDT4	hsa-miR-644a
NUDT4	hsa-miR-454-3p
NUDT4	hsa-miR-4662b
NUDT4	hsa-miR-4753-5p
NUDT4	hsa-miR-5692bc
NUDT4	hsa-miR-3079-5p
NUDT4	hsa-miR-340-5p
NUDT4	hsa-miR-6130
NUDT4	hsa-miR-4742-3p
NUDT4	hsa-miR-4478
NUDT4	hsa-miR-4671-3p
NUDT4	hsa-miR-1545c-3p
NUDT4	hsa-miR-9768-5p
NUDT4	hsa-miR-8886
NUDT4	hsa-miR-891-3p
NUDT4	hsa-miR-497-5p
NUDT4	hsa-miR-501-3p
NUDT4	hsa-miR-4452
NUDT4	hsa-miR-548ak
NUDT4	hsa-miR-518d-5p
NUDT4	hsa-miR-7262a-5p
NUDT4	hsa-miR-2462
NUDT4	hsa-miR-7011-3p
NUDT4	hsa-miR-7029-5p
NUDT4	hsa-miR-1251-3p
NUDT4	hsa-miR-6519
NUDT4	hsa-miR-2294

NUDT4	hsa-miR-186-3p
NUDT4	hsa-miR-743b-3p
NUDT4	hsa-miR-8908-3p
NUDT4	hsa-miR-137
NUDT4	hsa-miR-7386f-3p
NUDT4	hsa-miR-7270-5p
NUDT4	hsa-miR-7197-3p
NUDT4	hsa-miR-214-5p
NUDT4	hsa-miR-7227-3p
NUDT4	hsa-miR-4649-3p
NUDT4	hsa-miR-520g-5p
NUDT4	hsa-miR-126-3p.2
NUDT4	hsa-miR-6732-5p
NUDT4	hsa-miR-23
NUDT4	hsa-miR-3679-3p
NUDT4	hsa-miR-4328
NUDT4	hsa-miR-145-5p
NUDT4	hsa-miR-6074
NUDT4	hsa-miR-207
NUDT4	hsa-let-7g-3p
NUDT4	hsa-miR-6843-3p
NUDT4	hsa-miR-483-3p.2
NUDT4	hsa-miR-200a-3p
NUDT4	hsa-miR-103-2-5p
NUDT4	hsa-miR-33-5p
NUDT4	hsa-miR-6769a-5p
NUDT4	hsa-miR-7258-5p
NUDT4	hsa-miR-881-5p
NUDT4	hsa-miR-548a-3p
NUDT4	hsa-miR-1227-3p
NUDT4	hsa-miR-320a
NUDT4	hsa-miR-7292-5p
NUDT4	hsa-miR-7160-5p
NUDT4	hsa-miR-7202-3p
NUDT4	hsa-miR-6134
NUDT4	hsa-miR-200-3p
NUDT4	hsa-miR-6581-3p
NUDT4	hsa-miR-567
NUDT4	hsa-miR-382-5p
NUDT4	hsa-miR-6797-5p
NUDT4	hsa-miR-8810
NUDT4	hsa-miR-1545a-3p
NUDT4	hsa-miR-2467-5p
NUDT4	hsa-miR-7398u-3p
NUDT4	hsa-miR-4801
NUDT4	hsa-miR-7041-5p

NUDT4	hsa-miR-518-5p
NUDT4	hsa-miR-7173-3p
NUDT4	hsa-miR-7664-5p
NUDT4	hsa-miR-548t-5p
NUDT4	hsa-miR-3942-3p
NUDT4	hsa-miR-330-3p.2
NUDT4	hsa-miR-5588-5p
NUDT4	hsa-miR-8821
NUDT4	hsa-miR-5584-5p
NUDT4	hsa-miR-8906
NUDT4	hsa-miR-7-3p
NUDT4	hsa-miR-7026-5p
NUDT4	hsa-miR-616-5p
NUDT4	hsa-miR-465-5p
NUDT4	hsa-miR-203-3p.2
NUDT4	hsa-miR-4496
NUDT4	hsa-miR-2410
NUDT4	hsa-miR-4699-5p
NUDT4	hsa-miR-371b-5p
NUDT4	hsa-miR-3616-5p
NUDT4	hsa-miR-6986-3p
NUDT4	hsa-miR-224-5p
NUDT4	hsa-miR-4271
NUDT4	hsa-miR-132-3p
NUDT4	hsa-miR-1285-3p
NUDT4	hsa-miR-2681-3p
NUDT4	hsa-miR-2329-3p
NUDT4	hsa-miR-6826-3p
NUDT4	hsa-miR-4775
NUDT4	hsa-miR-2345
NUDT4	hsa-miR-6946-5p
NUDT4	hsa-miR-6825-5p
NUDT4	hsa-miR-1292-5p
NUDT4	hsa-miR-922
NUDT4	hsa-miR-7021-3p
NUDT4	hsa-miR-1-5p
NUDT4	hsa-miR-1197-5p
NUDT4	hsa-miR-546
NUDT4	hsa-miR-7359-5p
NUDT4	hsa-miR-130
NUDT4	hsa-miR-345-5p
NUDT4	hsa-miR-295-3p
NUDT4	hsa-miR-411-5p.2
NUDT4	hsa-miR-432-3p
NUDT4	hsa-miR-7164-5p
NUDT4	hsa-miR-3978

NUDT4	hsa-miR-526b-5p
NUDT4	hsa-miR-2313-3p
NUDT4	hsa-miR-3145-5p
NUDT4	hsa-miR-6502-3p
NUDT4	hsa-miR-193-3p
NUDT4	hsa-miR-4802-3p
NUDT4	hsa-miR-6908-5p
NUDT4	hsa-miR-23b-5p
NUDT4	hsa-miR-8822
NUDT4	hsa-miR-1933-5p
NUDT4	hsa-miR-513c-3p
NUDT4	hsa-miR-6663-5p
NUDT4	hsa-miR-3913-5p
NUDT4	hsa-miR-4471
NUDT4	hsa-miR-6871-5p
NUDT4	hsa-miR-2365
NUDT4	hsa-miR-1-2-5p
NUDT4	hsa-miR-607
NUDT4	hsa-miR-6216
NUDT4	hsa-miR-7194-5p
NUDT4	hsa-miR-510-5p
NUDT4	hsa-miR-7052-5p
NUDT4	hsa-miR-4720-3p
NUDT4	hsa-miR-491-5p
NUDT4	hsa-miR-597
NUDT4	hsa-miR-4277
NUDT4	hsa-miR-1982-3p
NUDT4	hsa-miR-4524a-3p
NUDT4	hsa-miR-2444
NUDT4	hsa-miR-2367-3p
NUDT4	hsa-miR-26-5p
NUDT4	hsa-miR-516-3p
NUDT4	hsa-miR-1251-5p
NUDT4	hsa-miR-758-3p
NUDT4	hsa-miR-592-3p
NUDT4	hsa-miR-4645-5p
NUDT4	hsa-miR-6680-3p
NUDT4	hsa-miR-181-5p
NUDT4	hsa-miR-2468
NUDT4	hsa-miR-2377
NUDT4	hsa-miR-101b-3p.1
NUDT4	hsa-miR-7205-3p
NUDT4	hsa-miR-8842
NUDT4	hsa-miR-3133
NUDT4	hsa-miR-4256
NUDT4	hsa-miR-2390

NUDT4	hsa-miR-3586-5p
NUDT4	hsa-miR-32-5p
NUDT4	hsa-miR-2372
NUDT4	hsa-miR-5011-3p
NUDT4	hsa-miR-200bc
NUDT4	hsa-miR-6903-5p
NUDT4	hsa-miR-6818-3p
NUDT4	hsa-miR-190-5p
NUDT4	hsa-miR-193b-5p
NUDT4	hsa-miR-33a-3p
NUDT4	hsa-miR-8837
NUDT4	hsa-miR-214-3p
NUDT4	hsa-miR-196-3p
NUDT4	hsa-miR-29-5p
NUDT4	hsa-miR-524-3p
NUDT4	hsa-miR-147-5p
NUDT4	hsa-miR-6894-5p
NUDT4	hsa-miR-147a
NUDT4	hsa-miR-518a-5p
NUDT4	hsa-miR-5003-3p
NUDT4	hsa-miR-7062-3p
NUDT4	hsa-miR-302-3p
NUDT4	hsa-miR-3596d
NUDT4	hsa-miR-7090-5p
NUDT4	hsa-miR-8895
NUDT4	hsa-miR-7398e-3p
NUDT4	hsa-miR-9-3p
NUDT4	hsa-miR-622
NUDT4	hsa-miR-653-5p
NUDT4	hsa-miR-183-5p.2
NUDT4	hsa-miR-3121-3p
NUDT4	hsa-miR-4501
NUDT4	hsa-miR-7271-3p
NUDT4	hsa-miR-4652-3p
NUDT4	hsa-miR-1388-5p
NUDT4	hsa-miR-466n-3p
NUDT4	hsa-miR-3614-5p
NUDT4	hsa-miR-5572
NUDT4	hsa-miR-15-5p
NUDT4	hsa-miR-566
NUDT4	hsa-miR-6911-5p
NUDT4	hsa-miR-744-3p
NUDT4	hsa-miR-2284aa
NUDT4	hsa-miR-8799
NUDT4	hsa-miR-378a-5p
NUDT4	hsa-miR-1295b-5p

NUDT4	hsa-miR-6857-5p
NUDT4	hsa-miR-93-3p
NUDT4	hsa-miR-7386e-3p
NUDT4	hsa-miR-1910-5p
NUDT4	hsa-miR-1-1-5p
NUDT4	hsa-miR-6948-3p
NUDT4	hsa-miR-4656
NUDT4	hsa-miR-490-5p
NUDT4	hsa-miR-1547-3p
NUDT4	hsa-miR-4639-5p
NUDT4	hsa-miR-5197-5p
NUDT4	hsa-miR-7355-5p
NUDT4	hsa-miR-137-3p
NUDT4	hsa-miR-6738-3p
NUDT4	hsa-miR-764
NUDT4	hsa-miR-92
NUDT4	hsa-miR-301-3p
NUDT4	hsa-miR-7176-3p
NUDT4	hsa-miR-7028-3p
NUDT4	hsa-miR-429-3p
NUDT4	hsa-miR-513b-5p
NUDT4	hsa-miR-8888
NUDT4	hsa-miR-3923
NUDT4	hsa-miR-125a-3p
NUDT4	hsa-miR-6772-5p
NUDT4	hsa-miR-434-3p
NUDT4	hsa-miR-31-3p
NUDT4	hsa-miR-874
NUDT4	hsa-miR-4274
NUDT4	hsa-miR-372-5p
NUDT4	hsa-miR-2486-5p
NUDT4	hsa-miR-548j-5p
NUDT4	hsa-miR-3117-5p
NUDT4	hsa-miR-4804-3p
NUDT4	hsa-miR-506-3p
NUDT4	hsa-miR-466bcp-3p
NUDT4	hsa-miR-2285y
NUDT4	hsa-miR-3662
NUDT4	hsa-miR-4320
NUDT4	hsa-miR-181b-3p
NUDT4	hsa-miR-4756-3p
NUDT4	hsa-miR-514a-3p
NUDT4	hsa-miR-4762-3p
NUDT4	hsa-miR-4678
NUDT4	hsa-miR-6408
NUDT4	hsa-miR-30-3p

NUDT4	hsa-miR-548ar-3p
NUDT4	hsa-miR-3611
NUDT4	hsa-miR-5118
NUDT4	hsa-miR-4786-5p
NUDT4	hsa-miR-106-3p
NUDT4	hsa-miR-200c-5p
NUDT4	hsa-miR-134
NUDT4	hsa-miR-448
NUDT4	hsa-miR-25-3p
NUDT4	hsa-miR-4676-5p
NUDT4	hsa-miR-4778-5p
NUDT4	hsa-miR-6778-5p
NUDT4	hsa-miR-7266-5p
NUDT4	hsa-miR-6959-5p
NUDT4	hsa-miR-129-5p
NUDT4	hsa-miR-4743-3p
NUDT4	hsa-miR-712-5p
NUDT4	hsa-miR-2188-3p
NUDT4	hsa-miR-211-5p
NUDT4	hsa-miR-7246-3p
NUDT4	hsa-miR-2052
NUDT4	hsa-miR-3135a
NUDT4	hsa-miR-609
NUDT4	hsa-miR-1283
NUDT4	hsa-miR-1912-3p
NUDT4	hsa-miR-7070-5p
NUDT4	hsa-miR-758-3p
NUDT4	hsa-miR-34-5p
NUDT4	hsa-miR-221-3p
NUDT4	hsa-miR-6529a
NUDT4	hsa-miR-2284g
NUDT4	hsa-miR-4265
NUDT4	hsa-miR-467eh-5p
NUDT4	hsa-miR-4521
NUDT4	hsa-miR-1968-5p
NUDT4	hsa-miR-488-3p
NUDT4	hsa-miR-872-5p
NUDT4	hsa-miR-5001-3p
NUDT4	hsa-miR-17-3p
NUDT4	hsa-miR-141-3p
NUDT4	hsa-miR-3660
NUDT4	hsa-miR-7020-5p
NUDT4	hsa-miR-541-5p
NUDT4	hsa-miR-4432
NUDT4	hsa-miR-5571-5p
NUDT4	hsa-miR-7324-3p

NUDT4	hsa-miR-1953
NUDT4	hsa-miR-491-5p
NUDT4	hsa-miR-324-3p
NUDT4	hsa-miR-23-3p
NUDT4	hsa-miR-300-5p
NUDT4	hsa-miR-6684-5p
NUDT4	hsa-miR-4762-5p
NUDT4	hsa-miR-6934-5p
NUDT4	hsa-miR-223-5p
NUDT4	hsa-miR-706
NUDT4	hsa-miR-421-5p
NUDT4	hsa-miR-7385d-3p
NUDT4	hsa-miR-376a-2-5p
NUDT4	hsa-miR-7265-5p
NUDT4	hsa-miR-6769b-3p
NUDT4	hsa-miR-548w
NUDT4	hsa-miR-1207
NUDT4	hsa-miR-7338-5p
NUDT4	hsa-miR-7380-3p
NUDT4	hsa-miR-4278
NUDT4	hsa-miR-2381
NUDT4	hsa-miR-664
NUDT4	hsa-miR-375
NUDT4	hsa-miR-3096-5p
NUDT4	hsa-miR-4422
NUDT4	hsa-miR-379-3p
NUDT4	hsa-miR-3174
NUDT4	hsa-miR-2284y
NUDT4	hsa-miR-4446
NUDT4	hsa-miR-344b-3p
NUDT4	hsa-miR-518g-3p
NUDT4	hsa-miR-7246-5p
NUDT4	hsa-miR-4494
NUDT4	hsa-miR-3688-5p
NUDT4	hsa-miR-578
NUDT4	hsa-miR-206
NUDT4	hsa-miR-6794-3p
NUDT4	hsa-miR-107
NUDT4	hsa-miR-4654
NUDT4	hsa-miR-3093-3p
NUDT4	hsa-miR-7307-5p
NUDT4	hsa-miR-669
NUDT4	hsa-miR-6984-5p
NUDT4	hsa-miR-2351
NUDT4	hsa-miR-678
NUDT4	hsa-miR-7245-5p

NUDT4	hsa-miR-3116
NUDT4	hsa-miR-6965-5p
NUDT4	hsa-miR-142-3p.2
NUDT4	hsa-miR-6883-5p
NUDT4	hsa-miR-6728-3p
NUDT4	hsa-miR-7168-5p
NUDT4	hsa-miR-4499
NUDT4	hsa-miR-3562
NUDT4	hsa-miR-742-5p
NUDT4	hsa-miR-1894-5p
NUDT4	hsa-miR-4677-5p
NUDT4	hsa-miR-6833-5p
NUDT4	hsa-miR-3156-5p
NUDT4	hsa-miR-204-5p
NUDT4	hsa-miR-3607-5p
NUDT4	hsa-miR-22-3p
NUDT4	hsa-miR-539-5p
NUDT4	hsa-miR-6981-3p
NUDT4	hsa-miR-6874-3p
NUDT4	hsa-miR-548n
NUDT4	hsa-miR-6417
NUDT4	hsa-miR-4787-5p
NUDT4	hsa-miR-548av-5p
NUDT4	hsa-miR-4728-5p
NUDT4	hsa-miR-4785
NUDT4	hsa-miR-881-3p
NUDT4	hsa-miR-5622-3p
NUDT4	hsa-miR-6129
NUDT4	hsa-miR-93-5p
NUDT4	hsa-miR-500b-3p
NUDT4	hsa-miR-6864-3p
NUDT4	hsa-miR-1931
NUDT4	hsa-miR-409-3p
NUDT4	hsa-miR-6810-5p
NUDT4	hsa-miR-1981-5p
NUDT4	hsa-miR-495-3p
NUDT4	hsa-miR-647
NUDT4	hsa-miR-486-5p
NUDT4	hsa-miR-1179
NUDT4	hsa-miR-6931-5p
NUDT4	hsa-miR-139-5p
NUDT4	hsa-miR-5581-3p
NUDT4	hsa-miR-326-5p
NUDT4	hsa-miR-7007-5p
NUDT4	hsa-miR-199-3p
NUDT4	hsa-miR-200c-3p

NUDT4	hsa-miR-504
NUDT4	hsa-miR-378
NUDT4	hsa-miR-4284
NUDT4	hsa-miR-655
NUDT4	hsa-miR-6387
NUDT4	hsa-miR-1292-5p
NUDT4	hsa-miR-3656
NUDT4	hsa-miR-2363
NUDT4	hsa-miR-183-5p
NUDT4	hsa-miR-7398b-3p
NUDT4	hsa-miR-2398
NUDT4	hsa-miR-1284
NUDT4	hsa-miR-1892
NUDT4	hsa-miR-7340-5p
NUDT4	hsa-miR-7a-1-3p
NUDT4	hsa-miR-151-3p
NUDT4	hsa-miR-4493
NUDT4	hsa-miR-6971-5p
NUDT4	hsa-miR-766-3p
NUDT4	hsa-miR-500a-5p
NUDT4	hsa-miR-548as-5p
NUDT4	hsa-miR-550-5p
NUDT4	hsa-miR-7184-5p
NUDT4	hsa-miR-532-5p
NUDT4	hsa-miR-684
NUDT4	hsa-miR-7662-5p
NUDT4	hsa-miR-2291
NUDT4	hsa-miR-505-3p
NUDT4	hsa-miR-548aw
NUDT4	hsa-miR-6883-3p
NUDT4	hsa-miR-7398t-3p
NUDT4	hsa-miR-373-5p
NUDT4	hsa-miR-655-3p
NUDT4	hsa-miR-7043-5p
NUDT4	hsa-miR-496.2
NUDT4	hsa-miR-7302-5p
NUDT4	hsa-miR-759
NUDT4	hsa-miR-142-3p
NUDT4	hsa-miR-7013-3p
NUDT4	hsa-miR-8902
NUDT4	hsa-miR-410-3p
NUDT4	hsa-miR-455-3p.1
NUDT4	hsa-miR-4272
NUDT4	hsa-miR-6912-5p
NUDT4	hsa-miR-3078-3p
NUDT4	hsa-miR-6511-5p

NUDT4	hsa-miR-5124a
NUDT4	hsa-miR-3573-3p
NUDT4	hsa-miR-6842-5p
NUDT4	hsa-miR-381-3p
NUDT4	hsa-miR-6981-5p
NUDT4	hsa-miR-548aq-3p
NUDT4	hsa-miR-7383-3p
NUDT4	hsa-miR-6991-5p
NUDT4	hsa-miR-7377-5p
NUDT4	hsa-miR-450b
NUDT4	hsa-miR-202-3p
NUDT4	hsa-miR-2464-3p
NUDT4	hsa-miR-3600
NUDT4	hsa-miR-298-3p
NUDT4	hsa-miR-1256
NUDT4	hsa-miR-2284f
NUDT4	hsa-miR-6964-5p
NUDT4	hsa-miR-6860
NUDT4	hsa-miR-4275
NUDT4	hsa-miR-2373-5p
NUDT4	hsa-miR-743a-3p
NUDT4	hsa-miR-2466-3p
NUDT4	hsa-miR-7665-5p
NUDT4	hsa-miR-144-3p
NUDT4	hsa-miR-215-3p
NUDT4	hsa-miR-330-3p
NUDT4	hsa-miR-4290
NUDT4	hsa-miR-3085-5p
NUDT4	hsa-miR-8838
NUDT4	hsa-miR-8901
NUDT4	hsa-miR-3085-3p
NUDT4	hsa-miR-770-5p
NUDT4	hsa-miR-300
NUDT4	hsa-miR-4795-3p
NUDT4	hsa-miR-202-3p
NUDT4	hsa-miR-335-5p
NUDT4	hsa-miR-6723-5p
NUDT4	hsa-miR-597-5p
NUDT4	hsa-miR-1662
NUDT4	hsa-miR-1233-5p
NUDT4	hsa-miR-4693-5p
NUDT4	hsa-miR-8809
NUDT4	hsa-miR-7078-5p
NUDT4	hsa-miR-2307
NUDT4	hsa-miR-871-5p
NUDT4	hsa-miR-6877-3p

NUDT4	hsa-miR-351-3p
NUDT4	hsa-miR-301
NUDT4	hsa-miR-3180-3p
NUDT4	hsa-miR-670-3p
NUDT4	hsa-miR-6388
NUDT4	hsa-miR-466mo-3p
NUDT4	hsa-miR-122
NUDT4	hsa-miR-2439-5p
NUDT4	hsa-miR-4777-3p
NUDT4	hsa-miR-8908e
NUDT4	hsa-miR-5000-5p
NUDT4	hsa-miR-8843
NUDT4	hsa-miR-219a-1-3p
NUDT4	hsa-miR-1254
NUDT4	hsa-miR-3612
NUDT4	hsa-miR-30ad-3p
NUDT4	hsa-miR-7018-3p
NUDT4	hsa-miR-4766-3p
NUDT4	hsa-miR-1299
NUDT4	hsa-miR-195-5p
NUDT4	hsa-miR-7189-3p
NUDT4	hsa-miR-219a-5p
NUDT4	hsa-miR-2422
NUDT4	hsa-miR-374
NUDT4	hsa-miR-4744
NUDT4	hsa-miR-362-3p
NUDT4	hsa-miR-218-5p
NUDT4	hsa-miR-455-3p
NUDT4	hsa-miR-7160-3p
NUDT4	hsa-miR-4750-3p
NUDT4	hsa-miR-1250-3p
NUDT4	hsa-miR-6744-5p
NUDT4	hsa-miR-103
NUDT4	hsa-miR-7332-5p
NUDT4	hsa-miR-6416-3p
NUDT4	hsa-miR-1343-5p
NUDT4	hsa-miR-875-3p
NUDT4	hsa-miR-496a-3p.1
NUDT4	hsa-miR-1206
NUDT4	hsa-miR-1275
NUDT4	hsa-miR-31-5p
NUDT4	hsa-miR-6541
NUDT4	hsa-miR-6133
NUDT4	hsa-miR-6899-3p
NUDT4	hsa-miR-155-5p
NUDT4	hsa-miR-1938

NUDT4	hsa-miR-520c-3p
NUDT4	hsa-miR-5128
NUDT4	hsa-miR-2485
NUDT4	hsa-miR-9-5p
NUDT4	hsa-miR-1838
NUDT4	hsa-miR-411a
NUDT4	hsa-miR-655-5p
NUDT4	hsa-miR-4668-3p
NUDT4	hsa-miR-8074
NUDT4	hsa-miR-1906
NUDT4	hsa-miR-2298
NUDT4	hsa-miR-8085
NUDT4	hsa-miR-1252-3p
NUDT4	hsa-miR-6796-5p
NUDT4	hsa-miR-7235-5p
NUDT4	hsa-miR-2284q
NUDT4	hsa-miR-3156-3p
NUDT4	hsa-miR-1193-5p
NUDT4	hsa-miR-3714
NUDT4	hsa-miR-6719-3p
NUDT4	hsa-miR-4302
NUDT4	hsa-miR-766-3p
NUDT4	hsa-miR-380-3p
NUDT4	hsa-miR-3076-5p
NUDT4	hsa-miR-196c-3p
NUDT4	hsa-miR-514a-3p
NUDT4	hsa-miR-363-5p
NUDT4	hsa-miR-8067
NUDT4	hsa-miR-3120-3p
NUDT4	hsa-miR-467-3p
NUDT4	hsa-miR-4461
NUDT4	hsa-miR-8066
NUDT4	hsa-miR-1224
NUDT4	hsa-miR-6917-3p
NUDT4	hsa-miR-3934-5p
NUDT4	hsa-miR-466b-4-3p
NUDT4	hsa-miR-2895
NUDT4	hsa-miR-874-3p
NUDT4	hsa-miR-7173-5p
NUDT4	hsa-miR-376a-1-5p
NUDT4	hsa-miR-7150
NUDT4	hsa-miR-939-5p
NUDT4	hsa-miR-3151
NUDT4	hsa-miR-1827
NUDT4	hsa-miR-3622b-5p
NUDT4	hsa-miR-6960-5p

NUDT4	hsa-miR-2399-5p
NUDT4	hsa-miR-4291
NUDT4	hsa-miR-8070
NUDT4	hsa-miR-8862
NUDT4	hsa-miR-3606-3p
NUDT4	hsa-miR-4430
NUDT4	hsa-miR-7006-3p
NUDT4	hsa-miR-101-3p
NUDT4	hsa-miR-5087
NUDT4	hsa-miR-7093-3p
NUDT4	hsa-miR-516
NUDT4	hsa-miR-6944-3p
NUDT4	hsa-miR-7168-3p
NUDT4	hsa-miR-3060-3p
NUDT4	hsa-miR-4423
NUDT4	hsa-miR-296-5p
NUDT4	hsa-miR-4477b
NUDT4	hsa-miR-6906-5p
NUDT4	hsa-miR-132-3p
NUDT4	hsa-let-7f-2-3p
NUDT4	hsa-miR-222-3p
NUDT4	hsa-miR-204-5p
NUDT4	hsa-miR-612
NUDT4	hsa-miR-2461-5p
NUDT4	hsa-miR-4728-3p
NUDT4	hsa-miR-3919
NUDT4	hsa-miR-3151-5p
NUDT4	hsa-miR-7328-3p
NUDT4	hsa-miR-29a-1-5p
NUDT4	hsa-miR-496
NUDT4	hsa-miR-2326
NUDT4	hsa-miR-5120
NUDT4	hsa-miR-3065-5p
NUDT4	hsa-miR-373-3p
NUDT4	hsa-miR-4424
NUDT4	hsa-miR-1681
NUDT4	hsa-miR-3475-3p
NUDT4	hsa-miR-146a-3p
NUDT4	hsa-miR-18a-3p
NUDT4	hsa-miR-9-5p
NUDT4	hsa-miR-6952-5p
NUDT4	hsa-miR-548
NUDT4	hsa-miR-449-5p
NUDT4	hsa-miR-3121-5p
NUDT4	hsa-miR-2116-3p
NUDT4	hsa-miR-6976-3p

NUDT4	hsa-miR-203-3p.1
NUDT4	hsa-miR-135a-1-3p
NUDT4	hsa-miR-330-3p.1
NUDT4	hsa-miR-702-5p
NUDT4	hsa-miR-487-3p
NUDT4	hsa-miR-683
NUDT4	hsa-miR-3671
NUDT4	hsa-miR-503-5p
NUDT4	hsa-miR-197-3p
NUDT4	hsa-miR-6756-5p
NUDT4	hsa-miR-661
NUDT4	hsa-miR-7660-3p
NUDT4	hsa-miR-5699-3p
NUDT4	hsa-miR-1249-5p
NUDT4	hsa-miR-149-3p
NUDT4	hsa-miR-7316-5p
NUDT4	hsa-miR-583
NUDT4	hsa-miR-378j
NUDT4	hsa-miR-1247-3p
NUDT4	hsa-miR-8890
NUDT4	hsa-miR-570-5p
NUDT4	hsa-miR-7045-3p
NUDT4	hsa-miR-365-3p
NUDT4	hsa-miR-4714-3p
NUDT4	hsa-miR-7194-3p
NUDT4	hsa-miR-21-3p
NUDT4	hsa-miR-2448-3p
NUDT4	hsa-miR-876
NUDT4	hsa-miR-5186
NUDT4	hsa-miR-891a-3p
NUDT4	hsa-miR-6123
NUDT4	hsa-miR-4519
NUDT4	hsa-miR-7256-3p
NUDT4	hsa-miR-7394b-5p
NUDT4	hsa-miR-2475
NUDT4	hsa-miR-4666b
NUDT4	hsa-miR-3976
NUDT4	hsa-miR-5189-5p
NUDT4	hsa-miR-3937
NUDT4	hsa-miR-7398s-5p
NUDT4	hsa-miR-1185-3p
NUDT4	hsa-miR-7275-5p
NUDT4	hsa-miR-4709-5p
NUDT4	hsa-miR-4506
NUDT4	hsa-miR-1983
NUDT4	hsa-miR-7326-5p

NUDT4	hsa-miR-219b-3p
NUDT4	hsa-miR-890-5p
NUDT4	hsa-miR-1296-5p
NUDT4	hsa-miR-122-5p
NUDT4	hsa-let-7a-2-3p
NUDT4	hsa-miR-1964-5p
NUDT4	hsa-miR-490-3p
NUDT4	hsa-miR-545-3p
NUDT4	hsa-miR-2897
NUDT4	hsa-miR-6855-5p
NUDT4	hsa-miR-8826
NUDT4	hsa-miR-4727-3p
NUDT4	hsa-miR-6718-5p
NUDT4	hsa-miR-216b-3p
NUDT4	hsa-miR-150-5p
NUDT4	hsa-miR-376c
NUDT4	hsa-miR-548g-3p
NUDT4	hsa-miR-5689
NUDT4	hsa-miR-500-5p
NUDT4	hsa-miR-222-3p
NUDT4	hsa-miR-6846-5p
NUDT4	hsa-miR-548ah-3p
NUDT4	hsa-miR-425-5p
NUDT4	hsa-miR-2380
NUDT4	hsa-miR-7861
NUDT4	hsa-miR-185-5p
NUDT4	hsa-miR-1825
NUDT4	hsa-miR-7352-3p
NUDT4	hsa-miR-15
NUDT4	hsa-miR-7297-5p
NUDT4	hsa-miR-185-5p
NUDT4	hsa-miR-7248-5p
NUDT4	hsa-miR-6951-3p
NUDT4	hsa-miR-2319
NUDT4	hsa-miR-4447
NUDT4	hsa-miR-6127
NUDT4	hsa-miR-344i
NUDT4	hsa-miR-515
NUDT4	hsa-miR-6922-3p
NUDT4	hsa-miR-3672
NUDT4	hsa-miR-218a-2-3p
NUDT4	hsa-miR-382-5p
NUDT4	hsa-miR-423-5p
NUDT4	hsa-miR-573
NUDT4	hsa-miR-7374a-3p
NUDT4	hsa-miR-7024-5p

NUDT4	hsa-miR-2349
NUDT4	hsa-miR-7461-3p
NUDT4	hsa-miR-7277-5p
NUDT4	hsa-miR-5681a
NUDT4	hsa-miR-575
NUDT4	hsa-miR-4297
NUDT4	hsa-miR-658
NUDT4	hsa-miR-516b
NUDT4	hsa-miR-519e-3p
NUDT4	hsa-miR-6762-5p
NUDT4	hsa-miR-1285-5p
NUDT4	hsa-miR-433-5p
NUDT4	hsa-miR-6876-5p
NUDT4	hsa-miR-7317-3p
NUDT4	hsa-miR-2469
NUDT4	hsa-miR-511-5p
NUDT4	hsa-miR-4677-3p
NUDT4	hsa-miR-4746-3p
NUDT4	hsa-miR-3110-5p
NUDT4	hsa-miR-3574
NUDT4	hsa-miR-7047-5p
NUDT4	hsa-miR-2452
NUDT4	hsa-miR-592
NUDT4	hsa-miR-3973
NUDT4	hsa-miR-7b-3p
NUDT4	hsa-miR-7279-3p
NUDT4	hsa-miR-6819-3p
NUDT4	hsa-miR-124-3p.2
NUDT4	hsa-miR-4426
NUDT4	hsa-miR-548cej-3p
NUDT4	hsa-miR-433
NUDT4	hsa-miR-6742-3p
NUDT4	hsa-miR-4446-5p
NUDT4	hsa-miR-6811-3p
NUDT4	hsa-miR-133b-5p
NUDT4	hsa-miR-16-3p
NUDT4	hsa-miR-7673-3p
NUDT4	hsa-miR-4735-5p
NUDT4	hsa-miR-3551-5p
NUDT4	hsa-miR-944
NUDT4	hsa-miR-6923-5p
NUDT4	hsa-miR-5616-3p
NUDT4	hsa-miR-1288-3p
NUDT4	hsa-miR-1207-3p
NUDT4	hsa-miR-707
NUDT4	hsa-miR-608

NUDT4	hsa-miR-219b-5p
NUDT4	hsa-miR-548k
NUDT4	hsa-miR-1950
NUDT4	hsa-miR-3087-3p
NUDT4	hsa-miR-1947-5p
NUDT4	hsa-miR-148-5p
NUDT4	hsa-miR-6881-5p
NUDT4	hsa-miR-466l-3p
NUDT4	hsa-miR-340-3p
NUDT4	hsa-miR-470-5p
NUDT4	hsa-miR-1273f
NUDT4	hsa-miR-544a
NUDT4	hsa-miR-3128
NUDT4	hsa-miR-1276
NUDT4	hsa-miR-7369-3p
NUDT4	hsa-let-7a-3p
NUDT4	hsa-miR-4648
NUDT4	hsa-miR-135-5p
NUDT4	hsa-miR-6401
NUDT4	hsa-miR-3106-3p
NUDT4	hsa-miR-4796-5p
NUDT4	hsa-miR-3675-3p
NUDT4	hsa-miR-4789-5p
NUDT4	hsa-miR-4502
NUDT4	hsa-miR-133
NUDT4	hsa-miR-6794-5p
NUDT4	hsa-miR-106
NUDT4	hsa-miR-140-5p
NUDT4	hsa-miR-651-5p
NUDT4	hsa-miR-7166-3p
NUDT4	hsa-miR-8072
NUDT4	hsa-miR-664a-3p
NUDT4	hsa-miR-153-3p
NUDT4	hsa-miR-4782-5p
NUDT4	hsa-miR-125-5p
NUDT4	hsa-miR-3123
NUDT4	hsa-miR-103-3p
NUDT4	hsa-miR-200c-3p
NUDT4	hsa-miR-6747-5p
NUDT4	hsa-miR-7350-5p
NUDT4	hsa-miR-1230
NUDT4	hsa-miR-4293
NUDT4	hsa-miR-4777-5p
NUDT4	hsa-miR-7335-3p
NUDT4	hsa-miR-7354-3p
NUDT4	hsa-miR-2461-3p

NUDT4	hsa-miR-3667-5p
NUDT4	hsa-miR-456-5p
NUDT4	hsa-miR-548b-5p
NUDT4	hsa-miR-4475
NUDT4	hsa-miR-2479
NUDT4	hsa-miR-6867-3p
NUDT4	hsa-miR-374c-3p
NUDT4	hsa-miR-7028-5p
NUDT4	hsa-miR-485-5p
NUDT4	hsa-miR-7396-5p
NUDT4	hsa-miR-2284b
NUDT4	hsa-miR-324
NUDT4	hsa-miR-30b-3p
NUDT4	hsa-miR-5099
NUDT4	hsa-miR-556-5p
NUDT4	hsa-miR-3150a-3p
NUDT4	hsa-miR-6961-3p
NUDT4	hsa-miR-105b
NUDT4	hsa-miR-6830-3p
NUDT4	hsa-miR-642-3p
NUDT4	hsa-miR-6776-5p
NUDT4	hsa-miR-591
NUDT4	hsa-miR-4799-3p
NUDT4	hsa-miR-548c-3p
NUDT4	hsa-miR-6878-5p
NUDT4	hsa-miR-4253
NUDT4	hsa-miR-3200-5p
NUDT4	hsa-miR-8084
NUDT4	hsa-miR-7235-3p
NUDT4	hsa-miR-632
NUDT4	hsa-let-7-3p
NUDT4	hsa-miR-8054
NUDT4	hsa-miR-6819-5p
NUDT4	hsa-miR-125
NUDT4	hsa-miR-7203-5p
NUDT4	hsa-miR-134-3p
NUDT4	hsa-miR-6414
NUDT4	hsa-miR-7327-5p
NUDT4	hsa-miR-3646
NUDT4	hsa-miR-4711-5p
NUDT4	hsa-miR-10-5p
NUDT4	hsa-miR-2285z
NUDT4	hsa-miR-519-3p
NUDT4	hsa-miR-887
NUDT4	hsa-miR-205-5p
NUDT4	hsa-miR-186-5p

NUDT4	hsa-miR-513a-3p
NUDT4	hsa-miR-411-5p
NUDT4	hsa-miR-218-5p
NUDT4	hsa-miR-6355
NUDT4	hsa-miR-6508-5p
NUDT4	hsa-miR-548aq-5p
NUDT4	hsa-miR-552-5p
NUDT4	hsa-miR-6720-5p
NUDT4	hsa-miR-25-5p
NUDT4	hsa-miR-128
NUDT4	hsa-miR-146-5p
NUDT4	hsa-miR-383-3p
NUDT4	hsa-miR-8083
NUDT4	hsa-miR-4504
NUDT4	hsa-miR-22-3p
NUDT4	hsa-miR-450a
NUDT4	hsa-miR-7244-3p
NUDT4	hsa-miR-126-5p
NUDT4	hsa-miR-7977
NUDT4	hsa-miR-669aflp-5p
NUDT4	hsa-miR-2311
NUDT4	hsa-miR-1544-5p
NUDT4	hsa-miR-7073-5p
NUDT4	hsa-miR-7289-3p
NUDT4	hsa-miR-2285b
NUDT4	hsa-miR-7200-5p
NUDT4	hsa-miR-4420
NUDT4	hsa-miR-6080
NUDT4	hsa-miR-1205
NUDT4	hsa-miR-3075-3p
NUDT4	hsa-miR-7154-3p
NUDT4	hsa-miR-6771-5p
NUDT4	hsa-miR-298
NUDT4	hsa-miR-574-3p
NUDT4	hsa-miR-606
NUDT4	hsa-miR-450b-5p
NUDT4	hsa-miR-8892
NUDT4	hsa-miR-208-5p
NUDT4	hsa-miR-7341-5p
NUDT4	hsa-miR-571
NUDT4	hsa-miR-17-5p
NUDT4	hsa-miR-5588-3p
NUDT4	hsa-miR-379-5p
NUDT4	hsa-miR-96-5p
NUDT4	hsa-miR-190-3p
NUDT4	hsa-miR-3064-5p

NUDT4	hsa-miR-4307
NUDT4	hsa-miR-495-3p
NUDT4	hsa-miR-873-3p
NUDT4	hsa-miR-3146
NUDT4	hsa-miR-6842-3p
NUDT4	hsa-miR-7119-5p
NUDT4	hsa-miR-7116-3p
NUDT4	hsa-miR-561-3p
NUDT4	hsa-miR-1260a
NUDT4	hsa-miR-4698
NUDT4	hsa-miR-6906-3p
NUDT4	hsa-miR-1194
NUDT4	hsa-miR-135a-5p
NUDT4	hsa-miR-890
NUDT4	hsa-miR-5114
NUDT4	hsa-miR-8904b
NUDT4	hsa-miR-671-5p
NUDT4	hsa-miR-6949-5p
NUDT4	hsa-miR-6331
NUDT4	hsa-let-7c-3p
NUDT4	hsa-miR-3101-3p
NUDT4	hsa-miR-8807
NUDT4	hsa-miR-6513-3p
NUDT4	hsa-miR-6344
NUDT4	hsa-miR-3082-5p
NUDT4	hsa-miR-320b
NUDT4	hsa-miR-3577-5p
NUDT4	hsa-miR-191-5p
NUDT4	hsa-miR-1227-3p
NUDT4	hsa-miR-7059-3p
NUDT4	hsa-miR-499b-3p
NUDT4	hsa-miR-506-3p
NUDT4	hsa-miR-6815-5p
NUDT4	hsa-miR-4421
NUDT4	hsa-miR-488-3p
NUDT4	hsa-miR-7274-5p
NUDT4	hsa-miR-6988-3p
NUDT4	hsa-miR-6756-3p
NUDT4	hsa-miR-4455
NUDT4	hsa-miR-7051-3p
NUDT4	hsa-miR-3670
NUDT4	hsa-miR-2284o
NUDT4	hsa-miR-628-5p
NUDT4	hsa-miR-2917
NUDT4	hsa-miR-302-5p
NUDT4	hsa-miR-6779-3p

NUDT4	hsa-miR-182-5p
NUDT4	hsa-miR-130b-5p
NUDT4	hsa-miR-219-1-3p
NUDT4	hsa-miR-7647-3p
NUDT4	hsa-miR-140-3p.2
NUDT4	hsa-miR-2335
NUDT4	hsa-miR-3575
NUDT4	hsa-miR-3691-3p
NUDT4	hsa-miR-942-3p
NUDT4	hsa-miR-6323
NUDT4	hsa-miR-2285n
NUDT4	hsa-miR-615-3p
NUDT4	hsa-miR-7026-3p
NUDT4	hsa-miR-4318
NUDT4	hsa-miR-16-5p
NUDT4	hsa-miR-5581-5p
NUDT4	hsa-miR-136-5p
NUDT4	hsa-miR-3169
NUDT4	hsa-miR-6996-3p
NUDT4	hsa-miR-7236-3p
NUDT4	hsa-miR-485
NUDT4	hsa-miR-653
NUDT4	hsa-miR-4701-3p
NUDT4	hsa-miR-743-5p
NUDT4	hsa-miR-4477a
NUDT4	hsa-miR-6731-5p
NUDT4	hsa-miR-3163
NUDT4	hsa-miR-2328-5p
NUDT4	hsa-miR-106a-3p
NUDT4	hsa-miR-6764-5p
NUDT4	hsa-miR-6481
NUDT4	hsa-miR-629-3p
NUDT4	hsa-miR-551-5p
NUDT4	hsa-miR-1812-3p
NUDT4	hsa-miR-4523
NUDT4	hsa-miR-572
NUDT4	hsa-miR-3094-3p
NUDT4	hsa-miR-548c-3p
NUDT4	hsa-miR-30e
NUDT4	hsa-miR-154-3p
NUDT4	hsa-miR-2483
NUDT4	hsa-miR-7272-3p
NUDT4	hsa-miR-7021-5p
NUDT4	hsa-miR-8818
NUDT4	hsa-miR-138-1-3p
NUDT4	hsa-miR-4459

NUDT4	hsa-miR-7047-3p
NUDT4	hsa-miR-5006-3p
NUDT4	hsa-miR-548b-3p
NUDT4	hsa-miR-2482
NUDT4	hsa-miR-339-5p
NUDT4	hsa-miR-7680-3p
NUDT4	hsa-miR-877-3p
NUDT4	hsa-miR-7252-3p
NUDT4	hsa-miR-548aj-3p
NUDT4	hsa-miR-548bb-5p
NUDT4	hsa-miR-196b-3p
NUDT4	hsa-miR-2447
NUDT4	hsa-miR-151
NUDT4	hsa-miR-7110-5p
NUDT4	hsa-miR-7862
NUDT4	hsa-miR-6131
NUDT4	hsa-miR-361-3p
NUDT4	hsa-miR-487a
NUDT4	hsa-miR-2489
NUDT4	hsa-miR-7169-5p
NUDT4	hsa-miR-322-5p
NUDT4	hsa-miR-524-5p
NUDT4	hsa-miR-192-3p
NUDT4	hsa-miR-548aef
NUDT4	hsa-miR-4720-5p
NUDT4	hsa-miR-6083
NUDT4	hsa-miR-7365-3p
NUDT4	hsa-miR-183-3p
NUDT4	hsa-miR-7161-5p
NUDT4	hsa-miR-4266
NUDT4	hsa-miR-7023-3p
NUDT4	hsa-miR-294-3p
NUDT4	hsa-miR-7019-3p
NUDT4	hsa-miR-4311
NUDT4	hsa-miR-3113-3p
NUDT4	hsa-miR-582-5p
NUDT4	hsa-miR-2284a
NUDT4	hsa-miR-2330-3p
NUDT4	hsa-miR-3561-5p
NUDT4	hsa-miR-651
NUDT4	hsa-miR-1643b-5p
NUDT4	hsa-miR-2384
NUDT4	hsa-miR-346
NUDT4	hsa-miR-718
NUDT4	hsa-miR-6845-5p
NUDT4	hsa-miR-674-5p

NUDT4	hsa-miR-5195-5p
NUDT4	hsa-miR-190a-3p
NUDT4	hsa-miR-501-5p
NUDT4	hsa-miR-432-5p
NUDT4	hsa-miR-29
NUDT4	hsa-miR-2285x
NUDT4	hsa-miR-873b
NUDT4	hsa-miR-1277-5p
NUDT4	hsa-miR-548c-5p
NUDT4	hsa-miR-518
NUDT4	hsa-miR-544a
NUDT4	hsa-miR-125b-2-3p
NUDT4	hsa-miR-6801-5p
NUDT4	hsa-miR-4512
NUDT4	hsa-miR-6340
NUDT4	hsa-miR-20-3p
NUDT4	hsa-miR-30
NUDT4	hsa-miR-92a-1-5p
NUDT4	hsa-miR-892a
NUDT4	hsa-miR-33-3p
NUDT4	hsa-miR-4716-3p
NUDT4	hsa-miR-409-5p
NUDT4	hsa-miR-550
NUDT4	hsa-miR-10b-3p
NUDT4	hsa-miR-7025-3p
NUDT4	hsa-miR-20
NUDT4	hsa-miR-2385-3p
NUDT4	hsa-miR-548d-5p
NUDT4	hsa-miR-344a-3p
NUDT4	hsa-miR-708-5p
NUDT4	hsa-miR-2296
NUDT4	hsa-miR-4723-3p
NUDT4	hsa-miR-3140-3p
NUDT4	hsa-miR-3674
NUDT4	hsa-miR-545-5p
NUDT4	hsa-miR-7094b-2-5p
NUDT4	hsa-miR-6732-3p
NUDT4	hsa-miR-4717-3p
NUDT4	hsa-miR-140-5p
NUDT4	hsa-miR-409b
NUDT4	hsa-miR-599
NUDT4	hsa-miR-4684-5p
NUDT4	hsa-miR-504-5p
NUDT4	hsa-miR-1253
NUDT4	hsa-miR-1914-5p
NUDT4	hsa-miR-7976

NUDT4	hsa-miR-694
NUDT4	hsa-miR-766-5p
NUDT4	hsa-miR-6362
NUDT4	hsa-miR-6799-5p
NUDT4	hsa-miR-704
NUDT4	hsa-miR-8868
NUDT4	hsa-miR-6942-5p
NUDT4	hsa-miR-548az-3p
NUDT4	hsa-miR-4419b
NUDT4	hsa-miR-520g-3p
NUDT4	hsa-miR-18b-3p
NUDT4	hsa-miR-126a-5p
NUDT4	hsa-miR-686
NUDT4	hsa-miR-892b
NUDT4	hsa-miR-7457-5p
NUDT4	hsa-miR-485-3p
NUDT4	hsa-miR-182-3p
NUDT4	hsa-miR-7348-5p
NUDT4	hsa-miR-548a-5p
NUDT4	hsa-miR-212-3p
NUDT4	hsa-miR-199-5p
NUDT4	hsa-miR-6516-3p
NUDT4	hsa-miR-28-3p
NUDT4	hsa-miR-4524a-5p
NUDT4	hsa-miR-7112-3p
NUDT4	hsa-miR-7320-5p
NUDT4	hsa-miR-425
NUDT4	hsa-miR-4724-3p
NUDT4	hsa-miR-520
NUDT4	hsa-miR-101-5p
NUDT4	hsa-miR-216a
NUDT4	hsa-miR-6758-3p
NUDT4	hsa-miR-7222-5p
NUDT4	hsa-miR-3173-5p
NUDT4	hsa-miR-4695-3p
NUDT4	hsa-miR-4427
NUDT4	hsa-miR-3059-3p
NUDT4	hsa-miR-2373-3p
NUDT4	hsa-miR-510-3p
NUDT4	hsa-miR-3100-3p
NUDT4	hsa-miR-152-5p
NUDT4	hsa-miR-663a
NUDT4	hsa-miR-3473g
NUDT4	hsa-miR-6757-3p
NUDT4	hsa-miR-6380
NUDT4	hsa-miR-6515-5p

NUDT4	hsa-miR-8873a
NUDT4	hsa-miR-765
NUDT4	hsa-miR-30-5p
NUDT4	hsa-miR-16-2-3p
NUDT4	hsa-miR-135b-5p
NUDT4	hsa-miR-653-3p
NUDT4	hsa-miR-4288
NUDT4	hsa-miR-519
NUDT4	hsa-miR-4322
NUDT4	hsa-miR-465c-5p
NUDT4	hsa-miR-203b-5p
NUDT4	hsa-miR-505-3p
NUDT4	hsa-miR-1285-3p
NUDT4	hsa-miR-7296-3p
NUDT4	hsa-miR-197-3p
NUDT4	hsa-miR-6895-3p
NUDT4	hsa-miR-1927
NUDT4	hsa-miR-33
NUDT4	hsa-miR-660-5p
NUDT4	hsa-let-7abf-3p
NUDT4	hsa-miR-889
NUDT4	hsa-miR-7310-5p
NUDT4	hsa-miR-942-5p
NUDT4	hsa-miR-7170-5p
NUDT4	hsa-miR-301-5p
NUDT4	hsa-miR-380
NUDT4	hsa-miR-549a
NUDT4	hsa-miR-4296
NUDT4	hsa-miR-200b-3p
NUDT4	hsa-miR-548l
NUDT4	hsa-miR-769-5p
NUDT4	hsa-miR-20b-3p
NUDT4	hsa-miR-7398a-3p
NUDT4	hsa-miR-4488
NUDT4	hsa-miR-6733-3p
NUDT4	hsa-miR-1612
NUDT4	hsa-miR-450a-2-3p
NUDT4	hsa-miR-5010-3p
NUDT4	hsa-miR-586
NUDT4	hsa-miR-526
NUDT4	hsa-miR-7074-5p
NUDT4	hsa-miR-5684
NUDT4	hsa-miR-7151-5p
NUDT4	hsa-miR-550a-5p
NUDT4	hsa-miR-489
NUDT4	hsa-miR-548au-5p

NUDT4	hsa-miR-548ag
NUDT4	hsa-miR-3558-5p
NUDT4	hsa-miR-6500-3p
NUDT4	hsa-miR-489-3p
NUDT4	hsa-miR-4476
NUDT4	hsa-miR-7084-3p
NUDT4	hsa-miR-363-3p
NUDT4	hsa-miR-7109-3p
NUDT4	hsa-miR-672-3p
NUDT4	hsa-miR-7331-5p
NUDT4	hsa-miR-1267
NUDT4	hsa-miR-5618-5p
NUDT4	hsa-miR-23-5p
NUDT4	hsa-miR-124
NUDT4	hsa-miR-374-5p
NUDT4	hsa-miR-188-3p
NUDT4	hsa-miR-939-5p
NUDT4	hsa-miR-92-3p
NUDT4	hsa-miR-669k-5p
NUDT4	hsa-miR-887-5p
NUDT4	hsa-miR-342-5p
NUDT4	hsa-miR-679
NUDT4	hsa-miR-10a-3p
NUDT4	hsa-miR-1912
NUDT4	hsa-miR-7192-5p
NUDT4	hsa-miR-3099-3p
NUDT4	hsa-miR-7650-3p
NUDT4	hsa-miR-762
NUDT4	hsa-miR-7351-3p
NUDT4	hsa-miR-2285j
NUDT4	hsa-miR-7232-5p
NUDT4	hsa-miR-4719
NUDT4	hsa-miR-3609
NUDT4	hsa-miR-7178-3p
NUDT4	hsa-miR-698-5p
NUDT4	hsa-miR-7360-5p
NUDT4	hsa-miR-8550
NUDT4	hsa-miR-126-3p
NUDT4	hsa-miR-335-3p
NUDT4	hsa-miR-3692-5p
NUDT4	hsa-miR-761
NUDT4	hsa-miR-692
NUDT4	hsa-miR-4705
NUDT4	hsa-miR-3913-3p
NUDT4	hsa-miR-6998-5p
NUDT4	hsa-miR-3183

NUDT4	hsa-miR-2113
NUDT4	hsa-miR-4470
NUDT4	hsa-miR-2424
NUDT4	hsa-miR-3596c
NUDT4	hsa-miR-8104
NUDT4	hsa-miR-4795-5p
NUDT4	hsa-miR-7332-3p
NUDT4	hsa-miR-6086
NUDT4	hsa-miR-338-3p
NUDT4	hsa-miR-548o-5p
NUDT4	hsa-miR-410
NUDT4	hsa-miR-1185-5p
NUDT4	hsa-miR-605-5p
NUDT4	hsa-miR-3098-3p
NUDT4	hsa-miR-7281-3p
NUDT4	hsa-miR-7251-5p
NUDT4	hsa-miR-1264-3p
NUDT4	hsa-miR-5132-3p
NUDT4	hsa-miR-429-5p
NUDT4	hsa-miR-548at-5p
NUDT4	hsa-miR-7677-5p
NUDT4	hsa-miR-580
NUDT4	hsa-miR-7a-2-3p
NUDT4	hsa-miR-302c-5p
NUDT4	hsa-miR-18
NUDT4	hsa-miR-16-5p
NUDT4	hsa-miR-6128
NUDT4	hsa-miR-327
NUDT4	hsa-miR-513b-3p
NUDT4	hsa-miR-4287
NUDT4	hsa-miR-2466-5p
NUDT4	hsa-let-7f-1-3p
NUDT4	hsa-miR-4325
NUDT4	hsa-miR-6907-5p
NUDT4	hsa-miR-129-5p
NUDT4	hsa-miR-1224-3p
NUDT4	hsa-miR-6867-5p
NUDT4	hsa-miR-624-5p
NUDT4	hsa-miR-1255b-5p
NUDT4	hsa-miR-8816
NUDT4	hsa-miR-124-3p.1
NUDT4	hsa-miR-452-5p
NUDT4	hsa-miR-101-2-5p
NUDT4	hsa-miR-200b-5p
NUDT4	hsa-miR-3088-5p
NUDT4	hsa-miR-551b-5p

NUDT4	hsa-miR-1231
NUDT4	hsa-miR-570-3p
NUDT4	hsa-miR-7068-3p
NUDT4	hsa-miR-935
NUDT4	hsa-miR-6946-3p
NUDT4	hsa-miR-1245b-3p
NUDT4	hsa-miR-8802
NUDT4	hsa-miR-376a-5p
NUDT4	hsa-miR-7269-5p
NUDT4	hsa-miR-3975
NUDT4	hsa-miR-7294-5p
NUDT4	hsa-miR-7215-5p
NUDT4	hsa-miR-195-5p
NUDT4	hsa-miR-522-3p
NUDT4	hsa-miR-555
NUDT4	hsa-miR-7023-5p
NUDT4	hsa-miR-3190-3p
NUDT4	hsa-miR-770
NUDT4	hsa-miR-4684-3p
NUDT4	hsa-miR-452-3p
NUDT4	hsa-miR-3074-2-3p
NUDT4	hsa-miR-3150a-5p
NUDT4	hsa-miR-513
NUDT4	hsa-miR-544-5p
NUDT4	hsa-miR-7648-3p
NUDT4	hsa-miR-3175
NUDT4	hsa-miR-3134
NUDT4	hsa-miR-4673
NUDT4	hsa-miR-1258
NUDT4	hsa-miR-376e
NUDT4	hsa-miR-4999-5p
NUDT4	hsa-miR-7106-5p
NUDT4	hsa-miR-548am-5p
NUDT4	hsa-miR-1584-3p
NUDT4	hsa-miR-4540
NUDT4	hsa-miR-5110
NUDT4	hsa-miR-7863
NUDT4	hsa-miR-338-5p
NUDT4	hsa-miR-1915-3p
NUDT4	hsa-miR-548i
NUDT4	hsa-miR-6514-3p
NUDT4	hsa-miR-1290
NUDT4	hsa-miR-7188-3p
NUDT4	hsa-miR-3191-5p
NUDT4	hsa-miR-539-5p
NUDT4	hsa-miR-3065-3p

NUDT4	hsa-miR-7330-3p
NUDT4	hsa-miR-548h-5p
NUDT4	hsa-miR-6893-5p
NUDT4	hsa-miR-3129-5p
NUDT4	hsa-miR-548y
NUDT4	hsa-miR-6970-5p
NUDT4	hsa-miR-8806
NUDT4	hsa-miR-8880
NUDT4	hsa-miR-449c-3p
NUDT4	hsa-miR-548j-3p
NUDT4	hsa-miR-2371
NUDT4	hsa-miR-1896
NUDT4	hsa-miR-7238-3p
NUDT4	hsa-miR-763
NUDT4	hsa-miR-96-3p
NUDT4	hsa-miR-516b-5p
NUDT4	hsa-miR-450a-1-3p
NUDT4	hsa-miR-7213-5p
NUDT4	hsa-miR-522-3p
NUDT4	hsa-miR-885-3p
NUDT4	hsa-miR-875-5p
NUDT4	hsa-miR-24-3p
NUDT4	hsa-miR-23a-5p
NUDT4	hsa-miR-2352
NUDT4	hsa-miR-4480
NUDT4	hsa-miR-8814
NUDT4	hsa-miR-6943-5p
NUDT4	hsa-miR-876-5p
NUDT4	hsa-miR-3145-3p
NUDT4	hsa-miR-335-5p
NUDT4	hsa-miR-181b-1-3p
NUDT4	hsa-miR-210
NUDT4	hsa-miR-344-5p
NUDT4	hsa-miR-4704-5p
NUDT4	hsa-miR-3152-3p
NUDT4	hsa-miR-450b-3p
NUDT4	hsa-miR-4727-5p
NUDT4	hsa-miR-126b-5p
NUDT4	hsa-miR-3560
NUDT4	hsa-miR-7024-3p
NUDT4	hsa-miR-4433b-3p
NUDT4	hsa-miR-7219-5p
NUDT4	hsa-miR-3925-5p
NUDT4	hsa-miR-4793-5p
NUDT4	hsa-miR-4679
NUDT4	hsa-miR-3588

NUDT4	hsa-miR-2356
NUDT4	hsa-miR-2284w
NUDT4	hsa-miR-550b-2-5p
NUDT4	hsa-miR-3692-3p
NUDT4	hsa-miR-26-2-3p
NUDT4	hsa-miR-7180-5p
NUDT4	hsa-miR-6988-5p
NUDT4	hsa-miR-4694-3p
NUDT4	hsa-miR-6746-5p
NUDT4	hsa-miR-548ai
NUDT4	hsa-miR-875
NUDT4	hsa-miR-1295b-3p
NUDT4	hsa-miR-743ab-3p
NUDT4	hsa-miR-101b-3p.2
NUDT4	hsa-miR-548ba
NUDT4	hsa-miR-4282
NUDT4	hsa-miR-5697-5p
NUDT4	hsa-miR-6737-5p
NUDT4	hsa-miR-377-5p
NUDT4	hsa-miR-644a
NUDT4	hsa-miR-7277-3p
NUDT4	hsa-miR-6371
NUDT4	hsa-miR-8801
NUDT4	hsa-miR-302d-5p
NUDT4	hsa-miR-127-5p
NUDT4	hsa-miR-3090-3p
NUDT4	hsa-miR-2320-5p
NUDT4	hsa-miR-1243-5p
NUDT4	hsa-miR-8797
NUDT4	hsa-miR-888-3p
NUDT4	hsa-miR-1542-5p
NUDT4	hsa-miR-181-3p
NUDT4	hsa-miR-2284z
NUDT4	hsa-miR-203a-3p.1
NUDT4	hsa-miR-7850-5p
NUDT4	hsa-miR-563
NUDT4	hsa-miR-6941-5p
NUDT4	hsa-miR-6403
NUDT4	hsa-miR-4790-3p
NUDT4	hsa-miR-7301-5p
NUDT4	hsa-miR-2284u
NUDT4	hsa-miR-7655-5p
NUDT4	hsa-miR-548m
NUDT4	hsa-miR-5580-3p
NUDT4	hsa-miR-138-3p
NUDT4	hsa-miR-7323-5p

NUDT4	hsa-miR-5093
NUDT4	hsa-miR-7163-5p
NUDT4	hsa-miR-3200-3p
NUDT4	hsa-miR-369-3p
NUDT4	hsa-miR-4731-3p
NUDT4	hsa-miR-367-3p
NUDT4	hsa-miR-7389-5p
NUDT4	hsa-miR-486-5p
NUDT4	hsa-miR-6736-5p
NUDT4	hsa-miR-135
NUDT4	hsa-miR-4760-5p
NUDT4	hsa-miR-6358
NUDT4	hsa-miR-1550-3p
NUDT4	hsa-miR-6956-3p
NUDT4	hsa-miR-30e-3p
NUDT4	hsa-miR-1190
NUDT4	hsa-miR-6780a-5p
NUDT4	hsa-miR-7287-3p
NUDT4	hsa-miR-1257
NUDT4	hsa-miR-337-3p
NUDT4	hsa-miR-3200-3p
NUDT4	hsa-miR-4263
NUDT4	hsa-miR-1954
NUDT4	hsa-miR-5002-5p
NUDT4	hsa-miR-8791
NUDT4	hsa-miR-6720-3p
NUDT4	hsa-miR-1271-5p
NUDT4	hsa-miR-7093-5p
NUDT4	hsa-miR-556-3p
NUDT4	hsa-miR-135a-3p
NUDT4	hsa-miR-3613-3p
NUDT4	hsa-miR-7360-3p
NUDT4	hsa-miR-936
NUDT4	hsa-miR-6372
NUDT4	hsa-miR-6071
NUDT4	hsa-miR-498
NUDT4	hsa-miR-6376
NUDT4	hsa-miR-3187-5p
NUDT4	hsa-miR-454
NUDT4	hsa-miR-7663-5p
NUDT4	hsa-miR-3122
NUDT4	hsa-miR-3092-3p
NUDT4	hsa-miR-4699-3p
NUDT4	hsa-miR-7114-5p
NUDT4	hsa-miR-4768-5p
NUDT4	hsa-miR-3533

NUDT4	hsa-miR-892-5p
NUDT4	hsa-miR-2346
NUDT4	hsa-miR-542-3p
NUDT4	hsa-miR-5617-3p
NUDT4	hsa-miR-8069
NUDT4	hsa-miR-548ay-5p
NUDT4	hsa-miR-519-5p
NUDT4	hsa-miR-6769b-5p
NUDT4	hsa-miR-503-5p
NUDT4	hsa-miR-7385b-3p
NUDT4	hsa-miR-4685-3p
NUDT4	hsa-miR-7089-3p
NUDT4	hsa-miR-2338
NUDT4	hsa-miR-6513-5p
NUDT4	hsa-miR-5683
NUDT4	hsa-miR-2488
NUDT4	hsa-miR-6905-3p
NUDT4	hsa-miR-1237-5p
NUDT4	hsa-miR-7090-3p
NUDT4	hsa-miR-1-3p
NUDT4	hsa-miR-7243-3p
NUDT4	hsa-miR-6759-5p
NUDT4	hsa-miR-6766-5p
NUDT4	hsa-miR-336-3p
NUDT4	hsa-miR-5706
NUDT4	hsa-miR-4769-5p
NUDT4	hsa-let-7b-3p
NUDT4	hsa-miR-7667-5p
NUDT4	hsa-miR-8107
NUDT4	hsa-miR-590-3p
NUDT4	hsa-miR-6916-5p
NUDT4	hsa-miR-7386i-5p
NUDT4	hsa-miR-6927-5p
NUDT4	hsa-miR-7386g-3p
NUDT4	hsa-miR-541-3p
NUDT4	hsa-miR-2313-5p
NUDT4	hsa-miR-508-5p
NUDT4	hsa-miR-7321-5p
NUDT4	hsa-miR-3105-3p
NUDT4	hsa-miR-29b-1-5p
NUDT4	hsa-miR-2285ad
NUDT4	hsa-miR-3188
NUDT4	hsa-miR-455-5p
NUDT4	hsa-miR-216
NUDT4	hsa-miR-6865-5p
NUDT4	hsa-miR-32-3p

NUDT4	hsa-miR-650abc
NUDT4	hsa-miR-1258-5p
NUDT4	hsa-miR-378-5p
NUDT4	hsa-miR-2396
NUDT4	hsa-miR-7682-3p
NUDT4	hsa-miR-92a-2-5p
NUDT4	hsa-miR-4796-3p
NUDT4	hsa-miR-153-5p
NUDT4	hsa-miR-6848-3p
NUDT4	hsa-miR-5100
NUDT4	hsa-miR-1966-3p
NUDT4	hsa-miR-3154
NUDT4	hsa-miR-741-3p
NUDT4	hsa-miR-940
NUDT4	hsa-miR-8060
NUDT4	hsa-miR-1b-5p
NUDT4	hsa-miR-3961
NUDT4	hsa-miR-8817
NUDT4	hsa-miR-4469
NUDT4	hsa-miR-6087
NUDT4	hsa-miR-4729
NUDT4	hsa-miR-221-3p
NUDT4	hsa-miR-101a-3p.2
NUDT4	hsa-miR-6827-5p
NUDT4	hsa-miR-574-5p
NUDT4	hsa-miR-4755-5p
NUDT4	hsa-miR-3529-3p
NUDT4	hsa-miR-7017-3p
NUDT4	hsa-miR-6809-3p
NUDT4	hsa-miR-460-5p
NUDT4	hsa-miR-4443
NUDT4	hsa-miR-4524-5p
NUDT4	hsa-miR-140-3p
NUDT4	hsa-miR-6132
NUDT4	hsa-miR-1-3p
NUDT4	hsa-miR-6805-5p
NUDT4	hsa-miR-4773
NUDT4	hsa-miR-7678-3p
NUDT4	hsa-miR-8061
NUDT4	hsa-miR-143-3p
NUDT4	hsa-miR-6536
NUDT4	hsa-miR-6771-3p
NUDT4	hsa-miR-6881-3p
NUDT4	hsa-miR-625-3p
NUDT4	hsa-miR-6895-5p
NUDT4	hsa-miR-6940-5p

NUDT4	hsa-miR-630
NUDT4	hsa-miR-4696
NUDT4	hsa-miR-3071-5p
NUDT4	hsa-miR-2322-3p
NUDT4	hsa-miR-7333-3p
NUDT4	hsa-miR-6520
NUDT4	hsa-miR-628-3p
NUDT4	hsa-miR-367-5p
NUDT4	hsa-miR-6332
NUDT4	hsa-miR-3552
NUDT4	hsa-miR-4651
NUDT4	hsa-miR-587
NUDT4	hsa-miR-2285u
NUDT4	hsa-miR-2317
NUDT4	hsa-miR-3945
NUDT4	hsa-miR-7651-3p
NUDT4	hsa-miR-381-5p
NUDT4	hsa-miR-4645-3p
NUDT4	hsa-miR-196a-3p
NUDT4	hsa-miR-599-3p
NUDT4	hsa-miR-3607-3p
NUDT4	hsa-miR-24-3p
NUDT4	hsa-miR-6862-5p
NUDT4	hsa-miR-371
NUDT4	hsa-miR-466c-5p
NUDT4	hsa-miR-219c-3p
NUDT4	hsa-miR-6335
NUDT4	hsa-miR-7080-5p
NUDT4	hsa-miR-4647
NUDT4	hsa-miR-1841
NUDT4	hsa-miR-4460
NUDT4	hsa-miR-4709-3p
NUDT4	hsa-miR-3164
NUDT4	hsa-miR-4666a-5p
NUDT4	hsa-miR-9768-3p
NUDT4	hsa-miR-290b-5p
NUDT4	hsa-miR-4419a
NUDT4	hsa-miR-133a-5p
NUDT4	hsa-miR-7350-3p
NUDT4	hsa-miR-1462-5p
NUDT4	hsa-miR-511
NUDT4	hsa-miR-4689
NUDT4	hsa-miR-6730-5p
NUDT4	hsa-miR-367-3p
NUDT4	hsa-miR-4438
NUDT4	hsa-miR-3062-5p

NUDT4	hsa-miR-369-3p
NUDT4	hsa-miR-3967
NUDT4	hsa-miR-2362
NUDT4	hsa-miR-668
NUDT4	hsa-miR-31-5p
NUDT4	hsa-miR-4722-5p
NUDT4	hsa-miR-5591-3p
NUDT4	hsa-miR-3929
NUDT4	hsa-miR-605
NUDT4	hsa-miR-7-5p
NUDT4	hsa-miR-296-3p
NUDT4	hsa-miR-2480
NUDT4	hsa-miR-3185
NUDT4	hsa-miR-294
NUDT4	hsa-miR-8824
NUDT4	hsa-miR-200a-3p
NUDT4	hsa-miR-3613b
NUDT4	hsa-miR-1434-5p
NUDT4	hsa-miR-497-5p
NUDT4	hsa-miR-593-3p
NUDT4	hsa-miR-1324
NUDT4	hsa-miR-2110
NUDT4	hsa-miR-467f
NUDT4	hsa-miR-1255a
NUDT4	hsa-miR-7366-5p
NUDT4	hsa-miR-548am-3p
NUDT4	hsa-miR-218a-1-3p
NUDT4	hsa-miR-665
NUDT4	hsa-miR-559
NUDT4	hsa-miR-1238-3p
NUDT4	hsa-miR-4446-3p
NUDT4	hsa-miR-6770-5p
NUDT4	hsa-miR-600
NUDT4	hsa-miR-409
NUDT4	hsa-miR-7061-5p
NUDT4	hsa-miR-1247-5p
NUDT4	hsa-miR-8086
NUDT4	hsa-miR-760-5p
NUDT4	hsa-miR-15a-3p
NUDT4	hsa-miR-155-5p
NUDT4	hsa-miR-145-5p
NUDT4	hsa-miR-5787
NUDT4	hsa-miR-343
NUDT4	hsa-miR-769
NUDT4	hsa-miR-381-3p
NUDT4	hsa-miR-105-5p

NUDT4	hsa-miR-8846
NUDT4	hsa-miR-517
NUDT4	hsa-miR-548ab
NUDT4	hsa-miR-545-3p
NUDT4	hsa-miR-2358
NUDT4	hsa-miR-138-2-3p
NUDT4	hsa-miR-885
NUDT4	hsa-miR-378-3p
NUDT4	hsa-miR-182-5p
NUDT4	hsa-miR-4666a-3p
NUDT4	hsa-miR-130a-5p
NUDT4	hsa-miR-449b-3p
NUDT4	hsa-miR-6777-3p
NUDT4	hsa-miR-25-3p
NUDT4	hsa-miR-451b
NUDT4	hsa-miR-4755-3p
NUDT4	hsa-miR-7370-3p
NUDT4	hsa-miR-186-5p
NUDT4	hsa-miR-3089-5p
NUDT4	hsa-miR-3084-3p
NUDT4	hsa-miR-3619-5p
NUDT4	hsa-miR-548u
NUDT4	hsa-miR-7388a-3p
NUDT4	hsa-miR-340-5p
NUDT4	hsa-miR-7221-5p
NUDT4	hsa-miR-194-5p
NUDT4	hsa-miR-590-5p
NUDT4	hsa-miR-5196-3p
NUDT4	hsa-miR-6779-5p
NUDT4	hsa-miR-411-5p
NUDT4	hsa-miR-6793-3p
NUDT4	hsa-let-7c-1-3p
NUDT4	hsa-miR-7111-3p
NUDT4	hsa-miR-124-3p
NUDT4	hsa-miR-527
NUDT4	hsa-miR-329-3p
NUDT4	hsa-miR-6996-5p
NUDT4	hsa-miR-3059-5p
NUDT4	hsa-miR-3170
NUDT4	hsa-miR-499b-3p
NUDT4	hsa-miR-1323-3p
NUDT4	hsa-miR-150-5p
NUDT4	hsa-miR-8106
NUDT4	hsa-miR-10
NUDT4	hsa-miR-1904
NUDT4	hsa-miR-599-5p

NUDT4	hsa-miR-7192-3p
NUDT4	hsa-miR-2402
NUDT4	hsa-miR-544-3p
NUDT4	hsa-miR-6972-3p
NUDT4	hsa-miR-713
NUDT4	hsa-miR-4276
NUDT4	hsa-miR-376b-3p
NUDT4	hsa-miR-3121-3p
NUDT4	hsa-miR-1271-3p
NUDT4	hsa-miR-377-3p
NUDT4	hsa-miR-939-3p
NUDT4	hsa-miR-153
NUDT4	hsa-miR-550-3p
NUDT4	hsa-miR-4463
NUDT4	hsa-miR-466b-2-3p
NUDT4	hsa-miR-32-5p
NUDT4	hsa-miR-467b-5p
NUDT4	hsa-miR-493-5p
NUDT4	hsa-miR-6975-3p
NUDT4	hsa-miR-7058-5p
NUDT4	hsa-miR-101-1-5p
NUDT4	hsa-miR-3108-5p
NUDT4	hsa-miR-7256-5p
NUDT4	hsa-miR-6079
NUDT4	hsa-miR-548h-3p
NUDT4	hsa-miR-6742-5p
NUDT4	hsa-miR-4694-5p
NUDT4	hsa-miR-7649-3p
NUDT4	hsa-miR-4682
NUDT4	hsa-miR-8891
NUDT4	hsa-miR-2284l
NUDT4	hsa-miR-3150b-5p
NUDT4	hsa-miR-3558-3p
NUDT4	hsa-miR-7208-3p
NUDT4	hsa-miR-15c-3p
NUDT4	hsa-miR-7247-5p
NUDT4	hsa-miR-3150b-3p
NUDT4	hsa-miR-1930-5p
NUDT4	hsa-miR-1239
NUDT4	hsa-miR-412-3p
NUDT4	hsa-miR-2322-5p
NUDT4	hsa-miR-3551-3p
NUDT4	hsa-miR-7231-3p
NUDT4	hsa-miR-6834-5p
NUDT4	hsa-miR-1255-5p
NUDT4	hsa-miR-6752-5p

NUDT4	hsa-miR-3549
NUDT4	hsa-miR-2446
NUDT4	hsa-miR-3157-3p
NUDT4	hsa-miR-183-5p.1
NUDT4	hsa-miR-183-5p
NUDT4	hsa-miR-7009-5p
NUDT4	hsa-miR-6982-3p
NUDT4	hsa-miR-512-5p
NUDT4	hsa-miR-6785-5p
NUDT4	hsa-miR-142-5p
NUDT4	hsa-miR-6941-3p
NUDT4	hsa-miR-6839-5p
NUDT4	hsa-miR-211-5p
NUDT4	hsa-miR-5119
NUDT4	hsa-miR-2355-5p
NUDT4	hsa-miR-6828-3p
NUDT4	hsa-miR-3658
NUDT4	hsa-miR-633
NUDT4	hsa-miR-6858-3p
NUDT4	hsa-miR-503-3p
NUDT4	hsa-miR-3102-3p.2-3p
NUDT4	hsa-miR-5098
NUDT4	hsa-miR-212-5p
NUDT4	hsa-miR-5089-5p
NUDT4	hsa-miR-124-5p
NUDT4	hsa-miR-8853
NUDT4	hsa-miR-624-3p
NUDT4	hsa-miR-7347-5p
NUDT4	hsa-miR-6858-5p
NUDT4	hsa-miR-666-3p
NUDT4	hsa-miR-3910
NUDT4	hsa-miR-548f-3p
NUDT4	hsa-miR-700-5p
NUDT4	hsa-miR-26
NUDT4	hsa-miR-299a-5p
NUDT4	hsa-miR-3099
NUDT4	hsa-miR-548b-3p
NUDT4	hsa-miR-7208-5p
NUDT4	hsa-miR-6800-3p
NUDT4	hsa-miR-548x-3p
NUDT4	hsa-miR-520gh
NUDT4	hsa-miR-468-3p
NUDT4	hsa-miR-432-5p
NUDT4	hsa-miR-1195
NUDT4	hsa-miR-7386k-5p
NUDT4	hsa-miR-2421

NUDT4	hsa-miR-4760-3p
NUDT4	hsa-miR-668-3p
NUDT4	hsa-miR-3960
NUDT4	hsa-miR-548ae-5p
NUDT4	hsa-miR-139-5p
NUDT4	hsa-miR-6973a-5p
NUDT4	hsa-miR-3570
NUDT4	hsa-miR-138-5p
NUDT4	hsa-miR-320a
NUDT4	hsa-miR-6507-5p
NUDT4	hsa-miR-764-3p
NUDT4	hsa-miR-1942
NUDT4	hsa-miR-4697-5p
NUDT4	hsa-miR-542
NUDT4	hsa-miR-7074-3p
NUDT4	hsa-miR-6359
NUDT4	hsa-miR-298-5p
NUDT4	hsa-miR-5589-5p
NUDT4	hsa-miR-129-2-3p
NUDT4	hsa-miR-6976-5p
NUDT4	hsa-miR-3135b
NUDT4	hsa-miR-595
NUDT4	hsa-miR-7286-3p
NUDT4	hsa-miR-651-3p
NUDT4	hsa-miR-146b-3p
NUDT4	hsa-miR-625-5p
NUDT4	hsa-miR-466c-3p
NUDT4	hsa-miR-2455
NUDT4	hsa-miR-7297-3p
NUDT4	hsa-miR-7009-3p
NUDT4	hsa-miR-3971
NUDT4	hsa-miR-3572-5p
NUDT4	hsa-miR-8874
NUDT4	hsa-miR-6506-3p
NUDT4	hsa-miR-463-5p
NUDT4	hsa-miR-502-3p
NUDT4	hsa-miR-6849-3p
NUDT4	hsa-miR-3082-3p
NUDT4	hsa-miR-2285
NUDT4	hsa-miR-3083-5p
NUDT4	hsa-miR-6994-5p
NUDT4	hsa-miR-7385e-3p
NUDT4	hsa-miR-7398l-5p
NUDT4	hsa-miR-4768-3p
NUDT4	hsa-miR-500
NUDT4	hsa-miR-376c-5p

NUDT4	hsa-miR-4772-3p
NUDT4	hsa-miR-500-3p
NUDT4	hsa-miR-520f-5p
NUDT4	hsa-miR-7260-5p
NUDT4	hsa-miR-200bc-3p
NUDT4	hsa-miR-584-3p
NUDT4	hsa-miR-8869
NUDT4	hsa-miR-18-3p
NUDT4	hsa-miR-550a-3-5p
NUDT4	hsa-miR-361-5p
NUDT4	hsa-miR-548ah-5p
NUDT4	hsa-miR-680
NUDT4	hsa-miR-380-5p
NUDT4	hsa-miR-224-5p
NUDT4	hsa-miR-429
NUDT4	hsa-miR-6992-5p
NUDT4	hsa-miR-654-5p
NUDT4	hsa-miR-3081-5p
NUDT4	hsa-miR-6780b-5p
NUDT4	hsa-miR-465a-5p
NUDT4	hsa-miR-6077
NUDT4	hsa-miR-203b-3p
NUDT4	hsa-miR-4310
NUDT4	hsa-miR-4797-5p
NUDT4	hsa-miR-5125
NUDT4	hsa-miR-7298-5p
NUDT4	hsa-miR-6812-5p
NUDT4	hsa-miR-7264-3p
NUDT4	hsa-miR-4662-5p
NUDT4	hsa-miR-3103-5p
NUDT4	hsa-miR-2284-5p
NUDT4	hsa-miR-374b-3p
NUDT4	hsa-miR-7702
NUDT4	hsa-miR-7113-3p
SNUPN	hsa-miR-8901
SNUPN	hsa-miR-324-3p
SNUPN	hsa-miR-2489
SNUPN	hsa-miR-7035-3p
SNUPN	hsa-miR-1247-3p
SNUPN	hsa-miR-376b-5p
SNUPN	hsa-miR-8868
SNUPN	hsa-miR-520f-5p
SNUPN	hsa-miR-4260
SNUPN	hsa-miR-660-3p
SNUPN	hsa-miR-9-3p
SNUPN	hsa-miR-375

SNUPN	hsa-miR-336-3p
SNUPN	hsa-miR-2329-3p
SNUPN	hsa-miR-2376
SNUPN	hsa-miR-1207
SNUPN	hsa-miR-205-3p
SNUPN	hsa-miR-708-5p
SNUPN	hsa-miR-18-3p
SNUPN	hsa-miR-378-3p
SNUPN	hsa-miR-6945-3p
SNUPN	hsa-miR-1540-5p
SNUPN	hsa-miR-378
SNUPN	hsa-miR-3925-3p
SNUPN	hsa-miR-2477
SNUPN	hsa-miR-7010-5p
SNUPN	hsa-miR-1298-3p
SNUPN	hsa-miR-346
SNUPN	hsa-miR-7069-3p
SNUPN	hsa-miR-4639-3p
SNUPN	hsa-miR-493
SNUPN	hsa-miR-3104-5p
SNUPN	hsa-miR-7215-3p
SNUPN	hsa-miR-335-5p
SNUPN	hsa-miR-7340-3p
SNUPN	hsa-miR-3192
SNUPN	hsa-miR-1227-5p
SNUPN	hsa-miR-1193-3p
SNUPN	hsa-miR-665
SNUPN	hsa-miR-128-1-5p
SNUPN	hsa-miR-370-5p
SNUPN	hsa-miR-6807-5p
SNUPN	hsa-miR-582-3p
SNUPN	hsa-miR-8872
SNUPN	hsa-miR-463-5p
SNUPN	hsa-miR-766-3p
SNUPN	hsa-miR-1839-3p
SNUPN	hsa-miR-3180-5p
SNUPN	hsa-miR-2458
SNUPN	hsa-miR-7297-5p
SNUPN	hsa-miR-7391-5p
SNUPN	hsa-miR-2465
SNUPN	hsa-miR-5193
SNUPN	hsa-miR-6897-5p
SNUPN	hsa-miR-212-3p
SNUPN	hsa-miR-103-2-5p
SNUPN	hsa-miR-3076-5p
SNUPN	hsa-miR-4289

SNUPN	hsa-miR-619-5p
SNUPN	hsa-miR-8056
SNUPN	hsa-miR-135-5p
SNUPN	hsa-miR-7027-5p
SNUPN	hsa-miR-181c-3p
SNUPN	hsa-miR-758-3p
SNUPN	hsa-miR-8866
SNUPN	hsa-miR-7363-5p
SNUPN	hsa-miR-182-5p
SNUPN	hsa-miR-6506-5p
SNUPN	hsa-miR-7339-5p
SNUPN	hsa-miR-8874
SNUPN	hsa-miR-3978
SNUPN	hsa-miR-7864
SNUPN	hsa-miR-877-3p
SNUPN	hsa-miR-10b-5p
SNUPN	hsa-miR-411a
SNUPN	hsa-miR-708-5p
SNUPN	hsa-miR-3622b-3p
SNUPN	hsa-miR-193b-5p
SNUPN	hsa-miR-6760-3p
SNUPN	hsa-miR-136-3p
SNUPN	hsa-miR-4427
SNUPN	hsa-miR-4509
SNUPN	hsa-miR-490-3p
SNUPN	hsa-miR-3569-3p
SNUPN	hsa-miR-6119-3p
SNUPN	hsa-miR-372-5p
SNUPN	hsa-miR-10-5p
SNUPN	hsa-miR-7015-3p
SNUPN	hsa-miR-2356
SNUPN	hsa-miR-3909
SNUPN	hsa-miR-92a-2-5p
SNUPN	hsa-miR-3942-3p
SNUPN	hsa-miR-7330-5p
SNUPN	hsa-miR-8059
SNUPN	hsa-miR-4515
SNUPN	hsa-miR-7291-3p
SNUPN	hsa-miR-7239-5p
SNUPN	hsa-miR-7157-5p
SNUPN	hsa-miR-224-5p
SNUPN	hsa-miR-6523b
SNUPN	hsa-miR-6720-5p
SNUPN	hsa-miR-3093-3p
SNUPN	hsa-miR-7261-5p
SNUPN	hsa-miR-483-5p

SNUPN	hsa-miR-7084-3p
SNUPN	hsa-miR-1306-3p
SNUPN	hsa-miR-206
SNUPN	hsa-miR-1208
SNUPN	hsa-miR-28-5p
SNUPN	hsa-miR-422
SNUPN	hsa-miR-577
SNUPN	hsa-miR-135
SNUPN	hsa-miR-4640-5p
SNUPN	hsa-miR-452-5p
SNUPN	hsa-miR-5100
SNUPN	hsa-miR-763
SNUPN	hsa-miR-452-5p
SNUPN	hsa-miR-1914-5p
SNUPN	hsa-miR-23-3p
SNUPN	hsa-miR-7683-3p
SNUPN	hsa-miR-7227-3p
SNUPN	hsa-miR-22-5p
SNUPN	hsa-miR-6765-3p
SNUPN	hsa-miR-6833-5p
SNUPN	hsa-miR-7052-3p
SNUPN	hsa-miR-7383-3p
SNUPN	hsa-miR-543-3p
SNUPN	hsa-miR-505-5p
SNUPN	hsa-miR-7066-3p
SNUPN	hsa-miR-7007-3p
SNUPN	hsa-miR-6769b-3p
SNUPN	hsa-miR-664-3p
SNUPN	hsa-miR-2897
SNUPN	hsa-miR-186-5p
SNUPN	hsa-miR-6735-3p
SNUPN	hsa-miR-592-3p
SNUPN	hsa-miR-5187-3p
SNUPN	hsa-miR-383-3p
SNUPN	hsa-miR-8827
SNUPN	hsa-miR-3192-5p
SNUPN	hsa-miR-6934-3p
SNUPN	hsa-miR-6858-3p
SNUPN	hsa-miR-3622a-3p
SNUPN	hsa-miR-3691-5p
SNUPN	hsa-miR-2380
SNUPN	hsa-miR-4448
SNUPN	hsa-miR-7384-3p
SNUPN	hsa-miR-4752
SNUPN	hsa-miR-320e
SNUPN	hsa-miR-2387

SNUPN	hsa-miR-7051-3p
SNUPN	hsa-miR-128-3p
SNUPN	hsa-miR-338-3p
SNUPN	hsa-miR-875-5p
SNUPN	hsa-miR-23b-5p
SNUPN	hsa-miR-880-3p
SNUPN	hsa-miR-224-5p
SNUPN	hsa-miR-8055
SNUPN	hsa-miR-216c-5p
SNUPN	hsa-miR-125-5p
SNUPN	hsa-miR-7382-5p
SNUPN	hsa-miR-8895
SNUPN	hsa-miR-376c
SNUPN	hsa-miR-558
SNUPN	hsa-miR-186-5p
SNUPN	hsa-miR-7385b-3p
SNUPN	hsa-miR-874-3p
SNUPN	hsa-miR-7296-5p
SNUPN	hsa-miR-6526
SNUPN	hsa-miR-4699-3p
SNUPN	hsa-miR-330-5p
SNUPN	hsa-miR-6927-3p
SNUPN	hsa-miR-132-3p
SNUPN	hsa-miR-2317
SNUPN	hsa-miR-4314
SNUPN	hsa-miR-326-3p
SNUPN	hsa-miR-875-3p
SNUPN	hsa-miR-96-5p
SNUPN	hsa-miR-328-5p
SNUPN	hsa-miR-4474-3p
SNUPN	hsa-miR-376c-5p
SNUPN	hsa-miR-1292-5p
SNUPN	hsa-miR-668
SNUPN	hsa-miR-122
SNUPN	hsa-miR-128
SNUPN	hsa-miR-28-5p
SNUPN	hsa-miR-551b-5p
SNUPN	hsa-miR-766-3p
SNUPN	hsa-miR-3098-5p
SNUPN	hsa-miR-7298-3p
SNUPN	hsa-miR-8825
SNUPN	hsa-miR-6755-3p
SNUPN	hsa-miR-4310
SNUPN	hsa-miR-1-5p
SNUPN	hsa-miR-1269a
SNUPN	hsa-miR-7646-5p

SNUPN	hsa-miR-29
SNUPN	hsa-miR-5619-5p
SNUPN	hsa-miR-600
SNUPN	hsa-miR-4793-5p
SNUPN	hsa-miR-7054-3p
SNUPN	hsa-miR-1207-3p
SNUPN	hsa-miR-10b-3p
SNUPN	hsa-miR-629-5p
SNUPN	hsa-miR-7158-5p
SNUPN	hsa-miR-4762-5p
SNUPN	hsa-miR-6936-5p
SNUPN	hsa-miR-103-3p
SNUPN	hsa-miR-1288
SNUPN	hsa-miR-3580-3p
SNUPN	hsa-miR-767-3p
SNUPN	hsa-miR-107-3p
SNUPN	hsa-miR-4740-5p
SNUPN	hsa-miR-5572
SNUPN	hsa-miR-6992-3p
SNUPN	hsa-miR-338-3p
SNUPN	hsa-miR-145-3p
SNUPN	hsa-miR-7000-3p
SNUPN	hsa-miR-3183
SNUPN	hsa-miR-668-3p
SNUPN	hsa-miR-669bf-3p
SNUPN	hsa-miR-7212-3p
SNUPN	hsa-miR-4471
SNUPN	hsa-miR-7111-3p
SNUPN	hsa-miR-1292-5p
SNUPN	hsa-miR-6845-5p
SNUPN	hsa-miR-29b-1-5p
SNUPN	hsa-miR-4267
SNUPN	hsa-miR-524-5p
SNUPN	hsa-miR-2290
SNUPN	hsa-miR-6762-5p
SNUPN	hsa-miR-4723-3p
SNUPN	hsa-miR-1-3p
SNUPN	hsa-miR-1288-3p
SNUPN	hsa-miR-6401
SNUPN	hsa-miR-490-3p
SNUPN	hsa-miR-377-3p
SNUPN	hsa-miR-27
SNUPN	hsa-miR-6794-3p
SNUPN	hsa-miR-892-3p
SNUPN	hsa-miR-6984-5p
SNUPN	hsa-miR-27-3p

SNUPN	hsa-miR-4680-3p
SNUPN	hsa-miR-4646-5p
SNUPN	hsa-miR-4744
SNUPN	hsa-miR-204-3p
SNUPN	hsa-miR-450a-2-3p
SNUPN	hsa-miR-134
SNUPN	hsa-miR-3133
SNUPN	hsa-miR-6839-3p
WDR4	hsa-miR-5571-3p
WDR4	hsa-miR-4672
WDR4	hsa-miR-7-3p
WDR4	hsa-miR-3148
WDR4	hsa-miR-942-3p
WDR4	hsa-miR-363-3p
WDR4	hsa-miR-1285-5p
WDR4	hsa-miR-5683
WDR4	hsa-miR-7975
WDR4	hsa-miR-6784-3p
WDR4	hsa-miR-6874-5p
WDR4	hsa-miR-199-3p
WDR4	hsa-miR-222-5p
WDR4	hsa-miR-421
WDR4	hsa-miR-1306-3p
WDR4	hsa-miR-936
WDR4	hsa-miR-367-3p
WDR4	hsa-miR-6716-3p
WDR4	hsa-miR-548g-3p
WDR4	hsa-miR-495-3p
WDR4	hsa-miR-377-3p
WDR4	hsa-miR-4716-5p
WDR4	hsa-miR-8082
WDR4	hsa-miR-6792-5p
WDR4	hsa-miR-185-3p
WDR4	hsa-miR-221-3p
WDR4	hsa-miR-6847-5p
WDR4	hsa-miR-4797-3p
WDR4	hsa-miR-485
WDR4	hsa-miR-7846-3p
WDR4	hsa-miR-1229-3p
WDR4	hsa-miR-495-3p
WDR4	hsa-miR-302b-5p
WDR4	hsa-miR-188-3p
WDR4	hsa-miR-3613-3p
WDR4	hsa-miR-203b-5p
WDR4	hsa-miR-32-5p
WDR4	hsa-miR-216b-3p

WDR4	hsa-miR-4534
WDR4	hsa-miR-4633-3p
WDR4	hsa-miR-4283
WDR4	hsa-miR-6500-5p
WDR4	hsa-miR-222-3p
WDR4	hsa-miR-4495
WDR4	hsa-miR-2467-3p
WDR4	hsa-miR-4699-3p
WDR4	hsa-miR-342-3p
WDR4	hsa-miR-6511-3p
WDR4	hsa-miR-4802-3p
WDR4	hsa-miR-27a-5p
WDR4	hsa-miR-3129-5p
WDR4	hsa-miR-4665-3p
WDR4	hsa-miR-4804-3p
WDR4	hsa-miR-7198-3p
WDR4	hsa-miR-543-3p
WDR4	hsa-miR-3914
WDR4	hsa-miR-6862-3p
WDR4	hsa-miR-505-3p
WDR4	hsa-miR-4448
WDR4	hsa-miR-1323-5p
WDR4	hsa-miR-4282
WDR4	hsa-miR-221-3p
WDR4	hsa-miR-596
WDR4	hsa-miR-222-3p
WDR4	hsa-miR-140-3p.1
WDR4	hsa-miR-1908-3p
WDR4	hsa-miR-384
WDR4	hsa-miR-28-3p
WDR4	hsa-miR-4733-5p
WDR4	hsa-miR-4326
WDR4	hsa-miR-582-5p
WDR4	hsa-miR-6772-3p
WDR4	hsa-miR-9-5p
WDR4	hsa-miR-5582-3p
WDR4	hsa-miR-92-3p
WDR4	hsa-miR-5004-5p
WDR4	hsa-miR-6768-5p
WDR4	hsa-miR-3675-5p
WDR4	hsa-miR-6718-5p
WDR4	hsa-miR-4489
WDR4	hsa-miR-216a-5p
WDR4	hsa-miR-302d-5p
WDR4	hsa-miR-25-3p
WDR4	hsa-miR-7186-3p

WDR4	hsa-miR-4268
WDR4	hsa-miR-194-5p
WDR4	hsa-miR-196b-3p
WDR4	hsa-miR-1323
